# Unveiling Crucivirus Diversity by Mining Metagenomic Data

**DOI:** 10.1128/mBio.01410-20

**Published:** 2020-09-01

**Authors:** Ignacio de la Higuera, George W. Kasun, Ellis L. Torrance, Alyssa A. Pratt, Amberlee Maluenda, Jonathan Colombet, Maxime Bisseux, Viviane Ravet, Anisha Dayaram, Daisy Stainton, Simona Kraberger, Peyman Zawar-Reza, Sharyn Goldstien, James V. Briskie, Robyn White, Helen Taylor, Christopher Gomez, David G. Ainley, Jon S. Harding, Rafaela S. Fontenele, Joshua Schreck, Simone G. Ribeiro, Stephen A. Oswald, Jennifer M. Arnold, François Enault, Arvind Varsani, Kenneth M. Stedman

**Affiliations:** a Department of Biology, Center for Life in Extreme Environments, Portland State University, Portland, Oregon, USA; b Université Clermont Auvergne, CNRS, Laboratoire Microorganismes: Génome et Environnement, UMR 6023, Clermont–Ferrand, France; c Institut für Neurophysiology, Charité-Universitätsmedizin, Berlin, Germany; d Department of Entomology and Plant Pathology, Division of Agriculture, University of Arkansas System, Fayetteville, Arkansas, USA; e The Biodesign Center for Fundamental and Applied Microbiomics, Center for Evolution and Medicine, School of Life Sciences, Arizona State University, Tempe, Arizona, USA; f School of Earth and Environment, University of Canterbury, Christchurch, New Zealand; g School of Biological Sciences, University of Canterbury, Christchurch, New Zealand; h Department of Anatomy, University of Otago, Dunedin, New Zealand; i Graduate School of Maritime Sciences, Laboratory of Sediment Hazards and Disaster Risk, Kobe University, Kobe City, Japan; j HT Harvey and Associates, Los Gatos, California, USA; k Embrapa Recursos Genéticos e Biotecnologia, Brasília, DF, Brazil; l Division of Science, Pennsylvania State University, Reading, Pennsylvania, USA; m Structural Biology Research Unit, Department of Clinical Laboratory Sciences, University of Cape Town, Rondebosch, Cape Town, South Africa; Oregon State University

**Keywords:** crucivirus, CRESS-DNA viruses, gene transfer, recombination, virus evolution, environmental virology

## Abstract

The discovery of cruciviruses revealed the most explicit example of a common protein homologue between DNA and RNA viruses to date. Cruciviruses are a novel group of circular Rep-encoding single-stranded DNA (ssDNA) (CRESS-DNA) viruses that encode capsid proteins that are most closely related to those encoded by RNA viruses in the family *Tombusviridae*. The apparent chimeric nature of the two core proteins encoded by crucivirus genomes suggests horizontal gene transfer of capsid genes between DNA and RNA viruses. Here, we identified and characterized 451 new crucivirus genomes and 10 capsid-encoding circular genetic elements through *de novo* assembly and mining of metagenomic data. These genomes are highly diverse, as demonstrated by sequence comparisons and phylogenetic analysis of subsets of the protein sequences they encode. Most of the variation is reflected in the replication-associated protein (Rep) sequences, and much of the sequence diversity appears to be due to recombination. Our results suggest that recombination tends to occur more frequently among groups of cruciviruses with relatively similar capsid proteins and that the exchange of Rep protein domains between cruciviruses is rarer than intergenic recombination. Additionally, we suggest members of the stramenopiles/alveolates/Rhizaria supergroup as possible crucivirus hosts. Altogether, we provide a comprehensive and descriptive characterization of cruciviruses.

## Abstract (precis)

Viruses are the most abundant biological entities on Earth. In addition to their impact on animal and plant health, viruses have important roles in ecosystem dynamics as well as in the evolution of the biosphere. Circular Rep-encoding single-stranded (CRESS) DNA viruses are ubiquitous in nature, many are agriculturally important, and they appear to have multiple origins from prokaryotic plasmids. A subset of CRESS-DNA viruses, the cruciviruses, have homologues of capsid proteins encoded by RNA viruses. The genetic structure of cruciviruses attests to the transfer of capsid genes between disparate groups of viruses. However, the evolutionary history of cruciviruses is still unclear. By collecting and analyzing cruciviral sequence data, we provide a deeper insight into the evolutionary intricacies of cruciviruses. Our results reveal an unexpected diversity of this virus group, with frequent recombination as an important determinant of variability.

## INTRODUCTION

In the last decade, metagenomics has allowed for the study of viruses from a new angle; viruses are not merely agents of disease but abundant and diverse members of ecosystems (1, 2). Viruses have been shaping the biosphere probably since the origin of life, as they are important drivers of the evolution of the organisms they infect (3–5). However, the origin of viruses is not entirely clear. Viruses, as replicons and mobile elements, are also subject to evolution. Virus variability is driven by various mutation rates, recombination, and reassortment of genetic components (6). These attributes, coupled with many types of genomes (RNA or DNA, single or double stranded, and circular or linear), lead to a large genetic diversity in the “viral world.”

Viruses are generally classified based on the nature of their transmitted genetic material (7). Viral genetic information is coded in either RNA or DNA. Moreover, these genomes can be single (positive or negative sense) or double stranded, or linear or circular, and can be comprised of a single or multiple molecules of nucleic acid (monopartite or multipartite, respectively). These different groups of viruses have different replication strategies, and they harbor distinct taxa based on their genome arrangement and composition (1). The striking differences between viral groups with disparate genome types suggest polyphyletic virus origins (8).

For example, the highly abundant circular Rep-encoding single-stranded DNA (CRESS-DNA; Rep being the replication-associated protein) viruses may have been derived from plasmids on multiple occasions by acquiring capsid genes from RNA viruses (9–11). Eukaryotic CRESS-DNA viruses, recently classified into the phylum *Cressdnaviricota* (12), constitute a diverse and widespread group of viruses with circular genomes—some of them multipartite—that contains the families *Geminiviridae*, *Circoviridae*, *Nanoviridae*, *Alphasatellitidae*, *Genomoviridae*, *Bacilladnaviridae*, *Smacoviridae*, and *Redondoviridae*, in addition to vast numbers of unclassified viruses (13, 14). Universal to all CRESS-DNA viruses is the Rep protein, which is involved in the initiation of the virus’ rolling-circle replication. Rep homologues are also encoded in plasmids (14, 15). Some pathogenic CRESS-DNA viruses are agriculturally important, such as porcine circoviruses, and nanoviruses and geminiviruses that infect a wide range of plant hosts (13). However, many CRESS-DNA viruses have been identified in apparently healthy organisms, and metagenomic studies have revealed their presence in most environments (13).

In 2012, a metagenomic survey of a hot and acidic lake in the volcanic Cascade Range of the western United States uncovered a new type of circular DNA virus (16). The genome of this virus appears to make it a CRESS-DNA virus based on the circularity of its sequence, the presence of a *rep* gene, and a predicted stem-loop structure with a conserved nucleotide sequence (*ori*) that serves as an origin for CRESS-DNA virus rolling-circle replication (reviewed in references 17 and 18). Interestingly, the amino acid sequence of the capsid protein encoded by this genome resembles those encoded by RNA viruses in the family *Tombusviridae* (16). It was hypothesized that this virus originated by the acquisition of a capsid gene from an RNA virus through a yet-to-be-demonstrated RNA-DNA recombination event (16, 19). Since the discovery of this putatively “chimeric virus,” 80 circular sequences encoding a Rep that shares homology to ssDNA viruses and a capsid protein that shares homology to tombusvirus capsid proteins have been found in different environments around the globe (20–32). This growing group of viruses have been branded “cruciviruses,” as they imply the crossing between CRESS-DNA viruses and RNA tombusviruses (28). Cruciviruses have been found associated with forams (21), alveolates hosted by isopods (27), arthropods (20, 23) and in peatland ecosystems (28), but no host for cruciviruses has been elucidated to date.

The circular genome of known cruciviruses is variable in size, ranging from 2.7 to 5.7 kb, and often contains open reading frames (ORFs) in addition to the Rep and capsid genes, which have been found in either a unisense or an ambisense orientation (21, 28). The function of additional crucivirus ORFs is unclear due to their lack of sequence similarity with any characterized protein. The genome replication of CRESS-DNA viruses is initiated by the Rep protein, which binds to direct repeats present just downstream of the stem of the *ori*-containing stem-loop structure and nicks the ssDNA (33, 34). The exposed 3′ OH serves as a primer for cellular enzymes to replicate the viral genome via rolling-circle replication (34–36). The exact terminating events of CRESS-DNA virus replication are poorly understood for most CRESS-DNA viruses, but Rep is known to be involved in the sealing of newly replicated genomes (34, 36–38).

Rep has a domain in the N terminus that belongs to the HUH endonuclease superfamily (39). This family of proteins is characterized by a HUH motif (motif II), in which two histidine residues are separated by a bulky hydrophobic amino acid, and a Tyr-containing motif (motif III) that catalyzes the nicking of the ssDNA (33, 39–41). CRESS-DNA virus Reps also contain a third conserved motif in the N-terminal portion of the protein (motif I), likely responsible for double-stranded DNA (dsDNA) binding specificity (42). In many CRESS-DNA viruses, the HUH motif has been replaced with a similar motif that lacks the second histidine residue (e.g., circoviruses have replaced HUH with HLQ) (10, 39). The C-terminal portion of eukaryotic CRESS-DNA virus Reps contains a superfamily 3 helicase domain (S3H) that may be responsible for unwinding dsDNA replicative intermediates (43, 44). This helicase domain is characterized by Walker A and B motifs, motif C, and an Arg finger. Previous studies have identified evidence of recombination in the endonuclease and helicase domains of Rep, which contributes to the potential ambiguity of Rep phylogenies (45). Interestingly, the Rep proteins of different cruciviruses have been shown to be similar to CRESS-DNA viruses in different families, including circoviruses, nanoviruses, and geminiviruses (21, 28). In some cruciviruses, these differences in phylogeny have been observed between the individual domains of a single Rep protein (22, 28). The apparent polyphyly of crucivirus Reps suggests recombination events involving cruciviruses and other CRESS-DNA viruses, even within Reps (21, 22).

All characterized CRESS-DNA viruses package their DNA into small capsids with icosahedral symmetry or their geminate variants, built from multiple copies of the capsid protein encoded in their genome (13). The capsid protein of these CRESS-DNA viruses appears to fold into an eight-strand β-barrel that conforms to the single jelly-roll (SJR) architecture, which is also commonly found in eukaryotic RNA viruses (46). The capsid protein of cruciviruses has no detectable sequence similarity with the capsid of other CRESS-DNA viruses and is predicted to adopt the SJR conformation found in the capsid protein of tombusviruses (16, 21, 22). Three domains can be distinguished in tombusviral capsid proteins (47, 48). From the N to the C terminus, they are (i) the RNA-interacting or R-domain, a disordered region that faces the interior of the viral particle to interact with the nucleic acid through abundant basic residues (49, 50); (ii) the shell or S-domain containing the single jelly-roll fold and the architectural base of the capsid (48); and (iii) the protruding or P-domain, which decorates the surface of the virion and is involved in host transmission (51). In tombusviruses, the S-domains of 180 capsid protein subunits interact with each other to assemble around the viral RNA in a T=3 fashion, forming an Ø∼35-nm virion (48, 52).

The study of cruciviruses suggests evidence for the transfer of capsid genes between disparate viral groups, which can shed light on virus origins and the phenotypic plasticity of virus capsids. Here, we document the discovery of 461 new crucivirus (CruV) genomes and cruci-like circular genetic elements (CruCGEs) identified in metagenomic data obtained from different environments and organisms. This study provides a comprehensive analysis of this greatly expanded data set and explores the extent of cruciviral diversity—mostly due to Rep heterogeneity—impacted by rampant recombination.

## RESULTS AND DISCUSSION

### Expansion of the crucivirus group.

To broaden our understanding of the diversity and relationships of cruciviruses, 461 uncharacterized circular DNA sequences containing predicted coding sequences (CDSs) with sequence similarity to the capsid protein of tombusviruses were compiled from metagenomic sequencing data (see Table S1 in the supplemental material). The data came from published and unpublished metagenomic studies, carried out in a wide variety of environments, from permafrost to temperate lakes, and on various organisms from red algae to invertebrates (metagenomes and their metadata are provided in Table S2 in the supplemental material).

**FIG 1:**
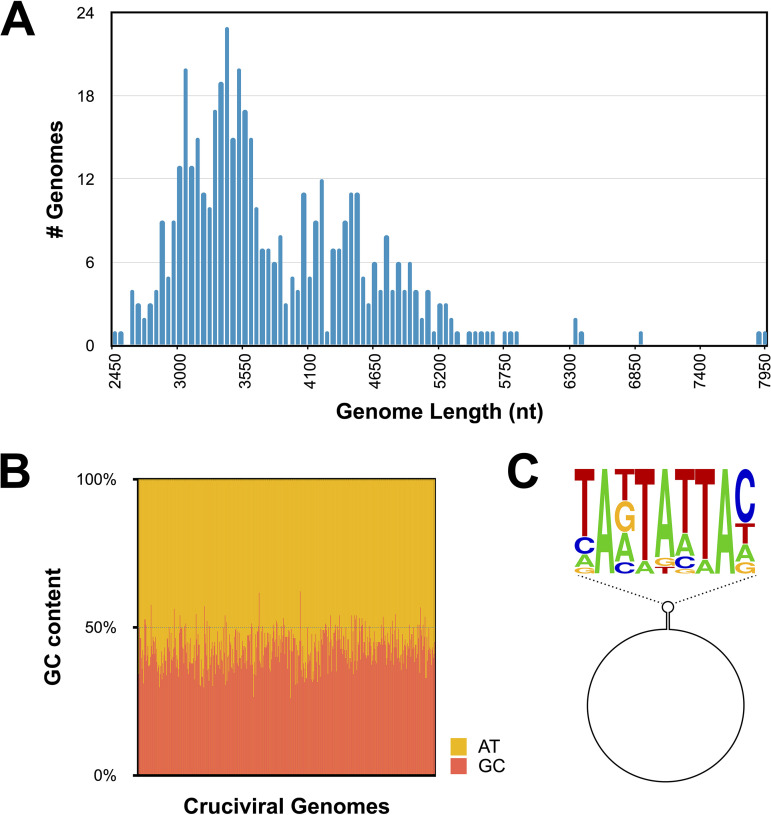
Genome properties of 461 new cruciviral circular sequences. (A) Histogram of cruciviral genome lengths categorized in 50-nt bins. (B) Percentage of G+C content versus A+T in each of the sequences described in this study. (C) Relative abundance of nucleotides in the conserved nonanucleotide sequence of the 211 stem-loops and putative origins of replication represented predicted with StemLoop-Finder (A. A. Pratt et al., unpublished) in Sequence Logo format.

The cruciviral sequences were named sequentially, beginning with the smallest genome, which was named CruV-81 to account for the 80 crucivirus genomes reported in prior literature (16, 20–32). The average GC content of the newly described cruciviral sequences is 42.9% ± 4.9% (Fig. 1B) with genome lengths spanning from 2,474 to 7,947 bases (Fig. 1A), some exceeding the size of described bacilladnaviruses (≤6,000 nucleotides [nt] [53]), the largest CRESS-DNA viruses known (12).

Of the 461 sequences that contain a capsid protein ORF, 451 have putative coding regions with sequence similarity to Rep of CRESS-DNA viruses (10). The capsid protein and Rep ORFs are encoded in a unisense orientation in 40% of the genomes and an ambisense orientation in 58% of the genomes. The remaining ∼2% correspond to 10 CruCGEs with no clear Rep CDS. Five of these CruCGEs contain a predicted origin of rolling-circle replication (Table S1), indicating that they are circular genomes that undergo rolling-circle replication characteristic of other CRESS-DNA virus genomes (17, 18).

10.1128/mBio.01410-20.2 TABLE S1 Properties of cruciviruses (CruV) and cruci-like circular genetic elements (CruCGE). Download Table S1, PDF file, 1.9 MB. Copyright © 2020 de la Higuera et al. 2020 de la Higuera et al. This content is distributed under the terms of the Creative Commons Attribution 4.0 International license.

10.1128/mBio.01410-20.3 TABLE S2 Viromes analyzed. Download Table S2, PDF file, 1.1 MB. Copyright © 2020 de la Higuera et al. 2020 de la Higuera et al. This content is distributed under the terms of the Creative Commons Attribution 4.0 International license.

One possible reason for the lack of a Rep ORF in certain sequences is that some of these may be subgenomic molecules or possible components of multipartite viruses (54). Some CRESS-DNA viruses, such as geminiviruses and nanoviruses, have multipartite genomes (55). Moreover, some ssRNA tombunodaviruses, including Plasmopara halstedii virus A and Sclerophthora macrospora virus A—viruses that contain the capsid sequences most similar to cruciviral capsids (16, 28)—also have multipartite genomes (56). Unfortunately, no reliable method yet exists to match different sequences belonging to the same multisegmented virus in metagenomes, making identification of multipartite or segmented viruses from metagenomic data challenging (55).

Stem-loop structures with conserved nonanucleotide motifs as putative origins of replication were predicted and annotated in 277 cruciviral sequences with StemLoop-Finder (A. A. Pratt, I. de la Higuera, E. L. Torrance, G. W. Kasun, and K. M. Stedman, unpublished data). In some cases, more than one nonanucleotide motif with similar scores were found for a single genome, resulting in more than one stem-loop annotation. Of the annotated genomes, 223 contain a stem-loop with a nonanucleotide with a NANTANTAN pattern, with the most common sequence being the canonical circovirus motif TAGTATTAC, found in 64 of the genomes (Table S1) (57). The majority of the 54 sequences that do not correspond to NANTANTAN contain a TAWWDHWAN nonanucleotide motif, typical of genomoviruses (58). The frequency of bases at each position in the nonanucleotide sequence is given in Fig. 1C and reflects similarity to motifs found in other CRESS-DNA viruses (10).

### Crucivirus capsid protein.

**FIG 2:**
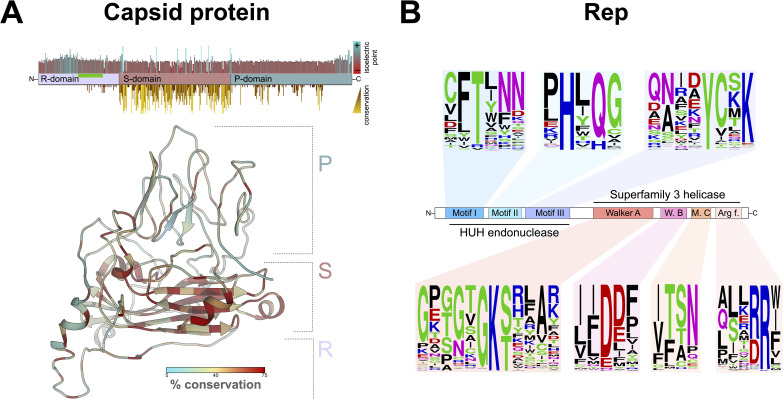
Protein conservation in cruciviruses. (A) (Top) Distribution of domains, isoelectric point, and conservation in a consensus capsid protein. Four hundred sixty-one capsid protein sequences were aligned in Geneious 11.0.4 with MAFFT (G-INS-i, BLOSUM 45, open gap penalty 1.53, offset 0.123) and trimmed manually. The conservation of the physicochemical properties at each position was obtained with Jalview v2.11.0 (88), and the isoelectric point was estimated in Geneious 11.0.4. The region of the capsid protein rich in glycine is highlighted with a green bar. (Bottom) Structure of a cruciviral capsid protein (CruV-359) as predicted by Phyre^2^ showing sequence conservation based on an alignment of the 47 capsid protein sequences from the capsid protein-based clusters. (B) Conserved motifs found in cruciviral Reps after aligning all the extracted Rep protein sequences using PSI-Coffee (94). Sequence logos were generated at http://weblogo.threeplusone.com to indicate the frequency of residues at each position.

**FIG 3:**
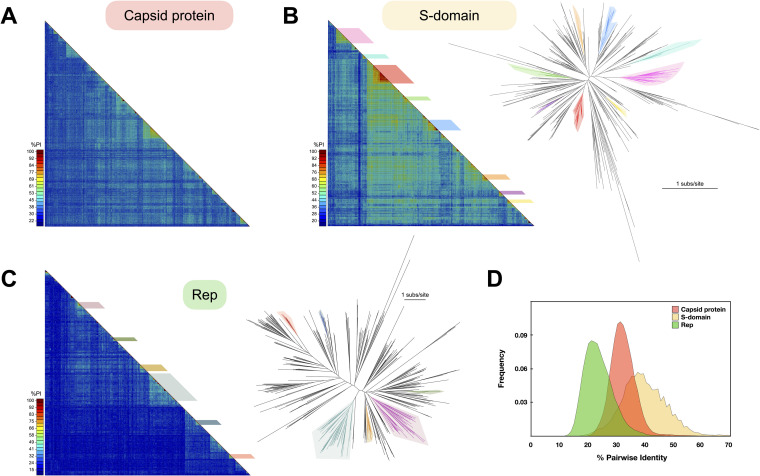
Diversity of cruciviral proteins. (A) Capsid protein diversity. Pairwise amino acid identity (%PI) between the capsid proteins predicted for 461 cruciviral sequences. The alignment and analysis were carried out with SDT, using the integrated MAFFT algorithm. (B) S-domain diversity. (Left) Pairwise identity matrix between the capsid protein predicted S-domains of the 461 sequences described in this study. The alignment and analysis were carried out with SDT, using the integrated MAFFT algorithm (87). The colored boxes indicate the different clusters of sequences used to create the capsid protein-based cluster sequence subset. (Right) Unrooted phylogenetic tree obtained with FastTree from a manually curated MAFFT alignment of the translated sequences of the S-domain (G-INS-i, BLOSUM 45, open gap penalty 1.53, offset 0.123) (93, 96). The colored branches represent the different clusters observed in the matrix. Scale bar indicates substitutions per site. (C) Rep diversity. (Left) Pairwise identity matrix between all Reps found in cruciviral genomes in this study. The alignment and analysis were carried out with SDT, using the integrated MUSCLE algorithm (87). (Right) Unrooted phylogenetic tree obtained with FastTree from a PSI-Coffee alignment of the translated sequences of Rep trimmed with TrimAl v1.3 (93–96). The colored branches represent the different clusters that contain the Rep-based cluster sequence subset. Scale bar indicates substitutions per site. (D) Pairwise identity frequency distribution. The frequency of pairwise identity values for each of the putative proteins or domains analyzed is shown.

The capsid protein of cruciviruses is predicted to have a single jelly-roll (SJR) architecture, based on its homology to tombusvirus capsid proteins, for which three-dimensional (3D) structures have been determined (Fig. 2A) (59–61). The SJR conformation is found in capsid proteins of both RNA and DNA viruses (46). The SJR capsid protein of tombusviruses and cruciviruses contains three distinct domains: the RNA-binding or R-domain, the shell or S-domain, and the protruding or P-domain (Fig. 2A). All 461 crucivirus capsid proteins analyzed in this study contain a complete S-domain. This domain contains a distinct jelly-roll fold and interacts with the S-domain of other capsid subunits in the virion of related tombusviruses (48). The S-domain of these new crucivirus sequences has greater sequence conservation than the remaining regions of the capsid protein (Fig. 2A), likely due to its functional importance in capsid structure. In tombusviruses, the S-domain contains a calcium-binding motif (DxDxxD), which was not identified in previously described cruciviruses (62). However, we detected this Ca-binding motif in 68 capsid proteins of the newly identified cruciviral sequences. These crucivirus sequences form a distinct cluster, shown in red in Fig. 3B. The S-domain is flanked on the N terminus by the R-domain, which in cruciviruses appears variable in size (up to 320 amino acids long) and appears to be truncated in some of the capsid protein sequences (e.g., CruV-386 and CruV-493). The R-domain is characterized by an abundance of basic residues at the N terminus, followed by a Gly-rich tract (Fig. 2A). The P-domain, on the C-terminal end of the capsid protein sequence, is generally the largest domain, with the exception of CruV-385, where it appears to be truncated. The conservation of the capsid protein suggests a similar structure for all cruciviruses. However, those cruciviruses with larger genomes may assemble their capsids in a different arrangement to accommodate their genome. While the capsids of tombusviruses have been shown to adopt a T=1 icosahedral conformation, rather than the usual T=3, when the R-domain is partially or totally removed (61), we have not seen a correlation between the length of capsid protein domains and genome size in our data set that could be indicative of alternative capsid arrangements. Furthermore, no packaging dynamics relating genome size and virion T-number arrangement have been determined in CRESS-DNA viruses, although subgenomic elements of geminiviruses can be packaged in nongeminate capsids (63, 64).

Interestingly, CruV-420 contains not one but two different tombusvirus-related capsid proteins. A recent compilation of CRESS-DNA viruses from animal metagenomes also contains four genomes with two different capsid proteins each (32). Whether these viruses use two different capsid proteins in their capsid (as some RNA viruses do [65]), or whether these are intermediates in the exchange of capsid genes, as predicted from the gene capture mechanism proposed by Stedman (19), is unclear. If the latter is true, capsid gene acquisition by CRESS-DNA viruses may be much more common than previously thought.

### Crucivirus Rep.

The Reps of CRESS-DNA viruses typically contain an endonuclease domain characterized by conserved motifs I, II, and III and a helicase domain with Walker A and B motifs, motif C, and an Arg finger (Fig. 2B) (13). The majority (85.9%) of the crucivirus genomes described in this data set contain all of the expected Rep motifs (Table S4). However, five genomes (CruCGE-110, CruCGE-296, CruCGE-436, CruCGE-471, and CruCGE-533) with overall sequence homology to other Reps (35.8, 32.7, 49.7, 60.2, and 57.2% pairwise identity with other putative Reps in the databases, respectively) lack any detectable conserved motifs within their sequence. Thus, these sequences are considered capsid-encoding crucivirus-like circular genetic elements (CruCGEs).

The endonuclease catalytic domain of Rep (motif II), including HUH, was identified in 441 of the genomes, 95.2% of which had an alternative HUH, with the most common arrangement being HUQ (70.0%), also found in circoviruses and nanoviruses (10, 25, 39) (Fig. 2B). Crucivirus motif II deviates from the HUH motif by additionally replacing the second hydrophobic residue (U) with a polar amino acid in 26.2% of genomes (Fig. 2B; Table S4), with 53 Reps with the sequence HYQ (12.0%) also found in smacoviruses (10, 24, 45).

We identified 13 putative Reps in these crucivirus genomes that lack all four motifs typically found in S3H helicases (e.g., CruV-166, CruV-202, and CruV-499 [Table S4]). Recent work has shown that the deletion of individual conserved motifs in the helicase domain of the Rep protein of beak and feather disease virus does not abolish ATPase and GTPase activity (66). The absence of all four motifs may prevent these putative Reps from performing helicase and ATPase activity using previously characterized mechanisms. However, it is possible that crucivirus Reps that lack these motifs are still capable of ATP hydrolysis and associated helicase activity. Alternatively, these activities may be provided by host factors (67), or by a viral replication-enhancer protein—as is the case with the AC3 protein of begomoviruses (68).

We identified 36 crucivirus genomes whose putative *rep* genes contain in-frame stop codons or in which the HUH and SF3 helicase are in different frames, suggesting that their transcripts may require intron splicing prior to translation. Acceptor and donor splicing sites identical to those found in maize streak virus (69) were found in all these sequences, and the putatively spliced Reps were annotated accordingly. In five of the 36 spliced Reps, we were unable to detect any of the four conserved motifs associated with helicase/ATPase activity, which are encoded in the predicted second exon in most cases. CruV-513 and CruV-518 also contain predicted splicing sites in their capsid gene.

No geminivirus Rep sequence (GRS) motifs—which have been identified as necessary for geminivirus replication (70) and have also been found in genomoviruses (58)—were detected in Reps in our data set. We were unable to detect any conserved Rep motifs present in cruciviruses that are absent in other CRESS-DNA viruses. Given the conservation of Rep motifs in these newly described cruciviruses, we expect most to be active in rolling-circle replication.

### Crucivirus capsid proteins share higher genetic identity than their Rep proteins.

To assess the diversity in the proteins of cruciviruses, the percent pairwise identity between the protein sequences was calculated for capsid protein and Rep using SDTv1.2 (Fig. 3). The average pairwise identity for the capsid protein was found to be 33.1% ± 4.9% (mean ± SD) (Fig. 3A and D), likely due to the high levels of conservation found in the S-domain (40.5% ± 8.4%) (Fig. 3B and D), while the average pairwise identity for Rep is quite low at 24.7% ± 5.6% (Fig. 3C and D). The differences in average pairwise identities between Rep, capsid protein, and S-domain are statistically significant (one-way analysis of variance [ANOVA]; *P* < 0.0001). The high variation of the Rep protein sequence relative to the capsid protein in cruciviruses correlates with a previous observation on a smaller data set (21).

**FIG 4:**
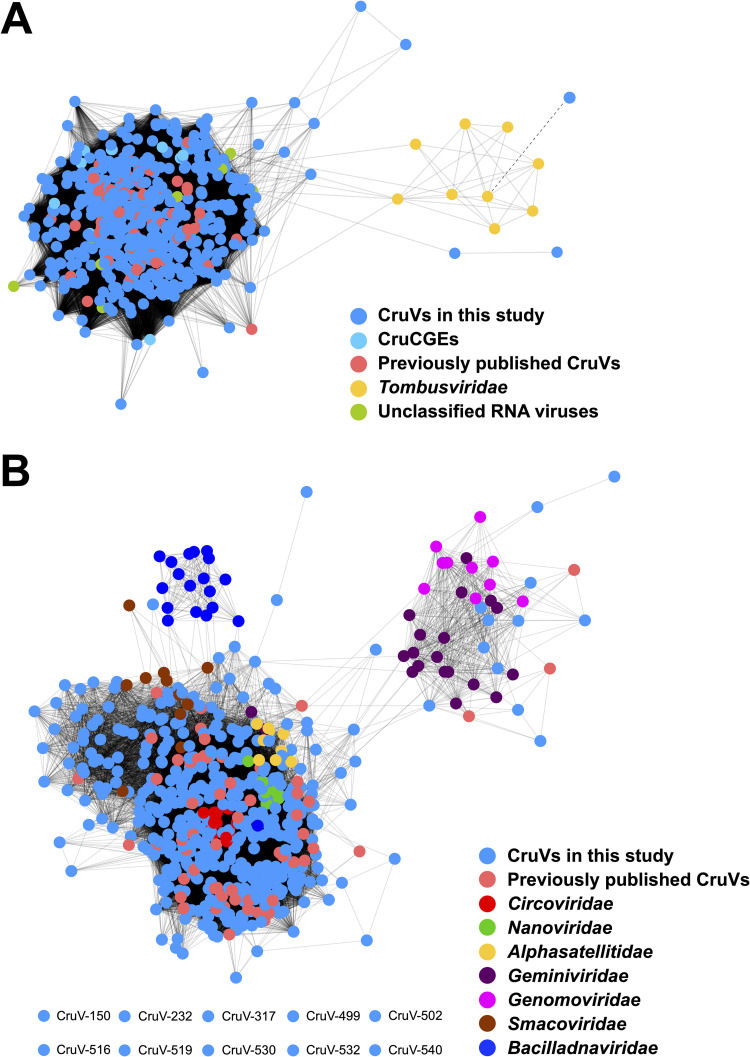
Similarity networks of cruciviral proteins with related viruses. (A) Capsid proteins represented by colored dots are connected with a solid line when the pairwise similarity, as assessed by the EFI-EST web server (100), has an E value of <10**^−^**^20^. The dashed line represents an E value of 6 × 10^−7^ between the nodes corresponding to the capsid protein of CruV-523 and turnip crinkle virus, as given by BLASTp. (B) Replication-associated protein (Rep) translations, represented by colored dots, are connected with a solid line when the pairwise similarity has an E value of <10^−10^. The eight nodes at the bottom left did not connect to any other node. All networks were carried out with pairwise identities calculated in the EFI–EST web server and visualized in Cytoscape v3.7.2 (100, 101).

To compare cruciviruses to other viral groups with homologous proteins, sequence similarity networks were built for the capsid protein and Rep (Fig. 4). For the capsid protein, related protein sequences from tombusviruses and unclassified RNA viruses were included. The virus sequences were connected when the similarity between their protein sequence had an E value of <10^−20^, sufficient to connect all cruciviruses and tombusviruses, with the exception of CruV-523 (Fig. 4A). However, using BLASTp, CruV-523 showed similarity to other RNA viruses with an E value of <10^−9^, which were not included in the analysis. The capsid protein sequence similarity network analysis demonstrates the apparent homology of the capsid proteins in our data set with the capsid protein of RNA viruses: specifically, to unclassified RNA viruses that have RNA-dependent RNA polymerases (RdRPs) similar to those of either tombusviruses—also described as tombus-like viruses (56, 71, 72)—or nodaviruses. The latter RNA viruses are proposed to belong to a chimeric group of viruses named tombunodaviruses (73).

For sequence similarity network analysis of Rep, sequences from CRESS-DNA viruses belonging to the families *Circoviridae*, *Nanoviridae*, *Alphasatellitidae*, *Geminiviridae*, *Genomoviridae*, *Smacoviridae*, and *Bacilladnaviridae* were used (Fig. 4B). Due to the heterogeneity of Rep (Fig. 3C), the score cutoff for the network was relaxed to an E value of <10^−10^; nonetheless, 10 divergent sequences lacked sufficient similarity to form connections within the network. While the Reps of the different viral families clustered in specific regions of the network, the similarity of cruciviral Reps spans the diversity of all CRESS-DNA viruses and blurs the borders between them. Though there are cruciviruses that appear to be closely related to geminiviruses and genomoviruses, these connections are less common than with other classified CRESS-DNA families (Fig. 4B). While still highly divergent from each other, the conserved motifs in the Rep still share the most sequence similarity with CRESS-DNA viruses (Fig. 2B).

The broad sequence space distribution of cruciviral Rep sequences has been proposed to reflect multiple Rep acquisition events through recombination with viruses from different CRESS-DNA viral families (21). However, the apparent larger diversity of cruciviral Reps relative to classified CRESS-DNA viruses can be due to the method of study, as most classified CRESS-DNA viruses have been discovered from infected organisms and are grouped mainly based on Rep similarity (1). In contrast, here crucivirus sequences are selected according to the presence of a tombusvirus-like capsid protein. Moreover, the Rep of cruciviruses could be subject to higher substitution rates than the capsid protein (27). It is possible that sequence divergence in capsid protein is more limited than in the Rep due to structural constraints.

### Horizontal gene transfer among cruciviruses.

To gain insight into the evolutionary history of cruciviruses, we carried out phylogenetic analyses of their capsid proteins and Reps. Due to the high sequence diversity in the data set, two smaller subsets of sequences were analyzed.

**FIG 5:**
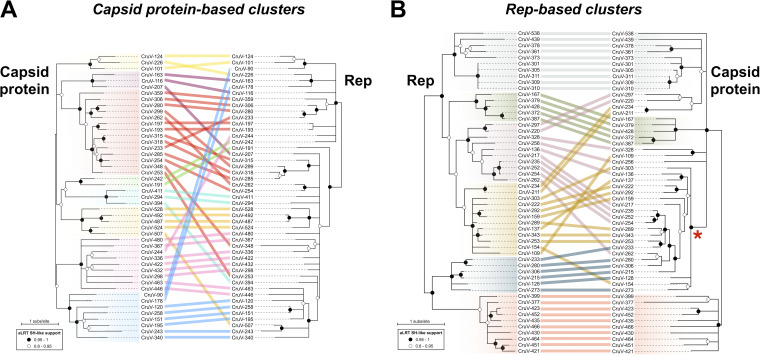
Comparison of phylogenies of capsid and Rep proteins of representative cruciviruses. (A) Tanglegram calculated with Dendroscope v3.5.10 from phylogenetic trees generated with PhyML from capsid protein (PhyML automatic model selection LG+G+I+F) and Rep (PhyML automatic model selection RtREV+G+I) alignments (97, 99). The tips corresponding to the same viral genome are linked by lines that are color coded according to the clusters obtained from Fig. 3A (capsid protein-based clusters). (B) Tanglegram calculated with Dendroscope v3.5.10 from phylogenetic trees generated with PhyML from capsid protein (PhyML automatic model selection LG+G+I+F) and Rep (PhyML automatic model selection RtREV+G+I) alignments (99). The tips corresponding to the same viral sequence are linked by lines that are color coded according to the clusters obtained from Fig. 3B (Rep-based clusters). The clade marked with a red asterisk is formed by members of the red capsid protein-based cluster. Branch support is given according to aLRT SH-like (97). All nodes with an aLRT SH-like branch support inferior to 0.8 were collapsed with Dendroscope prior to constructing the tanglegram.

**(i) Capsid protein-based clusters.** Clusters with more than six nonidentical capsid protein sequences whose S-domains share a pairwise identity greater than 70% were visually identified from Fig. 3B. This resulted in the identification of seven clusters, and one more divergent, yet clearly distinct, cluster was included (pink in Fig. 3B). A total of 47 genomes from the eight different clusters were selected for sequence comparison. The protein sequences of capsid and Rep were extracted and aligned, and their phylogenies were inferred and analyzed using tanglegrams (Fig. 5A). The capsid protein phylogeny shows that the sequences from the eight capsid protein-based clusters form separate clades (Fig. 5A). On the other hand, the phylogeny of Rep shows a different pattern of relatedness between those genomes (Fig. 5A). This suggests different evolutionary histories for the capsid and Rep proteins, which could be due to recombination events between cruciviruses, as previously proposed with smaller data sets (21, 22).

**(ii) Rep-based clusters.** To account for the possible bias introduced by selecting genomes from capsid protein cluster groups and to increase the resolution in the phylogeny of the Rep sequences, clusters of crucivirus genomes with more than six Rep sequences sharing pairwise identity of >45% and <98% were identified. The cutoff values were chosen to allow for the selection of six clusters containing a total of 53 genomes (Fig. 3C), whose capsid and Rep protein sequences were analyzed. The phylogeny of Reps shows distinct clades between the sequences from different Rep-based clusters (Fig. 5B). When the phylogeny of Rep was compared to that of their corresponding capsid proteins, we observed cruciviruses that group together in both Rep and capsid protein phylogenies. Discrepancies in topology between Rep and capsid protein trees were observed as well, particularly in the capsid protein clade marked with an asterisk in Fig. 5B. This clade corresponds to the highly homogeneous red capsid protein-based cluster shown in Fig. 3B and suggests that gene transfer is more common in cruciviruses with a more similar capsid protein, likely infecting the same type of organism. On the other hand, the presence of cruciviral groups with no trace of genetic exchange may indicate that lineages within the cruciviral group may have undergone speciation in the course of evolution.

**FIG 6:**
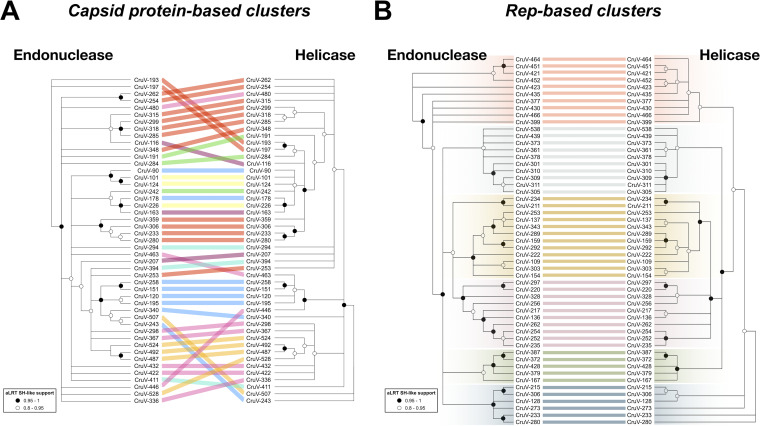
Comparison of phylogenies between the endonuclease and helicase domains of Reps from representative cruciviruses. (A) Tanglegram calculated with Dendroscope v3.5.10 from phylogenetic trees generated with PhyML from separate alignments of Rep endonuclease and helicase domains (97, 99). The tips corresponding to the same viral genome are linked by lines that are color coded according to the clusters obtained from Fig. 3A (capsid protein-based clusters). (B) Same as panel A but with sequences from the clusters obtained from Fig. 3B (Rep-based clusters). All nodes with an aLRT SH-like branch support inferior to 0.8 were collapsed with Dendroscope v3.5.10 prior to constructing the tanglegram (99).

To investigate possible exchanges of individual Rep domains among cruciviruses, the Rep alignments of the analyses of the capsid protein-based and Rep-based clusters were split at the beginning of the Walker A motif to separate endonuclease and helicase domains. From the analysis of the capsid protein-based clusters, we observed incongruence in the phylogenies between endonuclease and helicase domains (Fig. 6A), suggesting recombination within crucivirus Reps, as has been previously hypothesized with a much smaller data set (22). This incongruency is not observed in the analyzed Rep-based clusters (Fig. 6B). This is likely due to the higher similarity between Reps in this subset of sequences, biased by the clustering based on Rep. We do observe different topologies between the trees, which may be a consequence of different evolutionary constraints to which the endonuclease and helicase domains are subjected. The detection of capsid protein/Rep exchange and not of individual Rep domains in Rep-based clusters suggests that the rate of intergenic recombination is higher than intragenic recombination in cruciviruses.

### Members of the stramenopiles/alveolates/Rhizaria (SAR) supergroup are potential crucivirus hosts.

While no crucivirus host has been identified to date, the architecture of the Rep protein found in most cruciviruses, as well as the presence of introns in some of the genomes, suggests a eukaryotic host. The fusion of an endonuclease domain to an S3H helicase domain is observed in other CRESS-DNA viruses which are known to infect eukaryotes (39). This is distinct from Reps found in prokaryote-infecting CRESS-DNA viruses—which lack a fused S3H helicase domain (74)—and other related HUH endonucleases involved in plasmid rolling-circle replication and HUH transposases (39). Additionally, the capsid protein of cruciviruses, a suggested determinant of tropism (75, 76), is homologous to the capsid of RNA viruses known to infect eukaryotes. The RNA viruses with a known host with capsids most similar to cruciviral capsids (tombunodaviruses) infect oomycetes, a group of filamentous eukaryotic stramenopiles (56).

Cruciviruses have been found as contaminants of spin columns made of diatomaceous silica (22), in aquatic metagenomes enriched with unicellular algae (21), in the metagenome of Astrammina rara—a foraminiferan protist part of the Rhizaria (21)—and associated with epibionts of isopods, mainly comprised of apicomplexans and ciliates, both belonging to the alveolates (27). These pieces of evidence point toward the stramenopiles/alveolates/Rhizaria (SAR) supergroup as a candidate taxon to contain potential crucivirus hosts (77). No host prediction can be articulated from our sequence data. However, at least five of the crucivirus genomes render complete translated capsid protein and Rep sequences only when using a relaxed genetic code. Such alternative genetic codes have been detected in ciliates, in which the hypothetical termination codons UAA and UAG encode a glutamine (78). The usage of an alternative genetic code seems evident in CruV-502—found in the metagenome from seawater collected above diseased coral colonies (79) that uses a UAA codon for a glutamine of the S-domain conserved in 33.5% of the sequences. While the data accumulated suggest unicellular eukaryotes and SAR members as crucivirus-associated organisms, the host of cruciviruses remains elusive, and further investigations are necessary.

### Classification of cruciviruses.

Cruciviruses have circular genomes that encode a Rep protein probably involved in rolling-circle replication. The single-stranded nature of packaged crucivirus genomes has not been demonstrated experimentally; however, the overall genomic structure and sequence similarity underpin the placement of cruciviruses within the CRESS-DNA viruses.

The classification of the CRESS-DNA viruses is primarily based upon the phylogeny of the Rep proteins, although commonalities in capsid protein and genome organization are also considered (14). This taxonomic criterion is challenging in cruciviruses, whose Rep proteins are highly diverse and apparently paralogous. Whether the use of proteins involved in replication for virus classification should be preferred over structural proteins has been previously questioned (80).

The capsid of cruciviruses, as well as the capsid of other CRESS-DNA virus families like circoviruses, geminiviruses, and bacilladnaviruses, possesses the single jelly-roll architecture (46). However, there is no obvious sequence similarity between the capsid protein of cruciviruses and that of classified CRESS-DNA viruses. The crucivirus capsid protein—homologous to the capsid of tombusviruses—is an orthologous trait within the CRESS-DNA viruses. Hence, the capsid protein constitutes a synapomorphic character that demarcates this group of viruses from the rest of the CRESS-DNA viral families.

CRESS-DNA viruses appear to have multiple origins from plasmids. Their Rep proteins appear to have arisen from these plasmids, and the viruses have diverged into different ssDNA virus groups on acquisition of nonorthologous capsid proteins from RNA viruses (10, 81). Cruciviruses, however, are classified as such due to shared capsid protein genes but encode Rep proteins that span many different viral clusters within the phylum *Cressdnaviricota*, as we have shown. Thus, it is unlikely that cruciviruses will form a formal taxon, as they appear to be a collection of viruses from multiple *Cressdnaviricota* groups. However, like Baltimore classes, the label crucivirus can aid in understanding virus evolution, particularly the transfer of capsid protein genes, which appears to have been rampant not only in ssDNA viruses but throughout the virosphere (46, 81).

### Concluding remarks.

Cruciviruses are a growing group of CRESS-DNA viruses that encode capsid proteins that are homologous to those encoded by tombusviruses. Over 500 crucivirus genomes have been recovered from various environments across the globe. These genomes vary in size, sequence, and genome organization. While crucivirus capsid proteins are relatively homogeneous, the Reps are relatively diverse among the cruciviruses, spanning the diversity of all classified CRESS-DNA viruses. It has been hypothesized that cruciviruses emerged from the recombination between a CRESS-DNA virus and a tombus-like RNA virus (16, 19). Furthermore, cruciviruses seem to have recombined with each other to exchange functional modules between themselves, and probably with other viral groups, which blurs their evolutionary history. Cruciviruses show evidence of genetic transfer, not just between viruses with similar genomic properties but also between disparate groups of viruses such as CRESS-DNA and RNA viruses.

## MATERIALS AND METHODS

### Assembly and recovery of viral genomes.

A total of 461 crucivirus-related sequences were identified from 1,168 metagenomic surveys (see Tables S1 and S2 in the supplemental material). One thousand one hundred sixty-seven viromes from 57 published data sets and one unpublished virome were obtained from different types of environments: (i) aquatic systems (freshwater, seawater, hypersaline ponds, thermal springs, and hydrothermal vents), (ii) engineered systems (bioreactor and food production), and (iii) eukaryote-associated flora (human, insect and other animal feces, human saliva and fluids, cnidarians, and plants). The raw reads from metagenomes were assembled using multiple different programs (for details see Table S1), except for the sequences from the work of de Cárcer et al. (82), which were already assembled. New cruciviral sequences were identified in these viromes by screening circular contigs for the presence of capsid proteins from previously known cruciviruses (21) and tombusviruses, using a BLASTx bit-score threshold of 50. The selected genomes are assumed to be complete and circular based on the terminal redundancy identified in *de novo*-assembled genomes.

Additionally, sequences CruV-240, CruV-300, CruV-331, CruV-338, and CruV-367 were retrieved as assembled contigs from the Joint Genome Institute (JGI)’s IMG/VR repository (83), by searching scaffolds with a function set including the protein family pfam00729, corresponding to the S-domain of tombusvirus capsids. The sequences with an RdRP coding region were excluded, and the circularity of the sequences, as well as the presence of an ORF encoding a tombusvirus-like capsid, was confirmed with Geneious 11.0.4 (Biomatters, Ltd.).

### Annotation of crucivirus putative genes.

The 461 cruciviral sequences were annotated and analyzed in Geneious 11.0.4. Coding sequences (CDSs) were semiautomatically annotated from a custom database (Table S3) of protein sequences of published cruciviruses and close homologues obtained from GenBank, using Geneious 11.0.4’s annotation function with a 25% nucleotide similarity threshold. Annotated CDSs were rechecked with the GenBank database using BLASTx to identify sequences similar to previously described cruciviruses and putative relatives. Sequences containing in-frame stop codons were checked for putative splicing sites (69) or translated using a ciliate genetic code only when usage rendered a complete ORF with similarity to other putative crucivirus CDSs. Predicted ORFs longer than 300 bases with no obvious homologues and no overlap with capsid protein or Rep-like ORFs were annotated as “putative ORFs.”

10.1128/mBio.01410-20.4 TABLE S3 Custom library for crucivirus CDS annotation. Download Table S3, PDF file, 0.1 MB. Copyright © 2020 de la Higuera et al. 2020 de la Higuera et al. This content is distributed under the terms of the Creative Commons Attribution 4.0 International license.

10.1128/mBio.01410-20.5 TABLE S4 Crucivirus Rep motifs. Download Table S4, PDF file, 0.3 MB. Copyright © 2020 de la Higuera et al. 2020 de la Higuera et al. This content is distributed under the terms of the Creative Commons Attribution 4.0 International license.

### Putative stem-loop annotation.

Stem-loop structures that could serve as an origin of replication for circular ssDNA viruses were identified and annotated using StemLoop-Finder (34, 84; A. A. Pratt et al., unpublished data). The 461 cruciviral sequences were scanned for the presence of conserved nonanucleotide motifs described for other CRESS-DNA viruses (NANTANTAN, NAKWRTTAC, TAWWDHWAN, and TRAKATTRC) (13). The integrated ViennaRNA 2.0 library was used to predict secondary structures of DNA around the detected motif, including the surrounding 15 to 20 nucleotides on either side (85, 86). Predicted structures with a stem longer than 4 bp and a loop including seven or more bases were subjected to the default scoring system, which increases the score by one point for each deviation from ideal stem lengths of 11 bp and loop lengths of 11 nucleotides. A set of annotations for stem-loops and nonanucleotides was created with StemLoop-Finder for those with a score of 15 or below. Putative stem-loops were excluded from annotation when a separate stem-loop was found with the same first base, but they attained a greater score, as well as those that appeared to have a nonanucleotide within four bases of their stem-loop structure’s first or last nucleotide.

### Conservation analysis and visualization.

**(i) Pairwise identity matrices.** The pairwise identity between the protein sequence from translated cruciviral genes was calculated with SDTv1.2 (87), with MAFFT alignment option for capsid proteins and S-domains and MUSCLE alignment options for Reps. The raw data were further analyzed with Prism v8.4.3.

**(ii) Sequence conservation annotation.** Capsid protein sequence conservation represented in Fig. 2A was generated with Jalview v2.11.0 (88) and reflects the conservation of the physicochemical properties for each column of the alignment (89).

**(iii) Sequence logos.** Sequence logos showing frequency of bases in nonanucleotides at the origin of replication or residue in conserved Rep motifs were made using the WebLogo server (http://weblogo.threeplusone.com/) (90).

**(iv) Structural representation of capsid conservation.** The 3D structure of the capsid protein was modeled with Phyre^2^ (91). The generated graphic was colored by sequence conservation with Chimera v.1.13 (92), from the alignment of the 47 capsid sequences from each of the capsid protein-based clusters (Fig. 3B).

### Phylogenetic analyses.

**(i) Multiple sequence alignments.** Capsid protein sequences were aligned using MAFFT (93) in Geneious 11.0.4, with a G-INS-i algorithm and BLOSUM 45 as exchange matrix, with an open gap penalty of 1.53 and an offset value of 0.123, and manually curated. Rep protein sequences were aligned using PSI-Coffee (http://tcoffee.crg.cat/) (94). Rep alignments were manually inspected and corrected in Geneious 11.0.4 and trimmed using TrimAI v1.3 with a *strict plus* setting (95). To produce individual alignments of the endonuclease and helicase domains, the full-length trimmed alignments were split at the Walker A motif (45).

**(ii) Phylogenetic trees.** Phylogenetic trees containing the entire data set of cruciviral sequences were built in Geneious using the FastTree plugin (96). For the analysis of sequence subsets, trees were inferred with the PhyML 3.0 web server (http://www.atgc-montpellier.fr/phyml/) (97), using an aLRT SH-like support (98). The substitution model for each analysis was automatically selected by the program.

**(iii) Intergene and interdomain comparison.** Tanglegrams were made using Dendroscope v3.5.10 (99) to compare the phylogenies between different genes or domains within the same set of crucivirus genomes.

**(iv) Sequence similarity networks.** A total of 540 capsid amino acid sequences and 600 Rep amino acid sequences were uploaded tothe EFI–EST web server for the calculation of pairwise identities (https://efi.igb.illinois.edu/efi-est/) (100). A specific alignment score cutoff was established for each data set analyzed, and xgmml files generated by EFI-EST were visualized and edited in Cytoscape v3.7.2 (101).

### Data availability.

Accession numbers are provided in Table S1, and all sequences are provided in Text S1.

10.1128/mBio.01410-20.1 TEXT S1 Annotated cruciviral sequences in GenBank format. Download Text S1, DOCX file, 1.9 MB. Copyright © 2020 de la Higuera et al. 2020 de la Higuera et al. This content is distributed under the terms of the Creative Commons Attribution 4.0 International license.
