# Supplementary material for: Unveiling Crucivirus Diversity by Mining Metagenomic Data
Source: mBio. 2020 Sep 1;11(5):e01410-20. doi: 10.1128/mBio.01410-20 (PMC7468197; doi:10.1128/mBio.01410-20)
Supplement: TABLE S1 [file mBio.01410-20-st001.pdf]

Supplementary Table 1: Properties of cruciviruses (CruV) and cruci-like circular genetic elements (CruCGE)

Name	Length	%GC	Genetic Code	Genome organization	ori (nona)	Sequences Subset	Location	Publication	Source	Sequencing	Assembly software	Notes	Accession number*
CruV-081	2472	34.1%	Standard	Ambisense	TAATATTAC		River (NZ)		vDNA, MDA	Illumina HiSeq	metaSPAdes		MT263540
CruCGE-082	2535	50.1%	Standard	no rep	CATTATTAC		unnamed Arctic pond (78°02.935′N; 13°41.973′E)	Aguirre de Cárcer et al., 2015	vDNA, MDA	Illumina HiSeq	IDBA-UD		PRJEB5265
CruV-083	2612	46.3%	Standard	Ambisense	#N/A		unnamed Arctic pond (78°02.935′N; 13°41.973′E)	Aguirre de Cárcer et al., 2015	vDNA, MDA	Illumina HiSeq	IDBA-UD	Spliced Rep	PRJEB5265
CruV-084	2612	46.3%	Standard	Ambisense	#N/A		Lake Tunsjøen (78°03.375′N; 13°40.313′E)	Aguirre de Cárcer et al., 2015	vDNA, MDA	Illumina HiSeq	IDBA-UD	Spliced Rep	PRJEB5265
CruCGE-085	2618	39.6%	Standard	no rep	#N/A		Chirominidae (NZ)		vDNA, MDA	Illumina HiSeq	metaSPAdes		MT263538
CruV-086	2621	35.8%	Standard	Unisense	#N/A		Lake Tunsjøen (78°03.375′N; 13°40.313′E)	Aguirre de Cárcer et al., 2015	vDNA, MDA	Illumina HiSeq	IDBA-UD		PRJEB5265
CruV-087	2667	42.2%	Standard	Ambisense	#N/A		Lake Nordammen (78°38.279′N; 16°44.025′E)	Aguirre de Cárcer et al., 2015	vDNA, MDA	Illumina HiSeq	IDBA-UD		PRJEB5265
CruV-088	2671	31.1%	Standard	Ambisense	TAGTATTAC		Green lipped muscles (NZ)		vDNA, MDA	Illumina HiSeq	metaSPAdes		MT263541
CruV-089	2690	40.4%	Standard	Unisense	#N/A		Lake Tunsjøen (78°03.375′N; 13°40.313′E)	Aguirre de Cárcer et al., 2015	vDNA, MDA	Illumina HiSeq	IDBA-UD		PRJEB5265
CruV-090	2702	52.5%	Standard	Ambisense	#N/A	Capsid protein-based clusters	Lake Tunsjøen (78°03.375′N; 13°40.313′E)	Aguirre de Cárcer et al., 2015	vDNA, MDA	Illumina HiSeq	IDBA-UD		PRJEB5265
CruV-091	2702	52.6%	Standard	Ambisense	#N/A		unnamed Arctic pond (78°02.935′N; 13°41.973′E)	Aguirre de Cárcer et al., 2015	vDNA, MDA	Illumina HiSeq	IDBA-UD		PRJEB5265
CruV-092	2789	50.8%	Standard	Unisense	#N/A		Lake Aydat (45°39'52.859''N; 2°59'11.943''E) surface water		vDNA, MDA	Illumina HiSeq	IDBA-UD		MT478561
CruV-093	2795	38.4%	Standard	Ambisense	TAATACTAA		River (NZ)		vDNA, MDA	Illumina HiSeq	metaSPAdes		MT263542
CruV-094	2797	40.2%	Standard	Ambisense	AAATATTAT		Soil (NZ)		vDNA, MDA	Illumina HiSeq	metaSPAdes		MT263543
CruV-095	2810	43.5%	Standard	Unisense	GATTAATAT		Lake Aydat (45°39'52.859''N; 2°59'11.943''E) surface water		vDNA, MDA	Illumina HiSeq	IDBA-UD		MT478560
CruV-096	2839	37.0%	Standard	Ambisense	TATTATAAT		Lake Tenndammen (78°06.118′N; 15°02.024′E)	Aguirre de Cárcer et al., 2015	vDNA, MDA	Illumina HiSeq	IDBA-UD		PRJEB5265
CruV-097	2839	36.8%	Standard	Ambisense	CAGTATTAC		River (NZ)		vDNA, MDA	Illumina HiSeq	metaSPAdes		MT263544
CruV-098	2849	37.2%	Standard	Ambisense	GATTATTAC / TACTATTAA / TAATAGTAA / TATTACTAT		Lake Tenndammen (78°06.118′N; 15°02.024′E)	Aguirre de Cárcer et al., 2015	vDNA, MDA	Illumina HiSeq	IDBA-UD		PRJEB5265
CruV-099	2860	40.1%	Standard	Ambisense	TATAGCTAC		River bank soil (NZ)		vDNA, MDA	Illumina HiSeq	metaSPAdes		MT263545
CruV-100	2867	57.7%	Standard	Ambisense	#N/A		Lake Tunsjøen (78°03.375′N; 13°40.313′E)	Aguirre de Cárcer et al., 2015	vDNA, MDA	Illumina HiSeq	IDBA-UD		PRJEB5265
CruV-101	2871	43.5%	Standard	Ambisense	TAATGTTAA	Capsid protein-based clusters	Lake Nordammen (78°38.279′N; 16°44.025′E)	Aguirre de Cárcer et al., 2015	vDNA, MDA	Illumina HiSeq	IDBA-UD		PRJEB5265
CruV-102	2874	40.9%	Standard	Ambisense	TAGTATTAC		River bank soil (NZ)		vDNA, MDA	Illumina HiSeq	metaSPAdes		MT263546
CruV-103	2881	46.9%	Standard	Ambisense	TAATATTAC		Lake Nordammen (78°38.279′N; 16°44.025′E)	Aguirre de Cárcer et al., 2015	vDNA, MDA	Illumina HiSeq	IDBA-UD	Spliced Rep	PRJEB5265
CruV-104	2883	45.2%	Standard	Ambisense	TATTATAAT		Lake Tunsjøen (78°03.375′N; 13°40.313′E)	Aguirre de Cárcer et al., 2015	vDNA, MDA	Illumina HiSeq	IDBA-UD	Spliced Rep	PRJEB5265
CruV-105	2883	45.1%	Standard	Ambisense	TAATATTAC		unnamed Arctic pond (78°02.935′N; 13°41.973′E)	Aguirre de Cárcer et al., 2015	vDNA, MDA	Illumina HiSeq	IDBA-UD	Spliced Rep	PRJEB5265
CruV-106	2886	40.7%	Standard	Ambisense	#N/A		Lake Aydat (45°39'52.859''N; 2°59'11.943''E) surface water		vDNA, MDA	Illumina HiSeq	IDBA-UD		MT478559
CruV-107	2899	46.8%	Standard	Ambisense	#N/A		unnamed Arctic pond (78°02.935′N; 13°41.973′E)	Aguirre de Cárcer et al., 2015	vDNA, MDA	Illumina HiSeq	IDBA-UD	Spliced Rep	PRJEB5265
CruV-108	2905	42.8%	Standard	Ambisense	CAATAATAT		Lake Tenndammen (78°06.118′N; 15°02.024′E)	Aguirre de Cárcer et al., 2015	vDNA, MDA	Illumina HiSeq	IDBA-UD		PRJEB5265
CruV-109	2911	38.1%	Standard	Ambisense	CATTAATAA / CATTACTAA	Rep-based clusters	unnamed Arctic pond (78°02.935′N; 13°41.973′E)	Aguirre de Cárcer et al., 2015	vDNA, MDA	Illumina HiSeq	IDBA-UD		PRJEB5265
CruV-110	2919	34.8%	Standard	Ambisense	TACTATTAC		Lake Tenndammen (78°06.118′N; 15°02.024′E)	Aguirre de Cárcer et al., 2015			IDBA-UD		PRJEB5265
CruV-111	2929	36.5%	Standard	Ambisense	AAGTAATAA		River (NZ)		vDNA, MDA	Illumina HiSeq	metaSPAdes		MT263547
CruV-112	2942	37.4%	Standard	Ambisense	AATTACTAT		River (NZ)		vDNA, MDA	Illumina HiSeq	metaSPAdes		MT263548
CruV-113	2952	29.7%	Standard	Ambisense	GACTATTAC		River (NZ)		vDNA, MDA	Illumina HiSeq	metaSPAdes		MT263549
CruCGE-114	2953	33.9%	Standard	no rep	TAATATTAC		unnamed Arctic pond (78°02.935′N; 13°41.973′E)	Aguirre de Cárcer et al., 2015	vDNA, MDA	Illumina HiSeq	IDBA-UD		PRJEB5265
CruV-115	2954	37.0%	Standard	Ambisense	TATTTCAAG		unnamed Arctic pond (78°02.935′N; 13°41.973′E)	Aguirre de Cárcer et al., 2015	vDNA, MDA	Illumina HiSeq	IDBA-UD		PRJEB5265
CruV-116	2963	41.8%	Standard	Ambisense	#N/A	Capsid protein-based clusters	Lake Nordammen (78°38.279′N; 16°44.025′E)	Aguirre de Cárcer et al., 2015	vDNA, MDA	Illumina HiSeq	IDBA-UD		PRJEB5265
CruV-117	2965	37.9%	Standard	Unisense	TAAAATTAC / CATTATTAA		Borgdammane pond (78°04.254′N; 13°47.652′E)	Aguirre de Cárcer et al., 2015	vDNA, MDA	Roche 454	IDBA-UD		PRJEB5265
CruCGE-118	2965	32.5%	Standard	Unisense	#N/A		unnamed Arctic pond (78°02.935′N; 13°41.973′E)	Aguirre de Cárcer et al., 2015	vDNA, MDA	Illumina HiSeq	IDBA-UD		PRJEB5265
CruV-119	2966	34.5%	Standard	Unisense	#N/A		River (NZ)		vDNA, MDA	Illumina HiSeq	metaSPAdes		MT263550
CruV-120	2983	40.6%	Standard	Ambisense	TATATAAAA	Capsid protein-based clusters	Lake Tenndammen (78°06.118′N; 15°02.024′E)	Aguirre de Cárcer et al., 2015	vDNA, MDA	Illumina HiSeq	IDBA-UD		PRJEB5265
CruV-121	2991	43.1%	Standard	Unisense	AAATACTAC		unnamed Arctic pond (78°02.935′N; 13°41.973′E)	Aguirre de Cárcer et al., 2015	vDNA, MDA	Illumina HiSeq	IDBA-UD		PRJEB5265

CruV-122	3003	38.4%	Standard	Unisense	TAATGTTAA		River sediments (NZ)		vDNA, MDA	Illumina HiSeq	metaSPAdes		MT263551
CruV-123	3009	38.6%	Standard	Ambisense	CAGTATTAC		unnamed Arctic pond (78°02.935′N; 13°41.973′E)	Aguirre de Cárcer et al., 2015	vDNA, MDA	Illumina HiSeq	IDBA-UD		PRJEB5265
CruV-124	3012	33.6%	Standard	Ambisense	#N/A	Capsid protein-based clusters	Sewage Oxydation Pond (NZ)		vDNA, MDA	Illumina HiSeq	metaSPAdes		MT263552
CruV-125	3012	46.1%	Standard	Ambisense	#N/A		Lake Aydat (45°39'52.859''N; 2°59'11.943''E) surface water		vDNA, MDA	Illumina HiSeq	IDBA-UD		MT478558
CruV-126	3019	46.0%	Standard	Unisense	#N/A		Lake Tunsjøen (78°03.375′N; 13°40.313′E)	Aguirre de Cárcer et al., 2015	vDNA, MDA	Illumina HiSeq	IDBA-UD		PRJEB5265
CruV-127	3020	43.2%	Standard	Ambisense	#N/A		Lake Tenndammen (78°06.118′N; 15°02.024′E)	Aguirre de Cárcer et al., 2015	vDNA, MDA	Illumina HiSeq	IDBA-UD		PRJEB5265
CruV-128	3023	49.7%	Standard	Unisense	TAGTATTAC	Rep-based clusters	Lake Aydat (45°39'52.859''N; 2°59'11.943''E) surface water		vDNA, MDA	Illumina HiSeq	IDBA-UD		MT478557
CruV-129	3026	41.8%	Standard	Unisense	#N/A		Lake Aydat (45°39'52.859''N; 2°59'11.943''E) surface water		vDNA, MDA	Illumina HiSeq	IDBA-UD		MT478556
CruV-130	3026	34.4%	Standard	Ambisense	#N/A		Lake Aydat (45°39'52.859''N; 2°59'11.943''E) surface water		vDNA, MDA	Illumina HiSeq	IDBA-UD		MT478555
CruV-131	3030	48.0%	Standard	Ambisense	#N/A		Lake Tenndammen (78°06.118′N; 15°02.024′E)	Aguirre de Cárcer et al., 2015	vDNA, MDA	Illumina HiSeq	IDBA-UD		PRJEB5265
CruV-132	3030	39.2%	Standard	Ambisense	TAGTATTAC		Lake Nordammen (78°38.279′N; 16°44.025′E)	Aguirre de Cárcer et al., 2015	vDNA, MDA	Illumina HiSeq	IDBA-UD		PRJEB5265
CruV-133	3040	38.7%	Standard	Unisense	TACTATTAC		Sewage Oxydation Pond (NZ)		vDNA, MDA	Illumina HiSeq	metaSPAdes		MT263553
CruV-134	3049	40.1%	Standard	Unisense	#N/A		Lake Aydat (45°39'52.859''N; 2°59'11.943''E) surface water		vDNA, MDA	Illumina HiSeq	IDBA-UD		MT478554
CruV-135	3052	42.8%	Standard	Ambisense	AATTACTAT		Lake Aydat (45°39'52.859''N; 2°59'11.943''E) surface water		vDNA, MDA	Illumina HiSeq	IDBA-UD		MT478553
CruV-136	3054	56.2%	Standard	Ambisense	TAGTAATAG TATTACTAC / AACTATTAG	Rep-based clusters	Lake Aydat (45°39'52.859''N; 2°59'11.943''E) surface water		vDNA, MDA	Illumina HiSeq	IDBA-UD		MT478552
CruV-137	3056	43.0%	Standard	Ambisense	#N/A	Rep-based clusters	Lake Aydat (45°39'52.859''N; 2°59'11.943''E) surface water		vDNA, MDA	Illumina HiSeq	IDBA-UD		MT478551
CruV-138	3060	46.3%	Standard	Ambisense	TAATACTAC		Lake Aydat (45°39'52.859''N; 2°59'11.943''E) surface water		vDNA, MDA	Illumina HiSeq	IDBA-UD		MT478550
CruV-139	3065	36.2%	Standard	Ambisense	#N/A		unnamed Arctic pond (78°02.935′N; 13°41.973′E)	Aguirre de Cárcer et al., 2015	vDNA, MDA	Illumina HiSeq	IDBA-UD		PRJEB5265
CruV-140	3066	40.2%	Standard	Ambisense	#N/A		Lake Aydat (45°39'52.859''N; 2°59'11.943''E) surface water		vDNA, MDA	Illumina HiSeq	IDBA-UD		MT478549
CruV-141	3068	39.0%	Standard	Ambisense	TATTTCTAC		Soil (NZ)		vDNA, MDA	Illumina HiSeq	metaSPAdes		MT263554
CruV-142	3068	35.8%	Standard	Ambisense	TAGTATTAC		Lake Tenndammen (78°06.118′N; 15°02.024′E)	Aguirre de Cárcer et al., 2015	vDNA, MDA	Illumina HiSeq	IDBA-UD		PRJEB5265
CruV-143	3069	33.3%	Standard	Ambisense	#N/A		Lake Nordammen (78°38.279′N; 16°44.025′E)	Aguirre de Cárcer et al., 2015	vDNA, MDA	Illumina HiSeq	IDBA-UD		PRJEB5265
CruV-144	3070	47.9%	Standard	Unisense	TAGTATTAC		Lake Tenndammen (78°06.118′N; 15°02.024′E)	Aguirre de Cárcer et al., 2015	vDNA, MDA	Illumina HiSeq	IDBA-UD		PRJEB5265
CruV-145	3076	54.1%	Standard	Ambisense	TATAGTAAG / TATAAAAAC / TATTATAAG		Bat (China)	Wu et al. 2015	vDNA, MDA	Illumina	IDBA-UD		SRR206392 1
CruV-146	3084	49.6%	Standard	Unisense	TACTACTAA / TAGTAGTAA		River (NZ)		vDNA, MDA	Illumina HiSeq	metaSPAdes		MT263555
CruV-147	3084	49.7%	Standard	Unisense	AATTATTAA		River (NZ)		vDNA, MDA	Illumina HiSeq	metaSPAdes		MT263556
CruV-148	3084	49.6%	Standard	Unisense	#N/A		River (NZ)		vDNA, MDA	Illumina HiSeq	metaSPAdes		MT263557
CruV-149	3089	43.2%	Standard	Unisense	#N/A		Lake Tunsjøen (78°03.375′N; 13°40.313′E)	Aguirre de Cárcer et al., 2015	vDNA, MDA	Illumina HiSeq	IDBA-UD		PRJEB5265
CruV-150	3094	42.6%	Standard	Unisense	TAGTATTAC		Lake Pavin (45°29'45.11''N; 2°53'14.60''E), sampling depth = 22 meters		vDNA, MDA	Illumina HiSeq	IDBA-UD		MT478548
CruV-151	3096	37.6%	Standard	Unisense	TAGTATTAC	Capsid protein-based clusters	Lake Tenndammen (78°06.118′N; 15°02.024′E)	Aguirre de Cárcer et al., 2015	vDNA, MDA	Illumina HiSeq	IDBA-UD		PRJEB5265
CruV-152	3096	50.5%	Standard	Unisense	TAGTATTAC		River (NZ)		vDNA, MDA	Illumina HiSeq	metaSPAdes		MT263558
CruV-153	3097	43.9%	Standard	Ambisense	TATTGTTAC		Lake Aydat (45°39'52.859''N; 2°59'11.943''E) surface water		vDNA, MDA	Illumina HiSeq	IDBA-UD		MT478547
CruV-154	3099	51.4%	Standard	Unisense	#N/A	Rep-based clusters	Lake Aydat (45°39'52.859''N; 2°59'11.943''E) surface water		vDNA, MDA	Illumina HiSeq	IDBA-UD		MT478546
CruCGE-155	3102	41.2%	Standard	no rep	TAGTATTAC		Lake Aydat (45°39'52.859''N; 2°59'11.943''E) surface water		vDNA, MDA	Illumina HiSeq	IDBA-UD		MT478562
CruV-156	3105	44.7%	Standard	Unisense	#N/A		Lake Nordammen (78°38.279′N; 16°44.025′E)	Aguirre de Cárcer et al., 2015	vDNA, MDA	Illumina HiSeq	IDBA-UD		PRJEB5265
CruV-157	3113	35.8%	Standard	Unisense	TAGTATTAC		Lake Aydat (45°39'52.859''N; 2°59'11.943''E) surface water		vDNA, MDA	Illumina HiSeq	IDBA-UD		MT478545
CruV-158	3121	34.2%	Standard	Unisense	#N/A		River (NZ)		vDNA, MDA	Illumina HiSeq	metaSPAdes		MT263559
CruV-159	3122	44.6%	Standard	Ambisense	#N/A	Rep-based clusters	Lake Aydat (45°39'52.859''N; 2°59'11.943''E) surface water		vDNA, MDA	Illumina HiSeq	IDBA-UD		MT478544
CruV-160	3129	38.2%	Standard	Unisense	TAGTATTAC		Lake Aydat (45°39'52.859''N; 2°59'11.943''E) surface water		vDNA, MDA	Illumina HiSeq	IDBA-UD		MT478543
CruV-161	3137	46.1%	Standard	Unisense	GAATATTAT / GACTATTAT / GAATAATAG		unnamed Arctic pond (78°02.935′N; 13°41.973′E)	Aguirre de Cárcer et al., 2015	vDNA, MDA	Illumina HiSeq	IDBA-UD		PRJEB5265

**CruV-162**	3139	37.8%	Standard	Ambisense	#N/A		Lake Tunsjøen (78°03.375′N; 13°40.313′E)	Aguirre de Cárcer et al., 2015	vDNA, MDA	Illumina HiSeq	IDBA-UD		PRJEB5265
**CruV-163**	3142	37.6%	Standard	Unisense	#N/A	Capsid protein-based clusters	River bank soil (NZ)		vDNA, MDA	Illumina HiSeq	metaSPAdes		MT263560
**CruV-164**	3142	37.7%	Standard	Ambisense	TAGTATTAC		unnamed Arctic pond (78°02.935′N; 13°41.973′E)	Aguirre de Cárcer et al., 2015	vDNA, MDA	Illumina HiSeq	IDBA-UD		PRJEB5265
**CruV-165**	3142	37.8%	Standard	Unisense	#N/A		unnamed Arctic pond (78°02.935′N; 13°41.973′E)	Aguirre de Cárcer et al., 2015	vDNA, MDA	Illumina HiSeq	IDBA-UD		PRJEB5265
**CruV-166**	3144	39.5%	Standard	Unisense	AACTAATAA		unnamed Arctic pond (78°02.935′N; 13°41.973′E)	Aguirre de Cárcer et al., 2015	vDNA, MDA	Illumina HiSeq	IDBA-UD		PRJEB5265
**CruV-167**	3145	44.0%	Standard	Unisense	CAATACTAG	Rep-based clusters	unnamed Arctic pond (78°02.935′N; 13°41.973′E)	Aguirre de Cárcer et al., 2015	vDNA, MDA	Illumina HiSeq	IDBA-UD		PRJEB5265
**CruV-168**	3150	32.4%	Standard	Unisense	#N/A		River (NZ)		vDNA, MDA	Illumina HiSeq	metaSPAdes		MT263561
**CruV-169**	3150	38.5%	Standard	Unisense	TATTGCTAC / TAATTATAG		Lake Nordammen (78°38.279′N; 16°44.025′E)	Aguirre de Cárcer et al., 2015	vDNA, MDA	Illumina HiSeq	IDBA-UD		PRJEB5265
**CruV-170**	3154	54.5%	Standard	Unisense	TATTTAAAA / AAATAATAA		Soil (NZ)		vDNA, MDA	Illumina HiSeq	metaSPAdes	Spliced Rep	MT263562
**CruV-171**	3157	45.7%	Standard	Ambisense	TAGTATTAC		Lake Aydat (45°39'52.859''N; 2°59'11.943''E) surface water		vDNA, MDA	Illumina HiSeq	IDBA-UD		MT478542
**CruV-172**	3168	40.4%	Standard	Ambisense	TATTGCTAG		Lake Nordammen (78°38.279′N; 16°44.025′E)	Aguirre de Cárcer et al., 2015	vDNA, MDA	Illumina HiSeq	IDBA-UD		PRJEB5265
**CruV-173**	3171	47.5%	Standard	Ambisense	TATAATAAT / TATTATTAT / TAATAATAA / GATTATTAT / CACTATTAA		Lake Aydat (45°39'52.859''N; 2°59'11.943''E) surface water		vDNA, MDA	Illumina HiSeq	IDBA-UD		MT478541
**CruV-174**	3172	43.9%	Standard	Ambisense	#N/A		River (NZ)		vDNA, MDA	Illumina HiSeq	metaSPAdes	Spliced Rep	MT263563
**CruV-175**	3172	43.8%	Standard	Ambisense	TAGTATTAC		Chirominidae (NZ)		vDNA, MDA	Illumina HiSeq	metaSPAdes		MT263564
**CruV-176**	3176	30.3%	Standard	Unisense	TATTGCTAG		Lake Aydat (45°39'52.859''N; 2°59'11.943''E) surface water		vDNA, MDA	Illumina HiSeq	IDBA-UD		MT478540
**CruV-177**	3176	30.1%	Standard	Unisense	#N/A		unnamed Arctic pond (78°02.935′N; 13°41.973′E)	Aguirre de Cárcer et al., 2015	vDNA, MDA	Illumina HiSeq	IDBA-UD		PRJEB5265
**CruV-178**	3177	39.1%	Standard	Ambisense	TAGTATTAC	Capsid protein-based clusters	Lake Aydat (45°39'52.859''N; 2°59'11.943''E) surface water		vDNA, MDA	Illumina HiSeq	IDBA-UD		MT478539
**CruV-179**	3180	33.1%	Standard	Unisense	#N/A		Lake Tunsjøen (78°03.375′N; 13°40.313′E)	Aguirre de Cárcer et al., 2015	vDNA, MDA	Illumina HiSeq	IDBA-UD		PRJEB5265
**CruV-180**	3180	33.1%	Standard	Unisense	TAGTATTAC		unnamed Arctic pond (78°02.935′N; 13°41.973′E)	Aguirre de Cárcer et al., 2015	vDNA, MDA	Illumina HiSeq	IDBA-UD		PRJEB5265
**CruV-181**	3181	36.0%	Standard	Unisense	AACTATTAC		Lake Tunsjøen (78°03.375′N; 13°40.313′E)	Aguirre de Cárcer et al., 2015	vDNA, MDA	Illumina HiSeq	IDBA-UD		PRJEB5265
**CruV-182**	3199	29.5%	Standard	Unisense	AACTATTAC		Estuary benthic sediments (NZ)		vDNA, MDA	Illumina HiSeq	metaSPAdes		MT263565
**CruV-183**	3202	57.2%	Standard	Ambisense	GATTAATAT		Lake Mary (AZ)		vDNA, MDA	Illumina HiSeq	metaSPAdes		MT263566
**CruV-184**	3206	47.6%	Standard	Ambisense	CATTAATAT				vDNA, MDA	Illumina HiSeq	metaSPAdes		MT263533
**CruV-185**	3212	44.5%	Standard	Ambisense	CAATAATAT		unnamed Arctic pond (78°02.935′N; 13°41.973′E)	Aguirre de Cárcer et al., 2015	vDNA, MDA	Illumina HiSeq	IDBA-UD		PRJEB5265
**CruV-186**	3222	40.2%	Standard	Ambisense	CAGTATTAC		Lake Tenndammen (78°06.118′N; 15°02.024′E)	Aguirre de Cárcer et al., 2015	vDNA, MDA	Illumina HiSeq	IDBA-UD		PRJEB5265
**CruV-187**	3227	52.3%	Standard	Ambisense	CAGTATTAC		Lake Aydat (45°39'52.859''N; 2°59'11.943''E) surface water		vDNA, MDA	Illumina HiSeq	IDBA-UD		MT478538
**CruV-188**	3230	35.3%	Standard	Unisense	#N/A		Lake Aydat (45°39'52.859''N; 2°59'11.943''E) surface water		vDNA, MDA	Illumina HiSeq	IDBA-UD		MT478537
**CruV-189**	3230	35.7%	Standard	Unisense	TAGTATTAC		unnamed Arctic pond (78°02.935′N; 13°41.973′E)	Aguirre de Cárcer et al., 2015	vDNA, MDA	Illumina HiSeq	IDBA-UD		PRJEB5265
**CruV-190**	3241	31.7%	Standard	Unisense	#N/A		River (NZ)		vDNA, MDA	Illumina HiSeq	metaSPAdes		MT263567
**CruV-191**	3243	50.4%	Standard	Ambisense	TAATACTAC	Capsid protein-based clusters	Lake Tunsjøen (78°03.375′N; 13°40.313′E)	Aguirre de Cárcer et al., 2015	vDNA, MDA	Illumina HiSeq	IDBA-UD		PRJEB5265
**CruV-192**	3244	37.3%	Standard	Ambisense	#N/A		Lake Aydat (45°39'52.859''N; 2°59'11.943''E) surface water		vDNA, MDA	Illumina HiSeq	IDBA-UD		MT478536
**CruV-193**	3248	39.9%	Standard	Ambisense	CAATAATAA	Capsid protein-based clusters	Lake Aydat (45°39'52.859''N; 2°59'11.943''E) surface water		vDNA, MDA	Illumina HiSeq	IDBA-UD		MT478535
**CruV-194**	3250	42.6%	Standard	Unisense	#N/A		Lake Aydat (45°39'52.859''N; 2°59'11.943''E) surface water		vDNA, MDA	Illumina HiSeq	IDBA-UD		MT478534
**CruV-195**	3256	45.1%	Standard	Unisense	#N/A	Capsid protein-based clusters	Chirominidae (NZ)		vDNA, MDA	Illumina HiSeq	metaSPAdes		MT263568
**CruV-196**	3267	29.9%	Standard	Ambisense	#N/A		Lake Tenndammen (78°06.118′N; 15°02.024′E)	Aguirre de Cárcer et al., 2015	vDNA, MDA	Illumina HiSeq	IDBA-UD		PRJEB5265
**CruV-197**	3273	40.5%	Standard	Ambisense	TATTATTAC	Capsid protein-based clusters	Lake Aydat (45°39'52.859''N; 2°59'11.943''E) surface water		vDNA, MDA	Illumina HiSeq	IDBA-UD		MT478533
**CruV-198**	3279	43.5%	Standard	Ambisense	#N/A		unnamed Arctic pond (78°02.935′N; 13°41.973′E)	Aguirre de Cárcer et al., 2015	vDNA, MDA	Illumina HiSeq	IDBA-UD		PRJEB5265
**CruV-199**	3279	44.9%	Standard	Unisense	#N/A		Lake Aydat (45°39'52.859''N; 2°59'11.943''E) surface water		vDNA, MDA	Illumina HiSeq	IDBA-UD		MT478532
**CruV-200**	3282	34.0%	Standard	Unisense	#N/A		Lake Aydat (45°39'52.859''N; 2°59'11.943''E) surface water		vDNA, MDA	Illumina HiSeq	IDBA-UD		MT478531
**CruV-201**	3283	40.9%	Standard	Ambisense	TAATAATAG / TATTATTAC		unnamed Arctic pond (78°02.935′N; 13°41.973′E)	Aguirre de Cárcer et al., 2015	vDNA, MDA	Illumina HiSeq	IDBA-UD		PRJEB5265

CruV-202	3285	40.3%	Standard	Unisense	#N/A		Lake Aydat (45°39'52.859''N; 2°59'11.943''E) surface water		vDNA, MDA	Illumina HiSeq	IDBA-UD		MT478530
CruV-203	3299	48.3%	Standard	Ambisense	TAGTATTAC		unnamed Arctic pond (78°02.935′N; 13°41.973′E)	Aguirre de Cárcer et al., 2015	vDNA, MDA	Illumina HiSeq	IDBA-UD		PRJEB5265
CruV-204	3303	37.1%	Standard	Ambisense	#N/A		River (NZ)		vDNA, MDA	Illumina HiSeq	metaSPAdes		MT263569
CruV-205	3303	37.1%	Standard	Ambisense	TATAACTAG / CAGTATTAG		River (NZ)		vDNA, MDA	Illumina HiSeq	metaSPAdes		MT263570
CruV-206	3304	45.1%	Standard	Ambisense	#N/A		Lake Aydat (45°39'52.859''N; 2°59'11.943''E) surface water		vDNA, MDA	Illumina HiSeq	IDBA-UD		MT478529
CruV-207	3304	35.8%	Standard	Unisense	#N/A	Capsid protein-based clusters	River sediments (NZ)		vDNA, MDA	Illumina HiSeq	metaSPAdes		MT263571
CruV-208	3306	42.4%	Standard	Unisense	#N/A		Lake Tunsjøen (78°03.375′N; 13°40.313′E)	Aguirre de Cárcer et al., 2015	vDNA, MDA	Illumina HiSeq	IDBA-UD		PRJEB5265
CruV-209	3309	45.4%	Standard	Ambisense	#N/A		Lake Tenndammen (78°06.118′N; 15°02.024′E)	Aguirre de Cárcer et al., 2015	vDNA, MDA	Illumina HiSeq	IDBA-UD		PRJEB5265
CruV-210	3310	39.1%	Standard	Ambisense	TATTATTAC		Lake Aydat (45°39'52.859''N; 2°59'11.943''E) surface water		vDNA, MDA	Illumina HiSeq	IDBA-UD		MT478528
CruV-211	3310	38.8%	Standard	Ambisense	#N/A	Rep-based clusters	River (NZ)		vDNA, MDA	Illumina HiSeq	metaSPAdes		MT263572
CruV-212	3311	34.0%	Standard	Unisense	TATTATTAC		River (NZ)		vDNA, MDA	Illumina HiSeq	metaSPAdes		MT263573
CruV-213	3314	49.7%	Standard	Ambisense	TATTATTAC		unnamed Arctic pond (78°02.935′N; 13°41.973′E)	Aguirre de Cárcer et al., 2015	vDNA, MDA	Illumina HiSeq	IDBA-UD		PRJEB5265
CruV-214	3316	41.5%	Standard	Ambisense	TAGTATTAC		Lake Nordammen (78°38.279′N; 16°44.025′E)	Aguirre de Cárcer et al., 2015	vDNA, MDA	Illumina HiSeq	IDBA-UD		PRJEB5265
CruV-215	3326	54.0%	Standard	Unisense	TAATATTAC	Rep-based clusters	Lake Tenndammen (78°06.118′N; 15°02.024′E)	Aguirre de Cárcer et al., 2015	vDNA, MDA	Illumina HiSeq	IDBA-UD		PRJEB5265
CruV-216	3330	30.6%	Standard	Unisense	CAGTATTAC		unnamed Arctic pond (78°02.935′N; 13°41.973′E)	Aguirre de Cárcer et al., 2015	vDNA, MDA	Illumina HiSeq	IDBA-UD		PRJEB5265
CruV-217	3333	51.8%	Standard	Ambisense	...G	Rep-based clusters	Lake Aydat (45°39'52.859''N; 2°59'11.943''E) surface water		vDNA, MDA	Illumina HiSeq	IDBA-UD		MT478527
CruV-218	3344	31.8%	Standard	Ambisense	TATTTAAAT		Lake Aydat (45°39'52.859''N; 2°59'11.943''E) surface water		vDNA, MDA	Illumina HiSeq	IDBA-UD		MT478526
CruV-219	3344	40.1%	Standard	Unisense	#N/A		Lake Aydat (45°39'52.859''N; 2°59'11.943''E) surface water		vDNA, MDA	Illumina HiSeq	IDBA-UD		MT478525
CruV-220	3344	40.2%	Standard	Ambisense	#N/A	Rep-based clusters	Lake Nordammen (78°38.279′N; 16°44.025′E)	Aguirre de Cárcer et al., 2015	vDNA, MDA	Illumina HiSeq	IDBA-UD		PRJEB5265
CruV-221	3350	35.0%	Standard	Unisense	#N/A		unnamed Arctic pond (78°02.935′N; 13°41.973′E)	Aguirre de Cárcer et al., 2015	vDNA, MDA	Illumina HiSeq	IDBA-UD		PRJEB5265
CruV-222	3352	47.7%	Standard	Ambisense	TAAAGATAT	Rep-based clusters	Lake Aydat (45°39'52.859''N; 2°59'11.943''E) surface water		vDNA, MDA	Illumina HiSeq	IDBA-UD		MT478524
CruV-223	3354	44.9%	Standard	Ambisense	TAAAGTTAT		unnamed Arctic pond (78°02.935′N; 13°41.973′E)	Aguirre de Cárcer et al., 2015	vDNA, MDA	Illumina HiSeq	IDBA-UD		PRJEB5265
CruV-224	3359	43.8%	Standard	Ambisense	TAGTATTAC		River bank soil (NZ)		vDNA, MDA	Illumina HiSeq	metaSPAdes		MT263574
CruV-225	3362	36.2%	Standard	Unisense	#N/A		Lake Pavin (45°29'45.11''N; 2°53'14.60''E), sampling depth = 22 meters		vDNA, MDA	Illumina HiSeq	IDBA-UD		MT478523
CruV-226	3362	35.8%	Standard	Ambisense	#N/A	Capsid protein-based clusters	unnamed Arctic pond (78°02.935′N; 13°41.973′E)	Aguirre de Cárcer et al., 2015	vDNA, MDA	Illumina HiSeq	IDBA-UD		PRJEB5265
CruV-227	3365	42.5%	Standard	Unisense	#N/A		unnamed Arctic pond (78°02.935′N; 13°41.973′E)	Aguirre de Cárcer et al., 2015	vDNA, MDA	Illumina HiSeq	IDBA-UD		PRJEB5265
CruV-228	3369	37.8%	Standard	Ambisense	#N/A		Lake Tunsjøen (78°03.375′N; 13°40.313′E)	Aguirre de Cárcer et al., 2015	vDNA, MDA	Illumina HiSeq	IDBA-UD		PRJEB5265
CruV-229	3372	34.4%	Standard	Unisense	#N/A		Lake Aydat (45°39'52.859''N; 2°59'11.943''E) surface water		vDNA, MDA	Illumina HiSeq	IDBA-UD		MT478522
CruV-230	3373	46.8%	Standard	Ambisense	CAATATTAC		Lake Aydat (45°39'52.859''N; 2°59'11.943''E) surface water		vDNA, MDA	Illumina HiSeq	IDBA-UD		MT478521
CruV-231	3375	48.8%	Standard	Ambisense	TATTATTAA		Lake Aydat (45°39'52.859''N; 2°59'11.943''E) surface water		vDNA, MDA	Illumina HiSeq	IDBA-UD		MT478520
CruV-232	3383	49.6%	Standard	Ambisense	TAGTATTAC		Lake Tenndammen (78°06.118′N; 15°02.024′E)	Aguirre de Cárcer et al., 2015	vDNA, MDA	Illumina HiSeq	IDBA-UD		PRJEB5265
CruV-233	3384	42.2%	Standard	Ambisense	TATTATTAC	Capsid protein- and Rep-based clusters	Lake Aydat (45°39'52.859''N; 2°59'11.943''E) surface water		vDNA, MDA	Illumina HiSeq	IDBA-UD		MT478519
CruV-234	3385	37.6%	Standard	Ambisense	AAATAATAT	Rep-based clusters	River (NZ)		vDNA, MDA	Illumina HiSeq	metaSPAdes		MT263575
CruV-235	3386	48.3%	Standard	Ambisense	TACTATTAC	Rep-based clusters	Lake Aydat (45°39'52.859''N; 2°59'11.943''E) surface water		vDNA, MDA	Illumina HiSeq	IDBA-UD		MT478518
CruV-236	3391	37.0%	Standard	Ambisense	GATTATTAT		unnamed Arctic pond (78°02.935′N; 13°41.973′E)	Aguirre de Cárcer et al., 2015	vDNA, MDA	Illumina HiSeq	IDBA-UD		PRJEB5265
CruV-237	3394	48.1%	Standard	Ambisense	#N/A		Lake Tenndammen (78°06.118′N; 15°02.024′E)	Aguirre de Cárcer et al., 2015	vDNA, MDA	Illumina HiSeq	IDBA-UD		PRJEB5265
CruV-238	3396	39.5%	Standard	Unisense	#N/A		unnamed Arctic pond (78°02.935′N; 13°41.973′E)	Aguirre de Cárcer et al., 2015	vDNA, MDA	Illumina HiSeq	IDBA-UD		PRJEB5265
CruV-239	3398	39.6%	Standard	Unisense	CAGTATTAC		Lake Tunsjøen (78°03.375′N; 13°40.313′E)	Aguirre de Cárcer et al., 2015	vDNA, MDA	Illumina HiSeq	IDBA-UD		PRJEB5265
CruV-240	3400	35.8%	Standard	Ambisense	#N/A		Arabidopsis rhizosphere microbial communities from the University of North Carolina	Lundberg et al. 2012	eDNA	Illumina HiSeq	MEGAHIT v1.0.6		PRJNA336851

**CruV-241**	3402	40.6%	Standard	Ambisense	TATTAGTAA / TACTAATAA		River (NZ)		vDNA, MDA	Illumina HiSeq	metaSPAdes		MT263576
**CruV-242**	3403	45.5%	Standard	Ambisense	AATTATTAC	Capsid protein-based clusters	Lake Nordammen (78°38.279′N; 16°44.025′E)	Aguirre de Cárcer et al., 2015	vDNA, MDA	Illumina HiSeq	IDBA-UD		PRJEB5265
**CruV-243**	3405	43.5%	Standard	Ambisense	TAGTATTAC	Capsid protein-based clusters	River (NZ)		vDNA, MDA	Illumina HiSeq	metaSPAdes		MT263577
**CruV-244**	3405	42.4%	Standard	Unisense	CACTAATAT	Capsid protein-based clusters	unnamed Arctic pond (78°02.935′N; 13°41.973′E)	Aguirre de Cárcer et al., 2015	vDNA, MDA	Illumina HiSeq	IDBA-UD		PRJEB5265
**CruV-245**	3406	43.5%	Standard	Ambisense	#N/A		River (NZ)		vDNA, MDA	Illumina HiSeq	metaSPAdes		MT263578
**CruV-246**	3408	37.8%	Standard	Ambisense			Lake Tenndammen (78°06.118′N; 15°02.024′E)	Aguirre de Cárcer et al., 2015	vDNA, MDA	Illumina HiSeq	IDBA-UD		PRJEB5265
**CruV-247**	3413	43.2%	Standard	Ambisense	TAGTATTAC		Lake Aydat (45°39'52.859''N; 2°59'11.943''E) surface water		vDNA, MDA	Illumina HiSeq	IDBA-UD		MT478517
**CruV-248**	3413	36.0%	Standard	Ambisense	#N/A		Lake Linnevatnet (78°03.864′N; 13°46.308′E)	Aguirre de Cárcer et al., 2015	vDNA, MDA	Illumina HiSeq	IDBA-UD		PRJEB5265
**CruV-249**	3419	38.6%	Standard	Unisense	#N/A		Lake Tenndammen (78°06.118′N; 15°02.024′E)	Aguirre de Cárcer et al., 2015	vDNA, MDA	Illumina HiSeq	IDBA-UD		PRJEB5265
**CruV-250**	3420	47.2%	Standard	Ambisense	#N/A		River (NZ)		vDNA, MDA	Illumina HiSeq	metaSPAdes		MT263579
**CruV-251**	3420	47.2%	Standard	Ambisense	#N/A		River bank soil (NZ)		vDNA, MDA	Illumina HiSeq	metaSPAdes		MT263580
**CruV-252**	3423	47.0%	Standard	Ambisense	#N/A	Rep-based clusters	unnamed Arctic pond (78°02.935′N; 13°41.973′E)	Aguirre de Cárcer et al., 2015	vDNA, MDA	Illumina HiSeq	IDBA-UD		PRJEB5265
**CruV-253**	3424	45.3%	Standard	Ambisense	#N/A	Capsid protein-and Rep-based clusters	Lake Aydat (45°39'52.859''N; 2°59'11.943''E) surface water		vDNA, MDA	Illumina HiSeq	IDBA-UD		MT478516
**CruV-254**	3425	47.4%	Standard	Ambisense	#N/A	Capsid protein-and Rep-based clusters	Lake Tenndammen (78°06.118′N; 15°02.024′E)	Aguirre de Cárcer et al., 2015	vDNA, MDA	Illumina HiSeq	IDBA-UD		PRJEB5265
**CruV-255**	3426	37.2%	Standard	Ambisense	GATTATTAT		Sewage Oxydation Pond (NZ)		vDNA, MDA	Illumina HiSeq	metaSPAdes		MT263581
**CruV-256**	3426	51.3%	Standard	Unisense	GAATAATAA	Rep-based clusters	Lake Aydat (45°39'52.859''N; 2°59'11.943''E) surface water		vDNA, MDA	Illumina HiSeq	IDBA-UD		MT478515
**CruV-257**	3434	49.8%	Standard	Ambisense	TACTATTAA / TAATAGTAT		Lake Nordammen (78°38.279′N; 16°44.025′E)	Aguirre de Cárcer et al., 2015	vDNA, MDA	Illumina HiSeq	IDBA-UD		PRJEB5265
**CruV-258**	3435	37.4%	Standard	Unisense	TACTATTAA / TAATAGTAT	Capsid protein-based clusters	unnamed Arctic pond (78°02.935′N; 13°41.973′E)	Aguirre de Cárcer et al., 2015	vDNA, MDA	Illumina HiSeq	IDBA-UD		PRJEB5265
**CruV-259**	3437	26.6%	Ciliate	Unisense	TAATAATAT		Lake Aydat (45°39'52.859''N; 2°59'11.943''E) surface water		vDNA, MDA	Illumina HiSeq	IDBA-UD		MT478514
**CruV-260**	3439	34.2%	Standard	Ambisense	AATTATTAT		Lake Aydat (45°39'52.859''N; 2°59'11.943''E) surface water		vDNA, MDA	Illumina HiSeq	IDBA-UD		MT478513
**CruV-261**	3444	38.1%	Standard	Unisense	#N/A		Lake Aydat (45°39'52.859''N; 2°59'11.943''E) surface water		vDNA, MDA	Illumina HiSeq	IDBA-UD		MT478512
**CruV-262**	3447	47.5%	Standard	Ambisense	GAATAATAA	Capsid protein-and Rep-based clusters	unnamed Arctic pond (78°02.935′N; 13°41.973′E)	Aguirre de Cárcer et al., 2015	vDNA, MDA	Illumina HiSeq	IDBA-UD		PRJEB5265
**CruV-263**	3451	33.7%	Standard	Ambisense	TATAGTAAC		Lake Aydat (45°39'52.859''N; 2°59'11.943''E) surface water		vDNA, MDA	Illumina HiSeq	IDBA-UD		MT478511
**CruV-264**	3454	43.2%	Standard	Ambisense	#N/A		River (NZ)		vDNA, MDA	Illumina HiSeq	metaSPAdes		MT263582
**CruV-265**	3457	48.7%	Standard	Ambisense	TATAGTAAC		River sediments (NZ)		vDNA, MDA	Illumina HiSeq	metaSPAdes		MT263583
**CruV-266**	3458	48.6%	Standard	Ambisense	#N/A		River sediments (NZ)		vDNA, MDA	Illumina HiSeq	metaSPAdes		MT263584
**CruV-267**	3465	50.3%	Standard	Ambisense	CAGTATTAC		Lake Aydat (45°39'52.859''N; 2°59'11.943''E) surface water		vDNA, MDA	Illumina HiSeq	IDBA-UD		MT478510
**CruV-268**	3470	61.5%	Standard	Ambisense	#N/A		Lake Nordammen (78°38.279′N; 16°44.025′E)	Aguirre de Cárcer et al., 2015	vDNA, MDA	Illumina HiSeq	IDBA-UD		PRJEB5265
**CruV-269**	3475	38.7%	Standard	Unisense	TAGTATTAC		unnamed Arctic pond (78°02.935′N; 13°41.973′E)	Aguirre de Cárcer et al., 2015	vDNA, MDA	Illumina HiSeq	IDBA-UD		PRJEB5265
**CruV-270**	3477	37.5%	Standard	Ambisense	TAGTATTAC		Lake Tenndammen (78°06.118′N; 15°02.024′E)	Aguirre de Cárcer et al., 2015	vDNA, MDA	Illumina HiSeq	IDBA-UD		PRJEB5265
**CruV-271**	3479	36.4%	Standard	Unisense	TAGTATTAC		unnamed Arctic pond (78°02.935′N; 13°41.973′E)	Aguirre de Cárcer et al., 2015	vDNA, MDA	Illumina HiSeq	IDBA-UD		PRJEB5265
**CruV-272**	3479	48.3%	Standard	Ambisense	#N/A		Dragonfly larvae (NZ)		vDNA, MDA	Illumina HiSeq	metaSPAdes		MT263585
**CruV-273**	3485	50.2%	Standard	Unisense	#N/A	Rep-based clusters	Lake Aydat (45°39'52.859''N; 2°59'11.943''E) surface water		vDNA, MDA	Illumina HiSeq	IDBA-UD		MT478509
**CruV-274**	3485	36.2%	Standard	Unisense	#N/A		unnamed Arctic pond (78°02.935′N; 13°41.973′E)	Aguirre de Cárcer et al., 2015	vDNA, MDA	Illumina HiSeq	IDBA-UD		PRJEB5265
**CruV-275**	3497	40.6%	Standard	Ambisense	#N/A		South Island Robin feces (NZ)		vDNA, MDA	Illumina HiSeq	metaSPAdes		MT263586
**CruV-276**	3498	43.0%	Standard	Ambisense	TAGTATTAC		River (NZ)		vDNA, MDA	Illumina HiSeq	metaSPAdes		MT263587
**CruV-277**	3499	48.2%	Standard	Ambisense	AAATATTAA / AATTAATAT		River (NZ)		vDNA, MDA	Illumina HiSeq	metaSPAdes		MT263588
**CruV-278**	3500	42.9%	Standard	Ambisense	#N/A		River (NZ)		vDNA, MDA	Illumina HiSeq	metaSPAdes		MT263589
**CruV-279**	3501	42.9%	Standard	Unisense	#N/A		unnamed Arctic pond (78°02.935′N; 13°41.973′E)	Aguirre de Cárcer et al., 2015	vDNA, MDA	Illumina HiSeq	IDBA-UD	Spliced Rep	PRJEB5265
**CruV-280**	3507	44.1%	Standard	Ambisense	TAGTATTAC / TAATACTAG	Capsid protein-and Rep-based clusters	Lake Aydat (45°39'52.859''N; 2°59'11.943''E) surface water		vDNA, MDA	Illumina HiSeq	IDBA-UD		MT478508
**CruV-281**	3508	48.2%	Standard	Ambisense	TAGTATTAC		Blood worms (NZ)		vDNA, MDA	Illumina HiSeq	metaSPAdes		MT263590
**CruV-282**	3509	48.2%	Standard	Ambisense	TATTTCTAA		River (NZ)		vDNA, MDA	Illumina HiSeq	metaSPAdes		MT263591

CruV-283	3511	45.1%	Standard	Ambisense	TAATGTTAA		Lake Aydat (45°39'52.859''N; 2°59'11.943''E) surface water		vDNA, MDA	Illumina HiSeq	IDBA-UD		MT478507
CruV-284	3515	36.1%	Standard	Unisense	#N/A		unnamed Arctic pond (78°02.935′N; 13°41.973′E)	Aguirre de Cárcer et al., 2015	vDNA, MDA	Illumina HiSeq	IDBA-UD		PRJEB5265
CruV-285	3517	47.8%	Standard	Ambisense	CAATATTAC	Capsid protein-based clusters	Lake Aydat (45°39'52.859''N; 2°59'11.943''E) surface water		vDNA, MDA	Illumina HiSeq	IDBA-UD		MT478506
CruV-286	3518	34.8%	Standard	Unisense	TATAGTAAC		Lake Pavin (45°29'45.11''N; 2°53'14.60''E), sampling depth = 22 meters		vDNA, MDA	Illumina HiSeq	IDBA-UD		MT478505
CruV-287	3521	38.9%	Standard	Unisense	TAGTATTAC		Lake Tunsjøen (78°03.375′N; 13°40.313′E)	Aguirre de Cárcer et al., 2015	vDNA, MDA	Illumina HiSeq	IDBA-UD		PRJEB5265
CruV-288	3526	41.7%	Standard	Unisense	TAGTATTAC		Lake Aydat (45°39'52.859''N; 2°59'11.943''E) surface water		vDNA, MDA	Illumina HiSeq	IDBA-UD		MT478504
CruV-289	3536	46.1%	Standard	Ambisense	TACTATTAC	Rep-based clusters	Lake Aydat (45°39'52.859''N; 2°59'11.943''E) surface water		vDNA, MDA	Illumina HiSeq	IDBA-UD		MT478503
CruV-290	3538	50.9%	Standard	Ambisense	#N/A		Lake Aydat (45°39'52.859''N; 2°59'11.943''E) surface water		vDNA, MDA	Illumina HiSeq	IDBA-UD		MT478502
CruV-291	3538	48.6%	Standard	Ambisense	#N/A		River (NZ)		vDNA, MDA	Illumina HiSeq	metaSPAdes		MT263592
CruV-292	3538	45.1%	Standard	Ambisense	#N/A	Rep-based clusters	Lake Aydat (45°39'52.859''N; 2°59'11.943''E) surface water		vDNA, MDA	Illumina HiSeq	IDBA-UD		MT478501
CruV-293	3545	45.8%	Standard	Ambisense	TATAACTAG		Odonatan larvae (NZ)		vDNA, MDA	Illumina HiSeq	metaSPAdes		MT263593
CruV-294	3546	39.8%	Standard	Unisense	#N/A	Capsid protein-based clusters	Lake Aydat (45°39'52.859''N; 2°59'11.943''E) surface water		vDNA, MDA	Illumina HiSeq	IDBA-UD		MT478500
CruV-295	3548	52.3%	Standard	Ambisense	#N/A		Sewage Oxydation Pond (NZ)		vDNA, MDA	Illumina HiSeq	metaSPAdes		MT263594
CruV-296	3549	51.2%	Standard	Unisense	TATAAATAC		Lake Tunsjøen (78°03.375′N; 13°40.313′E)	Aguirre de Cárcer et al., 2015	vDNA, MDA	Illumina HiSeq	IDBA-UD		PRJEB5265
CruV-297	3549	40.1%	Standard	Ambisense	AAGTATTAT	Rep-based clusters	unnamed Arctic pond (78°02.935′N; 13°41.973′E)	Aguirre de Cárcer et al., 2015	vDNA, MDA	Illumina HiSeq	IDBA-UD		PRJEB5265
CruV-298	3551	43.6%	Standard	Ambisense	TAGTATTAC	Capsid protein-based clusters	Lake Mary (AZ)		vDNA, MDA	Illumina HiSeq	metaSPAdes		MT263595
CruV-299	3552	47.1%	Standard	Ambisense	AACTAGTAT	Capsid protein-based clusters	unnamed Arctic pond (78°02.935′N; 13°41.973′E)	Aguirre de Cárcer et al., 2015	vDNA, MDA	Illumina HiSeq	IDBA-UD		PRJEB5265
CruV-300	3558	38.2%	Standard	Ambisense	TATTGTTAC		Arabidopsis rhizosphere microbial communities from the University of North Carolina	Lundberg et al. 2012	eDNA	Illumina GAIIx	MEGAHIT v1.0.6		PRJNA336798
CruV-301	3560	46.0%	Standard	Unisense	AACTACTAT	Rep-based clusters	Lake Aydat (45°39'52.859''N; 2°59'11.943''E) surface water		vDNA, MDA	Illumina HiSeq	IDBA-UD		MT478499
CruV-302	3563	41.4%	Standard	Unisense	TATAGTAAC		Lake Nordammen (78°38.279′N; 16°44.025′E)	Aguirre de Cárcer et al., 2015	vDNA, MDA	Illumina HiSeq	IDBA-UD		PRJEB5265
CruV-303	3564	50.2%	Standard	Unisense	#N/A	Rep-based clusters	Lake ALake Aydat (45°39'52.859''N; 2°59'11.943''E) surface waterydat		vDNA, MDA	Illumina HiSeq	IDBA-UD		MT478498
CruV-304	3567	45.0%	Standard	Ambisense	TATAAATAC		River (NZ)		vDNA, MDA	Illumina HiSeq	metaSPAdes		MT263596
CruV-305	3569	47.1%	Standard	Unisense	AAGTATTAG	Rep-based clusters	River (NZ)		vDNA, MDA	Illumina HiSeq	metaSPAdes		MT263597
CruV-306	3576	53.0%	Standard	Unisense	#N/A	Capsid protein- and Rep-based clusters	River sediments (NZ)		vDNA, MDA	Illumina HiSeq	metaSPAdes		MT263598
CruV-307	3577	50.6%	Standard	Ambisense	TATAGTAAC		unnamed Arctic pond (78°02.935′N; 13°41.973′E)	Aguirre de Cárcer et al., 2015	vDNA, MDA	Illumina HiSeq	IDBA-UD		PRJEB5265
CruV-308	3578	40.8%	Standard	Ambisense	#N/A		Lake Aydat (45°39'52.859''N; 2°59'11.943''E) surface water		vDNA, MDA	Illumina HiSeq	IDBA-UD		MT478497
CruV-309	3584	46.6%	Standard	Unisense	#N/A	Rep-based clusters	River (NZ)		vDNA, MDA	Illumina HiSeq	metaSPAdes		MT263599
CruV-310	3584	46.6%	Standard	Unisense	#N/A	Rep-based clusters	River (NZ)		vDNA, MDA	Illumina HiSeq	metaSPAdes		MT263600
CruV-311	3584	46.6%	Standard	Unisense	#N/A	Rep-based clusters	River (NZ)		vDNA, MDA	Illumina HiSeq	metaSPAdes		MT263601
CruV-312	3593	35.8%	Standard	Ambisense	TAGTATTAC		River (NZ)		vDNA, MDA	Illumina HiSeq	metaSPAdes		MT263602
CruV-313	3594	46.8%	Standard	Unisense	TATAAATAC		Lake Tenndammen (78°06.118′N; 15°02.024′E)	Aguirre de Cárcer et al., 2015	vDNA, MDA	Illumina HiSeq	IDBA-UD		PRJEB5265
CruV-314	3597	49.0%	Standard	Unisense	TATAAATAC		Lake Nordammen (78°38.279′N; 16°44.025′E)	Aguirre de Cárcer et al., 2015	vDNA, MDA	Illumina HiSeq	IDBA-UD		PRJEB5265
CruV-315	3600	48.7%	Standard	Ambisense	#N/A	Capsid protein-based clusters	unnamed Arctic pond (78°02.935′N; 13°41.973′E)	Aguirre de Cárcer et al., 2015	vDNA, MDA	Illumina HiSeq	IDBA-UD		PRJEB5265
CruV-316	3603	42.4%	Standard	Unisense	GAATATTAT		Lake Aydat (45°39'52.859''N; 2°59'11.943''E) surface water		vDNA, MDA	Illumina HiSeq	IDBA-UD		MT478496
CruV-317	3611	26.1%	Standard	Unisense	#N/A		Lake Pavin (45°29'45.11''N; 2°53'14.60''E), sampling depth = 22 meters		vDNA, MDA	Illumina HiSeq	IDBA-UD		MT478495
CruV-318	3611	49.6%	Standard	Ambisense	#N/A	Capsid protein-based clusters	Lake Aydat (45°39'52.859''N; 2°59'11.943''E) surface water		vDNA, MDA	Illumina HiSeq	IDBA-UD		MT478494
CruV-319	3613	41.8%	Standard	Unisense	TAAAACAAA		Lake Tunsjøen (78°03.375′N; 13°40.313′E)	Aguirre de Cárcer et al., 2015	vDNA, MDA	Illumina HiSeq	IDBA-UD		PRJEB5265
CruV-320	3613	41.8%	Standard	Unisense	AACTATTAC		unnamed Arctic pond (78°02.935′N; 13°41.973′E)	Aguirre de Cárcer et al., 2015	vDNA, MDA	Illumina HiSeq	IDBA-UD		PRJEB5265

CruV-321	3614	47.6%	Standard	Ambisense	#N/A		Lake Aydat (45°39'52.859''N; 2°59'11.943''E) surface water		vDNA, MDA	Illumina HiSeq	IDBA-UD		MT478493
CruV-322	3616	46.1%	Standard	Unisense	TATTTAAAT		River (NZ)		vDNA, MDA	Illumina HiSeq	metaSPAdes		MT263603
CruV-323	3616	46.1%	Standard	Unisense	TATAAATAC		River (NZ)		vDNA, MDA	Illumina HiSeq	metaSPAdes		MT263604
CruV-324	3616	46.1%	Standard	Unisense	#N/A		River bank soil (NZ)		vDNA, MDA	Illumina HiSeq	metaSPAdes		MT263605
CruV-325	3619	30.3%	Standard	Unisense	#N/A		unnamed Arctic pond (78°02.935′N; 13°41.973′E)	Aguirre de Cárcer et al., 2015	vDNA, MDA	Illumina HiSeq	IDBA-UD		PRJEB5265
CruV-326	3620	44.0%	Standard	Ambisense	AATTATTAA		River bank soil (NZ)		vDNA, MDA	Illumina HiSeq	metaSPAdes		MT263606
CruV-327	3630	31.1%	Standard	Ambisense	TAATACTAC		Lake Nordammen (78°38.279′N; 16°44.025′E)	Aguirre de Cárcer et al., 2015	vDNA, MDA	Illumina HiSeq	IDBA-UD		PRJEB5265
CruV-328	3630	42.1%	Standard	Ambisense	#N/A	Rep-based clusters	unnamed Arctic pond (78°02.935′N; 13°41.973′E)	Aguirre de Cárcer et al., 2015	vDNA, MDA	Illumina HiSeq	IDBA-UD	Capsid ORF has smaller ORF in opposite orientation that has homology to Circo Cap	PRJEB5265
CruV-329	3635	40.2%	Standard	Ambisense	#N/A		unnamed Arctic pond (78°02.935′N; 13°41.973′E)	Aguirre de Cárcer et al., 2015	vDNA, MDA	Illumina HiSeq	IDBA-UD		PRJEB5265
CruV-330	3656	40.4%	Standard	Unisense	TAGTATTAC		unnamed Arctic pond (78°02.935′N; 13°41.973′E)	Aguirre de Cárcer et al., 2015	vDNA, MDA	Illumina HiSeq	IDBA-UD		PRJEB5265
CruV-331	3657	45.2%	Standard	Unisense	#N/A		Marine microbial communities from Delaware Coast		eDNA	Illumina HiSeq	SOAPdenovo, Newbler, Minimus2		PRJNA336828
CruV-332	3658	62.1%	Standard	Unisense	#N/A		Lake Nordammen (78°38.279′N; 16°44.025′E)	Aguirre de Cárcer et al., 2015	vDNA, MDA	Illumina HiSeq	IDBA-UD		PRJEB5265
CruV-333	3665	45.3%	Standard	Ambisense	TAATAATAT / TATTATTAG		Lake Aydat (45°39'52.859''N; 2°59'11.943''E) surface water		vDNA, MDA	Illumina HiSeq	IDBA-UD		MT478492
CruV-334	3666	35.4%	Standard	Ambisense	TAGTATTAC		Lake Aydat (45°39'52.859''N; 2°59'11.943''E) surface water		vDNA, MDA	Illumina HiSeq	IDBA-UD		MT478491
CruV-335	3667	33.0%	Standard	Ambisense	TATAGATAA		unnamed Arctic pond (78°02.935′N; 13°41.973′E)	Aguirre de Cárcer et al., 2015	vDNA, MDA	Illumina HiSeq	IDBA-UD		PRJEB5265
CruV-336	3676	43.5%	Standard	Unisense	#N/A	Capsid protein-based clusters	unnamed Arctic pond (78°02.935′N; 13°41.973′E)	Aguirre de Cárcer et al., 2015	vDNA, MDA	Illumina HiSeq	IDBA-UD		PRJEB5265
CruV-337	3682	37.5%	Standard	Unisense	#N/A		Lake Tunsjøen (78°03.375′N; 13°40.313′E)	Aguirre de Cárcer et al., 2015	vDNA, MDA	Illumina HiSeq	IDBA-UD		PRJEB5265
CruV-338	3686	36.1%	Standard	Ambisense	TAGTATTAC		Host-associated microbial communities from peat moss Sphagnum species from Minnesota, USA		eDNA	Illumina HiSeq	MEGAHIT v1.0.3		PRJNA364930
CruV-339	3691	31.3%	Standard	Ambisense	CAATATTAG		River (NZ)		vDNA, MDA	Illumina HiSeq	metaSPAdes		MT263607
CruV-340	3708	38.0%	Standard	Ambisense	TAATAAAAT	Capsid protein-based clusters	River (NZ)		vDNA, MDA	Illumina HiSeq	metaSPAdes	Spliced Rep	MT263608
CruV-341	3708	32.6%	Standard	Ambisense	TAATATTAC		unnamed Arctic pond (78°02.935′N; 13°41.973′E)	Aguirre de Cárcer et al., 2015	vDNA, MDA	Illumina HiSeq	IDBA-UD		PRJEB5265
CruV-342	3709	32.9%	Standard	Ambisense	TAGTATTAC		unnamed Arctic pond (78°02.935′N; 13°41.973′E)	Aguirre de Cárcer et al., 2015	vDNA, MDA	Illumina HiSeq	IDBA-UD		PRJEB5265
CruV-343	3719	49.7%	Standard	Ambisense	AATTATTAA	Rep-based clusters	Lake Aydat (45°39'52.859''N; 2°59'11.943''E) surface water		vDNA, MDA	Illumina HiSeq	IDBA-UD		MT478490
CruV-344	3726	37.5%	Standard	Unisense	TAGTATTAC		unnamed Arctic pond (78°02.935′N; 13°41.973′E)	Aguirre de Cárcer et al., 2015	vDNA, MDA	Illumina HiSeq	IDBA-UD		PRJEB5265
CruV-345	3728	41.0%	Standard	Ambisense	TAGTATTAC		River biofilm (NZ)		vDNA, MDA	Illumina HiSeq	metaSPAdes		MT263609
CruV-346	3749	40.3%	Standard	Unisense	#N/A		unnamed Arctic pond (78°02.935′N; 13°41.973′E)	Aguirre de Cárcer et al., 2015	vDNA, MDA	Illumina HiSeq	IDBA-UD	Spliced Rep	PRJEB5265
CruV-347	3766	38.4%	Standard	Unisense	TAATAGTAG / TACTATTAC		unnamed Arctic pond (78°02.935′N; 13°41.973′E)	Aguirre de Cárcer et al., 2015	vDNA, MDA	Illumina HiSeq	IDBA-UD	Spliced Rep	PRJEB5265
CruV-348	3769	41.2%	Standard	Ambisense	#N/A	Capsid protein-based clusters	Lake Tenndammen (78°06.118′N; 15°02.024′E)	Aguirre de Cárcer et al., 2015	vDNA, MDA	Illumina HiSeq	IDBA-UD		PRJEB5265
CruV-349	3770	32.3%	Standard	Ambisense	#N/A		River (NZ)		vDNA, MDA	Illumina HiSeq	metaSPAdes		MT263610
CruV-350	3777	47.8%	Standard	Ambisense	TATT...		River (NZ)		vDNA, MDA	Illumina HiSeq	metaSPAdes		MT263611
CruV-351	3786	31.3%	Standard	Ambisense	#N/A		Lake Nordammen (78°38.279′N; 16°44.025′E)	Aguirre de Cárcer et al., 2015	vDNA, MDA	Illumina HiSeq	IDBA-UD		PRJEB5265
CruV-352	3790	34.9%	Standard	Ambisense	#N/A		Lake Tunsjøen (78°03.375′N; 13°40.313′E)	Aguirre de Cárcer et al., 2015	vDNA, MDA	Illumina HiSeq	IDBA-UD		PRJEB5265
CruV-353	3791	32.1%	Standard	Ambisense	TACTATTAC		River (NZ)		vDNA, MDA	Illumina HiSeq	metaSPAdes		MT263612
CruV-354	3800	44.8%	Standard	Ambisense	TACTATTAC		unnamed Arctic pond (78°02.935′N; 13°41.973′E)	Aguirre de Cárcer et al., 2015	vDNA, MDA	Illumina HiSeq	IDBA-UD		PRJEB5265
CruV-355	3807	43.2%	Standard	Unisense	TATTATTAC		unnamed Arctic pond (78°02.935′N; 13°41.973′E)	Aguirre de Cárcer et al., 2015	vDNA, MDA	Illumina HiSeq	IDBA-UD		PRJEB5265
CruV-356	3823	47.4%	Standard	Unisense	CAGTATTAC		unnamed Arctic pond (78°02.935′N; 13°41.973′E)	Aguirre de Cárcer et al., 2015	vDNA, MDA	Illumina HiSeq	IDBA-UD		PRJEB5265
CruV-357	3828	38.1%	Standard	Ambisense	CAGTATTAC		Lake Aydat (45°39'52.859''N; 2°59'11.943''E) surface water		vDNA, MDA	Illumina HiSeq	IDBA-UD		MT478489
CruV-358	3835	41.7%	Standard	Ambisense	CAGTATTAC		Lake Aydat (45°39'52.859''N; 2°59'11.943''E) surface water		vDNA, MDA	Illumina HiSeq	IDBA-UD		MT478488
CruV-359	3848	31.5%	Standard	Ambisense	TAATGATAA	Capsid protein-based clusters	River (NZ)		vDNA, MDA	Illumina HiSeq	metaSPAdes		MT263613

**CruV-360**	3852	41.8%	Standard	Ambisense	#N/A		Lake Tunsjøen (78°03.375′N; 13°40.313′E)	Aguirre de Cárcer et al., 2015	vDNA, MDA	Illumina HiSeq	IDBA-UD		PRJEB5265
**CruV-361**	3864	45.3%	Standard	Ambisense	#N/A	Rep-based clusters	Lake Tunsjøen (78°03.375′N; 13°40.313′E)	Aguirre de Cárcer et al., 2015	vDNA, MDA	Illumina HiSeq	IDBA-UD		PRJEB5265
**CruV-362**	3866	37.3%	Standard	Ambisense	TAAAATAAA		River (NZ)		vDNA, MDA	Illumina HiSeq	metaSPAdes		MT263614
**CruV-363**	3875	33.0%	Standard	Ambisense	TAGTATTAC		unnamed Arctic pond (78°02.935′N; 13°41.973′E)	Aguirre de Cárcer et al., 2015	vDNA, MDA	Illumina HiSeq	IDBA-UD		PRJEB5265
**CruV-364**	3876	49.6%	Standard	Ambisense	#N/A		River (NZ)		vDNA, MDA	Illumina HiSeq	metaSPAdes		MT263615
**CruV-365**	3879	45.9%	Standard	Ambisense	TATTTATAC / TATAAATAG / CAGTGTTAC		unnamed Arctic pond (78°02.935′N; 13°41.973′E)	Aguirre de Cárcer et al., 2015	vDNA, MDA	Illumina HiSeq	IDBA-UD		PRJEB5265
**CruV-366**	3882	48.8%	Standard	Ambisense	#N/A		Lake Tunsjøen (78°03.375′N; 13°40.313′E)	Aguirre de Cárcer et al., 2015	vDNA, MDA	Illumina HiSeq	IDBA-UD		PRJEB5265
**CruV-367**	3899	44.1%	Standard	Ambisense	#N/A	Capsid protein-based clusters	Delisea pulchra microbial communities from Sydney, Australia, affected by bleaching disease	Zozaya-Valdés et al., 2017	eDNA	Illumina HiSeq	IDBA-UD + SOAPdenovo + GAA		Gp0060493 (GOLD)
**CruV-368**	3908	48.9%	Standard	Ambisense	TATTATTAC		Lake Aydat (45°39'52.859''N; 2°59'11.943''E) surface water		vDNA, MDA	Illumina HiSeq	IDBA-UD		MT478487
**CruV-369**	3929	49.2%	Standard	Ambisense	CATTATTAC		Lake Nordammen (78°38.279′N; 16°44.025′E)	Aguirre de Cárcer et al., 2015	vDNA, MDA	Illumina HiSeq	IDBA-UD		PRJEB5265
**CruV-370**	3948	47.9%	Standard	Ambisense	AACTATTAC		unnamed Arctic pond (78°02.935′N; 13°41.973′E)	Aguirre de Cárcer et al., 2015	vDNA, MDA	Illumina HiSeq	IDBA-UD	Spliced Rep	PRJEB5265
**CruV-371**	3970	51.1%	Standard	Unisense	TAATACTAA		River (NZ)		vDNA, MDA	Illumina HiSeq	metaSPAdes		MT263616
**CruV-372**	3974	51.0%	Standard	Unisense	#N/A	Rep-based clusters	River (NZ)		vDNA, MDA	Illumina HiSeq	metaSPAdes		MT263617
**CruV-373**	3981	49.2%	Standard	Ambisense	TAAAGATAC / TATTTCAAG	Rep-based clusters	River (NZ)		vDNA, MDA	Illumina HiSeq	metaSPAdes		MT263618
**CruV-374**	3994	46.6%	Standard	Unisense	#N/A		unnamed Arctic pond (78°02.935′N; 13°41.973′E)	Aguirre de Cárcer et al., 2015	vDNA, MDA	Illumina HiSeq	IDBA-UD		PRJEB5265
**CruV-375**	3998	34.1%	Standard	Ambisense	TATTATAAC		Lake Nordammen (78°38.279′N; 16°44.025′E)	Aguirre de Cárcer et al., 2015	vDNA, MDA	Illumina HiSeq	IDBA-UD		PRJEB5265
**CruV-376**	4026	50.1%	Standard	Ambisense	TATAGATAG / TATTAAAAT		Lake Aydat (45°39'52.859''N; 2°59'11.943''E) surface water		vDNA, MDA	Illumina HiSeq	IDBA-UD		MT478486
**CruV-377**	4037	40.2%	Standard	Ambisense	TAATATTAC	Rep-based clusters	unnamed Arctic pond (78°02.935′N; 13°41.973′E)	Aguirre de Cárcer et al., 2015	vDNA, MDA	Illumina HiSeq	IDBA-UD		PRJEB5265
**CruV-378**	4042	45.1%	Standard	Unisense	#N/A	Rep-based clusters	Lake Tunsjøen (78°03.375′N; 13°40.313′E)	Aguirre de Cárcer et al., 2015	vDNA, MDA	Illumina HiSeq	IDBA-UD		PRJEB5265
**CruV-379**	4049	54.0%	Standard	Unisense	#N/A	Rep-based clusters	unnamed Arctic pond (78°02.935′N; 13°41.973′E)	Aguirre de Cárcer et al., 2015	vDNA, MDA	Illumina HiSeq	IDBA-UD		PRJEB5265
**CruV-380**	4056	45.6%	Standard	Ambisense	CAGTATTAC		Tern feces (Canada)		vDNA, MDA	Illumina HiSeq	metaSPAdes		MT263619
**CruV-381**	4057	35.2%	Standard	Unisense	TAGTATTAC		Lake Tunsjøen (78°03.375′N; 13°40.313′E)	Aguirre de Cárcer et al., 2015	vDNA, MDA	Illumina HiSeq	IDBA-UD		PRJEB5265
**CruV-382**	4058	43.5%	Standard	Ambisense	#N/A		Lake Tenndammen (78°06.118′N; 15°02.024′E)	Aguirre de Cárcer et al., 2015	vDNA, MDA	Illumina HiSeq	IDBA-UD		PRJEB5265
**CruV-383**	4064	42.8%	Standard	Ambisense	#N/A		unnamed Arctic pond (78°02.935′N; 13°41.973′E)	Aguirre de Cárcer et al., 2015	vDNA, MDA	Illumina HiSeq	IDBA-UD		PRJEB5265
**CruV-384**	4065	48.1%	Standard	Unisense	TATATAAAA		Sewage Oxydation Pond (NZ)		vDNA, MDA	Illumina HiSeq	metaSPAdes		MT263620
**CruV-385**	4067	47.9%	Standard	Unisense	#N/A		Lake Pavin (45°29'45.11''N; 2°53'14.60''E), sampling depth = 22 meters		vDNA, MDA	Illumina HiSeq	IDBA-UD		MT478485
**CruV-386**	4072	49.2%	Standard	Unisense	TATAACAAC		Lake Nordammen (78°38.279′N; 16°44.025′E)	Aguirre de Cárcer et al., 2015	vDNA, MDA	Illumina HiSeq	IDBA-UD		PRJEB5265
**CruV-387**	4076	50.8%	Standard	Unisense	CAGTATTAC	Rep-based clusters	Chirominidae (NZ)		vDNA, MDA	Illumina HiSeq	metaSPAdes		MT263621
**CruV-388**	4077	34.9%	Standard	Ambisense	TAGTATTAC		Lake Nordammen (78°38.279′N; 16°44.025′E)	Aguirre de Cárcer et al., 2015	vDNA, MDA	Illumina HiSeq	IDBA-UD		PRJEB5265
**CruV-389**	4087	45.4%	Standard	Ambisense	TAGTATTAC		River (NZ)		vDNA, MDA	Illumina HiSeq	metaSPAdes		MT263622
**CruV-390**	4094	50.5%	Standard	Ambisense	TAGTATTAC		Lake Nordammen (78°38.279′N; 16°44.025′E)	Aguirre de Cárcer et al., 2015	vDNA, MDA	Illumina HiSeq	IDBA-UD		PRJEB5265
**CruV-391**	4115	49.1%	Standard	Ambisense	#N/A		Lake Aydat (45°39'52.859''N; 2°59'11.943''E) surface water		vDNA, MDA	Illumina HiSeq	IDBA-UD		MT478484
**CruV-392**	4132	45.8%	Standard	Ambisense	CATTAATAT		River (NZ)		vDNA, MDA	Illumina HiSeq	metaSPAdes		MT263623
**CruV-393**	4134	53.9%	Standard	Unisense	#N/A		Lake Aydat (45°39'52.859''N; 2°59'11.943''E) surface water		vDNA, MDA	Illumina HiSeq	IDBA-UD		MT478483
**CruV-394**	4137	45.6%	Standard	Ambisense	TAGTATTAC	Capsid protein-based clusters	unnamed Arctic pond (78°02.935′N; 13°41.973′E)	Aguirre de Cárcer et al., 2015	vDNA, MDA	Illumina HiSeq	IDBA-UD		PRJEB5265
**CruV-395**	4141	42.8%	Standard	Ambisense	#N/A		Lake Nordammen (78°38.279′N; 16°44.025′E)	Aguirre de Cárcer et al., 2015	vDNA, MDA	Illumina HiSeq	IDBA-UD		PRJEB5265
**CruV-396**	4153	49.7%	Standard	Unisense	#N/A		unnamed Arctic pond (78°02.935′N; 13°41.973′E)	Aguirre de Cárcer et al., 2015	vDNA, MDA	Illumina HiSeq	IDBA-UD		PRJEB5265
**CruV-397**	4158	42.5%	Standard	Unisense	TAGTATTAC		Lake Tunsjøen (78°03.375′N; 13°40.313′E)	Aguirre de Cárcer et al., 2015	vDNA, MDA	Illumina HiSeq	IDBA-UD		PRJEB5265
**CruV-398**	4169	40.9%	Standard	Ambisense	TATTATTAG / TAATAATAG		Lake Aydat (45°39'52.859''N; 2°59'11.943''E) surface water		vDNA, MDA	Illumina HiSeq	IDBA-UD		MT478482
**CruV-399**	4174	47.3%	Standard	Ambisense	CAGTATTAC	Rep-based clusters	unnamed Arctic pond (78°02.935′N; 13°41.973′E)	Aguirre de Cárcer et al., 2015	vDNA, MDA	Illumina HiSeq	IDBA-UD		PRJEB5265

CruV-400	4184	46.9%	Standard	Unisense	#N/A		Lake Aydat (45°39'52.859''N; 2°59'11.943''E) surface water		vDNA, MDA	Illumina HiSeq	IDBA-UD		MT478481
CruV-401	4185	42.3%	Standard	Ambisense	TAATAGTAA / CAATACTAA / AATTAATAT		unnamed Arctic pond (78°02.935′N; 13°41.973′E)	Aguirre de Cárcer et al., 2015	vDNA, MDA	Illumina HiSeq	IDBA-UD		PRJEB5265
CruV-402	4188	39.5%	Standard	Ambisense	TATATTTAT		River (NZ)		vDNA, MDA	Illumina HiSeq	metaSPAdes		MT263624
CruV-403	4191	50.1%	Standard	Ambisense	TAGTATTAC		unnamed Arctic pond (78°02.935′N; 13°41.973′E)	Aguirre de Cárcer et al., 2015	vDNA, MDA	Illumina HiSeq	IDBA-UD		PRJEB5265
CruV-404	4197	42.4%	Standard	Unisense	AAATAATAC		Lake Nordammen (78°38.279′N; 16°44.025′E)	Aguirre de Cárcer et al., 2015	vDNA, MDA	Illumina HiSeq	IDBA-UD		PRJEB5265
CruV-405	4209	37.6%	Standard	Unisense	AATTAGTAA		Lake Tunsjøen (78°03.375′N; 13°40.313′E)	Aguirre de Cárcer et al., 2015	vDNA, MDA	Illumina HiSeq	IDBA-UD		PRJEB5265
CruV-406	4212	45.8%	Standard	Ambisense	#N/A		River (NZ)		vDNA, MDA	Illumina HiSeq	metaSPAdes		MT263625
CruV-407	4212	45.8%	Standard	Ambisense	TAGTATTAC		River (NZ)		vDNA, MDA	Illumina HiSeq	metaSPAdes		MT263626
CruV-408	4213	35.9%	Standard	Ambisense	#N/A		Lake Aydat (45°39'52.859''N; 2°59'11.943''E) surface water		vDNA, MDA	Illumina HiSeq	IDBA-UD		MT478480
CruV-409	4223	39.4%	Standard	Ambisense	CAATACTAT		River sediments (NZ)		vDNA, MDA	Illumina HiSeq	metaSPAdes		MT263627
CruV-410	4227	39.4%	Standard	Ambisense	#N/A		River sediments (NZ)		vDNA, MDA	Illumina HiSeq	metaSPAdes		MT263628
CruV-411	4227	38.3%	Standard	Ambisense	TAATATTAC	Capsid protein-based clusters	River (NZ)		vDNA, MDA	Illumina HiSeq	metaSPAdes		MT263629
CruV-412	4231	53.8%	Standard	Unisense	TAATATTAC		Lake Pavin (45°29'45.11''N; 2°53'14.60''E), sampling depth = 22 meters		vDNA, MDA	Illumina HiSeq	IDBA-UD		MT478479
CruV-413	4234	41.9%	Standard	Unisense	#N/A		unnamed Arctic pond (78°02.935′N; 13°41973′E)	Aguirre de Cárcer et al., 2015	vDNA, MDA	Illumina HiSeq	IDBA-UD	Spliced Rep	PRJEB5265
CruV-414	4237	48.4%	Standard	Unisense	#N/A		Lake Tunsjøen (78°03.375′N; 13°40.313′E)	Aguirre de Cárcer et al., 2015	vDNA, MDA	Illumina HiSeq	IDBA-UD		PRJEB5265
CruV-415	4241	34.0%	Standard	Ambisense	TAATAGTAA / TACTATTAT		unnamed Arctic pond (78°02.935′N; 13°41.973′E)	Aguirre de Cárcer et al., 2015	vDNA, MDA	Illumina HiSeq	IDBA-UD		PRJEB5265
CruV-416	4249	53.9%	Standard	Unisense	#N/A		River (NZ)		vDNA, MDA	Illumina HiSeq	metaSPAdes		MT263630
CruV-417	4268	38.4%	Standard	Unisense	TAATGTTAC		unnamed Arctic pond (78°02.935′N; 13°41.973′E)	Aguirre de Cárcer et al., 2015	vDNA, MDA	Illumina HiSeq	IDBA-UD		PRJEB5265
CruV-418	4317	52.4%	Standard	Ambisense	#N/A		Lake Aydat (45°39'52.859''N; 2°59'11.943''E) surface water		vDNA, MDA	Illumina HiSeq	IDBA-UD		MT478478
CruV-419	4319	53.9%	Standard	Unisense	#N/A		Sewage Oxydation Pond (NZ)		vDNA, MDA	Illumina HiSeq	metaSPAdes		MT263631
CruV-420	4320	35.5%	Standard	Unisense	#N/A		unnamed Arctic pond (78°02.935′N; 13°41.973′E)	Aguirre de Cárcer et al., 2015	vDNA, MDA	Illumina HiSeq	IDBA-UD	Double Capsid protein	PRJEB5265
CruV-421	4329	38.4%	Standard	Unisense	AAATAGTAT	Rep-based clusters	unnamed Arctic pond (78°02.935′N; 13°41.973′E)	Aguirre de Cárcer et al., 2015	vDNA, MDA	Illumina HiSeq	IDBA-UD		PRJEB5265
CruV-422	4336	44.8%	Standard	Ambisense	CAGTATTAC	Capsid protein-based clusters	unnamed Arctic pond (78°02.935′N; 13°41.973′E)	Aguirre de Cárcer et al., 2015	vDNA, MDA	Illumina HiSeq	IDBA-UD		PRJEB5265
CruV-423	4344	43.9%	Standard	Ambisense	GATTATTAG	Rep-based clusters	unnamed Arctic pond (78°02.935′N; 13°41.973′E)	Aguirre de Cárcer et al., 2015	vDNA, MDA	Illumina HiSeq	IDBA-UD		PRJEB5265
CruV-424	4348	53.2%	Standard	Unisense	#N/A		unnamed Arctic pond (78°02.935′N; 13°41.973′E)	Aguirre de Cárcer et al., 2015	vDNA, MDA	Illumina HiSeq	IDBA-UD		PRJEB5265
CruV-425	4361	48.4%	Standard	Unisense	TAGTATTAC		Lake Nordammen (78°38.279′N; 16°44.025′E)	Aguirre de Cárcer et al., 2015	vDNA, MDA	Illumina HiSeq	IDBA-UD		PRJEB5265
CruV-426	4368	39.2%	Standard	Ambisense	#N/A		unnamed Arctic pond (78°02.935′N; 13°41.973′E)	Aguirre de Cárcer et al., 2015	vDNA, MDA	Illumina HiSeq	IDBA-UD	Spliced Rep	PRJEB5265
CruV-427	4371	50.3%	Standard	Unisense	CACTATTAC		River (NZ)		vDNA, MDA	Illumina HiSeq	metaSPAdes		MT263632
CruV-428	4379	53.2%	Standard	Unisense	AAATATTAA	Rep-based clusters	unnamed Arctic pond (78°02.935′N; 13°41.973′E)	Aguirre de Cárcer et al., 2015	vDNA, MDA	Illumina HiSeq	IDBA-UD		PRJEB5265
CruV-429	4383	50.6%	Standard	Ambisense	#N/A		River biofilm (NZ)		vDNA, MDA	Illumina HiSeq	metaSPAdes		MT263633
CruV-430	4383	41.1%	Standard	Ambisense	#N/A	Rep-based clusters	unnamed Arctic pond (78°02.935′N; 13°41.973′E)	Aguirre de Cárcer et al., 2015	vDNA, MDA	Illumina HiSeq	IDBA-UD		PRJEB5265
CruV-431	4384	44.8%	Standard	Ambisense	TAGTATTAC		Lake Nordammen (78°38.279′N; 16°44.025′E)	Aguirre de Cárcer et al., 2015	vDNA, MDA	Illumina HiSeq	IDBA-UD		PRJEB5265
CruV-432	4420	45.0%	Standard	Unisense	#N/A	Capsid protein-based clusters	Sewage Oxydation Pond (NZ)		vDNA, MDA	Illumina HiSeq	metaSPAdes		MT263634
CruV-433	4422	52.1%	Standard	Unisense	CACTAATAC		Lake Aydat (45°39'52.859''N; 2°59'11.943''E) surface water		vDNA, MDA	Illumina HiSeq	IDBA-UD		MT478477
CruV-434	4427	40.5%	Standard	Unisense	TAATTTTAC		Lake Tunsjøen (78°03.375′N; 13°40.313′E)	Aguirre de Cárcer et al., 2015	vDNA, MDA	Illumina HiSeq	IDBA-UD	Spliced Rep; Capsid contains an endomucin domain	PRJEB5265
CruV-435	4428	39.3%	Standard	Ambisense	#N/A	Rep-based clusters	unnamed Arctic pond (78°02.935′N; 13°41.973′E)	Aguirre de Cárcer et al., 2015	vDNA, MDA	Illumina HiSeq	IDBA-UD		PRJEB5265
CruCGE-436	4430	40.4%	Standard	Unisense	CATTAATAT			Aguirre de Cárcer et al., 2015	vDNA, MDA	Illumina HiSeq	IDBA-UD		PRJEB5265
CruV-437	4436	46.7%	Standard	Unisense	#N/A		unnamed Arctic pond (78°02.935′N; 13°41.973′E)	Aguirre de Cárcer et al., 2015	vDNA, MDA	Illumina HiSeq	IDBA-UD		PRJEB5265
CruV-438	4441	40.2%	Standard	Ambisense	AAATAATAT		Soil (NZ)		vDNA, MDA	Illumina HiSeq	metaSPAdes		MT263635
CruV-439	4447	44.8%	Standard	Unisense	GATTACTAT	Rep-based clusters	unnamed Arctic pond (78°02.935′N; 13°41.973′E)	Aguirre de Cárcer et al., 2015	vDNA, MDA	Illumina HiSeq	IDBA-UD		PRJEB5265
CruV-440	4448	50.4%	Standard	Unisense	#N/A		unnamed Arctic pond (78°02.935′N; 13°41.973′E)	Aguirre de Cárcer et al., 2015	vDNA, MDA	Illumina HiSeq	IDBA-UD		PRJEB5265

CruV-441	4451	46.1%	Standard	Ambisense	#N/A		unnamed Arctic pond (78°02.935′N; 13°41.973′E)	Aguirre de Cárcer et al., 2015	vDNA, MDA	Illumina HiSeq	IDBA-UD		PRJEB5265
CruV-442	4451	43.4%	Standard	Ambisense	TAGTATTAC		unnamed Arctic pond (78°02.935′N; 13°41.973′E)	Aguirre de Cárcer et al., 2015	vDNA, MDA	Illumina HiSeq	IDBA-UD		PRJEB5265
CruV-443	4452	37.1%	Standard	Ambisense	#N/A		Lake Nordammen (78°38.279′N; 16°44.025′E)	Aguirre de Cárcer et al., 2015	vDNA, MDA	Illumina HiSeq	IDBA-UD		PRJEB5265
CruV-444	4457	38.5%	Standard	Ambisense	TAGTATTAC		unnamed Arctic pond (78°02.935′N; 13°41.973′E)	Aguirre de Cárcer et al., 2015	vDNA, MDA	Illumina HiSeq	IDBA-UD		PRJEB5265
CruV-445	4457	40.3%	Standard	Ambisense	AAATACTAA		Lake Nordammen (78°38.279′N; 16°44.025′E)	Aguirre de Cárcer et al., 2015	vDNA, MDA	Illumina HiSeq	IDBA-UD		PRJEB5265
CruV-446	4459	44.7%	Standard	Unisense	#N/A	Capsid protein-based clusters	Lake Tunsjøen (78°03.375′N; 13°40.313′E)	Aguirre de Cárcer et al., 2015	vDNA, MDA	Illumina HiSeq	IDBA-UD		PRJEB5265
CruV-447	4462	48.7%	Standard	Ambisense	AAATATTAT		unnamed Arctic pond (78°02.935′N; 13°41.973′E)	Aguirre de Cárcer et al., 2015	vDNA, MDA	Illumina HiSeq	IDBA-UD		PRJEB5265
CruV-448	4464	42.7%	Standard	Unisense	#N/A		Lake Nordammen (78°38.279′N; 16°44.025′E)	Aguirre de Cárcer et al., 2015	vDNA, MDA	Illumina HiSeq	IDBA-UD		PRJEB5265
CruV-449	4482	38.5%	Standard	Unisense	#N/A		Lake Nordammen (78°38.279′N; 16°44.025′E)	Aguirre de Cárcer et al., 2015	vDNA, MDA	Illumina HiSeq	IDBA-UD		PRJEB5265
CruV-450	4485	44.9%	Standard	Unisense	TAAATAAAC / TAATATAAT		unnamed Arctic pond (78°02.935′N; 13°41.973′E)	Aguirre de Cárcer et al., 2015	vDNA, MDA	Illumina HiSeq	IDBA-UD	Spliced Rep	PRJEB5265
CruV-451	4487	37.7%	Standard	Ambisense	TAGTATTAC	Rep-based clusters	Lake Aydat (45°39'52.859''N; 2°59'11.943''E) surface water		vDNA, MDA	Illumina HiSeq	IDBA-UD		MT478476
CruV-452	4507	43.7%	Standard	Ambisense	TACTAGTAA / GACTAGTAT	Rep-based clusters	unnamed Arctic pond (78°02.935′N; 13°41.973′E)	Aguirre de Cárcer et al., 2015	vDNA, MDA	Illumina HiSeq	IDBA-UD		PRJEB5265
CruV-453	4520	44.9%	Standard	Unisense	TAATACTAG		Green lipped muscles (NZ)		vDNA, MDA	Illumina HiSeq	metaSPAdes		MT263534
CruV-454	4520	44.8%	Standard	Unisense	TATATAAAT		Green lipped muscles (NZ)		vDNA, MDA	Illumina HiSeq	metaSPAdes		MT263535
CruV-455	4520	44.8%	Standard	Unisense			Green lipped muscles (NZ)		vDNA, MDA	Illumina HiSeq	metaSPAdes		MT263536
CruV-456	4520	44.9%	Standard	Unisense	CAGTATTAC		Green lipped muscles (NZ)		vDNA, MDA	Illumina HiSeq	metaSPAdes		MT263537
CruV-457	4520	44.2%	Standard	Ambisense	TAGTATTAC		Lake Pavin (45°29'45.11''N; 2°53'14.60''E), sampling depth = 22 meters		vDNA, MDA	Illumina HiSeq	IDBA-UD		MT478475
CruV-458	4521	50.9%	Standard	Ambisense	TAGTATTAC				vDNA, MDA	Illumina HiSeq	metaSPAdes	Spliced Rep	MT263636
CruV-459	4525	46.1%	Standard	Unisense	TAGTATTAC		Lake Tunsjøen (78°03.375′N; 13°40.313′E)	Aguirre de Cárcer et al., 2015	vDNA, MDA	Illumina HiSeq	IDBA-UD		PRJEB5265
CruV-460	4538	44.0%	Standard	Unisense	#N/A		unnamed Arctic pond (78°02.935′N; 13°41.973′E)	Aguirre de Cárcer et al., 2015	vDNA, MDA	Illumina HiSeq	IDBA-UD	Spliced Rep	PRJEB5265
CruV-461	4542	53.6%	Standard	Ambisense	TAGTATTAC		unnamed Arctic pond (78°02.935′N; 13°41.973′E)	Aguirre de Cárcer et al., 2015	vDNA, MDA	Illumina HiSeq	IDBA-UD		PRJEB5265
CruV-462	4547	49.8%	Standard	Ambisense	TAGTATTAC		Lake Pavin (45°29'45.11''N; 2°53'14.60''E), sampling depth = 80 meters		vDNA, MDA	Illumina HiSeq	IDBA-UD	Spliced Rep; Capsid protein similar to bufiviruses	MT478474
CruV-463	4552	45.4%	Standard	Ambisense	TAGTATTAC	Capsid protein-based clusters	Lake Pavin (45°29'45.11''N; 2°53'14.60''E), sampling depth = 22 meters		vDNA, MDA	Illumina HiSeq	IDBA-UD		MT478473
CruV-464	4560	47.3%	Standard	Unisense	AAGTAGTAA	Rep-based clusters	unnamed Arctic pond (78°02.935′N; 13°41.973′E)	Aguirre de Cárcer et al., 2015	vDNA, MDA	Illumina HiSeq	IDBA-UD		PRJEB5265
CruV-465	4568	43.9%	Standard	Unisense	#N/A		unnamed Arctic pond (78°02.935′N; 13°41.973′E)	Aguirre de Cárcer et al., 2015	vDNA, MDA	Illumina HiSeq	IDBA-UD	Spliced Rep	PRJEB5265
CruV-466	4580	37.2%	Standard	Ambisense	AAGTATTAA	Rep-based clusters	unnamed Arctic pond (78°02.935′N; 13°41.973′E)	Aguirre de Cárcer et al., 2015	vDNA, MDA	Illumina HiSeq	IDBA-UD		PRJEB5265
CruV-467	4588	39.5%	Standard	Ambisense	#N/A		Lake Tunsjøen (78°03.375′N; 13°40.313′E)	Aguirre de Cárcer et al., 2015	vDNA, MDA	Illumina HiSeq	IDBA-UD		PRJEB5265
CruV-468	4601	48.1%	Standard	Ambisense	#N/A		River (NZ)		vDNA, MDA	Illumina HiSeq	metaSPAdes		MT263637
CruV-469	4605	44.7%	Standard	Ambisense	TAGTATTAC		unnamed Arctic pond (78°02.935′N; 13°41.973′E)	Aguirre de Cárcer et al., 2015	vDNA, MDA	Illumina HiSeq	IDBA-UD		PRJEB5265
CruV-470	4619	44.6%	Standard	Unisense	#N/A		Lake Aydat (45°39'52.859''N; 2°59'11.943''E) surface water		vDNA, MDA	Illumina HiSeq	IDBA-UD		MT478472
CruCGE-471	4650	43.8%	Standard	Unisense	CAATATTAC		River (NZ)		vDNA, MDA	Illumina HiSeq	metaSPAdes		MT263539
CruV-472	4656	49.8%	Standard	Ambisense	#N/A		Tern feces (Canada)		vDNA, MDA	Illumina HiSeq	metaSPAdes		MT263638
CruV-473	4661	38.7%	Ciliate	Ambisense	#N/A		unnamed Arctic pond (78°02.935′N; 13°41.973′E)	Aguirre de Cárcer et al., 2015	vDNA, MDA	Illumina HiSeq	IDBA-UD		PRJEB5265
CruV-474	4673	41.1%	Standard	Ambisense	CAGTATTAT		Odonatan larvae (NZ)		vDNA, MDA	Illumina HiSeq	metaSPAdes		MT263639
CruV-475	4676	52.2%	Standard	Ambisense	#N/A		Sewage Oxydation Pond (NZ)		vDNA, MDA	Illumina HiSeq	metaSPAdes		MT263640
CruV-476	4679	47.8%	Standard	Ambisense	#N/A		unnamed Arctic pond (78°02.935′N; 13°41.973′E)	Aguirre de Cárcer et al., 2015	vDNA, MDA	Illumina HiSeq	IDBA-UD		PRJEB5265
CruV-477	4711	37.4%	Standard	Ambisense	#N/A		unnamed Arctic pond (78°02.935′N; 13°41.973′E)	Aguirre de Cárcer et al., 2015	vDNA, MDA	Illumina HiSeq	IDBA-UD		PRJEB5265
CruV-478	4712	43.0%	Standard	Unisense	#N/A		unnamed Arctic pond (78°02.935′N; 13°41.973′E)	Aguirre de Cárcer et al., 2015	vDNA, MDA	Illumina HiSeq	IDBA-UD		PRJEB5265
CruV-479	4714	49.1%	Standard	Ambisense	CAGTATTAC		River (NZ)		vDNA, MDA	Illumina HiSeq	metaSPAdes		MT263641
CruV-480	4727	54.2%	Standard	Unisense	TACTATTAC	Capsid protein-based clusters	River (NZ)		vDNA, MDA	Illumina HiSeq	metaSPAdes		MT263642
CruV-481	4764	43.0%	Standard	Ambisense	#N/A		Lake Pavin (45°29'45.11''N; 2°53'14.60''E), sampling depth = 8 meters		vDNA, MDA	Illumina HiSeq	IDBA-UD		MT478471
CruV-482	4765	45.7%	Standard	Ambisense	CAGTATTAC		River (NZ)		vDNA, MDA	Illumina HiSeq	metaSPAdes		MT263643

CruV-483	4768	39.5%	Standard	Unisense	TATTATTAC		River bank soil (NZ)		vDNA, MDA	Illumina HiSeq	metaSPAdes	Spliced Rep	MT263644
CruV-484	4778	45.9%	Standard	Ambisense	AATTACTAG		Lake Pavin (45°29'45.11''N; 2°53'14.60''E), sampling depth = 22 meters		vDNA, MDA	Illumina HiSeq	IDBA-UD		MT478470
CruV-485	4778	49.3%	Standard	Ambisense	#N/A		unnamed Arctic pond (78°02.935′N; 13°41.973′E)	Aguirre de Cárcer et al., 2015	vDNA, MDA	Illumina HiSeq	IDBA-UD		PRJEB5265
CruV-486	4778	39.5%	Standard	Ambisense	CACTAATAG		Lake Pavin (45°29'45.11''N; 2°53'14.60''E), sampling depth = 22 meters		vDNA, MDA	Illumina HiSeq	IDBA-UD		MT478469
CruV-487	4779	38.3%	Standard	Unisense	AAGTATTAC	Capsid protein-based clusters	Lake Tenndammen (78°06.118′N; 15°02.024′E)	Aguirre de Cárcer et al., 2015	vDNA, MDA	Illumina HiSeq	IDBA-UD		PRJEB5265
CruV-488	4789	40.3%	Standard	Ambisense	#N/A		Lake Nordammen (78°38.279′N; 16°44.025′E)	Aguirre de Cárcer et al., 2015	vDNA, MDA	Illumina HiSeq	IDBA-UD		PRJEB5265
CruV-489	4817	35.3%	Ciliate	Unisense	CAGTACTAC		Lake Tunsjøen (78°03.375′N; 13°40.313′E)	Aguirre de Cárcer et al., 2015	vDNA, MDA	Illumina HiSeq	IDBA-UD		PRJEB5265
CruV-490	4826	40.7%	Standard	Unisense	#N/A		unnamed Arctic pond (78°02.935′N; 13°41.973′E)	Aguirre de Cárcer et al., 2015	vDNA, MDA	Illumina HiSeq	IDBA-UD	Spliced Rep	PRJEB5265
CruV-491	4831	47.4%	Standard	Unisense	GATTAGTAC		unnamed Arctic pond (78°02.935′N; 13°41.973′E)	Aguirre de Cárcer et al., 2015	vDNA, MDA	Illumina HiSeq	IDBA-UD		PRJEB5265
CruV-492	4833	37.5%	Standard	Unisense	GATTAATAA	Capsid protein-based clusters	unnamed Arctic pond (78°02.935′N; 13°41.973′E)	Aguirre de Cárcer et al., 2015	vDNA, MDA	Illumina HiSeq	IDBA-UD		PRJEB5265
CruV-493	4860	42.0%	Standard	Unisense	TAATACTAA / GATTAATAT / TAATATTAA		Lake Nordammen (78°38.279′N; 16°44.025′E)	Aguirre de Cárcer et al., 2015	vDNA, MDA	Illumina HiSeq	IDBA-UD		PRJEB5265
CruV-494	4862	44.0%	Standard	Ambisense	AACTAATAA		Lake Nordammen (78°38.279′N; 16°44.025′E)	Aguirre de Cárcer et al., 2015	vDNA, MDA	Illumina HiSeq	IDBA-UD		PRJEB5265
CruV-495	4869	45.9%	Standard	Ambisense	#N/A		Blackfly (NZ)		vDNA, MDA	Illumina HiSeq	metaSPAdes		MT263645
CruV-496	4874	43.6%	Standard	Unisense	#N/A		unnamed Arctic pond (78°02.935′N; 13°41.973′E)	Aguirre de Cárcer et al., 2015	vDNA, MDA	Illumina HiSeq	IDBA-UD		PRJEB5265
CruV-497	4881	36.0%	Standard	Ambisense	#N/A		River (NZ)		vDNA, MDA	Illumina HiSeq	metaSPAdes	Spliced Rep	MT263646
CruV-498	4899	40.6%	Standard	Unisense	AATTAATAC / GACTAATAT		Lake Tunsjøen (78°03.375′N; 13°40.313′E)	Aguirre de Cárcer et al., 2015	vDNA, MDA	Illumina HiSeq	IDBA-UD		PRJEB5265
CruV-499	4920	44.5%	Standard	Unisense	#N/A		Penguin guano (Antarctica)		vDNA, MDA	Illumina HiSeq	metaSPAdes	Spliced Rep	MT263647
CruV-500	4940	43.6%	Ciliate	Unisense	TAGTATTAC		unnamed Arctic pond (78°02.935′N; 13°41.973′E)	Aguirre de Cárcer et al., 2015	vDNA, MDA	Illumina HiSeq	IDBA-UD		PRJEB5265
CruV-501	4942	42.7%	Standard	Unisense	CACTAGTAG		Bivalve (NZ)		vDNA, MDA	Illumina HiSeq	metaSPAdes		MT263648
CruV-502	4948	50.7%	Ciliate	Ambisense	TACTATTAC		Brewers Bay, St. Thomas, U.S. Virgin Islands (18°20'34.526''N; 64°58'51.24''W)	Soffer et al., 2014	vDNA, MDA	Roche 454	newbler		N/A
CruV-503	4955	45.6%	Standard	Unisense	#N/A		Lake Aydat (45°39'52.859''N; 2°59'11.943''E) surface water		vDNA, MDA	Illumina HiSeq	IDBA-UD		MT478468
CruV-504	4979	39.9%	Standard	Unisense	#N/A		Lake Aydat (45°39'52.859''N; 2°59'11.943''E) surface water		vDNA, MDA	Illumina HiSeq	IDBA-UD		MT478467
CruV-505	4991	47.7%	Standard	Ambisense	#N/A		River (NZ)		vDNA, MDA	Illumina HiSeq	metaSPAdes		MT263649
CruV-506	4991	47.7%	Standard	Ambisense	#N/A		River (NZ)		vDNA, MDA	Illumina HiSeq	metaSPAdes		MT263650
CruV-507	4992	42.8%	Standard	Ambisense	TAATATAAG / TATTATAAT / TAAATAAAT	Capsid protein-based clusters	Lake Nordammen (78°38.279′N; 16°44.025′E)	Aguirre de Cárcer et al., 2015	vDNA, MDA	Illumina HiSeq	IDBA-UD		PRJEB5265
CruV-508	4998	49.9%	Standard	Ambisense	TAATATAAG / TATTATAAT / TAAATAAAG		Lake Aydat (45°39'52.859''N; 2°59'11.943''E) surface water		vDNA, MDA	Illumina HiSeq	IDBA-UD	Spliced Rep; Capsid protein similar to bufiviruses	MT478466
CruV-509	5004	43.2%	Standard	Unisense	TAATATAAG / TATTATAAT / TAAATAAAT		River (NZ)		vDNA, MDA	Illumina HiSeq	metaSPAdes		MT263651
CruV-510	5010	38.3%	Standard	Ambisense	TAATATAAG / TATTATAAT / TAAATAAAT		Lake Aydat (45°39'52.859''N; 2°59'11.943''E) surface water		vDNA, MDA	Illumina HiSeq	IDBA-UD	Rep is homologous to Smittium Rep	MT478465
CruV-511	5014	44.1%	Standard	Unisense	CACTACTAG		unnamed Arctic pond (78°02.935′N; 13°41.973′E)	Aguirre de Cárcer et al., 2015	vDNA, MDA	Illumina HiSeq	IDBA-UD		PRJEB5265
CruV-512	5039	44.1%	Standard	Ambisense	CAGTATTAC		unnamed Arctic pond (78°02.935′N; 13°41.973′E)	Aguirre de Cárcer et al., 2015	vDNA, MDA	Illumina HiSeq	IDBA-UD	Spliced Rep	PRJEB5265
CruV-513	5051	39.3%	Standard	Unisense	TAGTATTAC		River bank soil (NZ)		vDNA, MDA	Illumina HiSeq	metaSPAdes	Spliced Rep and Capsid protein	MT263652
CruV-514	5087	40.7%	Ciliate	Ambisense	AACTAATAT		Gastropods (NZ)		vDNA, MDA	Illumina HiSeq	metaSPAdes		MT263653
CruV-515	5111	51.0%	Standard	Ambisense	#N/A		Lake Pavin (45°29'45.11''N; 2°53'14.60''E), sampling depth = 80 meters		vDNA, MDA	Illumina HiSeq	IDBA-UD	Spliced Rep	MT478464
CruV-516	5115	46.4%	Ciliate	Unisense	CAGTATTAC		unnamed Arctic pond (78°02.935′N; 13°41.973′E)	Aguirre de Cárcer et al., 2015	vDNA, MDA	Illumina HiSeq	IDBA-UD		PRJEB5265
CruV-517	5120	50.9%	Standard	Ambisense	TAATTCAAG		Lake Pavin (45°29'45.11''N; 2°53'14.60''E), sampling depth = 22 meters		vDNA, MDA	Illumina HiSeq	IDBA-UD	Spliced Rep	MT478463
CruV-518	5126	34.6%	Standard	Unisense	#N/A		Lake Aydat (45°39'52.859''N; 2°59'11.943''E) surface water		vDNA, MDA	Illumina HiSeq	IDBA-UD	Spliced Rep and Capsid protein	MT478462
CruV-519	5164	56.6%	Standard	Unisense	#N/A		Lake Nordammen (78°38.279′N; 16°44.025′E)	Aguirre de Cárcer et al., 2015	vDNA, MDA	Illumina HiSeq	IDBA-UD		PRJEB5265
CruV-520	5206	48.9%	Standard	Unisense	TAATGTAAA		River (NZ)		vDNA, MDA	Illumina HiSeq	metaSPAdes		MT263654

**CruV-521**	5219	47.1%	Standard	Ambisense	#N/A		Lake Aydat (45°39'52.859''N; 2°59'11.943''E) surface water		vDNA, MDA	Illumina HiSeq	IDBA-UD		MT478461
**CruV-522**	5225	42.2%	Standard	Unisense	CATTAATAT		Lake Aydat (45°39'52.859''N; 2°59'11.943''E) surface water		vDNA, MDA	Illumina HiSeq	IDBA-UD	Spliced Rep	MT478460
**CruV-523**	5257	50.8%	Standard	Unisense	GATTACTAC		River (NZ)		vDNA, MDA	Illumina HiSeq	metaSPAdes	Spliced Rep, low similarity Capsid protein	MT263655
**CruV-524**	5271	43.6%	Standard	Ambisense	CATTATTAC	Capsid protein-based clusters	Lake Aydat (45°39'52.859''N; 2°59'11.943''E) surface water		vDNA, MDA	Illumina HiSeq	IDBA-UD		MT478459
**CruV-525**	5284	46.9%	Standard	Ambisense	TAAATCTAC		Lake Aydat (45°39'52.859''N; 2°59'11.943''E) surface water		vDNA, MDA	Illumina HiSeq	IDBA-UD		MT478458
**CruV-526**	5312	53.7%	Standard	Unisense	TAGTATTAC		Lake Nordammen (78°38.279′N; 16°44.025′E)	Aguirre de Cárcer et al., 2015	vDNA, MDA	Illumina HiSeq	IDBA-UD	Spliced Rep	PRJEB5265
**CruV-527**	5318	32.7%	Standard	Ambisense	AAATAATAA		Soil (NZ)		vDNA, MDA	Illumina HiSeq	metaSPAdes		MT263656
**CruV-528**	5370	32.7%	Standard	Ambisense	AAGTAATAA	Capsid protein-based clusters	Soil (NZ)		vDNA, MDA	Illumina HiSeq	metaSPAdes		MT263657
**CruV-529**	5452	46.9%	Standard	Ambisense	TAGTATTAC		Lake Aydat (45°39'52.859''N; 2°59'11.943''E) surface water		vDNA, MDA	Illumina HiSeq	IDBA-UD	Spliced Rep	MT478457
**CruV-530**	5549	38.4%	Standard	Unisense	#N/A		Chirominidae (NZ)		vDNA, MDA	Illumina HiSeq	metaSPAdes		MT263658
**CruV-531**	5582	42.3%	Standard	Ambisense	TAAATTTAA / TAAATTTAT		Lake Aydat (45°39'52.859''N; 2°59'11.943''E) surface water		vDNA, MDA	Illumina HiSeq	IDBA-UD		MT478456
**CruV-532**	5603	49.9%	Ciliate	Ambisense	#N/A		unnamed Arctic pond (78°02.935′N; 13°41.973′E)	Aguirre de Cárcer et al., 2015	vDNA, MDA	Illumina HiSeq	IDBA-UD		PRJEB5265
**CruCGE-533**	5688	42.9%	Standard	Unisense	CATTAATAT		Lake Nordammen (78°38.279′N; 16°44.025′E)	Aguirre de Cárcer et al., 2015	vDNA, MDA	Illumina HiSeq	IDBA-UD		PRJEB5265
**CruV-534**	5797	40.9%	Standard	Ambisense	TATATAAAA		Soil (NZ)		vDNA, MDA	Illumina HiSeq	metaSPAdes		MT263659
**CruV-535**	5803	42.7%	Standard	Ambisense	CAGTATTAC		Lake Aydat (45°39'52.859''N; 2°59'11.943''E) surface water		vDNA, MDA	Illumina HiSeq	IDBA-UD		MT478455
**CruV-536**	5856	37.3%	Standard	Unisense	TATTGAAAG		River bank soil (NZ)		vDNA, MDA	Illumina HiSeq	metaSPAdes		MT263660
**CruV-537**	6350	42.3%	Standard	Unisense	CAGTATTAC		Lake Aydat (45°39'52.859''N; 2°59'11.943''E) surface water		vDNA, MDA	Illumina HiSeq	IDBA-UD	Spliced Rep	MT478454
**CruV-538**	6385	42.9%	Standard	Unisense	#N/A	Rep-based clusters	River (NZ)		vDNA, MDA	Illumina HiSeq	metaSPAdes		MT263661
**CruV-539**	6421	39.3%	Standard	Ambisense	TGATATTAC		River (NZ)		vDNA, MDA	Illumina HiSeq	metaSPAdes		MT263662
**CruV-540**	6902	40.8%	Standard	Ambisense	#N/A		River bank soil (NZ)		vDNA, MDA	Illumina HiSeq	metaSPAdes		MT263663
**CruV-541**	7947	45.1%	Standard	Unisense	CAATATTAC / TAATGTTAT / TAATGAAAT / TAAAGTTAC		Lake Nordammen (78°38.279′N; 16°44.025′E)	Aguirre de Cárcer et al., 2015	vDNA, MDA	Illumina HiSeq	IDBA-UD		PRJEB5265

REFERENCES

De Cárcer, D. A., López-Bueno, A., Pearce, D. A. & Alcamí, A. Biodiversity and distribution of polar freshwater DNA viruses. *Sci. Adv.* (2015). doi:10.1126/sciadv.1400127

Lundberg, D. S. *et al.* Defining the core Arabidopsis thaliana root microbiome. *Nature* 488, 86–90 (2012).

Roux, S., Enault, F., Ravet, V., Pereira, O. & Sullivan, M. B. Genomic characteristics and environmental distributions of the uncultivated Far-T4 phages. *Front. Microbiol.* (2015). doi:10.3389/fmicb.2015.00199

Soffer, N., Brandt, M. E., Correa, A. M. S., Smith, T. B. & Thurber, R. V. Potential role of viruses in white plague coral disease. *ISME J.* (2014). doi:10.1038/ismej.2013.137

Zozaya-Valdés, E., Roth-Schulze, A. J., Egan, S. & Thomas, T. Microbial community function in the bleaching disease of the marine macroalgae Delisea pulchra. *Environ. Microbiol.* (2017). doi:10.1111/1462-2920.1

Wu, Z. *et al.* Deciphering the bat virome catalog to better understand the ecological diversity of bat viruses and the bat origin of emerging infectious diseases. *ISME J.* (2016). doi:10.1038/ismej.2015.138

*** GenBank accession numbers are provided for previously unpublished sequences. SRA and JGI's GOLD accession numbers are provided for previously published sequences 461 annotated cruciviral sequences are provided as a GenBank flatfile in Supp. File 1.**

3758

s. All
