# Supplementary material for: Unveiling Crucivirus Diversity by Mining Metagenomic Data
Source: mBio. 2020 Sep 1;11(5):e01410-20. doi: 10.1128/mBio.01410-20 (PMC7468197; doi:10.1128/mBio.01410-20)
Supplement: TABLE S2 [file mBio.01410-20-st002.pdf]

Supplementary Table 2: Viromes analyzed

Project Name	Virome Code	Habitat Type (code)	Ecosystem Type (text)	Location	GPS	Source	Sequencing Technology	Assembly Software	# crucivirus	Reference
Santiago_chemostatCulture	Donor10Day12	Engineered_Bioreactor	Bioreactor	USA : California	32.879696 -117.234507	reads MG-RAST	ion torrent	Newbler	0	Santiago-Rodriguez TM. Ly M. Daigneault MC. Brown IH. McDonald JA. Bonilla N. Vercoe EA. Pride DT. Chemostat culture systems support diverse bacteriophage communities from human feces. Microbiome. 2015 Nov 9;3(1):58. doi: 10.1186/s40168-015-0124-3.
Santiago_chemostatCulture	Donor10Day16	Engineered_Bioreactor	Bioreactor	USA : California	32.879696 -117.234507	reads MG-RAST	ion torrent	Newbler	0	
Santiago_chemostatCulture	Donor10Day4	Engineered_Bioreactor	Bioreactor	USA : California	32.879696 -117.234507	reads MG-RAST	ion torrent	Newbler	0	
Santiago_chemostatCulture	Donor10Day8	Engineered_Bioreactor	Bioreactor	USA : California	32.879696 -117.234507	reads MG-RAST	ion torrent	Newbler	0	
Santiago_chemostatCulture	Donor10Liq	Engineered_Bioreactor	Bioreactor	USA : California	32.879696 -117.234507	reads MG-RAST	ion torrent	Newbler	0	
Santiago_chemostatCulture	Donor10Stool	Engineered_Bioreactor	Bioreactor	USA : California	32.879696 -117.234507	reads MG-RAST	ion torrent	Newbler	0	
Santiago_chemostatCulture	Donor1Day12	Engineered_Bioreactor	Bioreactor	USA : California	32.879696 -117.234507	reads MG-RAST	ion torrent	Newbler	0	
Santiago_chemostatCulture	Donor1Day16	Engineered_Bioreactor	Bioreactor	USA : California	32.879696 -117.234507	reads MG-RAST	ion torrent	Newbler	0	
Santiago_chemostatCulture	Donor1Day4	Engineered_Bioreactor	Bioreactor	USA : California	32.879696 -117.234507	reads MG-RAST	ion torrent	Newbler	0	
Santiago_chemostatCulture	Donor1Day8	Engineered_Bioreactor	Bioreactor	USA : California	32.879696 -117.234507	reads MG-RAST	ion torrent	Newbler	0	
Santiago_chemostatCulture	Donor1Liq	Engineered_Bioreactor	Bioreactor	USA : California	32.879696 -117.234507	reads MG-RAST	ion torrent	Newbler	0	
Santiago_chemostatCulture	Donor1Stool	Engineered_Bioreactor	Bioreactor	USA : California	32.879696 -117.234507	reads MG-RAST	ion torrent	Newbler	0	
Santiago_chemostatCulture	Donor2Day12	Engineered_Bioreactor	Bioreactor	USA : California	32.879696 -117.234507	reads MG-RAST	ion torrent	Newbler	0	
Santiago_chemostatCulture	Donor2Day16	Engineered_Bioreactor	Bioreactor	USA : California	32.879696 -117.234507	reads MG-RAST	ion torrent	Newbler	0	
Santiago_chemostatCulture	Donor2Day4	Engineered_Bioreactor	Bioreactor	USA : California	32.879696 -117.234507	reads MG-RAST	ion torrent	Newbler	0	
Santiago_chemostatCulture	Donor2Day8	Engineered_Bioreactor	Bioreactor	USA : California	32.879696 -117.234507	reads MG-RAST	ion torrent	Newbler	0	
Santiago_chemostatCulture	Donor2Liq	Engineered_Bioreactor	Bioreactor	USA : California	32.879696 -117.234507	reads MG-RAST	ion torrent	Newbler	0	
Santiago_chemostatCulture	Donor2Stool	Engineered_Bioreactor	Bioreactor	USA : California	32.879696 -117.234507	reads MG-RAST	ion torrent	Newbler	0	
Santiago_chemostatCulture	Donor8Day12	Engineered_Bioreactor	Bioreactor	USA : California	32.879696 -117.234507	reads MG-RAST	ion torrent	Newbler	0	
Santiago_chemostatCulture	Donor8Day18	Engineered_Bioreactor	Bioreactor	USA : California	32.879696 -117.234507	reads MG-RAST	ion torrent	Newbler	0	
Santiago_chemostatCulture	Donor8Day3	Engineered_Bioreactor	Bioreactor	USA : California	32.879696 -117.234507	reads MG-RAST	ion torrent	Newbler	0	
Santiago_chemostatCulture	Donor8Day6	Engineered_Bioreactor	Bioreactor	USA : California	32.879696 -117.234507	reads MG-RAST	ion torrent	Newbler	0	
Santiago_chemostatCulture	Donor8Liq	Engineered_Bioreactor	Bioreactor	USA : California	32.879696 -117.234507	reads MG-RAST	ion torrent	Newbler	0	
Santiago_chemostatCulture	Donor8Stool	Engineered_Bioreactor	Bioreactor	USA : California	32.879696 -117.234507	reads MG-RAST	ion torrent	Newbler	0	
Santiago_chemostatCulture	Donor9Day12	Engineered_Bioreactor	Bioreactor	USA : California	32.879696 -117.234507	reads MG-RAST	ion torrent	Newbler	0	
Santiago_chemostatCulture	Donor9Day18	Engineered_Bioreactor	Bioreactor	USA : California	32.879696 -117.234507	reads MG-RAST	ion torrent	Newbler	0	
Santiago_chemostatCulture	Donor9Day3	Engineered_Bioreactor	Bioreactor	USA : California	32.879696 -117.234507	reads MG-RAST	ion torrent	Newbler	0	
Santiago_chemostatCulture	Donor9Day6	Engineered_Bioreactor	Bioreactor	USA : California	32.879696 -117.234507	reads MG-RAST	ion torrent	Newbler	0	
Santiago_chemostatCulture	Donor9Liq	Engineered_Bioreactor	Bioreactor	USA : California	32.879696 -117.234507	reads MG-RAST	ion torrent	Newbler	0	
Santiago_chemostatCulture	Donor9Stool	Engineered_Bioreactor	Bioreactor	USA : California	32.879696 -117.234507	reads MG-RAST	ion torrent	Newbler	0	
Park_FermentedFood	Cabbage	Engineered_FoodProduction	Food	Korea : Seoul	37.528853 126.985222	reads Metavir	454	Newbler	0	Park EJ. Kim KH. Abell GC. Kim MS. Roh SW. Bae JW. Metagenomic analysis of the viral communities in fermented foods. Appl Environ Microbiol. 2011 Feb;77(4):1284-91. doi: 10.1128/AEM.01859-10.
Park_FermentedFood	Sauerkraut	Engineered_FoodProduction	Food	Korea : Seoul	37.528853 126.985222	reads Metavir	454	Newbler	0	
Park_FermentedFood	Shrimp	Engineered_FoodProduction	Food	Korea : Seoul	37.528853 126.985222	reads Metavir	454	Newbler	0	
Bellas_CryoconiteArctic	Cryoconite	EnvAquatic_Freshwater	Polar Freshwater	Greenland sea	67.161028 -50.014639	contigs Metavir	?	?	0	Bellas CM. Anesio AM. Barker G. Analysis of virus genomes from glacial environments reveals novel virus groups with unusual host interactions. Front Microbiol. 2015 Jul 3;6:656. doi: 10.3389/fmicb.2015.00656. eCollection 2015.
Cai_SubtropicalJiulongRiverEstuary	JRE6750	EnvAquatic_Freshwater	Tropical Freshwater	China : Jiulong River Estuary	24.432207 117.930848	reads Metavir/ contigs Metavir	?	?	0	Cai L. Zhang R. He Y. Feng X. Jiao N. Metagenomic Analysis of Virioplankton of the Subtropical Jiulong River Estuary. China. Viruses. 2016 Feb 2;8(2). pii: E35. doi: 10.3390/v8020035.
Carcer_ArcticFresh	ArctSumIII	EnvAquatic_Freshwater	Polar Freshwater	Borgdammane pond	78.070900 13.794200	contigs Metavir	Illumina HiSeq	IDBA_UD	1	Aguirre de Cárcer D. López-Bueno A. Pearce DA. Alcamí A. Biodiversity and distribution of polar freshwater DNA viruses. Sci Adv. 2015 Jun 19;1(5):e1400127. eCollection 2015 Jun. PMID:26601189
Carcer_ArcticFresh	IR1	EnvAquatic_Freshwater	Polar Freshwater	Lake Tunsjøen	78.056250 13.671883	contigs Metavir	Illumina HiSeq	IDBA_UD	34	
Carcer_ArcticFresh	IR2	EnvAquatic_Freshwater	Polar Freshwater	unnamed Arctic pond	78.048917 13.699550	contigs Metavir	Illumina HiSeq	IDBA_UD	114	
Carcer_ArcticFresh	Lv1	EnvAquatic_Freshwater	Polar Freshwater	Lake Linnevatnet	78.064400 13.771800	contigs Metavir	Illumina HiSeq	IDBA_UD	1	

Project Name	Virome Code	Habitat Type (code)	Ecosystem Type (text)	Location	GPS	Source	Sequencing Technology	Assembly Software	# crucivirus	Reference
Carcer_ArcticFresh	SvL1	EnvAquatic_Freshwater	Polar Freshwater	Lake Nordammen	78.637983 16.733750	contigs Metavir	Illumina HiSeq	IDBA_UD	40	
Carcer_ArcticFresh	SvL2	EnvAquatic_Freshwater	Polar Freshwater	Lake Tenndammen	78.101967 15.033733	contigs Metavir	Illumina HiSeq	IDBA_UD	24	
Fancello_Gueltas	ElBerbera	EnvAquatic_Freshwater	Freshwater	Mauritania	20.03361111 13.85666667	reads Metavir	454	Newbler	0	Fancello L. Trape S. Robert C. Boyer M. Popgeorgiev N. Raoult D. Desnues C. Viruses in the desert: a metagenomic survey of viral communities in four perennial ponds of the Mauritanian Sahara. ISME J. 2013 Feb;7(2):359-69. doi: 10.1038/ismej.2012.101. Epub 2012 Oct 4.
Fancello_Gueltas	Hamdoun	EnvAquatic_Freshwater	Freshwater	Mauritania	20.42222222 13.28611111	reads Metavir	454	Newbler	0	
Fancello_Gueltas	Ilij	EnvAquatic_Freshwater	Freshwater	Mauritania	20.64611111 13.26944444	reads Metavir	454	Newbler	0	
Fancello_Gueltas	Molomhar	EnvAquatic_Freshwater	Freshwater	Mauritania	20.64694444 13.35388889	reads Metavir	454	Newbler	0	
Ge_EastLakeChina	EastLake210	EnvAquatic_Freshwater	Freshwater	China : Lake Donghu	30.557213 114.391886	contigs MG-RAST	solexa	?	0	Ge X. Wu Y. Wang M. Wang J. Wu L. Yang X. Zhang Y. Shi Z. Viral metagenomics analysis of planktonic viruses in East Lake. Wuhan. China. Virol Sin. 2013 Oct;28(5):280-90. doi: 10.1007/s12250-013-3365-y. Epub 2013 Sep 30.
Ge_EastLakeChina	EastLake208	EnvAquatic_Freshwater	Freshwater	China : Lake Donghu	30.557213 114.391886	contigs MG-RAST	solexa	Newbler	0	
Ge_EastLakeChina	EastLake209	EnvAquatic_Freshwater	Freshwater	China : Lake Donghu	30.557213 114.391886	contigs MG-RAST	solexa	Newbler	0	
Ge_EastLakeChina	EastLake211	EnvAquatic_Freshwater	Freshwater	China : Lake Donghu	30.557213 114.391886	contigs MG-RAST	solexa	?	0	
Green_LakeMatoaka	CrimDell	EnvAquatic_Freshwater	Freshwater	USA : Virginia	37.267189 -76.725779	reads Metavir	454	Newbler	0	Jasmin C. Green. Faraz Rahman. Matthew A. Saxton. Kurt E. Williamson . Metagenomic assessment of viral diversity in Lake Matoaka. a temperate. eutrophic freshwater lake in southeastern Virginia. USA . AME 75:117-128 (2015) - doi:10.3354/ame0175
Green_LakeMatoaka	Matoaka	EnvAquatic_Freshwater	Freshwater	USA : Virginia	37.267189 -76.725779	reads Metavir	454	Newbler	0	
Green_LakeMatoaka	Pogonian	EnvAquatic_Freshwater	Freshwater	USA : Virginia	37.267189 -76.725779	reads Metavir	454	Newbler	0	
Lopezbueno_RNAantarctic	L2006RNA	EnvAquatic_Freshwater	Polar Freshwater	Antarctica	-62.562386 -60.040952	reads MG-RAST	454	Newbler	0	López-Bueno A1. Rastrojo A1. Peiró R1. Arenas M1. Alcamí A1. Ecological connectivity shapes quasispecies structure of RNA viruses in an Antarctic lake. Mol Ecol. 2015 Oct;24(19):4812-4825. doi: 10.1111/mec.13321. Epub 2015 Aug 11.
Lopezbueno_RNAantarctic	L2007RNA	EnvAquatic_Freshwater	Polar Freshwater	Antarctica	-62.562386 -60.040952	reads Metavir	454	Newbler	0	
Lopezbueno_RNAantarctic	L2010RNA	EnvAquatic_Freshwater	Polar Freshwater	Antarctica	-62.562386 -60.040952	reads Metavir	454	Newbler	0	
Mohiuddin_FreshLakes	50pOnta3215e	EnvAquatic_Freshwater	Freshwater	Lake Ontario	43.2247 -79.6183	reads SRA	Illumina HiSeq2000	IDBA_UD	0	Mohiuddin M. Schellhorn HE . Spatial and temporal dynamics of virus occurrence in two freshwater lakes captured through metagenomic analysis. Front Microbiol. 2015 Sep 15;6:960. doi: 10.3389/fmicb.2015.00960. eCollection 2015.
Mohiuddin_FreshLakes	50pOnta3224v	EnvAquatic_Freshwater	Freshwater	Lake Ontario	43.2247 -79.6183	reads SRA	Illumina HiSeq2000	IDBA_UD	0	
Mohiuddin_FreshLakes	Lb1Erie3223v	EnvAquatic_Freshwater	Freshwater	Lake Erie	42.8638 -79.3862	reads SRA	Illumina HiSeq2000	IDBA_UD	0	
Mohiuddin_FreshLakes	Lb2Erie3222v	EnvAquatic_Freshwater	Freshwater	Lake Erie	42.8698 -79.3982	reads SRA	Illumina HiSeq2000	IDBA_UD	0	
Mohiuddin_FreshLakes	Lb3Erie3221v	EnvAquatic_Freshwater	Freshwater	Lake Erie	42.8723 -79.4187	reads SRA	Illumina HiSeq2000	IDBA_UD	0	
Mohiuddin_FreshLakes	Lb4Erie3220v	EnvAquatic_Freshwater	Freshwater	Lake Erie	42.8638 -79.3862	reads SRA	Illumina HiSeq2000	IDBA_UD	0	
Mohiuddin_FreshLakes	LbCEErie2964e	EnvAquatic_Freshwater	Freshwater	Lake Erie	42.8722 -79.4272	reads SRA	Illumina HiSeq2000	IDBA_UD	0	
Mohiuddin_FreshLakes	LbCEErie3218v	EnvAquatic_Freshwater	Freshwater	Lake Erie	42.8722 -79.4272	reads SRA	Illumina HiSeq2000	IDBA_UD	0	
Mohiuddin_FreshLakes	LbErie3214e	EnvAquatic_Freshwater	Freshwater	Lake Erie	42.8638 -79.3862	reads SRA	Illumina HiSeq2000	IDBA_UD	0	
Mohiuddin_FreshLakes	Ls1Onta3504v	EnvAquatic_Freshwater	Freshwater	Lake Ontario	43.2043 -79.2669	reads SRA	Illumina HiSeq2000	IDBA_UD	0	
Mohiuddin_FreshLakes	Ls2Onta3231v	EnvAquatic_Freshwater	Freshwater	Lake Ontario	43.2047 -79.266	reads SRA	Illumina HiSeq2000	IDBA_UD	0	
Mohiuddin_FreshLakes	Ls3Onta3230v	EnvAquatic_Freshwater	Freshwater	Lake Ontario	43.2052 -79.2648	reads SRA	Illumina HiSeq2000	IDBA_UD	0	
Mohiuddin_FreshLakes	Ls4Onta3227v	EnvAquatic_Freshwater	Freshwater	Lake Ontario	43.2043 -79.2669	reads SRA	Illumina HiSeq2000	IDBA_UD	0	
Mohiuddin_FreshLakes	LsOnta3217e	EnvAquatic_Freshwater	Freshwater	Lake Ontario	43.2043 -79.2669	reads SRA	Illumina HiSeq2000	IDBA_UD	0	
Mohiuddin_FreshLakes	NbErie3213e	EnvAquatic_Freshwater	Freshwater	Lake Erie	42.8746 -79.2323	reads SRA	Illumina HiSeq2000	IDBA_UD	0	
Mohiuddin_FreshLakes	NbErie3219v	EnvAquatic_Freshwater	Freshwater	Lake Erie	42.8746 -79.2323	reads SRA	Illumina HiSeq2000	IDBA_UD	0	
Mohiuddin_FreshLakes	QrOnta3216e	EnvAquatic_Freshwater	Freshwater	Lake Ontario	43.2579 -79.0687	reads SRA	Illumina HiSeq2000	IDBA_UD	0	
Mohiuddin_FreshLakes	QrOnta3225v	EnvAquatic_Freshwater	Freshwater	Lake Ontario	43.2579 -79.0687	reads SRA	Illumina HiSeq2000	IDBA_UD	0	
Roux_Caviar	PavinA15	EnvAquatic_Freshwater	Freshwater	France : Pavin lake	45.495210 2.888074	reads OwnSite	Illumina HiSeq2000	IDBA_UD	0	Roux S, Enault F, Ravet V, Pereira O, Sullivan MB. Genomic characteristics and environmental distributions of the uncultivated Far-T4 phages. Front Microbiol. 2015 Mar 16;6:199. doi: 10.3389/fmicb.2015.00199.
Roux_Caviar	PavinA22	EnvAquatic_Freshwater	Freshwater	France : Pavin lake	45.495210 2.888074	reads OwnSite	Illumina HiSeq2000	IDBA_UD	11	
Roux_Caviar	PavinA4	EnvAquatic_Freshwater	Freshwater	France : Pavin lake	45.495210 2.888074	reads OwnSite	Illumina HiSeq2000	IDBA_UD	0	
Roux_Caviar	PavinA8	EnvAquatic_Freshwater	Freshwater	France : Pavin lake	45.495210 2.888074	reads OwnSite	Illumina HiSeq2000	IDBA_UD	1	
Roux_Caviar	PavinA80	EnvAquatic_Freshwater	Freshwater	France : Pavin lake	45.495210 2.888074	reads OwnSite	Illumina HiSeq2000	IDBA_UD	2	
Roux_Caviar	PavinART15	EnvAquatic_Freshwater	Freshwater	France : Pavin lake	45.495210 2.888074	reads OwnSite	Illumina HiSeq2000	IDBA_UD	0	
Roux_Caviar	PavinART22	EnvAquatic_Freshwater	Freshwater	France : Pavin lake	45.495210 2.888074	reads OwnSite	Illumina HiSeq2000	IDBA_UD	0	
Roux_Caviar	PavinART4	EnvAquatic_Freshwater	Freshwater	France : Pavin lake	45.495210 2.888074	reads OwnSite	Illumina HiSeq2000	IDBA_UD	0	

Project Name	Virome Code	Habitat Type (code)	Ecosystem Type (text)	Location	GPS	Source	Sequencing Technology	Assembly Software	# crucivirus	Reference
Roux_Caviar	PavinART8	EnvAquatic_Freshwater	Freshwater	France : Pavin lake	45.495210 2.888074	reads OwnSite	Illumina HiSeq2000	IDBA_UD	0	
Roux_Caviar	PavinARt80	EnvAquatic_Freshwater	Freshwater	France : Pavin lake	45.495210 2.888074	reads OwnSite	Illumina HiSeq2000	IDBA_UD	0	
Roux_Caviar	virusbaton	EnvAquatic_Freshwater	Freshwater	France : Pavin lake	45.495210 2.888074	reads OwnSite	Illumina HiSeq2000	IDBA_UD	95	
Skvortsov_LakeLoughNeagh	LoughNeagh	EnvAquatic_Freshwater	Freshwater	UK : Lough Neagh	54.591791 -6.434395	reads SRA	Illumina	IDBA_UD	0	Skvortsov T. de Leeuwe C. Quinn JP. McGrath JW. Allen CC. McElarney Y. Watson C. Arkhipova K. Lavigne R. Kulakov LA. Metagenomic Characterisation of the Viral Community of Lough Neagh. the Largest Freshwater Lake in Ireland. PLoS One. 2016 Feb 29;11(2):e0150361. doi: 10.1371/journal.pone.0150361.
Tseng_SubTropicalFresh	subTropFresh74	EnvAquatic_Freshwater	Freshwater	Taiwan : Taipei	24.905627 121.553608	reads SRA	454 GS FLX	Newbler	0	Tseng CH. Chiang PW. Shiah FK. Chen YL. Liou JR. Hsu TC. Maheswararajah S. Saeed I. Halgamuge S. Tang SL. Microbial and viral metagenomes of a subtropical freshwater reservoir subject to climatic disturbances. ISME J. 2013 Dec;7(12):2374-86. doi: 10.1038/ismej.2013.118.
Tseng_SubTropicalFresh	SRR6483	EnvAquatic_Freshwater	Freshwater	Taiwan : Taipei	24.905627 121.553608	reads SRA	454 GS FLX	Newbler	0	
Tseng_SubTropicalFresh	subTropFresh11	EnvAquatic_Freshwater	Freshwater	Taiwan : Taipei	24.905627 121.553608	reads SRA	454 GS FLX	Newbler	0	
Tseng_SubTropicalFresh	subTropFresh13	EnvAquatic_Freshwater	Freshwater	Taiwan : Taipei	24.905627 121.553608	reads SRA	454 GS FLX	Newbler	0	
Tseng_SubTropicalFresh	subTropFresh14	EnvAquatic_Freshwater	Freshwater	Taiwan : Taipei	24.905627 121.553608	reads SRA	454 GS FLX	Newbler	0	
Tseng_SubTropicalFresh	subTropFresh73	EnvAquatic_Freshwater	Freshwater	Taiwan : Taipei	24.905627 121.553608	reads SRA	454 GS FLX	Newbler	0	
Watkins_LakeMichigan	Mont05Jul13	EnvAquatic_Freshwater	Freshwater	USA : Michigan	43.376762 -86.994278	reads Metavir	?	IDBA_UD	0	Watkins SC. Kuehnle N. Ruggeri CA. Malki K. Bruder K. Elayyan J. Damisch K. Vahora N. O'Malley P. Ruggles-Sage B. Romer Z. Putonti C. Assessment of a metaviromic dataset generated from nearshore Lake Michigan. Marine and Freshwater Research - http://dx.doi.org/10.1071/MF15172
Watkins_LakeMichigan	Mont05Jun13	EnvAquatic_Freshwater	Freshwater	USA : Michigan	43.376762 -86.994278	reads Metavir	?	IDBA_UD	0	
Watkins_LakeMichigan	Mont15Jun13	EnvAquatic_Freshwater	Freshwater	USA : Michigan	43.376762 -86.994278	reads Metavir	?	IDBA_UD	0	
Watkins_LakeMichigan	Mont25Jul13	EnvAquatic_Freshwater	Freshwater	USA : Michigan	43.376762 -86.994278	reads Metavir	?	IDBA_UD	0	
Watkins_LakeMichigan	Mont25Jun13	EnvAquatic_Freshwater	Freshwater	USA : Michigan	43.376762 -86.994278	reads Metavir	?	IDBA_UD	0	
Watkins_LakeMichigan	s57th05Jun13	EnvAquatic_Freshwater	Freshwater	USA : Michigan	43.376762 -86.994278	reads Metavir	?	IDBA_UD	0	
Watkins_LakeMichigan	s57th14Aug13	EnvAquatic_Freshwater	Freshwater	USA : Michigan	43.376762 -86.994278	reads Metavir	?	IDBA_UD	0	
Watkins_LakeMichigan	s57th15Jul13	EnvAquatic_Freshwater	Freshwater	USA : Michigan	43.376762 -86.994278	reads Metavir	?	IDBA_UD	0	
Watkins_LakeMichigan	s57th25Jun13	EnvAquatic_Freshwater	Freshwater	USA : Michigan	43.376762 -86.994278	reads Metavir	?	IDBA_UD	0	
Chow_SaanichInletBC	Saanich10m	EnvAquatic_Marine	Marine water	Canada : British columbia	48.591667 -123.506111	reads Metavir	2 x 250 bp paired-end reads MiSeq v2.0 : avail on Metavir no qual files	Newbler	0	Chow CE. Winget DM. White RA. Hallam SJ. Suttle CA. Combining genomic sequencing methods to explore viral diversity and reveal potential virus-host interactions. Front Microbiol. 2015 Apr 10;6:265. doi: 10.3389/fmicb.2015.00265. eCollection 2015.
Chow_SaanichInletBC	Saanich200m	EnvAquatic_Marine	Marine water	Canada : British columbia	48.591667 -123.506111	reads Metavir	2 x 250 bp paired-end reads MiSeq v2.0 : avail on Metavir no qual files	Newbler	0	
Hurwitz_POV	Dunk	EnvAquatic_Marine	Marine water	Coral sea	-17.933420 146.162830	reads Metavir	454	Newbler	0	
Hurwitz_POV	Fitzroy	EnvAquatic_Marine	Marine water	Coral sea	-16.928549 145.986021	reads Metavir	454	Newbler	0	
Hurwitz_POV	LA26A1397	EnvAquatic_Marine	Marine water	Pacific Ocean	46.956867 -145.666980	reads Metavir	454	Newbler	0	
Hurwitz_POV	LA26D1400	EnvAquatic_Marine	Marine water	Pacific Ocean	46.956867 -145.666980	reads Metavir	454	Newbler	0	
Hurwitz_POV	LA26O1398	EnvAquatic_Marine	Marine water	Pacific Ocean	46.956867 -145.666980	reads Metavir	454	Newbler	0	
Hurwitz_POV	LA26S1396	EnvAquatic_Marine	Marine water	Pacific Ocean	46.956867 -145.666980	reads Metavir	454	Newbler	0	
Hurwitz_POV	LF26A1402	EnvAquatic_Marine	Marine water	Pacific Ocean	46.956867 -145.666980	reads Metavir	454	Newbler	0	
Hurwitz_POV	LF26D1404	EnvAquatic_Marine	Marine water	Pacific Ocean	46.956867 -145.666980	reads Metavir	454	Newbler	0	
Hurwitz_POV	LF26O1403	EnvAquatic_Marine	Marine water	Pacific Ocean	46.956867 -145.666980	reads Metavir	454	Newbler	0	
Hurwitz_POV	LF26S1401	EnvAquatic_Marine	Marine water	Pacific Ocean	46.956867 -145.666980	reads Metavir	454	Newbler	0	
Hurwitz_POV	LJ12A1393	EnvAquatic_Marine	Marine water	Pacific Ocean	46.956867 -145.666980	reads Metavir	454	Newbler	0	
Hurwitz_POV	LJ12D1395	EnvAquatic_Marine	Marine water	Pacific Ocean	46.956867 -145.666980	reads Metavir	454	Newbler	0	
Hurwitz_POV	LJ12O1394	EnvAquatic_Marine	Marine water	Pacific Ocean	46.956867 -145.666980	reads Metavir	454	Newbler	0	
Hurwitz_POV	LJ12S1392	EnvAquatic_Marine	Marine water	Pacific Ocean	46.956867 -145.666980	reads Metavir	454	Newbler	0	
Hurwitz_POV	LJ26D1408	EnvAquatic_Marine	Marine water	Pacific Ocean	46.956867 -145.666980	reads Metavir	454	Newbler	0	
Hurwitz_POV	LJ26O1407	EnvAquatic_Marine	Marine water	Pacific Ocean	46.956867 -145.666980	reads Metavir	454	Newbler	0	Hurwitz BL. Sullivan MB. The Pacific Ocean virome (POV): a marine viral metagenomic dataset and [illegible]

Project Name	Virome Code	Habitat Type (code)	Ecosystem Type (text)	Location	GPS	Source	Sequencing Technology	Assembly Software	# crucivirus	Reference
Hurwitz_POV	LJ26S1405	EnvAquatic_Marine	Marine water	Pacific Ocean	46.956867 -145.666980	reads Metavir	454	Newbler	0	associated protein clusters for quantitative viral ecology. PLoS One. 2013;8(2):e57355. doi: 10.1371/ journal.pone.0057355.
Hurwitz_POV	LJ4A1410	EnvAquatic_Marine	Marine water	Pacific Ocean	46.956867 -145.666980	reads Metavir	454	Newbler	0	
Hurwitz_POV	LJ4D1415	EnvAquatic_Marine	Marine water	Pacific Ocean	46.956867 -145.666980	reads Metavir	454	Newbler	0	
Hurwitz_POV	LJ4O1414	EnvAquatic_Marine	Marine water	Pacific Ocean	46.956867 -145.666980	reads Metavir	454	Newbler	0	
Hurwitz_POV	LJ4S1409	EnvAquatic_Marine	Marine water	Pacific Ocean	46.956867 -145.666980	reads Metavir	454	Newbler	0	
Hurwitz_POV	M1CS1440	EnvAquatic_Marine	Marine water	Pacific Ocean	35.575213 -123.563863	reads Metavir	454	Newbler	0	
Hurwitz_POV	M2MS1438	EnvAquatic_Marine	Marine water	Pacific Ocean	35.575213 -123.563863	reads Metavir	454	Newbler	0	
Hurwitz_POV	M3MD1439	EnvAquatic_Marine	Marine water	Pacific Ocean	35.575213 -123.563863	reads Metavir	454	Newbler	0	
Hurwitz_POV	M4OS1430	EnvAquatic_Marine	Marine water	Pacific Ocean	35.575213 -123.563863	reads Metavir	454	Newbler	0	
Hurwitz_POV	M5OD1431	EnvAquatic_Marine	Marine water	Pacific Ocean	35.575213 -123.563863	reads Metavir	454	Newbler	0	
Hurwitz_POV	M6O1K1432	EnvAquatic_Marine	Marine water	Pacific Ocean	35.575213 -123.563863	reads Metavir	454	Newbler	0	
Hurwitz_POV	M7O4K1433	EnvAquatic_Marine	Marine water	Pacific Ocean	35.575213 -123.563863	reads Metavir	454	Newbler	0	
Hurwitz_POV	SFCS1442	EnvAquatic_Marine	Marine water	Pacific Ocean	32.108588 -118.106031	reads Metavir	454	Newbler	0	
Hurwitz_POV	SFDS1443	EnvAquatic_Marine	Marine water	Pacific Ocean	32.108588 -118.106031	reads Metavir	454	Newbler	0	
Hurwitz_POV	SFSS1444	EnvAquatic_Marine	Marine water	Pacific Ocean	32.108588 -118.106031	reads Metavir	454	Newbler	0	
Hurwitz_POV	STCS1445	EnvAquatic_Marine	Marine water	Pacific Ocean	32.108588 -118.106031	reads Metavir	454	Newbler	0	
McDaniel_inducedProphage	030409X2a	EnvAquatic_Marine	Marine water	USA : Florida	27.694309 -82.636907	reads Moore	454 GS FLX pyrosequencer (Roche)	Newbler	0	McDaniel LD. Rosario K. Breitbart M. Paul JH. Comparative metagenomics: natural populations of induced prophages demonstrate highly unique. lower diversity viral sequences. Environ Microbiol. 2014 Feb;16(2):570-85. doi: 10.1111/1462-2920.12184.
McDaniel_inducedProphage	030409X2i	EnvAquatic_Marine	Marine water	USA : Florida	27.694309 -82.636907	reads Moore	454 GS FLX pyrosequencer (Roche)	Newbler	0	
McDaniel_inducedProphage	tampBay	EnvAquatic_Marine	Marine water	USA : Florida	27.694309 -82.636907	reads Moore	454 GS FLX pyrosequencer (Roche)	Newbler	0	
Williamson_IndianOcean	indOcean1478	EnvAquatic_Marine	Marine water	Indian Ocean	-8.63 95.38	reads Metavir	454	Newbler	0	Williamson SJ. Allen LZ. Lorenzi HA. Fadrosh DW. Brami D. Thiagarajan M. McCrow JP. Tovchigrechko A. Yooseph S. Venter JC. Metagenomic exploration of viruses throughout the Indian Ocean. PLoS One. 2012;7(10):e42047. doi: 10.1371/journal.pone.0042047.
Williamson_IndianOcean	indOcean1477	EnvAquatic_Marine	Marine water	Indian Ocean	-8.15 80.38	reads Metavir	454	Newbler	0	
Williamson_IndianOcean	indOcean1479	EnvAquatic_Marine	Marine water	Indian Ocean	-8.14 54.21	reads Metavir	454	Newbler	0	
Williamson_IndianOcean	indOcean1480	EnvAquatic_Marine	Marine water	Indian Ocean	-29.55 40.68	reads Metavir	454	Newbler	0	
Adriaenssens_NamibDesertSaltPans	Gobabeb	EnvAquatic_Saline	Hypersaline	Namibia : Gobabeb	-23.507153 15.07125	contigs NCBI	MiSeq	CLC 99 %	0	Adriaenssens EM. van Zyl LJ. Cowan DA. Trindade MI. Metaviromics of Namib Desert Salt Pans: A Novel Lineage of Haloarchaeal Salterproviruses and a Rich Source of ssDNA Viruses. Viruses. 2016 Jan 8;8(1). pii: E14. doi: 10.3390/v8010014.
Adriaenssens_NamibDesertSaltPans	Swakopmund	EnvAquatic_Saline	Hypersaline	Namibia : Dorob national park	-22.484728 14.571683	contigs NCBI	MiSeq	CLC 99 %	0	
Adriaenssens_NamibHypoliths	hypolith	EnvAquatic_Saline	Hypersaline	Namibia : Gobabeb	-23.561111 15.041389	reads SRA	Illumina	IDBA_UD	0	Adriaenssens EM. Van Zyl L. De Maayer P. Rubagotti E. Rybicki E. Tuffin M. Cowan DA. Metagenomic analysis of the viral community in Namib Desert hypoliths. Environ Microbiol. 2015 Feb;17(2):480-95. Doi: 10.1111/1462-2920.12528. &&&& Zablocki O1. Adriaenssens EM1. Cowan D2. Diversity and Ecology of Viruses in Hyperarid Desert Soils. Appl Environ Microbiol. 2015 Nov 20;82(3):770-7. doi: 10.1128/AEM.02651-15.
GarciaHeredia_SalineFosmids	SalFosmids	EnvAquatic_Saline	Hypersaline	Spain : Santa Pola	38.191242 -0.615222	contigs NCBI	?	?	0	Garcia-Heredia I. Martin-Cuadrado AB. Mojica FJ. Santos F. Mira A. Antón J. Rodriguez-Valera F. Reconstructing viral genomes from the environment using fosmid clones: the case of haloviruses. PLoS One. 2012;7(3):e33802. doi: 10.1371/journal.pone.0033802.
Pacton_LagoaVermelhaOrganoMineral	Lagoa	EnvAquatic_Saline		Brazil	-23.083000 -43.950000	reads Metavir	454	Newbler	0	Pacton M. Wacey D. Corinaldesi C. Tangherlini M. Kilburn MR. Gorin GE. Danovaro R. Vasconcelos C. Viruses as new agents of organomineralization in the geological record. Nat Commun. 2014 Jul 3;5:4298. doi: 10.1038/ncomms5298.
Roux_Archevir	P2Saloon	EnvAquatic_Saline	Saline	Senegal	14.131228 -16.471595	contigs Metavir	Illumina HiSeq2000	IDBA_UD	0	Roux S. Enault F. Ravet V. Colombet J. Bettarel Y. Auguet JC. Bouvier T. Lucas-Staat S. Vellet A. Prangishvili D. Forterre P. Debroas D. Sime-Ngando T. Analysis of metagenomic data reveals common features of halophilic viral communities across continents. Environ Microbiol. 2015 Oct 16. doi: 10.1111/1462-2920.13084.
Roux_Archevir	P5Ngallou	EnvAquatic_Saline	Hypersaline	Senegal	14.077154 -16.737001	contigs Metavir	Illumina HiSeq2000	IDBA_UD	0	
Roux_Archevir	P6Retba	EnvAquatic_Saline	Hypersaline	Senegal	14.837910 -17.234274	contigs Metavir	Illumina HiSeq2000	IDBA_UD	0	
Roux_Archevir	P7Ngallou	EnvAquatic_Saline	Hypersaline	Senegal	14.077154 -16.737001	contigs Metavir	Illumina HiSeq2000	IDBA_UD	0	
Roux_Archevir	P8Ngallou	EnvAquatic_Saline	Hypersaline	Senegal	14.077154 -16.737001	contigs Metavir	Illumina HiSeq2000	IDBA_UD	0	
Roux_Archevir	P9Ngallou	EnvAquatic_Saline	Hypersaline	Senegal	14.077154 -16.737001	contigs Metavir	Illumina HiSeq2000	IDBA_UD	0	
Bolduc_YellowstoneHotSpring	NL10X0809	EnvAquatic_ThermalSprings	Hot spring	USA : Yellowstone national park	44.753600 -110.723700	contigs NCBI	454 GS FLX	Newbler	0	

Project Name	Virome Code	Habitat Type (code)	Ecosystem Type (text)	Location	GPS	Source	Sequencing Technology	Assembly Software	# crucivirus	Reference
Bolduc_Yellowstone HotSpring	NL10X0901	EnvAquatic_ThermalSprings	Hot spring	USA : Yellowstone national park	44.753600 -110.723700	contigs NCBI	454 GS FLX	Newbler	0	Bolduc B. Wirth JF. Mazurie A. Young MJ. Viral assemblage composition in Yellowstone acidic hot springs assessed by network analysis. ISME J. 2015 Oct;9(10):2162-77. doi: 10.1038/ismej.2015.28.
Bolduc_Yellowstone HotSpring	NL10X0903	EnvAquatic_ThermalSprings	Hot spring	USA : Yellowstone national park	44.753600 -110.723700	contigs NCBI	454 GS FLX	Newbler	0	
Bolduc_Yellowstone HotSpring	NL10X0904	EnvAquatic_ThermalSprings	Hot spring	USA : Yellowstone national park	44.753600 -110.723700	contigs NCBI	454 GS FLX	Newbler	0	
Bolduc_Yellowstone HotSpring	NL10X0908	EnvAquatic_ThermalSprings	Hot spring	USA : Yellowstone national park	44.753600 -110.723700	contigs NCBI	454 GS FLX	Newbler	0	
Bolduc_Yellowstone HotSpring	NL10X0909	EnvAquatic_ThermalSprings	Hot spring	USA : Yellowstone national park	44.753600 -110.723700	contigs NCBI	454 GS FLX	Newbler	0	
Bolduc_Yellowstone HotSpring	NL10X0910	EnvAquatic_ThermalSprings	Hot spring	USA : Yellowstone national park	44.753600 -110.723700	contigs NCBI	454 GS FLX	Newbler	0	
Bolduc_Yellowstone HotSpring	NL10X1002	EnvAquatic_ThermalSprings	Hot spring	USA : Yellowstone national park	44.753600 -110.723700	contigs NCBI	454 GS FLX	Newbler	0	
Bolduc_Yellowstone HotSpring	NL10X1006	EnvAquatic_ThermalSprings	Hot spring	USA : Yellowstone national park	44.753600 -110.723700	contigs NCBI	454 GS FLX	Newbler	0	
Bolduc_Yellowstone HotSpring	NL10X1X3	EnvAquatic_ThermalSprings	Hot spring	USA : Yellowstone national park	44.753600 -110.723700	contigs NCBI	MiSeq	IDBA_UD	0	
Bolduc_Yellowstone HotSpring	NL10X1X4	EnvAquatic_ThermalSprings	Hot spring	USA : Yellowstone national park	44.753600 -110.723700	contigs NCBI	MiSeq	IDBA_UD	0	
Bolduc_Yellowstone HotSpring	NL10X1X5	EnvAquatic_ThermalSprings	Hot spring	USA : Yellowstone national park	44.753600 -110.723700	contigs NCBI	MiSeq	IDBA_UD	0	
Anderson_HydrothermalVents	G2810	EnvAquatic_Vents	Hydrothermal Vents	Pacific Ocean	45.999969 -129.999700	reads Moore	454 GS FLX	Newbler	0	Anderson RE. Brazelton WJ. Baross JA (2011) Using CRISPRs as a metagenomic tool to identify microbial hosts of a diffuse flow hydrothermal vent viral assemblage. FEMS Microbiology Ecology 77(1): 120-133.
Appelt_14thCoprolite	Coprolite	EnvTerrestrial_Other		Belgium : Namur	50.466667 4.866667	reads Metavir	454 GS FLX	IDBA_UD	0	Appelt S1. Fancello L. Le Bailly M. Raoult D. Drancourt M. Desnues C. Viruses in a 14th-century coprolite. Appl Environ Microbiol. 2014 May;80(9):2648-55. doi: 10.1128/AEM.03242-13.
Reavy_ssDNAIn2Soils	brownEarth	EnvTerrestrial_Soil	soil	Scotland	55.279472 -3.8906851	reads MG-RAST	454	Newbler	0	Reavy B. Swanson MM. Cock PJ. Dawson L. Freitag TE. Singh BK. Torrance L. Mushegian AR. Taliansky M. Distinct circular single-stranded DNA viruses exist in different soil types. Appl Environ Microbiol. 2015 Jun 15;81(12):3934-45. doi: 10.1128/AEM.03878-14. Epub 2015 Apr 3.
Reavy_ssDNAIn2Soils	machair	EnvTerrestrial_Soil	soil	Scotland	56.599975 -6.6453090	reads MG-RAST	454	Newbler	0	
Zablocki_AntarcticSoils	Antarchypolith	EnvTerrestrial_Soil	Polar soil	Antarctica	-78.093333 163.810000	contigs Metavir		CLC	0	Zablocki O. van Zyl L. Adriaenssens EM. Rubagotti E. Tuffin M. Cary SC. Cowan D. High-level diversity of tailed phages. eukaryote-associated viruses. and virophage-like elements in the metaviromes of antarctic soils. Appl Environ Microbiol. 2014 Nov;80(22):6888-97. doi: 10.1128/AEM.01525-14. Epub 2014 Aug 29.
Zablocki_AntarcticSoils	AntarcSoils	EnvTerrestrial_Soil	Polar soil	Antarctica	-78.093333 163.810000	contigs Metavir		CLC	0	
Carlos_CoralBrazil	Mdecactis	HostAssoc_Cnidaria	Coral	Brazil : Buzios Island	-22.748090 -41.881290	reads MG-RAST	454	Newbler	0	Carlos C. Castro DB. Ottoboni LM. Comparative metagenomic analysis of coral microbial communities using a reference-independent approach. PLoS One. 2014 Nov 7;9(11):e111626. doi: 10.1371/journal.pone.0111626. eCollection 2014.
Carlos_CoralBrazil	Mhispida	HostAssoc_Cnidaria	Coral	Brazil : Buzios Island	-22.748090 -41.881290	reads MG-RAST	454	Newbler	0	
Correa_CoralBleaching	coralBleach	HostAssoc_Cnidaria	Coral	Australia : Heron Island	-23.443075 151.908442	reads ENA	MiSeq	IDBA_UD	0	AM Correa. TD Ainsworth. SM Rosales. AR Thurber. CR Butler and RL Vega Thurber. Viral outbreak in corals associated with an in situ bleaching event: atypical herpes-like viruses and a new megavirus infecting Symbiodinium. Front. Microbiol. I doi: 10.3389/fmicb.2016.00127
Soffer_CoralPlague	A5Coral853	HostAssoc_Cnidaria	Coral	USA : Virgin Islands	18.342267 -64.984840	reads Metavir	454 GS FLX	Newbler	0	Soffer N. Brandt ME. Correa AM. Smith TB. Thurber RV. Potential role of viruses in white plague coral disease. ISME J. 2014 Feb;8(2):271-83. doi: 10.1038/ismej.2013.137. Epub 2013 Aug 15.
Soffer_CoralPlague	A10Coral879	HostAssoc_Cnidaria	Coral	USA : Virgin Islands	18.342267 -64.984840	reads Metavir	454 GS FLX	Newbler	0	
Soffer_CoralPlague	A2Coral851	HostAssoc_Cnidaria	Coral	USA : Virgin Islands	18.342267 -64.984840	reads Metavir	454 GS FLX	Newbler	0	
Soffer_CoralPlague	A6Coral854	HostAssoc_Cnidaria	Coral	USA : Virgin Islands	18.342267 -64.984840	reads Metavir	454 GS FLX	Newbler	0	
Soffer_CoralPlague	D10Coral886	HostAssoc_Cnidaria	Coral	USA : Virgin Islands	18.342267 -64.984840	reads Metavir	454 GS FLX	Newbler	0	
Soffer_CoralPlague	D1Coral880	HostAssoc_Cnidaria	Coral	USA : Virgin Islands	18.342267 -64.984840	reads Metavir	454 GS FLX	Newbler	0	
Soffer_CoralPlague	D2Coral881	HostAssoc_Cnidaria	Coral	USA : Virgin Islands	18.342267 -64.984840	reads Metavir	454 GS FLX	Newbler	0	
Soffer_CoralPlague	D3Coral882	HostAssoc_Cnidaria	Coral	USA : Virgin Islands	18.342267 -64.984840	reads Metavir	454 GS FLX	Newbler	0	
Soffer_CoralPlague	D6Coral884	HostAssoc_Cnidaria	Coral	USA : Virgin Islands	18.342267 -64.984840	reads Metavir	454 GS FLX	Newbler	0	
Soffer_CoralPlague	H10Coral892	HostAssoc_Cnidaria	Coral	USA : Virgin Islands	18.342267 -64.984840	reads Metavir	454 GS FLX	Newbler	0	
Soffer_CoralPlague	SWdCoral895	HostAssoc_Cnidaria	Coral	USA : Virgin Islands	18.342267 -64.984840	reads Metavir	454 GS FLX	Newbler	1	
Soffer_CoralPlague	swHCoral896	HostAssoc_Cnidaria	Coral	USA : Virgin Islands	18.342267 -64.984840	reads Metavir	454 GS FLX	Newbler	0	
Soffer_CoralPlague	Ub2Coral894	HostAssoc_Cnidaria	Coral	USA : Virgin Islands	18.342267 -64.984840	reads Metavir	454 GS FLX	Newbler	0	
Weynberg_7coral	AtenuisDSISPA	HostAssoc_Cnidaria	Coral	Coral sea	-18.496944 146.596111	reads SRA		Newbler	0	
Weynberg_7coral	AtenuisRSISPA	HostAssoc_Cnidaria	Coral	Coral sea	-18.496944 146.596111	reads SRA		Newbler	0	
Weynberg_7coral	PdamicornisCFM	HostAssoc_Cnidaria	Coral	Coral sea	-18.496944 146.596111	reads SRA		Newbler	0	Weynberg KD. Wood-Charlson EM. Suttle CA. van Oppen

Project Name	Virome Code	Habitat Type (code)	Ecosystem Type (text)	Location	GPS	Source	Sequencing Technology	Assembly Software	# crucivirus	Reference
Weynberg_7coral	PdamicornisLN2	HostAssoc_Cnidaria	Coral	Coral sea	-18.496944 146.596111	reads SRA		Newbler	0	MJ. Generating viral metagenomes from the coral holobiont. Front Microbiol. 2014 May 7;5:206. doi: 10.3389/fmicb.2014.00206.
Weynberg_7coral	PdamicornisLN2S	HostAssoc_Cnidaria	Coral	Coral sea	-18.496944 146.596111	reads SRA		Newbler	0	
Weynberg_7coral	PdamicornisNLN	HostAssoc_Cnidaria	Coral	Coral sea	-18.496944 146.596111	reads SRA		Newbler	0	
Weynberg_7coral	PdamicornisNLNS	HostAssoc_Cnidaria	Coral	Coral sea	-18.496944 146.596111	reads SRA		Newbler	0	
WoodCharlson_CoralsRNA	CoralCtrlRNA	HostAssoc_Cnidaria	Coral	USA : Florida	24.541370 -81.790761	reads Metavir	454	Newbler	0	Wood-Charlson EM. Weynberg KD. Suttle CA. Roux S. van Oppen MJ. Metagenomic characterisation of viral communities in corals: Mining biological signal from methodological noise. Environ Microbiol Rep. 2015 Feb 13. doi: 10.1111/1758-2229.12275.
WoodCharlson_CoralsRNA	CoralStressRNA	HostAssoc_Cnidaria	Coral	USA : Florida	24.541370 -81.790761	reads Metavir	454	Newbler	0	
Lim_EarlyLifeDynamics	A109167M	HostAssoc_Human Feces	Human feces	USA : Missouri	38.130000 -90.000000	reads SRA	2 x 250 paired-end, MiSeq v2	Newbler	0	
Lim_EarlyLifeDynamics	A109167S	HostAssoc_Human Feces	Human feces	USA : Missouri	38.130000 -90.000000	reads SRA	2 x 250 paired-end, MiSeq v2	Newbler	0	
Lim_EarlyLifeDynamics	A1120834M	HostAssoc_Human Feces	Human feces	USA : Missouri	38.130000 -90.000000	reads SRA	2 x 250 paired-end, MiSeq v2	Newbler	0	
Lim_EarlyLifeDynamics	A1120834S	HostAssoc_Human Feces	Human feces	USA : Missouri	38.130000 -90.000000	reads SRA	2 x 250 paired-end, MiSeq v2	Newbler	0	
Lim_EarlyLifeDynamics	A1186364M	HostAssoc_Human Feces	Human feces	USA : Missouri	38.130000 -90.000000	reads SRA	2 x 250 paired-end, MiSeq v2	Newbler	0	
Lim_EarlyLifeDynamics	A1186364S	HostAssoc_Human Feces	Human feces	USA : Missouri	38.130000 -90.000000	reads SRA	2 x 250 paired-end, MiSeq v2	Newbler	0	
Lim_EarlyLifeDynamics	A1246377M	HostAssoc_Human Feces	Human feces	USA : Missouri	38.130000 -90.000000	reads SRA	2 x 250 paired-end, MiSeq v2	Newbler	0	
Lim_EarlyLifeDynamics	A1246377S	HostAssoc_Human Feces	Human feces	USA : Missouri	38.130000 -90.000000	reads SRA	2 x 250 paired-end, MiSeq v2	Newbler	0	
Lim_EarlyLifeDynamics	A133483M	HostAssoc_Human Feces	Human feces	USA : Missouri	38.130000 -90.000000	reads SRA	2 x 250 paired-end, MiSeq v2	Newbler	0	
Lim_EarlyLifeDynamics	A133483S	HostAssoc_Human Feces	Human feces	USA : Missouri	38.130000 -90.000000	reads SRA	2 x 250 paired-end, MiSeq v2	Newbler	0	
Lim_EarlyLifeDynamics	A163707M	HostAssoc_Human Feces	Human feces	USA : Missouri	38.130000 -90.000000	reads SRA	2 x 250 paired-end, MiSeq v2	Newbler	0	
Lim_EarlyLifeDynamics	A163707S	HostAssoc_Human Feces	Human feces	USA : Missouri	38.130000 -90.000000	reads SRA	2 x 250 paired-end, MiSeq v2	Newbler	0	
Lim_EarlyLifeDynamics	A208584M	HostAssoc_Human Feces	Human feces	USA : Missouri	38.130000 -90.000000	reads SRA	2 x 250 paired-end, MiSeq v2	Newbler	0	
Lim_EarlyLifeDynamics	A208584S	HostAssoc_Human Feces	Human feces	USA : Missouri	38.130000 -90.000000	reads SRA	2 x 250 paired-end, MiSeq v2	Newbler	0	
Lim_EarlyLifeDynamics	A2120860M	HostAssoc_Human Feces	Human feces	USA : Missouri	38.130000 -90.000000	reads SRA	2 x 250 paired-end, MiSeq v2	Newbler	0	
Lim_EarlyLifeDynamics	A2120860S	HostAssoc_Human Feces	Human feces	USA : Missouri	38.130000 -90.000000	reads SRA	2 x 250 paired-end, MiSeq v2	Newbler	0	
Lim_EarlyLifeDynamics	A2186574M	HostAssoc_Human Feces	Human feces	USA : Missouri	38.130000 -90.000000	reads SRA	2 x 250 paired-end, MiSeq v2	Newbler	0	
Lim_EarlyLifeDynamics	A2186574S	HostAssoc_Human Feces	Human feces	USA : Missouri	38.130000 -90.000000	reads SRA	2 x 250 paired-end, MiSeq v2	Newbler	0	
Lim_EarlyLifeDynamics	A2246583M	HostAssoc_Human Feces	Human feces	USA : Missouri	38.130000 -90.000000	reads SRA	2 x 250 paired-end, MiSeq v2	Newbler	0	
Lim_EarlyLifeDynamics	A2246583S	HostAssoc_Human Feces	Human feces	USA : Missouri	38.130000 -90.000000	reads SRA	2 x 250 paired-end, MiSeq v2	Newbler	0	
Lim_EarlyLifeDynamics	A233467M	HostAssoc_Human Feces	Human feces	USA : Missouri	38.130000 -90.000000	reads SRA	2 x 250 paired-end, MiSeq v2	Newbler	0	
Lim_EarlyLifeDynamics	A233467S	HostAssoc_Human Feces	Human feces	USA : Missouri	38.130000 -90.000000	reads SRA	2 x 250 paired-end, MiSeq v2	Newbler	0	
Lim_EarlyLifeDynamics	A263710M	HostAssoc_Human Feces	Human feces	USA : Missouri	38.130000 -90.000000	reads SRA	2 x 250 paired-end, MiSeq v2	Newbler	0	
Lim_EarlyLifeDynamics	A263710S	HostAssoc_Human Feces	Human feces	USA : Missouri	38.130000 -90.000000	reads SRA	2 x 250 paired-end, MiSeq v2	Newbler	0	
Lim_EarlyLifeDynamics	B108199M	HostAssoc_Human Feces	Human feces	USA : Missouri	38.130000 -90.000000	reads SRA	2 x 250 paired-end, MiSeq v2	Newbler	0	
Lim_EarlyLifeDynamics	B108199S	HostAssoc_Human Feces	Human feces	USA : Missouri	38.130000 -90.000000	reads SRA	2 x 250 paired-end, MiSeq v2	Newbler	0	
Lim_EarlyLifeDynamics	B1123480M	HostAssoc_Human Feces	Human feces	USA : Missouri	38.130000 -90.000000	reads SRA	2 x 250 paired-end, MiSeq v2	Newbler	0	
Lim_EarlyLifeDynamics	B1123480S	HostAssoc_Human Feces	Human feces	USA : Missouri	38.130000 -90.000000	reads SRA	2 x 250 paired-end, MiSeq v2	Newbler	0	
Lim_EarlyLifeDynamics	B1181568M	HostAssoc_Human Feces	Human feces	USA : Missouri	38.130000 -90.000000	reads SRA	2 x 250 paired-end, MiSeq v2	Newbler	0	
Lim_EarlyLifeDynamics	B1181568S	HostAssoc_Human Feces	Human feces	USA : Missouri	38.130000 -90.000000	reads SRA	2 x 250 paired-end, MiSeq v2	Newbler	0	
Lim_EarlyLifeDynamics	B1246604M	HostAssoc_Human Feces	Human feces	USA : Missouri	38.130000 -90.000000	reads SRA	2 x 250 paired-end, MiSeq v2	Newbler	0	
Lim_EarlyLifeDynamics	B1246604S	HostAssoc_Human Feces	Human feces	USA : Missouri	38.130000 -90.000000	reads SRA	2 x 250 paired-end, MiSeq v2	Newbler	0	
Lim_EarlyLifeDynamics	B134708M	HostAssoc_Human Feces	Human feces	USA : Missouri	38.130000 -90.000000	reads SRA	2 x 250 paired-end, MiSeq v2	Newbler	0	
Lim_EarlyLifeDynamics	B134708S	HostAssoc_Human Feces	Human feces	USA : Missouri	38.130000 -90.000000	reads SRA	2 x 250 paired-end, MiSeq v2	Newbler	0	
Lim_EarlyLifeDynamics	B160912M	HostAssoc_Human Feces	Human feces	USA : Missouri	38.130000 -90.000000	reads SRA	2 x 250 paired-end, MiSeq v2	Newbler	0	
Lim_EarlyLifeDynamics	B160912S	HostAssoc_Human Feces	Human feces	USA : Missouri	38.130000 -90.000000	reads SRA	2 x 250 paired-end, MiSeq v2	Newbler	0	

Project Name	Virome Code	Habitat Type (code)	Ecosystem Type (text)	Location	GPS	Source	Sequencing Technology	Assembly Software	# crucivirus	Reference
Lim_EarlyLifeDynamics	B208680M	HostAssoc_Human Feces	Human feces	USA : Missouri	38.130000 -90.000000	reads SRA	2 x 250 paired-end, MiSeq v2	Newbler	0	Lim ES. Zhou Y. Zhao G. Bauer IK. Droit L. Ndao IM. Warner BB. Tarr PI. Wang D. Holtz LR. Early life dynamics of the human gut virome and bacterial microbiome in infants. Nat Med. 2015 Oct;21(10):1228-34. doi: 10.1038/nm.3950.
Lim_EarlyLifeDynamics	B208680S	HostAssoc_Human Feces	Human feces	USA : Missouri	38.130000 -90.000000	reads SRA	2 x 250 paired-end, MiSeq v2	Newbler	0	
Lim_EarlyLifeDynamics	B2120802M	HostAssoc_Human Feces	Human feces	USA : Missouri	38.130000 -90.000000	reads SRA	2 x 250 paired-end, MiSeq v2	Newbler	0	
Lim_EarlyLifeDynamics	B2120802S	HostAssoc_Human Feces	Human feces	USA : Missouri	38.130000 -90.000000	reads SRA	2 x 250 paired-end, MiSeq v2	Newbler	0	
Lim_EarlyLifeDynamics	B2181797M	HostAssoc_Human Feces	Human feces	USA : Missouri	38.130000 -90.000000	reads SRA	2 x 250 paired-end, MiSeq v2	Newbler	0	
Lim_EarlyLifeDynamics	B2181797S	HostAssoc_Human Feces	Human feces	USA : Missouri	38.130000 -90.000000	reads SRA	2 x 250 paired-end, MiSeq v2	Newbler	0	
Lim_EarlyLifeDynamics	B2241824M	HostAssoc_Human Feces	Human feces	USA : Missouri	38.130000 -90.000000	reads SRA	2 x 250 paired-end, MiSeq v2	Newbler	0	
Lim_EarlyLifeDynamics	B2241824S	HostAssoc_Human Feces	Human feces	USA : Missouri	38.130000 -90.000000	reads SRA	2 x 250 paired-end, MiSeq v2	Newbler	0	
Lim_EarlyLifeDynamics	B234724M	HostAssoc_Human Feces	Human feces	USA : Missouri	38.130000 -90.000000	reads SRA	2 x 250 paired-end, MiSeq v2	Newbler	0	
Lim_EarlyLifeDynamics	B234724S	HostAssoc_Human Feces	Human feces	USA : Missouri	38.130000 -90.000000	reads SRA	2 x 250 paired-end, MiSeq v2	Newbler	0	
Lim_EarlyLifeDynamics	B263463M	HostAssoc_Human Feces	Human feces	USA : Missouri	38.130000 -90.000000	reads SRA	2 x 250 paired-end, MiSeq v2	Newbler	0	
Lim_EarlyLifeDynamics	B263463S	HostAssoc_Human Feces	Human feces	USA : Missouri	38.130000 -90.000000	reads SRA	2 x 250 paired-end, MiSeq v2	Newbler	0	
Lim_EarlyLifeDynamics	C100245M	HostAssoc_Human Feces	Human feces	USA : Missouri	38.130000 -90.000000	reads SRA	2 x 250 paired-end, MiSeq v2	Newbler	0	
Lim_EarlyLifeDynamics	C100245S	HostAssoc_Human Feces	Human feces	USA : Missouri	38.130000 -90.000000	reads SRA	2 x 250 paired-end, MiSeq v2	Newbler	0	
Lim_EarlyLifeDynamics	C1129721M	HostAssoc_Human Feces	Human feces	USA : Missouri	38.130000 -90.000000	reads SRA	2 x 250 paired-end, MiSeq v2	Newbler	0	
Lim_EarlyLifeDynamics	C1129721S	HostAssoc_Human Feces	Human feces	USA : Missouri	38.130000 -90.000000	reads SRA	2 x 250 paired-end, MiSeq v2	Newbler	0	
Lim_EarlyLifeDynamics	C1181815M	HostAssoc_Human Feces	Human feces	USA : Missouri	38.130000 -90.000000	reads SRA	2 x 250 paired-end, MiSeq v2	Newbler	0	
Lim_EarlyLifeDynamics	C1181815S	HostAssoc_Human Feces	Human feces	USA : Missouri	38.130000 -90.000000	reads SRA	2 x 250 paired-end, MiSeq v2	Newbler	0	
Lim_EarlyLifeDynamics	C1246608M	HostAssoc_Human Feces	Human feces	USA : Missouri	38.130000 -90.000000	reads SRA	2 x 250 paired-end, MiSeq v2	Newbler	0	
Lim_EarlyLifeDynamics	C1246608S	HostAssoc_Human Feces	Human feces	USA : Missouri	38.130000 -90.000000	reads SRA	2 x 250 paired-end, MiSeq v2	Newbler	0	
Lim_EarlyLifeDynamics	C130019M	HostAssoc_Human Feces	Human feces	USA : Missouri	38.130000 -90.000000	reads SRA	2 x 250 paired-end, MiSeq v2	Newbler	0	
Lim_EarlyLifeDynamics	C130019S	HostAssoc_Human Feces	Human feces	USA : Missouri	38.130000 -90.000000	reads SRA	2 x 250 paired-end, MiSeq v2	Newbler	0	
Lim_EarlyLifeDynamics	C161171M	HostAssoc_Human Feces	Human feces	USA : Missouri	38.130000 -90.000000	reads SRA	2 x 250 paired-end, MiSeq v2	Newbler	0	
Lim_EarlyLifeDynamics	C161171S	HostAssoc_Human Feces	Human feces	USA : Missouri	38.130000 -90.000000	reads SRA	2 x 250 paired-end, MiSeq v2	Newbler	0	
Lim_EarlyLifeDynamics	C200238M	HostAssoc_Human Feces	Human feces	USA : Missouri	38.130000 -90.000000	reads SRA	2 x 250 paired-end, MiSeq v2	Newbler	0	
Lim_EarlyLifeDynamics	C2129723M	HostAssoc_Human Feces	Human feces	USA : Missouri	38.130000 -90.000000	reads SRA	2 x 250 paired-end, MiSeq v2	Newbler	0	
Lim_EarlyLifeDynamics	C2129723S	HostAssoc_Human Feces	Human feces	USA : Missouri	38.130000 -90.000000	reads SRA	2 x 250 paired-end, MiSeq v2	Newbler	0	
Lim_EarlyLifeDynamics	C2186333M	HostAssoc_Human Feces	Human feces	USA : Missouri	38.130000 -90.000000	reads SRA	2 x 250 paired-end, MiSeq v2	Newbler	0	
Lim_EarlyLifeDynamics	C2186333S	HostAssoc_Human Feces	Human feces	USA : Missouri	38.130000 -90.000000	reads SRA	2 x 250 paired-end, MiSeq v2	Newbler	0	
Lim_EarlyLifeDynamics	C2249052M	HostAssoc_Human Feces	Human feces	USA : Missouri	38.130000 -90.000000	reads SRA	2 x 250 paired-end, MiSeq v2	Newbler	0	
Lim_EarlyLifeDynamics	C2249052S	HostAssoc_Human Feces	Human feces	USA : Missouri	38.130000 -90.000000	reads SRA	2 x 250 paired-end, MiSeq v2	Newbler	0	
Lim_EarlyLifeDynamics	C233337M	HostAssoc_Human Feces	Human feces	USA : Missouri	38.130000 -90.000000	reads SRA	2 x 250 paired-end, MiSeq v2	Newbler	0	
Lim_EarlyLifeDynamics	C233337S	HostAssoc_Human Feces	Human feces	USA : Missouri	38.130000 -90.000000	reads SRA	2 x 250 paired-end, MiSeq v2	Newbler	0	
Lim_EarlyLifeDynamics	C260838M	HostAssoc_Human Feces	Human feces	USA : Missouri	38.130000 -90.000000	reads SRA	2 x 250 paired-end, MiSeq v2	Newbler	0	
Lim_EarlyLifeDynamics	C260838S	HostAssoc_Human Feces	Human feces	USA : Missouri	38.130000 -90.000000	reads SRA	2 x 250 paired-end, MiSeq v2	Newbler	0	
Lim_EarlyLifeDynamics	D103848M	HostAssoc_Human Feces	Human feces	USA : Missouri	38.130000 -90.000000	reads SRA	2 x 250 paired-end, MiSeq v2	Newbler	0	
Lim_EarlyLifeDynamics	D103848S	HostAssoc_Human Feces	Human feces	USA : Missouri	38.130000 -90.000000	reads SRA	2 x 250 paired-end, MiSeq v2	Newbler	0	
Lim_EarlyLifeDynamics	D1128367M	HostAssoc_Human Feces	Human feces	USA : Missouri	38.130000 -90.000000	reads SRA	2 x 250 paired-end, MiSeq v2	Newbler	0	
Lim_EarlyLifeDynamics	D1128367S	HostAssoc_Human Feces	Human feces	USA : Missouri	38.130000 -90.000000	reads SRA	2 x 250 paired-end, MiSeq v2	Newbler	0	
Lim_EarlyLifeDynamics	D1188340M	HostAssoc_Human Feces	Human feces	USA : Missouri	38.130000 -90.000000	reads SRA	2 x 250 paired-end, MiSeq v2	Newbler	0	
Lim_EarlyLifeDynamics	D1188340S	HostAssoc_Human Feces	Human feces	USA : Missouri	38.130000 -90.000000	reads SRA	2 x 250 paired-end, MiSeq v2	Newbler	0	
Lim_EarlyLifeDynamics	D1247512M	HostAssoc_Human Feces	Human feces	USA : Missouri	38.130000 -90.000000	reads SRA	2 x 250 paired-end, MiSeq v2	Newbler	0	

Project Name	Virome Code	Habitat Type (code)	Ecosystem Type (text)	Location	GPS	Source	Sequencing Technology	Assembly Software	# crucivirus	Reference
Lim_EarlyLifeDynamics	D1247512S	HostAssoc_Human Feces	Human feces	USA : Missouri	38.130000 -90.000000	reads SRA	2 x 250 paired-end, MiSeq v2	Newbler	0	
Lim_EarlyLifeDynamics	D132131M	HostAssoc_Human Feces	Human feces	USA : Missouri	38.130000 -90.000000	reads SRA	2 x 250 paired-end, MiSeq v2	Newbler	0	
Lim_EarlyLifeDynamics	D132131S	HostAssoc_Human Feces	Human feces	USA : Missouri	38.130000 -90.000000	reads SRA	2 x 250 paired-end, MiSeq v2	Newbler	0	
Lim_EarlyLifeDynamics	D166691M	HostAssoc_Human Feces	Human feces	USA : Missouri	38.130000 -90.000000	reads SRA	2 x 250 paired-end, MiSeq v2	Newbler	0	
Lim_EarlyLifeDynamics	D166691S	HostAssoc_Human Feces	Human feces	USA : Missouri	38.130000 -90.000000	reads SRA	2 x 250 paired-end, MiSeq v2	Newbler	0	
Lim_EarlyLifeDynamics	D203840M	HostAssoc_Human Feces	Human feces	USA : Missouri	38.130000 -90.000000	reads SRA	2 x 250 paired-end, MiSeq v2	Newbler	0	
Lim_EarlyLifeDynamics	D203840S	HostAssoc_Human Feces	Human feces	USA : Missouri	38.130000 -90.000000	reads SRA	2 x 250 paired-end, MiSeq v2	Newbler	0	
Lim_EarlyLifeDynamics	D2122062M	HostAssoc_Human Feces	Human feces	USA : Missouri	38.130000 -90.000000	reads SRA	2 x 250 paired-end, MiSeq v2	Newbler	0	
Lim_EarlyLifeDynamics	D2122062S	HostAssoc_Human Feces	Human feces	USA : Missouri	38.130000 -90.000000	reads SRA	2 x 250 paired-end, MiSeq v2	Newbler	0	
Lim_EarlyLifeDynamics	D2188216M	HostAssoc_Human Feces	Human feces	USA : Missouri	38.130000 -90.000000	reads SRA	2 x 250 paired-end, MiSeq v2	Newbler	0	
Lim_EarlyLifeDynamics	D2188216S	HostAssoc_Human Feces	Human feces	USA : Missouri	38.130000 -90.000000	reads SRA	2 x 250 paired-end, MiSeq v2	Newbler	0	
Lim_EarlyLifeDynamics	D2247517M	HostAssoc_Human Feces	Human feces	USA : Missouri	38.130000 -90.000000	reads SRA	2 x 250 paired-end, MiSeq v2	Newbler	0	
Lim_EarlyLifeDynamics	D2247517S	HostAssoc_Human Feces	Human feces	USA : Missouri	38.130000 -90.000000	reads SRA	2 x 250 paired-end, MiSeq v2	Newbler	0	
Lim_EarlyLifeDynamics	D232028M	HostAssoc_Human Feces	Human feces	USA : Missouri	38.130000 -90.000000	reads SRA	2 x 250 paired-end, MiSeq v2	Newbler	0	
Lim_EarlyLifeDynamics	D232028S	HostAssoc_Human Feces	Human feces	USA : Missouri	38.130000 -90.000000	reads SRA	2 x 250 paired-end, MiSeq v2	Newbler	0	
Lim_EarlyLifeDynamics	D268614M	HostAssoc_Human Feces	Human feces	USA : Missouri	38.130000 -90.000000	reads SRA	2 x 250 paired-end, MiSeq v2	Newbler	0	
Lim_EarlyLifeDynamics	D268614S	HostAssoc_Human Feces	Human feces	USA : Missouri	38.130000 -90.000000	reads SRA	2 x 250 paired-end, MiSeq v2	Newbler	0	
Minot_RapidEvolution	d00X1	HostAssoc_Human Feces	Human feces	USA : Pennsylvania	39.947802 -75.193702	reads SRA	Illumina HiSeq2000	IDBA_UD	0	
Minot_RapidEvolution	d00X2	HostAssoc_Human Feces	Human feces	USA : Pennsylvania	39.947802 -75.193702	reads SRA	Illumina HiSeq2000	IDBA_UD	0	
Minot_RapidEvolution	d01X1	HostAssoc_Human Feces	Human feces	USA : Pennsylvania	39.947802 -75.193702	reads SRA	Illumina HiSeq2000	IDBA_UD	0	
Minot_RapidEvolution	d02X1	HostAssoc_Human Feces	Human feces	USA : Pennsylvania	39.947802 -75.193702	reads SRA	Illumina HiSeq2000	IDBA_UD	0	
Minot_RapidEvolution	d03X1	HostAssoc_Human Feces	Human feces	USA : Pennsylvania	39.947802 -75.193702	reads SRA	Illumina HiSeq2000	IDBA_UD	0	
Minot_RapidEvolution	d03X2	HostAssoc_Human Feces	Human feces	USA : Pennsylvania	39.947802 -75.193702	reads SRA	Illumina HiSeq2000	IDBA_UD	0	
Minot_RapidEvolution	d04X1	HostAssoc_Human Feces	Human feces	USA : Pennsylvania	39.947802 -75.193702	reads SRA	Illumina HiSeq2000	IDBA_UD	0	
Minot_RapidEvolution	d05X1	HostAssoc_Human Feces	Human feces	USA : Pennsylvania	39.947802 -75.193702	reads SRA	Illumina HiSeq2000	IDBA_UD	0	
Minot_RapidEvolution	d11X1	HostAssoc_Human Feces	Human feces	USA : Pennsylvania	39.947802 -75.193702	reads SRA	Illumina HiSeq2000	IDBA_UD	0	
Minot_RapidEvolution	d11X2	HostAssoc_Human Feces	Human feces	USA : Pennsylvania	39.947802 -75.193702	reads SRA	Illumina HiSeq2000	IDBA_UD	0	
Minot_RapidEvolution	d12X1	HostAssoc_Human Feces	Human feces	USA : Pennsylvania	39.947802 -75.193702	reads SRA	Illumina HiSeq2000	IDBA_UD	0	
Minot_RapidEvolution	d12X2	HostAssoc_Human Feces	Human feces	USA : Pennsylvania	39.947802 -75.193702	reads SRA	Illumina HiSeq2000	IDBA_UD	0	
Minot_RapidEvolution	d13X1	HostAssoc_Human Feces	Human feces	USA : Pennsylvania	39.947802 -75.193702	reads SRA	Illumina HiSeq2000	IDBA_UD	0	Minot S. Bryson A. Chehoud C. Wu GD. Lewis JD. Bushman FD. (2013) Rapid evolution of the human gut virome. Proc Natl Acad Sci U S A. 110(30):12450-5. doi: 10.1073/pnas.1300833110.
Minot_RapidEvolution	d13X2	HostAssoc_Human Feces	Human feces	USA : Pennsylvania	39.947802 -75.193702	reads SRA	Illumina HiSeq2000	IDBA_UD	0	
Minot_RapidEvolution	d14X1	HostAssoc_Human Feces	Human feces	USA : Pennsylvania	39.947802 -75.193702	reads SRA	Illumina HiSeq2000	IDBA_UD	0	
Minot_RapidEvolution	d15X1	HostAssoc_Human Feces	Human feces	USA : Pennsylvania	39.947802 -75.193702	reads SRA	Illumina HiSeq2000	IDBA_UD	0	
Minot_RapidEvolution	d21X1	HostAssoc_Human Feces	Human feces	USA : Pennsylvania	39.947802 -75.193702	reads SRA	Illumina HiSeq2000	IDBA_UD	0	
Minot_RapidEvolution	d21X2	HostAssoc_Human Feces	Human feces	USA : Pennsylvania	39.947802 -75.193702	reads SRA	Illumina HiSeq2000	IDBA_UD	0	
Minot_RapidEvolution	d22X1	HostAssoc_Human Feces	Human feces	USA : Pennsylvania	39.947802 -75.193702	reads SRA	Illumina HiSeq2000	IDBA_UD	0	
Minot_RapidEvolution	d22X2	HostAssoc_Human Feces	Human feces	USA : Pennsylvania	39.947802 -75.193702	reads SRA	Illumina HiSeq2000	IDBA_UD	0	
Minot_RapidEvolution	d23X1	HostAssoc_Human Feces	Human feces	USA : Pennsylvania	39.947802 -75.193702	reads SRA	Illumina HiSeq2000	IDBA_UD	0	
Minot_RapidEvolution	d23X2	HostAssoc_Human Feces	Human feces	USA : Pennsylvania	39.947802 -75.193702	reads SRA	Illumina HiSeq2000	IDBA_UD	0	
Minot_RapidEvolution	d24X1	HostAssoc_Human Feces	Human feces	USA : Pennsylvania	39.947802 -75.193702	reads SRA	Illumina HiSeq2000	IDBA_UD	0	
Minot_RapidEvolution	d25X1	HostAssoc_Human Feces	Human feces	USA : Pennsylvania	39.947802 -75.193702	reads SRA	Illumina HiSeq2000	IDBA_UD	0	
Minot_RapidEvolution	Mic1014X01	HostAssoc_Human Feces	Human feces	USA : Pennsylvania	39.947802 -75.193702	reads SRA	Illumina HiSeq2000	IDBA_UD	0	

Project Name	Virome Code	Habitat Type (code)	Ecosystem Type (text)	Location	GPS	Source	Sequencing Technology	Assembly Software	# crucivirus	Reference
Minot_RapidEvolution	Mic1014X02	HostAssoc_Human Feces	Human feces	USA : Pennsylvania	39.947802 -75.193702	reads SRA	Illumina HiSeq2000	IDBA_UD	0	
Minot_RapidEvolution	Mic1014X03	HostAssoc_Human Feces	Human feces	USA : Pennsylvania	39.947802 -75.193702	reads SRA	Illumina HiSeq2000	IDBA_UD	0	
Minot_VariableLoci	Loci1470	HostAssoc_Human Feces	Human feces	USA : Pennsylvania	39.947802 -75.193702	contigs Metavir	Illumina HiSeq2000	?	0	Minot S. Grunberg S. Wu GD. Lewis JD. Bushman FD. Hypervariable loci in the human gut virome. Proc Natl Acad Sci U S A. 2012 Mar 6;109(10):3962-6. doi: 10.1073/pnas.1119061109.
Norman_EntericCrohnUlcere	10568	HostAssoc_Human Feces	Human feces	UK : Cambridge	52.175148 0.140482	reads ENA	Miseq paired end	IDBA_UD	0	
Norman_EntericCrohnUlcere	2409	HostAssoc_Human Feces	Human feces	USA : Illinois	41.874204 -87.668708	reads ENA	Miseq paired end	IDBA_UD	0	
Norman_EntericCrohnUlcere	2411	HostAssoc_Human Feces	Human feces	USA : Illinois	41.874204 -87.668708	reads ENA	Miseq paired end	IDBA_UD	0	
Norman_EntericCrohnUlcere	2412	HostAssoc_Human Feces	Human feces	USA : Illinois	41.874204 -87.668708	reads ENA	Miseq paired end	IDBA_UD	0	
Norman_EntericCrohnUlcere	2413	HostAssoc_Human Feces	Human feces	USA : Illinois	41.874204 -87.668708	reads ENA	Miseq paired end	IDBA_UD	0	
Norman_EntericCrohnUlcere	2414	HostAssoc_Human Feces	Human feces	USA : Illinois	41.874204 -87.668708	reads ENA	Miseq paired end	IDBA_UD	0	
Norman_EntericCrohnUlcere	2415	HostAssoc_Human Feces	Human feces	USA : Illinois	41.874204 -87.668708	reads ENA	Miseq paired end	IDBA_UD	0	
Norman_EntericCrohnUlcere	2419	HostAssoc_Human Feces	Human feces	USA : Illinois	41.874204 -87.668708	reads ENA	Miseq paired end	IDBA_UD	0	
Norman_EntericCrohnUlcere	2420	HostAssoc_Human Feces	Human feces	USA : Illinois	41.874204 -87.668708	reads ENA	Miseq paired end	IDBA_UD	0	
Norman_EntericCrohnUlcere	2422	HostAssoc_Human Feces	Human feces	USA : Illinois	41.874204 -87.668708	reads ENA	Miseq paired end	IDBA_UD	0	
Norman_EntericCrohnUlcere	2424	HostAssoc_Human Feces	Human feces	USA : Illinois	41.874204 -87.668708	reads ENA	Miseq paired end	IDBA_UD	0	
Norman_EntericCrohnUlcere	2426	HostAssoc_Human Feces	Human feces	USA : Illinois	41.874204 -87.668708	reads ENA	Miseq paired end	IDBA_UD	0	
Norman_EntericCrohnUlcere	2448	HostAssoc_Human Feces	Human feces	USA : Illinois	41.874204 -87.668708	reads ENA	Miseq paired end	IDBA_UD	0	
Norman_EntericCrohnUlcere	7544	HostAssoc_Human Feces	Human feces	UK : Cambridge	52.175148 0.140482	reads ENA	Miseq paired end	IDBA_UD	0	
Norman_EntericCrohnUlcere	7547	HostAssoc_Human Feces	Human feces	UK : Cambridge	52.175148 0.140482	reads ENA	Miseq paired end	IDBA_UD	0	
Norman_EntericCrohnUlcere	7550	HostAssoc_Human Feces	Human feces	UK : Cambridge	52.175148 0.140482	reads ENA	Miseq paired end	IDBA_UD	0	
Norman_EntericCrohnUlcere	7552	HostAssoc_Human Feces	Human feces	UK : Cambridge	52.175148 0.140482	reads ENA	Miseq paired end	IDBA_UD	0	
Norman_EntericCrohnUlcere	7554	HostAssoc_Human Feces	Human feces	UK : Cambridge	52.175148 0.140482	reads ENA	Miseq paired end	IDBA_UD	0	
Norman_EntericCrohnUlcere	7556	HostAssoc_Human Feces	Human feces	UK : Cambridge	52.175148 0.140482	reads ENA	Miseq paired end	IDBA_UD	0	
Norman_EntericCrohnUlcere	7560	HostAssoc_Human Feces	Human feces	UK : Cambridge	52.175148 0.140482	reads ENA	Miseq paired end	IDBA_UD	0	
Norman_EntericCrohnUlcere	7562	HostAssoc_Human Feces	Human feces	UK : Cambridge	52.175148 0.140482	reads ENA	Miseq paired end	IDBA_UD	0	
Norman_EntericCrohnUlcere	7564	HostAssoc_Human Feces	Human feces	UK : Cambridge	52.175148 0.140482	reads ENA	Miseq paired end	IDBA_UD	0	
Norman_EntericCrohnUlcere	7577	HostAssoc_Human Feces	Human feces	UK : Cambridge	52.175148 0.140482	reads ENA	Miseq paired end	IDBA_UD	0	
Norman_EntericCrohnUlcere	7589	HostAssoc_Human Feces	Human feces	UK : Cambridge	52.175148 0.140482	reads ENA	Miseq paired end	IDBA_UD	0	
Norman_EntericCrohnUlcere	7605	HostAssoc_Human Feces	Human feces	UK : Cambridge	52.175148 0.140482	reads ENA	Miseq paired end	IDBA_UD	0	
Norman_EntericCrohnUlcere	7612	HostAssoc_Human Feces	Human feces	UK : Cambridge	52.175148 0.140482	reads ENA	Miseq paired end	IDBA_UD	0	
Norman_EntericCrohnUlcere	7614	HostAssoc_Human Feces	Human feces	UK : Cambridge	52.175148 0.140482	reads ENA	Miseq paired end	IDBA_UD	0	
Norman_EntericCrohnUlcere	7631	HostAssoc_Human Feces	Human feces	UK : Cambridge	52.175148 0.140482	reads ENA	Miseq paired end	IDBA_UD	0	
Norman_EntericCrohnUlcere	7632	HostAssoc_Human Feces	Human feces	UK : Cambridge	52.175148 0.140482	reads ENA	Miseq paired end	IDBA_UD	0	
Norman_EntericCrohnUlcere	7634	HostAssoc_Human Feces	Human feces	UK : Cambridge	52.175148 0.140482	reads ENA	Miseq paired end	IDBA_UD	0	
Norman_EntericCrohnUlcere	7647	HostAssoc_Human Feces	Human feces	UK : Cambridge	52.175148 0.140482	reads ENA	Miseq paired end	IDBA_UD	0	
Norman_EntericCrohnUlcere	7650	HostAssoc_Human Feces	Human feces	UK : Cambridge	52.175148 0.140482	reads ENA	Miseq paired end	IDBA_UD	0	
Norman_EntericCrohnUlcere	7652	HostAssoc_Human Feces	Human feces	UK : Cambridge	52.175148 0.140482	reads ENA	Miseq paired end	IDBA_UD	0	
Norman_EntericCrohnUlcere	10569	HostAssoc_Human Feces	Human feces	UK : Cambridge	52.175148 0.140482	reads ENA	Miseq paired end	IDBA_UD	0	
Norman_EntericCrohnUlcere	10571	HostAssoc_Human Feces	Human feces	UK : Cambridge	52.175148 0.140482	reads ENA	Miseq paired end	IDBA_UD	0	
Norman_EntericCrohnUlcere	10577	HostAssoc_Human Feces	Human feces	UK : Cambridge	52.175148 0.140482	reads ENA	Miseq paired end	IDBA_UD	0	
Norman_EntericCrohnUlcere	10578	HostAssoc_Human Feces	Human feces	UK : Cambridge	52.175148 0.140482	reads ENA	Miseq paired end	IDBA_UD	0	
Norman_EntericCrohnUlcere	10579	HostAssoc_Human Feces	Human feces	UK : Cambridge	52.175148 0.140482	reads ENA	Miseq paired end	IDBA_UD	0	
Norman_EntericCrohnUlcere	10586	HostAssoc_Human Feces	Human feces	UK : Cambridge	52.175148 0.140482	reads ENA	Miseq paired end	IDBA_UD	0	

Project Name	Virome Code	Habitat Type (code)	Ecosystem Type (text)	Location	GPS	Source	Sequencing Technology	Assembly Software	# crucivirus	Reference
Norman_EntericCrohnUlcere	10587	HostAssoc_Human Feces	Human feces	UK : Cambridge	52.175148 0.140482	reads ENA	Miseq paired end	IDBA_UD	0	
Norman_EntericCrohnUlcere	10599	HostAssoc_Human Feces	Human feces	UK : Cambridge	52.175148 0.140482	reads ENA	Miseq paired end	IDBA_UD	0	
Norman_EntericCrohnUlcere	10613	HostAssoc_Human Feces	Human feces	UK : Cambridge	52.175148 0.140482	reads ENA	Miseq paired end	IDBA_UD	0	
Norman_EntericCrohnUlcere	10615	HostAssoc_Human Feces	Human feces	UK : Cambridge	52.175148 0.140482	reads ENA	Miseq paired end	IDBA_UD	0	
Norman_EntericCrohnUlcere	10618	HostAssoc_Human Feces	Human feces	UK : Cambridge	52.175148 0.140482	reads ENA	Miseq paired end	IDBA_UD	0	
Norman_EntericCrohnUlcere	12274	HostAssoc_Human Feces	Human feces	UK : Cambridge	52.175148 0.140482	reads ENA	Miseq paired end	IDBA_UD	0	
Norman_EntericCrohnUlcere	13463	HostAssoc_Human Feces	Human feces	USA : Illinois	41.874204 -87.668708	reads ENA	Miseq paired end	IDBA_UD	0	
Norman_EntericCrohnUlcere	13469	HostAssoc_Human Feces	Human feces	USA : Illinois	41.874204 -87.668708	reads ENA	Miseq paired end	IDBA_UD	0	
Norman_EntericCrohnUlcere	13475	HostAssoc_Human Feces	Human feces	USA : Illinois	41.874204 -87.668708	reads ENA	Miseq paired end	IDBA_UD	0	
Norman_EntericCrohnUlcere	13478	HostAssoc_Human Feces	Human feces	USA : Illinois	41.874204 -87.668708	reads ENA	Miseq paired end	IDBA_UD	0	
Norman_EntericCrohnUlcere	13481	HostAssoc_Human Feces	Human feces	USA : Illinois	41.874204 -87.668708	reads ENA	Miseq paired end	IDBA_UD	0	
Norman_EntericCrohnUlcere	13484	HostAssoc_Human Feces	Human feces	USA : Illinois	41.874204 -87.668708	reads ENA	Miseq paired end	IDBA_UD	0	
Norman_EntericCrohnUlcere	13487	HostAssoc_Human Feces	Human feces	USA : Illinois	41.874204 -87.668708	reads ENA	Miseq paired end	IDBA_UD	0	
Norman_EntericCrohnUlcere	13490	HostAssoc_Human Feces	Human feces	USA : Illinois	41.874204 -87.668708	reads ENA	Miseq paired end	IDBA_UD	0	
Norman_EntericCrohnUlcere	13519	HostAssoc_Human Feces	Human feces	UK : Cambridge	52.175148 0.140482	reads ENA	Miseq paired end	IDBA_UD	0	
Norman_EntericCrohnUlcere	13520	HostAssoc_Human Feces	Human feces	UK : Cambridge	52.175148 0.140482	reads ENA	Miseq paired end	IDBA_UD	0	
Norman_EntericCrohnUlcere	13527	HostAssoc_Human Feces	Human feces	UK : Cambridge	52.175148 0.140482	reads ENA	Miseq paired end	IDBA_UD	0	
Norman_EntericCrohnUlcere	13530	HostAssoc_Human Feces	Human feces	UK : Cambridge	52.175148 0.140482	reads ENA	Miseq paired end	IDBA_UD	0	
Norman_EntericCrohnUlcere	14881	HostAssoc_Human Feces	Human feces	UK : Cambridge	52.175148 0.140482	reads ENA	Miseq paired end	IDBA_UD	0	
Norman_EntericCrohnUlcere	14882	HostAssoc_Human Feces	Human feces	UK : Cambridge	52.175148 0.140482	reads ENA	Miseq paired end	IDBA_UD	0	
Norman_EntericCrohnUlcere	14887	HostAssoc_Human Feces	Human feces	UK : Cambridge	52.175148 0.140482	reads ENA	Miseq paired end	IDBA_UD	0	
Norman_EntericCrohnUlcere	14888	HostAssoc_Human Feces	Human feces	UK : Cambridge	52.175148 0.140482	reads ENA	Miseq paired end	IDBA_UD	0	
Norman_EntericCrohnUlcere	14894	HostAssoc_Human Feces	Human feces	UK : Cambridge	52.175148 0.140482	reads ENA	Miseq paired end	IDBA_UD	0	
Norman_EntericCrohnUlcere	14898	HostAssoc_Human Feces	Human feces	UK : Cambridge	52.175148 0.140482	reads ENA	Miseq paired end	IDBA_UD	0	
Norman_EntericCrohnUlcere	14900	HostAssoc_Human Feces	Human feces	UK : Cambridge	52.175148 0.140482	reads ENA	Miseq paired end	IDBA_UD	0	
Norman_EntericCrohnUlcere	14918	HostAssoc_Human Feces	Human feces	UK : Cambridge	52.175148 0.140482	reads ENA	Miseq paired end	IDBA_UD	0	
Norman_EntericCrohnUlcere	14950	HostAssoc_Human Feces	Human feces	UK : Cambridge	52.175148 0.140482	reads ENA	Miseq paired end	IDBA_UD	0	
Norman_EntericCrohnUlcere	14978	HostAssoc_Human Feces	Human feces	UK : Cambridge	52.175148 0.140482	reads ENA	Miseq paired end	IDBA_UD	0	
Norman_EntericCrohnUlcere	14986	HostAssoc_Human Feces	Human feces	UK : Cambridge	52.175148 0.140482	reads ENA	Miseq paired end	IDBA_UD	0	
Norman_EntericCrohnUlcere	14998	HostAssoc_Human Feces	Human feces	UK : Cambridge	52.175148 0.140482	reads ENA	Miseq paired end	IDBA_UD	0	
Norman_EntericCrohnUlcere	14999	HostAssoc_Human Feces	Human feces	UK : Cambridge	52.175148 0.140482	reads ENA	Miseq paired end	IDBA_UD	0	
Norman_EntericCrohnUlcere	15011	HostAssoc_Human Feces	Human feces	UK : Cambridge	52.175148 0.140482	reads ENA	Miseq paired end	IDBA_UD	0	
Norman_EntericCrohnUlcere	15019	HostAssoc_Human Feces	Human feces	UK : Cambridge	52.175148 0.140482	reads ENA	Miseq paired end	IDBA_UD	0	
Norman_EntericCrohnUlcere	15027	HostAssoc_Human Feces	Human feces	UK : Cambridge	52.175148 0.140482	reads ENA	Miseq paired end	IDBA_UD	0	
Norman_EntericCrohnUlcere	15078	HostAssoc_Human Feces	Human feces	UK : Cambridge	52.175148 0.140482	reads ENA	Miseq paired end	IDBA_UD	0	
Norman_EntericCrohnUlcere	15105	HostAssoc_Human Feces	Human feces	UK : Cambridge	52.175148 0.140482	reads ENA	Miseq paired end	IDBA_UD	0	
Norman_EntericCrohnUlcere	15763	HostAssoc_Human Feces	Human feces	UK : Cambridge	52.175148 0.140482	reads ENA	Miseq paired end	IDBA_UD	0	
Norman_EntericCrohnUlcere	15767	HostAssoc_Human Feces	Human feces	UK : Cambridge	52.175148 0.140482	reads ENA	Miseq paired end	IDBA_UD	0	
Norman_EntericCrohnUlcere	15777	HostAssoc_Human Feces	Human feces	UK : Cambridge	52.175148 0.140482	reads ENA	Miseq paired end	IDBA_UD	0	
Norman_EntericCrohnUlcere	15783	HostAssoc_Human Feces	Human feces	UK : Cambridge	52.175148 0.140482	reads ENA	Miseq paired end	IDBA_UD	0	
Norman_EntericCrohnUlcere	15785	HostAssoc_Human Feces	Human feces	UK : Cambridge	52.175148 0.140482	reads ENA	Miseq paired end	IDBA_UD	0	
Norman_EntericCrohnUlcere	15787	HostAssoc_Human Feces	Human feces	UK : Cambridge	52.175148 0.140482	reads ENA	Miseq paired end	IDBA_UD	0	

Project Name	Virome Code	Habitat Type (code)	Ecosystem Type (text)	Location	GPS	Source	Sequencing Technology	Assembly Software	# crucivirus	Reference
Norman_EntericCrohnUlcere	15789	HostAssoc_Human Feces	Human feces	UK : Cambridge	52.175148 0.140482	reads ENA	Miseq paired end	IDBA_UD	0	Norman JM. Handley SA. Baldridge MT. Droit L. Liu CY. Keller BC. Kambal A. Monaco CL. Zhao G. Fleshner P. Stappenbeck TS. McGovern DP. Keshavarzian A. Mutlu EA. Sauk J. Gevers D. Xavier RJ. Wang D. Parkes M. Virgin HW. Disease-specific alterations in the enteric virome in inflammatory bowel disease. Cell. 2015 Jan 29;160(3):447-60. doi: 10.1016/j.cell.2015.01.002.
Norman_EntericCrohnUlcere	15791	HostAssoc_Human Feces	Human feces	UK : Cambridge	52.175148 0.140482	reads ENA	Miseq paired end	IDBA_UD	0	
Norman_EntericCrohnUlcere	15800	HostAssoc_Human Feces	Human feces	UK : Cambridge	52.175148 0.140482	reads ENA	Miseq paired end	IDBA_UD	0	
Norman_EntericCrohnUlcere	15802	HostAssoc_Human Feces	Human feces	UK : Cambridge	52.175148 0.140482	reads ENA	Miseq paired end	IDBA_UD	0	
Norman_EntericCrohnUlcere	15804	HostAssoc_Human Feces	Human feces	UK : Cambridge	52.175148 0.140482	reads ENA	Miseq paired end	IDBA_UD	0	
Norman_EntericCrohnUlcere	15819	HostAssoc_Human Feces	Human feces	UK : Cambridge	52.175148 0.140482	reads ENA	Miseq paired end	IDBA_UD	0	
Norman_EntericCrohnUlcere	15821	HostAssoc_Human Feces	Human feces	UK : Cambridge	52.175148 0.140482	reads ENA	Miseq paired end	IDBA_UD	0	
Norman_EntericCrohnUlcere	15825	HostAssoc_Human Feces	Human feces	UK : Cambridge	52.175148 0.140482	reads ENA	Miseq paired end	IDBA_UD	0	
Norman_EntericCrohnUlcere	15828	HostAssoc_Human Feces	Human feces	UK : Cambridge	52.175148 0.140482	reads ENA	Miseq paired end	IDBA_UD	0	
Norman_EntericCrohnUlcere	15829	HostAssoc_Human Feces	Human feces	UK : Cambridge	52.175148 0.140482	reads ENA	Miseq paired end	IDBA_UD	0	
Norman_EntericCrohnUlcere	15830	HostAssoc_Human Feces	Human feces	UK : Cambridge	52.175148 0.140482	reads ENA	Miseq paired end	IDBA_UD	0	
Norman_EntericCrohnUlcere	15831	HostAssoc_Human Feces	Human feces	UK : Cambridge	52.175148 0.140482	reads ENA	Miseq paired end	IDBA_UD	0	
Norman_EntericCrohnUlcere	15832	HostAssoc_Human Feces	Human feces	UK : Cambridge	52.175148 0.140482	reads ENA	Miseq paired end	IDBA_UD	0	
Norman_EntericCrohnUlcere	15834	HostAssoc_Human Feces	Human feces	UK : Cambridge	52.175148 0.140482	reads ENA	Miseq paired end	IDBA_UD	0	
Norman_EntericCrohnUlcere	15837	HostAssoc_Human Feces	Human feces	UK : Cambridge	52.175148 0.140482	reads ENA	Miseq paired end	IDBA_UD	0	
Norman_EntericCrohnUlcere	15839	HostAssoc_Human Feces	Human feces	UK : Cambridge	52.175148 0.140482	reads ENA	Miseq paired end	IDBA_UD	0	
Norman_EntericCrohnUlcere	15841	HostAssoc_Human Feces	Human feces	UK : Cambridge	52.175148 0.140482	reads ENA	Miseq paired end	IDBA_UD	0	
Norman_EntericCrohnUlcere	16043	HostAssoc_Human Feces	Human feces	USA : Massachusetts	42.363482 -71.068472	reads ENA	Miseq paired end	IDBA_UD	0	
Norman_EntericCrohnUlcere	16045	HostAssoc_Human Feces	Human feces	USA : Massachusetts	42.363482 -71.068472	reads ENA	Miseq paired end	IDBA_UD	0	
Norman_EntericCrohnUlcere	16046	HostAssoc_Human Feces	Human feces	USA : Massachusetts	42.363482 -71.068472	reads ENA	Miseq paired end	IDBA_UD	0	
Norman_EntericCrohnUlcere	16047	HostAssoc_Human Feces	Human feces	USA : Massachusetts	42.363482 -71.068472	reads ENA	Miseq paired end	IDBA_UD	0	
Norman_EntericCrohnUlcere	16048	HostAssoc_Human Feces	Human feces	USA : Massachusetts	42.363482 -71.068472	reads ENA	Miseq paired end	IDBA_UD	0	
Norman_EntericCrohnUlcere	16049	HostAssoc_Human Feces	Human feces	USA : Massachusetts	42.363482 -71.068472	reads ENA	Miseq paired end	IDBA_UD	0	
Norman_EntericCrohnUlcere	16050	HostAssoc_Human Feces	Human feces	USA : Massachusetts	42.363482 -71.068472	reads ENA	Miseq paired end	IDBA_UD	0	
Norman_EntericCrohnUlcere	16051	HostAssoc_Human Feces	Human feces	USA : Massachusetts	42.363482 -71.068472	reads ENA	Miseq paired end	IDBA_UD	0	
Norman_EntericCrohnUlcere	16052	HostAssoc_Human Feces	Human feces	USA : Massachusetts	42.363482 -71.068472	reads ENA	Miseq paired end	IDBA_UD	0	
Norman_EntericCrohnUlcere	16053	HostAssoc_Human Feces	Human feces	USA : Massachusetts	42.363482 -71.068472	reads ENA	Miseq paired end	IDBA_UD	0	
Norman_EntericCrohnUlcere	16054	HostAssoc_Human Feces	Human feces	USA : Massachusetts	42.363482 -71.068472	reads ENA	Miseq paired end	IDBA_UD	0	
Norman_EntericCrohnUlcere	16055	HostAssoc_Human Feces	Human feces	USA : Massachusetts	42.363482 -71.068472	reads ENA	Miseq paired end	IDBA_UD	0	
Norman_EntericCrohnUlcere	16057	HostAssoc_Human Feces	Human feces	USA : Massachusetts	42.363482 -71.068472	reads ENA	Miseq paired end	IDBA_UD	0	
Norman_EntericCrohnUlcere	16058	HostAssoc_Human Feces	Human feces	USA : Massachusetts	42.363482 -71.068472	reads ENA	Miseq paired end	IDBA_UD	0	
Norman_EntericCrohnUlcere	16060	HostAssoc_Human Feces	Human feces	USA : Massachusetts	42.363482 -71.068472	reads ENA	Miseq paired end	IDBA_UD	0	
Norman_EntericCrohnUlcere	16061	HostAssoc_Human Feces	Human feces	USA : Massachusetts	42.363482 -71.068472	reads ENA	Miseq paired end	IDBA_UD	0	
Norman_EntericCrohnUlcere	16063	HostAssoc_Human Feces	Human feces	USA : Massachusetts	42.363482 -71.068472	reads ENA	Miseq paired end	IDBA_UD	0	
Norman_EntericCrohnUlcere	16064	HostAssoc_Human Feces	Human feces	USA : Massachusetts	42.363482 -71.068472	reads ENA	Miseq paired end	IDBA_UD	0	
Norman_EntericCrohnUlcere	16065	HostAssoc_Human Feces	Human feces	USA : Massachusetts	42.363482 -71.068472	reads ENA	Miseq paired end	IDBA_UD	0	
Norman_EntericCrohnUlcere	16067	HostAssoc_Human Feces	Human feces	USA : Massachusetts	42.363482 -71.068472	reads ENA	Miseq paired end	IDBA_UD	0	
Norman_EntericCrohnUlcere	16070	HostAssoc_Human Feces	Human feces	USA : Massachusetts	42.363482 -71.068472	reads ENA	Miseq paired end	IDBA_UD	0	
Norman_EntericCrohnUlcere	16071	HostAssoc_Human Feces	Human feces	USA : Massachusetts	42.363482 -71.068472	reads ENA	Miseq paired end	IDBA_UD	0	
Norman_EntericCrohnUlcere	16073	HostAssoc_Human Feces	Human feces	USA : Massachusetts	42.363482 -71.068472	reads ENA	Miseq paired end	IDBA_UD	0	
Norman_EntericCrohnUlcere	16074	HostAssoc_Human Feces	Human feces	USA : Massachusetts	42.363482 -71.068472	reads ENA	Miseq paired end	IDBA_UD	0	
Norman_EntericCrohnUlcere	16075	HostAssoc_Human Feces	Human feces	USA : Massachusetts	42.363482 -71.068472	reads ENA	Miseq paired end	IDBA_UD	0	

Project Name	Virome Code	Habitat Type (code)	Ecosystem Type (text)	Location	GPS	Source	Sequencing Technology	Assembly Software	# crucivirus	Reference
Norman_EntericCrohnUlcere	16076	HostAssoc_Human Feces	Human feces	USA : Massachusetts	42.363482 -71.068472	reads ENA	Miseq paired end	IDBA_UD	0	
Norman_EntericCrohnUlcere	16079	HostAssoc_Human Feces	Human feces	USA : Massachusetts	42.363482 -71.068472	reads ENA	Miseq paired end	IDBA_UD	0	
Norman_EntericCrohnUlcere	16080	HostAssoc_Human Feces	Human feces	USA : Massachusetts	42.363482 -71.068472	reads ENA	Miseq paired end	IDBA_UD	0	
Norman_EntericCrohnUlcere	16081	HostAssoc_Human Feces	Human feces	USA : Massachusetts	42.363482 -71.068472	reads ENA	Miseq paired end	IDBA_UD	0	
Norman_EntericCrohnUlcere	16082	HostAssoc_Human Feces	Human feces	USA : Massachusetts	42.363482 -71.068472	reads ENA	Miseq paired end	IDBA_UD	0	
Norman_EntericCrohnUlcere	16083	HostAssoc_Human Feces	Human feces	USA : Massachusetts	42.363482 -71.068472	reads ENA	Miseq paired end	IDBA_UD	0	
Norman_EntericCrohnUlcere	16084	HostAssoc_Human Feces	Human feces	USA : Massachusetts	42.363482 -71.068472	reads ENA	Miseq paired end	IDBA_UD	0	
Norman_EntericCrohnUlcere	16085	HostAssoc_Human Feces	Human feces	USA : Massachusetts	42.363482 -71.068472	reads ENA	Miseq paired end	IDBA_UD	0	
Norman_EntericCrohnUlcere	16087	HostAssoc_Human Feces	Human feces	USA : Massachusetts	42.363482 -71.068472	reads ENA	Miseq paired end	IDBA_UD	0	
Norman_EntericCrohnUlcere	16089	HostAssoc_Human Feces	Human feces	USA : Illinois	41.874204 -87.668708	reads ENA	Miseq paired end	IDBA_UD	0	
Norman_EntericCrohnUlcere	16090	HostAssoc_Human Feces	Human feces	USA : Illinois	41.874204 -87.668708	reads ENA	Miseq paired end	IDBA_UD	0	
Norman_EntericCrohnUlcere	16092	HostAssoc_Human Feces	Human feces	USA : Illinois	41.874204 -87.668708	reads ENA	Miseq paired end	IDBA_UD	0	
Norman_EntericCrohnUlcere	16094	HostAssoc_Human Feces	Human feces	USA : Illinois	41.874204 -87.668708	reads ENA	Miseq paired end	IDBA_UD	0	
Norman_EntericCrohnUlcere	16096	HostAssoc_Human Feces	Human feces	USA : Illinois	41.874204 -87.668708	reads ENA	Miseq paired end	IDBA_UD	0	
Norman_EntericCrohnUlcere	16097	HostAssoc_Human Feces	Human feces	USA : Illinois	41.874204 -87.668708	reads ENA	Miseq paired end	IDBA_UD	0	
Norman_EntericCrohnUlcere	16098	HostAssoc_Human Feces	Human feces	USA : Illinois	41.874204 -87.668708	reads ENA	Miseq paired end	IDBA_UD	0	
Norman_EntericCrohnUlcere	16099	HostAssoc_Human Feces	Human feces	USA : Illinois	41.874204 -87.668708	reads ENA	Miseq paired end	IDBA_UD	0	
Norman_EntericCrohnUlcere	16100	HostAssoc_Human Feces	Human feces	USA : Illinois	41.874204 -87.668708	reads ENA	Miseq paired end	IDBA_UD	0	
Norman_EntericCrohnUlcere	16101	HostAssoc_Human Feces	Human feces	USA : Illinois	41.874204 -87.668708	reads ENA	Miseq paired end	IDBA_UD	0	
Norman_EntericCrohnUlcere	16102	HostAssoc_Human Feces	Human feces	USA : Illinois	41.874204 -87.668708	reads ENA	Miseq paired end	IDBA_UD	0	
Norman_EntericCrohnUlcere	16103	HostAssoc_Human Feces	Human feces	USA : Illinois	41.874204 -87.668708	reads ENA	Miseq paired end	IDBA_UD	0	
Norman_EntericCrohnUlcere	16104	HostAssoc_Human Feces	Human feces	USA : Illinois	41.874204 -87.668708	reads ENA	Miseq paired end	IDBA_UD	0	
Norman_EntericCrohnUlcere	16105	HostAssoc_Human Feces	Human feces	USA : Illinois	41.874204 -87.668708	reads ENA	Miseq paired end	IDBA_UD	0	
Norman_EntericCrohnUlcere	16106	HostAssoc_Human Feces	Human feces	USA : Illinois	41.874204 -87.668708	reads ENA	Miseq paired end	IDBA_UD	0	
Norman_EntericCrohnUlcere	16107	HostAssoc_Human Feces	Human feces	USA : Illinois	41.874204 -87.668708	reads ENA	Miseq paired end	IDBA_UD	0	
Norman_EntericCrohnUlcere	16108	HostAssoc_Human Feces	Human feces	USA : Illinois	41.874204 -87.668708	reads ENA	Miseq paired end	IDBA_UD	0	
Norman_EntericCrohnUlcere	16110	HostAssoc_Human Feces	Human feces	USA : Illinois	41.874204 -87.668708	reads ENA	Miseq paired end	IDBA_UD	0	
Norman_EntericCrohnUlcere	16111	HostAssoc_Human Feces	Human feces	USA : Illinois	41.874204 -87.668708	reads ENA	Miseq paired end	IDBA_UD	0	
Norman_EntericCrohnUlcere	16112	HostAssoc_Human Feces	Human feces	USA : Illinois	41.874204 -87.668708	reads ENA	Miseq paired end	IDBA_UD	0	
Norman_EntericCrohnUlcere	16113	HostAssoc_Human Feces	Human feces	USA : Illinois	41.874204 -87.668708	reads ENA	Miseq paired end	IDBA_UD	0	
Norman_EntericCrohnUlcere	16114	HostAssoc_Human Feces	Human feces	USA : Illinois	41.874204 -87.668708	reads ENA	Miseq paired end	IDBA_UD	0	
Norman_EntericCrohnUlcere	16115	HostAssoc_Human Feces	Human feces	USA : Illinois	41.874204 -87.668708	reads ENA	Miseq paired end	IDBA_UD	0	
Norman_EntericCrohnUlcere	16116	HostAssoc_Human Feces	Human feces	USA : Illinois	41.874204 -87.668708	reads ENA	Miseq paired end	IDBA_UD	0	
Norman_EntericCrohnUlcere	16117	HostAssoc_Human Feces	Human feces	USA : Illinois	41.874204 -87.668708	reads ENA	Miseq paired end	IDBA_UD	0	
Norman_EntericCrohnUlcere	16118	HostAssoc_Human Feces	Human feces	USA : Illinois	41.874204 -87.668708	reads ENA	Miseq paired end	IDBA_UD	0	
Norman_EntericCrohnUlcere	16119	HostAssoc_Human Feces	Human feces	USA : Illinois	41.874204 -87.668708	reads ENA	Miseq paired end	IDBA_UD	0	
Norman_EntericCrohnUlcere	16120	HostAssoc_Human Feces	Human feces	USA : Illinois	41.874204 -87.668708	reads ENA	Miseq paired end	IDBA_UD	0	
Norman_EntericCrohnUlcere	16121	HostAssoc_Human Feces	Human feces	USA : Massachusetts	42.363482 -71.068472	reads ENA	Miseq paired end	IDBA_UD	0	
Norman_EntericCrohnUlcere	16122	HostAssoc_Human Feces	Human feces	USA : Massachusetts	42.363482 -71.068472	reads ENA	Miseq paired end	IDBA_UD	0	
Norman_EntericCrohnUlcere	16123	HostAssoc_Human Feces	Human feces	USA : Massachusetts	42.363482 -71.068472	reads ENA	Miseq paired end	IDBA_UD	0	
Norman_EntericCrohnUlcere	16124	HostAssoc_Human Feces	Human feces	USA : Massachusetts	42.363482 -71.068472	reads ENA	Miseq paired end	IDBA_UD	0	
Norman_EntericCrohnUlcere	16125	HostAssoc_Human Feces	Human feces	USA : Massachusetts	42.363482 -71.068472	reads ENA	Miseq paired end	IDBA_UD	0	

Project Name	Virome Code	Habitat Type (code)	Ecosystem Type (text)	Location	GPS	Source	Sequencing Technology	Assembly Software	# crucivirus	Reference
Norman_EntericCrohnUlcere	16126	HostAssoc_Human Feces	Human feces	USA : Massachusetts	42.363482 -71.068472	reads ENA	Miseq paired end	IDBA_UD	0	
Norman_EntericCrohnUlcere	16127	HostAssoc_Human Feces	Human feces	USA : Massachusetts	42.363482 -71.068472	reads ENA	Miseq paired end	IDBA_UD	0	
Norman_EntericCrohnUlcere	16128	HostAssoc_Human Feces	Human feces	USA : Massachusetts	42.363482 -71.068472	reads ENA	Miseq paired end	IDBA_UD	0	
Norman_EntericCrohnUlcere	16129	HostAssoc_Human Feces	Human feces	USA : Massachusetts	42.363482 -71.068472	reads ENA	Miseq paired end	IDBA_UD	0	
Norman_EntericCrohnUlcere	16130	HostAssoc_Human Feces	Human feces	USA : Massachusetts	42.363482 -71.068472	reads ENA	Miseq paired end	IDBA_UD	0	
Norman_EntericCrohnUlcere	16131	HostAssoc_Human Feces	Human feces	USA : Massachusetts	42.363482 -71.068472	reads ENA	Miseq paired end	IDBA_UD	0	
PerezBrocal_CDViromes	CDv5176	HostAssoc_Human Feces	Human feces	Spain : Valencia	39.468228 -0.378304	reads ENA	454 GS-FLX Titanium	Newbler	0	
PerezBrocal_CDViromes	CDv5177	HostAssoc_Human Feces	Human feces	Spain : Valencia	39.468228 -0.378304	reads ENA	454 GS-FLX Titanium	Newbler	0	
PerezBrocal_CDViromes	CDv5178	HostAssoc_Human Feces	Human feces	Spain : Valencia	39.468228 -0.378304	reads ENA	454 GS-FLX Titanium	Newbler	0	
PerezBrocal_CDViromes	CDv5180	HostAssoc_Human Feces	Human feces	Spain : Valencia	39.468228 -0.378304	reads ENA	454 GS-FLX Titanium	Newbler	0	
PerezBrocal_CDViromes	CDv5181	HostAssoc_Human Feces	Human feces	Spain : Valencia	39.468228 -0.378304	reads ENA	454 GS-FLX Titanium	Newbler	0	
PerezBrocal_CDViromes	CDv5182	HostAssoc_Human Feces	Human feces	Spain : Valencia	39.468228 -0.378304	reads ENA	454 GS-FLX Titanium	Newbler	0	
PerezBrocal_CDViromes	CDv5184	HostAssoc_Human Feces	Human feces	Spain : Valencia	39.468228 -0.378304	reads ENA	454 GS-FLX Titanium	Newbler	0	
PerezBrocal_CDViromes	CDv5185	HostAssoc_Human Feces	Human feces	Spain : Valencia	39.468228 -0.378304	reads ENA	454 GS-FLX Titanium	Newbler	0	
PerezBrocal_CDViromes	CDv5186	HostAssoc_Human Feces	Human feces	Spain : Valencia	39.468228 -0.378304	reads ENA	454 GS-FLX Titanium	Newbler	0	
PerezBrocal_CDViromes	CDv5188	HostAssoc_Human Feces	Human feces	Spain : Valencia	39.468228 -0.378304	reads ENA	454 GS-FLX Titanium	Newbler	0	
PerezBrocal_CDViromes	CDv5189	HostAssoc_Human Feces	Human feces	Spain : Valencia	39.468228 -0.378304	reads ENA	454 GS-FLX Titanium	Newbler	0	
PerezBrocal_CDViromes	CDv5190	HostAssoc_Human Feces	Human feces	Spain : Valencia	39.468228 -0.378304	reads ENA	454 GS-FLX Titanium	Newbler	0	
PerezBrocal_CDViromes	CDv5192	HostAssoc_Human Feces	Human feces	Spain : Valencia	39.468228 -0.378304	reads ENA	454 GS-FLX Titanium	Newbler	0	
PerezBrocal_CDViromes	CDv5193	HostAssoc_Human Feces	Human feces	Spain : Valencia	39.468228 -0.378304	reads ENA	454 GS-FLX Titanium	Newbler	0	
PerezBrocal_CDViromes	CDv5194	HostAssoc_Human Feces	Human feces	Spain : Valencia	39.468228 -0.378304	reads ENA	454 GS-FLX Titanium	Newbler	0	
PerezBrocal_CDViromes	CDv5196	HostAssoc_Human Feces	Human feces	Spain : Valencia	39.468228 -0.378304	reads ENA	454 GS-FLX Titanium	Newbler	0	
PerezBrocal_CDViromes	CDv5197	HostAssoc_Human Feces	Human feces	Spain : Valencia	39.468228 -0.378304	reads ENA	454 GS-FLX Titanium	Newbler	0	
PerezBrocal_CDViromes	CDv5198	HostAssoc_Human Feces	Human feces	Spain : Valencia	39.468228 -0.378304	reads ENA	454 GS-FLX Titanium	Newbler	0	
PerezBrocal_CDViromes	CDv5200	HostAssoc_Human Feces	Human feces	Spain : Valencia	39.468228 -0.378304	reads ENA	454 GS-FLX Titanium	Newbler	0	
PerezBrocal_CDViromes	CDv5202	HostAssoc_Human Feces	Human feces	Spain : Valencia	39.468228 -0.378304	reads ENA	454 GS-FLX Titanium	Newbler	0	
PerezBrocal_CDViromes	CDv5204	HostAssoc_Human Feces	Human feces	Spain : Valencia	39.468228 -0.378304	reads ENA	454 GS-FLX Titanium	Newbler	0	
PerezBrocal_CDViromes	CDv5205	HostAssoc_Human Feces	Human feces	Spain : Valencia	39.468228 -0.378304	reads ENA	454 GS-FLX Titanium	Newbler	0	
PerezBrocal_CDViromes	CDv5206	HostAssoc_Human Feces	Human feces	Spain : Valencia	39.468228 -0.378304	reads ENA	454 GS-FLX Titanium	Newbler	0	
PerezBrocal_CDViromes	CDv5208	HostAssoc_Human Feces	Human feces	Spain : Valencia	39.468228 -0.378304	reads ENA	454 GS-FLX Titanium	Newbler	0	
PerezBrocal_CDViromes	CDv5210	HostAssoc_Human Feces	Human feces	Spain : Valencia	39.468228 -0.378304	reads ENA	454 GS-FLX Titanium	Newbler	0	
PerezBrocal_CDViromes	CDv5212	HostAssoc_Human Feces	Human feces	Spain : Valencia	39.468228 -0.378304	reads ENA	454 GS-FLX Titanium	Newbler	0	
PerezBrocal_CDViromes	CDv5213	HostAssoc_Human Feces	Human feces	Spain : Valencia	39.468228 -0.378304	reads ENA	454 GS-FLX Titanium	Newbler	0	
PerezBrocal_CDViromes	CDv5214	HostAssoc_Human Feces	Human feces	Spain : Valencia	39.468228 -0.378304	reads ENA	454 GS-FLX Titanium	Newbler	0	
PerezBrocal_CDViromes	CDv5216	HostAssoc_Human Feces	Human feces	Spain : Valencia	39.468228 -0.378304	reads ENA	454 GS-FLX Titanium	Newbler	0	
PerezBrocal_CDViromes	CDv5217	HostAssoc_Human Feces	Human feces	Spain : Valencia	39.468228 -0.378304	reads ENA	454 GS-FLX Titanium	Newbler	0	
PerezBrocal_CDViromes	CDv5218	HostAssoc_Human Feces	Human feces	Spain : Valencia	39.468228 -0.378304	reads ENA	454 GS-FLX Titanium	Newbler	0	
PerezBrocal_CDViromes	CDv5220	HostAssoc_Human Feces	Human feces	Spain : Valencia	39.468228 -0.378304	reads ENA	454 GS-FLX Titanium	Newbler	0	
PerezBrocal_CDViromes	CDv5222	HostAssoc_Human Feces	Human feces	Spain : Valencia	39.468228 -0.378304	reads ENA	454 GS-FLX Titanium	Newbler	0	
PerezBrocal_CDViromes	CDv5224	HostAssoc_Human Feces	Human feces	Spain : Valencia	39.468228 -0.378304	reads ENA	454 GS-FLX Titanium	Newbler	0	
PerezBrocal_CDViromes	CDv5225	HostAssoc_Human Feces	Human feces	Spain : Valencia	39.468228 -0.378304	reads ENA	454 GS-FLX Titanium	Newbler	0	
PerezBrocal_CDViromes	CDv5226	HostAssoc_Human Feces	Human feces	Spain : Valencia	39.468228 -0.378304	reads ENA	454 GS-FLX Titanium	Newbler	0	

Project Name	Virome Code	Habitat Type (code)	Ecosystem Type (text)	Location	GPS	Source	Sequencing Technology	Assembly Software	# crucivirus	Reference
PerezBrocal_CDViromes	CDv5228	HostAssoc_Human Feces	Human feces	Spain : Valencia	39.468228 -0.378304	reads ENA	454 GS-FLX Titanium	Newbler	0	
PerezBrocal_CDViromes	CDv5229	HostAssoc_Human Feces	Human feces	Spain : Valencia	39.468228 -0.378304	reads ENA	454 GS-FLX Titanium	Newbler	0	
PerezBrocal_CDViromes	CDv5231	HostAssoc_Human Feces	Human feces	Spain : Valencia	39.468228 -0.378304	reads ENA	454 GS-FLX Titanium	Newbler	0	
PerezBrocal_CDViromes	CDv5232	HostAssoc_Human Feces	Human feces	Spain : Valencia	39.468228 -0.378304	reads ENA	454 GS-FLX Titanium	Newbler	0	
PerezBrocal_CDViromes	CDv5233	HostAssoc_Human Feces	Human feces	Spain : Valencia	39.468228 -0.378304	reads ENA	454 GS-FLX Titanium	Newbler	0	
PerezBrocal_CDViromes	CDv5235	HostAssoc_Human Feces	Human feces	Spain : Valencia	39.468228 -0.378304	reads ENA	454 GS-FLX Titanium	Newbler	0	
PerezBrocal_CDViromes	CDv5236	HostAssoc_Human Feces	Human feces	Spain : Valencia	39.468228 -0.378304	reads ENA	454 GS-FLX Titanium	Newbler	0	
PerezBrocal_CDViromes	CDv5237	HostAssoc_Human Feces	Human feces	Spain : Valencia	39.468228 -0.378304	reads ENA	454 GS-FLX Titanium	Newbler	0	
PerezBrocal_CDViromes	CDv5239	HostAssoc_Human Feces	Human feces	Spain : Valencia	39.468228 -0.378304	reads ENA	454 GS-FLX Titanium	Newbler	0	
PerezBrocal_CDViromes	CDv5240	HostAssoc_Human Feces	Human feces	Spain : Valencia	39.468228 -0.378304	reads ENA	454 GS-FLX Titanium	Newbler	0	
PerezBrocal_CDViromes	CDv5241	HostAssoc_Human Feces	Human feces	Spain : Valencia	39.468228 -0.378304	reads ENA	454 GS-FLX Titanium	Newbler	0	
PerezBrocal_CDViromes	CDv5243	HostAssoc_Human Feces	Human feces	Spain : Valencia	39.468228 -0.378304	reads ENA	454 GS-FLX Titanium	Newbler	0	
PerezBrocal_CDViromes	CDv5245	HostAssoc_Human Feces	Human feces	Spain : Valencia	39.468228 -0.378304	reads ENA	454 GS-FLX Titanium	Newbler	0	
PerezBrocal_CDViromes	CDv5247	HostAssoc_Human Feces	Human feces	Spain : Valencia	39.468228 -0.378304	reads ENA	454 GS-FLX Titanium	Newbler	0	
PerezBrocal_CDViromes	CDv5248	HostAssoc_Human Feces	Human feces	Spain : Valencia	39.468228 -0.378304	reads ENA	454 GS-FLX Titanium	Newbler	0	
PerezBrocal_CDViromes	CDv5249	HostAssoc_Human Feces	Human feces	Spain : Valencia	39.468228 -0.378304	reads ENA	454 GS-FLX Titanium	Newbler	0	
PerezBrocal_CDViromes	CDv5251	HostAssoc_Human Feces	Human feces	Spain : Valencia	39.468228 -0.378304	reads ENA	454 GS-FLX Titanium	Newbler	0	
PerezBrocal_CDViromes	CDv5252	HostAssoc_Human Feces	Human feces	Spain : Valencia	39.468228 -0.378304	reads ENA	454 GS-FLX Titanium	Newbler	0	
PerezBrocal_CDViromes	CDv5253	HostAssoc_Human Feces	Human feces	Spain : Valencia	39.468228 -0.378304	reads ENA	454 GS-FLX Titanium	Newbler	0	
PerezBrocal_CDViromes	CDv5255	HostAssoc_Human Feces	Human feces	Spain : Valencia	39.468228 -0.378304	reads ENA	454 GS-FLX Titanium	Newbler	0	
PerezBrocal_CDViromes	CDv5256	HostAssoc_Human Feces	Human feces	Spain : Valencia	39.468228 -0.378304	reads ENA	454 GS-FLX Titanium	Newbler	0	Pérez-Brocal V. García-López R. Nos P. Beltrán B. Moret I. Moya A. Metagenomic Analysis of Crohn's Disease Patients Identifies Changes in the Virome and Microbiome Related to Disease Status and Therapy. and Detects Potential Interactions and Biomarkers. Inflamm Bowel Dis. 2015 Nov;21(11):2515-32. doi: 10.1097/MIB.
PerezBrocal_CDViromes	CDv5257	HostAssoc_Human Feces	Human feces	Spain : Valencia	39.468228 -0.378304	reads ENA	454 GS-FLX Titanium	Newbler	0	
PerezBrocal_CDViromes	CDv5259	HostAssoc_Human Feces	Human feces	Spain : Valencia	39.468228 -0.378304	reads ENA	454 GS-FLX Titanium	Newbler	0	
PerezBrocal_CDViromes	CDv5260	HostAssoc_Human Feces	Human feces	Spain : Valencia	39.468228 -0.378304	reads ENA	454 GS-FLX Titanium	Newbler	0	
PerezBrocal_CDViromes	CDv5261	HostAssoc_Human Feces	Human feces	Spain : Valencia	39.468228 -0.378304	reads ENA	454 GS-FLX Titanium	Newbler	0	
PerezBrocal_CDViromes	CDv5263	HostAssoc_Human Feces	Human feces	Spain : Valencia	39.468228 -0.378304	reads ENA	454 GS-FLX Titanium	Newbler	0	
PerezBrocal_CDViromes	CDv5264	HostAssoc_Human Feces	Human feces	Spain : Valencia	39.468228 -0.378304	reads ENA	454 GS-FLX Titanium	Newbler	0	
PerezBrocal_CDViromes	CDv5265	HostAssoc_Human Feces	Human feces	Spain : Valencia	39.468228 -0.378304	reads ENA	454 GS-FLX Titanium	Newbler	0	
PerezBrocal_CDViromes	CDv5267	HostAssoc_Human Feces	Human feces	Spain : Valencia	39.468228 -0.378304	reads ENA	454 GS-FLX Titanium	Newbler	0	
PerezBrocal_CDViromes	CDv5268	HostAssoc_Human Feces	Human feces	Spain : Valencia	39.468228 -0.378304	reads ENA	454 GS-FLX Titanium	Newbler	0	
PerezBrocal_CDViromes	CDv5269	HostAssoc_Human Feces	Human feces	Spain : Valencia	39.468228 -0.378304	reads ENA	454 GS-FLX Titanium	Newbler	0	
PerezBrocal_CDViromes	CDv5271	HostAssoc_Human Feces	Human feces	Spain : Valencia	39.468228 -0.378304	reads ENA	454 GS-FLX Titanium	Newbler	0	
PerezBrocal_CDViromes	CDv5272	HostAssoc_Human Feces	Human feces	Spain : Valencia	39.468228 -0.378304	reads ENA	454 GS-FLX Titanium	Newbler	0	
PerezBrocal_CDViromes	CDv5273	HostAssoc_Human Feces	Human feces	Spain : Valencia	39.468228 -0.378304	reads ENA	454 GS-FLX Titanium	Newbler	0	
PerezBrocal_CDViromes	CDv5275	HostAssoc_Human Feces	Human feces	Spain : Valencia	39.468228 -0.378304	reads ENA	454 GS-FLX Titanium	Newbler	0	
PerezBrocal_CDViromes	CDv5276	HostAssoc_Human Feces	Human feces	Spain : Valencia	39.468228 -0.378304	reads ENA	454 GS-FLX Titanium	Newbler	0	
PerezBrocal_CDViromes	CDv5277	HostAssoc_Human Feces	Human feces	Spain : Valencia	39.468228 -0.378304	reads ENA	454 GS-FLX Titanium	Newbler	0	
PerezBrocal_CDViromes	CDv5279	HostAssoc_Human Feces	Human feces	Spain : Valencia	39.468228 -0.378304	reads ENA	454 GS-FLX Titanium	Newbler	0	
PerezBrocal_CDViromes	CDv5280	HostAssoc_Human Feces	Human feces	Spain : Valencia	39.468228 -0.378304	reads ENA	454 GS-FLX Titanium	Newbler	0	
PerezBrocal_CDViromes	CDv5281	HostAssoc_Human Feces	Human feces	Spain : Valencia	39.468228 -0.378304	reads ENA	454 GS-FLX Titanium	Newbler	0	
PerezBrocal_CDViromes	CDv5283	HostAssoc_Human Feces	Human feces	Spain : Valencia	39.468228 -0.378304	reads ENA	454 GS-FLX Titanium	Newbler	0	
PerezBrocal_CDViromes	CDv5284	HostAssoc_Human Feces	Human feces	Spain : Valencia	39.468228 -0.378304	reads ENA	454 GS-FLX Titanium	Newbler	0	

Project Name	Virome Code	Habitat Type (code)	Ecosystem Type (text)	Location	GPS	Source	Sequencing Technology	Assembly Software	# crucivirus	Reference
PerezBrocal_CDViromes	CDv5285	HostAssoc_Human Feces	Human feces	Spain : Valencia	39.468228 -0.378304	reads ENA	454 GS-FLX Titanium	Newbler	0	
PerezBrocal_CDViromes	CDv5287	HostAssoc_Human Feces	Human feces	Spain : Valencia	39.468228 -0.378304	reads ENA	454 GS-FLX Titanium	Newbler	0	
PerezBrocal_CDViromes	CDv5288	HostAssoc_Human Feces	Human feces	Spain : Valencia	39.468228 -0.378304	reads ENA	454 GS-FLX Titanium	Newbler	0	
PerezBrocal_CDViromes	CDv5289	HostAssoc_Human Feces	Human feces	Spain : Valencia	39.468228 -0.378304	reads ENA	454 GS-FLX Titanium	Newbler	0	
PerezBrocal_CDViromes	CDv5291	HostAssoc_Human Feces	Human feces	Spain : Valencia	39.468228 -0.378304	reads ENA	454 GS-FLX Titanium	Newbler	0	
PerezBrocal_CDViromes	CDv5292	HostAssoc_Human Feces	Human feces	Spain : Valencia	39.468228 -0.378304	reads ENA	454 GS-FLX Titanium	Newbler	0	
PerezBrocal_CDViromes	CDv5293	HostAssoc_Human Feces	Human feces	Spain : Valencia	39.468228 -0.378304	reads ENA	454 GS-FLX Titanium	Newbler	0	
PerezBrocal_CDViromes	CDv5295	HostAssoc_Human Feces	Human feces	Spain : Valencia	39.468228 -0.378304	reads ENA	454 GS-FLX Titanium	Newbler	0	
PerezBrocal_CDViromes	CDv5296	HostAssoc_Human Feces	Human feces	Spain : Valencia	39.468228 -0.378304	reads ENA	454 GS-FLX Titanium	Newbler	0	
PerezBrocal_CDViromes	CDv5298	HostAssoc_Human Feces	Human feces	Spain : Valencia	39.468228 -0.378304	reads ENA	454 GS-FLX Titanium	Newbler	0	
PerezBrocal_CDViromes	CDv5299	HostAssoc_Human Feces	Human feces	Spain : Valencia	39.468228 -0.378304	reads ENA	454 GS-FLX Titanium	Newbler	0	
PerezBrocal_CDViromes	CDv5300	HostAssoc_Human Feces	Human feces	Spain : Valencia	39.468228 -0.378304	reads ENA	454 GS-FLX Titanium	Newbler	0	
PerezBrocal_CDViromes	CDv5302	HostAssoc_Human Feces	Human feces	Spain : Valencia	39.468228 -0.378304	reads ENA	454 GS-FLX Titanium	Newbler	0	
PerezBrocal_CDViromes	CDv5303	HostAssoc_Human Feces	Human feces	Spain : Valencia	39.468228 -0.378304	reads ENA	454 GS-FLX Titanium	Newbler	0	
PerezBrocal_CDViromes	CDv5304	HostAssoc_Human Feces	Human feces	Spain : Valencia	39.468228 -0.378304	reads ENA	454 GS-FLX Titanium	Newbler	0	
PerezBrocal_CDViromes	CDv5306	HostAssoc_Human Feces	Human feces	Spain : Valencia	39.468228 -0.378304	reads ENA	454 GS-FLX Titanium	Newbler	0	
PerezBrocal_CDViromes	CDv5307	HostAssoc_Human Feces	Human feces	Spain : Valencia	39.468228 -0.378304	reads ENA	454 GS-FLX Titanium	Newbler	0	
PerezBrocal_CDViromes	CDv5308	HostAssoc_Human Feces	Human feces	Spain : Valencia	39.468228 -0.378304	reads ENA	454 GS-FLX Titanium	Newbler	0	
PerezBrocal_CDViromes	CDv5310	HostAssoc_Human Feces	Human feces	Spain : Valencia	39.468228 -0.378304	reads ENA	454 GS-FLX Titanium	Newbler	0	
PerezBrocal_CDViromes	CDv5312	HostAssoc_Human Feces	Human feces	Spain : Valencia	39.468228 -0.378304	reads ENA	454 GS-FLX Titanium	Newbler	0	
PerezBrocal_CDViromes	CDv5314	HostAssoc_Human Feces	Human feces	Spain : Valencia	39.468228 -0.378304	reads ENA	454 GS-FLX Titanium	Newbler	0	
PerezBrocal_CDViromes	CDv5315	HostAssoc_Human Feces	Human feces	Spain : Valencia	39.468228 -0.378304	reads ENA	454 GS-FLX Titanium	Newbler	0	
PerezBrocal_CDViromes	CDv5316	HostAssoc_Human Feces	Human feces	Spain : Valencia	39.468228 -0.378304	reads ENA	454 GS-FLX Titanium	Newbler	0	
PerezBrocal_CDViromes	CDv5318	HostAssoc_Human Feces	Human feces	Spain : Valencia	39.468228 -0.378304	reads ENA	454 GS-FLX Titanium	Newbler	0	
PerezBrocal_CDViromes	CDv5319	HostAssoc_Human Feces	Human feces	Spain : Valencia	39.468228 -0.378304	reads ENA	454 GS-FLX Titanium	Newbler	0	
PerezBrocal_CDViromes	CDv5320	HostAssoc_Human Feces	Human feces	Spain : Valencia	39.468228 -0.378304	reads ENA	454 GS-FLX Titanium	Newbler	0	
PerezBrocal_CDViromes	CDv5322	HostAssoc_Human Feces	Human feces	Spain : Valencia	39.468228 -0.378304	reads ENA	454 GS-FLX Titanium	Newbler	0	
PerezBrocal_CDViromes	CDv5323	HostAssoc_Human Feces	Human feces	Spain : Valencia	39.468228 -0.378304	reads ENA	454 GS-FLX Titanium	Newbler	0	
PerezBrocal_CDViromes	CDv5324	HostAssoc_Human Feces	Human feces	Spain : Valencia	39.468228 -0.378304	reads ENA	454 GS-FLX Titanium	Newbler	0	
PerezBrocal_CDViromes	CDv5326	HostAssoc_Human Feces	Human feces	Spain : Valencia	39.468228 -0.378304	reads ENA	454 GS-FLX Titanium	Newbler	0	
PerezBrocal_CDViromes	CDv5327	HostAssoc_Human Feces	Human feces	Spain : Valencia	39.468228 -0.378304	reads ENA	454 GS-FLX Titanium	Newbler	0	
PerezBrocal_CDViromes	CDv5328	HostAssoc_Human Feces	Human feces	Spain : Valencia	39.468228 -0.378304	reads ENA	454 GS-FLX Titanium	Newbler	0	
PerezBrocal_CDViromes	CDv5330	HostAssoc_Human Feces	Human feces	Spain : Valencia	39.468228 -0.378304	reads ENA	454 GS-FLX Titanium	Newbler	0	
PerezBrocal_CDViromes	CDv5331	HostAssoc_Human Feces	Human feces	Spain : Valencia	39.468228 -0.378304	reads ENA	454 GS-FLX Titanium	Newbler	0	
PerezBrocal_CDViromes	CDv5332	HostAssoc_Human Feces	Human feces	Spain : Valencia	39.468228 -0.378304	reads ENA	454 GS-FLX Titanium	Newbler	0	
PerezBrocal_CDViromes	CDv5334	HostAssoc_Human Feces	Human feces	Spain : Valencia	39.468228 -0.378304	reads ENA	454 GS-FLX Titanium	Newbler	0	
PerezBrocal_CDViromes	CDv5336	HostAssoc_Human Feces	Human feces	Spain : Valencia	39.468228 -0.378304	reads ENA	454 GS-FLX Titanium	Newbler	0	
Pride_LongTermAntibio	ELA100A	HostAssoc_Human Feces	Human feces	USA : California	32.876609 -117.236885	reads MG-RAST	Ion Torrent	Newbler -large	0	
Pride_LongTermAntibio	ELA100B	HostAssoc_Human Feces	Human feces	USA : California	32.876609 -117.236885	reads MG-RAST	Ion Torrent	Newbler -large	0	
Pride_LongTermAntibio	ELA100C	HostAssoc_Human Feces	Human feces	USA : California	32.876609 -117.236885	reads MG-RAST	Ion Torrent	Newbler -large	0	
Pride_LongTermAntibio	ELA1A	HostAssoc_Human Feces	Human feces	USA : California	32.876609 -117.236885	reads MG-RAST	Ion Torrent	Newbler -large	0	
Pride_LongTermAntibio	ELA1B	HostAssoc_Human Feces	Human feces	USA : California	32.876609 -117.236885	reads MG-RAST	Ion Torrent	Newbler -large	0	

Project Name	Virome Code	Habitat Type (code)	Ecosystem Type (text)	Location	GPS	Source	Sequencing Technology	Assembly Software	# crucivirus	Reference
Pride_LongTermAntibio	ELA1C	HostAssoc_Human Feces	Human feces	USA : California	32.876609 -117.236885	reads MG-RAST	Ion Torrent	Newbler -large	0	Abeles SR. Ly M. Santiago-Rodriguez TM. Pride DT. Effects of Long Term Antibiotic Therapy on Human Oral and Fecal Viromes. PLoS One. 2015 Aug 26;10(8):e0134941. doi: 10.1371/journal.pone.0134941. eCollection 2015.
Pride_LongTermAntibio	ELA2B	HostAssoc_Human Feces	Human feces	USA : California	32.876609 -117.236885	reads MG-RAST	Ion Torrent	Newbler -large	0	
Pride_LongTermAntibio	ELA2C	HostAssoc_Human Feces	Human feces	USA : California	32.876609 -117.236885	reads MG-RAST	Ion Torrent	Newbler -large	0	
Pride_LongTermAntibio	ELA33A	HostAssoc_Human Feces	Human feces	USA : California	32.876609 -117.236885	reads MG-RAST	Ion Torrent	Newbler -large	0	
Pride_LongTermAntibio	ELA33B	HostAssoc_Human Feces	Human feces	USA : California	32.876609 -117.236885	reads MG-RAST	Ion Torrent	Newbler -large	0	
Pride_LongTermAntibio	ELA33C	HostAssoc_Human Feces	Human feces	USA : California	32.876609 -117.236885	reads MG-RAST	Ion Torrent	Newbler -large	0	
Pride_LongTermAntibio	ELA3A	HostAssoc_Human Feces	Human feces	USA : California	32.876609 -117.236885	reads MG-RAST	Ion Torrent	Newbler -large	0	
Pride_LongTermAntibio	ELA3B	HostAssoc_Human Feces	Human feces	USA : California	32.876609 -117.236885	reads MG-RAST	Ion Torrent	Newbler -large	0	
Pride_LongTermAntibio	ELA3C	HostAssoc_Human Feces	Human feces	USA : California	32.876609 -117.236885	reads MG-RAST	Ion Torrent	Newbler -large	0	
Pride_LongTermAntibio	ELA4A	HostAssoc_Human Feces	Human feces	USA : California	32.876609 -117.236885	reads MG-RAST	Ion Torrent	Newbler -large	0	
Pride_LongTermAntibio	ELA4B	HostAssoc_Human Feces	Human feces	USA : California	32.876609 -117.236885	reads MG-RAST	Ion Torrent	Newbler -large	0	
Pride_LongTermAntibio	ELA4C	HostAssoc_Human Feces	Human feces	USA : California	32.876609 -117.236885	reads MG-RAST	Ion Torrent	Newbler -large	0	
Pride_LongTermAntibio	ELA7A	HostAssoc_Human Feces	Human feces	USA : California	32.876609 -117.236885	reads MG-RAST	Ion Torrent	Newbler -large	0	
Pride_LongTermAntibio	ELA7B	HostAssoc_Human Feces	Human feces	USA : California	32.876609 -117.236885	reads MG-RAST	Ion Torrent	Newbler -large	0	
Pride_LongTermAntibio	ELA7C	HostAssoc_Human Feces	Human feces	USA : California	32.876609 -117.236885	reads MG-RAST	Ion Torrent	Newbler -large	0	
Pride_LongTermAntibio	ELA8A	HostAssoc_Human Feces	Human feces	USA : California	32.876609 -117.236885	reads MG-RAST	Ion Torrent	Newbler -large	0	
Pride_LongTermAntibio	ELA8B	HostAssoc_Human Feces	Human feces	USA : California	32.876609 -117.236885	reads MG-RAST	Ion Torrent	Newbler -large	0	
Pride_LongTermAntibio	ELA8C	HostAssoc_Human Feces	Human feces	USA : California	32.876609 -117.236885	reads MG-RAST	Ion Torrent	Newbler -large	0	
Pride_LongTermAntibio	ELA9A	HostAssoc_Human Feces	Human feces	USA : California	32.876609 -117.236885	reads MG-RAST	Ion Torrent	Newbler -large	0	
Pride_LongTermAntibio	ELA9B	HostAssoc_Human Feces	Human feces	USA : California	32.876609 -117.236885	reads MG-RAST	Ion Torrent	Newbler -large	0	
Pride_LongTermAntibio	ELA9C	HostAssoc_Human Feces	Human feces	USA : California	32.876609 -117.236885	reads MG-RAST	Ion Torrent	Newbler -large	0	
Reyes_Malnutrition	Malnut4990	HostAssoc_Human Feces	Human feces	Malawi	-13.754313 33.929313	reads ENA	Illumina MiSeq	Newbler	0	
Reyes_Malnutrition	Malnut4991	HostAssoc_Human Feces	Human feces	Malawi	-13.754313 33.929313	reads ENA	Illumina MiSeq	Newbler	0	
Reyes_Malnutrition	Malnut4992	HostAssoc_Human Feces	Human feces	Malawi	-13.754313 33.929313	reads ENA	Illumina MiSeq	Newbler	0	
Reyes_Malnutrition	Malnut4993	HostAssoc_Human Feces	Human feces	Malawi	-13.754313 33.929313	reads ENA	Illumina MiSeq	Newbler	0	
Reyes_Malnutrition	Malnut4994	HostAssoc_Human Feces	Human feces	Malawi	-13.754313 33.929313	reads ENA	Illumina MiSeq	Newbler	0	
Reyes_Malnutrition	Malnut4995	HostAssoc_Human Feces	Human feces	Malawi	-13.754313 33.929313	reads ENA	Illumina MiSeq	Newbler	0	
Reyes_Malnutrition	Malnut4996	HostAssoc_Human Feces	Human feces	Malawi	-13.754313 33.929313	reads ENA	Illumina MiSeq	Newbler	0	
Reyes_Malnutrition	Malnut4997	HostAssoc_Human Feces	Human feces	Malawi	-13.754313 33.929313	reads ENA	Illumina MiSeq	Newbler	0	
Reyes_Malnutrition	Malnut4998	HostAssoc_Human Feces	Human feces	Malawi	-13.754313 33.929313	reads ENA	Illumina MiSeq	Newbler	0	
Reyes_Malnutrition	Malnut4999	HostAssoc_Human Feces	Human feces	Malawi	-13.754313 33.929313	reads ENA	Illumina MiSeq	Newbler	0	
Reyes_Malnutrition	Malnut5000	HostAssoc_Human Feces	Human feces	Malawi	-13.754313 33.929313	reads ENA	Illumina MiSeq	Newbler	0	
Reyes_Malnutrition	Malnut5002	HostAssoc_Human Feces	Human feces	Malawi	-13.754313 33.929313	reads ENA	Illumina MiSeq	Newbler	0	
Reyes_Malnutrition	Malnut5003	HostAssoc_Human Feces	Human feces	Malawi	-13.754313 33.929313	reads ENA	Illumina MiSeq	Newbler	0	
Reyes_Malnutrition	Malnut5004	HostAssoc_Human Feces	Human feces	Malawi	-13.754313 33.929313	reads ENA	Illumina MiSeq	Newbler	0	
Reyes_Malnutrition	Malnut5005	HostAssoc_Human Feces	Human feces	Malawi	-13.754313 33.929313	reads ENA	Illumina MiSeq	Newbler	0	
Reyes_Malnutrition	Malnut5006	HostAssoc_Human Feces	Human feces	Malawi	-13.754313 33.929313	reads ENA	Illumina MiSeq	Newbler	0	
Reyes_Malnutrition	Malnut5007	HostAssoc_Human Feces	Human feces	Malawi	-13.754313 33.929313	reads ENA	Illumina MiSeq	Newbler	0	
Reyes_Malnutrition	Malnut5008	HostAssoc_Human Feces	Human feces	Malawi	-13.754313 33.929313	reads ENA	Illumina MiSeq	Newbler	0	
Reyes_Malnutrition	Malnut5009	HostAssoc_Human Feces	Human feces	Malawi	-13.754313 33.929313	reads ENA	Illumina MiSeq	Newbler	0	
Reyes_Malnutrition	Malnut5010	HostAssoc_Human Feces	Human feces	Malawi	-13.754313 33.929313	reads ENA	Illumina MiSeq	Newbler	0	
Reyes_Malnutrition	Malnut5011	HostAssoc_Human Feces	Human feces	Malawi	-13.754313 33.929313	reads ENA	Illumina MiSeq	Newbler	0	

Project Name	Virome Code	Habitat Type (code)	Ecosystem Type (text)	Location	GPS	Source	Sequencing Technology	Assembly Software	# crucivirus	Reference
Reyes_Malnutrition	Malnut5012	HostAssoc_Human Feces	Human feces	Malawi	-13.754313 33.929313	reads ENA	Illumina MiSeq	Newbler	0	
Reyes_Malnutrition	Malnut5013	HostAssoc_Human Feces	Human feces	Malawi	-13.754313 33.929313	reads ENA	Illumina MiSeq	Newbler	0	
Reyes_Malnutrition	Malnut5014	HostAssoc_Human Feces	Human feces	Malawi	-13.754313 33.929313	reads ENA	Illumina MiSeq	Newbler	0	
Reyes_Malnutrition	Malnut5015	HostAssoc_Human Feces	Human feces	Malawi	-13.754313 33.929313	reads ENA	Illumina MiSeq	Newbler	0	
Reyes_Malnutrition	Malnut5016	HostAssoc_Human Feces	Human feces	Malawi	-13.754313 33.929313	reads ENA	Illumina MiSeq	Newbler	0	
Reyes_Malnutrition	Malnut5017	HostAssoc_Human Feces	Human feces	Malawi	-13.754313 33.929313	reads ENA	Illumina MiSeq	Newbler	0	
Reyes_Malnutrition	Malnut5018	HostAssoc_Human Feces	Human feces	Malawi	-13.754313 33.929313	reads ENA	Illumina MiSeq	Newbler	0	
Reyes_Malnutrition	Malnut5019	HostAssoc_Human Feces	Human feces	Malawi	-13.754313 33.929313	reads ENA	Illumina MiSeq	Newbler	0	
Reyes_Malnutrition	Malnut5020	HostAssoc_Human Feces	Human feces	Malawi	-13.754313 33.929313	reads ENA	Illumina MiSeq	Newbler	0	
Reyes_Malnutrition	Malnut5021	HostAssoc_Human Feces	Human feces	Malawi	-13.754313 33.929313	reads ENA	Illumina MiSeq	Newbler	0	
Reyes_Malnutrition	Malnut5022	HostAssoc_Human Feces	Human feces	Malawi	-13.754313 33.929313	reads ENA	Illumina MiSeq	Newbler	0	
Reyes_Malnutrition	Malnut5023	HostAssoc_Human Feces	Human feces	Malawi	-13.754313 33.929313	reads ENA	Illumina MiSeq	Newbler	0	
Reyes_Malnutrition	Malnut5024	HostAssoc_Human Feces	Human feces	Malawi	-13.754313 33.929313	reads ENA	Illumina MiSeq	Newbler	0	
Reyes_Malnutrition	Malnut5025	HostAssoc_Human Feces	Human feces	Malawi	-13.754313 33.929313	reads ENA	Illumina MiSeq	Newbler	0	
Reyes_Malnutrition	Malnut5026	HostAssoc_Human Feces	Human feces	Malawi	-13.754313 33.929313	reads ENA	Illumina MiSeq	Newbler	0	
Reyes_Malnutrition	Malnut5027	HostAssoc_Human Feces	Human feces	Malawi	-13.754313 33.929313	reads ENA	Illumina MiSeq	Newbler	0	
Reyes_Malnutrition	Malnut5028	HostAssoc_Human Feces	Human feces	Malawi	-13.754313 33.929313	reads ENA	Illumina MiSeq	Newbler	0	
Reyes_Malnutrition	Malnut5029	HostAssoc_Human Feces	Human feces	Malawi	-13.754313 33.929313	reads ENA	Illumina MiSeq	Newbler	0	
Reyes_Malnutrition	Malnut5030	HostAssoc_Human Feces	Human feces	Malawi	-13.754313 33.929313	reads ENA	Illumina MiSeq	Newbler	0	
Reyes_Malnutrition	Malnut5031	HostAssoc_Human Feces	Human feces	Malawi	-13.754313 33.929313	reads ENA	Illumina MiSeq	Newbler	0	
Reyes_Malnutrition	Malnut5032	HostAssoc_Human Feces	Human feces	Malawi	-13.754313 33.929313	reads ENA	Illumina MiSeq	Newbler	0	
Reyes_Malnutrition	Malnut5033	HostAssoc_Human Feces	Human feces	Malawi	-13.754313 33.929313	reads ENA	Illumina MiSeq	Newbler	0	
Reyes_Malnutrition	Malnut5034	HostAssoc_Human Feces	Human feces	Malawi	-13.754313 33.929313	reads ENA	Illumina MiSeq	Newbler	0	
Reyes_Malnutrition	Malnut5035	HostAssoc_Human Feces	Human feces	Malawi	-13.754313 33.929313	reads ENA	Illumina MiSeq	Newbler	0	
Reyes_Malnutrition	Malnut5036	HostAssoc_Human Feces	Human feces	Malawi	-13.754313 33.929313	reads ENA	Illumina MiSeq	Newbler	0	
Reyes_Malnutrition	Malnut5037	HostAssoc_Human Feces	Human feces	Malawi	-13.754313 33.929313	reads ENA	Illumina MiSeq	Newbler	0	
Reyes_Malnutrition	Malnut5038	HostAssoc_Human Feces	Human feces	Malawi	-13.754313 33.929313	reads ENA	Illumina MiSeq	Newbler	0	
Reyes_Malnutrition	Malnut5039	HostAssoc_Human Feces	Human feces	Malawi	-13.754313 33.929313	reads ENA	Illumina MiSeq	Newbler	0	
Reyes_Malnutrition	Malnut5040	HostAssoc_Human Feces	Human feces	Malawi	-13.754313 33.929313	reads ENA	Illumina MiSeq	Newbler	0	
Reyes_Malnutrition	Malnut5041	HostAssoc_Human Feces	Human feces	Malawi	-13.754313 33.929313	reads ENA	Illumina MiSeq	Newbler	0	
Reyes_Malnutrition	Malnut5042	HostAssoc_Human Feces	Human feces	Malawi	-13.754313 33.929313	reads ENA	Illumina MiSeq	Newbler	0	
Reyes_Malnutrition	Malnut5043	HostAssoc_Human Feces	Human feces	Malawi	-13.754313 33.929313	reads ENA	Illumina MiSeq	Newbler	0	
Reyes_Malnutrition	Malnut5044	HostAssoc_Human Feces	Human feces	Malawi	-13.754313 33.929313	reads ENA	Illumina MiSeq	Newbler	0	
Reyes_Malnutrition	Malnut5045	HostAssoc_Human Feces	Human feces	Malawi	-13.754313 33.929313	reads ENA	Illumina MiSeq	Newbler	0	
Reyes_Malnutrition	Malnut5046	HostAssoc_Human Feces	Human feces	Malawi	-13.754313 33.929313	reads ENA	Illumina MiSeq	Newbler	0	
Reyes_Malnutrition	Malnut5047	HostAssoc_Human Feces	Human feces	Malawi	-13.754313 33.929313	reads ENA	Illumina MiSeq	Newbler	0	
Reyes_Malnutrition	Malnut5048	HostAssoc_Human Feces	Human feces	Malawi	-13.754313 33.929313	reads ENA	Illumina MiSeq	Newbler	0	
Reyes_Malnutrition	Malnut5049	HostAssoc_Human Feces	Human feces	Malawi	-13.754313 33.929313	reads ENA	Illumina MiSeq	Newbler	0	
Reyes_Malnutrition	Malnut5050	HostAssoc_Human Feces	Human feces	Malawi	-13.754313 33.929313	reads ENA	Illumina MiSeq	Newbler	0	
Reyes_Malnutrition	Malnut5051	HostAssoc_Human Feces	Human feces	Malawi	-13.754313 33.929313	reads ENA	Illumina MiSeq	Newbler	0	
Reyes_Malnutrition	Malnut5052	HostAssoc_Human Feces	Human feces	Malawi	-13.754313 33.929313	reads ENA	Illumina MiSeq	Newbler	0	
Reyes_Malnutrition	Malnut5053	HostAssoc_Human Feces	Human feces	Malawi	-13.754313 33.929313	reads ENA	Illumina MiSeq	Newbler	0	

Project Name	Virome Code	Habitat Type (code)	Ecosystem Type (text)	Location	GPS	Source	Sequencing Technology	Assembly Software	# crucivirus	Reference
Reyes_Malnutrition	Malnut5054	HostAssoc_Human Feces	Human feces	Malawi	-13.754313 33.929313	reads ENA	Illumina MiSeq	Newbler	0	
Reyes_Malnutrition	Malnut5055	HostAssoc_Human Feces	Human feces	Malawi	-13.754313 33.929313	reads ENA	Illumina MiSeq	Newbler	0	
Reyes_Malnutrition	Malnut5056	HostAssoc_Human Feces	Human feces	Malawi	-13.754313 33.929313	reads ENA	Illumina MiSeq	Newbler	0	
Reyes_Malnutrition	Malnut5057	HostAssoc_Human Feces	Human feces	Malawi	-13.754313 33.929313	reads ENA	Illumina MiSeq	Newbler	0	
Reyes_Malnutrition	Malnut5058	HostAssoc_Human Feces	Human feces	Malawi	-13.754313 33.929313	reads ENA	Illumina MiSeq	Newbler	0	
Reyes_Malnutrition	Malnut5059	HostAssoc_Human Feces	Human feces	Malawi	-13.754313 33.929313	reads ENA	Illumina MiSeq	Newbler	0	
Reyes_Malnutrition	Malnut5060	HostAssoc_Human Feces	Human feces	Malawi	-13.754313 33.929313	reads ENA	Illumina MiSeq	Newbler	0	
Reyes_Malnutrition	Malnut5061	HostAssoc_Human Feces	Human feces	Malawi	-13.754313 33.929313	reads ENA	Illumina MiSeq	Newbler	0	
Reyes_Malnutrition	Malnut5062	HostAssoc_Human Feces	Human feces	Malawi	-13.754313 33.929313	reads ENA	Illumina MiSeq	Newbler	0	
Reyes_Malnutrition	Malnut5063	HostAssoc_Human Feces	Human feces	Malawi	-13.754313 33.929313	reads ENA	Illumina MiSeq	Newbler	0	
Reyes_Malnutrition	Malnut5064	HostAssoc_Human Feces	Human feces	Malawi	-13.754313 33.929313	reads ENA	Illumina MiSeq	Newbler	0	
Reyes_Malnutrition	Malnut5065	HostAssoc_Human Feces	Human feces	Malawi	-13.754313 33.929313	reads ENA	Illumina MiSeq	Newbler	0	
Reyes_Malnutrition	Malnut5066	HostAssoc_Human Feces	Human feces	Malawi	-13.754313 33.929313	reads ENA	Illumina MiSeq	Newbler	0	
Reyes_Malnutrition	Malnut5067	HostAssoc_Human Feces	Human feces	Malawi	-13.754313 33.929313	reads ENA	Illumina MiSeq	Newbler	0	
Reyes_Malnutrition	Malnut5068	HostAssoc_Human Feces	Human feces	Malawi	-13.754313 33.929313	reads ENA	Illumina MiSeq	Newbler	0	
Reyes_Malnutrition	Malnut5069	HostAssoc_Human Feces	Human feces	Malawi	-13.754313 33.929313	reads ENA	Illumina MiSeq	Newbler	0	
Reyes_Malnutrition	Malnut5070	HostAssoc_Human Feces	Human feces	Malawi	-13.754313 33.929313	reads ENA	Illumina MiSeq	Newbler	0	
Reyes_Malnutrition	Malnut5071	HostAssoc_Human Feces	Human feces	Malawi	-13.754313 33.929313	reads ENA	Illumina MiSeq	Newbler	0	
Reyes_Malnutrition	Malnut5072	HostAssoc_Human Feces	Human feces	Malawi	-13.754313 33.929313	reads ENA	Illumina MiSeq	Newbler	0	
Reyes_Malnutrition	Malnut5073	HostAssoc_Human Feces	Human feces	Malawi	-13.754313 33.929313	reads ENA	Illumina MiSeq	Newbler	0	
Reyes_Malnutrition	Malnut5074	HostAssoc_Human Feces	Human feces	Malawi	-13.754313 33.929313	reads ENA	Illumina MiSeq	Newbler	0	
Reyes_Malnutrition	Malnut5075	HostAssoc_Human Feces	Human feces	Malawi	-13.754313 33.929313	reads ENA	Illumina MiSeq	Newbler	0	
Reyes_Malnutrition	Malnut5076	HostAssoc_Human Feces	Human feces	Malawi	-13.754313 33.929313	reads ENA	Illumina MiSeq	Newbler	0	
Reyes_Malnutrition	Malnut5077	HostAssoc_Human Feces	Human feces	Malawi	-13.754313 33.929313	reads ENA	Illumina MiSeq	Newbler	0	
Reyes_Malnutrition	Malnut5078	HostAssoc_Human Feces	Human feces	Malawi	-13.754313 33.929313	reads ENA	Illumina MiSeq	Newbler	0	
Reyes_Malnutrition	Malnut5079	HostAssoc_Human Feces	Human feces	Malawi	-13.754313 33.929313	reads ENA	Illumina MiSeq	Newbler	0	
Reyes_Malnutrition	Malnut5080	HostAssoc_Human Feces	Human feces	Malawi	-13.754313 33.929313	reads ENA	Illumina MiSeq	Newbler	0	
Reyes_Malnutrition	Malnut5081	HostAssoc_Human Feces	Human feces	Malawi	-13.754313 33.929313	reads ENA	Illumina MiSeq	Newbler	0	
Reyes_Malnutrition	Malnut5082	HostAssoc_Human Feces	Human feces	Malawi	-13.754313 33.929313	reads ENA	Illumina MiSeq	Newbler	0	
Reyes_Malnutrition	Malnut5084	HostAssoc_Human Feces	Human feces	Malawi	-13.754313 33.929313	reads ENA	Illumina MiSeq	Newbler	0	
Reyes_Malnutrition	Malnut5086	HostAssoc_Human Feces	Human feces	Malawi	-13.754313 33.929313	reads ENA	Illumina MiSeq	Newbler	0	
Reyes_Malnutrition	Malnut5087	HostAssoc_Human Feces	Human feces	Malawi	-13.754313 33.929313	reads ENA	Illumina MiSeq	Newbler	0	
Reyes_Malnutrition	Malnut5088	HostAssoc_Human Feces	Human feces	Malawi	-13.754313 33.929313	reads ENA	Illumina MiSeq	Newbler	0	
Reyes_Malnutrition	Malnut5089	HostAssoc_Human Feces	Human feces	Malawi	-13.754313 33.929313	reads ENA	Illumina MiSeq	Newbler	0	
Reyes_Malnutrition	Malnut5090	HostAssoc_Human Feces	Human feces	Malawi	-13.754313 33.929313	reads ENA	Illumina MiSeq	Newbler	0	
Reyes_Malnutrition	Malnut5091	HostAssoc_Human Feces	Human feces	Malawi	-13.754313 33.929313	reads ENA	Illumina MiSeq	Newbler	0	
Reyes_Malnutrition	Malnut5092	HostAssoc_Human Feces	Human feces	Malawi	-13.754313 33.929313	reads ENA	Illumina MiSeq	Newbler	0	
Reyes_Malnutrition	Malnut5093	HostAssoc_Human Feces	Human feces	Malawi	-13.754313 33.929313	reads ENA	Illumina MiSeq	Newbler	0	
Reyes_Malnutrition	Malnut5094	HostAssoc_Human Feces	Human feces	Malawi	-13.754313 33.929313	reads ENA	Illumina MiSeq	Newbler	0	
Reyes_Malnutrition	Malnut5095	HostAssoc_Human Feces	Human feces	Malawi	-13.754313 33.929313	reads ENA	Illumina MiSeq	Newbler	0	
Reyes_Malnutrition	Malnut5096	HostAssoc_Human Feces	Human feces	Malawi	-13.754313 33.929313	reads ENA	Illumina MiSeq	Newbler	0	
Reyes_Malnutrition	Malnut5097	HostAssoc_Human Feces	Human feces	Malawi	-13.754313 33.929313	reads ENA	Illumina MiSeq	Newbler	0	

Project Name	Virome Code	Habitat Type (code)	Ecosystem Type (text)	Location	GPS	Source	Sequencing Technology	Assembly Software	# crucivirus	Reference
Reyes_Malnutrition	Malnut5098	HostAssoc_Human Feces	Human feces	Malawi	-13.754313 33.929313	reads ENA	Illumina MiSeq	Newbler	0	
Reyes_Malnutrition	Malnut5099	HostAssoc_Human Feces	Human feces	Malawi	-13.754313 33.929313	reads ENA	Illumina MiSeq	Newbler	0	
Reyes_Malnutrition	Malnut5100	HostAssoc_Human Feces	Human feces	Malawi	-13.754313 33.929313	reads ENA	Illumina MiSeq	Newbler	0	
Reyes_Malnutrition	Malnut5101	HostAssoc_Human Feces	Human feces	Malawi	-13.754313 33.929313	reads ENA	Illumina MiSeq	Newbler	0	
Reyes_Malnutrition	Malnut5102	HostAssoc_Human Feces	Human feces	Malawi	-13.754313 33.929313	reads ENA	Illumina MiSeq	Newbler	0	
Reyes_Malnutrition	Malnut5103	HostAssoc_Human Feces	Human feces	Malawi	-13.754313 33.929313	reads ENA	Illumina MiSeq	Newbler	0	
Reyes_Malnutrition	Malnut5104	HostAssoc_Human Feces	Human feces	Malawi	-13.754313 33.929313	reads ENA	Illumina MiSeq	Newbler	0	
Reyes_Malnutrition	Malnut5105	HostAssoc_Human Feces	Human feces	Malawi	-13.754313 33.929313	reads ENA	Illumina MiSeq	Newbler	0	
Reyes_Malnutrition	Malnut5106	HostAssoc_Human Feces	Human feces	Malawi	-13.754313 33.929313	reads ENA	Illumina MiSeq	Newbler	0	
Reyes_Malnutrition	Malnut5107	HostAssoc_Human Feces	Human feces	Malawi	-13.754313 33.929313	reads ENA	Illumina MiSeq	Newbler	0	
Reyes_Malnutrition	Malnut5108	HostAssoc_Human Feces	Human feces	Malawi	-13.754313 33.929313	reads ENA	Illumina MiSeq	Newbler	0	
Reyes_Malnutrition	Malnut5109	HostAssoc_Human Feces	Human feces	Malawi	-13.754313 33.929313	reads ENA	Illumina MiSeq	Newbler	0	
Reyes_Malnutrition	Malnut5110	HostAssoc_Human Feces	Human feces	Malawi	-13.754313 33.929313	reads ENA	Illumina MiSeq	Newbler	0	
Reyes_Malnutrition	Malnut5111	HostAssoc_Human Feces	Human feces	Malawi	-13.754313 33.929313	reads ENA	Illumina MiSeq	Newbler	0	
Reyes_Malnutrition	Malnut5112	HostAssoc_Human Feces	Human feces	Malawi	-13.754313 33.929313	reads ENA	Illumina MiSeq	Newbler	0	
Reyes_Malnutrition	Malnut5113	HostAssoc_Human Feces	Human feces	Malawi	-13.754313 33.929313	reads ENA	Illumina MiSeq	Newbler	0	
Reyes_Malnutrition	Malnut5114	HostAssoc_Human Feces	Human feces	Malawi	-13.754313 33.929313	reads ENA	Illumina MiSeq	Newbler	0	
Reyes_Malnutrition	Malnut5115	HostAssoc_Human Feces	Human feces	Malawi	-13.754313 33.929313	reads ENA	Illumina MiSeq	Newbler	0	
Reyes_Malnutrition	Malnut5116	HostAssoc_Human Feces	Human feces	Malawi	-13.754313 33.929313	reads ENA	Illumina MiSeq	Newbler	0	
Reyes_Malnutrition	Malnut5117	HostAssoc_Human Feces	Human feces	Malawi	-13.754313 33.929313	reads ENA	Illumina MiSeq	Newbler	0	
Reyes_Malnutrition	Malnut5118	HostAssoc_Human Feces	Human feces	Malawi	-13.754313 33.929313	reads ENA	Illumina MiSeq	Newbler	0	
Reyes_Malnutrition	Malnut5119	HostAssoc_Human Feces	Human feces	Malawi	-13.754313 33.929313	reads ENA	Illumina MiSeq	Newbler	0	
Reyes_Malnutrition	Malnut5120	HostAssoc_Human Feces	Human feces	Malawi	-13.754313 33.929313	reads ENA	Illumina MiSeq	Newbler	0	
Reyes_Malnutrition	Malnut5121	HostAssoc_Human Feces	Human feces	Malawi	-13.754313 33.929313	reads ENA	Illumina MiSeq	Newbler	0	
Reyes_Malnutrition	Malnut5122	HostAssoc_Human Feces	Human feces	Malawi	-13.754313 33.929313	reads ENA	Illumina MiSeq	Newbler	0	
Reyes_Malnutrition	Malnut5123	HostAssoc_Human Feces	Human feces	Malawi	-13.754313 33.929313	reads ENA	Illumina MiSeq	Newbler	0	
Reyes_Malnutrition	Malnut5124	HostAssoc_Human Feces	Human feces	Malawi	-13.754313 33.929313	reads ENA	Illumina MiSeq	Newbler	0	
Reyes_Malnutrition	Malnut5125	HostAssoc_Human Feces	Human feces	Malawi	-13.754313 33.929313	reads ENA	Illumina MiSeq	Newbler	0	
Reyes_Malnutrition	Malnut5126	HostAssoc_Human Feces	Human feces	Malawi	-13.754313 33.929313	reads ENA	Illumina MiSeq	Newbler	0	
Reyes_Malnutrition	Malnut5127	HostAssoc_Human Feces	Human feces	Malawi	-13.754313 33.929313	reads ENA	Illumina MiSeq	Newbler	0	
Reyes_Malnutrition	Malnut5128	HostAssoc_Human Feces	Human feces	Malawi	-13.754313 33.929313	reads ENA	Illumina MiSeq	Newbler	0	
Reyes_Malnutrition	Malnut5129	HostAssoc_Human Feces	Human feces	Malawi	-13.754313 33.929313	reads ENA	Illumina MiSeq	Newbler	0	
Reyes_Malnutrition	Malnut5130	HostAssoc_Human Feces	Human feces	Malawi	-13.754313 33.929313	reads ENA	Illumina MiSeq	Newbler	0	
Reyes_Malnutrition	Malnut5131	HostAssoc_Human Feces	Human feces	Malawi	-13.754313 33.929313	reads ENA	Illumina MiSeq	Newbler	0	
Reyes_Malnutrition	Malnut5132	HostAssoc_Human Feces	Human feces	Malawi	-13.754313 33.929313	reads ENA	Illumina MiSeq	Newbler	0	
Reyes_Malnutrition	Malnut5133	HostAssoc_Human Feces	Human feces	Malawi	-13.754313 33.929313	reads ENA	Illumina MiSeq	Newbler	0	
Reyes_Malnutrition	Malnut5134	HostAssoc_Human Feces	Human feces	Malawi	-13.754313 33.929313	reads ENA	Illumina MiSeq	Newbler	0	
Reyes_Malnutrition	Malnut5135	HostAssoc_Human Feces	Human feces	Malawi	-13.754313 33.929313	reads ENA	Illumina MiSeq	Newbler	0	
Reyes_Malnutrition	Malnut5136	HostAssoc_Human Feces	Human feces	Malawi	-13.754313 33.929313	reads ENA	Illumina MiSeq	Newbler	0	
Reyes_Malnutrition	Malnut5137	HostAssoc_Human Feces	Human feces	Malawi	-13.754313 33.929313	reads ENA	Illumina MiSeq	Newbler	0	
Reyes_Malnutrition	Malnut5138	HostAssoc_Human Feces	Human feces	Malawi	-13.754313 33.929313	reads ENA	Illumina MiSeq	Newbler	0	
Reyes_Malnutrition	Malnut5139	HostAssoc_Human Feces	Human feces	Malawi	-13.754313 33.929313	reads ENA	Illumina MiSeq	Newbler	0	

Project Name	Virome Code	Habitat Type (code)	Ecosystem Type (text)	Location	GPS	Source	Sequencing Technology	Assembly Software	# crucivirus	Reference
Reyes_Malnutrition	Malnut5140	HostAssoc_Human Feces	Human feces	Malawi	-13.754313 33.929313	reads ENA	Illumina MiSeq	Newbler	0	
Reyes_Malnutrition	Malnut5141	HostAssoc_Human Feces	Human feces	Malawi	-13.754313 33.929313	reads ENA	Illumina MiSeq	Newbler	0	
Reyes_Malnutrition	Malnut5142	HostAssoc_Human Feces	Human feces	Malawi	-13.754313 33.929313	reads ENA	Illumina MiSeq	Newbler	0	
Reyes_Malnutrition	Malnut5143	HostAssoc_Human Feces	Human feces	Malawi	-13.754313 33.929313	reads ENA	Illumina MiSeq	Newbler	0	
Reyes_Malnutrition	Malnut5144	HostAssoc_Human Feces	Human feces	Malawi	-13.754313 33.929313	reads ENA	Illumina MiSeq	Newbler	0	
Reyes_Malnutrition	Malnut5145	HostAssoc_Human Feces	Human feces	Malawi	-13.754313 33.929313	reads ENA	Illumina MiSeq	Newbler	0	
Reyes_Malnutrition	Malnut5146	HostAssoc_Human Feces	Human feces	Malawi	-13.754313 33.929313	reads ENA	Illumina MiSeq	Newbler	0	
Reyes_Malnutrition	Malnut5147	HostAssoc_Human Feces	Human feces	Malawi	-13.754313 33.929313	reads ENA	Illumina MiSeq	Newbler	0	
Reyes_Malnutrition	Malnut5148	HostAssoc_Human Feces	Human feces	Malawi	-13.754313 33.929313	reads ENA	Illumina MiSeq	Newbler	0	
Reyes_Malnutrition	Malnut5149	HostAssoc_Human Feces	Human feces	Malawi	-13.754313 33.929313	reads ENA	Illumina MiSeq	Newbler	0	
Reyes_Malnutrition	Malnut5150	HostAssoc_Human Feces	Human feces	Malawi	-13.754313 33.929313	reads ENA	Illumina MiSeq	Newbler	0	
Reyes_Malnutrition	Malnut5151	HostAssoc_Human Feces	Human feces	Malawi	-13.754313 33.929313	reads ENA	Illumina MiSeq	Newbler	0	
Reyes_Malnutrition	Malnut5152	HostAssoc_Human Feces	Human feces	Malawi	-13.754313 33.929313	reads ENA	Illumina MiSeq	Newbler	0	
Reyes_Malnutrition	Malnut5153	HostAssoc_Human Feces	Human feces	Malawi	-13.754313 33.929313	reads ENA	Illumina MiSeq	Newbler	0	
Reyes_Malnutrition	Malnut5154	HostAssoc_Human Feces	Human feces	Malawi	-13.754313 33.929313	reads ENA	Illumina MiSeq	Newbler	0	
Reyes_Malnutrition	Malnut5155	HostAssoc_Human Feces	Human feces	Malawi	-13.754313 33.929313	reads ENA	Illumina MiSeq	Newbler	0	
Reyes_Malnutrition	Malnut5156	HostAssoc_Human Feces	Human feces	Malawi	-13.754313 33.929313	reads ENA	Illumina MiSeq	Newbler	0	Reyes A. Blanton LV. Cao S. Zhao G. Manary M. Trehan I. Smith MI. Wang D. Virgin HW. Rohwer F. Gordon JI. Gut DNA viromes of Malawian twins discordant for severe acute malnutrition. Proc Natl Acad Sci U S A. 2015 Sep 22;112(38):11941-6. doi: 10.1073/pnas.1514285112. Epub 2015 Sep 8.
Reyes_Malnutrition	Malnut5157	HostAssoc_Human Feces	Human feces	Malawi	-13.754313 33.929313	reads ENA	Illumina MiSeq	Newbler	0	
Reyes_Malnutrition	Malnut5158	HostAssoc_Human Feces	Human feces	Malawi	-13.754313 33.929313	reads ENA	Illumina MiSeq	Newbler	0	
Reyes_Malnutrition	Malnut5159	HostAssoc_Human Feces	Human feces	Malawi	-13.754313 33.929313	reads ENA	Illumina MiSeq	Newbler	0	
Reyes_Malnutrition	Malnut5160	HostAssoc_Human Feces	Human feces	Malawi	-13.754313 33.929313	reads ENA	Illumina MiSeq	Newbler	0	
Reyes_Malnutrition	Malnut5161	HostAssoc_Human Feces	Human feces	Malawi	-13.754313 33.929313	reads ENA	Illumina MiSeq	Newbler	0	
Reyes_Malnutrition	Malnut5162	HostAssoc_Human Feces	Human feces	Malawi	-13.754313 33.929313	reads ENA	Illumina MiSeq	Newbler	0	
Reyes_Malnutrition	Malnut5163	HostAssoc_Human Feces	Human feces	Malawi	-13.754313 33.929313	reads ENA	Illumina MiSeq	Newbler	0	
Reyes_Malnutrition	Malnut5164	HostAssoc_Human Feces	Human feces	Malawi	-13.754313 33.929313	reads ENA	Illumina MiSeq	Newbler	0	
Reyes_Malnutrition	Malnut5165	HostAssoc_Human Feces	Human feces	Malawi	-13.754313 33.929313	reads ENA	Illumina MiSeq	Newbler	0	
Reyes_Malnutrition	Malnut5166	HostAssoc_Human Feces	Human feces	Malawi	-13.754313 33.929313	reads ENA	Illumina MiSeq	Newbler	0	
Reyes_Malnutrition	Malnut5167	HostAssoc_Human Feces	Human feces	Malawi	-13.754313 33.929313	reads ENA	Illumina MiSeq	Newbler	0	
Reyes_Malnutrition	Malnut5168	HostAssoc_Human Feces	Human feces	Malawi	-13.754313 33.929313	reads ENA	Illumina MiSeq	Newbler	0	
Reyes_Malnutrition	Malnut5169	HostAssoc_Human Feces	Human feces	Malawi	-13.754313 33.929313	reads ENA	Illumina MiSeq	Newbler	0	
Reyes_Malnutrition	Malnut5170	HostAssoc_Human Feces	Human feces	Malawi	-13.754313 33.929313	reads ENA	Illumina MiSeq	Newbler	0	
Reyes_Malnutrition	Malnut5171	HostAssoc_Human Feces	Human feces	Malawi	-13.754313 33.929313	reads ENA	Illumina MiSeq	Newbler	0	
Reyes_Malnutrition	Malnut5172	HostAssoc_Human Feces	Human feces	Malawi	-13.754313 33.929313	reads ENA	Illumina MiSeq	Newbler	0	
Reyes_Malnutrition	Malnut5173	HostAssoc_Human Feces	Human feces	Malawi	-13.754313 33.929313	reads ENA	Illumina MiSeq	Newbler	0	
Reyes_Malnutrition	Malnut5174	HostAssoc_Human Feces	Human feces	Malawi	-13.754313 33.929313	reads ENA	Illumina MiSeq	Newbler	0	
Reyes_Malnutrition	Malnut5175	HostAssoc_Human Feces	Human feces	Malawi	-13.754313 33.929313	reads ENA	Illumina MiSeq	Newbler	0	
Reyes_Malnutrition	Malnut5176	HostAssoc_Human Feces	Human feces	Malawi	-13.754313 33.929313	reads ENA	Illumina MiSeq	Newbler	0	
Reyes_Malnutrition	Malnut5177	HostAssoc_Human Feces	Human feces	Malawi	-13.754313 33.929313	reads ENA	Illumina MiSeq	Newbler	0	
Reyes_Malnutrition	Malnut5178	HostAssoc_Human Feces	Human feces	Malawi	-13.754313 33.929313	reads ENA	Illumina MiSeq	Newbler	0	
Reyes_Malnutrition	Malnut5179	HostAssoc_Human Feces	Human feces	Malawi	-13.754313 33.929313	reads ENA	Illumina MiSeq	Newbler	0	
Reyes_Malnutrition	Malnut5180	HostAssoc_Human Feces	Human feces	Malawi	-13.754313 33.929313	reads ENA	Illumina MiSeq	Newbler	0	
Reyes_Malnutrition	Malnut5181	HostAssoc_Human Feces	Human feces	Malawi	-13.754313 33.929313	reads ENA	Illumina MiSeq	Newbler	0	

Project Name	Virome Code	Habitat Type (code)	Ecosystem Type (text)	Location	GPS	Source	Sequencing Technology	Assembly Software	# crucivirus	Reference
Reyes_Malnutrition	Malnut5182	HostAssoc_Human Feces	Human feces	Malawi	-13.754313 33.929313	reads ENA	Illumina MiSeq	Newbler	0	
Reyes_Malnutrition	Malnut5183	HostAssoc_Human Feces	Human feces	Malawi	-13.754313 33.929313	reads ENA	Illumina MiSeq	Newbler	0	
Reyes_Malnutrition	Malnut5184	HostAssoc_Human Feces	Human feces	Malawi	-13.754313 33.929313	reads ENA	Illumina MiSeq	Newbler	0	
Reyes_Malnutrition	Malnut5185	HostAssoc_Human Feces	Human feces	Malawi	-13.754313 33.929313	reads ENA	Illumina MiSeq	Newbler	0	
Reyes_Malnutrition	Malnut5186	HostAssoc_Human Feces	Human feces	Malawi	-13.754313 33.929313	reads ENA	Illumina MiSeq	Newbler	0	
Reyes_Malnutrition	Malnut5187	HostAssoc_Human Feces	Human feces	Malawi	-13.754313 33.929313	reads ENA	Illumina MiSeq	Newbler	0	
Reyes_Malnutrition	Malnut5188	HostAssoc_Human Feces	Human feces	Malawi	-13.754313 33.929313	reads ENA	Illumina MiSeq	Newbler	0	
Reyes_Malnutrition	Malnut5189	HostAssoc_Human Feces	Human feces	Malawi	-13.754313 33.929313	reads ENA	Illumina MiSeq	Newbler	0	
Reyes_Malnutrition	Malnut5190	HostAssoc_Human Feces	Human feces	Malawi	-13.754313 33.929313	reads ENA	Illumina MiSeq	Newbler	0	
Reyes_Malnutrition	Malnut5191	HostAssoc_Human Feces	Human feces	Malawi	-13.754313 33.929313	reads ENA	Illumina MiSeq	Newbler	0	
Reyes_Malnutrition	Malnut5192	HostAssoc_Human Feces	Human feces	Malawi	-13.754313 33.929313	reads ENA	Illumina MiSeq	Newbler	0	
Reyes_Malnutrition	Malnut5193	HostAssoc_Human Feces	Human feces	Malawi	-13.754313 33.929313	reads ENA	Illumina MiSeq	Newbler	0	
Reyes_Malnutrition	Malnut5194	HostAssoc_Human Feces	Human feces	Malawi	-13.754313 33.929313	reads ENA	Illumina MiSeq	Newbler	0	
Reyes_Malnutrition	Malnut5195	HostAssoc_Human Feces	Human feces	Malawi	-13.754313 33.929313	reads ENA	Illumina MiSeq	Newbler	0	
Reyes_Malnutrition	Malnut5196	HostAssoc_Human Feces	Human feces	Malawi	-13.754313 33.929313	reads ENA	Illumina MiSeq	Newbler	0	
Reyes_Malnutrition	Malnut5197	HostAssoc_Human Feces	Human feces	Malawi	-13.754313 33.929313	reads ENA	Illumina MiSeq	Newbler	0	
Reyes_Malnutrition	Malnut5198	HostAssoc_Human Feces	Human feces	Malawi	-13.754313 33.929313	reads ENA	Illumina MiSeq	Newbler	0	
Reyes_Malnutrition	Malnut5199	HostAssoc_Human Feces	Human feces	Malawi	-13.754313 33.929313	reads ENA	Illumina MiSeq	Newbler	0	
Reyes_Malnutrition	Malnut5200	HostAssoc_Human Feces	Human feces	Malawi	-13.754313 33.929313	reads ENA	Illumina MiSeq	Newbler	0	
Reyes_Malnutrition	Malnut5201	HostAssoc_Human Feces	Human feces	Malawi	-13.754313 33.929313	reads ENA	Illumina MiSeq	Newbler	0	
Reyes_Malnutrition	Malnut5202	HostAssoc_Human Feces	Human feces	Malawi	-13.754313 33.929313	reads ENA	Illumina MiSeq	Newbler	0	
Reyes_Malnutrition	Malnut5203	HostAssoc_Human Feces	Human feces	Malawi	-13.754313 33.929313	reads ENA	Illumina MiSeq	Newbler	0	
Reyes_Malnutrition	Malnut5204	HostAssoc_Human Feces	Human feces	Malawi	-13.754313 33.929313	reads ENA	Illumina MiSeq	Newbler	0	
Reyes_Malnutrition	Malnut5205	HostAssoc_Human Feces	Human feces	Malawi	-13.754313 33.929313	reads ENA	Illumina MiSeq	Newbler	0	
Reyes_Malnutrition	Malnut5206	HostAssoc_Human Feces	Human feces	Malawi	-13.754313 33.929313	reads ENA	Illumina MiSeq	Newbler	0	
Reyes_Malnutrition	Malnut5207	HostAssoc_Human Feces	Human feces	Malawi	-13.754313 33.929313	reads ENA	Illumina MiSeq	Newbler	0	
Reyes_Malnutrition	Malnut5208	HostAssoc_Human Feces	Human feces	Malawi	-13.754313 33.929313	reads ENA	Illumina MiSeq	Newbler	0	
Reyes_Malnutrition	Malnut5209	HostAssoc_Human Feces	Human feces	Malawi	-13.754313 33.929313	reads ENA	Illumina MiSeq	Newbler	0	
Reyes_Malnutrition	Malnut5210	HostAssoc_Human Feces	Human feces	Malawi	-13.754313 33.929313	reads ENA	Illumina MiSeq	Newbler	0	
Reyes_Malnutrition	Malnut5211	HostAssoc_Human Feces	Human feces	Malawi	-13.754313 33.929313	reads ENA	Illumina MiSeq	Newbler	0	
Reyes_Malnutrition	Malnut5212	HostAssoc_Human Feces	Human feces	Malawi	-13.754313 33.929313	reads ENA	Illumina MiSeq	Newbler	0	
Reyes_Malnutrition	Malnut5213	HostAssoc_Human Feces	Human feces	Malawi	-13.754313 33.929313	reads ENA	Illumina MiSeq	Newbler	0	
Reyes_Malnutrition	Malnut5214	HostAssoc_Human Feces	Human feces	Malawi	-13.754313 33.929313	reads ENA	Illumina MiSeq	Newbler	0	
Reyes_Malnutrition	Malnut5215	HostAssoc_Human Feces	Human feces	Malawi	-13.754313 33.929313	reads ENA	Illumina MiSeq	Newbler	0	
Reyes_Malnutrition	Malnut5216	HostAssoc_Human Feces	Human feces	Malawi	-13.754313 33.929313	reads ENA	Illumina MiSeq	Newbler	0	
Reyes_Malnutrition	Malnut5217	HostAssoc_Human Feces	Human feces	Malawi	-13.754313 33.929313	reads ENA	Illumina MiSeq	Newbler	0	
Reyes_Malnutrition	Malnut5218	HostAssoc_Human Feces	Human feces	Malawi	-13.754313 33.929313	reads ENA	Illumina MiSeq	Newbler	0	
Reyes_Malnutrition	Malnut5219	HostAssoc_Human Feces	Human feces	Malawi	-13.754313 33.929313	reads ENA	Illumina MiSeq	Newbler	0	
Reyes_Malnutrition	Malnut5220	HostAssoc_Human Feces	Human feces	Malawi	-13.754313 33.929313	reads ENA	Illumina MiSeq	Newbler	0	
Reyes_Malnutrition	Malnut5221	HostAssoc_Human Feces	Human feces	Malawi	-13.754313 33.929313	reads ENA	Illumina MiSeq	Newbler	0	
Reyes_Malnutrition	Malnut5222	HostAssoc_Human Feces	Human feces	Malawi	-13.754313 33.929313	reads ENA	Illumina MiSeq	Newbler	0	
Reyes_Malnutrition	Malnut5223	HostAssoc_Human Feces	Human feces	Malawi	-13.754313 33.929313	reads ENA	Illumina MiSeq	Newbler	0	

Project Name	Virome Code	Habitat Type (code)	Ecosystem Type (text)	Location	GPS	Source	Sequencing Technology	Assembly Software	# crucivirus	Reference
Reyes_Malnutrition	Malnut5224	HostAssoc_Human Feces	Human feces	Malawi	-13.754313 33.929313	reads ENA	Illumina MiSeq	Newbler	0	
Reyes_Malnutrition	Malnut5225	HostAssoc_Human Feces	Human feces	Malawi	-13.754313 33.929313	reads ENA	Illumina MiSeq	Newbler	0	
Reyes_Malnutrition	Malnut5226	HostAssoc_Human Feces	Human feces	Malawi	-13.754313 33.929313	reads ENA	Illumina MiSeq	Newbler	0	
Reyes_Malnutrition	Malnut5227	HostAssoc_Human Feces	Human feces	Malawi	-13.754313 33.929313	reads ENA	Illumina MiSeq	Newbler	0	
Reyes_Malnutrition	Malnut5228	HostAssoc_Human Feces	Human feces	Malawi	-13.754313 33.929313	reads ENA	Illumina MiSeq	Newbler	0	
Reyes_Malnutrition	Malnut5229	HostAssoc_Human Feces	Human feces	Malawi	-13.754313 33.929313	reads ENA	Illumina MiSeq	Newbler	0	
Reyes_Malnutrition	Malnut5230	HostAssoc_Human Feces	Human feces	Malawi	-13.754313 33.929313	reads ENA	Illumina MiSeq	Newbler	0	
Reyes_Malnutrition	Malnut5231	HostAssoc_Human Feces	Human feces	Malawi	-13.754313 33.929313	reads ENA	Illumina MiSeq	Newbler	0	
Reyes_Malnutrition	Malnut5232	HostAssoc_Human Feces	Human feces	Malawi	-13.754313 33.929313	reads ENA	Illumina MiSeq	Newbler	0	
Reyes_Malnutrition	Malnut5233	HostAssoc_Human Feces	Human feces	Malawi	-13.754313 33.929313	reads ENA	Illumina MiSeq	Newbler	0	
Reyes_Malnutrition	Malnut5234	HostAssoc_Human Feces	Human feces	Malawi	-13.754313 33.929313	reads ENA	Illumina MiSeq	Newbler	0	
Reyes_Malnutrition	Malnut5235	HostAssoc_Human Feces	Human feces	Malawi	-13.754313 33.929313	reads ENA	Illumina MiSeq	Newbler	0	
Reyes_Malnutrition	Malnut5236	HostAssoc_Human Feces	Human feces	Malawi	-13.754313 33.929313	reads ENA	Illumina MiSeq	Newbler	0	
Reyes_Malnutrition	Malnut5237	HostAssoc_Human Feces	Human feces	Malawi	-13.754313 33.929313	reads ENA	Illumina MiSeq	Newbler	0	
Reyes_Malnutrition	Malnut5238	HostAssoc_Human Feces	Human feces	Malawi	-13.754313 33.929313	reads ENA	Illumina MiSeq	Newbler	0	
Reyes_Malnutrition	Malnut5239	HostAssoc_Human Feces	Human feces	Malawi	-13.754313 33.929313	reads ENA	Illumina MiSeq	Newbler	0	
Reyes_Malnutrition	Malnut5240	HostAssoc_Human Feces	Human feces	Malawi	-13.754313 33.929313	reads ENA	Illumina MiSeq	Newbler	0	
Reyes_Malnutrition	Malnut5241	HostAssoc_Human Feces	Human feces	Malawi	-13.754313 33.929313	reads ENA	Illumina MiSeq	Newbler	0	
Reyes_Malnutrition	Malnut5242	HostAssoc_Human Feces	Human feces	Malawi	-13.754313 33.929313	reads ENA	Illumina MiSeq	Newbler	0	
Reyes_Malnutrition	Malnut5243	HostAssoc_Human Feces	Human feces	Malawi	-13.754313 33.929313	reads ENA	Illumina MiSeq	Newbler	0	
Reyes_Malnutrition	Malnut5244	HostAssoc_Human Feces	Human feces	Malawi	-13.754313 33.929313	reads ENA	Illumina MiSeq	Newbler	0	
Reyes_Malnutrition	Malnut5245	HostAssoc_Human Feces	Human feces	Malawi	-13.754313 33.929313	reads ENA	Illumina MiSeq	Newbler	0	
Reyes_Malnutrition	Malnut5246	HostAssoc_Human Feces	Human feces	Malawi	-13.754313 33.929313	reads ENA	Illumina MiSeq	Newbler	0	
Reyes_Malnutrition	Malnut5247	HostAssoc_Human Feces	Human feces	Malawi	-13.754313 33.929313	reads ENA	Illumina MiSeq	Newbler	0	
Reyes_Malnutrition	Malnut5248	HostAssoc_Human Feces	Human feces	Malawi	-13.754313 33.929313	reads ENA	Illumina MiSeq	Newbler	0	
Reyes_Malnutrition	Malnut5249	HostAssoc_Human Feces	Human feces	Malawi	-13.754313 33.929313	reads ENA	Illumina MiSeq	Newbler	0	
Reyes_Malnutrition	Malnut5250	HostAssoc_Human Feces	Human feces	Malawi	-13.754313 33.929313	reads ENA	Illumina MiSeq	Newbler	0	
Reyes_Malnutrition	Malnut5251	HostAssoc_Human Feces	Human feces	Malawi	-13.754313 33.929313	reads ENA	Illumina MiSeq	Newbler	0	
Reyes_Malnutrition	Malnut5252	HostAssoc_Human Feces	Human feces	Malawi	-13.754313 33.929313	reads ENA	Illumina MiSeq	Newbler	0	
Reyes_Malnutrition	Malnut5253	HostAssoc_Human Feces	Human feces	Malawi	-13.754313 33.929313	reads ENA	Illumina MiSeq	Newbler	0	
Reyes_Malnutrition	Malnut5254	HostAssoc_Human Feces	Human feces	Malawi	-13.754313 33.929313	reads ENA	Illumina MiSeq	Newbler	0	
Reyes_Malnutrition	Malnut5255	HostAssoc_Human Feces	Human feces	Malawi	-13.754313 33.929313	reads ENA	Illumina MiSeq	Newbler	0	
Reyes_Malnutrition	Malnut5256	HostAssoc_Human Feces	Human feces	Malawi	-13.754313 33.929313	reads ENA	Illumina MiSeq	Newbler	0	
Reyes_Malnutrition	Malnut5257	HostAssoc_Human Feces	Human feces	Malawi	-13.754313 33.929313	reads ENA	Illumina MiSeq	Newbler	0	
Reyes_Malnutrition	Malnut5258	HostAssoc_Human Feces	Human feces	Malawi	-13.754313 33.929313	reads ENA	Illumina MiSeq	Newbler	0	
Reyes_Malnutrition	Malnut5259	HostAssoc_Human Feces	Human feces	Malawi	-13.754313 33.929313	reads ENA	Illumina MiSeq	Newbler	0	
Reyes_Malnutrition	Malnut5260	HostAssoc_Human Feces	Human feces	Malawi	-13.754313 33.929313	reads ENA	Illumina MiSeq	Newbler	0	
Reyes_Malnutrition	Malnut5261	HostAssoc_Human Feces	Human feces	Malawi	-13.754313 33.929313	reads ENA	Illumina MiSeq	Newbler	0	
Reyes_Malnutrition	Malnut5262	HostAssoc_Human Feces	Human feces	Malawi	-13.754313 33.929313	reads ENA	Illumina MiSeq	Newbler	0	
Reyes_Malnutrition	Malnut5263	HostAssoc_Human Feces	Human feces	Malawi	-13.754313 33.929313	reads ENA	Illumina MiSeq	Newbler	0	
Reyes_Malnutrition	Malnut5264	HostAssoc_Human Feces	Human feces	Malawi	-13.754313 33.929313	reads ENA	Illumina MiSeq	Newbler	0	
Reyes_Malnutrition	Malnut5265	HostAssoc_Human Feces	Human feces	Malawi	-13.754313 33.929313	reads ENA	Illumina MiSeq	Newbler	0	

Project Name	Virome Code	Habitat Type (code)	Ecosystem Type (text)	Location	GPS	Source	Sequencing Technology	Assembly Software	# crucivirus	Reference
Reyes_Malnutrition	Malnut5266	HostAssoc_Human Feces	Human feces	Malawi	-13.754313 33.929313	reads ENA	Illumina MiSeq	Newbler	0	
Reyes_Malnutrition	Malnut5267	HostAssoc_Human Feces	Human feces	Malawi	-13.754313 33.929313	reads ENA	Illumina MiSeq	Newbler	0	
Reyes_Malnutrition	Malnut5268	HostAssoc_Human Feces	Human feces	Malawi	-13.754313 33.929313	reads ENA	Illumina MiSeq	Newbler	0	
Reyes_Malnutrition	Malnut5269	HostAssoc_Human Feces	Human feces	Malawi	-13.754313 33.929313	reads ENA	Illumina MiSeq	Newbler	0	
Reyes_Malnutrition	Malnut5270	HostAssoc_Human Feces	Human feces	Malawi	-13.754313 33.929313	reads ENA	Illumina MiSeq	Newbler	0	
Reyes_Malnutrition	Malnut5271	HostAssoc_Human Feces	Human feces	Malawi	-13.754313 33.929313	reads ENA	Illumina MiSeq	Newbler	0	
Reyes_Malnutrition	Malnut5272	HostAssoc_Human Feces	Human feces	Malawi	-13.754313 33.929313	reads ENA	Illumina MiSeq	Newbler	0	
Reyes_Malnutrition	Malnut5273	HostAssoc_Human Feces	Human feces	Malawi	-13.754313 33.929313	reads ENA	Illumina MiSeq	Newbler	0	
Reyes_Malnutrition	Malnut5274	HostAssoc_Human Feces	Human feces	Malawi	-13.754313 33.929313	reads ENA	Illumina MiSeq	Newbler	0	
Reyes_Malnutrition	Malnut5275	HostAssoc_Human Feces	Human feces	Malawi	-13.754313 33.929313	reads ENA	Illumina MiSeq	Newbler	0	
Reyes_Malnutrition	Malnut5276	HostAssoc_Human Feces	Human feces	Malawi	-13.754313 33.929313	reads ENA	Illumina MiSeq	Newbler	0	
Reyes_Malnutrition	Malnut5277	HostAssoc_Human Feces	Human feces	Malawi	-13.754313 33.929313	reads ENA	Illumina MiSeq	Newbler	0	
Reyes_Malnutrition	Malnut5278	HostAssoc_Human Feces	Human feces	Malawi	-13.754313 33.929313	reads ENA	Illumina MiSeq	Newbler	0	
Reyes_Malnutrition	Malnut5279	HostAssoc_Human Feces	Human feces	Malawi	-13.754313 33.929313	reads ENA	Illumina MiSeq	Newbler	0	
Reyes_Malnutrition	Malnut5280	HostAssoc_Human Feces	Human feces	Malawi	-13.754313 33.929313	reads ENA	Illumina MiSeq	Newbler	0	
Reyes_Malnutrition	Malnut5281	HostAssoc_Human Feces	Human feces	Malawi	-13.754313 33.929313	reads ENA	Illumina MiSeq	Newbler	0	
Reyes_Malnutrition	Malnut5282	HostAssoc_Human Feces	Human feces	Malawi	-13.754313 33.929313	reads ENA	Illumina MiSeq	Newbler	0	
Reyes_Malnutrition	Malnut5283	HostAssoc_Human Feces	Human feces	Malawi	-13.754313 33.929313	reads ENA	Illumina MiSeq	Newbler	0	
Reyes_Malnutrition	Malnut5284	HostAssoc_Human Feces	Human feces	Malawi	-13.754313 33.929313	reads ENA	Illumina MiSeq	Newbler	0	
Reyes_Malnutrition	Malnut5285	HostAssoc_Human Feces	Human feces	Malawi	-13.754313 33.929313	reads ENA	Illumina MiSeq	Newbler	0	
Reyes_Malnutrition	Malnut5286	HostAssoc_Human Feces	Human feces	Malawi	-13.754313 33.929313	reads ENA	Illumina MiSeq	Newbler	0	
Reyes_Malnutrition	Malnut5287	HostAssoc_Human Feces	Human feces	Malawi	-13.754313 33.929313	reads ENA	Illumina MiSeq	Newbler	0	
Reyes_Malnutrition	Malnut5288	HostAssoc_Human Feces	Human feces	Malawi	-13.754313 33.929313	reads ENA	Illumina MiSeq	Newbler	0	
Reyes_Malnutrition	Malnut5289	HostAssoc_Human Feces	Human feces	Malawi	-13.754313 33.929313	reads ENA	Illumina MiSeq	Newbler	0	
Reyes_Malnutrition	Malnut5290	HostAssoc_Human Feces	Human feces	Malawi	-13.754313 33.929313	reads ENA	Illumina MiSeq	Newbler	0	
Reyes_Malnutrition	Malnut5291	HostAssoc_Human Feces	Human feces	Malawi	-13.754313 33.929313	reads ENA	Illumina MiSeq	Newbler	0	
Reyes_Malnutrition	Malnut5292	HostAssoc_Human Feces	Human feces	Malawi	-13.754313 33.929313	reads ENA	Illumina MiSeq	Newbler	0	
Reyes_Malnutrition	Malnut5293	HostAssoc_Human Feces	Human feces	Malawi	-13.754313 33.929313	reads ENA	Illumina MiSeq	Newbler	0	
Reyes_Malnutrition	Malnut5294	HostAssoc_Human Feces	Human feces	Malawi	-13.754313 33.929313	reads ENA	Illumina MiSeq	Newbler	0	
Reyes_Malnutrition	Malnut5295	HostAssoc_Human Feces	Human feces	Malawi	-13.754313 33.929313	reads ENA	Illumina MiSeq	Newbler	0	
Reyes_Malnutrition	Malnut5296	HostAssoc_Human Feces	Human feces	Malawi	-13.754313 33.929313	reads ENA	Illumina MiSeq	Newbler	0	
Reyes_Malnutrition	Malnut5297	HostAssoc_Human Feces	Human feces	Malawi	-13.754313 33.929313	reads ENA	Illumina MiSeq	Newbler	0	
Reyes_Malnutrition	Malnut5298	HostAssoc_Human Feces	Human feces	Malawi	-13.754313 33.929313	reads ENA	Illumina MiSeq	Newbler	0	
Reyes_Malnutrition	Malnut5299	HostAssoc_Human Feces	Human feces	Malawi	-13.754313 33.929313	reads ENA	Illumina MiSeq	Newbler	0	
Reyes_Malnutrition	Malnut5300	HostAssoc_Human Feces	Human feces	Malawi	-13.754313 33.929313	reads ENA	Illumina MiSeq	Newbler	0	
Reyes_Malnutrition	Malnut5301	HostAssoc_Human Feces	Human feces	Malawi	-13.754313 33.929313	reads ENA	Illumina MiSeq	Newbler	0	
Reyes_Malnutrition	Malnut5302	HostAssoc_Human Feces	Human feces	Malawi	-13.754313 33.929313	reads ENA	Illumina MiSeq	Newbler	0	
Reyes_Malnutrition	Malnut5303	HostAssoc_Human Feces	Human feces	Malawi	-13.754313 33.929313	reads ENA	Illumina MiSeq	Newbler	0	
Reyes_Malnutrition	Malnut5304	HostAssoc_Human Feces	Human feces	Malawi	-13.754313 33.929313	reads ENA	Illumina MiSeq	Newbler	0	
Reyes_Malnutrition	Malnut5305	HostAssoc_Human Feces	Human feces	Malawi	-13.754313 33.929313	reads ENA	Illumina MiSeq	Newbler	0	
Reyes_Malnutrition	Malnut5306	HostAssoc_Human Feces	Human feces	Malawi	-13.754313 33.929313	reads ENA	Illumina MiSeq	Newbler	0	
Reyes_Malnutrition	Malnut5307	HostAssoc_Human Feces	Human feces	Malawi	-13.754313 33.929313	reads ENA	Illumina MiSeq	Newbler	0	

Project Name	Virome Code	Habitat Type (code)	Ecosystem Type (text)	Location	GPS	Source	Sequencing Technology	Assembly Software	# crucivirus	Reference
Reyes_Malnutrition	Malnut5308	HostAssoc_Human Feces	Human feces	Malawi	-13.754313 33.929313	reads ENA	Illumina MiSeq	Newbler	0	
Reyes_Malnutrition	Malnut5309	HostAssoc_Human Feces	Human feces	Malawi	-13.754313 33.929313	reads ENA	Illumina MiSeq	Newbler	0	
Reyes_Malnutrition	Malnut5310	HostAssoc_Human Feces	Human feces	Malawi	-13.754313 33.929313	reads ENA	Illumina MiSeq	Newbler	0	
Reyes_Malnutrition	Malnut5311	HostAssoc_Human Feces	Human feces	Malawi	-13.754313 33.929313	reads ENA	Illumina MiSeq	Newbler	0	
Reyes_Malnutrition	Malnut5312	HostAssoc_Human Feces	Human feces	Malawi	-13.754313 33.929313	reads ENA	Illumina MiSeq	Newbler	0	
Reyes_Malnutrition	Malnut5313	HostAssoc_Human Feces	Human feces	Malawi	-13.754313 33.929313	reads ENA	Illumina MiSeq	Newbler	0	
Reyes_Malnutrition	Malnut5314	HostAssoc_Human Feces	Human feces	Malawi	-13.754313 33.929313	reads ENA	Illumina MiSeq	Newbler	0	
Reyes_Malnutrition	Malnut5315	HostAssoc_Human Feces	Human feces	Malawi	-13.754313 33.929313	reads ENA	Illumina MiSeq	Newbler	0	
Reyes_Malnutrition	Malnut5316	HostAssoc_Human Feces	Human feces	Malawi	-13.754313 33.929313	reads ENA	Illumina MiSeq	Newbler	0	
Reyes_Malnutrition	Malnut5317	HostAssoc_Human Feces	Human feces	Malawi	-13.754313 33.929313	reads ENA	Illumina MiSeq	Newbler	0	
Reyes_Malnutrition	Malnut5318	HostAssoc_Human Feces	Human feces	Malawi	-13.754313 33.929313	reads ENA	Illumina MiSeq	Newbler	0	
Reyes_Malnutrition	Malnut5319	HostAssoc_Human Feces	Human feces	Malawi	-13.754313 33.929313	reads ENA	Illumina MiSeq	Newbler	0	
Reyes_Malnutrition	Malnut5320	HostAssoc_Human Feces	Human feces	Malawi	-13.754313 33.929313	reads ENA	Illumina MiSeq	Newbler	0	
Reyes_Malnutrition	Malnut5321	HostAssoc_Human Feces	Human feces	Malawi	-13.754313 33.929313	reads ENA	Illumina MiSeq	Newbler	0	
Reyes_TwinsFeces	TwinFeces	HostAssoc_Human Feces	Human feces	USA : Missouri	38.622948 -92.550521	contigs Metavir	?	Newbler	0	Reyes A. Haynes M. Hanson N. Angly FE. Heath AC. Rohwer F. Gordon JI. Viruses in the faecal microbiota of monozygotic twins and their mothers. Nature. 2010 Jul 15;466(7304):334-8. doi: 10.1038/nature09199.
Willner_CFibrosis	CF10	HostAssoc_Human Feces	Human feces	USA : California	32.754288 -117.166328	reads Metavir	454	Newbler	0	
Willner_CFibrosis	CF6	HostAssoc_Human Feces	Human feces	USA : California	32.754288 -117.166328	reads Metavir	454	Newbler	0	
Willner_CFibrosis	CF7	HostAssoc_Human Feces	Human feces	USA : California	32.754288 -117.166328	reads Metavir	454	Newbler	0	
Willner_CFibrosis	CF8	HostAssoc_Human Feces	Human feces	USA : California	32.754288 -117.166328	reads Metavir	454	Newbler	0	
Willner_CFibrosis	CF9	HostAssoc_Human Feces	Human feces	USA : California	32.754288 -117.166328	reads Metavir	454	Newbler	0	Willner D, Furlan M, Haynes M, et al. Metagenomic analysis of respiratory tract DNA viral communities in cystic fibrosis and non-cystic fibrosis individuals. PLoS One. 2009;4(10). doi:10.1371/journal.pone.0007370.
Willner_CFibrosis	Norm3	HostAssoc_Human Feces	Human feces	USA : California	32.754288 -117.166328	reads Metavir	454	Newbler	0	
Willner_CFibrosis	Norm4	HostAssoc_Human Feces	Human feces	USA : California	32.754288 -117.166328	reads Metavir	454	Newbler	0	
Willner_CFibrosis	Norm5	HostAssoc_Human Feces	Human feces	USA : California	32.754288 -117.166328	reads Metavir	454	Newbler	0	
Willner_CFibrosis	Norm6	HostAssoc_Human Feces	Human feces	USA : California	32.754288 -117.166328	reads Metavir	454	Newbler	0	
Willner_CFibrosis	Norm7	HostAssoc_Human Feces	Human feces	USA : California	32.754288 -117.166328	reads Metavir	454	Newbler	0	
Dinakaran_CardioDiseaseCircDNA	CON029	HostAssoc_Human Other		India : Madurai, Tamil Nadu	9.926890 78.132085	contigs Metavir	Ion Torrent (PGM)	?	0	
Dinakaran_CardioDiseaseCircDNA	CON030	HostAssoc_Human Other		India : Madurai, Tamil Nadu	9.926890 78.132085	contigs Metavir	Ion Torrent (PGM)	?	0	Dinakaran V. Rathinavel A. Pushpanathan M. Sivakumar R. Gunasekaran P. Rajendhran J. Elevated levels of circulating DNA in cardiovascular disease patients: metagenomic profiling of microbiome in the circulation. PLoS One. 2014 Aug 18;9(8):e105221. doi: 10.1371/journal.pone.0105221.
Dinakaran_CardioDiseaseCircDNA	CON064	HostAssoc_Human Other		India : Madurai, Tamil Nadu	9.926890 78.132085	contigs Metavir	Ion Torrent (PGM)	?	0	
Dinakaran_CardioDiseaseCircDNA	CVD008	HostAssoc_Human Other		India : Madurai, Tamil Nadu	9.926890 78.132085	contigs Metavir	Ion Torrent (PGM)	?	0	
Dinakaran_CardioDiseaseCircDNA	CVD010	HostAssoc_Human Other		India : Madurai, Tamil Nadu	9.926890 78.132085	contigs Metavir	Ion Torrent (PGM)	?	0	
Dinakaran_CardioDiseaseCircDNA	CVD014	HostAssoc_Human Other		India : Madurai, Tamil Nadu	9.926890 78.132085	contigs Metavir	Ion Torrent (PGM)	?	0	
Fancello_PeriCardialFluids	PeriCardCtrl2	HostAssoc_Human Other	human fluids	France	46.445546 2.888748	reads Metavir	454	Newbler	0	
Fancello_PeriCardialFluids	PeriCard1	HostAssoc_Human Other	human fluids	France	46.445546 2.888748	reads Metavir	454	Newbler	0	
Fancello_PeriCardialFluids	PeriCard2	HostAssoc_Human Other	human fluids	France	46.445546 2.888748	reads Metavir	454	Newbler	0	
Fancello_PeriCardialFluids	PeriCard3	HostAssoc_Human Other	human fluids	France	46.445546 2.888748	reads Metavir	454	Newbler	0	
Fancello_PeriCardialFluids	PeriCard4	HostAssoc_Human Other	human fluids	France	46.445546 2.888748	reads Metavir	454	Newbler	0	Fancello L. Monteil S. Popgeorgiev N. Rivet R. Gouriet F. Fournier PE. Raoult D. Desnues C. Viral communities associated with human pericardial fluids in idiopathic pericarditis. PLoS One. 2014 Apr 1;9(4):e93367. doi: 10.1371/journal.pone.0093367. ECollection 2014.
Fancello_PeriCardialFluids	PeriCard5	HostAssoc_Human Other	human fluids	France	46.445546 2.888748	reads Metavir	454	Newbler	0	
Fancello_PeriCardialFluids	PeriCard6	HostAssoc_Human Other	human fluids	France	46.445546 2.888748	reads Metavir	454	Newbler	0	
Fancello_PeriCardialFluids	PeriCard7	HostAssoc_Human Other	human fluids	France	46.445546 2.888748	reads Metavir	454	Newbler	0	
Fancello_PeriCardialFluids	PeriCard8	HostAssoc_Human Other	human fluids	France	46.445546 2.888748	reads Metavir	454	Newbler	0	
Fancello_PeriCardialFluids	PeriCardCtrl1	HostAssoc_Human Other	human fluids	France	46.445546 2.888748	reads Metavir	454	Newbler	0	
Fancello_PeriCardialFluids	PeriCardCtrlpos	HostAssoc_Human Other	human fluids	France	46.445546 2.888748	reads Metavir	454	Newbler	0	

Project Name	Virome Code	Habitat Type (code)	Ecosystem Type (text)	Location	GPS	Source	Sequencing Technology	Assembly Software	# crucivirus	Reference
Gibbons_restrooms	F1FH81130	HostAssoc_Human Other	human fluids	USA : San Diego	32.777655 -117.071384	reads MG-RAST		Newbler	0	Gibbons SM. Schwartz T. Fouquier J. Mitchell M. Sangwan N. Gilbert JA. Kelley ST. Ecological succession and viability of human-associated microbiota on restroom surfaces. Appl Environ Microbiol. 2015 Jan;81(2):765-73. doi: 10.1128/AEM.03117-14.
Gibbons_restrooms	F1MH21130	HostAssoc_Human Other	human fluids	USA : San Diego	32.777655 -117.071384	reads MG-RAST		Newbler	0	
Gibbons_restrooms	F3FH41205	HostAssoc_Human Other	human fluids	USA : San Diego	32.777655 -117.071384	reads MG-RAST		Newbler	0	
Gibbons_restrooms	F3FH71130	HostAssoc_Human Other	human fluids	USA : San Diego	32.777655 -117.071384	reads MG-RAST		Newbler	0	
Gibbons_restrooms	F3MH41205	HostAssoc_Human Other	human fluids	USA : San Diego	32.777655 -117.071384	reads MG-RAST		Newbler	0	
Gibbons_restrooms	F3MH61130	HostAssoc_Human Other	human fluids	USA : San Diego	32.777655 -117.071384	reads MG-RAST		Newbler	0	
Gibbons_restrooms	F3MH81130	HostAssoc_Human Other	human fluids	USA : San Diego	32.777655 -117.071384	reads MG-RAST		Newbler	0	
Pride_LongTermAntibio	ELA100ASal	HostAssoc_Human Other	Human saliva	USA : California	32.876609 -117.236885	reads MG-RAST	Ion Torrent	Newbler-large	0	
Pride_LongTermAntibio	ELA100BSal	HostAssoc_Human Other	Human saliva	USA : California	32.876609 -117.236885	reads MG-RAST	Ion Torrent	Newbler-large	0	
Pride_LongTermAntibio	ELA100CSal	HostAssoc_Human Other	Human saliva	USA : California	32.876609 -117.236885	reads MG-RAST	Ion Torrent	Newbler-large	0	
Pride_LongTermAntibio	ELA1ASal	HostAssoc_Human Other	Human saliva	USA : California	32.876609 -117.236885	reads MG-RAST	Ion Torrent	Newbler-large	0	
Pride_LongTermAntibio	ELA1BSal	HostAssoc_Human Other	Human saliva	USA : California	32.876609 -117.236885	reads MG-RAST	Ion Torrent	Newbler-large	0	
Pride_LongTermAntibio	ELA1CSal	HostAssoc_Human Other	Human saliva	USA : California	32.876609 -117.236885	reads MG-RAST	Ion Torrent	Newbler-large	0	
Pride_LongTermAntibio	ELA2BSal	HostAssoc_Human Other	Human saliva	USA : California	32.876609 -117.236885	reads MG-RAST	Ion Torrent	Newbler-large	0	
Pride_LongTermAntibio	ELA2CSal	HostAssoc_Human Other	Human saliva	USA : California	32.876609 -117.236885	reads MG-RAST	Ion Torrent	Newbler-large	0	
Pride_LongTermAntibio	ELA33ASal	HostAssoc_Human Other	Human saliva	USA : California	32.876609 -117.236885	reads MG-RAST	Ion Torrent	Newbler-large	0	
Pride_LongTermAntibio	ELA33BSal	HostAssoc_Human Other	Human saliva	USA : California	32.876609 -117.236885	reads MG-RAST	Ion Torrent	Newbler-large	0	
Pride_LongTermAntibio	ELA33CSal	HostAssoc_Human Other	Human saliva	USA : California	32.876609 -117.236885	reads MG-RAST	Ion Torrent	Newbler-large	0	
Pride_LongTermAntibio	ELA3ASal	HostAssoc_Human Other	Human saliva	USA : California	32.876609 -117.236885	reads MG-RAST	Ion Torrent	Newbler-large	0	
Pride_LongTermAntibio	ELA3BSal	HostAssoc_Human Other	Human saliva	USA : California	32.876609 -117.236885	reads MG-RAST	Ion Torrent	Newbler-large	0	
Pride_LongTermAntibio	ELA3CSal	HostAssoc_Human Other	Human saliva	USA : California	32.876609 -117.236885	reads MG-RAST	Ion Torrent	Newbler-large	0	
Pride_LongTermAntibio	ELA4ASal	HostAssoc_Human Other	Human saliva	USA : California	32.876609 -117.236885	reads MG-RAST	Ion Torrent	Newbler-large	0	
Pride_LongTermAntibio	ELA4BSal	HostAssoc_Human Other	Human saliva	USA : California	32.876609 -117.236885	reads MG-RAST	Ion Torrent	Newbler-large	0	
Pride_LongTermAntibio	ELA4CSal	HostAssoc_Human Other	Human saliva	USA : California	32.876609 -117.236885	reads MG-RAST	Ion Torrent	Newbler-large	0	
Pride_LongTermAntibio	ELA7ASal	HostAssoc_Human Other	Human saliva	USA : California	32.876609 -117.236885	reads MG-RAST	Ion Torrent	Newbler-large	0	
Pride_LongTermAntibio	ELA7BSal	HostAssoc_Human Other	Human saliva	USA : California	32.876609 -117.236885	reads MG-RAST	Ion Torrent	Newbler-large	0	
Pride_LongTermAntibio	ELA7CSal	HostAssoc_Human Other	Human saliva	USA : California	32.876609 -117.236885	reads MG-RAST	Ion Torrent	Newbler-large	0	
Pride_LongTermAntibio	ELA8ASal	HostAssoc_Human Other	Human saliva	USA : California	32.876609 -117.236885	reads MG-RAST	Ion Torrent	Newbler-large	0	
Pride_LongTermAntibio	ELA8BSal	HostAssoc_Human Other	Human saliva	USA : California	32.876609 -117.236885	reads MG-RAST	Ion Torrent	Newbler-large	0	
Pride_LongTermAntibio	ELA8CSal	HostAssoc_Human Other	Human saliva	USA : California	32.876609 -117.236885	reads MG-RAST	Ion Torrent	Newbler-large	0	
Pride_LongTermAntibio	ELA9ASal	HostAssoc_Human Other	Human saliva	USA : California	32.876609 -117.236885	reads MG-RAST	Ion Torrent	Newbler-large	0	
Pride_LongTermAntibio	ELA9BSal	HostAssoc_Human Other	Human saliva	USA : California	32.876609 -117.236885	reads MG-RAST	Ion Torrent	Newbler-large	0	
Pride_LongTermAntibio	ELA9CSal	HostAssoc_Human Other	Human saliva	USA : California	32.876609 -117.236885	reads MG-RAST	Ion Torrent	Newbler-large	0	
Rosario_RNAwhitefly	Whitefly89	HostAssoc_Insecta	Animal feces	USA : Florida	29.411571 -82.108277	reads Metavir	Solexa	IDBA_UD	0	Karyna Rosario. Heather Capobianco. Terry Fei Fan Ng. Mya Breitbart. and Jane E. Polston. RNA Viral Metagenome of Whiteflies Leads to the Discovery and Characterization of a Whitefly-Transmitted Carlavirus in North America. PLoS One. 2014; 9(1): e86748.
Rosario_RNAwhitefly	Whitefly90	HostAssoc_Insecta	Animal feces	USA : Florida	29.411571 -82.108277	reads Metavir	Solexa	IDBA_UD	0	
Temmam_ArthroVirome	midges43	HostAssoc_Insecta	Animal feces	Senegal	14.059243 -16.288288	reads Metavir	Illumina	IDBA_UD	0	Temmam S. Monteil-Bouchard S. Sambou M. Aubadie-Ladrix M. Azza S. Decloquement P. Bou Khalil JY. Baudoin J-P. Jardot P. Robert C. La Scola B. Mediannikov OY. Raoult D and Desnues C (2015) Faustovirus-Like Asfarvirus in Hematophagous Biting Midges and Their Vertebrate Hosts. Front. Microbiol. 6:1406. doi: 10.3389/fmicb.2015.01406
Temmam_ArthroVirome	imicola44	HostAssoc_Insecta	Animal feces	Senegal	14.059243 -16.288288	reads Metavir	Illumina	IDBA_UD	0	
Temmam_ArthroVirome	imicola45	HostAssoc_Insecta	Animal feces	Senegal	14.059243 -16.288288	reads Metavir	Illumina	IDBA_UD	0	
Chen_yakDiarrhea	JNVQ011	HostAssoc_MammalFeces	Animal feces	Tibet	30.152745 88.787819	contigs NCBI	no reads	SOAP De Novo	0	Chen X. Zhang B. Yue H. Wang Y. Zhou F. Zhang Q. Tang C. A novel astrovirus species in the gut of yaks with diarrhea in the Qinghai Tibetan plateau. 2013. J Gen Virol. 2015 Sep 29. doi: 10.1099/jgv.0.000303.
Sachsenroder_pigFeces	PigletC12	HostAssoc_MammalFeces	Animal feces	Germany	52.528393 13.400384	reads SRA	454	Newbler	0	
Sachsenroder_pigFeces	AllVirPig	HostAssoc_MammalFeces	Animal feces	Germany	52.528393 13.400384	reads SRA	454	Newbler	0	

Project Name	Virome Code	Habitat Type (code)	Ecosystem Type (text)	Location	GPS	Source	Sequencing Technology	Assembly Software	# crucivirus	Reference
Sachsenroder_pigFeces	PigletC54	HostAssoc_MammalFeces	Animal feces	Germany	52.528393 13.400384	reads SRA	454	Newbler	0	Sachsenröder J. Twardziok SO. Scheuch M. Johne R. The general composition of the faecal virome of pigs depends on age. but not on feeding with a probiotic bacterium. PLoS One. 2014 Feb 19;9(2):e88888. Doi: 10.1371/journal.pone.0088888. eCollection 2014.
Sachsenroder_pigFeces	PigletP12	HostAssoc_MammalFeces	Animal feces	Germany	52.528393 13.400384	reads SRA	454	Newbler	0	
Sachsenroder_pigFeces	PigletP54	HostAssoc_MammalFeces	Animal feces	Germany	52.528393 13.400384	reads SRA	454	Newbler	0	
Sachsenroder_pigFeces	SowC14pp	HostAssoc_MammalFeces	Animal feces	Germany	52.528393 13.400384	reads SRA	454	Newbler	0	
Sachsenroder_pigFeces	SowC28ap	HostAssoc_MammalFeces	Animal feces	Germany	52.528393 13.400384	reads SRA	454	Newbler	0	
Sachsenroder_pigFeces	SowP14pp	HostAssoc_MammalFeces	Animal feces	Germany	52.528393 13.400384	reads SRA	454	Newbler	0	
Shan_Pigfeces	Pig136828	HostAssoc_MammalFeces	Animal feces	USA : North Carolina	35.587952 -79.988361	reads SRA	454	IDBA_UD	0	Shan T. Li L. Simmonds P. Wang C. Moeser A. Delwart E. The fecal virome of pigs on a high-density farm. J Virol. 2011 Nov;85(22):11697-708. doi: 10.1128/JVI.05217-11.
Woo_CamelFeces	camelFeces	HostAssoc_MammalFeces	Animal feces	UAE	25.210798 55.289773	reads SRA	HiSeq 2500 paired-end	IDBA_UD	0	Woo PC1. Lau SK2. Teng JL3. Tsang AK3. Joseph M4. Wong EY3. Tang Y3. Sivakumar S4. Bai R3. Wernery R4. Wernery U5. Yuen KY2. Metagenomic analysis of viromes of dromedary camel fecal samples reveals large number and high diversity of circoviruses and picobirnaviruses. Virology. 2014 Dec;471-473:117-25. doi: 10.1016/j.virol.2014.09.020. Epub 2014 Oct 29.
Wu_batGuano	Bat2063878	HostAssoc_MammalFeces	Animal feces	China : Beijing	39.920239 116.415238	reads SRA	Illumina	IDBA_UD IGS	0	
Wu_batGuano	Bat2063879	HostAssoc_MammalFeces	Animal feces	China : Beijing	39.920239 116.415238	reads SRA	Illumina	IDBA_UD IGS	0	
Wu_batGuano	Bat2063880	HostAssoc_MammalFeces	Animal feces	China : Beijing	39.920239 116.415238	reads SRA	Illumina	IDBA_UD IGS	0	
Wu_batGuano	Bat2063881	HostAssoc_MammalFeces	Animal feces	China : Beijing	39.920239 116.415238	reads SRA	Illumina	IDBA_UD IGS	0	
Wu_batGuano	Bat2063882	HostAssoc_MammalFeces	Animal feces	China : Beijing	39.920239 116.415238	reads SRA	Illumina	IDBA_UD IGS	0	
Wu_batGuano	Bat2063883	HostAssoc_MammalFeces	Animal feces	China : Beijing	39.920239 116.415238	reads SRA	Illumina	IDBA_UD IGS	0	
Wu_batGuano	Bat2063884	HostAssoc_MammalFeces	Animal feces	China : Beijing	39.920239 116.415238	reads SRA	Illumina	IDBA_UD IGS	0	
Wu_batGuano	Bat2063885	HostAssoc_MammalFeces	Animal feces	China : Beijing	39.920239 116.415238	reads SRA	Illumina	IDBA_UD IGS	0	
Wu_batGuano	Bat2063886	HostAssoc_MammalFeces	Animal feces	China : Beijing	39.920239 116.415238	reads SRA	Illumina	IDBA_UD IGS	0	
Wu_batGuano	Bat2063887	HostAssoc_MammalFeces	Animal feces	China : Beijing	39.920239 116.415238	reads SRA	Illumina	IDBA_UD IGS	0	
Wu_batGuano	Bat2063888	HostAssoc_MammalFeces	Animal feces	China : Beijing	39.920239 116.415238	reads SRA	Illumina	IDBA_UD IGS	0	
Wu_batGuano	Bat2063891	HostAssoc_MammalFeces	Animal feces	China : Beijing	39.920239 116.415238	reads SRA	Illumina	IDBA_UD IGS	0	
Wu_batGuano	Bat2063894	HostAssoc_MammalFeces	Animal feces	China : Beijing	39.920239 116.415238	reads SRA	Illumina	IDBA_UD IGS	0	
Wu_batGuano	Bat2063898	HostAssoc_MammalFeces	Animal feces	China : Beijing	39.920239 116.415238	reads SRA	Illumina	IDBA_UD IGS	0	
Wu_batGuano	Bat2063899	HostAssoc_MammalFeces	Animal feces	China : Beijing	39.920239 116.415238	reads SRA	Illumina	IDBA_UD IGS	0	
Wu_batGuano	Bat2063900	HostAssoc_MammalFeces	Animal feces	China : Beijing	39.920239 116.415238	reads SRA	Illumina	IDBA_UD IGS	0	
Wu_batGuano	Bat2063901	HostAssoc_MammalFeces	Animal feces	China : Beijing	39.920239 116.415238	reads SRA	Illumina	IDBA_UD IGS	0	
Wu_batGuano	Bat2063902	HostAssoc_MammalFeces	Animal feces	China : Beijing	39.920239 116.415238	reads SRA	Illumina	IDBA_UD IGS	0	
Wu_batGuano	Bat2063903	HostAssoc_MammalFeces	Animal feces	China : Beijing	39.920239 116.415238	reads SRA	Illumina	IDBA_UD IGS	0	
Wu_batGuano	Bat2063904	HostAssoc_MammalFeces	Animal feces	China : Beijing	39.920239 116.415238	reads SRA	Illumina	IDBA_UD IGS	0	
Wu_batGuano	Bat2063905	HostAssoc_MammalFeces	Animal feces	China : Beijing	39.920239 116.415238	reads SRA	Illumina	IDBA_UD IGS	0	
Wu_batGuano	Bat2063906	HostAssoc_MammalFeces	Animal feces	China : Beijing	39.920239 116.415238	reads SRA	Illumina	IDBA_UD IGS	0	
Wu_batGuano	Bat2063907	HostAssoc_MammalFeces	Animal feces	China : Beijing	39.920239 116.415238	reads SRA	Illumina	IDBA_UD IGS	0	
Wu_batGuano	Bat2063908	HostAssoc_MammalFeces	Animal feces	China : Beijing	39.920239 116.415238	reads SRA	Illumina	IDBA_UD IGS	0	
Wu_batGuano	Bat2063909	HostAssoc_MammalFeces	Animal feces	China : Beijing	39.920239 116.415238	reads SRA	Illumina	IDBA_UD IGS	0	
Wu_batGuano	Bat2063910	HostAssoc_MammalFeces	Animal feces	China : Beijing	39.920239 116.415238	reads SRA	Illumina	IDBA_UD IGS	0	
Wu_batGuano	Bat2063911	HostAssoc_MammalFeces	Animal feces	China : Beijing	39.920239 116.415238	reads SRA	Illumina	IDBA_UD IGS	0	
Wu_batGuano	Bat2063912	HostAssoc_MammalFeces	Animal feces	China : Beijing	39.920239 116.415238	reads SRA	Illumina	IDBA_UD IGS	0	
Wu_batGuano	Bat2063913	HostAssoc_MammalFeces	Animal feces	China : Beijing	39.920239 116.415238	reads SRA	Illumina	IDBA_UD IGS	0	
Wu_batGuano	Bat2063914	HostAssoc_MammalFeces	Animal feces	China : Beijing	39.920239 116.415238	reads SRA	Illumina	IDBA_UD IGS	0	
Wu_batGuano	Bat2063915	HostAssoc_MammalFeces	Animal feces	China : Beijing	39.920239 116.415238	reads SRA	Illumina	IDBA_UD IGS	0	
Wu_batGuano	Bat2063916	HostAssoc_MammalFeces	Animal feces	China : Beijing	39.920239 116.415238	reads SRA	Illumina	IDBA_UD IGS	0	

Project Name	Virome Code	Habitat Type (code)	Ecosystem Type (text)	Location	GPS	Source	Sequencing Technology	Assembly Software	# crucivirus	Reference
Wu_batGuano	Bat2063917	HostAssoc_MammalFeces	Animal feces	China : Beijing	39.920239 116.415238	reads SRA	Illumina	IDBA_UD IGS	0	Wu Z. Yang L. Ren X. He G. Zhang J. Yang J. Qian Z. Dong J. Sun L. Zhu Y. Du J. Yang F. Zhang S. Jin Q. Deciphering the bat virome catalog to better understand the ecological diversity of bat viruses and the bat origin of emerging infectious diseases. ISME J. 2015 Aug 11. doi: 10.1038/ismej.2015.138.
Wu_batGuano	Bat2063918	HostAssoc_MammalFeces	Animal feces	China : Beijing	39.920239 116.415238	reads SRA	Illumina	IDBA_UD IGS	0	
Wu_batGuano	Bat2063919	HostAssoc_MammalFeces	Animal feces	China : Beijing	39.920239 116.415238	reads SRA	Illumina	IDBA_UD IGS	0	
Wu_batGuano	Bat2063920	HostAssoc_MammalFeces	Animal feces	China : Beijing	39.920239 116.415238	reads SRA	Illumina	IDBA_UD IGS	0	
Wu_batGuano	Bat2063921	HostAssoc_MammalFeces	Animal feces	China : Beijing	39.920239 116.415238	reads SRA	Illumina	IDBA_UD IGS	1	
Wu_batGuano	Bat2063922	HostAssoc_MammalFeces	Animal feces	China : Beijing	39.920239 116.415238	reads SRA	Illumina	IDBA_UD IGS	0	
Wu_batGuano	Bat2063923	HostAssoc_MammalFeces	Animal feces	China : Beijing	39.920239 116.415238	reads SRA	Illumina	IDBA_UD IGS	0	
Wu_batGuano	Bat2063924	HostAssoc_MammalFeces	Animal feces	China : Beijing	39.920239 116.415238	reads SRA	Illumina	IDBA_UD IGS	0	
Wu_batGuano	Bat2063925	HostAssoc_MammalFeces	Animal feces	China : Beijing	39.920239 116.415238	reads SRA	Illumina	IDBA_UD IGS	0	
Wu_batGuano	Bat2063926	HostAssoc_MammalFeces	Animal feces	China : Beijing	39.920239 116.415238	reads SRA	Illumina	IDBA_UD IGS	0	
Wu_batGuano	Bat2063927	HostAssoc_MammalFeces	Animal feces	China : Beijing	39.920239 116.415238	reads SRA	Illumina	IDBA_UD IGS	0	
Wu_batGuano	Bat2063928	HostAssoc_MammalFeces	Animal feces	China : Beijing	39.920239 116.415238	reads SRA	Illumina	IDBA_UD IGS	0	
Wu_batGuano	Bat2063929	HostAssoc_MammalFeces	Animal feces	China : Beijing	39.920239 116.415238	reads SRA	Illumina	IDBA_UD IGS	0	
Wu_batGuano	Bat2063931	HostAssoc_MammalFeces	Animal feces	China : Beijing	39.920239 116.415238	reads SRA	Illumina	IDBA_UD IGS	0	
Wu_batGuano	Bat2063933	HostAssoc_MammalFeces	Animal feces	China : Beijing	39.920239 116.415238	reads SRA	Illumina	IDBA_UD IGS	0	
Wu_batGuano	Bat2063934	HostAssoc_MammalFeces	Animal feces	China : Beijing	39.920239 116.415238	reads SRA	Illumina	IDBA_UD IGS	0	
Wu_batGuano	Bat2063935	HostAssoc_MammalFeces	Animal feces	China : Beijing	39.920239 116.415238	reads SRA	Illumina	IDBA_UD IGS	0	
Wu_batGuano	Bat2063936	HostAssoc_MammalFeces	Animal feces	China : Beijing	39.920239 116.415238	reads SRA	Illumina	IDBA_UD IGS	0	
Wu_batGuano	Bat2063937	HostAssoc_MammalFeces	Animal feces	China : Beijing	39.920239 116.415238	reads SRA	Illumina	IDBA_UD IGS	0	
Wu_batGuano	Bat2063938	HostAssoc_MammalFeces	Animal feces	China : Beijing	39.920239 116.415238	reads SRA	Illumina	IDBA_UD IGS	0	
Wu_batGuano	Bat2063939	HostAssoc_MammalFeces	Animal feces	China : Beijing	39.920239 116.415238	reads SRA	Illumina	IDBA_UD IGS	0	
Wu_batGuano	Bat2063940	HostAssoc_MammalFeces	Animal feces	China : Beijing	39.920239 116.415238	reads SRA	Illumina	IDBA_UD IGS	0	
Wu_batGuano	Bat2063942	HostAssoc_MammalFeces	Animal feces	China : Beijing	39.920239 116.415238	reads SRA	Illumina	IDBA_UD IGS	0	
Wu_batGuano	Bat2063943	HostAssoc_MammalFeces	Animal feces	China : Beijing	39.920239 116.415238	reads SRA	Illumina	IDBA_UD IGS	0	
Wu_batGuano	Bat2063944	HostAssoc_MammalFeces	Animal feces	China : Beijing	39.920239 116.415238	reads SRA	Illumina	IDBA_UD IGS	0	
Wu_batGuano	Bat2063945	HostAssoc_MammalFeces	Animal feces	China : Beijing	39.920239 116.415238	reads SRA	Illumina	IDBA_UD IGS	0	
Wu_batGuano	Bat2063946	HostAssoc_MammalFeces	Animal feces	China : Beijing	39.920239 116.415238	reads SRA	Illumina	IDBA_UD IGS	0	
Wu_batGuano	Bat2063948	HostAssoc_MammalFeces	Animal feces	China : Beijing	39.920239 116.415238	reads SRA	Illumina	IDBA_UD IGS	0	
Wu_batGuano	Bat2063950	HostAssoc_MammalFeces	Animal feces	China : Beijing	39.920239 116.415238	reads SRA	Illumina	IDBA_UD IGS	0	
Wu_batGuano	Bat2063951	HostAssoc_MammalFeces	Animal feces	China : Beijing	39.920239 116.415238	reads SRA	Illumina	IDBA_UD IGS	0	
Wu_batGuano	Bat2063952	HostAssoc_MammalFeces	Animal feces	China : Beijing	39.920239 116.415238	reads SRA	Illumina	IDBA_UD IGS	0	
Wu_batGuano	Bat2063954	HostAssoc_MammalFeces	Animal feces	China : Beijing	39.920239 116.415238	reads SRA	Illumina	IDBA_UD IGS	0	
Wu_batGuano	Bat2063955	HostAssoc_MammalFeces	Animal feces	China : Beijing	39.920239 116.415238	reads SRA	Illumina	IDBA_UD IGS	0	
Wu_batGuano	Bat2063956	HostAssoc_MammalFeces	Animal feces	China : Beijing	39.920239 116.415238	reads SRA	Illumina	IDBA_UD IGS	0	
Wu_batGuano	Bat2063957	HostAssoc_MammalFeces	Animal feces	China : Beijing	39.920239 116.415238	reads SRA	Illumina	IDBA_UD IGS	0	
Wu_batGuano	Bat2063958	HostAssoc_MammalFeces	Animal feces	China : Beijing	39.920239 116.415238	reads SRA	Illumina	IDBA_UD IGS	0	
Wu_batGuano	Bat2063960	HostAssoc_MammalFeces	Animal feces	China : Beijing	39.920239 116.415238	reads SRA	Illumina	IDBA_UD IGS	0	
Wu_batGuano	Bat2063961	HostAssoc_MammalFeces	Animal feces	China : Beijing	39.920239 116.415238	reads SRA	Illumina	IDBA_UD IGS	0	
Wu_batGuano	Bat2063962	HostAssoc_MammalFeces	Animal feces	China : Beijing	39.920239 116.415238	reads SRA	Illumina	IDBA_UD IGS	0	
Wu_batGuano	Bat2063967	HostAssoc_MammalFeces	Animal feces	China : Beijing	39.920239 116.415238	reads SRA	Illumina	IDBA_UD IGS	0	
Wu_batGuano	Bat546088	HostAssoc_MammalFeces	Animal feces	China : Beijing	39.920239 116.415238	reads SRA	Illumina	IDBA_UD IGS	0	
Wu_batGuano	Bat546089	HostAssoc_MammalFeces	Animal feces	China : Beijing	39.920239 116.415238	reads SRA	Illumina	IDBA_UD IGS	0	

Project Name	Virome Code	Habitat Type (code)	Ecosystem Type (text)	Location	GPS	Source	Sequencing Technology	Assembly Software	# crucivirus	Reference
Zhang_catFeces	catFeces	HostAssoc_MammalFeces	Animal feces	USA : California	37.778896 -122.446804	reads SRA		Newbler	0	Zhang W. Li L. Deng X. Kapusinszky B. Pesavento PA. Delwart E. Faecal virome of cats in an animal shelter. J Gen Virol. 2014 Nov;95(Pt 11):2553-64. doi: 10.1099/vir.0.069674-0. Epub 2014 Jul 30.
Coetzee_VineyardRNA	RNAVine	HostAssoc_Plants		South Africa : Stellenbosch	-33.935322 18.855379	contigs Metavir	no reads		0	Coetzee B1. Freeborough MJ. Maree HJ. Celton JM. Rees DJ. Burger JT. Deep sequencing analysis of viruses infecting grapevines: Virome of a vineyard. Virology. 2010 May 10;400(2):157-63. doi: 10.1016/j.virol.2010.01.023.
Hansen_CressCompletRattusFeces	cressComp	HostAssoc_Rodent Feces	Animal feces	Malaysia and Denmark		contigs NCBI			0	Hansen TA. Fridholm H. Frøslev TG. Kjartansdóttir KR. Willerslev E. Nielsen LP. Hansen AJ. New Type of Papillomavirus and Novel Circular Single Stranded DNA Virus Discovered in Urban Rattus norvegicus Using Circular DNA Enrichment and Metagenomics. PLoS One. 2015 Nov 11;10(11):e0141952. doi: 10.1371/journal.pone.0141952
Modi_MouseFeces	AmpiCtrl	HostAssoc_Rodent Feces	Animal feces	USA : Massachusetts	41.732542 -72.793500	reads SRA	454 GS FLX	Newbler	0	Modi SR. Lee HH. Spina CS. Collins JJ. Antibiotic treatment expands the resistance reservoir and ecological network of the phage metagenome. Nature. 2013 Jul 11;499(7457):219-22. doi: 10.1038/nature12212.
Modi_MouseFeces	AmpiTreat	HostAssoc_Rodent Feces	Animal feces	USA : Massachusetts	41.732542 -72.793500	reads SRA	454 GS FLX	Newbler	0	
Modi_MouseFeces	CiproCtrl	HostAssoc_Rodent Feces	Animal feces	USA : Massachusetts	41.732542 -72.793500	reads SRA	454 GS FLX	Newbler	0	
Modi_MouseFeces	CiproTreat	HostAssoc_Rodent Feces	Animal feces	USA : Massachusetts	41.732542 -72.793500	reads SRA	454 GS FLX	Newbler	0	
Reyes_Gnotobiotic Mouse	Coproseq	HostAssoc_Rodent Feces	Animal feces	USA : Missouri	38.635007 -90.262387	reads ENA	Illumina	IDBA_UD	0	
Reyes_Gnotobiotic Mouse	01HAE4ODE	HostAssoc_Rodent Feces	Animal feces	USA : Missouri	38.635007 -90.262387	reads ENA	454 GS FLX pyrosequencer (Roche)	Newbler	0	
Reyes_Gnotobiotic Mouse	08G3ZYMMR	HostAssoc_Rodent Feces	Animal feces	USA : Missouri	38.635007 -90.262387	reads ENA	454 GS FLX pyrosequencer (Roche)	Newbler	0	
Reyes_Gnotobiotic Mouse	09HAE4ODE	HostAssoc_Rodent Feces	Animal feces	USA : Missouri	38.635007 -90.262387	reads ENA	454 GS FLX pyrosequencer (Roche)	Newbler	0	
Reyes_Gnotobiotic Mouse	09HAE4ODE2	HostAssoc_Rodent Feces	Animal feces	USA : Missouri	38.635007 -90.262387	reads ENA	454 GS FLX pyrosequencer (Roche)	Newbler	0	
Reyes_Gnotobiotic Mouse	09HCZ1WD0	HostAssoc_Rodent Feces	Animal feces	USA : Missouri	38.635007 -90.262387	reads ENA	454 GS FLX pyrosequencer (Roche)	Newbler	0	
Reyes_Gnotobiotic Mouse	10GVVF7D3	HostAssoc_Rodent Feces	Animal feces	USA : Missouri	38.635007 -90.262387	reads ENA	454 GS FLX pyrosequencer (Roche)	Newbler	0	
Reyes_Gnotobiotic Mouse	10HAE4ODE	HostAssoc_Rodent Feces	Animal feces	USA : Missouri	38.635007 -90.262387	reads ENA	454 GS FLX pyrosequencer (Roche)	Newbler	0	
Reyes_Gnotobiotic Mouse	10HAE4ODE2	HostAssoc_Rodent Feces	Animal feces	USA : Missouri	38.635007 -90.262387	reads ENA	454 GS FLX pyrosequencer (Roche)	Newbler	0	
Reyes_Gnotobiotic Mouse	10HAE4ODE3	HostAssoc_Rodent Feces	Animal feces	USA : Missouri	38.635007 -90.262387	reads ENA	454 GS FLX pyrosequencer (Roche)	Newbler	0	
Reyes_Gnotobiotic Mouse	10HCZ1WD0	HostAssoc_Rodent Feces	Animal feces	USA : Missouri	38.635007 -90.262387	reads ENA	454 GS FLX pyrosequencer (Roche)	Newbler	0	
Reyes_Gnotobiotic Mouse	10HDVKH9M	HostAssoc_Rodent Feces	Animal feces	USA : Missouri	38.635007 -90.262387	reads ENA	454 GS FLX pyrosequencer (Roche)	Newbler	0	
Reyes_Gnotobiotic Mouse	12GVVF7D3	HostAssoc_Rodent Feces	Animal feces	USA : Missouri	38.635007 -90.262387	reads ENA	454 GS FLX pyrosequencer (Roche)	Newbler	0	
Reyes_Gnotobiotic Mouse	12GVVF7D32	HostAssoc_Rodent Feces	Animal feces	USA : Missouri	38.635007 -90.262387	reads ENA	454 GS FLX pyrosequencer (Roche)	Newbler	0	
Reyes_Gnotobiotic Mouse	12GVVF7D33	HostAssoc_Rodent Feces	Animal feces	USA : Missouri	38.635007 -90.262387	reads ENA	454 GS FLX pyrosequencer (Roche)	Newbler	0	
Reyes_Gnotobiotic Mouse	23GVL26ZJ	HostAssoc_Rodent Feces	Animal feces	USA : Missouri	38.635007 -90.262387	reads ENA	454 GS FLX pyrosequencer (Roche)	Newbler	0	
Reyes_Gnotobiotic Mouse	23GVRF1TC	HostAssoc_Rodent Feces	Animal feces	USA : Missouri	38.635007 -90.262387	reads ENA	454 GS FLX pyrosequencer (Roche)	Newbler	0	
Reyes_Gnotobiotic Mouse	23GVVF7D3	HostAssoc_Rodent Feces	Animal feces	USA : Missouri	38.635007 -90.262387	reads ENA	454 GS FLX pyrosequencer (Roche)	Newbler	0	
Reyes_Gnotobiotic Mouse	24HAE4ODE	HostAssoc_Rodent Feces	Animal feces	USA : Missouri	38.635007 -90.262387	reads ENA	454 GS FLX pyrosequencer (Roche)	Newbler	0	
Reyes_Gnotobiotic Mouse	24HCZ1WD0	HostAssoc_Rodent Feces	Animal feces	USA : Missouri	38.635007 -90.262387	reads ENA	454 GS FLX pyrosequencer (Roche)	Newbler	0	
Reyes_Gnotobiotic Mouse	34GVVF7D3	HostAssoc_Rodent Feces	Animal feces	USA : Missouri	38.635007 -90.262387	reads ENA	454 GS FLX pyrosequencer (Roche)	Newbler	0	

Project Name	Virome Code	Habitat Type (code)	Ecosystem Type (text)	Location	GPS	Source	Sequencing Technology	Assembly Software	# crucivirus	Reference
Reyes_Gnotobiotic Mouse	34GVVF7D32	HostAssoc_Rodent Feces	Animal feces	USA : Missouri	38.635007 -90.262387	reads ENA	454 GS FLX pyrosequencer (Roche)	Newbler	0	Reyes A. Wu M. McNulty NP. Rohwer FL. Gordon JI. Gnotobiotic mouse model of phage-bacterial host dynamics in the human gut. Proc Natl Acad Sci U S A. 2013 Dec 10;110(50):20236-41. doi: 10.1073/pnas.1319470110. Epub 2013 Nov 20.
Reyes_Gnotobiotic Mouse	34HAE4ODE	HostAssoc_Rodent Feces	Animal feces	USA : Missouri	38.635007 -90.262387	reads ENA	454 GS FLX pyrosequencer (Roche)	Newbler	0	
Reyes_Gnotobiotic Mouse	45GVL26ZJ	HostAssoc_Rodent Feces	Animal feces	USA : Missouri	38.635007 -90.262387	reads ENA	454 GS FLX pyrosequencer (Roche)	Newbler	0	
Reyes_Gnotobiotic Mouse	45GVRF1TC	HostAssoc_Rodent Feces	Animal feces	USA : Missouri	38.635007 -90.262387	reads ENA	454 GS FLX pyrosequencer (Roche)	Newbler	0	
Reyes_Gnotobiotic Mouse	45GVVF7D3	HostAssoc_Rodent Feces	Animal feces	USA : Missouri	38.635007 -90.262387	reads ENA	454 GS FLX pyrosequencer (Roche)	Newbler	0	
Reyes_Gnotobiotic Mouse	45GVVF7D32	HostAssoc_Rodent Feces	Animal feces	USA : Missouri	38.635007 -90.262387	reads ENA	454 GS FLX pyrosequencer (Roche)	Newbler	0	
Reyes_Gnotobiotic Mouse	51HAE4ODE	HostAssoc_Rodent Feces	Animal feces	USA : Missouri	38.635007 -90.262387	reads ENA	454 GS FLX pyrosequencer (Roche)	Newbler	0	
Reyes_Gnotobiotic Mouse	67G3ZYMMR	HostAssoc_Rodent Feces	Animal feces	USA : Missouri	38.635007 -90.262387	reads ENA	454 GS FLX pyrosequencer (Roche)	Newbler	0	
Reyes_Gnotobiotic Mouse	67G3ZYMMR2	HostAssoc_Rodent Feces	Animal feces	USA : Missouri	38.635007 -90.262387	reads ENA	454 GS FLX pyrosequencer (Roche)	Newbler	0	
Reyes_Gnotobiotic Mouse	78GVVF7D3	HostAssoc_Rodent Feces	Animal feces	USA : Missouri	38.635007 -90.262387	reads ENA	454 GS FLX pyrosequencer (Roche)	Newbler	0	
Reyes_Gnotobiotic Mouse	78GVVF7D32	HostAssoc_Rodent Feces	Animal feces	USA : Missouri	38.635007 -90.262387	reads ENA	454 GS FLX pyrosequencer (Roche)	Newbler	0	
Reyes_Gnotobiotic Mouse	78HAE4ODE	HostAssoc_Rodent Feces	Animal feces	USA : Missouri	38.635007 -90.262387	reads ENA	454 GS FLX pyrosequencer (Roche)	Newbler	0	
Reyes_Gnotobiotic Mouse	78HCZ1WD0	HostAssoc_Rodent Feces	Animal feces	USA : Missouri	38.635007 -90.262387	reads ENA	454 GS FLX pyrosequencer (Roche)	Newbler	0	
Reyes_Gnotobiotic Mouse	89GVVF7D3	HostAssoc_Rodent Feces	Animal feces	USA : Missouri	38.635007 -90.262387	reads ENA	454 GS FLX pyrosequencer (Roche)	Newbler	0	
Reyes_Gnotobiotic Mouse	89HAE4ODE	HostAssoc_Rodent Feces	Animal feces	USA : Missouri	38.635007 -90.262387	reads ENA	454 GS FLX pyrosequencer (Roche)	Newbler	0	
Reyes_Gnotobiotic Mouse	lumGVL26ZJ	HostAssoc_Rodent Feces	Animal feces	USA : Missouri	38.635007 -90.262387	reads ENA	454 GS FLX pyrosequencer (Roche)	Newbler	0	
Reyes_Gnotobiotic Mouse	lumGVRF1TC	HostAssoc_Rodent Feces	Animal feces	USA : Missouri	38.635007 -90.262387	reads ENA	454 GS FLX pyrosequencer (Roche)	Newbler	0	
Reyes_Gnotobiotic Mouse	M10G4PULPT	HostAssoc_Rodent Feces	Animal feces	USA : Missouri	38.635007 -90.262387	reads ENA	454 GS FLX pyrosequencer (Roche)	Newbler	0	
Reyes_Gnotobiotic Mouse	M1G3ZYMMR	HostAssoc_Rodent Feces	Animal feces	USA : Missouri	38.635007 -90.262387	reads ENA	454 GS FLX pyrosequencer (Roche)	Newbler	0	
Reyes_Gnotobiotic Mouse	M2G3ZYMMR	HostAssoc_Rodent Feces	Animal feces	USA : Missouri	38.635007 -90.262387	reads ENA	454 GS FLX pyrosequencer (Roche)	Newbler	0	
Reyes_Gnotobiotic Mouse	M3G4PULPT	HostAssoc_Rodent Feces	Animal feces	USA : Missouri	38.635007 -90.262387	reads ENA	454 GS FLX pyrosequencer (Roche)	Newbler	0	
Reyes_Gnotobiotic Mouse	M3HCZ1WD0	HostAssoc_Rodent Feces	Animal feces	USA : Missouri	38.635007 -90.262387	reads ENA	454 GS FLX pyrosequencer (Roche)	Newbler	0	
Reyes_Gnotobiotic Mouse	M4G4PULPT	HostAssoc_Rodent Feces	Animal feces	USA : Missouri	38.635007 -90.262387	reads ENA	454 GS FLX pyrosequencer (Roche)	Newbler	0	
Reyes_Gnotobiotic Mouse	M5G4PULPT	HostAssoc_Rodent Feces	Animal feces	USA : Missouri	38.635007 -90.262387	reads ENA	454 GS FLX pyrosequencer (Roche)	Newbler	0	
Reyes_Gnotobiotic Mouse	M5HAE4ODE	HostAssoc_Rodent Feces	Animal feces	USA : Missouri	38.635007 -90.262387	reads ENA	454 GS FLX pyrosequencer (Roche)	Newbler	0	
Reyes_Gnotobiotic Mouse	M5HCZ1WD0	HostAssoc_Rodent Feces	Animal feces	USA : Missouri	38.635007 -90.262387	reads ENA	454 GS FLX pyrosequencer (Roche)	Newbler	0	
Reyes_Gnotobiotic Mouse	M6G4PULPT	HostAssoc_Rodent Feces	Animal feces	USA : Missouri	38.635007 -90.262387	reads ENA	454 GS FLX pyrosequencer (Roche)	Newbler	0	
Reyes_Gnotobiotic Mouse	M7G4PULPT	HostAssoc_Rodent Feces	Animal feces	USA : Missouri	38.635007 -90.262387	reads ENA	454 GS FLX pyrosequencer (Roche)	Newbler	0	
Reyes_Gnotobiotic Mouse	M8G4PULPT	HostAssoc_Rodent Feces	Animal feces	USA : Missouri	38.635007 -90.262387	reads ENA	454 GS FLX pyrosequencer (Roche)	Newbler	0	

Project Name	Virome Code	Habitat Type (code)	Ecosystem Type (text)	Location	GPS	Source	Sequencing Technology	Assembly Software	# crucivirus	Reference
Reyes_Gnotobiotic Mouse	M9G4PULPT	HostAssoc_Rodent Feces	Animal feces	USA : Missouri	38.635007 -90.262387	reads ENA	454 GS FLX pyrosequencer (Roche)	Newbler	0	
