# Supplementary material for: Unveiling Crucivirus Diversity by Mining Metagenomic Data
Source: mBio. 2020 Sep 1;11(5):e01410-20. doi: 10.1128/mBio.01410-20 (PMC7468197; doi:10.1128/mBio.01410-20)
Supplement: TABLE S4 [file mBio.01410-20-st004.pdf]

Supplementary Table 4: Crucivirus Rep motifs

	Endonuclease domain			Helicase domain			
	Motif I	Motif II	Motif III	Walker A	Walker B	Motif C	Arg finger
CruV-081	FFTWNN	KHLQG	KANAYCCK	EMGENGKTVFCK	IFHFP	VFSN	LSQDRW
CruV-083	QPRISK			GRTGTGKSRRAW	VIDEF	ITSN	ALRRRL
CruV-084	QPRISK			GRTGTGKSRRAW	VIDEF	ITSN	ALRRRL
CruV-086	TFTINN	PHIQG	NNANYCSK				ETVKKY
CruV-087	DFTSSC	THFQG	LNMFYVLK	PKGGKGKTHVCR	LVDIP	VFTN	LSSDRW
CruV-088	IFTINN	PHIQG	QNYNYCSK	EDGNKGKSYLCK	LLDIP	AFSN	MSSDRW
CruV-089	IATIPK	QHWQI	AAKDYVWK	GPTGTGKSHNAW	IIDEF	ILSN	ALARRL
CruV-090	LLTYPG	DHTHC	NQIAYIAK	PKGGAGKTWLAR	ILDLP	VFAN	MSADRW
CruV-091	LLTYPG	DHTHC	NQIAYIAK	PKGGAGKTWLAR	ILDLP	VFAN	MSADRW
CruV-092	CFTVFD	HHWQC	DNIAYCTK	PNGNAGKTTMSK	FNFV	CFAN	MGSYKW
CruV-093	TGTWNN	PHIQF	ACMKYCSK	HEGNSGKTTFAK	MFDLT	IFAN	LSLDRW
CruV-094	DLTIPC	LHYQC	GNEFYVTK	TIGQRGKTFLTM	FIDLP	VFTN	LSIDRW
CruV-095	VFTWNN	PHIQG	KDQDYCKK	GPTATGKTSRAR	IIDDL	ITSC	QLYRRI
CruV-096	DFTAPA	IHYQG	GNDWYVTK	EKGNEGKSILCQ	TIDMP	VFTN	LSMDRW
CruV-097	CWTSYE	NHWQG	ENRIYCG	GPTGTGKSLKAR	IIDDF	ITSP	QFERRF
CruV-098	SWTSYE	KHWQG	ENRIYCGA	GPTGCGKSFKAY	LINDF	ITSS	QFQRRF
CruV-099	FFTWNN	PHIQG	AAENYCSK	PLTCQGKTQLTK	IFNFA	IFSN	LSKDRW
CruV-100	VFTAWD	VHWQG	QARDYTMK	GPSGTGKSRLAE	IVNEF	FTTP	QLLRRI
CruV-101	MLTYKT	KHTHV	NFVLYIAK	KKGCVGKTVLCK	LINLS	VFAN	LSLDRW
CruV-102	AFTLFY	KHLQG	QNIKYCSK	PTGDIGKSELCK	VIDIP	VFSN	LSKDRW
CruV-103	FLTYAQ	RHFHA	ATATYIQK		SRDEF		
CruV-104	FLTYPK	PHFHV	ACAAYLQK		TQDEF		
CruV-105	FLTYPK	PHFHV	ACAAYLQK		TQDEF		
CruV-106	RFTWNN	PHLQG	ANIAYAQK	EKGNQGKSWIAQ	IIDLT		
CruV-107					DSDDF		
CruV-108	FFTWNN	PHIQG	RAQEYCCK	PIGNNGKTFFMK	IFDLA	VFSN	MSADRW
CruV-109	FFTLNN	PHVQG	ACIKYCKK	GNAGTLKTRTAF	IIDDF	ITCE	QVNRRI
CruV-111	DFTLYD	LHYQG	DNNFYVCK	KNGNIGKSSICR	LIDMP	VFTN	LSKDMW
CruV-112	FFTFNN	PHLQG	KADEYCEK	KSGNNGKTEFCK	LFDFP	VMSN	MSIDRW
CruV-113	FITYPQ	PHIHA	NSKAYVCK	GAPDFGKTYAIE	IYDDI	VVTN	AFESRF
CruV-115	FITIKG		QSICYGTK	KIGGNGKSFFSR	FIDLP	IFSN	LSKDRW
CruV-116	FLTWH	RHFQG	ASKKYCTK	KIGGRGKSFLAK	LFDVA	VFAN	LSADRW
CruV-117	DFRANE	VHWQG	ESFFYAMK	IDGNNGKSFISA	FCDMP	VFTN	LSRDRW
CruV-119	DMRLSQ	VHFQI	KNFNYVLK	FEGNNGKSTIAS	LLDMP	VFTN	LSKDRW
CruV-120	GCTWNN	PHLQF	VASNYCKK	GPAGYGKSHEAK	IVDDF	VTSN	PIEQRF
CruV-121	LLTWPL	EHTHT	NAVKYLSK	EEGGKGKSRLAR	IFDLP	IFAN	LSADRW
CruV-122	CFTYNN	PHLQG	KAIAYCKK	GEPGSGKSQLAR	VLDDI	ITTL	QLLRRI
CruV-123	CITKHL	PHIHI	QADDYVKK	GKAGLGKSRLAH	LIEDL	VTSD	QLTRRI
CruV-124	LLTYST	EHTHV	DAKQYIAK	TVGLCQKTDLTR	IIDLP	VLAN	LSFDKW
CruV-125	CFTQNF	PHWQG	AAREYCMK	GPTGCGKSHDAF	VFDEF	ISSR	EVLRRI
CruV-126	MVTIRE	RHWQV	AAKDYVWK	GDTGTGKSRRAW	VFDEF	ITSN	ALMRRL
CruV-127	QITVFD	EHWHI	ENIDYVKK	GPSGVGKTERAK	IYDEW	ITSV	QWERRI
CruV-128	LFTWNN	AHLQG	QNKTYCSK	SAGGVGKSAMYK	TFDFA	CFAN	LTLSRW
CruV-129	CFTWNN	PHVQG	ANLAYCSK	GTTGVGKSYDAL	IIDDF	ATTC	QLYRRI

	Endonuclease domain			Helicase domain			
	Motif I	Motif II	Motif III	Walker A	Walker B	Motif C	Arg finger
CruV-130	CWTLNN	PHLQG	DAVNYCKK	GSSGTGKSHKAR	IIDDF	VTSI	QLLRRI
CruV-131	LFTLNN	PHLQA	INWLYCSK	GESGLGKTQYAC	VFDDC	FTIN	AIDSRV
CruV-132	AFTAWY	KHIQG	GNIKYCKK	GKTGAGKSYSAF	IIDEF	ITSN	QLLRRI
CruV-133	CFTLNN	PHIQG	DASNYCKK	GATSTGKSETSH	MIDEF	ITSC	QLVRRC
CruV-134	CFTINN	RHIQG	RYLYCMK				
CruV-135	VLTIWM	IHIQA	DAREYCMV	GETDLGKTRFVF	LLDEF	ITSN	ALMRRI
CruV-136	AFTLNN	PHHQG	QNYNYCSK	GKTNVGKSHLAM	VLDEL	ITSA	QLGRRI
CruV-137	CFTLNN	PHLQG	QASEYCKK	GLAGVGKSRGAF	VIDDF	ITCE	QVMRRI
CruV-138	CFTYWG	LHHQG	QNIKYCSK	GPTGCSKTHSAY	IIDDF	VTSC	QLLRRI
CruV-139	CFTSYK	THIQG	QNINYCKK	GEPGTGKTRAVW	LIDDF	FTSN	AFFRRV
CruV-140	FFTWNN	PHIQG	KAEIYCSK	EKTNMGKTQLCK	IFNIS	IFAN	MSIDRW
CruV-141	LLTYKT	LHTHV	GALKYLSK	SLGRKGKTQFGK	IINLT	LTCN	LSQDRW
CruV-142	TFVSWE	LHWQG	QNRMYCCK	GDAGSGKTEWCR	LFDDF	ITCS	QLYRRI
CruV-143	FFTYNN	KHLQG	SANMYCCK	KSGNIGKTVFTK	IFHYS	VFSN	LTLDRW
CruV-144	VFTFNN	PHYQG	KAAAYCMK	GPTGTGKTHRAR	VIDEY	ITCP	EITRRL
CruV-145	CFTINN	PHIQG	QNIDYCSK	GPTGTGKSALAG	VIDDF	VTSP	QLLRRI
CruV-146	CFTVNN	PHLQG	HNLAYCTK	GPSGAGKSYWCH	YLDDL	VTSQ	ALLRRF
CruV-147	CFTVNN	PHLQG	HNLAYCTK	GPSGAGKSYWCH	YLDDL	VTSQ	ALLRRF
CruV-148	CFTVNN	PHLQG	HNLAYCTK	GPSGAGKSYWCH	YLDDL	VTSQ	ALLRRF
CruV-149	CYTYWG	RHIQG	QAATYCMK	GGPGTGKSAAAD	IFDDF	FTAN	PFMRRV
CruV-150	SLTINN	EHYQL	ALEKYVKK				
CruV-151	CFTINH	EHLQC	QASDYCKK	GESGVGKSKSVR	LIDDL	ITSQ	AITRRF
CruV-152	CFTVNN	PHLQG	HNLAYCTK	GPSGAGKSYWCH	YLDDL	VTSQ	ALLRRF
CruV-153	TFTWNN	PHLQG	ANRLYCMK	TVGGMGKSWMTD	VFDFS	CFAN	MSQDRW
CruV-154	CFTLNN	PHLQG	SAAAYCKK	GPAGAGKTAGPV	IIDDF	ITCD	QVIRRI
CruV-156	CYTYWG	RHIQG	QAADYCMK	GGPGTGKSAAAA	IFDDF	FTAN	PFMRRV
CruV-157	DFRHTA	VHFQG	DDFFYTMK	DSGNNGKSTIAS	FVDMP	VFSN	LSRDRW
CruV-158	VFTLNN	PHLQG	SNKTYCSK	EKGSKGKTFLAK	IYDVP	IFTN	FSVDRL
CruV-159	CFTLNN	PHLQG	QASNYCKK	GATGVGKSRGAR	IIDDF	ITCE	QVLRRI
CruV-160	EFRLSE	RHFQG	NYDCYASK	NIGNNGKSTIAH	FVDLP	VFTN	VSRDRW
CruV-161	DFTYHS	LHFQG	TSFDYVMK	PKGNLGKTIFRK	ILDFA	IFTN	MSDDRW
CruV-162	CWTLNN	PHLQG	LAIRYCVK	ENGNTGKSELTK	IIDIP	VFSN	LSLDRW
CruV-163	DFTAHG	IHLQG	KNSYYVSK	KSGNIGKSTLCT	LFDLP	IFTN	LSADRW
CruV-164	CWTLNN	PHLQG	LAIRYCVK	ENGNTGKSELTK	IIDIP	VFSN	LSLDRW
CruV-165	DFRANQ	LHWQG	DLFFYVMK	PTGGVGKSTIAA	FLDMP	VFTN	LSRDRW
CruV-166	CFTINN	PHIQG	RAAAYCQ				
CruV-167	CYTYWG	RHIQG	QAADYCMK	GGPGTGKSAAAD	IFDDF	FTAN	PFMRRV
CruV-168	TFTLYD	LHYQG	HNYNYCTK	KESETGKTIFKQ		VTTN	LLPKRI
CruV-169	DFRANQ	LHWQG	DLFFYVMK	PTGGVGKSTIAA	FLDMP	VFTN	LSRDRW
CruV-170		VHFHV		GPPDIGKTHWIN	VYDDV	IFLN	LFVARF
CruV-171	VFTWNN	PHLQG	ANLKYCSK	KDGNTGKSDMLK	VFDYS	CFSN	LSQDRW
CruV-172	DFTLFD	IHYQG	DNDFYVLK	ENGNVGKTTLVR	FVDMP	IFTN	LSKDMW
CruV-173		PHYHI	DNIHYIKK	GPSGVGKTTKAM	LYDEW	ITSV	QWERRI
CruV-174	VIVTNN	PHLQG	SQRAYCTK	GEAGVGKSVKAR			
CruV-175	VIVTNN	PHLQG	SQRAYCTK	GEAGVGKSVKAR			
CruV-176	VFTINN	KHLQG	QNVKYCTK	KNGNSGKSKIVK	LLDIS	IFSN	MSLDRW

	Endonuclease domain			Helicase domain			
	Motif I	Motif II	Motif III	Walker A	Walker B	Motif C	Arg finger
CruV-177	DFTYVK	EHYQG	KNFNYVLK	INGNNGKSYLSE	IMDMP	LFCN	LSKDRM
CruV-178	DMTWHQ	LHYQC	GNLNYVTK	SKGVVGKSTLIN	LIDIP	VFTN	LSNGRW
CruV-179	CFTAYH	PHLQG	EAIDYCKK	GGPGSGKSHHCK	ILDEF	ITSN	PLIRRI
CruV-180	CFTAYH	PHLQG	EAIDYCKK	GGPGSGKSHHCK	ILDEF	ITSN	PLIRRI
CruV-181	DFTLRR	EHFQG	SNFNYVLK	PAGNVGKSTLSG	FLDLP	VFTN	LSYDRW
CruV-182	VFTSFN	KHLQG	QAIDYCKK	GDTQTGKTKYVY	LLDDF	ITSN	ALKRRI
CruV-183	RFTINN	FHFQG	SNVVYASK	GPSGTGKTHRAT	IIDDL	ITAP	QLLRRI
CruV-184	CFTMFF	LHYQG	QNVKYCSK	GPTGVGKSKLAF	IFDDF	ITSY	QLGRRI
CruV-185	QLTVFD	EHWHI	DNIHYIKK	GPSGIGKTQRAK	VYDEW	ITSV	QWQRRI
CruV-186	CFTAWD	FHWQG	QASEYCKK	HNSGTGKSTLID	TFNIP	VFAN	LMPKRC
CruV-187	CFTIHD	LHVQG	QNKEYCSK	GPPRTGKSLLVR	VFDEF	FTSN	IRGPRF
CruV-188	DFRISK	IHYQG	RTFNYQLK	PVGNNGKSTIKD	LVDIP	IFTN	LSPDRM
CruV-189	MIRISR	DHIQG	ALSKYSMK	KIGNTGKSKFCK	LFNLT	VFAN	MSLDRW
CruV-190	VFTYNN	KHLQG	QAITYCKE	DNGRSGKSELVA	LFDLP	IFAN	LTMDKW
CruV-191	TWTIFD	IHLQG	QNKVYCSK	GPPNIGKSGLAR	VFGEF	FLSN	IRGPRF
CruV-192	VFVLPN	KHLQG	ANIAYCKK	PEGNQGKSWMCN	VFDFS	CFSN	MSQDRW
CruV-193	CFTNFA	LHHQG	QNVDYCSK	LTGNLGKSFFST	IFDFE	AMSN	FSIDRW
CruV-194	VFTSWC	KHWQG	QASSYCMK	LKSATGKSTMRD	IFDLP	VFAN	EMPERC
CruV-195	VFTLNN	PHLQG	QAVDYCKK	GPTGTGKSRTAR	LIEDV	ITSN	PILRRF
CruV-196	CFTWNN	PHLQG	SNMTYCTK	KEGKTGKTEFAK	IYPLS	IFAN	MSKDRW
CruV-197	CFTNFA	LHHQG	QNVEYCSK	ITGNLGKSFFSD	VFDFE	CLSN	FSLDRW
CruV-198	PWTLNN	PHLQG	QNIDYIVL	GPSGIGKTYAAE	FLDEF	ITTP	QLSRRI
CruV-199	FITYFA	IHFHC	QAIAYILK				
CruV-200	CITINN	LHIQG	QAIDYCKK	GESGCSKSHTAK	IIDEL	ITTS	QLIRRI
CruV-201	LLTYKT	EHTHV	DTFFYLTK	DVGGSGKTNLSC	MIDIP	ILAN	IKWDRW
CruV-202	CFTLNN	PHLQG	ERYLYCMK				
CruV-203	VFTFNN	PHLQG	QCRTYCSK	GPTGSGKSRGVR	LLDDV	VTSQ	ALERRF
CruV-204	CFTFNN	PHLQG	QNTAYCSK	GETGTGKSHCVE	YLEDF	VTSN	PLERRF
CruV-205	CFTFNN	PHLQG	QNTAYCSK	GETGTGKSHCVE	YLEDF	VTSN	PLERRF
CruV-206	VFTWNN	PHLQG	ASLKYCSK	KEGNTGKSDMLK	VFDYS	CFSN	LSQDRW
CruV-207	CFTFNN	KHLQG	QAIAYCKK	GEPEAGKSFYAR	ILDDI	ITTL	QFTRRI
CruV-208	LGTIPR	EHWQI	AAEDYVWK	GKTGTGKSRRAW	IIDEF	ITSN	ALMRRL
CruV-209	MFTYAT	LHTHA	QKVLYLCK	IKGCCGKTIFAQ	LEDVG	VLAN	LTWDKW
CruV-210	LLTYRT	LHTHV	DASKYIAK	GNGNDGKSLGLC	IIDLP	VMAN	LSLDRW
CruV-211	CYTINN	PHLQG	EASDYCKK	GKAGVGKTKLAH	IIDDY	ITCE	QITRRI
CruV-212	FFTFNN	THLQG	ASINYCSK	GNGNDGKSLGLC	ILDLP	IFTN	LTLDRW
CruV-213	TFTLNN	KHVQG	QNYEYCSK	GKSGSGKSREAR	IIDDV	VTSQ	ALKRRF
CruV-214	CCTDYV	EHYQV	QNKRYCSK	GPPGTCKSRTAI	LFDDF	FTSN	AWIRRL
CruV-215	CFTHNN	PHLQG	SNKTYCSK	PEGNVGKSDMAK	VIDLA	CFAN	LSADRW
CruV-216	DLSYNK	EHYQI	KNFNYVMK	KNGNNGKSYLSE	IMDMP	VFCN	LSKDRM
CruV-217	CFTLNN	PHHQG	DNYDYCSK	GPTGVGKTHLAH	IFDDI	VTTC	QLGRRI
CruV-218	CFTINN	PHIQG	QNRTYCSK	GGARLGKSKLAH	LWDEF	ITSI	QIMGRI
CruV-219	VFTSWC	KHWQG	QAAQYCKK	QKSGTGKSTMRD	VFDLP	VFAN	DMPMRC
CruV-220	CFTINN	PHIQG	QASDYCKE	GPTGTGKTRTAM	IIDDF	ITCP	QLTRRI
CruV-221	DFRCNE	IHWQG	DLFFYCMK	PTGGKGKSTIAA	FFDMP	VFSN	LSRDRW
CruV-222	IFVYNN	PHLQG	EAAAYCKK	GETGVGKTRGAI	IIDDF	ITCD	QLTRRI

	Endonuclease domain			Helicase domain			
	Motif I	Motif II	Motif III	Walker A	Walker B	Motif C	Arg finger
CruV-223	FLTSYA	PHSHT	QAIEYVVK	ALRHMGMIERAQ			
CruV-224	CWTLNN	PHLQG	QARDYCLK	TKGGRGKSYLAK	VFDYS	CFSN	
CruV-225	DVTIWE	RHFQI	RNFNYVLK	PTGNYGKGFAAD	ILDMP	VFTN	YSKDRF
CruV-226	DFTLFD	IHFQG	TNDFYVTK	PEGCKGKTALTR	MIDMP	VFTN	MSKDMW
CruV-227	CFTFNN	RHLQG	QNDDYINK	GNTGTGKSHAVE	YLEDI	VTSN	PLQRRF
CruV-228	ILTINN	VHIQA	AAAAYCIK	GDGGVGKTKYVI	CIDDF	ITCE	QVMRRI
CruV-229	VFTINN	LHIQG	DSINYTKK	GKANTGKTYKAR	VMEDF	FSTN	CENGRI
CruV-230	VFTWNN	PHHQG	QNFNYSNK	GPSGAGKTRWCY	IVDDF	FTSC	QLLRRI
CruV-231	CFTMFW	EHYQG	QNVKYCAK	GPTGVFKTRYAS	IFDDF	ITSC	QLGRRI
CruV-232	IPTIWI	LHAHL				ICFS	
CruV-233	VFTWNN	PHLQG	ASLKYCSK	KRGNTGKTAMFK	IFDLA	CFSN	LSIDRW
CruV-234	CYTINN	PHLQG	EASDYCKK	GKAGVGKTKLAH	IIDDY	ITCE	QITRRI
CruV-235	CFTMYW	EHYQG	QNVHYCAK	GPTGTGKSKMAF	IFDDF	ITSC	QLGRRI
CruV-236	CFTYNN	PHLQG	ENIKYCSK	LVGNKGKTAMTK	IFDFS	IFAN	LSKDRW
CruV-237	CFTHHI	PHYQG	EASEYCKE	GVMDAGKTTIIR	FMDEV	VASN	AIKKRF
CruV-238	DFTVWP	LHFQG	KNFFYVMK	GVGNNGKTICCQ	IFDFP	LFMN	LSADRW
CruV-239	DFTVWP	LHFQG	KNFFYVMK	GVGNNGKTICCQ	IFDFP	LFMN	LSADRW
CruV-240	DFTLDV	LHYQG	NSFIYVMK	AQPNLGKTHATQ	FIDMP	VFTN	LGDDRW
CruV-241	DFTYFG	LHYQG	DNNFYVCK	KLGNKGKSYLSF	IFDMP	IFTN	LSEDRW
CruV-242	AITISR	PHFQG	ALKKYCMK	QEGNQGKSEFVK	LVDLT	VMAN	LSNDRW
CruV-243	LYTWFN	PHVQG	NNFEYCSK	GPSNTGKTHYAA	IFDDM	FTHN	AIERRF
CruV-244	CFTSFN	EHFQG	QARVYCMK	DAGNNGKTFLSK	ICDYA	VMAN	LSVDRI
CruV-245	LYTWFN	PHVQG	NNFEYCSK	GPSNTGKTHYAA	IFDDM	FTHN	AIERRF
CruV-246	FFTYKT	EHTHA	GSIAYCIK	KAGGKGKSTFLD	INLPR	IFAN	MSLRKW
CruV-247	CFTINN	HHFQC	SNIVYCTK	GPTGTGKTYHVM	LIDDL	VTSN	TLWRRF
CruV-248	CFTYNN	PHLQG	ENIKYCSK	LVGNKGKTAMTK	IFDFS	IFAN	LSKDRW
CruV-249	VFTHHC	RHFQG	GSLDDCLN	GDGDLGKSDLCK	IFDFP	CLAN	YTSNRW
CruV-250	CFTLNN	PHVQG	QNYEYCSK	GESGAGKSHKSR	ILEDV	VTSQ	ALHRRF
CruV-251	CFTLNN	PHVQG	QNYEYCSK	GESGAGKSHKSR	ILEDV	VTSQ	ALHRRF
CruV-252	CFTMFW	EHYQG	QNVRYCAK	GPTGTGKSKIAF	IFDDF	ITSC	QLGRRL
CruV-253	CFTLNN	PHLQG	QASEYCKK	GLAGVGKTRHIY	IIDDF	ITCE	QIMRRI
CruV-254	CFTMYW	EHYQG	QNVRYCAK	GPTGTGKSKLAF	IFDDF	ITSC	QLGRRL
CruV-255	FLTINN	KHLHC	DCIVYCQK	GASGTGKTRCIF	LWDEI	ITSS	EIMRRL
CruV-256	CFTAYP	QHHQG	DAAQYCWK	GKAGTGKTRLAL	IIDDF	ITSC	QLLRRI
CruV-257	VYTYFD	LHLQG	QASTYCKE	GLAGTGKSRTAK	VIDDF	ITCE	QIMRRL
CruV-258	CFTVNN	QHLQC	QAADYCKK	GDSGVGKSMSVR	LIDDL	ITSQ	AISRRF
CruV-259	TLTIWD	QHFQA	HNNIYTSK	GKSGCGKTHQAQ	IFDEF	FTSN	AFQRRI
CruV-260	FYTFNN	PHLQG	QAANYCLK	GTTGTHKSYLSR	ILEDF	INTP	QLLRRI
CruV-261	WLTYKT	EHTHC	RIMRYMAK	ETGNVGKSWMAL	IFDFP	VLAN	MSLDRW
CruV-262	CFTMFW	EHYQG	QNVKYCSK	GPAGVGKTQSAL	IIDDF	ITCD	QIGRRI
CruV-263	CFVINN	PHIQG	QNRAYCSK	GDTGAGKSRLGH	LWDEF	ITSI	QILRRI
CruV-264	LYTWFN	PHVQG	NNFEYCSK	GPSNTGKTHYAA	IFDDM	FTHN	AIERRF
CruV-265	CFTLNN	PHVQG	QNYEYCSK	GESGVGKSHKAR	ILEDV	VTSQ	ALGRRF
CruV-266	CFTLNN	PHVQG	QNYEYCSK	GESGVGKSHKAR	ILEDV	VTSQ	ALGRRF
CruV-267	CFTIFG	PHLQG	NNVIYCSK	AQGDTGKSFFVK	CIDYA	CFAN	LSMDRW
CruV-268	VFTAWD	GHWQG	QAREYALK	GPTSTGKTRLVM	LVDDF	ITSP	QLLRRI

	Endonuclease domain			Helicase domain			
	**Motif I**	**Motif II**	**Motif III**	**Walker A**	**Walker B**	**Motif C**	**Arg finger**
**CruV-269**	IFVKHY	EHLQG	QASDYCRG	VVGSAGKSELAK	AYDLP	VFAN	MSADRW
**CruV-270**	FFTYKT	EHTHA	GSIAYCIK	KAGGKGKSTFLD	INLPR	IFAN	MSLRKW
**CruV-271**	EIEINN	PHIHC	RYVDYCKG				VLHREI
**CruV-272**	CFTDFV	PHIQG	QAADYCKE	GEPGCGKSHRAV	VLDDF	ITTN	QWLRRV
**CruV-273**	TFTLFY	PHLQG	ENNTYCSK	PEGGVGKSDMFK	VFDLA	CFAN	LSRDRW
**CruV-274**	DLTGWT	LHFQC	KNFDYVMK	KIGGIGKTTLTD	IVDLP	VFTN	ISKFRW
**CruV-275**	LLTYGS	LHTHI	NSLKYLAK	SIGGSGKSWLID	MNLT	VMAN	LTTDRW
**CruV-276**	LYTWFN	PHVQG	NNFEYCSK	GPSNTGKTHYAA	IFDDM	FTHN	AIERRF
**CruV-277**	CFTDFV	PHIQG	QAADYCKE	GEPGCGKSHRAV	VLDDF	ITTN	QWLRRV
**CruV-278**	LYTWFN	PHVQG	NNFEYCSK	GPSNTGKTHYAA	IFDDM	FTHN	AIERRF
**CruV-279**		EHMQC	QASDYCKK	GPPGVGKSHIAR		VTSN	AIVRRF
**CruV-280**	AFTWNN	PHLQG	ANLKYCSK	KRGNTGKTAMFK	IFDLA	CFSN	LSIDRW
**CruV-281**	CFTDFV	PHIQG	QAADYCKE	GEPGCGKSHRAV	VLDDF	ITTN	QWLRRV
**CruV-282**	CFTDFV	PHIQG	QAADYCKE	GEPGCGKSHRAV	VLDDF	ITTN	QWLRRV
**CruV-283**	EITIQP	LHFQG	DNNFYVMK	QKGGIGKSKLVE	LIDYP	IFTN	LTKNRW
**CruV-284**	DFRISQ	LHFQG	QNFNYVLK	PVGNNGKSTVAS	LIDMP	VFTN	LSADRW
**CruV-285**	VFTNFK	LHHQG	SNNKYTSK	GSAGRGKSHIAK	WFDEF	VTSP	QMLRRI
**CruV-286**	DLTIYE	RHFQI	KNFNYVMK	PDGNRGKGFIAD	ILDMP	LFTN	YSKDRY
**CruV-287**	DFTYFI	WHLQG	AFYRYPMK	SHGNTGKTTFKS	VIDIP	VMSN	LSIDRW
**CruV-288**	VFTAWQ	LHWQG	EAAAYCEK	EASATGKSTMYD	FNVPR	VFAN	DMPDRC
**CruV-289**	CFTLNN	PHLQG	QASEYCKK	GATGVGKTRTAR	IIDDF	ITCE	QVIRRI
**CruV-290**	RWTHNN	PHLQG	ANIVYVRK	PLGKCGKTVFAA	IIDLM	VLSN	MAQERF
**CruV-291**	CFTDFV	PHIQG	QAADYCKE	GEPGCGKSHRAV	VLDDF	ITTN	QWLRRV
**CruV-292**	CFTLNN	PHLQG	QASMYCKK	GATGVGKSRTAR	IIDDF	ITCE	QVLRRI
**CruV-293**	MFTYYI	PHLQF	QNYKYCSK	GPPGVGKSRRAR	LIDDL	VTSN	SFDRML
**CruV-294**	CFTINN	PHMQC	HNQRYCKK	GTTGSAKTREYT	LSRLR	ITSC	QLHRRY
**CruV-295**	CFTMFW	DHYQG	DNVKYCSK	GPTGTGKSKFAF	IFDDF	ITSC	QLGRRI
**CruV-297**	CFTINN	PHIQG	QASDYCKK	GPTGTGKTRTAI	LIDDF	ITCP	QVTRRI
**CruV-298**	CWTLNN	PHLQG	QAAEYCKK	GPTGTGKSRRAH	IIDDF	VTSN	ALRRRI
**CruV-299**	VFTNFK	PHHQG	SNEKYTQK	GESGRGKTYIAK	WFDEF	FTSP	QLKRRI
**CruV-300**	DATIGE	RHYQC	DNHFYVCK	PDGGIGKSTFCT	LMDMP	IFTN	LTRNRW
**CruV-301**	CFTVNN	PHLQG	QNRVYCMK	GPTGIGKSRYCF	ILEDV	ITSN	PLLRRF
**CruV-302**	KFTWNN	PHIQG	NHVRYIQG	LSSTTGKTTFMQ	HIDIP	VTSN	KLPNRF
**CruV-303**	CFTLNN	PHLQG	EAAMYCKK	GLAGVGKTRKPI	IVDDF	ITCE	QVLRRI
**CruV-304**	LFTYYI	PHLQF	QNYKYCSK	GPPGVGKSRRAR	LIDDL	VTSN	SILRRF
**CruV-305**	CFTVNN	PHLQG	QNRVYCMK	GPTGIGKSRYCF	ILEDV	ITSN	PLLRRF
**CruV-306**	CFTHNN	PHLQG	ANKTYCSK	PEGNVGKSDMAK	VIDLA	CFAN	LSADRW
**CruV-307**	LTTYWP	LHGHM	QAKDYVTK				
**CruV-308**	CYTSRE	PHIHV	KWTNYVLK				
**CruV-309**	CFTVNN	PHLQG	QNRVYCMK	GPTGIGKSRYCF	ILEDV	ITSN	PLLRRF
**CruV-310**	CFTVNN	PHLQG	QNRVYCMK	GPTGIGKSRYCF	ILEDV	ITSN	PLLRRF
**CruV-311**	CFTVNN	PHLQG	QNRVYCMK	GPTGIGKSRYCF	ILEDV	ITSN	PLLRRF
**CruV-312**	DFTSYF	LHLQG	TDDAYVMK	KKGNIGKTTLVG	FIDLP	VFTN	MSSDRW
**CruV-313**	TWTLNN	PHLQG	QNRNYCGK	GKTGQAKTRLAG	IIDEV	YTSP	QLHRRI
**CruV-314**	VFTWNN	LHHQG	QNFNYSNK	GPAGIGKTRWCA	IVDDF	FTSC	QLLRRI
**CruV-315**	IFTWNN	PHHQG	QNFNYSNK	GPAGIGKTRWCA	IVDDF	FTSC	QLLRRI

	Endonuclease domain			Helicase domain			
	Motif I	Motif II	Motif III	Walker A	Walker B	Motif C	Arg finger
CruV-316	CFTINN	PHMQC	QNQIYCKK	GTTGSAKTREFT	MNEVR	ITSC	QFYRRY
CruV-317	FITYPK	HLHSI	YAIDYCDK				
CruV-318	CFTAFD	PHYQG	SNEAYTQK	GGAGKGKSTVAR	WFDEF	ITSS	QLLRRI
CruV-319	VFTYNN	PHLQG	ENEDYCSK	GDTGTGKSHAVE	YLEDF	VTSN	PLMRRF
CruV-320	VFTYNN	PHLQG	ENEDYCSK	GDTGTGKSHAVE	YLEDF	VTSN	PLMRRF
CruV-321	VFTINN	PHLQG	NNAIYCSK	KDGNAGKSDMCK	CMDYA	CFSN	LSTDRW
CruV-322	CFTLNN	PHLQG	DNIDYCSK	GVTGVGKSTKYR	ILEDI	VTSQ	ALKRRF
CruV-323	CFTLNN	PHLQG	DNIDYCSK	GVTGVGKSTKYR	ILEDI	VTSQ	ALKRRF
CruV-324	CFTLNN	PHLQG	DNIDYCSK	GVTGVGKSTKYR	ILEDI	VTSQ	ALKRRF
CruV-325	MIRISK	HHIQG	ALEKYSMK	QIGNTGKSKFCK	FNLSR	VMAN	LSSDRW
CruV-326	IFTWNN	PHLQG	TNTSYCSK	GQSGSGKSQFAE	VIEEF	ITSV	QIVRRM
CruV-327	LLTYKT	EHTHT	DMKRYISK	PMGNSGKSTFAK	IFDFP	VFAN	LSFDRW
CruV-328	CFTLNN	PHLQG	QASNYCKK	GPTGSGKTRHAL	VIDDF	ITSC	QLLRRI
CruV-329	DLTISL	DHIQA	SGDFYCCK	EKGNIGKSILVA	MFDMP	IFTN	LSRDRW
CruV-330	FLTYWP	IHAHM	QAIDYCTK				
CruV-331	CFTLNN	PHLQG	SNIAYCTK	GPTGTGKSRAAN	IIDDY	ITTP	QLMRRI
CruV-332	CFTYYS	LHVQG	EAAAYCEK	GPSRCGKSVLAR	FSEFA	FTSN	PWLKRI
CruV-333	VFTINN	PHLQG	NNAIYCSK	HKGNAGKSDMCK	CFDYA	CFAN	LSADRW
CruV-334	CFTINN	FHLQG	QAREYCMK	GEPGCGKTRLAK	LFDDF	ITSN	ALERRI
CruV-335	MLTYKT	EHTHV	KCINYLCK	GGSGIGKTQLAL	VFDDI	FTSN	AIKRRF
CruV-336	CFTIFD	PHLQG	EASEYCKK	GPTGTGKTSMSM	IIDEY	FTSN	PIARRV
CruV-337	VLTVSQ	WHYQG	NLKEYCIK	FDGGKGKSSLCK	FNLSR	VFTN	FSPDRW
CruV-338	DITAPC	LHWQC	GDEFYVIK	PTGQVGKSTLAL	FIDLP	VFTN	LSRDRW
CruV-339	TFTWNN	PHLQG	DSIRYCSK	KDGCAGKSSLID	IVDLS	VMAN	FSLDRW
CruV-340	CITLNN	PHLQG	QNKTYCSK	HWGPTGKFLQEC		IMSN	
CruV-341	CFTEFD	KHMQG	ENQKYCTK	GKSGSGKTKYVY	LLDDF	ITSN	AVERRI
CruV-342	LLTYKT	EHTHT	DMKRYISK	PMGNSGKSTFAK	IFDFP	VFAN	LSFDRW
CruV-343	CFTLNN	PHLQC	QASEYCKK	GLAGVGKSRGAY	VIDDF	ITCE	QVMRRL
CruV-344	CFTVNL	HHYQG	QAIDYVSK	GVSDSGKTRKVY	LIDDY	YTSN	AFKRRI
CruV-345	CYTWNN	PHIQG	QSLTYCSK	GKTAVGKSHLAF		ITSS	QLLRRL
CruV-346	CFTLNN	PHLQG	SNIKYCSK	GKSRAAREHNGY	ILDDF	IVTSN	
CruV-347	CFTLNN	PHLQG	QNIDYCIK	GSTGTGKSMKAR	IIDDF	VTSN	PLLRRF
CruV-348	AFTHHT	LHWQT	DNARYCKK	GKGGTGKSRLAR	MIEEF	FTSN	QTERRF
CruV-349	IITSYL	MHHQG	QGAEYCGY	GESGTGKTELIY	LIDDI	LTSN	AIIRRI
CruV-350	SWTLFN	KHLQG	ENRAYCTK	GDTGTGKTLFAR	ILDDI	VTGP	QLLRRC
CruV-351	MLTYPD	EHSHV	NSVRYLGK	AIGGKGKSCFAD	IFDWP	IFAN	LSLDRW
CruV-352	CFTINK	LHIQG	QARDYCMG	GKSGTGKSKLAW	IIDDY	FTTN	TFERRI
CruV-353	IITSYL	MHHQG	QGAEYCGY	GESGTGKTELIY	LIDDI	LTSN	AIMRRI
CruV-354	VFTNWK	LHHQG	SNDKYTSK	GKSGRGKTHIAK	WLDEF	VTSS	QLRRRI
CruV-355	AVTINN	RHLQC	QAAEYCKK	GPTRIGKSTRAR	LIEDI	VTSQ	AIVARC
CruV-356	CFTINQ	LHLQG	QARDYCMK	GPPGTGKTTLIA	IFDDF	FTSN	AIRRRC
CruV-357	AFTWFN	KHLQG	ANIAYCSK	QIGNVGKTHMAM	VFDFT	IFAN	MSLDRW
CruV-358	SFNFFI	EHYQC	NFDYVMK	VGGVHMKSSFEE	IIDIE	VFAN	LTGGRF
CruV-359	QFTLNN	PHLQG	QNITYCKK	NLGNIGKSDTVM	IFDLP	IYAN	LSLDRW
CruV-360	LFVLNN	PHLQG	VNIKYCQK	GKTSTGKSHYIH	FIDDF	ITSP	QLLRRV
CruV-361	VGTLNN	PHLQF	QNKDYCSK	GAAGTGKSMTAR	LIDDF	VTSQ	AIKRRF

	Endonuclease domain			Helicase domain			
	Motif I	Motif II	Motif III	Walker A	Walker B	Motif C	Arg finger
CruV-362	DFTLDM	RHWQG	NVFTYIEK	NKGNNGKTWFAG	IIDIP	VFVN	LSADRM
CruV-363	CFTNFD	PHHQG	DNVKYCSE	YDGQLGKSYFQK	VNLTR	IFAN	LSIHRW
CruV-364	CFTDFV	PHIQG	QAADYCKE	GEPGCGKSHRAV	VLDDF	ITTN	QWLRRV
CruV-365	CFTLNN	PHLQG	QNDAYCKK	GLSGCGKSRYAR	IIEDL	ITSQ	ALTRRC
CruV-366	EIRANE	RHYQG	GKAFYSMK	FAGCDGKSTCAS	FFDMP	VFTN	MSKDRW
CruV-367	VFTLNN	PHLQG	KAIDYCKK	GNTGLGKTRFVH	LFDDY	ITSN	ALERRI
CruV-368			QARDYTMK	GPTGINKSRNAY	IIDDY	LTSH	ELARRV
CruV-369	CFTDFV	KHLQG	QNIEYCSK	GESGAGKSYPIN	LFDDF	FTAN	AWNRRI
CruV-370					VFDEF	ILSN	AFLSRV
CruV-371	SSTTIP	PHAMI	SISRYAEV				CVARRV
CruV-372	CYTLNS	LHIQG	QAADYCRD	GESGTGKSQAAY	LFDEF	FTSN	PLTRRI
CruV-373	LVTCNV	KHLQG	EMMMYCKK	GPAGSGKSHYAR	LLDDF	VTSQ	ALNRRF
CruV-374	VFTLHN	PHLQG	HSIIYCKK	GESGTGKSRHAR	IIEDI	ITSN	AIERRY
CruV-375	FLTYKT	KHSHM	KALNYIGK	PPGQGGKTSLLR	VNLTR	VFAN	MSRGRI
CruV-376	HFVYKH	LHTHV	TLVRYLAK	ETGACGKSTFIR	LFDLP	VFAN	LSLDRW
CruV-377	SFTWNK	LHYQG	NAEFYCCK	AEGCTGKSNFAT	IFDVP	VFAN	LSVDRW
CruV-378	VFTLNN	PHLQG	QNKEYTQK	GPAGAGKSRGAR	IMDDF	ITSQ	AIKRRF
CruV-379	CYTLNN	LHCQG	QAADYCKR	GATGVGKSAAAY	IFDEM	FTSN	PFMRRI
CruV-380	VFTCNN	PHLQG	ASIEYCEK	GPTGSGKSRAAR	ILDDV	VTSN	ALHRRF
CruV-381	PLTRFN	FHWQI	QNYKYCTK	GPSGIGKTSWAL	VFDEI	FCSN	AIERRV
CruV-382	CFTIHD	PHLQG	LARHYCMK	GKSRSGKSSSAI	VNEMS	MTTN	IFNRIT
CruV-383	MVTYND	PHLQM	DAAGYCTL	GDGATLKSSCIR	AIDDP	ITAN	PMIRRF
CruV-384	VYTLNN	PHLQG	EAVTYCQK	GNPRTGKSRSAR	LIEDI	ITSN	PLQERF
CruV-385	CVTYNL	RHIQG	QNVRYCSK	GRTGLAKTHGTI	LIDDL	VTSN	ALKRRM
CruV-386		WHYQC	AAWRYCYN	AEGQSGTTQMFK	CIDIT	IKWN	VSAGRM
CruV-387	CYTLNS	LHIQG	QAADYCRD	GESGTGKSQAAY	LFDEF	FTSN	PLTRRI
CruV-388	CWTHYD	KHMQG	KNEEYVGK	KGGGAGKSACAD	CFDFS	VFAN	LSEDKL
CruV-389	LLTYKT	PHTHA	RVLKYITK	RVGNMGKTVLAK	TIDLP	VLAN	LSLDRW
CruV-390	VFTLNN	LHLQG	KNFTYCTK	GPSGTGKSRYAR	LVEDV	VTSQ	ALKRRF
CruV-391	FLTYSQ	IHIHA	GLVRYLRD	APSGWGKSEWIQ	IFDDI	VLSN	QIKQRF
CruV-392	FLTYAQ	VHRHA	GSLKYVLE	GGTNIGKTTLMN	VFDEF	FLSN	TLMNRL
CruV-393	CYTLNN	PHLQG	QNRAYCTK	GPTGSGKSHAAR	IFDDF	FTAP	QFTRRI
CruV-394	IFTLNN	HHLQG	QAEAYATK	GPTGTGKTKAAA	IIDDY	ITAP	QLGRRL
CruV-395	AFTWFG	PHLQG	ANFRYASK	IAGGQGKTLFSK	IVDLT	VFCN	LSADRV
CruV-396	CITLNN	PHIQG	QNREYCSK	GPTGTGKTFRAI	IFDDF	ITSN	PLYRRI
CruV-397	NFTDPN	PHLQC	ACIEYCGK	GPTGSGKSRWAM	IIDDF	FTTP	QFSRRL
CruV-398	FFTWNN	KHIQG	EALAYCTK	PNGGAGKTVLAT	IWDIP	IFSN	LSKDRW
CruV-399	SFTWNK	LHYQG	EAEFYCMK	ETGRTGKSEFAT	IFDVP	VFSN	LSHDRW
CruV-400		LHWQV	AVLSYCMK	TTGQAGFTSLMS	IIDIT	IKTN	LSEGRM
CruV-401	AFTMNN	RHLQG	KSIDYCKK	GPTGTGKSSTAR	IMDDI	ITSN	ALNRRF
CruV-402	CFTLNN	AHVQG	ENQAYCSK	GSTGTGKSRFAN	IIDDY	ITTP	QLERRV
CruV-403	AFTWNS	DHYQG	GLDFYVVK	QSGGVGKSKLQK	CNIP	VFSN	ASPDRW
CruV-404	CFTLNN	PHLQG	SCIDYCKK	GKTGVGKTRYLY	IFDDI	FTSS	QLLRRV
CruV-405	CGTLNN	PHLQF	QATDYCRK	GPPGTGKTRAAY	LLDEF	ISAN	ALRRRF
CruV-406	LVTWFI	KHYQG	QAIAYCTK	GETGTGKSRKVY	LIDDF	ITSN	AFFRRV
CruV-407	LVTWFI	KHYQG	QAIAYCTK	GETGTGKSRKVY	LIDDF	ITSN	AFFRRV

	Endonuclease domain			Helicase domain			
	Motif I	Motif II	Motif III	Walker A	Walker B	Motif C	Arg finger
CruV-408	CFTVFG	VHIQG	KNTKYCTK	GGSGMGKTFSTY	LIDDF	ITSE	NLRRRA
CruV-409	CWTWNN	PHLQA	QNYTYCSK	GTAGAGKTRAAF	ILDDF	ITSN	ALERRL
CruV-410		PHLQA	QNYTYCSK	GTAGAGKTRAAF	ILDDF	ITSN	ALERRL
CruV-411	CFTWNN	PHLQG	QNYAYCSK	GTAGAGKTRAAF	ILDDF	ITSN	ALERRL
CruV-412	LLTTKL	IHWQW	QACEYVIK	EEGGKGKSMLAR	IIDIA	IMTN	LSSDRW
CruV-413	CFTLNN	PHLQG	DNYLYCSK	RTGGVGKSWLAT	CFDIS	VFAN	FSADRL
CruV-414	VFTLNN	PHLQG	QARDYCRK	GPTGSGKSRAAF	VIDDY	ITSP	QLVRRV
CruV-415	CFTSFN	EHAQG	QAIYYCEK	GSSRVRKTSVAY	IIDEI	FTSN	ALKNRF
CruV-416	MLTIYN	PHLQG	SNTAYCKK	GTTGTGKSRWTK	VIDDL	ITNP	QLTRRI
CruV-417	FGTVNG	PHLHF	KCLDYMKK	GESGSGKSLSAR	IIDDL	VTSN	ALLRRF
CruV-418	IFTWWC	RHLQG	GSRDYCLS	GSAGKNKTRAAY	LIDEF	ITTN	AILRRI
CruV-419	CFTMNN	PHLQG	ASIEYCRK	GPTGTGKSTRAR	ILDDV	VTSQ	ALRRRF
CruV-420	VFRHSN	PHLQG	DSQNYCMK	GPTGTGKTRTIY	VLDEF	ITSP	QLIRRI
CruV-421	SWTWHL	LHYQG	EAEFYCLK	EEGCTGKSHFAT	IFDIP	VFSN	LSKDRW
CruV-422	SFTLNN	PHLQG	ENIVYCSK	GRTGSGKSKFAF	IVDEF	ITSQ	ALLRRI
CruV-423	SWTWNK	LHYQG	DAEWYCLK	NEGCNGKSHFAT	IFDIP	VLAN	LSVDRW
CruV-424	CFTHNN	RHLQG	ASIEYCKK	GPTGTGKSTLAR	IIDDL	VTSQ	ALERRF
CruV-425	CFTINH	PHLQG	QAADYCKK	GASGTGKSKTAR	IIEEW	ITSN	PLKRRI
CruV-426	VVTVNN	PHLQC	QNFIYCSK		IIEDM		
CruV-427	MFTWFD	PHLQG	DNYNYCRK	GDTGCGKSVAIK	VMDDI	FISN	QLTRRI
CruV-428	CYTLNN	LHCQG	QAADYCRD	GETGTGKSNAAY	LMEEF	FTSN	PFLRRI
CruV-429	CFTLNN	PHLQG	QNIDYCFK	GKTGFGKTRCSM	ICDDF	ITTP	QLTRRV
CruV-430	SFTWNK	LHYQG	DAEFYCTK	NIGCVGKTNFAT	IFDVP	ILAN	MSADRW
CruV-431	MLTYMC	LHTHV	NRVKYLSK	RSGSVGKTDLVK	LIDIP	IFSN	LKIDRW
CruV-432	CFTLNN	PHLQG	SNKVYCSK	GATGSGKSFAAH	VLDDY	VTSN	AACRRV
CruV-433	CPVCAD	PHLQM	KSITYCTK	GPPGTGKSLLAA	ILDDF	ITTN	AILRRM
CruV-434	FITSSN	PHLQF	SNILYVLK	ATGNVGKSWMAT	CFDIS	VFAN	FSQDRL
CruV-435	SWTWNK	LHYQG	DAEFYCMK	ETGCTGKSYLAT	LFDIP	VLAN	LSLDRW
CruV-437	CFTMWD	PHLQG	ACRAYCLK	GPPGTGKTHLAK	FIDEL	ITSN	PLKRRL
CruV-438	AFTLNN	PHLQG	SSIAYCKK	GPTGTGKSRFAR	LIEDV	ITSN	PLLRRC
CruV-439	CFTDYD	PHLQG	DCISYCEK	GKTGVGKTYAAI	IIDDF	VTSN	PLRRRF
CruV-440	KLTTPA	QHSKA	LSLLCQEV	GESTGSTGRTVG	LSDEL	ALVP	TPNKRC
CruV-441	VFTSFP	RHVHV	GWIDYCQK			IELI	
CruV-442	AFTWNN	PHLQG	ASIEYCKK	GPTGSGKSRSAR	IVDDV	VTSN	PLMRRF
CruV-443	CFTFNN	PHLQA	QASDYCKK	GESGSGKTELAK	IIDDF	VTCD	QIKSRF
CruV-444	MFTIFQ	EHLQG	QCKTYCSK	QDGNSGKTLLAK	VMDYT	VFAN	LSIDRF
CruV-445	CLTVYN	VHQHW	EAVMYFLK	GPTGTGKSLLCT	VLDEV	ITAP	QLRRRL
CruV-446	CFTDYD	PHLQG	DCISYCEK	GKTGVGKTYAAI	IIDDF	VTSN	PLRRRF
CruV-447	CFTLNN	RHLQG	ASIAYCKK	GETGSGKSRCAR	VIEDV	VTSN	ALFRRF
CruV-448	AFTQWN	RHLQG	DNIDYCSK	GKSRAGKSHDAK	FENL	MCGN	HTERKE
CruV-449	CYTVNN	EHIQG	QAKHYCMK	GPSGSGKSRIAE	ILDDF	FTSN	ALHRRI
CruV-450	CLTGHF	PHLQG	QNIAYCSK	GPTGSGKSFSAR	ILDDF	ITSN	PILRRF
CruV-451	SWTFNK	LHYQG	QAEFYCLK	TEGCTGKSSFAT	IFDVP	VFAN	LSMDRW
CruV-452	SWTWNR	LHYQG	DAEWYCMK	DQGCKGKSHFAT	IFDIP	VLAN	MSVDRW
CruV-453	LVYNFN	VHLQC	HATEYCKK	GPTGTGKSRDAN	FWDEF	FTAP	QFLRRL
CruV-454	LVYNFN	VHLQC	HATEYCKK	GPTGTGKSRDAN	FWDEF	FTAP	QFLRRL

	Endonuclease domain			Helicase domain			
	Motif I	Motif II	Motif III	Walker A	Walker B	Motif C	Arg finger
CruV-455	LVYNFN	VHLQC	HATEYCKK	GPTGTGKSRDAN	FWDEF	FTAP	QFLRRL
CruV-456	LVYNFN	VHLQC	HATEYCKK	GPTGTGKSRDAN	FWDEF	FTAP	QFLRRL
CruV-457	CFTIYD	IHLQC	QNRDYCKK	RTPGRGKTDCLS	VIDDF	VTSN	PIKARF
CruV-458	CFTQKF	KHYQG	QNFEYVTK	GPSGCGKSHAAM	IMDDF	ITAI	QFRRRF
CruV-459	CFTLND	QHLQG	QAKQYCEK	GPTGSGKSRRAL	VLDDF	ITSN	ALVRRL
CruV-460	CLTGHY	PHLQA	DNIAYCSK	GKTGTGKSHAMR	IIDDF	VTSN	PILRRF
CruV-461	MFTWNN	RHYQG	QADAYCSK	GGPGVGKTRDAL	VLDEI	IFLC	QVERRI
CruV-462	VFTLNN	PHLQG	QASDYCKK	GAPGTGKSLCAR	LIEEW	VTSN	ALKRRF
CruV-463	CVTINN	KHFQM	QNEDYCAG	RTSGVGKTSAVR	LLDDF	VTSN	PINNRF
CruV-464	SWTFHM	LHYQG	NAEFYCMK	DTGCTGKTDFSI	IFDIP	VFSN	LSKDRW
CruV-465	PFTYNY	EHVQG	QNRDYCAK	GASGTGKSYYAR	IDDFD	VTSN	PILRRF
CruV-466	SITFNK	LHYQL	NAEFYCMK	ENGCKGKSHFAT	IFDIP	VLSN	LSADRW
CruV-467	CFTLNN	PHLQG	ENRVYCSK	GPTGCGKSKSVR	LIEDI	VTSQ	ALERRF
CruV-468	LLTYKS	RHTHV	DAKAYIGK	KRGNTGKSWLSR	IMDLA	VFAN	MSLDRW
CruV-469	VFTCFQ	VHWQG	RARAYCKK	GPTGTGKSLWAH	IMEEF	LTSN	AAQRRL
CruV-470	CFTVFF	PHFQG	QAIDYCHT	TDGRCGKTAFTR	LFDIE	VFSN	ATPDVW
CruV-472	VFTLNG	SHLQG	HVRSYCVK	GPSNSGKTYQAN	INEF	VTCV	QFLRRI
CruV-473	MSLAKN			GPSGTGKSHAAR	IIEDF	VTSN	PLLRRF
CruV-474	CFTLNN	PHLQG	KAWDYCDK	VEGGTGKSTFAR	LFDFA	IFTN	LSRDRW
CruV-475	FYTARE	LSTTG	RASRYSAR	GGTGTGKTRSVY	LLDEL	ITAP	QLLRRI
CruV-476	FCTYSD	LHFHV	RTYEYCTK	GPPDCGKSFWVN	IYDDV	IVCN	TFTTRF
CruV-477	CFTQHQ	LHLQG	QAINYCKK	GPSGVGKSREAA	IIDDY	ITSN	ALNRRI
CruV-478	CFVHWI	DHYQG	DNYDYITC	YNFGTGKSHSAK	LIDEI	ITSN	CLKRRM
CruV-479	LLTYKS	RHTHV	DAKAYIGK	KRGNTGKSWFSR	IMDLA	VFAN	MSLDRW
CruV-480	CLTAYN	AHLQG	ANYKYCSK	GTTGVGKTFSVD	VIDDM	ITNP	QLMRRI
CruV-481	VFTLNH	PDIQG	QSLDYCTK	GPTGTQKTRSSF	IFDDY	VTCP	QLSRRI
CruV-482	CFTKFL	LHIQG	SAREYCRK	GASGAGKTRRAI	IIDDF	ITSN	ALRRRI
CruV-483	CWTLNN	PHLQG	IAIEYCKK				
CruV-484	TITYNK	PHLQM	FNSDYCEG	KASGVGKTTAIR	LLDDF	VTSN	PICNRF
CruV-485	DFRANE	RHYQG	GQAFYALK	HTGNLGKSFLVA	LIDMP	VFTN	LSADRW
CruV-486	HLTYRS	DHTHV	KNVVYTTY	GETGLAKTQWAV	IFDDM	FTSN	AVLRRV
CruV-487	CGTLHN	PHLQI	QAREYCMK	GKPNAGKTYKFY	LIDDF	VTTN	ALLRRI
CruV-488	MFTIFQ	EHLQG	QCKAYCSK	QDGNSGKTVLAK	VMDYT	VLAN	LSQDRW
CruV-489		PHLQG	QNERYCSK	GRPGCGKTRQCY	LLDEF	LTSN	ALRRRI
CruV-490	CFTVNN	PHLQG	EAWDYCLK	GPTGSGKSRAAF	ILDDF	ITSC	QLLRRI
CruV-491		KHLQG	ASIRYCKK	GPTGTGKSHFAR	IIEEL	VTSN	PLRRRF
CruV-492	CGTLHN	PHLQI	QAREYCMK	GKPGCGKTYKFY	LIDDF	VTTN	ALLRRF
CruV-493			ANFEYCSK	GVPGCGKTEYAL	LIDDF	ITTN	ALFRRI
CruV-494	CFTINN	VHLQG	QNFLYCAK	GPSGTGKTQFAL	VFDDM	FTHN	AIERRY
CruV-495			DSNRYCRK	GKTGRGKTRAVY	LFDDF	FTTP	QLSRRV
CruV-496		AHTHV	RLWEYHQK	GPTGIGKTQWAL	VFDDT	ITSN	AIKRRC
CruV-497	PWTLNN	PHLQGSHI TG	QAINYCKK	GKPGTGKTRKAY	LLDEF	VTSN	ALRRRF
CruV-498	VFTINN	LHLQG	LARNYVLK	GDTNIGKTSFAK	VFDDM	FTCN	AILRRV
CruV-499	SLTVNN	PHLQC	SLHKYCEK				
CruV-500	VFTLNN	PHLQS	NTTQYYPT	SCSNGRCSQIHL			PMSNRK

	Endonuclease domain			Helicase domain			
	Motif I	Motif II	Motif III	Walker A	Walker B	Motif C	Arg finger
CruV-501	LYTKFN	PHIQG	QNFVYCSK	GSSGIGKTHYAA	IFDDM	FTHN	AIERRL
CruV-502	CFTIHR	VHLQG	SNWDYCTK	GATGTGKTYYAS	IIDDY	ITSA	QLTDRC
CruV-503	FGTCNN	RHLQF	QNITYCSK	GESGSGKSTLAD	CQEFR	FTQC	QLLRRV
CruV-504	CFTLNN	PHLQG	QARDYCRK	GETYSGKTHQAF	LLDDF	ITTN	ALKRRI
CruV-505	CFRANA	LHHQG	QAWAYSTK	GDSNCGKTHFVL	VFDDL	FTHN	AIDRRV
CruV-506	CFRANA	LHHQG	QAWAYSTK	GDSNCGKTHFVL	VFDDL	FTHN	AIDRRV
CruV-507	CFTINN	PHIQG	QNMLYCKK	GPSGTGKTQWAL	VFDDM	FTTN	AIERRY
CruV-508	VFTLNN	PHLQG	QASDYCKK	GAPGTGKSLCAR	LIEEW	VTSN	QALRRR
CruV-509	LYTKFN	PHIQG	QNFVYCSK	GSSGIGKTHYAA	IFDDM	FTHN	AIERRL
CruV-510	TFRKSA	LHFQG	QNWAYATK	GPSNTGKTSFVK	IFDDM	FTHN	AIDRRV
CruV-511	CFSHWN	HHYQG	ENHVYCTK	GVKGAGKTQDAM	IIDEA	ITMN	YPVRHP
CruV-512	HLTYPS	PHTHA	ACWNYHEK	GRTGIGKTQWAL	VFDDL	FTSN	AIARRC
CruV-513		LHYQG	QVWAYSTK	GEPGSGKTEFAR	IIEDF		
CruV-514	TFVLNN	KHFQG	TNAKYCSK	GPPGTGKSFMAW	IIDDF	ITTN	QLRRRV
CruV-515	VFTLNN	PHLQG	QASDYCKK				
CruV-516	SLVVER	GHSWM	RHRAYFSQ	GGSGIGKSMFAQ	IFDDC	ITSN	AIERRM
CruV-517	VFTLNN	PHLQG	QASDYCKK				
CruV-518	IFRMSN	PHLQG	QNEKYCSK	GPPGCGKTELAK	LIDDF	ITSN	ALFRRF
CruV-519		THWWA		GAAGLGKTHAAL	FNEA	LTTN	AFRRRG
CruV-520	CFTVNN	PHLQG	QNRDYCLK	GSPGTGKSVWAR	LIEDV	VTSN	ALRRRF
CruV-521	CFTLND	KHYQG	QARDYCMK	GKPGSGKTQLFW	LIDDF	ITTN	ALKRRV
CruV-522		LHYQG	QNWAYCTK	GPPGVGKSEFAR	ILEDF	ITTN	
CruV-523	CVTYFG	LHCQA	ENIVYCTK	GETGGGKTRAAF	IFDDF	ITCP	QLLRRI
CruV-524	VFTWND	KHYQG	QAREYAMK	GPPGTGKSRCVR	LIDDF	ITTN	ALKRRI
CruV-525	CFTLND	KHYQG	QARDYCMK	GTPGTGKTQNFW	LIDDF	VTSN	ALKRRF
CruV-526	LLTQQG	AHAHI	RAVEYVKK	GPSRTGKSYHAR	LIEDL	VTSN	PICNRF
CruV-527	CFTWFK	EHLQC	RARLYCMK	GKPGTGKTRRAY	LLDDF	ITTN	ALKRRF
CruV-528	CFTWFK	EHLQC	RARLYCMK	GKPGTGKTRRAY	LLDDF	ITTN	ALKRRF
CruV-529	VFTTNN	PHLQG	EAATYCKK	QVAVRNGGFPKC	ILDDW	ITTT	QLLRRV
CruV-530	ISTPTL		AAIQYYRV	GASRTGKSTHMK	VHDCA	IDSQ	AFCKRF
CruV-531	VLTHNN	PHVQA	GGVDYILR	GVSGSGKSHTAR	INDFY	ITSN	AVERRI
CruV-532		LHWQV	QSLDYCNK	GPSGAGKSTRCR	IIEDL	ITTN	RWARRF
CruV-534	VFRSNA	LHHQG	QAWAYSVK	GPPGTGKTDFAY	ILDEF	ITTN	ALFRRI
CruV-535	VFTARA	LHYQG	SNWTYCTK	GPPGTGKTDFAY	IIDEF	ITTN	ALFRRI
CruV-536	VFTLNN	PHFQG	QAIEYCKK	GKTGTGKTYKAF	LLDEV	ITTN	ALLRRF
CruV-537	CWTLNN	PHLQG					
CruV-538	IVVINN	PHFQC	NNQDYCKK	GPTGLGKSKLAR	LIEDF	VTSN	PLLRRF
CruV-539	FLTKNN	PHLHA	SCITYCKK	GETGFGKTSFAF	IIDEI	ITAP	HLLRRI
CruV-540	FFTLFV	LHIQG	FGMNYCEK	GPTSTGKTTYAE	IDDLT	LTSN	
CruV-541	VLTCNN	PHLQC		GKPGTGKTHAAT	IIDDF	VTSN	AIMRRC
