# Supplementary material for: Unveiling Crucivirus Diversity by Mining Metagenomic Data
Source: mBio. 2020 Sep 1;11(5):e01410-20. doi: 10.1128/mBio.01410-20 (PMC7468197; doi:10.1128/mBio.01410-20)
Supplement: TEXT S1 [file mBio.01410-20-s0001.docx]

```
LOCUS       CruV-81                 2472 bp    DNA     circular     24-FEB-2020
DEFINITION  Crucivirus-81 isolate SR5_32407.
ACCESSION   CruV-81
KEYWORDS    .
SOURCE      Crucivirus-81
  ORGANISM  Crucivirus-81
            Unclassified.
REFERENCE   1  (bases 1 to 2472)
  AUTHORS   de la Higuera,I., Kasun,G.W., Torrance,E.L., Pratt,A.A.,
            Maluenda,A., Colombet,J., Bisseux,M., Ravet,V., Dayaram,A.,
            Stainton,D., Kraberger,S., Zawar-Reza,P., Goldstien,S., Briskie,J.,
            White,R., Taylor,H., Gomez,C., Ainley,D.G., Harding,J.S.,
            Fontenele,R.S., Schreck,J., Ribeiro,S.G., Oswald,S.A., Arnold,J.M.,
            Varsani,A., Enault,F. and Stedman,K.M.
  TITLE     Direct Submission
  JOURNAL   Submitted (24-FEB-2020) Biology, Portland State University, 1719 SW
            10th Ave, Portland, Oregon 97201, USA
COMMENT     Bankit Comment: ALT EMAIL:ide@pdx.edu
            Bankit Comment: TOTAL # OF SEQS:451
            Bankit Comment: GAP: est. length - alignment

            ##Assembly-Data-START##
            Assembly Method       :: MetaSPAdes v. 3.12; IDBA-UD v. 1.1.1;
                                     MEGAHIT v. 1.0.3
            Sequencing Technology :: 454; Illumina
            ##Assembly-Data-END##
FEATURES             Location/Qualifiers
     source          1..2472
                     /organism="Crucivirus-81"
                     /mol_type="genomic DNA"
                     /isolate="SR5_32407"
                     /isolation_source="Water"
                     /country="New Zealand"
                     /collection_date=2012
     CDS             1..1308
                     /codon_start=1
                     /product="Putative CruV Capsid Protein"
                     /translation="MSKNYSGNRRPKRSGFQQSKNRRVQIIEAPRIRRERAPRQIAAA
                     PAAAQPEQSIGQKIGSGLGGLVGGGLQQLFKAVTGLGDYTINKNVLMEQDPLSIQNKS
                     DRGGVVIRHREYLADIYTSAAIGAFRLQAFGLNPGQENTFPFLSQVAANFEEYSFEGV
                     IFEFRSMSADALNSTNTALGSVMMATNYNAGSANFTTKSEMENYEYGSSCKPSINMMH
                     PIECAPRQTAISTELYVRPGPLPVGEDIRLYDWGKFQIATVGFQAASVNIGELWVSYQ
                     VCLQKPKMYASLGKMNEYSSFAFVGGSVADPFLQSGLDTRVNQIGIIFQNNQSFTFPG
                     AVGVPRTYQLQYVVNGSVPVVYVPPVISYSTGVTASVLYATTPHPQSGLAGSLAVDIL
                     WEIQIDANVLSPTVTFGVAGTYPTGGTSYLKVSQVSNAIFLLG*"
     CDS             complement(1486..2376)
                     /codon_start=1
                     /product="Putative Replication Associated Protein"
                     /translation="MQKLERSGAGKIGNINQSSNPKKDNERNQRVHWFFTWNNYTEEN
                     IEMLIKFFKANSKKYIFQEEIGDLNKTKHLQGNVQLKKRMRWSEFGLPKQICWEPTRN
                     IDKANAYCCKEETKNGRTFKFGFPSELKLITTLRPWQSKICTIINGEINDRFVYWIYD
                     EMGENGKTVFCKYLYANQDAIIATDGANKDVSCMLAELVKNGRDLNDKTTFIFHFPRS
                     TEGLSYKAIESVKDGLITSTKFHSNTLVFNCPHVFVFSNELPNLAKLSQDRWKLKTII
                     NGDLFDYEEEILDPIFIDNL*"
     misc_feature    2321..2347
                     /note="stem-loop"
     misc_feature    2331..2339
                     /note="nonanucleotide"
ORIGIN
        1 atgtcaaaaa attattcagg aaataggaga ccaaaaagga gtggatttca acaatctaag
       61 aacagaagag ttcagattat tgaggcacct agaattcgtc gtgagcgtgc ccctcgtcag
      121 atagctgcag ctcctgcagc tgcgcaaccc gaacaaagta ttggtcaaaa gattggatct
      181 ggtttaggtg gtttagtggg tggtggttta caacagttat ttaaagctgt aactggatta
      241 ggggattata caataaataa gaatgtgtta atggaacaag atcccctatc tattcaaaat
      301 aaaagcgata gaggtggagt cgtgattagg catcgagaat atcttgcaga tatttatact
      361 tctgctgcta tcggtgcatt tagacttcaa gcttttggat tgaatcctgg acaagaaaat
      421 acttttcctt ttctgtctca agttgcagcg aatttcgaag aatattcgtt cgaaggagta
      481 atatttgaat ttagaagtat gtctgcagac gctctaaatt cgactaatac tgctttaggt
      541 agtgttatga tggcaaccaa ttataacgct ggttctgcta attttacaac taaatcagag
      601 atggaaaatt atgagtacgg ttcaagttgt aaaccatcaa tcaatatgat gcatcctatt
      661 gaatgtgctc ctcgtcagac tgctatctct actgagttat atgttcgccc aggtccttta
      721 cctgttggtg aagatataag gctgtatgat tggggtaaat tccagatagc aactgttggt
      781 tttcaagctg cttctgttaa tatcggagaa ttatgggtta gttatcaggt ttgtttgcaa
      841 aagccaaaga tgtatgcgtc attgggtaaa atgaatgaat attcttcttt tgcttttgtt
      901 ggcggttcag ttgcagatcc ctttttgcaa tctggtttgg ataccagagt taatcaaata
      961 gggattattt ttcaaaataa tcaatctttt acctttccag gtgctgttgg tgttcctcga
     1021 acatatcagc ttcaatatgt tgttaatggt tcagttcctg tagtttacgt acctccagta
     1081 atatcatatt caactggggt tactgctagt gttttatatg caacgacacc tcatcctcaa
     1141 tcaggattag ctggttcgtt ggcagttgat attttatggg aaatacaaat agatgcaaat
     1201 gtattgtctc ctacagttac atttggggtt gctggtacat atccaactgg aggtacctct
     1261 tatcttaaag tatcacaagt atcaaatgct atatttttat tgggataatt taaattattt
     1321 aataaatgtt ttgtaataag atatttatta aatttgtttc cactcctttt tatatagttt
     1381 ttcgtaacgg atagcgagca acgtagtgta acgaggctgt cgatgcggtg aagaaaaacc
     1441 gaggaatttt attccgagct taaaacatat aagatggttt atttttcata aattatcgat
     1501 gaatattgga tcgagaattt cttcttcgta atcgaatagg tctccgttta ttattgtctt
     1561 tagtttccat ctatcttggc ttagtttagc caaatttgga agttcgttgc tgaaaacgaa
     1621 aacatgagga caattaaaca ctaaagtgtt actgtggaat ttagtacttg taattaatcc
     1681 atctttaaca ctttcgatag ctttatagct gagtccttct gtcgatctgg gaaaatgaaa
     1741 aataaaagtt gttttatcgt ttaagtcacg accgtttttt acaagttctg ctagcatgca
     1801 tgagacgtct ttattggctc catcggttgc aataatagcg tcttgatttg cgtaaagata
     1861 tttgcaaaaa acggtttttc cattttctcc catttcgtcg tatatccagt agacgaatct
     1921 atcattaatt tcaccattta taattgtgca aatttttgat tgccatggtc ttaaagtagt
     1981 aattaattta agttctgaag gaaatccaaa tttaaacgtt ctaccatttt ttgtttcttc
     2041 tttacaacaa tatgcattag ctttgtctat gtttcttgtt ggttcccagc aaatttgttt
     2101 aggtagacca aattcgctcc atctcattct ttttttgagt tgaacgtttc cttgtaaatg
     2161 tttagtttta tttaagtctc caatctcttc ttgaaatata tatttcttag aattggcttt
     2221 aaaaaacttt attaacattt ctatattttc ttcagtataa ttgttccaag tgaaaaacca
     2281 atgtactctt tggtttcttt cattgtcttt tttagggtta gaagattgat taatattacc
     2341 aatcttcccc gctccagatc gctccaattt ttgcatattt tataattact atagataata
     2401 aattttagtt taattaaacg cgcgtaaatt agtttagaat tattttgtat atagtaatta
     2461 taatatagaa at
//
LOCUS       CruV-83                 2612 bp    DNA     circular     24-FEB-2020
DEFINITION  Crucivirus-83 isolate 120_2207_IR2_3297.
ACCESSION   CruV-83
KEYWORDS    .
SOURCE      Crucivirus-83
  ORGANISM  Crucivirus-83
            Unclassified.
REFERENCE   1  (bases 1 to 2612)
  AUTHORS   de la Higuera,I., Kasun,G.W., Torrance,E.L., Pratt,A.A.,
            Maluenda,A., Colombet,J., Bisseux,M., Ravet,V., Dayaram,A.,
            Stainton,D., Kraberger,S., Zawar-Reza,P., Goldstien,S., Briskie,J.,
            White,R., Taylor,H., Gomez,C., Ainley,D.G., Harding,J.S.,
            Fontenele,R.S., Schreck,J., Ribeiro,S.G., Oswald,S.A., Arnold,J.M.,
            Varsani,A., Enault,F. and Stedman,K.M.
  TITLE     Direct Submission
  JOURNAL   Submitted (24-FEB-2020) Biology, Portland State University, 1719 SW
            10th Ave, Portland, Oregon 97201, USA
COMMENT     Bankit Comment: ALT EMAIL:ide@pdx.edu
            Bankit Comment: TOTAL # OF SEQS:451
            Bankit Comment: GAP: est. length - alignment

            ##Assembly-Data-START##
            Assembly Method       :: MetaSPAdes v. 3.12; IDBA-UD v. 1.1.1;
                                     MEGAHIT v. 1.0.3
            Sequencing Technology :: 454; Illumina
            ##Assembly-Data-END##
FEATURES             Location/Qualifiers
     source          1..2612
                     /organism="Crucivirus-83"
                     /mol_type="genomic DNA"
                     /isolate="120_2207_IR2_3297"
                     /isolation_source="Water"
                     /country="Norway"
                     /collection_date=2011
     CDS             join(1..291,338..835)
                     /codon_start=1
                     /product="Putative Replication Associated Protein"
                     /translation="MAYRQWIGTISAGTWTVPTNLEAPTTYLKGQKEIGEGGYEHYQL
                     IAYFSSPVRLSRVQLLLNAPSAHFEKTRSKAAEAYVWKEATRVPGSQFELGRKDWDKI
                     LVAAKEGDFDGIPSDVIIRCYNQLSRISKDYMAPVGQEKTVYVYWGRTGTGKSRRAWD
                     EATLVAYPKDPCTKWWCGYRGQSTVVIDEFRGRIGIEHLLRWTDRYPVVVESKGGAVV
                     LRCTSIWITSNLSPDVWYPDIDAESLAALRRRLNIIHFNYVVFL*"
     CDS             complement(1035..2414)
                     /codon_start=1
                     /product="Putative CruV Capsid Protein"
                     /translation="MPKGTPCTIAGSIYPRPPKPKSNKRKARGQPSGPRRPAKIGGMP
                     MGPVMTGSGDYNLGPYSYKGDTPYARVGRKLGKAAGLFGLPSWVADAGEALGHGVGRL
                     FGSGDYTTGPMVRSNCLTNSTDVPMFSGNGRGTIITHREYVNDITTSSVIGAFKNQSF
                     TIQPGNAITFPWLSSIAEHYEQYRIHGMVFYFKSTSGDSVGSTNTAIGTVVLSTDYNV
                     NSTAYTTKQQMENAEFAQSAKASANQVHGIECDVAERSVAVYFVRSDSINATDSLKWY
                     DFANFQIATQGFQAANVNVGELWVSYTIEFFKPQVATTIGGTVQGLAIERTGITNALP
                     LGTGTNYGRGDLPFSITNTALTLSGLQDGTLLFFNVTWIGSVAAVIVDPIITFTNATL
                     NTVFYGVATGVPVPGTSTARYMQFLSIRVNNASLGVVTMTVGAAGTLPTGTVVCNILV
                     TQIDSSLLG*"
ORIGIN
        1 atggcctacc gacaatggat cggcacgatc tcagccggca cctggactgt ccccaccaac
       61 cttgaagcgc caaccacgta tctcaaaggc caaaaagaaa tcggtgaagg aggctatgag
      121 cattatcagc tcattgccta cttctcctct cccgttagac tttcacgagt tcagctgctc
      181 ctcaacgccc ccagcgctca tttcgagaag acccgctcta aagcagccga agcgtacgtc
      241 tggaaagaag caacccgcgt tcccggttct cagttcgagc tcggaagaaa ggttaatttt
      301 tgtataaaat taggcttttg atcgatcgaa cgctaaggac tgggataaga tcctcgtcgc
      361 cgcaaaagaa ggtgattttg atgggatccc atccgatgtc attatccgct gttataacca
      421 gctctcccga atcagcaaag attatatggc accggttggt caagaaaaaa cagtctatgt
      481 ttactgggga cgtacaggaa ccggtaagag tagacgtgcc tgggatgagg ccactctggt
      541 tgcttatcct aaagacccat gtacaaaatg gtggtgcgga tatcgagggc aatcaacagt
      601 tgttatcgat gaatttagag gtaggattgg tattgagcat ttgttgcgat ggacagacag
      661 atatcctgtc gttgtggaaa gcaaaggagg agctgttgtg ctcagatgta ctagtatatg
      721 gattacctca aatctttccc ctgatgtgtg gtatcccgat atagatgctg aaagtcttgc
      781 ggctttgaga cgaagattaa atataatcca ttttaattac gttgttttcc tgtagcttag
      841 taagtttgcc gcaaaatatt ataatatcca agtagaatat tttgcggcga acctaaccct
      901 aaccctaacc ctaaccctaa ccctaaccct aaaacgtgac tttattaaaa cttcctttaa
      961 aaaaagagga ggccttggac tccgataagg acttgctaaa atgaaaatgt aagcattttg
     1021 taataacttt attattaccc taataacgaa gaatcaattt gtgtcaccaa aatgttgcaa
     1081 actactgtgc ctgtgggtaa ggtgccggct gcccctactg tcatagttac aacccctaag
     1141 gatgcattgt taactcgaat tgacaagaat tgcatataac gtgccgtaga ggttcctgga
     1201 acaggtactc ctgtcgcaac accgtaaaag acggtgttga gagtcgcgtt agtgaatgta
     1261 ataattgggt caacgatcac tgcagctaca ctaccaatcc acgtaacatt gaaaaagagt
     1321 agagttccat cctgtagccc actcagggtc agggcagtgt ttgtgataga aaagggcaaa
     1381 tcccctcgcc cgtaatttgt acctgttcct agaggtagcg cgttggttat gccagttcgc
     1441 tcaatcgcaa ggccttgcac agttccacct atggttgttg caacttgggg tttgaaaaat
     1501 tcgatggtgt aacttaccca taactcgcct acgtttacgt tcgccgcttg gaatccctgt
     1561 gttgcgattt gaaaattggc aaaatcatac cacttgagac tgtcagttgc atttatggag
     1621 tcgcttcgaa cgaaatatac agctaccgac ctttctgcta catcacattc aatgccatga
     1681 acctgattcg cggacgcttt agcactctgc gcaaactctg cgttttccat ttgttgcttg
     1741 gtagtgtacg ccgtcgagtt aacattgtaa tccgtactca atactacagt tccaatggcg
     1801 gtgttagtcg agccgacgct atccccagag gtgcttttaa agtaaaagac cataccgtga
     1861 atgcggtatt gctcatagtg ctcggcaata gacgaaagcc aggggaacgt gattgcattg
     1921 cccggttgta tcgtgaatga ctggttcttg aaggctccaa tgacactgct tgttgtaata
     1981 tcatttacgt attcacgatg cgtaataatt gttcctcgac cgttgccaga aaacataggc
     2041 acatccgttg agttggtgag acaatttgaa cgaaccatcg gacccgtagt ataatcccct
     2101 gaaccaaata accgtcctac cccgtggccg agtgcctctc cggcgtctgc gacccaggaa
     2161 ggtaggccaa agaggcctgc cgctttgcct agctttctgc ctactctagc gtatggagta
     2221 tcgcccttat acgaatatgg gccgaggttg tagtcgccgg atcctgtcat gacgggcccc
     2281 atcggcatcc cgccgatctt agccggtcgt ctcgggccag agggttgtcc tctagccttg
     2341 cgcttgttgg acttgggttt tggtggccgt ggatagatac tacctgctat ggtgcatggc
     2401 gttcctttgg gcatataggg taaatattaa ggaggagtag atctataata acctagatgt
     2461 attttgatac atcttagtat tacttataga tcgacgtatc ctgtgtcact cttgggttcc
     2521 caaagaccct tacccttggt gccaaaaaag gacccttacc cgtataaaaa gggagtataa
     2581 aacaactttc ttatgtttga ttacgacgag ca
//
LOCUS       CruV-84                 2612 bp    DNA     circular     24-FEB-2020
DEFINITION  Crucivirus-84 isolate 120_820_IR1_3296.
ACCESSION   CruV-84
KEYWORDS    .
SOURCE      Crucivirus-84
  ORGANISM  Crucivirus-84
            Unclassified.
REFERENCE   1  (bases 1 to 2612)
  AUTHORS   de la Higuera,I., Kasun,G.W., Torrance,E.L., Pratt,A.A.,
            Maluenda,A., Colombet,J., Bisseux,M., Ravet,V., Dayaram,A.,
            Stainton,D., Kraberger,S., Zawar-Reza,P., Goldstien,S., Briskie,J.,
            White,R., Taylor,H., Gomez,C., Ainley,D.G., Harding,J.S.,
            Fontenele,R.S., Schreck,J., Ribeiro,S.G., Oswald,S.A., Arnold,J.M.,
            Varsani,A., Enault,F. and Stedman,K.M.
  TITLE     Direct Submission
  JOURNAL   Submitted (24-FEB-2020) Biology, Portland State University, 1719 SW
            10th Ave, Portland, Oregon 97201, USA
COMMENT     Bankit Comment: ALT EMAIL:ide@pdx.edu
            Bankit Comment: TOTAL # OF SEQS:451
            Bankit Comment: GAP: est. length - alignment

            ##Assembly-Data-START##
            Assembly Method       :: MetaSPAdes v. 3.12; IDBA-UD v. 1.1.1;
                                     MEGAHIT v. 1.0.3
            Sequencing Technology :: 454; Illumina
            ##Assembly-Data-END##
FEATURES             Location/Qualifiers
     source          1..2612
                     /organism="Crucivirus-84"
                     /mol_type="genomic DNA"
                     /isolate="120_820_IR1_3296"
                     /isolation_source="Water"
                     /country="Norway"
                     /collection_date=2011
     CDS             join(1..291,338..835)
                     /codon_start=1
                     /product="Putative Replication Associated Protein"
                     /translation="MAYRQWIGTISAGTWTVPTNLEAPTTYLKGQKEIGEGGYEHYQL
                     IAYFSSPVRLSRVQLLLNAPSAHFEKTRSKAAEAYVWKEATRVPGSQFELGRKDWDKI
                     LVAAKEGDFDGIPSDVIIRCYNQLSRISKDYMAPVGQEKTVYVYWGRTGTGKSRRAWD
                     EATLVAYPKDPCTKWWCGYRGQSTVVIDEFRGRIGIEHLLRWTDRYPVVVESKGGAVV
                     LRCTSIWITSNLSPDVWYPDIDAESLAALRRRLNIIHFNYVVFL*"
     CDS             complement(1035..2414)
                     /codon_start=1
                     /product="Putative CruV Capsid Protein"
                     /translation="MPKGTPCTIAGSIYPRPPKPKSNKRKARGQPSGPRRPAKIGGMP
                     MGPVMTGSGDYNLGPYSYKGDTPYARVGRKLGKAAGLFGLPSWVADAGEALGHGVGRL
                     FGSGDYTTGPMVRSNCLTNSTDVPMFSGNGRGTIITHREYVNDITTSSVIGAFKNQSF
                     TIQPGNAITFPWLSSIAEHYEQYRIHGMVFYFKSTSGDSVGSTNTAIGTVVLSTDYNV
                     NSTAYTTKQQMENAEFAQSAKASANQVHGIECDVAERSVALYFVRSDSINATDSLKWY
                     DFANFQIATQGFQAANVNVGELWVSYTIEFFKPQVATTIGGTVQGLAIERTGITNALP
                     LGTGTNYGRGDLPFSITNTALTLSGLQDGTLLFFNVTWIGSVAAVIVDPIITFTNATL
                     NTVFYGVATGVPVPGTSTARYMQFLSIRVNNASLGVVTMTVGAAGTLPTGTVVCNILV
                     TQIDSSLLG*"
ORIGIN
        1 atggcctacc gacaatggat cggcacgatc tcagccggca cctggactgt ccccaccaac
       61 cttgaagcgc caaccacgta tctcaaaggc caaaaagaaa tcggtgaagg aggctatgag
      121 cattatcagc tcattgccta cttctcctct cccgttagac tttcacgagt tcagctgctc
      181 ctcaacgccc ccagcgctca tttcgagaag acccgctcta aagcagccga agcgtacgtc
      241 tggaaagaag caacccgcgt tcccggttct cagttcgagc tcggaagaaa ggttaatttt
      301 tgtataaaat taggcttttg atcgatcgaa cgctaaggac tgggataaga tcctcgtcgc
      361 cgcaaaagaa ggtgattttg atgggatccc atccgatgtc attatccgct gttataacca
      421 gctctcccga atcagcaaag attatatggc accggttggt caagaaaaaa cagtctatgt
      481 ttactgggga cgtacaggaa ccggtaagag tagacgtgcc tgggatgagg ccactctggt
      541 tgcttatcct aaagacccat gtacaaaatg gtggtgcgga tatcgagggc aatcaacagt
      601 tgttatcgat gaatttagag gtaggattgg tattgagcat ttgttgcgat ggacagacag
      661 atatcctgtc gttgtggaaa gcaaaggagg agctgttgtg ctcagatgta ctagtatatg
      721 gattacctca aatctttccc ctgatgtgtg gtatcccgat atagatgctg aaagtcttgc
      781 ggctttgaga cgaagattaa atataatcca ttttaattac gttgttttcc tgtagcttag
      841 taagtttgcc gcaaaatatt ataatatcca agtagaatat tttgcggcga acctaaccct
      901 aaccctaacc ctaaccctaa ccctaaccct aaaacgtgac tttattaaaa cttcctttaa
      961 aaaaagagga ggccttggac tccgataagg acttgctaaa atgaaaatgt aagcattttg
     1021 taataacttt attattaccc taataacgaa gaatcaattt gtgtcaccaa aatgttgcaa
     1081 actactgtgc ctgtgggtaa ggtgccggct gcccctactg tcatagttac aacccctaag
     1141 gatgcattgt taactcgaat tgacaagaat tgcatataac gtgccgtaga ggttcctgga
     1201 acaggtactc ctgtcgcaac accgtaaaag acggtgttga gagtcgcgtt agtgaatgta
     1261 ataattgggt caacgatcac tgcagctaca ctaccaatcc acgtaacatt gaaaaagagt
     1321 agagttccat cctgtagccc actcagggtc agggcagtgt ttgtgataga aaagggcaaa
     1381 tcccctcgcc cgtaatttgt acctgttcct agaggtagcg cgttggttat gccagttcgc
     1441 tcaatcgcaa ggccttgcac agttccacct atggttgttg caacttgggg tttgaagaat
     1501 tcgatggtgt aacttaccca taactcgcct acgtttacgt tcgccgcttg gaatccctgt
     1561 gttgcgattt gaaaattggc aaaatcatac cacttgagac tgtcagttgc atttatggag
     1621 tcgcttcgaa cgaaatatag agctaccgat ctttctgcta catcacattc aatgccatga
     1681 acctgattcg cggacgcttt agcactctgc gcaaactctg cgttttccat ttgttgcttg
     1741 gtagtgtacg ccgtcgagtt aacattgtaa tccgtactca atactacagt tccaatggcg
     1801 gtgttagtcg agccgacgct atccccagag gtgcttttaa agtaaaagac cataccgtga
     1861 atgcggtatt gctcatagtg ctcggcaata gacgaaagcc aggggaacgt gattgcattg
     1921 cccggttgta tcgtgaatga ctggttcttg aaggctccaa tgacactgct tgttgtaata
     1981 tcatttacgt attcacgatg cgtaataatt gttcctcgac cgttgccaga aaacataggc
     2041 acatccgttg agttggtgag acaatttgaa cgaaccatcg gacccgtagt ataatcccct
     2101 gaaccaaata accgtcctac cccgtggccg agtgcctctc cggcgtctgc gacccaggaa
     2161 ggtaggccaa agaggcctgc cgctttgcct agctttctgc ctactctagc gtatggagta
     2221 tcgcccttat acgaatatgg gccgaggttg tagtcgccgg atcctgtcat gaccggtccc
     2281 atcggcatcc cgccgatttt ggccggtcgt ctcgggcccg agggttgtcc tctagccttg
     2341 cgcttgttcg acttgggttt tggtggccgt ggatagatac tacctgctat ggtgcatggc
     2401 gttcctttgg gcatataggg taaatattaa ggaggagtag atctataata acctagatgt
     2461 attttgatac atcttagtat tacttataga tcgacgtatc ctgtgtcact cttgggttcc
     2521 caaagaccct tacccttggt gccaaaaaag gacccttacc cgtataaaaa gggagtataa
     2581 aacaactttc ttatgtttga ttacgacgag ca
//
LOCUS       CruV-86                 2621 bp    DNA     circular     24-FEB-2020
DEFINITION  Crucivirus-86 isolate 120_812_IR1_3296.
ACCESSION   CruV-86
KEYWORDS    .
SOURCE      Crucivirus-86
  ORGANISM  Crucivirus-86
            Unclassified.
REFERENCE   1  (bases 1 to 2621)
  AUTHORS   de la Higuera,I., Kasun,G.W., Torrance,E.L., Pratt,A.A.,
            Maluenda,A., Colombet,J., Bisseux,M., Ravet,V., Dayaram,A.,
            Stainton,D., Kraberger,S., Zawar-Reza,P., Goldstien,S., Briskie,J.,
            White,R., Taylor,H., Gomez,C., Ainley,D.G., Harding,J.S.,
            Fontenele,R.S., Schreck,J., Ribeiro,S.G., Oswald,S.A., Arnold,J.M.,
            Varsani,A., Enault,F. and Stedman,K.M.
  TITLE     Direct Submission
  JOURNAL   Submitted (24-FEB-2020) Biology, Portland State University, 1719 SW
            10th Ave, Portland, Oregon 97201, USA
COMMENT     Bankit Comment: ALT EMAIL:ide@pdx.edu
            Bankit Comment: TOTAL # OF SEQS:451
            Bankit Comment: GAP: est. length - alignment

            ##Assembly-Data-START##
            Assembly Method       :: MetaSPAdes v. 3.12; IDBA-UD v. 1.1.1;
                                     MEGAHIT v. 1.0.3
            Sequencing Technology :: 454; Illumina
            ##Assembly-Data-END##
FEATURES             Location/Qualifiers
     source          1..2621
                     /organism="Crucivirus-86"
                     /mol_type="genomic DNA"
                     /isolate="120_812_IR1_3296"
                     /isolation_source="Water"
                     /country="Norway"
                     /collection_date=2011
     misc_feature    join(2612..2621,1..16)
                     /note="stem-loop"
     misc_feature    1..9
                     /note="nonanucleotide"
     CDS             90..989
                     /codon_start=1
                     /product="Putative Replication Associated Protein"
                     /translation="MSSRFRAWTFTINNYSWKDMCLLMDGEFKYLVFGFEVGGKKNVP
                     HIQGYIYLYNAMTMTALAKSILPRAHLIPSKGTVENNANYCSKDGEWYEFGDKPEQGK
                     AQWEKIEDVMKDPRSNPHLFNQYNKMYRQLTLTSPKEHTRRLIAIPTCDKFTHAKACE
                     CPVSFDTSLETYDGESVVFLRAYSQNEFILEWINGFPSKIRRGFEIICVDPMVIYLMY
                     DNLTEKSYIVRTYSDYLEDVIAILPEKTTMNDSEYSFEETNTEETVKKYKISIKKDRS
                     NTNDNLGFLIEESDFYTTSGGEE*"
     CDS             1053..2360
                     /codon_start=1
                     /product="Putative CruV Capsid Protein"
                     /translation="MPSKTSRTKPKPKTKTPVVVVQAPVRRKPRRSINKPKTTPADTN
                     YEYQNSIGGKIGAYLGHGIGRVVSHLAGFGDYQIEQNSLMDATLGGDPPIVRNSPNGM
                     IIRHREYIADIYAAIPFTIDTYPIQPGLLSTFPWLSQIADSFEQYRFRGLVFEYKSMS
                     SDAILSASSSSALGTVIMSTQYNSNETVFVDKRTMENYEYSNSCKPSVDMYHPVECKN
                     SETSISELYTRNGPAVGDLRLYDLGNFSIAVQGMQNALTENQVVGELWATFEIELFKP
                     KLLANGGYDSLTDHFRILQATFADPILEAVATSGSNLGCDISGHPGFVQFPPNITSGI
                     YLLTYYVEGTSTTANSGGILITPVYNIQVYTVLQNGTTNTELAPAAGSTTTRLMFQIT
                     FRIIQTTPGIPATFQLSGINGSIWPIAPVTGDMIITLLNIGFD*"
ORIGIN
        1 tactattacc ccagaccggt tctgtgcaat tccttgtgaa atatcatttc taacttctgt
       61 gcaaatttta atttctaata tagtaatata tgtcttcacg atttagagcc tggactttca
      121 caatcaataa ctattcctgg aaggatatgt gtttattgat ggatggtgaa tttaaatacc
      181 tagtatttgg ttttgaagtt ggaggtaaaa agaatgttcc tcatatacaa ggttatatat
      241 atctttataa tgcaatgaca atgacagctt tagcaaagag catattacca agagcccact
      301 taataccatc gaaaggaact gttgaaaata atgcaaatta ttgttcaaag gatggagaat
      361 ggtatgaatt tggcgataaa cccgaacaag ggaaagccca atgggaaaaa atagaggatg
      421 taatgaagga cccccgaagt aatccgcact tatttaatca atataataag atgtatcgac
      481 agttaacatt aaccagtcca aaagaacata cacgtagatt gatcgcaata ccaacttgtg
      541 ataaatttac acacgctaaa gcttgtgaat gtcctgtttc ttttgatact tcacttgaaa
      601 catacgatgg tgaaagtgta gtatttctac gagcatattc ccaaaacgag tttatattag
      661 agtggataaa tggttttcca tctaagataa gacgaggttt tgaaataata tgcgtcgatc
      721 ctatggttat atatttaatg tacgacaatt taaccgaaaa gagttacatt gttaggacat
      781 attctgatta tctcgaggat gtcatagcga ttttgcctga aaaaacgact atgaatgatt
      841 ccgagtattc tttcgaagaa actaatacag aagaaactgt taagaaatat aagatttcca
      901 ttaaaaaaga tagatcgaat acaaatgata atctaggatt tttaatagaa gaatctgatt
      961 tctatacaac ttctggagga gaagaataat actcttaaaa attacattta tgtataaata
     1021 ataaatatat aatctttatt atctatataa aaatgccaag caaaacttct cgcactaaac
     1081 caaaaccaaa gactaagacg ccagtagtcg ttgtacaggc tccagttaga cgtaagccac
     1141 gccgatccat aaacaaacct aagacaactc ctgccgatac gaattatgag tatcagaata
     1201 gtataggcgg gaaaataggt gcttatttgg gtcatggtat aggacgtgtt gtgtcacatt
     1261 tagcaggttt tggtgattac caaatcgaac aaaactcatt aatggatgca accttaggag
     1321 gtgatccacc tatcgtccgt aattcaccca atgggatgat aatacgtcat agggaatata
     1381 ttgcggacat ttatgcagca ataccattca ctatagatac gtatcctatt caacctggtt
     1441 tactaagtac atttccatgg ttatcgcaaa tagcggacag ctttgagcaa tatcgcttta
     1501 gaggattagt atttgagtac aaatctatgt catctgatgc aatattatca gcgagtagca
     1561 gtagcgcgtt aggtacggta atcatgagta cacaatacaa tagtaatgaa acagtgttcg
     1621 ttgataaacg aactatggag aattacgagt attcaaattc ttgtaaacca tctgttgata
     1681 tgtatcatcc tgtagaatgt aaaaacagcg agacgtctat ttctgagttg tataccagaa
     1741 atggaccagc tgttggtgac cttcgattgt acgatttagg taattttagt attgcagtcc
     1801 aaggaatgca aaacgcactt accgaaaatc aagtagtagg tgaattatgg gcaacatttg
     1861 agattgaatt atttaagcca aagttacttg ccaatggagg atatgattcg cttacggatc
     1921 atttcaggat tttgcaagca acttttgctg atccaatact ggaagcagtt gcgacttctg
     1981 gaagtaattt aggatgtgat ataagtggac acccagggtt tgtccagttt ccgccaaata
     2041 ttacgagtgg tatatatttg ttaacatatt atgttgaagg tacctcgaca acagctaatt
     2101 ctggtggtat attaataacc ccggtatata atatacaagt ttacacagta ctgcagaatg
     2161 gtacaacgaa tactgagttg gcaccagcag caggatcaac tacaaccaga cttatgtttc
     2221 agattacatt ccgtattatt caaactacgc caggtatacc agctacattc caattaagtg
     2281 gaattaatgg ttcaatctgg cctatagctc cagttactgg tgatatgata attacacttt
     2341 tgaacatagg attcgattag taaatgatat acccgctggt ccatcaagcg agctcgtgat
     2401 aaaaaaaggt ttaccttttt ttagacacgg cgacgaatgg agcgggtcca gggttgaaaa
     2461 ccatggtcaa ggagcgcaaa gcctgcgact gcgcgggctg aaagcccagc atacgataga
     2521 tctatctaat atagtttttc ctaaaaattc tattagatat attttttgca caaaattttt
     2581 ttataaacta tagaaatatt ttttcctaaa agtctgggtc a
//
LOCUS       CruV-87                 2667 bp    DNA     circular     24-FEB-2020
DEFINITION  Crucivirus-87 isolate 120_1319_SvL1_3295.
ACCESSION   CruV-87
KEYWORDS    .
SOURCE      Crucivirus-87
  ORGANISM  Crucivirus-87
            Unclassified.
REFERENCE   1  (bases 1 to 2667)
  AUTHORS   de la Higuera,I., Kasun,G.W., Torrance,E.L., Pratt,A.A.,
            Maluenda,A., Colombet,J., Bisseux,M., Ravet,V., Dayaram,A.,
            Stainton,D., Kraberger,S., Zawar-Reza,P., Goldstien,S., Briskie,J.,
            White,R., Taylor,H., Gomez,C., Ainley,D.G., Harding,J.S.,
            Fontenele,R.S., Schreck,J., Ribeiro,S.G., Oswald,S.A., Arnold,J.M.,
            Varsani,A., Enault,F. and Stedman,K.M.
  TITLE     Direct Submission
  JOURNAL   Submitted (24-FEB-2020) Biology, Portland State University, 1719 SW
            10th Ave, Portland, Oregon 97201, USA
COMMENT     Bankit Comment: ALT EMAIL:ide@pdx.edu
            Bankit Comment: TOTAL # OF SEQS:451
            Bankit Comment: GAP: est. length - alignment

            ##Assembly-Data-START##
            Assembly Method       :: MetaSPAdes v. 3.12; IDBA-UD v. 1.1.1;
                                     MEGAHIT v. 1.0.3
            Sequencing Technology :: 454; Illumina
            ##Assembly-Data-END##
FEATURES             Location/Qualifiers
     source          1..2667
                     /organism="Crucivirus-87"
                     /mol_type="genomic DNA"
                     /isolate="120_1319_SvL1_3295"
                     /isolation_source="Water"
                     /country="Norway"
                     /collection_date=2012
     CDS             1..1074
                     /codon_start=1
                     /product="Putative Replication Associated Protein"
                     /translation="MCFFSNTHLDVKYTPFYFCYNYIMNRCCVHDFTSSCETGPNWDT
                     CIAWLKENSKRWCFQKERGASGYTHFQGRFSLISKQKMRDIIHPFSHLSVTSKENYLN
                     MFYVLKDDTRVDGPWQDTDVYVPKSCKDTALLPFQKHIVEHRDDDRTINCIIDPKGGK
                     GKTHVCRYIDATKRGVLITFTKDYKDLMRCVFNQDPSLLYLVDIPRGMKKDKLDELFA
                     ALETLKDGLSFDDRYKYRKRWMEPPTIWVFTNTIPNLRWLSSDRWKLWSICPSTNVLL
                     PKVVLGTENTQVLPSTSSQEGFSGGSSSTPCNTSINRFTLNTLEKPGLVGRELDSVRN
                     SSIRLDDVPIDNIEDDYFDLEST*"
     CDS             complement(1280..2659)
                     /codon_start=1
                     /product="Putative CruV Capsid Protein"
                     /translation="MFAILMSTRSRKSTSRKQYTDAEKRAYYAKMARLAEGKLKVMGS
                     GKYRIGRPYLRGQGNYNFNAPKFGSMLGSAGGAAIGNMIAPGIGGKIGGEFGSGIGGE
                     LGKLFKNITGFGDYTIRQNSLLFPDQIVPSFGEDSIRVKKREYITDINATTVFTNNSF
                     PINPGLSDTFPWLSSIANNFEQYRFNGLIFQFVSTSSDAIASTTDLGLGQVILAADYN
                     SADAAYVNAPQMLGAMFSNSGKPSENILHAIECAPTDQAQKLYYIRSGDVPANTDIRL
                     YDMASFQIATQKMPAPYDGCGQLWVSYDVTFCKSVQNNQLGFDLNTDKYQLVGQAANI
                     PFGTSRVLAEHSNLGTTVTNTVISFPPTLSTGYFLLVWGCYGTPDPTTLPTETLTNCA
                     LVTGWDNNVSATISNGGETTSTYVIVKLYRLTDRDATIAWAGPSLLPAAITSGDLLIT
                     QINGEIFLP*"
ORIGIN
        1 ttgtgttttt tttctaacac tcacttagat gtaaaatata ctccgtttta cttttgttac
       61 aactacataa tgaatcgctg ttgcgttcac gattttacat ctagctgcga aaccggtcct
      121 aattgggaca cttgtattgc ttggcttaaa gaaaattcta aacgttggtg ttttcaaaaa
      181 gaacgtggtg cctccggcta cactcacttt caaggtcgct tttctttaat ttccaagcaa
      241 aaaatgcggg acatcataca tcccttttct cacctttccg ttacctctaa agaaaattat
      301 ttgaatatgt tttatgtatt aaaagatgac actagggtcg atggtccttg gcaggacact
      361 gatgtttatg ttcctaaaag ttgtaaagac actgctctgc ttccgttcca gaagcacatt
      421 gttgaacata gagatgatga cagaactata aactgcatca tcgaccctaa aggtggaaaa
      481 ggtaaaacgc acgtatgtag atacattgat gctacaaaac gtggtgtgct cattacattc
      541 actaaagatt acaaagatct catgcgttgc gttttcaacc aagatccatc tcttttgtac
      601 ctagtcgaca ttccccgcgg aatgaagaag gataaattgg atgaactgtt tgctgcgctt
      661 gaaaccctaa aggatggttt atccttcgac gacagataca aataccgtaa acggtggatg
      721 gaacctccaa ccatctgggt gttcacgaac acaataccca accttcgttg gttatcttcc
      781 gacagatgga aactttggtc catttgccct tcgacaaatg ttttgctgcc caaagttgta
      841 cttgggaccg agaatacgca agtcctccct tcgacttcgt ctcaggaagg tttttctggc
      901 ggttcgtctt cgactccctg taacactagt attaatcgtt tcactcttaa tactttagaa
      961 aaacctggac ttgtcggacg tgagttggac tcagtccgga attcatcgat tcggttggac
     1021 gatgtaccga ttgataacat agaagatgac tattttgatt tggaatccac ataatttaac
     1081 gatattctta ttggtaattc gtaattcttc attacgaatt gttaacttat accactcggc
     1141 gctccgcttc gctccgctcc ttgcctcgtg gagggtggcc gtctgacata tgtatctttg
     1201 ctcgggacgc aaagatacaa acccactcaa ccaccctctt ccccttcggg gaattcacca
     1261 acggttagaa aggcagcttt tatggtaaga atatctctcc gttaatttgt gtgataagta
     1321 aatcaccgct cgtgattgct gctggtaaca aagatgggcc agcccatgcg attgttgcat
     1381 ccctatcggt taatctgtat aatttaacta tcacatatgt tgaagttgtt tctcctccgt
     1441 tcgaaattgt tgcacttacg ttgttatccc atcccgtcac caatgcgcag ttcgtcaacg
     1501 tttctgttgg cagggttgtt ggatctgggg ttccgtaaca tccccacacc aatagaaagt
     1561 atcctgtgct taatgtgggt gggaatgaga taacggtgtt tgtaactgtt gtacctaaat
     1621 tactgtgctc tgcaagcact cttgatgtac cgaacgggat gttagcagcc tgtccaacaa
     1681 gttggtactt atctgtattt aaatcaaatc ccagctgatt attctgtaca cttttacaaa
     1741 atgttacatc ataactgacc caaagttggc cacagccatc atagggtgct ggcatcttct
     1801 gtgttgcaat ctgaaatgat gccatatcgt atagtctaat atctgtattt gcgggtacat
     1861 caccactacg gatatagtac agcttttgtg cctggtctgt gggtgcacat tctattgcgt
     1921 gcaatatatt ctccgatggt tttccactgt tggaaaacat cgctcctagc atctgcggcg
     1981 cgttcacgta tgctgcgtcg gccgaattat agtctgctgc taatataact tgacccagac
     2041 ctaaatccgt tgttgatgca attgcgtctg aagacgtcga aacaaattgg aatatcagtc
     2101 cgttgaaccg atattgctca aaattgtttg caattgatga cagccaagga aatgtatcgg
     2161 acagtcctgg attaattgga aacgagttgt ttgtaaacac cgttgtggcg tttatgtcgg
     2221 tgatatattc tcgctttttc actcgaatac tgtcttcacc gaaactcggt acaatttgat
     2281 ctgggaacag taatgaattt tgtcggatag tgtaatcgcc aaaacctgtt atgtttttga
     2341 agagctttcc tagctctccg ccaattccac ttccaaattc acctccaatt ttaccaccta
     2401 tacctggtgc tatcatattg cctattgctg ctcctcctgc ggatccgagc atgcttccaa
     2461 atttgggggc gttaaaattg taatttcctt gtcctctcaa atacggacga cctatacgat
     2521 atttcccgct tcccattacc ttcaactttc cctctgccaa tcttgccatt ttggcatagt
     2581 atgctcgctt ttcagcgtcg gtatactgtt ttcgtgaagt tgatttcctg cttcgtgttg
     2641 acattaatat agcaaacatc ttttttt
//
LOCUS       CruV-88                 2671 bp    DNA     circular     26-FEB-2020
DEFINITION  Crucivirus-88 isolate TM03_45.
ACCESSION   CruV-88
KEYWORDS    .
SOURCE      Crucivirus-88
  ORGANISM  Crucivirus-88
            Unclassified.
REFERENCE   1  (bases 1 to 2671)
  AUTHORS   de la Higuera,I., Kasun,G.W., Torrance,E.L., Pratt,A.A.,
            Maluenda,A., Colombet,J., Bisseux,M., Ravet,V., Dayaram,A.,
            Stainton,D., Kraberger,S., Zawar-Reza,P., Goldstien,S., Briskie,J.,
            White,R., Taylor,H., Gomez,C., Ainley,D.G., Harding,J.S.,
            Fontenele,R.S., Schreck,J., Ribeiro,S.G., Oswald,S.A., Arnold,J.M.,
            Varsani,A., Enault,F. and Stedman,K.M.
  TITLE     Direct Submission
  JOURNAL   Submitted (24-FEB-2020) Biology, Portland State University, 1719 SW
            10th Ave, Portland, Oregon 97201, USA
COMMENT     Bankit Comment: ALT EMAIL:ide@pdx.edu
            Bankit Comment: TOTAL # OF SEQS:451
            Bankit Comment: GAP: est. length - alignment

            ##Assembly-Data-START##
            Assembly Method       :: MetaSPAdes v. 3.12; IDBA-UD v. 1.1.1;
                                     MEGAHIT v. 1.0.3
            Sequencing Technology :: 454; Illumina
            ##Assembly-Data-END##
FEATURES             Location/Qualifiers
     source          1..2671
                     /organism="Crucivirus-88"
                     /mol_type="genomic DNA"
                     /isolate="TM03_45"
                     /isolation_source="Green lipped muscles"
                     /country="New Zealand"
                     /collection_date=2014
     CDS             1..1032
                     /codon_start=1
                     /product="Putative CruV Capsid Protein"
                     /translation="MSTRKTYKRNGYRTRRQTARRTYYSPRYERKAYAPLPRAAPAKK
                     VNDNVLGTVGGAIGSAYGPVGTVLGTVGGSLLGDFLGIGDYTEMPNSSHNISTVETNS
                     LIKDRLPKQLPPFMNGKEPYIIVHHREYCFDVLSHIGFVGVVANINPTDTKLFPWLKN
                     VAMNYEKYKVLGMVVEYVSLSGESSSLTVPTLGSVMMAHQTNNYDPVFPDKQHMLNHR
                     GAISSSPNKNMMLGIECDPEYLFAENKFIRHADQIVGGDNRVYDFGSVSVFCEGQNQI
                     GSIIGEIWCSYQIALINPRVNTSAFETPEFLSPSDINEKKKNKIEEDYVISSGKSSKT
                     HSRAHSPRR*"
     CDS             complement(1553..2512)
                     /codon_start=1
                     /product="Putative Replication Associated Protein"
                     /translation="MTDEIVVSVIGRPEKHTGGGNTNTPPLYSDSEYSVLETQDLKTQ
                     KHTKNRKWIFTINNYSDEEVKNLKNIFEKNNRKYIMGYEVAPTTGTPHIQGYMESKNA
                     VTFTTMKGYNGRMKLIMAKGSIIQNYNYCSKEGNFITNITDEDLMPKGRKSKKKKENE
                     LWLEQYKNDILDEEYGEDIKWKDWQKEIINILDSKPDKRSIYWYYSEDGNKGKSYLCK
                     YLTLTRKVIICSGKQADIFNQVKLCLDEMIAPEIILLDIPRTNNEYVNYSAIESLKNG
                     CLYSGKYEGGQVLIKTPHIIAFSNELPNIKAMSSDRWKIKEIL*"
     misc_feature    2435..2462
                     /note="stem-loop"
     misc_feature    2446..2454
                     /note="nonanucleotide"
ORIGIN
        1 atgtcaaccc gaaaaacata caaacggaac ggttatagaa ctcgtcgcca gacggccagg
       61 cgaacttatt attctcctcg ttatgaacga aaagcttatg cacctctacc tagagccgct
      121 ccagctaaaa aagttaatga taatgtttta ggcactgttg gcggtgccat tggttccgcc
      181 tatggtcctg tcggtaccgt tttaggaacg gttggcggtt ctctattagg tgattttcta
      241 ggaattggcg attacactga aatgcctaat tcttcacata atatatctac tgttgaaact
      301 aatagcctta taaaggatag gcttcctaag caattaccgc cttttatgaa tggaaaagag
      361 ccgtatatta tagtacacca tagggaatat tgttttgatg tattaagcca tattggcttt
      421 gttggtgttg tcgcgaatat taatcctacc gatactaaac tttttccttg gttaaaaaat
      481 gttgctatga attatgaaaa atataaggtc cttggtatgg tcgtggaata tgtaagttta
      541 tctggcgaat catcaagttt aaccgtgcct actttaggca gtgttatgat ggcgcaccaa
      601 actaataatt acgaccctgt ttttcctgac aagcagcata tgttaaatca tagaggtgca
      661 atttcttcct cccctaataa aaatatgatg ttaggcattg aatgcgaccc tgaatatctt
      721 tttgctgaaa ataaatttat tagacatgct gaccagattg tcggtggtga caatcgtgta
      781 tatgattttg gaagtgtatc tgtattttgt gagggtcaaa atcaaatcgg cagcattatc
      841 ggcgaaatat ggtgctctta tcaaattgct ttaattaatc cccgggttaa tacctcggca
      901 ttcgaaactc cagagtttct ctctccttct gatattaatg aaaaaaaaaa aaataaaatc
      961 gaagaagatt atgtcatctc ttcaggtaaa tccagtaaaa cccattcaag agcccatagt
     1021 ccccgtcgct aataatattc ctctcgcctc tgaatataat attccagata tagaaattta
     1081 tgatattaca ttacctccta agctttctga ttcttcttat atttcttatt ttattccctc
     1141 cttcatattt tcttaaatat tccgttgttc ggattttccg agcagctgat tctttcatat
     1201 aaatataaaa aaaattaata ttgatttaga ttaatatttt ttttctcgcc ctattactta
     1261 cttatatatt aaaacaaaaa acatataaaa aaaaaataat aataatttaa aactattttt
     1321 ttctcgccct attatttact tatatataaa aacaaaaaac atataaaaaa aaaattaata
     1381 ttttttctta gcttatttag agcttactat tattagaggg tattatcata atctacactc
     1441 gccccggcaa cattgcccca agggggcagt gttgggttgt gataacaacc cggtccacta
     1501 cttaagagcc gtaagtgagc gacggagtcg tgaatctcgg cggtccacta acttataaaa
     1561 tctcttttat cttccatctg tcgctagaca ttgctttaat gttaggtaat tcattactaa
     1621 aagctataat atgtggtgtt ttaattaaaa cttgccctcc ttcatatttg ccactatata
     1681 gacaaccatt ttttaaactt tcaatagcac tataatttac atattcgtta ttagtccttg
     1741 gtatatctaa taatattatt tctggtgcta tcatttcgtc taaacataat ttaacttgat
     1801 tgaatatatc cgcctgttta ccagaacata taataacttt tcttgtaagt gttaagtatt
     1861 tacataaata gctttttcct ttgtttccat cttctgaata ataccagtaa atactccttt
     1921 tatctggttt actatctaat atatttatta tctctttttg ccaatctttc catttaatat
     1981 cttctccata ttcttcatct aaaatatcat ttttatattg ttcaagccat aattcatttt
     2041 cttttttttt tttacttttt ctacccttag gcattaaatc ttcatctgta atattagtta
     2101 taaaattacc ttctttagaa caataattat aattttgtat tatacttcct ttagccataa
     2161 ttagtttcat tcttccattg taacctttca ttgttgtaaa tgtaacggca tttttacttt
     2221 ccatatatcc ttgtatatga ggggtcccag ttgttggggc gacttcataa cccataatat
     2281 attttctatt atttttttca aaaatatttt ttaaattttt tacttcttcg tctgaataat
     2341 tattaatagt aaaaatccat tttctatttt tagtgtgttt ttgtgttttt aaatcttgtg
     2401 tttctaaaac actatattct gagtcagaat ataacggggg agtattagta ttacctcccc
     2461 cggtgtgttt ttcgggtctt ccgataacag aaactacgat ttcatcagtc atttttagta
     2521 agtttatcac aatttataat ttgtgttttt tgtgtttttt tgtgtttttg tgtttttata
     2581 ttatacaata ttttttatct ttagaataaa ataattaatt attttaattt ccttttaaac
     2641 ttatatctat atatatatat agaaaaaaag a
//
LOCUS       CruV-89                 2690 bp    DNA     circular     26-FEB-2020
DEFINITION  Crucivirus-89 isolate 120_765_IR1_3296.
ACCESSION   CruV-89
KEYWORDS    .
SOURCE      Crucivirus-89
  ORGANISM  Crucivirus-89
            Unclassified.
REFERENCE   1  (bases 1 to 2690)
  AUTHORS   de la Higuera,I., Kasun,G.W., Torrance,E.L., Pratt,A.A.,
            Maluenda,A., Colombet,J., Bisseux,M., Ravet,V., Dayaram,A.,
            Stainton,D., Kraberger,S., Zawar-Reza,P., Goldstien,S., Briskie,J.,
            White,R., Taylor,H., Gomez,C., Ainley,D.G., Harding,J.S.,
            Fontenele,R.S., Schreck,J., Ribeiro,S.G., Oswald,S.A., Arnold,J.M.,
            Varsani,A., Enault,F. and Stedman,K.M.
  TITLE     Direct Submission
  JOURNAL   Submitted (24-FEB-2020) Biology, Portland State University, 1719 SW
            10th Ave, Portland, Oregon 97201, USA
COMMENT     Bankit Comment: ALT EMAIL:ide@pdx.edu
            Bankit Comment: TOTAL # OF SEQS:451
            Bankit Comment: GAP: est. length - alignment

            ##Assembly-Data-START##
            Assembly Method       :: MetaSPAdes v. 3.12; IDBA-UD v. 1.1.1;
                                     MEGAHIT v. 1.0.3
            Sequencing Technology :: 454; Illumina
            ##Assembly-Data-END##
FEATURES             Location/Qualifiers
     source          1..2690
                     /organism="Crucivirus-89"
                     /mol_type="genomic DNA"
                     /isolate="120_765_IR1_3296"
                     /isolation_source="Water"
                     /country="Norway"
                     /collection_date=2011
     CDS             1..819
                     /codon_start=1
                     /product="Putative Replication Associated Protein"
                     /translation="MSHRYWIATIPKDDWTPPTTLEGTQIKYLRGQQENGESGYQHWQ
                     IYMETFSTCRLPRAKALLGIQSHMEPTRSTAAKDYVWKEHTAVMETRFELGSQGTRGK
                     NKVDWKKAMNLAIDGMAHHIDEGLQIRHFGNLMKIQAFHAKPAFRKDLAVNVYWGPTG
                     TGKSHNAWEEALDFGSVYVKQPSTKWWDGYQGEENVIIDEFCGEIGVTHLLRWLDKYP
                     CSVEVKGSQVPLKATRFWILSNIDPARWYSCPISQQEALARRLTNVVNKTEKYV*"
     CDS             847..2229
                     /created_by="nacho"
                     /modified_by="nacho"
                     /label="Putative CruV Capsid Protein"
ORIGIN
        1 atgtctcaca ggtattggat tgcaacgatt ccgaaggacg attggacacc tccaacaaca
       61 ttagagggga ctcaaatcaa atacctgaga ggacaacaag agaacgggga gtcgggatat
      121 caacattggc agatatacat ggagactttc agtacgtgcc gattaccccg cgcgaaagcc
      181 ctcctaggta tacagtccca tatggaacca acgcgctcaa cagcagcgaa agattacgtt
      241 tggaaagagc atacagccgt tatggaaaca cgtttcgaac ttggctctca gggaacgcgc
      301 gggaagaaca aagtcgattg gaagaaagca atgaatctcg cgatagatgg aatggcacac
      361 catatcgacg aagggcttca gattcgccat tttgggaatt tgatgaagat tcaagctttt
      421 cacgccaagc ctgcgtttcg gaaagacctg gccgttaacg tatattgggg ccctacaggt
      481 actggtaagt ctcataatgc ctgggaagaa gctcttgatt ttgggtctgt atacgtgaaa
      541 caacctagta ctaaatggtg ggatggatac cagggtgagg aaaatgttat catcgacgaa
      601 ttttgtggcg aaatcggtgt aactcattta ttaagatggc ttgataaata tccatgttca
      661 gttgaagtaa aaggttcaca ggttccgttg aaagctaccc ggttttggat tttatctaat
      721 attgatcctg cacgttggta tagttgtcct atttctcagc aggaagcttt agcgcgtcgt
      781 ctcactaatg tagtgaataa aactgagaaa tatgtttaaa aaaataattt tacaatacca
      841 taaaagatgc cacttgacct taaagggaaa ttttatggaa aggctgcgaa gacgttgcca
      901 aagtttggcc cgaagaattt gtcgcaggcc ggtcgtaaga aattggaact tgttagtgga
      961 aacaaacgag ctcggtctgc ttcgttttct cagagcgttt ctggtagcgg tgattatcgg
     1021 cttattaaaa atggcgttcg ggctaggacg ccatttggtg atgtaggtcg ccatcttgga
     1081 tcgtatattg gatcacctga tattggatat gctgctggtc atttagtcgg ttcagtactt
     1141 ggtagtggtg attatactat ggtgaatgcc aatagtccac ctaaatttaa ttccggaagg
     1201 atatccaata ttgtttgtca tcgtgagttt atacaagata taaccactgg aacagctaat
     1261 acattcacta accaagcttt tgctttaaat cctggtttag cccagacttt tccttggttg
     1321 gctgttgtcg cacagaatta tgaacaatac gaaattcatg gaatgatttt tgagtttaaa
     1381 actacttcaa gcgacgcgtt aaatagtacg aatacggcac ttggttctgt tatcatggcc
     1441 acttcctata actctcctga tcctattttc acaaataagc aggacatgga aaactatgaa
     1501 tatgctcaaa gtgctaaacc atctgtttct caattgcatg gtattgaatg tgcagctagt
     1561 caaacaccag tgaaacagtt atttgttagg agtggttcag taccctctgg gcaggatcag
     1621 aggtggtacg atatgggtgt ttttcagatt gcaacagctg gttttcaagc tagtaatgtt
     1681 gttgtaggag agttatgggt gacgtatgag atagaattct taaaaccacg aattcctcga
     1741 actattggtg gaaatgttca gtcatttcat ttgtacaata ctgctggtgt tgcacaagct
     1801 actccttttg gaacagcgat tgtgaccgat agttctaaat acaaaggaag tattagtttg
     1861 tctactaata ctgcctatac aaccgctact actttggcgt ttgataattt gcaaagtaat
     1921 tcccagtatt atatatacat aatttggggt tgtggatctg ctttattgac aactcctgct
     1981 gtgtctgcta ctggtgattg ggcgattagc tctaccggtt gtacttctag ggggggtgta
     2041 tatactgctt atgaaggtgg tcagaacggt aagactactg ataaaatgtc tgttggcgct
     2101 gtttttacta ctgattccat tcctaataca ggatatgggg gacttgtact tagtgcaaca
     2161 gctgtttttg gcacctcccc tgtttgtgat gtgttcattt tcaaaattga cgattctgta
     2221 acaaattaat aaacgattta accttcgact tggatcgagt cccaaactta cagctttggt
     2281 ggctcgattc aatctttacg atttaaccta atataatgta agggttaggg tttttataaa
     2341 accataaccc tggctatact cgattattaa cccctccccg tttctcttct agagaaacat
     2401 atttgagtac tacatccaaa ccctaataat aaggggtaaa tgtgtcaatt gtcatgaggt
     2461 agtaacactt cagggtgtgt gtcaaccacc ctattattac ttactacctc attgacactt
     2521 gacacatttc gtttaattga cacaaatccc ctttacccct tatttcattt aacccctgtg
     2581 gctaacccta agccacatta tattaaatat tatattaaat ttaatataaa cagccacatc
     2641 ttttacataa atagcctgga ttttgtttac tttttttaaa aattttatgc
//
LOCUS       CruV-90                 2702 bp    DNA     circular     24-FEB-2020
DEFINITION  Crucivirus-90 isolate 120_756_IR1_3296.
ACCESSION   CruV-90
KEYWORDS    .
SOURCE      Crucivirus-90
  ORGANISM  Crucivirus-90
            Unclassified.
REFERENCE   1  (bases 1 to 2702)
  AUTHORS   de la Higuera,I., Kasun,G.W., Torrance,E.L., Pratt,A.A.,
            Maluenda,A., Colombet,J., Bisseux,M., Ravet,V., Dayaram,A.,
            Stainton,D., Kraberger,S., Zawar-Reza,P., Goldstien,S., Briskie,J.,
            White,R., Taylor,H., Gomez,C., Ainley,D.G., Harding,J.S.,
            Fontenele,R.S., Schreck,J., Ribeiro,S.G., Oswald,S.A., Arnold,J.M.,
            Varsani,A., Enault,F. and Stedman,K.M.
  TITLE     Direct Submission
  JOURNAL   Submitted (24-FEB-2020) Biology, Portland State University, 1719 SW
            10th Ave, Portland, Oregon 97201, USA
COMMENT     Bankit Comment: ALT EMAIL:ide@pdx.edu
            Bankit Comment: TOTAL # OF SEQS:451
            Bankit Comment: GAP: est. length - alignment

            ##Assembly-Data-START##
            Assembly Method       :: MetaSPAdes v. 3.12; IDBA-UD v. 1.1.1;
                                     MEGAHIT v. 1.0.3
            Sequencing Technology :: 454; Illumina
            ##Assembly-Data-END##
FEATURES             Location/Qualifiers
     source          1..2702
                     /organism="Crucivirus-90"
                     /mol_type="genomic DNA"
                     /isolate="120_756_IR1_3296"
                     /isolation_source="Water"
                     /country="Norway"
                     /collection_date=2011
     CDS             1..1080
                     /codon_start=1
                     /product="Putative Replication Associated Protein"
                     /translation="MDSTKKFRFAGVKMLLTYPGHLNKPWLIDLLTIASNQPIKFIRC
                     AHEIGKTTGVDYDHTHCLIRWVNQLTTTDCRRFDVVLDGNTIHPNIAPIRTETHWRNQ
                     IAYIAKEDPDNSDLIVRSCVPKIWGAESLADALADNVKKPADTLGVIALYNARPAAEI
                     QVDTPDRPWQLEVLDFVQTKPDRRSVHWYYDPKGGAGKTWLARYMMANKLAYMVKQCG
                     GSSNFATIMAGAVASGWDQRCFILDLPRSAEDYKFYGCLEEICDGCVTATKYQGGTIL
                     FNQPHVLVFANFLPNRSAMSADRWVVHWLPQIEESIKAPPLQAAPGLGGLTLITLTEF
                     QPPKITRCDNVLSQEDIEEVLNDLL*"
     CDS             complement(1100..2467)
                     /codon_start=1
                     /product="Putative CruV Capsid Protein"
                     /translation="MPTTYYTQSGRPYTIVNGRRKYGAAVAAPRQLTGFGAYKTPEQY
                     AAKKQRKRVAAGAVVTVPRSAAYRLPPAVSEPDPWVEWGSKGGKAVAGFAKGILGLGD
                     YHVKQNVLMGRLPQVVNLPRGGGTIIRFCEYLGDITTSSSANTFAVSSFDINAGLFST
                     FPWLSQIAQNYEQYSFEGLLFEFRSTSADALNSTNTALGSVIMATNYDSSDPAFLSKG
                     EMLNYEFSTSIKPAESAMHMVECAPRQTVLTELYTRSAAVPAEDDQRFYDLGKFQIAT
                     AGFQGTSVVVGELHCTYQVRLLKPKLHQSLGNSISFAQMGNSTGVAAGTPLGTTTGWS
                     SVASVQTLAIVFTSSTVITFPVTNVIQQYLLRYGIQGTIAGALVYPVLTAANMTIGTG
                     TLQTATFPVAATAATSGVMHVWLKTTPNGLAPTLTFGVGGTFPTGTTSCAIGIIQVPD
                     AVTIT*"
ORIGIN
        1 atggatagca ccaagaagtt ccggttcgca ggtgtcaaga tgctcttgac ataccctggc
       61 cacctcaata agccatggct tattgacctc ttgactatag catccaacca accgatcaaa
      121 ttcatccgct gcgcgcacga aatcggtaaa actacaggtg tggactacga ccacacccac
      181 tgcctgatcc gatgggtcaa ccagctaaca accaccgact gccgtcggtt cgacgttgtt
      241 ctggatggta acaccatcca ccccaacata gctcccattc gcacagagac tcactggcgt
      301 aaccagatcg cctacattgc gaaggaagac cctgacaaca gcgacctaat cgtcagatct
      361 tgcgtaccta agatctgggg cgccgagtcc ctcgctgacg ccctcgctga caatgtcaaa
      421 aaaccggccg acacccttgg ggtgatcgcc ctatacaatg ctcgtcctgc ggccgagata
      481 caagtagata ctccagaccg accctggcag ctggaagtct tggacttcgt ccaaactaaa
      541 ccagaccgca ggtcggtaca ctggtactac gaccccaaag ggggtgcggg caagacatgg
      601 ctagcccgct acatgatggc taataaatta gcttacatgg taaaacaatg tggcggctcg
      661 tcaaacttcg ccaccatcat ggccggagcc gtcgcctcgg ggtgggacca gcgatgcttc
      721 atcttggacc tcccccgctc ggcggaagac tataaattct acggctgcct cgaggaaata
      781 tgcgatggct gtgtcacagc cactaaatac caaggtggta ctatcctctt caaccagcct
      841 cacgtactcg tcttcgctaa ctttctaccg aaccggagcg ccatgtcggc cgaccgatgg
      901 gtcgtccact ggttacctca aatcgaagaa agtataaaag cccctccgct gcaagcagct
      961 ccggggcttg ggggcttaac attaataact cttacagagt ttcagccccc aaaaataact
     1021 cgctgtgata acgtgttatc ccaagaggac atcgaagaag tcctcaatga ccttctctaa
     1081 ggtcgaaacc ttcttttgtt tacgtaatcg taacagcatc cggaacctgt ataatcccta
     1141 tcgcacaaga agttgtcccc gtcgggaacg taccaccaac accaaaggta agcgttggtg
     1201 caagaccatt cggagttgtt ttcaaccaca catgcatcac accagaagta gccgcagtcg
     1261 cggcaaccgg aaaggtcgct gtttgcagtg ttcccgtgcc aattgtcata ttggcagccg
     1321 taagaacagg gtacacaagc gctcccgcga tagttccctg aataccgtac ctcaagaggt
     1381 actgctgaat cacattcgta accgggaaag ttatcaccgt cgacgatgtg aacacaatag
     1441 caagcgtttg cacactcgcc acagacgacc acccagttgt cgtccccaac ggagtaccag
     1501 ctgctacacc cgtagaattg cccatttggg caaaggaaat actattccca agagattgat
     1561 gaagcttcgg cttcaacagc cgcacctggt aagtgcaatg aagctcacca acaacgacac
     1621 tggttccctg gaacccagcg gtcgcgatct gaaattttcc aagatcgtag aaacgctgat
     1681 catcttccgc cggaactgcc gccgaacgag tatacagctc agtcagaaca gtctgacgtg
     1741 gagcacactc caccatatgc atcgcgctct cagctggctt aatgcttgtg ctgaactcat
     1801 agttcagcat ctctcccttc gagaggaaag caggatcact actatcgtag ttcgtagcca
     1861 taataacact cccaagagca gtgtttgtag agttaagagc atccgcagaa gtactgcgaa
     1921 actcaaacaa gagaccctca aagctgtact gctcataatt ctgagcgatc tgcgacaacc
     1981 aaggaaaggt cgagaagaga ccagcattga tgtcaaagga cgacaccgcg aacgtattcg
     2041 cagacgatga cgtcgtgata tctccaagat actcacagaa ccgtataatc gtcccacctc
     2101 cacgaggaag gttaaccacc tgaggaagcc gtcccataag gacattctgc ttcacatgat
     2161 aatcaccaag gccaagtatg cccttagcaa acccggcaac agccttacca cccttacttc
     2221 cccattcgac ccaagggtca ggctcagaca cggctggagg aagacgatac gcagccgatc
     2281 gaggtactgt cacaacagca ccagcggcta ctcgtttccg ctgcttttta gcagcatact
     2341 gctccggcgt tttatatgct ccaaaacccg taagctgtct aggcgccgcc acagcggcac
     2401 catacttacg tcgcccgttc acaatcgtgt acggtctacc tgactgagtg taatatgttg
     2461 taggcatagg agtaccccat acctaccccg ctctaataga gcgcaccatt ctatacaccg
     2521 gacccatgcc tcgaggccca gacccgcacg tctcgtgaca ttcgggccgg gacaagcccg
     2581 gcccggccag ggcacattcg agccgacagg tcttaagagc tctgtggtgg gtcctcacac
     2641 catcccctgg aattccagga gatgccaatt agagaacaca aggttcctaa caagagagat
     2701 ag
//
LOCUS       CruV-91                 2702 bp    DNA     circular     24-FEB-2020
DEFINITION  Crucivirus-91 isolate 120_2087_IR2_3297.
ACCESSION   CruV-91
KEYWORDS    .
SOURCE      Crucivirus-91
  ORGANISM  Crucivirus-91
            Unclassified.
REFERENCE   1  (bases 1 to 2702)
  AUTHORS   de la Higuera,I., Kasun,G.W., Torrance,E.L., Pratt,A.A.,
            Maluenda,A., Colombet,J., Bisseux,M., Ravet,V., Dayaram,A.,
            Stainton,D., Kraberger,S., Zawar-Reza,P., Goldstien,S., Briskie,J.,
            White,R., Taylor,H., Gomez,C., Ainley,D.G., Harding,J.S.,
            Fontenele,R.S., Schreck,J., Ribeiro,S.G., Oswald,S.A., Arnold,J.M.,
            Varsani,A., Enault,F. and Stedman,K.M.
  TITLE     Direct Submission
  JOURNAL   Submitted (24-FEB-2020) Biology, Portland State University, 1719 SW
            10th Ave, Portland, Oregon 97201, USA
COMMENT     Bankit Comment: ALT EMAIL:ide@pdx.edu
            Bankit Comment: TOTAL # OF SEQS:451
            Bankit Comment: GAP: est. length - alignment

            ##Assembly-Data-START##
            Assembly Method       :: MetaSPAdes v. 3.12; IDBA-UD v. 1.1.1;
                                     MEGAHIT v. 1.0.3
            Sequencing Technology :: 454; Illumina
            ##Assembly-Data-END##
FEATURES             Location/Qualifiers
     source          1..2702
                     /organism="Crucivirus-91"
                     /mol_type="genomic DNA"
                     /isolate="120_2087_IR2_3297"
                     /isolation_source="Water"
                     /country="Norway"
                     /collection_date=2011
     CDS             1..1080
                     /codon_start=1
                     /product="Putative Replication Associated Protein"
                     /translation="MDSTKKFRFAGVKMLLTYPGHLNKPWLIDLLTIASNQPIKFIRC
                     AHEIGKTTGVDYDHTHCLIRWVNQLTTTDCRRFDVVLDGNTIHPNIAPIRTETHWRNQ
                     IAYIAKEDPDNSDLIVRSCVPKIWGAESLADALADNVKKPADTLGVIALYNARPAAEI
                     QVDTPDRPWQLEVLDFVQTKPDRRSVHWYYDPKGGAGKTWLARYMMANKLAYMVKQCG
                     GSSNFATIMAGAVASGWDQRCFILDLPRSAEDYKFYGCLEEICDGCVTATKYQGGTIL
                     FNQPHVLVFANFLPNRSAMSADRWVVHWLPQIEESIKAPPLQAAPGLGGLTLITLTEF
                     QPPKITRCDNVLSQEDIEEVLNDLL*"
     CDS             complement(1100..2467)
                     /codon_start=1
                     /product="Putative CruV Capsid Protein"
                     /translation="MPTTYYTQSGRPYTIVNGRRKYGAAVAAPRQLTGFGAYKTPEQY
                     AAKKQRKRVAAGAVVTVPRSAAYRLPPAVSEPDPWVEWGSKGGKAVAGFAKGILGLGD
                     YHVKQNVLMGRLPQVVNLPRGGGTIIRFCEYLGDITTSSSANTFAVSSFDINAGLFST
                     FPWLSQIAQNYEQYSFEGLLFEFRSTSADALNSTNTALGSVIMATNYDSSDPAFLSKG
                     EMLNYEFSTSIKPAESAMHMVECAPRQTVLTELYTRSAAVPAEDDQRFYDLGKFQIAT
                     AGFQGTSVVVGELHCTYQVRLLKPKLHQSLGNSISFAQMGNSTGVAAGTPLGTTTGWS
                     SVANVQTLAIVFTSSTVITFPVTNVIQQYLLRYGIQGTIAGALVYPVLTAANMTIGTG
                     TLQTATFPVAATAATSGVMHVWLKTTPNGLAPTLTFGVGGTFPTGTVVCAIGITQVPD
                     AVTIT*"
ORIGIN
        1 atggatagca ccaagaagtt ccggttcgca ggtgtcaaga tgctcttgac ataccctggc
       61 cacctcaata agccatggct tattgacctc ttgactatag catccaacca accgatcaaa
      121 ttcatccgct gcgcgcacga aatcggtaaa actacaggtg tggactacga ccacacccac
      181 tgcctgatcc gatgggtcaa ccagctaaca accaccgact gccgtcggtt cgacgttgtt
      241 ctggatggta acaccatcca ccccaacata gctcccattc gcacagagac tcactggcgt
      301 aaccagatcg cctacattgc gaaggaagac cctgacaaca gcgacctaat cgtcagatct
      361 tgcgtaccta agatctgggg cgccgagtcc ctcgctgacg ccctcgctga caatgtcaaa
      421 aaaccggccg acacccttgg ggtgatcgcc ctatacaatg ctcgtcctgc ggccgagata
      481 caagtagata ctccagaccg accctggcag ctggaagtct tggacttcgt ccaaactaaa
      541 ccagaccgca ggtcggtaca ctggtactac gaccccaaag gaggtgcggg caagacatgg
      601 ctagcccgct acatgatggc taataaatta gcttacatgg taaaacaatg tggcggctcg
      661 tcaaacttcg ccaccatcat ggccggagcc gtcgcctcgg ggtgggacca gcgatgcttc
      721 atcttggacc tcccccgctc ggcggaagac tataaattct acggctgcct cgaggaaata
      781 tgcgatggct gcgtcacagc cactaagtac caaggtggta ctatcctctt caaccagcct
      841 cacgtactcg tcttcgctaa ctttctaccg aaccggagcg ccatgtcggc cgaccgatgg
      901 gtcgtccact ggctacctca aatcgaagaa agtataaaag cccctccgct gcaagcagct
      961 ccggggcttg ggggcttaac attaataact cttacagagt ttcagccccc aaaaataact
     1021 cgctgtgata acgtgttatc ccaagaggac atcgaagaag tcctcaatga ccttctctaa
     1081 ggtcgaaacc ttcttttgtt tacgtaatcg taacagcatc cggaacctgg gtaatcccaa
     1141 ttgcacatac caccgtcccc gtcgggaacg taccaccaac accaaaggta agcgttggtg
     1201 caagaccatt cggagttgtt ttcaaccaca catgcatcac accagaagta gccgcagtcg
     1261 cggcaaccgg aaaggtcgct gtttgcagtg ttcccgtgcc aattgtcata ttggcagccg
     1321 taagaacagg gtacacaagc gctcccgcga tagttccctg aataccgtac ctcaagaggt
     1381 actgctgaat cacattcgta accgggaaag ttatcaccgt cgacgatgtg aacacaatag
     1441 caagcgtttg cacattcgcc acagacgacc acccagttgt cgtccccaac ggagtaccag
     1501 ctgctacacc cgtagaattg cccatttggg caaaggaaat actattccca agagattgat
     1561 gaagcttcgg cttcaacagc cgcacctggt aagtgcaatg aagctcacca acaacgacac
     1621 tggttccctg gaacccagcg gtcgcgatct gaaattttcc aagatcgtag aaacgctgat
     1681 catcttccgc cggaactgcc gccgaacgag tatacagctc agtcagaaca gtctgacgtg
     1741 gagcacactc caccatatgc atcgcgctct cagctggctt aatgcttgtg ctgaactcat
     1801 agttcagcat ctctcctttt gagaggaaag caggatcact actatcgtag ttcgtagcca
     1861 taataacact cccaagagca gtgtttgtag agttaagagc atccgcagaa gtactgcgaa
     1921 actcaaacaa gagaccctca aagctgtact gctcataatt ctgagcgatc tgcgacaacc
     1981 aaggaaaggt cgagaagaga ccagcattga tgtcaaagga cgacaccgcg aacgtattcg
     2041 cagacgatga cgtcgtgata tctccaagat actcacagaa ccgtataatc gtcccacctc
     2101 cacgaggaag gttaaccacc tgaggaagcc gtcccataag gacattctgc ttcacatgat
     2161 aatcaccaag gccaagtatg cccttagcaa acccggcaac agccttacca cccttacttc
     2221 cccattcgac ccaagggtca ggctcagaca cggctggagg aagacgatac gcagccgatc
     2281 gaggtactgt cacaacagca ccagcggcta ctcgtttccg ctgcttttta gcagcatact
     2341 gctccggcgt tttatatgct ccaaaacccg taagctgtct aggcgccgcc acagcggcac
     2401 catacttacg tcgcccgttc acaatcgtgt acggtctacc tgactgagtg taatatgttg
     2461 taggcatagg agtaccccat acctaccccg ctctaataga gcgcaccatt ctatacaccg
     2521 gacccatgcc tcgaggccca gacccgcacg tctcgtgaca ttcgggccgg gacaagcccg
     2581 gcccggccag ggcacattcg agccgacagg tcttaagagc tctgtggtgg gtcctcacac
     2641 catcccctgg aattccagga gatgccaatt agagaacaca aggttcctaa caagagagat
     2701 ag
//
LOCUS       CruV-92                 2789 bp    DNA     circular     24-FEB-2020
DEFINITION  Crucivirus-92 isolate 002052_virusbaton.
ACCESSION   CruV-92
KEYWORDS    .
SOURCE      Crucivirus-92
  ORGANISM  Crucivirus-92
            Unclassified.
REFERENCE   1  (bases 1 to 2789)
  AUTHORS   de la Higuera,I., Kasun,G.W., Torrance,E.L., Pratt,A.A.,
            Maluenda,A., Colombet,J., Bisseux,M., Ravet,V., Dayaram,A.,
            Stainton,D., Kraberger,S., Zawar-Reza,P., Goldstien,S., Briskie,J.,
            White,R., Taylor,H., Gomez,C., Ainley,D.G., Harding,J.S.,
            Fontenele,R.S., Schreck,J., Ribeiro,S.G., Oswald,S.A., Arnold,J.M.,
            Varsani,A., Enault,F. and Stedman,K.M.
  TITLE     Direct Submission
  JOURNAL   Submitted (24-FEB-2020) Biology, Portland State University, 1719 SW
            10th Ave, Portland, Oregon 97201, USA
COMMENT     Bankit Comment: ALT EMAIL:ide@pdx.edu
            Bankit Comment: TOTAL # OF SEQS:451
            Bankit Comment: GAP: est. length - alignment

            ##Assembly-Data-START##
            Assembly Method       :: MetaSPAdes v. 3.12; IDBA-UD v. 1.1.1;
                                     MEGAHIT v. 1.0.3
            Sequencing Technology :: 454; Illumina
            ##Assembly-Data-END##
FEATURES             Location/Qualifiers
     source          1..2789
                     /organism="Crucivirus-92"
                     /mol_type="genomic DNA"
                     /isolate="002052_virusbaton"
                     /isolation_source="Water"
                     /country="France"
                     /collection_date=2013
     CDS             1..1329
                     /codon_start=1
                     /product="Putative Replication Associated Protein"
                     /translation="MTGQHKRVCFTVFDENWLPDDIDRDKIKFFVGQEEICPDTQRHH
                     WQCYAECFKRRGWKKFEAAVGLPRGDDNKPRFHHSAAIASAEDNIAYCTKDASRMPNG
                     ERITFGDARPTRSVWDLIAIDIEKGMKLRDIKRKYVSTAGMYPTGLRDIYEVFKPVYR
                     EDAPEKWKPWQQHIVDLTATPCKDKSTIHWFWEPNGNAGKTTMSKYLYDHCGAFLVFN
                     GKTADLAYPYDSEPIIVFNFVMANEEKVNWQPIECLKDGIIYSSKYQSCLKRFASPHV
                     ICFANFRPPNGVMGSYKWNIVDIRNYGAADSVGGGPSPPNVPFRPDTVAAVKLSDIDF
                     DELVGTLCETSSNSNAASVVASPPPVIHFPTGSGNNSVPTAPDPVIIIRNRRRRLRRG
                     WFSTPSARGSAPSRPVMYAGGSPSGLAADLRTSASGGLSPLTSRCAVEPG*"
     CDS             1229..2731
                     /codon_start=1
                     /product="Putative CruV Capsid Protein"
                     /translation="MQAARPPGSPPICEHRRAGAYHRLHPGVRLNPARPIIYPHTDEY
                     CSPLLAKIESKYFSPRKLFAAIMSMSGRAYTAAQKRAFGARMAAARAAKKTKTPRKRR
                     IAPKSPDAITGFGKYKAGDPSLSNVGMELGRWVGKGADKIVKTLFGFGDYHIEHNTLM
                     AGGLSPPEMINSIDRGGFIVRHREYIGDINATTDFTVQSFFINPALIESFPWLSSVAE
                     AFEEYEFRGLVYEFKSMSSDALLSATTSTALGTVVMATQYNSLNPPFSSKIQMENYEF
                     ANSSKPSCDFLHPVECKRSLTPVTHLYTRSGPPPGGDIRLYDLGEFQIATQGMQAASG
                     VVGELWCTFEIELYKPKLIIDEEEILSGHWQLTNVTAASPLGAGEAVMVEGSSLPATI
                     TGGTITFPSTLSEGNFIIYWTTVGGNATIVAPTITALSNCTIRTWQRADTVTVIHDMN
                     GSTSGTLSEFFVLGLTGPSATVVFDDNGTLPTATTSGDLWITQLTELIAS*"
ORIGIN
        1 atgactggcc aacacaagcg cgtctgcttt accgtattcg acgaaaattg gctgcctgac
       61 gatatcgacc gcgacaaaat taaattcttt gtcggccaag aagaaatctg tccggataca
      121 caacgtcatc attggcaatg ttacgcagaa tgctttaaac gccgcggatg gaagaaattc
      181 gaggctgctg tcggtctccc tcgcggggac gacaataagc ctcgcttcca ccacagtgcc
      241 gccatcgctt cggctgaaga taacattgct tattgtacca aagatgcgtc ccgtatgcct
      301 aatggtgaac ggattacgtt tggcgatgcc agacctaccc gttctgtctg ggatcttatc
      361 gctatcgata tcgaaaaagg catgaaactc cgcgatataa aacgcaaata cgtctctacg
      421 gccggtatgt accctacagg tcttcgcgac atctacgaag tcttcaaacc tgtatatcgt
      481 gaagatgctc ctgaaaaatg gaaaccctgg caacaacaca ttgttgacct tacggctacc
      541 ccatgcaaag acaagtctac cattcactgg ttctgggaac ctaacgggaa tgctggaaaa
      601 actacaatga gtaaatatct ctacgatcat tgcggcgcct tcttggtttt caacggcaag
      661 accgctgatc ttgcctaccc ctacgacagt gaaccgatca ttgtgtttaa cttcgttatg
      721 gctaacgaag aaaaggtaaa ctggcaacct atcgaatgct taaaagacgg tatcatttac
      781 tcttcgaaat atcaaagctg cttaaaacgg ttcgcttcgc cacacgttat ctgtttcgct
      841 aactttcgac cgcctaacgg tgttatgggc tcttataaat ggaacattgt agatattagg
      901 aattacgggg ccgccgactc cgtcggcggc ggcccctctc cccctaatgt tccgtttagg
      961 cccgacaccg ttgcggcggt taaactgagc gatatcgact ttgatgaact tgttggcact
     1021 ttgtgcgaaa catcatccaa ttctaatgct gcttcagtgg tcgcttcgcc cccaccggtt
     1081 atccactttc ccacagggtc aggtaataat agcgtgccta cggcacctga ccctgtaatt
     1141 ataattcgaa atcgccgccg ccgactccgt cgcggatggt tcagcacacc ttcagcaagg
     1201 gggtcagccc cctctcgccc ggtgatgtat gcaggcggct cgccctccgg gctcgccgcc
     1261 gatctgcgaa catcggcgag cgggggctta tcaccgctta catccaggtg tgcggttgaa
     1321 cccggctaga ccaataatct atccgcacac ggatgaatat tgctcgccgt tactagccaa
     1381 gattgagagc aaatattttt ctccgcgaaa acttttcgca gctatcatgt ctatgtctgg
     1441 aagagcctac actgctgcgc aaaaacgcgc cttcggcgct cgtatggccg ccgctcgcgc
     1501 tgctaaaaaa acaaaaacgc ctagaaaacg tagaattgcg cctaaaagtc ccgacgccat
     1561 caccggcttt ggtaaataca aagctggcga tccaagcctc tccaatgtcg gcatggaact
     1621 cggaagatgg gtcggaaaag gcgctgataa aatagtgaaa actctattcg ggttcggcga
     1681 ttatcatatt gagcacaata cgctcatggc cggcggcctt tcgccgccag aaatgataaa
     1741 cagcatagac agaggcggat tcattgttcg ccaccgcgaa tacattgggg acatcaacgc
     1801 cacgacagac ttcacagtgc agtctttctt tataaatccc gccctaatag aaagctttcc
     1861 gtggctttct tccgtcgccg aagcattcga agaatatgaa ttccgcggcc ttgtctacga
     1921 gtttaaatct atgagctcag acgctctcct ctccgccacc acaagcactg ctttaggtac
     1981 cgtcgttatg gctactcaat acaattcgct gaaccctcct ttctcatcca aaatacaaat
     2041 ggagaactac gagtttgcca actcttccaa accctcgtgc gacttcctcc acccagttga
     2101 atgcaaacgt tcgcttactc ccgttactca tctctatact cgtagtggtc caccgcctgg
     2161 cggcgatatc cgtctctacg atctgggcga atttcagatc gctacacagg gtatgcaagc
     2221 cgcctccggc gtcgttggcg aactgtggtg cacgttcgaa atcgaactct ataaacctaa
     2281 actcatcatc gatgaagaag aaatcctctc cggccactgg cagctaacta acgttaccgc
     2341 cgcgagccct ttaggggccg gagaggctgt aatggtggaa ggctcttctc taccggcaac
     2401 cattaccggc ggcacaatca cgtttccctc tacgttgtcg gagggaaact ttataatata
     2461 ctggacaaca gtcggtggca acgcgacgat cgtcgccccg actattaccg cccttagtaa
     2521 ctgtacgata cgcacttggc agcgcgccga caccgttacg gttatccatg atatgaatgg
     2581 cagcacctcc ggcaccctct ccgaattctt cgttctaggg cttaccggtc ccagcgccac
     2641 agtcgtcttc gacgacaatg gtactcttcc taccgctacc acgtcgggcg atctctggat
     2701 cacccaactc acggaactta tcgcctctta atctacttgc ggccgcaggt tatttttcgt
     2761 tttagaaaat ctgtcgcact agtatagcc
//
LOCUS       CruV-93                 2795 bp    DNA     circular     24-FEB-2020
DEFINITION  Crucivirus-93 isolate GP1_93001.
ACCESSION   CruV-93
KEYWORDS    .
SOURCE      Crucivirus-93
  ORGANISM  Crucivirus-93
            Unclassified.
REFERENCE   1  (bases 1 to 2795)
  AUTHORS   de la Higuera,I., Kasun,G.W., Torrance,E.L., Pratt,A.A.,
            Maluenda,A., Colombet,J., Bisseux,M., Ravet,V., Dayaram,A.,
            Stainton,D., Kraberger,S., Zawar-Reza,P., Goldstien,S., Briskie,J.,
            White,R., Taylor,H., Gomez,C., Ainley,D.G., Harding,J.S.,
            Fontenele,R.S., Schreck,J., Ribeiro,S.G., Oswald,S.A., Arnold,J.M.,
            Varsani,A., Enault,F. and Stedman,K.M.
  TITLE     Direct Submission
  JOURNAL   Submitted (24-FEB-2020) Biology, Portland State University, 1719 SW
            10th Ave, Portland, Oregon 97201, USA
COMMENT     Bankit Comment: ALT EMAIL:ide@pdx.edu
            Bankit Comment: TOTAL # OF SEQS:451
            Bankit Comment: GAP: est. length - alignment

            ##Assembly-Data-START##
            Assembly Method       :: MetaSPAdes v. 3.12; IDBA-UD v. 1.1.1;
                                     MEGAHIT v. 1.0.3
            Sequencing Technology :: 454; Illumina
            ##Assembly-Data-END##
FEATURES             Location/Qualifiers
     source          1..2795
                     /organism="Crucivirus-93"
                     /mol_type="genomic DNA"
                     /isolate="GP1_93001"
                     /isolation_source="Water"
                     /country="New Zealand"
                     /collection_date=2014
     CDS             1..1110
                     /codon_start=1
                     /product="Putative Replication Associated Protein"
                     /translation="MEPKDSKKNRNAGTSGTASVILKGKARCYTGTWNNYSEEEYVKL
                     GQFCIDEKCVEYAYQKEEGEIDGTPHIQFAMKFENPRSWAAIVKKLTKNVQDEDKTKY
                     DVEGKPKNNFHLEKAKNWIACMKYCSKDDTCVGGKVKVEKVKDPYILDDPLEGNDLHP
                     WQKELELELEVKADKRSIIWYYDHEGNSGKTTFAKYLCIKYPGEVIYMAGKAADLKYG
                     VFDYHQTQDAMVLKGFKRQYLRIVMFDLTRSTEDFISWQGIEEVKNGIFYNTKYKSGM
                     CLYNCPHVIIFANFKPNKLKLSLDRWNIRILGKINGEIRILTKLEAKKEKYKREGKYE
                     SDDEDEKPKPSNLLVEDENIEKSDLDLFEYDSLKL*"
     misc_feature    51..82
                     /note="stem-loop"
     misc_feature    59..67
                     /note="nonanucleotide"
     CDS             complement(1316..2731)
                     /codon_start=1
                     /product="Putative CruV Capsid Protein"
                     /translation="MAYRNTGRRQYSPAEKRAYYASMSKAVVPRVRAQRSGAYTGYGA
                     YVKAPVEKSPAKVKKPRREHHGAFKTGGRYAGGLAGGALGTALGGPAGGALGSALGTF
                     LGGKLGHLASEITGFGDYKIEDNTIMTGGMSPPEIVNSVDTGSTIVRHREYIGDITAT
                     TAFTNREYLVQPGLSTTFPWLSSMASSFEQYRLRGVLFEFQSTSADALLSSSTSTALG
                     TVLMSTDYDIADLPPTSKREMLNAMFSTSGKPSITMIHPIECKKSLSANDIMYTRSAL
                     VTPEGYDPRLYDFCRFNIATEGMQADGGVLGELWVTYEIELFKQQYHYQSLANHFRQS
                     SVSNTAPLGLVTDFSNINSGGTIGGTISGDGTHYNFPQQVSNGKYLMTYCADGGSAAL
                     VAPALTYTNCTELSWFDGDTRTFVNSGGTFTRFIQQSLFEITGEGAVVTFGAAGTLPT
                     SSNVDLFIIRLPDVMTAGVAP*"
ORIGIN
        1 atggaaccta aagattcaaa aaaaaaccgg aacgctggaa ccagtggaac agcgagcgta
       61 atactaaaag gtaaagctcg ctgttatact gggacatgga ataactattc cgaagaggaa
      121 tatgttaaat taggacagtt ttgcatagat gaaaaatgtg ttgaatatgc gtatcaaaag
      181 gaagaagggg aaatagatgg aaccccacat atacaatttg ctatgaaatt tgagaatcct
      241 agatcttggg cagcaatagt aaaaaaattg acaaaaaatg tacaagatga agataaaact
      301 aaatatgatg tagagggtaa acctaaaaac aattttcact tagaaaaagc caagaactgg
      361 attgcatgta tgaaatactg tagcaaagat gatacatgtg ttgggggtaa agttaaagta
      421 gaaaaagtta aagatccgta tattctagat gatcccttag aagggaatga tttacatcca
      481 tggcaaaaag aattagaatt agaattagaa gttaaagcag ataaacgaag tattatctgg
      541 tattatgacc atgaagggaa tagtggaaag actacatttg ctaaatacct gtgtataaaa
      601 tatcctgggg aggtaattta tatggcagga aaggcagcag atttaaaata tggagttttc
      661 gattatcacc aaactcaaga tgctatggta ctaaaaggtt ttaaaaggca atatttaagg
      721 attgtaatgt ttgatctaac gagaagtact gaagacttca tatcttggca aggaattgaa
      781 gaagttaaaa atggtatatt ctacaacact aaatataaaa gtggaatgtg cttatataat
      841 tgcccacatg taattatatt cgccaatttt aaaccaaata aattaaaatt atctttagat
      901 agatggaata taagaatatt aggaaaaata aatggagaga taaggatcct taccaagtta
      961 gaagcaaaga aagaaaaata taaaagagaa ggtaaatatg aaagtgatga tgaagatgaa
     1021 aaaccaaaac caagcaatct tttggttgaa gatgaaaaca tagaaaaatc tgacttagac
     1081 ttatttgaat atgatagtct taagttatag gggtactaaa gacccccccc cttcatgttc
     1141 ggtcgatggc gacacttgat cctgcgtcgt ggacagggat agcccttcgg actatccact
     1201 cctcggatcg gcgatgtctg actccctcca taaggacaaa gggttttctc ctaggtcgtg
     1261 tataattaat aatgaaaaaa tattgttttt tatatagaca tcgcgaactt tttttttatg
     1321 gagcaactcc ggcagtcata acgtctggca atctaattat aaatagatcg acattagaac
     1381 tagttggcaa agtacctgca gcaccgaaag taacaacagc accttcaccg gtaatctcaa
     1441 agagagattg ttgaatgaaa cgggtgaatg taccccccga attaacaaag gttctggtat
     1501 ctccatcaaa ccaagacaat tctgtgcagt tagtatatgt aagagctgga gcaactaaag
     1561 cagcagaacc accgtcagca cagtatgtca ttagatactt accattagaa acctgttgtg
     1621 gaaaattata gtgggtacca tcaccactaa tcgttcctcc aattgtacct cctgaattga
     1681 tgttactaaa atcggttact agacccaagg gagcggtatt gcttacacta gactgtctaa
     1741 aatgattggc aagactttga taatgatatt gttgtttaaa taactctatt tcataggtta
     1801 cccacaactc acctagaact cctccgtcgg cttgcatacc ttcagtcgca atgttgaaac
     1861 ggcaaaaatc atataatcga ggatcatatc cctcaggcgt gactaaagca ctacgggtat
     1921 acatgatatc attggctgaa agggatttct tgcattcaat aggatggatc attgttatag
     1981 aaggctttcc ggatgttgag aacattgcat taagcatctc tctcttactt gttggtggaa
     2041 gatcggctat atcataatcg gttgacatca gaactgtacc caaagctgta cttgtagagg
     2101 aagacaataa tgcatctgcc gatgtggact ggaattcgaa taaaacaccc ctcaatcgat
     2161 attgttcaaa tgaactagcc atagaagaca accaagggaa agttgttgac aatccaggtt
     2221 gtacaagata ttcacgattg gtaaaagcag tagtggcagt tatatctccg atatactccc
     2281 tatgtcgaac tattgtcgat ccagtatcta cactattcac aatctcggga ggtgacattc
     2341 ctccagtcat aatggtattg tcttcgattt tataatctcc gaaaccagtt atttctgatg
     2401 cgagatggcc gagtttacca cctaaaaagg tacccagagc agatccgaga gccccaccag
     2461 cgggtccccc taatgcagta cctaaggcac caccggcaag tcctccagca taacgaccac
     2521 ctgtcttgaa agcaccatga tgttcacgtc taggtttttt gaccttagcc ggagactttt
     2581 caacaggggc tttgacgtat gcaccatatc cggtataggc accagatcgt tgagcacgca
     2641 cacgaggtac taccgcttta ctcatagagg cataatatgc cctcttctca gcaggtgagt
     2701 actggcgacg accggtattc ctataggcca ttttctagtg gaattatatt ttacaaaaat
     2761 tttttcaaat ataaagaaaa gaccgaccgg aacaa
//
LOCUS       CruV-94                 2797 bp    DNA     circular     24-FEB-2020
DEFINITION  Crucivirus-94 isolate C1C10_147.
ACCESSION   CruV-94
KEYWORDS    .
SOURCE      Crucivirus-94
  ORGANISM  Crucivirus-94
            Unclassified.
REFERENCE   1  (bases 1 to 2797)
  AUTHORS   de la Higuera,I., Kasun,G.W., Torrance,E.L., Pratt,A.A.,
            Maluenda,A., Colombet,J., Bisseux,M., Ravet,V., Dayaram,A.,
            Stainton,D., Kraberger,S., Zawar-Reza,P., Goldstien,S., Briskie,J.,
            White,R., Taylor,H., Gomez,C., Ainley,D.G., Harding,J.S.,
            Fontenele,R.S., Schreck,J., Ribeiro,S.G., Oswald,S.A., Arnold,J.M.,
            Varsani,A., Enault,F. and Stedman,K.M.
  TITLE     Direct Submission
  JOURNAL   Submitted (24-FEB-2020) Biology, Portland State University, 1719 SW
            10th Ave, Portland, Oregon 97201, USA
COMMENT     Bankit Comment: ALT EMAIL:ide@pdx.edu
            Bankit Comment: TOTAL # OF SEQS:451
            Bankit Comment: GAP: est. length - alignment

            ##Assembly-Data-START##
            Assembly Method       :: MetaSPAdes v. 3.12; IDBA-UD v. 1.1.1;
                                     MEGAHIT v. 1.0.3
            Sequencing Technology :: 454; Illumina
            ##Assembly-Data-END##
FEATURES             Location/Qualifiers
     source          1..2797
                     /organism="Crucivirus-94"
                     /mol_type="genomic DNA"
                     /isolate="C1C10_147"
                     /isolation_source="Soil"
                     /country="New Zealand"
                     /collection_date=2012
     CDS             1..1065
                     /codon_start=1
                     /product="Putative Replication Associated Protein"
                     /translation="MQQEQSRTVLTNNNPLTMWDLTIPCDRFTLDTVRSFMRTYCHRW
                     CFQQEKGDETGYLHYQCRISLITKKRPHNMHQFIQQVLPTSHCSPTSNATHNTGNEFY
                     VTKVDTRVNGPWTDRDDINTDKIPTRFRNEPTWNPMQQSILNIISLPPDDRTINVLID
                     TIGQRGKTFLTMWLATRNKAERIPQQKDARDIMRMVMDMPKRTCYFIDLPRGTSHKDQ
                     HSIYSAIEEIKNGYAYDDRFKFRKEYFEPPHIWVFTNEKPNTSLLSIDRWKFWSVSND
                     YHLIPQGTTINISEDINRPSGTHTPLTLNIITNNITNKLIEVPTSITLPLIPVTPTVV
                     PKTPTPIINHHTFQNNLKTN*"
     CDS             complement(1366..2721)
                     /codon_start=1
                     /product="Putative CruV Capsid Protein"
                     /translation="MPGINRGIRQYTAAEKRAYYAKKGKKQVKVAPGISMAGSGAYKQ
                     RVQKYQSVPRSVGGQGKYYFRDNYGKDLGSQIAGNLGGLIGEGIQSLGRAIGFGDYKV
                     EHNVLMNELQGGPPVMQTTSKRGQVIRHREYLQNIVTASDGSFKLQSFALNPGLVQTF
                     PWLSQVAAGFEQYKINGMIWEFKSTSADSLSSTTTSLGSVIMATEYDSSRAVFANQQQ
                     MANHEFAMSCRQSASMLHAVECKKSLTPVSELWVRTGAVPSGDDERLYDWGLFQIASV
                     GGPSVANTVGELWVTYEVEFYKPQLIGGLGLELLSDHYSTTSTISTSHYFGLQSGSDP
                     KAVTGSNLGTTISGPTIIFPVGLSEGNFMVQYAVVGNSTANTKPTLTFANCAAQNLFA
                     NDTSGSSDNSGSTSVTLLVNLVVKVTGQGATITFSAGTLPAAATAMDLWVNQLNANIV
                     T*"
     misc_feature    1655..1677
                     /note="stem-loop"
     misc_feature    1663..1671
                     /note="nonanucleotide"
ORIGIN
        1 atgcaacaag aacagtctag aactgttcta acaaataata acccactcac tatgtgggac
       61 cttaccatcc cctgtgacag atttacccta gacaccgtcc gctccttcat gcgaacctac
      121 tgtcacagat ggtgctttca acaagaaaaa ggtgatgaaa caggatactt acactaccaa
      181 tgtagaatat cactcatcac taagaaacgc ccccacaata tgcaccaatt tatccaacaa
      241 gtacttccta cttcacactg ttctccaacc agtaatgcta cgcacaatac tggtaatgaa
      301 ttttatgtca ctaaagtcga caccagagtc aacggccctt ggaccgatcg tgacgatatc
      361 aatacagaca aaattcctac taggttcaga aatgaaccca catggaaccc catgcaacaa
      421 tcgatcttaa atataatatc actaccccca gatgatagaa caataaatgt tctcatcgac
      481 accattggac aaagaggtaa aaccttcctc actatgtggc tcgccaccag aaataaagcc
      541 gagcgcattc ctcaacagaa agacgccaga gacattatgc gtatggtcat ggatatgccc
      601 aaacgaactt gttatttcat tgatcttcct agaggtacct cacacaaaga tcaacactcc
      661 atctactccg ccattgaaga aatcaaaaac ggctacgcct atgatgacag gttcaaattc
      721 cgcaaagaat actttgaacc tcctcacatt tgggttttca ctaatgaaaa acccaataca
      781 tcccttctgt ctatagacag atggaaattt tggtctgtgt ctaacgacta tcacctcatc
      841 cctcaaggca ccaccatcaa tatatctgaa gatatcaatc gcccaagtgg aacacatact
      901 ccacttacat taaacattat tactaacaat attactaata aattgattga ggtccctacc
      961 tcaatcacac ttccactcat accagtcact cctaccgtcg tgcctaaaac tcctacgcca
     1021 ataataaatc atcatacttt tcaaaataat ttgaaaacca attaacaata atttctttct
     1081 tttttttggc gctgacgctg taatactata caaatatata ttatcatata tatttttatt
     1141 acaaaaaaaa gaacactgga acgggggcgc attaactcaa cctgcccccc cagtgggcct
     1201 taattcaacc tgcctaggtt aattcaacct agaacaataa tagaacacta tagaacacac
     1261 tagaacatta atactattat taccgggcac ccctccgggg aatagaccac taagaccggg
     1321 gaaaaggaca ctgcgccggc cttcggccgt cgttacttag ttcatttaag ttactatatt
     1381 agcattcaat tgattaaccc aaagatccat tgcagtcgcc gcagcaggta aagttcctgc
     1441 agaaaatgta atagtagcac cctgaccagt aactttaaca acaagattaa ctaagagcgt
     1501 cacagacgtc gaacccgaat tatctgatga accagacgta tcatttgcaa ataaattttg
     1561 agcagcacaa tttgcaaaag ttaaagtagg ctttgtattt gcggtagaat tacccactac
     1621 cgcatattgt accatgaagt taccttccga caaacctact ggaaatatta tagtaggacc
     1681 cgatatagtc gtgcctaaat tagagcctgt tacagccttt ggatcactcc cactttgtaa
     1741 cccaaaataa tgagatgtag aaatagtact agttgttgaa taatgatcac tcaacaattc
     1801 aagacccaaa cctccaatca attgaggctt gtaaaattca acctcataag taacccataa
     1861 ctcaccaaca gtattagcta ctgatggacc cccaactgat gctatttgaa ataaacccca
     1921 atcatacaac ctctcatcat ctcctgaagg tacagctcct gtacgaaccc aaagttcaga
     1981 cactggtgtc aatgacttct tgcactcaac cgcatgcaac atagatgcag attgcctgca
     2041 tgacatggca aattcatgat ttgccatctg ttgctgatta gcaaatactg cacgagaaga
     2101 atcatattct gtcgccataa taacactacc caacgaagta gtggtagaag aaagagaatc
     2161 agctgaggta gatttgaatt cccaaatcat accattgatc ttatattgct caaaaccagc
     2221 tgctacctga gaaagccacg gaaacgtttg aaccaatcct ggattcaacg caaatgactg
     2281 caacttaaaa ctaccatctg atgcagtcac tatattttgc aaatactctc gatgacgaat
     2341 tacttgaccc ctcttcgaag tagtctgcat aacaggtgga cccccttgta actcattcat
     2401 caaaacatta tgctccacct tataatcacc gaatccaatc gctctaccaa gagattggat
     2461 tccttctcca atcaaccctc ccaaattacc agctatttga gagccaagat cctttccata
     2521 attatcacgg aaataatact ttccttgacc acctacactt cttggtacgg actgatactt
     2581 ttgtactctt tgcttatatg caccacttcc cgccatagat ataccgggtg caactttaac
     2641 ttgcttctta cccttcttag cataataagc cctcttctct gctgcagtat actgcctaat
     2701 gccacgatta attcctggca tacttggttg tgtttataca ctcattacca ccatttatac
     2761 tatttatatg gtataaatac atgctttatt actataa
//
LOCUS       CruV-95                 2810 bp    DNA     circular     24-FEB-2020
DEFINITION  Crucivirus-95 isolate 002033_virusbaton.
ACCESSION   CruV-95
KEYWORDS    .
SOURCE      Crucivirus-95
  ORGANISM  Crucivirus-95
            Unclassified.
REFERENCE   1  (bases 1 to 2810)
  AUTHORS   de la Higuera,I., Kasun,G.W., Torrance,E.L., Pratt,A.A.,
            Maluenda,A., Colombet,J., Bisseux,M., Ravet,V., Dayaram,A.,
            Stainton,D., Kraberger,S., Zawar-Reza,P., Goldstien,S., Briskie,J.,
            White,R., Taylor,H., Gomez,C., Ainley,D.G., Harding,J.S.,
            Fontenele,R.S., Schreck,J., Ribeiro,S.G., Oswald,S.A., Arnold,J.M.,
            Varsani,A., Enault,F. and Stedman,K.M.
  TITLE     Direct Submission
  JOURNAL   Submitted (24-FEB-2020) Biology, Portland State University, 1719 SW
            10th Ave, Portland, Oregon 97201, USA
COMMENT     Bankit Comment: ALT EMAIL:ide@pdx.edu
            Bankit Comment: TOTAL # OF SEQS:451
            Bankit Comment: GAP: est. length - alignment

            ##Assembly-Data-START##
            Assembly Method       :: MetaSPAdes v. 3.12; IDBA-UD v. 1.1.1;
                                     MEGAHIT v. 1.0.3
            Sequencing Technology :: 454; Illumina
            ##Assembly-Data-END##
FEATURES             Location/Qualifiers
     source          1..2810
                     /organism="Crucivirus-95"
                     /mol_type="genomic DNA"
                     /isolate="002033_virusbaton"
                     /isolation_source="Water"
                     /country="France"
                     /collection_date=2013
     CDS             complement(216..1673)
                     /codon_start=1
                     /product="Putative CruV Capsid Protein"
                     /translation="MSQGLTTIIFFHYKYLIFYIVSHGTSFVFFNMSGRKSYTDAQKA
                     AYYRRRAAAGKPKKKYSASKTVRTSNTRRLRGHGDYSYDEPGPFGKVGRLLGGGLGQM
                     VGGSTGREIGEKVGGLAHWIGKLFGSGDYKMGENPDYNVLTNAHQIPQFSSSKMTNMV
                     CHREYIQDIISSATPGAFNLQSFDINAGLAASFPWLSNLAQNYEQYKVHGMVFEFRTM
                     SADALNSTNTALGQVIMACEYNSANPNFINKQQMENYEYAISCKPSESAYCAVECAKN
                     QTPVAELYVRGHAVPSGQDQRLYDLGNFQIATNGLQGASVNVGELWVTFCVELLKPRL
                     FGGTLGDAILTAHINLSTSIAVTTGYLIIHPTLADYDGIGLLPFTTATTLNFPANIID
                     GTYMIVYTCKGNSTAAVVGPTITATSGCSAFNMYDGEAASAIVLPSATSTIAITTKFI
                     KITSGGAIVTFSGGTFPNVISSGDLYVCQLPGDIT*"
     misc_feature    620..644
                     /note="stem-loop"
     misc_feature    626..634
                     /note="nonanucleotide"
     CDS             complement(1839..2810)
                     /codon_start=1
                     /product="Putative Replication Associated Protein"
                     /translation="MEVDQLVEATGIKSKDVKETPKKRIREPKKCAWVFTWNNYPTEW
                     EDKLGKLPDRAYMVAGKELAPTTGTPHIQGYLRFHNGVTLTWLTKRIAGAHFAMAKGT
                     DKDQDYCKKDGDFISYGAPSQQGKRTDIIAMRNAVLSGMNAREIFEQVAVNQQGLKIM
                     DRAFDLFEPPRDPSWGEFKVSWWYGPTATGKTSRARREYPDYYFHTGDTMQWQQGFDG
                     HRGMIIDDLRDTTCTYVYLLKLCQAFPLGAPFKGGMRQMTAMHIIITSCYSPVDAYRH
                     TTESKSQLYRRITDLAYFPVYGKWEDQKIDLFGNITDNTRSGLSQRS*"
ORIGIN
        1 gtcgcgctat ataaagtatg tgaaacttta taacacttcc cgcgccaggt ggcgcggggt
       61 ggcgcgacaa gtccggggta ataataggta aacctgcccg gacttgctca tcggcgcact
      121 aaagtgcacc gattcgccgc ttcgcgtgtt caaaaagtac aaaatctatg tcactcgaaa
      181 aaacttttta gttattttca gaccaaaaga caaccttagg tgatgtctcc aggaagttgg
      241 cagacataaa gatcaccaga agatatcaca ttcgggaaag tacctcctga aaaggtaact
      301 atagcacctc cagaagtaat cttgataaac ttagtagtaa tcgcaattgt tgaagtagca
      361 ctaggaagta caatagcaga agcagcctca ccatcataca tgttgaaagc agaacaacca
      421 ctcgttgcgg taatcgtagg accgacaact gcggctgttg aattaccctt acaggtataa
      481 actatcatat aagtaccatc aatgatgttt gcaggaaagt tcaaagtagt tgccgtagta
      541 aacggaagta aaccgatacc atcataatcg gccaaagtag gatggattat caaataacca
      601 gtagtaactg caattgaagt tgacagatta atatgagcag tcaaaatggc atctccaaga
      661 gtacctccaa agaggcgagg tttgagtaac tctacacaga aggttaccca tagctcacca
      721 acatttacgg aagctccttg gagaccattt gtggcaattt ggaagtttcc aagatcataa
      781 agcctctgat cctgtccact tggtactgca tgacctcgaa catacagttc ggcaacagga
      841 gtctgattct ttgcgcactc aactgcacaa taggcagatt cggaaggctt acaagatatg
      901 gcatactcgt aattctccat ctgttgttta tttataaaat tcggatttgc cgaattgtat
      961 tcgcacgcca taatcacctg accgagagct gtattggtcg agttcaacgc atctgctgac
     1021 attgtacgaa actcaaagac cataccatga accttgtatt gttcataatt ttgtgccaaa
     1081 ttactcaacc aaggaaaact tgctgccaaa ccggcattaa tatcaaaact ttggagatta
     1141 aatgcacctg gagtggcaga agatataata tcctgaatat attcacgatg acagaccatg
     1201 tttgtcattt ttgaagatga aaactgagga atctgatgag cattggtcaa aacgttataa
     1261 tctgggtttt cacccatctt atagtctccg gatccgaaga gttttccgat ccagtgggct
     1321 aaaccaccca ctttctctcc aatttctctt cctgtggacc ctccaaccat ttgaccaaga
     1381 ccgccaccaa gaagacgacc tactttgccg aacggacctg gttcgtcgta ggaataatct
     1441 ccgtgtcctc ggagacgtct tgtgttagaa gttctaaccg tcttggacgc agaatacttc
     1501 tttttgggct taccagctgc ggctctacgc cgatagtagg ctgctttttg agcatctgtg
     1561 taactttttc ggcctgacat gttgaaaaac acgaagcttg tcccgtggct gactatataa
     1621 aagattagat atttataatg aaaaaatata atagttgtca gcccctgcga caaggacctt
     1681 acgacccggt ttggttgtgt cttttcaaga acttatcctc gaatatccgg tggattcgag
     1741 gatcgttgct tgaacacaac gtcctcgggt cttcagctcc tcgtcttttg ggctctttcg
     1801 ttagatgtaa acatcgactt cgtcttggtt gccttcgctc agctacgctg gcttaggccg
     1861 cttcgcgtgt tatcagttat attgccaaat aaatctattt tttggtcttc ccactttcca
     1921 taaactggga aatatgcaag atcagttata cgcctgtaca gctgactttt gctctcagtt
     1981 gtatgtctat aagcatccac aggagaataa caagatgtta taatgatatg cattgctgtc
     2041 atttgccgca ttccaccctt aaaaggtgct ccaagcggaa atgcctgaca caattttaac
     2101 aaatatacat acgtacaagt tgtatctctc aggtcatcga tgatcatacc tcgatgcccg
     2161 tcgaaacctt gttgccactg catcgtgtca cccgtatgaa aataataatc gggatattct
     2221 cgtctcgcgc gagaagtttt acctgttgca gttggtccat accaccaact aaccttaaat
     2281 tccccccacg aaggatcgcg aggaggttca aataaatcga atgctcgatc cataatcttc
     2341 aatccttgct gattaacagc aacttgctca aagatttctc gggcattcat accggaaagt
     2401 acagcatttc gcatagcaat aatgtctgtt ctctttccct gttgtgatgg tgctccataa
     2461 cttatgaagt ccccatcttt tttgcaatag tcttggtcct tatcagtacc ttttgccatt
     2521 gcaaaatggg ccccagctat acgcttggtc aaccacgtaa gtgttacacc attatgaaaa
     2581 cggagataac cctgtatatg tggagttccg gtagtcggag ctagctcctt tccggctacc
     2641 atatacgcgc gatcgggtaa ctttcccaac ttatcttccc attcggtcgg ataattgttc
     2701 caagtaaaca cccaagcaca tttcttgggt tcccgaatac gctttttcgg tgtctctttg
     2761 acatccttag acttaattcc ggttgcctca acaagttggt caacctccat
//
LOCUS       CruV-96                 2839 bp    DNA     circular     24-FEB-2020
DEFINITION  Crucivirus-96 isolate 120_320_SvL2_3298.
ACCESSION   CruV-96
KEYWORDS    .
SOURCE      Crucivirus-96
  ORGANISM  Crucivirus-96
            Unclassified.
REFERENCE   1  (bases 1 to 2839)
  AUTHORS   de la Higuera,I., Kasun,G.W., Torrance,E.L., Pratt,A.A.,
            Maluenda,A., Colombet,J., Bisseux,M., Ravet,V., Dayaram,A.,
            Stainton,D., Kraberger,S., Zawar-Reza,P., Goldstien,S., Briskie,J.,
            White,R., Taylor,H., Gomez,C., Ainley,D.G., Harding,J.S.,
            Fontenele,R.S., Schreck,J., Ribeiro,S.G., Oswald,S.A., Arnold,J.M.,
            Varsani,A., Enault,F. and Stedman,K.M.
  TITLE     Direct Submission
  JOURNAL   Submitted (24-FEB-2020) Biology, Portland State University, 1719 SW
            10th Ave, Portland, Oregon 97201, USA
COMMENT     Bankit Comment: ALT EMAIL:ide@pdx.edu
            Bankit Comment: TOTAL # OF SEQS:451
            Bankit Comment: GAP: est. length - alignment

            ##Assembly-Data-START##
            Assembly Method       :: MetaSPAdes v. 3.12; IDBA-UD v. 1.1.1;
                                     MEGAHIT v. 1.0.3
            Sequencing Technology :: 454; Illumina
            ##Assembly-Data-END##
FEATURES             Location/Qualifiers
     source          1..2839
                     /organism="Crucivirus-96"
                     /mol_type="genomic DNA"
                     /isolate="120_320_SvL2_3298"
                     /isolation_source="Water"
                     /country="Norway"
                     /collection_date=2012
     CDS             1..927
                     /codon_start=1
                     /product="Putative Replication Associated Protein"
                     /translation="MTNPVCVFDFTAPADRASVEDLKQWLAKIAKQWVFQKEQGDETG
                     YIHYQGRISLKVKTRLEKLIKDAPHEWGFHWSPTSKNNQGNDWYVTKEETRIEGPWSD
                     KDKTVTIPKQVQEVINKGWYPWQISIYNLSKVWDTRTIHCIYNEKGNEGKSILCQYLR
                     CMGIAMNIPFCNDFKDIMRMVMDMPKVGCYTIDMPRAINKEKLFQLFAAIEVVKGGYA
                     YDDRYSFKSEDFDCPNIFVFTNKIPDFNLLSMDRWRLWEIKNHQLVPYDPNSLSNASL
                     TQQLLNQNANASNPEVVGTISVPIRIPPTGGQ*"
     CDS             complement(1364..2752)
                     /codon_start=1
                     /product="Putative CruV Capsid Protein"
                     /translation="MSYSSGYRNYRRPSYTNSYYSRNYYNRKPMYNVPPPPPPPPKPR
                     VSSSSKKTLSSSAPRKRSFNFKPLTSALGGIAGTFLGGPAGAAAGSSLGGLAGDLLGK
                     ITGLGDYKIQYNTLANNSVKLLSGDAVPAVQNTHTGVRICHREYITDISSSIAFSIQN
                     YNINPGLSSTFPWLSAVAQNFQQYKLHGMIFTFVSTSADALNSTNTALGTVILGTNYN
                     ASSSNFSSKQQMENNEFTTSSKPSCNVMHMIECDPKQTLQEGKFYVRTGVLPANQDIK
                     TYDIGNFQIATVGSQAVAVIGELHVSYDIELMKPIDLSITDQGAGLSHYYSNTNVNTS
                     AYFGTVQANRFDNIGLTISTTGITFPNTLQPGQVYQVFVWYGGTAANVATPTITLGNC
                     TQLSIYTSGSANILTTGGNTGSTVLAMNFTLRVSGTASIVTPVSFTFSGGTLPGSPVC
                     ELFVSQEPNSIA*"
     misc_feature    2252..2283
                     /note="stem-loop"
     misc_feature    2266..2274
                     /note="nonanucleotide"
ORIGIN
        1 atgaccaacc cagtttgtgt gtttgacttt acagctcccg ctgatcgcgc ttctgtcgaa
       61 gacttaaagc aatggcttgc aaaaattgcc aagcaatggg tgtttcaaaa agaacaaggt
      121 gatgaaactg gttacataca ctaccaaggt agaatcagtc taaaagtcaa aacaagattg
      181 gaaaaattga ttaaagatgc tccacacgaa tggggattcc attggtcacc aacaagtaaa
      241 aataatcaag gtaatgattg gtacgttacc aaagaagaaa ccagaataga aggtccttgg
      301 tcagacaagg ataaaacggt taccattcca aaacaagttc aagaagttat aaacaaaggt
      361 tggtaccctt ggcaaatatc aatttataac ctctcaaaag tatgggacac cagaactatt
      421 cattgtatat acaatgaaaa aggaaatgaa ggtaagtcaa tattatgtca atatctaaga
      481 tgcatgggta tagcaatgaa cattccattc tgtaatgact tcaaagatat catgagaatg
      541 gtcatggaca tgcccaaagt cggttgttac accattgata tgccaagagc aatcaacaaa
      601 gaaaagttat ttcaactttt cgctgctata gaagtagtaa aaggaggcta cgcctacgat
      661 gacagatact cattcaaatc tgaagatttt gactgtccca acatattcgt attcacaaat
      721 aaaataccag actttaatct cctctcaatg gaccgctgga gactctggga aataaaaaac
      781 catcagttag ttccttacga tcctaactca ctatccaacg cttcgctaac acaacaacta
      841 ttgaaccaaa atgctaacgc atcaaaccca gaggttgtcg gaaccatatc agttccgatc
      901 agaattcctc ccacaggagg ccaataatct cattatattc gatttaattt tgcattgcaa
      961 acttaacatg ctcgctaccg ctcgaccctt ttgcttatag caaaaactgc aacggacacc
     1021 gttgcataac cctcggcttc gccgaggggc cgctggcgct cgtcgcttcg ctcctcactg
     1081 taatactcca gcggctacaa aaaaaaatct ccgctccgct ccgatttttc tatatatata
     1141 tattttagat caccggaaca actggaacaa cttttcgatg atctttgccc aggggggaat
     1201 tttttttttt tttttttcaa aaattccagt gattatcact ttcagtgttt atatgaaata
     1261 cgatatatcg taatttagct tcaagtgaac taaatatttc attgtctgat tgatctcaaa
     1321 tcaattatat tatatattat aatataacaa ctgaatcgta taactatgca attgaatttg
     1381 gttcttgtga aacgaataat tcacaaacag gagaacctgg taaagttcct cccgaaaaag
     1441 taaaagaaac tggagtaact atgctagcag ttcctgaaac tctcagagta aaattcattg
     1501 ccaacactgt agaacctgtg tttccaccag tagtaagtat atttgcgcta ccgcttgtat
     1561 aaatagataa ttgagtacaa ttacccaaag taattgtcgg tgttgcaaca tttgctgctg
     1621 tcccaccata ccacacaaaa acctgataca cctgaccagg ttgtaatgta ttcgggaatg
     1681 taattcccgt tgtagatatc gttaaaccga tattatcaaa cctattggct tgaacggttc
     1741 caaaataagc tgatgtatta acattcgtat tagaataata atgagacaaa ccagcacctt
     1801 gatcagtgat tgataaatca attggtttca ttaattctat atcatagcta acatgtaatt
     1861 caccaatcac cgctaccgct tgtgatccta cagttgcaat ttgaaaattg ccgatatcat
     1921 aagttttgat atcttgatta gccggtaaaa caccagttct aacataaaac ttaccctctt
     1981 gtagggtctg tttaggatca cattcaatca tatgcatcac attacacgaa ggtttcgaag
     2041 aagtagtgaa ctcattattt tccatctgtt gcttcgatga aaaattagat gatgaagcat
     2101 tataattagt tcctaatatt acagttccca gtgcagtgtt tgtagaatta agagcatccg
     2161 ctgatgtact tacaaacgta aatatcatac catgtaactt atactgttga aaattttgtg
     2221 ctaccgctga caaccatgga aaagtagacg acaatccagg atttatatta taattctgga
     2281 ttgaaaatgc aatagaacta gaaatatccg ttatatattc cctatgacat attctcacac
     2341 cagtatgtgt attttgaact gctggtacag catcacctga caataatttt acgctattgt
     2401 tagctaacgt attatattga atcttataat cacctaaacc ggtgatttta cccagaagat
     2461 cccctgccaa gcctccaagg cttgagcctg ctgctgctcc cgcaggtcct cctaaaaatg
     2521 tacctgctat accacctaac gctgaagtta atggtttaaa attaaaactc ctcttcctag
     2581 gcgctgaaga ggataatgtc ttcttagatg aagaagaaac cctaggttta ggtggtggtg
     2641 gaggtggagg tggaacatta tacataggtt tacgattata ataattccta ctgtaataac
     2701 tgttagtata agaaggacga cgatagttgc gatatccaga agagtaggac attgttgact
     2761 tttagagaaa aaaatttttt tttaaggaaa aaattgaaaa aaaaaaaaaa tttttttccc
     2821 cccacctaaa agttccggt
//
LOCUS       CruV-97                 2839 bp    DNA     circular     24-FEB-2020
DEFINITION  Crucivirus-97 isolate GP1_93008.
ACCESSION   CruV-97
KEYWORDS    .
SOURCE      Crucivirus-97
  ORGANISM  Crucivirus-97
            Unclassified.
REFERENCE   1  (bases 1 to 2839)
  AUTHORS   de la Higuera,I., Kasun,G.W., Torrance,E.L., Pratt,A.A.,
            Maluenda,A., Colombet,J., Bisseux,M., Ravet,V., Dayaram,A.,
            Stainton,D., Kraberger,S., Zawar-Reza,P., Goldstien,S., Briskie,J.,
            White,R., Taylor,H., Gomez,C., Ainley,D.G., Harding,J.S.,
            Fontenele,R.S., Schreck,J., Ribeiro,S.G., Oswald,S.A., Arnold,J.M.,
            Varsani,A., Enault,F. and Stedman,K.M.
  TITLE     Direct Submission
  JOURNAL   Submitted (24-FEB-2020) Biology, Portland State University, 1719 SW
            10th Ave, Portland, Oregon 97201, USA
COMMENT     Bankit Comment: ALT EMAIL:ide@pdx.edu
            Bankit Comment: TOTAL # OF SEQS:451
            Bankit Comment: GAP: est. length - alignment

            ##Assembly-Data-START##
            Assembly Method       :: MetaSPAdes v. 3.12; IDBA-UD v. 1.1.1;
                                     MEGAHIT v. 1.0.3
            Sequencing Technology :: 454; Illumina
            ##Assembly-Data-END##
FEATURES             Location/Qualifiers
     source          1..2839
                     /organism="Crucivirus-97"
                     /mol_type="genomic DNA"
                     /isolate="GP1_93008"
                     /isolation_source="Water"
                     /country="New Zealand"
                     /collection_date=2014
     CDS             1..1371
                     /codon_start=1
                     /product="Putative CruV Capsid Protein"
                     /translation="MPYKKTAKSSKPVKSSKPAKRYSKSYSKSYVRKAPVDHGPSKYL
                     ATGLSTLGGMIGANLAGPPGALAGFTAGALVGDTIRDITGYGDYHVQSNTLVMGQPPG
                     VRNSARPGGSIILSHKEYIGDVVSSSSANTFKIETFPINPGVDKTFPWLSQVASNFQE
                     YSIRGMVFHFRSMSADALNSTNTALGSVILATQYNAAEPAFTSKSEMENAQFSNSIKP
                     SQSCLHLIECARSSSVLSNLYVRSDAVDSSSQDIRFYDLGNFSIASTGCQGTSVNLGE
                     LWVSFELELMRPKLYAELGETIDYYNAYTLTGVTNALPLGTADFVNSDNSNLGVVISA
                     SVSSQLVTFPASSAPKSYMIRLFWRGSRTASLDSPALTYTNCNANTNIFVYTSAGAEI
                     QSLQSPDDNANAQSIYLEECVAIQTSGSNKIPTISFGSAGVLPASVVVFNITIMQIPN
                     TYAFTP*"
     CDS             complement(1553..2593)
                     /codon_start=1
                     /product="Putative Replication Associated Protein"
                     /translation="MLNVSAQHSQKGNTGPSVLNDNLILASNIKKKDDGKKSRAWCWT
                     SYEIEKIPLLDNIRYVCYSPEICPTTQRNHWQGYVYFKDAVTISAIQKKFKANNWVFG
                     KLKKALGTSTENRIYCGFDRYEKTVNGVLKIKEKNPQFFEWGCIPMQGVHDLKSMTDD
                     IASGKITPLEILKENPFSYHMYGRVLRDAVYARSAGKERTWMTKGIWYYGPTGTGKSL
                     KAREGTDKTNRYNLVVEDNGFWNGYTGQETVIIDDFRGEIKFAQLLKIVDMHEMNVKI
                     KGGDPVPLLAKTVIITSPLHPRDVYFNVLSDDDNFDQFERRFEIVHLTKKYDKHIEVP
                     EIDIHSDFFVFA*"
     misc_feature    2542..2566
                     /note="stem-loop"
     misc_feature    2551..2559
                     /note="nonanucleotide"
ORIGIN
        1 atgccatata aaaagactgc gaaatcatct aaacctgtga aatcatctaa acccgcgaag
       61 aggtactcga agagttactc gaagagttat gttcgaaaag ctcctgttga tcatggaccg
      121 tcaaagtatc ttgcaacggg tttgtcgact cttggtggta tgataggggc taatttagcc
      181 ggtccacctg gcgcacttgc aggttttaca gcaggtgcac ttgtgggcga cactatacgt
      241 gacatcaccg gatatggtga ttatcatgtt caatctaata cattagttat gggacaacct
      301 ccaggtgtcc gtaattcggc acgacctgga ggttcgataa ttttatctca taaagagtat
      361 atcggtgatg tagtttctag ctcatccgcc aatactttta aaatcgaaac cttccctatc
      421 aacccaggag tcgataaaac atttccctgg ttgtcacaag tagcttctaa cttccaagaa
      481 tatagtattc gagggatggt ctttcacttt aggagtatgt cagcagatgc cctaaattca
      541 acaaacactg ctctaggttc tgtgattcta gcgacacaat ataatgccgc cgaaccagct
      601 tttacaagta aaagtgaaat ggaaaatgcg caattctcaa atagtataaa gccttcacaa
      661 tcgtgtctac atttaataga atgtgcacgg tcatcatctg tcctttctaa tttatatgtc
      721 agatctgatg ccgttgattc atcttcacaa gatattagat tttatgactt aggtaatttt
      781 tccattgcat ctacaggatg tcaagggaca tccgtaaatt tgggcgaatt atgggtgtct
      841 ttcgagctcg aactcatgcg cccgaaattg tacgcagaac tcggagaaac tatagattat
      901 tacaacgctt atactttaac cggtgtaacg aatgcgttgc ctttaggaac tgcggatttt
      961 gtcaactctg acaatagcaa tcttggtgtt gttatttctg ctagcgtttc ttcacaattg
     1021 gtcacttttc cggcatcgtc tgcgccaaag tcttatatga taagactgtt ctggcgtggg
     1081 tctagaactg caagtttaga ctcaccggcg cttacatata ctaattgtaa cgctaataca
     1141 aatatatttg tatacacatc cgcaggagca gaaatacaat cattgcaatc cccagatgat
     1201 aacgcgaacg cacagagtat atacctagag gaatgtgtag caatccaaac gtcagggtct
     1261 aataagatcc cgacaatctc ttttggatcc gcaggggtat tacctgccag tgtagttgta
     1321 tttaacatta ctataatgca aatacccaat acatacgcat ttactcctta atttttttca
     1381 cacagcaaaa ccaagcgaag cataataata agatttatct tattattata atggtacaat
     1441 gcggatatga ttgttctttt ttgtctctgg acaaaaagga atgagaatga agcacgagtt
     1501 cacgtatgtg agtttacgaa cgaagtgtta cgagatcttg caagcagaac cactaagcaa
     1561 aaacaaagaa atctgaatga atatcaattt ctggtacctc tatatgttta tcatattttt
     1621 tggttaagtg tacaatttcg aaccttcttt caaattggtc aaagttatca tcatctgaca
     1681 atacgttaaa gtacacatca cggggatgaa gaggagaagt tataataact gttttagcta
     1741 aaagaggcac agggtcacca cctttaattt ttacattcat ttcatgcata tcaactattt
     1801 taagcaactg tgcgaattta atttcacctc tgaaatcgtc aattataact gtttcttgac
     1861 cggtataacc attccagaaa ccgttatctt ctaccactaa attataacga ttagttttat
     1921 cagtaccttc acgtgctttc aaacttttac cggttcctgt tggtccataa taccaaatac
     1981 ctttcgtcat ccatgttctt tcttttcctg cagacctggc ataaacggca tctctaagta
     2041 cacgaccata catatgataa gaaaaaggat tttcttttaa tatttctaac ggagtgatct
     2101 taccactagc tatgtcatca gtcatgcttt ttaaatcatg cactccttgc ataggtatac
     2161 aaccccattc aaagaactga gggttttttt cttttatttt aagtacacca ttaacggtct
     2221 tttcatatct atcgaaacca cagtaaatac gattttcagt agaagtgcct aaagcttttt
     2281 ttaatttacc aaacacccag ttgttagctt taaacttttt ctgaattgct gagatagtaa
     2341 cggcatcttt aaaatataca taaccttgcc aatgattacg ttgtgtagtt gggcagattt
     2401 caggagaata acaaacatat ctaatattat caagtaacgg tattttttca atttcatatg
     2461 aagtccaaca ccaagctctg ctttttttgc catcatcttt tttcttaata ttagaagcta
     2521 atattaagtt atcatttagc acagaaggtc cagtattacc cttctggctg tgttgtgcag
     2581 aaacattaag catttgaata aatttaatac aagtcaattg tagtcttatc tttaagtcac
     2641 tttatttaag tatcttatat gttcaaacaa aaaactgggt ggtaaatgtt agcatcaata
     2701 ggcatcttta ggctcatgga caaataggca tctttaggct catggacaaa taggcatctt
     2761 taggctcatg gacaaatagg catctttatt tacatggaca aaaaggaatt ttttttatat
     2821 atatagtata ttataaaat
//
LOCUS       CruV-98                 2849 bp    DNA     circular     24-FEB-2020
DEFINITION  Crucivirus-98 isolate 120_312_SvL2_3298.
ACCESSION   CruV-98
KEYWORDS    .
SOURCE      Crucivirus-98
  ORGANISM  Crucivirus-98
            Unclassified.
REFERENCE   1  (bases 1 to 2849)
  AUTHORS   de la Higuera,I., Kasun,G.W., Torrance,E.L., Pratt,A.A.,
            Maluenda,A., Colombet,J., Bisseux,M., Ravet,V., Dayaram,A.,
            Stainton,D., Kraberger,S., Zawar-Reza,P., Goldstien,S., Briskie,J.,
            White,R., Taylor,H., Gomez,C., Ainley,D.G., Harding,J.S.,
            Fontenele,R.S., Schreck,J., Ribeiro,S.G., Oswald,S.A., Arnold,J.M.,
            Varsani,A., Enault,F. and Stedman,K.M.
  TITLE     Direct Submission
  JOURNAL   Submitted (24-FEB-2020) Biology, Portland State University, 1719 SW
            10th Ave, Portland, Oregon 97201, USA
COMMENT     Bankit Comment: ALT EMAIL:ide@pdx.edu
            Bankit Comment: TOTAL # OF SEQS:451
            Bankit Comment: GAP: est. length - alignment

            ##Assembly-Data-START##
            Assembly Method       :: MetaSPAdes v. 3.12; IDBA-UD v. 1.1.1;
                                     MEGAHIT v. 1.0.3
            Sequencing Technology :: 454; Illumina
            ##Assembly-Data-END##
FEATURES             Location/Qualifiers
     source          1..2849
                     /organism="Crucivirus-98"
                     /mol_type="genomic DNA"
                     /isolate="120_312_SvL2_3298"
                     /isolation_source="Water"
                     /country="Norway"
                     /collection_date=2012
     CDS             1..1056
                     /codon_start=1
                     /product="Putative Replication Associated Protein"
                     /translation="MNCIKFIKMLNISAEGCLQGNYETCKQKKMIPHIMIKDDGKKTR
                     NYSWTSYEAEKIPLLEKIKYVCYSPEICPSSGRKHWQGFIHLKDQMTITNLQKYFIKN
                     GWTFGYLCKALGTPDENRIYCGADRYQKNVDGVLKIKEKNPLFFEYGTIPNPGARTDL
                     KIISNNIINGKLHPLDVLNEDPMLYHQYGRTLNMCLIARTFRKKRNWMTKGIWYWGPT
                     GCGKSFKAYEGHDEDTHYELVTDDSGFWQGYYGQETVLINDFRGEIKFAQLLRIVDMY
                     PLKVKVKGCDTVPLLAKTVIITSSQHPRDVYSNVLSDDENFDQFQRRFEIIHMTEKYD
                     KNVEVPEIDIHADFLVF*"
     CDS             complement(1241..2599)
                     /codon_start=1
                     /product="Putative CruV Capsid Protein"
                     /translation="MSYAKAPPKPARTKYVYAPAPPKPARKPKKNYNYTPASAEFRTG
                     AGALGGALGGLLGPAGVPIGAAGGVLAADLFKTITGYGDYRVKSNSLAMGQAPSVRNG
                     SLPGGSIILSHKEYLGDIISSATANTFNLQSFPINPGMSQSFPFLSALAGNYQEYSFR
                     GLVYSFRSMSADALNSTNVSLGSVIMSTNYNSSSADFASKSEMENAQYSNSIRPSESC
                     IHLIECARSSNVLSNLYVRTDAVDRSSEDVRFYDLGKFQIATAAVQGTSVNLGELHVS
                     YEVMLMKPKLWAELGEAGDYYNAYSIGAAMTNALPLGTDEWVESINSNIDITITASLT
                     SQLFTLPTSSPPKSYMFIIKWKGTRTVSLAAPAYTFTNASAALTPFTSLATPTFANAL
                     QAPDTNASSDSTILVNCVCFKTSGNNTVPTISVGAAGVLPASVTLAQIIFIQLPNTYD
                     FS*"
     misc_feature    1622..1643
                     /note="stem-loop"
     misc_feature    1627..1635
                     /note="nonanucleotide"
     misc_feature    complement(1629..1637)
                     /note="nonanucleotide"
     misc_feature    1630..1638
                     /note="nonanucleotide"
     misc_feature    1702..1725
                     /note="stem-loop"
     misc_feature    complement(1709..1717)
                     /note="nonanucleotide"
ORIGIN
        1 ttgaattgta ttaaatttat taaaatgctt aatatttctg ctgaggggtg cttacaggga
       61 aattatgaaa cctgtaagca aaaaaagatg attccacata ttatgataaa agatgacgga
      121 aaaaagacta gaaattattc gtggacttct tatgaagctg agaaaattcc tttgttggaa
      181 aaaataaaat acgtctgtta ttcaccggaa atatgcccat caagtgggcg aaaacactgg
      241 caaggtttta ttcatcttaa agatcagatg acgattacca atttacaaaa atactttatt
      301 aaaaatggtt ggacattcgg ttacctctgt aaggcgttgg gaactcctga tgaaaatcgt
      361 atatattgtg gtgctgatcg ttatcaaaaa aatgtagacg gcgtattaaa aattaaggaa
      421 aaaaacccgc tattcttcga atacggtact atcccaaatc ctggtgctcg taccgattta
      481 aaaattatat caaataatat aattaatggt aaattacatc cgttagatgt tttaaatgag
      541 gaccctatgt tatatcatca atatggtcga acgctcaaca tgtgtttaat cgctaggact
      601 tttagaaaaa aaagaaattg gatgactaaa ggcatttggt attggggtcc aactggatgc
      661 gggaaatcat tcaaagctta tgaaggacat gatgaagata cacactacga attagtaacc
      721 gacgactcag gattttggca aggttactac ggacaagaaa ccgtcttaat aaatgacttc
      781 agaggcgaaa ttaaatttgc ccaattatta agaattgtag atatgtaccc tttgaaagta
      841 aaagtcaaag ggtgtgatac cgtcccgctt ctagctaaaa cggttattat cacatcttca
      901 caacatccac gtgatgtata ctctaatgtt ttatcagacg acgaaaactt cgatcagttt
      961 caaaggagat ttgaaattat tcatatgact gaaaaatatg ataaaaatgt agaagtaccc
     1021 gaaattgaca ttcacgctga ttttttagtt ttttaatttt tgtgttactg ctgctaattc
     1081 tcgtaacact tcgttcgtaa actcacatac gtgaactcgt gcttcattct cattcctttt
     1141 tgtccacgac aaaaaagaac aatcatatcc gcatgaacca ttataataat aagataaatc
     1201 ttattattat gctacgcttg ttatcatacg gtggattatt ttaagaaaaa tcatatgtat
     1261 ttggtagttg gataaaaata atttgagcta aggttaccga ggctggaagt actcccgctg
     1321 caccaactga tattgtaggc acagtgttgt ttcctgatgt tttgaaacaa acacaattca
     1381 ccaaaattgt tgaatctgat gatgcgttgg tgtcaggagc ttgcaatgca tttgcaaaag
     1441 ttggtgttgc caatgatgta aatggggtaa gtgctgctga tgcatttgta aatgtatagg
     1501 caggagccgc caaactcacg gtacgtgtac ctttccattt tattataaac atgtaagatt
     1561 ttggaggact agaagtaggt aaagtaaaaa gctgtgaggt aagagatgca gtaatcgtta
     1621 tatcaatatt actattaatt gattcgaccc actcatccgt tcccaatggt aacgcgttgg
     1681 tcatcgctgc accgatagaa taagcgttgt aataatcccc cgcttctcct aactccgccc
     1741 ataatttggg cttcatgagc atcacctcat aactgacgtg taattctccc aaatttacag
     1801 aagtcccctg tactgctgct gttgcgattt gaaactttcc taaatcataa aatcgtacat
     1861 cctctgacga tctatcaacg gcgtctgtcc ttacataaag attagataat acatttgaag
     1921 atcgcgcaca ttctattaaa tgaatacaag attcactagg acgtatacta ttagaatatt
     1981 gtgcgttttc catctcgctt tttgaggcaa aatccgctga tgacgaatta taattagtac
     2041 tcattatcac cgatcccaac gaaacattgg tactattcaa tgcatccgca ctcatagaac
     2101 ggaatgagta tacgagtcca cgaaatgaat attcttgata attacctgcc aatgccgaca
     2161 aaaaaggaaa actttgtgac atgccaggat taatgggaaa agattgaaga ttaaaggtgt
     2221 ttgccgtggc tgagcttatt atgtccccca agtattcctt gtgtgataaa attatactcc
     2281 ctccgggtaa cgatccattc ctaacactcg gagcttgccc catcgctagc gaattactct
     2341 taaccctata gtcaccatat ccagttatgg ttttaaataa atcagctgct agaacacctc
     2401 cggctgctcc tattgggacc cctgcaggtc ccaataaacc gcctaaggca ccgcctaggg
     2461 cgcctgcacc agtcctaaac tctgcgctag ctggagtgta gttataattc ttctttggtt
     2521 ttcgagctgg ctttggtgga gcaggcgcat aaacgtattt tgtacgagct ggtttgggag
     2581 gagcttttgc ataagacatg attttaataa tattttatat atcttatata cataaaataa
     2641 atatgccctt ttgtccgtga aaataaaagt cccctcctgt ccgtgtaact aaagttccta
     2701 tctttaagtc actttattgt ccatgtgtct aattatgccc ttttgtccgt gaaaaaagaa
     2761 aagcctagtc cttacattac cccatccacc gaattttttg cagaacatat aagatactta
     2821 aataaagtga cttaaagata agactacaa
//
LOCUS       CruV-99                 2860 bp    DNA     circular     24-FEB-2020
DEFINITION  Crucivirus-99 isolate PCD_321.
ACCESSION   CruV-99
KEYWORDS    .
SOURCE      Crucivirus-99
  ORGANISM  Crucivirus-99
            Unclassified.
REFERENCE   1  (bases 1 to 2860)
  AUTHORS   de la Higuera,I., Kasun,G.W., Torrance,E.L., Pratt,A.A.,
            Maluenda,A., Colombet,J., Bisseux,M., Ravet,V., Dayaram,A.,
            Stainton,D., Kraberger,S., Zawar-Reza,P., Goldstien,S., Briskie,J.,
            White,R., Taylor,H., Gomez,C., Ainley,D.G., Harding,J.S.,
            Fontenele,R.S., Schreck,J., Ribeiro,S.G., Oswald,S.A., Arnold,J.M.,
            Varsani,A., Enault,F. and Stedman,K.M.
  TITLE     Direct Submission
  JOURNAL   Submitted (24-FEB-2020) Biology, Portland State University, 1719 SW
            10th Ave, Portland, Oregon 97201, USA
COMMENT     Bankit Comment: ALT EMAIL:ide@pdx.edu
            Bankit Comment: TOTAL # OF SEQS:451
            Bankit Comment: GAP: est. length - alignment

            ##Assembly-Data-START##
            Assembly Method       :: MetaSPAdes v. 3.12; IDBA-UD v. 1.1.1;
                                     MEGAHIT v. 1.0.3
            Sequencing Technology :: 454; Illumina
            ##Assembly-Data-END##
FEATURES             Location/Qualifiers
     source          1..2860
                     /organism="Crucivirus-99"
                     /mol_type="genomic DNA"
                     /isolate="PCD_321"
                     /isolation_source="Soil"
                     /country="New Zealand"
                     /collection_date=2012
     CDS             1..1578
                     /codon_start=1
                     /product="Putative CruV Capsid Protein"
                     /translation="MLYDIEGNPIRYHPNLDPNEGFRIMHGVDYGPITKVTMVSVPEE
                     INNYKNNYSSHNIDRMSIVKRKSDYTLEQQEAYAKGKAAVKKRTAKRKPARRRKTSYR
                     RGGGSSRDVIYPNIVGRGSYFGDIGHSLGQSAGGYLGRIAGDAVGGLIEPFSGIAGLG
                     DYHVNRNAILVDPPPVINMDPRGGVVVRHREYLGDIRTDLNTAGAFNIASYGINPGDE
                     RTFPWLSQIACNYEEYDMQGCVFEFRSMSANALNSTNTALGTVIMATQYNAASDPFES
                     KAQMENYQYGCSSKPSENLMHPIECEPRQTVLGNLLYTRGGAIPAGDDPRLFDLGNFS
                     IATQGGQATNVNLGELWVTYQVCLLKPKLYSFLGNDINAYYMTNAAATATDPLGANAS
                     DVVTLNNFPNFRAAAVTGGFNSPHSNSTQIALPNSAQFAYQIEVYWGGTIAAAVVVPV
                     LTLTRCAYVDYGVGTAGSTQVPASGVSSIYVIQKFSILTDGSNQTALVVFGVAGTLPT
                     GTTAMRLTITQLPADQF*"
     misc_feature    987..1015
                     /note="stem-loop"
     misc_feature    1002..1010
                     /note="nonanucleotide"
     CDS             complement(1809..2681)
                     /codon_start=1
                     /product="Putative Replication Associated Protein"
                     /translation="MLTSDQHSRGVILDEVCPRESALIRSIQRIHWFFTWNNYPMNFV
                     ELLETSLQPLCKTYVFQQEVGDNGTPHIQGCLTLHKKMRWTQFGLPKSIHWEPTVKKS
                     AAENYCSKEASKAGNTYWWPPLESTPLKLIDTLLPWQQTVCDLCKVLPDDRSVNWIYD
                     PLTCQGKTQLTKYLVAKYNAIALGGCARDATFILANAVNNGRDLNKPTTIIFNFAKDS
                     SSPNYSLIESIKDGMIINGKYESCALIFNSPHIWIFSNKLPNTNGLSKDRWRIWTIID
                     SQLTAYNVAHYTYI*"
ORIGIN
        1 atgttatacg acatcgaagg taatccaatt aggtatcatc cgaacctcga tcctaacgag
       61 ggtttccgta ttatgcacgg agttgattac ggacctataa cgaaagtgac tatggtctct
      121 gtccccgagg aaataaataa ttataaaaat aattattctt cacacaatat agatcgtatg
      181 tcgattgtga agcgtaaaag tgattataca cttgagcaac aggaagctta tgccaagggt
      241 aaagctgctg ttaaaaagag gacggctaaa agaaagccgg cccgtagacg aaaaacatcc
      301 taccgaagag gtggaggttc ttctcgagac gtgatatatc caaatattgt cggtcgagga
      361 agttatttcg gagatattgg ccattcctta ggccaatctg caggaggtta cttaggccga
      421 attgcaggtg atgcagtagg tggtttgata gaaccattct ctggcatcgc aggtttaggt
      481 gattatcacg tgaaccgtaa tgccatatta gttgacccac caccagtcat taatatggat
      541 ccaagaggcg gtgtcgtagt tagacatcga gaatatctag gtgatattcg tactgatttg
      601 aatactgctg gagcattcaa tatcgcttcg tacggtatca atccaggaga cgaacgaaca
      661 tttccttggt tatcgcaaat tgcttgcaat tacgaggaat acgacatgca aggttgcgta
      721 ttcgaattca gatcaatgtc agctaatgct ctgaattcaa ccaatactgc tctcggtacc
      781 gttattatgg caactcaata taacgctgct tccgatccat ttgagtcaaa agcacaaatg
      841 gaaaattacc agtatggttg ctcaagcaag ccatctgaaa atttgatgca tccgatcgag
      901 tgtgaacctc gtcaaacggt acttggtaac ctattgtata caagaggtgg agcaattccc
      961 gcaggagatg atccccgttt atttgacttg ggtaatttct ctatagctac ccaaggaggt
     1021 caagccacta atgtcaactt aggtgaatta tgggtgacct atcaggtttg tttgttaaaa
     1081 ccaaaactgt attcgtttct aggaaatgat attaatgcat attatatgac taatgcagca
     1141 gcaacagcta ctgatccttt aggtgcgaat gcaagtgatg ttgtgactct taataatttt
     1201 ccaaatttta gagctgcagc agtcactggt ggatttaata gtcctcattc aaattccact
     1261 cagattgcat tacccaactc tgctcaattt gcttaccaga ttgaagttta ttggggagga
     1321 acaatcgctg ccgcagttgt tgtccctgta ttaaccttaa ctcgatgtgc atatgtcgat
     1381 tatggtgttg gaactgcagg tagtacacag gtccctgcat ctggagtaag ttccatttat
     1441 gtaatccaaa agttcagtat actgactgat ggatctaacc aaactgcttt agtcgtattt
     1501 ggtgttgctg gtactctgcc aacaggtact acagcaatgc ggttgacaat tacccaattg
     1561 cccgcagatc aattttaatc ttgcgtgtcc acaacgcaca aaaaaatata cccgagcccc
     1621 acatgcgacc agggctatag taattccatt gcatggttac acctcaagga attactttat
     1681 ttttttagat taacggtcag ccgggcttgc cctcgcccct tgtgggcggc tgtccgctac
     1741 acaaaatata taataacttg agggatactc catgcaatgg atgtactata gcccgttggg
     1801 tcccgccatt atatatatgt gtaatgtgcg acattataag cagtcaattg actgtctata
     1861 atagtccata ttctccatct atctttggac aagccattgg tattcggtaa cttattactg
     1921 aatatccaga tatgtggaga attaaatatg agagcacacg attcatattt accattaata
     1981 atcatcccgt ctttgattga ttcaattaaa gaataattag gtgatgatga atctttagca
     2041 aagttaaaga tgatggtggt aggtttgttt aaatctctac cattattcac agcgttagca
     2101 agaataaatg ttgcgtctct agcacatccg ccaagtgcaa tagcattata cttagcaact
     2161 aagtatttag taagctgtgt cttaccttgg catgtaagtg gatcataaat ccaatttaca
     2221 gatctatcgt caggtaacac cttacataga tcgcatactg tctgttgcca aggtaataga
     2281 gtatctatta acttcaacgg agtggactct aatggaggcc accagtaagt gttgccagct
     2341 ttactggctt ccttgctgca gtaattttca gccgctgatt tcttaacagt aggctcccaa
     2401 tggatcgatt taggtaatcc aaattgagtc catctcatct tcttatgcaa ggtcaaacaa
     2461 ccttgaatat gaggtgtacc attatctccg acttcttgtt gaaaaacata agtcttgcat
     2521 aatggttgta gagacgtctc caacagctct acaaaattca ttgggtaatt attccatgtg
     2581 aaaaaccaat gaatgcgttg aatagacctt attaacgcgc tctcacgggg gcagacttcg
     2641 tctagtatta ccccccgtga atgttgatcg gatgtcaaca tgatactaac gggatgttgt
     2701 aaaaccttac aatcatcatc gtgtgcaata ttatctgcta aagccagtaa taaattgtca
     2761 ctatctctaa gtatgtctcc atgcgtctcc attttattga tatataaatg agtagagtaa
     2821 atatttaata aataattaaa tatccgtata tatatgtata
//
LOCUS       CruV-100                2867 bp    DNA     circular     24-FEB-2020
DEFINITION  Crucivirus-100 isolate 120_667_IR1_3296.
ACCESSION   CruV-100
KEYWORDS    .
SOURCE      Crucivirus-100
  ORGANISM  Crucivirus-100
            Unclassified.
REFERENCE   1  (bases 1 to 2867)
  AUTHORS   de la Higuera,I., Kasun,G.W., Torrance,E.L., Pratt,A.A.,
            Maluenda,A., Colombet,J., Bisseux,M., Ravet,V., Dayaram,A.,
            Stainton,D., Kraberger,S., Zawar-Reza,P., Goldstien,S., Briskie,J.,
            White,R., Taylor,H., Gomez,C., Ainley,D.G., Harding,J.S.,
            Fontenele,R.S., Schreck,J., Ribeiro,S.G., Oswald,S.A., Arnold,J.M.,
            Varsani,A., Enault,F. and Stedman,K.M.
  TITLE     Direct Submission
  JOURNAL   Submitted (24-FEB-2020) Biology, Portland State University, 1719 SW
            10th Ave, Portland, Oregon 97201, USA
COMMENT     Bankit Comment: ALT EMAIL:ide@pdx.edu
            Bankit Comment: TOTAL # OF SEQS:451
            Bankit Comment: GAP: est. length - alignment

            ##Assembly-Data-START##
            Assembly Method       :: MetaSPAdes v. 3.12; IDBA-UD v. 1.1.1;
                                     MEGAHIT v. 1.0.3
            Sequencing Technology :: 454; Illumina
            ##Assembly-Data-END##
FEATURES             Location/Qualifiers
     source          1..2867
                     /organism="Crucivirus-100"
                     /mol_type="genomic DNA"
                     /isolate="120_667_IR1_3296"
                     /isolation_source="Water"
                     /country="Norway"
                     /collection_date=2011
     CDS             1..1203
                     /codon_start=1
                     /product="Putative Replication Associated Protein"
                     /translation="MTLLRASHWCDSNAVGHLEPNGSMDPMESKRPNGTKWIGRCRHA
                     DERSLAKHWVFTAWDEPDITDDLPDWATFISFGREVCPDTRRVHWQGYVELAKKARVG
                     PIRKLFSDVGVTSIWLAMRLGNPCQARDYTMKDGDYVELGELSSDASHQGQRTDIQRI
                     IDAVDDRTLTSVVGGCRSGLIQNWQQLRIVDRLIASCEPVRAWRPDIVVHWGPSGTGK
                     SRLAEHLFPNAYRAPACTPRWWPKYDGHDAVIVNEFRGSWLPFHELLAFTDRYEYQLE
                     YKGGHRQLIAHTMVFTTPLHPRDWYPNCREQLAQLLRRISVIRYFFAEVDERSDVSDI
                     KYTETTGLALPLTRPFVPLQRLDSSNLVVPDLDPEVGVGNIALLAPRADNVGTYPDFA
                     DVLADLGL*"
     CDS             complement(1271..2620)
                     /codon_start=1
                     /product="Putative CruV Capsid Protein"
                     /translation="MPKVYTTASGRPYTIVNGKRKYGAAAPAAPRARAPRARAPRAPR
                     QLPALPPPYAVAYNNPPVPKSEVDQWFDYGQKAGAAVGGFGKDLARVFGFGAYHIKQN
                     VLTGRLPRVMNIPQGGGTIIRFQEYLGDITTSAVANTFDIKQFDINAGLFATFPWLSQ
                     IAQNYEQYSFEGLLFEFRSTSADALNSTNTALGSVMLATNYDSSDPPFGSKGEMLNYE
                     YSTSIKPAESCVHMVECAPRQTVLTELYTRPGAVPTGDDQRFYDLGKFQIATTGFQGT
                     SVNVGELHCTYQVRLLKPKLHSALGNGISFAQMGNSTGVAAGTPLGTTTGWSSVASVQ
                     TLAIVFSSATLITFPATNVIQQYLLRYGIQGTVAGALVYPVLTAANMTIGTGALQTAT
                     FPVTPTSATSGVMHVWLKTTPNGLAPTLTFGVGGTFPTGTTSCAIGIIQVPDDVTIT*
                     "
ORIGIN
        1 atgactctgc ttcgcgcgtc acattggtgt gactccaatg ccgtcgggca tttggagcct
       61 aatgggtcca tggatcctat ggaatccaag cgacccaacg gcaccaagtg gattgggcgc
      121 tgtcggcatg ccgacgagcg ctcgcttgcc aagcactggg tgttcaccgc ttgggacgag
      181 ccagacatca ctgatgactt gcccgattgg gctacgttca tctccttcgg tcgtgaggtc
      241 tgccccgaca cgcggcgcgt gcattggcaa ggctacgtcg agctagcgaa aaaggcccgg
      301 gtgggcccca ttcgcaagct cttctctgac gttggtgtca cgtccatctg gcttgctatg
      361 cgtcttggca atccttgtca ggctcgcgac tatacaatga aggacggtga ctacgttgag
      421 ctcggtgagc tctcatctga tgcttcgcac cagggtcaac gtacagacat tcagcgcatc
      481 atcgacgctg tcgatgaccg caccctcacg tctgttgtcg gaggctgtcg ctccgggctc
      541 atccagaact ggcaacagct gcggatcgtt gatcggctta ttgcgtcctg tgagcctgtc
      601 agggcttggc gccccgacat cgtcgtccat tggggtccct caggtactgg caagtcacga
      661 ctcgccgagc acctgttccc caatgcgtac cgcgcacctg cgtgcactcc ccgttggtgg
      721 cccaagtacg acggtcacga tgctgtgatc gtgaacgagt ttcgtgggtc atggctgcct
      781 ttccacgaac tcttagcgtt caccgaccgt tatgaatacc agctcgagta caagggtggt
      841 caccgtcagc ttattgctca caccatggtg ttcacaacgc ctcttcatcc tcgcgactgg
      901 tatcctaact gccgtgagca gcttgcgcag ctccttcggc gaatctcggt catccgttac
      961 ttctttgcag aagtagacga acgctctgac gtctctgaca tcaagtacac cgagaccact
     1021 gggttggcgc ttccgcttac taggcccttc gtgcctctac agcggctcga ttcttcgaat
     1081 ctcgtggttc cggatctgga tccggaagtt ggggtaggta atattgcgtt gctggcacct
     1141 cgtgccgaca acgttgggac ctaccccgac tttgctgacg tcctcgccga ccttggtctc
     1201 tgaggactcc aaaatgcgct cttgcgttcc gcgcggatcc actccgtgca tcccgcttct
     1261 tttgtgttgt ttacgttatc gtgacatcgt ccgggacctg tatgatccct atcgcgcatg
     1321 acgtggtccc tgttgggaag gttccgccca caccgaaggt tagcgttggc gctaacccgt
     1381 tcggcgttgt ctttaaccac acgtgcatga ctcccgacgt tgccgacgtt ggtgtcactg
     1441 gaaacgttgc cgtctgtaac gcgcccgtcc ctatcgtcat attggctgcc gttagcacag
     1501 gatacactaa cgctcccgcg accgttccct gtatgccata tcgcaatagg tactgctgga
     1561 tcacgttggt tgccgggaat gtgattagcg tcgctgagct aaacacgatt gcgagtgtct
     1621 gcacactcgc tactgaggac cagcccgtgg ttgtccccag tggtgtccct gcggcgaccc
     1681 ctgtggaatt ccccatctgt gcgaagctga ttccgttccc tagggcgctg tgcaacttcg
     1741 gcttcagcag tcgcacctgg taggtgcaat gcagctcgcc tacgttcacg ctagtccctt
     1801 ggaaaccggt tgttgcgatc tggaattttc ccaggtcgta aaacctttgg tcgtcccccg
     1861 ttggcacggc gcccggccgt gtgtaaagct ccgtcaacac tgtttgtcta ggggcgcact
     1921 ccaccatgtg cacgcagctt tcagccggtt tgatgcttgt gctgtactca tagttcagca
     1981 tctcaccttt cgacccgaac ggtggatcac tgctgtcata attcgtcgcc agcatcacgc
     2041 tgcccagtgc ggtgtttgtg ctgttaagcg cgtctgccga cgtgctccga aactcgaaca
     2101 acagtccttc gaagctgtac tgttcgtagt tttgtgctat ctgcgatagc catgggaacg
     2161 tggcaaacaa acctgcattg atatcgaatt gcttgatatc aaaggtgttc gctactgccg
     2221 acgttgtgat gtctccaaga tactcttgga aacggataat ggtccctccc ccttggggga
     2281 tgttcattac ccttgggagc cgtcccgtca gcacattctg tttgatgtga taggctccga
     2341 agccgaagac cctggcgagg tcttttccaa agccccctac ggccgctccg gctttttgtc
     2401 catagtcgaa ccattggtcc acctctgact ttgggactgg ggggttattg tatgccacgg
     2461 catacggtgg tggtagcgct ggcaactgac gaggcgctcg cggggctcga gctcgtggtg
     2521 cccttgcgcg cggcgctgcc ggcgcggcgg caccatactt gcgcttgcca ttcactattg
     2581 tgtacggcct accactggct gttgtataca ctttgggcat atcgggtgcc cgtagccggg
     2641 cacctctaac tgcccgccat gtttggcttt ggacggacgt gatcctcggg gcccagaccc
     2701 atcaccctct tgtcactggg cccggggaca agccccgggc ctgccggcgg cacgttcgag
     2761 agcgacgggt cttaagacca ctgtggtacg tcctgttggg aaagtggcgg gtcccacttg
     2821 tccgcgccct gcattaatta cgccggcggc tcttatctgc gggctac
//
LOCUS       CruV-101                2871 bp    DNA     circular     24-FEB-2020
DEFINITION  Crucivirus-101 isolate 120_1180_SvL1_3295.
ACCESSION   CruV-101
KEYWORDS    .
SOURCE      Crucivirus-101
  ORGANISM  Crucivirus-101
            Unclassified.
REFERENCE   1  (bases 1 to 2871)
  AUTHORS   de la Higuera,I., Kasun,G.W., Torrance,E.L., Pratt,A.A.,
            Maluenda,A., Colombet,J., Bisseux,M., Ravet,V., Dayaram,A.,
            Stainton,D., Kraberger,S., Zawar-Reza,P., Goldstien,S., Briskie,J.,
            White,R., Taylor,H., Gomez,C., Ainley,D.G., Harding,J.S.,
            Fontenele,R.S., Schreck,J., Ribeiro,S.G., Oswald,S.A., Arnold,J.M.,
            Varsani,A., Enault,F. and Stedman,K.M.
  TITLE     Direct Submission
  JOURNAL   Submitted (24-FEB-2020) Biology, Portland State University, 1719 SW
            10th Ave, Portland, Oregon 97201, USA
COMMENT     Bankit Comment: ALT EMAIL:ide@pdx.edu
            Bankit Comment: TOTAL # OF SEQS:451
            Bankit Comment: GAP: est. length - alignment

            ##Assembly-Data-START##
            Assembly Method       :: MetaSPAdes v. 3.12; IDBA-UD v. 1.1.1;
                                     MEGAHIT v. 1.0.3
            Sequencing Technology :: 454; Illumina
            ##Assembly-Data-END##
FEATURES             Location/Qualifiers
     source          1..2871
                     /organism="Crucivirus-101"
                     /mol_type="genomic DNA"
                     /isolate="120_1180_SvL1_3295"
                     /isolation_source="Water"
                     /country="Norway"
                     /collection_date=2012
     misc_feature    join(1..9,2857..2871)
                     /note="nonanucleotide"
     misc_feature    1..19
                     /note="stem-loop"
     CDS             complement(80..1180)
                     /codon_start=1
                     /product="Putative Replication Associated Protein"
                     /translation="MDKVKIEVEKTGDKVFPGGRGRIANQKLMLTYKTHINKEGLIEF
                     LTDISERKGYTMVFCRVAHENGEGDTVTPYKHTHVVVDFGGKIDTTNIRMFDWKVNGD
                     HRDDIHPNIKPCAAGKRCWVNFVLYIAKEDPENEDLLDEYGDQKKVTVDELQACKSLK
                     DCFDKHGKNFNDILGMERLYNLGRNTLKRFDWEPTTEWQREIVKLNEVNAGHREIRWL
                     WEKKGCVGKTVLCKYMEFKFPDRWRACRDLGTSKDCSTNIMGFFESGWDGHGILINLS
                     RTSSHDKRMYAYLEDIKDGMVTVQKYKGGSKLFDNPHVIVFANFPPRVDLLSLDRWDI
                     RQIDGDIAVKENAWQMYAEQLGSEEGLYFPDE*"
     CDS             1307..2740
                     /codon_start=1
                     /product="Putative CruV Capsid Protein"
                     /translation="MPRRSRESYQPRRQYSDAEKIAYYKRLANARVSGYGDYKINRAP
                     AQKSYSNRRATYYKYPGTGRNIGRALGGTVGNLISPGVGGIIGSELGGYAGQGAQALV
                     KRITGFGDYTVSKNSLMYNQDAVPEFSVNNQRCTMIAHREFISDIKSSITFESTEYRI
                     NPAIASTFPWLANVAENYEQYVVQGMIFEFKTTSATAIGSTNTSLGTVVMATQYNSLA
                     PIFANKQQMENYEFAQSSVPSQSIIHPIECDPSQTQCGGIFNMFNPLDESGDTRLYDI
                     GRFTIATVGMQADDAIIGELWVTYKICLLKPRLQGSSEVLDMYRLDSATITNTKPLSD
                     SQFAYNGNSNFTTILGTNENEIHFSPSFVGVVLIQYSVQVASNLNDANSPHTVAVNAL
                     AVDVTNIVNPPLTAASIITHSESGSTNTMFITYGYYRILGGFTSTGDPPIVSIIGLTS
                     SGAISYTASSGCLVITAVPPGFADRQA*"
ORIGIN
        1 taatgttaac cccccttttt ggcaagggcc cacttgtgag cccgagcttt tgtcacagat
       61 catgtctaat agacattatc tactcatcag ggaagtagag cccctcttcg ctccctagct
      121 gttcagcgta catctgccaa gcgttttcct tgacagcaat gtcgccatct atttgcctaa
      181 tatcccatct gtcgagagac aagaggtcca cacgtggagg gaaattagcg aacacaatca
      241 catgcggatt gtcaaatagt ttactgcccc ccttgtattt ttggacagtt accattccat
      301 cctttatgtc ttcaaggtat gcatacattc ttttgtcatg tgaagatgtc ctcgagagat
      361 ttatcaagat accatgacca tcccatccac tttcgaagaa acccatgata ttagttgagc
      421 aatctttact agttcccaaa tctctacatg ctctccatct gtcagggaat ttaaattcca
      481 tatacttaca gagtactgtc tttcctacac accccttctt ttcccataac caccgtattt
      541 ctctatgccc cgcatttacc tcattgagct ttacgatttc tctctgccat tcagttgtag
      601 gctcccagtc aaatcttttc aaggtatttc ttcctaagtt gtatagtctc tccataccca
      661 gtatgtcatt gaagttcttc ccatgtttgt caaagcagtc cttcaaactc ttacacgcct
      721 gtaactcatc gacggtcact tttttttggt caccatattc atcgagtagg tcctcatttt
      781 caggatcttc ctttgcgatg tacaatacga agttaaccca acacctctta cccgcggcac
      841 agggtttgat gttagggtgg atatcatccc ggtgatctcc atttaccttc cagtcgaaca
      901 tcctgatatt agtagtgtcg attttgcccc cgaagtcgac cactacgtga gtatgcttat
      961 aaggagtaac agtatcaccc tctccgtttt catgagctac tcgacagaat accatggtgt
     1021 aacccttccg ctccgagata tcggttagga actctatcaa tccctctttg ttaatgtgag
     1081 tcttgtaggt tagcatcaat ttctggttag caattctgcc tcttccccca gggaacactt
     1141 tgtctcctgt cttctctact tctattttta ccttgtccat atgtatgtat gtgttgtttg
     1201 tatgtatact ataatccttt aagagacctc tataaggaca cttcccccta aatatacttt
     1261 aacggaatat ctatagtcct aaaagcctaa acgttcccta aagtaaatgc cacgcagatc
     1321 aagagaatcg taccaaccac gaagacagta ctctgacgca gagaagatag cgtattacaa
     1381 gagactagca aatgcaagag tctcaggcta tggagactac aagatcaata gagcaccagc
     1441 tcaaaagagc tacagtaata gacgcgccac atattacaag tacccaggca ccggacgtaa
     1501 cataggcaga gccctaggag gaacagtagg taatctcata tccccaggag taggtggtat
     1561 aataggtagt gaattaggcg ggtacgccgg tcaaggagcc caagcattag ttaagcgaat
     1621 cacaggattc ggagattaca ctgtctccaa gaactctcta atgtacaacc aagacgcggt
     1681 acctgaattt agcgtcaata accaacggtg cactatgatc gcccatcgag aattcatctc
     1741 agatatcaag tcttcaatca ctttcgagtc aaccgagtac aggattaacc ccgccatagc
     1801 ctcgactttt ccatggcttg cgaacgttgc agagaattac gaacagtacg tcgtccaagg
     1861 aatgatattt gagttcaaga cgacctcagc aacagcaata ggatctacta atacgtcctt
     1921 aggtacagtt gttatggcta ctcaatacaa ctccttagcc cctatcttcg ctaataagca
     1981 acagatggag aactacgaat ttgctcaatc atcagtacct tctcaatcaa tcattcatcc
     2041 tatagaatgc gacccttctc agacccaatg tggaggtatc ttcaacatgt tcaatcctct
     2101 agatgaatca ggagacacca gattatacga cataggacgc ttcactatag ctaccgtagg
     2161 aatgcaagca gatgatgcaa taattggaga attatgggta acatataaga tatgtcttct
     2221 taagcccaga ctacaaggta gcagtgaagt tttagatatg tatcgacttg actctgcaac
     2281 tattaccaac actaagccac ttagtgacag tcaattcgcg tataacggta acagtaattt
     2341 caccaccatc ttaggaacta acgagaatga aatacacttt agcccatctt ttgtaggtgt
     2401 agtcctcatt caatactcag tacaggtcgc ttctaacctt aacgatgcta actcaccaca
     2461 tacagttgct gttaatgcct tggccgtcga tgtaaccaat atcgtcaatc ctcctcttac
     2521 tgcagctagt ataattactc actccgaatc aggatccact aacaccatgt tcatcaccta
     2581 cggatattac cgtattctag gaggatttac ctctacagga gacccaccta tagtctctat
     2641 tatcggactc acaagttcag gtgctatctc atacaccgct tcatctggat gtctagttat
     2701 tactgcagtt cctccaggct ttgccgatag acaagcttaa atcattgaat actcacatag
     2761 tatgtgaaat atgcgcagca agaggagcga agcgactcgt tatcggaacc gcaggtgccc
     2821 ggagcgaagc ggagggggag cgaagcgacg ggtgaaaaaa ggggggaaat t
//
LOCUS       CruV-102                2874 bp    DNA     circular     24-FEB-2020
DEFINITION  Crucivirus-102 isolate PCD_311.
ACCESSION   CruV-102
KEYWORDS    .
SOURCE      Crucivirus-102
  ORGANISM  Crucivirus-102
            Unclassified.
REFERENCE   1  (bases 1 to 2874)
  AUTHORS   de la Higuera,I., Kasun,G.W., Torrance,E.L., Pratt,A.A.,
            Maluenda,A., Colombet,J., Bisseux,M., Ravet,V., Dayaram,A.,
            Stainton,D., Kraberger,S., Zawar-Reza,P., Goldstien,S., Briskie,J.,
            White,R., Taylor,H., Gomez,C., Ainley,D.G., Harding,J.S.,
            Fontenele,R.S., Schreck,J., Ribeiro,S.G., Oswald,S.A., Arnold,J.M.,
            Varsani,A., Enault,F. and Stedman,K.M.
  TITLE     Direct Submission
  JOURNAL   Submitted (24-FEB-2020) Biology, Portland State University, 1719 SW
            10th Ave, Portland, Oregon 97201, USA
COMMENT     Bankit Comment: ALT EMAIL:ide@pdx.edu
            Bankit Comment: TOTAL # OF SEQS:451
            Bankit Comment: GAP: est. length - alignment

            ##Assembly-Data-START##
            Assembly Method       :: MetaSPAdes v. 3.12; IDBA-UD v. 1.1.1;
                                     MEGAHIT v. 1.0.3
            Sequencing Technology :: 454; Illumina
            ##Assembly-Data-END##
FEATURES             Location/Qualifiers
     source          1..2874
                     /organism="Crucivirus-102"
                     /mol_type="genomic DNA"
                     /isolate="PCD_311"
                     /isolation_source="Soil"
                     /country="New Zealand"
                     /collection_date=2012
     CDS             1..1353
                     /codon_start=1
                     /product="Putative CruV Capsid Protein"
                     /translation="MNRYAYSSNRAPRRQAYQRPHPSYPVVSGQGAYAHRLAKRVYDQ
                     AKARKTKSAYLAPALGVIGGVAGSRFGKPGSMVGAAIGSAVGQAIHQVSGLGNYKVNS
                     NVLYEGAQIPKINNKSNIPNGTVIRHKEYLCDIISSSTANTFNLQSFLINPGQAATFP
                     WLSQIAANYDEYILQGCLFEYRSMSADALNSTNTALGTVIMATEYNAALANFASKGEM
                     EAYEFSMSEKPSCSMIHPIECDPRQNPMKELYIRSGAVPSGQDQRLYDLGNFQIATTG
                     CQGTSVNLGELWISYQVCLLKPKLYATLGLAGAYYHSTTITGVANATPLGSGPVATSS
                     SNLSVTITSGTTINFPLDAVPQTYYVYIHWVGGAAAWVPPTVSGGGGLLFSSVLPGGV
                     LDAPQSGLAGTTRASKSISCVSPGQYAASSIVLGAAGTLPAAVTAFQLIILQMPNTAT
                     *"
     CDS             complement(1706..2689)
                     /codon_start=1
                     /product="Putative Replication Associated Protein"
                     /translation="MANVEIVETGEILMEGNTKSIKISAGDVLSSKVKGSKRVGKKKQ
                     DVQRKYWAFTLFYLDDVMVETVETHLKPVCDKYVIGREVCPTTGRKHLQGFIALKGKG
                     KRFSQVRDLIKCEWHLEPCNGSEEQNIKYCSKEGNYVKHGMKIDVEVITNLFPYQKLV
                     EDIIKGKPDKRAVYWFYEPTGDIGKSELCKYLIKYYNAILITNGKKTDIVNLVFNRKE
                     DIEFGRTNLIVIDIPRNEYNACSYNAIEEIKNGMVVNTKYETGSFIFNSPHIIVFSNF
                     YPETTKLSKDRWHIYKINKDDKTATRENVIEKKLDLTFSDTDVYSDDSSLW*"
     misc_feature    2620..2658
                     /note="stem-loop"
     misc_feature    2635..2643
                     /note="nonanucleotide"
ORIGIN
        1 atgaatcgtt atgcgtattc ttccaaccgt gctccacgtc gtcaggcgta tcagagacct
       61 catccgtctt atccagttgt atcaggccag ggcgcatatg ctcaccgcct tgcaaagcga
      121 gtatacgacc aggccaaggc gcgtaagaca aagtcagcct acttagcgcc tgctcttgga
      181 gttataggag gtgttgcagg atctaggttt ggaaagcccg gctcaatggt tggcgctgcc
      241 atcggctcag ccgttgggca agcaatacat caggtgagtg gtctcgggaa ttacaaggtt
      301 aattccaacg tattgtatga gggagcgcag atcccgaaga tcaataacaa gtctaatatt
      361 cctaatggca cggtgatccg tcataaggag tacttgtgcg atattatttc gtcatcaacc
      421 gcgaatacat ttaatttaca aagttttctt atcaatcctg ggcaagcagc gacattccca
      481 tggttgtctc aaatcgcagc caattatgac gagtacattt tacagggctg cttattcgag
      541 tatcgctcga tgtcagccga tgcgttgaac agtactaaca cagcgttggg tactgtcatt
      601 atggcaacgg aatacaacgc cgcactagcc aatttcgcat ctaagggtga aatggaggcg
      661 tatgagtttt caatgtctga gaaaccatca tgttcgatga tacatcccat tgagtgtgat
      721 cctagacaga acccaatgaa ggagttatat attcgttcag gggctgtacc atcgggacag
      781 gaccaacgtt tgtacgattt gggtaatttc caaatcgcga ccacgggttg ccaaggtacg
      841 tcggtcaacc ttggagagtt gtggatttca taccaagtat gtcttcttaa gcctaagttg
      901 tatgcgacat taggtttagc aggagcttac taccattcta ctactatcac aggggtagct
      961 aacgctaccc cgttgggtag tggaccagtg gcaacgtcat catctaacct aagtgtaact
     1021 attacatcag gtactacgat taactttcct ttagacgcgg tcccacaaac atattatgtg
     1081 tacatacatt gggttggagg tgccgcagca tgggtaccac caacggttag tggtggaggt
     1141 ggactcttat tttcttctgt gttacctggt ggtgtgctag acgcacccca gtcaggttta
     1201 gcaggaacca caagagcatc taagtctatt tcttgtgtgt ctccaggaca atatgcagct
     1261 agttcgattg ttctaggcgc agccggaaca cttccagctg cggtaacagc attccagtta
     1321 attatactgc agatgcctaa tactgcgaca taacataaca aagagagaca gaggagggtt
     1381 agtgtttata tattacaggt taaggtgtgt accacacctt aacataacaa aaaaaagagt
     1441 acttgaacca ttcatctaca tccttccata atatcatatc ttctcaacct tataattatt
     1501 aacatagcca atataatcta tgataaatgc gttgcgaagc ccgagggtcg aggatagact
     1561 ccattgtctg agcggagcga gtggagacgc cgcagacgag ggcgagcaat cgcatttata
     1621 atatttatct ttggcttaac tcacatacat tattagatta acaacgtatg tattatgtaa
     1681 gtagtgttgt atcaagtacg ttttattacc acagactact gtcatcgcta taaacatccg
     1741 tatcgctaaa agttaagtct aactttttct caataacgtt ttctcttgta gcagtcttgt
     1801 catccttatt tattttataa atatgccatc tatccttaga cagtttagtc gtttcaggat
     1861 agaaattact gaatactata atatgagggg agttaaaaat gaacgaaccg gtttcatatt
     1921 tagtgtttac caccatacca ttttttattt cttcaatagc attatagctg catgcattat
     1981 attcattacg agggatatcg ataactataa ggttggttcg accgaattcg atatcttcct
     2041 tcctgttaaa cacaagatta acgatgtctg tcttcttacc gttggtaatg aggatagcat
     2101 tgtaatattt aataagatat ttacacagtt cagacttacc aatatcacca gttggttcat
     2161 aaaaccaata tacagctcgt ttatcaggtt tacctttgat aatatcttca actaatttct
     2221 gatatgggaa aaggttagtg ataacctcaa catcaatttt cataccatgc tttacatagt
     2281 taccttcttt agaacaatat tttatattct gttcttcaga gccattacat ggctctagat
     2341 gccattcgca ttttattagg tctctgacct gagaaaagcg tttaccttta ccttttaagg
     2401 ctataaatcc ttggaggtgc ttccggccag tggtcgggca cacttctcta ccgataacat
     2461 acttgtcaca gacaggcttg agatgagtct caaccgtctc taccataaca tcatcaagat
     2521 aaaaaagagt aaaagcccag tattttctct gaacatcttg tttcttctta ccaactctct
     2581 tagaaccctt tactttacta gaaagaacat cacccgcgga gatcttaatg gatttagtat
     2641 taccttccat taagatctct cccgtctcta cgatctctac gtttgccata actataccta
     2701 tagcaaatat tatatattta aatcatttta aacgcacgta aaataatatt atatagagtc
     2761 cgttaggctc gcggtgctcg ccttggtgcg attttttgtc catgacagga tcgaagatta
     2821 aaatgtgccg cgaaaatata tagtaaatta ttttctaaac ctatagtatt atca
//
LOCUS       CruV-103                2881 bp    DNA     circular     24-FEB-2020
DEFINITION  Crucivirus-103 isolate 120_1154_SvL1_3295.
ACCESSION   CruV-103
KEYWORDS    .
SOURCE      Crucivirus-103
  ORGANISM  Crucivirus-103
            Unclassified.
REFERENCE   1  (bases 1 to 2881)
  AUTHORS   de la Higuera,I., Kasun,G.W., Torrance,E.L., Pratt,A.A.,
            Maluenda,A., Colombet,J., Bisseux,M., Ravet,V., Dayaram,A.,
            Stainton,D., Kraberger,S., Zawar-Reza,P., Goldstien,S., Briskie,J.,
            White,R., Taylor,H., Gomez,C., Ainley,D.G., Harding,J.S.,
            Fontenele,R.S., Schreck,J., Ribeiro,S.G., Oswald,S.A., Arnold,J.M.,
            Varsani,A., Enault,F. and Stedman,K.M.
  TITLE     Direct Submission
  JOURNAL   Submitted (24-FEB-2020) Biology, Portland State University, 1719 SW
            10th Ave, Portland, Oregon 97201, USA
COMMENT     Bankit Comment: ALT EMAIL:ide@pdx.edu
            Bankit Comment: TOTAL # OF SEQS:451
            Bankit Comment: GAP: est. length - alignment

            ##Assembly-Data-START##
            Assembly Method       :: MetaSPAdes v. 3.12; IDBA-UD v. 1.1.1;
                                     MEGAHIT v. 1.0.3
            Sequencing Technology :: 454; Illumina
            ##Assembly-Data-END##
FEATURES             Location/Qualifiers
     source          1..2881
                     /organism="Crucivirus-103"
                     /mol_type="genomic DNA"
                     /isolate="120_1154_SvL1_3295"
                     /isolation_source="Water"
                     /country="Norway"
                     /collection_date=2012
     misc_feature    join(2870..2881,1..19)
                     /note="stem-loop"
     misc_feature    1..9
                     /note="nonanucleotide"
     CDS             187..1602
                     /codon_start=1
                     /product="Putative CruV Capsid Protein"
                     /translation="MPPYSAVRKRYGRKNGRALVYRSGPAYKRVAIVPAGRATCVPEF
                     PQRTTGQMDRRSKRPRGPNGRFLVGNGAYHHWGPSPGADLGRSVGKWFGFEKQGAALG
                     HFAGTLLGSGDYHSSDRMINTNSIINPSSSPPMLISGTGNGFIIRRREYIKDIVSSSV
                     PNTFVQDTYSINPGQAKTFPWLATVAENFEQYRIRGMVFEFVSLSGDATSTTATSLGY
                     VAMATQYDVLDPPFRNKEDLQNYDMSQSCKPSRNQIHGVECDPGRGVMSELYTRVGAQ
                     PPLSDLRLYDFAAFTIANAAPGVSVTLGELWVSYDIELIKPKLPANPGGSIPSLWLSR
                     VNNTVSPVMGFGQVSTKASGNTAFSCDNTTLTLRDLRIGHKYQVSAYWFCTTAINARP
                     ALGLTSGALPFSMFGNTVGDPNFSSAFAAGAIPDITAMFSITFIAASSTVTVVCTPGT
                     MAAAALFGCNISVVGLDATVT*"
     CDS             join(complement(2224..2824),complement(1843..2162))
                     /codon_start=1
                     /product="Putative Replication Associated Protein"
                     /translation="MPYQLKAKNVFLTYAQTPEGWTKESLLAALSLLPSGVPAGYCIA
                     QESHADGGRHFHALLQFSEPVRTKDARFFDISDIHPNVQGARDPKATATYIQKDGDFI
                     EFGLSVGAKRTWGDLVEGCTSRDEFMLEVSKSFPRDSVLSLEKLEYYAAKKFKPATPP
                     YSPQYTDFTVPETLQAWKDENLFVDKDRPRSLFLVGASRLGKTEWARSLGSHTYFNGM
                     FNLDDWNADADYLIIDDIDWKYVPAKKALFGAQKQFTITDKYIKKKTLLWGKPVIYLC
                     NQDMDVYNSCDEKLWLRDNCDYVILHTKMF*"
ORIGIN
        1 taatattacc ctccatttgc agtccctagt cccctatatg tagcttggtc cccaccagca
       61 aaatggctag gctttgctgt gtcctaaccc taaccctaac atgtccgaat cttgttcgga
      121 tggatcgagg ccctgtataa atacctgtct ttgcattggt taactcctaa ccaaaattac
      181 aaattcatgc caccttattc tgctgtccga aaacgatacg gaaggaaaaa cggtcgcgcc
      241 cttgtctaca gatcaggacc agcgtataaa cgagtggcga ttgtcccagc aggaagagcc
      301 acctgtgtac cagagtttcc ccaacgaaca acgggccaga tggatcgaag atcaaaacgt
      361 ccgcgtgggc ccaatggtcg ttttcttgtc ggtaatggtg cttatcatca ttggggccct
      421 tctcctggtg ccgatttggg tcgaagtgtc ggaaagtggt tcggttttga gaaacaagga
      481 gcagccttgg gtcatttcgc tgggaccctc cttggctccg gagattacca ttcgtccgat
      541 cgtatgatta atacgaattc tatcatcaac ccctcgtctt ccccaccaat gttgatttct
      601 ggcacgggga atggtttcat cattcgccga cgagagtaca ttaaagatat tgtgtcttca
      661 tctgttccga atacgtttgt tcaggataca tattccatca atccagggca agccaagact
      721 tttccttggt tggctacggt tgccgagaat tttgagcagt atcgcattcg tgggatggtg
      781 tttgaatttg tgtcgttgtc tggtgatgct acatcaacga ctgcaacgtc acttggttat
      841 gttgctatgg ctacgcagta tgatgttctt gatcctcctt ttcgtaataa ggaggatttg
      901 caaaactatg acatgtctca gtcgtgtaag ccttctcgca atcaaattca cggtgttgaa
      961 tgtgatccag ggcgtggtgt tatgtctgag ttgtacactc gtgttggagc tcagcctcct
     1021 ttgtctgatt tgcgtttgta cgattttgca gcctttacga ttgcgaatgc ggctccggga
     1081 gtcagtgtaa ctcttggaga actttgggtc agttatgaca tcgaattgat caagcccaag
     1141 ttacctgcca acccaggagg atccattcca tctctttggc tgagtagagt gaacaatacc
     1201 gtgtctccgg ttatgggctt tggtcaggtg tcgaccaagg cctctggaaa tactgcgttt
     1261 tcgtgtgata acacgacgtt gacgctgcgt gaccttcgta ttggtcacaa gtaccaggtg
     1321 tctgcgtact ggttttgtac aactgctatt aacgctcgtc ctgcacttgg attgacaagt
     1381 ggtgcgttgc cgtttagtat gtttggaaac acggttggtg atcccaattt tagttctgcg
     1441 tttgctgcag gggctattcc agacatcaca gccatgtttt cgattacatt tatcgctgcc
     1501 agttccactg ttacggtagt ttgtacaccc ggtacaatgg cggctgctgc ccttttcgga
     1561 tgtaacattt cagttgttgg attggacgcc actgttacct agtgtagcta gttattcttg
     1621 taaataaaac ttgtgttaaa ggaccgtact tcgacagcgt ttacagaaca tccattggcg
     1681 ccctaaggcg ggaacgccgt gggcaagaca gcctcggagc aggtgccgaa caggcatctg
     1741 cgggtataac aaccaagctt tatttttaaa cttgtgttta gggttatttt ttaaaaagaa
     1801 aaaaaacagt cttagggtta ggtaagttat tacaaaatta tattaaaaca ttttagtgtg
     1861 tagaatgacg tagtcacagt tgtctcgtag ccacaacttt tcatcacagc tattatatac
     1921 atccatatct tgattacaaa ggtagatgac cggtttaccc caaagcagcg tctttttttt
     1981 aatgtacttg tcggtgattg taaattgttt ttgggcgcca aacagggctt ttttcgctgg
     2041 tacgtatttc cagtcgatat catcaatgat taagtagtca gcgtcggcat tccaatcgtc
     2101 gaggttgaac atcccattaa agtatgtgtg ggatcccaaa gaccttgccc attctgtttt
     2161 gcctaatatg atttattagt gaacctcaac aatatcatca atgaccaaca ccctttttcc
     2221 cacctagtcg agatgctccg acgagaaaga gtgatcttgg tctgtctttg tccacaaaga
     2281 gattttcgtc tttccatgct tggagagtct caggcacggt gaagtcggtg tattgtgggg
     2341 agtacggggg ggtggcaggc ttgaattttt tagcggcgta gtactccagc ttttccaagg
     2401 ataggacgga gtcacgaggg aatgactttg acacttccag catgaattca tctcgtgaag
     2461 tacacccttc cacgagatct ccccaagttc ttttggctcc aacggacagt ccgaactcaa
     2521 taaagtcccc gtccttttgg atgtacgtgg cagtcgcttt agggtcacgt gctccttgaa
     2581 cgttcgggtg gatatcggag atatcgaaaa atcgagcgtc tttggtccgc accggttcgg
     2641 agaactggag tagtgcgtga aagtgccttc ctccatccgc atgcgactct tgggcgatgc
     2701 agtatccggc aggaactcca gagggcagca gagacaaggc tgcgagcaag gattctttag
     2761 tccatccttc aggtgtttgg gcgtaagtga gaaagacatt tttggctttg agttgatatg
     2821 gcataaagtg ggcggacgaa tttcggacgg cggggactag agcgggaccc aaatggaggt
     2881 a
//
LOCUS       CruV-104                2883 bp    DNA     circular     24-FEB-2020
DEFINITION  Crucivirus-104 isolate 120_649_IR1_3296.
ACCESSION   CruV-104
KEYWORDS    .
SOURCE      Crucivirus-104
  ORGANISM  Crucivirus-104
            Unclassified.
REFERENCE   1  (bases 1 to 2883)
  AUTHORS   de la Higuera,I., Kasun,G.W., Torrance,E.L., Pratt,A.A.,
            Maluenda,A., Colombet,J., Bisseux,M., Ravet,V., Dayaram,A.,
            Stainton,D., Kraberger,S., Zawar-Reza,P., Goldstien,S., Briskie,J.,
            White,R., Taylor,H., Gomez,C., Ainley,D.G., Harding,J.S.,
            Fontenele,R.S., Schreck,J., Ribeiro,S.G., Oswald,S.A., Arnold,J.M.,
            Varsani,A., Enault,F. and Stedman,K.M.
  TITLE     Direct Submission
  JOURNAL   Submitted (24-FEB-2020) Biology, Portland State University, 1719 SW
            10th Ave, Portland, Oregon 97201, USA
COMMENT     Bankit Comment: ALT EMAIL:ide@pdx.edu
            Bankit Comment: TOTAL # OF SEQS:451
            Bankit Comment: GAP: est. length - alignment

            ##Assembly-Data-START##
            Assembly Method       :: MetaSPAdes v. 3.12; IDBA-UD v. 1.1.1;
                                     MEGAHIT v. 1.0.3
            Sequencing Technology :: 454; Illumina
            ##Assembly-Data-END##
FEATURES             Location/Qualifiers
     source          1..2883
                     /organism="Crucivirus-104"
                     /mol_type="genomic DNA"
                     /isolate="120_649_IR1_3296"
                     /isolation_source="Water"
                     /country="Norway"
                     /collection_date=2011
     misc_feature    join(2872..2883,1..17)
                     /note="stem-loop"
     misc_feature    1..9
                     /note="nonanucleotide"
     CDS             209..1609
                     /codon_start=1
                     /product="Putative CruV Capsid Protein"
                     /translation="MSVPSTKVSGTINGSYKPWSAYKGVRKPIQGTKRQAERTIARPK
                     KRKTYISGSGDYMIREVGSRKNDYTPFGSVGAKLGSYVGAPGIGWAGGHLLGRILGSG
                     DYKMGPCNLSGNVLTNKTEVPQFSSSARSNIVAHREYIKDISTGSVAGAFNVETIVIQ
                     PGDGSTFPWLSTIAQNYEQYRVHGMVFEFKTTSCDALNSTNTALGTVIMATEYNVNSP
                     AYTNKISMENSEFAQSAKPSQSQMHGVECAPAEGLVRNLFTRSAGLSSTSLALQDKRL
                     YDLANFQIATVGGQAANIVVGELWVTYLIEFLKPQIPITLGGDLDTLKFNKNSAVATT
                     TLDFGLIGLSATGSLGYSIVSAASFTVSKLVIGNLYEFTVAWSASAAITTLPTLSVSS
                     GGTATAAYPTSAGVNLLTVLSTGGGAGVTTAAQGALTFTATSTSATIAITGGILTSGT
                     FYCQGVVSGIDASITN*"
     CDS             join(complement(2490..2826),complement(1774..2402))
                     /codon_start=1
                     /product="Putative Replication Associated Protein"
                     /translation="MAGKFQLSTKSLFLTYPKVPADWTKNLVRDTVYKIASDAGNQPY
                     GYLVARELHEDGTPHFHVLLQFVVPFRTRNERIFDIQAGVDTWHHPNIQSTRDPAACA
                     AYLQKDGMDWNFITEGLVLKATKATWSSILATATTQDEFMEAAKQDFPRDYVLNYEKL
                     EYFAKQHYKAKVEEYVSKFTSFIVPQVLRDWVLESLDPTANRPKSLFLVGASRMGKTE
                     WARSLGCHTYFNHMFNLDDWNHAGRYLVIDDIEWKYFPARKALLGAQSTFTMTDKYRK
                     KVTLAWGKPCIYLCNKDMDVFNSCDEYEWLKDNCVYYNLQGKLFE*"
ORIGIN
        1 taatattacc ccccatttcc caatcccact cccaccccag ggttaggggc ctcgcgtagg
       61 gttacccagg gttagggtcg gagaattaaa tcacgtgagt attttaattc tccgacgtct
      121 ggttccttct ctggtcttca tgctcgtgtc ttggcccttc tcccaaaaat tacacaaaca
      181 caaacacggc ctcagaaatt attagtaaat gtcagtgccg tccacgaaag tttctggcac
      241 tattaacggt tcttataagc cttggtctgc ttacaagggt gttcgtaagc ctattcaagg
      301 taccaagcgt caagctgaaa gaaccattgc ccgcccaaaa aagcgcaaga cctacatatc
      361 cggcagtggc gattatatga ttcgcgaagt tggttctcgc aagaatgact atactccttt
      421 tggttctgta ggagctaaac ttggatctta tgttggtgct ccaggtattg gatgggcagg
      481 tggacatttg ttgggccgta ttttaggatc tggtgactac aagatgggtc cttgtaattt
      541 gagcggtaat gttttgacga ataagactga ggttcctcag ttttctagtt cagccagatc
      601 caatattgtc gctcatcgtg agtatattaa ggatatttcg actggttctg ttgctggagc
      661 ttttaacgtt gaaactattg ttattcaacc tggcgatggc agtaccttcc cttggttgtc
      721 tactattgcc cagaattatg agcagtatcg tgttcatggg atggtgtttg agtttaaaac
      781 tacctcatgt gatgctctta attccaccaa tacggcttta ggaactgtta ttatggctac
      841 tgagtacaat gttaattctc ctgcttatac caacaagata tctatggaaa attctgagtt
      901 tgcgcaaagt gcgaaaccct ctcagtctca aatgcacggg gttgagtgtg ctcctgctga
      961 ggggttggtt aggaatttgt ttactcgatc tgcagggttg tcttctacat ctttagctct
     1021 tcaagacaaa cgtctttatg atttggctaa ctttcagatt gccactgttg gtggtcaagc
     1081 tgctaatatt gttgttggag agctttgggt tacatacttg attgagtttt tgaagcctca
     1141 aattcctatt actcttggtg gtgatcttga tactttgaaa ttcaacaaga attctgctgt
     1201 tgccactact acattggact ttggtttgat aggattatca gctactggtt ctttgggtta
     1261 ttctattgtg tctgcagcat cttttactgt tagtaaattg gtcatcggca atctgtatga
     1321 atttactgta gcatggtcag cttctgctgc cattactact ttgccaacat tgtctgttag
     1381 cagtggagga accgctacgg ctgcttatcc tacatccgct ggtgttaatc ttttgactgt
     1441 tctttctact ggaggaggtg caggtgtgac tacggctgct caaggggctc ttacgtttac
     1501 ggcaacttct acttctgcga ctattgctat tacaggtgga atcttgactt ctggaacatt
     1561 ttattgtcaa ggtgttgtta gtggcattga tgcttctatt acaaattagt tttgccagga
     1621 ttactggcta ggtgtgtgtg cgatatgttt gtattaacct ttagggaaaa ggccttaggg
     1681 accccccgga ctttaggcag ggagggatgg cctttggtcc cgacttgaaa aagaactaat
     1741 aaatgcccgc gggagactgt attctatcta tttttattca aaaagctttc cttgcaggtt
     1801 gtagtagaca cagttgtcct tgagccactc atactcgtca catgagttga agacatccat
     1861 gtccttgttg cataggtaga tgcacggctt gccccaggca agggttactt tcttgcggta
     1921 tttgtcagtc atggtgaatg tagactgtgc tccaagtaag gcctttcttg cagggaagta
     1981 cttccattcg atatcgtcta tcaccagata gcgacctgca tggttccagt catcgagatt
     2041 gaacatgtga ttaaagtatg tatgacagcc caaggatctc gcccattcag tttttcccat
     2101 gcgcgaggct ccgacaagga agagtgattt gggtcggtta gccgtaggat cgaggctttc
     2161 caatacccag tcacgtaata cttgcggtac tatgaaagaa gtaaacttcg atacatactc
     2221 ttcaactttg gccttgtagt gttgcttggc aaagtactcg agcttttcgt aattcaacac
     2281 gtagtcacgt ggaaagtctt gctttgctgc ttccatgaac tcgtcttgtg ttgttgcggt
     2341 tgcaagtatt gaggaccatg tggccttggt tgctttgagg acgagtccct cagtgatgaa
     2401 gtctcctatt tattcatatg aaaagtaagt caatatatca gacatgtcgt ccccatccca
     2461 ctcatcatct ccaaggcaga aaattccact ccagtccata ccatcctttt gcaagtaggc
     2521 cgcacatgct gctgggtctc gcgtgctttg gatgttcgga tgatgccacg tgtccactcc
     2581 agcttggatg tcgaagatgc gctcgttcct tgtccggaag ggtaccacga attggagcag
     2641 cacatggaag tgaggagtcc cgtcctcgtg tagctcccgt gccactaggt atccatatgg
     2701 ttggtttcca gcgtcactgg cgattttgta tacggtgtcg cgtaccagat tttttgtcca
     2761 gtccgcaggc acctttgggt aggtcaggaa cagtgacttg gtggagagtt gaaatttacc
     2821 ggccattttg aagtgaggat gtccggatga gttggtgaat tgattgggag gaatggggga
     2881 tta
//
LOCUS       CruV-105                2883 bp    DNA     circular     24-FEB-2020
DEFINITION  Crucivirus-105 isolate 120_1846_IR2_3297.
ACCESSION   CruV-105
KEYWORDS    .
SOURCE      Crucivirus-105
  ORGANISM  Crucivirus-105
            Unclassified.
REFERENCE   1  (bases 1 to 2883)
  AUTHORS   de la Higuera,I., Kasun,G.W., Torrance,E.L., Pratt,A.A.,
            Maluenda,A., Colombet,J., Bisseux,M., Ravet,V., Dayaram,A.,
            Stainton,D., Kraberger,S., Zawar-Reza,P., Goldstien,S., Briskie,J.,
            White,R., Taylor,H., Gomez,C., Ainley,D.G., Harding,J.S.,
            Fontenele,R.S., Schreck,J., Ribeiro,S.G., Oswald,S.A., Arnold,J.M.,
            Varsani,A., Enault,F. and Stedman,K.M.
  TITLE     Direct Submission
  JOURNAL   Submitted (24-FEB-2020) Biology, Portland State University, 1719 SW
            10th Ave, Portland, Oregon 97201, USA
COMMENT     Bankit Comment: ALT EMAIL:ide@pdx.edu
            Bankit Comment: TOTAL # OF SEQS:451
            Bankit Comment: GAP: est. length - alignment

            ##Assembly-Data-START##
            Assembly Method       :: MetaSPAdes v. 3.12; IDBA-UD v. 1.1.1;
                                     MEGAHIT v. 1.0.3
            Sequencing Technology :: 454; Illumina
            ##Assembly-Data-END##
FEATURES             Location/Qualifiers
     source          1..2883
                     /organism="Crucivirus-105"
                     /mol_type="genomic DNA"
                     /isolate="120_1846_IR2_3297"
                     /isolation_source="Water"
                     /country="Norway"
                     /collection_date=2011
     misc_feature    join(2872..2883,1..17)
                     /note="stem-loop"
     misc_feature    1..9
                     /note="nonanucleotide"
     CDS             209..1609
                     /codon_start=1
                     /product="Putative CruV Capsid Protein"
                     /translation="MSVPSTKVSGTINGSYKPWSAYKGVRKPIQGTKRQAERTIARPK
                     KRKAYISGSGDYMIREVGSRKSDYTPFGSVGAKLGSYFGAPGIGWAGGHLLGRILGSG
                     DYKMGPCNLSGNVLMNKTEVPQFSSSARSNIVAHREYIKDISTGSVAGAFNVETIVIQ
                     PGDGSTFPWLSTIAQNYEQYRVHGMVFEFKTTSCDALNSTNTALGTVIMATEYNVNSP
                     AYTNKISMENSEFAQSAKPSQSQMHGVECAPAEGLVRNLFTRSAGLSSTSLALQDKRL
                     YDLANFQIATVGGQAANIVVGELWVTYLIEFLKPQIPITLGGDLDTLKFNKNSAVATT
                     TLDFGLIGLSATGSLGYSIVSAASFTVSKLVIGNVYEFTVAWSASAAITTLPTLSLSS
                     GGTATAAYPTSAGVNLLTVLSTGGGAGVTTAAQGALTFTATSTSATIATTGGILTSGT
                     FYCQGVVSGIDASITN*"
     CDS             join(complement(2490..2826),complement(1774..2402))
                     /codon_start=1
                     /product="Putative Replication Associated Protein"
                     /translation="MAGKFQLSTKSLFLTYPKVPADWTKNLVRDTVYKIASDAGNQPY
                     GYLVARELHEDGTPHFHVLLQFVVPFRTRNERIFDIQAGVDTWHHPNIQSTRDPAACA
                     AYLQKDGMDWNFITEGLVLKATKATWSSILATATTQDEFMEAAKQDFPRDYVLNYEKL
                     EYFAKQHYKAKVEEYVSKFTSFIVPQVLRDWVLESLDPTANRPKSLFLVGASRMGKTE
                     WARSLGCHTYFNHMFNLDDWNHAGRYLVIDDIEWKYFPARKALLGAQSTFTMTDKYRK
                     KVTLAWGKPCIYLCNKDMDVFNSCDEYEWLKDNCVYYNLQGKLFE*"
ORIGIN
        1 taatattacc ccccatttcc caatcccact cccaccccag ggttaggggc ctcgcgtagg
       61 gttacccagg gttagggtcg gagaattaaa tcacgtgagt attttaattc tccgacgtct
      121 ggtcccttct ctggtcttca tgctcgtgtc ttggcccttc tcccaaaaat tacacaaaca
      181 caaacacggc ctcagaaatt attagtaaat gtcagtgccg tccacgaaag tttctggcac
      241 tattaacggt tcttataagc cttggtctgc ttacaagggt gttcgtaagc ctattcaagg
      301 taccaagcgt caagctgaaa gaaccattgc ccgcccaaaa aagcgcaagg cctacatatc
      361 cggcagcggc gattatatga ttcgcgaagt tggttctcgc aagagtgact atactccttt
      421 tggttctgta ggagctaaac tgggatctta ttttggtgct ccaggtattg gatgggcagg
      481 tggacatttg ttgggccgta ttttaggatc tggtgactac aagatgggtc cttgtaattt
      541 gagcggtaat gttttgatga ataagactga ggttcctcag ttttctagtt cagctagatc
      601 caatattgtc gctcatcgtg agtatattaa ggatatttcg actggttctg ttgctggagc
      661 ttttaacgtt gaaactattg ttattcaacc tggcgatggc agtaccttcc cttggttgtc
      721 tactattgcc cagaattatg agcagtatcg tgttcatggg atggtgtttg agtttaaaac
      781 gacctcatgt gatgctctta attccaccaa tacggcttta ggaactgtta ttatggctac
      841 tgagtacaat gttaattctc ctgcttatac caacaagata tctatggaaa attctgagtt
      901 tgcgcaaagt gcgaaaccct ctcagtctca aatgcacggg gttgagtgtg ctcctgctga
      961 ggggttggtt aggaatttgt ttactcgatc tgcagggttg tcttctacat ctttagctct
     1021 tcaagacaaa cgtctttatg atttggctaa ctttcagatt gccactgttg gtggtcaagc
     1081 tgctaatatt gttgttggag agctttgggt tacatacttg attgagtttt tgaagcctca
     1141 aattcctatt actcttggtg gtgatcttga tactttgaaa tttaacaaga attctgctgt
     1201 tgccactact acattggact ttggtttgat aggattatca gctactggtt ctttgggtta
     1261 ttctattgtg tctgcagcat cttttactgt tagtaaattg gtcatcggca atgtgtatga
     1321 atttactgta gcatggtcag cttctgctgc cattactact ttgccaacat tgtctcttag
     1381 cagtggagga acagctacag ctgcttatcc tacatccgct ggtgttaatc ttttgactgt
     1441 tctttctact ggaggaggtg caggtgtgac tacggctgct caaggggctc ttacgtttac
     1501 ggcaacttct acttctgcga ctattgctac tacaggtgga atcttgactt ctggaacatt
     1561 ttattgtcaa ggtgttgtta gtggcattga tgcttctatt acaaattagt tttgccagga
     1621 ttactggcta ggtgtgtgtg cgatatgttt gtattaacct ttagggaaaa ggccttaggg
     1681 accccccgga ctttaggcag ggagggatgg cctttggtcc cgacttgaaa aagaactaat
     1741 aaatgcccgc gggagactgt attctatcta tttttattca aaaagctttc cttgcaggtt
     1801 gtagtagaca cagttgtcct tgagccactc atactcgtca catgagttga agacatccat
     1861 gtccttgttg cataggtaga tgcacggctt gccccaggca agggttactt tcttgcggta
     1921 tttgtcagtc atggtgaatg tagactgtgc tccaagtaag gcctttcttg cagggaagta
     1981 cttccattcg atatcgtcta tcaccagata gcgacctgca tggttccagt catcgagatt
     2041 gaacatgtga ttaaagtatg tatgacagcc caaggatctc gcccattcag tttttcccat
     2101 gcgcgaggct ccgacaagga agagtgactt gggtcggtta gccgtaggat cgaggctttc
     2161 caatacccag tcacgtaata cttgcggtac tatgaaagaa gtaaacttcg atacatactc
     2221 ttcaactttg gccttgtagt gttgcttggc aaagtactcg agcttttcgt aattcaacac
     2281 gtagtcacgt ggaaagtctt gctttgctgc ttccatgaac tcatcttgtg ttgttgcggt
     2341 tgcaagtatt gaggaccatg tggccttggt tgctttgagg acgagtccct cagtgatgaa
     2401 gtctcctatt tattcatatg aaaagtaagt caatatatca gacatgtcgt ccccatccca
     2461 ctcatcatct ccaaggcaga aaatgccact ccagtccata ccatcctttt gcaagtaggc
     2521 cgcacatgct gctgggtctc gcgtgctttg gatgtttgga tgatgccatg tgtccactcc
     2581 agcttggatg tcgaagatgc gctcgttcct tgtccggaag ggtaccacga attggagcag
     2641 cacatggaag tgaggagtcc cgtcctcgtg tagctcccgt gccactaggt atccatacgg
     2701 ttggtttcca gcgtcactgg cgattttgta taccgtatca cgtactagat tttttgtcca
     2761 gtccgcaggc acctttgggt aggtgaggaa cagtgacttg gtggagagtt gaaatttacc
     2821 ggccattttg aagtgaggat gtccggatga gttggtgaat tgattgggaa gaatggggga
     2881 tta
//
LOCUS       CruV-106                2886 bp    DNA     circular     24-FEB-2020
DEFINITION  Crucivirus-106 isolate 001971_virusbaton.
ACCESSION   CruV-106
KEYWORDS    .
SOURCE      Crucivirus-106
  ORGANISM  Crucivirus-106
            Unclassified.
REFERENCE   1  (bases 1 to 2886)
  AUTHORS   de la Higuera,I., Kasun,G.W., Torrance,E.L., Pratt,A.A.,
            Maluenda,A., Colombet,J., Bisseux,M., Ravet,V., Dayaram,A.,
            Stainton,D., Kraberger,S., Zawar-Reza,P., Goldstien,S., Briskie,J.,
            White,R., Taylor,H., Gomez,C., Ainley,D.G., Harding,J.S.,
            Fontenele,R.S., Schreck,J., Ribeiro,S.G., Oswald,S.A., Arnold,J.M.,
            Varsani,A., Enault,F. and Stedman,K.M.
  TITLE     Direct Submission
  JOURNAL   Submitted (24-FEB-2020) Biology, Portland State University, 1719 SW
            10th Ave, Portland, Oregon 97201, USA
COMMENT     Bankit Comment: ALT EMAIL:ide@pdx.edu
            Bankit Comment: TOTAL # OF SEQS:451
            Bankit Comment: GAP: est. length - alignment

            ##Assembly-Data-START##
            Assembly Method       :: MetaSPAdes v. 3.12; IDBA-UD v. 1.1.1;
                                     MEGAHIT v. 1.0.3
            Sequencing Technology :: 454; Illumina
            ##Assembly-Data-END##
FEATURES             Location/Qualifiers
     source          1..2886
                     /organism="Crucivirus-106"
                     /mol_type="genomic DNA"
                     /isolate="001971_virusbaton"
                     /isolation_source="Water"
                     /country="France"
                     /collection_date=2013
     CDS             1..873
                     /codon_start=1
                     /product="Putative Replication Associated Protein"
                     /translation="METMNKKARRFRFTWNNYTEQEVNNLSSLTKKDCEYITFGFELA
                     PTTLTPHLQGYIEFAHPVPGSTCKRKLDPINKLKSKVSILEANASREANIAYAQKTET
                     KDPNRESPFIELNFRESTQGQRSDWQEMRSFLKSEPDFEKFADRYPEQAIKYHGGIDR
                     MISGINNQRCLEEFRSEYPNTSLMYTWQRKLVNRCKGTPDKRKFIWFADEKGNQGKSW
                     IAQWLVANAGATRFTNAASKDIAHAYKGEPIVIIDLTRSNEERINYSILEGLKDGMLF
                     SPKYNSHNKLFKSP*"
     CDS             883..1230
                     /codon_start=1
                     /product="putative ORF"
                     /translation="MANFYPNRSACSDDRWEIHTLTGDPIMEQVHDTPLENLKVITAC
                     NTGISDSYNAAVNLPITSDTPEDNLTPPVGSLTFPDGHGQIPCGGGVRGEPPPQGSRP
                     LSGNSRYPPTSVG*"
     CDS             complement(1383..2750)
                     /codon_start=1
                     /product="Putative CruV Capsid Protein"
                     /translation="MSKVVRVPDDAIIEMVPARRAPARRAPARRAPAKRVVTKSRTVR
                     NSRGKRVPRLREAGIISQSGSALGGALGSAFGPLGVGLGSFLGGKAGHLIEQITGFGD
                     YAIEGNSIMGLSPPEMRNSSGDSYIVRHREFITDISPSTTFTNQSFRINPGISSSFPW
                     LSQIAGSFDEYRMRGMIFEFKSMSSDSILSSGASTSLGSVVMATQYNSLQAPFADKKT
                     MENYQFANSAKPSQSFMHPIECARSLTAVDQLFVRNSSNITNLVGDLRLYDAGNFQIA
                     TVGMQNTTGVIGELWVTYEIEFLKPRYRQFSLEDHYLLDGVTNAAPFGLTTGAHTVAG
                     YGTNGIYNMNNIGGTINAAGTAYSFPHSISSGKFLIVYVVNGGATAVTLPTVTLVNCS
                     NYYGWLDQSDYKVCNTGSVGAFFWNQIITVTGSGASFTWATAVLPTSIVDGDLYVVAL
                     SDTVL*"
ORIGIN
        1 atggaaacca tgaacaaaaa agcaagaaga tttcgattta cttggaacaa ttacaccgaa
       61 caagaagtta acaacttatc atctctaaca aaaaaagatt gcgaatacat tacatttgga
      121 ttcgagcttg cgccaacaac tttaactcct catttacaag gatacataga gttcgcacat
      181 cctgtacctg gctctacctg taaaagaaaa ctggatccca ttaacaaact caaaagcaaa
      241 gttagcatcc tggaagctaa cgcctctcgc gaggccaata ttgcttatgc tcagaaaact
      301 gagacaaagg accctaatag ggaatccccc tttatagaac taaactttcg agaaagtaca
      361 caaggacaaa gatctgattg gcaggaaatg cgttctttcc ttaaatccga acctgacttt
      421 gaaaaattcg ctgaccgtta cccggaacaa gcaattaaat accacggtgg tattgatcgc
      481 atgatcagtg gaattaataa ccaacgatgt ttggaagaat tccgttcaga atatccaaat
      541 acatctctga tgtacacatg gcaacgtaaa ctggttaatc gctgcaaggg aacacctgac
      601 aaacgcaaat tcatttggtt cgcagatgaa aaaggaaacc agggtaaatc ctggattgca
      661 caatggcttg ttgctaatgc aggagctacc cgtttcacaa atgcggcgtc taaagacatc
      721 gcacatgcat acaaaggtga acctattgtt atcattgact taactcgaag taatgaagaa
      781 agaataaact attctatcct tgaaggtctc aaagatggaa tgttatttag cccaaaatac
      841 aactctcata ataaattatt caagtctcct tgactcatcg ttatggctaa cttctatcct
      901 aacagatctg cttgcagtga cgacagatgg gaaatccata cactaactgg ggaccctatc
      961 atggaacaag ttcatgatac cccattagaa aacctaaagg ttataactgc gtgtaatact
     1021 ggaatctccg attcctataa cgcagcagtt aacctaccaa taacttcaga cactcctgaa
     1081 gataacttaa ccccccccgt cgggtctctt acattcccag acggccacgg ccaaatcccc
     1141 tgtggtggag gggttcgggg cgaacctcct ccacagggga gtcgtccgtt gtctgggaat
     1201 tccagatacc ctcctacgtc ggtggggtga tgctgaatga gaagattctc tctgagagtg
     1261 ctccatatca ccatatatat agtctcgggg atcgatcggg gatctcaacg acatacaata
     1321 gaggagacct atgctccaaa aacctatata aacacaaaaa aaatattctc ttcttttttt
     1381 aactaaagta cggtatcaga taatgcaaca acatataaat ctccatctac aatggatgtt
     1441 ggcaatactg cagtagccca cgtaaacgaa gctccagatc ctgtcacggt aattatctgg
     1501 ttccagaaaa atgccccaac tgatcctgta ttacaaacct tgtaatcaga ctggtcaagc
     1561 catccataat aatttgaaca attaacaaga gttaccgttg gtaacgtaac agcggttgcc
     1621 ccgccattaa caacatacac gataagaaac ttaccggatg aaatggaatg aggaaatgaa
     1681 taagctgtac cagctgcatt aattgttcct ccaatattgt tcatattgta aataccatta
     1741 gttccataac cagcaaccgt atgtgcacct gtggtcaatc caaacggcgc tgcattagta
     1801 actccatcaa gaagataatg atcttctaaa gaaaactgcc tataccgagg tttaagaaac
     1861 tcgatttcat aggtaaccca taattctcca atcacacctg tggtattttg catacctacg
     1921 gttgcaattt gaaaattacc tgcgtcataa agacgtaaat ctccaactaa attcgtaatg
     1981 tttgatgaat tacgaacaaa taactgatca acagcggtca aagatcgagc acactcgatc
     2041 ggatgcataa aagattgcga aggcttcgca ctattagcaa attgataatt ctccatcgtt
     2101 ttcttatcag caaacggtgc ctgaagagaa ttatactgtg ttgccataac aactgatcca
     2161 agagaagttg aagcaccaga tgataatata gaatcagaag acatggattt gaactcaaat
     2221 atcattcctc tcatcctgta ttcatcaaac gaaccggcta tctgtgataa ccagggaaaa
     2281 gaagaagaaa tcccaggatt tatcctaaac gactgatttg taaaagtcgt cgatggtgaa
     2341 atatcagtaa taaattcgcg atggcgaaca atataactat cacccgatga attcctcatc
     2401 tctgggggac tcaaccccat tatgctatta ccctcaatag cataatcacc aaaacctgta
     2461 atttgttcaa tcaaatggcc agctttacca cctaaaaacg atccaagtcc tacacctaaa
     2521 ggtccgaacg ctgatccaag agcacctccg agagcagagc cgctttgaga gataatccct
     2581 gcctctctca atcgtggaac cctctttcca cgagaattcc taacagtacg actcttagta
     2641 actacacgct tcgccggtgc acgccttgct ggtgcccgtc ttgctggtgc acgccttgct
     2701 ggcaccatct caataattgc atcgtctgga actctaacaa ctttcgacat tatacaaaag
     2761 tataaaaatt ctttaaataa attaaataac tattttggct acagaagtat aaatataaga
     2821 ccaactgttg tcaatttttg gcaacattat tttctacgaa attatattca cacatcagta
     2881 tacacc
//
LOCUS       CruV-107                2899 bp    DNA     circular     24-FEB-2020
DEFINITION  Crucivirus-107 isolate 120_1833_IR2_3297.
ACCESSION   CruV-107
KEYWORDS    .
SOURCE      Crucivirus-107
  ORGANISM  Crucivirus-107
            Unclassified.
REFERENCE   1  (bases 1 to 2899)
  AUTHORS   de la Higuera,I., Kasun,G.W., Torrance,E.L., Pratt,A.A.,
            Maluenda,A., Colombet,J., Bisseux,M., Ravet,V., Dayaram,A.,
            Stainton,D., Kraberger,S., Zawar-Reza,P., Goldstien,S., Briskie,J.,
            White,R., Taylor,H., Gomez,C., Ainley,D.G., Harding,J.S.,
            Fontenele,R.S., Schreck,J., Ribeiro,S.G., Oswald,S.A., Arnold,J.M.,
            Varsani,A., Enault,F. and Stedman,K.M.
  TITLE     Direct Submission
  JOURNAL   Submitted (24-FEB-2020) Biology, Portland State University, 1719 SW
            10th Ave, Portland, Oregon 97201, USA
COMMENT     Bankit Comment: ALT EMAIL:ide@pdx.edu
            Bankit Comment: TOTAL # OF SEQS:451
            Bankit Comment: GAP: est. length - alignment

            ##Assembly-Data-START##
            Assembly Method       :: MetaSPAdes v. 3.12; IDBA-UD v. 1.1.1;
                                     MEGAHIT v. 1.0.3
            Sequencing Technology :: 454; Illumina
            ##Assembly-Data-END##
FEATURES             Location/Qualifiers
     source          1..2899
                     /organism="Crucivirus-107"
                     /mol_type="genomic DNA"
                     /isolate="120_1833_IR2_3297"
                     /isolation_source="Water"
                     /country="Norway"
                     /collection_date=2011
     misc_feature    join(2889..2899,1..18)
                     /note="stem-loop"
     misc_feature    1..9
                     /note="nonanucleotide"
     CDS             196..1632
                     /codon_start=1
                     /product="Putative CruV Capsid Protein"
                     /translation="MPPTVVSGPTPYQYQKNRSTYGVAGGRIGPAKPKGSVPDRYRRM
                     AVNMKKRKISGSGDYMIRETGSSKNEYTPFGSIGHKIGSFFGAPGIGWAGGHLIGRIL
                     GSGDYKMGPCNLSSNVLVNKTEVPQFSSSARSNIVAHREYIKDISTGPTIGAFNVESF
                     TVQPGDGTTFPWLSTIAQNYEQYRVHGMVFEFKTTSSDALNSTNTALGTVIMATEYNV
                     NSPAYTNKISMENSEFAQSAKPSQSQMHGLECAPAEGLVKNLFTRTAGLSSTSLALQD
                     KRLYDFATFQIATAGGQAANIVIGELWVTYLIEFLKPQIPTTLGGDLDTLKINGTESA
                     VTAIRITDNLSNSTTNTVTGSLGYSIAASGIAPFTESLTLTRMVIGNLYQISSAYACS
                     QAITTAPAWSITSGATAKLAYPSGTAFNANSTVSINGGGTGACQFMITFTASASTVVL
                     SLTNGLLAASGNYFGQHIVSGLDSTITN*"
     CDS             join(complement(2554..2857),complement(1840..2471))
                     /codon_start=1
                     /product="Putative Replication Associated Protein"
                     /translation="MPGAFQLKSSCIFLTYPQANPNWTKEQVLDVIKKRCGFNEPFGW
                     MVAKEAHKDGSPHYHALLQFVVPFRTKDERIFDIDGKHPNIQSSRDPAAVALYLEKEG
                     DFIKHGLAFKEKKRTWGQLVEESKDSDDFMNLVASQYPRDFVLNHEKLEYYCKQKYKI
                     KIAKYVSRFSSFANVPQSLVDWASINLNGRGDRPLSLFLVGASRLGKTEWARSLGTHT
                     YFNSMFNLDDWNPMADYLVIDDIEWKYVPAKKALLGAQAQFTMTDKYRKKVTLTWGKP
                     CIYLCNKDMDVYNSCEEAMWLRDNCVYVILHNKLY*"
ORIGIN
        1 taatattacc ctccttttcc cggtccccag tccccggtat ctgattggtc cattgatttc
       61 gtccgggctc ccgtttgttg tgccaagtca cgtgatcttg tgctaaggcc ctagccaaga
      121 ttactatata agcagcttgc cagttcattc cctcttccca aaaataatct caacttgatc
      181 taaactgtta caaatatgcc tcctacagtt gtttctggac ctactcctta tcaataccag
      241 aagaaccgta gtacttatgg ggttgcagga ggtcggattg gtccagctaa acctaaagga
      301 tctgttcctg atcgttatag gcgtatggct gtgaatatga agaagcgcaa gatttctgga
      361 tctggagatt atatgattcg agaaacggga tcatctaaga acgaatatac gccctttgga
      421 tctattgggc ataagatcgg ttccttcttt ggtgcacccg ggatcgggtg ggcgggtggt
      481 catttgattg gtcggatttt aggatctggt gattacaaga tgggtccgtg taatttgagt
      541 agcaatgtct tggtgaacaa gactgaggtt cctcagttct ctagttcggc taggtccaac
      601 attgttgctc atcgcgagta catcaaggat attagcacag gtccgactat tggtgccttt
      661 aacgtggaat cgtttacggt gcaacctggt gatggcacta ctttcccttg gttgtccacc
      721 atagcccaga attatgagca gtaccgtgtg catgggatgg tgtttgagtt taaaacgacc
      781 tcctcggatg ctttgaattc caccaataca gccttaggaa cggtcattat ggccaccgag
      841 tacaacgtca atagtcctgc ttacacgaac aagatctcga tggaaaattc tgagtttgcc
      901 caaagcgcta aaccctctca gtcccaaatg cacggtcttg aatgtgcacc tgctgagggt
      961 ttggttaaga atttgtttac tcgaactgct ggattatcgt ctacctcttt ggctcttcaa
     1021 gacaaacgtc tttatgactt tgccacgttt cagattgcca ctgcaggtgg tcaagctgct
     1081 aacatcgtta ttggagagct ttgggttaca tatttgattg agtttttgaa gccccagatc
     1141 cctactacac ttggtggtga tcttgatacg ttgaagatta acggtacaga atctgctgtt
     1201 acggcaattc gtattacgga taatctttcc aattccacca ccaacactgt tacgggttcg
     1261 cttggatata gtatcgcagc atcgggtatt gctccgttta cggagtcttt gactttgacc
     1321 agaatggtta ttggcaattt ataccagatt tcgtcggcgt atgcatgttc tcaggctatt
     1381 accacggctc ctgcatggtc cattacttca ggcgctactg ccaagttagc atatccttcg
     1441 ggtactgcgt ttaatgccaa ttcgactgtt agtattaatg gtggtggtac tggtgcgtgt
     1501 caatttatga tcactttcac tgctagtgcg tctactgtag ttttgtcttt gaccaacggt
     1561 ttattggcag cctctggaaa ctactttgga cagcatattg ttagtggtct tgactccacc
     1621 atcaccaact agggttaggg ttgtagactt gtatataacc aaaattaggg ttagggagag
     1681 ggcctaggga cccgccggaa gcatgaaggc aggggtggcc ctcggtcccg acttgactca
     1741 actggaacca cactcgaagg gagactttat atctgatcgc cggcgggacc ccccgaaggg
     1801 agggcggcga tgcgaataat aaacgaatgt taaccaaaat taatacaatt tattgtgaag
     1861 aattacgtat acacaattat ctcttaacca catagcctcc tcgcaggagt tataaacatc
     1921 catgtctttg ttgcataagt agatgcaagg tttgccccaa gtcaaagtga ctttcttgcg
     1981 atacttgtcc gtcatggtaa attgtgcttg ggccccaagt aacgcctttt ttgcaggtac
     2041 gtatttccac tctatgtcat caatgaccaa ataatcagcc ataggattcc aatcatcgag
     2101 gttaaacata ctattaaagt acgtatgagt gccaagcgag cgcgcccact ctgtctttcc
     2161 aagtcgggag gcgccgacga ggaagaggct gagtggtctg tctcctctcc cgttgagatt
     2221 gatggaggcc cagtcgacaa ggctctgagg aacattggca aaagacgaga aacggctgac
     2281 atactttgcg atcttgattt tatatttttg cttgcagtag tactcgagct tctcatgatt
     2341 caagacgaag tcgcgtgggt attgagaggc taccaaattc atgaaatcat ctgagtcctt
     2401 ggactcttcg acgagttgtc cccaggtacg tttcttctct ttgaaagcaa ggccatgttt
     2461 gatgaagtca cctatattat tcatagatta gggttcctga agtgaagcgg aaggcgccgt
     2521 cgtcgtggca ccagactcca tcccattcca taccctcctt ttcgagataa agagccactg
     2581 ctgcagggtc tcggcttgat tggatgttgg ggtgcttgcc gtcgatatcg aagattcgct
     2641 cgtccttggt cctgaagggt acgacgaact ggagcaaggc gtggtagtgg ggggacccgt
     2701 ctttgtgtgc ttccttggcc accatccacc cgaagggttc attgaagcca catctctttt
     2761 tgatgacatc gaggacttgc tccttggtcc aattagggtt ggcttggggg tatgtgagaa
     2821 atatacacga agatttaagt tggaaggcac caggcattac gaattagctg ggggactcgc
     2881 ggggaccgaa aaggaggtt
//
LOCUS       CruV-108                2905 bp    DNA     circular     24-FEB-2020
DEFINITION  Crucivirus-108 isolate 120_289_SvL2_3298.
ACCESSION   CruV-108
KEYWORDS    .
SOURCE      Crucivirus-108
  ORGANISM  Crucivirus-108
            Unclassified.
REFERENCE   1  (bases 1 to 2905)
  AUTHORS   de la Higuera,I., Kasun,G.W., Torrance,E.L., Pratt,A.A.,
            Maluenda,A., Colombet,J., Bisseux,M., Ravet,V., Dayaram,A.,
            Stainton,D., Kraberger,S., Zawar-Reza,P., Goldstien,S., Briskie,J.,
            White,R., Taylor,H., Gomez,C., Ainley,D.G., Harding,J.S.,
            Fontenele,R.S., Schreck,J., Ribeiro,S.G., Oswald,S.A., Arnold,J.M.,
            Varsani,A., Enault,F. and Stedman,K.M.
  TITLE     Direct Submission
  JOURNAL   Submitted (24-FEB-2020) Biology, Portland State University, 1719 SW
            10th Ave, Portland, Oregon 97201, USA
COMMENT     Bankit Comment: ALT EMAIL:ide@pdx.edu
            Bankit Comment: TOTAL # OF SEQS:451
            Bankit Comment: GAP: est. length - alignment

            ##Assembly-Data-START##
            Assembly Method       :: MetaSPAdes v. 3.12; IDBA-UD v. 1.1.1;
                                     MEGAHIT v. 1.0.3
            Sequencing Technology :: 454; Illumina
            ##Assembly-Data-END##
FEATURES             Location/Qualifiers
     source          1..2905
                     /organism="Crucivirus-108"
                     /mol_type="genomic DNA"
                     /isolate="120_289_SvL2_3298"
                     /isolation_source="Water"
                     /country="Norway"
                     /collection_date=2014
     CDS             1..1026
                     /codon_start=1
                     /product="Putative Replication Associated Protein"
                     /translation="MSPMSPMKTGVEVILNSTPTLSDSKKPSQKRPSCARNWFFTWNN
                     YPTRLVSDVSSNLDEIAEEWLVLLLTVFKKRCEAWVFQKELGTTPHIQGCITCKVKDR
                     WSDFGLPKDIHWELTKNNTRAQEYCCKDNTRVGDSWWVFPEPEEVIVYERDTLKTLYP
                     WQQWVFNNCKGKADHRTINWIFDPIGNNGKTFFMKHMFDTVGALCTDGGQQKDITCYI
                     ASTQEVKKGVTPKNLNKPGTTFIFDLARCCEKISYHSLEAVKNGFIFSPKYGSQSYSF
                     NCPHVWVFSNELPDCSKMSADRWKLYTIDKTKHLVEITVDENNNIVTRCPLIKTPEVD
                     DKFILKF*"
     misc_feature    945..967
                     /note="stem-loop"
     misc_feature    954..962
                     /note="nonanucleotide"
     CDS             complement(1480..2850)
                     /codon_start=1
                     /product="Putative CruV Capsid Protein"
                     /translation="MAAVRRRVPRKRRVAVRKVRVIKGKGDYVIADPAHVTKKDVKDA
                     YKQGLAEGAESSGPQSVGATIGQYLGHGAQKVVKMLTGFGDYAVNENSLMHGGATLGA
                     NPPSVENTQTGFIVRHREYIQDVFSTTAFINVPLEINPGLSSTFPWLSQVADSFEQYS
                     FRGLVFEYKSMSSDAVLSGGSSSALGTVIMSTQYNSLDLPFTDKRTMENYEYANSDKP
                     SNSFMHPIECKKNQNSISELYIRTGPINGAVGDKRLYDLGVFNIATQGMQNASPNQVI
                     GELWCTFEIEFYKAKLSTGDPALASDTFHRSGVANYFNLLAPFDSSCLPSLQNTIGCT
                     IPVRGTIRFPLNVVDGIYQITYFMTGSTATTVNSLAYTAGGVTVNVDIVSTDVKFFFG
                     ASDSFALAPNAGSTTNNIQITQICKILQTNPGLPAQVQLLIDSGTLPQGSQTVDIIVN
                     EMSPYI*"
ORIGIN
        1 atgtctccaa tgtctccaat gaaaactggg gtagaggtaa tactaaactc tacccccacg
       61 cttagtgatt caaagaaacc ctctcaaaag agaccaagtt gtgcaagaaa ctggtttttc
      121 acttggaata actatccgac ccggcttgtc tccgatgtct ccagtaattt ggatgaaatc
      181 gccgaagaat ggttagttct tctgctcacg gttttcaaaa aaagatgcga ggcttgggtc
      241 ttccaaaaag aacttggtac gacgcctcac attcaaggct gcatcacatg taaagtgaag
      301 gaccggtgga gtgactttgg tttaccaaag gacattcact gggaactcac taaaaataac
      361 actcgggccc aagaatactg ctgtaaagac aacacaaggg ttggtgactc gtggtgggtc
      421 tttcctgaac ctgaggaagt tatcgtatac gaacgcgata ctttaaaaac tttgtatcct
      481 tggcagcaat gggtgttcaa caattgcaaa ggcaaagctg accacagaac cattaattgg
      541 atatttgacc ctattggaaa taacgggaag accttcttca tgaaacatat gtttgacacc
      601 gttggtgcgt tgtgtactga tggtggacag caaaaagaca ttacctgcta tattgcaagt
      661 actcaagaag tgaaaaaagg agtcacccct aaaaacttga acaaacctgg aactaccttt
      721 atcttcgacc tggcacggtg ctgcgaaaaa atatcatacc actccctcga agcagttaaa
      781 aacggattca tcttctctcc taaatacggt tcgcagtcgt actcgtttaa ctgtcctcat
      841 gtctgggtat tctcaaatga gttaccagat tgttcaaaaa tgtcagcgga taggtggaag
      901 ctttatacta ttgataaaac caagcacctc gttgagatta cagttgacga aaacaataat
      961 atcgtcactc ggtgcccact tattaaaaca cctgaagttg atgacaagtt tatcctcaaa
     1021 ttttaatgag gcaaatgctc tatggatgct cgggacgccc ctacggccta tttttgtcgc
     1081 tttgcagcgc caaaaattag tcggcctacg cgttccgctg gggctaaaac tcgcatcgcc
     1141 tacgctttgg tcgggggggg ccctggactc ccttccccac tccctccgct ccgctgaggg
     1201 tggctcacgc cctaacgggc tccgctgggg acaaaacata cactatattg tgttacatat
     1261 attggtgact cgtgcaccaa aaataaactt gtaatcataa tccgaaactt aaaaagttcc
     1321 tgattattag tggtttcttt tttgattgcc cgcgcaccaa taggcgatgc aatcgtatta
     1381 aaacacctaa aagttactgc tccatccgct accatgtatt gggcgatgat gtagactgcc
     1441 gctccttgcg tagtttagcg tcatttcgga cgttactact taaatgtatg gactcatttc
     1501 gttaacgata atatccacag tctgagagcc ttgtggtaat gtaccagagt caatcaacag
     1561 ttgaacttga gctggtaatc ctggattagt ttgaagaatt ttacaaattt gagtaatttg
     1621 aatattgttc gtggtactac cagcatttgg agcgagagcg aatgaatctg atgctccaaa
     1681 gaaaaattta acatcagttg aaactatgtc tacgttgacg gttactccac cagcagtata
     1741 tgccagggag tttacggtag tcgctgtgct tcctgtcata aagtaagtta tttgatatat
     1801 accgtcaaca acgttcaatg gaaatcggat tgtacctcta acaggtatag tacaaccgat
     1861 ggtgttttgc agtgatggga gacatgaact gtcgaaaggt gctaacaaat taaaataatt
     1921 tgctactccc gaacgatgaa atgtatcgga tgctaatgct gggtctccag tagagagttt
     1981 cgctttgtaa aactcaatct caaaagtaca ccacaactca ccaatgactt gattaggtga
     2041 agcattctgc attccctgag ttgcaatgtt aaaaactcct aaatcgtata aacgcttatc
     2101 accgacagca ccattaattg gtccagtacg aatatagagc tccgaaatag aattttggtt
     2161 cttcttacat tcgataggat gcatgaacga gtttgatggt ttatcagaat ttgcgtactc
     2221 ataattctcc atagttctct tatccgtaaa cggcaaatcg agggaattat attgcgtact
     2281 cataattaca gtaccaaggg cagatgaact gccaccactc aaaactgcat ctgaactcat
     2341 tgacttgtat tcaaatacaa gacctctaaa agagtattgc tcgaatgagt ctgcgacttg
     2401 agataaccac gggaacgtac tcgataaacc agggtttatt tcgagaggta catttataaa
     2461 agctgttgtt gagaatacat cctgtatgta ctcacgatgc cttacaatga aaccggtttg
     2521 tgtgttctcg acgctcggag gatttgcgcc taaagttgcc cccccatgca tcaaactgtt
     2581 ttcgttgact gcatagtccc cgaaaccagt taacatcttt acgacttttt gggcgccgtg
     2641 accgaggtat tgacctatag ttgcacccac ggactgcgga ccagaggatt ccgcaccctc
     2701 agcaagacct tgcttataag cgtctttgac gtctttcttc gtcacatgtg ctgggtcggc
     2761 aattacataa tcacctttcc ccttaataac tcgtaccttg cgaactgcta ctcgtctttt
     2821 tctcggaact ctccttcgaa ctgctgccat tttatattat agtgcgagaa tttattttct
     2881 aaacggatag tataatatga cccaa
//
LOCUS       CruV-109                2911 bp    DNA     circular     24-FEB-2020
DEFINITION  Crucivirus-109 isolate 120_1803_IR2_3297.
ACCESSION   CruV-109
KEYWORDS    .
SOURCE      Crucivirus-109
  ORGANISM  Crucivirus-109
            Unclassified.
REFERENCE   1  (bases 1 to 2911)
  AUTHORS   de la Higuera,I., Kasun,G.W., Torrance,E.L., Pratt,A.A.,
            Maluenda,A., Colombet,J., Bisseux,M., Ravet,V., Dayaram,A.,
            Stainton,D., Kraberger,S., Zawar-Reza,P., Goldstien,S., Briskie,J.,
            White,R., Taylor,H., Gomez,C., Ainley,D.G., Harding,J.S.,
            Fontenele,R.S., Schreck,J., Ribeiro,S.G., Oswald,S.A., Arnold,J.M.,
            Varsani,A., Enault,F. and Stedman,K.M.
  TITLE     Direct Submission
  JOURNAL   Submitted (24-FEB-2020) Biology, Portland State University, 1719 SW
            10th Ave, Portland, Oregon 97201, USA
COMMENT     Bankit Comment: ALT EMAIL:ide@pdx.edu
            Bankit Comment: TOTAL # OF SEQS:451
            Bankit Comment: GAP: est. length - alignment

            ##Assembly-Data-START##
            Assembly Method       :: MetaSPAdes v. 3.12; IDBA-UD v. 1.1.1;
                                     MEGAHIT v. 1.0.3
            Sequencing Technology :: 454; Illumina
            ##Assembly-Data-END##
FEATURES             Location/Qualifiers
     source          1..2911
                     /organism="Crucivirus-109"
                     /mol_type="genomic DNA"
                     /isolate="120_1803_IR2_3297"
                     /isolation_source="Water"
                     /country="Norway"
                     /collection_date=2011
     CDS             1..1353
                     /codon_start=1
                     /product="Putative CruV Capsid Protein"
                     /translation="MSSYNTYSRQYSRKKRFVPYEEYMRRKQMPKAQRAPYRKKAYRP
                     SYKKRAASAQNNEIGRAIGSTIGAAIAGAPGSVIGSALGGGAQSLISKITGSGDYKVN
                     YNVLVNEQVPQFAMGNNKFRETRISHKEFIQDIISSATPGAFNIDTFVVNPGVSATFP
                     WLSSLAVNYEEYALDGLIFEFKSTSSNALNSTNTALGTVVLATQYNALSAPFVNKQQM
                     ENYEFAVSCNPSCNVIHPLECAQFENPVNMLYVRELDNASIANSDKRLYDMGNFSIAS
                     VGLQGASVNIGELWVSYSLRLLKPKLGQAGDIADHFILDAASVTTGSWMGNVALAVLS
                     QSSDFGVSLTQTTIVIPSFFSGNICIELALNVASASQTAPTLTGTLGATPLNLLFNNT
                     INYWITPAGETVTKIISTSYWTIANGGTITFSGGSTGAPGACTVADLVVSAISSSLVD
                     *"
     CDS             complement(1610..2659)
                     /codon_start=1
                     /product="Putative Replication Associated Protein"
                     /translation="MSLTTQNSEVHSDGSSNSIDATENTEKIITDEFPENQKTKIRGT
                     FFTLNNYTDDDIKRMISLVIGKEDSRYLVYQLEEGEVDHTPHVQGFFYFHNQIQLGTL
                     NKALPTAHFMKPRCIKACIKYCKKDKTRIAGPFEYGLMPEQGRRTDLEEVARKFLTLS
                     MKEFVEQCPTEYVRYHRGLQALKMASTTHRDRNTPPIVKWYWGNAGTLKTRTAFESHT
                     TSYIKDGTTWWNGYEQQVAIIIDDFDGKWPYRDLLRLLDRYPYQGQVKGDYVKINSPY
                     IYITCEHPPGMFWQGNELAQVNRRISEVRCFNPVMETEALPGSKEETHVQAHAAEAED
                     PDDILDVLNHMAQTK*"
     misc_feature    2593..2621
                     /note="stem-loop"
     misc_feature    2605..2613
                     /note="nonanucleotide"
ORIGIN
        1 atgtcatcgt acaacacgta ttcgcgacaa tattccagaa agaaacgatt cgttccatat
       61 gaagagtaca tgaggcgcaa acaaatgcca aaggcacaaa gggctcctta tcgaaagaaa
      121 gcatatcgtc cgtcgtacaa gaaaagagca gcatctgctc aaaataatga gattggaagg
      181 gcaatcggaa gcaccattgg agccgcaatt gcgggagctc caggaagtgt gatcggttca
      241 gctcttggcg ggggagctca atctctgatt agtaaaatca caggtagtgg agactacaaa
      301 gtaaattata atgttctggt caacgaacag gttccacagt ttgcaatggg taataacaag
      361 tttagagaga ctcgtatttc acacaaagaa ttcattcaag atattatatc gtcagcgact
      421 cctggcgcct ttaacattga cacatttgtt gtcaatcctg gtgtttcagc aacgtttcct
      481 tggctttcat ctcttgcagt gaattatgaa gaatatgctc ttgatggtct catttttgag
      541 tttaaatcaa cttcatccaa tgctctcaac tcaacaaaca ctgctcttgg tactgttgta
      601 ttggcgactc agtataatgc tttgagcgct ccttttgtta acaaacaaca gatggaaaac
      661 tatgaatttg cagtttcttg caatccttct tgtaacgtga ttcatccact tgagtgtgca
      721 caattcgaaa atccagtaaa catgttatat gttcgagaac ttgacaatgc ttcaatcgca
      781 aactctgaca aacgattgta tgatatgggt aactttagca ttgcttccgt gggacttcaa
      841 ggagcatcgg tcaacatcgg tgaattgtgg gtatcttatt ctcttagact tctcaaacca
      901 aaactcggac aagcgggtga tattgccgac cattttatat tagatgctgc atctgtgaca
      961 actggctcat ggatgggaaa cgtagcctta gccgttcttt ctcagtcatc tgattttggt
     1021 gtgtcattaa cacaaacaac tattgttatt ccttcttttt tctcgggtaa tatttgtatc
     1081 gaacttgcat tgaatgttgc atctgcgtca caaactgcac caacattaac tggtactttg
     1141 ggagccactc ctctcaatct tctcttcaat aatactatta attattggat cacaccagca
     1201 ggagagaccg taactaaaat tatatctact tcatattgga ccattgccaa tggagggaca
     1261 atcacctttt ctggtggttc aacaggagcc cctggagctt gtactgttgc cgatttagta
     1321 gtttcagcta tttcttcatc tcttgtcgac taattatatg ttatcaaaca tacaatttaa
     1381 ttatcttctt cactattttc ttcaggccaa ttgcaccaat aacatgggaa atcatcatta
     1441 tcatatacta acattccaca atttggacaa gacacgtcat caataatttc ttcctctatg
     1501 tgtacttctt cagccaaatt tatttcttct ggaataattt gcattatcaa acatatatct
     1561 ttttgcacac acatttataa tgttataaat atgtctttag gtcatttatt tattttgttt
     1621 gcgccatatg attcaaaaca tccaaaatat catctggatc ctcggcttca gccgcatgtg
     1681 cttgcacatg cgtttcttct ttgcttcctg gtaaagcttc ggtttccata actgggttga
     1741 aacatctaac ttcactaatt cttctgttaa cttgggcaag ttcgtttcct tgccaaaaca
     1801 ttcctggtgg gtgttcacaa gtaatatata tgtaggggct atttatttta acatagtctc
     1861 cttttacttg gccttgatag ggatatctgt caagcaatct taatagatct ctgtatggcc
     1921 attttccatc aaagtcatca ataataatag caacttgttg ttcgtatcca ttccaccatg
     1981 tggttccatc tttaatgtac gatgttgtgt gtgattcaaa agctgtccga gttttgagag
     2041 ttccagcgtt tccccaatac catttaacaa ttggaggagt atttctatct ctgtgtgtcg
     2101 ttgaagccat cttcaaagct tgaagtcctc ggtgatatct tacatattca gtgggacatt
     2161 gttcaacaaa ttctttcata gagagtgtta gaaattttct tgcaacttct tccagatctg
     2221 ttcttcttcc ttgttcaggc atcagtccgt attcaaaagg tccagcaatg cgggttttat
     2281 cctttttgca atatttaata catgctttaa tacatcttgg tttcatgaaa tgggctgtcg
     2341 gaagagcttt gttcaaagtg cctagttgaa tctgattgtg aaaatagaaa aagccttgaa
     2401 catgaggggt gtgatcaact tctccttctt ccagctggta taccaagtat ctgctatctt
     2461 ctttgccaat gacaagagag atcattcgtt tgatatcatc atctgtgtaa ttattgagag
     2521 tgaaaaacgt tcctcgtatc ttagttttct gattttctgg gaattcatca gtaattattt
     2581 tttcagtgtt ttcggtagca tctatactat tactgctacc gtctgagtga acctctgagt
     2641 tttgtgttgt gagtgacatt tctgggctct gtacttgtct ttaggtcatt tattttgtgg
     2701 tgtgaaaaat ggtctagagc atctgaaatg tgttatattt ttttgtttgc gtgataataa
     2761 ctggtctaaa gctatcgtat ctgtgtgaat ttttgtgtgt tgagtgataa taactggtct
     2821 atagcaataa atatgtgtca aagtttttgt gttcgcgaag ataaataact ggtctaaaga
     2881 cacatatttg taataaaata acacaccaac c
//
LOCUS       CruV-111                2929 bp    DNA     circular     24-FEB-2020
DEFINITION  Crucivirus-111 isolate SR1_3042.
ACCESSION   CruV-111
KEYWORDS    .
SOURCE      Crucivirus-111
  ORGANISM  Crucivirus-111
            Unclassified.
REFERENCE   1  (bases 1 to 2929)
  AUTHORS   de la Higuera,I., Kasun,G.W., Torrance,E.L., Pratt,A.A.,
            Maluenda,A., Colombet,J., Bisseux,M., Ravet,V., Dayaram,A.,
            Stainton,D., Kraberger,S., Zawar-Reza,P., Goldstien,S., Briskie,J.,
            White,R., Taylor,H., Gomez,C., Ainley,D.G., Harding,J.S.,
            Fontenele,R.S., Schreck,J., Ribeiro,S.G., Oswald,S.A., Arnold,J.M.,
            Varsani,A., Enault,F. and Stedman,K.M.
  TITLE     Direct Submission
  JOURNAL   Submitted (24-FEB-2020) Biology, Portland State University, 1719 SW
            10th Ave, Portland, Oregon 97201, USA
COMMENT     Bankit Comment: ALT EMAIL:ide@pdx.edu
            Bankit Comment: TOTAL # OF SEQS:451
            Bankit Comment: GAP: est. length - alignment

            ##Assembly-Data-START##
            Assembly Method       :: MetaSPAdes v. 3.12; IDBA-UD v. 1.1.1;
                                     MEGAHIT v. 1.0.3
            Sequencing Technology :: 454; Illumina
            ##Assembly-Data-END##
FEATURES             Location/Qualifiers
     source          1..2929
                     /organism="Crucivirus-111"
                     /mol_type="genomic DNA"
                     /isolate="SR1_3042"
                     /isolation_source="Water"
                     /country="New Zealand"
                     /collection_date=2012
     misc_feature    join(2921..2929,1..16)
                     /note="stem-loop"
     misc_feature    1..9
                     /note="nonanucleotide"
     CDS             42..1046
                     /codon_start=1
                     /product="Putative Replication Associated Protein"
                     /translation="MSKKCAGTIGTKQNPCCVYDFTLYDTLEVNVIRSNIEDICKKYC
                     FQLEKGEKSGKLHYQGRISLTQKKRLNELIKILNKKGWANFHLSITSKENRDNNFYVC
                     KEETRVSGPYTNENNIYIPKDVSKMQVLRPWQKSLRDELSTYNERSVDVIYEKNGNIG
                     KSSICRYMMIYDDAELLPFCNDYKDVMRMAYDVGPKKIYLIDMPRAISKEKLFQFYSA
                     IETLKSGYCYDDRYKFTRRLFDRPRICVFTNTPPDTTMLSKDMWNIWTVSNNNLEKYD
                     DLIKSLDPHYINVDDIVFISDYENDNDICDFDLIDIKNIDTPPVVRKTISRKPTKKEP
                     *"
     CDS             complement(1531..2922)
                     /codon_start=1
                     /product="Putative CruV Capsid Protein"
                     /translation="MPRYTDAEKAAYYKRKSQTVYHRRPVRKTRSSGSKTRYGKRYLK
                     EQPDYRYPGVGRRIGGYLGSMAGNVLAPGVGGAVGNVVGSLAGQGAQALIKKISGFGD
                     YSVSKNSLMYNVDAVPEFSNNNERCTVVTHREFISDIQSSTGFVSRTFRINPALAETF
                     PWLAALAENYEQYVVQGMVFEYKTTSATAIGSTNTSLGTVVMATQYNSLAPPFSNKQQ
                     MENYEFSQSTVPSQSILHPIECDPMQTQCGGIFNMFNPNDTAGDTRLYDIGRFTIATV
                     GMQAGAATVGELWVSYKICLLKPRLQAISNVADGYILEASSVTNALPLGNFLNATTFA
                     ENSNFTNLVSSSEFRFDPNFVGVVQVLLCYVCSGTAITPPTISPLANCVQMNPNYINS
                     NLTLTGQNDLTAGVSPHNVYSLGFFNVTGGFNAGGSRPGVSIFGLTTTGVTFISARIL
                     FISVPSQFTPGFN*"
ORIGIN
        1 aattactata taaaataata ttttctctag aatatataga aatgtctaaa aagtgtgctg
       61 gaacaattgg aacaaaacaa aacccatgtt gtgtatacga ttttacgtta tacgacactc
      121 tcgaagtaaa tgtcatcaga tctaatatcg aagacatatg caaaaagtac tgttttcaac
      181 tagaaaaagg agagaaatcg ggtaagctac actatcaagg aagaatatct cttactcaaa
      241 aaaagagatt aaatgaacta attaaaatac taaacaaaaa aggatgggca aactttcatc
      301 tttcgataac atcaaaagaa aatagagaca ataacttcta tgtttgcaaa gaagaaacta
      361 gagtaagtgg accttacact aatgaaaata atatatatat accaaaagac gtttctaaaa
      421 tgcaagttct ccgaccatgg caaaaatcat taagagatga acttagcaca tataatgaaa
      481 gatcagtcga cgtaatatat gaaaaaaatg gaaatatcgg aaaatcatca atatgtagat
      541 atatgatgat ttatgatgat gccgaattac tacccttctg taatgactac aaagatgtca
      601 tgagaatggc atatgatgta ggacctaaaa aaatatactt aatcgatatg ccgagagcaa
      661 tctccaaaga aaaacttttt caattctatt cagcaattga aacattaaaa agcggatact
      721 gttatgatga tcgatacaaa ttcactagac gtttatttga tcgtccacgt atctgtgttt
      781 ttactaatac accaccagat acaaccatgt taagcaaaga tatgtggaac atatggacag
      841 tatctaataa taacttagag aaatacgatg atttaattaa gtcacttgac ccacactaca
      901 ttaacgtaga tgatattgtc ttcataagtg actatgaaaa tgataatgac atttgcgatt
      961 ttgacctaat cgacataaaa aatatagata caccaccggt tgttagaaaa accattagtc
     1021 ggaaacccac taaaaaggaa ccatgaaata actcataaaa tgaggatttt tcggcgctca
     1081 catccgttcc ctgtaatact gcatcgaaaa atctattatt taataatata tctagtaatt
     1141 cttactatat atattattga agtttctgga acattggaac attattgaag cttatccgtt
     1201 tttctcggaa aattactgga acattctaga tcattgttcc aaatctaaat ttactatata
     1261 aattatcgga taaaggggca acgtgcccct cttgctatcg caattcaccc accagggcaa
     1321 cgtgccctct cgccgtcgcg attcacctcc gaaggggcaa gccccctctt gccttcgcaa
     1381 ttcacccaag gtgcgtacgc cgccccttta ccccggcgcc cttgctgggg cagggcgacg
     1441 tgaaaaaagc agggcttttt tcgcttacgc catttactct atcttctgat acacataata
     1501 attcataaac atgcattatt atatgaatag ttaattaaaa cctggtgtaa actgacttgg
     1561 aactgaaata aataagatcc gtgctgaaat aaaagttaca ccagttgtag taagaccaaa
     1621 tattgatact cccggtcgag aacctccggc attaaaacct ccggtcacat taaagaaacc
     1681 caatgaataa acattatgag gtgatacacc agctgttaaa tcattctgac ccgtcagagt
     1741 aagattacta tttatataat tcggattcat ttgaacacaa ttagcaagag gagatattgt
     1801 cggaggcgta atagccgttc ccgaacaaac ataacaaaga agaacttgaa ccactcctac
     1861 aaaattagga tcaaacctaa actcagatga actaacaaga ttagtaaaat tactgttctc
     1921 tgcaaaagtt gttgcattaa gaaaattacc aagaggtaaa gcattagtaa cactagatgc
     1981 ttcgagtatg taaccatcag caacattact tatggcttgc aatctcggct taagcaggca
     2041 tatcttgtaa gatacccata gctctcctac ggtggctgcg cctgcctgca tccctacggt
     2101 cgctatcgta aatctcccga tgtcatataa acgtgtatct ccggctgtat cgtttggatt
     2161 aaacatatta aagatacctc cacactgagt ttgcatagga tcacactcaa taggatgtaa
     2221 tatactctgg gatggaaccg tcgattgtga gaattcgtag ttctccattt gttgcttatt
     2281 agaaaatgga ggagctaaac tattatattg agtcgccatc acaactgtac ctaatgaagt
     2341 attagttgaa ccaattgcag ttgcagaagt ggttttgtac tcgaaaacca ttccttgaac
     2401 cacatattgt tcatagttct ccgctaatgc tgctaaccat ggaaatgtct ctgctaacgc
     2461 tggatttatt ctaaatgttc tcgaaacaaa accagttgat gattgtatat ccgatataaa
     2521 ctctcgatga gtaactaccg tacatcgttc gttattatta gagaactcag gaacagcatc
     2581 aacattatac atcaacgaat tcttagaaac tgaataatct ccgaaccctg aaatcttctt
     2641 tattaatgct tgtgcacctt gacctgcaag tgaacccaca acattaccta ctgctccacc
     2701 tacacctggt gctaatacat taccagccat cgaacccaaa tatcctccga tccgtcttcc
     2761 gactccagga tatctataat caggttgctc ttttaaatat cgcttcccat atctagtctt
     2821 tgatccacta gatctagtct ttcgaacagg ccttcgatga taaacagtct gtgacttacg
     2881 cttataataa gctgctttct ctgcatcagt atatcttggc attttataa
//
LOCUS       CruV-112                2942 bp    DNA     circular     24-FEB-2020
DEFINITION  Crucivirus-112 isolate H_425.
ACCESSION   CruV-112
KEYWORDS    .
SOURCE      Crucivirus-112
  ORGANISM  Crucivirus-112
            Unclassified.
REFERENCE   1  (bases 1 to 2942)
  AUTHORS   de la Higuera,I., Kasun,G.W., Torrance,E.L., Pratt,A.A.,
            Maluenda,A., Colombet,J., Bisseux,M., Ravet,V., Dayaram,A.,
            Stainton,D., Kraberger,S., Zawar-Reza,P., Goldstien,S., Briskie,J.,
            White,R., Taylor,H., Gomez,C., Ainley,D.G., Harding,J.S.,
            Fontenele,R.S., Schreck,J., Ribeiro,S.G., Oswald,S.A., Arnold,J.M.,
            Varsani,A., Enault,F. and Stedman,K.M.
  TITLE     Direct Submission
  JOURNAL   Submitted (24-FEB-2020) Biology, Portland State University, 1719 SW
            10th Ave, Portland, Oregon 97201, USA
COMMENT     Bankit Comment: ALT EMAIL:ide@pdx.edu
            Bankit Comment: TOTAL # OF SEQS:451
            Bankit Comment: GAP: est. length - alignment

            ##Assembly-Data-START##
            Assembly Method       :: MetaSPAdes v. 3.12; IDBA-UD v. 1.1.1;
                                     MEGAHIT v. 1.0.3
            Sequencing Technology :: 454; Illumina
            ##Assembly-Data-END##
FEATURES             Location/Qualifiers
     source          1..2942
                     /organism="Crucivirus-112"
                     /mol_type="genomic DNA"
                     /isolate="H_425"
                     /isolation_source="Water"
                     /country="New Zealand"
                     /collection_date=2012
     CDS             1..1305
                     /codon_start=1
                     /product="Putative CruV Capsid Protein"
                     /translation="MSRRAPPRSAPRRAQTTRAPRPQLKGRGAYNVDSSYKKTKQARK
                     IAKRAPDYEPSLGRNLLTEGGSMLGGLFGMPGLGKSAGSALSNIFGLGSYEVKENVFM
                     EGRLPQVMNVPTGGGTVIRFQEYLGDVITSATVGAFQNTSWIVNSANRDSFPFLAQIA
                     ANYEQYQFEGLVYEFRSTSADALNSTNTALGSVMMATQYDFQDTPFTSKAEMLNYEYS
                     NSVKPSENCMHMIECAPSQSVLPILYTLDGSVPAGADSRLYHLGRFQVATIGFQAPSV
                     RIGEIHVTYQVRLLKPKIYSALGLDNDLWRLQSNSYTNALPLGPAVSSTPEFDNFGVT
                     HDSTIITIPPSSLVKNYRLEMYWKGNTTIVTVPTINFVNATALQTRVTTNGTSTQEVF
                     INTAFVTQGLNLPVNIIFGVLGTLPAAGTEVIARLIEVASPA*"
     CDS             complement(1557..2597)
                     /codon_start=1
                     /product="Putative Replication Associated Protein"
                     /translation="MTQKIETIRGMRIGNTKQSASSKKGISKGKSKKNRDILKIVETE
                     ETKDEVCEEFKTSTEIKKPKQPIQLKHWFFTFNNYSKGDIGILEKKFKAICVKYVFQE
                     ETGESGTPHLQGSIHLIYAMRPSEFKLNNKIHWEKTNNETKADEYCEKIDSRTGDVFK
                     WGFPPPKVPIEYITELYEWQQEIYNLCLPKPNPRDIVWVYDKSGNNGKTEFCKYMYDK
                     IDAVLSDDATKKDIACLIADMVEDDDRDFGKQTTFLFDFPRSCKSVDYGALESVKNGL
                     IMSPKFHSKTLIFNKPHVIVMSNSLPEFSKMSIDRWIVYELNDKKLHKMDIKKYLKNI
                     CEDDNMFIEDEE*"
     misc_feature    1705..1723
                     /note="stem-loop"
     misc_feature    1710..1718
                     /note="nonanucleotide"
ORIGIN
        1 atgtcccgaa gagctccccc aagaagtgcc ccaagacgag cccaaaccac cagagcgcca
       61 agaccgcaat taaaaggtcg tggtgcttat aatgttgatt cttcttacaa gaagaccaaa
      121 caagcgcgca agattgcaaa gcgtgcccct gattacgaac catcactcgg aaggaactta
      181 ctgactgagg gagggagtat gttagggggt ctgtttggga tgccaggact cggcaaatca
      241 gccgggtccg ccctttctaa tatattcggt ttaggttcat acgaagttaa agagaatgta
      301 tttatggagg gtagattacc acaagttatg aacgttccga caggtggagg aacggtaata
      361 agatttcaag agtatcttgg ggatgtgata acatccgcca cggttggagc ttttcaaaat
      421 acatcatgga ttgtgaacag tgcaaaccgg gactccttcc catttcttgc ccagattgcc
      481 gcgaattatg agcagtatca atttgaggga cttgtctatg agtttcggag tacatccgcc
      541 gacgcgctga attcaacaaa tacagccctc ggctcggtca tgatggccac acaatatgac
      601 tttcaagaca ccccatttac atccaaagca gaaatgctta attatgaata ttcaaacagt
      661 gttaagccct ctgaaaactg tatgcatatg atagagtgcg ccccgtcgca atcggtttta
      721 cctattcttt ataccttaga cgggtctgtg ccagctggtg cagattcccg attataccat
      781 ttaggtcggt ttcaagtcgc aacaattggg tttcaggccc caagtgtgag aattggagag
      841 atccatgtta cttaccaagt tagattactt aaaccaaaaa tatactcagc cttaggtctt
      901 gataatgacc tctggcgcct tcaatcaaac tcttatacca atgctttacc actcggccca
      961 gccgtttcct caactcctga atttgataat tttggtgtga ctcatgactc aacaataatt
     1021 acaataccac catcttcatt ggtaaaaaac tatcgtttag aaatgtattg gaaaggtaac
     1081 actacaattg ttacagttcc aactatcaat ttcgtaaacg ctacagcctt acaaacaaga
     1141 gtcactacaa acggaacatc tactcaagaa gtttttatta atacagcctt tgtaacccag
     1201 ggattaaatt taccagtaaa tataatcttt ggggtcttag gaaccttacc agcagcaggg
     1261 accgaagtaa tcgccagact tattgaggtc gctagtccag cataagacaa gtccctcaaa
     1321 ttgattgaac attattaact aataactact attaaacttt aacctatata atatatagat
     1381 taaattatgc gtagagcatc gtaggccgtt aggccgtcaa tggctgtagc ctccgaacgc
     1441 cagggggtgg ggtgggggcc ctacctagat acctttaggt atctaccgga tttatccggc
     1501 ccggttagtg gagcgcggaa gctggagggt tcttaaaaac ccgacataca gaataattat
     1561 tcttcatctt caataaacat attatcatct tcacaaatat ttttcaaata ttttttaata
     1621 tccattttat gcaacttttt atcattaagt tcatatacta tccatctatc tattgacatt
     1681 tttgagaatt caggcaatga attactcatg actattacat gaggtttatt aaaaataagt
     1741 gtcttagaat gaaattttgg tgacattata agaccatttt taacactttc aagagcccca
     1801 tagtcaacac ttttacagga cctcgggaaa tcaaacaaaa atgttgtttg ttttccgaaa
     1861 tctctatcat catcttccac catatcagca attaaacatg caatatcttt tttagttgca
     1921 tcatctgata aaacggcatc aattttatca tacatatatt tacaaaattc agttttgcca
     1981 ttatttccac ttttatcata aacccaaaca atatccctag gatttggttt tggtaaacat
     2041 aaattataaa tttcttgttg ccactcataa agctcggtga tatattcaat cgggacttta
     2101 ggaggtggaa acccccattt aaacacatcg cctgtccgac tgtctatttt ttcacaatac
     2161 tcatcggctt tagtttcatt attagttttt tcccaatgaa ttttattatt caatttaaat
     2221 tctgatgggc gcatagcata tataaggtgt atactacctt gtagatgtgg tgtgccactt
     2281 tcaccagttt cttcttggaa aacatattta acgcatatag ctttaaattt tttttctaag
     2341 attccaatat ctcctttact gtaattatta aacgtaaaga accaatgctt taattgtatt
     2401 ggttgtttag gttttttaat ttcagtagag gttttgaact cttcacaaac ctcatcctta
     2461 gtctcttcgg tctctactat ctttaaaata tccctatttt tcttactttt acctttactt
     2521 ataccctttt tactagatgc ggattgttta gtgttaccaa tccgcatccc tctaatggtc
     2581 tctatttttt gggtcatttt atactataag attatataat aattctttat gtatatttgg
     2641 tgcgtttgcc cgaagggttg ggggatggtc ccagattgct taagtaagca agctggttgg
     2701 ggccccccta gtcgcgtctt gggacataac atactgagga aactattaat tttatattag
     2761 tatgggggag taatttaatt ttgataattt tatgaattct gaccaggaat gaataacgga
     2821 gttatcttta agtgtaatta acataatttt tataattatg ttaaatatac ttaaaaataa
     2881 cgactttcag aatttaatta actcccccaa tatttattat ctataaatat agtataataa
     2941 at
//
LOCUS       CruV-113                2952 bp    DNA     circular     24-FEB-2020
DEFINITION  Crucivirus-113 isolate GP1_93048.
ACCESSION   CruV-113
KEYWORDS    .
SOURCE      Crucivirus-113
  ORGANISM  Crucivirus-113
            Unclassified.
REFERENCE   1  (bases 1 to 2952)
  AUTHORS   de la Higuera,I., Kasun,G.W., Torrance,E.L., Pratt,A.A.,
            Maluenda,A., Colombet,J., Bisseux,M., Ravet,V., Dayaram,A.,
            Stainton,D., Kraberger,S., Zawar-Reza,P., Goldstien,S., Briskie,J.,
            White,R., Taylor,H., Gomez,C., Ainley,D.G., Harding,J.S.,
            Fontenele,R.S., Schreck,J., Ribeiro,S.G., Oswald,S.A., Arnold,J.M.,
            Varsani,A., Enault,F. and Stedman,K.M.
  TITLE     Direct Submission
  JOURNAL   Submitted (24-FEB-2020) Biology, Portland State University, 1719 SW
            10th Ave, Portland, Oregon 97201, USA
COMMENT     Bankit Comment: ALT EMAIL:ide@pdx.edu
            Bankit Comment: TOTAL # OF SEQS:451
            Bankit Comment: GAP: est. length - alignment

            ##Assembly-Data-START##
            Assembly Method       :: MetaSPAdes v. 3.12; IDBA-UD v. 1.1.1;
                                     MEGAHIT v. 1.0.3
            Sequencing Technology :: 454; Illumina
            ##Assembly-Data-END##
FEATURES             Location/Qualifiers
     source          1..2952
                     /organism="Crucivirus-113"
                     /mol_type="genomic DNA"
                     /isolate="GP1_93048"
                     /isolation_source="Water"
                     /country="New Zealand"
                     /collection_date=2014
     misc_feature    join(2940..2952,1..19)
                     /note="stem-loop"
     misc_feature    1..9
                     /note="nonanucleotide"
     CDS             106..1398
                     /codon_start=1
                     /product="Putative CruV Capsid Protein"
                     /translation="MPKYTQAQKIAYYKKKAVVAGKGKYHLRKAVKRIRGKGSYFTDV
                     LGAIAKPLTNVLGSGAYDFGEEVKAPVGMVKNSLFDKGMDSVPAMHSTGASVRICHRE
                     YIRDISSTVAFSNTKFELNPGLSESFPWLSQLAGAFTNYKWNGLAVQLKTMAGNSVAS
                     TTSTLGIYGMVAQYNTSLDNYANKSQCENAQGAVSAIISNSLMLGVECDNSASPFKNL
                     YVRQGAVPDGNPPQMYDLCNVQIFSSGAQAAQTCAELWITYDIELLNPLMSSHQTQGL
                     ATLFDIPVDCTVASGNFFGSSPSTLVPSFDDIGVTLSKNTGAATGIITFPVGSDGSYL
                     INYSTHGTAATVVAPPTVTYSNCTTVKLYKHVSPVTNLISSTESGASTSLDFVMNVCI
                     LVTDPSVVATVTFNGGVLPSTSNLSTLLITQLPADILA*"
     CDS             complement(1941..2930)
                     /codon_start=1
                     /product="Putative Replication Associated Protein"
                     /translation="MNFQFNNDKLITKENDSDKENKNPNIDKKFRINAKAFFITYPQC
                     NLDKNKIYDLLPEKHTITEYYIAEEKHEDGNPHIHALIKYSYKRNITNCKHFDIEGFH
                     PNVQAVKNWNNSKAYVCKDNNFISYVSLDTSTPENYNKRKKDFNEWKEDQELLSFKDI
                     TYPIKFLHYKIDKPNPNIKKRHYWFIGAPDFGKTYAIEKTFAGLKIFKRGNNKYPFEG
                     YNNEDIIIYDDIEKISFEEISNVSNTYHTKTLVYGDTRYNKIYWKIGHTRTIIVVTNT
                     YPHYNKLQNAFESRFYTIMLFNDRILIDDPMEDKDLILPNNIPILERQKAIYF*"
ORIGIN
        1 taatattacc aattctggtg tttctggcgt caatatttat ttatatatag atttatgttt
       61 tatctatatt atatagattt atatttcttg caaagaaaat aaataatgcc taaatacacc
      121 caagctcaaa aaatagctta ttataaaaag aaagctgttg tagctggtaa aggaaaatat
      181 catttacgta aagcagttaa acgtattaga ggtaaaggat cgtattttac tgatgtttta
      241 ggtgctattg ctaaaccatt aactaatgtt ttaggatcag gtgcttatga ttttggagaa
      301 gaagtaaaag cacctgttgg tatggtaaaa aattctttat ttgataaagg tatggattca
      361 gttccagcta tgcatagtac tggagctagt gttcgaattt gtcatagaga atatattcga
      421 gatatatctt ctactgtagc ttttagtaat actaaattcg aattaaatcc tggtttatca
      481 gaatcatttc catggctttc tcaattagca ggtgctttta ctaattataa atggaatggt
      541 ttagcagttc aacttaagac tatggctggt aattcagtag cttcgactac ttcaacttta
      601 ggtatttatg gaatggtagc tcaatataat acttcacttg ataattatgc aaataaatct
      661 caatgtgaaa atgcacaggg tgcagtttcg gctattatat ctaattctct tatgttagga
      721 gtagaatgtg ataattctgc ttctcctttt aaaaatttat atgttcgtca gggtgcagta
      781 cctgatggta atcctcctca aatgtatgat ttatgtaatg ttcagatatt ttctagtgga
      841 gctcaagctg cgcagacttg tgcagagctt tggataacat atgatattga attactaaat
      901 cctttgatgt ctagtcatca aactcaagga ttagcaactt tatttgatat acctgtagat
      961 tgtactgttg ctagtggtaa tttctttgga tcttctccta gtactttagt accatctttt
     1021 gatgatattg gagttacatt gtcaaaaaat actggtgctg ctacaggcat tattactttt
     1081 ccagtaggat ctgatggttc ttatttaatt aattatagta ctcatggaac tgcagctaca
     1141 gttgtagcac ctcctactgt aacatattcg aactgtacta cagttaagtt atacaaacat
     1201 gtttcacctg ttactaatct tatttcatct acagagtctg gtgcttctac atctttggat
     1261 tttgttatga atgtttgtat tttagttaca gatccttcag tagtagctac agttacattt
     1321 aatggaggag tactaccatc tacttctaat ttgtctacat tgcttattac tcaattacct
     1381 gctgatattc ttgcttaatt aatttattaa tttatttatt tatggaagaa tatataaagt
     1441 atatatacaa cataaaatta ttatgggttt gtttagtagg cttcgtgagt tttttaaaga
     1501 tttgttgtgt attagtaggt gttgtattca tgagatagtt gttttagaaa aacaagaaaa
     1561 gaaaaataaa catgtaaaaa agtaaaaaat aggagtatta attatttttt tttacaattc
     1621 gcagacggcg aagccggcaa gaattttaaa aaaaatataa ttaaacggac ttataagttt
     1681 ttttaaaata aaatattaag tttgtttaag tctgaaagct aaaatttttc ttcttatttt
     1741 acaacgcaaa aactaaaacg gaaaaaagcg ttggaattac ccggggggtt ctcgtagagg
     1801 gcccccccgg gtaattccaa cgcgtttgat gtttattagt ttaaagtata agtattgtat
     1861 tttctttaaa taaacccgcg aagcggggtg gggttttagg gtgctaaccc taaggctaga
     1921 cagtgacata aagtattttt ttaaaaatat atagcttttt gtctttctag tataggtata
     1981 ttatttggaa gtataagatc tttatcttcc atagggtcat ctattagtat acgatcgtta
     2041 aataacatta ttgtatagaa tcttgattcg aaagcatttt gtaatttgtt ataatgtgga
     2101 taagtatttg ttaccactat tattgttcta gtatgtccta ttttccaata aattttgttg
     2161 tatcttgtat ctccgtatac tagtgtttta gtgtgataag tattgcttac attactaatt
     2221 tcttcaaaag atattttctc tatgtcatca tagatgataa tatcttcatt attatatcct
     2281 tcgaatggat atttattatt tcctctttta aaaattttaa gtcctgcgaa agttttttct
     2341 atagcataag tttttccaaa atctggagct ccaataaacc aataatgtct ttttttgata
     2401 ttagggtttg gtttatcaat tttataatgt agaaatttga taggatatgt gatatcttta
     2461 aaagataata attcttggtc ttctttccat tcgttaaaat cttttttccg tttgttgtag
     2521 ttttcaggtg tgcttgtatc aagacttaca taactgataa agttattgtc tttacataca
     2581 taggctttac tgttattcca gtttttaact gcttgaacat ttggatgaaa tccttctata
     2641 tcaaagtgtt tacagtttgt gatatttcgt ttatagctat atttaattag agcatgtata
     2701 tgcggattac catcttcatg tttttcttct gctatgtagt attctgttat agtatgtttt
     2761 tcaggaagta aatcgtagat tttattctta tccaaattgc attgaggata agtaatgaaa
     2821 aaagctttag cgttgattct aaatttttta tctatgtttg gatttttatt ttctttatca
     2881 gagtcgtttt cttttgttat taatttatca ttattaaact gaaagttcat taagaaagag
     2941 ccagaattgt at
//
LOCUS       CruV-115                2954 bp    DNA     circular     24-FEB-2020
DEFINITION  Crucivirus-115 isolate 120_1745_IR2_3297.
ACCESSION   CruV-115
KEYWORDS    .
SOURCE      Crucivirus-115
  ORGANISM  Crucivirus-115
            Unclassified.
REFERENCE   1  (bases 1 to 2954)
  AUTHORS   de la Higuera,I., Kasun,G.W., Torrance,E.L., Pratt,A.A.,
            Maluenda,A., Colombet,J., Bisseux,M., Ravet,V., Dayaram,A.,
            Stainton,D., Kraberger,S., Zawar-Reza,P., Goldstien,S., Briskie,J.,
            White,R., Taylor,H., Gomez,C., Ainley,D.G., Harding,J.S.,
            Fontenele,R.S., Schreck,J., Ribeiro,S.G., Oswald,S.A., Arnold,J.M.,
            Varsani,A., Enault,F. and Stedman,K.M.
  TITLE     Direct Submission
  JOURNAL   Submitted (24-FEB-2020) Biology, Portland State University, 1719 SW
            10th Ave, Portland, Oregon 97201, USA
COMMENT     Bankit Comment: ALT EMAIL:ide@pdx.edu
            Bankit Comment: TOTAL # OF SEQS:451
            Bankit Comment: GAP: est. length - alignment

            ##Assembly-Data-START##
            Assembly Method       :: MetaSPAdes v. 3.12; IDBA-UD v. 1.1.1;
                                     MEGAHIT v. 1.0.3
            Sequencing Technology :: 454; Illumina
            ##Assembly-Data-END##
FEATURES             Location/Qualifiers
     source          1..2954
                     /organism="Crucivirus-115"
                     /mol_type="genomic DNA"
                     /isolate="120_1745_IR2_3297"
                     /isolation_source="Water"
                     /country="Norway"
                     /collection_date=2011
     CDS             1..1065
                     /codon_start=1
                     /product="Putative Replication Associated Protein"
                     /translation="MENYTETLITPKTPKTPKNGFRVTAKNLFITIKGHIDMSYIHFI
                     HNKFPIQTWFYCHETGKTRTYPHSHIMLKLFNKANITDPRSLDFNDVHPDIQALKNWE
                     QSICYGTKEGTFFTNIPESVFKKILNKNKSAGGNGFKQVINNIVNSKNTIEAFENNAT
                     DFKDIVAIERIFANKNLIIDKELVDKYLNSTYLDWQSTIANWLSQPPSDRTVHWIVDK
                     IGGNGKSFFSRKFRFEHQDNCLIIGSTGKPSDIADVIRNWMTQGNIPKYIFIDLPRSC
                     EYKDNIYTSIESIKNGMITCTKYKGQTLEFSSPHVIIFSNWYPSIKKLSKDRWNIHCL
                     DNKILKHMTLEDMKCIDPFA*"
     CDS             complement(1527..2900)
                     /codon_start=1
                     /product="Putative CruV Capsid Protein"
                     /translation="MAARKAMVRKMRGPGADYKYRGDWDDDDRIYDTLNNRRNASVYY
                     NRGFPKPMKRAKKKPVSRRSKIFAPIGGMLGGAAGGWLGGPAGAVLGSSIGSSAGSLI
                     SKITGFGSYQVARNTLVNNSIPQFQSRLGDGSIRIKHKEFISDVVSSATPGAFNPIQY
                     HISASDQNTFPYLSQLAVNFEEYAFEGLVFQYITSSGSISTTGQLGTVVGATQFNALA
                     APYANKQQMEASTFGQSTVASQSCLFPVECDAKQTPSNGIFYNQRPGISNVDNDLRWS
                     RLGNFTLATVGMPNASETVGELYVSYDIVLMKPILIQDSGASADHWQLGSGIVAGSVE
                     FGTNPVLTAQSDNFTRLGGTTILINPSFNGNLCVMYYVNGVAGLWVSPDMTAGVGVTD
                     LNVINLGTANNLNVATAATSAYAYSVGFFTVQSTGVESVITLGSGTYFSPTNGDLYIM
                     QIPADFD*"
ORIGIN
        1 atggagaatt atacagaaac actgataaca ccaaaaacac caaaaacacc aaaaaatgga
       61 tttagagtca cagctaaaaa tctatttatt actattaaag gtcatattga catgagttat
      121 attcatttca ttcataacaa gtttcctata caaacttggt tctattgcca tgaaactggc
      181 aaaacaagaa catatcctca ttcacatatt atgcttaagc tctttaacaa agctaacata
      241 accgatccaa gatctcttga ctttaatgat gtacatcctg acattcaagc tctcaaaaat
      301 tgggaacaaa gtatttgtta tggaacaaaa gaaggtactt tcttcactaa cattcctgag
      361 agtgtattta aaaaaatact caacaaaaac aaaagtgctg gaggcaatgg atttaaacaa
      421 gtaattaaca atattgttaa ttctaaaaat accatcgaag ctttcgaaaa taacgctacc
      481 gatttcaaag atatcgtagc tattgaaaga atctttgcta acaaaaatct tatcattgac
      541 aaagaacttg ttgataaata tctcaactcg acctacttgg attggcaatc aactatcgct
      601 aattggctat cgcaaccacc aagtgatcga acagtccatt ggattgttga caaaattggt
      661 ggtaatggta agtcattctt ttcaagaaaa ttcagattcg agcaccaaga taattgtcta
      721 attattggtt ctacaggtaa accaagtgac atcgctgatg taatccgaaa ctggatgacc
      781 caaggcaata tacctaagta tattttcatc gacttacctc gatcctgtga gtataaagat
      841 aacatttata ccagtattga atctatcaaa aatggtatga tcacttgcac taagtacaag
      901 ggacagactc ttgaattctc tagtccacat gtcatcattt tctctaattg gtatccaagc
      961 attaaaaaac tatctaaaga cagatggaac atccattgct tggacaataa aattcttaaa
     1021 catatgactc tcgaagacat gaaatgcatc gatccttttg cttaaatact tgtggggaaa
     1081 gtggtttacc acgtttcccc ttcgcttttg gatcactttc gtgctcaaaa gctcttcaca
     1141 agtattatcc ttatgttatt tctattagaa ataatataat gtgaccttcg tccctcctgc
     1201 accaaaaatg gctcaagaga atggatcccc ccacatcgct cgcaagctcg ctcggaggcc
     1261 cccccattca ctttcgcaat ttttggctcc gggggactac ggtttcttgg ggagtcgcta
     1321 cgctcctttc ttgaaagtgc tctggggacc gttgatcgct ccgctcctag gtccgactga
     1381 tccttttcaa gttcaagaat ttttccgaaa agggaaccct cttatttttt tagatttatt
     1441 ctttacagaa ctcttacgag gatcctaaaa agagtccctt ttgccggggt aacactatcg
     1501 ctacgctctt cccggcttta aattatttag tcaaaatctg caggtatttg cataatatac
     1561 aaatccccat tcgtgggcga aaaataagta cctgaaccta aggttattac actttcaaca
     1621 cctgttgatt gaactgtaaa aaaaccaaca gaatatgcat atgcagacgt cgctgcagtt
     1681 gcaacattca aattatttgc tgtacctaaa ttgattacat tcagatctgt gactccaact
     1741 cctgccgtca tatctggaga tacccacaat cctgctactc catttacata atacataaca
     1801 caaagattac cattaaaaga tggatttatt aaaatagtag taccacctaa tctagtaaaa
     1861 ttgtcagatt gtgctgttag aaccggatta gtaccaaact ctactgaacc agcaacaatt
     1921 cctgaaccaa gctgccaatg atcagctgat gcaccagaat cttgaatcaa gattggtttc
     1981 atcaaaacaa tatcataaga gacataaagc tctcctacag tctcacttgc attcggcata
     2041 cctactgtag ctaatgtaaa attacctaat ctagaccatc taagatcatt atcgacattc
     2101 gatataccag gtctttgatt ataaaatata ccattcgaag gcgtctgctt tgcatcacat
     2161 tctactggaa acaaacaaga ttgactcgca accgtactct gaccaaatgt cgaagcctcc
     2221 atttgttgtt tattcgcata aggcgctgct aacgcattga attgagtcgc acccacaacc
     2281 gtacccaatt gacctgtcgt agaaattgaa ccactcgatg ttatatattg aaacacaaga
     2341 ccttcaaaag catactcttc aaagttgact gccaactgtg aaagatatgg aaatgtgttt
     2401 tgatcagatg cagaaatatg atactgaatt ggattaaatg ctccaggagt tgcagatgac
     2461 acaacatcag atataaactc cttatgttta attctaatag aaccatctcc aagtctactc
     2521 tgaaactgtg gaatggaatt atttaccaat gtattcctag caacttgata tgaaccaaaa
     2581 cctgtaattt tagatatcaa actgcctgca gacgatccta tagaagaacc taatactgca
     2641 cctgcaggac ctcctaacca acctcctgca gctcctccta acattcctcc aataggagcg
     2701 aatatcttag atcgtctcga tactggcttt ttcttagccc tcttcatagg tttaggaaaa
     2761 cctctattat aatagacaga tgcattacga cgattattta atgtatcata aattcgatca
     2821 tcatcatccc aatcacctcg atacttatag tccgctcctg gtcctctcat ctttctaacc
     2881 atcgcttttc tcgctgccat attataatat aagaaaataa ttattatata taattatttt
     2941 attggtgtta ttat
//
LOCUS       CruV-116                2963 bp    DNA     circular     24-FEB-2020
DEFINITION  Crucivirus-116 isolate 120_1105_SvL1_3295.
ACCESSION   CruV-116
KEYWORDS    .
SOURCE      Crucivirus-116
  ORGANISM  Crucivirus-116
            Unclassified.
REFERENCE   1  (bases 1 to 2963)
  AUTHORS   de la Higuera,I., Kasun,G.W., Torrance,E.L., Pratt,A.A.,
            Maluenda,A., Colombet,J., Bisseux,M., Ravet,V., Dayaram,A.,
            Stainton,D., Kraberger,S., Zawar-Reza,P., Goldstien,S., Briskie,J.,
            White,R., Taylor,H., Gomez,C., Ainley,D.G., Harding,J.S.,
            Fontenele,R.S., Schreck,J., Ribeiro,S.G., Oswald,S.A., Arnold,J.M.,
            Varsani,A., Enault,F. and Stedman,K.M.
  TITLE     Direct Submission
  JOURNAL   Submitted (24-FEB-2020) Biology, Portland State University, 1719 SW
            10th Ave, Portland, Oregon 97201, USA
COMMENT     Bankit Comment: ALT EMAIL:ide@pdx.edu
            Bankit Comment: TOTAL # OF SEQS:451
            Bankit Comment: GAP: est. length - alignment

            ##Assembly-Data-START##
            Assembly Method       :: MetaSPAdes v. 3.12; IDBA-UD v. 1.1.1;
                                     MEGAHIT v. 1.0.3
            Sequencing Technology :: 454; Illumina
            ##Assembly-Data-END##
FEATURES             Location/Qualifiers
     source          1..2963
                     /organism="Crucivirus-116"
                     /mol_type="genomic DNA"
                     /isolate="120_1105_SvL1_3295"
                     /isolation_source="Water"
                     /country="Norway"
                     /collection_date=2012
     CDS             1..951
                     /codon_start=1
                     /product="Putative Replication Associated Protein"
                     /translation="MSKKVVKSKYLSEKPVEPLQDRVSETHDSRGFQTHLDTGASNKV
                     VAPILENELKNDEKDDKKRRSWFLTWHFSDDDTQDTLDTVKMRLMTWWDQYSFQLEKC
                     PTTGRRHFQGAFNMANARSWKQIVAAVGEGGFWKPCRSYKASKKYCTKNPTRIEGPYT
                     NVEDDIIKDPFDIRLMELVENLWQKDVIGLTQAEADARLIYWFWEKIGGRGKSFLAKH
                     ICLVNERAIYVGGKVADIKCAIADLEKKPNVVLFDVARAAFGGIDYSALEEVKNGIFF
                     SGKFKSAMVIMNIPQIIVFANAPPDQRQLSADRWRIIEIQ*"
     CDS             complement(1137..2483)
                     /codon_start=1
                     /product="Putative CruV Capsid Protein"
                     /translation="MPRKAMKRGYTQAEKLEYYKRKSQRAYRGRGAYSKPRRDAPISR
                     AYGESMGNLVGSAASFIPGIGAAVGPVASVLASKLGGYLGNKLGSYMGWGDYQVKFNS
                     LVDEGNSPAAMHNAGKATRICHREYIGDVLSSAVAGQFQLARYAIQPGLRANFPWLSP
                     VALQYQKYRLLGAIVEFKSGSGDAITGTNTALGEVIIASNYNCADANFVNRQQMENTQ
                     YCSSAKPSISFVHVIECDPELQTNVNLYISPSEVPPTGMTANEINWVNVQVATVGCQG
                     GSVNLGSLYITYDVELIQPIDDSIDRQPKGDYFSASGASVLNNAIMGTTPVTDPTNSI
                     GGSFTATNTYSFPASLASGVYKVEYTVLGSAAVWTAPVLTFTNAVTRNCMGNGTFPAA
                     GFPQNALAGVITGCIIYTFELTGPNASITLGVAGTIPTSVTMATFMIDQIDGDFAR*"
ORIGIN
        1 atgtctaaaa aagttgttaa gtcaaaatat ttgtctgaaa aacctgttga gccattgcaa
       61 gaccgtgtat ctgaaactca cgattctcgt ggtttccaga cacacttgga cacaggggct
      121 agtaataaag ttgtagcccc tattttagaa aatgaattga aaaatgatga gaaagatgat
      181 aagaaaagaa gaagttggtt tttgacttgg catttctccg atgacgatac ccaagacaca
      241 ctagacacag ttaaaatgag gttgatgact tggtgggatc aatattcttt ccaacttgaa
      301 aagtgtccta ctacgggcag gcgtcacttt caaggtgctt ttaatatggc taacgcaaga
      361 tcatggaaac agattgtagc tgctgtcggc gagggcggtt tctggaaacc ttgtcgtagt
      421 tataaggcat ctaagaaata ctgcacaaaa aacccaacac gtatagaagg tccttatacg
      481 aatgttgaag atgacataat caaagatcca tttgatatta gattaatgga attagttgaa
      541 aatttatggc aaaaagatgt tataggtcta acgcaagctg aagctgacgc tagactaatt
      601 tattggtttt gggaaaaaat aggtggtaga ggaaaatcat ttcttgcgaa acatatatgt
      661 ttagtgaacg aacgtgctat ttatgtgggt ggaaaagtag cggatatcaa atgtgctatt
      721 gcagacttgg aaaagaaacc aaacgtagtg ctcttcgacg ttgcacgggc agcattcggg
      781 ggaatagact actcggcact cgaagaggta aagaatggaa tatttttcag tggaaaattc
      841 aaatctgcaa tggttataat gaacatacca caaataatag tcttcgctaa cgcaccgcct
      901 gatcaaagac aattatctgc ggatcgttgg aggataattg aaattcaata aaattcgagg
      961 aattcaatga aattgtaaat ggctcaaatg atgtgaagtt taacctacgg tgaatttgta
     1021 acggcctgcg gcccgctcca aaacccttga atcattgcga ttgtaaattt aattactcga
     1081 ataagattga aataagattg gaataagatt ggaataaaac aagatttatc aaattattag
     1141 cgagcaaaat caccatcgat ctggtcgatc atgaatgttg ccatggttac cgacgttgga
     1201 atagtgcctg cgactccaag tgtaattgat gcatttggac ctgttagttc aaatgtataa
     1261 attatacaac ctgtgattac tccagccaac gcgttttgtg ggaaccctgc agctgggaat
     1321 gttccattac ccatacaatt tcgcgtaact gcgtttgtga aagtaagtac tggagctgtc
     1381 catactgctg ctgatcccaa gacggtatat tcaactttgt atacacctga tgcaagtgat
     1441 gctggaaaag agtatgtgtt ggttgcggta aacgaaccac caatactgtt tgttgggtcc
     1501 gtaactggcg ttgtacccat aattgcattg ttaagaacgc tcgctccact cgctgagaaa
     1561 taatcacctt tgggttgtcg atcgatagaa tcatcaatgg gttgaatgag ttctacgtcg
     1621 taagtgatat ataatgaacc aaggttaacg ctaccacctt ggcagccaac tgttgctacc
     1681 tgcacgttaa cccaattgat ttcattcgca gtcattccag taggtggaac ttcgctcggt
     1741 gaaatataaa ggttaacgtt tgtttgcaat tctggatcac actcaattac gtgaacaaat
     1801 gaaatcgacg gtttcgcaga cgaacaatac tgcgtattct ccatctgctg tcggttaacg
     1861 aaattggcgt cggcgcagtt ataattgctc gcaatgataa cttcacctag ggcagtgttt
     1921 gtgcctgtga ttgcatcacc tgagcctgac ttaaattcaa ctatagctcc aaggagtcta
     1981 tatttttgat attgaagtgc aacaggtgat agccatggga aatttgcacg caatcctggt
     2041 tggatggcgt accttgccag ttgaaattgt cctgcaactg cagaagagag cacatctccg
     2101 atatactctc tatggcaaat gcgggtggct ttgcctgcgt tatgcattgc ggctggtgaa
     2161 ttaccttcgt caaccaacga gttgaacttg acttgataat ctccccatcc catataagat
     2221 cctagcttat tgcctaggta tccaccgagt ttggatgcga gtacggacgc aacgggtcct
     2281 actgcggcac caatccctgg tataaagcta gcggctgatc caactaaatt gcccatggat
     2341 tctccatagg cccttgagat tggagcatca cgtctaggtt ttgagtaagc accacgacct
     2401 cggtaggcac gctgggactt acgtttatag tattcaagct tttcagcttg agtatagcct
     2461 cgtttcattg cttttctcgg cataataatg tgtgtctttg tctggggtgg gcttttctta
     2521 gaaaccacga aaatttataa accaccaaaa attctcggtg tttctacgaa accctcaaat
     2581 tgtcgatgtt tgataacttg tcgtggttta taagcagttt gaaggtttca aagttgggag
     2641 ccgaggtttc tctgaaacac tcgtcgcgcc aaactcgagg tttctaagaa acacccactt
     2701 cgtggttgtt tctgataaac atcgactgtt gccgaattca gtgtttctac gaaacaccca
     2761 ctacgtggtt gttttacttg aaacaccgag ttcgagctgt cgtggtttta ccacggactt
     2821 atgatttttt cgtccgattt tttcttaaaa atttctcaga ataagttgcg cggccgaagt
     2881 tcgacatttc tttcagaaac atcgaacgtt cgtcaaggcg caacattttc taacgctacg
     2941 acaaaaccca gctaccacct acc
//
LOCUS       CruV-117                2965 bp    DNA     circular     24-FEB-2020
DEFINITION  Crucivirus-117 isolate 120_213_ArcticSummerIll_3300.
ACCESSION   CruV-117
KEYWORDS    .
SOURCE      Crucivirus-117
  ORGANISM  Crucivirus-117
            Unclassified.
REFERENCE   1  (bases 1 to 2965)
  AUTHORS   de la Higuera,I., Kasun,G.W., Torrance,E.L., Pratt,A.A.,
            Maluenda,A., Colombet,J., Bisseux,M., Ravet,V., Dayaram,A.,
            Stainton,D., Kraberger,S., Zawar-Reza,P., Goldstien,S., Briskie,J.,
            White,R., Taylor,H., Gomez,C., Ainley,D.G., Harding,J.S.,
            Fontenele,R.S., Schreck,J., Ribeiro,S.G., Oswald,S.A., Arnold,J.M.,
            Varsani,A., Enault,F. and Stedman,K.M.
  TITLE     Direct Submission
  JOURNAL   Submitted (24-FEB-2020) Biology, Portland State University, 1719 SW
            10th Ave, Portland, Oregon 97201, USA
COMMENT     Bankit Comment: ALT EMAIL:ide@pdx.edu
            Bankit Comment: TOTAL # OF SEQS:451
            Bankit Comment: GAP: est. length - alignment

            ##Assembly-Data-START##
            Assembly Method       :: MetaSPAdes v. 3.12; IDBA-UD v. 1.1.1;
                                     MEGAHIT v. 1.0.3
            Sequencing Technology :: 454; Illumina
            ##Assembly-Data-END##
FEATURES             Location/Qualifiers
     source          1..2965
                     /organism="Crucivirus-117"
                     /mol_type="genomic DNA"
                     /isolate="120_213_ArcticSummerIll_3300"
                     /isolation_source="Water"
                     /country="Norway"
                     /collection_date=2010
     misc_feature    join(2961..2965,1..15)
                     /note="stem-loop"
     misc_feature    1..9
                     /note="nonanucleotide"
     CDS             176..1060
                     /codon_start=1
                     /product="Putative Replication Associated Protein"
                     /translation="MYVYPIPELEDVSNYTGTCAVWDFRANEDLYTSSVLLKFLKGNC
                     KLYVFQKEKSDTGYVHWQGRFSLIKKRNKSSLLKLFEIIGKPNYLAPTSETNRNESFF
                     YAMKEDTRIEGPFMDPKHKIDNDELYIPKQFRDKALYPFQQTILDSYNEFTDRIVDVI
                     VDIDGNNGKSFISAYASLKVNAVNIPMLNDFKDIISFVCNRCQDMGTHDPKLIFCDMP
                     RALNKRHLDGFYSAIEQIKKGELFDTRYHAKSWWIDSPRIWVFTNYLPDNNLLSRDRW
                     RLWRIVNKVLVPYFNDNE*"
     misc_feature    388..415
                     /note="stem-loop"
     misc_feature    399..407
                     /note="nonanucleotide"
     CDS             1123..2472
                     /codon_start=1
                     /product="Putative CruV Capsid Protein"
                     /translation="MPRAPAKRAVSFKKKSAVSSMKKSNTAGTGKYKTSSRSGNHSRR
                     DRGSHPFENLGTGIGKMLGSWTGVGDYVEELASASAPRSGVVLAPDPPRFKNDKDGTT
                     IIHREYLGDILTSSVAGAFNVNQYGLNPSDSNTFPWLSQLCQPNFQQYKFEQLIFEFR
                     TFSSDALNSTNTALGSVFGCINYDYSDPSVSSRTEVENTDWSNSCKPSQSMLIPVECD
                     PKQTGLNSGLLYIINGNNVPAGADPKTYYLGKLFIGTTGLQAASVNIGSLYVSYRVKL
                     YKPFMTKPLSNGLITMLARTGASAGFLWGNANTNFFGNCDSIGVSFAGNVMTINRTRL
                     QVGQIFRLFFSVTGTAVAGGTPATLALSANATGVTYFSTIPGGNGFDTTVVSFPPSPA
                     STTQVAFEVFFIILNNNSDVTATLSLGVYPTGAQMSNLQITQVCGIPSQQIGFYLPN*
                     "
ORIGIN
        1 taaaattaca tgttttatca tttaaattta ttttttatct ttattattag ataatatcaa
       61 acatgcataa atagaaaaaa aaaacgcact tattttttgt aaaaaaattt tttctggtat
      121 agatttatat atggaaaagc tggaagattt ggaaaataaa atatttgaca gaaaaatgta
      181 cgtttatccc atccctgaat tagaagatgt ttctaattac actggaacat gtgcagtatg
      241 ggattttaga gctaacgagg atttatacac tagctcggtt ttattaaagt ttttaaaggg
      301 taactgtaag ttatacgtct ttcaaaaaga gaaaagtgac actggttatg tacattggca
      361 gggtcgtttt tctttaatta aaaaaaggaa caaaagttca ttattaaaat tgttcgaaat
      421 aataggtaag cctaattatt tggcacctac aagtgagact aatcgtaatg aatcattttt
      481 ttatgcgatg aaggaggata caaggatcga aggtcctttt atggatccta agcataaaat
      541 tgataatgat gaattataca taccaaaaca gtttcgcgac aaagcattat atccttttca
      601 acaaacgata ttagattcat ataatgaatt tacagatcgt atagttgatg ttattgtaga
      661 cattgacgga aataacggta aaagttttat aagcgcatat gcttcattaa aagttaatgc
      721 tgtcaatata ccaatgttaa atgattttaa agatattata tcctttgtat gtaatagatg
      781 tcaggatatg ggtactcacg atcctaaact tattttttgt gatatgccta gggcattaaa
      841 taaacgtcat cttgacggtt tttattccgc cattgaacaa ataaaaaaag gggagttatt
      901 tgatacccga taccatgcaa aatcatggtg gattgacagt ccaagaatat gggtctttac
      961 taattattta cctgataata atttacttag tcgtgatcgt tggcgattat ggcgaattgt
     1021 taataaagtt ttagttcctt attttaatga taacgaataa taaaaacgca tttaaaaaat
     1081 ttatactata taagtataaa tttttttcta tatttatttt atatgcctcg tgctcccgct
     1141 aaaagagctg tttcttttaa aaagaaatca gccgtttcat ctatgaaaaa atccaatact
     1201 gccggtaccg gtaagtataa aacatcttct cgctcaggaa atcatagccg cagagataga
     1261 ggatctcatc cttttgaaaa tctgggaacc ggaattggaa aaatgttagg aagctggaca
     1321 ggagtaggtg attatgttga agaactagca tctgcttctg cacctagatc aggtgttgtt
     1381 ttagcacctg atccacctag atttaagaat gataaagacg gtactaccat tatacatcgt
     1441 gaatatctcg gtgatatttt aacatcatca gttgctggtg cattcaatgt taatcaatac
     1501 ggacttaatc cttcggatag taacacgttt ccgtggttgt ctcaattatg tcaaccaaat
     1561 tttcaacaat ataaattcga acaactaatt tttgagttta gaacatttag ctctgacgct
     1621 ttaaattcaa ctaatacggc acttggttcc gtatttggtt gtattaatta tgattatagc
     1681 gatccgtcag tttcatctcg tacagaggtt gaaaatacag attggtctaa tagttgtaag
     1741 ccttcacaaa gtatgttaat acctgttgaa tgcgatccaa aacaaacagg cttaaattct
     1801 ggattgttat atattattaa tgggaacaac gttcccgccg gtgcagaccc aaaaacatat
     1861 taccttggta aattgtttat aggtacaacc ggattacaag ctgcaagcgt taatattggc
     1921 tcattatacg tttcatatcg cgtaaaatta tataagcctt tcatgacgaa accattgagt
     1981 aatggcttaa ttactatgtt agcccgtaca ggtgccagtg ctggtttctt atggggtaac
     2041 gcaaatacaa atttttttgg taattgtgat agtataggcg tatcatttgc tggaaatgtt
     2101 atgactataa atagaacccg attacaagta ggacaaatat tcagattgtt cttctctgtt
     2161 actggtactg ccgttgctgg tggaactcct gctactttgg cgttatctgc aaatgcaacg
     2221 ggtgttacat acttttctac catacctggt ggaaatggat ttgatactac cgttgtcagt
     2281 ttcccccctt cgcctgcttc tactacacag gtagcatttg aggtattttt tattatatta
     2341 aataacaatt ccgatgtaac tgctacactg tcgttaggag tttacccaac aggtgcacaa
     2401 atgagtaatt tacaaattac acaggtgtgc ggtataccat cgcaacagat tgggttttat
     2461 ttacctaatt aatttattta taaattaatc tatcaattat cattaataca ttttatgcgt
     2521 tatacttaaa atgagtcaag atatataaac ccggcctcgt agaggtccaa taggttaggt
     2581 cgcgctagtg acgggtccct acgggcaaaa tcagtccctt ttggttggta cgactaaaag
     2641 gttttcatta tatttaaaaa attaattaat atataaacta tctgtataca atgcctgatt
     2701 gtttaaaaga ttttttggaa attattcttc ttattataag aatgatattt tctaaaaatc
     2761 aaaattccaa taattccaat aattccaatt gttaaaggat atatatatat gtatgaaata
     2821 tcatatatat ataatttaaa agcgtgtgcg ttcttccaga taatgttaat tatcgcccac
     2881 acgctgcgaa gctacacacg tcaccgtgta gcgtagcatt ctatttattt attttttatt
     2941 tttatttcat cataaatatc aaaca
//
LOCUS       CruV-119                2966 bp    DNA     circular     24-FEB-2020
DEFINITION  Crucivirus-119 isolate SR1_16099.
ACCESSION   CruV-119
KEYWORDS    .
SOURCE      Crucivirus-119
  ORGANISM  Crucivirus-119
            Unclassified.
REFERENCE   1  (bases 1 to 2966)
  AUTHORS   de la Higuera,I., Kasun,G.W., Torrance,E.L., Pratt,A.A.,
            Maluenda,A., Colombet,J., Bisseux,M., Ravet,V., Dayaram,A.,
            Stainton,D., Kraberger,S., Zawar-Reza,P., Goldstien,S., Briskie,J.,
            White,R., Taylor,H., Gomez,C., Ainley,D.G., Harding,J.S.,
            Fontenele,R.S., Schreck,J., Ribeiro,S.G., Oswald,S.A., Arnold,J.M.,
            Varsani,A., Enault,F. and Stedman,K.M.
  TITLE     Direct Submission
  JOURNAL   Submitted (24-FEB-2020) Biology, Portland State University, 1719 SW
            10th Ave, Portland, Oregon 97201, USA
COMMENT     Bankit Comment: ALT EMAIL:ide@pdx.edu
            Bankit Comment: TOTAL # OF SEQS:451
            Bankit Comment: GAP: est. length - alignment

            ##Assembly-Data-START##
            Assembly Method       :: MetaSPAdes v. 3.12; IDBA-UD v. 1.1.1;
                                     MEGAHIT v. 1.0.3
            Sequencing Technology :: 454; Illumina
            ##Assembly-Data-END##
FEATURES             Location/Qualifiers
     source          1..2966
                     /organism="Crucivirus-119"
                     /mol_type="genomic DNA"
                     /isolate="SR1_16099"
                     /isolation_source="Water"
                     /country="New Zealand"
                     /collection_date=2012
     misc_feature    join(2958..2966,1..22)
                     /note="stem-loop"
     misc_feature    1..9
                     /note="nonanucleotide"
     CDS             11..1369
                     /codon_start=1
                     /product="Putative CruV Capsid Protein"
                     /translation="MLPKNVYMNLSKKDKAKVKRNIASKAKGSGAYDLATQPYGYYGS
                     KIGRALGTSFYGPAGGAVGDVAAGYLGHRIGRMFGSGAYKSKRIPNYTGAHKMIRGSG
                     AYDMPAQGRVITEGTSPPSFGSNRTIIRHREYIGNLNSSTAFNLNSYNVNPGDETTFP
                     WLAGIATNYQKYRPLGVLFEFKSTSANALNSTNTALGTVMMAVSYNAIASSAPGSKME
                     MLQLEGCTSICPAESAMCAVECAKNYNPLSTLYIRNGVAFQVPNGAEQFYDFANFYIA
                     TEGMQAANVVIGELWATYEFELLTPVLNDGQVGNDVNFSNYLLSSGASTSNYFPSPSA
                     SLNIQTMNLTFTATTISLPANITTGCYQLLWYLVGSTGASSAPSIAFSNAVALPIFSG
                     NTVSRPTSTAQSATTQIVVQSFKVTGPNATLTFSGGTILTGSTGGSVELVQLPGVVGQ
                     GI*"
     CDS             2045..2932
                     /codon_start=1
                     /product="Putative Replication Associated Protein"
                     /translation="MKQCYTYDMRLSQAKSDGKSSYQKDEVGKLLQSIAKKFVFQLEK
                     GEETGYVHFQIRASLHKKVTKFGLIKLIQDILKCDIKDVPQFIEPSLVDVHNKKNFNY
                     VLKEQTKLDGPWTDATFALEREVYIPRQYKGIIDKLYPYQKVIFDSGDVFDDRIINVI
                     VDFEGNNGKSTIASICELYGKGMDVPPLNDMKEIIQMICDECMASGNRSPSPLLLDMP
                     RAMNKEKLYGFYSALEQIKKGKLYDPRHSYKKFWIDSPQIWVFTNNIPNIEFLSKDRW
                     RFWSINEKKELVIFVDEIE*"
ORIGIN
        1 tatataaaag atgctaccaa aaaacgttta tatgaatctt tcgaaaaagg ataaagctaa
       61 agtcaaaaga aacattgctt ctaaagctaa gggttctggt gcatatgatt tagctacaca
      121 accatatggt tattatggtt ctaaaatcgg tagagcttta ggaacatcat tttatggacc
      181 agcaggaggt gctgttggtg atgttgcagc tggttattta ggtcatcgta ttggtagaat
      241 gtttgggtct ggagcttata agtctaagag gattcctaat tatacaggtg ctcataaaat
      301 gattagaggt tctggagctt acgatatgcc tgctcaagga agagttataa ctgagggaac
      361 ctcacctcca tcatttggtt caaatagaac cataattaga catagagaat atattggaaa
      421 tttaaattca tcaactgctt ttaacttgaa ttcttacaat gttaatccag gtgatgagac
      481 gactttccca tggttggcag gtatagctac gaattatcaa aaatacagac ctttaggtgt
      541 attgtttgag tttaaatcta cctctgccaa tgctttgaat tctacaaata cagcattagg
      601 aacagtcatg atggctgttt catataatgc tatagcatcg tcagcaccag gatcaaagat
      661 ggaaatgtta cagcttgaag gatgtacttc tatctgtcct gctgaatcag ctatgtgtgc
      721 tgtagaatgc gctaagaatt ataacccctt atcgactttg tatattagaa atggggttgc
      781 attccaagtt ccaaatggag ctgaacaatt ctatgacttt gcaaactttt atattgcaac
      841 agaaggtatg caagcagcaa atgtcgtgat tggagaattg tgggcgacat atgaatttga
      901 acttttaact cctgttctta acgatggtca ggttggaaat gatgttaatt tttctaatta
      961 cttattgtca tctggagcat caacaagtaa ttatttccca tctccatcag catctttgaa
     1021 tatacaaacg atgaatttga cttttacggc aactacgatt agcctccctg caaatataac
     1081 gactggatgt tatcaacttt tgtggtatct tgtcggatcg actggtgcat cttctgcgcc
     1141 cagtattgcg tttagtaatg cggttgcttt gccaatcttt tctggcaata ctgtatcaag
     1201 accaacttca acagctcaat cagcaacaac tcagattgtt gttcagtcgt ttaaagttac
     1261 aggaccaaat gcaactttaa catttagtgg aggtactata ttgacaggtt caacaggtgg
     1321 ttcagttgaa cttgttcaat tacctggagt tgtcggtcaa ggaatttgac tacgagagtc
     1381 ctcgggcata gagccctcgg acttttatga gatagtttaa atctctcttt tttatcactt
     1441 tttatacatt aaataaatga tatttaatat attttttcat tatttcataa taaaaataat
     1501 aatataaaca attcctctct cactgaaaaa aagaaaatat ttatatatat tcttagctca
     1561 ctgacgaact gaaagacgaa agagaagacg ttttcgcgag tgagcaggcc gcgccgcccc
     1621 cccgtgggcg atacggagcg aaatactcgg agcgcgagtc ttgtgaggaa atagtgagcg
     1681 tactcgaacg cggagagagt tatcctaaaa aagccatata tatccaagta gcgtttagct
     1741 tctgtatata tatcgatgag gtattatttt agtattgatt tattggtgca aacgtataaa
     1801 ttacgtgcga gataaatatg agtagggtat ccagatatcc tgattctctt atataaaaat
     1861 agatattcga agaatatgtt tccatttgtg ttgttccagg tagtttacct accgccaaat
     1921 ggaaacattt aaagtgaatt aagaagaaat ctttaaattt tattaagttt atgttttgat
     1981 aaaaatctaa acgcacttta tttcatatcc taaagaaata tctataatta ggatatatca
     2041 ggatatgaaa cagtgttata cgtatgatat gagattgagt caagcgaagt ctgatggtaa
     2101 atcatcatat caaaaagatg aagtaggtaa gttacttcag tcaattgcaa aaaagtttgt
     2161 ttttcaattg gagaaaggtg aagagactgg ttatgttcac tttcaaatta gagcttcttt
     2221 acataagaag gtaactaaat ttggattaat taaattaatc caagacatcc taaaatgtga
     2281 tataaaggat gttcctcaat ttattgaacc atctcttgta gatgtacata ataagaagaa
     2341 ttttaattat gttttaaaag agcaaactaa gcttgatggt ccttggaccg atgcaacgtt
     2401 tgctctcgag agagaggtat atatacctcg acaatataag gggataattg ataaattata
     2461 cccttatcaa aaagttatat ttgatagtgg agatgtattc gacgatagaa taattaacgt
     2521 tatagttgat tttgaaggga ataacggtaa atcaactatt gcaagtattt gcgaacttta
     2581 tggtaagggc atggatgtcc caccattaaa cgatatgaaa gaaatcattc agatgatttg
     2641 tgatgagtgt atggcgagtg gaaacaggag cccaagtcct ttattattag atatgccaag
     2701 agctatgaat aaagagaagt tatatggttt ttattcggca cttgagcaga ttaaaaaagg
     2761 gaaattatat gatccaagac attcttataa gaagttttgg attgatagcc cacagatatg
     2821 ggtcttcaca aataatatcc ctaatataga atttttgagt aaagacagat ggagattctg
     2881 gtctataaat gaaaaaaagg aattagttat ttttgtggat gaaattgaat aaataatata
     2941 gattttttat ttttttgttt tggtaa
//
LOCUS       CruV-120                2983 bp    DNA     circular     24-FEB-2020
DEFINITION  Crucivirus-120 isolate 120_252_SvL2_3298.
ACCESSION   CruV-120
KEYWORDS    .
SOURCE      Crucivirus-120
  ORGANISM  Crucivirus-120
            Unclassified.
REFERENCE   1  (bases 1 to 2983)
  AUTHORS   de la Higuera,I., Kasun,G.W., Torrance,E.L., Pratt,A.A.,
            Maluenda,A., Colombet,J., Bisseux,M., Ravet,V., Dayaram,A.,
            Stainton,D., Kraberger,S., Zawar-Reza,P., Goldstien,S., Briskie,J.,
            White,R., Taylor,H., Gomez,C., Ainley,D.G., Harding,J.S.,
            Fontenele,R.S., Schreck,J., Ribeiro,S.G., Oswald,S.A., Arnold,J.M.,
            Varsani,A., Enault,F. and Stedman,K.M.
  TITLE     Direct Submission
  JOURNAL   Submitted (24-FEB-2020) Biology, Portland State University, 1719 SW
            10th Ave, Portland, Oregon 97201, USA
COMMENT     Bankit Comment: ALT EMAIL:ide@pdx.edu
            Bankit Comment: TOTAL # OF SEQS:451
            Bankit Comment: GAP: est. length - alignment

            ##Assembly-Data-START##
            Assembly Method       :: MetaSPAdes v. 3.12; IDBA-UD v. 1.1.1;
                                     MEGAHIT v. 1.0.3
            Sequencing Technology :: 454; Illumina
            ##Assembly-Data-END##
FEATURES             Location/Qualifiers
     source          1..2983
                     /organism="Crucivirus-120"
                     /mol_type="genomic DNA"
                     /isolate="120_252_SvL2_3298"
                     /isolation_source="Water"
                     /country="Norway"
                     /collection_date=2012
     CDS             1..1398
                     /codon_start=1
                     /product="Putative CruV Capsid Protein"
                     /translation="MEMIPANQAYNVPTVQDRAEGPYMPGGRFVATQAYKRKAYKRLS
                     GKRTYKRRKRSTYRKSYGRTTRGGNGGNIIIGRGDYTSFGGMLGAKAGGYLGDMAQNA
                     LMGLVTGLGDYTVQKNAFLNGRLPEMVNISDSGGIVVRYQEYLFDVVTSATIGAFSTQ
                     AVDINAANPLCFPFLAQLAANFEQYEIQGLIFEFRSTSADALNSTNTALGSVMLATQY
                     DVLDPLFTSKYEMLNYEFANSIKPAGNCLHMVECARSQTSISDLYTAWNGQVAVGSDP
                     RLYNLGRFVIACTGFQAASVNVGEVHITYQVRLLKPKLFGGLGNNISYAKFVIPTWSN
                     AKPIGTAAPSATVTNQPLITLDQTNREIIFPNTTGKLSYSVMIWWESGGANTAFTAPI
                     ITAFNCAVNGGGVIPAPLNGDTVYAAMLWFQLTTDGSGLAPYVLVGTGGALPGTSGGS
                     TQTQMWIVVEQKANI*"
     misc_feature    1090..1109
                     /note="stem-loop"
     misc_feature    1095..1103
                     /note="nonanucleotide"
     CDS             complement(1408..2463)
                     /codon_start=1
                     /product="Putative Replication Associated Protein"
                     /translation="MQFDRMASPPQNLKNKPIVFNPKARHWAGCTWNNYTPDDYEHLK
                     LGMMQHCIYGVIGKETGEHGTPHLQFMFSFAKPKNYIWIVSHLLRGNRLHCEVKHGPF
                     SVASNYCKKGSQPHDEWTNMHETGPHFGNDADFWEHGVLPQDQGTAGGNATAKVWDEI
                     KESAKAGALDDIPSDVYIKHYSTLNRIAEDHREDPLPLTWIRYPGYTETPNTWIWGPA
                     GYGKSHEAKRLAPNAYRMNPNLKFWQNYKDEDNVIVDDFDRCHVKQGKLFDLKIWGDK
                     DIFFADIKNKGHFIRPKIIIVTSNSSPKEMFGRCASEIRDYLEPIEQRFNIVHWDKPW
                     KEYEKATKLNLFENIEF*"
ORIGIN
        1 atggagatga taccagctaa tcaggcatat aacgtgccta cagtacaaga tagggcagag
       61 ggaccctata tgcctggtgg acgttttgta gcaacacagg cctacaaacg taaggcttat
      121 aaacgtttat caggtaaacg tacttataag agacgtaaac gatctacgta tcgtaagtct
      181 tatggtcgaa ccacaagagg tggtaatggt ggtaatataa tcatcggtag aggtgattat
      241 acatcatttg gagggatgtt aggagccaag gcaggaggtt accttggtga tatggcccag
      301 aatgcactta tgggcctagt taccggctta ggtgactata cagttcagaa gaacgccttt
      361 cttaatggaa gattaccaga gatggttaat atttccgatt cagggggtat tgtagtaaga
      421 tatcaagaat atctgtttga tgttgtaaca tcagctacaa ttggagcatt tagcacacag
      481 gctgttgata tcaatgctgc caatcccctt tgtttcccat ttttggcaca attagctgct
      541 aattttgaac aatatgaaat tcaaggtctt atctttgaat ttagaagtac atcagcagat
      601 gctttgaact cgacaaacac tgctcttgga tcagtcatgc ttgccacgca atatgatgtg
      661 ttagatccat tgtttacttc caaatatgag atgctcaatt atgaatttgc aaattcaatt
      721 aaacctgcag gtaattgttt gcatatggtt gaatgcgcta gaagtcaaac ctcaatatct
      781 gatctttaca ctgcttggaa tggccaagtt gctgtaggat cagaccctcg attatataat
      841 ctaggaagat ttgtaatcgc ttgtacagga tttcaagcag ccagtgttaa cgttggagaa
      901 gttcatatta catatcaagt tcgactcttg aagcctaagt tgtttggagg tcttggaaac
      961 aatatttcgt atgctaaatt tgttattcca acatggtcca atgccaagcc aattggaaca
     1021 gccgccccaa gtgcaactgt cactaatcaa cctttgatta cccttgatca aacaaataga
     1081 gaaataatat ttccaaatac tactggaaaa ttatcttact ctgttatgat ctggtgggag
     1141 tcaggaggag ctaacactgc atttactgca cctattatca cagcgtttaa ctgtgctgtt
     1201 aatggtggag gtgttattcc tgccccactt aatggtgata ctgtgtatgc agccatgtta
     1261 tggttccagc ttacaactga tggaagtgga cttgctccat atgtacttgt tggaacaggt
     1321 ggagcattac ccggaactag tggaggttct acacagacac aaatgtggat tgtagttgag
     1381 cagaaagcta acatatagta aagtttatta aaattcaata ttttcaaata aatttagttt
     1441 tgtagccttt tcgtattctt tccagggttt atcccaatga actatattga atctttgttc
     1501 aatgggttct aagtaatctc taatctcact tgcacatcta ccaaacattt cttttggtga
     1561 tgagttagat gttactatta taattttggg tcttataaag tgacctttat ttttaatatc
     1621 agcaaaaaat atatctttat ccccccaaat ttttaaatca aatagtttgc cttgcttgac
     1681 atggcaacgg tcaaaatcat cgacaattac attgtcttca tctttataat tttgccaaaa
     1741 ctttaggttt ggattcattc tataagcatt aggcgctagt cgctttgctt catgggattt
     1801 cccatatcct gcaggtcccc agatccatgt atttggggtt tctgtataac caggatagcg
     1861 aatccatgta agtggtagag ggtcctccct atgatcttca gcaatacgat taagtgttga
     1921 gtaatgttta atgtaaacat ctgaagggat gtcatctaat gcaccagctt tagcagactc
     1981 ctttatctca tcccacactt tagctgtggc atttccacca gcagtacctt gatcttgtgg
     2041 tagaactcca tgttcccaga agtcagcatc attaccaaag tgtggtcctg tctcatgcat
     2101 gtttgtccat tcatcatgag gttgactacc cttcttacaa tagttgctag caacagagaa
     2161 gggtccatgt ttaacttcac agtgaagcct gttacctctt aacaggtgag aaactatcca
     2221 tatatagttt ttaggtttgg caaaactaaa cataaactgg aggtgagggg tgccatgttc
     2281 acctgtctct ttgcctatga caccatagat gcagtgttgc atcatcccta gcttgaggtg
     2341 ttcatagtca tcaggagtgt agttgttcca agtacagcct gcccaatgtc tggcctttgg
     2401 gttgaagacg attggcttgt tttttaaatt ttgaggaggt gatgccatcc gatcaaattg
     2461 caaataaaaa cgtgttcacg tgtaaggaca cgggatttaa ttttttattg gataaataaa
     2521 aaaaaaggta ctggtgactc ccgtaaggac gacacctccc cgtaggacgg atctttaaga
     2581 ggacgtctac accagtacct ggaggttata taaccctata acccccttaa tgtaagctgg
     2641 cccgtgcgag aagcacggga cggggccccc ccttgggggg tggaggtacc tttaggtacc
     2701 gacttagcct ctagggaaac tttagttagt attaccccta gaggcgttga acctgatacc
     2761 gttgaacccg cacgtgttca cgtgtatcca atgcctcacg ttcttaggaa atgcagaaca
     2821 cgtgtcgcac gtttatttgc attttgttag agtcaccacg gctctacata gaaagcgaaa
     2881 agttgaggag gactattctc aaccgcatat tagtgctata cgtgctaata aggctaacaa
     2941 gaacgcccat atagattttg aagaaatcga tttatcagcg cca
//
LOCUS       CruV-121                2991 bp    DNA     circular     24-FEB-2020
DEFINITION  Crucivirus-121 isolate 120_1710_IR2_3297.
ACCESSION   CruV-121
KEYWORDS    .
SOURCE      Crucivirus-121
  ORGANISM  Crucivirus-121
            Unclassified.
REFERENCE   1  (bases 1 to 2991)
  AUTHORS   de la Higuera,I., Kasun,G.W., Torrance,E.L., Pratt,A.A.,
            Maluenda,A., Colombet,J., Bisseux,M., Ravet,V., Dayaram,A.,
            Stainton,D., Kraberger,S., Zawar-Reza,P., Goldstien,S., Briskie,J.,
            White,R., Taylor,H., Gomez,C., Ainley,D.G., Harding,J.S.,
            Fontenele,R.S., Schreck,J., Ribeiro,S.G., Oswald,S.A., Arnold,J.M.,
            Varsani,A., Enault,F. and Stedman,K.M.
  TITLE     Direct Submission
  JOURNAL   Submitted (24-FEB-2020) Biology, Portland State University, 1719 SW
            10th Ave, Portland, Oregon 97201, USA
COMMENT     Bankit Comment: ALT EMAIL:ide@pdx.edu
            Bankit Comment: TOTAL # OF SEQS:451
            Bankit Comment: GAP: est. length - alignment

            ##Assembly-Data-START##
            Assembly Method       :: MetaSPAdes v. 3.12; IDBA-UD v. 1.1.1;
                                     MEGAHIT v. 1.0.3
            Sequencing Technology :: 454; Illumina
            ##Assembly-Data-END##
FEATURES             Location/Qualifiers
     source          1..2991
                     /organism="Crucivirus-121"
                     /mol_type="genomic DNA"
                     /isolate="120_1710_IR2_3297"
                     /isolation_source="Water"
                     /country="Norway"
                     /collection_date=2011
     CDS             complement(1..1218)
                     /codon_start=1
                     /product="Putative Replication Associated Protein"
                     /translation="MDTADGTSTISDLGEYIDESKFKFQGQKLLLTWPLHLDKKCVQD
                     HIIEEIGFTPKFLRSAIETGDTGHEHTHTLIDFGKKVNYTKCDKFDMLVSVESEDGED
                     DTINVHPNWKPIKSKQHWDNAVKYLSKEDPENADLAEAKVNIIQAIWNEPNRAAAVLK
                     YAETPSQAQAVIATYAAKPTNRVASQDAPDAWHPWQQDVVDFIKTKPDDRTVHWFYDE
                     EGGKGKSRLARHLITEKLAYMVRSTGGDRDFATIMRGALADGWDQRCFIFDLPRVSEA
                     HAIYGPIEACKDGTVTATKYEGGTFTFNSPHVLIFANFLPDCSKLSADRWRIHRLLGD
                     KTMDKPLFTRKLATPQQGDLNIIRSPKEEAVEKKDNVLSNTLDIESAVAQLLKLYTDS
                     IKLKSLCTPEKDD*"
     misc_feature    139..166
                     /note="stem-loop"
     misc_feature    150..158
                     /note="nonanucleotide"
     CDS             complement(1391..2698)
                     /codon_start=1
                     /product="Putative CruV Capsid Protein"
                     /translation="MRKTMAMPAAKKIYNAAKSYVPKGSAERIGAKMGASTGFPYGEY
                     VGKQVGKQFAKITGVGDYYMDGGSKSIMNPKTKTPRVKNSDSDVVISKCEYICDISSG
                     PTGTPTDFDQRVFFLNPGLGEEEGGLFTFLPSLARNFSQYSFDQCVIEFRSTSGNATG
                     ANTSLGSVILCANYVPSDDPCANKLEALNSQFAVSTKTSSSAFFPIECKNKQQNLRFH
                     QIRDGALKPNQNIDLFDYTQVEVNTIGCPTANQLLGELWITYTVRLSKYKFTKQVPVS
                     SSLFAKFRYDGGAADWSTNPLGPSSGTALALVSTSNFTPTLNRVGKTITIPSDVVSKH
                     FWLYCKYRGTSTLEWNTFAIACSGNTSPLVSILSTTAGQTDVVSAGTEKADTEFTVSI
                     MIAVSAGGGGTLTFNEASCILDDVTNVEIGLIEMPEGAFVAGE*"
ORIGIN
        1 ctagtcatcc ttttctggag tacataacga tttcaatttg atggaatcgg tgtagagttt
       61 taataactgt gccaccgcag actcaatgtc gagtgtgttt gaaagcacgt tatccttttt
      121 ttctaccgct tcctctttgg gtgaccgaat aatgttaagg tcaccctgtt gcggtgtagc
      181 gagcttgcga gtgaacaatg gcttatccat tgttttatca cccaacagcc gatggattct
      241 ccatcgatct gcagacaatt tactacagtc agggaggaaa tttgcgaaga ttaggacgtg
      301 gggactatta aatgtgaagg ttccgccttc gtacttagtc gccgttacag tcccatcctt
      361 gcaggcctcg atagggccgt atatggcgtg ggcctcactc actctgggga ggtcgaagat
      421 gaagcaccgc tgatcccacc cgtccgcaag ggctcctctc atgatagtag cgaagtctct
      481 atcccctcca gtcgagcgca ccatgtacgc gagcttctca gtaatgaggt gtctggctag
      541 gcgtgatttt ccttttccgc cttcttcatc gtagaaccag tgtactgttc gatcatcagg
      601 tttcgtttta atgaaatcca caacatcttg ttgccacggg tgccatgcat ccggcgcatc
      661 ttgtgatgcg actctattag tcggcttagc tgcatatgta gcaatgacgg cttgagcttg
      721 cgacggagtc tcggcgtact tcaatacagc ggccgcgcgg tttggctcgt tccaaatagc
      781 ctgaataatg tttacctttg cttctgctaa atcggcattt tcagggtcct ccttcgagag
      841 gtattttacg gcattgtccc aatgttgttt agatttgatg ggtttccagt tggggtgtac
      901 gtttatcgta tcatcttctc cgtcttcaga ttctacggag accaacatgt caaatttgtc
      961 gcattttgtg tagttcacct tcttaccaaa atcgatgagg gtatgtgtat gttcatgacc
     1021 cgtatctccg gtctctatgg ccgacctgag aaatttaggc gtaaagccaa tttcttctat
     1081 aatatggtcc tgcacgcact tcttgtctag gtgcaaaggc cacgtcaata acagtttctg
     1141 tccttgaaac ttgaattttg attcatcaat atattctcct aaatcagaga ttgtacttgt
     1201 accatctgca gtatccattt gtaatatcga ctcgggagat aactattaca agggtataaa
     1261 gttccggcaa aaataaataa aatagggaat tgtgctactt tgtatcagca attaattatt
     1321 ttttgtcccc cggcaactat atgtgttatt ctagaatact agaacaaaaa tacaattgtc
     1381 atttttatga ttattctcca gcaacgaatg ctccttcagg catttcaatg agaccgattt
     1441 ctacatttgt cacgtcgtct aatatgcacg acgcctcatt aaacgtcaaa gttccaccac
     1501 cacctgcgct tactgcaatc attattgata cagtaaattc agtatctgcc ttttctgtac
     1561 ccgcagacac aacatcagtt tgaccagctg tagttgaaag aattgaaacg aggggtgaag
     1621 tattacctga gcaggcaatc gcgaacgtat tccattccaa tgtactagtt cctctatatt
     1681 tacaatatag ccagaagtgt tttgacacga cgtcagacgg tatagttatc gtctttccta
     1741 ccctatttag tgtaggcgtg aagttagatg tcgacacaag cgctaacgct gtacctgaag
     1801 acggtccgag aggattagtc gaccagtcag cagcacctcc atcatatcgg aactttgcaa
     1861 atagagatga ggacacggga acctgcttgg tgaatttata cttcgacaat ctgacagtgt
     1921 aggtaatcca caactcacct aagagttggt tagcagtagg acaaccgata gtgttgactt
     1981 caacctgcgt ataatcaaat aagtcaatat tttggttagg tttaagggct ccatcacgaa
     2041 tttgatgaaa tctcaaattc tgttgcttgt ttttacactc gataggaaag aatgccgagc
     2101 tagaagtctt cgtactcacg gcaaattgac tattaagcgc ctcgagctta tttgcgcatg
     2161 ggtcatctga tggcacatag ttagcgcaaa gaataaccga tcccaatgag gtgtttgcac
     2221 cggttgcgtt accagaagta gagcggaact cgattacgca ctggtcgaaa gagtattggg
     2281 agaagtttct agcaagactg ggaaggaatg tgaacaaacc tccctcttct tcgcctaacc
     2341 cggggttaag gaagaatact ctttggtcga aatctgtcgg ggtaccagta ggacccgatg
     2401 agatatcgca gatatattcg cacttcgaaa ttacgacatc ggagtcagag tttttaactc
     2461 ttggtgtttt cgttttgggg ttcataatgg acttggatcc tccatccatg tagtaatccc
     2521 ccactcctgt tatttttgca aactgcttac ccacctgctt tccgacatac tcaccataag
     2581 gaaagccagt agacgcgccc atttttgcgc cgattctttc tgcactacct ttaggtacgt
     2641 atgatttagc agcgttgtat atctttttag ctgctggcat tgccattgtt ttacgcatga
     2701 tgattatact tctttgtata acataagtaa aatattttat gtgaaaaaaa ttaataaatt
     2761 cttctccagt ctagttggtt ccactcccaa ataccagtcc aacgagacct gggatatcta
     2821 tatatatgag atatgacgct cggggagaag aattttaaat ttagtaatta attcgtaaac
     2881 aggaccgggg cttgtccccg gtcacgatta attatacatt tcaaattatt ataccctagg
     2941 attatcacat atttatatgg tattctaggt cgaggatctg gtatttcatt a
//
LOCUS       CruV-122                3003 bp    DNA     circular     24-FEB-2020
DEFINITION  Crucivirus-122 isolate GP2_73255.
ACCESSION   CruV-122
KEYWORDS    .
SOURCE      Crucivirus-122
  ORGANISM  Crucivirus-122
            Unclassified.
REFERENCE   1  (bases 1 to 3003)
  AUTHORS   de la Higuera,I., Kasun,G.W., Torrance,E.L., Pratt,A.A.,
            Maluenda,A., Colombet,J., Bisseux,M., Ravet,V., Dayaram,A.,
            Stainton,D., Kraberger,S., Zawar-Reza,P., Goldstien,S., Briskie,J.,
            White,R., Taylor,H., Gomez,C., Ainley,D.G., Harding,J.S.,
            Fontenele,R.S., Schreck,J., Ribeiro,S.G., Oswald,S.A., Arnold,J.M.,
            Varsani,A., Enault,F. and Stedman,K.M.
  TITLE     Direct Submission
  JOURNAL   Submitted (24-FEB-2020) Biology, Portland State University, 1719 SW
            10th Ave, Portland, Oregon 97201, USA
COMMENT     Bankit Comment: ALT EMAIL:ide@pdx.edu
            Bankit Comment: TOTAL # OF SEQS:451
            Bankit Comment: GAP: est. length - alignment

            ##Assembly-Data-START##
            Assembly Method       :: MetaSPAdes v. 3.12; IDBA-UD v. 1.1.1;
                                     MEGAHIT v. 1.0.3
            Sequencing Technology :: 454; Illumina
            ##Assembly-Data-END##
FEATURES             Location/Qualifiers
     source          1..3003
                     /organism="Crucivirus-122"
                     /mol_type="genomic DNA"
                     /isolate="GP2_73255"
                     /isolation_source="Sediments"
                     /country="New Zealand"
                     /collection_date=2014
     CDS             1..1335
                     /codon_start=1
                     /product="Putative CruV Capsid Protein"
                     /translation="MPGKKKSYTQAEKLAYYKRKAAMNYTGRGAYRMRKPAPKSRAVG
                     EAAGAALGGMVKYIPGGAPFAAPAAILASKLGGYLGNKIGSYMGFGEYTVKNNSLVIP
                     EGNSPGQMHSDGKRTRICHREYITDIISSGTTGQFRLQSFQMQPGNNLIFPWLSSVAL
                     SFQKYKVHGAIVEFKSGSGDAINGTNTALGEVIISSNYNCADSNFVNRMQMENTQYCS
                     SAKPSVSFVHIIECDPSLQAQETLYVSPSLVPQAGLGINDINFVNVQVATVGVQGTNV
                     NLGSLYITYDIELIQPLQDYIDKYPKSDWFAPTLATVSAANPCGTSNVPDNRNSLGGT
                     LSGATYIFPPLLQEGLFKFQWYAIGTAAVFTAPGVTLTNCVIRATMGGDVVGALAFPQ
                     NGANATNVCLIYVIEVTGQNASFVINSGGTIPTAVTGAALMVDQVDRDLFGV*"
     CDS             1401..2672
                     /codon_start=1
                     /product="Putative Replication Associated Protein"
                     /translation="MPLPRYVNWCFTYNNYTESGIESLKTFEHIKYCIFGKEIGESGT
                     PHLQGYFELYNKKSIAGLKESLIAHSEELNFHFESRKSNQEKAIAYCKKGEQSHEDWK
                     KYGITGKTYGLNADITEWGVKARPGTRNDLDAARQCVEDNGMREVSARFGLQAIQVAR
                     HYLTYCEPVRELEFNPLVIYIYGEPGSGKSQLARDLCKDLDTYWWRPALFEKWWDGYD
                     QHSCVVLDDIRLSHVSEALMLALLDKYDCAVQVKGSTRQMLAHMFIITTLLPPEKFWN
                     SHEVVTQLLRRISITVKVSPDHSYHVEKVNENLESTLTGFKIENKFLKKLIHGSRSGG
                     NTVPPTSSPVDVENIEKCLEIEDIFDNDEFNEGDDENCKEWDDIESDDESKRNYIFDE
                     LSESDLPELIGLNENQFLENIKDNEIYDNIV*"
     misc_feature    2391..2421
                     /note="stem-loop"
     misc_feature    complement(2401..2409)
                     /note="nonanucleotide"
     misc_feature    2899..2919
                     /note="stem-loop"
ORIGIN
        1 atgcctggca agaaaaagtc ctatacccaa gctgaaaaat tggcttacta caagcgtaaa
       61 gctgccatga attatactgg tcgaggtgct tatagaatgc gtaaacctgc tcctaaatct
      121 cgtgcagttg gtgaagctgc tggcgccgct cttggtggta tggtcaaata tattcctggt
      181 ggtgcgccat ttgccgctcc tgccgctatt ctagctagta aacttggtgg atatttggga
      241 aataagatcg gaagctatat gggattcgga gaatacactg ttaaaaataa tagtttagtt
      301 ataccggagg gcaattctcc gggtcagatg catagtgatg gtaaacgtac tcgtatttgt
      361 catagggaat atattactga tattataagt tctggtacta caggtcaatt tagacttcaa
      421 agcttccaaa tgcagcccgg aaataatctt atattccctt ggctttcatc tgttgctttg
      481 agttttcaaa agtataaagt acatggtgct atcgttgaat ttaagtcagg ttccggtgat
      541 gctatcaatg gtactaacac tgcgttgggt gaagttatca taagttccaa ttacaactgt
      601 gctgactcta atttcgtgaa tcgcatgcag atggaaaaca ctcaatattg ttcaagcgct
      661 aaaccttcgg ttagcttcgt acacattatt gaatgtgatc catctttgca agctcaagaa
      721 actctgtacg tttctccttc attagttcct caagctggac ttggaattaa tgatattaac
      781 tttgttaacg tgcaggttgc tactgttggt gtgcaaggaa ctaacgtaaa tttgggtagt
      841 ttatatatta cttatgatat tgaattaatc caacctctac aggattatat tgataaatac
      901 cctaagagcg attggtttgc gcctactctt gctacggtta gcgctgctaa tccttgtgga
      961 acatctaacg tgcccgataa tcgtaactca cttggtggta ctctttctgg tgctacttat
     1021 atatttccac cacttctaca agaaggtttg tttaaatttc agtggtatgc aattggtacg
     1081 gctgctgtct tcacagcgcc tggtgttacg ctcactaact gcgttattcg agctactatg
     1141 ggtggtgatg ttgtcggtgc acttgcgttt cctcaaaatg gtgccaatgc taccaatgta
     1201 tgtttaatat atgtaattga agtaactgga caaaatgcta gttttgttat taactcgggt
     1261 ggtactatcc ctactgctgt tacgggtgct gctcttatgg ttgatcaagt tgatcgcgac
     1321 ttattcggtg tataaatgca taattaagag ataattaatt attttttttt ttgtattctg
     1381 taccatatac aaaaaaaaca atgccgcttc ctcgctacgt taattggtgt ttcacgtaca
     1441 ataactacac tgagtcaggc attgaaagtt taaaaacttt tgaacatatt aaatattgta
     1501 tctttggaaa agaaattggt gaaagtggca cgccacattt acaaggttat tttgaattat
     1561 ataataaaaa aagtattgcg ggcttaaaag aatctttaat tgctcattct gaggaattaa
     1621 attttcattt tgaatcacgc aaaagtaatc aggaaaaagc tattgcatac tgtaaaaaag
     1681 gtgaacaatc tcatgaagat tggaaaaagt atggtattac aggtaaaacc tatggtttaa
     1741 acgccgacat tactgagtgg ggcgttaaag ctcgtcccgg tacccgtaac gatcttgacg
     1801 ctgcccgtca gtgtgtcgaa gataatggta tgcgtgaagt ttcagcacgc ttcggacttc
     1861 aagctattca agttgcccgg cactatctca catactgcga gcctgtgcgt gaacttgagt
     1921 ttaatccctt agtaatttac atttacggcg aacctgggtc tggcaaaagt caattagcta
     1981 gggatttgtg taaagacctt gatacctatt ggtggagacc agcgctgttc gagaaatggt
     2041 gggacggata tgatcagcat tcctgcgtcg tgctcgatga tatccgcctt tcccatgtca
     2101 gcgaagcgct tatgcttgca ttattagata aatatgattg tgcggtacag gtcaagggca
     2161 gtacgcggca aatgctagca catatgttta tcattacaac tttattacca cctgaaaagt
     2221 tctggaatag tcacgaagtt gttacacaac tgctcagacg tatttccatc actgttaagg
     2281 tgtctcctga tcattcttat catgtcgaaa aagtgaacga aaacctagaa tctactttaa
     2341 cggggtttaa gatagagaat aaatttttaa aaaaattaat ccacggctca aggtcggggg
     2401 gtaatactgt acccccgacc tcttcacctg tggatgttga aaatattgaa aaatgtttag
     2461 aaattgaaga tatttttgat aatgatgaat ttaatgaagg tgatgatgaa aattgtaaag
     2521 aatgggatga tattgaaagt gatgatgaaa gtaaaagaaa ttatatattt gatgaattaa
     2581 gtgaaagtga tctacctgaa ttaataggat taaatgaaaa tcaatttctt gaaaacatta
     2641 aagataatga aatatatgat aatattgttt gaaatagtgg gaataaaaca gaatggatag
     2701 gcacgccctg ttcattgcgg tgcgtctcat tgtctcaaaa ccaagtccca ccccttcggg
     2761 gcgtgccgtg gtttcgagcc aacagagtcg taaagtacgc aactccatag gtgcctactg
     2821 tctgatatag ttgatatagt taatattttt ttgatgcggc taaaaatccc tcgcattcgc
     2881 tcgatcaagt cgagaactgt attttttttt tttaaatact tttgttgcac tgcacggcta
     2941 gaggttggaa ttaattattt ttttttttat tttatgtttt cgctaggata tattacttaa
     3001 atc
//
LOCUS       CruV-123                3009 bp    DNA     circular     24-FEB-2020
DEFINITION  Crucivirus-123 isolate 120_1711_IR2_3297.
ACCESSION   CruV-123
KEYWORDS    .
SOURCE      Crucivirus-123
  ORGANISM  Crucivirus-123
            Unclassified.
REFERENCE   1  (bases 1 to 3009)
  AUTHORS   de la Higuera,I., Kasun,G.W., Torrance,E.L., Pratt,A.A.,
            Maluenda,A., Colombet,J., Bisseux,M., Ravet,V., Dayaram,A.,
            Stainton,D., Kraberger,S., Zawar-Reza,P., Goldstien,S., Briskie,J.,
            White,R., Taylor,H., Gomez,C., Ainley,D.G., Harding,J.S.,
            Fontenele,R.S., Schreck,J., Ribeiro,S.G., Oswald,S.A., Arnold,J.M.,
            Varsani,A., Enault,F. and Stedman,K.M.
  TITLE     Direct Submission
  JOURNAL   Submitted (24-FEB-2020) Biology, Portland State University, 1719 SW
            10th Ave, Portland, Oregon 97201, USA
COMMENT     Bankit Comment: ALT EMAIL:ide@pdx.edu
            Bankit Comment: TOTAL # OF SEQS:451
            Bankit Comment: GAP: est. length - alignment

            ##Assembly-Data-START##
            Assembly Method       :: MetaSPAdes v. 3.12; IDBA-UD v. 1.1.1;
                                     MEGAHIT v. 1.0.3
            Sequencing Technology :: 454; Illumina
            ##Assembly-Data-END##
FEATURES             Location/Qualifiers
     source          1..3009
                     /organism="Crucivirus-123"
                     /mol_type="genomic DNA"
                     /isolate="120_1711_IR2_3297"
                     /isolation_source="Water"
                     /country="Norway"
                     /collection_date=2011
     CDS             1..1080
                     /codon_start=1
                     /product="Putative Replication Associated Protein"
                     /translation="MTQRSRAWCITKHLNWTIPKNCENPEEFHEGTFIHKSVLRGIGH
                     SEDGPDIDLLEISLNQLFLKFDFKGLARPYAPYSYISIGLEVGEDTQRPHIHIYVEYK
                     NARSFNTLKKIDETTHIEKRMGTAKQADDYVKKDGRSWDDGEMTLPQGKRTDLDACRE
                     LIVEDKCNMRDACMTVRSYQSIKTCQILLTYLEEERDWKPTVIWIWGKAGLGKSRLAH
                     DYARLQENKMHTVATSPDFKWWDGYDKHEIVLIEDLREEDCRFNVLLKILDRYPYRVE
                     YKGGTRQLLAKTMFVTSDESPRCCYPQQDEVKIKQLTRRIDHVIELQSEEEYQLLIDY
                     NKGKKDWEWDLGNILLNSFLETEPN*"
     CDS             complement(1544..2923)
                     /codon_start=1
                     /product="Putative CruV Capsid Protein"
                     /translation="MSLTMKPGMARQYTDAEKAAYYKSKAQMVARPTRKSKPYAPKKR
                     KYVKKSNKSNSYSSMGRNLGALAGNALYPGVGGIIGGALGGAAQGIISKVSGFGDYHV
                     SKNSFIHNQDAVPEFSSDNERCTVITHREFISDVLSSTNFAVDEYRLNPGVFETFPWL
                     NSIADNYEQYVVQGMLFEYKTTSATAIGSTNTALGTVVMATQYNSLSQTFTNKQQMEN
                     YEFSQSTVPSQSILHPIECDPFQTQCGGVFNVADPNATNGDLRLYDLGKFNIATVGMQ
                     TAGIPIGELWVTYKICLLKPRLRSVVDVWDEFSLQLTTVANNVPLGDVSQAARFSQGS
                     SFTTLININTVEIDPGFSGVIQVIYYVRAQGFTSLIAPQIVPGATSPQVTDITGTFLN
                     SPDVRTVCDPLASPNDCVSIAYLRCGGGFTSSGVGQRFTLPSFGVTGGYAIDIARIQI
                     ISMPASMNF*"
ORIGIN
        1 atgacacaaa ggtctagagc atggtgcatc acaaaacact taaactggac aattcctaag
       61 aattgcgaaa atcctgaaga atttcatgaa ggtaccttta ttcacaaatc ggtacttaga
      121 gggattggac acagcgaaga tggtcctgat atagatttgc tagaaatcag cttaaatcag
      181 ttattcctaa agtttgactt taaaggattg gctaggccat atgcgcctta ctcgtatatc
      241 tcgattggct tagaagttgg tgaggatact caaagacctc atattcatat ctacgtagaa
      301 tacaaaaacg ccagatcttt taatacttta aagaagatag atgagactac tcatattgaa
      361 aaaagaatgg gtacggctaa acaagccgat gattatgtta agaaggatgg cagatcatgg
      421 gatgatggtg aaatgacatt accgcaaggc aaacgtacag accttgatgc gtgtagggaa
      481 ctaatcgttg aagataaatg caatatgcga gacgcatgca tgacggtaag atcatatcaa
      541 tcaatcaaga catgccaaat cctcttaaca tatttagaag aagaacggga ttggaaacca
      601 accgtcatct ggatttgggg aaaagcagga cttggaaagt caagactcgc tcatgattac
      661 gctcgactac aagaaaataa aatgcataca gtagcaacgt caccagattt caaatggtgg
      721 gacggttatg acaagcacga aatcgtacta attgaagatc tcagagaaga agattgcaga
      781 ttcaatgtgc tgctaaaaat actagacaga tatccataca gagtggaata taaaggaggt
      841 acaagacaac tcctagcaaa aacaatgttc gtaacgagcg atgaatcgcc acgatgctgc
      901 tatccgcaac aagacgaagt aaaaatcaaa cagctaacgc gccgtattga tcacgtaata
      961 gaattacaat cagaagaaga atatcaactt ttaattgact ataacaaagg caaaaaagat
     1021 tgggaatggg atttgggcaa catattgcta aattcgttcc tcgagaccga accaaattag
     1081 ctttccttgg tgctcactaa agttcacaga cgttaaaaag ctgtgatggc tcgttctctc
     1141 gttcgctcgg caagctcgct cactacgttc tcttcatcgt ctctcgctct ctccgttcgg
     1201 ctcgttcctc gtccatccaa tggcagacta cagcgtcgaa agacttctcc ttgcacactt
     1261 gcacaaaggt cggggggtaa tactaggcca aggccacccc ctcctaccct cgttttttga
     1321 actcgagaca ttttttgtta cttcagcctt attcaaggca attcagaaat atagtcgctc
     1381 cgctccccca gtgttcattt tcttatacaa aaaatagatg attaacagta cctccataac
     1441 caatagtcgg aacaaagttg ttagattaat tcatgtagaa acatacttta agaagtgaat
     1501 aactaattgt gttgcctagt cacagataga ggcataaatg ggtttagaag ttcatagaag
     1561 caggcataga aataatttga attctagcaa tatcaatagc ataaccacct gtaacaccaa
     1621 aactgggaag agtaaatcgt tgaccaacac cactagaagt aaaacctcca ccacaacgta
     1681 aataagcaat agaaacacag tcattaggac tagcaagtgg atcacaaaca gtacgaacat
     1741 caggagaatt caagaaagta ccagtaatat cagtaacttg aggagaagta gcaccaggaa
     1801 caatttgagg tgcaattaaa ctagtaaaac cttgagcacg aacgtaataa attacttgaa
     1861 taacaccact gaaaccagga tcaatctcga cagtatttat attaataaga gtagtaaaag
     1921 aagaaccttg actgaatcga gcagcttgag aaacatcacc aagaggaaca ttattagcaa
     1981 cagtagtcaa ttgaagtgaa aattcatccc aaacatcaac aacagaacgg agacgaggct
     2041 ttaataaaca aatcttataa gttacccaaa gttcaccaat aggaatacct gcagtctgca
     2101 taccaacagt agcaatatta aatttaccta aatcataaag tcgaagatct ccattagtag
     2161 catttgggtc agcaacatta aagactccac cacactgggt ttggaaagga tcgcattcga
     2221 taggatgtaa aatagattga gatggtacag tagattgaga aaattcataa ttttccatct
     2281 gttgtttgtt agtaaaagtt tgagataaac tattgtattg agtagccatg actactgttc
     2341 ctaaagctgt attcgtactt ccgatagcag tggcagaagt ggttttatac tcgaataaca
     2401 taccttggac tacatattgt tcataattat cagcaataga attcaaccat ggaaaggtct
     2461 caaaaacacc aggattaaga cgatattcat cgacagcaaa attagttgaa gacaatacat
     2521 cagaaataaa ctctctatga gttataacag tacatctttc gttatctgaa gagaactcag
     2581 gaacagcatc ttgattatga ataaaagaat tcttagaaac atgataatca ccaaaaccgg
     2641 ataccttact aataatacct tgagcggcac cacctagggc acctccaatg atacctccaa
     2701 cacctggata aagggcatta cctgccaaag cgccgagatt acggcccata ctagagtaac
     2761 tattagattt attggacttt ttgacatact tccgcttctt aggagcgtaa ggcttagact
     2821 tgcgagtagg tcgggccacc atttgggcct tagatttgta gtaagctgcc ttttcggcat
     2881 cagtatactg tctcgccata cctggtttca tagtaagaga caaaatttaa aattaattat
     2941 aagtcaaaaa atgaaaagtc caaaaagttg aaattaattt gtataaaaaa gtttggtcgg
     3001 aacctaaaa
//
LOCUS       CruV-124                3012 bp    DNA     circular     24-FEB-2020
DEFINITION  Crucivirus-124 isolate BS_313.
ACCESSION   CruV-124
KEYWORDS    .
SOURCE      Crucivirus-124
  ORGANISM  Crucivirus-124
            Unclassified.
REFERENCE   1  (bases 1 to 3012)
  AUTHORS   de la Higuera,I., Kasun,G.W., Torrance,E.L., Pratt,A.A.,
            Maluenda,A., Colombet,J., Bisseux,M., Ravet,V., Dayaram,A.,
            Stainton,D., Kraberger,S., Zawar-Reza,P., Goldstien,S., Briskie,J.,
            White,R., Taylor,H., Gomez,C., Ainley,D.G., Harding,J.S.,
            Fontenele,R.S., Schreck,J., Ribeiro,S.G., Oswald,S.A., Arnold,J.M.,
            Varsani,A., Enault,F. and Stedman,K.M.
  TITLE     Direct Submission
  JOURNAL   Submitted (24-FEB-2020) Biology, Portland State University, 1719 SW
            10th Ave, Portland, Oregon 97201, USA
COMMENT     Bankit Comment: ALT EMAIL:ide@pdx.edu
            Bankit Comment: TOTAL # OF SEQS:451
            Bankit Comment: GAP: est. length - alignment

            ##Assembly-Data-START##
            Assembly Method       :: MetaSPAdes v. 3.12; IDBA-UD v. 1.1.1;
                                     MEGAHIT v. 1.0.3
            Sequencing Technology :: 454; Illumina
            ##Assembly-Data-END##
FEATURES             Location/Qualifiers
     source          1..3012
                     /organism="Crucivirus-124"
                     /mol_type="genomic DNA"
                     /isolate="BS_313"
                     /isolation_source="Water"
                     /country="New Zealand"
                     /collection_date=2012
     CDS             1..1221
                     /codon_start=1
                     /product="Putative Replication Associated Protein"
                     /translation="MTETKTETNVEQKGFRFQNTAALLTYSTHINKKEYIEWFKTLSK
                     QTPTFIRLAHETGSNENKEEKYEHTHVVFKVSKRIDTINQRFFDYNNIHPHIKILKGN
                     KAIDDAKQYIAKEDPENKDLLIEPSIVHGIWAETSIQDALSKFVQKPSDAMGIVSIYN
                     YKKNEVFIDEEDEPNFTWHKEIMEEFKERARYARKRHVTWIYETVGLCQKTDLTRYLM
                     LTQPNAWFSCKDLGNAKDAATIIANAIAGGWTGHGCIIDLPRSASNHKRIYNYIEEIK
                     DGMVTSQKYSGRTFVFNKPHVLVLANWAPKLFMDGKPTLSFDKWSIRQLTKKTIDGKL
                     DVFQEKIDIFTDKRFLNNTRSKKLIEDFNNESTYNTNIYGTDDTDITDFVDELSESDN
                     KSNDNIDYLLGIKQ*"
     CDS             complement(1461..2831)
                     /codon_start=1
                     /product="Putative CruV Capsid Protein"
                     /translation="MPRQYSDKEKAAYYKKKATMVANPQRRYKSKYPKKRYVKKRYDY
                     PGAGYSIGSSLGGNVGGPMGSIIGGAVGQGAQSLVKRITGFGDYKVSKNALVYNVDAI
                     PEFSNDNERCTMICHREFISDIISSVSFNANTFRINPSIVTTFPWLASVARNWEQYVV
                     QGMVFEFKTTSATAIGSTNTGLGTVIMATQYNSLAPEFSSKQQMENYEFSQSTIPSAS
                     ILHPIECDPTQTQCGGIFNTYDPDYDVGDRRLYDIGRFTLATVGMQEANSVIGELWVT
                     YKICLLKPRLTSISDVYDFFTLDTGQMSPTYPIGDPVAAIPDSSNSGIFKVKGTIAAG
                     QYLEIDAEFIGIMQVVVAYVLDGGGSNSFAPPYANNFSSSIIDVTGNFIGVPSVAQGQ
                     NVRESGGPTFSGKCYTATFFLRCLGGRNPDGTNPRFQLISMAAASITFQYATIQATSI
                     NVSILP*"
ORIGIN
        1 atgacagaaa caaaaacaga aacaaacgta gaacaaaaag gattccgttt tcaaaacaca
       61 gcagctttac taacatatag cacacacata aacaaaaaag aatatataga atggttcaaa
      121 acattatcaa aacaaacacc cactttcata agactagcac atgaaacagg atcaaatgaa
      181 aacaaagaag aaaaatatga acatacacac gtagttttca aagtatcaaa aagaatagat
      241 actatcaacc aaagattctt cgattataat aacatacacc cacacataaa aatactaaaa
      301 ggtaacaaag caatagatga tgcaaaacaa tatatagcaa aagaagaccc agaaaataaa
      361 gacttactta tagaacctag tatagtacac ggtatatggg cagaaacatc tatacaagac
      421 gcactatcta aatttgttca aaaaccttcg gatgctatgg gcatagtaag tatttataac
      481 tataagaaaa atgaagtatt catagatgaa gaagatgaac caaatttcac atggcataaa
      541 gaaataatgg aagaatttaa agaaagagca aggtacgcaa ggaaaagaca tgtaacatgg
      601 atatatgaaa cagttggtct gtgccaaaaa actgatctaa ctagatatct aatgctaaca
      661 caaccaaatg cttggttctc atgtaaagat ttaggtaatg caaaagacgc agcaacaata
      721 atagcaaacg cgatagcagg tggatggaca ggacacggat gcataataga ccttcctaga
      781 tctgcatcaa atcacaaaag aatatacaac tatatagaag aaataaaaga cggaatggta
      841 acatcacaaa aatactcagg cagaactttc gtattcaata aaccacatgt attagttcta
      901 gctaactggg cacctaaact atttatggat ggaaaaccaa ctttatcatt tgataaatgg
      961 tcaataaggc aacttaccaa aaaaacaata gatggtaaat tagatgtttt tcaagaaaaa
     1021 atagacatat tcacagacaa acgattccta aacaatacta gatccaaaaa acttatagaa
     1081 gattttaaca acgaatccac atataataca aacatttatg gcactgatga tacagacata
     1141 acagatttcg tagacgaatt atcagaatcg gacaacaaat caaatgataa catcgattac
     1201 cttttaggta taaaacaata gttgattaaa aaatttctgg aattttttaa taaacaatgg
     1261 ctcccaccac ttcgttcctc ccattgacgc cgtgccgggc cgtcttctag ccgactaaca
     1321 ttacaatttt gtcggctagt ctgttttatg tatgcagatt tatgtattca tacgaattaa
     1381 tcgctcaggc cgctcggcca cgggaaacat ctttcttatt taatatatca aattagatgt
     1441 atttaaataa gaattcagat ttatggcaga atagatacat taatacttgt agcttgaata
     1501 gttgcatatt ggaaagtaat tgatgcagca gccatactga tcaactggaa tctcggatta
     1561 gttccatcag gatttctccc tccaagacat ctcaagaaaa atgtagcagt ataacattta
     1621 cctgaaaaag taggtccacc agattctctc acattctgtc cctgtgctac tgacggaaca
     1681 cctataaagt ttccagtaac atcaattatt gatgatgaaa aattattggc ataaggcgga
     1741 gcaaacgaat ttgaacctcc accatcaaga acatacgcaa caacaacttg cattatacca
     1801 ataaactctg catctatttc taaatattga ccagctgcta tcgttccttt gaccttaaat
     1861 atacccgaat ttgaagaatc aggtatagct gccacaggat caccaattgg atatgttgga
     1921 ctcatttgtc cagtatctaa tgtgaaaaaa tcatatacat cagatatact ggtcaatcta
     1981 ggtttcagta aacatatctt atatgttacc cataattctc caataactga attagcttct
     2041 tgcataccta ccgtcgcaag tgtaaatctt cctatatcat acaagcgacg atcaccaaca
     2101 tcataatcag gatcatatgt attaaatata cccccacact gcgtttgtgt cgggtcacat
     2161 tctatgggat gtaatatgct tgctgatgga atagttgatt gactaaattc ataattttcc
     2221 atctgttgct tacttgaaaa ttcaggcgcc aatgaattat actgtgtagc cataatcaca
     2281 gttcctaaac ctgtatttgt tgacccaatt gctgtagccg atgtagtttt gaactcaaaa
     2341 accatacctt gaacaacata ttgctcccaa ttacgcgcaa ctgaggctaa ccacggaaat
     2401 gtggtgacta ttgaaggatt tatgcgaaac gtatttgcat taaaactaac tgaagaaata
     2461 atatcagata taaactctct gtgacaaatc attgtacacc tttcattatc attcgaaaat
     2521 tcaggtatag catcaacatt atataccaat gcattcttac ttactttata atctccaaaa
     2581 cctgtaatac gtttaaccaa agattgcgca ccttgtccta ctgcaccacc tataatagaa
     2641 cccattggac ctcccacatt tcctcctaaa ctagaaccaa tagaataacc agcaccagga
     2701 taatcatatc tcttctttac gtatctcttt tttgggtact tggatttata ccttctctgt
     2761 ggattagcta ccatagttgc ttttttctta taataagcag ctttctcttt gtctgaatat
     2821 tgtcttggca tttttttata ttctattata agaatatttt tcttcaattc actataatat
     2881 atactaactc acttactcac tcacttactc attgtatttt tacatataat ccctattcac
     2941 ttactcactc acttactcac tcatattatt attccctata aaaaaataaa atatttataa
     3001 tatatagatt ta
//
LOCUS       CruV-125                3012 bp    DNA     circular     24-FEB-2020
DEFINITION  Crucivirus-125 isolate 001835_virusbaton.
ACCESSION   CruV-125
KEYWORDS    .
SOURCE      Crucivirus-125
  ORGANISM  Crucivirus-125
            Unclassified.
REFERENCE   1  (bases 1 to 3012)
  AUTHORS   de la Higuera,I., Kasun,G.W., Torrance,E.L., Pratt,A.A.,
            Maluenda,A., Colombet,J., Bisseux,M., Ravet,V., Dayaram,A.,
            Stainton,D., Kraberger,S., Zawar-Reza,P., Goldstien,S., Briskie,J.,
            White,R., Taylor,H., Gomez,C., Ainley,D.G., Harding,J.S.,
            Fontenele,R.S., Schreck,J., Ribeiro,S.G., Oswald,S.A., Arnold,J.M.,
            Varsani,A., Enault,F. and Stedman,K.M.
  TITLE     Direct Submission
  JOURNAL   Submitted (24-FEB-2020) Biology, Portland State University, 1719 SW
            10th Ave, Portland, Oregon 97201, USA
COMMENT     Bankit Comment: ALT EMAIL:ide@pdx.edu
            Bankit Comment: TOTAL # OF SEQS:451
            Bankit Comment: GAP: est. length - alignment

            ##Assembly-Data-START##
            Assembly Method       :: MetaSPAdes v. 3.12; IDBA-UD v. 1.1.1;
                                     MEGAHIT v. 1.0.3
            Sequencing Technology :: 454; Illumina
            ##Assembly-Data-END##
FEATURES             Location/Qualifiers
     source          1..3012
                     /organism="Crucivirus-125"
                     /mol_type="genomic DNA"
                     /isolate="001835_virusbaton"
                     /isolation_source="Water"
                     /country="France"
                     /collection_date=2013
     CDS             1..951
                     /codon_start=1
                     /product="Putative Replication Associated Protein"
                     /translation="MELRSTRLVIMPSARGWCFTQNFVTNVPKFVDESMKYLVYQKEG
                     SPSTGKPHWQGYVEYKITVGLKTLQKDFDGAHVEVRKGPRHAAREYCMKDESRLEPAK
                     EFGIWSDGQGDRTDIKAAAALAKAGKWKDIDASTYIKFHRGLREYARVHSQVPLRRDV
                     KVYVWWGPTGCGKSHDAFAGAENDPKACAIDWHGGWADPYDDQKTVVFDEFDGDTLPV
                     NIFLKLLDKYRTTVNCKGVTAKPWLVEKIFISSRKHPGDWYPGRRSEVLRRITTITHK
                     PDPYVAPLGGDKKTVKTPSPPDIRPDNVAMPDDSWLDEFV*"
     CDS             complement(1133..2842)
                     /codon_start=1
                     /product="Putative CruV Capsid Protein"
                     /translation="MLPKAQYMKLPKSAKAAVKRSLGMSKRKGPRAAVINLAGHGDYF
                     SYDPKGGMIRTPDWRKSVGAQIGGKFGPLGEVIGGGAHALFKKVTGWGDYKVGSNSIV
                     TGNTPALFKSDDRITIIRHREFVKDIVSSATPGAFKVESFRINAALPDTMPWLSQIAN
                     NFEQYCVSGMIFEYKSTSSDALNSTNTALGSVILATEYNTVNPNFISKSQMENHEFAS
                     AGKPSTELIRHPIECARGETPVSCLYTRNGAVPTGADPRLYDLGNFQIASSGMQAASV
                     TIGELWITYEVGLLKPRLQDALSYDVLCDHYRLPTTASVSKIFGVVLPTAETGSNLGT
                     TLTNVAEGECHFPVVPYDRYYLVWYQVVGASTALTDHFAWGGAGTTGFDLFTVGTEGA
                     VYLPSGITSTKQHIVQLFKVAANVAAYINYDQGTLPGTVTSGDLYITELSAAVLTKSP
                     YARNWRSLSAESEARRTKMISAKETSDRKLTSRQHDLVRKYEEETKALQEKLKSSLEA
                     DEVSEDEDDYLEYLKGRKAARDAAKASHIAEVSRPTVKPEEKAVAPPPVEAPKPLKIA
                     SKK*"
ORIGIN
        1 ctggagcttc gctcgacacg ccttgttatt atgccttcgg ctagaggttg gtgcttcacc
       61 caaaactttg tgactaatgt cccgaagttt gtggatgaat ctatgaaata ccttgtatac
      121 caaaaagaag gttcaccatc tactggtaaa cctcattggc aaggttatgt agaatataaa
      181 attactgtag gcctaaagac cctacagaaa gattttgatg gcgcacatgt tgaggtccgc
      241 aaggggcctc gccatgctgc tagagaatat tgtatgaaag atgaaagcag gttagaacct
      301 gctaaagaat ttggcatctg gtcagatggc caaggagata gaactgatat caaagctgct
      361 gcagctttgg ctaaagcagg taaatggaaa gacatagatg cttccacata tataaaattt
      421 cataggggat tgagagagta cgctcgcgta cactctcaag tacccctaag aagagatgta
      481 aaagtatatg tttggtgggg acctaccggt tgcggtaagt cccatgatgc atttgcagga
      541 gctgaaaatg atcccaaagc atgtgcaatt gattggcatg ggggttgggc tgatccttat
      601 gacgatcaga aaaccgttgt ctttgatgaa tttgatggtg atactttgcc cgtcaatata
      661 tttcttaagc ttcttgataa atataggact accgtaaact gcaaaggggt tacggctaag
      721 ccgtggcttg ttgagaaaat atttatctct tccaggaaac atccggggga ttggtatcct
      781 ggtcgtaggt ctgaagtatt gagaagaatt acgaccatca cccacaagcc ggacccctac
      841 gtggccccgc ttgggggtga taagaaaact gttaagactc cttcaccccc tgacattcgt
      901 cccgacaatg tcgcaatgcc agacgatagc tggttagatg aatttgtgta aaaaagattt
      961 gataaatcaa attatattgg ttcacaccga tacgccggaa acgttatgtg ctatgcgggg
     1021 cttcgccccg cccatggaca taagattccg gtttatcgaa ccggcgcgtg catatatttt
     1081 tcacatttat ctttggctaa tggccttgct ccatagttgg gactcatgac aatcacttct
     1141 ttgaagcgat tttcaggggc ttcggagcct cgacaggcgg tggtgccacc gctttttctt
     1201 ccggtttcac cgttggccgt gatacttctg caatgtgcga agcctttgcc gcgtcgcgcg
     1261 cagcctttcg ccccttcaga tactcgagat aatcatcctc atcttcgctg acctcgtcag
     1321 cttcgaggga ggactttaat ttttcttgca gagcttttgt ttcctcctcg tactttcgca
     1381 cgaggtcatg ttgacgtgat gtcagctttc gatccgatgt ctctttagct gatatcatct
     1441 ttgtccgtcg agcttcgctt tcggcggaca aagatcgcca attgcgagca tatggcgact
     1501 ttgtcaacac agcggcggat aattctgtaa tatacagatc gccggaagta acggtacctg
     1561 gtaatgtacc ttgatcataa ttgatatagg ctgcgacatt tgccgcaacc ttaaataatt
     1621 gtacgatatg ttgttttgtt gaggtgattc cggagggtag atatacggca ccttcagtgc
     1681 caactgtaaa cagatcaaac cctgttgttc ctgcaccccc ccatgcaaag tgatctgtaa
     1741 gagctgtaga tgcgccgacg acctgatacc acacaaggta gtatcgatcg tatggtacga
     1801 cggggaaatg acattctccc tcagcaacat ttgtcaatgt tgtgccgagg ttcgaccccg
     1861 tctcggcggt tggcaacacc actccgaaga ttttactaac ggatgcagtt gttggtagac
     1921 gatagtgatc acacaatacg tcataagata acgcgtcttg cagacgtggt tttagcagac
     1981 caacctcata tgtgatccat agctcaccta ttgtaacgct tgctgcttgc attccggagg
     2041 atgctatctg aaagtttccg agatcataca ggcgcggatc cgcgccggtt ggcacggcac
     2101 cattacgagt gtagaggcaa gacactggtg tctcgcctct tgcacactcg atagggtgcc
     2161 ttatgagctc ggttgatggc ttaccagccg atgcgaactc atggttctcc atctgcgact
     2221 tactaataaa gtttggattt accgtattgt attctgttgc cagaattacg gaacccaagg
     2281 ctgtattagt gctattcaaa gcatcgcttg acgttgattt gtattcgaat atcattcctg
     2341 atacacaata ctgctcaaag ttattggcta tctgcgataa ccatggcata gtatccggca
     2401 atgcggcatt tatgcggaag gattctacct tgaatgcacc tggggtggcc gacgagacta
     2461 tgtctttgac aaactctcga tgtcgtatga tcgttattcg atcatcagac ttaaataatg
     2521 ctggagtatt gcctgtgaca atactattgg aaccgacttt atagtcgccc caaccagtta
     2581 cttttttgaa gagtgcgtgg gctccgcccc ctataacttc ccctaacggg ccgaacttac
     2641 caccaatctg tgctccgaca gatttgcgcc agtcgggcgt gcggatcata ccgccctttg
     2701 gatcataaga gaaataatct ccatgaccgg ctaaattgat aactgctgct cgaggacctt
     2761 tcctcttcga catccccaaa gatcgcttaa cagcggcctt tgctgacttt ggcagtttca
     2821 tatattgtgc ttttggcaac atgatgcgtc ggatatgttc ggcgtataat acacctatac
     2881 aaatccgcgc ggcaatatta attaatattg cacaagaaat atttgtgcga cttacgcttc
     2941 gcggcgcgca acatttatta acatacgtta attaatacat tttttgaaaa aaaacgatgt
     3001 ggcgtggtaa ta
//
LOCUS       CruV-126                3019 bp    DNA     circular     24-FEB-2020
DEFINITION  Crucivirus-126 isolate 120_586_IR1_3296.
ACCESSION   CruV-126
KEYWORDS    .
SOURCE      Crucivirus-126
  ORGANISM  Crucivirus-126
            Unclassified.
REFERENCE   1  (bases 1 to 3019)
  AUTHORS   de la Higuera,I., Kasun,G.W., Torrance,E.L., Pratt,A.A.,
            Maluenda,A., Colombet,J., Bisseux,M., Ravet,V., Dayaram,A.,
            Stainton,D., Kraberger,S., Zawar-Reza,P., Goldstien,S., Briskie,J.,
            White,R., Taylor,H., Gomez,C., Ainley,D.G., Harding,J.S.,
            Fontenele,R.S., Schreck,J., Ribeiro,S.G., Oswald,S.A., Arnold,J.M.,
            Varsani,A., Enault,F. and Stedman,K.M.
  TITLE     Direct Submission
  JOURNAL   Submitted (24-FEB-2020) Biology, Portland State University, 1719 SW
            10th Ave, Portland, Oregon 97201, USA
COMMENT     Bankit Comment: ALT EMAIL:ide@pdx.edu
            Bankit Comment: TOTAL # OF SEQS:451
            Bankit Comment: GAP: est. length - alignment

            ##Assembly-Data-START##
            Assembly Method       :: MetaSPAdes v. 3.12; IDBA-UD v. 1.1.1;
                                     MEGAHIT v. 1.0.3
            Sequencing Technology :: 454; Illumina
            ##Assembly-Data-END##
FEATURES             Location/Qualifiers
     source          1..3019
                     /organism="Crucivirus-126"
                     /mol_type="genomic DNA"
                     /isolate="120_586_IR1_3296"
                     /isolation_source="Water"
                     /country="Norway"
                     /collection_date=2011
     CDS             1..963
                     /codon_start=1
                     /product="Putative Replication Associated Protein"
                     /translation="MNHRFMDEPRFVRGRPGLSWAARAPRVRAWPCYKTIRTWLCLFY
                     YVMGERIGGGKARYWMVTIREEDWNPTLPEGVEYIRGQLEVGEGGFRHWQVVMYFRKQ
                     VRLGAAKHSLGVPSAHLEPTRSRAAKDYVWKEETRVDGTKFELGSESVSRAEPTDWER
                     IWEFAKNGELTNIPADIRVRCYSSLRRIHADFAEPVGVVRSCVVFWGDTGTGKSRRAW
                     EEAGMDAYPKDPRSKFWCGYRGQKNIVFDEFRGGIDIGHLLRWLDRYPVNVEIKGGSV
                     PFAGEKVWITSNLDPRFWYPELDSATLEALMRRLTVTHFNKSLM*"
     CDS             983..2356
                     /codon_start=1
                     /product="Putative CruV Capsid Protein"
                     /translation="MPNPSGNVSIYGKLTALGPRKRKMAGKSRAGKRKRDDGMFDGYS
                     SKRRVIGSGDYKLFQQDVADISGRYKSNYQNRAQRVGRAIGSGVGAFGGWVADKVFGS
                     GDYVLGGRSATILNGKELPMFGNTSGNGGSTFIAHKEYLKDIQTGTAGTFSIEKFALQ
                     IADEKTFPWLSSIAENFEEYKLHGCIFEFKTTSSDALNSTNTALGTVILATNYNASRA
                     VFSSKQEMENYEFSQSAKPSLSQMHGVELATRDTPMTTYYIRNEALNANEDIKMYDYG
                     NFQIATTGFQASNVVIGELWVSYYVELLKPRIPKTIGGTPDSGWWAFTRISTAIDYAG
                     ATGTLVAKKGTLANNPDTSLTDTVLTVAGLEVGNLYEIMVQTICSTPAGVLTITATSG
                     AVVSNILQNYTQNGFATFGGTSSVSVYTFKATSTTGVFTAAGSTCASALQYVNIQIIT
                     LDKNIAP*"
ORIGIN
        1 atgaaccata ggtttatgga tgagccccgg tttgtccgtg gccgccccgg tttgtcctgg
       61 gcagcgcgtg ccccccgtgt ccgtgcatgg ccgtgttata agactataag aacgtggtta
      121 tgcttattct attatgtcat gggagaaaga attgggggag gaaaggcgag atactggatg
      181 gttaccattc gagaagagga ttggaacccc acacttccag aaggcgtcga atatattcga
      241 ggacagcttg aagttggaga aggaggcttc cgacactggc aggtcgtcat gtatttccga
      301 aagcaagtac gattgggagc tgcaaagcac agcttgggag tcccatctgc acacttggaa
      361 cccaccagat caagagccgc caaagactac gtgtggaaag aagaaacccg tgttgacgga
      421 acgaagttcg agcttggctc agaatcagtc tcgagagcgg aacccacaga ctgggaacgc
      481 atttgggagt ttgccaaaaa tggggagctt accaatatac cggcggatat tcgagtacgc
      541 tgttacagca gcttgcgaag aatccatgcg gattttgcgg aaccagttgg agtggtccgt
      601 tcttgtgttg tattctgggg tgacactgga acaggtaagt cgcggcgtgc ttgggaagaa
      661 gccggaatgg atgcttatcc taaagatcca aggagcaagt tctggtgtgg atacagaggt
      721 cagaaaaata ttgtatttga tgaatttcga ggaggaattg atatcggcca cttgctacgg
      781 tggttggata gatatccggt caacgtggaa attaaaggag ggtctgtccc ttttgccgga
      841 gaaaaagtat ggatcaccag caatctcgac ccgcgattct ggtatccaga actagattct
      901 gccaccctgg aagcgttaat gcggagatta acggttactc actttaataa aagtttgatg
      961 taaggaaata gtttactaat ttatgcccaa cccaagtgga aacgtgtcta tttatggaaa
     1021 gcttacagcc ctcggtcctc gcaagagaaa gatggcggga aagtctagag cgggaaaacg
     1081 aaaacgtgac gatggaatgt ttgacggata ttcttcgaag cgaagggtta tcggtagtgg
     1141 agactacaag ttgttccaac aagatgtcgc agacattagt ggccgctata agtctaatta
     1201 tcaaaatcgc gctcagcgtg ttggtcgtgc tattggttct ggcgtgggtg catttggtgg
     1261 atgggttgct gacaaagtat ttgggtctgg ggactatgtg ctcggaggac gaagtgctac
     1321 cattttgaat ggtaaagaac tgccaatgtt cggaaatacg tctggcaacg gcggatccac
     1381 ttttattgca cacaaggaat atctgaaaga tattcaaacc ggtactgctg gtacttttag
     1441 tatcgaaaag ttcgcacttc aaatagccga tgagaagact ttcccgtggc tttctagtat
     1501 tgccgagaat tttgaggagt ataagttgca cggatgtatt tttgaattca aaacaacttc
     1561 ctctgatgct ttgaacagta ctaatactgc gttgggtacc gtgattttag ccacaaatta
     1621 caatgcttcg cgtgctgtgt tttcgtcaaa acaggagatg gagaattatg aattttctca
     1681 aagtgccaag ccttcgctta gtcaaatgca tggagtagaa ttggccaccc gcgatactcc
     1741 tatgactact tactacattc gcaatgaagc actgaatgcg aatgaggaca ttaaaatgta
     1801 cgattatggt aattttcaaa ttgccacgac tggtttccag gctagtaatg tggtcatagg
     1861 agaattgtgg gtgtcctatt atgtggaatt gttgaaacca agaattccta aaaccattgg
     1921 cgggactccc gacagtggtt ggtgggcgtt tacgcgcatt tcgactgcta ttgattatgc
     1981 gggggctact ggcactttgg tggccaaaaa aggtactttg gctaacaatc cggacacttc
     2041 acttactgac actgtgttaa cagtggcagg acttgaagtg ggtaatttgt acgaaatcat
     2101 ggtgcaaaca atttgttcaa cgccagccgg tgttcttact atcaccgcta ccagtggtgc
     2161 tgttgtgtcg aacatcttgc aaaattatac tcagaatggt tttgcaacct ttggaggaac
     2221 tagctctgtt agtgtgtaca catttaaggc taccagcact actggtgtat ttacggctgc
     2281 tggttcaact tgcgcatcgg cgttacagta tgtgaatatt caaattatca cacttgataa
     2341 gaatattgct ccttaaaata aagtagggtt ttaagttttt attgtgtttt tcaatgtatt
     2401 tgattgggtc aatatctcgt atattgctac taggataagg aacatcataa gccaggcaga
     2461 gttcaaatag cgttcgacat ttggcggcat ggcgttcggc atcagcaatt gttttgatgt
     2521 tggtgttagt catactctgc gtgggaatgg tggttcttat atactagtgt ggctttgctg
     2581 cttacataat atgggctgtg ctcacccgat taggccgaag gccgtgtaga gagaatgttc
     2641 aaacttaaga tgatgttttg tgagttttgg tgcctatgac acccacataa tagtaataag
     2701 ccaaactttg agtgattgtt cgttacgttt tgtttgccta ctggtgcgga atgattcctg
     2761 gaagataatg gcggaggggc cccccgtagg gagggagggg ccctattagc tgatatccgg
     2821 gaatcattca cggttgggca aacaaaacgt aacgaacaat ttcctcaatt ttgctgatac
     2881 ggccaactgc tcaccccagc ttgggctggg ccggtccggg tgccctgtgg gctgggccgg
     2941 ccaggggggc cgcctgttga tctgtaaaaa cgttaacgaa ctgtggttcg ttatagtatt
     3001 acttacagat caacgatcc
//
LOCUS       CruV-127                3020 bp    DNA     circular     24-FEB-2020
DEFINITION  Crucivirus-127 isolate 120_287_SvL2_3298.
ACCESSION   CruV-127
KEYWORDS    .
SOURCE      Crucivirus-127
  ORGANISM  Crucivirus-127
            Unclassified.
REFERENCE   1  (bases 1 to 3020)
  AUTHORS   de la Higuera,I., Kasun,G.W., Torrance,E.L., Pratt,A.A.,
            Maluenda,A., Colombet,J., Bisseux,M., Ravet,V., Dayaram,A.,
            Stainton,D., Kraberger,S., Zawar-Reza,P., Goldstien,S., Briskie,J.,
            White,R., Taylor,H., Gomez,C., Ainley,D.G., Harding,J.S.,
            Fontenele,R.S., Schreck,J., Ribeiro,S.G., Oswald,S.A., Arnold,J.M.,
            Varsani,A., Enault,F. and Stedman,K.M.
  TITLE     Direct Submission
  JOURNAL   Submitted (24-FEB-2020) Biology, Portland State University, 1719 SW
            10th Ave, Portland, Oregon 97201, USA
COMMENT     Bankit Comment: ALT EMAIL:ide@pdx.edu
            Bankit Comment: TOTAL # OF SEQS:451
            Bankit Comment: GAP: est. length - alignment

            ##Assembly-Data-START##
            Assembly Method       :: MetaSPAdes v. 3.12; IDBA-UD v. 1.1.1;
                                     MEGAHIT v. 1.0.3
            Sequencing Technology :: 454; Illumina
            ##Assembly-Data-END##
FEATURES             Location/Qualifiers
     source          1..3020
                     /organism="Crucivirus-127"
                     /mol_type="genomic DNA"
                     /isolate="120_287_SvL2_3298"
                     /isolation_source="Water"
                     /country="Norway"
                     /collection_date=2013
     CDS             1..1095
                     /codon_start=1
                     /product="Putative Replication Associated Protein"
                     /translation="MDTQLTLKDGERYGTVGKWDPTLGKMVWEENKACILLKGEEQTS
                     YTLTNTKKPLILPPDRDEKKEPPKACHARKYQITVFDKNKTGIYNQVLAYLQSRKQLT
                     YIITCWEMCPDTKNEHWHIFCAFNNSVQINAKKICHSHVEVCRGSVQENIDYVKKCGK
                     YADEWNSSEEHHKNLFEWGTPPREANGKLSGAQLMEMTYKEIATEEPIYHRVYFAAKQ
                     YLEASIDIDDWAKKITVYYIQGPSGVGKTERAKQIVRDTYPIGQRQCDRVKFQGEFWR
                     GVNDAKICIYDEWRSSQMKPSEFISFIDYNRQSMNIKGGDKINNYELIIITSVEPLCN
                     IYGNVTGEPREQWERRITLIDMAPETPPPQ*"
     CDS             complement(1476..2879)
                     /codon_start=1
                     /product="Putative CruV Capsid Protein"
                     /translation="MSGRVSKEVWDAMSSSDQAVLREQLKASRPKKTYKKRAYRRAPP
                     SRQPRRTYRVPAGRGGSQNSVIFGNGDYFSDAASGLLKHGLPLVIKALTGFGDYSVAQ
                     NSMVPKLGGDPPIMMSTNQGGFIMRHREYVSDVYAQSSFTNNVLPINPGLLGTFPLAS
                     QIADTFEQYEIRGMVMEFKSMSSDAVLSAGSSSALGTVIFATQYNSLDLPFEDKRTME
                     NYEFANSTKPSCSMLHPIECKRSQTPVDLLYVRTGPVTTGDLRMYDLGNFNYAVQGCQ
                     NAAPGEVIGELWISFELEFFKPKLLPLGGSSTYSDHYTLSGNSLIAVGNPFYTQNGLT
                     LVSGSNLGTYIQVAALAPYAARSIIFPAQITDGIFKIDYYMVGSAASANSPNAGSSTL
                     VNCTLHPYWGGGTAGSIQLDPEPANTTKNFHFSEVINVNQVLPGIRAGINLDMTGVMP
                     SGNQILDIVITLLQASA*"
ORIGIN
        1 atggacactc aactgacatt gaaagatggt gaacgatacg gaacagtggg aaaatgggat
       61 cccacattag gcaagatggt gtgggaagag aacaaagcct gcatcttact caaaggagaa
      121 gagcagacaa gctacactct caccaacacc aaaaagcccc ttattctacc tccagacaga
      181 gatgagaaaa aggaacctcc caaagcatgc catgctagga aataccagat aactgtcttt
      241 gacaagaaca agactggtat ttacaaccaa gtcctagctt atctacaaag cagaaagcaa
      301 ctgacttata tcataacatg ctgggagatg tgtccagata cgaaaaacga acactggcac
      361 atattctgcg cctttaacaa ctctgtgcag atcaatgcca agaaaatctg ccacagccat
      421 gttgaagtct gcagagggtc tgttcaagag aacattgact atgtcaagaa atgtggcaag
      481 tatgccgacg aatggaatag ctctgaagag caccataaaa atctctttga atggggtact
      541 ccacctagag aagctaatgg taagctttct ggagcacagc tcatggagat gacatacaag
      601 gagattgcta ccgaagaacc aatctaccac cgtgtatact ttgctgctaa acaatactta
      661 gaagcatcta tagatataga tgactgggca aagaaaatca ctgtctacta catccaaggt
      721 ccaagtggtg taggaaagac agaacgagct aaacagatcg ttagagatac ttatcctata
      781 ggacaaagac aatgtgatcg agtcaaattt caaggagaat tctggcgagg tgtcaacgat
      841 gcaaaaatct gtatctatga tgaatggaga tcctcacaga tgaaaccctc tgaattcatc
      901 agctttatcg actacaatcg acaatcgatg aacatcaagg gtggtgataa gataaacaac
      961 tacgagttga ttatcatcac atctgtggaa cctctatgta acatttacgg aaatgtaact
     1021 ggagaaccca gagaacaatg ggagcgaaga atcactctca ttgatatggc tccggagact
     1081 cctcctccgc aataaactcc tcaatacaat gtacatcgta ttgaagactt tcaatcgctt
     1141 gctctaatct agcagtgaca tcaccgcaag acagagccaa gcgatgaata gtaagttgga
     1201 gatcttcaac agcgctctct aacttcacaa ccttaatttg catagcatga aggagaggga
     1261 acaaaccagg atactctaca gtgtccattt atataacgat atatattttc ttggtagatt
     1321 cccctctcct ggatcctccc ttccggaagg gcgcaagcgc ccttccttgt agggccaccc
     1381 acccctcgag gggggatttc ccctccggat tacctggcac agcctattct atgcatattc
     1441 tagtagaaat gctatacacc tgttcagttg tatacttaag cactagcttg aagaagagta
     1501 atgacaatat ctaagatctg gttacccgat ggcattacac cagtcatatc aagattgata
     1561 ccagcacgaa taccaggaag aacctggttt acattgatga cttcagagaa gtgaaaattc
     1621 ttagttgtat tagcaggctc aggatcgagc tgaatagaac ctgcagtacc tccaccccaa
     1681 taaggatgta acgtacaatt cacaagagtt gatgaacctg cattgggact gttagcagaa
     1741 gcagcagatc ccaccatgta ataatcaatc ttgaaaatac cgtcagtaat ctgcgcaggg
     1801 aagataatcg aacgtgccgc ataaggggca agtgcagcca cttgaatata cgtcccgaga
     1861 ttacttcccg acacaagggt taatccattc tgtgtataga atggattacc cacagcgatc
     1921 aaactgttac cagacaaggt ataatgatcg gaataggtag atgaaccacc taatgggaga
     1981 agcttaggct taaagaactc aagttcgaat gaaatccata actcacctat aacctcacca
     2041 ggagctgcat tttgacatcc ctgaactgca tagttgaaat taccaagatc atacattcga
     2101 agatcaccgg tagtaacggg accagttctc acatagagga gatcaacagg agtctgactc
     2161 ctcttacatt caattggatg aagcatacta caactaggtt tagttgaatt ggcgaactca
     2221 tagttctcca tggtacgctt atcctcgaaa ggcaagtcca aagaattgta ctgagttgcg
     2281 aagataaccg tacctaatgc cgatgacgaa cctgcgctaa gaacagcatc agatgacatc
     2341 gacttaaact ccatgaccat tcccctaatc tcatactgct cgaacgtatc ggcaatctga
     2401 gatgcaagag ggaaggttcc taggagtcca ggattgatag gaagtacgtt gttagtaaac
     2461 gacgactgag cataaacatc gctaacatac tcacgatgac gcataatgaa tccaccctga
     2521 ttagtgctca tcataatcgg tggatcacca ccaagcttag gaaccataga attttgagct
     2581 acagagtagt ctccaaaacc tgtcaaagct ttgataacca atggaaggcc gtgtttaaga
     2641 agacctgaag cagcgtcaga gaaatagtca ccatttccaa aaatgacact gttctgagaa
     2701 cctccacgac ctgcaggaac gcggtacgtt cgacggggtt gacgggaagg aggagcacgg
     2761 cgatatgccc tcttcttgta ggtcttctta ggtctcgagg ctttaagctg ctcacggaga
     2821 acagcttgat cggatgaact catcgcatcc catacctctt tagaaacacg cccagacata
     2881 ttccttttat taacaaaaaa agtaaaataa aaaaataaat tttagctgct gttttttcat
     2941 cctcatggcg agccataagg ctaaaaaaaa acagctaaaa gaatcaacgc ctcaggtaat
     3001 actaaacctt cggcgtttta
//
LOCUS       CruV-128                3023 bp    DNA     circular     24-FEB-2020
DEFINITION  Crucivirus-128 isolate 001819_virusbaton.
ACCESSION   CruV-128
KEYWORDS    .
SOURCE      Crucivirus-128
  ORGANISM  Crucivirus-128
            Unclassified.
REFERENCE   1  (bases 1 to 3023)
  AUTHORS   de la Higuera,I., Kasun,G.W., Torrance,E.L., Pratt,A.A.,
            Maluenda,A., Colombet,J., Bisseux,M., Ravet,V., Dayaram,A.,
            Stainton,D., Kraberger,S., Zawar-Reza,P., Goldstien,S., Briskie,J.,
            White,R., Taylor,H., Gomez,C., Ainley,D.G., Harding,J.S.,
            Fontenele,R.S., Schreck,J., Ribeiro,S.G., Oswald,S.A., Arnold,J.M.,
            Varsani,A., Enault,F. and Stedman,K.M.
  TITLE     Direct Submission
  JOURNAL   Submitted (24-FEB-2020) Biology, Portland State University, 1719 SW
            10th Ave, Portland, Oregon 97201, USA
COMMENT     Bankit Comment: ALT EMAIL:ide@pdx.edu
            Bankit Comment: TOTAL # OF SEQS:451
            Bankit Comment: GAP: est. length - alignment

            ##Assembly-Data-START##
            Assembly Method       :: MetaSPAdes v. 3.12; IDBA-UD v. 1.1.1;
                                     MEGAHIT v. 1.0.3
            Sequencing Technology :: 454; Illumina
            ##Assembly-Data-END##
FEATURES             Location/Qualifiers
     source          1..3023
                     /organism="Crucivirus-128"
                     /mol_type="genomic DNA"
                     /isolate="001819_virusbaton"
                     /isolation_source="Water"
                     /country="France"
                     /collection_date=2013
     CDS             1..1086
                     /codon_start=1
                     /product="Putative Replication Associated Protein"
                     /translation="MTEEIKTVSLEYVDVKYDEVGRVIYTGDFLKQRSRNWLFTWNNY
                     TEADIGTVKTAWPASGNFRGLMFELEVAPTTGTAHLQGFICFDSMKSGKQIKELSPKV
                     NFRVMLGSIEQNKTYCSKDASGVVVLGTFPMSQKDKGVRGIAGAKAPNTWGALVEDMK
                     AGKTRRELYELYPKLAGTSPSGFEKLYQEFCPVGTYSILEHFGAFLPWQKELADLVQA
                     GPDERSVIWIYSSAGGVGKSAMYKHLVYNAGIQPMKNATTRDLACAWKCGSVTFDFAR
                     DMGDEKINYSFIENVKDGICFSSKYESASKLADPNVHRFVICFANEPPRVEALTLSRW
                     MIYDIVNSKLVSMPASLYVKNMNTDNR*"
     CDS             1593..2921
                     /codon_start=1
                     /product="Putative CruV Capsid Protein"
                     /translation="MPRVAGAKAAPRKRAPRKKPAGSVGRLHGHGDYTYDKPGPWGKI
                     GRSLGEHAGGMIGSGYGFKGAGSALGGKLGSYLHYIGKIFGSGDYVTAANQLKHNVLT
                     NSTQIPVFSDGANDVRIRHREFLGDIISSNTAAAFRLQSFPINPGLLSSFPWLSQVCG
                     ATWQQYRVNGMVFEFRSMSADALNSTNTALGSVVMATDYDSADAVFTSKADMENTEFG
                     VSCKPSSSMIHGIECARYQTAMSEQYIRSAAVPANKDIRMYDLGNFQIATVGFQGQSV
                     NCGELWVSYDVSLLKTKGSPPGAVMGLANLQIAGATAALPLGTGFTIAAAGQDSIGIT
                     WVTGNLLSFPVLPIGTTFLLTYNLSVASAALTLPTITAGGGMTILAGFKAPAAGETAT
                     KGMCATWVQITSLVSTTAPYVNYAGMTSGAPGVTQYLDIYQVNGTTLQSP*"
ORIGIN
        1 atgactgaag agattaagac ggtgtcactg gagtatgtcg atgtgaagta tgacgaagta
       61 ggacgcgtca tctacacagg cgactttctc aagcaacgca gtcgtaattg gttgtttacg
      121 tggaacaact acacagaagc ggatattggc acagtaaaaa cggcctggcc ggcctctggc
      181 aacttccgcg gtctgatgtt tgaacttgaa gtcgcgccta ccacgggaac ggcacacctt
      241 caggggttca tttgcttcga ttctatgaag tctggcaagc agatcaaaga actcagccca
      301 aaggtcaact ttagagtcat gctcggttct atcgaacaga acaagactta ctgcagtaag
      361 gacgcttcgg gtgtagtggt cttggggacc tttccaatgt cgcaaaaaga caaaggtgtt
      421 agaggcatag ctggcgctaa agccccaaac acttggggtg ctcttgttga agacatgaaa
      481 gctggcaaaa ctaggcgcga gctctacgag ctatacccca agttagccgg cactagtcct
      541 tctggcttcg agaagcttta tcaggaattc tgcccggttg ggacatactc aatacttgag
      601 catttcgggg cattccttcc ttggcagaag gaattagccg acctcgtcca ggcaggacca
      661 gatgaacgat cggttatctg gatttactcg agcgctgggg gcgtcggtaa gtccgcaatg
      721 tataagcacc tcgtttacaa cgcgggcata cagcccatga agaacgccac tacgcgcgat
      781 cttgcctgtg cgtggaaatg cggttctgta acgtttgact ttgctcgtga tatgggagac
      841 gagaaaatca attacagctt catcgagaac gtcaaggacg gtatctgctt cagtagcaaa
      901 tatgagtccg cgagcaagct agcagaccca aatgtacacc ggttcgtcat ttgcttcgct
      961 aacgaacctc ctcgtgtaga agctcttacg ctcagccgtt ggatgatcta cgacatagtc
     1021 aatagcaagc tagtgagcat gcctgcaagc ttatacgtga agaatatgaa cacggataat
     1081 agataaggct aaaacattat tatgattctt actgttcaat tcaataatcg tgatctgtga
     1141 tcactagggg gtctaggtaa tactaggctg cgccaccgac cccctaaaga tcagacttag
     1201 atcaagatat agagctatag aattcaatat cgattcgaat cgagatagac attcgcttgc
     1261 cacacctggc aagcaaaagt agaattcttt ttttgatgga tccggtttca ttgcccttgc
     1321 tggggattgc tgcgccggcc gcacgtggac gctatccgca ggcggcgttc ggggcgaacg
     1381 cctgcgggcg tccccatgct ggcgcatcaa ccgccagcaa ggacaactcc cggatcttat
     1441 atggtattgt atgaataggt acggcctttc gaccgattgg ccggactgga gtaactatag
     1501 atagttctag agttaggcca ttatttaatt ttttgattaa aaggtttcgt gtacctactg
     1561 tatacgacag tcagtcaaac gattgttttt gaatgcctcg agttgccgga gctaaagcag
     1621 cgcctcgcaa gagagcaccc agaaagaagc cagcgggttc tgttgggcgt ctccatgggc
     1681 atggtgacta cacttatgac aagccggggc cttgggggaa gattggtcga tctctaggag
     1741 agcacgccgg tggcatgata ggttcaggct atggcttcaa aggcgccggt tcggcacttg
     1801 gtggtaagct aggtagctat cttcactata tcggcaaaat ctttggttct ggtgactacg
     1861 tcactgcggc taaccagctc aaacacaatg tgctgaccaa ttcgacgcaa attcccgttt
     1921 tctctgatgg agcgaacgat gtccgcatta ggcataggga gttccttggt gatatcatca
     1981 gttcgaacac agcggctgca ttcagacttc aaagcttccc gatcaatcct ggcttactaa
     2041 gctccttccc ttggctatcg caagtgtgcg gcgcgacgtg gcagcagtat cgagtcaatg
     2101 ggatggtctt cgagttccga agcatgtccg ccgatgcgtt gaactccacg aatacagccc
     2161 ttgggtcagt agtcatggct acggattacg acagtgcaga cgctgtcttt acttcgaaag
     2221 cagatatgga gaacaccgaa tttggggtct cgtgcaagcc aagctcaagc atgatccatg
     2281 ggatcgagtg cgctcgctac caaactgcga tgtctgagca atacatcagg tctgctgctg
     2341 tccctgccaa caaggatata cgtatgtacg atctcggtaa cttccaaatt gcaactgttg
     2401 gttttcaagg ccaaagtgta aattgcggtg aactgtgggt cagttacgat gtttcattac
     2461 tcaagacaaa gggaagtcct ccgggagctg tgatgggcct tgccaatctg cagatcgccg
     2521 gggctaccgc cgcgttgccc cttggtactg gatttactat agcagcggct ggtcaagata
     2581 gcatagggat tacgtgggtc acgggtaatc ttctgtcttt cccggttcta ccgataggaa
     2641 caacatttct gttgacttac aacctcagcg ttgcttctgc cgcattgacg ttaccgacaa
     2701 tcacagcagg tggaggtatg acgatattgg ctgggttcaa ggccccagct gcaggtgaga
     2761 cagcaaccaa aggtatgtgt gcaacctggg ttcaaatcac gtcgctggtt tccacaactg
     2821 ctccgtatgt gaattacgcc ggtatgacaa gtggagcgcc tggggtcact caataccttg
     2881 atatctacca ggtaaacgga acaactctac aatcgccata agacggcatc gatgggtggt
     2941 tcgggggtgt tagctagtaa caccctggat tattattttt tttgtcttaa gtaagttgaa
     3001 cggtccatgt atagacacgc taa
//
LOCUS       CruV-129                3026 bp    DNA     circular     24-FEB-2020
DEFINITION  Crucivirus-129 isolate 001814_virusbaton.
ACCESSION   CruV-129
KEYWORDS    .
SOURCE      Crucivirus-129
  ORGANISM  Crucivirus-129
            Unclassified.
REFERENCE   1  (bases 1 to 3026)
  AUTHORS   de la Higuera,I., Kasun,G.W., Torrance,E.L., Pratt,A.A.,
            Maluenda,A., Colombet,J., Bisseux,M., Ravet,V., Dayaram,A.,
            Stainton,D., Kraberger,S., Zawar-Reza,P., Goldstien,S., Briskie,J.,
            White,R., Taylor,H., Gomez,C., Ainley,D.G., Harding,J.S.,
            Fontenele,R.S., Schreck,J., Ribeiro,S.G., Oswald,S.A., Arnold,J.M.,
            Varsani,A., Enault,F. and Stedman,K.M.
  TITLE     Direct Submission
  JOURNAL   Submitted (24-FEB-2020) Biology, Portland State University, 1719 SW
            10th Ave, Portland, Oregon 97201, USA
COMMENT     Bankit Comment: ALT EMAIL:ide@pdx.edu
            Bankit Comment: TOTAL # OF SEQS:451
            Bankit Comment: GAP: est. length - alignment

            ##Assembly-Data-START##
            Assembly Method       :: MetaSPAdes v. 3.12; IDBA-UD v. 1.1.1;
                                     MEGAHIT v. 1.0.3
            Sequencing Technology :: 454; Illumina
            ##Assembly-Data-END##
FEATURES             Location/Qualifiers
     source          1..3026
                     /organism="Crucivirus-129"
                     /mol_type="genomic DNA"
                     /isolate="001814_virusbaton"
                     /isolation_source="Water"
                     /country="France"
                     /collection_date=2013
     CDS             1..984
                     /codon_start=1
                     /product="Putative Replication Associated Protein"
                     /translation="MEAAKSNEKDEMEITETKVEVYREVGGKKVRVYEAKKNCWCFTW
                     NNYPIDYKEKLAKLLPHASFMVYGLEVGDKCHTPHVQGYVRFAKGKAATTLHNIIEKC
                     YFVPAKGDDQANLAYCSKQDPDFFQYGEPKSQGKRNDIIAIREAVQEKKSMRDMLTDG
                     TLVNYQGIRVAEKLLDYFEPARHPSDGTFTFEWWYGTTGVGKSYDALMEYPGYYSFTQ
                     KDMKWWNGYDAHIGVIIDDFRDTMGPMSEFLRIWQPFPHRVEAKGVMRQLRAMSFIAT
                     TCHSPLQVYRGILENRNQLYRRITGIRRYYDKNTYVKQKFNDDYTALVDIA*"
     CDS             1275..2660
                     /codon_start=1
                     /product="Putative CruV Capsid Protein"
                     /translation="MSSRKYSDAQKAAYYAKRARVGKPKKKYSKSKSYSMISGSGDYT
                     YARPGPFGRVGRAVGKVIGGIGGGLVGAPYGPGSAIVLGKAGSAVGGELGGLAHYIGK
                     IFGSGDYKTGVMPSSNVLTNTRQIPQFSTSKMTNVVCHREYLQDVISSATPGAFKIQS
                     FDINAGLSGSFPWLAQVAANYEQFKIHGMVFEFRTMSADALNSTNTALGQVIMAVEYN
                     SANPNFVNKQQMENYEYAVSCKPSDSMLCAVECARGQTPVIELYVRTGAVPTNQDQRL
                     YDLGNFQIATNGLQAASVNVGELWVTFCVELLKPRLYSGPLGYNIPTLHRQLTSSVTT
                     SDYFNSISTPLDTIGLTVGLTTLTFPAAILAGTYLIQWSMIGSSTASVQCPTFAATTN
                     CVGVAIYNNNSGLTVRQPVAPTTTNTVNCVACFSITGPSAVITLSGGTMPGSLLYGDL
                     VVSQCSALLVA*"
ORIGIN
        1 atggaagccg ccaagagcaa cgagaaggat gagatggaga ttaccgagac aaaagttgag
       61 gtttatcgtg aagttggtgg aaagaaagtt cgagtttacg aggccaaaaa gaattgttgg
      121 tgctttactt ggaacaacta tccgattgat tataaggaaa aacttgcaaa gttgcttccg
      181 catgcatctt ttatggttta tggacttgag gtaggtgaca agtgtcacac tcctcatgtt
      241 cagggttatg tacgatttgc taaaggtaaa gccgctacaa ctctccataa tattattgaa
      301 aagtgttact ttgtgccagc aaagggtgac gatcaggcta atctggcata ttgctctaag
      361 caagatccag atttcttcca gtatggagag cctaagtcac aaggtaagcg taatgatatt
      421 atagctattc gcgaagccgt tcaggaaaaa aaaagtatgc gcgacatgct taccgatgga
      481 acattagtga attatcaagg cattcgagtc gccgaaaagt tacttgatta ctttgagcca
      541 gctagacatc cgtcagatgg tacttttact tttgagtggt ggtatggtac gactggcgtt
      601 ggtaaatctt acgacgcatt aatggagtat ccaggttatt attctttcac acagaaagat
      661 atgaaatggt ggaatggtta tgatgctcat attggagtca ttattgacga ctttcgagat
      721 acaatgggac ctatgtccga gtttcttcgt atatggcaac catttccaca tagggttgaa
      781 gctaaaggag ttatgcgaca gcttcgagcc atgtcattta ttgcaacaac ttgccattct
      841 ccactccaag tttatcgagg aattttagag aatcgcaatc agttgtatcg gcgtataaca
      901 ggtattcgta ggtactatga taaaaatact tatgttaaac aaaaatttaa tgatgattat
      961 actgccttag tagacattgc gtgagcgacc gacaagcgtt agcgaggaag ggtagctctt
     1021 cttccattaa ttttagcctt ttctttttag tagacaaact gatttaattt tgttaaaaga
     1081 agttacttga gcaccagaat gagatgcgaa agtagatcgg aatgaacgcg cggacttcgc
     1141 ccacccattg gatagtgggc ggagtctaag tgtttgtgta gatcgaatga agcatctgaa
     1201 tggaggtgca agtatacttt ggtcctaaat tgtatttagt ttaacaaagt ttatatattt
     1261 ctatatatct caggatgtct tctcgaaagt attcggatgc ccaaaaggcc gcgtactacg
     1321 ctaagcgtgc acgtgttggg aagcctaaaa agaagtactc taagtcaaag agttactcca
     1381 tgatttccgg ttccggtgat tatacttatg ctcgaccagg tccgtttggc cgtgttggta
     1441 gagctgttgg aaaggttatt ggcggtattg gtggaggtct tgttggagct ccttatggtc
     1501 ctggttctgc cattgttctt ggcaaagcag gttccgctgt tggcggtgaa cttggtggtc
     1561 ttgctcatta cataggcaag attttcggtt ccggtgatta taagaccgga gtgatgccta
     1621 gttcaaatgt gttgacaaac acccgacaaa ttccacagtt ttccacctca aaaatgacga
     1681 acgttgtttg tcatcgtgag tatcttcagg atgttatttc atccgctact cctggtgctt
     1741 ttaagatcca gtcatttgac attaatgctg gtctttcagg atcctttcct tggttggctc
     1801 aagttgctgc caattatgaa cagtttaaga ttcatggtat ggtgtttgaa tttcgcacaa
     1861 tgtctgctga tgcgttgaat tccaccaata cagcacttgg ccaagtaatt atggctgttg
     1921 agtacaattc agccaatccc aattttgtca ataagcaaca gatggaaaat tatgaatatg
     1981 cagtttcttg taaaccatca gattcaatgt tgtgtgctgt ggagtgtgct cgtggacaga
     2041 ccccagttat tgaactttat gttcgaacag gagccgttcc aacaaatcaa gatcagcgtc
     2101 tttacgatct tggtaacttt cagattgcaa ctaatggtct gcaggctgct tcagttaatg
     2161 ttggtgaact ttgggttacg ttctgtgttg aacttcttaa accccgtctt tatagtggac
     2221 ctcttgggta caatattcct acgttgcatc gtcaacttac atcgtccgtt actacgtcag
     2281 attatttcaa ttctatcagt acacctcttg atactattgg tttgactgtt ggtctcacca
     2341 ctttaacttt ccctgcagcc attttggcag gaacttatct gattcaatgg tcaatgattg
     2401 gttcttctac tgcgtcagtt cagtgtccga cttttgctgc tacaactaat tgtgttggtg
     2461 ttgcaattta caacaacaac tctggtctta cggttcgtca acctgtagct ccgactacta
     2521 cgaacactgt taattgcgta gcttgcttta gcattaccgg accttctgca gtaatcactc
     2581 tttctggtgg tactatgcct ggcagcttgc tgtatggtga tttggttgta tcgcagtgtt
     2641 ctgcactatt ggttgcatga aaaaccgaat cagtttatga tccagaagat tacgcaagcg
     2701 tttaccttag tacttctagc agcttcgtct ttgaagataa tgtatctgac aattcgtcgc
     2761 ttgatggatt cgacgtcata tgatgtttaa aaaaagaaat atggcaggac gctcggcgga
     2821 tgaaagacgc cgttttgtgt ccataagtcc aattttttgt ttttggagtg tttaacttag
     2881 tgggaggacg cccgtagggt ggacgaaaag tcggggtgga acatagttcc tttattaccc
     2941 ccgacttgtc cccactagtc cccccacata ggtggtgggg agaagtgtta taaagtttct
     3001 aattttatat atagacaagt acctcc
//
LOCUS       CruV-130                3026 bp    DNA     circular     24-FEB-2020
DEFINITION  Crucivirus-130 isolate 001815_virusbaton.
ACCESSION   CruV-130
KEYWORDS    .
SOURCE      Crucivirus-130
  ORGANISM  Crucivirus-130
            Unclassified.
REFERENCE   1  (bases 1 to 3026)
  AUTHORS   de la Higuera,I., Kasun,G.W., Torrance,E.L., Pratt,A.A.,
            Maluenda,A., Colombet,J., Bisseux,M., Ravet,V., Dayaram,A.,
            Stainton,D., Kraberger,S., Zawar-Reza,P., Goldstien,S., Briskie,J.,
            White,R., Taylor,H., Gomez,C., Ainley,D.G., Harding,J.S.,
            Fontenele,R.S., Schreck,J., Ribeiro,S.G., Oswald,S.A., Arnold,J.M.,
            Varsani,A., Enault,F. and Stedman,K.M.
  TITLE     Direct Submission
  JOURNAL   Submitted (24-FEB-2020) Biology, Portland State University, 1719 SW
            10th Ave, Portland, Oregon 97201, USA
COMMENT     Bankit Comment: ALT EMAIL:ide@pdx.edu
            Bankit Comment: TOTAL # OF SEQS:451
            Bankit Comment: GAP: est. length - alignment

            ##Assembly-Data-START##
            Assembly Method       :: MetaSPAdes v. 3.12; IDBA-UD v. 1.1.1;
                                     MEGAHIT v. 1.0.3
            Sequencing Technology :: 454; Illumina
            ##Assembly-Data-END##
FEATURES             Location/Qualifiers
     source          1..3026
                     /organism="Crucivirus-130"
                     /mol_type="genomic DNA"
                     /isolate="001815_virusbaton"
                     /isolation_source="Water"
                     /country="France"
                     /collection_date=2013
     CDS             1..1296
                     /codon_start=1
                     /product="Putative CruV Capsid Protein"
                     /translation="MSEKKGNKKSNPKSSNSAKKSKRPKKITAERSFMKTYVPKGSFE
                     SGGKMLGNFLGGPLGGTLGGKAGDLLSKIVGFGDYSLQGNSIMTGGLSPPEIVNSVNR
                     GGIIVRHREYLQDVIGSTVPFQLTNFSINPGLQSSFPWLAQIAGAFEEYEFRGLIYEF
                     KSLSSDSTPLTTTTALGYVAMATQYNAAAPDFANKKELENYEFANSAKPSCSFIHPIE
                     CKRSLNVDTHLYTRTGAVPLGQDQKTYDLGDFQIAVGGMPVNGGIIGELWCTYEIEFF
                     HPKYVIANNVDSDHFFLSGAGIFTNGQPVSLGTTLTRSTGSNLGMSCANNNMTFRNDI
                     SGGTFLFVLKWSGTGAVAVAYPVITTSNCTLLQVMSGGSTTSTSAPPAGTSSTQCMLE
                     FYVRVDAQNASVILNGAGTLPTGTQQIEYWIMESATITA*"
     CDS             complement(1782..2927)
                     /codon_start=1
                     /product="Putative Replication Associated Protein"
                     /translation="MSKSFCWTLNNYSEKEENTIKDLYDKYANYIVFGKEKGEECHTP
                     HLQGYIELTNIMKLTTLKNIIGKRCHLELRSGSQNDAVNYCKKGEQTKSEWREKKEKG
                     ANFGLNADVFEIGILAVKHQGKRTDIAKCRSLAKIGGMRAVTKQATSLQSIKVAEKFL
                     EYNEKKRDFKTNVIWIWGSSGTGKSHKARERCADINDIFTKNDGSKWWIGYDGHESVI
                     IDDFRDSWWGITEMLSLLDKYEKRIETKGGSRQLLAKTIIVTSIHSPNDCYKGCADEP
                     IQQLLRRIDETIHLTSVYNSTANEKNIEDLSAFFPVEEVAVEEVEGNTRDLDFINEDF
                     LDAPKYKFEVKKPLFENKKIEKKNCICTCCQYCDCCDCDSSICIFAD*"
     misc_feature    1942..1972
                     /note="stem-loop"
     misc_feature    1955..1963
                     /note="nonanucleotide"
ORIGIN
        1 atgtcagaaa aaaagggaaa caaaaaatca aatccaaagt caagtaattc tgctaaaaag
       61 tctaagagac ccaagaaaat tacagccgaa agaagtttta tgaaaactta tgttcctaaa
      121 ggttcatttg aatctggagg aaaaatgctg ggtaattttc tgggtggtcc tttagggggc
      181 actttaggtg gaaaagcggg tgatttatta tcaaaaattg ttggcttcgg ggattatagt
      241 ctccaaggta attcaattat gacaggtgga ttatcccctc cagagattgt caatagtgtc
      301 aatcgagggg gtataattgt gagacatcgc gagtatctac aagatgtaat tggatctact
      361 gtaccatttc aattgactaa tttttctatc aatccgggac tacagtcttc ttttccatgg
      421 ttagcccaga ttgctggagc ttttgaagaa tatgaatttc gtggtttaat ttatgaattc
      481 aaatcattat cttcagattc tacaccattg acaacgacaa ccgccttagg ttatgttgca
      541 atggcaacac aatataatgc ggccgcacca gattttgcaa ataaaaaaga attagagaat
      601 tatgagttcg caaattcagc aaaaccgtcc tgttcattta ttcatccaat tgaatgtaag
      661 cggtctttaa atgtagatac tcatttatat acgcgtacag gagccgttcc tcttggacaa
      721 gatcaaaaaa cctatgattt gggtgatttt caaatagccg ttggagggat gcccgtaaat
      781 ggtggtatta taggggaact ttggtgtacc tatgagattg aattttttca tcctaaatat
      841 gtaatagcga ataacgtaga ttctgatcat ttctttttga gtggcgcagg catttttact
      901 aatggtcagc cagtgtctct cggaacaact ttaacgcgct caactggttc aaatttggga
      961 atgtcttgtg caaataataa tatgacgttt cgaaatgata tatcaggagg aactttctta
     1021 tttgtattaa aatggtcagg tacaggagcg gttgcagtag cctatcctgt aatcacaact
     1081 tcaaattgca cattattgca agtgatgagt ggtggttcga ctacaagtac aagtgctccg
     1141 ccagctggta cttcatcaac tcaatgtatg ttggaatttt atgtaagagt tgatgctcaa
     1201 aatgcttcag taattttgaa tggcgcagga acattaccta cagggactca acaaattgag
     1261 tactggataa tggaaagtgc aacgattacc gcttaaatta aaagagaaag gggtaacaag
     1321 ataaataata taaattaatt tttgggttat cgggccctcc gcccgcggaa taagcccgaa
     1381 tccaaaaaaa tattttctca taatatagta tgggaaaata aaaataaaaa attgttgtga
     1441 gtaacttggt tgtgtcattt tgataattaa atagtataat ataataatac tatttgataa
     1501 agtccattgt aggacaaatt ttatagacat tttttaaaaa actgttaaaa ttctaggccc
     1561 gttaggtccg aattttcagt tttttaataa ttgtctataa aattcgttcg ttggtcgcag
     1621 tttttgccag cacgtagtgt acagagcgaa gcaatatttt cacttgtgaa aatttgatga
     1681 gttcgggcaa aaacgttaaa ttatattaga aaaatgattt aactaaatta gcgaagcgtc
     1741 ctttaaggcg tagccttagt gtccgttagg acccatatat tttaatcagc gaagatacat
     1801 attgagctgt cacaatcgca acaatcgcag tattgacagc atgtacatat gcagtttttt
     1861 ttttctattt ttttattttc aaataatggc ttttttactt caaatttgta ttttggagca
     1921 tctaaaaagt cttcatttat aaagtcgagg tctctagtat taccctcgac ttcctctacc
     1981 gctacttcct ctactggaaa aaatgcactt aaatcttcga tatttttctc attagcggta
     2041 gagttgtaaa cggacgtaag atgaattgtt tcatcaattc gtctgagtaa ctgttgtatt
     2101 ggttcatcgg cacaaccttt gtagcagtcg tttggactat gtatggaggt gacaataatg
     2161 gttttagcta ataattgtct cgaaccgcct ttagtctcaa ttcttttttc gtatttatct
     2221 aataaagata acatttctgt tataccccac caactgtcgc gaaagtcatc tattatgact
     2281 gattcgtgac cgtcgtaacc aatccaccat ttactaccat catttttcgt aaaaatgtca
     2341 ttaatatcag cacatcgttc tcttgctttg tgtgatttac cagttccact ggatccccat
     2401 atccaaataa catttgtttt gaaatccctt tttttctcat tatattctaa aaacttttca
     2461 gcaaccttta tgctttgtaa gctagtggct tgcttagtga ctgccctcat tccgccaatt
     2521 ttggctaatg aacggcattt agcaatatcc gtccgtttac cttgatgttt tacggctaat
     2581 atacctattt cgaaaacatc ggcgttcaaa ccaaagttag cccctttctc ttttttttct
     2641 ctccactcac ttttagtttg ttcgcctttc ttacaataat taactgcatc attctgacta
     2701 ccagatctta attctaaatg acatcgttta ccgataatgt ttttaagtgt agttaatttc
     2761 attatatttg taagttctat atatccttgt aaatgaggag tgtgacattc ttcacctttc
     2821 tctttaccga atacgatata attagcgtat ttatcatata aatctttaat agtattttct
     2881 tctttttcgc tatagttgtt taatgtccaa caaaaacttt tgctcattta atatactata
     2941 gtaaatataa tattttatat gtatttaatt aaattaaatt aaataaaata cttaaagatt
     3001 attttctcta tatatagtat taacaa
//
LOCUS       CruV-131                3030 bp    DNA     circular     24-FEB-2020
DEFINITION  Crucivirus-131 isolate 120_232_SvL2_3298.
ACCESSION   CruV-131
KEYWORDS    .
SOURCE      Crucivirus-131
  ORGANISM  Crucivirus-131
            Unclassified.
REFERENCE   1  (bases 1 to 3030)
  AUTHORS   de la Higuera,I., Kasun,G.W., Torrance,E.L., Pratt,A.A.,
            Maluenda,A., Colombet,J., Bisseux,M., Ravet,V., Dayaram,A.,
            Stainton,D., Kraberger,S., Zawar-Reza,P., Goldstien,S., Briskie,J.,
            White,R., Taylor,H., Gomez,C., Ainley,D.G., Harding,J.S.,
            Fontenele,R.S., Schreck,J., Ribeiro,S.G., Oswald,S.A., Arnold,J.M.,
            Varsani,A., Enault,F. and Stedman,K.M.
  TITLE     Direct Submission
  JOURNAL   Submitted (24-FEB-2020) Biology, Portland State University, 1719 SW
            10th Ave, Portland, Oregon 97201, USA
COMMENT     Bankit Comment: ALT EMAIL:ide@pdx.edu
            Bankit Comment: TOTAL # OF SEQS:451
            Bankit Comment: GAP: est. length - alignment

            ##Assembly-Data-START##
            Assembly Method       :: MetaSPAdes v. 3.12; IDBA-UD v. 1.1.1;
                                     MEGAHIT v. 1.0.3
            Sequencing Technology :: 454; Illumina
            ##Assembly-Data-END##
FEATURES             Location/Qualifiers
     source          1..3030
                     /organism="Crucivirus-131"
                     /mol_type="genomic DNA"
                     /isolate="120_232_SvL2_3298"
                     /isolation_source="Water"
                     /country="Norway"
                     /collection_date=2012
     misc_feature    join(3019..3030,1..21)
                     /note="stem-loop"
     misc_feature    1..9
                     /note="nonanucleotide"
     CDS             45..1250
                     /codon_start=1
                     /product="Putative Replication Associated Protein"
                     /translation="MMDLDPRIVYDYSTSRSELTGVLASEPPVQGGSEQGSPAREDPN
                     WLDEAQALSERLAVDPAEQVLTPHTAGDRTPLAVHWLFTLNNYTEQDEAAFSANLNLF
                     KGACYGREVGEQGTPHLQAFVSFAKQSRGSQLAKIWPRAHMAIAKASPWINWLYCSKG
                     EQPKTEWTAYREKGPTYGRNAQFFHFGTRPVCPKKGSNRRDLHDVTAECYAMLEDGTI
                     QSAMDHLKVHRPADHAKYNVQYRNAFTAETKPKTFQHIYVDTDFVRPLEIFHNNTHIL
                     LRGESGLGKTQYACAHFLNPLVVPGSDLERIQRLTPDHDGVVFDDCDLRHFTPTQVIG
                     ILGRDNTPRAFPCRWHNKAIPDGLRCIFTINDVYPFYNDTCSVGQRRAIDSRVRVVDL
                     TERLFVVQQ*"
     CDS             complement(1252..2673)
                     /codon_start=1
                     /product="Putative CruV Capsid Protein"
                     /translation="MAFAITPYRAPEAQPPLDTAVTYGALRKYLKNDSYNPAIAKAVR
                     GSGGRYKRRAYGRYQPRYRRSSRPYARRRSYRGYGSYSMDGGANFGTRWGGHYGARAG
                     ERIGGAVQGLVNRIGGFGDYSIKANALMPGEGGEIVNPNTHGGTVYRGSEFLGDVLSG
                     APNTFSLQNFFINPALEITFPKLAQFLANYDQYVIEGLYFEYRTMSADALNSTNTALG
                     QVIMCCNYNSLAPNFPTKAEMEEYDGGVSVKPSCSCKFFIECARSESPLDVLYTRSGG
                     IPTGADQRMYDLGNFQIATNGLQGTNVNCGELWVTYQVAGLKPKIFSGLGNYNSAFEY
                     TENSFTNSAPLGSGTTTVLYQAPTGNIGWFLDAAGTTITFPDSPLRQTYGVYIGWNGT
                     AAVTVTYPSITLLNASQTSSVNVPSGGTTASRMSMSFEVRTGPCNDINPASFTLGAGG
                     TLPTGTQVLTVKIWQIPNLVIPG*"
ORIGIN
        1 tactattact ctatgcccac cacatccatc catccacacg tgcgatgatg gatttagacc
       61 cgcgcattgt ttatgattat tcgaccagca ggtcggaatt aacgggcgta ctggcgtccg
      121 aaccgccggt ccaaggagga tccgagcagg gtagccccgc tcgggaggat ccgaattggc
      181 ttgacgaggc tcaagccttg tctgagcgtt tagctgtcga ccctgctgag caagtcttga
      241 ctccgcatac cgctggggat cgcactcctt tggcggtcca ttggttattt actctaaata
      301 actacaccga acaagatgag gctgcgttta gtgccaatct aaaccttttc aaaggcgcct
      361 gctatggtcg tgaagttggg gaacagggga ccccacattt acaagccttt gtatcctttg
      421 cgaagcaatc ccgtggatca cagttggcca agatatggcc acgcgcacat atggcaattg
      481 ccaaggcctc gccttggatc aattggcttt actgttcgaa gggtgaacaa cccaagaccg
      541 aatggacggc ctaccgtgag aaggggccga cttatggaag gaacgctcag ttcttccact
      601 ttggaacgcg acctgtgtgt ccaaagaagg gtagtaatcg tcgtgatctt cacgatgtta
      661 ctgcagaatg ttatgctatg cttgaagacg gtaccattca atccgctatg gatcatttga
      721 aggtacaccg tcctgctgat catgcgaagt ataatgtaca gtatcgcaat gcgtttactg
      781 ctgagacgaa gccgaagact tttcagcata tttatgtcga cacagacttt gttcgtccct
      841 tggagatctt tcacaacaac acgcatattc ttcttagggg agaaagtggt ttaggtaaga
      901 cacagtatgc ctgtgctcac tttctcaatc ctttggtcgt tcctggatca gatctggaac
      961 gtattcaacg cctcactccg gatcatgatg gcgttgtatt tgacgactgt gatcttagac
     1021 attttactcc aactcaagtt attggaatac ttggtcgaga taacactcca agagcctttc
     1081 cttgtcgttg gcataacaag gctatccctg atggtcttcg ttgtattttt actatcaacg
     1141 atgtttatcc tttttataat gatacatgca gtgttggcca acgtcgggcc attgatagtc
     1201 gtgttagggt cgttgatctt actgaacgtt tatttgtagt acaacaataa attaaccagg
     1261 aatgactaag tttggaattt gccaaatctt taccgttagt acttgtgttc ctgttggtaa
     1321 tgttcctcca gcaccgaggg taaaggatgc tgggttgatg tcattgcacg gaccagttct
     1381 aacttcgaat gacattgaca ttctgcttgc tgtagttcca cctgatggta cgtttaccga
     1441 cgatgtttgc gacgcattca gtaacgttat actcgggtac gttacggtta ctgcggcagt
     1501 tccattccat ccaatgtata cgccatacgt ttgtcgtagt ggcgagtctg ggaacgtgat
     1561 tgtggttcct gctgcgtcta ggaaccaccc aatgtttcct gttggtgcct ggtaaagcac
     1621 tgttgttgtt ccacttccta atggggcaga gttggtgaat gaattttctg tgtactcaaa
     1681 tgcactgttg tagtttccga gtccggaaaa gatttttggt ttcagtccgg caacttggta
     1741 ggtgacccaa agttctccgc agttcacgtt tgttccttgt agaccatttg tagcgatttg
     1801 gaagtttccg agatcgtaca ttctctggtc agcaccggtt gggatgcctc cacttctcgt
     1861 gtagagaaca tctaacgggc tttcacttct tgcgcattcg atgaagaact tgcacgaaca
     1921 tgatggtttc acggacacac ctccgtcata ttcttccatt tctgcttttg ttggaaagtt
     1981 gggtgctaaa ctgttgtagt tgcagcacat gatgacttga ccaagtgcag tgtttgttga
     2041 gttgagcgcg tctgcactca ttgttctgta ttcaaaataa aggccttcga tcacgtattg
     2101 atcataattg gccagaaatt gtgctagttt cgggaacgtg atttcgagag ctgggttgat
     2161 gaagaagttt tgtagcgaaa acgtattagg tgctccgctc agaacatctc ccaggaattc
     2221 gcttcctctg tagaccgttc ctccgtgggt gttcgggttg acgatttctc ctccttctcc
     2281 cggcataagc gcattagctt tgatgctgta atccccgaac ccgccaatgc ggttgaccag
     2341 accttgaaca gctcctccga tacgctctcc ggcacgagcg ccgtagtgtc ccccccagcg
     2401 agttccaaaa tttgcaccgc catccatgct atatgatcca tatcctcgat agcttcttcg
     2461 acgagcgtac ggacgtgagg atcttctgta ccttggctgg taacgtccat aagcccttcg
     2521 cttgtatcga ccgccacttc cgcgcaccgc tttagcgatg gctggattgt agctgtcgtt
     2581 tttgaggtac tttctaagtg ctccgtaggt aactgcggtg tcgagaggag gttgtgcttc
     2641 tggtgccctg tagggcgtga ttgcgaaggc catggggttg atattgcttc tttcgatatc
     2701 aatgttttct tcgtcggcga gtctgattct cgcgttctcc ctgttgtcag gtaagtcccc
     2761 gtctgcgcgg cgcttcgtgg taactcgcgt cgcataaacg ctatcacaaa ttcacaaaaa
     2821 ataatgtgaa tttttaagga tgacccctga caaaacaaat ttttataaac tttgtgaatt
     2881 cttgggaggc cgaaggccga cctgctcaca ggggaactct tccagggggt ggaggccgaa
     2941 ggccgactct tctaactttg tgaattttta gttcactaat tttttaattc ttgggatccg
     3001 agccgaaggc gaggatgggg tgggcatagt
//
LOCUS       CruV-132                3030 bp    DNA     circular     24-FEB-2020
DEFINITION  Crucivirus-132 isolate 120_1072_SvL1_3295.
ACCESSION   CruV-132
KEYWORDS    .
SOURCE      Crucivirus-132
  ORGANISM  Crucivirus-132
            Unclassified.
REFERENCE   1  (bases 1 to 3030)
  AUTHORS   de la Higuera,I., Kasun,G.W., Torrance,E.L., Pratt,A.A.,
            Maluenda,A., Colombet,J., Bisseux,M., Ravet,V., Dayaram,A.,
            Stainton,D., Kraberger,S., Zawar-Reza,P., Goldstien,S., Briskie,J.,
            White,R., Taylor,H., Gomez,C., Ainley,D.G., Harding,J.S.,
            Fontenele,R.S., Schreck,J., Ribeiro,S.G., Oswald,S.A., Arnold,J.M.,
            Varsani,A., Enault,F. and Stedman,K.M.
  TITLE     Direct Submission
  JOURNAL   Submitted (24-FEB-2020) Biology, Portland State University, 1719 SW
            10th Ave, Portland, Oregon 97201, USA
COMMENT     Bankit Comment: ALT EMAIL:ide@pdx.edu
            Bankit Comment: TOTAL # OF SEQS:451
            Bankit Comment: GAP: est. length - alignment

            ##Assembly-Data-START##
            Assembly Method       :: MetaSPAdes v. 3.12; IDBA-UD v. 1.1.1;
                                     MEGAHIT v. 1.0.3
            Sequencing Technology :: 454; Illumina
            ##Assembly-Data-END##
FEATURES             Location/Qualifiers
     source          1..3030
                     /organism="Crucivirus-132"
                     /mol_type="genomic DNA"
                     /isolate="120_1072_SvL1_3295"
                     /isolation_source="Water"
                     /country="Norway"
                     /collection_date=2012
     CDS             1..1080
                     /codon_start=1
                     /product="Putative Replication Associated Protein"
                     /translation="MPQHLVDAINKLGKKVNSPKKRRIIRCAKATPELKEPEEVLDHD
                     FPEPDEDEIMIPNFKARRYAFTAWYEDKDLDTKKILEAFSAVACKYWILGYEKTKEGV
                     KHIQGYVEFVNTITFPRTGIPCHIAKAIKNKVGNIKYCKKAKSKKSKDDWICMPEMEN
                     YENNGISQGKRTDITTIRDSTLDGESVRSMLLNDKMKNAQSFYIARECQRLLPVLQDC
                     KPRVLWFWGKTGAGKSYSAFQECAKADGDTWKSMNSLKWFDGYDGQENVIIDEFRGDF
                     CTFHFLLQLLDEYPLMVEIKGGVTSWRPKMIIITSNRTPEEIYENRTQESIDQLLRRI
                     DEIVEFKRKSTPKTPIRRIVEEPNC*"
     CDS             complement(1558..2868)
                     /codon_start=1
                     /product="Putative CruV Capsid Protein"
                     /translation="MAQRIPYADFKKLAPADKKRLASAVRKSSSSGHPPSSRTITISG
                     RGGYKATVKRNPARNFRNSAVKAVGAGAEFLADKAMSLAGFGDYTISANSVMNAMTSS
                     GPPVMKSTRNREFVVRHREFIQDVYTGPVAGVFNLESFKINPGQVSTFPWVSQIASNF
                     EQYRVEGMVFEFKSTSGDALTSTNTAIGTVIMATDYNAASPNFSNKQQMENHEFANSC
                     RQSNNMLHPIECKRGLTPVSELYIRTGAVPSGQDPRLYDLGNFQVATVGSQAANINIG
                     ELWITYEVSFLKPRLGATAISDHVYSSSGVTNAAPFGPVASQVFRTNSNLGIQLSAGA
                     TITFPSYISQGSYLVSYHVKGTSGGSASCSVGLTTNCDAYFLWDNGANRIIESADLAL
                     TYILTFNVFITGPNAVVTLSPSGLPTGTVTADLIVTQLNSNIQD*"
ORIGIN
        1 atgccccaac acctcgtaga tgcaattaat aaattaggga aaaaagtaaa ttctcctaaa
       61 aaaagaagaa ttattaggtg tgctaaagca acgccagaat taaaagaacc cgaagaggtt
      121 cttgatcatg attttcccga gcctgacgaa gatgagatta tgatccctaa cttcaaggct
      181 cgacgttatg cctttactgc gtggtacgaa gataaagacc ttgacactaa aaaaatccta
      241 gaagcctttt cggctgtagc ctgcaagtac tggatacttg gatatgaaaa aacaaaagaa
      301 ggtgtaaaac atatccaagg ttatgtggaa tttgttaata ccataacatt cccgcgtacg
      361 ggtattcctt gtcatatcgc taaagctatt aagaacaagg taggtaatat caaatattgt
      421 aaaaaggcaa aatccaaaaa atctaaagat gattggatct gtatgcctga aatggaaaat
      481 tacgaaaata atggaatttc ccaaggtaaa agaacagata taacaactat tcgtgattca
      541 acccttgatg gtgaatccgt cagaagtatg cttctgaatg ataagatgaa aaatgcgcaa
      601 agcttttata ttgcgcgtga gtgtcagcgc ttacttccgg tattacaaga ctgtaaacct
      661 cgtgtccttt ggttttgggg caaaactggc gctggtaaat cttattcagc tttccaggaa
      721 tgcgccaagg cagatgggga cacctggaaa tccatgaact ccctaaagtg gtttgatgga
      781 tatgatggac aagaaaatgt catcattgat gaatttcgtg gagatttctg tacctttcac
      841 tttctcttac aattacttga cgagtacccg cttatggttg aaatcaaagg aggtgtaaca
      901 tcttggagac caaagatgat tataatcacc tctaatagaa ctcctgagga aatctacgaa
      961 aatcgaactc aggagtcaat cgaccaatta cttcgtagga tcgatgagat cgtagaattt
     1021 aaaagaaaat ctactcctaa aacaccaatt cgtagaattg ttgaagaacc aaactgttaa
     1081 cgtctacgcc gggttaccct aaatccagca cttcggctgg acccaactca acaattagtt
     1141 tggaaaggat ttttaaaaaa aatcactgaa aaaatttttt ttaaaaattt tattgaactt
     1201 ctgacttcag ctgaagcaga agcagaagtc gactagtaat aataacgccc gtaggttcct
     1261 acggcctcgt cgtcgacttc ttttttccct gcgggacaac gtgtatatat atacacgatt
     1321 attatattaa gctgttagat aacagactta aatgctaccg cgcttcgctt ggagataata
     1381 tttataaaga acgcttatcg tcatgcgata agatggtagg gggggctccc gccccccatc
     1441 tcgtcgctgg acacgacgag gatccgctac agatcaaaat aatatcgggt ctatccggtc
     1501 ttcgacctcc gattttatcg agtacatgga aagtgttttt aaaaacacac aaatatatta
     1561 atcttgaata ttagaattca attgagtgac aatcaagtca gctgtgacag tgccagtagg
     1621 caacccagaa ggggataaag ttacaacggc attaggacct gtaataaaaa cattaaatgt
     1681 taaaatataa gtaagtgcta aatcagcact ctcaataata cgattagcac cattatccca
     1741 aaggaaatag gcatcgcaat tagttgttaa accaacggaa catgatgcag agccaccaga
     1801 agtgccctta acatggtaac taacaagata agatccctga gaaatataag agggaaatgt
     1861 aatagtagct ccagcactaa gctgaatacc caaattagaa ttggtacgaa agacctgact
     1921 agcaacagga ccaaacggtg cagcgttagt aactccagag ctagaataaa cgtgatcaga
     1981 aattgccgta gctccaaggc gcggtttcaa aaatgaaact tcataagtaa tccaaagctc
     2041 accaatatta atattagcag cttgtgaacc tactgtggca acttgaaaat tacctaaatc
     2101 ataaagacga ggatcttgtc ctgacggaac tgcccctgta cgaatataca attcggaaac
     2161 aggggttaaa cctcgcttac actcgattgg atgtaacatg ttgttacttt gacggcatga
     2221 attggcgaat tcatggtttt ccatctgctg cttattggaa aaattaggtg atgcagcgtt
     2281 gtaatctgta gccataataa cagtgccaat tgcagtgttg gtagatgtca atgcatcacc
     2341 ggaagtggat ttgaactcaa ataccattcc ttcaacacga tattgctcga aattactcgc
     2401 aatctgagaa acccaaggaa aagttgaaac ttgacctggg ttaattttaa aactctccaa
     2461 attaaaaact ccagctactg gacccgtata aacatcttga ataaattctc gatgacgaac
     2521 aacaaactca cggtttcttg tagacttcat aacaggagga cccgatgaag tcattgcatt
     2581 cataactgaa tttgctgaaa ttgtgtaatc gccaaaacct gctaaagaca tagctttgtc
     2641 ggccaaaaat tcggcaccag caccaacggc ttttaccgcc gaattacgga aattacgtgc
     2701 tggatttcgc ttgacagtag ctttatagcc gccacgacca ctaatagtaa tcgtgcgact
     2761 agatggagga tgtccagagg atgaactctt acgaactgca gaagccaatc gtttcttgtc
     2821 tgcgggtgcg agtttcttaa aatctgcata tggaatgcgc tgcgccatgg taatttttct
     2881 acagaaaaaa tttttgcaga ttttttttca tttttttaaa taaaggtttt aaaaaaaaac
     2941 ccaaaaaata ttttccggaa atcaaaagaa agcaattcta aatttgctat gagtgattcc
     3001 gaagaattct acgacgaacc aagtggagct
//
LOCUS       CruV-133                3040 bp    DNA     circular     24-FEB-2020
DEFINITION  Crucivirus-133 isolate BS_350.
ACCESSION   CruV-133
KEYWORDS    .
SOURCE      Crucivirus-133
  ORGANISM  Crucivirus-133
            Unclassified.
REFERENCE   1  (bases 1 to 3040)
  AUTHORS   de la Higuera,I., Kasun,G.W., Torrance,E.L., Pratt,A.A.,
            Maluenda,A., Colombet,J., Bisseux,M., Ravet,V., Dayaram,A.,
            Stainton,D., Kraberger,S., Zawar-Reza,P., Goldstien,S., Briskie,J.,
            White,R., Taylor,H., Gomez,C., Ainley,D.G., Harding,J.S.,
            Fontenele,R.S., Schreck,J., Ribeiro,S.G., Oswald,S.A., Arnold,J.M.,
            Varsani,A., Enault,F. and Stedman,K.M.
  TITLE     Direct Submission
  JOURNAL   Submitted (24-FEB-2020) Biology, Portland State University, 1719 SW
            10th Ave, Portland, Oregon 97201, USA
COMMENT     Bankit Comment: ALT EMAIL:ide@pdx.edu
            Bankit Comment: TOTAL # OF SEQS:451
            Bankit Comment: GAP: est. length - alignment

            ##Assembly-Data-START##
            Assembly Method       :: MetaSPAdes v. 3.12; IDBA-UD v. 1.1.1;
                                     MEGAHIT v. 1.0.3
            Sequencing Technology :: 454; Illumina
            ##Assembly-Data-END##
FEATURES             Location/Qualifiers
     source          1..3040
                     /organism="Crucivirus-133"
                     /mol_type="genomic DNA"
                     /isolate="BS_350"
                     /isolation_source="Water"
                     /country="New Zealand"
                     /collection_date=2012
     CDS             complement(1..1332)
                     /codon_start=1
                     /product="Putative Replication Associated Protein"
                     /translation="MAPKFKNWCFTLNNYQTHLNIEAELASNLTAEVPKSIEELYALK
                     FYDLCGGATKVKYIVMGFEKGEEEETPHIQGYMDFTNGRYVGGLKKLQKQTHWEPRGG
                     SWLDASNYCKKGTQSKKEFDLSHEKGENFGAGAIFYEYGTPNEQGKRYDIEDMKASLT
                     KDCKIRNVINTVTNVQQIKFAESYIKYMEKKRTWAPIVIWRYGATSTGKSETSHSDFL
                     ELSNGNSEDIYHLGSGLKWWDGYDAHTYVMIDEFRGSHGKYSDLLRLLDKYPYQIETK
                     GGSRQMLATHIYITSCYHPYEVYKVGEKMNESANQLVRRCTKIFLHRKDLPPLDVTLM
                     TYSETLALYDASTVDTVGIPTFPVITKDTPVHFTTRPSKTVVLTPGRGLVPGTKDGSG
                     TRVKGVILESPEPVVPPSAPLTKREIFTPTFGKLERVFTRRSKDVFGRDAE*"
     misc_feature    731..749
                     /note="stem-loop"
     misc_feature    736..744
                     /note="nonanucleotide"
     CDS             complement(1425..2783)
                     /codon_start=1
                     /product="Putative CruV Capsid Protein"
                     /translation="MSKVSKKTVSRKSEPRRKKSSVEKTFHRFRKNYNKEYMFPGVGR
                     EIGGTIGSFAGPTGNLIGKAIGHGAHSLMKTVTGFGDYQVKGNSLMYNKDAVPEFTNN
                     PRCTIIAHREYIGDVFSGPTLSAGATTFNATSFRINPGVAGTFPWLSSVAQNYEQYVI
                     QGMIFEFKTNSATAVSSTNTALGTVIIASQYNSLAPEFNSKQQMENYEFCQSSVPSMS
                     VAHPIECDPHQTQCGGIFNVWDPVFSTGDIRLYDIGRFTIATQGMQAVGINLGELWVT
                     YKICMLKPRLTQIAYGESDVWELEVASTTSSAPFGHPVNALQKSLGIPISSLTNTNTF
                     TISPNFTGYIICLWTLVNPNVESTDKSMLGSLVAGSGSTLVPVAALGDGNVNLNLMAV
                     ENSGFNANGQSSVIFFCPGGPNQTFTNTTANFNASVWLPELNNNTMIIASINWNNSIN
                     PA*"
ORIGIN
        1 ttattccgcg tctctaccga atacatcttt ggaccgtctt gtaaatacac gttcgagttt
       61 accgaacgta ggtgtaaaaa tctctcgttt cgtcaagggg gctgagggag gaacaactgg
      121 ctccggactc tctagtatta cccccttgac cctggtgcca gagccatctt tggtgccagg
      181 aacgagtccc cgccccggag ttagcacgac ggttttagag ggacgagtag tgaaatgtac
      241 tggggtatct tttgtgataa ccggaaatgt aggaattcct acagtgtcca cagtactagc
      301 gtcatataga gctagtgttt cactgtatgt cataagtgtc acgtctaaag gtggtaagtc
      361 ttttcgatgg agaaatattt tcgtacatcg tctgactagt tggtttgcac tttcgttcat
      421 tttttcacca actttatata cctcgtatgg atgatagcaa ctcgtaatgt atatgtgagt
      481 agctagcatt tgcctagagc cacctttagt ttcaatttga tacgggtatt tatctagtag
      541 tctgagtaag tcactatatt ttccgtgact acctctaaat tcgtcaatca ttacgtaagt
      601 atgggcgtca taaccgtccc accattttaa accactacct aggtggtaaa tgtcttccga
      661 atttccatta cttaattcta ggaaatccga atgggaagtt tcagatttcc cagtactagt
      721 tgcaccgtat ctccaaatta ctattggagc ccaagttctt tttttttcca tgtattttat
      781 atacgactca gcaaatttaa tttgctgtac gttagtgact gtattaatta cgtttctaat
      841 tttacagtct ttagtcagcg aagctttcat gtcttcgatg tcgtaacgtt taccttgttc
      901 gttaggagta ccatattcat agaatatggc tccggctccg aagttctcac ctttttcgtg
      961 tgataagtca aactcttttt tactctgagt accttttttg cagtaattac tagcgtctaa
     1021 ccaagagcca ccccttggtt cccaatgagt ttgtttctgt aattttttca aaccgcctac
     1081 gtatctgcca ttcgtgaagt ccatatatcc ctgtatatgg ggagtttctt cctcttcacc
     1141 tttttcgaat cccattacaa tgtatttgac tttcgttgca ccaccgcata ggtcgtagaa
     1201 ttttagagcg tacaattcct caattgattt aggaacttcg gccgttaggt tagatgctaa
     1261 ttctgcctct atatttaagt gagtttgata gttgttgagc gtgaaacacc aatttttgaa
     1321 ctttggagcc atgttaattt ccaaaattaa aaatttcgaa ttacgatgtc gttttttttt
     1381 gaaattttca aaataatttg aaaatctgaa ctttactttt cggattaagc aggattaatt
     1441 gaattgttcc aattaatact tgcaataatc attgtgttat tgtttaattc gggtagccat
     1501 acactggcgt tgaagtttgc tgtagtatta gtaaatgtct gatttggtcc tcctggacaa
     1561 aagaagatta cacttgattg accatttgcg ttaaatcctg aattctctac tgccattaag
     1621 tttaaattta catttccgtc tcctagtgca gctactggaa ccaaagtgct tcctgagcca
     1681 gctactaacg agccaagcat tgatttatca gtgctttcaa catttggatt tacaagagtc
     1741 caaaggcata taatatatcc tgtgaagtta ggacttatag tgaacgtatt agtgtttgtt
     1801 aaagaactaa tcggaatacc taacgatttt tggagagcgt taacggggtg accgaaggga
     1861 gctgagctag ttgtactagc tacttccaat tcccacacgt cgctttctcc gtaagcgatt
     1921 tgagtaagtc taggttttaa catacaaatt ttgtatgtta cccataattc ccctaaattt
     1981 ataccaactg cttgcatacc ttgtgttgca atagtaaacc tacctatgtc ataaagtcta
     2041 atgtctcctg ttgaaaatac agggtcccaa acattaaaaa tgcctccgca ttgggtttgg
     2101 tgagggtcac actctatagg gtgtgcaacc gacattgatg gcactgaaga ttggcaaaat
     2161 tcataatttt ccatttgttg tttagagtta aattctggcg ccaaagagtt atattgcgaa
     2221 gcaattataa cggtacctaa tgcagtattg gtacttgaga cggcagtagc actgttagtt
     2281 ttaaattcaa atatcatacc ttgtataaca tattgttcgt agttttgagc gacacttgag
     2341 agccaaggaa aagtgccggc cacgcccggg ttaattctga atgaggtagc attaaatgta
     2401 gtagctccgg ccgatagtgt aggcccacta aatacatctc ctatatattc tctatgagca
     2461 attatagtac atctaggatt attagtgaat tccgggacag cgtctttatt atacattaga
     2521 gaattacctt ttacttgata atctccaaaa cctgttacag ttttcattaa actgtgtgcg
     2581 ccgtgaccaa ttgctttacc aattaggttt ccagttggcc cagcgaagga accaatagtt
     2641 cctccaattt ctctacctac tccaggaaac atatattctt tattgtaatt tttcctaaat
     2701 ctgtgaaatg ttttttcaac agaagacttt ttacgtcttg gttcagattt tctggaaaca
     2761 gtttttttgg atactttaga cattttatat agttttatat aattttagat tatttttaaa
     2821 acctagaatt taatagtctg attttgtcta tttcagcatg cacacaggcg cgaagcgccg
     2881 ttacatagat ttgtaaatca gcaagcgtta gcgcgctagt acaagcgtta gcgcgttagt
     2941 acaagcgtta gcgcgtagca caagcgaagc gcgtagttac atatattgcg aagcttaatt
     3001 acagataagt actgagtact tatttgttgg agcggaagta
//
LOCUS       CruV-134                3049 bp    DNA     circular     24-FEB-2020
DEFINITION  Crucivirus-134 isolate 001799_virusbaton.
ACCESSION   CruV-134
KEYWORDS    .
SOURCE      Crucivirus-134
  ORGANISM  Crucivirus-134
            Unclassified.
REFERENCE   1  (bases 1 to 3049)
  AUTHORS   de la Higuera,I., Kasun,G.W., Torrance,E.L., Pratt,A.A.,
            Maluenda,A., Colombet,J., Bisseux,M., Ravet,V., Dayaram,A.,
            Stainton,D., Kraberger,S., Zawar-Reza,P., Goldstien,S., Briskie,J.,
            White,R., Taylor,H., Gomez,C., Ainley,D.G., Harding,J.S.,
            Fontenele,R.S., Schreck,J., Ribeiro,S.G., Oswald,S.A., Arnold,J.M.,
            Varsani,A., Enault,F. and Stedman,K.M.
  TITLE     Direct Submission
  JOURNAL   Submitted (24-FEB-2020) Biology, Portland State University, 1719 SW
            10th Ave, Portland, Oregon 97201, USA
COMMENT     Bankit Comment: ALT EMAIL:ide@pdx.edu
            Bankit Comment: TOTAL # OF SEQS:451
            Bankit Comment: GAP: est. length - alignment

            ##Assembly-Data-START##
            Assembly Method       :: MetaSPAdes v. 3.12; IDBA-UD v. 1.1.1;
                                     MEGAHIT v. 1.0.3
            Sequencing Technology :: 454; Illumina
            ##Assembly-Data-END##
FEATURES             Location/Qualifiers
     source          1..3049
                     /organism="Crucivirus-134"
                     /mol_type="genomic DNA"
                     /isolate="001799_virusbaton"
                     /isolation_source="Water"
                     /country="France"
                     /collection_date=2013
     CDS             1..1329
                     /codon_start=1
                     /product="Putative CruV Capsid Protein"
                     /translation="MPAVKKTVKRPTRVYKAKPRAKPRARRPRRVDYDEDVAIDMGVR
                     SPGMLSKGGSALGTAAGGALGGPAGAAIGNFLGGKLGHLVEHVTGFGDYSISSNSILQ
                     GGLSPPQVINSIDKGGVIVRHREYIRDIPATVDFTSLKFPLNPGQASTFPWLSQMAAN
                     FEQYRFRGLLFEYISTSSDAILSSSTSTALGTVSIATDYDVLDIAYTDKRTMLNAEFS
                     TSSKPSMTFIHPIECKKSISPMALQYIRTTLNFPANSDARMYDLANTFVATEGMQAAA
                     GNVGELWVTYEVELYKQQSNFIAYTDHWQMSNMTSASLLGTVQSSIDAGGSIGGAVNG
                     PGTAYSFPPELSSGKFLCYYYAAGSAAATLGNLVPTITKGSLAALWRTDNWTSVQIPA
                     PGVSSAFVSLAFIVQINAPGCIITFPTAVIPTTSVFGDFYITQIANSIVD*"
     CDS             1962..2984
                     /codon_start=1
                     /product="Putative Replication Associated Protein"
                     /translation="MSSASLASPESEESFQANLSERNYRKKKFSEGLAGSSITPHGPF
                     QNFIFSIESRIMLKSRGYCFTINNYTDVILSQLMELFETGVATYLVVGFEVAPETGTR
                     HIQGYMHFGNPRSMSGIHKLVSNMHLEAPRATGAKYTLRYLYCMKGDQPKEEWESMSD
                     KGPNFGRNSDFIEYGVRPQDGISKVTTLITTAILNGSTYEDVFAEYPQYCLQHSRNVK
                     AFITDASVYKQPQLFTIKETPTLHGDIDHNFPDRRIHWVDIDNFYELEVEGDTSQDVV
                     VALVGLPNRTYQLWGYGKPITYKYGYECKKLRCAQLVLVSPRDPTKEFCYNMYKNLTP
                     GEYLKN*"
     misc_feature    2976..2998
                     /note="stem-loop"
     misc_feature    2982..2990
                     /note="nonanucleotide"
ORIGIN
        1 atgccagctg ttaaaaagac tgttaaacga cccacgagag tgtataaggc taagccacgt
       61 gctaaaccac gtgctagacg tccacgtcgt gtcgattatg acgaagatgt agccattgat
      121 atgggtgtta gatcacccgg tatgttatca aaaggaggct cagcgttagg tacagctgca
      181 ggaggcgctt taggcggacc tgcaggtgca gctatcggca attttttggg tggaaagctc
      241 ggacatcttg tagaacatgt cactggtttc ggtgactata gtatttcttc aaattctatt
      301 ctacaaggtg gtcttagtcc accacaagtt atcaatagta ttgataaggg tggagttatc
      361 gtaagacatc gagaatatat tcgagatatt ccggcaactg tcgatttcac atctttgaag
      421 ttccctttaa atccaggaca agcttcgact tttccttggt tatctcagat ggctgccaat
      481 tttgagcagt atcgatttag agggttgtta tttgaatata tatcaacttc atcagatgca
      541 atattgtctt caagcacatc tactgcctta ggtacagtat ctatagctac agactatgat
      601 gtacttgata tagcttatac agataagcga actatgttaa atgccgaatt ttcgacttct
      661 tctaaaccgt ctatgacgtt cattcatcct attgaatgta agaagtcgat tagtcctatg
      721 gcattacaat atatccgtac tactctgaat tttccagcaa attcagatgc tcgtatgtat
      781 gatttagcaa atacatttgt tgctactgaa ggtatgcaag ctgctgctgg taatgtcggt
      841 gaattatggg ttacatatga agtcgaatta tataagcaac agtctaattt cattgcatat
      901 acggaccatt ggcaaatgtc aaatatgaca agcgcatctt tattgggtac tgtacaatct
      961 agtattgatg caggtggttc aattggaggt gctgtcaatg gtcctggtac agcatatagc
     1021 ttcccgcctg aattatcttc aggtaagttt ctttgttatt attatgcagc tggttcagcg
     1081 gcagcaacgt taggtaatct tgtacctact ataacaaaag gatctttggc agctttatgg
     1141 cgtactgata attggacaag tgtacaaatc cccgctccag gtgtaagctc tgcttttgtg
     1201 agtttggcat ttattgtaca aataaatgca cctggatgta ttatcacatt tcctactgcg
     1261 gtaataccaa caactagtgt ttttggagat ttttacataa cacagatagc taattctatc
     1321 gtcgattaat tttattgatt gtaaagttat tttgaagaaa taattttata accgttttaa
     1381 tatttttata aataattcct ttgattaggg tatagacata acccttgttc catgacgtca
     1441 gcaggacaca tccgatttgg gtttaggatt tactacgttt ctgagttcgt ataacatcta
     1501 taacaatgga ggggtgtgat gtaatggagc gacgaggtgc ggaggcgtaa gggaggagga
     1561 cacaggacga ctgagagtga tgatcgagga gatatatgag gatgaagtgg tgtgagaagg
     1621 ttgcggagtt attggaattt ttacggacat agaacggaga ctatctggcg gcgccccggc
     1681 ggtgacatag aggcgaggtt atctgggagt agaaatggaa ataacgatca accgggcctg
     1741 taaggatcga aaaccgctac agacgaaata tagcgacgct ggaagaaccg aaggaggcat
     1801 ttggccgagc gaccgaaata acgcagcgcc gatttgcgca atggaagaac caccccggaa
     1861 tgacttcctt tataggtgct tatatgaatc tcagagaacg gttcctgtaa tatccgtata
     1921 gaacacagag agtatgtgcg cctgcggcta ggacggcatt tatgtcgtcc gcgagcttgg
     1981 cgagccctga aagtgaggag tcatttcagg ctaatctttc tgaaagaaac tatagaaaaa
     2041 aaaaattttc tgagggcctg gcggggtctt ctattacccc ccacggcccg ttccaaaatt
     2101 ttattttctc tatagaaagt agaattatgt taaaatcacg aggttattgt tttacaatca
     2161 ataattatac cgatgttatt ctttcccaac tcatggagct gtttgaaact ggtgttgcca
     2221 catacttagt tgttggcttc gaggttgctc ctgaaactgg gactcgtcat atccaaggat
     2281 atatgcattt tggtaatccc cgttcaatgt ctggtataca taaattagtt tctaatatgc
     2341 atttagaggc tccacgtgca accggggcaa agtatacttt gcgttatctt tactgtatga
     2401 agggcgacca accaaaagaa gaatgggagt ctatgtccga taaaggacct aatttcggaa
     2461 gaaattccga ttttatagag tatggtgtac gtccacagga cggtatcagt aaagtaacaa
     2521 cactcattac aacagcaata ctgaatggtt ctacatacga agacgtattc gctgagtatc
     2581 ctcagtattg tttacaacat tctcgtaatg ttaaggcgtt cataacagat gcatctgttt
     2641 acaaacaacc ccagttgttt actattaaag aaacgcctac actccatggc gatattgatc
     2701 acaattttcc agaccgtcgc atacattggg ttgatataga taatttttat gaattagagg
     2761 ttgaaggtga tacatcgcaa gacgtagtcg tagcacttgt tggattacct aaccgtactt
     2821 accaattatg gggttatgga aaaccaatta cttataaata tggatatgaa tgtaagaaat
     2881 tgcgttgtgc tcaattagta ttagtttctc ctcgggaccc aacgaaggag ttttgttaca
     2941 acatgtataa gaatttgact ccaggagaat atcttaaaaa ctaatactac cagtttttcc
     3001 acctttttaa tataataatt tctttactaa actttagtat agtaaagaa
//
LOCUS       CruV-135                3052 bp    DNA     circular     24-FEB-2020
DEFINITION  Crucivirus-135 isolate 001797_virusbaton.
ACCESSION   CruV-135
KEYWORDS    .
SOURCE      Crucivirus-135
  ORGANISM  Crucivirus-135
            Unclassified.
REFERENCE   1  (bases 1 to 3052)
  AUTHORS   de la Higuera,I., Kasun,G.W., Torrance,E.L., Pratt,A.A.,
            Maluenda,A., Colombet,J., Bisseux,M., Ravet,V., Dayaram,A.,
            Stainton,D., Kraberger,S., Zawar-Reza,P., Goldstien,S., Briskie,J.,
            White,R., Taylor,H., Gomez,C., Ainley,D.G., Harding,J.S.,
            Fontenele,R.S., Schreck,J., Ribeiro,S.G., Oswald,S.A., Arnold,J.M.,
            Varsani,A., Enault,F. and Stedman,K.M.
  TITLE     Direct Submission
  JOURNAL   Submitted (24-FEB-2020) Biology, Portland State University, 1719 SW
            10th Ave, Portland, Oregon 97201, USA
COMMENT     Bankit Comment: ALT EMAIL:ide@pdx.edu
            Bankit Comment: TOTAL # OF SEQS:451
            Bankit Comment: GAP: est. length - alignment

            ##Assembly-Data-START##
            Assembly Method       :: MetaSPAdes v. 3.12; IDBA-UD v. 1.1.1;
                                     MEGAHIT v. 1.0.3
            Sequencing Technology :: 454; Illumina
            ##Assembly-Data-END##
FEATURES             Location/Qualifiers
     source          1..3052
                     /organism="Crucivirus-135"
                     /mol_type="genomic DNA"
                     /isolate="001797_virusbaton"
                     /isolation_source="Water"
                     /country="France"
                     /collection_date=2013
     CDS             1..1251
                     /codon_start=1
                     /product="Putative Replication Associated Protein"
                     /translation="MKRGDSEEIDLAVDEEIDVVPDNETAEIIEIPPVIENQKNENFS
                     KKGRLRNFVLTIWMVDSFEVESLTGHKHFKYLVFQKEKCPKSGRIHIQAYVEFAEALS
                     YKQVHELLPQPGLPGFQRSWVRQRIGSRKDAREYCMVSTWKGKDKGQIPNTTEEHGEF
                     VADGERTDLTAFTTAILEGKKSWELCEQFPNQMLRHAKHYDKFKQLAVKPLIREPPQV
                     IVICGETDLGKTRFVFDNHPPEDIFRIESEWLWFDGYDGHPVALLDEFHHQWKITKIL
                     QVLDRYPLRVPIKGGFVDWRPNTIYITSNTHPDEWYPNVNMRHKDALMRRIHKIRVLV
                     KQQNAFDLLIKGDKARETASPQEICEEAVPGRRASVQQERVYVPGTGTLTSRPTRGER
                     SAPQVPKKASDIFEALNEYENMLY*"
     CDS             complement(1266..2855)
                     /codon_start=1
                     /product="Putative CruV Capsid Protein"
                     /translation="MSFSMTKRKRSDKPTKSSRKLHRSDTFSSTSDIARYIKPPNLSS
                     LANMATTTSGPMVTDRPGKQVFANKKLAREYAQSKGYKTFTFDGVKYHSSNGVAAKSR
                     SSGGRPKKRRRYSRGYSRGYGQSSSNVSVIQGRGDYSLGKILGTGLGMLGDLCGLGDY
                     RVANNSLVLGGDPPRIRNIKNGEAVIVSHREYLTDILSGGSGSPSTVFNLQNFVLQPG
                     NVATFPWLSDIAACFQEYEFLGVIFEYKSLSSDAVFSAASSGALGDVIMATNYNSAQG
                     NYTNKQEMLESEFSSDAKPSKTIIHPIECAPNLTAVSSHLYVRTGPVPTGQDQRLYDH
                     ANFQIATQGCQANGATLGELWVSYEVAFYKPILGGRASGLDVLTDKFLVVTPAGGVPL
                     GTSQIQTTNGIGGTITIGSVYTFPTYVNQGKFLFVLTAQGTVAANLVEPLITTSVGTA
                     VTSVWASNAGANLNTVAVAPVSGAGVNNANMEMAFIISIGTNAVLPVFVAFGVAGTLP
                     GGGAGNVFGNLLVTEVNSTGT*"
ORIGIN
        1 atgaaaagag gagattcgga agaaatcgac ttagcggtcg atgaggaaat cgatgttgtc
       61 ccggacaacg aaaccgcaga aataattgag atacccccgg ttatcgaaaa tcagaaaaat
      121 gaaaattttt caaaaaaagg ccgcctacgg aattttgttt taaccatatg gatggttgat
      181 tcttttgaag tggagagcct aacaggccac aaacacttca aatatttggt ttttcaaaag
      241 gaaaaatgtc caaaatccgg aaggattcac atacaagcat atgtggagtt tgcggaagca
      301 ctctcttata agcaagtcca cgaattgctg ccacaacctg gattacccgg cttccagaga
      361 agctgggtta gacaacgtat aggatcacga aaggatgccc gtgaatattg tatggtatcc
      421 acttggaaag gaaaggacaa gggacagata ccgaatacaa cagaagaaca cggagagttt
      481 gttgcagatg gagagagaac cgatctcact gcatttacga ctgctatcct ggaaggaaaa
      541 aagtcgtggg aattatgcga acaattccca aatcaaatgt tgaggcacgc aaaacattat
      601 gataagttta aacagctggc tgttaaacct ttaattagag aacctcctca ggttatcgtt
      661 atatgtggag aaactgatct tggaaagaca cgtttcgtgt ttgataatca tcctccagag
      721 gatatttttc gaatagagtc agaatggctc tggttcgatg gctacgatgg acatccagta
      781 gcccttttgg atgaattcca ccaccagtgg aagattacga aaatactgca agtattggat
      841 agatatcctt tgagagtacc tatcaaagga ggatttgtgg attggagacc aaacacaata
      901 tacattacga gtaacactca tccggatgag tggtatccta atgttaatat gagacataaa
      961 gatgctctca tgagacgaat tcataaaatt cgagttttgg tgaaacaaca aaacgctttt
     1021 gatttattga taaaagggga taaagccaga gagactgcct cccctcaaga aatctgcgag
     1081 gaggccgtcc ccggccgacg agcctccgtt cagcaggagc gagtttatgt tcctggaaca
     1141 ggaacattaa cgtcgcgacc aactagaggc gagcgaagcg caccgcaggt gcctaaaaag
     1201 gcatctgata tatttgaagc actcaacgag tacgaaaata tgctttatta gaataataaa
     1261 tttttttaag tcccagtgga gttaacttcg gtgactaata agttaccgaa aacgtttcca
     1321 gcgccaccgc ctggaagtgt tccggcaacg ccgaatgcta caaatactgg taaaacagca
     1381 tttgtgccaa ttgagataat gaaagccatc tccatattgg cattgttgac cccggcacca
     1441 gatacaggcg caaccgctac tgtattcaag tttgcacccg cattacttgc ccataccgag
     1501 gtaacggcag taccgacact tgtcgtaatc agaggttcga cgaggttcgc ggctacagtt
     1561 ccttgtgcag tgagaacaaa caagaatttt ccttgattca cgtaagtagg gaaagtataa
     1621 acacttccga tagtgattgt tccgccaatg ccattggtgg tttgtatttg tgatgttcct
     1681 agaggaacac caccggctgg agtaacgaca aggaacttgt cagtaagaac atctagccca
     1741 gatgctcttc ctccaaggat gggtttgtaa aaagccactt catatgatac ccacaactct
     1801 ccgagagttg ctccatttgc ctgacaaccc tgtgtggcaa tttggaagtt tgcatgatca
     1861 tacaagcgtt gatcttgccc agtcggaaca ggtcctgttc tgacataaag atgactgctg
     1921 actgcggtca aatttggtgc acattcaata ggatgaatga tggtttttga gggttttgcg
     1981 tcagacgaaa actccgattc aagcatttct tgcttgttgg tgtaattccc ctgggctgaa
     2041 ttatagttag tagccataat aacgtcacct agagctccag agctcgctgc gctgaagacg
     2101 gcgtctgaag ataagctttt gtattcgaat atcacgccga gaaattcgta ttcttgaaaa
     2161 catgcagcaa tgtctgaaag ccaaggaaat gtggccacat ttcctggttg caaaacaaag
     2221 ttttgcaagt tgaatactgt gctgggggat ccggatcccc cagatagaat atctgttaga
     2281 tattctctat gagaaacgat gaccgcctca ccgtttttga tatttctgat cctgggtgga
     2341 tcacctccaa gaacaaggga gttgttcgcg actctatagt cgcccagtcc gcagaggtct
     2401 ccaagcatgc cgagacctgt tccgaggatt tttccaaggg aataatcccc tcttccttgt
     2461 ataacggaca cgttactaga actctggcca tagccacggg aatagccgcg agaatagcgg
     2521 cgtctttttt tgggtcttcc cccggaagat ctagattttg cagcaactcc attcgaagaa
     2581 tggtatttga cgccatcaaa agtgaaagtt ttgtatcctt tgctctgagc atactcacga
     2641 gccagttttt tgtttgcaaa cacttgtttg ccaggacgat ctgtgaccat aggtccagac
     2701 gttgttgttg ccatattagc tagtgagctt aaatttggtg gtttgatata gcgagctata
     2761 tcagatgtag aggaaaatgt atcggaacga tgtagtttcc gagatgattt tgtcggtttg
     2821 tccgaccttt tgcgttttgt cattgaaaat gacaaaacgc attttttaca tagtaaaaaa
     2881 ttttagattt ttttgaagtc aaaaaaaata ggatgggtga aggtaattat attacccttc
     2941 accctgtgta atgtgtaatg gcattacgca ttacaaagac ttttggagaa tccgtaaagt
     3001 ctaataattt ttaggaaaaa ttataatttt tgttttcagg gaaaaatcaa aa
//
LOCUS       CruV-136                3054 bp    DNA     circular     24-FEB-2020
DEFINITION  Crucivirus-136 isolate 001794_virusbaton.
ACCESSION   CruV-136
KEYWORDS    .
SOURCE      Crucivirus-136
  ORGANISM  Crucivirus-136
            Unclassified.
REFERENCE   1  (bases 1 to 3054)
  AUTHORS   de la Higuera,I., Kasun,G.W., Torrance,E.L., Pratt,A.A.,
            Maluenda,A., Colombet,J., Bisseux,M., Ravet,V., Dayaram,A.,
            Stainton,D., Kraberger,S., Zawar-Reza,P., Goldstien,S., Briskie,J.,
            White,R., Taylor,H., Gomez,C., Ainley,D.G., Harding,J.S.,
            Fontenele,R.S., Schreck,J., Ribeiro,S.G., Oswald,S.A., Arnold,J.M.,
            Varsani,A., Enault,F. and Stedman,K.M.
  TITLE     Direct Submission
  JOURNAL   Submitted (24-FEB-2020) Biology, Portland State University, 1719 SW
            10th Ave, Portland, Oregon 97201, USA
COMMENT     Bankit Comment: ALT EMAIL:ide@pdx.edu
            Bankit Comment: TOTAL # OF SEQS:451
            Bankit Comment: GAP: est. length - alignment

            ##Assembly-Data-START##
            Assembly Method       :: MetaSPAdes v. 3.12; IDBA-UD v. 1.1.1;
                                     MEGAHIT v. 1.0.3
            Sequencing Technology :: 454; Illumina
            ##Assembly-Data-END##
FEATURES             Location/Qualifiers
     source          1..3054
                     /organism="Crucivirus-136"
                     /mol_type="genomic DNA"
                     /isolate="001794_virusbaton"
                     /isolation_source="Water"
                     /country="France"
                     /collection_date=2013
     CDS             1..1281
                     /codon_start=1
                     /product="Putative Replication Associated Protein"
                     /translation="MQNLFQPRKPIGVRPAATLFIFFLEIFRRIVYPRGTTLLYPWIY
                     DPMPPSSAAKSRKWAFTLNNPTGGAELYGDEWVVEYLVVGHEIAPETGTPHHQGYVRF
                     ANPRALAGVRKLLPRAHWEPVKGTEQQNYNYCSKEQVIVIELGEREVPEDASDQKQPG
                     KRSDIALARSIISDGGGMIDVVHAVNSYQAIKCAEVMLKYLEQPRNWKPEVIWLWGKT
                     NVGKSHLAMQLCPNAWCSADHSKWFEGYDAHENVVLDELREDWCPFTTMLRLLDKWPY
                     RIEFKGGSRQFLARKIVITSAQPPHVVYANRKGEDLQQLGRRIDQIVEIRGRGEALWV
                     PGKSIGLPYAGRALAYLYPEPEPELEVEGNTAEEDSKVPPMPSLERETSSSAPEPTAP
                     DPSTCTHQLSGCYVTEEQWEATFGWPPADYGETK*"
     CDS             complement(1372..2778)
                     /codon_start=1
                     /product="Putative CruV Capsid Protein"
                     /translation="MVRLASSRRTVPVRRKVVVAPRRSAPVSAAAVSEKAAAKAVQKV
                     MNLFGATPQALAAANKQRLARASARDSAMSGALGFAGRAAAAYFGGPAAALPSIMGSG
                     DYITSADPVKYNTVLNAGQIPQFSGGKNVTTIRHREYLGDIISSSSANTFQVQTFNIN
                     PGLSATFPWLSGVCGATYQQHRINGMVFEFRSTSADALNSVNTALGSVVMATDYDSAD
                     AAFTSKAQMENTEFGVSCKPSQSMIHAIECARNQTTVSEQYIRAFAVPTGADIRLYDL
                     GKFYIATNGFQGTSVNCGELWVSYDVTLFKAIQQPPGFLTPCAHFALAPASLATAPFT
                     VSTTVGTNFDNIGLVFAANGLSFTMPLSIPIKSRWLIIQREAGTAAAATIHTYGYSNG
                     LNALGTFVGQASGAAKAPSPSALTGTTGTLTIGTFSYDGSGTPSALPTVTIDAAATPL
                     ASYSSGDLMLVMMSGNWQ*"
ORIGIN
        1 atgcaaaatt tgtttcaacc aagaaaacca ataggagtcc ggcccgccgc gaccctattt
       61 attttctttt tagaaatctt tcgccgcatc gtatacccgc gcggaacaac tctgttgtac
      121 ccttggatat acgacccgat gcctccgtca tctgctgcga aatcgcgtaa atgggcattt
      181 acgctcaaca atccaaccgg cggagccgag ctgtatggtg atgaatgggt ggtggagtac
      241 ctcgtcgtcg ggcacgaaat cgcgcccgag acaggcaccc cccaccacca aggctacgtc
      301 cgcttcgcga accctcgggc tcttgcgggc gttcgcaagc tcctccctcg agcccattgg
      361 gagcctgtta aagggaccga acaacaaaac tacaactatt gctctaaaga gcaagtcata
      421 gttattgagc tcggcgagcg tgaagtccct gaagatgctt ctgaccagaa gcagcccggc
      481 aagcggtccg atatagcgct agcgcgctct atcatctctg atggtggtgg catgatcgac
      541 gtcgttcacg ctgtgaactc ctatcaagcc atcaagtgtg ctgaagtaat gctcaagtac
      601 cttgagcaac cacgcaactg gaagcccgaa gtgatatggc tttggggcaa aaccaacgtc
      661 ggcaagtcgc atctagcgat gcaactctgc cccaatgctt ggtgcagcgc cgaccattcg
      721 aaatggttcg aaggctatga cgctcacgag aacgtcgttc tcgatgagct ccgtgaggac
      781 tggtgtccgt tcaccacgat gcttcgactc ctcgataagt ggccttatcg aatcgagttc
      841 aaaggcggtt cgcgtcagtt cctggcccgc aaaatcgtga tcacctccgc ccagccccct
      901 cacgttgtct acgctaaccg caagggcgaa gaccttcaac agcttggtcg aagaatcgac
      961 caaatcgttg agattcgcgg ccgcggggaa gccctatggg ttcccggcaa atccattggt
     1021 ttgccctacg ctggccgcgc cctcgcgtac ctatatccgg aaccggaacc ggaactcgag
     1081 gtcgagggta atactgcgga agaggactct aaagtccctc ccatgccaag cctggagcgg
     1141 gaaacgtcct cttccgcgcc ggagcctacg gccccggacc cctcgacctg cacccatcaa
     1201 ctttctggat gctatgtaac tgaagaacag tgggaagcaa ccttcggttg gcctcccgct
     1261 gactatggtg aaaccaagtg agatcgaccc gaaaaatcgg gtcgcctccc tcctggcaac
     1321 atagttgcca agccagtgcc aagcactgag caagcttttt gcggaactgg ctcactgcca
     1381 gtttcccgac atcatcacga gcatcagatc ccctgatgaa tatgacgcaa gtggcgttgc
     1441 cgctgcgtca attgtgacgg tcgggagagc cgacggggtc ccactcccat cgtacgaaaa
     1501 cgtaccaata gtgagcgtac ccgttgtacc cgtcaaagct gacggcgaag gtgccttcgc
     1561 cgctccacta gcctgaccaa caaaggtccc aagcgcattg agcccattgc tgtatccgta
     1621 agtgtgaatc gttgcagccg cggccgtacc ggcctctctc tgaatgataa gccatctcga
     1681 ctttatcgga atcgaaagag gcatcgtaaa cgatagccca ttcgccgcaa agacgagacc
     1741 gatgttgtcg aagttggttc ccaccgtcgt cgacacggta aatggagccg ttgccaatga
     1801 agcaggggcc aacgcaaagt gagcacaagg agtcaagaag cctggcggct gctggatcgc
     1861 tttgaacagt gtaacgtcat acgacaccca cagttcgcca cagttgactg aagtcccctg
     1921 aaacccgttc gttgcaatgt agaacttgcc aagatcgtag agcctgatat cggcgccagt
     1981 cggcaccgca aaggctcgaa tgtattgctc actgacagtc gtctgattac gcgcacattc
     2041 gatcgcatgt atcatgctct gcgacggctt gcaagaaaca ccgaactcgg tgttctccat
     2101 ctgcgccttc gaagtgaaag cggcatccgc cgaatcatag tctgtcgcca tgacgacgga
     2161 gcccaacgcc gtgttcaccg aattcaacgc gtctgccgac gtcgatcgaa attcgaagac
     2221 catgccgtta atccggtgct gctggtacgt tgcaccacag accccagaaa gccacggaaa
     2281 cgtggctgac aaccctgggt tgatgttaaa tgtctgaacc tggaaggtat tcgccgagct
     2341 cgaagaaatg atgtccccga ggtactcgcg gtggcgaatc gttgtcacat tcttcccgcc
     2401 ggagaactga gggatctggc cagcgttcaa cactgtgttg tacttgaccg gatccgcact
     2461 tgtgatgtaa tcaccagaac ccataatgct gggaagcgca gcagcagggc caccaaagta
     2521 agcagccgca gcgcgaccgg caaagccgag tgcgccgctc attgctgagt cgcgggcaga
     2581 tgcccgcgct aagcgctgct tattggctgc cgccaacgcc tgcggcgttg ccccaaatag
     2641 gttcataacc ttctgaaccg ctttggctgc agctttttca gataccgcgg ccgccgatac
     2701 gggtgcggag cgacgcggcg ctaccacaac cttccggcgg accggaaccg tccgtctcga
     2761 tgaagctaat cgcaccatcg ttgtcggaag caggtatcta agcacaaata aaaatctgtg
     2821 aataaaaata tatatctctc ttttaattgc ttggtcaccg gggggtaccc gtggggtcaa
     2881 cccccctcgt ccgtgtgatc atactcggcc cttcccgcct gtcaccaggc ggggcgctgc
     2941 gcgccccgca atgcggtgtg accggcccgt acgggccgcc ttcggtcgta tgcacgcccg
     3001 ggggggtttc gcccgcagga cccacccccc taagagacca attaatttgc aaga
//
LOCUS       CruV-137                3056 bp    DNA     circular     24-FEB-2020
DEFINITION  Crucivirus-137 isolate 001791_virusbaton.
ACCESSION   CruV-137
KEYWORDS    .
SOURCE      Crucivirus-137
  ORGANISM  Crucivirus-137
            Unclassified.
REFERENCE   1  (bases 1 to 3056)
  AUTHORS   de la Higuera,I., Kasun,G.W., Torrance,E.L., Pratt,A.A.,
            Maluenda,A., Colombet,J., Bisseux,M., Ravet,V., Dayaram,A.,
            Stainton,D., Kraberger,S., Zawar-Reza,P., Goldstien,S., Briskie,J.,
            White,R., Taylor,H., Gomez,C., Ainley,D.G., Harding,J.S.,
            Fontenele,R.S., Schreck,J., Ribeiro,S.G., Oswald,S.A., Arnold,J.M.,
            Varsani,A., Enault,F. and Stedman,K.M.
  TITLE     Direct Submission
  JOURNAL   Submitted (24-FEB-2020) Biology, Portland State University, 1719 SW
            10th Ave, Portland, Oregon 97201, USA
COMMENT     Bankit Comment: ALT EMAIL:ide@pdx.edu
            Bankit Comment: TOTAL # OF SEQS:451
            Bankit Comment: GAP: est. length - alignment

            ##Assembly-Data-START##
            Assembly Method       :: MetaSPAdes v. 3.12; IDBA-UD v. 1.1.1;
                                     MEGAHIT v. 1.0.3
            Sequencing Technology :: 454; Illumina
            ##Assembly-Data-END##
FEATURES             Location/Qualifiers
     source          1..3056
                     /organism="Crucivirus-137"
                     /mol_type="genomic DNA"
                     /isolate="001791_virusbaton"
                     /isolation_source="Water"
                     /country="France"
                     /collection_date=2013
     CDS             1..1371
                     /codon_start=1
                     /product="Putative CruV Capsid Protein"
                     /translation="MPRASVSARRPARPPAARPMVRAAPKKRVPRAPSERKSSYSYDN
                     PGPVGRVGRIAGRALGTYLGGPMGGAAGDALGGMAHYIGKVFGSGDYITNADRVKRNV
                     LTNDVQIPQFGSDKNVVHIRHREFLGDVISSSSANTFQVQSYAIQPGQSASFPWLSNV
                     VGVNFQQYRVNGMLYEFRSMSSDALNSTNTALGSVVMATDYDSADSAFTTKQQMENTE
                     FGVSCKPSCSMIHAIECARSQTAVSEQYVRPGAVPSNADIRLYDLGKFYIATTGMQGT
                     NVNLGELWVTYDITFFKAIQLSPGLSIPSAYYPISLTNAGTLPFLVAGSAGPIPAVDT
                     IGLTFAANSLSFSFPISIPLNSVWVVSWSGVGTAANATAQTSFVLSNGFVNAVTALGL
                     RGYFSPNPTAVSANTTGVSVTEFIQYTGGATPNALPTVALLAFTTPLATYTFCDLGVT
                     MINGGL*"
     CDS             complement(2033..2884)
                     /codon_start=1
                     /product="Putative Replication Associated Protein"
                     /translation="MSIDNCRTRNWCFTLNNWTPEEYGIVEDLNVKASYLIIGKEVGS
                     NGTPHLQGYIEYPNAVRGSAIRKIIERIHWEVRKGTAQQASEYCKKDKNFIEFGSISA
                     QGKRTDLEEIGAAVVAGSSIREIAITFPGTYIKFHKGIAALKSALYEARTEPPKVIWR
                     WGLAGVGKSRGAFDAHKTFYIKDGTPWWDGYEQQEAIVIDDFDNGKWPFRDMLRLLDR
                     YPYRGQNKGGYVEINSPFIYITCEYPPYKFWSGNELSQVMRRITEIVEVRGSEMLPLK
                     EEIKTSL*"
     misc_feature    2178..2209
                     /note="stem-loop"
     misc_feature    2189..2197
                     /note="nonanucleotide"
ORIGIN
        1 atgcctcgcg cgtctgttag tgctcgtcgt cctgctcgtc ctcctgctgc ccgtcctatg
       61 gttcgagcag ctcctaagaa acgagttcct cgagctccat ccgaacggaa gtcttcctat
      121 tcttatgaca atcccggtcc cgttggaagg gttggaagaa ttgccggtcg agctcttgga
      181 acctatttag gcggtcctat gggtggagcc gcaggcgatg ccctcggtgg aatggcacat
      241 tacatcggta aagtattcgg ctctggggac tacattacaa atgcggatcg tgtcaagcgc
      301 aatgtgctta caaatgatgt tcaaatcccg cagtttggtt ctgataagaa tgtggtccat
      361 attcgtcatc gagaattcct cggagatgtt atttcaagct cttctgcaaa tacttttcaa
      421 gtacaatctt atgctattca gccaggacaa agtgcatctt ttccttggct ttcgaatgta
      481 gttggtgtta acttccaaca gtacagagta aatggaatgt tgtatgagtt tcgttctatg
      541 tcaagtgacg ccttaaattc taccaataca gctctcggat cagtcgttat ggctactgat
      601 tacgatagcg cagattctgc ttttactact aaacagcaga tggaaaatac tgaatttgga
      661 gtctcttgca aaccttcttg ttctatgatc catgcaattg agtgtgcgag gtctcagacc
      721 gcagtatctg aacaatacgt gcgtcctggc gccgttcctt ctaatgctga tatcaggctt
      781 tacgatcttg gaaagtttta tatcgcaact actggaatgc aaggtacaaa tgttaattta
      841 ggggagcttt gggtgaccta tgatattact ttctttaaag ctatacagtt gtcacctggt
      901 ttgtctattc catccgcata ttatcctatc tctcttacaa atgcaggaac attaccgttc
      961 ctcgttgcag gttctgcagg tcctattcct gcagtagata ctatcggtct tacttttgca
     1021 gcaaacagcc tgtctttctc tttcccaatc agtatacctt tgaattctgt ttgggtggtc
     1081 tcatggtcag gcgttggaac tgctgcgaat gctacagcac agacaagttt tgtcctttct
     1141 aatggttttg tcaatgctgt cacagctctt ggtttgagag gatatttttc tccaaatcct
     1201 actgctgtga gtgcaaatac cactggagtt tctgttactg agtttatcca gtatacagga
     1261 ggagctacac ctaatgctct tcctacagtt gctctgctcg catttacaac tcctcttgct
     1321 acgtatacat tttgcgatct aggagtcaca atgattaatg gaggtcttta acggtcaaat
     1381 ttgaccggta aaatggagga ggtaaatata cccccgcaaa aaacaaacaa aaagacctat
     1441 aaggtcatat tttttggcat attaaaaaac gcaccttgaa cagagggcgg cgtcagccgc
     1501 caaataaaca cattctgact ttcccccgcg aagcgggagg tcaaagggcc ggggcggcgg
     1561 cttagccgcc ccgtaagcca acacaagtat gtgcaatgcc gcgaagcggc gcggccttca
     1621 gccgcagcac cctaacgtag acaatcagcc cccacgcgaa gcgtgagggg cgtttcacca
     1681 agtaccatat tggtacttgg tcccttaaaa cagggagtaa aaaaagcagc gcagaataga
     1741 gcggagctcg taattgtggg ggtgagcctt tggcacaaag gctagtatta ccccccactt
     1801 ctgtgccaaa acgtccagta gccgcgcaat tgagaaataa gcgcatgacg tttacagaat
     1861 tggaaaaact tttgtgccaa gaataacttc tgtgccaagg ctatacataa gttcaaaagt
     1921 taaaaaaaag cttatatatc atcaccgaaa tttcggcgat gatagatcag ctctaatgta
     1981 agaatatatc taaaaaaata gggtgtccgt ttcagacacc ctttatttaa gcttataagc
     2041 tggttttgat ctcttctttc aggggtaaca tttcgctacc cctgacctct actatctcag
     2101 tgattcttct catcacttga gatagttcgt tacccgacca aaacttatat ggaggatact
     2161 cacatgtaat gtaaatgaaa ggtgagttta tttctacata tccacctttg ttttgtcccc
     2221 tgtaaggata acgatcaagg agacgtaaca tgtctctgaa cggccatttg ccattgtcga
     2281 agtcatctat gacaattgct tcttgctgtt cataaccatc ccaccaggga gtaccatctt
     2341 tgatgtaaaa ggtcttgtgt gcatcgaatg caccacgact ctttccaaca ccagcaagtc
     2401 cccatcgcca gatcacttta ggtggttctg tgcgagcttc ataaagtgca gatttcaatg
     2461 ctgctattcc tttatggaac ttgatgtatg ttcctggaaa tgtgatagct atctcacgga
     2521 tagagcttcc agcaactact gctgcaccaa tttcttcaag gtcagttctc ttaccttgag
     2581 cagaaataga accaaattcg ataaaatttt tatctttctt acaatattcg gacgcttgtt
     2641 gggcagtacc tttgcggact tcccaatgta tgcgttcaat tatctttcta atcgctgatc
     2701 ctctgacagc gttaggatac tctatataac cttgtaaatg aggagttcca ttggaaccta
     2761 cttctttacc aatgataaga tatgatgctt tgacattcag gtcttcaaca atgccgtact
     2821 cttcaggagt ccaattgttc aatgtaaaac accagttacg tgtacgacag ttatcgatac
     2881 tcatgatccc gccaaagaaa ttagtatcta tcactaacgt gtatgtcact ggtagtatat
     2941 aacttctgtg ccaagcttaa gaagaaaata aatatttatt aattaattaa taaattatca
     3001 taacaagttc gatcgtatac atatactcca actttttcat caacgagaat aaagaa
//
LOCUS       CruV-138                3060 bp    DNA     circular     24-FEB-2020
DEFINITION  Crucivirus-138 isolate 001787_virusbaton.
ACCESSION   CruV-138
KEYWORDS    .
SOURCE      Crucivirus-138
  ORGANISM  Crucivirus-138
            Unclassified.
REFERENCE   1  (bases 1 to 3060)
  AUTHORS   de la Higuera,I., Kasun,G.W., Torrance,E.L., Pratt,A.A.,
            Maluenda,A., Colombet,J., Bisseux,M., Ravet,V., Dayaram,A.,
            Stainton,D., Kraberger,S., Zawar-Reza,P., Goldstien,S., Briskie,J.,
            White,R., Taylor,H., Gomez,C., Ainley,D.G., Harding,J.S.,
            Fontenele,R.S., Schreck,J., Ribeiro,S.G., Oswald,S.A., Arnold,J.M.,
            Varsani,A., Enault,F. and Stedman,K.M.
  TITLE     Direct Submission
  JOURNAL   Submitted (24-FEB-2020) Biology, Portland State University, 1719 SW
            10th Ave, Portland, Oregon 97201, USA
COMMENT     Bankit Comment: ALT EMAIL:ide@pdx.edu
            Bankit Comment: TOTAL # OF SEQS:451
            Bankit Comment: GAP: est. length - alignment

            ##Assembly-Data-START##
            Assembly Method       :: MetaSPAdes v. 3.12; IDBA-UD v. 1.1.1;
                                     MEGAHIT v. 1.0.3
            Sequencing Technology :: 454; Illumina
            ##Assembly-Data-END##
FEATURES             Location/Qualifiers
     source          1..3060
                     /organism="Crucivirus-138"
                     /mol_type="genomic DNA"
                     /isolate="001787_virusbaton"
                     /isolation_source="Water"
                     /country="France"
                     /collection_date=2013
     CDS             1..1329
                     /codon_start=1
                     /product="Putative CruV Capsid Protein"
                     /translation="MQRRAYFARKSAAVSGRGAYHKAPAKRAPAKKPAASRSAPRSSQ
                     PWLAPALGVVGSAIGTAVGGPAGGLVGNAAGQAAGSIIRHVTGYGAYKVRSNVLYEGA
                     QIPMVRNRSVRNGTVIRHREYLMDVISSSSASTFNIQSFSLNPGQEGTFPWLAQIASN
                     YEEFIIEGCLFEFRSLSGDALTSTNTALGSVIMATNYNAANAAFASKAEMESYEYSMS
                     CKPSESMIHPIECDPRQTPISELYIRPGSVPSGQDVRLYDLGIFQIATTGMQGTSVNL
                     GELWISYQVSLLKPKLFATLGLLGGFWKNTATTGVTNAQPLGTTPVASSSSNLAITFT
                     GAGFNVINFPLTSAPQAYEIYFRWVGGSVVWVPPAVSFSGMTALSVYAGGTVDTPSSG
                     ATATTVSKICSFLTNGQYQLPTLTIGTAGTLPTAVSAFEMVVSQIPNNAN*"
     CDS             complement(1442..2680)
                     /codon_start=1
                     /product="Putative Replication Associated Protein"
                     /translation="MIHQNTDQDKYLNRAENCQTCNTMSKRSRAWCFTYWGVGGDHKK
                     GIQGVLPPKETYDIPCRYILVGLETSNAGWLHHQGFVMFANASTANAVKKVLPIGCHL
                     EACKGTALQNIKYCSKDGNIVAENGKRPEQGKRNDMETVFKIIKTTGRMADVTENVRS
                     YQAMKAGELILKYHEPKRFTKPFVGWFWGPTGCSKTHSAYELLGENGTWKSSGPMKWW
                     EGYDGEQNVIIDDFREHYCDFVTLLSLLDRYPFRVENKGGSRQLQARNIIVTSCYPPD
                     KCFRTSREDVKQLLRRIDTIKFYNVVYEGNLGCINYDSSVAHSAESRAEIPPGFEIPI
                     DTSSENICVDTPADTEVSVSLTIAPECIPFIKNVPTGPYVPVTQKCGRGNTMLSHSTT
                     VKETENEDLSDLLKELGCIF*"
     misc_feature    1514..1542
                     /note="stem-loop"
     misc_feature    1525..1533
                     /note="nonanucleotide"
ORIGIN
        1 atgcagcgcc gtgcatactt tgctcgtaaa agtgctgcag tctccggccg aggtgcttat
       61 cataaagcgc cggccaagcg agctcctgcg aagaagccag cagcctcgcg ttctgctcca
      121 cgatcctccc aaccttggtt ggcgccagca cttggtgttg ttggatcagc tattggaacc
      181 gctgttggcg gtcctgctgg tggtcttgtt ggaaacgctg ccggacaggc tgccggttct
      241 ataatccgtc atgtgacggg ttacggagct tataaggtcc gttctaacgt gttgtacgag
      301 ggtgctcaaa taccaatggt tcgtaaccgt tcggtccgta atggcacggt tattcgccat
      361 cgtgagtatt taatggatgt tatatccagt tcttcagcaa gcacgttcaa tatccagtca
      421 ttctctttga atcctggtca ggaaggtacc tttccttggt tggcacaaat tgcctccaac
      481 tatgaagaat ttatcattga gggttgcctc ttcgagtttc gatctctgtc aggtgatgct
      541 ttgacatcaa ccaataccgc tctcggttca gttattatgg caaccaacta caacgccgct
      601 aatgcggcct ttgcctctaa ggcagagatg gagtcttatg agtattcaat gtcttgtaag
      661 ccatccgaga gtatgatcca tcctattgag tgtgatcctc ggcaaacacc tatttcggag
      721 ctttacatcc gtcctggttc agtgccctcc ggacaagatg tccgcctgta tgatcttggc
      781 atctttcaga ttgccactac aggtatgcaa ggcactagcg tgaacctcgg tgagctctgg
      841 atttcttatc aggtgtcttt gcttaagcct aagttgttcg caaccttagg actattgggc
      901 gggttctgga agaacaccgc taccaccggt gtgacaaatg ctcagccatt gggaactact
      961 cctgttgcaa gttcgtcgag taatcttgcg atcacgttta ccggtgctgg cttcaatgta
     1021 atcaactttc cgttgacttc tgctccccaa gcgtatgaga tttactttcg ctgggttgga
     1081 ggttctgttg tatgggtgcc tcctgcagtg agtttctctg gtatgaccgc cctttcagtt
     1141 tatgcgggcg gaactgtcga tacaccaagt agcggagcta cagcgacaac agtcagcaag
     1201 atttgttctt tcttgactaa tggacaatac cagttaccga ctttgacgat cggtactgca
     1261 gggactcttc ctacggctgt atcagccttt gagatggtag tatctcaaat tcctaacaac
     1321 gccaattaat tgacataaaa aagcatctgg ctactgagcc tatggggtac tgtgtcgcac
     1381 cttgggtctt gcaagacccg ccccaaaggg gcggtgctac aagacacaat acaccacgag
     1441 gttagaagat gcatccgagc tctttgagca gatctgatag atcttcgttt tctgtttctt
     1501 tcacagtagt tgagtgggag agcatagtat tacctctccc acacttctgt gtaacgggta
     1561 cgtaaggacc cgttgggacg ttttttataa acggaatgca ctctggcgct attgttagcg
     1621 acacacttac ttctgtgtca gccggagtat caacacaaat gttctctgaa ctagtgtcga
     1681 ttggaatctc gaaccctggc ggtatctccg cccggctctc cgccgaatgt gcgacactgc
     1741 tgtcgtaatt gatgcaaccg aggttgcctt catacactac gttatagaac ttgattgtat
     1801 caattcttct gagtagttgt tttacatctt ctcggctggt tcgaaagcat ttatcagggg
     1861 gatagcacga agtgactatt atgttccttg cttgtagctg tctggaaccc cctttgtttt
     1921 ctacgcggaa aggatatctg tcaaggagag agagtaaagt gacaaagtca cagtaatgtt
     1981 ctctgaagtc atctattatg acgttttgtt ctccgtcata tccttcccac catttcattg
     2041 gtccgctgct tttccaggta ccgttctcac cgagcagttc ataagcgcta tgcgtcttac
     2101 tgcatccggt aggaccccag aaccatccaa caaacggttt tgtaaatcgt tttggttcgt
     2161 ggtattttaa gatgagttcg cctgctttca tagcttggta tgagcgaaca ttttcagtaa
     2221 catcagccat tctgcctgtt gtttttatta tcttaaacac tgtttccata tcattccgtt
     2281 tcccttgttc aggccttttg ccgttttcgg cgactatgtt gccgtctttc gagcagtact
     2341 ttatgttctg gagcgccgtg cctttgcagg cttctaagtg acacccgata gggagtactt
     2401 ttttgaccgc attagcggtc gaggcgttcg caaacattac aaacccttgg tggtgaagcc
     2461 acccggcatt acttgtttca aggcctacaa ggatgtatct acaaggtatg tcataagtct
     2521 cctttggagg caagacgcct tggatacctt ttttatgatc acctcctacg ccccagtagg
     2581 taaagcacca agctcttgag cgctttgaca tggtattgca tgtctggcag ttttctgcgc
     2641 gatttaaata tttgtcttgg tctgtatttt ggtgtatcat atttatttat tttgtagtcg
     2701 atccgaattt tcggtcgttt tactcaaatg acacagtgtc attttgagta tactcaaatg
     2761 atatacacaa atgctatgtc attttgtgta gcgatctctc cgcagtcgct atttaatttt
     2821 ctccgccgga actttttcgt acaacgttgt ccgaatgaca ccatgtcatt tgggttgtgc
     2881 aaaagacacc atggtgtcat atggacagtg tctattattt attttgtgtt ccgcgttttt
     2941 tcgcatgtgt tttacacaaa tgacatatat gtcaaatgtg tatacactca gtgggtaccc
     3001 cgcgtggtgt atatgcatta attatttgcg aaattttttt gtacggtatg tataccgact
//
LOCUS       CruV-139                3065 bp    DNA     circular     24-FEB-2020
DEFINITION  Crucivirus-139 isolate 120_1635_IR2_3297.
ACCESSION   CruV-139
KEYWORDS    .
SOURCE      Crucivirus-139
  ORGANISM  Crucivirus-139
            Unclassified.
REFERENCE   1  (bases 1 to 3065)
  AUTHORS   de la Higuera,I., Kasun,G.W., Torrance,E.L., Pratt,A.A.,
            Maluenda,A., Colombet,J., Bisseux,M., Ravet,V., Dayaram,A.,
            Stainton,D., Kraberger,S., Zawar-Reza,P., Goldstien,S., Briskie,J.,
            White,R., Taylor,H., Gomez,C., Ainley,D.G., Harding,J.S.,
            Fontenele,R.S., Schreck,J., Ribeiro,S.G., Oswald,S.A., Arnold,J.M.,
            Varsani,A., Enault,F. and Stedman,K.M.
  TITLE     Direct Submission
  JOURNAL   Submitted (24-FEB-2020) Biology, Portland State University, 1719 SW
            10th Ave, Portland, Oregon 97201, USA
COMMENT     Bankit Comment: ALT EMAIL:ide@pdx.edu
            Bankit Comment: TOTAL # OF SEQS:451
            Bankit Comment: GAP: est. length - alignment

            ##Assembly-Data-START##
            Assembly Method       :: MetaSPAdes v. 3.12; IDBA-UD v. 1.1.1;
                                     MEGAHIT v. 1.0.3
            Sequencing Technology :: 454; Illumina
            ##Assembly-Data-END##
FEATURES             Location/Qualifiers
     source          1..3065
                     /organism="Crucivirus-139"
                     /mol_type="genomic DNA"
                     /isolate="120_1635_IR2_3297"
                     /isolation_source="Water"
                     /country="Norway"
                     /collection_date=2011
     CDS             1..1458
                     /codon_start=1
                     /product="Putative Replication Associated Protein"
                     /translation="MAQRFNSKNSKKNHQVYGEKDTKKEENSTDTDVGQGNTSLSKTS
                     LRSESLRSESLRSSASDQSCNDDEKRVRNYCFTSYKGTIPFKKSMKYLLQGIEICPST
                     GKTHIQGFVIFNNARTCKAVRKEYNPIHFETCKGSIEQNINYCKKDKDFTEEGEIPKG
                     QGARTDKVSLTESIKEGLSDKEIIEKHGDKFIRNYRGIAMTRNILKEEIRKWDMDVRI
                     YWGEPGTGKTRAVWDEFGYEEVYPKMVGKWWDGYKGQKCVLIDDFNPENAFEITFDFY
                     LKLLDRYPMTIENKGGTSQFYSKVIIFTSNFNPEYWFEDKPEVARKAFFRRVKTIKEF
                     NFEEEKQENTEPKSSYSGVFGENDVTWEKQEAEENEPVLSKEDIQKIFDDIEKFKEEE
                     KNKQVTDPEDEYIIKEEKQENTEPKSSYSGVYEYVRNDFGRCVKMNVENMDIQERLRL
                     THRHNFNELNVYEKDNLIKCEKLRDIRDKNTKESN*"
     CDS             complement(1643..2977)
                     /codon_start=1
                     /product="Putative CruV Capsid Protein"
                     /translation="MNMYKPRAKSSYKPRAKPATAKKPYPKKTYYKKSYPRKNYAGSV
                     IKTIGTLGGGAIGSAFGGPAGGALGSAVGGVAADLFKQITGMGDYNVKNNSLFIQGDS
                     TPMFRNSGRSVRITHREYISDIITSSVALSFKLNSFLLNPATSDTYPWLATIAQQFEQ
                     YRIHGMVFHFKSNSADALNSTNTALGSVIMATQYNVQLPVFTTKQQMENYEFACSSRP
                     SCDLLHPVECDPSLTSFGPVFDIRLGGDDGGDQRLYDLGRFCLATVGMQAANVNIGEL
                     WVSYDIELLKPRMGDTVSVFGQWRVADPTDASPFLDAVISEDSDFFAIIGTNTITFPA
                     NFNGNVLVWYKSRSTLAATQASFTTVTLSSGITRLGILTGGGSDASQYATGSVSDTEC
                     CFIQMLRIVSNGVPQVATYTGGTWVNADLGSDLIINVIPYTVTVPYVAENFN*"
ORIGIN
        1 atggcacaaa ggttcaattc gaagaattca aagaaaaatc atcaggttta cggggaaaaa
       61 gatacaaaaa aagaggaaaa cagcacagac acagatgttg gacagggtaa tactagcctg
      121 tccaaaacgt cgcttcgctc cgagtcgctt cgctccgagt cgcttcgctc ctccgcatct
      181 gatcaatcct gcaacgacga tgaaaaaaga gttagaaatt actgctttac aagttacaaa
      241 ggaactatcc catttaagaa atcaatgaaa tatcttcttc aaggaataga aatatgtcct
      301 tcaactggca aaactcatat acaaggattc gttattttca ataatgccag aacttgcaaa
      361 gctgttagaa aagaatacaa tcctattcat tttgaaactt gcaaaggaag tattgaacaa
      421 aatataaatt attgcaaaaa agacaaagac tttactgaag aaggagaaat accaaaaggt
      481 caaggtgcgc gcacagacaa agtgtcactc actgaatcga taaaagaagg tctctcagat
      541 aaagaaataa tagaaaaaca tggagataaa ttcatcagaa actacagagg aatagcaatg
      601 actagaaaca ttttaaaaga agaaataaga aaatgggata tggatgtacg catctactgg
      661 ggcgagcctg gaacaggtaa aacccgagca gtatgggacg aattcggtta tgaagaagta
      721 tatcctaaaa tggtaggtaa atggtgggat ggttataaag gacaaaagtg cgttctaatt
      781 gacgacttta atcccgaaaa cgcatttgaa ataacgttcg acttttattt aaaactgctg
      841 gatagatatc caatgacaat agagaataaa ggtggaacct cacaatttta cagcaaagtt
      901 attattttca caagtaactt taatcctgaa tattggttcg aagataaacc agaggtagct
      961 agaaaagctt tctttagaag agtaaagact ataaaagaat ttaattttga agaagaaaaa
     1021 caagaaaata cagaacctaa aagttcctac agtggagtat ttggagaaaa tgacgtcaca
     1081 tgggaaaaac aggaagctga agaaaacgaa cctgtactgt ctaaagaaga tatacaaaaa
     1141 atattcgacg atattgaaaa attcaaagaa gaagaaaaaa ataaacaagt aacagatcca
     1201 gaagatgaat acataattaa agaagaaaaa caagaaaata cagaacctaa aagttcctac
     1261 agtggagtat atgaatatgt aagaaatgac tttggaagat gtgttaaaat gaatgtagaa
     1321 aacatggata ttcaagaaag attaaggctt acgcacagac acaacttcaa tgaattaaac
     1381 gtatacgaaa aagataatct tataaagtgc gaaaaactca gagatataag agataaaaat
     1441 acaaaagaaa gtaattaaac attggtctta ggacatatgt ttgaagatgc tgcgcttcgc
     1501 tccgcccgtc tcagaagctc acgcactgct acaggctctg ccgctaccgc tgcagccgct
     1561 aacgcggaca ccctcgcgat cttaaattaa tcgaaagtgc tacgctccgc ttcgccaatc
     1621 atatcttgta taagaaatga ttttaattaa agttttcggc aacataagga actgtaacag
     1681 tataaggaat aacatttatt attaagtcag aaccaagatc agcattgacc caagtacctc
     1741 ctgtataagt agcaacttga ggaacaccat tagaaacaat tcttaacatt tgaataaaac
     1801 agcactcggt gtcagaaaca gaaccagtag catattgact agcatccgaa ccacctccgg
     1861 ttaaaatacc taatctggtt attccagaag ataaagtaac tgtagtaaaa gaagcttgag
     1921 tagcagcaag agttgatcta gatttatacc aaacaaggac attaccattg aaattagcag
     1981 ggaaagttat agtatttgta ccaataatag caaaaaaatc ggaatcttct gatataacgg
     2041 catctagaaa aggagaagca tctgttggat ctgcaactct ccattgaccg aatactgaaa
     2101 cagtatcccc cattctgggt ttgagaagct caatgtcata agaaacccat aattctccaa
     2161 tatttacatt agctgcctgc atacctacag tagcgagaca gaatcgacca agatcgtata
     2221 atcgttgatc tccaccatca tcaccaccaa gccttatatc aaatacagga ccaaaagaag
     2281 ttaaagatgg atcacattct acaggatgaa gaagatcgca agatggacgt gaagaacaag
     2341 caaattcata attctccatt tgttgtttag tagtgaaaac tgggagttga acattgtact
     2401 gagtagccat aataacggaa cctagagcgg tattagtaga attgagagca tcggcagaat
     2461 tagatttaaa atgaaaaacc attccatgaa tcctatattg ttcaaattgt tgagcaatag
     2521 tagctaacca tggatatgta tcagatgtag cgggattgag aagaaaagaa ttgagtttaa
     2581 aagaaagagc aacagaagat gtaataatgt cagaaatata ttctcggtga gtgattctta
     2641 cagaacgtcc agaattcctg aacatgggag ttgagtcacc ctgaataaat aatgaattat
     2701 tttttacgtt ataatcaccc attcctgtga tttgtttaaa taaatcagca gcaactccac
     2761 ctacggcaga tccgagagca ccaccagcag gacctccgaa agctgaacct atggctcctc
     2821 caccaagagt tcctatggtc ttaataacag aaccagcata attctttctg ggataagatt
     2881 tcttgtaata agttttctta ggatatggtt tcttagcagt agcaggttta gcacgaggct
     2941 tgtaagacga tttggcgcgc ggcttataca tattcatttt ataagtaaat attttctctt
     3001 tatgattttc gcgattttaa aattgtctaa agaaacaaaa ataaacacaa aagaaaaata
     3061 cacgc
//
LOCUS       CruV-140                3066 bp    DNA     circular     24-FEB-2020
DEFINITION  Crucivirus-140 isolate 001778_virusbaton.
ACCESSION   CruV-140
KEYWORDS    .
SOURCE      Crucivirus-140
  ORGANISM  Crucivirus-140
            Unclassified.
REFERENCE   1  (bases 1 to 3066)
  AUTHORS   de la Higuera,I., Kasun,G.W., Torrance,E.L., Pratt,A.A.,
            Maluenda,A., Colombet,J., Bisseux,M., Ravet,V., Dayaram,A.,
            Stainton,D., Kraberger,S., Zawar-Reza,P., Goldstien,S., Briskie,J.,
            White,R., Taylor,H., Gomez,C., Ainley,D.G., Harding,J.S.,
            Fontenele,R.S., Schreck,J., Ribeiro,S.G., Oswald,S.A., Arnold,J.M.,
            Varsani,A., Enault,F. and Stedman,K.M.
  TITLE     Direct Submission
  JOURNAL   Submitted (24-FEB-2020) Biology, Portland State University, 1719 SW
            10th Ave, Portland, Oregon 97201, USA
COMMENT     Bankit Comment: ALT EMAIL:ide@pdx.edu
            Bankit Comment: TOTAL # OF SEQS:451
            Bankit Comment: GAP: est. length - alignment

            ##Assembly-Data-START##
            Assembly Method       :: MetaSPAdes v. 3.12; IDBA-UD v. 1.1.1;
                                     MEGAHIT v. 1.0.3
            Sequencing Technology :: 454; Illumina
            ##Assembly-Data-END##
FEATURES             Location/Qualifiers
     source          1..3066
                     /organism="Crucivirus-140"
                     /mol_type="genomic DNA"
                     /isolate="001778_virusbaton"
                     /isolation_source="Water"
                     /country="France"
                     /collection_date=2013
     CDS             1..1389
                     /codon_start=1
                     /product="Putative CruV Capsid Protein"
                     /translation="MYAKKTTKRKTAPQYARKTYSPRVPAQKVEIIEVVPKPKRVYKP
                     RQTMEVVQKSSGFNAVPNSFGAKLGGWLGHGIGSVVKAITGFGDYTVRENSLMNLNMG
                     GIDPPSIVNSRNGVIIRHREFIGNVLGATVFTLQSFKIQPGLVSSFPWLSQVADAYQE
                     WRPRGIIYEFKSLSSDAVIATNSSGSLGSVIMATQYDSTLANFPDKRTMENSEYANSC
                     KPSCSMLHPIECMKSQSVLSNLYLRAAGISLTGKDPRFYDLGNFQIAVEGMASGSAGQ
                     TIGELWVTFEVEMYKPELGLTGAGVLTDHFSTGVGTNTITSTNPFGNLDVFAPATGNT
                     IGCVIRQAAGNFDTIQFPSNIQSGIYLIAYFMNGTTPTVANSASIANGSSTNISTTGK
                     SALFLNGTTNIVFAPNNGATVSSFSYQQIVEVVQTTPGSKAAIQVALSGVLPSGTQNG
                     NLFITLLNPFIN*"
     CDS             complement(1736..2962)
                     /codon_start=1
                     /product="Putative Replication Associated Protein"
                     /translation="MPMNSSLSEISKKRSKKDREVDFEKVTSEGAGVILDPRMNPHQS
                     TRLKQHFFTWNNYCEKSLVMLLSWVKTHCVRWCFQEEIGESGTPHIQGCITLKEAARW
                     TTFDLPKQIHWEPTRNNKKAEIYCSKDETRLSGAKVYRYPLPLVKTPLKLIEKLRPFQ
                     LSIIDSLVPIPDDRTINWICDEKTNMGKTQLCKYLFVKHKAILFTGGACADIAQTLVG
                     VIEAGKIDLNDQLNPFIFNISKSSEHISYKAIEGCKDGLLNSPKYESQTLVFNSPHIW
                     IFANREPDYDKMSIDRWKIWTINDDFMLVPHNWNYRNRNIEPTGSYEERLTAFLNRPI
                     TNQLNLQPIIDSSDDEKEHIAPPGDNRWTWDQNGNKFYDCDLADAELKERDDIVADWN
                     LERAFSAEEFNYEDLV*"
     misc_feature    2849..2872
                     /note="stem-loop"
     misc_feature    2858..2866
                     /note="nonanucleotide"
ORIGIN
        1 atgtacgcta aaaaaacaac caagcgtaaa accgcacctc aatatgccag aaaaacatat
       61 tcacctcggg ttccagccca aaaggtggag attattgagg tggttcctaa acctaaacgt
      121 gtgtacaaac ctcgtcaaac gatggaagtc gttcaaaaga gcagcggctt caatgctgta
      181 cccaattcat tcggtgcaaa gcttggaggc tggcttggac acggtattgg ctcagtggta
      241 aaagcaatca ccggttttgg tgattacact gtaagagaga attcactcat gaatttgaac
      301 atgggaggaa tcgatccacc atcaatagta aatagccgga acggcgtgat aattcgccac
      361 agggaattta tcggtaatgt actgggtgca actgtattta ctctccaatc cttcaaaatt
      421 caaccaggat tggtatcaag ttttccttgg ttgtcacaag tcgcagacgc atatcaagag
      481 tggagaccaa gaggaattat atatgaattc aaatccttgt cgtcagatgc agtaattgca
      541 actaattcgt caggttctct tggttcagta atcatggcaa cgcaatatga ttcaactcta
      601 gctaattttc cagacaaacg tacaatggag aattcagaat acgcaaacag ctgtaaacca
      661 agctgttcca tgcttcatcc aattgaatgc atgaagagtc aaagcgttct tagtaattta
      721 tatctaagag ctgcaggtat ctcactaaca gggaaagatc cacgctttta tgatttaggt
      781 aattttcaaa ttgccgtaga aggtatggct tctggctccg caggacaaac aataggggaa
      841 ttatgggtaa cttttgaagt agaaatgtac aagcctgaac taggtttaac aggcgctggt
      901 gttctaactg atcattttag taccggagta ggtaccaata caataacatc aacaaatcca
      961 ttcggtaatc ttgatgtttt tgcgccagca acaggtaaca caattggttg tgttattcgt
     1021 caagctgcag gtaacttcga cacgattcaa ttcccgtcta atattcaatc aggtatatat
     1081 ctgatagctt atttcatgaa tggcacaaca ccaacagtag ccaattcagc aagtatagca
     1141 aatggttcat caactaatat tagcacaaca ggcaaatcag ctctgttctt aaatggaaca
     1201 accaacatcg tattcgcacc aaacaatgga gcaacagtat cttcgttctc ttatcaacaa
     1261 atagttgaag ttgtgcaaac aactcctggt tcaaaggccg ctattcaagt cgcattaagt
     1321 ggtgttcttc cgtcaggaac tcaaaatggt aatcttttca tcactttgtt aaatccgttc
     1381 ataaactaat tgtctccact cttcaattct ctaccaaata gatagagaat acagatataa
     1441 ttttatatca atctaggcgt ggccgctagg cccgccaccg tagggtagta gagccaatac
     1501 ttttgaaagt gagcgcgcgc agagcgcgct catcccggag gggccggaag ccactccgtg
     1561 gtgtctttct aaagtgttgg ctcctacgcc tacccttcgg gatgatcaat tagagctcaa
     1621 actcagagtt gggggtcgcc ccttagcaat ttgcgatagg ggggcgatcc cccaatctct
     1681 gatgttttag ctctaattag atcctaaatt ttatatattc atatattaaa tttcttcaaa
     1741 ctaaatcttc ataattaaat tcttctgcgc tgaaggcgcg ttctaaattc caatctgcga
     1801 cgatgtcgtc gcgttctttt agttcggcgt cagccaaatc gcaatcataa aacttatttc
     1861 cgttttgatc ccaagtccag cgattatcgc cgggaggcgc aatgtgttct ttctcatcat
     1921 cactactatc gataatcggt tgtaaattca attggttagt gataggtcta ttcaaaaagg
     1981 ctgtaagcct ttcttcatat gaaccggtag gttcaatatt tctgttccga taattccaat
     2041 tatgagggac tagcataaaa tcatcgttaa tggtccaaat tttccatctg tcgatagaca
     2101 ttttgtcgta atcgggttct ctattagcga agatccaaat atgtgggctg ttgaaaacca
     2161 aagtttgaga ttcatacttg ggtgagttaa gtagtccgtc tttacaacct tcaatagcct
     2221 tatagctaat atgttcagaa gacttgctta tgttgaagat aaaagggtta agttgatcgt
     2281 tcaggtcaat ctttcccgct tcaattacac caacgagtgt ttgagcaata tccgcgcaag
     2341 cgccaccggt gaacaatata gccttatgtt tgacaaacag atatttacac aattgtgttt
     2401 ttcccatatt ggtcttctca tcacatatcc agttgatagt tctatcatca ggtataggta
     2461 ctaatgagtc gataatactc agctgaaatg gtcgaagttt ctcgataagt ttaagcggag
     2521 ttttcacaag aggtaaagga tacctgtata ctttagcgcc ggagaggcgc gtttcatctt
     2581 tactgcaata aatctcagct ttcttgttat ttctagtagg ttcccagtga atctgtttag
     2641 gtaaatcaaa tgtagtccag cgagcagctt ctttcaaagt tatgcaacct tgtatatgag
     2701 gtgttccaga ttcaccaatt tcttcttgaa agcaccatcg gacacaatgg gttttgaccc
     2761 atgaaagtag catgactaat gacttttcac aatagttgtt ccaggtaaaa aagtgctgtt
     2821 tcaatcgagt actctgatga gggttcatgc ggggatctag tattaccccc gctccctctg
     2881 aagtgacttt ttcgaagtca acttctcgat cttttttcga gcgtttttta gagatctctg
     2941 ataaggaact attcataggc aaagtgacta tttcagtatt ggttacaata tctctaaata
     3001 atatttttca cgtttttttg aattaaaata atctaataca aatgtaagac ctataaggca
     3061 caaaat
//
LOCUS       CruV-141                3068 bp    DNA     circular     24-FEB-2020
DEFINITION  Crucivirus-141 isolate C1C10_122.
ACCESSION   CruV-141
KEYWORDS    .
SOURCE      Crucivirus-141
  ORGANISM  Crucivirus-141
            Unclassified.
REFERENCE   1  (bases 1 to 3068)
  AUTHORS   de la Higuera,I., Kasun,G.W., Torrance,E.L., Pratt,A.A.,
            Maluenda,A., Colombet,J., Bisseux,M., Ravet,V., Dayaram,A.,
            Stainton,D., Kraberger,S., Zawar-Reza,P., Goldstien,S., Briskie,J.,
            White,R., Taylor,H., Gomez,C., Ainley,D.G., Harding,J.S.,
            Fontenele,R.S., Schreck,J., Ribeiro,S.G., Oswald,S.A., Arnold,J.M.,
            Varsani,A., Enault,F. and Stedman,K.M.
  TITLE     Direct Submission
  JOURNAL   Submitted (24-FEB-2020) Biology, Portland State University, 1719 SW
            10th Ave, Portland, Oregon 97201, USA
COMMENT     Bankit Comment: ALT EMAIL:ide@pdx.edu
            Bankit Comment: TOTAL # OF SEQS:451
            Bankit Comment: GAP: est. length - alignment

            ##Assembly-Data-START##
            Assembly Method       :: MetaSPAdes v. 3.12; IDBA-UD v. 1.1.1;
                                     MEGAHIT v. 1.0.3
            Sequencing Technology :: 454; Illumina
            ##Assembly-Data-END##
FEATURES             Location/Qualifiers
     source          1..3068
                     /organism="Crucivirus-141"
                     /mol_type="genomic DNA"
                     /isolate="C1C10_122"
                     /isolation_source="Soil"
                     /country="New Zealand"
                     /collection_date=2012
     misc_feature    join(3062..3068,1..19)
                     /note="stem-loop"
     misc_feature    1..9
                     /note="nonanucleotide"
     CDS             119..1447
                     /codon_start=1
                     /product="Putative CruV Capsid Protein"
                     /translation="MAAAPIKKRAVKKPARAPAANRRTYPANARGYISGVGGYKKRTG
                     EKPYYKKYRAPHSTGGDLGSMLGSWLGHGAQHVVQALSGFGDYAVEENSIMPGIGVLS
                     PPEVINTPGGFIVRHREYIQDINATTAFTLQSFPVQPGLFASFPWLAGLADSFEEYRF
                     RGLVYEFKSLSSDSVLSASTSSALGSVIMATSYNVNNAVFPSKIAMENYEFANSSKPS
                     CSFMHPVECKKSISFGTGELYLRTGAVPSGQDGRLYDLGLFQIATVGMQAASGVAGEL
                     WATYEIEFFHAKFDVSGNGGNLDHLQITTATAAAPLGTQGNFNFRSLGGTVNNPTGTI
                     YTFPVTVAIGDKFHISYILVGSVSAAITAPVITLSGCTLKTLLGGNDSSGAVNSPANG
                     VTSVALIMEFFVQITSVATQPTITWGGAGTIPTGTIAADMFVIQIDPAVA*"
     misc_feature    255..280
                     /note="stem-loop"
     misc_feature    260..268
                     /note="nonanucleotide"
     CDS             complement(1736..2953)
                     /codon_start=1
                     /product="Putative Replication Associated Protein"
                     /translation="MTEDTFSKELEGKEGKEFFSNQRLLLTYKTHIPKDELSSFLLKT
                     AKAEKCSKFMCRIAHETGDKKNPYLHTHVVVDFGKVFRSTKQDALDFPTHNMEWDGST
                     GESRNMISYIHPHVRIIFNAHRMKGALKYLSKEDPECADLAEEICKINLIQNQETLTD
                     AFKLNVESPINTKIIFENRVRPLPEFDVEMFDRWQTEGFELLERKGKGPAYPIKTDRA
                     LWRSTVPIPRPPSDDRHITVIVESLGRKGKTQFGKGLMCYNPKRYFTTQGCGNMKDFS
                     TTIENALNGIWKGEVLIINLTRSNMKNQQIYGPIEAIRDGIITSQKFVGKTTIFQPQH
                     VVLTCNSMPDLSKLSQDRWKIYKVHEVHETLEPLTLSQAWDIRHAQISAKVAEEQQLA
                     FSIPQLEVREIRI*"
     misc_feature    2304..2324
                     /note="stem-loop"
     misc_feature    complement(2310..2318)
                     /note="nonanucleotide"
ORIGIN
        1 tatagtaagg tatattagag gtttttttta taaaaaagac aaattttagt cgtagagaac
       61 tcaacttctc tacaaatcag aagtttaaaa aaattttatt tgtcctacta aaggacaaat
      121 ggccgcagca ccaattaaaa agagagcagt taagaaacca gctcgagctc ctgctgcgaa
      181 tcgtagaact tacccagcaa acgctagagg ttatatctcc ggagtggggg gatataagaa
      241 gagaactggt gagaagcctt attataagaa gtatagggct ccacattcaa ctggcgggga
      301 ccttgggtcc atgctgggat catggttggg tcatggagca caacatgtag tacaagcatt
      361 atcaggtttt ggtgattatg cggtagaaga gaactccata atgccaggca tcggagttct
      421 cagtcctcca gaggtgataa atactccagg aggatttatc gtacgacata gagagtacat
      481 acaagatata aatgcgacaa cagcatttac tttacagtct ttcccggtcc aaccaggatt
      541 atttgcatcg tttccatggt tggcagggtt agcagatagt tttgaagagt atagatttcg
      601 aggacttgtt tacgagttta aatcgttatc ttcagattca gtattatctg catctacttc
      661 tagtgcttta ggtagtgtta ttatggcaac aagttataac gttaataacg cggtatttcc
      721 atcgaaaatt gcgatggaaa actatgaatt tgcaaattca tcgaaaccat cttgttcgtt
      781 tatgcatcca gtggagtgca aaaagtcaat atcttttggc accggagaat tatatctccg
      841 aactggagca gtaccatcag gacaagatgg taggttatat gacttaggat tatttcagat
      901 tgctacagta ggtatgcaag ctgccagtgg tgtagccgga gaattgtggg ctacatatga
      961 aatcgaattt ttccatgcta aatttgatgt atctggtaat ggaggtaatc ttgatcactt
     1021 acaaattaca actgcaacag cggcagctcc attaggaaca caaggaaact tcaattttag
     1081 aagtttagga ggtaccgtta ataatccaac aggaactatt tatacatttc cggttactgt
     1141 agcaatcgga gataagttcc atatatcata tattttagta ggttcagtgt cagcagcaat
     1201 tacagctcca gttattacgt tatctggatg tacactgaaa acattattag gtggtaacga
     1261 ctcatcagga gcagtgaact caccagcaaa cggagtgaca tccgttgcgt taataatgga
     1321 attttttgta cagattactt ctgtagctac tcaaccaaca attacttggg gaggagcagg
     1381 gacaatacca acaggtacta ttgctgcaga tatgtttgta atacaaatag atccagcagt
     1441 agcatagagt tacaacaacc aaggttggac agtatgttgt aaccgggacg cttgcgtccc
     1501 ggcgccatgc cgcttgcggc cggtgcttac aaccaacaaa ggtggctgcg ctaaactaat
     1561 atttaatgtt atgtttagcg cagtcggaga actttggttt atcgaagttc gtagacccta
     1621 ctacctatac cctttaggac tttgagcaac ccaagggcgt caaagcccgc ctattaaagt
     1681 tgcggttcca taaagggtat aggtagtatt atatatgtat tcattacata tataattata
     1741 ttcgaatttc tctaacttca agttgtggta tactgaaggc aagttgttgt tcttcggcaa
     1801 cttttgcgga tatctgagca tgtcttatat cccaagcttg agataaagtt agaggttcta
     1861 aagtttcatg cacttcgtga actttgtaaa ttttccatct atcttgagat agtttggaca
     1921 ggtctggcat ggaattacaa gtaagcacaa cgtgttgtgg ttggaagatt gtggttttac
     1981 caacgaactt ttggcttgta atgataccat cacggatagc ttctataggt ccgtatattt
     2041 gttggttctt catgttagac cgggttaaat ttatgattaa gacttcgccc ttccagatac
     2101 cgttaagggc gttttctatt gttgtagaga agtctttcat attaccacat ccttgtgtgg
     2161 taaagtatct tttaggatta taacacatta atcctttacc gaattgtgtt ttaccttttc
     2221 gccccaagct ttctacgatg acggttatat gtcggtcatc gcttgggggt ctaggtatag
     2281 gtactgtgga tctccacaat gctctatcag tttttatagg ataggcagga cctttacctt
     2341 ttctttctaa taattcgaat ccttcagttt gccaacggtc gaacatttca acgtcaaatt
     2401 caggaagggg tctgacccta ttttcaaata tgattttggt atttataggg ctttcaacgt
     2461 ttaatttgaa agcgtcagtt agagtttctt gattttgtat caaatttatt ttgcatattt
     2521 cttcagcaag atcagcacat tcaggatctt ctttgctgag atatttcaat gctcctttca
     2581 ttctatgagc attgaaaatt atacgtacat gggggtgaat atatgaaatc atatttctag
     2641 attcaccggt ggagccatcc cattccatat tatgtgtcgg aaagtctaga gcatcttgct
     2701 tggtgctcct gaataccttt ccgaagtcca caaccacatg tgtgtggaga taagggttct
     2761 tcttatctcc agtttcgtgg gctattctac acataaattt tgaacatttt tcagcttttg
     2821 ctgttttcaa aaggaaagat gatagttcat cttttggtat gtgtgttttg tacgtgagta
     2881 agagtcgttg attggaaaag aattctttac cttcttttcc ttcgagttct tttgagaagg
     2941 tatcttctgt catttgtagt ctacaaatta tttctttata ctatataaga actctacgac
     3001 tattttgctt tatacccccc ccccattagt ctccagtggg acttctctac gactatacaa
     3061 atctaata
//
LOCUS       CruV-142                3068 bp    DNA     circular     24-FEB-2020
DEFINITION  Crucivirus-142 isolate 120_211_SvL2_3298.
ACCESSION   CruV-142
KEYWORDS    .
SOURCE      Crucivirus-142
  ORGANISM  Crucivirus-142
            Unclassified.
REFERENCE   1  (bases 1 to 3068)
  AUTHORS   de la Higuera,I., Kasun,G.W., Torrance,E.L., Pratt,A.A.,
            Maluenda,A., Colombet,J., Bisseux,M., Ravet,V., Dayaram,A.,
            Stainton,D., Kraberger,S., Zawar-Reza,P., Goldstien,S., Briskie,J.,
            White,R., Taylor,H., Gomez,C., Ainley,D.G., Harding,J.S.,
            Fontenele,R.S., Schreck,J., Ribeiro,S.G., Oswald,S.A., Arnold,J.M.,
            Varsani,A., Enault,F. and Stedman,K.M.
  TITLE     Direct Submission
  JOURNAL   Submitted (24-FEB-2020) Biology, Portland State University, 1719 SW
            10th Ave, Portland, Oregon 97201, USA
COMMENT     Bankit Comment: ALT EMAIL:ide@pdx.edu
            Bankit Comment: TOTAL # OF SEQS:451
            Bankit Comment: GAP: est. length - alignment

            ##Assembly-Data-START##
            Assembly Method       :: MetaSPAdes v. 3.12; IDBA-UD v. 1.1.1;
                                     MEGAHIT v. 1.0.3
            Sequencing Technology :: 454; Illumina
            ##Assembly-Data-END##
FEATURES             Location/Qualifiers
     source          1..3068
                     /organism="Crucivirus-142"
                     /mol_type="genomic DNA"
                     /isolate="120_211_SvL2_3298"
                     /isolation_source="Water"
                     /country="Norway"
                     /collection_date=2012
     CDS             1..1083
                     /codon_start=1
                     /product="Putative Replication Associated Protein"
                     /translation="MEEYNALFDKNYDETTFKGQSQYWTFVSWEDPKCEDLSDWFTYI
                     VYGKEITPTTGKLHWQGYIECVAMLRVGTLRKKFLKEFKNVKNIKFLKRYIRSTGEQN
                     RMYCCKGLQSHEEYIEFKDKGENYGLNKETVEFGEIFKPEPGKRSDLKGISEAIKDNS
                     SIKHNIDNEIITTGPALKTFGALTPYYEKKRRLAKPPIVYVIFGDAGSGKTEWCRNMA
                     ESLNLGNIYEDYRGGGYWQGYDNEQIVLFDDFRDTWMDFELLLKILDKGPVTLGCKFG
                     SRQLAASIFFITCSKHPREWYKGVSESRYQLYRRISICFHFEFEIGWNCFKQMKLNEW
                     DAFKVKMTPVSLEKEEDYVKNTDFGW*"
     misc_feature    765..790
                     /note="stem-loop"
     misc_feature    complement(775..783)
                     /note="nonanucleotide"
     misc_feature    776..784
                     /note="nonanucleotide"
     CDS             complement(1568..2977)
                     /codon_start=1
                     /product="Putative CruV Capsid Protein"
                     /translation="MRKQYTDKEKVAYYKALALQKDIASGKLVLPSSRPRRQPAKTTA
                     PRTKKSTSGMYGQKKTYNNQQIARLGDFGGLAGSTLGTLVGGPLGGVIGGGLGTLAGG
                     LFKHFVGKGDYQVKNNAYLSKEGQLMTPVINKHRGGGDVFRRSEYLGDIISSATANTF
                     NLQSFDVNPGLESTFLWLSQIAANYEEYEFEGMYFEFRSMSADALNSVNTALGQVVCA
                     ANYNAASPNFANKQAMENYEGGVSVRPSESVRYFVECAKNKNVLSELYVRQGAVPTGQ
                     DQRLYDLCNFQIATNGMQGTSVNVGELWVCYQVVLRKPKMFSAFGLYNSWYSASPSTG
                     AYTNALPLGSSGAWTLGVANNLIGATITGTVITFSPPNIPQAYQMFLQWTGSSTAITY
                     PSLTLSNGLVLSSTFTSPAAGETCTRCSREVLMEYLPQNNAAFLPPSITLGTAGTLPS
                     AGSAFTVYIVQIPNTLFAI*"
ORIGIN
        1 atggaagaat ataacgcatt attcgataaa aattacgatg aaactacatt taaaggacaa
       61 tcacaatatt ggacttttgt atcgtgggaa gatccaaaat gtgaagatct atcggactgg
      121 tttacctata ttgtatacgg taaagaaatc acacctacta caggtaaatt acattggcaa
      181 ggctacatcg aatgcgtagc tatgttacgc gtaggtacac tacgtaaaaa attcttaaag
      241 gaatttaaaa acgttaagaa tataaaattc ttaaaacgct atatacgttc tactggtgaa
      301 cagaatcgta tgtactgttg taaaggtctt caatcacatg aagaatatat tgaatttaaa
      361 gataaaggtg aaaattatgg tcttaataaa gagaccgtag aatttggtga aatctttaaa
      421 cctgagcccg gtaaacggtc tgacttgaaa ggcattagtg aagccatcaa ggacaacagc
      481 agtattaaac ataatattga taatgaaatt atcactacag gtcctgcatt aaaaacattc
      541 ggtgcgctga ctccttatta tgaaaaaaaa agaagactag ctaaaccacc gatagtatac
      601 gttattttcg gtgatgcagg ttcgggtaaa accgaatggt gcagaaacat ggcagagtct
      661 cttaaccttg gtaacattta tgaagattat agaggtggag gttactggca gggttatgat
      721 aacgaacaaa tagtactatt tgacgatttt agagacacat ggatggattt tgaattacta
      781 ctaaaaatcc ttgacaaagg cccagtcacc ttgggatgca agtttggatc ccgacaatta
      841 gcagcaagca tatttttcat tacatgtagc aagcaccccc gagaatggta taagggtgta
      901 tcagaatcta ggtatcaatt ataccgtaga atttcgatat gtttccattt cgaatttgag
      961 ataggatgga actgtttcaa acaaatgaaa cttaatgaat gggatgcatt taaagttaag
     1021 atgacaccag tttctttaga aaaagaagaa gactacgtta aaaataccga ttttggttgg
     1081 taaagtattt ttgtgagttg tgagttgtga ctgaggttgg gtaagtaata gtagttaccc
     1141 aactcccaat tttttcttat cgatacttac gtataaacta tgggatcctt ccgataacgg
     1201 catcccaaac gtacctcata tgaggtttct tgttttcttc cctatgcatg attgatcgct
     1261 acatgcgata aatggaatat attattcagg taatctgaat aataatataa gaaacaagca
     1321 tataagagta aatcacacga aaatggtgaa catcatattt tatgtggaac cgaaggttcc
     1381 ccacaaaata tgaagattca cattttgtgt gagccattac gcttatttgg ttctttcata
     1441 tgttactatg catcatacat aaacgtaaca tatgttcttg cttcttgcac tgtgcatgat
     1501 caataagggt atagcaacag tattattttt tatgttattt ttaacataaa acttacgatt
     1561 attttactta gatagcaaaa agagtattag gtatttgtac tatatataca gtgaaagcag
     1621 atccagcaga tggtaaagtt ccagcagtac cgagagttat tgatggaggc aaaaatgccg
     1681 cattattttg tggtaaatat tccattaata cttctcgaga acaacgagta catgtctctc
     1741 ccgcagcagg acttgtaaaa gtagaagata aaacgagtcc attcgataat gttaacgaag
     1801 gatacgttat agcagttgat gaacctgtcc attgaaggaa catctgatat gcttgtggaa
     1861 tatttggagg tgaaaaagtt attactgttc cagtaattgt agcaccaatc aaattattag
     1921 ctacaccgag agtccatgct ccggaactac caagaggtaa agcatttgta taagcaccag
     1981 ttgatggact agcagaatac catgaattat acaatccaaa agctgaaaac atctttggct
     2041 ttcttaatac gacttgatag cacacccata attcacctac gttaactgac gttccttgca
     2101 ttccattagt ggctatttga aaattacata aatcatatag acgttggtct tgtcccgttg
     2161 gtactgcgcc ttgtctaaca tataattcac tgagaacgtt cttattttta gcacattcta
     2221 caaaatatcg aacactttca gatggtctaa cggaaacacc tccttcgtag ttttccattg
     2281 cttgtttatt agcaaaattt ggagatgcag cattataatt tgctgcacat acaacttgac
     2341 cgagagcagt attaacagaa ttcaatgcat cagctgacat tgatctaaat tcgaaataca
     2401 taccttcgaa ctcatattct tcataattag ctgctatttg agataaccat aaaaatgttg
     2461 attcgagtcc aggattgaca tcgaacgatt gaagattaaa tgtatttgca gtagcagatg
     2521 atataatatc acctaaatat tcggatcgtc taaatacatc tcctcctcct ctatgtttgt
     2581 taataactgg tgtcattaac tgaccttcct tactaaggta agcgttattc ttgacttgat
     2641 aatcaccttt ccctacaaaa tgtttaaaaa gacctcctgc gagggtacct agtcctcctc
     2701 ctatcactcc tcctaatgga ccacctacta atgtaccaag agtacttcct gctaaacctc
     2761 cgaaatctcc gagtctagct atctgttgat tattatacgt cttcttttgg ccgtacattc
     2821 cgcttgtaga tttctttgtg cgtggtgcag tcgtcttagc gggttgtctt ctaggtctag
     2881 aagaaggtaa aacaagttta ccagatgcaa tatccttttg aagagcaagt gctttataat
     2941 aagcaacttt ttccttatcg gtatattgtt tacgcatggg tatcacagtg ctagatatag
     3001 cttatatact agtacgcatc atttaaattc attttttgtg ttaaaaaaat gtgactgagg
     3061 ttataagc
//
LOCUS       CruV-143                3069 bp    DNA     circular     24-FEB-2020
DEFINITION  Crucivirus-143 isolate 120_1045_SvL1_3295.
ACCESSION   CruV-143
KEYWORDS    .
SOURCE      Crucivirus-143
  ORGANISM  Crucivirus-143
            Unclassified.
REFERENCE   1  (bases 1 to 3069)
  AUTHORS   de la Higuera,I., Kasun,G.W., Torrance,E.L., Pratt,A.A.,
            Maluenda,A., Colombet,J., Bisseux,M., Ravet,V., Dayaram,A.,
            Stainton,D., Kraberger,S., Zawar-Reza,P., Goldstien,S., Briskie,J.,
            White,R., Taylor,H., Gomez,C., Ainley,D.G., Harding,J.S.,
            Fontenele,R.S., Schreck,J., Ribeiro,S.G., Oswald,S.A., Arnold,J.M.,
            Varsani,A., Enault,F. and Stedman,K.M.
  TITLE     Direct Submission
  JOURNAL   Submitted (24-FEB-2020) Biology, Portland State University, 1719 SW
            10th Ave, Portland, Oregon 97201, USA
COMMENT     Bankit Comment: ALT EMAIL:ide@pdx.edu
            Bankit Comment: TOTAL # OF SEQS:451
            Bankit Comment: GAP: est. length - alignment

            ##Assembly-Data-START##
            Assembly Method       :: MetaSPAdes v. 3.12; IDBA-UD v. 1.1.1;
                                     MEGAHIT v. 1.0.3
            Sequencing Technology :: 454; Illumina
            ##Assembly-Data-END##
FEATURES             Location/Qualifiers
     source          1..3069
                     /organism="Crucivirus-143"
                     /mol_type="genomic DNA"
                     /isolate="120_1045_SvL1_3295"
                     /isolation_source="Water"
                     /country="Norway"
                     /collection_date=2012
     CDS             1..1032
                     /codon_start=1
                     /product="Putative Replication Associated Protein"
                     /translation="MSLENGVIGGDVDGNTISSTSVVKSLKRSNQCIYWFFTYNNYKI
                     EEMEGISLVFKEICLEYIFQEEIGTAGTKHLQGSIKLIKPMRWTEFKLPKGIHWEPTR
                     NIQSANMYCCKDETRNGQRFEWEKYPVRKLKIINNLRPWQLELFNIMKTEPDDRLIYW
                     IYNKSGNIGKTVFTKYLHSKLKNLFVVTQGSCKDVACMLAQAKENGTKLNEITTFIFH
                     YSRTTEKINYKCLESIKDGIISSPKYESTTLIMNCPHTFVFSNELPELDRLTLDRWCI
                     YNVVDTDGSLIKMSLDQVEFERNVCEQIIDEIPLWEEDEKNNLVEEKIFELERRKIND
                     KEWINFKDS*"
     misc_feature    394..416
                     /note="stem-loop"
     misc_feature    399..407
                     /note="nonanucleotide"
     CDS             complement(1561..2976)
                     /codon_start=1
                     /product="Putative CruV Capsid Protein"
                     /translation="MPRKPRVVRKGGKKVARKRYPKSGGSSTMSNLLKIGAGAALGSL
                     GAAAMGPLGRIAGEGAVGLYDRLPSWNALSEMAENAGLAYKVHQADDALKREESHLRS
                     NYRRNYGGISGQGDYVVNGNQILSGSMPKFGHSNGSIIIEDCEYLGDVISSNVADTFA
                     IHEYELNPGNEGTFAKLSQIAMNFEQYEFLGCVFQFKSKSADALNSTNTALGSVLMCA
                     LYDVADTNFGSKSEMLNYEFCQDCKPSENAMCMIECKPSQSTINRLYVLNGAVPSGKD
                     PRLYNFASFSIATMGMQGTNVNLGELYVSYRVRLLKPKLYAFYARNANTSHFSNPTGV
                     SATAPLGTVATISTLVDTIGITFDSPVQLRLTGPRNNGITTYFAQFLVAGNSASVVAP
                     LITYTNCTTIASFAMSSGTTTTQQFPVSGAASVQLLYQTIFTTDGKGLDPTLVFGVAG
                     TFPGSANASSVYIAQISTDSI*"
ORIGIN
        1 atgtcacttg aaaatggagt cattggaggg gacgtggatg gtaatactat ttcatccacg
       61 tctgtagtaa aaagtttgaa acgatcaaat caatgcattt attggttctt tacttacaat
      121 aattacaaaa tagaagaaat ggagggcatt tctctagttt ttaaagaaat atgtttagaa
      181 tacatttttc aagaagaaat tggaactgct ggtactaaac atttacaagg atctattaaa
      241 ttaatcaaac ctatgagatg gactgaattt aaactgccaa aaggtataca ttgggaacct
      301 accagaaaca ttcaatcggc taacatgtac tgttgcaaag atgaaacgag aaatggacaa
      361 agattcgaat gggaaaaata ccctgttaga aaacttaaaa ttattaacaa tttaagacct
      421 tggcagttag agttattcaa cattatgaaa actgaacctg atgacagatt aatttattgg
      481 atatacaata aatctggaaa cattggaaaa actgttttta ctaaatattt acattctaag
      541 ttaaaaaact tatttgttgt tactcaagga tcatgtaaag atgttgcttg tatgttagca
      601 caagctaaag aaaatggcac taagcttaat gaaattacca catttatttt ccattatagt
      661 agaacaactg aaaaaattaa ttataaatgt cttgaatcta ttaaagatgg tattataagc
      721 tcacctaaat atgaaagcac tacattaatt atgaactgcc ctcacacttt tgtgtttagt
      781 aatgaactgc ctgaattgga cagattgact ctagaccgat ggtgtatcta taatgtggtg
      841 gacactgacg ggtcacttat taaaatgtca cttgatcaag ttgaatttga aagaaatgtt
      901 tgtgaacaaa tcattgatga aataccttta tgggaagaag atgaaaaaaa caatttagta
      961 gaagaaaaaa tttttgaatt agagagaagg aagataaatg ataaagaatg gattaatttt
     1021 aaggatagtt gaaagattat ctcgtgaaga gataataact ttaatcagaa gattttattg
     1081 agatgccaaa aaagacacaa tttaaattgt atttgataaa aaagcaactt tgttccaaag
     1141 tttctgcttt tttctcaaat aaaattcaaa gtgtatattt tttgatccct acaacaaaac
     1201 atcctattaa tcttattcct actcacttac tatacaagat tatacttgaa tgttatgttt
     1261 actgtagtaa acataactta ggaagttagt gaagtagttg tagttattaa ttctttattt
     1321 gcgtcgccga ctgaggcttt ctatgaagaa cgactgattt ttttaaagta gtaaggtgtt
     1381 acctttaata ctttacccat tatagatact cattttaaag attgaaagat tgggttactt
     1441 ggagcacaag tgcacagtat aaacatttac atatattgta aacatatata aagttttact
     1501 tttagacaag tatcacacta acagtgtgat agggcacgaa cagtgcccta accgattgaa
     1561 ttatatactg tcagtagata tctgagcaat atatacactt gatgcattgg ctgatcctgg
     1621 aaaggtacct gctacaccaa acactaatgt tgggtctaat cctttaccat ctgtagtaaa
     1681 tattgtttga tacaataatt gaactgatgc tgcaccagat actggaaatt gctgggttgt
     1741 tgtggttcct gatgacatgg caaaacttgc aatggtcgta cagttagtat acgtgatcaa
     1801 gggagctact actgaagcag aattacctgc tactaaaaat tgagcaaaat aagtggttat
     1861 accattatta cgtggacctg ttaaacgtaa ttgaactggt gaatcaaatg tgatccctat
     1921 tgtatcaact aaagttgata ttgttgctac tgtacctaaa ggagctgtgg ctgatactcc
     1981 tgtcggatta ctaaagtgag atgtattggc atttcttgca taaaaagcat agagcttagg
     2041 ctttaataat ctaactctat aacttacata caattctccc aaatttacat tggttccttg
     2101 cattcccatg gtagctatgg aaaaagaggc aaagttgtac aaacgtggat cctttccact
     2161 tggaacggca ccatttaata catataacct attaatggtg gattgagatg gtttacattc
     2221 aatcatacac atggcgttct cacttggctt acaatcttgg caaaactcat aatttaacat
     2281 ttctgacttt gaaccaaaat ttgtatcggc tacatcatat aaagcacaca ttaatactga
     2341 accaagagca gtattggtac tgttcaaagc atctgctgac tttgacttaa attgaaatac
     2401 acaacctaaa aattcatatt gttcaaaatt cattgctatc tgagataact tggcaaaggt
     2461 accctcatta cctggattca attcatactc atgaattgca aatgtatccg ctacattgga
     2521 agagattaca tctcctaagt actcacaatc ctctattatg atacttccat tagaatgacc
     2581 gaactttggc atagatcctg ataggatttg attaccgttt actacataat caccttgacc
     2641 ggatatacca ccataattac gcctgtaatt agaccttaag tgagattcct ctcgcttaag
     2701 agcatcatct gcttgatgta ccttataagc taaacctgca ttctctgcca tttctgacag
     2761 agcattccat gatggtaacc gatcatataa tcctacagct ccctcgcctg ctatacgacc
     2821 taaaggtccc attgctgccg ctcctagaga acctagtgca gcaccggctc ctatctttaa
     2881 cagattggac attgttgatg acccacctga cttaggatac cttttccttg ccactttttt
     2941 accccccttt ctaacaactc tcggctttct tggcatttta tactatagta ttataattaa
     3001 ttattgatat aaaataattt attataaata tacttaaaga tttattttct cattctaagt
     3061 catattaaa
//
LOCUS       CruV-144                3070 bp    DNA     circular     24-FEB-2020
DEFINITION  Crucivirus-144 isolate 120_210_SvL2_3298.
ACCESSION   CruV-144
KEYWORDS    .
SOURCE      Crucivirus-144
  ORGANISM  Crucivirus-144
            Unclassified.
REFERENCE   1  (bases 1 to 3070)
  AUTHORS   de la Higuera,I., Kasun,G.W., Torrance,E.L., Pratt,A.A.,
            Maluenda,A., Colombet,J., Bisseux,M., Ravet,V., Dayaram,A.,
            Stainton,D., Kraberger,S., Zawar-Reza,P., Goldstien,S., Briskie,J.,
            White,R., Taylor,H., Gomez,C., Ainley,D.G., Harding,J.S.,
            Fontenele,R.S., Schreck,J., Ribeiro,S.G., Oswald,S.A., Arnold,J.M.,
            Varsani,A., Enault,F. and Stedman,K.M.
  TITLE     Direct Submission
  JOURNAL   Submitted (24-FEB-2020) Biology, Portland State University, 1719 SW
            10th Ave, Portland, Oregon 97201, USA
COMMENT     Bankit Comment: ALT EMAIL:ide@pdx.edu
            Bankit Comment: TOTAL # OF SEQS:451
            Bankit Comment: GAP: est. length - alignment

            ##Assembly-Data-START##
            Assembly Method       :: MetaSPAdes v. 3.12; IDBA-UD v. 1.1.1;
                                     MEGAHIT v. 1.0.3
            Sequencing Technology :: 454; Illumina
            ##Assembly-Data-END##
FEATURES             Location/Qualifiers
     source          1..3070
                     /organism="Crucivirus-144"
                     /mol_type="genomic DNA"
                     /isolate="120_210_SvL2_3298"
                     /isolation_source="Water"
                     /country="Norway"
                     /collection_date=2012
     CDS             1..1089
                     /codon_start=1
                     /product="Putative Replication Associated Protein"
                     /translation="MSSTQVVPTIVDVPRRRSTQTSLSLAWVFTFNNPTTTMKVPQKT
                     PAELEDLLKPIASFYIFQLEVGEKGTPHYQGFIRLKKALRLSALRKIVQMNWQVRYPK
                     GSDKKAAAYCMKEVGRLDGPWQYGDLPANEQGKRSDLVRVFESIKSGMTIDEIIEEHP
                     AAYIRYHAGIKDIVSRRKTVKPVPRVSWFHGPTGTGKTHRARGLDVGLDQNLVFVTNA
                     DKDRIFEGYECDQTRVVIDEYSPAKIPFVALKQYIDKYPTKVKVLYGARDFVPEDIVI
                     TCPRAPNEIWTGNPLNEITRRLNGGIHLFDVPYVEPIEEPTRPVVDEMQTEAEDVEIE
                     SSIDTTEDFEGRYDPIVLNKTSIIRKSK*"
     CDS             1145..1567
                     /codon_start=1
                     /product="putative ORF"
                     /translation="METTSTNDVPRMTDPRLADPRQIIVTVGPDISTLTQLADWAKMR
                     LGYRPEPAIWAALKAVNPQIKPKRKVTSASAKRVAANKRRRTTYTPQPRYTGRGGGSA
                     NIIYPNVTGLGAYDVTGYANVDLGDTFRGGLQGNFSLD*"
     CDS             1684..2751
                     /codon_start=1
                     /product="Putative CruV Capsid Protein"
                     /translation="MGSVDLGTSPPTVRNTHKGEAFVLSHREYLGDLLSANNGPPTLF
                     EVTKTFALNPGNPDLFPWLAPIAAMFQEYEIRGMLVELKTLSSDFTAALNMGAVFMSA
                     DYNVLDAAPIDKKHLENMEYASSCKPSCSLIMPIECDPKNDSNTHLYIARNEVYEGGD
                     KRLFDLCNIFIGSQGIPVANAPIAEIWVTYEVALFKPIIPDIVSEIIAGAHFQLISAT
                     VAKPLANPVGSPANASDITITTNDTIHFPGTVGSRWLVLVDWSSVNANFGVALGTTTL
                     TGGVGTPPLFATGGGNNLSQIFDVPNTAAALTATQNMAYHAIITVTAPGCTLAFNHDW
                     TFPAATFGDVHITQYPLDILT*"
ORIGIN
        1 atgtcctcta cccaagtcgt accaactatt gtcgacgtac ctcgacgtcg ctcgactcag
       61 acgtccttat cgttggcttg ggtttttacc tttaacaacc caacgacgac gatgaaggtc
      121 cctcaaaaaa cgcctgcgga gctagaggat ttgcttaaac ctatagcgtc tttctacata
      181 tttcaacttg aagttggaga aaaagggacc cctcactatc aaggatttat tcgactgaag
      241 aaagccttgc gattgtccgc cttgcgaaaa atcgtacaaa tgaattggca ggtccgttat
      301 cctaaaggat ctgacaagaa agcggccgct tattgtatga aagaagtagg ccgattggac
      361 ggaccttggc aatacggcga tctaccggcg aacgaacaag gcaaacgtag cgatctagtg
      421 cgcgtgtttg agtccataaa gtctggcatg acgatcgacg agattatcga ggagcatcct
      481 gccgcctata tccgttatca cgcagggata aaggatattg tgagccgtcg taagacggtg
      541 aaacctgtgc cgcgtgtgag ttggtttcac ggtcctactg gaacgggtaa aacgcaccgt
      601 gcgcgcggct tggacgttgg tctcgatcaa aaccttgttt tcgtgaccaa cgctgataaa
      661 gatcgtattt ttgaaggcta cgaatgtgat caaacacgtg tcgtgattga tgaatattcg
      721 cctgcgaaga ttccgttcgt agccttaaaa caatacatag ataagtatcc gacgaaggtg
      781 aaggtcctct atggagctag agacttcgtc ccagaggaca tcgttataac gtgtcctcgt
      841 gcaccaaatg aaatttggac gggtaatccg ttaaatgaaa ttactcgccg tcttaacggc
      901 ggtattcatt tgttcgacgt tccatacgtc gaaccgatcg aagaaccgac tcgaccagtc
      961 gtagacgaaa tgcaaacgga ggcggaagac gtggagatag aatcttcgat cgatacgacc
     1021 gaagatttcg aaggtcgtta tgatccgatc gttttgaata aaacgtcgat cattagaaag
     1081 tcgaaataaa aattttttta ttgtagagtt tacgtttttt ttgtaagtca ctgacggaaa
     1141 aaaaatggaa acgacatcga cgaacgatgt ccctcgaatg accgatcctc gattggctga
     1201 tcctagacag ataatcgtga ctgtcgggcc cgatatctcg actcttacgc agttggccga
     1261 ctgggccaaa atgaggttgg gatatcgtcc tgagccggcg atttgggccg ctcttaaggc
     1321 tgtgaacccc caaatcaagc ctaagcgtaa ggtgacgtct gcttctgcaa aacgagtcgc
     1381 cgctaataag cgacgacgaa cgacgtatac tcctcaacct aggtataccg ggcgaggagg
     1441 aggatcggcg aatataatct atcctaacgt caccggcctt ggcgcgtatg acgttaccgg
     1501 gtatgcaaat gtcgacttgg gcgacacgtt tcgcggaggt ttacaaggta atttttcttt
     1561 agattaactt gggactggag tttgttcccg aaaggatctc ccgttaacct tggtttctca
     1621 gggagattat acagcgacgc tgtaaccggt ctaggcggtt atcaagtcaa tcggaattcc
     1681 cttatgggat ccgttgatct tggaacttct ccgccgaccg ttcgaaatac gcacaaaggc
     1741 gaagctttcg tcctttctca tagagagtat ttgggggatc tcttgtccgc caacaacggc
     1801 ccgccgacgt tgttcgaagt aaccaaaacc tttgcgttga atccgggtaa cccggattta
     1861 ttcccgtggt tagcgcctat cgctgccatg tttcaggaat acgaaattcg ggggatgttg
     1921 gtcgaactta agaccttatc gtccgatttt acggcggcgt tgaatatggg tgccgtattc
     1981 atgtccgccg attacaacgt tctcgatgcg gcgcctatcg ataaaaagca tctagagaac
     2041 atggaatatg cgagctcttg taagccgtcg tgttctttga tcatgcctat agagtgtgat
     2101 ccaaagaacg actctaatac gcacttgtac atcgcccgta acgaagtgta cgaaggcggt
     2161 gacaagcgat tgtttgactt gtgcaatatc tttattggca gtcaaggcat acctgtggcg
     2221 aatgctccga tcgccgaaat atgggtgact tacgaagtag ccctatttaa accgattatc
     2281 cccgatatcg ttagcgagat tatcgccggt gctcactttc agttgatctc cgcgacggtt
     2341 gccaaacctc tcgctaatcc ggtcggtagc cctgctaacg cttcggatat tacgattaca
     2401 accaacgaca ccatccactt tcctggaacc gtaggatcca ggtggttggt tcttgtcgat
     2461 tggtctagcg ttaacgctaa ctttggcgtt gcccttggaa cgacgacctt gacgggaggc
     2521 gttggaacgc cgcctctttt tgcgacgggg ggaggcaaca atctttctca aatttttgac
     2581 gtccccaata cggcggccgc tttgacggcc acccaaaata tggcttatca tgccattata
     2641 acggtgactg cacctggttg cacgttagcg tttaatcatg attggacgtt cccagctgca
     2701 acttttgggg acgttcatat cactcaatat cctctcgata ttttaacgta aataaaattg
     2761 acgtttattt tttttttgtc ccgtgtacgt gtaccaaaaa aaaagcacgc gtgtacatgt
     2821 atgcgctcgt gccttttttt accctcctgt cctttggagc caagggaagg gccctatggg
     2881 ccctgaccgc aggcccattg gactttcccg tgtacatggg ataagtccac caaaaaagtc
     2941 cgctggctaa aaaaagtgat gttcactttc ggcgaaaaac tttttcgctc tatataagga
     3001 agtggacacg gaacgaagtt cctattatta ctgtccactt ccacatctca catcacgtct
     3061 actttgtgag
//
LOCUS       CruV-145                3076 bp    DNA     circular     24-FEB-2020
DEFINITION  Crucivirus-145 isolate 00039_SRR2063921.
ACCESSION   CruV-145
KEYWORDS    .
SOURCE      Crucivirus-145
  ORGANISM  Crucivirus-145
            Unclassified.
REFERENCE   1  (bases 1 to 3076)
  AUTHORS   de la Higuera,I., Kasun,G.W., Torrance,E.L., Pratt,A.A.,
            Maluenda,A., Colombet,J., Bisseux,M., Ravet,V., Dayaram,A.,
            Stainton,D., Kraberger,S., Zawar-Reza,P., Goldstien,S., Briskie,J.,
            White,R., Taylor,H., Gomez,C., Ainley,D.G., Harding,J.S.,
            Fontenele,R.S., Schreck,J., Ribeiro,S.G., Oswald,S.A., Arnold,J.M.,
            Varsani,A., Enault,F. and Stedman,K.M.
  TITLE     Direct Submission
  JOURNAL   Submitted (24-FEB-2020) Biology, Portland State University, 1719 SW
            10th Ave, Portland, Oregon 97201, USA
COMMENT     Bankit Comment: ALT EMAIL:ide@pdx.edu
            Bankit Comment: TOTAL # OF SEQS:451
            Bankit Comment: GAP: est. length - alignment

            ##Assembly-Data-START##
            Assembly Method       :: MetaSPAdes v. 3.12; IDBA-UD v. 1.1.1;
                                     MEGAHIT v. 1.0.3
            Sequencing Technology :: 454; Illumina
            ##Assembly-Data-END##
FEATURES             Location/Qualifiers
     source          1..3076
                     /organism="Crucivirus-145"
                     /mol_type="genomic DNA"
                     /isolate="00039_SRR2063921"
                     /isolation_source="Rhinolophus affinis pharyngeal and
                     analswab"
                     /country="China"
                     /collection_date=2013
     CDS             1..1428
                     /codon_start=1
                     /product="Putative Replication Associated Protein"
                     /translation="MDHNAHEIVNAIRARRWCFTINNYSEEDLARCQDAINDGVCRYI
                     CFGLEIAPETGTPHIQGYLECAGQKARRTLSKAFPRAAFFPAKGSAEQNIDYCSKEGK
                     FFEDGDNPLAPAEDGSLKGQGRRNDIHRVKQIVTEGGGMADIIDECSSYQSLKAGELL
                     LKYSEPKRAVKPVVIWIHGPTGTGKSALAGELAPDAWWTAKSLKWWDGYDAHRNVVID
                     DFRADYCTFHELLRILDRHPYRVETKGGSRQLLATRIVVTSPFPPDHVYREREDIGQL
                     LRRIDRTIFVEAPIPPKVDPQAEGEFNRELNAVDSILSQYGHSPTVSEVEGNILPATP
                     HPSGSAQPDSTPQLPKHVDNVAVASHADINEALCELMGCNIESNNPYDHDYDEKPPIV
                     EPIYKPPTRDLPPKFPTNGEINKKQAEYGWNMLAGEPDHSIICFCKNCIKDTPKRETP
                     PAIATITPSARPSGKKAAPPRRKGR*"
     CDS             complement(1633..3009)
                     /codon_start=1
                     /product="Putative CruV Capsid Protein"
                     /translation="MSYRPYQPRSYGRTYVRGRGSYYAAPAPVRRRAPARRRAPVMYE
                     TIPRAPRIRGNGKYRVFDAPRLGTGLGSLTGGVLGNMIAPGVGGAIGAQAGGFIGNAL
                     GQGFRSITGFGDYTIHENSLVYPDRVVPSFGDDSIRVKKREFIANIDATTAFANNFFP
                     INPGLNDTFPWLSSIANNYEQYRWNGLVFQFVSTSSDAIASTTDLGLGQVVLATDYNA
                     ADAAYVNMPQMLGTMFANSGKPSQNIMHALECAPAEQAQKLYYVRSGGHPDGTDIRMY
                     DMGSFQLATENMPANYDGMGQLWVSYDVTFCKSVQNNQLGFDLNTDKYQLVAPTVTTA
                     YFGTSRTAADHNNLGTTVSATAIAFPPTLSSGYYEISYQAEGASTAVVTPTFTAVNCT
                     ALTVWSNNTSSAMTNSGSTSTKMIVTRIYRIDDRDATITLSGGTLPTVGTTGDLVITQ
                     INGEVFLA*"
ORIGIN
        1 atggaccaca acgcgcacga aatcgtcaac gctatacgcg ctcgcagatg gtgcttcacc
       61 atcaacaact acagcgagga agacctcgct cgatgccaag acgccatcaa cgacggcgta
      121 tgcagataca tctgctttgg cctcgaaatc gccccggaaa ccgggacccc acacattcaa
      181 ggataccttg aatgcgccgg acaaaaagcg cgcaggaccc taagcaaagc tttcccgcgc
      241 gctgccttct tccccgccaa aggctcggcc gagcagaaca tagattactg ctctaaagaa
      301 ggaaaattct tcgaagacgg cgacaatccc ctggctcccg ctgaagacgg gtccctcaaa
      361 ggccaaggcc gccgcaacga tatccatcgc gtcaaacaaa tcgtcactga aggtggcggc
      421 atggccgaca tcatcgacga atgcagctct taccagagcc tcaaagccgg tgaactacta
      481 ctaaagtaca gcgagcccaa acgcgcggtt aagcccgtcg tcatttggat tcacggcccc
      541 accggcactg gcaaaagcgc tctcgcgggc gaactcgccc cagacgcctg gtggacagct
      601 aaatcgctaa aatggtggga cggttacgac gcccaccgga acgtggtcat cgacgacttt
      661 cgcgctgact attgcacgtt ccacgaactg ctccgcattc tcgatcgcca cccctaccgg
      721 gttgaaacta aaggtggctc tcgccaactc ctcgcgacac gcatcgtcgt tacctccccc
      781 ttcccccctg atcatgtcta tcgcgaacgc gaagacatag ggcagctcct ccgtcgcatc
      841 gaccgaacaa tcttcgtcga agcccctata cccccaaagg tggaccccca agccgagggg
      901 gagttcaacc gcgaactaaa cgcggttgac tcaattctaa gccagtatgg gcatagccca
      961 actgtctcag aagttgaggg taatattctg ccggctaccc ctcacccttc gggctcggcc
     1021 cagccagaca gtacccctca acttccaaaa cacgtcgaca acgtcgccgt agcatcccac
     1081 gcagacataa acgaagctct ctgcgaactt atgggatgca acatcgaatc aaacaacccc
     1141 tacgaccatg attacgatga aaaaccccct atcgtggagc caatctataa accacctacg
     1201 cgggacttac cgccaaaatt cccaacaaac ggcgaaatca acaaaaaaca agccgaatat
     1261 ggctggaaca tgctcgccgg cgaacctgac cactctatta tatgcttctg caaaaactgc
     1321 ataaaagaca caccaaaacg cgaaacacca ccggccatcg caactatcac accttcggct
     1381 cgcccaagcg gcaaaaaagc cgcaccgcct cgtcgtaaag gcagataagc ccgcccacac
     1441 gcacctaccg gtgcgaacca cccctcgtcg ggtcgttacc agtaccgggc ttcgcccggt
     1501 acacacaacc actcgtcggg gatggctgcc acgattgtgg gcccatttaa catttatccc
     1561 gcgccacctc ctggaaacca gacgataaca cttggccgcg ggacgcccaa aaagtacccc
     1621 ttttgtgggc tcttacgcaa ggaaaacctc cccgttgatc tgtgttatca ccagatcacc
     1681 ggtcgttccc actgtcggca acgtccctcc cgacaacgta atcgtcgcgt cgcggtcatc
     1741 tatacgatat atacgcgtaa ctatcatctt cgtcgatgta ctaccgctat tcgtcatagc
     1801 cgacgaagtg ttattcgacc acaccgtcag cgccgtgcaa ttcacagcag taaacgtcgg
     1861 agtcacgacc gcagtcgacg ctccttccgc ctgatacgat atctcgtaat aaccagaact
     1921 aagcgtcgga ggaaacgcaa tagccgtagc acttacagtc gtacctaaat tattatgatc
     1981 cgcagcagtt cgactcgtac caaagtacgc cgtcgtcacc gtcggcgcaa ccaactgata
     2041 cttatccgta ttcaagtcga aaccaagctg gttgttctgc acggacttac agaacgttac
     2101 atcgtaacta acccaaagct gacccatgcc gtcatagttc gccggcatgt tttccgtcgc
     2161 gagctggaac gaacccatat cgtacatacg aatatcggtg ccatcaggat ggccaccgga
     2221 acgcacgtaa tacaactttt gagcctgctc agctggcgca cactccaaag catgcattat
     2281 attctgcgac ggcttaccac tgttggcgaa catcgtaccc aacatctgcg gcatgttcac
     2341 atacgccgca tccgcggcat tatagtccgt ggccaacaca acctgcccaa gacctaaatc
     2401 agtcgtagac gctatcgcgt ccgaactggt actcacaaac tgaaacacga gcccattcca
     2461 tcgatactgc tcgtagttgt tcgcaatgct cgacaaccac ggaaacgtat cattcaaccc
     2521 aggattgatc gggaagaagt tattcgcgaa cgcagttgtt gcgtcaatat tcgcgataaa
     2581 ctcacgcttc ttcacccgaa tcgaatcatc gccaaagctc ggaaccactc gatcaggata
     2641 caccaaagaa ttctcgtgaa tcgtataatc accaaaacca gtgatcgatc ggaacccttg
     2701 ccccaaagca ttcccgataa aacctccagc ttgcgcacca attgcgcctc cgactcccgg
     2761 cgcgatcatg tttcccaaca cgccgcccgt caacgaacct aatccggttc ccaatcgcgg
     2821 agcatcaaac acccgatact tcccattccc acgaatacgc ggggcccttg gaatcgtctc
     2881 atacataacc ggagcacggc gcctagccgg cgcacgacgc ctaaccggcg ccggtgctgc
     2941 ataataagaa ccacggccgc gcacatacgt gcgcccatag gaccgcggtt gatacggtcg
     3001 ataactcatg ctgtcgagat actacagcaa agataattag ctaaaagcta attatatacg
     3061 ctgcacatgc agcttc
//
LOCUS       CruV-146                3084 bp    DNA     circular     24-FEB-2020
DEFINITION  Crucivirus-146 isolate H1_85280.
ACCESSION   CruV-146
KEYWORDS    .
SOURCE      Crucivirus-146
  ORGANISM  Crucivirus-146
            Unclassified.
REFERENCE   1  (bases 1 to 3084)
  AUTHORS   de la Higuera,I., Kasun,G.W., Torrance,E.L., Pratt,A.A.,
            Maluenda,A., Colombet,J., Bisseux,M., Ravet,V., Dayaram,A.,
            Stainton,D., Kraberger,S., Zawar-Reza,P., Goldstien,S., Briskie,J.,
            White,R., Taylor,H., Gomez,C., Ainley,D.G., Harding,J.S.,
            Fontenele,R.S., Schreck,J., Ribeiro,S.G., Oswald,S.A., Arnold,J.M.,
            Varsani,A., Enault,F. and Stedman,K.M.
  TITLE     Direct Submission
  JOURNAL   Submitted (24-FEB-2020) Biology, Portland State University, 1719 SW
            10th Ave, Portland, Oregon 97201, USA
COMMENT     Bankit Comment: ALT EMAIL:ide@pdx.edu
            Bankit Comment: TOTAL # OF SEQS:451
            Bankit Comment: GAP: est. length - alignment

            ##Assembly-Data-START##
            Assembly Method       :: MetaSPAdes v. 3.12; IDBA-UD v. 1.1.1;
                                     MEGAHIT v. 1.0.3
            Sequencing Technology :: 454; Illumina
            ##Assembly-Data-END##
FEATURES             Location/Qualifiers
     source          1..3084
                     /organism="Crucivirus-146"
                     /mol_type="genomic DNA"
                     /isolate="H1_85280"
                     /isolation_source="Water"
                     /country="New Zealand"
                     /collection_date=2012
     misc_feature    join(3072..3084,1..21)
                     /note="stem-loop"
     misc_feature    1..9
                     /note="nonanucleotide"
     CDS             60..1019
                     /codon_start=1
                     /product="Putative Replication Associated Protein"
                     /translation="MAAAKQARNWCFTVNNWKEADLECLKKLEGSQVKYMVYGKEVGD
                     SGTPHLQGFVIFLNPKRFSEVKKLLPWGCHIEQAKGSWEHNLAYCTKEDTNAYVWGEA
                     PMSRKRQGEAGAEAEKERWTKIRALAKAGKIEELEVDFPREAILYEEKFARIAMKCAP
                     RADLPPETVVGVWIHGPSGAGKSYWCHHNLEVDGVTYRIEEIYDKDLSQWWDGWRSRD
                     ETEDRLKHEVAYLDDLDKFCIKMARDLKVWTQEYEFNAQVKGGYQRIRPKLVVVTSQS
                     TIEEIWDDDKTRCALLRRFKVGYKANRDAPVVWEPKYVPTFFA*"
     CDS             1271..2689
                     /codon_start=1
                     /product="Putative CruV Capsid Protein"
                     /translation="MYGAPRKPARANRGFRGYEQVNRPVYGRARRTAAKGKYVRARYK
                     PGPRYSYQRSTWLAPALRTVGSGVGNYLGGPVLGRMGGQAGAMLGDAIKTMTGFGDYQ
                     IKENSLVLGNPPSVMNGALPGGSIIISHKEYLGDVVSSSVSGSFQLRSYNINPGDENT
                     FPWLSQIAGNFQQYKVMGMAFQFRTMSCDALNSTNTALGQVIMATNYDCTQQVFLSKP
                     EMENCEYAQSVKPSESCLHLLECDTSTMPIETLYVRTDEIPAGTDRRMYDMGKFQIAT
                     NGFQGQSVNAGELWVTYQIMLLKPKLNDALGEDTAYWIGWNNVSIAASTPLGAIAYWT
                     EPENPQVNTSQVSITSDRIITFEPSAVPRCWRVGISWVGAATGTLVAPTLAYTNCTGA
                     YCIRPTSTQQVSEQYPHSAAGAASTRFYLEFYVRTNGAGIVSYVTLGTGGTLPASCTR
                     VQVQVISIPLDAGELDGDGMYE*"
ORIGIN
        1 tagtattacc ccagaccccc ccgtaacaag tcacattttt cattttcctc gtgagaagta
       61 tggcagcagc aaagcaagcg cgaaattggt gtttcactgt gaacaactgg aaggaggcgg
      121 acttggagtg cctgaagaaa cttgaaggca gccaagtgaa gtacatggtg tacggcaagg
      181 aggtgggtga ctctggaaca ccgcacttgc agggctttgt gattttttta aatccaaagc
      241 ggtttagtga agtgaagaag ttgcttccgt ggggttgtca cattgagcag gccaaaggct
      301 cgtgggagca caacttggcg tattgtacca aggaggacac aaatgcttat gtttggggcg
      361 aagcaccaat gtcgcgtaag cgacaaggcg aagcgggagc ggaggcggag aaggagcgat
      421 ggacgaagat ccgagcgttg gcgaaagccg gaaagattga ggaactggaa gtggatttcc
      481 ctcgtgaagc gatactttac gaagaaaagt tcgcaaggat agcgatgaag tgcgccccta
      541 gggcggactt gccgccggag acggtagtcg gagtgtggat acacgggccc agcggagcgg
      601 gcaagtcgta ttggtgtcac cacaaccttg aggttgatgg tgtgacgtat cggatagagg
      661 aaatctacga caaggacctc agtcaatggt gggacggctg gaggtcgagg gacgaaaccg
      721 aagatcggct aaagcatgaa gtggcatact tggacgacct cgacaagttt tgcattaaga
      781 tggcgcgtga tttgaaggtt tggactcaag aatatgagtt caacgcgcag gtgaaggggg
      841 gataccaacg gattcgccct aagttggtgg tcgtcacgtc tcaatctacg attgaggaga
      901 tatgggacga tgacaagacc cggtgtgcac ttcttcgtcg ttttaaagtt ggctataaag
      961 ccaataggga cgccccagtt gtttgggagc caaaatatgt cccaacattt tttgcttaaa
     1021 taaatctgta agatttatta cagttcgtgg tttttcttag ttttagaaat ttcgctgaaa
     1081 cttttagttg ctttagttct ttgctgcatt tttctgtttc accacggcga aacggaaatt
     1141 cgaggagtcg gtctacgacc aagggagtag tttagtgcca tacggagaag cgggctatat
     1201 accatatagc aatcgtaatt cgaagtttac gaagaattat cacgaggatc taggaaatat
     1261 tggagcgaat atgtatggag cgccacgaaa accagcgaga gctaaccgag ggttccgagg
     1321 ttacgagcag gtcaacaggc cagtatatgg acgtgcacgt cggactgctg ccaaaggcaa
     1381 gtacgtacgg gcacgatata agccaggtcc caggtactct tatcaaagaa gtacatggct
     1441 ggcgcctgct ctcaggactg ttggttctgg tgtggggaat tatctcggcg gtccggtttt
     1501 gggaagaatg ggtggacagg caggagccat gcttggggac gcgataaaga cgatgactgg
     1561 atttggtgac taccaaatca aggagaacag cctggtcttg ggcaatcccc caagcgtgat
     1621 gaatggagca cttcctggag gctccattat catcagtcac aaggagtact taggcgacgt
     1681 tgtgtcgagt tcagtgtctg gatcattcca gctcaggtcg tataacatca atcccggtga
     1741 tgagaataca ttcccatggc tgagtcaaat tgctggcaat tttcagcagt acaaggtgat
     1801 gggaatggcc tttcagttca ggacgatgtc ttgcgatgcg ctgaattcga cgaatacagc
     1861 gttggggcag gtcataatgg ctactaatta tgactgcacc cagcaagtgt tcttgtccaa
     1921 gcctgagatg gagaattgcg agtatgcgca atcggtgaag ccctctgagt cgtgcttgca
     1981 tcttttggag tgcgacacca gtacaatgcc tattgagaca ttgtacgttc gcactgatga
     2041 gataccggcg ggtaccgatc ggcggatgta cgacatgggc aagttccaga ttgccaccaa
     2101 tgggttccaa gggcaatctg tgaacgctgg agagctttgg gtgacctatc agattatgct
     2161 tctgaagccc aaattgaatg atgctttggg cgaagataca gcatactgga tagggtggaa
     2221 taacgtatcg attgcggcga gcacaccttt aggagcaatt gcgtattgga ctgaacctga
     2281 gaaccctcag gtgaacacga gtcaagtgtc gatcacaagt gatagaatta tcacttttga
     2341 accttctgct gttccaaggt gttggagagt tggaatctca tgggttggag ctgctacagg
     2401 gacattggtg gcacctactt tggcctatac taattgcact ggtgcatatt gtataaggcc
     2461 aacctcgaca cagcaggtgt cagagcagta tccgcactct gctgcgggtg ctgcgagtac
     2521 gagattctat ttagagtttt atgtgagaac caatggcgca gggattgtgt cttacgttac
     2581 cttgggcact ggaggcactt tgccggctag ttgcacaagg gttcaagttc aggttataag
     2641 cattcctttg gatgctggtg aacttgatgg agatggaatg tacgaataaa ttttttttca
     2701 acgaaacaaa acatttattt agttaggact ctgactctgc ctctgaccca cccatctcac
     2761 cactttacta attccgggaa gtcggcccgc ggctgcggga cgatggcaag gcaaaacacc
     2821 atcagtggct acctaacatc gagcgaagcg aagcgagcga gtcatacacc ctccaatact
     2881 cacgtctaaa aatggtgacg tggcccccgg acgagggggc cataaagtga gccgttaggc
     2941 gaatgttagg cgaacggacg tgacgccctg gttgtgcaaa ctaccaggcg tttatgcaat
     3001 tccaagaaaa gtgacgtggc aaggtgggaa gttagttcct accgccttat aaaaatggct
     3061 gtgttactgt aggggggtct gggc
//
LOCUS       CruV-147                3084 bp    DNA     circular     24-FEB-2020
DEFINITION  Crucivirus-147 isolate H2_33437.
ACCESSION   CruV-147
KEYWORDS    .
SOURCE      Crucivirus-147
  ORGANISM  Crucivirus-147
            Unclassified.
REFERENCE   1  (bases 1 to 3084)
  AUTHORS   de la Higuera,I., Kasun,G.W., Torrance,E.L., Pratt,A.A.,
            Maluenda,A., Colombet,J., Bisseux,M., Ravet,V., Dayaram,A.,
            Stainton,D., Kraberger,S., Zawar-Reza,P., Goldstien,S., Briskie,J.,
            White,R., Taylor,H., Gomez,C., Ainley,D.G., Harding,J.S.,
            Fontenele,R.S., Schreck,J., Ribeiro,S.G., Oswald,S.A., Arnold,J.M.,
            Varsani,A., Enault,F. and Stedman,K.M.
  TITLE     Direct Submission
  JOURNAL   Submitted (24-FEB-2020) Biology, Portland State University, 1719 SW
            10th Ave, Portland, Oregon 97201, USA
COMMENT     Bankit Comment: ALT EMAIL:ide@pdx.edu
            Bankit Comment: TOTAL # OF SEQS:451
            Bankit Comment: GAP: est. length - alignment

            ##Assembly-Data-START##
            Assembly Method       :: MetaSPAdes v. 3.12; IDBA-UD v. 1.1.1;
                                     MEGAHIT v. 1.0.3
            Sequencing Technology :: 454; Illumina
            ##Assembly-Data-END##
FEATURES             Location/Qualifiers
     source          1..3084
                     /organism="Crucivirus-147"
                     /mol_type="genomic DNA"
                     /isolate="H2_33437"
                     /isolation_source="Water"
                     /country="New Zealand"
                     /collection_date=2012
     misc_feature    join(3072..3084,1..21)
                     /note="stem-loop"
     misc_feature    1..9
                     /note="nonanucleotide"
     CDS             60..1019
                     /codon_start=1
                     /product="Putative Replication Associated Protein"
                     /translation="MAAAKQARNWCFTVNNWKEADLECLKKLEGSQVKYMVYGKEVGD
                     SGTPHLQGFVIFLNPKRFSEVKKLLPWGCHIEQAKGSWEHNLAYCTKEDTNAYVWGEA
                     PMSRKRQGEAGAEAEKERWTKIRALAKAGKIEELEVDFPREAILYEEKFARIAMKCAP
                     RADLPPETVVGVWIHGPSGAGKSYWCHHNLEVDGVTYRIEEIYDKDLSQWWDGWRSRD
                     ETEDRLKHEVAYLDDLDKFCIKMARDLKVWTQEYEFNAQVKGGYQRIRPKLVVVTSQS
                     TIEEIWDDDKTRCALLRRFQVGYKANRDAPVVWEPKYVPTFFA*"
     CDS             1271..2689
                     /codon_start=1
                     /product="Putative CruV Capsid Protein"
                     /translation="MYGAPRKPARANRGFRGYEQVNRPVYGRARRTAAKGKYVRARYK
                     PGPRYSYQRSTWLAPALRTVGSGVGNYLGGPVLGRMGGQAGAMLGDAIKTMTGFGDYQ
                     IKENSLVLGNPPSVMNGALPGGSIIISHKEYLGDVVSSSVSGSFQLRSYNINPGDENT
                     FPWLSQIAGNFQQYKVMGMAFQFRTMSCDALNSTNTALGQVIMATNYDCTQQVFLSKP
                     EMENCEYAQSVKPSESCLHLLECDTSTMPIETLYVRTDEIPAGTDRRMYDMGKFQIAT
                     NGFQGQSVNAGELWVTYQIMLLKPKLNDALGEDTAYWIGWNNVSIAASTPLGAIAYWT
                     EPENPQVNTSQVSITSDRIITFEPSAVPRCWRVGISWVGAATGTLVAPTLAYTNCTGA
                     YCIRPTSTQQVSEQYPHSAAGAASTRFYLEFYVRTNGAGIVSYVTLGTGGTLPASCTR
                     VQVQVISIPLDAGELDGDGMYE*"
ORIGIN
        1 tagtattacc ccagaccccc ccgtaacaag tcacattttt cattttcctc gtgagaagta
       61 tggcagcagc aaagcaagcg cgaaattggt gtttcactgt gaacaactgg aaggaggcgg
      121 acttggagtg cctgaagaaa cttgaaggca gccaagtgaa gtacatggtg tacggcaagg
      181 aggtgggtga ctctggaaca ccgcacttgc agggctttgt gattttttta aatccaaagc
      241 ggtttagtga agtgaagaag ttgcttccgt ggggttgtca cattgagcag gccaaaggct
      301 cgtgggagca caacttggcg tattgtacca aggaggacac aaatgcttat gtttggggcg
      361 aagcaccaat gtcgcgtaag cgacaaggcg aagcgggagc ggaggcggag aaggagcgat
      421 ggacgaagat ccgagcgttg gcgaaagccg gaaagattga ggaactggaa gtggatttcc
      481 ctcgtgaagc gatactttac gaagaaaagt tcgcaaggat agcgatgaag tgcgccccta
      541 gggcggactt gccgccggag acggtagtcg gagtgtggat acacgggccc agcggagcgg
      601 gcaagtcgta ttggtgtcac cacaaccttg aggttgatgg tgtgacgtat cggatagagg
      661 aaatctacga caaggacctc agtcaatggt gggacggctg gaggtcgagg gacgaaaccg
      721 aagatcggct aaagcatgaa gtggcatact tggacgacct cgacaagttt tgcattaaga
      781 tggcgcgtga tttgaaggtt tggactcaag aatatgagtt caacgcgcag gtgaaggggg
      841 gataccaacg gattcgccct aagttggtgg tcgtcacgtc tcaatctacg attgaggaga
      901 tatgggacga tgacaagacc cggtgtgcac ttcttcgtcg ttttcaagtt ggctataaag
      961 ccaataggga cgccccagtt gtttgggagc caaaatatgt cccaacattt tttgcttaaa
     1021 taaatctgta agatttatta cagttcgtgg tttttcttag ttttagaaat ttcgctgaaa
     1081 cttttagttg ctttagttct ttgctgcatt tttctgtttc accacggcga aacggaaatt
     1141 cgaggagtcg gtctacgacc aagggagtag tttagtgcca tacggagaag cgggctatat
     1201 accatatagc aatcgtaatt cgaagtttac gaagaattat cacgaggatc taggaaatat
     1261 tggagcgaat atgtatggag cgccacgaaa accagcgaga gctaaccgag ggttccgagg
     1321 ttacgagcag gtcaacaggc cagtatatgg acgtgcacgt cggactgctg ccaaaggcaa
     1381 gtacgtacgg gcacgatata agccaggtcc caggtactct tatcaaagaa gtacatggct
     1441 ggcgcctgct ctcaggactg ttggttctgg tgtggggaat tatctcggcg gtccggtttt
     1501 gggaagaatg ggtggacagg caggagccat gcttggggac gcgataaaga cgatgactgg
     1561 atttggtgac taccaaatca aggagaacag cctggtcttg ggcaatcccc caagcgtgat
     1621 gaatggagca cttcctggag gctccattat catcagtcac aaggagtact taggcgacgt
     1681 tgtgtcgagt tcagtgtctg gatcattcca gctcaggtcg tataacatca atcccggtga
     1741 tgagaataca ttcccatggc tgagtcaaat tgctggcaat tttcagcagt acaaggtgat
     1801 gggaatggcc tttcagttca ggacgatgtc ttgcgatgcg ctgaattcga cgaatacagc
     1861 gttggggcag gtcataatgg ctactaatta tgactgcacc cagcaagtgt tcttgtccaa
     1921 gcctgagatg gagaattgcg agtatgcgca atcggtgaag ccctctgagt cgtgcttgca
     1981 tctgttggag tgcgacacca gtacaatgcc tattgagaca ttgtacgttc gcactgatga
     2041 gataccggcg ggtaccgatc ggcggatgta cgacatgggc aagttccaga ttgccaccaa
     2101 tgggttccaa gggcaatctg tgaacgctgg agagctttgg gtgacctatc agattatgct
     2161 tctgaagccc aaattgaatg atgctttggg cgaagataca gcatactgga tagggtggaa
     2221 taacgtatcg attgcggcga gcacaccttt aggagcaatt gcgtattgga ctgaacctga
     2281 gaaccctcag gtgaacacga gtcaagtgtc gatcacaagt gatagaatta tcacttttga
     2341 accttctgct gttccaaggt gttggagagt tggaatctca tgggttggag ctgctacagg
     2401 gacattggtg gcacctactt tggcctatac taattgcact ggtgcatatt gtataaggcc
     2461 aacctcgaca cagcaggtgt cagagcagta tccgcactct gctgcgggtg ctgcgagtac
     2521 gagattctat ttagagtttt atgtgagaac caatggcgca gggattgtgt cttacgttac
     2581 cttgggcact ggaggcactt tgccggctag ttgcacaagg gttcaagttc aggttataag
     2641 cattcctttg gatgctggtg aacttgatgg agatggaatg tacgaataaa ttttttttca
     2701 acgaaacaaa acatttattt agttaggact ctgactctgc ctctgaccca cccatctcac
     2761 cactttacta attccgggaa gtcggcccgc ggctgcggga cgatggcaag gcaaaacacc
     2821 atcagtggct acctaacatc gagcgaagcg aagcgagcga gtcatacacc ctccaatact
     2881 cacgtctaaa aatggtgacg tggcccccgg acgagggggc cataaagtga gccgttaggc
     2941 gaatgttagg cgaacggacg tgacgccctg gttgtgcaaa ctaccaggcg tttatgcaat
     3001 tccaagaaaa gtgacgtggc aaggtgggaa gttagttcct accgccttat aaaaatggct
     3061 gtgttactgt aggggggtct gggc
//
LOCUS       CruV-148                3084 bp    DNA     circular     24-FEB-2020
DEFINITION  Crucivirus-148 isolate H7_21258.
ACCESSION   CruV-148
KEYWORDS    .
SOURCE      Crucivirus-148
  ORGANISM  Crucivirus-148
            Unclassified.
REFERENCE   1  (bases 1 to 3084)
  AUTHORS   de la Higuera,I., Kasun,G.W., Torrance,E.L., Pratt,A.A.,
            Maluenda,A., Colombet,J., Bisseux,M., Ravet,V., Dayaram,A.,
            Stainton,D., Kraberger,S., Zawar-Reza,P., Goldstien,S., Briskie,J.,
            White,R., Taylor,H., Gomez,C., Ainley,D.G., Harding,J.S.,
            Fontenele,R.S., Schreck,J., Ribeiro,S.G., Oswald,S.A., Arnold,J.M.,
            Varsani,A., Enault,F. and Stedman,K.M.
  TITLE     Direct Submission
  JOURNAL   Submitted (24-FEB-2020) Biology, Portland State University, 1719 SW
            10th Ave, Portland, Oregon 97201, USA
COMMENT     Bankit Comment: ALT EMAIL:ide@pdx.edu
            Bankit Comment: TOTAL # OF SEQS:451
            Bankit Comment: GAP: est. length - alignment

            ##Assembly-Data-START##
            Assembly Method       :: MetaSPAdes v. 3.12; IDBA-UD v. 1.1.1;
                                     MEGAHIT v. 1.0.3
            Sequencing Technology :: 454; Illumina
            ##Assembly-Data-END##
FEATURES             Location/Qualifiers
     source          1..3084
                     /organism="Crucivirus-148"
                     /mol_type="genomic DNA"
                     /isolate="H7_21258"
                     /isolation_source="Water"
                     /country="New Zealand"
                     /collection_date=2012
     misc_feature    join(3071..3084,1..22)
                     /note="stem-loop"
     misc_feature    1..9
                     /note="nonanucleotide"
     CDS             60..1019
                     /codon_start=1
                     /product="Putative Replication Associated Protein"
                     /translation="MAAAKQARNWCFTVNNWKEADLECLKKLEGSQVKYMVYGKEVGD
                     SGTPHLQGFVIFLNPKRFSEVKKLLPWGCHIEQAKGSWEHNLAYCTKEDTNAYVWGEA
                     PMSRKRQGEAGAEAEKERWTKIRALAKAGKIEELEVDFPREAILYEEKFARIAMKCAP
                     RADLPPETVVGVWIHGPSGAGKSYWCHHNLEVDGVTYRIEEIYDKDLSQWWDGWRSRD
                     ETEDRLKHEVAYLDDLDKFCIKMARDLKVWTQEYEFNAQVKGGYQRIRPKLVVVTSQS
                     TIEEIWDDDKTRCALLRRFKVGYKANRDAPVVWEPKYVPTFFA*"
     CDS             1271..2689
                     /codon_start=1
                     /product="Putative CruV Capsid Protein"
                     /translation="MYGAPRKPARANRGFRGYEQVNRPVYGRARRTAAKGKYVRARYK
                     PGPRYSYQRSTWLAPALRTVGSGVGNYLGGPVLGRMGGQAGAMLGDAIKTMTGFGDYQ
                     IKENSLVLGNPPSVMNGALPGGSIIISHKEYLGDVVSSSVSGSFQLRSYNINPGDENT
                     FPWLSQIAGNFQQYKVMGMAFQFRTMSCDALNSTNTALGQVIMATNYDCTQQVFLSKP
                     EMENCEYAQSVKPSESCLHLLECDTSTMPIETLYVRTDEIPAGTDRRMYDMGKFQIAT
                     NGFQGQSVNAGELWVTYQIMLLKPKLNDALGEDTAYWIGWNNVSIAASTPLGAIAYWT
                     EPENPQVNTSQVSITSDRIITFEPSAVPRCWRVGISWVGAATGTLVAPTLAYTNCTGA
                     YCIRPTSTQQVSEQYPHSAAGAASTRFYLEFYVRTNGAGIVSYVTLGTGGTLPASCTR
                     VQVQVISIPLDAGELDGDGMYE*"
ORIGIN
        1 tagtattacc ccagaccccc ccgtaacaag tcacattttt cattttcctc gtgagaagta
       61 tggcagcagc aaagcaagcg cgaaattggt gtttcactgt gaacaactgg aaggaggcgg
      121 acttggagtg cctgaagaaa cttgaaggca gccaagtgaa gtacatggtg tacggcaagg
      181 aggtgggtga ctctggaaca ccgcacttgc agggctttgt gattttttta aatccaaagc
      241 ggtttagtga agtgaagaag ttgcttccgt ggggttgtca cattgagcag gccaaaggct
      301 cgtgggagca caacttggcg tattgtacca aggaggacac aaatgcttat gtttggggcg
      361 aagcaccaat gtcgcgtaag cgacaaggcg aagcgggagc ggaggcggag aaggagcgat
      421 ggacgaagat ccgagcgttg gcgaaagccg gaaagattga ggaactggaa gtggatttcc
      481 ctcgtgaagc gatactttac gaagaaaagt tcgcaaggat agcgatgaag tgcgccccta
      541 gggcggactt gccgccggag acggtagtcg gagtgtggat acacgggccc agcggagcgg
      601 gcaagtcgta ttggtgtcac cacaaccttg aggttgatgg tgtgacgtat cggatagagg
      661 aaatctacga caaggacctc agtcaatggt gggacggctg gaggtcgagg gacgaaaccg
      721 aagatcggct aaagcatgaa gtggcatact tggacgacct cgacaagttt tgcattaaga
      781 tggcgcgtga tttgaaggtt tggactcaag aatatgagtt caacgcgcag gtgaaggggg
      841 gataccaacg gattcgccct aagttggtgg tcgtcacgtc tcaatctacg attgaggaga
      901 tatgggacga tgacaagacc cggtgtgcac ttcttcgtcg ttttaaagtt ggctataaag
      961 ccaataggga cgccccagtt gtttgggagc caaaatatgt cccaacattt tttgcttaaa
     1021 taaatctgta agatttatta cagttcgtgg tttttcttag ttttagaaat ttcgctgaaa
     1081 cttttagttg ctttagttct ttgctgcatt tttctgtttc accacggcga aacggaaatt
     1141 cgaggagtcg gtctacgacc aagggagtag tttagtgcca tacggagaag cgggctatat
     1201 accatatagc aatcgtaatt cgaagtttac gaagaattat cacgaggatc taggaaatat
     1261 tggagcgaat atgtatggag cgccacgaaa accagcgaga gctaaccgag ggttccgagg
     1321 ttacgagcag gtcaacaggc cagtatatgg acgtgcacgt cggactgctg ccaaaggcaa
     1381 gtacgtacgg gcacgatata agccaggtcc caggtactct tatcaaagaa gtacatggct
     1441 ggcgcctgct ctcaggactg ttggttctgg tgtggggaat tatctcggcg gtccggtttt
     1501 gggaagaatg ggtggacagg caggagccat gcttggggac gcgataaaga cgatgactgg
     1561 atttggtgac taccaaatca aggagaacag cctggtcttg ggcaatcccc caagcgtgat
     1621 gaatggagca cttcctggag gctccattat catcagtcac aaggagtact taggcgacgt
     1681 tgtgtcgagt tcagtgtctg gatcattcca gctcaggtcg tataacatca atcccggtga
     1741 tgagaataca ttcccatggc tgagtcaaat tgctggcaat tttcagcagt acaaggtgat
     1801 gggaatggcc tttcagttca ggacgatgtc ttgcgatgcg ctgaattcga cgaatacagc
     1861 gttggggcag gtcataatgg ctactaatta tgactgcacc cagcaagtgt tcttgtccaa
     1921 gcctgagatg gagaattgcg agtatgcgca atcggtgaag ccctctgagt cgtgcttgca
     1981 tcttttggag tgcgacacca gtacaatgcc tattgagaca ttgtacgttc gcactgatga
     2041 gataccggcg ggtaccgatc ggcggatgta cgacatgggc aagttccaga ttgccaccaa
     2101 tgggttccaa gggcaatctg tgaacgctgg agagctttgg gtgacctatc agattatgct
     2161 tctgaagccc aaattgaatg atgctttggg cgaagataca gcatactgga tagggtggaa
     2221 taacgtatcg attgcggcga gcacaccttt aggagcaatt gcgtattgga ctgaacctga
     2281 gaaccctcag gtgaacacga gtcaagtgtc gatcacaagt gatagaatta tcacttttga
     2341 accttctgct gttccaaggt gttggagagt tggaatctca tgggttggag ctgctacagg
     2401 gacattggtg gcacctactt tggcctatac taattgcact ggtgcatatt gtataaggcc
     2461 aacctcgaca cagcaggtgt cagagcagta tccgcactct gctgcgggtg ctgcgagtac
     2521 gagattctat ttagagtttt atgtgagaac caatggcgca gggattgtgt cttacgttac
     2581 cttgggcact ggaggcactt tgccggctag ttgcacaagg gttcaagttc aggttataag
     2641 cattcctttg gatgctggtg aacttgatgg agatggaatg tacgaataaa ttttttttca
     2701 acgaaacaaa acatttattt agttaggact ctgactctgc ctctgaccca cccatctcac
     2761 cactttacta attccgggaa gtcggcccgc ggctgcggga cgatggcaag gcaaaacacc
     2821 atcagtggct acctaacatc gagcgaagcg aagcgagcga gtcatacacc ctccaatact
     2881 cacgtctaaa aatggtgacg tggcccccgg acgagggggc cataaagtga gccgttaggc
     2941 gaatgttagg cgaacggacg tgacgccctg gttgtgcaca ctaccaggcg tttatgcaat
     3001 tccaagaaaa gtgacgtggc aaggtgggaa gttagttcct accgccttat aaaaatggct
     3061 gtgttacttt gggggggtct gggc
//
LOCUS       CruV-149                3089 bp    DNA     circular     24-FEB-2020
DEFINITION  Crucivirus-149 isolate 120_547_IR1_3296.
ACCESSION   CruV-149
KEYWORDS    .
SOURCE      Crucivirus-149
  ORGANISM  Crucivirus-149
            Unclassified.
REFERENCE   1  (bases 1 to 3089)
  AUTHORS   de la Higuera,I., Kasun,G.W., Torrance,E.L., Pratt,A.A.,
            Maluenda,A., Colombet,J., Bisseux,M., Ravet,V., Dayaram,A.,
            Stainton,D., Kraberger,S., Zawar-Reza,P., Goldstien,S., Briskie,J.,
            White,R., Taylor,H., Gomez,C., Ainley,D.G., Harding,J.S.,
            Fontenele,R.S., Schreck,J., Ribeiro,S.G., Oswald,S.A., Arnold,J.M.,
            Varsani,A., Enault,F. and Stedman,K.M.
  TITLE     Direct Submission
  JOURNAL   Submitted (24-FEB-2020) Biology, Portland State University, 1719 SW
            10th Ave, Portland, Oregon 97201, USA
COMMENT     Bankit Comment: ALT EMAIL:ide@pdx.edu
            Bankit Comment: TOTAL # OF SEQS:451
            Bankit Comment: GAP: est. length - alignment

            ##Assembly-Data-START##
            Assembly Method       :: MetaSPAdes v. 3.12; IDBA-UD v. 1.1.1;
                                     MEGAHIT v. 1.0.3
            Sequencing Technology :: 454; Illumina
            ##Assembly-Data-END##
FEATURES             Location/Qualifiers
     source          1..3089
                     /organism="Crucivirus-149"
                     /mol_type="genomic DNA"
                     /isolate="120_547_IR1_3296"
                     /isolation_source="Water"
                     /country="Norway"
                     /collection_date=2011
     misc_feature    join(3079..3089,1..18)
                     /note="stem-loop"
     misc_feature    1..9
                     /note="nonanucleotide"
     CDS             75..1442
                     /codon_start=1
                     /product="Putative Replication Associated Protein"
                     /translation="MKKRAIPPIDLVNACVSKNNSMNGEDTVWRKEKSVVKPTEFNGF
                     GDWTEGKPKDPIVVGGPNNISSQPTSGKGRPGSKKSSRDWCYTYWGPESSIDPGLHER
                     STVKFGDHVWLETLHDVPTNAVVYHVFQFEISPDTGGRHIQGFISFATRRRFATVRKV
                     LPRCHLSPRRGTVEQAATYCMKTDSKDGDYQFEYGTRPEDDLGQGHRSDIDAIKELID
                     EGASMKMVADHDFGYYVRNYRGLNQYRSISTKGRNKKSLVFLFVGGPGTGKSAAADSF
                     MDNYTVPPGSSGTVWYDDYEPGHHKTVIFDDFTGAWCQWRELLRLTDSYKMLVNTKGG
                     HIPFKPEAIVFTANYSPERWYSNEAVPDKAPFMRRVDYHWQYYLKHATNAEWMIPDEV
                     RSALLGFEERHPMLPVHSVCRCLKGDPTYHPHYGRYCAFDVNGETYYAVISQQKPVFV
                     QPKLW*"
     CDS             1487..2827
                     /codon_start=1
                     /product="Putative CruV Capsid Protein"
                     /translation="MPFGTYRRASTRRAPLKSYARSRRAVPKRRSFSGYRGRGAYTAP
                     KSAAWGQAAGNALGGVLGMIPQTKAFAAPIGALASKLGGFLGNKLGTYMGWGDYSVNV
                     NSLIVPEGNSPAAMHSDGKSVRICHREYIGDILSSPIPGQFLLQNLQLNPANAAFFPW
                     LWNISVNFQKYKLHGAIMEYKSASGDAITTDNTALGTVLIASNYNCADPLFTNRSQME
                     NTQYTSSAKPSVSFVHIIECDPHLQTQVNLYTAEGGVPHTGVGINESNWCNVQIATIG
                     CQGESVNLGSIYITYDIELIQPINQVDSTVFRGDWFSLTGNGSEWAAQNIPGSTPNAN
                     NSIGGIIVTGNTYRFPQYLQTGLFKVTYYYIPQTPGPGQSLPTITPNGSCVSPGSMPG
                     SLSGEGSSEDDSMTVIWMIKVTAGNASFHIGSGTFQPGTGNGALMIDQVDLNFT*"
ORIGIN
        1 tattgttacc ccccgagact tcgggttcag ggcggataaa aatcccctca tccacaaaac
       61 gcgcctagcg tgacttgaaa aaacgcgcca ttccccctat tgacttggtc aacgcctgtg
      121 tctcaaaaaa caattctatg aatggtgaag atacagtatg gcgtaaagaa aagtcagtcg
      181 ttaaaccgac tgaatttaat ggctttggtg attggactga aggcaaaccc aaagacccaa
      241 tcgttgttgg tggaccaaat aatatttcgt ctcagccgac aagcggaaag ggtagaccgg
      301 gctcaaagaa aagttcacga gactggtgtt acacttattg gggaccggaa tcatccattg
      361 atcctggact tcatgaacga agtacagtca aattcggaga tcatgtgtgg ttggaaactc
      421 ttcacgatgt tcccacaaac gctgttgtgt accacgtttt tcaatttgag atatcaccag
      481 atactggtgg aagacacatt cagggcttca tctcttttgc gactcgacgt cgtttcgcaa
      541 cagttcgtaa agtactgcca cgatgtcacc tttcgcctcg tagaggaact gttgaacaag
      601 ctgcaactta ttgcatgaaa acggattcaa aagatggtga ttatcaattc gagtatggta
      661 cgagacctga agatgatctt ggacaaggtc atcgaagtga tattgatgct atcaaggagt
      721 tgatagatga aggcgcttca atgaaaatgg ttgctgatca tgactttgga tattatgtta
      781 gaaactatcg aggtcttaac caatatcgat ctatatcaac aaaaggtcgt aacaaaaaaa
      841 gccttgtgtt tttatttgtt ggtggtcctg gtactggaaa gtctgctgct gctgattctt
      901 tcatggacaa ttatacagtc cctcctggtt cttcagggac tgtttggtac gatgattatg
      961 agcctggtca tcacaaaact gtaatttttg atgatttcac cggcgcttgg tgtcaatggc
     1021 gagagctctt gagacttacg gattcataca agatgcttgt caatacaaag ggtggacata
     1081 tcccttttaa acctgaggct attgttttta cagcgaatta ttctcctgaa agatggtata
     1141 gtaatgaagc cgtacctgat aaggcgcctt ttatgaggcg tgtagactat cattggcaat
     1201 actatcttaa acatgcaact aatgctgaat ggatgatacc tgatgaagtg cgttcagcac
     1261 ttttgggatt tgaggaacgg catcctatgc ttccggtgca ttctgtgtgc agatgcttga
     1321 aaggggaccc aacatatcat cctcactatg gtagatactg tgcatttgat gttaatggag
     1381 aaacatatta tgctgtaata tctcaacaaa aaccggtgtt tgttcaacca aaactttggt
     1441 agtataaact tttcaaagta tattcttatt atcacaagac taaagtatgc cttttggtac
     1501 atatcgtcgt gcttcaactc gtagagcacc actcaaaagt tatgctcgtt ctcgtagagc
     1561 cgttcctaaa cgtcgttctt ttagtggata tcgtggcaga ggtgcttaca ctgcaccaaa
     1621 atctgctgca tggggtcaag ccgcaggtaa tgcacttggt ggcgttcttg ggatgatccc
     1681 tcaaactaaa gcctttgctg ctcctattgg tgctttagca tcaaagttgg gaggatttct
     1741 tggcaacaag ttggggactt atatgggatg gggtgactat tctgtgaatg tcaactctct
     1801 tattgttcca gagggaaatt ctcctgctgc aatgcacagt gatggaaaaa gtgtccgtat
     1861 ttgccatcgt gaatatattg gagatatcct ttcttctcca attccaggac aatttcttct
     1921 tcaaaatctt cagctcaatc cagccaacgc tgcatttttc ccatggttgt ggaatatttc
     1981 agtgaacttt caaaagtata aacttcatgg agctattatg gaatataagt ctgcttcagg
     2041 tgatgccatc acaaccgaca ataccgccct tggaactgtt ctcattgcta gcaattacaa
     2101 ttgcgccgat cccttgttca ccaatcgttc ccaaatggag aacacacaat acacctcttc
     2161 tgccaagccc tcagtgagtt ttgttcatat tattgaatgt gatcctcatc ttcaaactca
     2221 agtgaactta tacactgctg aaggaggagt tcctcacact ggtgttggca tcaacgaatc
     2281 caattggtgc aatgttcaaa ttgcaactat cggttgtcaa ggagaatctg tcaatcttgg
     2341 gtcaatttac attacttatg acattgaatt gattcaacca atcaaccaag ttgactctac
     2401 tgttttccga ggagattggt tttctctcac aggtaatgga tctgagtggg ctgcacaaaa
     2461 cattcctggt tctactccaa atgctaacaa tagcattgga ggaattatcg ttactgggaa
     2521 tacttacaga tttcctcagt atttgcagac tggacttttc aaagtcacct actactatat
     2581 tcctcaaact cctggtccag gccaatctct tccaactatc acaccaaatg gaagctgcgt
     2641 gtctcctggc tcaatgcctg gctctctttc tggagaaggt tcttctgaag atgattctat
     2701 gactgtcatt tggatgatta aggttacagc aggcaacgcc tctttccata ttggaagtgg
     2761 aacatttcaa ccaggcactg gaaatggtgc tttgatgatt gatcaagttg atctcaactt
     2821 cacctgatcg tgtcccgcag ggcacgcaga ctcattaaac tctcattcca ctttaccttt
     2881 attactagct aaaattcctt tttaccacac tcaacactct gtatgggccg ccaacgaggc
     2941 gcgggacccg cttgcggggc gccgaggctg gtggaccagt atccctatca tatataatgt
     3001 agtagtcctt agaacccatt gaagtagtcg ttgaagagag cgccagtgat cgtcggcgtg
     3061 ttctccctag agggagaatc tcgggggtc
//
LOCUS       CruV-150                3094 bp    DNA     circular     24-FEB-2020
DEFINITION  Crucivirus-150 isolate 000763_ADNa22.
ACCESSION   CruV-150
KEYWORDS    .
SOURCE      Crucivirus-150
  ORGANISM  Crucivirus-150
            Unclassified.
REFERENCE   1  (bases 1 to 3094)
  AUTHORS   de la Higuera,I., Kasun,G.W., Torrance,E.L., Pratt,A.A.,
            Maluenda,A., Colombet,J., Bisseux,M., Ravet,V., Dayaram,A.,
            Stainton,D., Kraberger,S., Zawar-Reza,P., Goldstien,S., Briskie,J.,
            White,R., Taylor,H., Gomez,C., Ainley,D.G., Harding,J.S.,
            Fontenele,R.S., Schreck,J., Ribeiro,S.G., Oswald,S.A., Arnold,J.M.,
            Varsani,A., Enault,F. and Stedman,K.M.
  TITLE     Direct Submission
  JOURNAL   Submitted (24-FEB-2020) Biology, Portland State University, 1719 SW
            10th Ave, Portland, Oregon 97201, USA
COMMENT     Bankit Comment: ALT EMAIL:ide@pdx.edu
            Bankit Comment: TOTAL # OF SEQS:451
            Bankit Comment: GAP: est. length - alignment

            ##Assembly-Data-START##
            Assembly Method       :: MetaSPAdes v. 3.12; IDBA-UD v. 1.1.1;
                                     MEGAHIT v. 1.0.3
            Sequencing Technology :: 454; Illumina
            ##Assembly-Data-END##
FEATURES             Location/Qualifiers
     source          1..3094
                     /organism="Crucivirus-150"
                     /mol_type="genomic DNA"
                     /isolate="000763_ADNa22"
                     /isolation_source="Water"
                     /country="France"
                     /collection_date=2013
     misc_feature    join(3083..3094,1..20)
                     /note="stem-loop"
     misc_feature    1..9
                     /note="nonanucleotide"
     CDS             107..793
                     /codon_start=1
                     /product="Putative Replication Associated Protein"
                     /translation="MPRFDPKARGTAWSLTINNPTDDDEENMNQARQKGWKVEGQKEI
                     GANGTEHYQLILKTPQTRGSAVKKTFPRAHIELARDAIALEKYVKKEETRIGELPVSS
                     DKYPSLQKMWDMFAQYIEDKYPKGTYLDWIDTDDEKCLKVFDEFIRRAIRDGYVVETM
                     GVNPQMRSCVKNYCFAIFARSMNRIDEARQRVMDAEEYSDDESAVSPFTPRPTVFVSD
                     DEAEVTLPIA*"
     CDS             931..2307
                     /codon_start=1
                     /product="Putative CruV Capsid Protein"
                     /translation="MLRYIYKMQTSRVPRRRAVARPGGRALAVAPVAPRRRRAPAPRG
                     AVSGKGFYKGFGQHLGTAIGGAVSALVGVPGLDKVGGRLGAMGAKATGFGAYAISKNS
                     LVMQDPPEIHNARAKEGAMVVCHREYLTDIQGSTGFTIQGMYDLQPGLDAGFPWLSQI
                     ANAFEEWEPMGILVEFVTTSGNAISSTNPALGEVILSTQYNSYAPAFTTKQQMLNQIF
                     AVSSVPSQNVIHPIECKPSQNQVTRFYTRNSAPIGDTRLYDLGRLSVATSGQQAVSAI
                     GELWITYQIAFYKPKLLSPSALLSLPLAEYSFVYGGTTDPIFGQTGSAPILQSAGDPF
                     GLQFAPNRLAFTCPGSQGTTFEVTMVLNGVGANDFSSQPLASLSNCTASNTATDSTGA
                     VSRSLICAKVTSTLNMPVSCIQSYVVTVTDPTLPWSMVWLPTGTAGSNYANLPPSPGG
                     QLYVSKLS*"
ORIGIN
        1 tagtattaca ggatttctag aatcttataa ggaacgcata agttctaaaa tttgtctcgc
       61 cctcgatttt agacaaattt cttttctcag tttatagtta taaacaatgc ctcgctttga
      121 ccctaaagca agaggcacag cttggtcttt aaccattaat aatccaactg acgatgatga
      181 agagaatatg aatcaagctc gtcaaaaagg ttggaaggtt gagggacaaa aggagatagg
      241 agcgaatgga acagagcatt atcaactcat tttgaaaact cctcaaaccc gtgggtctgc
      301 tgttaagaag acatttccac gagctcacat cgaactagcg cgagatgcaa tcgcgttaga
      361 gaagtatgtt aaaaaggaag agacccgaat aggcgagctt cctgtatcat ccgataaata
      421 tccatctttg cagaagatgt gggatatgtt cgcacaatat atcgaagaca aatacccgaa
      481 gggaacttat ttggattgga tagacaccga tgatgaaaaa tgtttaaaag tatttgatga
      541 atttattcgt cgagccatta gagatggcta tgtggttgaa accatgggag tcaatccaca
      601 aatgcgtagc tgtgtaaaga attattgctt tgctattttt gcacgttcaa tgaatcgcat
      661 tgatgaagca cggcaacgtg tgatggatgc ggaagaatat tcagatgacg agtcagctgt
      721 ttctcctttt acaccaagac caaccgtttt tgtatctgac gatgaggcag aagttacgtt
      781 gccaattgca taagtgacta aaaacaatat ataatttcga ttagaaaaat cttacctaaa
      841 caggtaagaa aaacaaagca ttatcttatc gtcaaatgat gaataagatt atgttcgcgc
      901 caagttcgac ccccccccca tgaaaaaaaa atgttgcgtt atatatataa aatgcaaact
      961 tctcgtgttc ctcgtagacg tgctgtagcg cgtcctggtg gtcgtgcact tgctgtagct
     1021 cccgtagccc cccgtcgtcg tcgtgcgcct gcgccacgcg gtgcggtttc gggtaaggga
     1081 ttttacaaag gattcggaca acatttaggc actgccatcg gtggtgctgt aagtgcgctt
     1141 gtcggtgtcc ctggacttga taaagttggc ggacgtcttg gtgccatggg tgccaaggca
     1201 acgggttttg gtgcgtatgc tatttccaaa aatagtcttg ttatgcaaga cccgcccgag
     1261 attcataatg cccgcgcgaa ggaaggtgca atggtagtgt gccaccgtga gtatcttacg
     1321 gatattcagg gttcaactgg atttactatt cagggcatgt atgaccttca acctgggctt
     1381 gacgcaggtt ttccatggct ttctcagatt gctaatgcgt ttgaggaatg ggagccgatg
     1441 ggtattctcg tagaatttgt aaccacgtca ggtaatgcga tttcgagcac caatcctgcg
     1501 cttggtgaag tcattctatc gacgcaatac aacagctatg ctcctgcatt tacgactaaa
     1561 cagcagatgc ttaatcagat ttttgctgta agctcagtgc catctcagaa tgtcatacat
     1621 cctattgaat gtaagccatc gcagaatcaa gttacgcggt tttatactcg taactctgca
     1681 cccattggtg acacgcgtct atatgacctt gggcgtttat ctgttgcaac atcggggcaa
     1741 caggctgtga gtgcaattgg tgagctttgg attacctatc agattgcatt ttacaaaccg
     1801 aaacttcttt cgccatctgc actgctgagc ttgcctttgg cagagtattc ctttgtgtat
     1861 ggcggaacga cagaccctat tttcggtcag actggttctg caccgattct ccagtcagct
     1921 ggagacccat ttgggctaca gtttgcacct aatcgtcttg cgtttacatg ccctggttct
     1981 cagggcacaa cgttcgaagt tacaatggta ttgaacggtg ttggcgcgaa tgactttagt
     2041 tctcagccac ttgcttcgct aagcaattgc actgcatcta acacggcaac ggacagcacg
     2101 ggtgctgttt cgcgtagtct tatttgtgcg aaggttacga gcacgttgaa tatgccagta
     2161 agttgtattc agtcatatgt tgttacggtg actgacccta cacttccttg gtcgatggtg
     2221 tggcttccta cgggaacggc tggttcaaat tatgctaatt tgccacccag tccaggcggt
     2281 cagctttatg tgtctaaact tagttagata tatatttttt gactgagggt ggtctcgaag
     2341 cctttggtga gagctgtata ccgaagttac tttttttaaa aaaaaagttc catatgaaaa
     2401 ggtttttaac atttaaatca ttgttccttt aaaaaacaca atgtaattac aaacgtagtt
     2461 atattatgtt tcttacctaa atacgtaaca atatttttat aaaatagatc tattttattg
     2521 tgacctaaat aggtcatgtc aaaaggcttt gcattttgtt taattttttg caaacttgtt
     2581 ttgcactttt gcatttatgc ttttgcggac tcgaccgctc caggtttaag acccccccat
     2641 gaaaacaagt ctttgcggac tcgaccgctc ttggttttat acccccccat gaaaagttcc
     2701 agaataaaaa aggcgtcgcc ctcgataaat tattttctca gtaaaatagt tatagcctca
     2761 acttcaaaag atgcattcat tttttgaatt ttatcttgat ttttgtcgcc ataactgttg
     2821 tgaaataatt catttttttt ggtccgtaga caaaaaagac agtcagacaa tagacaagta
     2881 tagcataacc gacattctaa attgcaaacc cttttgcgat tttgaagttg ggtcccgtca
     2941 gatgtcaatc tgacttggcg gaaatcgggt tcccttccag ggtgattttc gacgaggcag
     3001 ttggcagatg gcggggcacg acatcaatga agttggggtg atgctactct gcgttatttc
     3061 acaatctttt gtacgcgcaa acctagaaat cccc
//
LOCUS       CruV-151                3096 bp    DNA     circular     24-FEB-2020
DEFINITION  Crucivirus-151 isolate 120_242_SvL2_3298.
ACCESSION   CruV-151
KEYWORDS    .
SOURCE      Crucivirus-151
  ORGANISM  Crucivirus-151
            Unclassified.
REFERENCE   1  (bases 1 to 3096)
  AUTHORS   de la Higuera,I., Kasun,G.W., Torrance,E.L., Pratt,A.A.,
            Maluenda,A., Colombet,J., Bisseux,M., Ravet,V., Dayaram,A.,
            Stainton,D., Kraberger,S., Zawar-Reza,P., Goldstien,S., Briskie,J.,
            White,R., Taylor,H., Gomez,C., Ainley,D.G., Harding,J.S.,
            Fontenele,R.S., Schreck,J., Ribeiro,S.G., Oswald,S.A., Arnold,J.M.,
            Varsani,A., Enault,F. and Stedman,K.M.
  TITLE     Direct Submission
  JOURNAL   Submitted (24-FEB-2020) Biology, Portland State University, 1719 SW
            10th Ave, Portland, Oregon 97201, USA
COMMENT     Bankit Comment: ALT EMAIL:ide@pdx.edu
            Bankit Comment: TOTAL # OF SEQS:451
            Bankit Comment: GAP: est. length - alignment

            ##Assembly-Data-START##
            Assembly Method       :: MetaSPAdes v. 3.12; IDBA-UD v. 1.1.1;
                                     MEGAHIT v. 1.0.3
            Sequencing Technology :: 454; Illumina
            ##Assembly-Data-END##
FEATURES             Location/Qualifiers
     source          1..3096
                     /organism="Crucivirus-151"
                     /mol_type="genomic DNA"
                     /isolate="120_242_SvL2_3298"
                     /isolation_source="Water"
                     /country="Norway"
                     /collection_date=2012
     CDS             1..1350
                     /codon_start=1
                     /product="Putative Replication Associated Protein"
                     /translation="MCVFYLKIYLYKRVGRHVLPINMNLDPANNYAPLSNEDGRSRAK
                     HWCFTINHPTIQDVSMFKDIAILTTYYVYGEEVADSGTEHLQCYICFDKAVTLKSCKG
                     YWPRAHFEVMRGTPIQASDYCKKDGKFVEHGLLPKAKNVAGGEATKAKWIEISTAATE
                     NNLQLIQSKHPKEFVSNYRSLKQISFDFQPPMPDLDGDLDNLWVYGESGVGKSKSVRE
                     QYGEDLYYKMCNKWWDNYEGEENVLIDDLDLTHSVIGHHIKIWADRYAFRAEVKNHSK
                     MIRPKRIIITSQYHPNEIWTDQKLVDAITRRFKVKHMIKLPDVDPLISKKSKIPYTKD
                     AYAYNVVPKKHDKAYMALDNQIQKKKLRLIGSKSSIQSKIDKPLVQVGPVLKYKTPEL
                     SKPSLDFTNLKNQSISIDSSSEEEFDSSLPDISYDEGSNHGEWDSYDMYEKFVNQKK*
                     "
     CDS             1637..3019
                     /codon_start=1
                     /product="Putative CruV Capsid Protein"
                     /translation="MSKRASPNAIRPRAMAKYGQAGAKPIYAKGPIPYEVSAPNRFWR
                     IKKDLPPQEYWKKRYWRRRITGKGAYSMNAGDSFGRRWGGYLGSKAGEFIGGYAQRLT
                     GLGDYKVTRNVFADGRLPEVVNNPQGGGTVIRFQEYLGDIITSATANTFDIQSYDINA
                     ANPRTFPWLSQIAANYDQYDFEGLLFEFKSTSADALNSTNTALGSVMMATQYDTIDPP
                     FDSKLDMLNYEYSTSIKPSMSTMHMVECDPKQTTINELYTLSNQSVPPNADPRLYFLG
                     RFQIATTGFQGLSVNVGQLHVTYQVRLLKPKLFTTLGNGIDDATYRFGVGTTYVGYDN
                     THPLTLVGEVPNSAVDNIPLVITPTTITFPVFEGKLAYRIEIIWRGATAVNTEHPDLS
                     IANGTLISVDSFNMDLNAQSRCGYLLGIVSDGSANPLVLTVADNGILPSGNNEVVIRV
                     MQTNPIFKAV*"
ORIGIN
        1 atgtgtgttt tttatttgaa aatctacctt tataaacgtg tcggaagaca cgttttacca
       61 atcaatatga acttagatcc tgctaacaac tacgctccct tatcaaatga agatggaaga
      121 tcacgcgcaa aacactggtg ttttacaatc aatcatccaa caattcaaga tgttagcatg
      181 tttaaagata ttgcaatcct tacaacatac tatgtttatg gagaagaagt tgctgattct
      241 gggactgaac acttacaatg ttatatatgc tttgacaaag cagttactct taaatcctgt
      301 aaaggatatt ggcctagagc ccactttgaa gtcatgcgag gaactccaat acaagcaagc
      361 gattattgta aaaaggatgg aaaatttgtg gagcatgggc ttctaccaaa agccaaaaat
      421 gttgctggag gagaagcgac gaaagctaaa tggatagaga tttcaactgc agccacagaa
      481 aataatctcc agttaattca gagcaaacat ccaaaagagt ttgtatctaa ttatagatca
      541 cttaaacaaa tctcttttga ttttcaaccg cctatgccag acttggatgg agacttagat
      601 aatctgtggg tatatggtga atctggcgtt ggtaaatcga agagtgtcag agagcaatat
      661 ggagaagatc tttattacaa aatgtgtaat aaatggtggg ataattatga aggcgaagaa
      721 aacgttctta ttgatgacct agatctcaca cattctgtca ttggacatca tatcaagata
      781 tgggctgatc gatacgcttt cagagccgaa gtcaaaaatc attccaagat gatacgtcca
      841 aaacgtatca ttattacttc tcaatatcat ccaaatgaaa tttggactga ccagaaactt
      901 gtagatgcta ttactcgtcg atttaaagtt aagcacatga tcaaacttcc tgatgtagat
      961 cctttgattt caaagaaatc aaaaattcct tataccaaag atgcttatgc ttataatgtt
     1021 gttccgaaga agcatgacaa agcatatatg gcgttagata atcagatcca gaagaagaaa
     1081 cttcgtttaa ttggttccaa atcttccatt caatcaaaaa ttgataagcc attagttcaa
     1141 gttggacctg ttttaaaata taaaacacct gaacttagta aaccatcttt agattttact
     1201 aatttaaaaa atcaaagtat ttcaattgat tcttcgtcag aagaagaatt tgattcttct
     1261 ttacctgata tatcatatga tgaaggttcg aatcatgggg aatgggactc ttatgatatg
     1321 tatgagaaat ttgttaatca aaagaaataa atacttgtta gccctaaatt agtaatgatt
     1381 attccctgtc tattattcgt taggtgttga tacgaactat cttaaaacga ataataacag
     1441 ctgcgcgcag cgtacattac ccctttaggg cgcgccgcag gctaggtcat gtcatcttga
     1501 cccgtctata aatgttagcc ctctaaggcc gaccgcaggt cgtagtatta cccttagagg
     1561 gcgttaaacc tttagggtga gttcactgca ctatttaaag gattttaatc cacgtgacgc
     1621 acatttgttg tacagtatgt ctaaacgtgc atcacctaac gcaattcgac ccagagccat
     1681 ggccaaatat ggccaggctg gggcgaaacc tatctatgcc aaaggtccaa ttccctacga
     1741 ggtatctgca ccgaatcgtt tctggcggat caagaaggat ctgccaccac aagagtattg
     1801 gaaaaagcga tattggcgtc gtaggatcac tggtaaggga gcctactcga tgaacgcagg
     1861 tgattcgttt gggcgtcgat ggggaggtta tttaggatca aaagcaggag agttcattgg
     1921 aggctacgca cagcgtttaa ctggtttagg cgactataaa gttacacgga acgtgtttgc
     1981 tgatggtcgc cttcctgagg ttgttaacaa tcctcagggt ggtggcaccg taattcgctt
     2041 tcaagaatat ttaggtgaca ttattacttc agcgacagct aatacttttg acatccaatc
     2101 ttatgatatc aacgctgcta atccaaggac atttccttgg ttatctcaaa tagcagccaa
     2161 ttatgatcag tatgattttg agggtctgct atttgaattt aagagtacgt cagccgacgc
     2221 tttaaattca acgaatactg ctttaggttc agtcatgatg gctacgcaat atgacacaat
     2281 tgatcctcct tttgactcaa aattggacat gttaaattat gagtattcca catcgatcaa
     2341 accttccatg tctaccatgc atatggtaga atgtgatccc aaacaaacca ctatcaatga
     2401 gctttataca cttagcaatc aatctgttcc tccaaatgct gacccccgtc tatattttct
     2461 aggacgattt cagattgcta caactggttt ccaaggtctt tcagtaaatg ttggtcaact
     2521 tcatgttact tatcaagtaa gattacttaa accaaaatta tttactactc ttgggaatgg
     2581 catagatgat gctacgtata gatttggcgt aggtactact tatgttgggt atgataatac
     2641 ccatcctctt acattggttg gtgaagttcc gaatagtgcc gtagacaata ttcctcttgt
     2701 tataacacca actacaatta catttcctgt atttgaaggt aagcttgctt accgaattga
     2761 aattatatgg agaggtgcta ctgctgttaa tactgaacat ccagatcttt ctatcgctaa
     2821 tggtacactt atatcagtcg attcttttaa tatggatctt aatgctcaaa gtcgctgtgg
     2881 atatttactt ggtattgttt cagatggttc agctaatcct cttgttctta ctgtagctga
     2941 taatggtata ttgccttcag gcaataatga agttgtcatt agagttatgc aaacgaaccc
     3001 tatatttaaa gctgtgtaaa ataaataaaa attattttca aaatagaatt atttttaaat
     3061 tgtgtaattc ccatttctaa aaacgtgcgg aagtac
//
LOCUS       CruV-152                3096 bp    DNA     circular     24-FEB-2020
DEFINITION  Crucivirus-152 isolate SR5_34386.
ACCESSION   CruV-152
KEYWORDS    .
SOURCE      Crucivirus-152
  ORGANISM  Crucivirus-152
            Unclassified.
REFERENCE   1  (bases 1 to 3096)
  AUTHORS   de la Higuera,I., Kasun,G.W., Torrance,E.L., Pratt,A.A.,
            Maluenda,A., Colombet,J., Bisseux,M., Ravet,V., Dayaram,A.,
            Stainton,D., Kraberger,S., Zawar-Reza,P., Goldstien,S., Briskie,J.,
            White,R., Taylor,H., Gomez,C., Ainley,D.G., Harding,J.S.,
            Fontenele,R.S., Schreck,J., Ribeiro,S.G., Oswald,S.A., Arnold,J.M.,
            Varsani,A., Enault,F. and Stedman,K.M.
  TITLE     Direct Submission
  JOURNAL   Submitted (24-FEB-2020) Biology, Portland State University, 1719 SW
            10th Ave, Portland, Oregon 97201, USA
COMMENT     Bankit Comment: ALT EMAIL:ide@pdx.edu
            Bankit Comment: TOTAL # OF SEQS:451
            Bankit Comment: GAP: est. length - alignment

            ##Assembly-Data-START##
            Assembly Method       :: MetaSPAdes v. 3.12; IDBA-UD v. 1.1.1;
                                     MEGAHIT v. 1.0.3
            Sequencing Technology :: 454; Illumina
            ##Assembly-Data-END##
FEATURES             Location/Qualifiers
     source          1..3096
                     /organism="Crucivirus-152"
                     /mol_type="genomic DNA"
                     /isolate="SR5_34386"
                     /isolation_source="Water"
                     /country="New Zealand"
                     /collection_date=2012
     misc_feature    join(3085..3096,1..20)
                     /note="stem-loop"
     misc_feature    1..9
                     /note="nonanucleotide"
     CDS             59..1027
                     /codon_start=1
                     /product="Putative Replication Associated Protein"
                     /translation="MAARNNNAARNWCFTVNNWKEADLDVLKTLGTGTQVKYMVVGKE
                     VGESGTPHLQGFVVFQKTERFGAVKKVLPPGCHIEKAKGTWEHNLAYCTKEDKEALVM
                     GEAPMSRKRQGEAGAEAEKERWLKIRALAKEGKTEELEMEFPREATLYSEKLARIHMK
                     CAPRADLPSDTVVGVWIHGPSGAGKSYWCHHHLEVEGVKYDINEIYDKDLSQWWDGWR
                     IKDEVEHRPKHRVAYLDDLDKFNIKLARDLKVWTQEYEFNAQVKGDYLRIRPELVVVT
                     SQSTIEEIWEDDKTRCALLRRFRVGYKANREAPVVWEPMYVPSFFA*"
     CDS             1284..2702
                     /codon_start=1
                     /product="Putative CruV Capsid Protein"
                     /translation="MYGAPRKPARVNRGFRGYEQVNRPVYGRARRPYTRGRMVRARYK
                     PGPRYSYQRSTWLAPALRTVGSGVGNYLGGPVLGRMGGQAGAMLGDAIKTMTGFGDYQ
                     IKENSLVLGSPPSVMNGALPGGSIIISHKEYLGDVVSSSVAGSFQLRSYNINPGDENT
                     FPWLSQIAGNFQQYKVMGMAFQFRTMSCDALNSTNTALGQVIMATNYDCTQQVFLSKP
                     EMENCEYAQSVKPSESCLHLLECDTSTMPIETLYVRTDEIPAGTDRRMYDMGKFQIAT
                     NGFQGNSVNAGELWVTYQIMLLKPKLNDALGEDTAYWIGWNNVSIAASTPLGAIAYWA
                     EPENPQVNTSQVSITSDRIITFEPSAVPRCWRVGISWVGAATGTLVAPTLAYTNCTGA
                     YCIRPTSTQQVSEQYPHSAAGAASTRFYLEFYVRTNGAGIVSYVTLGTGGTLPASCTR
                     VQVQVISIPLDAGELDGDGMYE*"
ORIGIN
        1 tagtattacc ccagaccccc gcgttacagt tacacttttc attttcctcg tgtgaagtat
       61 ggcagcgaga aacaacaacg ccgcgcgtaa ttggtgtttc accgtgaaca actggaagga
      121 ggccgacctt gacgtcttga agaccttggg tactggaacg caagtgaagt acatggtagt
      181 tggcaaggag gtgggcgaga gcggaacacc gcatttgcag ggcttcgtag tgttccaaaa
      241 aacggagcgt tttggagcgg tgaagaaagt gcttccaccg gggtgtcata ttgagaaggc
      301 gaaaggcact tgggagcaca acttggccta ctgtaccaag gaggacaagg aggccttggt
      361 gatgggcgaa gcacccatgt ctaggaagcg tcaaggcgaa gcgggagcgg aagccgagaa
      421 ggagcgctgg ttgaagatcc gagctttggc gaaggaagga aagactgagg aacttgagat
      481 ggagttccct cgagaagcca cgctttactc ggaaaagctg gcgaggatac acatgaagtg
      541 cgcccctcgg gcggacttgc cgtcagacac agtagtcggt gtgtggatac acggccctag
      601 cggagcgggc aagtcgtact ggtgtcacca ccacctggag gtggaaggtg tgaagtacga
      661 cataaatgaa atttatgaca aggacctcag tcaatggtgg gacggatggc ggattaagga
      721 tgaagtcgag catcggccca agcatcgtgt ggcctatctg gacgacttgg acaagttcaa
      781 catcaagttg gcgcgtgacc tgaaagtgtg gactcaggag tacgagttca atgcacaggt
      841 gaagggcgat tacctgagga ttcgcccgga gcttgtagtg gtgacctctc aatctacgat
      901 tgaggagatc tgggaagatg acaagacccg gtgtgcactt ctgaggcgtt ttagggttgg
      961 ttacaaggct aaccgggagg ccccagttgt gtgggagcca atgtatgttc cttctttttt
     1021 tgcttaaata aatccagaga tttttttaag ttgtctttta tttcttttgt gactttttca
     1081 ctgaaaagtc cacgcagctg cagactttgt attttgctgt atttttctgt ttcaccacgg
     1141 cgaaacggaa attcgaggag tcggtctacg accaagggag tagcttagtg ccatacggag
     1201 aagcgggcta tataccatat agcaatcgta attcgaagtt tacgaagaat tatcacgagg
     1261 atctaggaaa tattggagcg aatatgtatg gagcgccacg aaaaccagcg agagttaacc
     1321 gagggttccg aggttacgag caggtcaaca ggccagtata tggacgagca cgtcggcctt
     1381 acacgagagg ccgaatggta cgggcgaggt acaagccagg ccccaggtac tcgtatcaaa
     1441 gaagtacatg gctggcgcct gctctcagga ctgttggttc tggtgtgggg aattatctcg
     1501 gcggtccggt tttgggaaga atgggtggac aggcaggagc catgcttggg gacgcgataa
     1561 agacgatgac tggatttggt gactaccaaa tcaaggagaa cagcctggtt ttgggcagtc
     1621 ccccaagcgt gatgaatgga gcacttcctg gaggctccat tatcatcagt cacaaggagt
     1681 acttaggcga cgttgtgtcg agttcagtgg ctggatcatt ccagctcagg tcgtataaca
     1741 tcaatcccgg tgatgagaat acattcccat ggctgagtca aattgctggc aattttcagc
     1801 agtacaaggt gatgggaatg gcctttcagt tcaggacgat gtcttgcgat gcgctgaatt
     1861 cgacgaatac agcgttgggg caggtcataa tggctactaa ttatgactgc acccagcaag
     1921 tgttcttgtc caagcctgag atggagaatt gcgagtatgc gcaatctgtg aagccctctg
     1981 agtcgtgctt gcatcttttg gagtgcgaca ccagtacaat gcctattgag acattgtacg
     2041 ttcgcactga tgagataccg gcgggtaccg atcggcggat gtacgacatg ggcaagttcc
     2101 agattgccac caatgggttc caagggaact ctgtgaacgc tggagagctt tgggtgacct
     2161 atcagattat gcttctgaag cccaaattga atgatgcttt gggcgaagat acagcatact
     2221 ggatagggtg gaataacgta tcgattgcgg cgagcacacc tttaggagca attgcgtatt
     2281 gggctgaacc tgagaaccct caggtgaaca cgagtcaagt gtcgatcaca agtgatagaa
     2341 ttatcacttt tgaaccttct gctgttccaa ggtgttggag agttggaatc tcatgggttg
     2401 gagctgctac agggacattg gtggcaccta ctttggccta tactaattgc actggtgcat
     2461 attgtataag gccaacttcg acacagcagg tgtcagagca gtatccgcac tctgctgcgg
     2521 gtgctgcgag tacgagattc tatttagagt tttatgtgag aaccaatggc gcagggattg
     2581 tgtcttacgt taccttgggc actggaggca ctttgccggc tagttgcaca agggttcaag
     2641 ttcaggttat aagcattcct ttggatgctg gtgaacttga tggagatgga atgtacgaat
     2701 aaattttttt tcaacgaaac aaaacattta tttagttagg actctgactc tgcctctgac
     2761 ccacccatct caccacttta ctaatttcgg gaagtcggcc cgcggctgcg ggacgatggc
     2821 aaggcaaaac accatcagtg gctacctaac atcgagcgaa gcgaagcgag cgagtcatac
     2881 accctccaat actcacgtct aaaaatggtg acgtggcccc cggacgaggg ggccataaag
     2941 tgagccgtta ggcgaatgtt aggcgaacgg acgtgacgcc ctggttgtgc aaactaccag
     3001 gcgtttatgc aattccaaga aaagtgacgt ggcaaggtgg gaagttagtt cctaccgcct
     3061 tataaaaatg gcttgtcaca tcgggggggt ctgggc
//
LOCUS       CruV-153                3097 bp    DNA     circular     24-FEB-2020
DEFINITION  Crucivirus-153 isolate 001752_virusbaton.
ACCESSION   CruV-153
KEYWORDS    .
SOURCE      Crucivirus-153
  ORGANISM  Crucivirus-153
            Unclassified.
REFERENCE   1  (bases 1 to 3097)
  AUTHORS   de la Higuera,I., Kasun,G.W., Torrance,E.L., Pratt,A.A.,
            Maluenda,A., Colombet,J., Bisseux,M., Ravet,V., Dayaram,A.,
            Stainton,D., Kraberger,S., Zawar-Reza,P., Goldstien,S., Briskie,J.,
            White,R., Taylor,H., Gomez,C., Ainley,D.G., Harding,J.S.,
            Fontenele,R.S., Schreck,J., Ribeiro,S.G., Oswald,S.A., Arnold,J.M.,
            Varsani,A., Enault,F. and Stedman,K.M.
  TITLE     Direct Submission
  JOURNAL   Submitted (24-FEB-2020) Biology, Portland State University, 1719 SW
            10th Ave, Portland, Oregon 97201, USA
COMMENT     Bankit Comment: ALT EMAIL:ide@pdx.edu
            Bankit Comment: TOTAL # OF SEQS:451
            Bankit Comment: GAP: est. length - alignment

            ##Assembly-Data-START##
            Assembly Method       :: MetaSPAdes v. 3.12; IDBA-UD v. 1.1.1;
                                     MEGAHIT v. 1.0.3
            Sequencing Technology :: 454; Illumina
            ##Assembly-Data-END##
FEATURES             Location/Qualifiers
     source          1..3097
                     /organism="Crucivirus-153"
                     /mol_type="genomic DNA"
                     /isolate="001752_virusbaton"
                     /isolation_source="Water"
                     /country="France"
                     /collection_date=2013
     CDS             1..1137
                     /codon_start=1
                     /product="Putative Replication Associated Protein"
                     /translation="MHKYTTCKMANPKARCWTFTWNNYTDDQVNYLMDIPKERCDYLI
                     FGFEISPTDNTPHLQGYIEFSCPLAKTTVKSRLDPIKKSKSTVHVEMAVKGKEANRLY
                     CMKEDSADPDKTAKYGSKYIEIVHKFKKQGDRTDWHNMHDFLKETPNFGKFAAEFPEA
                     AIKYHGGIDRLIQSIEETQQAESFSAQFDETELRNWQQKLLDELKGKPNDRHIIWIYD
                     TVGGMGKSWMTDYLVSKHDAEFFENASTKDIACAWKGKPIAVFDFSRTTEERINYGVL
                     ESIKNGRIFSAKYNSTAKLFAKPHVVCFANWLPRMSAMSQDRWSIRCLDRNQEATVEN
                     PITNISEFINPEMHPRNTVSPKVKTGADAESEEDNIESREDVDN*"
     CDS             complement(1488..2840)
                     /codon_start=1
                     /product="Putative CruV Capsid Protein"
                     /translation="MKRQPARRQYTQAEKAAYYKKLSAPQQRIPRQPVQSRALQVAKP
                     RAVEKAPGMVSRAGQGVGGAIGSALGPVGTAIGSFLGGKLGHLVEKVTGFGDYKVEQN
                     SVLVGGMNVPQIVNSVEKGGVIIRHREFIQDIVATTAFTNQSFLIQPGLARTFPWLSQ
                     IANSFEQYRIRGMMFDFVSTSSDALLSSATSTALGTVVMMTDYDVADSAPTSKREMLQ
                     SEFASSDKPSCTFIHPIECKKSVTAQNILYTRGAIAPPENFDQRLYDFASFNIATEGM
                     QAAGGVLGELWVTYEIELLKPQFSYFGLADHYRMNTITTARPFGTVLGSNIGAGATLG
                     GAIAADALSYRFPPEVTSGKFLVTYSVVGSAVASAHTPSFSITGGSLLSIQVNDTLAW
                     INAPEPAATSGIIIITAVVTVTSGACYILLANNGLMPTSNNGDFWVVRLPDSIAARPT
                     *"
ORIGIN
        1 ttgcacaaat atacaacgtg caaaatggca aacccaaaag caagatgttg gacattcaca
       61 tggaacaact ataccgatga tcaagtgaac tatctcatgg atataccaaa agaaaggtgt
      121 gattatctta tattcggctt cgaaatctcg ccgaccgata acacccccca tttacaaggg
      181 tatatagaat tttcatgtcc gctggccaaa acgaccgtga agagtagact cgacccgatc
      241 aaaaaatcca aaagcacagt ccatgtagaa atggctgtaa aaggaaaaga agcaaatcgt
      301 ctgtactgca tgaaagaaga ctcagcagat ccagacaaaa ccgcaaaata cggtagcaaa
      361 tacatagaaa tcgttcacaa gttcaaaaaa caaggtgacc gcacagactg gcacaacatg
      421 cacgatttct taaaagaaac accaaacttc ggcaaatttg cggccgagtt cccggaagcc
      481 gcaataaaat accacggtgg gatcgaccga ttgatacaat cgatagaaga aactcagcaa
      541 gctgaatcct tctcagccca attcgacgaa accgagctga gaaactggca acagaagctc
      601 ttagatgagc taaaaggtaa accaaacgac agacatatca tttggattta cgacactgta
      661 ggaggaatgg gtaaatcatg gatgaccgat tacctcgtgt ccaaacacga cgccgaattc
      721 ttcgagaatg cgtcaaccaa ggacattgca tgcgcttgga aaggaaaacc tatagccgtg
      781 tttgacttca gcagaacgac tgaagaaaga atcaactacg gtgtattaga atctatcaaa
      841 aacggacgaa tcttcagtgc aaaatacaac tcaacagcta agctgttcgc taaaccacat
      901 gtggtttgtt ttgcgaactg gctgccaaga atgtcagcta tgtcacaaga ccgatggtct
      961 atacgctgtc ttgacagaaa tcaagaagcg acagtggaaa atcctataac caacatttcc
     1021 gaatttatca atccggaaat gcatccgcgt aatactgttt ctcctaaagt caaaaccggg
     1081 gcggatgctg aaagtgaaga agacaacatc gaatctagag aagatgttga taactaaact
     1141 gcggggcctc cctcgtcgct aaagctcctc gccctcgaca ggctcggggt aggcgggccc
     1201 ctaagaagaa tacacctctc agggggtacc cccctggccg gcagccgcat cgaccgtacc
     1261 ggtctcgcgt ctttggccaa cccccttctg tcacggtgtc ctccgggcct aggccctccg
     1321 gaaaatataa gaatacatag agatagatac tcgagtctca tctccggtgc tattttgatg
     1381 gaaaccatac aaagcccaaa atgcgctcct cgatttcgag cctcgcatgg ctcgggctaa
     1441 gtgcgccctc gctaaaagag acaaaaaaag tattcattat tttttgatta tgtaggacgg
     1501 gcagcaatgg agtctgggag acgaacaacc cagaaatctc cattgtttga agttggcata
     1561 agtccattat tagccaaaag aatataacat gcgccagacg taactgtgac tacagcagtt
     1621 ataattataa taccggaagt agctgccggt tcaggagcat taatccaagc aagagtatca
     1681 ttgacttgaa tagaaagcaa cgacccaccg gtgatggaaa aactaggagt gtgagcactg
     1741 gcaacagcgc taccaacaac ggaataagtg accaagaatt tacctgatgt aacttctgga
     1801 ggaaacctat aagacaatgc atcggcggca atagcaccac caagagtagc accagcgcca
     1861 atgttggaac ctagaactgt accaaaagga cgagcagtag taatcgtatt cattcgataa
     1921 tgatccgcta agccgaagta agagaattga ggtttaagaa gctcaatctc atatgtaacc
     1981 cataactctc cgagaacacc accggcagct tgcatacctt cggttgcaat gttaaaagaa
     2041 gcaaagtcat aaagacgttg atcaaaattc tctggaggag caattgcacc acgagtatag
     2101 agaatattct gagcagtaac cgatttcttg cattcaatcg gatggataaa agtacaagat
     2161 ggtttgtctg aagaagcaaa ctctgactga agcatctctc ttttagatgt cggcgcagaa
     2221 tctgcgacat cgtaatcggt catcataacc actgtaccga gtgctgtact tgtggctgaa
     2281 gacaacaacg catcggacga agtcgaaaca aagtcgaaca tcatacctct aattctatac
     2341 tgctcaaacg aattagcgat ctgggataac cagggaaaag ttcttgctaa ccctggttga
     2401 atcaaaaagg attgatttgt gaatgcagtc gtagctacaa tatcttgaat aaactcgcgg
     2461 tgtcgaataa tgactccacc cttctcaaca gaatttacaa tctgaggaac attcattcct
     2521 cctacaagaa cagagttttg ctctacctta tagtctccaa aaccagtaac cttctcgact
     2581 aagtgtccaa gttttccacc gaggaaggaa ccaatagcag taccaactgg tcctaatgct
     2641 gaaccaatag caccacctac accttgaccg gcgcgagaca ccataccagg ggccttttca
     2701 acagccctag gttttgcaac ctgaagagcc cgtgattgaa cgggttgacg gggaatccgt
     2761 tgttggggcg cagacaattt cttgtaatac gccgcctttt cggcttgcgt atattgtcga
     2821 cgagcaggtt gtcttttcat cgaacatact tagtgaaaga caaatttcta aaagaaataa
     2881 taaatattgc taaaatgtgc aatacgcttc tcatagatca gctacaaaag ttaaagccgt
     2941 gacaggaaac acaccgagga cttggcagcg cctctaaata ctgtagcaat ttttgctaca
     3001 gaagtatata gagttttgac ccgtagctaa aactcatctg acaaaatcaa ataattaatg
     3061 tgctacataa ttattattta aacaaacaaa gtatatt
//
LOCUS       CruV-154                3099 bp    DNA     circular     24-FEB-2020
DEFINITION  Crucivirus-154 isolate 001749_virusbaton.
ACCESSION   CruV-154
KEYWORDS    .
SOURCE      Crucivirus-154
  ORGANISM  Crucivirus-154
            Unclassified.
REFERENCE   1  (bases 1 to 3099)
  AUTHORS   de la Higuera,I., Kasun,G.W., Torrance,E.L., Pratt,A.A.,
            Maluenda,A., Colombet,J., Bisseux,M., Ravet,V., Dayaram,A.,
            Stainton,D., Kraberger,S., Zawar-Reza,P., Goldstien,S., Briskie,J.,
            White,R., Taylor,H., Gomez,C., Ainley,D.G., Harding,J.S.,
            Fontenele,R.S., Schreck,J., Ribeiro,S.G., Oswald,S.A., Arnold,J.M.,
            Varsani,A., Enault,F. and Stedman,K.M.
  TITLE     Direct Submission
  JOURNAL   Submitted (24-FEB-2020) Biology, Portland State University, 1719 SW
            10th Ave, Portland, Oregon 97201, USA
COMMENT     Bankit Comment: ALT EMAIL:ide@pdx.edu
            Bankit Comment: TOTAL # OF SEQS:451
            Bankit Comment: GAP: est. length - alignment

            ##Assembly-Data-START##
            Assembly Method       :: MetaSPAdes v. 3.12; IDBA-UD v. 1.1.1;
                                     MEGAHIT v. 1.0.3
            Sequencing Technology :: 454; Illumina
            ##Assembly-Data-END##
FEATURES             Location/Qualifiers
     source          1..3099
                     /organism="Crucivirus-154"
                     /mol_type="genomic DNA"
                     /isolate="001749_virusbaton"
                     /isolation_source="Water"
                     /country="France"
                     /collection_date=2013
     misc_feature    join(3088..3099,1..20)
                     /note="stem-loop"
     misc_feature    1..9
                     /note="nonanucleotide"
     CDS             complement(111..1097)
                     /codon_start=1
                     /product="Putative Replication Associated Protein"
                     /translation="MDRTAPPAPTAPEGAETAVSEPITDISKKSRNYCFTLNNYQPTD
                     AKTILGWKDTKYIVMGEEVGESGTPHLQGYVEFKEGKPVQPTLKRLDPRIHWETRRAL
                     ATSAAAYCKKGTQSKTEWSSLGIKGPNYGKGAVVHEFGKISEQGKRSDLKECAEMIVN
                     GEPMREVALHNPAAYVKYHTGFEALRTVLYEHRKTKPTVIWRWGPAGAGKTAGPVEAH
                     PDHYIKDGTIWWNGYTQQQAIIIDDFEIKGLDYRAFLRLLDRYHYLAQYKGGYIPINS
                     PYIYITCDHPPSSFWTGNELAQVIRRIDEIQEVTAKNGRPIAVPVVRKVDGV*"
     CDS             complement(1222..2562)
                     /codon_start=1
                     /product="Putative CruV Capsid Protein"
                     /translation="MPRAMIKAAAPRRKPAKAPKGPKPPKAPYSYDRPGPWGNFGRAA
                     GGAVGSILGGGPGKMLGSKLGSYLHYIGKIFGSGDYVTSADQVRNNILVSEAQAPAFG
                     HGRNTVHIRHREFLGDIVTSGSANTFNLQSYPIQPGLSSSFPWLSNVCGTSFQQYRIN
                     GMVFEFRSMSADALNSTNTALGSVVMATDYDSKDANFQSKAQMENTEFGVSCKPSSCM
                     IHAIECAKSQTSVSELYIRNTGVPSGADIRLYDMGNFQIATTGFQGTSVNVGELWVSY
                     DITLFKAIQLQPGAGILQYHRNLAATTAAQPLLQDVSVYATQPQYDNIGCSVNTANTI
                     LTLPVGLPLRTRFVMVYAMRGASTAGVVAPTATASQGLASHSGFINAGSAGFNAPQTA
                     TGTTLILVQMYEITAVSAASAPPIITFSGAGVFPGTTQGGDLYLIQMSASDGLV*"
ORIGIN
        1 tagtattacc ttacaccact tctgtggaga tttgttaaaa gctcctgtga gactattagt
       61 ctctgttggg tgccctagta aaaaataggg ctcatcaact tctgtggagg tcataccccg
      121 tcgacttttc gtactacggg gacagctata ggtcttccat tcttcgctgt gacctcttgt
      181 atctcgtcta tccgtcgaat tacttgcgcg agttcgttgc ccgtccagaa ggacgaaggc
      241 ggatgatcgc atgttatgta gatgtatggc gagttgatcg gtatatatcc tcctttgtac
      301 tgcgccaggt aatggtagcg gtcaaggagg cgcaggaatg ctcgatagtc gagacctttg
      361 atttcgaagt cgtcgattat gattgcttgc tgctgtgtat atccgttcca ccagattgtg
      421 ccatctttga tgtaatgatc tggatgcgct tctacaggtc cagctgtctt gcctgcgccc
      481 gcagggcccc agcgccagat tacagttggt tttgtttttc tgtgctcgta gagtacggtt
      541 cgtagcgctt caaatcccgt atggtatttg acatatgctg ccggattgtg taacgcaacc
      601 tctctcattg gttcgccgtt aacgatcatt tcggcgcatt ctttgaggtc agatctcttg
      661 ccttgctctg atatcttgcc gaactcgtga acaacggccc cttttccata attgggccct
      721 ttgatgccga gtgatgacca ctcggtctta gattgggtgc cttttttgca gtaagccgct
      781 gcgcttgtcg caagtgctct gcgggtctcc caatgtattc gcgggtcaag gcgttttaac
      841 gtcggttgga ctggtttacc ttctttaaac tcgacgtagc cttgaagatg cggggtgcct
      901 gattccccga cttcttcgcc catgacaatg tactttgtat ctttccaccc gaggatggtt
      961 ttagcgtccg tgggttggta gttgttcagg gtaaagcagt agttccggct tttctttgat
     1021 atgtccgtaa ttggttcact tactgctgtt tctgcgcctt caggtgctgt gggggcaggg
     1081 ggcgctgtgc ggtccatgca ctcatcgatc gctttttcta gctttcgatt cattgtttga
     1141 tcgctatata gcgaagccgg atattctggc tcaaaaaata ttaataccgt aaggtatcgt
     1201 caggaagcgg ccgtttgacg tttagactag cccgtcactc gctgacattt gaatcagata
     1261 caagtcacct ccttgagtcg ttccgggaaa cacaccagca ccgctgaatg tgataatcgg
     1321 tggcgctgat gcggcagaca ctgcggtgat ttcgtacatc tgtaccagaa tgagggtggt
     1381 tccagttgct gtctgtggtg cgttgaagcc cgcagatcca gcgttgataa atcccgagtg
     1441 gcttgcgagg ccttggcttg ctgtagctgt cggggctacg accccggcgg tagaagctcc
     1501 tcgcattgca tagaccatta cgaatcgggt ccggagaggc aatccgaccg gaagagttaa
     1561 gatagtgttg gcagtattta cgctacagcc gatattgtca tattgtggct gtgttgcgta
     1621 tacgctcaca tcttgtagca acggctgtgc ggccgttgta gctgctaggt tcctgtgata
     1681 ttgcaggata ccagcccctg gctgcagttg gatagctttg aacagtgtga tgtcgtagga
     1741 tacccatagc tctcctacat tcacgctggt tccttgaaac ccagtcgtag cgatctggaa
     1801 gtttcccatg tcgtagagcc gtatatcggc tcctgatggc actcctgtgt tcctgatgta
     1861 gagttctgac acgcttgtct gacttttcgc gcattctata gcgtgaatca tgcacgatga
     1921 gggcttgcac gacacaccga actcggtgtt ttccatttgc gctttcgatt gaaagtttgc
     1981 atctttgctg tcgtagtcag tggccattac cactgaacct agggcagtat ttgttgagtt
     2041 cagggcgtca gcgctcatgc ttcggaactc gaacaccatt ccgttaattc tgtattgctg
     2101 gaacgatgta ccgcatacgt tggacaacca ggggaacgat gaggacaaac cgggttgaat
     2161 cgggtacgat tgtaggttga acgtgttagc ggacccggag gtcacaatgt ctcctaggaa
     2221 ttcacgatga cgaatatgca cggtattcct gccgtgcccg aatgcgggcg cttgcgcttc
     2281 actcactaag atgttatttc ttacttggtc ggctgatgtc acgtagtcgc cagaaccgaa
     2341 gattttgccg atgtagtgga gatacgatcc aagttttgag cctagcattt tacccggccc
     2401 tcctcctagg atgcttccaa cagcccctcc ggcagctctg ccaaagtttc cccaaggccc
     2461 tggcctgtcg tatgagtagg gtgcttttgg aggtttgggc cctttgggtg ctttcgcggg
     2521 tttcctgcgt ggtgccgctg ctttgatcat agctctaggc atctttgaac gatgagggct
     2581 cgtatacggt gcccgctata gttcttattg gaatcaatta attaactatt aattatgagc
     2641 ggctagaact gtccggaaag tcttgctttc ggacctttta tcgctcgaat aaataattaa
     2701 tcatataagc catcggcttt cagatcgggc caattggtct cgtagatgtt aaaaaagggg
     2761 atcggctatc gatccggcca caggtcggct aatagatcag gggtgccgta ggcagaaacc
     2821 catccatata tgccccttcc aagcggggca ggctggccga atggccggcc ggggcgcccc
     2881 cggcgttagc cggcgaggag ggcggcgccg tagatcgcga agcgattacg ggcgcaacgc
     2941 cccgacgcag caacggccag ccggaatagg cgccacgagt cggtcagagg gcaccaggag
     3001 cgcagcgact ggccagctaa cccgcgcaca ctctcccccc taggtagagg tcaaaaaaga
     3061 gaactgtttc ttactgttca ttttcacagt ggtgtaagc
//
LOCUS       CruV-156                3105 bp    DNA     circular     24-FEB-2020
DEFINITION  Crucivirus-156 isolate 120_1015_SvL1_3295.
ACCESSION   CruV-156
KEYWORDS    .
SOURCE      Crucivirus-156
  ORGANISM  Crucivirus-156
            Unclassified.
REFERENCE   1  (bases 1 to 3105)
  AUTHORS   de la Higuera,I., Kasun,G.W., Torrance,E.L., Pratt,A.A.,
            Maluenda,A., Colombet,J., Bisseux,M., Ravet,V., Dayaram,A.,
            Stainton,D., Kraberger,S., Zawar-Reza,P., Goldstien,S., Briskie,J.,
            White,R., Taylor,H., Gomez,C., Ainley,D.G., Harding,J.S.,
            Fontenele,R.S., Schreck,J., Ribeiro,S.G., Oswald,S.A., Arnold,J.M.,
            Varsani,A., Enault,F. and Stedman,K.M.
  TITLE     Direct Submission
  JOURNAL   Submitted (24-FEB-2020) Biology, Portland State University, 1719 SW
            10th Ave, Portland, Oregon 97201, USA
COMMENT     Bankit Comment: ALT EMAIL:ide@pdx.edu
            Bankit Comment: TOTAL # OF SEQS:451
            Bankit Comment: GAP: est. length - alignment

            ##Assembly-Data-START##
            Assembly Method       :: MetaSPAdes v. 3.12; IDBA-UD v. 1.1.1;
                                     MEGAHIT v. 1.0.3
            Sequencing Technology :: 454; Illumina
            ##Assembly-Data-END##
FEATURES             Location/Qualifiers
     source          1..3105
                     /organism="Crucivirus-156"
                     /mol_type="genomic DNA"
                     /isolate="120_1015_SvL1_3295"
                     /isolation_source="Water"
                     /country="Norway"
                     /collection_date=2012
     CDS             1..1308
                     /codon_start=1
                     /product="Putative Replication Associated Protein"
                     /translation="MNGEDTVWRKEKSVVKPTEFNGFGDWTEGKPKDPIVVGGPNHIS
                     SQPTSGKGRPGSKKSSRDWCYTYWGPESSIDPGLYEKSTVKFGDHVWLETLHDVPTNA
                     VVYHVFQFEISPDTGGRHIQGFISFATRRRFATVRKVLPRCHLAPRRGTVNQAADYCM
                     KMDSKDGDYQFEYGTRPEDDLGQGHRSDIDAIKELIDDGASMKIVADFDFGYYVGHYR
                     GLEKYRSLSTKGRAKKSLVFLFVGGPGTGKSAAAATFLDNYAVPPGSSGTVWYDEYEP
                     GHHKTLIFDDFTGAWCQWRELLRITDEYPMLANTKGGHIPFKPEAIVFTANYSPERWY
                     SSEAVPDKAPFMRRVDYHWQYYLKHATNAEWMIPDAVRVALLGFEERHPMLPVHSVCR
                     CLKGDPTYHPHYGRYCAFDVDGETYYAVISQKKPEVVLPKLWV*"
     CDS             1348..2709
                     /codon_start=1
                     /product="Putative CruV Capsid Protein"
                     /translation="MPFPTYRRAARRAPLKTYVRPRRAYAPKRRSFPAYRGKGAYTAP
                     KSAAWGQAAGSALGSIAGFIPQTKAFAAPIAALASKLGGFLGNKLGTYMGWGDYSVNM
                     NSLLVPEGNSPAAMHSDGKSIRICHREYIGDVISSSNAKEFTLQNFQINPASSIAFPW
                     LWNIAVNFQKYRLHGAIVEFKSSSGDALTTADTSLGTVLIASNYNCADDNFQNRTQME
                     NTQYTSSAKPSVSFVHIIECDPHLQTQTSLYTAVDGIPHPGTSINEVNWVNVQVASLG
                     MQGVNVNLGSIYITYDIEMIQPINQADSTVFRGDWFALSGASGDWISNVFPNNEPNPE
                     NSLGGTIVSGDTYVFPFNIQSGLFRMIYYCGPETPAGALGLPAFTGLNCALVNTMPTP
                     NAGGDPTSSIAGQGGADCMMATFTFKITGSGAKISATAPGTFAAGISRGGLLIDQIDL
                     DYA*"
ORIGIN
        1 atgaatggtg aagatacagt atggcgtaaa gaaaagtcag tcgttaagcc gactgaattt
       61 aatggctttg gcgattggac ggaaggcaaa cctaaagacc caatcgttgt tggtggacca
      121 aatcatattt cgtctcagcc gacaagcgga aagggtagac cgggctcaaa gaaaagttca
      181 cgagactggt gttacactta ttggggacct gaatcatcca ttgaccctgg actttatgaa
      241 aaaagtacag tcaaattcgg agatcatgtg tggttggaaa ctcttcacga tgttcccaca
      301 aacgctgttg tgtaccacgt ttttcaattt gagatatcac cagatactgg tggaagacac
      361 attcagggct tcatctcttt tgcgactaga cgtcgtttcg caacagttcg taaggtattg
      421 ccacgttgtc accttgcgcc tcgtagagga actgttaatc aggctgcgga ttattgtatg
      481 aaaatggatt caaaagatgg tgattatcaa ttcgagtatg gtacgagacc tgaggatgat
      541 cttggacaag gtcaccgaag tgatattgat gctatcaaag agttgataga tgatggcgcc
      601 tcaatgaaaa tcgttgctga ttttgacttt ggctattatg ttggacatta tagaggtctt
      661 gagaaatatc gatctttatc aacaaaaggg cgtgccaaaa aaagccttgt gtttttattt
      721 gttggtggtc ctggtactgg aaagtctgct gctgctgcta ctttcttgga caattatgca
      781 gtccctcctg gttcttcagg gactgtttgg tacgatgagt atgagcctgg tcatcacaaa
      841 actttgattt ttgatgattt cactggcgca tggtgtcaat ggcgagagct attgaggatt
      901 acagatgaat atccaatgct tgctaacaca aagggtggac atatcccttt caaacctgag
      961 gccattgttt ttacagcaaa ttattctcct gaaagatggt atagtagtga agctgtacct
     1021 gataaggcgc cttttatgag gcgtgtagac tatcattggc aatactactt aaaacatgca
     1081 actaatgctg aatggatgat acctgatgcg gtgcgtgtag ctctgttggg atttgaggaa
     1141 cggcatccta tgcttccggt gcattctgtg tgcagatgct taaaagggga ccctacatat
     1201 catcctcact atggtagata ttgtgcattt gatgttgatg gagaaacata ttacgctgta
     1261 atatctcaaa agaaaccaga ggttgttcta ccaaaacttt gggtataaac ttttcaaagt
     1321 atattcttat tgtcacaaga ctaaagtatg ccttttccta catatcgtcg tgctgctcgt
     1381 agagcaccac tcaaaactta cgttcgtcct cgtagagctt atgctccgaa acgtcgttct
     1441 ttcccagcat accgaggtaa aggagcatat actgctccta aatctgctgc atggggtcaa
     1501 gcggccggta gtgcgcttgg gtctattgct gggttcattc ctcagacaaa ggccttcgct
     1561 gcaccaattg ctgctttggc atcaaagcta ggaggctttc ttgggaataa gttaggcact
     1621 tacatgggat ggggtgacta ttctgtcaat atgaatagtc ttcttgttcc tgagggcaac
     1681 tctcctgccg cgatgcattc tgatggaaaa tccattcgaa tttgtcatcg tgagtacatt
     1741 ggagatgtta tttcttcttc caatgctaag gaatttactc ttcaaaattt tcagatcaac
     1801 cctgctagct ccattgcttt tccatggtta tggaacatcg ctgtaaactt tcaaaagtac
     1861 agacttcatg gagctattgt tgagttcaag tcatcctctg gtgatgcgct cactactgct
     1921 gataccagcc ttggcacagt cctcattgcc agcaattaca attgtgctga tgacaatttt
     1981 caaaaccgca cgcagatgga gaacactcag tacactagtt ctgccaagcc ctctgtgagt
     2041 tttgttcaca tcattgaatg tgatcctcat cttcaaactc aaacttcatt gtacactgct
     2101 gttgatggaa tccctcatcc aggaaccagc atcaatgagg tcaattgggt gaatgttcaa
     2161 gtagcctcac ttggcatgca aggtgtcaat gtgaaccttg gttccattta catcacttat
     2221 gatattgaga tgattcaacc catcaatcaa gctgattcca ctgtgttccg aggagattgg
     2281 tttgctctct ctggcgcaag tggtgattgg atctccaatg tttttcccaa caatgagcca
     2341 aatcctgaga acagccttgg aggcactatt gttagtggcg atacttatgt attcccattc
     2401 aacattcaaa gtggcctctt tagaatgatc tattattgtg gtccagaaac tcctgctggt
     2461 gctcttggcc ttccagcttt tacagggctc aattgtgctc ttgtcaatac aatgcctaca
     2521 cctaacgctg gtggcgatcc tacaagctct attgctggac aaggaggtgc tgattgtatg
     2581 atggctacat tcaccttcaa aatcactggc tctggcgcca agatttctgc tactgctcca
     2641 ggcacctttg ctgctggaat ctcacgtggt ggtcttctca ttgaccagat tgatttagat
     2701 tacgcgtagt cgtgtgccgc aggccacgca gacccattaa actctcattc cactttacta
     2761 tgtcacctct ctacattcct ttttctatca ctcacctagt aaagggccgc caacgaggcg
     2821 cgggacccgc ttgcggggcg ccgaggctgg tggaccaatg tccccatcat atgtaatgta
     2881 gtagccctaa gaacccattg aagtagtcgt tgaagagagc gttagtgatc gtcggcgtgt
     2941 tctccctaga gggagaatct cgggggtcta ttgttacccc ccgagacttc gggttcaggg
     3001 cggataaaaa tcccctcatc cacaaaacgc gcctagcgtg tcttgaaaaa acgcgccatt
     3061 ccccctattg acttggtcaa cgactgtgtc tcaaaaaata attcc
//
LOCUS       CruV-157                3113 bp    DNA     circular     24-FEB-2020
DEFINITION  Crucivirus-157 isolate 001740_virusbaton.
ACCESSION   CruV-157
KEYWORDS    .
SOURCE      Crucivirus-157
  ORGANISM  Crucivirus-157
            Unclassified.
REFERENCE   1  (bases 1 to 3113)
  AUTHORS   de la Higuera,I., Kasun,G.W., Torrance,E.L., Pratt,A.A.,
            Maluenda,A., Colombet,J., Bisseux,M., Ravet,V., Dayaram,A.,
            Stainton,D., Kraberger,S., Zawar-Reza,P., Goldstien,S., Briskie,J.,
            White,R., Taylor,H., Gomez,C., Ainley,D.G., Harding,J.S.,
            Fontenele,R.S., Schreck,J., Ribeiro,S.G., Oswald,S.A., Arnold,J.M.,
            Varsani,A., Enault,F. and Stedman,K.M.
  TITLE     Direct Submission
  JOURNAL   Submitted (24-FEB-2020) Biology, Portland State University, 1719 SW
            10th Ave, Portland, Oregon 97201, USA
COMMENT     Bankit Comment: ALT EMAIL:ide@pdx.edu
            Bankit Comment: TOTAL # OF SEQS:451
            Bankit Comment: GAP: est. length - alignment

            ##Assembly-Data-START##
            Assembly Method       :: MetaSPAdes v. 3.12; IDBA-UD v. 1.1.1;
                                     MEGAHIT v. 1.0.3
            Sequencing Technology :: 454; Illumina
            ##Assembly-Data-END##
FEATURES             Location/Qualifiers
     source          1..3113
                     /organism="Crucivirus-157"
                     /mol_type="genomic DNA"
                     /isolate="001740_virusbaton"
                     /isolation_source="Water"
                     /country="France"
                     /collection_date=2013
     CDS             1..906
                     /codon_start=1
                     /product="Putative Replication Associated Protein"
                     /translation="MENTPVTKTTKLSKMSAVCMWDFRHTASKTNKDDIIKAMKSIAK
                     KWAFQLETSATGYVHFQGRFSLIKKHRKAELMTMWEAFTMPLPEYLEPSNSEAYKTDD
                     FFYTMKDETRTEGPWTNKTELDPSYIPKQYRGKMDNLYPFQKQIFDSANIFDDRIINL
                     IYDDSGNNGKSTIASLCELVGNGIDLPPINDMKELIQVACDICVAKETRNPSPVFVDM
                     PRAMDKARLNGIYTAIEQIKKGKLYDTRYHYKEWWIDAPQIWVFSNMEPDENLLSRDR
                     WKIWTINSNKELVAYETEEKNPLDL*"
     CDS             1504..2835
                     /codon_start=1
                     /product="Putative CruV Capsid Protein"
                     /translation="MYMPMKAGSSMRSRRRVTRPRRRVVSGRGFYRGFGRDVGKVIGY
                     GAGMVGRAYGAPEGTQNFLSNAGGYIGDKASQVTGWGSYKINQNNLLTNDPPMVANKK
                     LYGGDLATVIRHREFIGDVKSGPLPAGATSTEFNLNYELPINPGLPATFPWASAVAGN
                     YIQYSINGMLVEFVTTSGNAVSSTNSALGSVNLACQYDSILPSYANKQQMLAEEHAVS
                     GVPSCNLILPIECAPNQTTITKLYVRSGGTPVGTDIRMYDLGRISCATSGCQAPDVTL
                     GELWVTYDITLFKASLQNSVLDNGGYGAHWTIAFPSGVNQPTLGPNNLLANNIDGFNL
                     GVPATAGNNKFVFTNLAPNKYWNLQILASGTVGGGGVPTLILTNMVLFDQLGNQTTSS
                     AGQFSVTGVAYSVFLEIVDPNQNADFEVVGCPSSNGAAVFDFVVTPISDPF*"
ORIGIN
        1 atggagaata ctcctgttac taaaaccacg aaactttcta aaatgtccgc agtatgtatg
       61 tgggatttta gacatacagc ttccaaaacc aataaagatg acatcataaa agctatgaag
      121 tctattgcta aaaaatgggc ttttcaactt gaaacatcgg ctactggcta tgtccatttt
      181 caagggcgat tttctttaat aaagaaacat cgtaaagctg agttaatgac aatgtgggaa
      241 gcatttacta tgcccttacc agagtattta gaaccttcaa acagtgaagc gtataaaacg
      301 gatgatttct tctacactat gaaggatgag acaaggactg aaggaccttg gaccaataaa
      361 acggagcttg acccttcata tattcccaaa caatatagag gaaagatgga taacctttat
      421 ccatttcaga aacaaatatt tgattcagct aatatttttg atgaccgcat tataaacctt
      481 atttatgacg atagcggaaa taatggtaaa tctacgatag ctagtttatg cgaactggtg
      541 ggcaatggaa ttgacctacc accaattaat gatatgaaag aattaataca ggttgcttgt
      601 gatatatgcg tagctaagga aacgagaaac ccatctccag tatttgtaga tatgccaagg
      661 gcaatggata aagcacggtt aaacgggata tatacagcta ttgaacaaat aaaaaagggt
      721 aagttatacg acacccgcta ccattataaa gaatggtgga ttgatgctcc gcaaatatgg
      781 gtattttcta atatggaacc tgacgaaaac ttgttaagca gggacaggtg gaaaatatgg
      841 accatcaatt ccaacaaaga acttgtggct tatgagacag aagaaaaaaa ccctttagat
      901 ttataaaaaa taatatctaa tattttatat atggaatata gaaaatctag aataagaata
      961 caaaaatata ttgactatta ttaccaatgc ccgtcttgcg gtagagaatt taacgaaacg
     1021 atgggtcttg caggttacaa gagatttaaa atacatttaa acgggtgcaa tggtgttcct
     1081 cctgtggaac atgtgcaaca tgtggaacaa gtgaagactc ctgagcgtcc agaagaacca
     1141 ttttattatc aataaaaaaa tatctaatta ataatatata tatgagttca tatttatatt
     1201 ataacactaa gttttacaca ttcaacgaag ttgataaact tgttaagcag aaggaaacgg
     1261 agcttgacga tgcactctta ggctggaaga ctaatggcgg ttatgtatat tcagactata
     1321 acccaaaaat aaaggctaat acttttactg atgaagagaa ggaaaaagcg atacaaggca
     1381 ttagaagaat gaaaaatgat ttggagtttt taaaagctaa agttattaaa acttttacta
     1441 tagcccaaaa aaaaaaataa aaaaagtcta aaaataaaaa aataaaaata aaaatctctc
     1501 ttactgtata tgccaatgaa ggcaggttct agtatgcgtt cccgtagaag ggtaacaaga
     1561 cccagaagac gcgtagtttc tggtagaggt ttttacagag gttttggtcg tgatgtaggt
     1621 aaggtcatcg gctacggtgc aggtatggtt ggtcgtgctt atggtgctcc agaaggcaca
     1681 caaaattttt taagtaacgc tggtggatac atcggcgaca aggcttcaca agtgacaggt
     1741 tggggttctt acaaaattaa tcaaaataac ttattaacta atgaccctcc aatggtagcc
     1801 aataaaaaac tttatggtgg tgaccttgct acagttatta gacatcgtga gtttatcgga
     1861 gatgttaaaa gcggtccttt acccgctggt gctacatcaa cagaatttaa cttaaattat
     1921 gaattaccaa ttaatcctgg cttacccgcg actttccctt gggcatcagc ggtagctggt
     1981 aactatattc aatattctat taacggtatg ttagtagagt ttgtaacaac atcaggaaat
     2041 gcagtatcta gcacaaacag tgctttgggc tcagttaatc ttgcttgcca atacgacagt
     2101 atattaccat cttatgctaa taaacaacaa atgcttgcag aagaacacgc agtaagcggt
     2161 gttccatctt gcaatttaat acttcccata gaatgtgccc ctaatcaaac aacaattact
     2221 aaattatatg tccgtagcgg tggaacaccc gtaggaactg atatccgtat gtatgattta
     2281 ggaagaatta gttgtgcaac ttctggatgc caagccccag atgtgacatt gggcgaatta
     2341 tgggtaacat atgatataac attatttaaa gcttcattac aaaatagcgt attagacaat
     2401 gggggctacg gtgcacattg gactatagct ttcccttcag gtgttaacca acctacattg
     2461 ggtccaaaca atttacttgc caataatatt gatggtttta atcttggagt tcccgcgaca
     2521 gcgggtaaca acaagtttgt atttactaat cttgctccaa ataaatattg gaatttacaa
     2581 atacttgcat caggaactgt aggcggtggc ggtgtcccaa cattaatatt aactaatatg
     2641 gtattatttg accaattagg aaaccaaaca acatcttcag ctggtcaatt tagtgtaact
     2701 ggagttgcat attctgtatt tttagaaata gtagacccaa atcaaaatgc tgattttgaa
     2761 gtagttggtt gtccatcatc aaatggtgct gcggtttttg attttgtagt aactcccatc
     2821 tcagacccct tctgagcgta acatgtggaa catgtgaagc ctgattaaag tcttgatcaa
     2881 gctctgcttg actaagacgg ggctattatt ctatagccaa tcctataaag tatactttta
     2941 tactaaaata tactttatat aaaataaact ctttttatct taaactatgg aaaccctaaa
     3001 aaaatgtcta gttttttgtg ccaattgtgc caagaactta aagtatatta atagataata
     3061 atttgtgttt ttccagagtt tcactcgcca aattattatc ggggtgtata tta
//
LOCUS       CruV-158                3121 bp    DNA     circular     24-FEB-2020
DEFINITION  Crucivirus-158 isolate SR1_25615.
ACCESSION   CruV-158
KEYWORDS    .
SOURCE      Crucivirus-158
  ORGANISM  Crucivirus-158
            Unclassified.
REFERENCE   1  (bases 1 to 3121)
  AUTHORS   de la Higuera,I., Kasun,G.W., Torrance,E.L., Pratt,A.A.,
            Maluenda,A., Colombet,J., Bisseux,M., Ravet,V., Dayaram,A.,
            Stainton,D., Kraberger,S., Zawar-Reza,P., Goldstien,S., Briskie,J.,
            White,R., Taylor,H., Gomez,C., Ainley,D.G., Harding,J.S.,
            Fontenele,R.S., Schreck,J., Ribeiro,S.G., Oswald,S.A., Arnold,J.M.,
            Varsani,A., Enault,F. and Stedman,K.M.
  TITLE     Direct Submission
  JOURNAL   Submitted (24-FEB-2020) Biology, Portland State University, 1719 SW
            10th Ave, Portland, Oregon 97201, USA
COMMENT     Bankit Comment: ALT EMAIL:ide@pdx.edu
            Bankit Comment: TOTAL # OF SEQS:451
            Bankit Comment: GAP: est. length - alignment

            ##Assembly-Data-START##
            Assembly Method       :: MetaSPAdes v. 3.12; IDBA-UD v. 1.1.1;
                                     MEGAHIT v. 1.0.3
            Sequencing Technology :: 454; Illumina
            ##Assembly-Data-END##
FEATURES             Location/Qualifiers
     source          1..3121
                     /organism="Crucivirus-158"
                     /mol_type="genomic DNA"
                     /isolate="SR1_25615"
                     /isolation_source="Water"
                     /country="New Zealand"
                     /collection_date=2012
     misc_feature    join(3113..3121,1..16)
                     /note="stem-loop"
     misc_feature    1..9
                     /note="nonanucleotide"
     CDS             198..1244
                     /codon_start=1
                     /product="Putative Replication Associated Protein"
                     /translation="MSTASALNWVFTLNNYTPKGENELRESVLFEYIIYGHEVAPTTG
                     TPHLQGYFQLQKKKRLNYLLKKLPKGIHLEKAIGNYESNKTYCSKDAQDIYTRGTPSI
                     KGANTNQLYDLILQCRTWPEVLKLPNIERRMTFAREVFNNKPIIPQSNVKLLPWQQKV
                     VDIIKTPPDDRTIHIVYDEKGSKGKTFLAKYLYTNYDAYYISPSKSADLLYAYNGQQI
                     IIYDVPRSCDDDYVNWGAIEKLKDGIFFSGKFASGIKHREKHAHVIIFTNHPIPADKF
                     SVDRLNYIDLHTERNIHNLSEKSIHDILNSDDEDEEYEDEEELIPSTLDYIKKNIEAS
                     NEIDDEDKIILRFD*"
     CDS             1324..2643
                     /codon_start=1
                     /product="Putative CruV Capsid Protein"
                     /translation="MPNTKTATQQKFGPKNKPQPFHKKVWEQAYKKKNSWIRQGLKAG
                     GSALGSLLGGPVGSSLGTSAGNLIANLVGAGAYRQGEIKMNTSFNKLPETGVLHAGVP
                     TMHKVGSDIRVSHREYVCEVISSATANTFKVDKYPINPAMRNSFPWLSAVAQQYEQYK
                     VHGMVFEFRSNSGNALNSTNTALGSVVMGASYRPSTTTPTNKQMIMNFEWANETVPSK
                     DMIMGIECDPSSLAYKQYFTRGSNALPADDSVNQYDIANLFISTSGCQGTSVVLGSLF
                     VSYDIELQHSIDSADIGQWLHQAKYNIPSQTISSSNAGLSPCLGALMVSDGIGLSVSG
                     DTITFPIGSVGKYFINHSLRGSSATLGSWSFVLTNCTALNSFIGSSIGASMTPNAGVA
                     TGDENLEYTLTISDPAKIATFKFTNTALPSNWTSGEFFVIQLANSVF*"
ORIGIN
        1 tagtattacc caaaccttgg aacattttta taaatagaca acagtctatg ataaaaaccc
       61 tgacaacttt ttcagattat ttattaatca ccgatcaata aaccaaatat taatagtatg
      121 ctcatactct ttttcattta aacgcacctt ttttgaccaa aaaaaataaa taactatagt
      181 atttatataa ataaaccatg tccacagcat cagcgctcaa ttgggtgttc acgctaaata
      241 attatacacc aaagggtgaa aatgaattac gtgagagtgt attattcgaa tacataattt
      301 atgggcatga agtagcacct actacaggca caccgcattt acaagggtat tttcaattac
      361 aaaagaagaa aagactaaac tatttattaa agaaactacc gaaaggaatt caccttgaaa
      421 aagcaattgg aaattacgaa agtaataaaa cctattgttc aaaagatgca caagacatct
      481 atacacgtgg cacaccatca attaaaggcg ctaatacaaa tcaattatat gatttaatac
      541 tacaatgcag aacatggcca gaagttttaa aacttccaaa tatcgaacgc cgaatgacat
      601 tcgcgcgtga ggtttttaat aataaaccaa ttataccaca aagtaatgtg aaattactac
      661 catggcaaca aaaggtggta gatatcatca aaacaccacc agatgataga actatacata
      721 ttgtatacga tgaaaaaggc tcaaaaggta aaactttttt agcaaaatac ttatacacaa
      781 attatgacgc atactatata tcaccttcta aatcggcgga tttattatat gcatataatg
      841 gacaacaaat tattatttac gatgtcccgc gttcttgcga tgatgattat gttaactggg
      901 gcgcaattga aaaactcaaa gacggtatct tctttagtgg taaattcgca tcaggtataa
      961 aacatcgaga aaagcacgcg catgtaatta tatttacaaa tcatccaata cccgccgata
     1021 agttcagtgt cgatagatta aattatatcg atttacacac tgaacgaaat atacataatc
     1081 tatctgaaaa atctatccat gatattttga atagtgatga cgaagacgaa gaatatgagg
     1141 atgaggaaga acttatacca tcaacacttg attacatcaa aaaaaatatc gaggcgtcta
     1201 atgaaattga cgacgaagac aagattatac taagatttga ttagttttat ttaaaaaaat
     1261 attttatttt tttaaataat taatccacaa ctttattata cactataagt atataaatca
     1321 acaatgccaa acactaaaac agcgacacaa caaaagttcg gaccaaagaa caaaccccaa
     1381 cctttccata agaaagtatg ggaacaagcc tacaagaaaa aaaattcttg gattagacaa
     1441 ggtctaaagg cgggtggttc tgctcttggt tcacttttag gtggaccagt tggaagttct
     1501 ctcggaactt ctgcgggtaa tcttattgct aatcttgttg gagcgggcgc gtatcgtcaa
     1561 ggggaaatta aaatgaacac ttcatttaat aaattacccg agacgggagt tttgcatgca
     1621 ggcgtgccta caatgcataa agtcggtagc gatatacgag tatcacaccg tgaatacgta
     1681 tgcgaagtga tttcatctgc gacagcaaat acatttaaag tcgataaata cccaatcaat
     1741 ccagcaatgc gtaattcatt cccttggcta tcagcagttg cacaacaata cgaacagtac
     1801 aaagttcacg gtatggtttt tgaattccgt tcgaattcag gaaatgcgct aaatagcaca
     1861 aataccgctc ttggttctgt cgtaatgggt gcatcttata gaccaagcac tacaacacct
     1921 actaacaaac aaatgattat gaattttgaa tgggctaatg aaactgtccc atcaaaggat
     1981 atgattatgg gtattgaatg cgacccatca tcactagcat acaagcaata cttcactcgc
     2041 ggtagtaatg cactacccgc cgatgacagt gtcaatcagt atgatattgc taatctgttt
     2101 atctctacca gcggttgtca ggggacatct gtagttcttg gtagtctatt tgtatcatac
     2161 gatatcgaac tacaacattc aattgatagt gcagatatcg gacaatggtt gcatcaagca
     2221 aaatacaata tcccatcaca aaccatttca agttcaaacg ctggtttatc accatgttta
     2281 ggtgctttaa tggtttccga tggtatcggg ttatctgtat ctggagatac tataacattc
     2341 ccaattggta gcgttggtaa atattttatc aaccattccc tacgtggatc aagtgcaaca
     2401 ctgggttcgt ggtcatttgt tttaacaaat tgcactgctt tgaactcctt tattggtagt
     2461 tcaatcggtg caagtatgac acctaacgca ggtgttgcca ctggtgatga aaatttggag
     2521 tatacattaa caattagcga ccctgcaaaa attgcaacat ttaaatttac caataccgct
     2581 ctaccatcaa attggacatc tggagaattc tttgtaattc aacttgccaa ttcagttttc
     2641 taaatcattt ataaaaccaa taaattatct ttagttgttc tattgaaaca actaaataga
     2701 ctaccaagca ataaaaacac tttaataatt ggatgaacaa agacacggag ggaagacgcc
     2761 agacacctta gatttacaat ataaattttc attatgcatt ttatcacgct ttagcgtgat
     2821 aatgaaaatt tatattgtaa atatacggtt tccagtcttc ccgtatcttt tcatctatta
     2881 aaacaatcac ataaccaatg aatttcataa ttacataacc aatgaattca tcacataacc
     2941 aatgatttaa ttataactct aattataatc taaactataa ctatagatgt ctaaacaagt
     3001 atacttaaat ggaatgtata cgcgtagtca attaatacat attataattg tatcaaccat
     3061 taggcgattt actaaaaaac gcccattttt caactaaaaa aaatgttcca aaggtttggt
     3121 a
//
LOCUS       CruV-159                3122 bp    DNA     circular     24-FEB-2020
DEFINITION  Crucivirus-159 isolate 001731_virusbaton.
ACCESSION   CruV-159
KEYWORDS    .
SOURCE      Crucivirus-159
  ORGANISM  Crucivirus-159
            Unclassified.
REFERENCE   1  (bases 1 to 3122)
  AUTHORS   de la Higuera,I., Kasun,G.W., Torrance,E.L., Pratt,A.A.,
            Maluenda,A., Colombet,J., Bisseux,M., Ravet,V., Dayaram,A.,
            Stainton,D., Kraberger,S., Zawar-Reza,P., Goldstien,S., Briskie,J.,
            White,R., Taylor,H., Gomez,C., Ainley,D.G., Harding,J.S.,
            Fontenele,R.S., Schreck,J., Ribeiro,S.G., Oswald,S.A., Arnold,J.M.,
            Varsani,A., Enault,F. and Stedman,K.M.
  TITLE     Direct Submission
  JOURNAL   Submitted (24-FEB-2020) Biology, Portland State University, 1719 SW
            10th Ave, Portland, Oregon 97201, USA
COMMENT     Bankit Comment: ALT EMAIL:ide@pdx.edu
            Bankit Comment: TOTAL # OF SEQS:451
            Bankit Comment: GAP: est. length - alignment

            ##Assembly-Data-START##
            Assembly Method       :: MetaSPAdes v. 3.12; IDBA-UD v. 1.1.1;
                                     MEGAHIT v. 1.0.3
            Sequencing Technology :: 454; Illumina
            ##Assembly-Data-END##
FEATURES             Location/Qualifiers
     source          1..3122
                     /organism="Crucivirus-159"
                     /mol_type="genomic DNA"
                     /isolate="001731_virusbaton"
                     /isolation_source="Water"
                     /country="France"
                     /collection_date=2013
     CDS             1..933
                     /codon_start=1
                     /product="Putative Replication Associated Protein"
                     /translation="MGGIMSIDNIRVRNWCFTLNNYTEDEVEALTTWGVKNTSYLIFG
                     KEIGEEKATPHLQGYLEFTNAHRGSALKKLLNGRLHWSARIGTALQASNYCKKGDQTK
                     VEWNESKELGSNFGKNAIIFEHGEISNQGKRTDLADVGQAVLAGETMKEIATSFPSTF
                     IKYHKGILALKQAALGVFDFEDRKTKPKVIWRWGATGVGKSRGARDAHSNFYIKDGTP
                     WWDGYLQQEAIIIDDFDGKWPFRDLLRLLDYGPYQGQIKGGYVKINSPNIYITCEFPP
                     ESAFHIAPGDMAQVLRRIDEVIHVTEDIRGESRT*"
     CDS             complement(1593..2978)
                     /codon_start=1
                     /product="Putative CruV Capsid Protein"
                     /translation="MSGRAPVRERARKKAVKTVRRPATRRVPRARPSMISERKEQYSY
                     SRPGPVGKTGGMIGTALGGYLGGPAGAALGSALGGSAGHYIGKLFGSGDYMVTSPKPL
                     VQNTIVNPGQVPLFDSAGSKDTIRIRHRECLGDVFTSATIGAFSINNYPINPGMYQTF
                     PWLSQVVGGNFQQYRINGMVFHFRSMSADALNSTNTALGSVVLATEYDSLDTPFASKI
                     EMENTDYGVSSKPSCDVMHGIECARSQTTITELYVRNEAAPSGGDLRLYDLGRFSIAT
                     TGFQAASVNIGELWVTYDISLLKPIMAVPADVALVAVYNITPSATAPMGVAGSQVALL
                     DEIGLTFTENKIQWPNNIQVNSCWYWFWQVEGDSTASVPNPGVSRGNGMESPAALAVN
                     PTPMNNAVWQPVTTGTSTRAATSQVSVYTGGGTPAALPYVNIADQVIPTNFTYAQVIV
                     CQISSSYLTAR*"
ORIGIN
        1 ttgggaggga tcatgagcat cgacaacatt cgtgtgcgta actggtgttt cacactgaac
       61 aactacactg aagatgaagt tgaagcattg acaacgtggg gtgtgaaaaa tacatcgtat
      121 ttaatcttcg gcaaagaaat tggcgaagaa aaagccacac cacacttaca aggatacctt
      181 gaatttacta acgcacacag aggaagtgcg ctaaagaaat tactcaatgg acgtttgcac
      241 tggagtgcac gtattggtac ggccttacag gcctccaact actgcaaaaa aggtgatcaa
      301 acaaaagttg aatggaacga gtccaaagaa cttggatcca actttggcaa gaatgcgatc
      361 atttttgagc atggagaaat cagcaatcag ggtaaacgca ctgatttagc agatgtggga
      421 caagctgtat tagcgggtga gacaatgaag gaaatagcga cgtcatttcc ttccacgttc
      481 atcaaatacc acaaaggtat tctagccctc aaacaagctg ctttgggagt cttcgatttc
      541 gaagatcgaa aaacaaaacc caaagtgata tggcgatggg gtgcaacagg agttgggaaa
      601 tctcggggtg cacgagatgc gcactcgaat ttctacatca aagatggtac gccatggtgg
      661 gacggatact tacaacagga agcaataatc atagatgatt ttgacggtaa atggccattt
      721 agagacctgt tgagactcct tgattatgga ccttatcaag gacaaataaa gggtggatat
      781 gtcaagataa actctccaaa catctacatt acatgtgagt ttccacccga atctgccttt
      841 catatagcgc caggagacat ggcgcaggtc ctacgtagaa tcgacgaagt gattcacgtc
      901 acagaagaca tacgaggcga atccagaact taatgctaac gttcaccacc tacggttatc
      961 ttcactacgc gttctttttt tcacatctcc ttcttcctga cacagtggtc gtgaatcacg
     1021 atcccctgtt ttacgatcta aagatcattc aaacgcacag aggcgttttg gcacagaagt
     1081 ggtggggtaa tactgccgtg tcaagacacg tctacccacc acatactgtt ttttccgcat
     1141 attgacggga cctaatgagg gtcctttcaa tatcctgcca ggagactatt gaacgtcctg
     1201 gccggacgtc ggccctgcca gggtgtgggg ccgacgtcca gccaggacgt ccaacagcct
     1261 cccccctgga tgaggtattt tataagaggg taccagaaat ggtacctgga aatatacatt
     1321 tttttgtaca atataagaac gccgagcctc cctaaaatag ggagtctaaa ttcacggctt
     1381 ctataacgtt tcaacttcat tttcaaacat agtttgctct ccccgaatgg agcgcaaaaa
     1441 atgagactaa aaaatcattt tatgttgata catttgctcg tcaaaattaa cgagcaaaaa
     1501 atgagatatg acacagttac tagacccggc aaatcaccga ggtacatttt taattatcgg
     1561 attttcgata attagaccag atgtagagat gactaacggg cagtcaagta agaacttgag
     1621 atctgacaaa caataacctg agcataagtg aaattcgttg gaattacctg atcggcaata
     1681 ttaacgtatg gaagtgcagc tggagtacct ccacctgtat aaacagatac ttgagaagtt
     1741 gcagctcttg tagaggtacc tgtagtaaca ggctgccaaa ctgcattgtt cataggagtt
     1801 ggattaactg caagtgcagc tggactttcc ataccatttc ctcgtgagac gccaggatta
     1861 gggacgctag ctgtgctatc accctctacc tgccagaacc agtaccaaca agagttgacc
     1921 tggatgttgt taggccactg gattttgttc tccgtgaacg tcaaaccaat ctcatccaga
     1981 agcgcaacct gagaacctgc aacacccatt ggcgcagtag ctgatggggt aatgttgtac
     2041 actgctacga gtgcaacatc agcaggaaca gccatgatag gcttaagtaa agaaatatca
     2101 taggttaccc acaattcacc tatattgacc gacgcggctt gaaaaccggt cgttgcaatt
     2161 gaaaatcgac caagatcata caaacgaaga tctccaccag aaggtgcagc ttcgttacgt
     2221 acataaagct ctgtgatcgt tgtctgagat ctcgcacact cgatcccatg cataacatca
     2281 caagatggtt tagaactaac tccatagtcc gtgttctcca tctctatttt tgatgcaaaa
     2341 ggagtatcaa gactatcata ctctgtagca agcaccacgg aaccaagagc cgtgtttgtg
     2401 ctattcagag catcagcaga catagaacgg aaatggaaaa ccattccgtt gatacgatac
     2461 tgttgaaagt tgcctccgac aacttgactc agccatggaa atgtctgata cataccagga
     2521 ttaataggat aattattgat gctaaacgca ccaatagtag cagatgtaaa aacatcacct
     2581 aaacattcac gatgacggat acgaatcgta tccttgcttc cagcactatc gaaaagagga
     2641 acctgaccag gattgacaat ggtattttga acgagcggct tcgggctcgt aaccatatag
     2701 tcacctgatc cgaaaagctt gccaatataa tggccagctg aaccgccaag agcgcttcca
     2761 agagcggctc cagcaggacc gccaagataa ccgccaagag cggtaccaat cattcctccc
     2821 gttttaccaa ccggtccggg tctagaatag ctatactgct ctttcctctc gctgatcatc
     2881 gaaggacgag cacgaggcac gcgccgggta gctggccttc ggacagtctt aacagccttt
     2941 ttcctcgcac gctcacgaac cggagcacgt cccgacatta attgttttgg cggaaaagtt
     3001 tcggagtata tagggataca taaacttctt caaaacaaaa ttaaatatta attaatattt
     3061 ttacatgctt aagaaaatag catgtacata tatacacgca cgaactaagc gctgcacatt
     3121 tc
//
LOCUS       CruV-160                3129 bp    DNA     circular     24-FEB-2020
DEFINITION  Crucivirus-160 isolate 001727_virusbaton.
ACCESSION   CruV-160
KEYWORDS    .
SOURCE      Crucivirus-160
  ORGANISM  Crucivirus-160
            Unclassified.
REFERENCE   1  (bases 1 to 3129)
  AUTHORS   de la Higuera,I., Kasun,G.W., Torrance,E.L., Pratt,A.A.,
            Maluenda,A., Colombet,J., Bisseux,M., Ravet,V., Dayaram,A.,
            Stainton,D., Kraberger,S., Zawar-Reza,P., Goldstien,S., Briskie,J.,
            White,R., Taylor,H., Gomez,C., Ainley,D.G., Harding,J.S.,
            Fontenele,R.S., Schreck,J., Ribeiro,S.G., Oswald,S.A., Arnold,J.M.,
            Varsani,A., Enault,F. and Stedman,K.M.
  TITLE     Direct Submission
  JOURNAL   Submitted (24-FEB-2020) Biology, Portland State University, 1719 SW
            10th Ave, Portland, Oregon 97201, USA
COMMENT     Bankit Comment: ALT EMAIL:ide@pdx.edu
            Bankit Comment: TOTAL # OF SEQS:451
            Bankit Comment: GAP: est. length - alignment

            ##Assembly-Data-START##
            Assembly Method       :: MetaSPAdes v. 3.12; IDBA-UD v. 1.1.1;
                                     MEGAHIT v. 1.0.3
            Sequencing Technology :: 454; Illumina
            ##Assembly-Data-END##
FEATURES             Location/Qualifiers
     source          1..3129
                     /organism="Crucivirus-160"
                     /mol_type="genomic DNA"
                     /isolate="001727_virusbaton"
                     /isolation_source="Water"
                     /country="France"
                     /collection_date=2013
     misc_feature    join(3123..3129,1..18)
                     /note="stem-loop"
     misc_feature    1..9
                     /note="nonanucleotide"
     CDS             complement(519..1940)
                     /codon_start=1
                     /product="Putative CruV Capsid Protein"
                     /translation="MSSRAPRSRAPRSRAPRAPRARAPAPAPVVVAPAPKKKGGLFGK
                     LGRSLMKIARPVADAGIQKLQQMAIAKIAGSGDYAISDGVHAIEHNSIINPTHSDNIP
                     TFSSSGNVFRICHREYIGDLYSSTDFVNTTYTINPANSACFPWLSQIVTGNFQEYRFN
                     GLVFEYKTGSSDALNSTNTALGYVVMSSQYNTLSQPFTTKQSAENTEFAVSTKPSRSM
                     LHAIECKGNPNQPLYVQLEGGQQLSAGDARLYNLATLNVITTGMQQANTNLGELWVSY
                     DILLYRPILQNSLGLRIGTARYRFSQGDSYSWPLGTVQDRTENFDSIGIVIRDDAGAR
                     GDAVGNDIVFPVGLAGTYQISLLVDNIPEAYGPYQPYGLNNGRPELVPVLTNATFYNP
                     FLMSTSSVGYMTQQAEGQANGVQSMAFVFCIKITDANVPCVVRFPSLSPRTMLPPTSI
                     NGTAPYVQEGNLLITQLNGRISD*"
     CDS             complement(1996..3069)
                     /codon_start=1
                     /product="Putative Replication Associated Protein"
                     /translation="MSCCVDYEFRLSETATTGTKCTVERLTDALLKIAKNFVFQLEQG
                     EGGYRHFQGSIRLYKKVLPTGALELFKSNLSSNLAECPQYLKPSVKGIHTAHKFNYDC
                     YASKAQTRLGETITDKTAREIKLIEPEYIPRQYRGKIDTLYEYQKTILIMSEDFNTRR
                     INVIIDNIGNNGKSTIAHVARLYKGAVVLPPINDSKELMQACCDILKARGTRKSVPIF
                     VDLPRGMNKKNTQAIYIALEQILSGWLYDIRHNMKEWDIDTPSIWVFTNSEPDLSCVS
                     RDRWALWRISDSKELKPYTTEQTSDDEEIILKPIIAKPIRDLNKRLEVERREAEIKRL
                     NFELQDMKSEIINPDDIYNSDTD*"
ORIGIN
        1 aactaataaa taattaaacc actaaatggg attttttacg gcgaaactaa agtttctgga
       61 ataacaccgt aaaaaatttt cttatactaa tcttcttata tattctttat aaggaagttc
      121 ggaaagttcg gacataatgc ggtgcttatc tcctttatct ctggaaatct tcggaaagtc
      181 cggacataat ttctctgcgt ttattatgtg aaatttccat gttctgaggc tgaaaatata
      241 tttattaaag cactgaatat attatttatt tttccaatgt caaataaata atgccctaaa
      301 gggcatttcc ctccggtatt ttttgacgaa aaataaaaat atatcttaac cgtgttaata
      361 aatagtattt tctatgtatt agcctaaggc tctgaggctc gtttatgctc catcttgaaa
      421 gattacgcga gaaatccaga gcccgacggc taaggctcaa actcgtttat aaattatata
      481 ttaatatcat tgaatgaatt taatatatta ttatttgatt aatcagatat ccgcccattt
      541 aattgagtaa ttagtaggtt tccttcctgc acatagggag ctgttccatt aatagaagtt
      601 ggagggagca tagtgcgagg agacaacgaa ggaaacctga caacgcaagg cacgtttgca
      661 tcagttattt taatacaaaa tacaaaagcc atactttgaa cgccgtttgc ttgtccttcc
      721 gcttgttgcg tcatatatcc aactgacgaa gtagacatta agaaaggatt ataaaaggtt
      781 gcatttgtaa gaactggcac aagttccgga cggccattat ttaaaccata tggctgatat
      841 ggtccgtatg cttcaggtat attatcaact aataaagata tttgataagt gccagccaat
      901 ccaacaggaa agactatatc atttccgacg gcgtccccac gagctccggc gtcgtcccta
      961 attacaatgc cgatactatc aaaattttca gtgcggtctt gaactgttcc gagaggccat
     1021 gaataactat caccctgtga aaatctatag cgagcagtgc ctatacgaag gccgagcgaa
     1081 ttttgcaaaa ttggcctata tagtaagata tcatatgata cccataattc cccgaggtta
     1141 gtgtttgcct gttgcattcc ggtcgtaatt acattgagcg tcgcaagatt atataagcgg
     1201 gcatcacctg ctgatagttg ttgcccgcct tctaactgta catataaagg ctgatttgga
     1261 ttgcctttgc attctatggc gtgaagcatg gagcgactgg gcttagtaga tactgcaaac
     1321 tcagtatttt ctgcactttg cttagtagtg aatggttgcg ataaagtatt atattgcgat
     1381 gacataacaa cataaccgag agcggtatta gttgaattaa gagcatcact tgaaccggtt
     1441 ttatattcaa aaacaagacc attaaagcgg tattcttgaa aatttcctgt gacaatttga
     1501 gacagccaag gaaaacaagc cgaatttgcg gggttaatgg tgtaagtagt attaacgaaa
     1561 tcggttgagc tgtataaatc gccgatatac tcgcgatgac atatgcgaaa cacattgcca
     1621 gaacttgaaa aagtagggat attatccgaa tgagtaggat taattatgct gttatgttct
     1681 atagcgtgca cgccatcaga aatcgcataa tcacctgaac cggcgatttt agcaattgcc
     1741 atttgctgaa gtttctgaat accagcatca gcaactggac gggctatttt cattaaactt
     1801 cgccctaact taccaaataa accacctttt ttctttggag caggagcaac gacaacgggg
     1861 gcaggagctg gagcacgagc ccgcggagca cgaggggcac gagaacgagg agcacgagaa
     1921 cgaggagcac gagaagacat tattttatat atattactat atagaaaata aattttatga
     1981 aattatcgca tgcgtttaat ccgtgtcgga attataaata tcatcggggt tgattatttc
     2041 tgatttcata tcttgcaact caaaattaag gcgttttatc tcggcttctc tgcgttcaac
     2101 ctctaaccgt ttatttaaat ctctaatcgg tttagcaatt ataggcttta aaattatttc
     2161 ctcgtcgtcg cttgtttgtt cggtagtata tggctttaat tctttactgt cggaaatccg
     2221 ccaaagagcc cacctatcac gagatacgca tgataagtcg ggttcggaat ttgtaaatac
     2281 ccatatactc ggcgtgtcta tgtcccactc tttcatatta tggcggatgt catacagcca
     2341 accgcttaat atttgttcta atgcaatata tattgcttgt gtgttctttt tattcatccc
     2401 tcggggtaaa tcaacgaata tcggcacgct tttacgcgtt cctcttgcct ttaatatatc
     2461 acagcatgcc tgcattaatt ctttgctatc attaataggc ggtagaacaa ccgccccttt
     2521 atataagcga gccacgtgtg caatagttga ttttccatta ttgcctatat tatcaatgat
     2581 aacatttatt cggcgggtgt tgaaatcctc ggacatgatt aaaattgtct tttgatattc
     2641 atataaagta tcaatttttc cgcgatattg acggggtata tattcaggtt caattaattt
     2701 aatctccctt gccgttttat ccgttattgt ctcacctaaa cgagtttgag cctttgatgc
     2761 gtagcagtca taattaaatt tatgagcggt atgaatacct ttaacggacg gttttaaata
     2821 ttgcggacac tccgccaaat ttgaggacaa attagattta aataattcaa gtgccccagt
     2881 aggaagcact tttttatata accgtataga cccttgaaag tgtctatatc ccccctctcc
     2941 ttgctctaat tggaatacga aattttttgc tatttttaat agtgcgtcag tcagtcgctc
     3001 caccgtgcac tttgtgcccg tcgtcgctgt ctctgataac ctaaattcat aatccacaca
     3061 gcaagacatt tacttattta tataacatag aatgagaaaa taaattttaa gtaaatgacc
     3121 catttaatg
//
LOCUS       CruV-161                3137 bp    DNA     circular     24-FEB-2020
DEFINITION  Crucivirus-161 isolate 120_1564_IR2_3297.
ACCESSION   CruV-161
KEYWORDS    .
SOURCE      Crucivirus-161
  ORGANISM  Crucivirus-161
            Unclassified.
REFERENCE   1  (bases 1 to 3137)
  AUTHORS   de la Higuera,I., Kasun,G.W., Torrance,E.L., Pratt,A.A.,
            Maluenda,A., Colombet,J., Bisseux,M., Ravet,V., Dayaram,A.,
            Stainton,D., Kraberger,S., Zawar-Reza,P., Goldstien,S., Briskie,J.,
            White,R., Taylor,H., Gomez,C., Ainley,D.G., Harding,J.S.,
            Fontenele,R.S., Schreck,J., Ribeiro,S.G., Oswald,S.A., Arnold,J.M.,
            Varsani,A., Enault,F. and Stedman,K.M.
  TITLE     Direct Submission
  JOURNAL   Submitted (24-FEB-2020) Biology, Portland State University, 1719 SW
            10th Ave, Portland, Oregon 97201, USA
COMMENT     Bankit Comment: ALT EMAIL:ide@pdx.edu
            Bankit Comment: TOTAL # OF SEQS:451
            Bankit Comment: GAP: est. length - alignment

            ##Assembly-Data-START##
            Assembly Method       :: MetaSPAdes v. 3.12; IDBA-UD v. 1.1.1;
                                     MEGAHIT v. 1.0.3
            Sequencing Technology :: 454; Illumina
            ##Assembly-Data-END##
FEATURES             Location/Qualifiers
     source          1..3137
                     /organism="Crucivirus-161"
                     /mol_type="genomic DNA"
                     /isolate="120_1564_IR2_3297"
                     /isolation_source="Water"
                     /country="Norway"
                     /collection_date=2011
     CDS             1..870
                     /codon_start=1
                     /product="Putative Replication Associated Protein"
                     /translation="MSNTPVFRFDFTYHSSKELKDVDKGGVEAWCLAHCKHWCFQLEE
                     SSTGNLHFQGRVSLLTRTRLSSIISTTSGFHWSYTSSNCTSFDYVMKEETRVDGPWAD
                     NIIIPKPTRDIDGIVLRPWQASLFGMSKVYDGRAIDVILNPKGNLGKTIFRKWMSFHG
                     HAKTIPTINNSKDLMQAVYSMGVSKCYILDFAKAMDQKAYRPLYSAIEDVKSGYVWDT
                     RYKFKELHMDPPRIFIFTNNLPPPEFMSDDRWRYWQVTPAFELTKFDISVFNGFEIPT
                     TISCGISLPGLLF*"
     CDS             1190..2569
                     /codon_start=1
                     /product="Putative CruV Capsid Protein"
                     /translation="MSVRQDAPYGFTKSGKVRTKPLQGRTKGGPGEMSVVWIKGRGAY
                     KVGRPSVTGRGGYVTDTARRATKGWGGRAGTYLGGLAGGAIGSAIAPGIGSAAGAGIG
                     AGAGGWAGRQLARLTGWGDYDITQNTLTKQGSEVPIFGENTVRVKHRECIGHLPATTA
                     FTNNSYPLNPGLSDTFPWLSGIANNYEQYHFNGLVFYFKSTSSDAIADLTNLGLGQVI
                     LATDYNSLNAPFADDLQMLGSNFANSGKPSMDIMHAVECAPAETQSKVYNVRSGDISS
                     GDIRLFDLGHFQIATVNMPANYDGMGQLWVSYDVSFLKPVMNNQLGFDINTDRFDVTT
                     GAANNIPFGTDPVEETGSNLGCSIDGPSDTISFPVTIESGYYMIMYYCVGAIAANVSF
                     NQTVVNCTVMDTYTNSGSGVQTVGMYTVIVRIDNRLATLRLIAATLPGTITAATVIIT
                     QLNGEIFLL*"
ORIGIN
        1 atgtcaaata ctccagtatt tagattcgat tttacttatc actcctccaa ggagttgaag
       61 gatgttgata agggtggtgt ggaagcttgg tgtctagcgc attgcaaaca ttggtgtttc
      121 caactcgaag aaagctccac gggtaatcta cactttcaag gaagagtgtc attactgacg
      181 cggaccagat tatctagtat aatcagtacc acttccgggt tccattggag ctatactagt
      241 tccaactgta cttctttcga ttatgtaatg aaagaagaga cccgtgttga cggcccttgg
      301 gcagataata ttatcatccc aaagcctact agggatattg atggtattgt attacgaccg
      361 tggcaagcat cgctatttgg aatgtccaaa gtatacgatg gtcgtgctat tgatgtaatt
      421 cttaacccta aagggaatct aggtaagact atatttagaa agtggatgtc attccatggt
      481 catgcaaaga ccatccctac tattaacaat tccaaggatc tgatgcaggc cgtgtactcc
      541 atgggagtat ccaaatgtta tattttggac ttcgctaagg ccatggatca gaaggcgtat
      601 cgaccgctat actctgccat cgaagacgtc aagagtgggt acgtatggga tactcgatac
      661 aagttcaaag aactacacat ggatcctcca cgtatattca ttttcacgaa taacttaccg
      721 ccgccggaat ttatgtccga cgacagatgg cgttattggc aggtaacacc tgcttttgaa
      781 ttaacgaaat ttgatatttc ggtattcaat ggcttcgaaa ttcccacgac tatctcgtgc
      841 ggaatttctc ttccaggatt attattttag atgtttctaa tatactaatc tttttttttt
      901 cggcaaaaaa ctaaagttct tttgtattaa tatcttaatt cacctgtaca ctatgtacat
      961 tttgaaatta agactcgaaa aaaaaaacat gaccaactac atagcgaaaa attctatcgc
     1021 cgaactccgt tcgtcgaata gaatttttcg cgcctcgcaa tatactgtcg taaggtgacg
     1081 cgggcgttgg tgtaaaaaac atgtagtttt ttagctatcg cgaaaaattg atttttcgtc
     1141 gatgtgtttg gttttatttt aaccaatacc catacattta tgggatgaaa tgtctgtccg
     1201 ccaagatgct ccatacggct tcaccaaatc gggcaaagtc cgtacgaagc cgctccaagg
     1261 ccgtaccaag ggtggtccgg gcgagatgtc cgtcgtctgg atcaagggcc gcggagccta
     1321 taaggtcgga cgtccctcgg tcaccggtcg cggtggttat gtcaccgaca ccgcccgtag
     1381 ggccaccaaa ggctggggtg gacgagctgg aacctatctg ggaggtttgg cgggaggtgc
     1441 tattggctct gcaatcgctc ctggtattgg ttctgcggca ggcgcaggta ttggagcagg
     1501 cgcgggcggt tgggccggac gccagctagc ccgtttgacg ggctggggtg attacgatat
     1561 cactcagaat accttgacca agcaggggtc ggaggtgccg atttttggag aaaatacggt
     1621 ccgagtcaag catcgagagt gtatcggtca cctgccggct acgactgctt ttacaaacaa
     1681 ttcgtacccg ttgaatccag gtttgagtga cacgttcccg tggttgtcgg gcattgccaa
     1741 caactacgag cagtaccatt ttaatggact ggtgttctac ttcaaatcga catcctcaga
     1801 tgcgattgca gatctcacca acctgggtct gggtcaagtg atccttgcca cagactacaa
     1861 cagtctgaat gcgccgtttg ccgacgacct tcagatgttg ggctcgaact ttgccaactc
     1921 gggtaaaccg agtatggaca tcatgcatgc cgtcgagtgt gccccggccg aaacgcagtc
     1981 caaggtgtac aacgttcggt ctggtgatat ctccagtggg gatatccgcc tatttgacct
     2041 cggacatttc cagattgcaa ctgtgaacat gccggcaaat tacgatggta tgggacaact
     2101 gtgggtgtcc tatgatgtct cgttcctgaa gccagtgatg aacaatcagc taggcttcga
     2161 catcaatacc gatcggtttg acgtgacaac gggtgctgcg aacaacattc cctttggaac
     2221 agaccctgtg gaagagacgg gttccaacct aggttgctcg atcgatggac cgagtgatac
     2281 gatctctttc cctgtcacta ttgagtctgg ttactacatg atcatgtact actgcgttgg
     2341 agcaattgct gcaaacgtca gcttcaacca gacggtggtc aattgtactg tcatggacac
     2401 gtataccaac tctggaagcg gagtccagac tgttggtatg tacactgtta tcgttaggat
     2461 cgataaccga cttgccacac tccgactgat cgcagcgaca ttgccaggga caataacagc
     2521 tgctacagtc atcatcaccc agttgaatgg tgagatcttc ttgttgtaag aagggagttt
     2581 ttttgggttt tttttcccta cggggttttg gtggttttta aaaagctttc gcttttaccc
     2641 ggtccaatta tgacgaggta cattgtaagc gtcagccgtt gcattcaaag catgcacacg
     2701 attgcgttcc accattggat cggaagacgg cccattgatt ttcatcatat gaaactgcgg
     2761 attcggaaga ccggttaata ccggtcccag accgatggat ctgggatcat caatgatacc
     2821 atcgacatga ccaccatggg agatggtgtc gatggcggcc tgaagtttgg atacgtcttg
     2881 tacgtatatg tacgcgccgg tagagcgttc gcgtaccagg aaaccgattt tatcgttttc
     2941 cattttatct gattgtcaga taattttatt ttaggcaaaa cgacttccat gaagtcaatt
     3001 tttccacggt ataacttaaa gatgtttgta cccaaaattt ttaaagttgc ccgtacgagc
     3061 ttcgcatctg agtcgaactt atttttagct atcgcagaaa aattgaattt tcatatgtac
     3121 actatgtaca tttgaaa
//
LOCUS       CruV-162                3139 bp    DNA     circular     24-FEB-2020
DEFINITION  Crucivirus-162 isolate 120_534_IR1_3296.
ACCESSION   CruV-162
KEYWORDS    .
SOURCE      Crucivirus-162
  ORGANISM  Crucivirus-162
            Unclassified.
REFERENCE   1  (bases 1 to 3139)
  AUTHORS   de la Higuera,I., Kasun,G.W., Torrance,E.L., Pratt,A.A.,
            Maluenda,A., Colombet,J., Bisseux,M., Ravet,V., Dayaram,A.,
            Stainton,D., Kraberger,S., Zawar-Reza,P., Goldstien,S., Briskie,J.,
            White,R., Taylor,H., Gomez,C., Ainley,D.G., Harding,J.S.,
            Fontenele,R.S., Schreck,J., Ribeiro,S.G., Oswald,S.A., Arnold,J.M.,
            Varsani,A., Enault,F. and Stedman,K.M.
  TITLE     Direct Submission
  JOURNAL   Submitted (24-FEB-2020) Biology, Portland State University, 1719 SW
            10th Ave, Portland, Oregon 97201, USA
COMMENT     Bankit Comment: ALT EMAIL:ide@pdx.edu
            Bankit Comment: TOTAL # OF SEQS:451
            Bankit Comment: GAP: est. length - alignment

            ##Assembly-Data-START##
            Assembly Method       :: MetaSPAdes v. 3.12; IDBA-UD v. 1.1.1;
                                     MEGAHIT v. 1.0.3
            Sequencing Technology :: 454; Illumina
            ##Assembly-Data-END##
FEATURES             Location/Qualifiers
     source          1..3139
                     /organism="Crucivirus-162"
                     /mol_type="genomic DNA"
                     /isolate="120_534_IR1_3296"
                     /isolation_source="Water"
                     /country="Norway"
                     /collection_date=2011
     CDS             1..1059
                     /codon_start=1
                     /product="Putative Replication Associated Protein"
                     /translation="MMNNTRKNADAKKDANTKTIKSKFALTAKSGAVEQCGKGNTKPY
                     RAHINAEKSKKREVQSKVWCWTLNNFEKEKVEQLEQCFLTVFCEKWIYGFEVSESGTP
                     HLQGYFVLYDKRRMSEIKKIDEFCYKMHLENRKGTEELAIRYCVKDGNYRHSNNLPLH
                     LLKPKIVRKMEILHPADYWSWQKDVEEIIKSEIVDKRSIYWIFEENGNTGKSELTKSI
                     CFHYGCLLLNKAKNDDIMHAAYNYEGELNNVIIDIPRIVGNRINYESVESLKNGVIIN
                     NKYETGQKLIASPTIIVFSNEMPVLSGLSLDRWKVFKIVDKQLFMVNIDKSIDEMSVD
                     SLDGICRNHIQTPSRFWT*"
     misc_feature    432..461
                     /note="stem-loop"
     misc_feature    447..455
                     /note="nonanucleotide"
     CDS             complement(1393..2685)
                     /codon_start=1
                     /product="Putative CruV Capsid Protein"
                     /translation="MSYNRKKTVSMPSRGKKQVKNSRVKQQTPFGDSGQIVGKRLGSF
                     FGYPKTGAMLGRAASSLVGRALFGSGDYNDNFSKVEVNSIINPQTTPKVFSMEQNAAD
                     SVCIRRTEYIQDIISSPTANTFATTTFNINPGQAATFPYLSNIARSFEEYRIKGMVYH
                     FKSLSGDNSSGAQTGLGYVCIATQYDIGDATFTNKQALQNYAYAQSGKPTHDMIHGIE
                     CKDEINVLDRRFVRAGSVPSGSDPRFYDMGKTTIATSCPGTSVTLGELWVSYDIEFLK
                     PKLPETSSTSGSQISRSNISSASITFGSIGLQSVGSIIATVNSSTEQTWVGLLPDQVY
                     QYIFLIDTSFVFSVAPVITIAQGVPVNVLTNSGSTIDTVSTIQSVANSDIFIYVRYLK
                     PSTAGTISFVLTTPGTVVSAAPYNTDSLLLPIDLAIAS*"
     misc_feature    1920..1941
                     /note="stem-loop"
     misc_feature    complement(1925..1933)
                     /note="nonanucleotide"
ORIGIN
        1 ttgatgaata atacaagaaa aaacgctgac gctaaaaaag acgctaacac taagaccata
       61 aaatctaagt ttgctctaac tgctaaaagt ggagcagtgg agcaatgcgg taagggtaat
      121 actaaacctt accgcgcgca tattaatgca gaaaaatcca aaaaaaggga ggtgcaaagc
      181 aaagtatggt gttggacttt gaataacttt gaaaaagaaa aagtggagca attggagcaa
      241 tgttttctta ctgttttttg tgaaaaatgg atttacggct tcgaggtgag tgaatcaggg
      301 acacctcatc ttcaaggcta tttcgtttta tatgataaaa gacgtatgag tgaaattaaa
      361 aaaattgatg aattttgtta taaaatgcat ttggaaaacc ggaagggcac agaagagtta
      421 gctattagat actgtgtcaa agatggtaat tatagacata gcaataatct accactacat
      481 ttattaaagc ctaaaattgt tagaaagatg gaaattttac atcctgcaga ctactggagc
      541 tggcagaaag atgttgaaga gattatcaaa agtgagattg ttgataaacg aagcatttac
      601 tggatttttg aagaaaacgg gaacactggg aagagtgaat tgactaaatc aatttgtttt
      661 cattatggat gtttgctttt aaacaaagcc aaaaacgatg acattatgca cgctgcgtat
      721 aattatgaag gtgaattaaa taacgtgata attgatatac caaggattgt gggaaatcgt
      781 ataaattatg aaagcgtgga atcattgaaa aatggtgtga taattaataa caagtatgag
      841 acaggacaaa agttgatagc ttcgcctaca ataattgttt tcagtaatga aatgcctgta
      901 ttatcgggtc ttagtcttga cagatggaaa gttttcaaga ttgttgataa acaactcttt
      961 atggtaaata tcgataagag tatcgatgaa atgtcagtcg attcgctaga tggtatatgt
     1021 agaaatcata tacaaacccc aagtagattt tggacgtaat gtgaaaatga tgacacgatg
     1081 gtccttgggc tttagctctt ccccgccctc accagggtga gagtgcgacg aacacgcgtc
     1141 aagctctcct cctctgcata tagacttaaa gagatatgaa tgttgtgctg ttgccaaata
     1201 acgaatgaga agagttagcg aattccagta aattacttag attttatttt aaggacaagg
     1261 ggactacctt gtgattccta tatatggtaa ggaatatatt tttaagtggt atgatgacac
     1321 agctaaaggc tgcaagcttt tttaacataa aatgattaaa atgataaaaa ggtttttata
     1381 aaaaatagaa aattaagaag caatagctaa gtcgattgga agcaggagcg aatcggtatt
     1441 ataaggagct gcagaaacta ctgtacctgg agtagtaaga acaaacgaga ttgttcccgc
     1501 agtagaaggt tttagataac gaacatatat gaaaatgtct gaattagcaa cactctgtat
     1561 agtgctaaca gtgtctatgg tcgacccaga attcgttaaa acattcaccg gaacaccttg
     1621 tgcaattgtg atgactgggg ctacgctaaa tacaaatgaa gtatcaatga ggaatatata
     1681 ttgatatact tggtctggga ggagacctac ccaggtttgt tctgtggatg aattgactgt
     1741 agcaataata gacccaacgg attgaagacc tatagaacca aaagtaatgg aagcacttga
     1801 aatgttgctt cttgaaattt gagacccaga agttgaagag gtttccggaa gttttggctt
     1861 caaaaactca atgtcatagg atacccataa ctcacctagc gtaacgctgg tgccgggaca
     1921 agacgtagca atagtagtct ttcccatatc gtaaaatctc gggtcgctgc ccgacggtac
     1981 gctacctgcg cggacgaaac gtctgtctag aacgttaatt tcatccttac actcgatgcc
     2041 gtgaatcata tcgtgggtag gtttaccact ctgtgcgtat gcataattct gtaaagcttg
     2101 tttgttagtg aatgtggcgt ctccgatgtc gtactgcgtc gcaatgcaga cgtatccgag
     2161 cccagtctga gcgccggaag aattgtcacc agaaagtgac ttaaaatgat aaaccatacc
     2221 cttaattcta tactcttcga aacttcttgc gatgtttgac aagtaaggga atgttgcggc
     2281 ttgacctggg tttatattga atgtagtggt tgcaaaggta tttgcagtag gcgatgaaat
     2341 aatatcctga atatattcag ttctacgaat gcagacggag tcagctgcat tttgttccat
     2401 actaaacacc ttaggtgttg tctgagggtt aattatagaa ttaacttcaa ctttggagaa
     2461 gttgtcatta tagtcgcctg aaccaaacaa ggcacggcca acaagggaag aagctgcgcg
     2521 gccaagcata gcccctgttt ttggatagcc aaaaaagctt cccaaacgtt tcccaacgat
     2581 ttggcccgag tctccaaaag gagtttgttg ttttactctt gagtttttca cttgtttctt
     2641 tcctcgtgag ggcatcgaga cggttttttt acgattatac gacatactat gagtttatag
     2701 agttttatta agatgtaata aaacgtttta tccaatggat aatatagcat tagaaaaaaa
     2761 taattgagaa ttaaacgaga aatgttccta aagaaaaagt gttaattttt gtcttagtgt
     2821 taatacaaat aacataggga acccggagcc ctcattaatt tattctagaa agaagtaaat
     2881 attgtattag ttaaaggaag cttgcttctt ctccctctcc tcacttggac gtgctgcgcc
     2941 cgccactgcg ctacgtcgtt ccgtcggcac tacgcttgat gaatgaaatg tggtaattta
     3001 catagtgtat atacgacagg gatggtataa cgatagggaa cccggagccc tatatattat
     3061 atatgaataa tataacttta actacgcgtt tatatgcaga aatttaattt ctaacaataa
     3121 ttatataaga ggaactttt
//
LOCUS       CruV-163                3142 bp    DNA     circular     26-FEB-2020
DEFINITION  Crucivirus-163 isolate PCD_266.
ACCESSION   CruV-163
KEYWORDS    .
SOURCE      Crucivirus-163
  ORGANISM  Crucivirus-163
            Unclassified.
REFERENCE   1  (bases 1 to 3142)
  AUTHORS   de la Higuera,I., Kasun,G.W., Torrance,E.L., Pratt,A.A.,
            Maluenda,A., Colombet,J., Bisseux,M., Ravet,V., Dayaram,A.,
            Stainton,D., Kraberger,S., Zawar-Reza,P., Goldstien,S., Briskie,J.,
            White,R., Taylor,H., Gomez,C., Ainley,D.G., Harding,J.S.,
            Fontenele,R.S., Schreck,J., Ribeiro,S.G., Oswald,S.A., Arnold,J.M.,
            Varsani,A., Enault,F. and Stedman,K.M.
  TITLE     Direct Submission
  JOURNAL   Submitted (24-FEB-2020) Biology, Portland State University, 1719 SW
            10th Ave, Portland, Oregon 97201, USA
COMMENT     Bankit Comment: ALT EMAIL:ide@pdx.edu
            Bankit Comment: TOTAL # OF SEQS:451
            Bankit Comment: GAP: est. length - alignment

            ##Assembly-Data-START##
            Assembly Method       :: MetaSPAdes v. 3.12; IDBA-UD v. 1.1.1;
                                     MEGAHIT v. 1.0.3
            Sequencing Technology :: 454; Illumina
            ##Assembly-Data-END##
FEATURES             Location/Qualifiers
     source          1..3142
                     /organism="Crucivirus-163"
                     /mol_type="genomic DNA"
                     /isolate="PCD_266"
                     /isolation_source="Soil"
                     /country="New Zealand"
                     /collection_date=2012
     misc_feature    join(3132..3142,1..16)
                     /note="stem-loop"
     misc_feature    1..9
                     /note="nonanucleotide"
     misc_feature    complement(5..13)
                     /note="nonanucleotide"
                     /modified_by="nacho"
     CDS             complement(173..1126)
                     /codon_start=1
                     /product="Putative Replication Associated Protein"
                     /translation="MSINPVTCFDFTAHGVEPDEAKKMLMLHCKKWDFQQEQGERTGA
                     IHLQGRMWLKVKTRLLTIAKKFPGFHLSITSKANAKNSYYVSKEETRIAGPWSSDDKD
                     VYVPRQVKEVKDLYPWQESIIKDASTWDTRHINIIIDKSGNIGKSTLCTYMGVYGIGR
                     KIPYSNDFRDIMRMVMDTPVSRLYLFDLPKALKKEHLNQFFAGVEEIKNGYAYDDRYN
                     FREKYFDCPNVWIFTNSVPDKNYLSADRWVLWEVGTTFQLNPYSPPFAPELSVDFSES
                     YDISGAASPSDITDDMIPPNVEASPNFADNIIDDIDWDAPL*"
     CDS             complement(1222..2787)
                     /codon_start=1
                     /product="Putative CruV Capsid Protein"
                     /translation="MPRKVSKKAGYTQAQKLAYYKAKAARSYRGRGDYYSDKAPKSYE
                     VGGQVGESVGNAFNAIPGVGGAISKLASPLTKKLGQYLGSRLGSYFGWGSYKVNMNSL
                     TVPEGNSPASMHSNGMITRICHREYFGDVISSSSAGAFKLDSYKMQPGAPDMFPWLSD
                     IAPNFQKYRIRGAIVEFKSGSGDAITGTNTALGEIICSTNYNTVDPNFTSRNQMENTQ
                     YCSSAKPSVSFVHVIECDPSLQAQENLYTSSSTTPITGLNGNDINWCNVQVASIGCQG
                     TNVNLGSLYITYDIELIQPIEFSAIRRPYTDIFSGTAYTDALPIAQLTADPANSLGGT
                     TNSSSQYSFPKWVNDGIWMLYGYWQSSGGGAYTPPVVTISGGSLATILINDGTSQLQA
                     PQTGLAAVTRFSLMSIIKVTSSPCIVTVVTGVIGGATPLNAVFIVNAMDASFLPALTA
                     IPMAPPAPTSMKICKEPLHSRDTIREEYHTALKDCEDAGESEIIERLKRRLAELENKS
                     PALAYQVDRLNHQ*"
ORIGIN
        1 aaataataat aaaaaaagct ttttgtgcca atgtgccaaa aagttaaaat ttaatatata
       61 tactattact atcagggcgt aaataaaagg gagcgtagcg accgcattat tacaggggaa
      121 ggagcgtagc gactggaccg ccacgccctg agtaaaatat agtttactta ttttataaag
      181 gggcatccca atcgatatca tctatgatat tatccgcaaa atttggactc gcttccacat
      241 tgggcggaat catgtcatca gttatatcgc ttggagatgc ggcaccggaa atatcataag
      301 attcagaaaa atctactgaa agttcaggtg caaaaggtgg agagtatggg ttgagttgga
      361 aagtagttcc aacttcccat aatacccacc gatccgcgga aagatagttt ttatctggca
      421 cactgtttgt gaagatccaa acattgggac aatcaaaata tttttcccgg aagttatacc
      481 tatcatcata ggcataacca ttcttaattt cttccacacc tgcaaagaat tgattaagat
      541 gttctttttt tagtgcctta ggcaaatcaa ataaatataa acgacttaca ggtgtatcca
      601 tgaccattct catgatatcc ctgaaatcgt tagaatacgg tattttccgc cctattccat
      661 atactcccat atatgtgcat agtgtggatt taccgatatt cccactttta tctataatta
      721 tattaatgtg tcgagtatcc caagtgctcg catccttaat tatactttct tgccaagggt
      781 aaagatcttt aacttccttg acttgtcgag gcacgtaaac gtctttatca tcagatgacc
      841 aaggcccggc aatacgcgtt tcttctttgc taacataata actgtttttc gcattagcct
      901 tactagtaat actgaggtga aaaccaggaa attttttcgc aatagttaga agacgcgttt
      961 taacttttaa ccacattcta ccctgtaagt gaatcgcacc agtgcgttcg ccttgctctt
     1021 gctgaaaatc ccattttttg caatgcagca taagcatttt tttagcttca tcgggctcta
     1081 cgccgtgcgc ggtgaagtca aaacatgtta cggggttaat actcattttt caatattgtt
     1141 ccaaagttgt gccaagggta tatatcttat ggaacatttc tttaaaataa aaaaaataat
     1201 taattaattc attttataaa tttattgatg atttaaacgg tcaacttgat aagcaagggc
     1261 agggctctta ttctcaagtt cagcgagtct acgttttaaa cgctcaataa tttcactttc
     1321 accggcatct tcacaatctt taagagcggt atgatactct tctcgaatag tatcacgcga
     1381 gtgaaggggt tctttacaaa ttttcatgct tgtaggagca ggaggagcca taggaattgc
     1441 cgtcaaagca ggaagaaaac tcgcgtccat agcattaaca ataaaaactg cgttaagagg
     1501 agtggcaccg ccaattacac cagttacaac ggtaacaata caaggacttg aagtcacttt
     1561 aattatactc attagagaaa accttgtaac agctgccaaa cctgtctgcg gtgcttgtaa
     1621 ttgacttgtt ccatcattaa ttaaaatagt agctaaagaa cctccagaaa tagtaacaac
     1681 ggggggagta tatgcaccac ctccgctcga ttgccaatat ccatataaca tccaaatacc
     1741 gtcattaacc catttaggaa atgaatactg acttgaagaa tttgttgttc cacctaacga
     1801 atttgccgga tcagcagtaa gctgagcaat aggaagagca tcagtataag cagttccaga
     1861 gaaaatatcc gtatacgggc gacgaatagc actaaattca ataggttgaa taagttcaat
     1921 atcataagtg atataaagag atccaagatt tacatttgtt ccttgacatc caatactagc
     1981 aacctgaaca ttacaccaat taatatcatt tccatttaat ccagtaattg gtgtagttga
     2041 agaggaagta taaagatttt cttgtgcctg aaggcttgga tcacattcaa ttacatgaac
     2101 aaaactcacg gaaggcttag cactggaaca atactgagta ttttccattt gattgcgtga
     2161 cgtgaagttt gggtctactg tgttgtaatt tgtcgaacaa attatctcac cgagcgctgt
     2221 atttgttcca gtaatagcat cacctgaacc acttttaaat tctacaatag cacctcgtat
     2281 cctgtatttt tgaaagtttg gagcaatatc agacaaccag ggaaacatat caggagcacc
     2341 aggttgcatt ttataactat ctaatttaaa agcaccagca gaagaagaag aaataacatc
     2401 accaaaatat tcacgatgac aaatacgggt aatcattcca ttggaatgca ttgaagcggg
     2461 cgagtttcct tcaggcacag ttagagaatt catattaact ttataacttc cccatccgaa
     2521 ataacttccg agacgacttc caagatattg acctaatttt tttgtaagag gtgacgcaag
     2581 tttcgaaatt gctccaccaa ctccaggaat ggcattaaat gcatttccaa cagattcgcc
     2641 aacttgtcca ccaacttcat aagatttagg cgctttatca ctataataat caccacgacc
     2701 gcggtagctt cgtgcagctt tagccttgta atatgctaat ttttgcgctt gagtatatcc
     2761 ggcttttttt gagactttgc gaggcatggc ttacaaaaag tttatacgag agagtatata
     2821 ttagcattta caatttttta taataattaa atattttttt ttaatatttt aaaacccccc
     2881 gcagcgtcgt tattcgccct tgaggggtgc ccacgaagaa taaaaagact ttaaaaggaa
     2941 attaatgcga agcattaatt tcgtttttaa gtccttttta tgctgggcaa acttcacaag
     3001 agagcgaagc gaccagcaag gggttttgct gttttaaaat atttctttta aataattaaa
     3061 attgttccac atgtgccaca catgttccac aattgggcca acctttagag tcccaataag
     3121 gactagaaac attttttatt tt
//
LOCUS       CruV-164                3142 bp    DNA     circular     24-FEB-2020
DEFINITION  Crucivirus-164 isolate 120_1545_IR2_3297.
ACCESSION   CruV-164
KEYWORDS    .
SOURCE      Crucivirus-164
  ORGANISM  Crucivirus-164
            Unclassified.
REFERENCE   1  (bases 1 to 3142)
  AUTHORS   de la Higuera,I., Kasun,G.W., Torrance,E.L., Pratt,A.A.,
            Maluenda,A., Colombet,J., Bisseux,M., Ravet,V., Dayaram,A.,
            Stainton,D., Kraberger,S., Zawar-Reza,P., Goldstien,S., Briskie,J.,
            White,R., Taylor,H., Gomez,C., Ainley,D.G., Harding,J.S.,
            Fontenele,R.S., Schreck,J., Ribeiro,S.G., Oswald,S.A., Arnold,J.M.,
            Varsani,A., Enault,F. and Stedman,K.M.
  TITLE     Direct Submission
  JOURNAL   Submitted (24-FEB-2020) Biology, Portland State University, 1719 SW
            10th Ave, Portland, Oregon 97201, USA
COMMENT     Bankit Comment: ALT EMAIL:ide@pdx.edu
            Bankit Comment: TOTAL # OF SEQS:451
            Bankit Comment: GAP: est. length - alignment

            ##Assembly-Data-START##
            Assembly Method       :: MetaSPAdes v. 3.12; IDBA-UD v. 1.1.1;
                                     MEGAHIT v. 1.0.3
            Sequencing Technology :: 454; Illumina
            ##Assembly-Data-END##
FEATURES             Location/Qualifiers
     source          1..3142
                     /organism="Crucivirus-164"
                     /mol_type="genomic DNA"
                     /isolate="120_1545_IR2_3297"
                     /isolation_source="Water"
                     /country="Norway"
                     /collection_date=2011
     CDS             1..1296
                     /codon_start=1
                     /product="Putative CruV Capsid Protein"
                     /translation="MSYNRKKTVSMPSRGKKQVKNSRVKQQSTPFGDSGQIVGKRLGS
                     FFGYPKTGAMLGRAASSLVGRALFGSGDYNDNFSKVEVNSIINPQSTPKVFSMEQNAA
                     DSVCIRRTEYIQDIISSPTANTFATTTFNINPGQAATFPYLSNIARSFEEYRIKGMVY
                     HFKSLAGDTAIGAQTGLGYVAIATQYDIGDATFTNKQALQNYAYAQSGKPTHDMIHGI
                     ECKDEINVLDRRFVRAGSVPSGSDPRFYDMGKTTIATSCPGTSVTLGELWVSYDIEFL
                     KPKLPETSSTSGSQISRSNISSASITFGSIGLQSVGSIIATVNSSTEQTWVGLLPDQV
                     YQYIFLIDTSFVFSVAPVITIAQGVPVNVLTNSGSTIDTVSTIQSVANSDIFIYVRYL
                     KPSTAGTISFVLTTPGTVVSAAPYNTDSLLLPIDLAIAS*"
     CDS             complement(1630..2688)
                     /codon_start=1
                     /product="Putative Replication Associated Protein"
                     /translation="MMNNTRKNANAKKDANTKTIKSKFALTAKSGAVEQCGKGNTKPY
                     RAHINAEKSKKREVQSKVWCWTLNNFEKEKVEQLEQCFLTVFCEKWIYGFEVGESGTP
                     HLQGYFVLYDKRRMSEIKKIDEFCYKMHLENRKGTEELAIRYCVKDGNYRHSNNLPLH
                     LLKPKIVRKMEILHPADYWSWQKDVEEIIKSEIVDKRSIYWIFEENGNTGKSELTKSI
                     CFHYGCLLLNKAKNDDIMHAAYNYEGELNNVIIDIPRIVGNRINYESVESLKNGVIIN
                     NKYETGQKLIASPTIIVFSNEMPVLSGLSLDRWKVFKIVDKQLFMVNIDKSIDEMSVD
                     SLDGICRNHIQTPSRFWT*"
     misc_feature    2554..2582
                     /note="stem-loop"
     misc_feature    2565..2573
                     /note="nonanucleotide"
ORIGIN
        1 atgtcgtata atcgtaaaaa aaccgtctcg atgccctcac gaggaaagaa acaagtgaaa
       61 aactcaagag taaaacaaca atcgacaccc tttggagact cgggccaaat cgttgggaaa
      121 cgtttgggaa gcttttttgg ctatccaaaa acaggcgcta tgcttggcag agccgcttcc
      181 agccttgttg gccgtgctct gtttggttca ggcgactata atgacaactt ctctaaagtt
      241 gaagttaatt caataattaa cccacaatca acacctaagg tgttttctat ggaacaaaat
      301 gcagctgatt ccgtctgtat tcgtagaact gaatatattc aggatattat ttcatcgcct
      361 actgcaaata cttttgctac cactacattc aatattaatc caggtcaagc cgcaacattc
      421 ccttacttgt caaacatcgc tagaagtttc gaagagtata gaattaaggg tatggtttat
      481 cattttaaat cccttgctgg agataccgcc atcggcgctc agactggtct gggttatgta
      541 gccattgcaa cgcaatatga cattggtgac gccacattca ctaacaaaca agctttacag
      601 aattatgcat acgcacagag tggaaaacct acccacgata tgattcacgg catcgagtgc
      661 aaggatgaaa ttaacgttct agacagacgt ttcgtccgcg caggtagcgt cccgtcgggc
      721 agcgacccga gattttacga tatgggaaag actactattg ctacgtcttg tcccggcacc
      781 agcgttacgc taggtgagtt atgggtatcc tatgacattg agtttttgaa gccaaagctt
      841 ccggaaacct cttcaacttc tgggtctcaa atttcaagaa gcaacatttc aagtgcttcc
      901 attacgtttg gttctatagg tcttcaatcc gttgggtcta ttattgctac agtcaattca
      961 tccacagaac aaacctgggt aggtctcctc ccagaccagg tatatcaata tatattcctt
     1021 attgatactt catttgtatt tagcgtagcc ccagtcatca caattgcaca aggtgttccg
     1081 gtgaatgttt taacgaattc tgggtcgacc atagacactg ttagcactat acagagtgtt
     1141 gctaattcag acattttcat atatgttcgt tatctaaaac cttctactgc gggaacaatc
     1201 tcgtttgttc ttactactcc aggtacagta gtttctgcag ctccttataa taccgattcg
     1261 ctcctgcttc caatcgactt agctattgct tcttaatttt ttatttttta taaaaacctt
     1321 tttatcattt taatcatttt atgttaaaaa agcttgcagc ctttagctgt gtcatcatat
     1381 cacttaaaaa tatattcctt accatatata ggaatcacaa ggtagtcccc ttgtccttaa
     1441 aataaaatct aagtaattta ctggaattcg ctaactcttc tcattcgtta tttggcaaca
     1501 gcacaacatt catatctctt taagtctata tgcagaggag gagagcttga cgcgtgttcg
     1561 tcgcactctc accctggtga gggcggggaa gagctaaagc cccaggacca tcgtgtcatc
     1621 attttcacat tacgtccaaa atctacttgg ggtttgtata tgatttctac atataccatc
     1681 tagcgaatcg actgacattt catcgatact cttatcgata tttaccataa agagttgttt
     1741 atcaacaatc ttgaaaactt tccatctgtc aagactaaga cccgataata caggcatttc
     1801 attactgaaa acaattattg taggcgaagc tatcaacttt tgtcctgtct catacttgtt
     1861 attaattatc acaccatttt tcaatgattc cacgctttca taatttatac gatttcccac
     1921 aatccttggt atatcaatta tcacgttatt taattctcct tcataattat acgcagcgtg
     1981 cataatgtca tcgtttttgg ctttgtttaa aagcaaacat ccataatgaa aacaaattga
     2041 tttagtcaat tcactcttcc cagtgttccc gttttcttca aaaatccagt aaatgcttcg
     2101 tttatcaaca atctcacttt tgataatctc ttcaacatct ttctgccagc tccagtagtc
     2161 tgcaggatgt aaaatttcca tctttctaac aattttaggc tttaataaat gtagtggtag
     2221 attattgcta tgtctataat taccatcttt gacacagtat ctaatagcta actcttctgt
     2281 gcccttccgg ttttccaaat gcattttata acaaaattca tcaatttttt taatttcact
     2341 catacgtctt ttatcatata aaacgaaata gccttgaaga tgaggtgtcc ctgattcacc
     2401 cacctcgaag ccgtaaatcc atttttcaca aaaaacagta agaaaacatt gctccaattg
     2461 ctccactttt tctttttcaa agttattcaa agtccaacac catactttgc tttgcacctc
     2521 cctttttttg gatttttctg cattaatatg cgcgcggtaa ggtttagtat tacccttacc
     2581 gcattgctcc actgctccac ttttagcagt tagagcaaac ttagatttta tggtcttagt
     2641 gttagcgtct tttttagcgt tagcgttttt tcttgtatta ttcatcaaaa aagtttctct
     2701 tatataatta ttgttagaaa ttaaatttct gcatataaac gcgtagttaa agttatatta
     2761 ttcatatata atatataggg ccccgggttc cctatcgtta taccatccct gtcgtatata
     2821 cactatgtaa attaccacat ttcattcatc aagcgtagtg ccgacggaac gacgtagcgc
     2881 agtggcgggc gcagcacgtc caagtgagga gagggaggag aagcaagctt cctttaacta
     2941 atacaatatt tacttctctc tagaataaat taatgagggc tccgggttcc ctatgttatt
     3001 tgtattaaca ctaagacaaa aattaacact ttttctttag gaacatttct cgtttaattc
     3061 tcaattattt ttttctaatg ctatattatc cattggataa aacgttttat tacattctaa
     3121 taaaactcta taaactcata gt
//
LOCUS       CruV-165                3142 bp    DNA     circular     24-FEB-2020
DEFINITION  Crucivirus-165 isolate 120_1546_IR2_3297.
ACCESSION   CruV-165
KEYWORDS    .
SOURCE      Crucivirus-165
  ORGANISM  Crucivirus-165
            Unclassified.
REFERENCE   1  (bases 1 to 3142)
  AUTHORS   de la Higuera,I., Kasun,G.W., Torrance,E.L., Pratt,A.A.,
            Maluenda,A., Colombet,J., Bisseux,M., Ravet,V., Dayaram,A.,
            Stainton,D., Kraberger,S., Zawar-Reza,P., Goldstien,S., Briskie,J.,
            White,R., Taylor,H., Gomez,C., Ainley,D.G., Harding,J.S.,
            Fontenele,R.S., Schreck,J., Ribeiro,S.G., Oswald,S.A., Arnold,J.M.,
            Varsani,A., Enault,F. and Stedman,K.M.
  TITLE     Direct Submission
  JOURNAL   Submitted (24-FEB-2020) Biology, Portland State University, 1719 SW
            10th Ave, Portland, Oregon 97201, USA
COMMENT     Bankit Comment: ALT EMAIL:ide@pdx.edu
            Bankit Comment: TOTAL # OF SEQS:451
            Bankit Comment: GAP: est. length - alignment

            ##Assembly-Data-START##
            Assembly Method       :: MetaSPAdes v. 3.12; IDBA-UD v. 1.1.1;
                                     MEGAHIT v. 1.0.3
            Sequencing Technology :: 454; Illumina
            ##Assembly-Data-END##
FEATURES             Location/Qualifiers
     source          1..3142
                     /organism="Crucivirus-165"
                     /mol_type="genomic DNA"
                     /isolate="120_1546_IR2_3297"
                     /isolation_source="Water"
                     /country="Norway"
                     /collection_date=2011
     misc_feature    join(3133..3142,1..24)
                     /note="stem-loop"
     misc_feature    1..9
                     /note="nonanucleotide"
     CDS             complement(311..1729)
                     /codon_start=1
                     /product="Putative CruV Capsid Protein"
                     /translation="MKKTHKNNSLVMIKYNNLFIIYRMPRKRKSTARRASSKKRVSFK
                     ANTSKQGAYNYKPKSWSGQGSYVRPERGPMERFGRNLGTKAGAWLGNVFGTGDYVMDQ
                     AGVPVAASGVLAPDPPRMYSDNSGTYITHREYLGDIISSSSANTFKVQKFGLNPSDMN
                     TFPWLSQVAQPNFQQYKFEQLIFEYRTFSADSLSSTNTALGSISACINYDYNDIDVLS
                     RYAVENTDWANACKPSQSMLIPVECDPKQTGLNQGLLYVINGNNVPAGTDAKTYYLGK
                     LFICTTGCQGTSVNLGSLYVSYRVKLLKPIMSPPSSSSLIFTSSRTNAVGTGSLWGAA
                     EASSVVNADSLGVSISGDVLTIDQSRLIVGQRYILIHTLTGSSTASVIYGTVTMSAGI
                     TGYTYFTKTAPYDSNSFGSPAAAATSTSLSMIIVFAVVNNNANQTITLSGSPAYPSTN
                     LGTIMIAQICGVPLENIGEYTP*"
     CDS             complement(1836..2759)
                     /codon_start=1
                     /product="Putative Replication Associated Protein"
                     /translation="MTAIKNTKKDYGSICTTWDFRANQEYFSPEDLLKFMKLNCKKYV
                     FQLEKGKETGYLHWQGRFSLMAKTIKSSVVKSLGREPNYLEPTSKENHKDLFFYVMKE
                     DTRVGETYMDPYHKKALGIDEFYMPRQYRDKTLYPYQQTIRDSAEHFDDRMIDLIVDP
                     TGGVGKSTIAAICEILDHAIDLPIVNDHKDLIHSMCDTCMDRQTRNPKLVFLDMPRAL
                     DKRSLNGFYTAIEQIKKGKLYDLRYHTKTYWIDSPRIWVFTNEEPDENLLSRDRWRIW
                     RVNKQTKILEKYDSQTTASFSYRVNKTKAKA*"
ORIGIN
        1 tattgctagt gatgtatttc tccagacaac caattggaaa aacatggcaa aatttgccga
       61 tgttttaaat gtgttttgta ataataaaat aaatcttatt atataatttg atgtgtcgtc
      121 agccggggat ttcgacccct tcggggctgc gcgtgctccc tccggggcat gctcgtcaag
      181 tctcaacccc tctagcctct accgtccctt cgggcaaacc ccttcggggc gggccctgtc
      241 gggcgaaccc tcgatgcggg ttctgtctat atattataag atatttataa gtattttata
      301 ataatttatt ttatggtgta tattctccaa tattttctaa tggaacaccg catatctgcg
      361 caatcattat ggtacccaaa ttagttgatg gatatgcagg tgaaccagat aaagtaattg
      421 tttgatttgc attgttatta actactgcaa atacaataat catacttaat gatgtagaag
      481 tagctgcggc agctgggctg ccaaaagaat ttgaatcata tggtgcagtt ttggtgaaat
      541 atgtatagcc ggttatacct gcagacatag tcactgtgcc gtagataacg gatgctgtac
      601 ttgagcctgt aagtgtatgt attaatatgt acctttgtcc aacaatcaat ctagattgat
      661 ctattgtgag tacatcgcca gagattgaaa ctccgagtga atcggcgttt acaacgctgc
      721 tcgcctcggc tgcgccccaa aggcttccgg tacccactgc attggtacga gatgaagtaa
      781 atatcaaaga acttgatgaa ggaggggaca taatcggttt taaaagtttg acacgatatg
      841 aaacgtaaag tgatcctagg tttacggatg taccttgaca tcccgtagta catatgaata
      901 actttcctaa atagtatgtt ttagcgtctg taccagccgg gacattgttt ccgttaataa
      961 cgtaaagaag tccttggtta agaccagttt gcttgggatc gcattccact ggtattaaca
     1021 tcgattggga cggcttgcaa gcatttgccc aatcggtgtt ctcaacagcg taacgcgaaa
     1081 gtacgtcaat atcgttgtaa tcgtaattaa tacaggctga tatagagccc aatgctgtat
     1141 ttgttgaact caatgagtca gccgaaaagg ttctgtattc gaagattaat tgctcgaatt
     1201 tgtattgttg aaaatttggt tgcgcaacct ggcttaacca aggaaaagta ttcatgtccg
     1261 aaggatttag tccaaatttt tgtactttga atgtatttgc ggatgatgaa ctaatgatat
     1321 cacctaagta ttcacgatgt gtaatgtatg ttcctgagtt gtcactatac atgcgtggag
     1381 gatctggtgc caatactccg gatgcagcaa ctggtacacc agcctgatcc ataacataat
     1441 caccagttcc gaagacatta ccgagccatg ctccggcttt tgttccaaga tttctgccaa
     1501 atctttccat tggtccacgt tcaggtctca catatgatcc ctgacctgac caagattttg
     1561 gtttgtaatt gtatgcgcct tgcttagatg tatttgcttt aaaagaaact cttttctttg
     1621 aactagctct acgagctgta gatttacgct ttcgtggcat tctatatatg atgaaaagat
     1681 tattatattt tatcataact aaagaattgt ttttgtgcgt ttttttcata cactcgtaaa
     1741 aaattgtgtt gtttttaaat taggcttaaa gaaattattt ctttaagcct aaccggctag
     1801 tatcgccttt taaaaaccaa acataacaat ttagcctacg ccttcgcttt agtcttattt
     1861 actcggtatg aaaaagacgc tgtagtttga gaatcatact tttctaaaat ttttgtttgt
     1921 ttgttgacac gccatattcg ccaacgatcc cttgaaagca agttttcatc gggctcttca
     1981 tttgtgaata cccatattct tgggctatcg atccagtatg ttttagtatg gtagcgtaaa
     2041 tcataaagct tgcctttttt gatctgctct atcgcagtat agaaaccgtt caatgaacgt
     2101 ttatctaaag ctctgggcat atccaaaaaa acaagtttag gatttcgtgt ttgtcgatcc
     2161 atgcaagtgt cacacattga atgtataaga tctttgtggt cgtttacaat aggcaaatca
     2221 attgcgtgat ccagaatctc acatattgcc gcgattgttg acttacctac tccccctgtg
     2281 gggtcaacga taagatcaat cattctgtcg tcaaagtgct cagctgaatc acgaatggtt
     2341 tgctgataag gataaagggt tttgtccctg tattgtctgg gcatgtaaaa ttcatcaatt
     2401 cctaatgctt ttttatgata tggatccata tacgtctcgc ccactcgagt atcttccttc
     2461 ataacataaa aaaacaagtc cttgtgattt tcctttgaag ttggttccaa ataatttggt
     2521 tctctaccta atgattttac cacagaactc ttaatagttt tggccattag agaaaagcga
     2581 ccttgccagt gtaagtaccc tgtctccttg cctttctcta attgaaaaac atatttttta
     2641 caatttaatt tcatgaattt gagtaaatct tccggtgaaa aatattcttg gttggcccta
     2701 aagtcccaag tcgtacatat tgaaccgtaa tctttcttgg tattcttgat tgctgtcatt
     2761 cctgcactct ttcgttcgtc atttccatca ataaattcat cccagtttat ttttcctgtc
     2821 aagtaatttt ccatctatat attatatatc cggaaaatta cttggaaaaa taatcgcgcg
     2881 tttttttaat taatgcagtc gttaaaatca aataaaatga aaaataaaac gaatcaaaac
     2941 aagtatgttg tgatgctttt gatgtttaat ttgatgtgac ttgaaagacc gaatgaatca
     3001 aaataatcgc actctggcat cttaggcgtt tcgcttcgct cactggaatg accctaagat
     3061 gcctgaagta tattttaaca tttaattata ataatagtat actatatata tatatttaac
     3121 caattggaaa actggaaaaa tg
//
LOCUS       CruV-166                3144 bp    DNA     circular     24-FEB-2020
DEFINITION  Crucivirus-166 isolate 120_1568_IR2_3297.
ACCESSION   CruV-166
KEYWORDS    .
SOURCE      Crucivirus-166
  ORGANISM  Crucivirus-166
            Unclassified.
REFERENCE   1  (bases 1 to 3144)
  AUTHORS   de la Higuera,I., Kasun,G.W., Torrance,E.L., Pratt,A.A.,
            Maluenda,A., Colombet,J., Bisseux,M., Ravet,V., Dayaram,A.,
            Stainton,D., Kraberger,S., Zawar-Reza,P., Goldstien,S., Briskie,J.,
            White,R., Taylor,H., Gomez,C., Ainley,D.G., Harding,J.S.,
            Fontenele,R.S., Schreck,J., Ribeiro,S.G., Oswald,S.A., Arnold,J.M.,
            Varsani,A., Enault,F. and Stedman,K.M.
  TITLE     Direct Submission
  JOURNAL   Submitted (24-FEB-2020) Biology, Portland State University, 1719 SW
            10th Ave, Portland, Oregon 97201, USA
COMMENT     Bankit Comment: ALT EMAIL:ide@pdx.edu
            Bankit Comment: TOTAL # OF SEQS:451
            Bankit Comment: GAP: est. length - alignment

            ##Assembly-Data-START##
            Assembly Method       :: MetaSPAdes v. 3.12; IDBA-UD v. 1.1.1;
                                     MEGAHIT v. 1.0.3
            Sequencing Technology :: 454; Illumina
            ##Assembly-Data-END##
FEATURES             Location/Qualifiers
     source          1..3144
                     /organism="Crucivirus-166"
                     /mol_type="genomic DNA"
                     /isolate="120_1568_IR2_3297"
                     /isolation_source="Water"
                     /country="Norway"
                     /collection_date=2011
     misc_feature    join(3138..3144,1..22)
                     /note="stem-loop"
     misc_feature    1..9
                     /note="nonanucleotide"
     misc_feature    complement(2..10)
                     /note="nonanucleotide"
     misc_feature    3..11
                     /note="nonanucleotide"
     misc_feature    complement(5..13)
                     /note="nonanucleotide"
     CDS             complement(417..1388)
                     /codon_start=1
                     /product="Putative Replication Associated Protein"
                     /translation="MVRKAGNSPVKVKASQWTETRSWCFTINNYTDDDIAGAMDLQHF
                     AKYVVLAFEIGDQGTPHIQGYVYFIHEKKRDVVIRNWLRRAAMFKAIATNGRAAAYCQ
                     IQEGKTTDIWEYGDEPTPGMLSKDRLTEVMAKPLENFGLYNQYRRAYREYMSKQFKDH
                     DRTVIITYPENRFKYMVEASQVCTDMSCYDGEKILVFESIDRVKEIELVNWLRGFPMY
                     TRRGYEVIKIDPEVILFLAVSYKDASDYTSSLDKTHVILDFKVKDDEVNAEEAIMSYA
                     KKKSIVGLEDGTPKLLRKKKYSERNREGSPYDDIRFGEYSNESDLSD*"
     misc_feature    1295..1322
                     /note="stem-loop"
     misc_feature    complement(1306..1314)
                     /note="nonanucleotide"
     CDS             complement(1858..3060)
                     /codon_start=1
                     /product="Putative CruV Capsid Protein"
                     /translation="MSYGRKLRKRWNKPLDYPGAGANLGSYIGSYIGGPSGSALGRVV
                     GGGAHRLMKTITGFGDYKVTKNAILHPAETPYFKPSTNGNLIVAYREYIGDVVSGGID
                     TFFATTYTVNPGIALTFPWVSDIADNFDEWVPHGIVFQFKSNSADALNSTNTALGTVV
                     MASQYNPYSPRFVNKQEMEQSLFSSSCRPSVNMLHPIECDPSQTRPVFNTRKGDVPPT
                     ADSRLYDICNFTIATDGMQAANVSIGELWVTYMIEFRKPVQGDASLAFDHYQIVGATT
                     AAYFGASPVKTPNSNLGIALGATTIQFPNTAYYNVLITYNLLGDAGTWIGPVFTGTAG
                     ATSLNILEADTNNDIQSSFLAAQTRIQGFTYFKITGGGLITLSGATLPTNVTAGDVVI
                     TVISTIQD*"
ORIGIN
        1 tataataata atctgaacct gcaattcgaa taggatcctc attttttgca aaatatgtcc
       61 tcttatttat acgccatcta tacggtgctg caccatatgc agcaactgca ccacgattaa
      121 gtccaaccct tggatgggtt aaaggtacat aaccaatctc acgtccgccg cttggtatat
      181 atggacctac ctctcgcatt gatcgtggtt gcatattaaa atccatttta cctccaccat
      241 atccataatt catatatcgt ccgcgcatgg atgggggcgc aatataacgc ccataagata
      301 atttctcgct aacaatatca ttattaagat cgagatcatc catcacggaa tcaacaggac
      361 ctcgcataga ttcatcaacc acataactca taactaatac tatattatat tattagttaa
      421 tcactgagat cactctcatt actatattca ccaaaccgta tatcatcgta aggagaaccc
      481 tctcgattac gctcagaata tttcttcttt cgcaacaatt ttggggtacc atcttctaat
      541 cctacaatag attttttctt cgcataactc ataatagcct cctcggcatt cacctcatcg
      601 tctttcacct taaaatctaa tatcacatgt gttttatcta aagaacttgt ataatcagaa
      661 gcatccttat aagaaactgc taaaaacaaa atcacttctg gatctatttt tatcacttca
      721 tatccccttc gggtatacat tggaaaccct ctaagccaat tcacaagttc gatttctttg
      781 actctgtcaa tggattcgaa aactaatatt ttctcaccgt cataacatga catatcagta
      841 caaacttggg atgcttctac catatactta aaccgattct ctggataagt aattatgaca
      901 gttctatcat gatctttgaa ctgtttagac atatactcgc gatacgctcg tctatactga
      961 ttatacaacc caaaattctc gagcggtttg gccattacct cggtcaatct atctttcgat
     1021 aacattcctg gtgtaggttc atcaccatac tcccatatgt cagtggtctt accttcttgt
     1081 atctgacaat atgcagcggc tcttccatta gttgctatcg ccttaaacat tgctgctcgt
     1141 cgtaaccaat tacgtattac aacatcgcgc ttcttctcgt gaatgaaata tacataccct
     1201 tgtatgtgtg gtgtaccttg atcaccaatt tcgaatgcga gtacaacata ttttgcgaaa
     1261 tgttgtaaat ccattgcacc ggctatatca tcatcggtgt aattgttaat agtgaaacac
     1321 caactacgtg tctcagtcca ttgcgatgcc ttgactttga ctggagaatt gcctgctttc
     1381 ctaaccattt ctaattctat actagattaa aaattccgca gaagtgccct ggtaatatta
     1441 cgcgattgag aatcgctatt ttcctccgga aaatcagggc ttagcgaatt ttctatactt
     1501 ttatgccttc tgttttcacg tgtttttata gatagcgtat tgaataagca ttaaaagtat
     1561 attactgcgt ataccattgt cgctaaagct ccaacctacg tacgagcctc gacgataaat
     1621 gtctcagatc tatggttata aaggcttatc gatcgccctt atatatccgt ctccgtgtct
     1681 agtcatgtca taaaattatt taatttgtat gccatgacag cgacacgaac cggaatagcc
     1741 cctcgcttcg cttggggggc tattcctctc gcgtctagtc ttggccggcc tagatcagcg
     1801 gggggatgta tatctatctg ttgcatgcac aatttaagtg ccagcagaag atagaaatta
     1861 atcttgaata gtcgaaatta ccgtaattac tacgtcacca gcagttacat ttgtcggcaa
     1921 cgtggcacca gacaaagtaa tcagaccgcc accagtaatc ttaaaatatg taaacccttg
     1981 tatccgggtt tgtgcagcca aaaatgaact ttgtatatca ttatttgtat ctgcctccaa
     2041 tatattcaat gatgttgcac ctgctgtacc tgtaaacact ggtcctatcc atgttcctgc
     2101 atcacctagc aagttatacg taattaatac gttataatat gctgtattcg gaaactgaat
     2161 tgtggtcgcg ccaagtgcta tacccaaatt tgaattcggt gtttttactg gactcgctcc
     2221 gaaatatgca gccgtagttg cacctacaat ctgataatga tcgaaagcga gcgaagcatc
     2281 accctgtacc ggttttcgga actcaatcat atacgtcacc cacaattctc ctattgagac
     2341 atttgcggct tgcattccgt ctgtagcaat tgtaaaatta caaatatcat acagtcgaga
     2401 atcagcagtt ggaggtacat cacctttacg tgtattaaac actggtcgag tttgtgaagg
     2461 atcacactct attggatgca acatatttac tgatggacgg catgacgatg agaacaatga
     2521 ctgttccatc tcttgcttat ttacaaaacg tggactatac ggattatatt gggatgccat
     2581 tacaacagtt cccaatgccg tatttgtact attcaaagcg tctgcgctgt tcgatttaaa
     2641 ttgaaataca attccatggg gaacccattc atcgaaatta tcagctatat cagacaccca
     2701 agggaaagtc aaggcaattc caggattaac agtataagtt gttgcaaaaa atgtatcaat
     2761 tccaccggat acaacgtcac ctatgtattc acggtatgca acaatcaaat taccatttgt
     2821 acttggttta aaatatggtg tttcagcagg atgcaaaata gcattttttg tcaccttata
     2881 atctccaaaa ccagtaatgg ttttcattag acgatgggca cctccaccaa caactctacc
     2941 taatgcagag cctgatggtc cgccaatata tgatccaata taggatccta aattagcacc
     3001 ggcacctgga taatctaaag gcttattcca tcgtttacgt agcttccttc cgtaagacat
     3061 ctaattctta gtgagattat tttcgcattc tatacctccg gcgctttata gatgggcgtt
     3121 tacgaaattt gcgagataca ggtt
//
LOCUS       CruV-167                3145 bp    DNA     circular     24-FEB-2020
DEFINITION  Crucivirus-167 isolate 120_1548_IR2_3297.
ACCESSION   CruV-167
KEYWORDS    .
SOURCE      Crucivirus-167
  ORGANISM  Crucivirus-167
            Unclassified.
REFERENCE   1  (bases 1 to 3145)
  AUTHORS   de la Higuera,I., Kasun,G.W., Torrance,E.L., Pratt,A.A.,
            Maluenda,A., Colombet,J., Bisseux,M., Ravet,V., Dayaram,A.,
            Stainton,D., Kraberger,S., Zawar-Reza,P., Goldstien,S., Briskie,J.,
            White,R., Taylor,H., Gomez,C., Ainley,D.G., Harding,J.S.,
            Fontenele,R.S., Schreck,J., Ribeiro,S.G., Oswald,S.A., Arnold,J.M.,
            Varsani,A., Enault,F. and Stedman,K.M.
  TITLE     Direct Submission
  JOURNAL   Submitted (24-FEB-2020) Biology, Portland State University, 1719 SW
            10th Ave, Portland, Oregon 97201, USA
COMMENT     Bankit Comment: ALT EMAIL:ide@pdx.edu
            Bankit Comment: TOTAL # OF SEQS:451
            Bankit Comment: GAP: est. length - alignment

            ##Assembly-Data-START##
            Assembly Method       :: MetaSPAdes v. 3.12; IDBA-UD v. 1.1.1;
                                     MEGAHIT v. 1.0.3
            Sequencing Technology :: 454; Illumina
            ##Assembly-Data-END##
FEATURES             Location/Qualifiers
     source          1..3145
                     /organism="Crucivirus-167"
                     /mol_type="genomic DNA"
                     /isolate="120_1548_IR2_3297"
                     /isolation_source="Water"
                     /country="Norway"
                     /collection_date=2011
     CDS             1..1371
                     /codon_start=1
                     /product="Putative Replication Associated Protein"
                     /translation="MKKRAIPPIDLVNDCVSKNDSMNGEDTVWRKEKSVVKPTEFNGF
                     GDWTEGKPKDPIVVGGPNHISSQPTSGKGRPGSKKSSRDWCYTYWGPESSIDPGLHEK
                     STVKFGDHVWLETLHDIPTNAVVYHVFQFEISPDTGGRHIQGFISFATRRRFATVRKV
                     LPRCHLAPRRGTVNQAADYCMKTDSKDGDYQFEYGTRPEDDLGQGHRSDIDAIKELID
                     EGASMKMVADHDFGYYVRNHRGLNQYRSISTKGRNKKSLVFLFVGGPGTGKSAAADSF
                     MDNYAVPPGSSGTVWYDEYEPGHHKTVIFDDFTGAWCQWRELLRLTDAYKMLVNTKGG
                     HIPFKPEAIVFTANYSPERWYSNEAVPDKAPFMRRVDYHWQYYLKHATNAEWMIPDAV
                     RIALLGFEERHPMLPVHSVCRCLKGDPTYHPHYGRYCAFDVDGETYYAVISQKKPEVV
                     LPKLWV*"
     CDS             1411..2772
                     /codon_start=1
                     /product="Putative CruV Capsid Protein"
                     /translation="MPFPTYRRAARRAPLKAYVRPRRTYAPKRRSLPSYRGKGAYTAP
                     KSAAWGQAAGSALGSIAGFIPQTKAFAAPIAALASKLGGFLGNKLGTYMGWGDYSVNM
                     NSLLVPEGNSPAQMHSDGKSIRICHREYIGDIISSSNVKEFSLQNFQVNPASSIAFPW
                     LWNIAVNFQKYKLHGAIVEFKSSSGDALTTADTSLGTVLIASNYNCADDNFQNRTQME
                     NTQYTSSAKPSVSFVHIIECDPHLQTQTSLYTAVDGIPHPGTSINEVNWVNVQVASLG
                     MQGTNVNLGSLYITYDVELIQPINQSDSTVFRGDWFALSGASGDWISNVFPNNEANPE
                     NTLGGAIVSGDTYVFPFNIQSGLFRMTYYCGPETPSGALGLPAFTGLNCTLVNTMPTP
                     NAGGNPTSSIAGQGGADCMMATFTFKITGSGAKISATAPGTFAAGISRGGLLIDQIDL
                     DYA*"
ORIGIN
        1 ttgaaaaaac gcgccattcc ccctattgac ttggtcaacg actgtgtctc aaaaaacgat
       61 tccatgaatg gtgaagatac agtatggcgt aaagaaaagt cagtcgttaa gccgactgaa
      121 tttaatggct ttggtgattg gacggaaggc aaacctaaag acccaatcgt tgttggtgga
      181 ccaaatcata tttcgtctca gccgacaagc ggaaagggta gaccgggctc aaagaaaagt
      241 tcacgagact ggtgttacac ttattgggga cctgaatcgt ccattgaccc tggacttcat
      301 gaaaaaagta cagtcaaatt cggagatcat gtgtggttgg aaactcttca cgatattccc
      361 acaaacgctg ttgtgtacca cgtttttcaa tttgagatat caccagatac tggtggaaga
      421 cacattcagg gcttcatctc ttttgcgact agacgtcgtt tcgcaacagt tcgtaaggta
      481 ttgccacggt gtcaccttgc gcctcgtaga ggaactgtta atcaagctgc agattactgc
      541 atgaaaacgg attcaaaaga tggggattat caattcgagt atggtacgag acctgaagat
      601 gatcttggac aaggtcatcg aagtgatatt gatgctatca aggagttgat agatgaaggc
      661 gcttcaatga aaatggttgc tgatcatgac tttggatatt atgttagaaa ccatcgaggt
      721 cttaaccaat atcgatctat atcaacaaaa ggtcgtaaca aaaaaagcct tgtgttttta
      781 tttgttggtg gtcctggtac tggaaagtct gctgctgctg attctttcat ggacaattat
      841 gcagtccctc ctggttcttc agggactgtt tggtacgatg agtatgagcc tggtcatcac
      901 aaaactgtaa tttttgatga tttcactggc gcttggtgtc aatggcgaga gctcttgaga
      961 cttacggatg catacaagat gcttgtcaat acaaagggtg gacatatccc tttcaaacct
     1021 gaggctattg tttttacagc taattattct cctgaaagat ggtatagtaa tgaagccgta
     1081 cctgataagg cgccttttat gaggcgtgta gactatcatt ggcaatacta tttgaagcat
     1141 gcaaccaatg ctgaatggat gatacctgat gcggtgcgta tagctctgtt gggatttgag
     1201 gaacggcatc ctatgcttcc ggtgcattct gtgtgcagat gcttaaaagg ggaccctaca
     1261 tatcatcctc actatggtag atactgtgca tttgatgtcg atggagaaac atattacgct
     1321 gtaatatctc aaaagaaacc agaggttgtt ttaccaaaac tttgggtata aacttttcaa
     1381 agtatattct tattgtcata agactaaagt atgccttttc ctacatatcg tcgtgctgct
     1441 cgtagagcac cactcaaagc ttacgttcgt cctcgtagaa cttatgctcc aaaacgtcgt
     1501 tctctcccat cttatcgtgg aaagggagca tacactgctc ctaaatctgc tgcatggggt
     1561 caagcggccg gtagtgcgct tgggtctatt gctgggttta ttcctcagac aaaggccttc
     1621 gctgcgccaa ttgctgcttt ggcatcaaag ttaggaggct ttcttggaaa taagttgggc
     1681 acttacatgg gatggggaga ctattctgtc aatatgaata gccttcttgt tccagagggc
     1741 aactctcctg ctcaaatgca ttctgatgga aaatccattc gaatttgtca tcgtgagtat
     1801 attggagata ttatttcttc ttccaatgtt aaggaatttt ctcttcaaaa ttttcaagtc
     1861 aaccccgcca gctccattgc atttccatgg ttatggaata ttgctgtaaa ctttcaaaag
     1921 tacaaacttc atggagctat tgttgagttc aagtcatctt ctggtgacgc tctcactact
     1981 gctgatacca gtcttggtac tgttctcatt gcgagcaact acaattgtgc agatgacaat
     2041 tttcaaaatc gcactcaaat ggagaacact cagtacacta gttctgccaa gccttctgtg
     2101 agttttgttc acatcattga atgtgatcct catcttcaaa ctcaaacttc attgtacact
     2161 gccgttgatg gaattcctca tccaggaact agcatcaatg aggtcaattg ggtgaatgtc
     2221 caagttgctt cccttggaat gcagggcact aatgtcaatc ttggttctct ttacatcact
     2281 tatgatgttg agttgattca acccatcaat caatctgact ctactgtgtt ccgaggagat
     2341 tggtttgctc tttctggtgc aagtggtgat tggatttcca atgttttccc caacaacgag
     2401 gcaaatcctg agaacaccct tggaggcgct attgttagtg gtgatactta tgtatttcca
     2461 tttaacattc aaagtggtct ctttagaatg acctattatt gtggtccaga aactccttct
     2521 ggtgctcttg gccttccagc ttttacaggg ctcaattgta ctcttgtcaa tacaatgcct
     2581 acacctaacg ctggtggcaa tcctacaagc tcgattgccg gacaaggagg tgctgattgt
     2641 atgatggcta cattcacctt caaaatcact ggctctggcg ccaagatttc tgctactgct
     2701 ccaggcacct ttgctgctgg aatctcacgt ggtggtcttc tcattgacca gattgaccta
     2761 gattacgcgt agtcgtgtgc cgcaggccac gcagacccat taaactctcc ttatcactat
     2821 aatactctac attcctcttt actcctttac tttacctgcc ctaacccctt tactatcgtg
     2881 tcccgctagg gcacgcagac actatcaact agcaagggat accatagggg cgggagcgaa
     2941 gcggtaccgc ccctatggta taccagtata agtaaagaaa ggtagtagcc ctaagaatcc
     3001 attgaagtag tcgtgaagag agcgttagag atcgtcggcg tgttctccct agtgggagaa
     3061 tctcgggggt ctattgttac cccccgagac ttcgggttca gggcggataa aaatcccctc
     3121 atccacaaaa cgcgcctagc gtgtc
//
LOCUS       CruV-168                3150 bp    DNA     circular     24-FEB-2020
DEFINITION  Crucivirus-168 isolate H2_12919.
ACCESSION   CruV-168
KEYWORDS    .
SOURCE      Crucivirus-168
  ORGANISM  Crucivirus-168
            Unclassified.
REFERENCE   1  (bases 1 to 3150)
  AUTHORS   de la Higuera,I., Kasun,G.W., Torrance,E.L., Pratt,A.A.,
            Maluenda,A., Colombet,J., Bisseux,M., Ravet,V., Dayaram,A.,
            Stainton,D., Kraberger,S., Zawar-Reza,P., Goldstien,S., Briskie,J.,
            White,R., Taylor,H., Gomez,C., Ainley,D.G., Harding,J.S.,
            Fontenele,R.S., Schreck,J., Ribeiro,S.G., Oswald,S.A., Arnold,J.M.,
            Varsani,A., Enault,F. and Stedman,K.M.
  TITLE     Direct Submission
  JOURNAL   Submitted (24-FEB-2020) Biology, Portland State University, 1719 SW
            10th Ave, Portland, Oregon 97201, USA
COMMENT     Bankit Comment: ALT EMAIL:ide@pdx.edu
            Bankit Comment: TOTAL # OF SEQS:451
            Bankit Comment: GAP: est. length - alignment

            ##Assembly-Data-START##
            Assembly Method       :: MetaSPAdes v. 3.12; IDBA-UD v. 1.1.1;
                                     MEGAHIT v. 1.0.3
            Sequencing Technology :: 454; Illumina
            ##Assembly-Data-END##
FEATURES             Location/Qualifiers
     source          1..3150
                     /organism="Crucivirus-168"
                     /mol_type="genomic DNA"
                     /isolate="H2_12919"
                     /isolation_source="Water"
                     /country="New Zealand"
                     /collection_date=2012
     misc_feature    join(3141..3150,1..15)
                     /note="stem-loop"
     misc_feature    1..9
                     /note="nonanucleotide"
     CDS             334..1434
                     /codon_start=1
                     /product="Putative Replication Associated Protein"
                     /translation="MKKMLNNIKLLEEPYSIPTTRIIDPITKQVINEGETETIIEPVV
                     ETDEPKEIASGSVRISTFTLYDVCEETRLKLISMPVKLLVFQTEAAPTTGNLHYQGYW
                     EFEKPYPFKALKAKFPKIKLILRAKHATALHNYNYCTKEESKVDGPWMKGNWKDIIVP
                     KPKVNSKGKRLDLNIEPFISDIKAGMKVKELSLKYPNMFMKYNKAFYEYYEMNKPIIG
                     PKINIELFDWQKTLLKIFESEPVHRQILWIWSKESETGKTIFKQYCQQTMNILDINNF
                     VFRDIVHNYNDERIIWINLPRGEVLDTTKINVLERLSDGGYAQSTKYQGSKKYISSHI
                     VVTTNDEPPYDLLPKRIKSICVDFCKPKSLKK*"
     CDS             1544..2827
                     /codon_start=1
                     /product="Putative CruV Capsid Protein"
                     /translation="MAPAKKQVRSTVHQRKVRGKGAYNVDASYKRTKKARKVAKRAPD
                     YEPSFGRSLLTEGGSALGGLFGMPGIGKSIGSAVSNVFGLGSYEIKENVFMEGRLPQM
                     MNVPAGGGTVIRFQEYLGDIITSATPGAFSINSWDINAANRESFPFLAQIAANYESYQ
                     VMGLIYEFRSTSCDALNSTNTALGSVLLATQYDVADPVFTSKAEMLNYEYSNSVKPSD
                     NCMHMVECAPNQTVLPILYTLDGSIPANTDPRLYHLGRFQCATIGFQSGSVRVGEIHC
                     TYQIRLLKPKLYSALGLDNDYVYYIAPYVNASPLGSAPTTFFNNFGVAITPTTITIPG
                     SSLVKNYRIEVCWQGITAVAIAYPSIAGSGGAQLSNGFLGCQTTAVSQNCAYLINCKT
                     SGDDIPNVITYGVAGTLPTGTNTVQIRIMELPQSI*"
ORIGIN
        1 tagtattacc taggaacgta cggtatattg tataacaata taccgatacg gtccgacccc
       61 ctacgggaac ggttttaaag gccagttaaa taaaggaaag ttcaatttga ggattttctt
      121 atacattttg aatttttgta taacctaagc caagacctgt ataaatattt acgatagtaa
      181 aatatttagt ccaggatatt tggagctagg actaaacgca ccaattcttt aagtaatttt
      241 ataataaagt accgacaggt aatttattat acaaatatac tgagattcgg gtgagtttaa
      301 ttagtataaa aaataaacaa atactatagt ataatgaaga aaatgttaaa taacatcaaa
      361 ttattagaag aaccttactc tataccaaca acgagaataa tagatccaat aactaaacaa
      421 gttattaatg aaggagaaac cgaaactatt atcgaaccag ttgtagagac agatgaacca
      481 aaagagattg ctagtggatc tgtccgaatt tcaacattta cattatatga tgtatgcgag
      541 gaaactcgtt taaagttaat ttctatgcca gtaaaattat tagtttttca gacagaggca
      601 gcgcctacta caggtaattt acattatcag ggatattggg aatttgagaa accatatcca
      661 tttaaagctt taaaagcaaa gttcccgaaa atcaagttga tccttagagc taaacatgca
      721 acagctcttc acaattataa ttattgtacg aaagaagagt ctaaggttga cggtccttgg
      781 atgaagggaa attggaaaga tattattgta ccaaaaccaa aagtcaatag caagggtaag
      841 agacttgatt taaatataga accatttatt tctgatataa aagcaggtat gaaggttaaa
      901 gaactttctt taaaataccc aaatatgttt atgaaataca acaaggcctt ttatgagtat
      961 tatgagatga acaaacctat aattggtcca aaaatcaata ttgagctatt tgattggcaa
     1021 aaaacgttat taaagatatt tgaatctgaa ccagttcata gacaaatttt atggatctgg
     1081 tcgaaagaat cagagacagg aaagactatt tttaaacaat attgtcaaca aactatgaac
     1141 atattggaca taaacaattt cgtttttcga gatatagtac ataactataa tgatgaacga
     1201 ataatatgga taaacttacc gagaggtgaa gtattagaca caacaaaaat aaatgttcta
     1261 gaaaggttat cagatggagg atatgcacag tctaccaaat atcaaggatc aaagaaatat
     1321 atttcttctc atatagttgt aacaactaat gatgaaccac cttatgactt attgccaaaa
     1381 agaattaagt caatttgtgt agatttttgc aaaccaaaaa gtttaaaaaa ataaaaacgg
     1441 aaacggaatt ttaagacact taaaaacgga aacggaattt taagacgaat taaatatata
     1501 ataaataaat ttaatttttt ttctattact atactataac aaaatggctc cagcaaaaaa
     1561 acaagttcga tcaactgtcc atcaaagaaa agttcgaggt aaaggtgcct acaatgtaga
     1621 tgcatcttac aagagaacta agaaagctag aaaagtagct aaaagagctc ctgattatga
     1681 gccatcattt ggtaggtcat tacttacaga aggaggatca gccttaggtg gattatttgg
     1741 gatgccaggt attggcaaaa gtattggatc tgctgtatcc aatgtttttg gtttaggatc
     1801 ttatgaaatt aaggaaaatg tttttatgga aggtagattg cctcaaatga tgaatgttcc
     1861 agctggtgga ggtactgtaa ttagatttca agaatatttg ggagatatta taacatcagc
     1921 gactcctggg gcttttagta ttaatagttg ggatattaac gcagccaatc gggagtcatt
     1981 ccctttttta gctcaaatag cagcaaatta tgagtcctat caagtgatgg ggctcattta
     2041 tgaatttaga agtacgagtt gtgatgcttt aaattcaacc aatacagctt taggatctgt
     2101 attacttgct acacaatatg atgtagcaga tcctgtattt acttctaaag cagaaatgtt
     2161 aaattatgaa tattctaatt cagtcaagcc atctgataac tgcatgcata tggtagaatg
     2221 tgcaccaaat caaacggtgc taccaatttt atatacatta gacggatcta ttccagctaa
     2281 tacagatcca agattatatc atttgggccg gttccaatgt gctacaattg gatttcaatc
     2341 gggttcggtg agagtcgggg aaattcattg tacatatcaa attaggctcc ttaaacctaa
     2401 actttattca gctttaggac ttgataatga ttatgtttat tatattgccc cttatgttaa
     2461 tgcatcacca ttaggttcag cacctacaac attttttaat aattttggtg ttgcaattac
     2521 accaacaaca ataacgattc ctggatcatc attagttaaa aattatcgta tagaggtatg
     2581 ttggcaagga ataacagctg ttgctattgc ttatccatct attgccggtt ctggtggtgc
     2641 acaattaagc aatggatttt taggttgtca aactacggca gtatctcaga attgcgcata
     2701 tcttattaat tgtaaaacaa gtggagatga tataccaaat gtaattactt atggtgttgc
     2761 tggtacatta ccaacaggta caaatactgt ccaaattaga attatggaat tgccacaatc
     2821 aatttaattt aatatatact ttaataaaat atatattatt acatatcctt tcctttgccc
     2881 tctgcaagag tcgaaaaaaa aaatgttttt tcgaaatagt cggccagagg ccccagttgt
     2941 cgcgtgtaaa gcgggtcttt agaccccaac aataacataa aagtttataa gaaataatgt
     3001 cttattggat agcttaagca catccacata gtcaaaataa tagatttgca ttgacaaatt
     3061 ttacatttac gatgtctaca gttcatttgt tatactatag taatacaaaa aaaattaaat
     3121 ttatattatg agggggtcgg tcctagctgt
//
LOCUS       CruV-169                3150 bp    DNA     circular     24-FEB-2020
DEFINITION  Crucivirus-169 isolate 120_979_SvL1_3295.
ACCESSION   CruV-169
KEYWORDS    .
SOURCE      Crucivirus-169
  ORGANISM  Crucivirus-169
            Unclassified.
REFERENCE   1  (bases 1 to 3150)
  AUTHORS   de la Higuera,I., Kasun,G.W., Torrance,E.L., Pratt,A.A.,
            Maluenda,A., Colombet,J., Bisseux,M., Ravet,V., Dayaram,A.,
            Stainton,D., Kraberger,S., Zawar-Reza,P., Goldstien,S., Briskie,J.,
            White,R., Taylor,H., Gomez,C., Ainley,D.G., Harding,J.S.,
            Fontenele,R.S., Schreck,J., Ribeiro,S.G., Oswald,S.A., Arnold,J.M.,
            Varsani,A., Enault,F. and Stedman,K.M.
  TITLE     Direct Submission
  JOURNAL   Submitted (24-FEB-2020) Biology, Portland State University, 1719 SW
            10th Ave, Portland, Oregon 97201, USA
COMMENT     Bankit Comment: ALT EMAIL:ide@pdx.edu
            Bankit Comment: TOTAL # OF SEQS:451
            Bankit Comment: GAP: est. length - alignment

            ##Assembly-Data-START##
            Assembly Method       :: MetaSPAdes v. 3.12; IDBA-UD v. 1.1.1;
                                     MEGAHIT v. 1.0.3
            Sequencing Technology :: 454; Illumina
            ##Assembly-Data-END##
FEATURES             Location/Qualifiers
     source          1..3150
                     /organism="Crucivirus-169"
                     /mol_type="genomic DNA"
                     /isolate="120_979_SvL1_3295"
                     /isolation_source="Water"
                     /country="Norway"
                     /collection_date=2012
     misc_feature    join(3141..3150,1..24)
                     /note="stem-loop"
     misc_feature    1..9
                     /note="nonanucleotide"
     CDS             complement(321..1670)
                     /codon_start=1
                     /product="Putative CruV Capsid Protein"
                     /translation="MPRKRKSTARRASSKKRVSFKANTSKQGAYNYKPKPWSGQGSYV
                     RPERGPMERFGRNLGTKAGAWLGNIFGTGDYVMDQAGVPVAASGVLAPDPPRMYSDTT
                     GTYITHREYLGDIISSSSANTFKVQKFGLNPSDMNTFPWLSQVAQSSFQQYKFEQLIF
                     EYRTFSADSLSSTNTALGSISACINYDYNDPDVLSRYAVENTDWSNACKPSQSMLVPV
                     ECDPKQTGLNQGLLYVINGNNVPTGADAKTYYLGKLFICTTGCQGTSVNLGSLYVSYR
                     VKLLKPIMSPPSSSSLIFTSSRTNAVGTGSLWGAAEASSVVNADSLGVSISGDVLTID
                     QSRLIVGQRYLLIHTLTGSSTASVIYGTVTMSAGITGYTYFTKAAPYDSNSYGSPAAA
                     ATSTSLSMIIVFAVVDNNANQTITLGGAPSYPASNLGTIMIAQICGLPLENVGNYVP*
                     "
     CDS             complement(1848..2843)
                     /codon_start=1
                     /product="Putative Replication Associated Protein"
                     /translation="MENYLTGKINWDEFIDGNDERRAGMTAIKNTKKDYGSICTTWDF
                     RANQEYFSPEDLLKFMKLNCKKYVFQLEKGKETGYLHWQGRFSLMAKTIKSSVVKSLG
                     REPNYLEPTSKENHKDLFFYVMKEDTRVGETYMDPYHKKALGIDEFYMPRQYRDKTLY
                     PYQQTIRDSAEHFDDRMIDLIVDPTGGVGKSTIAAICEILDHAIDLPIVNDHKDLIHS
                     MCDTCMDRQTRNPKLVFLDMPRALDKRSLNGFYTAIEQIKKGKLYDLRYHTKTYWIDS
                     PRIWVFTNEEPDENLLSRDRWRIWRVNKQTKILEKYDSQTTASFSYRVNKPKAKA*"
ORIGIN
        1 tattgctagt gatgtatttc tccagacaac caattggaaa aacatggcaa aatttgccga
       61 tgttttaaat gtgttttgta ataataaaat aaatcttatt atataattta atatgtcgtc
      121 agcgtgtcgt cagccgggga tttcgacccc ttcggggctg cgcgtgctcc ctccggggca
      181 tgctcgtcaa gtctcaaccc ctctagcctc taccgtccct tcgggcaaac cccttcgggg
      241 cgggccctgt cgggcgaacc ctcgatgcgg gttctgtcta tatattataa gatatttata
      301 agtattttat aataatttat ttatggtacg taatttccaa cattttctaa tggaaggccg
      361 catatctgcg caatcattat ggtacccaaa ttagatgctg gatatgaagg tgcaccacct
      421 aaagtaattg tttgatttgc attgttatcg accactgcaa atacaataat catgcttaac
      481 gatgtagaag tagctgcggc agctgggctg ccataagaat ttgagtcgta tggtgcggct
      541 ttggtgaaat atgtatagcc ggttatacct gcagacatag tcactgtgcc gtagatgacg
      601 gatgctgtgc ttgagcctgt aagtgtatgt attaataggt acctttgtcc aacaatcaat
      661 ctagattgat ctattgtgag tacatcgcca gagattgaaa ctccgagtga atcggcattt
      721 acaacgctgc tcgcctcggc tgcgccccaa aggcttccgg tgccaactgc attggtacga
      781 gatgaagtaa atatcaaaga acttgatgaa ggaggggaca taatcggttt taaaagtttg
      841 acacgatatg aaacgtaaag tgatcctagg tttacggatg taccttgaca tcccgtagta
      901 catatgaata actttcctaa gtagtatgtt ttagcgtctg caccagtcgg gacattgttt
      961 ccgttaataa cgtaaagaag tccttggtta agaccagttt gcttgggatc gcattccact
     1021 ggcactaaca tcgattggga cggcttgcaa gcatttgacc aatcggtatt ctcgacagcg
     1081 taacgcgaaa gtacgtcagg gtcgttgtaa tcgtaattaa tacaggccga tatagagccc
     1141 aatgctgtat ttgttgaact caatgagtca gcagaaaagg tcctatattc gaagattaat
     1201 tgctcgaatt tgtattgttg aaaacttgat tgcgcaacct ggcttaacca aggaaaagta
     1261 ttcatgtccg aaggatttag tccaaatttt tgtactttga atgtatttgc ggatgatgaa
     1321 ctaatgatat cacctaagta ttcgcgatgt gtaatgtatg ttcctgtggt gtcactatac
     1381 atacgtggag gatctggtgc caatactccg gatgcagcaa ctggtacacc agcttgatcc
     1441 ataacataat caccagttcc gaagatattt ccgagccatg ctccggcttt tgttccaaga
     1501 tttctgccaa atctttccat tggtccacgt tcaggtctca catatgatcc ctgacctgac
     1561 caaggttttg gtttgtaatt gtatgcgcct tgcttagatg tatttgcttt aaaagaaact
     1621 cttttctttg aactagctct acgagctgta gatttgcgct ttcgtggcat tctatatata
     1681 tgatgaaaag attattatat tttatcataa ctaaagaatt gtttttgtgc gtttttttca
     1741 tacactcgta aaaaattgtg ttgtttttaa attaggctta aagaaattat ttctttaagc
     1801 ctaaccggct agtatcgcct tttaaaaacc aaacataaca atttagccta cgccttcgct
     1861 ttaggcttat ttactcggta tgaaaaagac gctgtagttt gagaatcata cttttctaaa
     1921 atttttgttt gtttgttgac acgccatatt cgccatcgat cccttgaaag caagttttca
     1981 tcgggctctt catttgtgaa tacccatatt cttgggctat cgatccagta tgttttagta
     2041 tggtaacgta aatcataaag cttgcctttt ttgatctgct ctatcgcagt atagaaaccg
     2101 ttcaatgaac gtttatctaa agctctgggc atatccaaaa aaacaagttt aggatttcgt
     2161 gtttgtcgat ccatgcaagt gtcgcacatt gaatgtataa gatctttgtg gtcgtttaca
     2221 ataggcaaat caattgcgtg atccaggatc tcacatattg ccgcgattgt tgacttacct
     2281 actccccctg tggggtcaac gataagatca atcattctgt cgtcaaaatg ctcagctgaa
     2341 tcacgaatgg tttgctgata aggataaagg gttttgtccc tgtattgtct gggcatataa
     2401 aattcatcaa ttcctaatgc ttttttatga tatggatcca tatacgtctc gcccactcga
     2461 gtatcttcct tcataacata aaaaaacaag tccttgtgat tttcctttga agttggttcc
     2521 aaataatttg gttctctacc taatgatttt accaccgaac tcttaatagt tttggccatt
     2581 agagaaaagc gaccttgcca gtgtaagtac cctgtctcct tgcctttctc taattgaaaa
     2641 acatatttct tacaatttaa tttcatgaat ttgagtaaat cttccggtga aaaatattct
     2701 tggttggccc taaagtccca agtcgtacat attgaaccgt aatctttctt ggtattcttg
     2761 attgctgtca ttcctgctct tcgttcgtca tttccatcaa taaattcatc ccagtttatt
     2821 tttcctgtca agtaattttc catctatata ttatatatcc ggaaaattat tggaaaaata
     2881 atcgcgcgtt tttttaatta atgcagtcgt taaaatcaaa taaaatgaaa aataaaacga
     2941 atcaaaacaa gtatgttgtg atgcttttga tgtttaattt gatgtgactt gaaagaccga
     3001 atgaatcaaa ataatcgcac tctggcatct taggcgtttc gcttcgctca ctggaatgac
     3061 cctaagatgc ctaaagtata ttttaacatt taattataat aatagtatac tatatatata
     3121 tatttaacca attggaaaac tggaaaaatg
//
LOCUS       CruV-170                3154 bp    DNA     circular     24-FEB-2020
DEFINITION  Crucivirus-170 isolate C5_126106.
ACCESSION   CruV-170
KEYWORDS    .
SOURCE      Crucivirus-170
  ORGANISM  Crucivirus-170
            Unclassified.
REFERENCE   1  (bases 1 to 3154)
  AUTHORS   de la Higuera,I., Kasun,G.W., Torrance,E.L., Pratt,A.A.,
            Maluenda,A., Colombet,J., Bisseux,M., Ravet,V., Dayaram,A.,
            Stainton,D., Kraberger,S., Zawar-Reza,P., Goldstien,S., Briskie,J.,
            White,R., Taylor,H., Gomez,C., Ainley,D.G., Harding,J.S.,
            Fontenele,R.S., Schreck,J., Ribeiro,S.G., Oswald,S.A., Arnold,J.M.,
            Varsani,A., Enault,F. and Stedman,K.M.
  TITLE     Direct Submission
  JOURNAL   Submitted (24-FEB-2020) Biology, Portland State University, 1719 SW
            10th Ave, Portland, Oregon 97201, USA
COMMENT     Bankit Comment: ALT EMAIL:ide@pdx.edu
            Bankit Comment: TOTAL # OF SEQS:451
            Bankit Comment: GAP: est. length - alignment

            ##Assembly-Data-START##
            Assembly Method       :: MetaSPAdes v. 3.12; IDBA-UD v. 1.1.1;
                                     MEGAHIT v. 1.0.3
            Sequencing Technology :: 454; Illumina
            ##Assembly-Data-END##
FEATURES             Location/Qualifiers
     source          1..3154
                     /organism="Crucivirus-170"
                     /mol_type="genomic DNA"
                     /isolate="C5_126106"
                     /isolation_source="Soil"
                     /country="New Zealand"
                     /collection_date=2012
     CDS             join(1..165,343..885)
                     /codon_start=1
                     /product="Putative Replication Associated Protein"
                     /translation="MRVGEERHQDGGVHFHVVVVFGTKLSTKNVRLFDITPPGGAAEH
                     PNIRKFDKKQMKKADHEAWMHDSKVSGQTTPYPFTLMGEVSIREPKPDGRGKLDKRRH
                     WLVIGPPDIGKTHWINVTFKGKSVYLRPNNKYPFEANGYSQQPVVVYDDVVPQWAELS
                     DVTNVHSLEKATYGESRYRGNWWKEDQARIVLIFLNSFNVPSYALEDGQDARYDLFVA
                     RFNTLRWNQRDKAWYVF*"
     CDS             complement(1081..2214)
                     /codon_start=1
                     /product="Putative CruV Capsid Protein"
                     /translation="MIPTGNVKAASSGGVVVSHREYLGDVYSKFSDSPAVPTAFSIAE
                     YILNPGNSITFTWLAPIARSFEEYRIRKMMFQFRSTSGNSVASANTALGAVMLATQYN
                     VTNMPFASKIEMENYAHGQSGVPSQDILHQVDLKKAASPLKTLYVREDNTPVPSGQDP
                     RLYDFGKFSIATQGQQANATDLGELWCSYVIELIKPKIPTAQTDVLLGSLTDHFALDP
                     GVISATSGKYFGVTGSTTQDPATGSTLGGKIADIPGNGNYYLFPGYGLDSGLRLPIGT
                     KALFHYDMVGAGTTLTVAQITWTPGINWSAFALWRNSSQAGPDYNNINGAINIAAQGG
                     LACMVKTSDGDSLGDHVSITAGTLCTSPGSGDLVVTLIPAAIV*"
ORIGIN
        1 atgcgggtcg gagaggagcg acatcaggac ggtggtgtgc actttcacgt cgtggttgtc
       61 ttcggtacga agctttccac caagaacgtg cgtctgtttg acatcacacc acctggcggg
      121 gcggcagagc atcccaacat ccgcaagttc gataagaagc agatggttgg caggtaagcc
      181 gttagtatca tgagctggaa gtgcagcggg cgtaaacccg ctgtacgaaa ctgatttgcg
      241 atatgatgca ggctttgcta cgccagtaaa gacgggaaga tcaccgactt cggagtttgg
      301 aagccacact ctttgttcgc ggactcgaac aattatctca agaagaaggc tgaccatgaa
      361 gcttggatgc acgactccaa ggtgtctggt cagacaacgc cgtatccctt caccctcatg
      421 ggtgaggtct ctattcgcga gcctaaacct gacgggcgcg gcaagcttga caagcgccgg
      481 cattggctcg tgataggccc tcccgacatt ggtaaaaccc actggattaa cgttaccttc
      541 aagggcaagt cggtgtatct ccgcccaaac aacaagtatc cgtttgaagc caacggttac
      601 tcgcagcagc ctgtcgtcgt atacgatgac gttgtgcctc agtgggcgga gttgtccgac
      661 gtgaccaatg tgcacagcct ggagaaggct acgtacgggg aatcccgtta ccgcggcaat
      721 tggtggaagg aggatcaagc gcgcatcgtg ctcatcttcc tcaacagttt caacgtgccg
      781 agctatgctc tggaggatgg gcaggacgca cgttacgact tgttcgttgc gcgcttcaac
      841 acgttgcgct ggaaccagcg cgacaaagcg tggtacgtgt tctaaacacg tgcgttgcct
      901 atttgcgtat ttcgtaaatt gctttttatg tggaaaagcg ccaactcacg cttgaaaaac
      961 cacgaatcac gtcccagtga agctcgcaag ataaaacgcg ggcatggcca tgcacgggta
     1021 tgcaacccct gtcggtctgc atggtgaccg cttcacgctc tccagctgcg tggtaggtta
     1081 ctacactatc gctgctggaa tcagcgtgac aaccaggtct ccgctgccag ggcttgtgca
     1141 cagtgttcct gctgtgatgg agacatggtc tcccaaactg tcaccgtctg acgttttcac
     1201 catgcaggcc aagcctcctt gtgccgcaat gttaattgcg ccgttgatat tgttgtagtc
     1261 cggtcctgct tgactggagt ttcgccacaa ggcaaacgcc gaccagttaa tgcccggcgt
     1321 ccacgtgatt tgcgccactg tcaaggtggt cccggcgccg accatgtcat agtggaacag
     1381 cgccttagtg ccgatcggca agcggagtcc agaatccaga ccgtagcccg ggaatagata
     1441 ataattcccg tttcctggga tgtctgcgat tttccctccc agcgtcgatc cagtggctgg
     1501 atcctgcgtg gtacttcctg tcacgccgaa gtacttcccg cttgttgccg atatcactcc
     1561 tggatctaga gcgaagtgat ccgtcagaga accgagcagt acgtcagtct gcgcagttgg
     1621 aatcttcggc ttgatcagct cgatgacgta gctgcaccac agctcgccca agtctgtggc
     1681 gtttgcttgc tggccttgcg tcgcaatgct gaactttcca aaatcgtaga gccgcgggtc
     1741 ttgacctgat ggcactggcg tgttgtcttc gcgcacgtac agcgtcttca atggagacgc
     1801 agccttcttg aggtccactt ggtgaagaat gtcctgcgag ggtactccgc tctggccgtg
     1861 cgcatagttc tccatttcga tcttgctagc gaaaggcatg ttggtcacgt tgtattgcgt
     1921 tgccagcatg acggctccga gagcagtgtt cgctgaggcc acagagtttc cgctcgtcga
     1981 gcggaactgg aacatcattt tcctgatccg atactcttcg aagctgcggg cgattggcgc
     2041 cagccaggtg aacgtgatgg agttccctgg gttgaggatg tactcggcaa tgctgaacgc
     2101 agtgggaact gctggtgagt ctgagaactt gctgtagaca tctccgaggt actctctgtg
     2161 agagacaacg acgccgccgg aggaagctgc tttgacgttg cctgtaggaa tcaattgtaa
     2221 gcgagcccgc gagcgaacaa gacttcaccg aatgaaggga ctcccatggg tccaggtcca
     2281 tagaggctgc ctcgtcgtcc tcgaggggtt gacttgacag aggaagctcg cttgcggctt
     2341 cctttctttc tcgaggcttt tccgcgcgca acgacggttt tgtcgaaagc atcgcgcatg
     2401 gacttgaccc gcttcgctga gtagccctta gcataccggc ctttacctgt tgaagcgcgt
     2461 ttgcgctttg gcattgctta atgaaagcaa ggacagctgg attgcaaggg attttcacgt
     2521 gtggagaatc cttaggatat ttctcttctt tgctcataaa ttcctgccca acagagcagg
     2581 tttattagcg aggtcgcgcc ctcgctaagt acatgccccc cggtcatacc agaaggttta
     2641 gatgtatatc aacttgcatc ccgggtccgg ggcgggtagc cccggccagg gggtgcaggg
     2701 gggcggagcc ccccgcaaaa aatattagtt aaaccgaaaa gtagttgttc cggccctcgg
     2761 tgggggacca actcaaacct tgtctatttt gctttctagg catggctgaa cataaagcgg
     2821 atgctatgga tgttcgccct gacggaaaaa atgcaaatgc gcagccggaa gcgcttccgc
     2881 actttgcgca acgcaaacag ttctggaaag cagcatttga ggctgctggc attgagcagg
     2941 aggacgagga cccggagttt gcctgcgatg atgtgccgga gccaaaagca ttccgcttgc
     3001 aggcgaagaa cctttttctg acctattccc agtcccgtct aacgccccaa ctgatttggg
     3061 atagctttag caaaggtccg cgtcttaaga gcggagtatt acattttgta accttttctt
     3121 tgtagcccaa gacgcacgtg gaacgtgcct gcgc
//
LOCUS       CruV-171                3157 bp    DNA     circular     24-FEB-2020
DEFINITION  Crucivirus-171 isolate 001711_virusbaton.
ACCESSION   CruV-171
KEYWORDS    .
SOURCE      Crucivirus-171
  ORGANISM  Crucivirus-171
            Unclassified.
REFERENCE   1  (bases 1 to 3157)
  AUTHORS   de la Higuera,I., Kasun,G.W., Torrance,E.L., Pratt,A.A.,
            Maluenda,A., Colombet,J., Bisseux,M., Ravet,V., Dayaram,A.,
            Stainton,D., Kraberger,S., Zawar-Reza,P., Goldstien,S., Briskie,J.,
            White,R., Taylor,H., Gomez,C., Ainley,D.G., Harding,J.S.,
            Fontenele,R.S., Schreck,J., Ribeiro,S.G., Oswald,S.A., Arnold,J.M.,
            Varsani,A., Enault,F. and Stedman,K.M.
  TITLE     Direct Submission
  JOURNAL   Submitted (24-FEB-2020) Biology, Portland State University, 1719 SW
            10th Ave, Portland, Oregon 97201, USA
COMMENT     Bankit Comment: ALT EMAIL:ide@pdx.edu
            Bankit Comment: TOTAL # OF SEQS:451
            Bankit Comment: GAP: est. length - alignment

            ##Assembly-Data-START##
            Assembly Method       :: MetaSPAdes v. 3.12; IDBA-UD v. 1.1.1;
                                     MEGAHIT v. 1.0.3
            Sequencing Technology :: 454; Illumina
            ##Assembly-Data-END##
FEATURES             Location/Qualifiers
     source          1..3157
                     /organism="Crucivirus-171"
                     /mol_type="genomic DNA"
                     /isolate="001711_virusbaton"
                     /isolation_source="Water"
                     /country="France"
                     /collection_date=2013
     misc_feature    join(3144..3157,1..21)
                     /note="stem-loop"
     misc_feature    1..9
                     /note="nonanucleotide"
     CDS             complement(197..1321)
                     /codon_start=1
                     /product="Putative Replication Associated Protein"
                     /translation="MISRGIYPREYSSMAAVETKESLVTPLGMGEQREIILSETEAID
                     DKSRKKNWVFTWNNYTVEDLKTVNEQWLKLPNMRCVVYEPEIGKQGTPHLQGFFIFDT
                     LKSFKQVKEMIDKRVWFAQMRKPIDANLKYCSKEGGTIYLGDVPKTQKEKGYAGKEYV
                     SKYGHVWSHLIDDIKNGMTFKTAAEKYPDCHGMYPRGFKEKFDLFAPKPVFNLPERVE
                     KLYDWQKECIRILGGDPDDRSVYWVWSKDGNTGKSDMLKHLVSVNGFQPMQNAPTRDL
                     ACAWKGGDVVFDYSRDEQAGGQINYAVLEHIKNRLLFSPKYESQTKMSEDFKSIHVVC
                     FSNSPPDVSKLSQDRWNVYRIENIENKPWLKQTVYENALF*"
     CDS             1359..2756
                     /codon_start=1
                     /product="Putative CruV Capsid Protein"
                     /translation="MFRTKSLHSIYPDFLINMPQRRVTRARVVPRAPVKRATYTAPAA
                     KRRPKAKSSSKSSGGSALGTAIGSALGTALGGPAGGAIGGIVGNAGGRFLSNIFGHGD
                     YAVTNADQIKENNLVLGNAANVPQFGAGKVSVRIKHREFLGDVISSHTAGAFQVFDYA
                     VNPGLVGTFPWLSGVVGSSFQQYRLNGAVFEFRSMSSDALNSTNTALGSVIMAADYDS
                     KDVPFTTKQQMENTEYGVSCKPSVNMCHAIECARTQTSVSELYIRAWGVPSGADIRLY
                     DLCRFSIATVGCQGTDVNLGELWVTYDIDLLKSIEQVPLYIAPYAEYNLLGLSNSSPF
                     GAAGTQVLIEDRIGLTIAGNRCYWPYNIQVGSCWQLYWEVDGASTAIVAPPTETFGNG
                     MTNFSDEIYMPFPVTVTSTKAAVSVVAKYTGGATPNAPPYWNLANGVFPAAAYTYRAM
                     SVKQIAGGYTLPAGV*"
ORIGIN
        1 tagtattaca gaccccctag atatcatatc attagatcta gaactagcgc tttagcgcgg
       61 ttattatgga ctttagacca taaattcatg ggtcgcgaag cgctaccctg aatatcattt
      121 tgcttaccct agatatcatc gataggtgag ctttaaatgt aaaaaaaatg agatttctat
      181 gtcgaaacat ttcgctttag aaaagagcgt tttcatacac cgtttgcttc agccacggtt
      241 tattttcgat attctcaata cgataaacat tccaacgatc ttgtgatagt ttcgatacat
      301 ccggaggact gttcgaaaag cacacaacat ggattgattt gaagtcttcg gacatttttg
      361 tctgagactc gtatttaggt gagaacaaaa gacggttttt aatgtgctca agcacagcat
      421 agtttatttg tcctccagct tgctcatcac gactgtagtc aaatacaacg tcaccgcctt
      481 tccaagcaca cgcaagatct cttgtcggtg cgttctgcat tggttgaaaa ccattgacag
      541 atacaagatg tttaagcata tcggacttgc ccgtattgcc atctttggac cagacccagt
      601 aaacggatct gtcatcggga tcgcctccaa gaatcctgat acattccttt tgccagtcgt
      661 agagtttctc tacacgttct ggtagattaa agacaggttt gggagcaaag agatcaaact
      721 tttctttgaa tccgcgagga tacataccat gacaatcagg gtatttttcc gctgctgtct
      781 tgaaggtcat gccgtttttg atgtcatcga tcagatgact ccaaacatgt ccgtatttag
      841 acacgtactc tttacctgcg tatcctttct ctttctgagt tttgggaacg tcaccaaggt
      901 aaatggtacc tccttccttt gagcaatact ttagatttgc atcaatgggt ttgcgcattt
      961 gtgcgaacca tactctttta tcgatcattt ccttaacttg cttgaaggat tttagcgtat
     1021 caaagataaa gaatccttga agatgaggcg tgccttgctt gcctatttca ggttcgtaaa
     1081 caacgcatcg catgtttggc agcttgagcc attgctcatt aactgttttc aaatcttcga
     1141 ctgtatagtt gttccacgtg aagacccagt tctttttacg tgatttatca tctattgctt
     1201 ctgtttcaga caggataatc tctcgttgct cccccatccc taaaggagta actagagatt
     1261 ctttcgtctc aacggcagcc atcgatgaat attctctagg atatataccc ctagatatca
     1321 ttctttagaa taaaattaat taatgtaatt atttaattat gtttagaaca aagtcattgc
     1381 atagtatata ccctgacttt ttgatcaaca tgcctcaacg aagagtaaca cgcgctcgtg
     1441 tggtacctcg agcaccagtc aagcgtgcaa cttacaccgc acctgctgca aagaggcgtc
     1501 ccaaagctaa gtcgtcaagc aagagcagcg gaggttccgc gttgggtaca gctattggtt
     1561 cagctctggg aacagctctg ggtggacccg cagggggagc cattggaggc atcgtaggaa
     1621 acgccggagg gcgattctta tcgaatatct tcggtcatgg cgattacgca gtaaccaacg
     1681 ctgaccagat caaagagaac aatcttgttc ttggaaatgc ggctaacgta ccacagttcg
     1741 gcgcaggaaa agtatccgtg aggataaagc accgtgaatt cctcggagat gtgataagct
     1801 cacacaccgc aggagcattt caggtctttg actacgcagt gaatcccggc cttgtcggga
     1861 ctttcccttg gctgtcaggt gttgtaggct caagcttcca acagtaccga ctcaatggag
     1921 cggtatttga gtttaggagc atgtcgtcag acgctctcaa ttcaaccaac acagcattgg
     1981 gatcggtcat catggctgcg gattacgaca gtaaagatgt cccgttcaca acaaaacaac
     2041 aaatggagaa tacggagtac ggagtatcgt gtaagccttc tgtgaacatg tgtcatgcga
     2101 tcgaatgcgc gcgcactcaa acgtctgtgt cagagctcta catacgagct tggggcgtac
     2161 cttcaggagc cgacataaga ctttacgatc tctgtcgatt ctctattgca accgtaggtt
     2221 gtcaaggaac agatgtcaat ctgggagagt tgtgggttac ctacgacatc gaccttctca
     2281 agtctattga gcaagttcct ctctacatag caccctatgc agagtacaat ttacttggac
     2341 tatcaaattc atcacctttt ggagctgcag gcacacaagt cctcatagag gatcgaatcg
     2401 gactgacaat tgctggaaac cgatgctatt ggccttacaa tattcaggtc ggatcttgtt
     2461 ggcaattgta ttgggaagtt gacggcgctt caaccgctat cgttgcgcct ccaacggaga
     2521 cgttcggtaa cgggatgact aatttcagtg atgaaattta tatgccattt ccagtgacag
     2581 ttacatcaac gaaggcagct gtatcagtgg ttgcgaagta caccggaggt gcaacaccca
     2641 acgcaccacc atactggaat cttgcaaatg gagtattccc tgctgcggca tatacatacc
     2701 gagctatgtc tgtgaagcaa attgcaggag gatacacgct ccctgctgga gtctagtttg
     2761 acagtgcaaa aaaatctgca cctaactctc gacttgccga tagatagaaa atgaacataa
     2821 acacattttt tgctataaaa acaggttacc caaaaaaggg taactgaaaa tagggcaaac
     2881 aacggataac gggccgcgta gcgaagcgga gtcggccctc ataatcctta ttgactactg
     2941 ctcgccgcag gcggccgcgc ggcagcgcct caggctgtcg gcgtaagccg acgaaacaaa
     3001 taatggacac gaagaggcca aaggccgcga agcggcccgg cccgcaggcc gaaatgctag
     3061 gtgagtaaca agttgctccc cgaactgagg gagggtggaa cacccccccg aaaaatgaac
     3121 aaaaagaggg caaagcccgt atgtctaggg ggtccta
//
LOCUS       CruV-172                3168 bp    DNA     circular     24-FEB-2020
DEFINITION  Crucivirus-172 isolate 120_984_SvL1_3295.
ACCESSION   CruV-172
KEYWORDS    .
SOURCE      Crucivirus-172
  ORGANISM  Crucivirus-172
            Unclassified.
REFERENCE   1  (bases 1 to 3168)
  AUTHORS   de la Higuera,I., Kasun,G.W., Torrance,E.L., Pratt,A.A.,
            Maluenda,A., Colombet,J., Bisseux,M., Ravet,V., Dayaram,A.,
            Stainton,D., Kraberger,S., Zawar-Reza,P., Goldstien,S., Briskie,J.,
            White,R., Taylor,H., Gomez,C., Ainley,D.G., Harding,J.S.,
            Fontenele,R.S., Schreck,J., Ribeiro,S.G., Oswald,S.A., Arnold,J.M.,
            Varsani,A., Enault,F. and Stedman,K.M.
  TITLE     Direct Submission
  JOURNAL   Submitted (24-FEB-2020) Biology, Portland State University, 1719 SW
            10th Ave, Portland, Oregon 97201, USA
COMMENT     Bankit Comment: ALT EMAIL:ide@pdx.edu
            Bankit Comment: TOTAL # OF SEQS:451
            Bankit Comment: GAP: est. length - alignment

            ##Assembly-Data-START##
            Assembly Method       :: MetaSPAdes v. 3.12; IDBA-UD v. 1.1.1;
                                     MEGAHIT v. 1.0.3
            Sequencing Technology :: 454; Illumina
            ##Assembly-Data-END##
FEATURES             Location/Qualifiers
     source          1..3168
                     /organism="Crucivirus-172"
                     /mol_type="genomic DNA"
                     /isolate="120_984_SvL1_3295"
                     /isolation_source="Water"
                     /country="Norway"
                     /collection_date=2012
     CDS             1..972
                     /codon_start=1
                     /product="Putative Replication Associated Protein"
                     /translation="MPKRGSKTAQTKKRTPATQCCRWDFTLFDEISPNEVRKTLAENC
                     KKYVFQKEKGEETGKIHYQGRISLKQKKRAPEVGKLFLKSWKAFHVTITSNENKDNDF
                     YVLKEDTRVDGPFSDQNEMFVPKDVEEVETLRPWQQSLVDHLSSYDKRTIDVVFEENG
                     NVGKTTLVRWMMCFKDAEEIPFCNEFRDVMRMGYDMGPKKIYFVDMPRALPKAKMEQF
                     FAAIEKLKSGYAYDDRYGFKKRLFDRPRVCIFTNVHPDLDLLSKDMWKIWTIKDNNLI
                     PYIQPHDIESPQAPIIEGDIELYTDRSDFTDSDSESVITPALTSIRK*"
     CDS             complement(1411..3108)
                     /codon_start=1
                     /product="Putative CruV Capsid Protein"
                     /translation="MAVKVKRTAGYKYVLTKSGKRKRVYPKGVKPKRRVSKKSQARGK
                     TTVLRGRGPYEITTADIDRQIQQTLGHDDPRIRYAQVDLQEPLAPIITERGRTTFPID
                     PILTAAANKANSSYYDDTTASFDSLIPNDGGGIVKTAGRILSNVVKGADMYGRYRKSN
                     FVKYGKKYVPKGTFSTIGSMVGGPLGGYAGSKFAELAGFGPYRLKRNSLVMGRNAIIG
                     EGQSPAYMHSGNSDAFITHREYIADITSSPVANAFQNQSFAINPGMFTTFPWLSALAQ
                     QYEQYNVEGMVFEYKTMYADAIATGAANATIGYVIMGTNYNSVMPPFINKQQMDNTEY
                     STSAKPSVSFYHPIECEPSSKPTKNLFIRSGDPPANADLRLYDLGNFQIATGGIAASS
                     VVVGELWVTYRVRLIKAISTTAHGLNVLSDHFQLDNPGGFTFLGNAQSAGVSGSSIGG
                     KIVNDPGGDMYVFAANLQDGNFQFTYTVQGSTTASPLVLSLPGTLVNCTMMTVYRNDT
                     VQVVDNSGTDSIVGLQTFVVNITGPGASVQWTGSFTLPTVNVGGDLWVTQLDADIVT*
                     "
ORIGIN
        1 atgccaaaac gtggatcaaa aactgctcaa acaaaaaaaa gaacaccagc aacacaatgt
       61 tgtcgctggg actttacttt atttgacgaa atttctccaa acgaagttag aaaaacactt
      121 gctgaaaatt gcaagaaata cgtttttcaa aaagaaaaag gtgaagaaac aggaaaaatc
      181 cactatcagg gacgtatttc gctaaagcaa aaaaaacgtg ctccagaagt gggaaaactg
      241 tttctaaaat catggaaagc cttccatgta accattacat ctaacgaaaa taaagacaac
      301 gacttctatg tcttaaaaga agacacaaga gtcgatggtc cattttcaga ccagaacgaa
      361 atgttcgttc ctaaagatgt cgaagaagta gaaactcttc gcccgtggca acaaagcctc
      421 gtcgatcatc tttcatctta cgataaaaga acaatcgacg tcgtctttga agaaaacggt
      481 aacgtcggca aaacaacact cgtccgttgg atgatgtgtt tcaaggatgc cgaagaaatt
      541 ccattttgca acgaatttag agacgtcatg cgtatgggat acgacatggg tcctaaaaag
      601 atatacttcg ttgacatgcc aagagcattg cctaaggcaa aaatggaaca attttttgcc
      661 gctattgaaa aactcaaaag cggatacgcc tacgatgatc gttacggctt caaaaaaaga
      721 ctctttgaca gaccaagagt ctgcatcttc actaacgttc atccagacct ggatctgtta
      781 tccaaagata tgtggaaaat atggacaata aaggataata atcttatccc ttacatccaa
      841 ccacacgata ttgagagccc acaagctcca attatcgaag gcgacataga actttacact
      901 gatcgatccg attttacgga ctctgacagt gaatcagtca ttacgccagc ccttacttcc
      961 ataagaaagt aagcccctaa tttataaatt agggctcctc catgtacgac gtccatcgtc
     1021 ggagggcctt ttggcggtac acttcgttac cctgtaatac tacgctcgct tcgctcgctc
     1081 aaaaggccaa tcttatatat atataacttg aaattcctgg atttttggct catcggagaa
     1141 gcctccgata aaccattaaa atcgaaattt caataaaaac ggatcaggat ccgaatgagc
     1201 cataatgatc caatatgaaa taacatggat acactagcgt tctcgctggc gcgcagtcgc
     1261 ttcgctttgc gctggctcta taaagggttc cacccttata cccggttcgc cccataggcg
     1321 tcgcgccagg tgaggcttac gcctcaccct tgcgctatca ctagcacaga atggcggaat
     1381 aaactacttt tttcaaaaaa agataaaagt ttatgttaca atgtcagcat ctaattgtgt
     1441 tacccacaaa tcacctccaa catttactgt tggtaatgta aatgatcctg tccactgaac
     1501 tgaagctcca ggtccagtaa tattcacaac aaatgtttgt aaaccaacaa tagaatcagt
     1561 accactatta tctaccactt gcacagtgtc attacgataa acagtcatca tagtacaatt
     1621 taccaacgta cctggtaaag ataaaaccaa aggagaagca gtcgttgatc cttgaacagt
     1681 atatgtaaac tggaaatttc catcctgtaa attagctgcg aacacataca tatcaccacc
     1741 tggatcatta acaatcttac ctcctatcga agatccagat acacctgcag attgtgcatt
     1801 tccaaggaaa gtaaatccac cgggattatc caattggaaa tgatcagaaa gcacattcaa
     1861 accatgtgct gttgtcgaaa tcgccttaat aagacgaact ctataggtaa cccaaagctc
     1921 acctacaaca acagatgatg cagcaattcc tcctgtcgca atttggaaat tacccaaatc
     1981 ataaagacga agatcagcat tagctggagg atcacccgaa cgaataaaca aattcttcgt
     2041 tggcttcgaa gatggctcgc actctatcgg atgatagaag cttaccgatg gcttagcact
     2101 cgtagaatac tcggtattat ccatctgctg tttattaata aatggaggca taaccgaatt
     2161 ataattcgtg cccattatta cataaccaat cgtagcattc gcagctcccg tagcaatagc
     2221 atcagcgtac attgtcttgt actcgaaaac cattccttca acattatact gctcgtattg
     2281 ttgagctaaa gcagataacc aaggaaatgt cgtaaacata cctggattaa tcgcgaaact
     2341 ctgattctga aaagcattcg ctaccggaga acttgtaata tctgcgatat attcacgatg
     2401 cgtaataaat gcatcagaat tacctgaatg catataagct ggggattgac cctcacctat
     2461 aattgcattc ctacccataa ctaatgaatt ccttttaagg cgataaggac caaaacctgc
     2521 aagctctgca aacttagaac cagcataacc acctaacggt ccaccaacca tagatcctat
     2581 agtagaaaat gttcccttcg gaacatactt tttcccatac ttaacaaagt tcgatttcct
     2641 ataccgtcca tacatatctg cacctttaac aacgttagat aatattcttc cggcagtttt
     2701 cacaataccg cctccatcat tcggtataag agaatcaaaa gatgcggtcg tatcatcata
     2761 ataagaacta ttcgccttat tcgctgccgc cgtcaaaatc ggatctattg gaaaagtagt
     2821 cctgccacgc tccgtaatta tcggagcaag tggctcttgc aaatcgactt gagcataacg
     2881 aatcctagga tcatcatgtc ccaaagtctg ctgtatctgc cgatcaatat ctgcagtcgt
     2941 aatctcatac ggtccacgac ctctcaaaac agttgtttta cctctagctt gagatttctt
     3001 cgaaactcta cgcttcggtt ttactccttt cggataaaca cgcttacgct taccactctt
     3061 agttaaaaca tatttataac ccgctgtacg cttaactttt acagccatcc tataccacta
     3121 aatgtaagat atatttaata taaaatatat cttacattta gtatagaa
//
LOCUS       CruV-173                3171 bp    DNA     circular     26-FEB-2020
DEFINITION  Crucivirus-173 isolate 001700_virusbaton.
ACCESSION   CruV-173
KEYWORDS    .
SOURCE      Crucivirus-173
  ORGANISM  Crucivirus-173
            Unclassified.
REFERENCE   1  (bases 1 to 3171)
  AUTHORS   de la Higuera,I., Kasun,G.W., Torrance,E.L., Pratt,A.A.,
            Maluenda,A., Colombet,J., Bisseux,M., Ravet,V., Dayaram,A.,
            Stainton,D., Kraberger,S., Zawar-Reza,P., Goldstien,S., Briskie,J.,
            White,R., Taylor,H., Gomez,C., Ainley,D.G., Harding,J.S.,
            Fontenele,R.S., Schreck,J., Ribeiro,S.G., Oswald,S.A., Arnold,J.M.,
            Varsani,A., Enault,F. and Stedman,K.M.
  TITLE     Direct Submission
  JOURNAL   Submitted (24-FEB-2020) Biology, Portland State University, 1719 SW
            10th Ave, Portland, Oregon 97201, USA
COMMENT     Bankit Comment: ALT EMAIL:ide@pdx.edu
            Bankit Comment: TOTAL # OF SEQS:451
            Bankit Comment: GAP: est. length - alignment

            ##Assembly-Data-START##
            Assembly Method       :: MetaSPAdes v. 3.12; IDBA-UD v. 1.1.1;
                                     MEGAHIT v. 1.0.3
            Sequencing Technology :: 454; Illumina
            ##Assembly-Data-END##
FEATURES             Location/Qualifiers
     source          1..3171
                     /organism="Crucivirus-173"
                     /mol_type="genomic DNA"
                     /isolate="001700_virusbaton"
                     /isolation_source="Water"
                     /country="France"
                     /collection_date=2013
     misc_feature    join(3163..3171,1..16)
                     /note="stem-loop"
                     /modified_by="nacho"
     misc_feature    1..9
                     /note="nonanucleotide"
     CDS             184..1590
                     /codon_start=1
                     /product="Putative CruV Capsid Protein"
                     /translation="MSGFTRVSKDVWDTMSPSEQAALRAQLQKAKPAKSSKKKPVKRT
                     TRRTTTTKRYGGGGRGGVRNTQYICGSGDYFSDAAAGLLKHGLPLVIKGLTGFGDYSV
                     AQNSMIPKLGGDPPIMMSTNNGGFVMRHREYVQDIYATEGFVNNTLAINPGLLSTFPL
                     ASQIADTFEQFEIRGMVMEYKSMSSDAVLSAGASSALGTIIFATQYNALDEPFEDKRT
                     MENYEFANSTKPSQSMLHPIECKRSQTPVDLLYVRTGPVETGDLRLYDLGNFNFAVQG
                     CQNAAAGQVLGELWISFELEFFKPKLLPLGGSSTLSDHYTLTGNSLIAVGNPFYTQNG
                     LVRAPESNLGTYIQIAAGSPYDANSIIFPPQITSGKFKVDYYMSATSVSANSPNVVQG
                     TLVNMVSEPFWANGTFYQIAPQPGAAAIANFHFSQVFKITQTAPGVRAGVGYTMTGVM
                     PTGNQSLDIVITLLGESA*"
     CDS             complement(2210..3079)
                     /codon_start=1
                     /product="Putative Replication Associated Protein"
                     /translation="MTVFGEEVYAEVSNYLQSRKQLDYIISCWEVCPTTGRPHYHIYC
                     HFINSVQLSEKKCFHSHLEIVRGSVDDNIHYIKKTGKHKSEFNDDPTHHKNLLEWGTI
                     PKESGGRLTAHELMSMSTAEIVEFDPRCHKAYLGAKNMLLNPVDIDIDDWAKVVTVYY
                     IQGPSGVGKTTKAMDMVRENYPVGQRTVNIVSYTSGGFWEGVGLANVALYDEWRSSHM
                     KPSEFIRFIDYNKQNMNVKGGIARNNYTTIFITSVERLKAIYGNVGGEPRLQWERRIK
                     LIDMYADASDEEN*"
ORIGIN
        1 tagtattacc cgaggcgcga ttccgattcc cgattctttt taggtgcaag gcgttagcct
       61 taagcctaaa agttgggaaa ccgaatcgca atttctaccc tcctttgata aggagggggg
      121 gtccctggag ggggggtgga tagaaaaaag aaaaatagat ttgttttcct cgtgttaaaa
      181 ccgatgtccg gcttcacacg agtttcgaag gacgtttggg acactatgtc accaagtgag
      241 caagctgcat tgagagctca acttcagaag gctaagcctg ctaagtcgtc caagaagaag
      301 cctgtcaaga gaaccacgcg tcgtaccacc acaactaagc gttatggtgg cggcggacgt
      361 ggtggcgtcc gcaacacaca gtacatctgt gggagtggcg actacttctc tgacgcagca
      421 gcaggcctct tgaaacatgg gctccccctt gttatcaaag gtctaactgg ctttggggac
      481 tactctgtag cccaaaacag tatgatcccc aagcttggcg gggatcctcc catcatgatg
      541 agtaccaaca atggtggttt cgtcatgcgc cacagggagt atgtccagga catttacgct
      601 actgaggggt ttgttaataa cactcttgct attaacccag gactcctgag tacatttccc
      661 cttgcatcgc aaattgccga cacgttcgag caattcgaga tcagaggaat ggttatggag
      721 tacaagtcta tgtcatctga cgctgttctt agcgcaggtg catcatctgc tcttggtact
      781 attatcttcg caacgcagta caatgctttg gacgaaccct tcgaagacaa gcgtaccatg
      841 gagaactatg agttcgccaa ctctactaag cctagtcaga gtatgttgca cccaattgag
      901 tgcaagagaa gtcagacccc cgttgatctt ctctacgtaa gaactggtcc tgtagaaaca
      961 ggagaccttc gattgtatga tcttggtaat ttcaactttg ctgtacaagg ctgtcagaat
     1021 gcagcagcag ggcaagttct aggtgaatta tggatctcat tcgaacttga gttcttcaag
     1081 cccaagcttc ttccacttgg tggatcaagt acgttgtctg atcattatac tttgacaggg
     1141 aacagtctta ttgctgtcgg caatccattc tatacacaga atggacttgt tagggcacct
     1201 gagagtaatc ttggaactta tatccaaatt gctgctggtt ctccttatga tgccaactcg
     1261 atcatcttcc ccccacagat aacgagtggc aagtttaagg tagactacta tatgtctgct
     1321 acatctgtat cagccaacag tcctaatgtt gtgcaaggta cattagtcaa catggtttct
     1381 gagccatttt gggctaatgg tactttctat cagatcgctc ctcagcctgg agcagctgcc
     1441 atagctaact tccacttctc gcaagtgttt aagattacac agactgctcc aggagttcga
     1501 gcaggagttg gctataccat gactggtgtt atgcctacag gtaaccagtc attggatata
     1561 gtcattactc ttcttggtga aagtgcataa atgaataatc gatcgattgt tcttagtctg
     1621 agtccgattc cagctcaggc gtgttgtatg ctagcgtcat ccgcttgtag tatgatggtg
     1681 gtctgtatac aacaccacgt tctgcgtgca tctctatgcg caccatcgct ctgttcttct
     1741 catgtatcgc attagttact tcaaagaaca gagcgatggg agtctcctcc atagcggtgt
     1801 ccgctgtgtt gcctgtgtgt ggtacgttat agaacgacat gttcggaatc gattcctttc
     1861 aggcgacatc gcttatcctt aagcctccac ggtgggttcg tcctgcacgc atcccagccg
     1921 gtgttacacg ccaccaccta gggacactcg ttgtccttaa gcctccacgg tggacacgtc
     1981 agaacggcgt ccagccgtgt gtggtagtgt gtgtgtggtg tgtactgtaa atgcacgata
     2041 tcacctaggg gcggccactg tccttaagcc tccacggtgg atgtcgtatg tcgcatccca
     2101 gccgtgtgtg gtagtgtgtg gtagtgtgtg tatgacatgt tctcgtgtta tactgtgctg
     2161 ctaccacccg cgcgcagcgc ccagcatgtt tataatcaac cgattataac tagttctctt
     2221 cgtcagacgc atcagcatac atgtctatga gtttgatacg acgctcccac tggagccgtg
     2281 gctctccacc gacattacca tagatggcct tcagtctttc tactgaagtg atgaatatgg
     2341 tagtgtagtt gttcctagct ataccacctt taacattcat gttctgtttg ttatagtcta
     2401 tgaaccttat gaactcagac ggtttcatat gtgagcttct ccattcatcg taaagtgcga
     2461 cgttagccaa acccacgcct tcccagaagc cgccagatgt gtagctcact atgttgaccg
     2521 tacgctgtcc tacagggtag ttctctctta ccatgtccat cgctttagta gtcttgccta
     2581 ctccagatgg accttgtatg tagtacaccg ttactacttt agcccaatcg tctatgtcta
     2641 tgtcgactgg gttgagtagc atattcttag cacctaagta tgctttgtga caccgtgggt
     2701 cgaactctac gatctccgct gtgctcatac tcatgagttc atgtgctgtg agcctaccac
     2761 cactttcctt tggtatggtt ccccattcca ataggttctt gtggtgagtg gggtcatcgt
     2821 tgaactcact cttgtgcttt cccgtcttct tgatgtagtg tatgttgtca tctactgacc
     2881 ctctcactat ctctagatgg ctgtggaagc acttcttctc acttagctgc acactgttta
     2941 tgaagtggca gtatatgtga tagtgtggtc tcccagttgt tggacatact tcccaacaac
     3001 tgatgatgta gtctaactgc tttctgctct gcaggtagtt actgacctca gcatacactt
     3061 cttctccaaa cactgtcatc tggaagtgac gtgctttgct cttatatgga ttccaagagt
     3121 cttcttttgg agccatattc ttcttcgttg gtttatccat gagcctcggt a
//
LOCUS       CruV-174                3172 bp    DNA     circular     26-FEB-2020
DEFINITION  Crucivirus-174 isolate H2_33461.
ACCESSION   CruV-174
KEYWORDS    .
SOURCE      Crucivirus-174
  ORGANISM  Crucivirus-174
            Unclassified.
REFERENCE   1  (bases 1 to 3172)
  AUTHORS   de la Higuera,I., Kasun,G.W., Torrance,E.L., Pratt,A.A.,
            Maluenda,A., Colombet,J., Bisseux,M., Ravet,V., Dayaram,A.,
            Stainton,D., Kraberger,S., Zawar-Reza,P., Goldstien,S., Briskie,J.,
            White,R., Taylor,H., Gomez,C., Ainley,D.G., Harding,J.S.,
            Fontenele,R.S., Schreck,J., Ribeiro,S.G., Oswald,S.A., Arnold,J.M.,
            Varsani,A., Enault,F. and Stedman,K.M.
  TITLE     Direct Submission
  JOURNAL   Submitted (24-FEB-2020) Biology, Portland State University, 1719 SW
            10th Ave, Portland, Oregon 97201, USA
COMMENT     Bankit Comment: ALT EMAIL:ide@pdx.edu
            Bankit Comment: TOTAL # OF SEQS:451
            Bankit Comment: GAP: est. length - alignment

            ##Assembly-Data-START##
            Assembly Method       :: MetaSPAdes v. 3.12; IDBA-UD v. 1.1.1;
                                     MEGAHIT v. 1.0.3
            Sequencing Technology :: 454; Illumina
            ##Assembly-Data-END##
FEATURES             Location/Qualifiers
     source          1..3172
                     /organism="Crucivirus-174"
                     /mol_type="genomic DNA"
                     /isolate="H2_33461"
                     /isolation_source="Water"
                     /country="New Zealand"
                     /collection_date=2012
     misc_feature    join(3163..3172,1..16)
                     /note="stem-loop"
                     /modified_by="nacho"
     misc_feature    1..9
                     /note="nonanucleotide"
     CDS             26..1576
                     /codon_start=1
                     /product="Putative CruV Capsid Protein"
                     /translation="MEPWSQHVPFHINLKFLFLIQLMPIGYRSPLLTRKPSTKKKVSR
                     PHDSMQQAKKRKSTYVAKQYANPPIPYEISQPNKFWRIKKGIPPVDYWRKRYYRRRIT
                     GRGDYSYSDSRSTGANIGGYLGSKAGEFLGGAAQNIGTALLSGLGSYSVKKNVLLDGN
                     MPRIDNKSGPGGITIRFQEYIGEVRSGPVAGAFNINTFLINPANSATFPWLSQIAANF
                     EQYEIEGMLFGFRTTASDLVTNDTNNLQLGTVILATQYDVADAVFQSKAEMLNYQFSS
                     SVKPSQGCIHMIECDPRQTSVNLLYCSPGSIPAGTDPRLYHLGRFSIATQGFQGDSVG
                     CGELHVTYQVRLLKPKLWSNLGQTIPFSSFFNQFTKAASTGPILGPIATISAQSQPNG
                     CNILFRAGTIASSTDMVFQANPAIPRKYLVCIFWNSAVAGNYWTSTNWPNLISPSGCN
                     VLVSAYAPTVGVLTSAYAQLNFYVTVTGTNIPELPYVNLPPADANDLPNQDIGCTIRV
                     TVVDPDLA*"
     CDS             join(complement(2238..2784),complement(1587..2158))
                     /codon_start=1
                     /product="Putative Replication Associated Protein"
                     /translation="MAKRDASRKGWVIVTNNYDAAEEARFANNLFEWSIFGREVAPTT
                     GTPHLQGYVHMKTKVRHPAMVKLFPKSKVIAADGNCTSQRAYCTKDGAFTEYGIQPKD
                     GLETIKASWEEVLVAAKAGDFEAIPAETTIKYYSTIKKIRDDTQNKIVPAKLDWKKGS
                     PPNVWIWGEAGVGKSVKARNMAGDDVYLKLCNKWWEDYDYEDNVLIEDFGMEHSVLLH
                     HVKIWADRYGFRAEKKFGSRTLRPKRIFITSNYSPNTIWPNEVECSPIRRRFQIIHMT
                     EIEKWDETPPVMKQKPKLVRTETQKFPMLQCRNGIWEPFQDDRRFVDQMMREIVTKAI
                     VISDDDDSVEITGAKAPCMFCSVKDEGLENCAFCGRVF*"
ORIGIN
        1 aactattacc cccccccgtg gagccatgga gccatggagc caacacgtgc cttttcatat
       61 aaatttaaaa tttttatttc ttattcaatt aatgccgatt gggtaccgat caccactact
      121 caccaggaaa ccatctacca agaaaaaagt ctccaggcca cacgattcaa tgcagcaagc
      181 taaaaaacgg aaatcaactt acgtagccaa acaatatgcc aatcctccaa ttccttatga
      241 aataagtcag ccgaacaagt tttggagaat caaaaaaggg attccgcctg tcgattactg
      301 gagaaaacga tattacagac gacgaattac tgggcgaggg gactattctt attccgactc
      361 cagatcaacc ggcgctaaca taggagggta cctcggttca aaggccggtg aatttttagg
      421 cggagctgcc cagaatattg ggactgcatt actatccggt cttggttcct actcagttaa
      481 aaagaatgtt cttttggatg gaaatatgcc ccggatagac aataagtcag gaccaggtgg
      541 aatcactata cgcttccagg aatatatcgg cgaggttagg agtggcccag ttgctggggc
      601 gtttaatata aacacctttt taattaaccc cgcgaactct gctacatttc cttggttatc
      661 tcagattgct gctaacttcg aacaatacga aatcgaagga atgttattcg gcttccgaac
      721 taccgcatca gatcttgtta caaacgacac aaataactta cagcttggta ctgttatttt
      781 agctactcaa tacgatgttg ctgatgctgt atttcaatcg aaagccgaga tgttgaacta
      841 ccaattttca tcatcagtta aaccctccca gggttgtatt cacatgattg agtgtgaccc
      901 acgccaaacg tcagttaatt tactttattg ttcccccgga tctattccag ccggaaccga
      961 cccaaggtta taccatttag gacgtttctc gattgcaacg caaggatttc aaggagactc
     1021 tgttggttgt ggagaactcc acgttaccta tcaagtgcgt ttactgaaac ctaagttgtg
     1081 gtctaattta ggccaaacta ttccctttag ttcatttttc aatcaattca ctaaagcggc
     1141 atctactggc cctattctcg gtcccattgc gacaattagt gcccagtcgc agccgaatgg
     1201 ctgcaacata ttatttcgag caggaactat tgcaagctca acagacatgg tttttcaagc
     1261 aaacccagcg atccccagga aatatttggt ttgcatattt tggaattctg ctgttgctgg
     1321 aaattattgg acttcaacta attggcccaa ccttatttct cctagtggct gtaatgtgct
     1381 cgttagcgct tatgcaccaa cagtaggagt tcttacatcg gcatatgcac agctaaattt
     1441 ttatgttacg gtaacgggaa caaacattcc tgagttaccc tatgttaatc tgccaccggc
     1501 agatgcaaat gatttgccta accaggatat tggttgcaca ataagggtaa cggtagtaga
     1561 tccagattta gcataataaa attttattaa aaaactcttc cacaaaaggc acaattctct
     1621 aggccctcat ctttaacgga gcaaaacatg caaggagcct tggctcctgt aatttcgacg
     1681 gaatcatcat catcagagat tacaatagcc ttcgtaacta tttcacgcat catttgatca
     1741 acaaatcttc gatcgtcttg gaagggctcc caaatgccat tcctacattg aagcatagga
     1801 aacttctgtg tttcggtacg aacaagttta ggcttctgct tcattaccgg aggcgtttcg
     1861 tcccatttct caatttcagt catatgaatg atttggaagc gacggcgtat aggagagcat
     1921 tcgacttcat ttggccaaat cgtatttgga ctgtaattgg aagtaataaa aatccgtttg
     1981 ggccttaatg ttcttgaacc aaacttcttt tcagctctga aaccatatct atcagcccat
     2041 atcttgacat ggtgtaagag gacagaatgt tccattccaa aatcttcgat aagaacatta
     2101 tcttcataat cataatcttc ccaccacttg ttgcaaagct taagatacac gtcgtcacct
     2161 acttgttagg agaaggcaaa aatcgatatt agggttaagc aacacaacta cttgttaggg
     2221 ttaaggtgta tgcatacctg ccatatttct agccttaaca gatttaccaa caccagcttc
     2281 tccccaaatc cacacattcg gaggggaacc tttcttccaa tcgagtttgg cggggacaat
     2341 cttgttttgg gtgtcgtcgc gaatcttctt gatggtagaa tagtacttga tggtcgtctc
     2401 ggcagggatg gcctcaaaat caccagcttt cgcagcgacc aacacttctt cccaagaagc
     2461 tttgattgtc tctagcccat ccttcggttg gattccatat tcggtaaatg cgccatcctt
     2521 tgtgcagtag gctctctgag aggtgcagtt accatcggcc gcaatgactt tggatttcgg
     2581 aaaaagctta accatagccg gatgcctgac cttggttttc atgtggacgt agccttgcaa
     2641 atgcggagtc ccagtagtag gtgcgacctc tcgaccaaag atcgaccact caaaaagatt
     2701 gttggcgaac cttgcctcct cagctgcatc ataattgtta gtgacaataa cccaaccctt
     2761 cctcgaagca tctctctttg ccatagtaaa ccttgaacag tcggtatagg gtgacgattc
     2821 gggagcccaa aatggtttga gcagcacgtg aaacctatca aacgaaattt caaaaaacac
     2881 gtgcgaaaac cctgttcagg gtaggcagca ggaggtcaaa aaaaaaccgt ggtacccagg
     2941 gcttgaacct gggaactggt gcaccagctg tgacggcgaa ccgctccact accacctccg
     3001 gtcctacgaa ggaccgaggc cgaaggcttg gggataccca aaattcaaag ggaaaagtct
     3061 tcactccgga tcctattatt ttccaagaat tgggtatttc caagaattag gacgaagtct
     3121 aagttggaaa ggatatttat tgacaacctg tcaaaaatca aagggggggg cc
//
LOCUS       CruV-175                3172 bp    DNA     circular     24-FEB-2020
DEFINITION  Crucivirus-175 isolate LSCO_103348.
ACCESSION   CruV-175
KEYWORDS    .
SOURCE      Crucivirus-175
  ORGANISM  Crucivirus-175
            Unclassified.
REFERENCE   1  (bases 1 to 3172)
  AUTHORS   de la Higuera,I., Kasun,G.W., Torrance,E.L., Pratt,A.A.,
            Maluenda,A., Colombet,J., Bisseux,M., Ravet,V., Dayaram,A.,
            Stainton,D., Kraberger,S., Zawar-Reza,P., Goldstien,S., Briskie,J.,
            White,R., Taylor,H., Gomez,C., Ainley,D.G., Harding,J.S.,
            Fontenele,R.S., Schreck,J., Ribeiro,S.G., Oswald,S.A., Arnold,J.M.,
            Varsani,A., Enault,F. and Stedman,K.M.
  TITLE     Direct Submission
  JOURNAL   Submitted (24-FEB-2020) Biology, Portland State University, 1719 SW
            10th Ave, Portland, Oregon 97201, USA
COMMENT     Bankit Comment: ALT EMAIL:ide@pdx.edu
            Bankit Comment: TOTAL # OF SEQS:451
            Bankit Comment: GAP: est. length - alignment

            ##Assembly-Data-START##
            Assembly Method       :: MetaSPAdes v. 3.12; IDBA-UD v. 1.1.1;
                                     MEGAHIT v. 1.0.3
            Sequencing Technology :: 454; Illumina
            ##Assembly-Data-END##
FEATURES             Location/Qualifiers
     source          1..3172
                     /organism="Crucivirus-175"
                     /mol_type="genomic DNA"
                     /isolate="LSCO_103348"
                     /isolation_source="Chirominidae"
                     /country="New Zealand"
                     /collection_date=2013
     misc_feature    join(3163..3172,1..16)
                     /note="stem-loop"
     misc_feature    1..9
                     /note="nonanucleotide"
     CDS             26..1576
                     /codon_start=1
                     /product="Putative CruV Capsid Protein"
                     /translation="MEPWSQHVPFHINLKFLFLIQLMPIGYRSPLLTRKPSTKKKVSR
                     PHDSMQQAKKRKSTYVAKQYANPPIPYEISQPNKFWRIKKGIPPVDYWRKRYYRRRIT
                     GRGDYSYSDSRSTGANIGGYLGSKAGEFLGGAAQNIGTALLSGLGSYSVKKNVLLDGN
                     MPRIDNKSGPGGITIRFQEYIGEVRSGPVAGAFNINTFLINPANSATFPWLSQIAANF
                     EQYEIEGMLFGFRTTASDLVTNDTNNLQLGTVILATQYDVADAVFQSKAEMLNYQFSS
                     SVKPSQGCIHMIECDPRQTSVNLLYCSPGSIPAGTDPRLYHLGRFSIATQGFQGDSVG
                     CGELHVTYQVRLLKPKLWSNLGQTIPFSSFFNQFTKAASIGPILGPIATISAQSQPNG
                     CNILFRAGTIASSTDMVFQANPAIPRKYLVCIFWNSAVAGNYWTSTNWPNLISPSGCN
                     VLVSAYAPTIGVLTSAYAQLNFYVTVTGTNIPELPYVNLPPADPNDLPNQDIGCTIRV
                     TVVDPDLA*"
     CDS             complement(1587..2075)
                     /codon_start=1
                     /product="putative ORF"
                     /translation="MEHSVLLHHVKIWADRYGFRAEKKFGSRTLRPKRIFITSNYSPN
                     TIWPNEVECSPIRRRFQIIHMTEIEKWDETPPVMKQKPKLVRTETQKFPMLQCRNGIW
                     EPFQDDRRFVDQMMREIVTKAIVISDDDDSVEITGAKAPCMFCSVKDEGLENCAFCGR
                     VF*"
     CDS             complement(2221..2784)
                     /codon_start=1
                     /product="Putative Replication Associated Protein"
                     /translation="MAKRDASRKGWVIVTNNYDAAEEARFANNLFEWSIFGREVAPTT
                     GTPHLQGYVHMKTKVRHPAMVKLFPKSKVIAADGNCTSQRAYCTKDGAFTEYGIQPKD
                     GLETIKASWEEVLVAAKAGDFEAIPAETTIKYYSTIKKIRDDTQNKIVPAKLDWKKGS
                     PPNVWIWGEAGVGKSVKARNMAGMHTP*"
ORIGIN
        1 aactattacc cccccccgtg gagccatgga gccatggagc caacacgtgc cttttcatat
       61 aaatttaaaa tttttatttc ttattcaatt aatgccgatt gggtaccgat caccactact
      121 caccaggaaa ccatctacca agaaaaaagt ctccaggcca cacgattcaa tgcagcaagc
      181 taaaaaacgg aaatcaactt acgtagccaa acaatatgcc aatcctccaa ttccttatga
      241 aataagtcag ccgaacaagt tttggagaat caaaaaaggg attccgcctg tcgattactg
      301 gagaaaacga tattacagac gacgaattac tgggcgaggg gactattctt attccgactc
      361 cagatcaacc ggcgctaaca taggagggta cctcggttca aaggccggtg aatttttagg
      421 cggagctgcc cagaatattg ggactgcatt actatccggt cttggttcct actcagttaa
      481 aaagaatgtt cttttggatg gaaatatgcc ccggatagac aataagtcag gaccaggtgg
      541 aatcactata cgcttccagg aatatatcgg cgaggttagg agtggcccag ttgctggggc
      601 gtttaatata aacacctttt taattaaccc cgcgaactct gctacatttc cttggttatc
      661 tcagattgct gctaacttcg aacaatacga aatcgaagga atgttattcg gcttccgaac
      721 taccgcatca gatcttgtta caaacgacac aaataactta cagcttggta ctgttatttt
      781 agctactcaa tacgatgttg ctgatgctgt atttcaatcg aaagccgaga tgttgaacta
      841 ccaattttca tcatcagtta aaccctccca gggttgtatt cacatgattg agtgtgaccc
      901 acgccaaacg tcagttaatt tactttattg ttcccccgga tctattccag ccggaaccga
      961 cccaaggtta taccatttag gacgtttctc gattgcaacg caaggatttc aaggagactc
     1021 tgttggttgt ggagaactcc acgttaccta tcaagtgcgt ttactgaaac ctaagttgtg
     1081 gtctaattta ggccaaacta ttccctttag ttcatttttc aatcaattca ctaaagcggc
     1141 atctattggc cctattctcg gtcccattgc gacaattagt gcccagtcgc agccgaatgg
     1201 ctgcaacata ttatttcgag caggaactat tgcaagctca acagacatgg tttttcaagc
     1261 aaacccagcg atccccagga aatatttggt ttgcatattt tggaattctg ctgttgctgg
     1321 aaattattgg acttcaacta attggcccaa ccttatttct cctagtggct gtaatgtgct
     1381 cgttagcgct tatgcaccaa caataggagt tcttacatcg gcatatgcac agctaaattt
     1441 ttatgttacg gtaacgggaa caaacattcc tgagttaccc tatgttaatc tgccaccggc
     1501 agatccaaat gatttgccta accaggatat tggttgcaca ataagggtaa cggtagtaga
     1561 tccagattta gcataataaa attttattaa aaaactcttc cacaaaaggc acaattctct
     1621 aggccctcat ctttaacgga gcaaaacatg caaggagcct tggctcctgt aatttcgacg
     1681 gaatcatcat catcagagat tacaatagcc ttcgtaacta tttcacgcat catttgatca
     1741 acaaatcttc gatcgtcttg gaagggctcc caaatgccat tcctacattg aagcatagga
     1801 aacttctgtg tttcggtacg aacaagttta ggcttctgct tcattaccgg aggcgtttcg
     1861 tcccatttct caatttcagt catatgaatg atttggaagc gacggcgtat aggagagcat
     1921 tcgacttcat ttggccaaat cgtatttgga ctgtaattgg aagtaataaa aatccgtttg
     1981 ggccttaatg ttcttgaacc aaacttcttt tcagctctga aaccatatct atcagcccat
     2041 atcttgacat ggtgtaagag gacagaatgt tccattccaa aatcttcgat aagaacatta
     2101 tcttcataat cataatcttc ccaccacttg ttgcaaagct taagatacac gtcgtcacct
     2161 acttgttagg agaaggcaaa aatcgatatt agggttaagc aacacaacta cttgttaggg
     2221 ttaaggtgta tgcatacctg ccatatttct agccttaaca gatttaccaa caccagcttc
     2281 tccccaaatc cacacattcg gaggggaacc tttcttccaa tcgagtttgg cggggacaat
     2341 cttgttttgg gtgtcgtcgc gaatcttctt gatggtagaa tagtacttga tggtcgtctc
     2401 ggcagggatg gcctcaaaat caccagcttt cgcagcgacc aacacttctt cccaagaagc
     2461 tttgattgtc tctagcccat ccttcggttg gattccatat tcggtaaatg cgccatcctt
     2521 tgtgcagtag gctctctgag aggtgcagtt accatcggcc gcaatgactt tggatttcgg
     2581 aaaaagctta accatagccg gatgcctgac cttggttttc atgtggacgt agccttgcaa
     2641 atgcggagtc ccagtagtag gtgcgacctc tcgaccaaag atcgaccact caaaaagatt
     2701 gttggcgaac cttgcctcct cagctgcatc ataattgtta gtgacaataa cccaaccctt
     2761 cctcgaagca tctctctttg ccatagtaaa ccttgaacag tcggtatagg gtgacgattc
     2821 gggagcccaa aatggtttga gcagcacgtg aaacctatca aacgaaattt caaaaaacac
     2881 gtgcgaaaac cctgttcagg gtaggcagca ggaggtcaaa aaaaaaccgt ggtacccagg
     2941 gcttgaacct gggaactggt gcaccagctg tgacggcgaa ccgctccact accacctccg
     3001 gtcctacgaa ggaccgaggc cgaaggcttg gggataccca aaattcaaag ggaaaagtct
     3061 tcactccgga tcctattatt ttccaagaat tgggtatttc caagaattag gacgaagtct
     3121 aagttggaaa ggatatttat tgacaacctg tcaaaaatca aagggggggg cc
//
LOCUS       CruV-176                3176 bp    DNA     circular     24-FEB-2020
DEFINITION  Crucivirus-176 isolate 001697_virusbaton.
ACCESSION   CruV-176
KEYWORDS    .
SOURCE      Crucivirus-176
  ORGANISM  Crucivirus-176
            Unclassified.
REFERENCE   1  (bases 1 to 3176)
  AUTHORS   de la Higuera,I., Kasun,G.W., Torrance,E.L., Pratt,A.A.,
            Maluenda,A., Colombet,J., Bisseux,M., Ravet,V., Dayaram,A.,
            Stainton,D., Kraberger,S., Zawar-Reza,P., Goldstien,S., Briskie,J.,
            White,R., Taylor,H., Gomez,C., Ainley,D.G., Harding,J.S.,
            Fontenele,R.S., Schreck,J., Ribeiro,S.G., Oswald,S.A., Arnold,J.M.,
            Varsani,A., Enault,F. and Stedman,K.M.
  TITLE     Direct Submission
  JOURNAL   Submitted (24-FEB-2020) Biology, Portland State University, 1719 SW
            10th Ave, Portland, Oregon 97201, USA
COMMENT     Bankit Comment: ALT EMAIL:ide@pdx.edu
            Bankit Comment: TOTAL # OF SEQS:451
            Bankit Comment: GAP: est. length - alignment

            ##Assembly-Data-START##
            Assembly Method       :: MetaSPAdes v. 3.12; IDBA-UD v. 1.1.1;
                                     MEGAHIT v. 1.0.3
            Sequencing Technology :: 454; Illumina
            ##Assembly-Data-END##
FEATURES             Location/Qualifiers
     source          1..3176
                     /organism="Crucivirus-176"
                     /mol_type="genomic DNA"
                     /isolate="001697_virusbaton"
                     /isolation_source="Water"
                     /country="France"
                     /collection_date=2013
     misc_feature    join(3172..3176,1..14)
                     /note="stem-loop"
     misc_feature    1..9
                     /note="nonanucleotide"
     CDS             332..1633
                     /codon_start=1
                     /product="Putative Replication Associated Protein"
                     /translation="MDNQVVKRKPRTTVKSVSKSSVKEDGRHRTYVFTINNYTDDMIE
                     LLQKIKCKCMQFGKEVAPSTGTKHLQGWIQFSDAKTLSAVCSIFKPFNKPHLEFMKGS
                     IEQNVKYCTKDNDVITVGVVPVAQKKGENKNTRLSEWESLDVDIQSGISMKDLMIKYP
                     RLSGECPSGFRMHFENLKPEYEYDISKETWFKGFLPFQSKIISILKSTPCHRSIYWIY
                     DKNGNSGKSKIVKYLSSCKEFKCIRISNGKTSDIAYCWKGEHILLDISRTSQEYINYD
                     LIEQLKNGQIFSSKYESVSKTYDVPHVFIFSNFEPDYVAMSLDRWRILEIDKKTNDFI
                     LCPDICKDNLKNKNIMYDNLNRMFSEFFPDVFLPQVFKPNHSLMCIKMPESLMCTEMP
                     ESLMCTEMPDSLMCTQDECYSSESSCISDMDIDFIQSSDEE*"
     CDS             1696..3009
                     /codon_start=1
                     /product="Putative CruV Capsid Protein"
                     /translation="MVKSVNTVGYRAPRSNAKPRVYGTTTKKQARHVRRKRIGQGIMK
                     ALPYVSKGLSYAAPFLPGPAQMAARGINSVLNTITGLGDYTVKYNSLMEGASTSLSGD
                     YVPIVTNSNGNVRIRHREYIQDINSTTVFNNTNFGINPGLRGSFPWLSAVAQNFQQYK
                     LHGCVFTFVSTSADALNSTNTALGTVMLGTNYNASQPVFTSKSQIENNEYTTSTKPSH
                     SVLHMIECDPEQTSSRGLLYVRTGNLPAGQDIKTYDIGNFQICTVGSQAITNIGELHV
                     SYDIELLKPQDLSILNYFEQVSHYYLPGILVAGNLHFGPAPFTRIDNIGITLTSNKIT
                     FPYNVNNGDVFLIMYSCTGVAAVLADPTLTFNACTRINYWLNGTNGGASDAGFNNTSY
                     ITSFAVQMNNPTTVNNPSITFTVGTLPTATCNGDLWIVQLPSNVS*"
ORIGIN
        1 gattaatata ataatttggg ggattgtgtt tacacaatgg ggggtctcag tattaccaga
       61 cccccccctg tggagtgttg aatagttttt agattgttat ctatgttcct ttttaggtgc
      121 tattcactcc acatcaagac gtaagtcttg tattattcgg tggttcatgt gtgtgtttta
      181 atttatttaa ataatatttt tgtgcattct tatataatat gttttcaatt gatgttgata
      241 cttattgttg agatttttat gctcaatttt ctaatatttt acgctacaca tgtcatccga
      301 ttttatttta gtgtaatata gtatatatta aatggacaac caagttgtta agagaaaacc
      361 aagaactaca gttaaatctg tttcaaaaag ttctgtaaag gaagatggta gacatcgtac
      421 ttatgttttt accattaata attatactga tgatatgatt gaattacttc aaaaaataaa
      481 atgtaaatgt atgcaatttg gtaaagaggt tgctccgtca actggtacaa agcatttaca
      541 aggatggatt caattttcag atgctaagac gctttctgct gtctgtagta tttttaagcc
      601 attcaataaa ccacatcttg aatttatgaa aggttctata gaacaaaatg tgaagtattg
      661 tactaaggat aacgatgtta tcacagtagg tgttgtacct gttgcacaaa agaaaggcga
      721 aaataaaaat actcgtttgt ctgaatggga atctttggat gtagatatac aatctggaat
      781 ttctatgaaa gatcttatga ttaaataccc aagattatca ggagaatgtc cttctggttt
      841 cagaatgcat ttcgagaatt taaaaccaga gtatgaatat gacatttcta aagagacatg
      901 gtttaagggt tttttgccat ttcaatcaaa aattatttca atattgaaat caactccatg
      961 tcatagatct atttattgga tatatgataa aaatggcaac tcgggtaaat caaaaatagt
     1021 gaaatatttg tcttcttgta aagaatttaa gtgtattaga atttctaatg gaaagacatc
     1081 agatatagca tattgttgga agggagaaca tattctttta gatatttcaa gaacttctca
     1141 agaatatatt aattatgatc tcattgaaca acttaaaaat ggtcaaatat tttcatcaaa
     1201 atatgaatca gtaagtaaaa cttatgatgt tcctcatgtg tttatatttt ctaattttga
     1261 accggattat gttgctatgt ctttagatag atggcgtatt ttagagattg ataaaaaaac
     1321 taatgatttt attttatgtc ctgatatttg taaggataat ttaaaaaata aaaatattat
     1381 gtatgataat ttaaacagaa tgttttctga gtttttccct gatgtttttt tacctcaggt
     1441 attcaaacca aatcactcct tgatgtgtat caagatgcct gaatccttga tgtgtaccga
     1501 gatgcctgaa tccttgatgt gtaccgagat gcctgattcc ttgatgtgta cacaagatga
     1561 atgttattct tctgaaagtt catgtataag tgatatggat attgatttta ttcaatcttc
     1621 agacgaagaa taataatatt gttgaaagtt tatcatccca ttaaatattt ttatatataa
     1681 tatgtatata aatatatggt taaatcagtt aatactgttg gatatcgtgc tccaagatct
     1741 aatgcaaagc caagagttta tggtacgaca acaaaaaagc aagcaaggca cgtaagaaga
     1801 aagcgtattg gacaaggtat aatgaaagca ttgccgtatg tttccaaagg tttgtcttac
     1861 gctgctccct ttttacctgg acctgctcaa atggcagcac gtggtattaa ttctgttctt
     1921 aatactataa ctggtttagg tgattatact gttaagtata atagtttaat ggaaggtgca
     1981 tctacttctt tatctggaga ttatgtacct attgttacta attctaatgg taatgtaaga
     2041 attagacatc gtgaatatat acaggatatt aattctacaa ctgtttttaa taacacaaat
     2101 tttggaatta atcctggttt acgtggttct tttccttggt tgtcagctgt agctcaaaac
     2161 tttcaacaat ataagcttca tggatgtgta tttacatttg tgtctacatc tgctgatgct
     2221 ttgaattcta caaatactgc tttgggaaca gttatgttag gaactaatta taatgcatct
     2281 cagcctgtat ttacttcaaa gtcacaaatt gaaaataatg aatatactac atccaccaaa
     2341 ccaagtcatt ctgttcttca tatgattgaa tgtgatccag agcagacttc ttctcgtggt
     2401 ttgctttatg tgagaaccgg aaatttacca gctggtcaag atatcaagac ctatgatatt
     2461 ggtaatttcc aaatatgtac tgttggttct caggcaatta caaatattgg agaacttcat
     2521 gtttcttatg atattgaatt gttgaagcct caagacttat caattttaaa ttattttgaa
     2581 caagtttctc attattatct tcctggaatt cttgtagctg gaaatcttca ttttgggcca
     2641 gctccattta caagaattga taatattggt attacattga cttctaataa aattacgttt
     2701 ccatataatg tgaataatgg tgatgtattt ttgataatgt attcatgtac aggagttgct
     2761 gctgttcttg cagatccgac acttacattt aatgcgtgta cgcgaatcaa ctattggttg
     2821 aatggtacaa atggtggagc atctgatgct ggttttaata atactagtta tattacttca
     2881 tttgcagttc aaatgaataa tccaacaact gttaataatc catctatcac atttaccgta
     2941 ggtactttac caacagctac atgtaatgga gatttgtgga tagttcaact tccatcaaat
     3001 gttagttaaa aacatgattt atttatttct gtttttttgt ttccaaataa ttcatatttg
     3061 aatttgttta ttcagatatt aaaattgatt aatataataa tttacattta tttgtttctt
     3121 gcaactttat tgattaatat aataatttac atttatttgt ttcttgcaac tttatt
//
LOCUS       CruV-177                3176 bp    DNA     circular     24-FEB-2020
DEFINITION  Crucivirus-177 isolate 120_1518_IR2_3297.
ACCESSION   CruV-177
KEYWORDS    .
SOURCE      Crucivirus-177
  ORGANISM  Crucivirus-177
            Unclassified.
REFERENCE   1  (bases 1 to 3176)
  AUTHORS   de la Higuera,I., Kasun,G.W., Torrance,E.L., Pratt,A.A.,
            Maluenda,A., Colombet,J., Bisseux,M., Ravet,V., Dayaram,A.,
            Stainton,D., Kraberger,S., Zawar-Reza,P., Goldstien,S., Briskie,J.,
            White,R., Taylor,H., Gomez,C., Ainley,D.G., Harding,J.S.,
            Fontenele,R.S., Schreck,J., Ribeiro,S.G., Oswald,S.A., Arnold,J.M.,
            Varsani,A., Enault,F. and Stedman,K.M.
  TITLE     Direct Submission
  JOURNAL   Submitted (24-FEB-2020) Biology, Portland State University, 1719 SW
            10th Ave, Portland, Oregon 97201, USA
COMMENT     Bankit Comment: ALT EMAIL:ide@pdx.edu
            Bankit Comment: TOTAL # OF SEQS:451
            Bankit Comment: GAP: est. length - alignment

            ##Assembly-Data-START##
            Assembly Method       :: MetaSPAdes v. 3.12; IDBA-UD v. 1.1.1;
                                     MEGAHIT v. 1.0.3
            Sequencing Technology :: 454; Illumina
            ##Assembly-Data-END##
FEATURES             Location/Qualifiers
     source          1..3176
                     /organism="Crucivirus-177"
                     /mol_type="genomic DNA"
                     /isolate="120_1518_IR2_3297"
                     /isolation_source="Water"
                     /country="Norway"
                     /collection_date=2011
     CDS             complement(1..957)
                     /codon_start=1
                     /product="Putative Replication Associated Protein"
                     /translation="MNKKCPCLGYDFTYVKTRADGKVNTFTMIGQALLKISNKFVFQL
                     EKGDIAGVEHYQGRCHYVKKTTQQHALNLFMSAMGLESEDYKNAPHVSTTTTGVHKHK
                     NFNYVLKLDTRIEGPWTDKDFVSDILIEPEYIQKRFRNNPLNTLTLLQQDVVRNCSDY
                     NDRIIDIIIDINGNNGKSYLSEVLTLFKNACTLPTTTSMKELIQYAYGYCNNKIINKV
                     KDCREISPFIMDMPRAVDSYELRTVIAALETIKKGRLTDLRYVNKEVIIEPPRVWLFC
                     NDVPPLQYLSKDRMRIWDIKEIDGANRLVPYIPTQKIVKKLV*"
     misc_feature    433..459
                     /note="stem-loop"
     misc_feature    441..449
                     /note="nonanucleotide"
     CDS             complement(1680..3116)
                     /codon_start=1
                     /product="Putative CruV Capsid Protein"
                     /translation="MSEKTYVRKTYVPKKKTYAKKKYAPKKRAYSSYVRGASSVSKRL
                     GNVLNSTDPRLSTLVKMSKNYISGMGDYDVSGITHNSLLGRLSPIIPEFNNNGAGGNI
                     KIVHREFIQDIKSSILFATNEFAINPADPITFPYLSRIASQFEQYRLDGLVFEFKSGS
                     SDALNSTNTALGYVIGAVEYNSLSAPFTSKLAMENAVFASSTKPSLSMITAVECSPDQ
                     TTIPLLYTRVNDAILAPSDLRLYDLGRFTLATIGMQAEDVNLGELWVSYSCTLMKCRL
                     NDPDPIPTNIKAWSFNFDDIALNNSPFGSYNSTNLDKFNNMGGVVDYSISGSDTRNLI
                     RIPAGNIGTYMLTYNVNGNAAPDPLSNTRPLFEDPFTPLVNITPVNYFAVDGQTPETS
                     PWTQTGLALDANNQNSVSLNYQMQYCFTIDDNNLESRLAMDINRSRFEDPTTPGDPGG
                     FVFTGLAGAPLTTGTCSIMEINPLMVSP*"
ORIGIN
        1 ttaaactaat ttcttaacaa ttttttgtgt agggatataa ggcactaatc tattagcacc
       61 atcaatctct tttatatccc atatcctcat tcggtcctta gacaaatatt gtaaaggcgg
      121 cacatcatta caaaataacc aaactcgtgg cggttctata ataacttctt tattaacata
      181 tcttaaatca gtcaaacgac cttttttaat cgtttctaaa gcagcaataa cagttcgtaa
      241 ttcataagaa tcaaccgcac ggggcatatc cataataaac ggggatattt cacgacaatc
      301 tttaacctta tttataatct tattattaca atacccataa gcatattgta taagttcttt
      361 catactcgtt gttgtaggaa gagtacatgc atttttaaaa agagttaata cctcagacaa
      421 ataactctta ccattattac cattaatatc tataataata tctattatcc tatcattata
      481 atcagaacaa tttctcacaa catcttgttg taaaagtgtt aatgtattta atggattatt
      541 tctaaaacgc ttttgtatat attcaggttc aatcaaaata tcactaacaa aatctttatc
      601 agtccatggt ccctcaattc gtgtatccaa ttttaataca taattaaaat tcttatgctt
      661 atgcaccccc gttgttgttg tactcacatg gggagcattc ttataatcct cagattctaa
      721 tcccatagca ctcataaaca aattcaatgc atgttgttgt gttgttttct taacataatg
      781 acaacgtcct tgataatgtt caacaccagc aatatcacct ttttctaatt gaaaaacaaa
      841 cttatttgat atcttcaata atgcttgacc aatcattgta aatgtattca cttttccatc
      901 cgcgcgagtt tttacataag taaaatcata acctaagcac ggacatttct tattcattat
      961 actatactat agataattta tttatccaat taaaacacac cctaatcgga taaattagaa
     1021 attctatata ttttatattt tttttcaaac attttaaaat atttttttaa aaagccataa
     1081 ggcatatata ctcgtgattt catcactcta cttgtatata tttttcaaaa agccataaga
     1141 taaaacataa ggcagacatt tttttaggca ttcatttcat tcattgtcat tccaactcat
     1201 atttcatatt cgttctaaaa aaatgtttat tcaatattta ttatatataa agtattccta
     1261 tatatataag tttaactatt tagataattc ggataaaggg catatgtcaa atatattatt
     1321 tagaaatcat tcggatatat atgcatttat ttggatatat atccaattaa tatttccagg
     1381 ttctattaca taatattgtt cttgtgtggg gggacacgcg gcgcaagcgc cttattgcat
     1441 aagcgtataa gtcgctcgcg acttatatcc cccctaacag ctcaactcat ctttatgtta
     1501 tcaccaggta ctcccccctt caactaagcc tctctttatc ctatatataa acactattat
     1561 taacatatcc tatcagatat attattaact atataattac ccattatata gggtatattt
     1621 ataagaattt attataaaac actatcattt atcactcatt tttaataata atattcacat
     1681 taaggactaa ccattaaagg attaatttcc ataatactac atgttccagt tgttaaaggg
     1741 gctccagcaa gcccagtaaa tacaaatcca ccagggtcgc caggggttgt tggatcttca
     1801 aatcttgatc tattaatatc catagctaat ctagattcaa gattattatc atcaatagtg
     1861 aaacaatatt gcatttgata attcaatgac acactatttt gattattcgc atccaacgct
     1921 aaaccagttt gagtccatgg agaagtttca ggagtttgac catccacagc aaaataatta
     1981 acaggagtaa tattaactaa cggagtaaat ggatcctcaa ataaagggcg tgtattactt
     2041 aatggatcag gagcagcatt accattaaca ttataagtaa gcatataagt cccaatatta
     2101 ccagcaggta ttctaataag atttcttgta tcactaccag aaattgaata atcaacaaca
     2161 ccacccatat tgttaaattt atctaaattt gtagaattgt aagacccaaa tggactatta
     2221 tttaaagcaa tatcatcaaa attaaaagac cacgctttaa tatttgtagg aataggatca
     2281 ggatcattca aacggcactt cattaaagta caactatagc taacccataa ttcaccaaga
     2341 ttaacatctt cagcttgcat tccaatagta gctaaagtaa agcgccccaa atcatataaa
     2401 cgtaaatcag aaggcgccaa tatagcgtca ttcacacgtg tatataataa aggtattgtt
     2461 gtttgatcag gagaacattc caccgcagta atcattgata atgaaggttt tgttgaactc
     2521 gcaaacacag cattttccat tgctaattta cttgtaaatg gagcagataa actattatat
     2581 tcaaccgcac caataacata ccccaacgca gtgttagtac tatttaaagc atcgcttgaa
     2641 ccacttttaa attcaaacac taatccatct aatctatatt gttcaaattg tgaagcaatt
     2701 cttgataaat atggaaatgt tataggatcc gcaggattaa tagcaaattc atttgtagca
     2761 aaaagtattg aacttttaat atcttgaata aattcacggt gtactatttt aatattacca
     2821 ccagccccat tattattaaa ttcaggaata atcggagata atcgtcctaa aagagaatta
     2881 tgagtaattc cactaacatc ataatccccc attccagaaa tataattttt tgacatttta
     2941 actaaagtcg ataatcgtgg atcagttgaa tttaatacat tacctaaccg cttactaaca
     3001 cttgaagcac ctctaacata acttgaataa gccctttttt taggagcata ttttttctta
     3061 gcatatgttt tctttttcgg cacataagtt ttcctcacat aagttttttc agacattata
     3121 aattaatata aagaaaatat tttttctata tattataaaa taattaatta ataatt
//
LOCUS       CruV-178                3177 bp    DNA     circular     24-FEB-2020
DEFINITION  Crucivirus-178 isolate 001694_virusbaton.
ACCESSION   CruV-178
KEYWORDS    .
SOURCE      Crucivirus-178
  ORGANISM  Crucivirus-178
            Unclassified.
REFERENCE   1  (bases 1 to 3177)
  AUTHORS   de la Higuera,I., Kasun,G.W., Torrance,E.L., Pratt,A.A.,
            Maluenda,A., Colombet,J., Bisseux,M., Ravet,V., Dayaram,A.,
            Stainton,D., Kraberger,S., Zawar-Reza,P., Goldstien,S., Briskie,J.,
            White,R., Taylor,H., Gomez,C., Ainley,D.G., Harding,J.S.,
            Fontenele,R.S., Schreck,J., Ribeiro,S.G., Oswald,S.A., Arnold,J.M.,
            Varsani,A., Enault,F. and Stedman,K.M.
  TITLE     Direct Submission
  JOURNAL   Submitted (24-FEB-2020) Biology, Portland State University, 1719 SW
            10th Ave, Portland, Oregon 97201, USA
COMMENT     Bankit Comment: ALT EMAIL:ide@pdx.edu
            Bankit Comment: TOTAL # OF SEQS:451
            Bankit Comment: GAP: est. length - alignment

            ##Assembly-Data-START##
            Assembly Method       :: MetaSPAdes v. 3.12; IDBA-UD v. 1.1.1;
                                     MEGAHIT v. 1.0.3
            Sequencing Technology :: 454; Illumina
            ##Assembly-Data-END##
FEATURES             Location/Qualifiers
     source          1..3177
                     /organism="Crucivirus-178"
                     /mol_type="genomic DNA"
                     /isolate="001694_virusbaton"
                     /isolation_source="Water"
                     /country="France"
                     /collection_date=2013
     misc_feature    join(3167..3177,1..18)
                     /note="stem-loop"
     misc_feature    1..9
                     /note="nonanucleotide"
     CDS             complement(681..1664)
                     /codon_start=1
                     /product="Putative Replication Associated Protein"
                     /translation="MEHKMNISNSPCKWFDMTWHQSFHEVEHSVLIDCFKKHCDKWGF
                     QLEICPETGNLHYQCRISMKVKIRHKQLVSMEDFNGFHIRPTVKENIGNLNYVTKDAS
                     RMEGPWVWDKMPAYVPRQYRETIECKPHPWQSEFLNYSEEWDTKHINILIESKGVVGK
                     STLINYCDIKGYGCIIPVMKEYKDLMRAVCSQPESKLYLIDIPRALDHGKMADLFAAI
                     ETIKGGYAWDDRYKFVKRHFDCPNVWVFTNHLPNLECLSNGRWRFWKLINNHLREVDV
                     KSFEEPQISRKFIQDKSSKNSETKSSGVLPMRYDFACAEHSIVGCRTCYIG*"
     CDS             1765..3150
                     /codon_start=1
                     /product="Putative CruV Capsid Protein"
                     /translation="MPRYSTRRAPVRRRAPARRRAPVRRRAPIRNRVTEVYDDSPAPI
                     VVYNKTSRRAQTGGTESSNGSLGRSLLSGGLGALGGAAFGPVGRGIGSLVGGGLSSLL
                     GFGAYQVKSNVFMDGKLPRVTNDPQGGGTIIRFQEYLGDVITSATANTFSLNSYLINA
                     ANPACFPWLSQIAENYEQYSFEGLLFEFKTTSADALNSTNTALGTVMFSTNYDSFDSN
                     FASKGEMLNYEYSTSCKPSHDVMHMIECSPRQNVLSELYTLNGAVPSGADPRLYHLGN
                     FQVATTGFQGTSVNCGMLYCTYQVRLMKPKLFSTLGLANNIAHYTLSAASAAAPLGTV
                     ASATKNVDSIGLTLTSSTVLTFPQSQIREYFMVYVTALGNSTATLTAPLLTFSGLTQV
                     SLGGLSASGSSPAQTPTTSDTGARLTYTQICYTNGGGVIPTITFGVAGTYPAAVSASH
                     IYVMQIPSSII*"
ORIGIN
        1 caataatata tgtaaaaaac tcactcacat aatttgggtt cggttacttt tctgtcttgc
       61 atgcttctgt gtgcacagtg caaaaaggca aaagtttatc atatagtatt aactactatt
      121 atgatatcca aaaccaaaca agcgtaataa agacaacata atattatatt gggaggattc
      181 cttgtatttt gtgtgcggag cactcaaaat ataaggaggg acccaatata atattatgtt
      241 gccattactc ttatgtgctt ttgtatatta tcatgtaggc atacattaga atgctcgttt
      301 ttataccgta cacttacgga gtgcataggg aaaaaataca aaacgacata tactaccata
      361 acgtataaag tatagtcggt gagcgtcact tacccgcatg ttctacaact tgttccaagt
      421 tgttccacct tagtggtatt tataaattag cgataatgtt cactaattta acgattggtt
      481 ccaacgttcc aaatagttaa aattatatat atagtgtaag cttcgcttac acgtgtagtt
      541 cctacatata aacgtagtat tatatggtta ttacagaaaa gcggcataac gcttttcgcc
      601 ataggaacta cacacatgcg gttttttcgc tagaacacct ccttgcgaaa taaattaaat
      661 agtgtaaccg agccgcataa ttatccgatg taacatgtac gacaccccac aatactatgt
      721 tcggcacatg caaaatcata cctcatgggc aatacaccac tagattttgt ctcgctattt
      781 ttactggatt tatcctgtat aaatttacga gatatttgag gttcttcaaa actttttaca
      841 tctacctccc gaaggtgatt gttaataagt ttccaaaacc tccatcggcc attagacaga
      901 cattctaaat tcggaaggtg attggtaaaa acccacacat tcggacaatc aaagtgcctc
      961 ttgacaaact tgtagcgatc atcccaagca tatccacctt tgatagtctc tatagctgca
     1021 aacagatcgg ccattttccc gtgatctaaa gcgcggggaa tatcgattaa atagagttta
     1081 gattctggtt gtgaacatac ggctctcata agatctttat attccttcat aacaggaatg
     1141 atacagccat aacctttaat atcacaatag ttaatcaaag tggacttacc gacaacccct
     1201 ttggattcta ttagtatatt aatatgttta gtatcccatt cctcggaata attaaggaat
     1261 tcggattgcc agggatgtgg tttacattca attgtctcgc gatattgcct tggcacatac
     1321 gcgggcattt tatcccaaac ccacggaccc tccatccgag acgcgtcttt agtgacataa
     1381 ttaagatttc caatgttttc tttaaccgta ggccttatat gaaatccatt gaaatcttcc
     1441 atagacacaa gctgtttatg acgaatctta actttcatag atatcctgca ctgataatgt
     1501 aaattacccg tctcaggaca tatctcgagt tgaaaccccc atttatcgca atgtttttta
     1561 aaacaatcga ttaacaccga atgttcaact tcgtgaaagg attgatgcca agtcatatca
     1621 aaccatttac atggtgaatt tgaaatattc atcttatgtt ccatggttcc agatcttatt
     1681 atactatata taaaaatgtt atttaaaata aaaaatttta tatatatata gtataagtta
     1741 cactatattc ataagtgata aaacatgcct cgatattcta cgcgtagagc cccggtaaga
     1801 agacgggcac cagcaaggag aagagcaccg gtacgaaggc gggcccctat ccgaaatagg
     1861 gtgactgaag tatatgatga ctctccagca ccaattgttg tttacaacaa aacgtcaagg
     1921 agagcacaga ccggaggcac cgaatcatct aatggctcac ttggacgatc acttttaagt
     1981 ggcggcttag gtgcattagg aggcgctgca tttggcccgg taggaagagg aataggatca
     2041 ctggtaggtg gtggattatc atcactactt ggattcgggg cttaccaagt aaagtccaac
     2101 gtctttatgg acggcaagtt accccgagtg actaacgatc ctcaaggcgg aggtactatt
     2161 attcgcttcc aagaatacct aggtgatgta ataaccagtg ctactgctaa cacatttagc
     2221 ttgaatagtt accttatcaa tgctgctaat ccagcgtgtt ttccatggct atctcaaatc
     2281 gcggaaaact acgaacaata cagctttgaa ggtttactgt ttgaattcaa gacaacaagc
     2341 gcagatgcac taaactctac taacactgca ttaggaactg ttatgttttc tactaattat
     2401 gattcgtttg attcaaactt tgcttcgaaa ggagaaatgt tgaactacga atattcaacg
     2461 agttgcaaac catcacatga cgtcatgcat atgattgaat gtagtccacg ccagaatgta
     2521 ttatccgaat tgtatacgtt gaacggagct gtcccaagcg gcgcggatcc acgtttatac
     2581 catctcggta attttcaagt ggcaaccacc gggttccaag gaacatcagt taactgtggt
     2641 atgctttatt gtacttatca agttcgatta atgaaaccta aattgttttc tacattagga
     2701 ttagcaaata atattgcaca ttatacgcta tccgccgcta gtgcagctgc accactggga
     2761 acagttgcta gtgctactaa gaatgtcgat tcaattggtt tgacacttac atcgtcgacg
     2821 gttttgacat ttccgcaaag tcaaatccgc gaatacttta tggtctatgt tactgcattg
     2881 ggcaattcta cggcaacgtt aactgcacca ttgctaacat ttagtggctt gacccaagtg
     2941 tctttgggcg gtcttagtgc ttcaggctct tcaccagctc aaacacctac cacatcagac
     3001 accggagcta ggttaacata tacgcagata tgttatacta atggtggtgg agtgataccc
     3061 acaattacat ttggggtagc cggaacctac ccagctgccg tatctgcttc acacatttat
     3121 gtcatgcaga ttccatcaag tattatttga tcagacaacg tgttaatttt tacatat
//
LOCUS       CruV-179                3180 bp    DNA     circular     24-FEB-2020
DEFINITION  Crucivirus-179 isolate 120_535_IR1_3296.
ACCESSION   CruV-179
KEYWORDS    .
SOURCE      Crucivirus-179
  ORGANISM  Crucivirus-179
            Unclassified.
REFERENCE   1  (bases 1 to 3180)
  AUTHORS   de la Higuera,I., Kasun,G.W., Torrance,E.L., Pratt,A.A.,
            Maluenda,A., Colombet,J., Bisseux,M., Ravet,V., Dayaram,A.,
            Stainton,D., Kraberger,S., Zawar-Reza,P., Goldstien,S., Briskie,J.,
            White,R., Taylor,H., Gomez,C., Ainley,D.G., Harding,J.S.,
            Fontenele,R.S., Schreck,J., Ribeiro,S.G., Oswald,S.A., Arnold,J.M.,
            Varsani,A., Enault,F. and Stedman,K.M.
  TITLE     Direct Submission
  JOURNAL   Submitted (24-FEB-2020) Biology, Portland State University, 1719 SW
            10th Ave, Portland, Oregon 97201, USA
COMMENT     Bankit Comment: ALT EMAIL:ide@pdx.edu
            Bankit Comment: TOTAL # OF SEQS:451
            Bankit Comment: GAP: est. length - alignment

            ##Assembly-Data-START##
            Assembly Method       :: MetaSPAdes v. 3.12; IDBA-UD v. 1.1.1;
                                     MEGAHIT v. 1.0.3
            Sequencing Technology :: 454; Illumina
            ##Assembly-Data-END##
FEATURES             Location/Qualifiers
     source          1..3180
                     /organism="Crucivirus-179"
                     /mol_type="genomic DNA"
                     /isolate="120_535_IR1_3296"
                     /isolation_source="Water"
                     /country="Norway"
                     /collection_date=2011
     CDS             join(complement(1..3),complement(2773..3180))
                     /codon_start=1
                     /product="putative ORF"
                     /translation="MMPGNYKIYYFLLYTYVSFFRIKMQEIPTQFRFTYGDGVIQRNN
                     LYYFSHQFANELILQNPLQKDIINSVREPTLEPSDRNIFHILNRINYTESQRTVRIRR
                     NKINRKFKNLATRSNTGYIPKDIYFPRHTDNQND*"
     misc_feature    join(3171..3180,1..16)
                     /note="stem-loop"
     misc_feature    1..9
                     /note="nonanucleotide"
     CDS             complement(94..981)
                     /codon_start=1
                     /product="Putative Replication Associated Protein"
                     /translation="MTSRNYCFTAYHERELENLRNFDKTKYICWGNEICPTTMRPHLQ
                     GYIELKEPIRITGFKKISPTTHFEKRKGTQDEAIDYCKKDGNFFEEGTRAKGQGTRTD
                     LEELKDSLDNNMDIKSISENHFNCFMKYNRSIKEYQSLHTEHRNWETNLEIHIGGPGS
                     GKSHHCKTTYPNAYWMMKPNGGSVFFDGYTGQDTIILDEFYGWLPWDLLLRLADKYPL
                     ILQTKGGSVNCIIKNLIITSNKNVPEWYKDTFDLDPLIRRITLYKDDYVTGKYIDKSK
                     FENMKNKIFNIKNNDDCQD*"
     CDS             complement(1014..2357)
                     /codon_start=1
                     /product="Putative CruV Capsid Protein"
                     /translation="MLPKSVYNKLSISDKKKVKSSLKKGKGPGSGAYTFDKNTNPFGH
                     YGEKIGKYISPFLGSYAPLARSVGAYGGHALGKLFGSGAYINSRGKYIPSYSGAHKMI
                     RGSGAYYEEPRGVNTTVTSPPSFGSNRTIIRHREFIGNIVGSAAFTINTWNVNPGDES
                     TFPWLASIAANYEKYRPIGIVFEFKSTSASALNSTNTALGTVMMAPRYNAIAQSVPQS
                     KMEMLQIENCSSICPAENGQVGIECAKNYNPLGVLYIRNQTQAVTGSEQMYDFCDFYL
                     ATEGMQAAATIGEIWVTYEIELLTPILASGQVGRNINMANYTLNSSISTSAYFSGATE
                     GLGHNMDLTLSGTTIIMPPDIVTGSYVLSYSVSGSSASVVQPTVSFTSGCTGYALYQS
                     PGINNYGTVATVNFLIKQVAFTITAGNAILTFSGATMPASVINGSLTISQIPLAE*"
ORIGIN
        1 cagtattacc ccgggacttt gtgacgtgac agcttgctgt tcggaataaa gtacataatg
       61 agaccgtagc ggtccgtaag gaaaaacaat gttttagtct tgacagtcat cattattttt
      121 aatattgaag attttattct tcatattttc aaatttcgat ttatcgatat atttgcctgt
      181 gacgtaatca tctttgtata aagttattcg tctaataagg gggtcaagat caaacgtatc
      241 tttataccat tctggtacgt tcttgttact agttatgatt aaatttttaa ttatacagtt
      301 aacagagccc cctttggttt gcaatattaa gggatattta tcagctagtc gcaataataa
      361 atcccatggc aaccatccat agaattcgtc tagaattata gtatcctgtc cagtgtatcc
      421 atcaaaaaat acggatcctc cgttaggttt catcatccaa taagcattgg ggtatgttgt
      481 tttacaatga tgagatttac ctgaaccagg tccaccaata tgaatctcta aattggtttc
      541 ccaatttcga tgttcagtat gtaaagattg atattcttta atcgatctgt tatatttcat
      601 aaaacagtta aagtgatttt cactaatcga tttaatatcc atattattat ctaaagaatc
      661 ttttaattct tctagatcag ttctagtacc ttgccctttg gcacgtgtac cttcctcgaa
      721 gaaattacca tcttttttac agtaatcaat agcttcgtct tgagtacctt tacgtttttc
      781 aaaatgagtt gttggcgata tttttttaaa accagtaatg cgaataggct cttttaattc
      841 gatataacct tgtaaatgcg gacgcatagt tgttggacat atttcattgc cccaacaaat
      901 atattttgtt ttatcaaaat tacgtaggtt ttctaattca cgttcatgat atgctgtaaa
      961 gcagtaattt ctagaggtca ttttatatat aaaaaaaata atatattatt tatttattct
     1021 gctagtggta tttgcgatat agttaacgat ccgttaataa cagatgctgg catcgtcgct
     1081 ccactaaatg ttaatatagc attaccagct gtgatggtaa aagcaacttg tttaattaaa
     1141 aagtttactg tagctacagt accataatta ttaataccag gcgattgata taatgcgtat
     1201 ccagtacatc ctgatgtgaa tgatactgta ggttgaacaa ccgaagcaga cgaaccagat
     1261 accgaataac ttaatacata tgatcctgta acaatatcag gaggcatgat gattgttgtt
     1321 cccgaaaggg ttaaatccat attatgtcct aatccttctg tagctcctga aaagtatgct
     1381 gaagttgaga tactagaatt caatgtataa ttagccatat tgatattgcg tccgacttgt
     1441 cctgatgcaa gaatgggagt aagtaactct atttcataag ttacccatat ctcaccgatg
     1501 gtagcagctg cttgcatacc ttctgtagct aaatagaaat cacaaaaatc atacatttgt
     1561 tcagatcctg taacagcttg tgtttgattt ctaatatata acactccgag aggattataa
     1621 tttttagcac attcaatacc tacttgacca ttctcagcag gacaaataga agagcaattt
     1681 tcaatttgca acatttccat tttagattga ggtacagatt gagcaatagc attatatcta
     1741 ggagccatca taacggtacc taacgcagta ttggtactgt tcaacgcact agctgacgtt
     1801 gatttaaatt caaatacaat accgataggt ctgtattttt cataattagc agcaatagat
     1861 gctaaccaag gaaacgtact ttcatcacca ggattaacat tccatgtatt aatcgtgaaa
     1921 gcagcggacc ctactatatt tccaataaat tctctatgtc taataattgt tctattagag
     1981 ccaaaacttg gcggtgatgt aacagttgta ttaactcctc ttggttcttc atagtaagca
     2041 ccagatcctc taatcatttt atgggcacca gaatatgatg gtatgtattt acctcgacta
     2101 ttaatataag cacctgaacc aaataattta cctaaagcat gaccaccata agcaccaact
     2161 gatcttgcta gtggagcgta agatccaagg aatggagaaa tatatttacc aattttttct
     2221 ccgtaatgac caaatggatt tgtattttta tcaaaagtat aagcacctga tccaggtcct
     2281 ttaccttttt taagagaaga ttttaccttc tttttatcag aaattgatag tttattataa
     2341 acagatttag gtagcatttt aatgcaaaaa aaaagaatat ttattatttt tttaaacgtc
     2401 ttaaaccagg agttttagat aaaactactg ttggtgtttg tggttcagga ttatttttaa
     2461 agacgaggtc actcatttgt aatttaagtt gagttatttc tccttgaagt aaagtcaacg
     2521 tattacgtat ttcaattatc tgctcgaagg tatcttctaa agcaggtata taaacttcat
     2581 atggtttaga atcattactg aatgattcaa catcagcatc atgtgtttta tgtgtataat
     2641 ctatattcat tttaatagta aaaaaaaaga atatttttta ttgggagaag gtttatacac
     2701 tggcaggggg cccctcagag agggggggcg cagccaaata agggcagacc gaaggggcgc
     2761 cagcgtttat gtttaatcat tttgattatc agtatgacga ggaaaataaa tatcctttgg
     2821 gatatatcct gtatttgatc tcgtagctaa atttttaaat tttctattta ttttatttct
     2881 tcttatacga actgtacgtt gtgattctgt ataattaatt cgatttaata tgtgaaatat
     2941 atttctatct gaaggttcta atgtaggttc tcgtactgaa ttaataatat ccttttgtaa
     3001 aggattttgt aaaatgagct cattagcgaa ttgatgagaa aagtagtaaa ggttattccg
     3061 ttgaataaca ccatcaccat aagtaaatct gaattgtgta ggtatttctt gcattttaat
     3121 gcgaaaaaaa gaaacgtaag tgtataagag aaaataatat attttataat tcccgggcat
//
LOCUS       CruV-180                3180 bp    DNA     circular     24-FEB-2020
DEFINITION  Crucivirus-180 isolate 120_1520_IR2_3297.
ACCESSION   CruV-180
KEYWORDS    .
SOURCE      Crucivirus-180
  ORGANISM  Crucivirus-180
            Unclassified.
REFERENCE   1  (bases 1 to 3180)
  AUTHORS   de la Higuera,I., Kasun,G.W., Torrance,E.L., Pratt,A.A.,
            Maluenda,A., Colombet,J., Bisseux,M., Ravet,V., Dayaram,A.,
            Stainton,D., Kraberger,S., Zawar-Reza,P., Goldstien,S., Briskie,J.,
            White,R., Taylor,H., Gomez,C., Ainley,D.G., Harding,J.S.,
            Fontenele,R.S., Schreck,J., Ribeiro,S.G., Oswald,S.A., Arnold,J.M.,
            Varsani,A., Enault,F. and Stedman,K.M.
  TITLE     Direct Submission
  JOURNAL   Submitted (24-FEB-2020) Biology, Portland State University, 1719 SW
            10th Ave, Portland, Oregon 97201, USA
COMMENT     Bankit Comment: ALT EMAIL:ide@pdx.edu
            Bankit Comment: TOTAL # OF SEQS:451
            Bankit Comment: GAP: est. length - alignment

            ##Assembly-Data-START##
            Assembly Method       :: MetaSPAdes v. 3.12; IDBA-UD v. 1.1.1;
                                     MEGAHIT v. 1.0.3
            Sequencing Technology :: 454; Illumina
            ##Assembly-Data-END##
FEATURES             Location/Qualifiers
     source          1..3180
                     /organism="Crucivirus-180"
                     /mol_type="genomic DNA"
                     /isolate="120_1520_IR2_3297"
                     /isolation_source="Water"
                     /country="Norway"
                     /collection_date=2011
     CDS             join(complement(1..3),complement(2773..3180))
                     /codon_start=1
                     /product="putative ORF"
                     /translation="MMPGNYKIYYFLLYTYVSFFRIKMQEIPTQFRFTYGDGVIQRNN
                     LYYFSHQFANELILQNPLQKDIINSVREPTLEPSDRNIFHILNRINYTESQRTIRIRR
                     NKINRKFKNLATRSNTGYIPKDIYFPRHTDNQND*"
     misc_feature    join(3171..3180,1..16)
                     /note="stem-loop"
     misc_feature    1..9
                     /note="nonanucleotide"
     CDS             complement(94..981)
                     /codon_start=1
                     /product="Putative Replication Associated Protein"
                     /translation="MTSRNYCFTAYHERELENLRNFDKTKYICWGNEICPTTMRPHLQ
                     GYIELKEPIRITGFKKISPTTHFEKRKGTQDEAIDYCKKDGNFFEEGTRAKGQGTRTD
                     LEELKDSLDNNMDIKSISENHFNCFMKYNRSIKEYQSLHTEHRNWETNLEIHIGGPGS
                     GKSHHCKTTYPNAYWMMKPNGGSVFFDGYTGQDTIILDEFYGWLPWDLLLRLADKYPL
                     ILQTKGGSVNCIIKNLIITSNKNVPEWYKDTFDLDPLIRRITLYKDDYVTGKYIDKSK
                     FENMKNKIFNIKNNDDRQD*"
     CDS             complement(1014..2357)
                     /codon_start=1
                     /product="Putative CruV Capsid Protein"
                     /translation="MLPKSVYNKLSISDKKKVKSSLKKGKGPGSGAYTFDKNTNPFGH
                     YGEKIGKYISPFLGSYAPLARSVGAYGGHALGKLFGSGAYINSRGKYIPSYSGAHKMI
                     RGSGAYYEEPRGVNTTVTSPPSFGSNRTIIRHREFIGNIVGSAAFTINTWNVNPGDES
                     TFPWLASIAANYEKYRPIGIVFEFKSTSASALNSTNTALGTVMMAPRYNAIAQSVPQS
                     KMEMLQIENCSSICPAENGQVGIECAKNYNPLGVLYIRNQTQAVTGSEQMYDFCDFYL
                     ATEGMQAAATIGEIWVTYEIELLTPILASGQVGRNINMANYTLNSSISTSAYFSGATE
                     GLGHNMDLTLSGTTIIMPPDIITGSYVLSYSVSGSSASVVQPTVSFTSGCTGYALYQS
                     PGINNYGTVATVNFLIKQVAFTITAGNAILTFSGATMPASVINGSLTISQIPLAE*"
ORIGIN
        1 cagtattacc ccgggacttt gtgacgtgac agcttgctgt tcggaataaa gtacataatg
       61 agaccgtagc ggtccgtaag gaaaaacaat gttttagtct tgacggtcat cattattttt
      121 aatattgaag attttattct tcatattttc aaatttcgat ttatcgatat atttgcctgt
      181 gacgtaatca tctttgtata aagttattcg tctaataagg gggtcaagat caaacgtatc
      241 tttataccat tctggtacgt tcttgttact agttatgatt aaatttttaa ttatacagtt
      301 aacagagccc cctttggttt gcaatattaa gggatattta tcagctagtc gcaataataa
      361 atcccatggc aaccatccat agaattcgtc tagaattata gtatcctgtc cagtgtatcc
      421 atcaaaaaat acggatcctc cgttaggttt catcatccaa taagcattgg ggtatgttgt
      481 tttacaatga tgagatttac ctgaaccagg tccaccaata tgaatctcta aattggtttc
      541 ccaatttcga tgttcagtat gtaaagattg atattcttta atcgatctgt tatatttcat
      601 aaaacagtta aagtgatttt cactaatcga tttaatatcc atattattat ctaaagaatc
      661 ttttaattct tctagatcag ttctagtacc ttgccctttg gcacgtgtac cttcctcgaa
      721 gaaattacca tcttttttac agtaatcaat agcttcgtct tgagtacctt tacgtttttc
      781 aaaatgagtt gttggcgata tttttttaaa accagtaatg cgaataggct cttttaattc
      841 gatataacct tgtaaatgcg gacgcattgt tgttggacat atttcattgc cccaacaaat
      901 atattttgtt ttatcaaaat tacgtaggtt ttctaattca cgttcatgat atgctgtaaa
      961 gcagtaattt ctagaggtca ttttatatat aaaaaaaata atatattatt tatttattct
     1021 gctagtggta tttgcgatat agttaacgat ccgttaataa cagatgctgg catcgtcgct
     1081 ccactaaatg ttaatatagc attaccagct gtgatggtaa aagcaacttg tttaattaaa
     1141 aagtttactg tagctacagt accataatta ttaataccag gcgattgata taatgcgtat
     1201 ccagtacatc ctgatgtgaa tgatactgta ggttgaacaa ccgaagcaga cgaaccagat
     1261 accgaataac ttaatacata tgatcctgta ataatatcag gaggcatgat gattgttgtt
     1321 cccgaaaggg ttaaatccat attatgtcct aacccttctg tagctcctga aaagtatgct
     1381 gaagttgaga tactagaatt caatgtataa ttagccatat tgatattgcg tccgacttgt
     1441 cctgatgcaa gaatgggagt aagtaactct atttcataag ttacccatat ctcaccgatg
     1501 gtagcagctg cttgcatacc ttctgtagct aaatagaaat cacaaaaatc atacatttgt
     1561 tcagatcctg taacagcttg tgtttgattt ctaatatata acactccgag aggattataa
     1621 tttttagcac attcaatacc tacttgacca ttctcagcag gacaaataga agagcaattt
     1681 tcaatttgca acatttccat tttagattga ggcacagatt gagcaatagc attatatcta
     1741 ggagccatca taacggtacc taacgcagta ttggtactgt tcaatgcact agctgacgtt
     1801 gatttaaatt caaatacaat accgataggt ctgtattttt cataattagc agcaatagat
     1861 gctaaccaag gaaacgtact ttcatcacca ggattaacat tccatgtatt aatcgtgaaa
     1921 gcagcggatc ctactatatt tccaataaat tctctatgtc taataattgt tctattagat
     1981 ccaaaacttg gcggtgatgt aacagttgta ttaactcccc ttggttcttc atagtaagca
     2041 ccagatcctc taatcatttt atgggcacca gaatatgacg gtatgtattt acctcgacta
     2101 ttaatataag cacctgagcc aaataattta cctaaagcat gaccaccata agcaccaact
     2161 gatcttgcta gtggagcgta agatccaagg aatggagaaa tatatttacc aattttttct
     2221 ccgtaatgac caaatggatt tgtattttta tcaaaagtat aagcacctga tccaggtcct
     2281 ttaccttttt taagagaaga ttttactttc tttttatcag aaattgatag tttattataa
     2341 acagatttag gtagcatttt aatgcaaaaa aaaagaatat ttattatttt tttaaacgtc
     2401 ttaaaccagg agttttagat aaaactactg ttggtgtttg tggttcagga ttatttttaa
     2461 agacgaggtc actcatttgt aatttaagtt gagttatttc tccttgaagt aaagtcaacg
     2521 tattacgtat ttcaattatc tgctcgaagg tatcttctaa agcaggtata taaacttcat
     2581 atggtttaga atcattactg aatgattcaa catcagcatc atgtgtttta tgtgtataat
     2641 ctatattcat tttaatagta aaaaaaaaga atatttttta ttgggagaag gtttatacac
     2701 tggcaggggg cccctcagag agggggggcg cagccaaata agggcagacc gaaggggcgc
     2761 cagcgtttat gtttaatcat tttgattatc agtatgacga ggaaaataaa tatcctttgg
     2821 gatatatcct gtatttgatc tcgtagctaa atttttaaat tttctattta ttttatttct
     2881 tcttatacga attgtacgtt gtgattctgt ataattaatt cgatttaata tgtgaaatat
     2941 atttctatct gaaggttcta atgtaggttc tcgtactgaa ttaataatat ccttttgtaa
     3001 aggattttgt aaaatgagct cattagcgaa ttgatgagaa aagtagtaaa ggttattccg
     3061 ttgaataaca ccatcaccat aagtaaatct gaattgtgta ggtatttctt gcattttaat
     3121 gcgaaaaaaa gaaacgtaag tgtataagag aaaataatat attttataat tcccgggcat
//
LOCUS       CruV-181                3181 bp    DNA     circular     24-FEB-2020
DEFINITION  Crucivirus-181 isolate 120_522_IR1_3296.
ACCESSION   CruV-181
KEYWORDS    .
SOURCE      Crucivirus-181
  ORGANISM  Crucivirus-181
            Unclassified.
REFERENCE   1  (bases 1 to 3181)
  AUTHORS   de la Higuera,I., Kasun,G.W., Torrance,E.L., Pratt,A.A.,
            Maluenda,A., Colombet,J., Bisseux,M., Ravet,V., Dayaram,A.,
            Stainton,D., Kraberger,S., Zawar-Reza,P., Goldstien,S., Briskie,J.,
            White,R., Taylor,H., Gomez,C., Ainley,D.G., Harding,J.S.,
            Fontenele,R.S., Schreck,J., Ribeiro,S.G., Oswald,S.A., Arnold,J.M.,
            Varsani,A., Enault,F. and Stedman,K.M.
  TITLE     Direct Submission
  JOURNAL   Submitted (24-FEB-2020) Biology, Portland State University, 1719 SW
            10th Ave, Portland, Oregon 97201, USA
COMMENT     Bankit Comment: ALT EMAIL:ide@pdx.edu
            Bankit Comment: TOTAL # OF SEQS:451
            Bankit Comment: GAP: est. length - alignment

            ##Assembly-Data-START##
            Assembly Method       :: MetaSPAdes v. 3.12; IDBA-UD v. 1.1.1;
                                     MEGAHIT v. 1.0.3
            Sequencing Technology :: 454; Illumina
            ##Assembly-Data-END##
FEATURES             Location/Qualifiers
     source          1..3181
                     /organism="Crucivirus-181"
                     /mol_type="genomic DNA"
                     /isolate="120_522_IR1_3296"
                     /isolation_source="Water"
                     /country="Norway"
                     /collection_date=2011
     CDS             1..1041
                     /codon_start=1
                     /product="Putative Replication Associated Protein"
                     /translation="MTKQLTGWDFTLRREKSDKSLYTKDDVILLLAPLCSKYVFQLEK
                     GESGYEHFQGRIRLIKKQIKNTLLKNFKDISLADLPSFTPTSDEVFQTSNFNYVLKSD
                     TRMDGPWSDKDAVREELREPVYVPKQYRIEYDKLYPWQKYIVDNCYQYDTRTINVLYD
                     PAGNVGKSTLSGYLYCNKDAIIVPDIDDSSRIVYSVCDILKSKQIRHTVPIFLDLPRS
                     IDPNELGHFYRSIERIKSGIIYDTRNRYQAWEFDSPCIWVFTNKLPDKGSLSYDRWKI
                     WQIGNDQTLVPYAPLEARKELKQLKPTKKVKINYNYHTDKYHDDNGMDVDFNRLLQDY
                     EITEPFKPRRLV*"
     CDS             1361..2779
                     /codon_start=1
                     /product="Putative CruV Capsid Protein"
                     /translation="MDIDYYQHQMPRAPIKEKIVYVEKPAKKVVKKLVKRKPKAQVAV
                     TSQTERDMRKSNEPTAFGSIIKSAGRALGGYMGGPGGGVVGGYIADKLSKLLGQGTYV
                     MSDTNSLSQNSIAKPSYGDVTHMRYNQDGTMRIRHREFIQDIVGSTAFVNNTFAINPG
                     NTRTFPWIYRIAQQFQTYQMEGLCFEFVSTSADALNSTNTALGAVIMGCQYDSIDRVF
                     QNKQEAENTQGTISGKPSSNMVMCVECRPGSNPLTDLYIVRPNQVAPSDIRFQNLGNF
                     QIMTVGMQAAATIGELWCSYDVILKKPILGSASAIARSTHVSLDSTVTDSSSTSFFFG
                     INPSILTNDLSIVLNPLINRITIPANTFASSQEPVRIVIAYQLTGADTGLTSLAYMDV
                     VSPNTIPFNVYTFNNVQLAVNTLSNGFGPGAQFFQYIALEIVDVSIDTDITLGGAAIF
                     PLNTGVPDPVLSGDMFISLFEA*"
ORIGIN
        1 atgacgaagc aattaactgg atgggatttt actcttagac gtgaaaaatc agacaaatcg
       61 ttatatacta aggatgatgt tattcttcta ttagcacccc tttgctctaa atatgtcttt
      121 caattagaga agggtgaatc cggatatgaa cactttcagg gacggataag gttaattaag
      181 aagcagatca agaatactct tttaaagaat ttcaaagata tatcattagc tgatcttcct
      241 tcattcacac caacatcaga tgaggtattt cagacctcta actttaatta tgtattaaaa
      301 tctgatactc gtatggatgg tccttggtca gacaaggatg ccgtccgaga agaactcaga
      361 gaacctgttt atgttccaaa acagtatcgc attgagtatg acaagcttta tccttggcaa
      421 aaatacatag ttgataattg ttatcaatat gacacaagaa caattaatgt tctatacgat
      481 cctgcaggga atgtaggtaa atccacatta tctgggtatt tatattgtaa caaggatgca
      541 atcattgtcc cagatatcga tgactcatca cgtatagttt attctgtatg tgatattctc
      601 aaatcaaaac aaatacggca cacagttcct atttttttag atctaccacg atcaattgat
      661 cctaatgaat taggtcattt ttatcgttct atcgagagga taaaatccgg tattatttat
      721 gatactcgta acagatatca ggcatgggaa tttgattctc catgtatttg ggtattcaca
      781 aataaactac ctgataaagg atcattatct tatgacagat ggaagatatg gcagatagga
      841 aatgatcaaa ctttagtccc ttatgcgccc ttagaggcgc gtaaagagct aaaacaatta
      901 aaaccaacaa aaaaagtaaa aatcaattac aattatcata ctgataaata tcatgatgat
      961 aacggaatgg atgtagattt caatagactt ttacaggatt atgaaataac agagccattc
     1021 aaaccaagac gtctcgtcta aatcaaaaac attacaccaa aacatcacat acgcatgttc
     1081 gtgggcttta acgcctgcga attttggtcg aggccccgcc cgagcgccaa aaccttcggc
     1141 atgaaccgtc actttagtcc aattacttca attagtactg ctcccggcga gggaatacca
     1201 ggcgaaagca atagaaaata gttatactgg attggggtga tcgggagcca tcgggagggg
     1261 cttgtagcgc tccggtcctg aaagggaggg gcagtagcgc tccggttcgt tagcggttcg
     1321 gaacccatta tttattttac atatccagta aaaatatttt ctggatatag attattatca
     1381 gcatcaaatg ccccgtgccc caatcaaaga gaaaatcgtt tacgtagaga aaccagcaaa
     1441 gaaagtagtt aaaaaattag ttaaaagaaa accaaaagct caagtagctg ttacatcaca
     1501 aacagaaaga gatatgagaa aatcaaatga accgactgct tttggatcta ttatcaaaag
     1561 tgcaggtcgt gcattaggag gttatatggg tggtcctgga ggaggagttg taggtggata
     1621 tatagcagac aaattatcaa aattattagg gcaaggtaca tatgttatga gtgatactaa
     1681 ttcattatcc caaaacagca tagcaaaacc ttcatatggg gacgtaacac atatgaggta
     1741 taatcaagac ggaacgatga gaataagaca tcgtgagttt atccaagata ttgttggttc
     1801 gacagcgttc gtaaacaata cattcgcaat caatccaggg aatactagaa cgtttccatg
     1861 gatttatcgg attgcacagc aattccaaac ttatcaaatg gaaggcttat gctttgaatt
     1921 tgtaagtaca tcggcagatg ccctcaattc tacgaataca gcattaggtg cagtcattat
     1981 gggatgtcaa tatgattcaa tagatcgagt attccaaaat aaacaagaag cagaaaatac
     2041 acaaggtaca atatcaggaa aaccatcatc aaatatggtt atgtgtgtgg aatgtagacc
     2101 tggttcaaac cctttgacag atctctatat tgtccgtcca aatcaagtag ccccatcaga
     2161 tattcgattc cagaatcttg gtaacttcca aataatgact gtaggtatgc aagctgctgc
     2221 tacgatcgga gaattgtggt gttcttacga tgtcatactt aaaaaaccaa ttttaggatc
     2281 agcttcagct atagcaagaa gcactcatgt atcattggat agtactgtta cggatagttc
     2341 atctactagt ttcttctttg gaataaatcc ttcaatacta acaaatgatt tgagcatagt
     2401 attaaatcca ctaattaata gaattacaat ccctgcaaac acttttgcat catcacaaga
     2461 acctgtcaga attgttatag catatcaatt gactggtgca gataccggcc ttacatcttt
     2521 agcatatatg gacgtagtat ctccaaatac aatacctttt aatgtgtata cttttaacaa
     2581 tgttcaatta gcggttaata ctttatcaaa cgggtttggc ccgggtgcac aattcttcca
     2641 gtatattgca ttggaaatag tagatgtctc tatagataca gatatcacat taggaggagc
     2701 tgcaatcttc ccattaaata cgggagtacc agatccagta ttatccggag acatgtttat
     2761 atcacttttt gaagcttaat agatctacgt ttatgtactc tatccagact tcaaaaatat
     2821 atccatgtct atccagaaat tatccatctc ataattaaca acagacatac tttatccaag
     2881 tatccaggaa ttctagttat aatatatata taaaaatata tactaaaagt gcaactatcg
     2941 gtgttacagg cacgtttacc tgccgccata gttgctttca tcgagctgcc tgatcgaacg
     3001 gctcgatgaa acagtacatt cagcattcat ttatcgatta atgattaatt accacatcaa
     3061 taaatattga aaatacttta tttattgaca tcatattact ttattaatta atattatgta
     3121 catttttatt tatctaattt atccagaaat tattttctct ggataaacat atagaaatca
     3181 a
//
LOCUS       CruV-182                3199 bp    DNA     circular     24-FEB-2020
DEFINITION  Crucivirus-182 isolate SG_167.
ACCESSION   CruV-182
KEYWORDS    .
SOURCE      Crucivirus-182
  ORGANISM  Crucivirus-182
            Unclassified.
REFERENCE   1  (bases 1 to 3199)
  AUTHORS   de la Higuera,I., Kasun,G.W., Torrance,E.L., Pratt,A.A.,
            Maluenda,A., Colombet,J., Bisseux,M., Ravet,V., Dayaram,A.,
            Stainton,D., Kraberger,S., Zawar-Reza,P., Goldstien,S., Briskie,J.,
            White,R., Taylor,H., Gomez,C., Ainley,D.G., Harding,J.S.,
            Fontenele,R.S., Schreck,J., Ribeiro,S.G., Oswald,S.A., Arnold,J.M.,
            Varsani,A., Enault,F. and Stedman,K.M.
  TITLE     Direct Submission
  JOURNAL   Submitted (24-FEB-2020) Biology, Portland State University, 1719 SW
            10th Ave, Portland, Oregon 97201, USA
COMMENT     Bankit Comment: ALT EMAIL:ide@pdx.edu
            Bankit Comment: TOTAL # OF SEQS:451
            Bankit Comment: GAP: est. length - alignment

            ##Assembly-Data-START##
            Assembly Method       :: MetaSPAdes v. 3.12; IDBA-UD v. 1.1.1;
                                     MEGAHIT v. 1.0.3
            Sequencing Technology :: 454; Illumina
            ##Assembly-Data-END##
FEATURES             Location/Qualifiers
     source          1..3199
                     /organism="Crucivirus-182"
                     /mol_type="genomic DNA"
                     /isolate="SG_167"
                     /isolation_source="Sediments"
                     /country="New Zealand"
                     /collection_date=2012
     misc_feature    join(3186..3199,1..20)
                     /note="stem-loop"
     misc_feature    1..9
                     /note="nonanucleotide"
     CDS             208..1194
                     /codon_start=1
                     /product="Putative Replication Associated Protein"
                     /translation="MSISKSKGGSKYFVFTSFNEDNYEIFKHLDLSQKGIRYICYQLE
                     ISKDNNKHLQGYIEFKGNKTSLNSIKKLLNDNTIHLEQRRGTQQQAIDYCKKSETSIK
                     PFFESGEPMPQRQGKRTDFTNLRDKVKNGATLSSIIDTDPGMFYKYAKHIKEHINIVK
                     ASKIKTYEKPAVYILYGDTQTGKTKYVYDHHKLEDIYSLPKGNQNNVWFNNYTNEPIL
                     LLDDFYGWIKYSYLLNLTDGYKLQLETKGGYCYKKWNTVYITSNKHPDEWYKHIYPNG
                     MEPALKRRITEIMHFKYNEENQKPSEPIYSTIINGVKTYDPPKDESDDEEEG*"
     CDS             1662..2945
                     /codon_start=1
                     /product="Putative CruV Capsid Protein"
                     /translation="MYKRYSKKQIAPAIYVRKRAPAKKKTYKKKSSWSAPQTFGNVGS
                     EIGKYVPMPYGSQIGRAVGTAGGYLYRKITGRGDYLLQNGGNPNITKPIPRFSSDNSI
                     RIKHKEFISNISSTTTFNNITYKINPGNQRLFPWMSMLSQCYEKYRINSMVIYYRSLS
                     GDSMSSSNVNLGKILLGCDYNASDQPWINADQFYGSMHSNSGKPSADLMLAIECKNSP
                     ELFIRTGGLKSGEDRKSYDFGTLQIGTDSSQSANEIGECFVSYDISLMSSVNNGGVGL
                     NVKTAKAQLSNCTTTRWFGDLTTWEHNDIDGLTISTDGNYLVFPAEMSSGTFQLIYTM
                     KGSTPATLALPTFDATSGVIGKTIFDDDTDQYLSNAGDSSTRFILTTIFTITDKNAAF
                     GMLTTTGTLPGGSFQTADLQVIKLNHADSLKASEY*"
ORIGIN
        1 tagtattacc tacaactttt atagaaaaac aacaaacaaa caaaaatatt ataccattcc
       61 atgtataaag ttattttact tatttcaaaa ttctattctg gattttttaa tgagatttat
      121 aaatataaat ctgttaatta attaattttt aaaaaattct attttgtatt atataataaa
      181 cacaaaataa taatatcata tattaaaatg tcaatttcaa aatctaaagg aggctctaaa
      241 tactttgttt ttacttcatt taatgaagat aattatgaga tatttaagca cttagattta
      301 tcccaaaaag gtattcgtta tatttgttat caattagaga tatctaaaga taataataaa
      361 caccttcaag gatatataga atttaaagga aataagactt ctcttaattc aattaagaag
      421 ttattaaatg ataatactat tcatttagaa cagcgaagag gaactcagca acaagctata
      481 gattactgca agaaatcaga aacatcaata aagccatttt ttgagagcgg ggagcctatg
      541 cctcaacgtc aagggaaacg tacagatttt acaaatctta gagacaaagt aaaaaatggg
      601 gcaactttaa gttctattat tgatacagac cctggcatgt tttataagta tgctaagcat
      661 attaaagaac atattaacat agttaaagct tctaaaatta agacttatga aaaaccagcc
      721 gtatatattt tatatggcga tacacaaaca ggtaaaacaa aatatgttta tgaccatcat
      781 aaacttgaag acatatattc acttcctaaa ggtaatcaaa ataacgtatg gtttaataac
      841 tatactaatg aacctatttt actcctggac gatttttacg gatggataaa atattcttat
      901 cttcttaatt taaccgatgg ttataaatta caattagaaa ctaaaggggg ttattgttat
      961 aaaaaatgga acactgtata tataacatct aataagcatc ccgatgaatg gtataaacat
     1021 atatatccta atggaatgga accagcactt aaaaggcgaa taactgagat aatgcatttt
     1081 aagtataatg aagaaaatca aaaaccttca gaacctattt atagtactat aataaatgga
     1141 gtaaaaactt acgaccctcc taaagatgaa tcagacgatg aagaagaggg ataaaaaata
     1201 ttcattattt ttttttatat ttttccgtaa ataatactta catatattta ttttacacat
     1261 tttatttttt gtaagggtcc cccccttacc taataaaaaa aactatttaa atcatctatt
     1321 taatacatct aaaaatttat taaggctaat accttaccta taattttttt acttatttat
     1381 tttacacatt ttattttttg taagggtccc ccccttacct aataaaaaaa actatttaaa
     1441 tcatctattt aatacattta ctaaaaaaaa tatttattta tttatttttt atatttttat
     1501 atttttatcc tcaaaaatta actttttaga tattttttta ttttttaatt aattaattat
     1561 tttaaaaaaa atatttattt atttatttat ttatttttta tatttttata ttttttctta
     1621 tatattattt aaaaaatatt atcaacaaaa tcttaacaac aatgtataaa cgatactcaa
     1681 aaaaacaaat tgcacccgcc atttatgttc gtaaaagagc accggcgaaa aagaaaactt
     1741 ataagaaaaa aagcagttgg tctgctccac agacattcgg aaatgtcggt tcagaaatcg
     1801 gtaagtacgt acccatgcct tacggctctc aaatcggccg tgcagtaggt acagccggag
     1861 ggtatttata tcgcaagatc acgggtagag gcgattatct tcttcaaaat ggaggtaatc
     1921 caaatattac aaaaccgatc cctcgatttt caagtgacaa ttcaattcgt atcaaacata
     1981 aagaatttat ttcaaatatt tcaagtacaa caacttttaa taatattact tataaaatca
     2041 atccgggaaa tcaacggctc tttccatgga tgtcaatgct ttctcaatgt tatgaaaaat
     2101 atcgaattaa ctcgatggta atttattacc gttctttaag tggtgactca atgtcatcaa
     2161 gcaacgttaa tttaggtaaa atccttcttg gatgtgatta taacgcatct gatcagccat
     2221 ggataaatgc agaccagttt tatggttcaa tgcatagtaa ctctggtaaa ccatctgccg
     2281 atctgatgtt agcgatcgaa tgcaaaaatt ctcctgaatt atttattaga actggaggtt
     2341 taaaatctgg tgaagaccgc aaaagctatg atttcggaac tttacaaatc ggtactgatt
     2401 cgagtcaaag tgcaaacgag attggtgaat gctttgtttc atatgatata agtcttatgt
     2461 ccagtgtaaa taatggaggc gtaggcctta acgtaaaaac tgcgaaagct cagctttcta
     2521 actgtacaac tactaggtgg tttggtgatt taactacatg ggaacataat gatatagacg
     2581 gattaactat aagcactgat ggtaactatc tcgtttttcc tgctgaaatg tcaagtggca
     2641 ctttccagct catttataca atgaaaggtt caacaccagc tacattggct cttcccacat
     2701 ttgatgcaac aagtggagta atcggaaaga ctatatttga tgacgatact gaccagtatt
     2761 taagcaacgc aggagacagt tcaactcgtt ttatattaac tactatattt acaataactg
     2821 ataaaaatgc agcttttggt atgctcacaa ctactggaac ccttcccggg ggttcttttc
     2881 aaactgctga tttacaagta ataaaattaa accatgctga ctcacttaag gcttctgaat
     2941 attaaacact tttaatatga tatttattaa gactattttt tttgacaaaa aaaagcaaga
     3001 taaaaaaccc atgggataca catcacgaga caaggggggg atacctatac ttaatgttaa
     3061 gcagaagtac cgcccaacgg cagcggttct tcgacttctg cttaacatta agtataggta
     3121 tcaccacctg atggggggct tatataatca agacaaaata tatttaaaaa ttctattcta
     3181 ataaaaaaag ttgtagtca
//
LOCUS       CruV-183                3202 bp    DNA     circular     24-FEB-2020
DEFINITION  Crucivirus-183 isolate AZL_25995.
ACCESSION   CruV-183
KEYWORDS    .
SOURCE      Crucivirus-183
  ORGANISM  Crucivirus-183
            Unclassified.
REFERENCE   1  (bases 1 to 3202)
  AUTHORS   de la Higuera,I., Kasun,G.W., Torrance,E.L., Pratt,A.A.,
            Maluenda,A., Colombet,J., Bisseux,M., Ravet,V., Dayaram,A.,
            Stainton,D., Kraberger,S., Zawar-Reza,P., Goldstien,S., Briskie,J.,
            White,R., Taylor,H., Gomez,C., Ainley,D.G., Harding,J.S.,
            Fontenele,R.S., Schreck,J., Ribeiro,S.G., Oswald,S.A., Arnold,J.M.,
            Varsani,A., Enault,F. and Stedman,K.M.
  TITLE     Direct Submission
  JOURNAL   Submitted (24-FEB-2020) Biology, Portland State University, 1719 SW
            10th Ave, Portland, Oregon 97201, USA
COMMENT     Bankit Comment: ALT EMAIL:ide@pdx.edu
            Bankit Comment: TOTAL # OF SEQS:451
            Bankit Comment: GAP: est. length - alignment

            ##Assembly-Data-START##
            Assembly Method       :: MetaSPAdes v. 3.12; IDBA-UD v. 1.1.1;
                                     MEGAHIT v. 1.0.3
            Sequencing Technology :: 454; Illumina
            ##Assembly-Data-END##
FEATURES             Location/Qualifiers
     source          1..3202
                     /organism="Crucivirus-183"
                     /mol_type="genomic DNA"
                     /isolate="AZL_25995"
                     /isolation_source="Water"
                     /country="USA"
                     /collection_date=2012
     CDS             1..1065
                     /codon_start=1
                     /product="Putative Replication Associated Protein"
                     /translation="MSRIPREPRFRRFRFTINNPTELNTEMCIAAKGFSYLIVGLEIG
                     SETGTFHFQGYAELPEQRTRSTILKMLGASSEEGHPGWAHILPCNASQHSNVVYASKD
                     CDILVEAGEPSSQGARNDIADVRRTILKGGSMEDVCMQATSYQAMRTGELMMKYLKPP
                     SKPRVEPLVTWLWGPSGTGKTHRATHFFPKPGLPDAPFIWSDGKWWDGYDRHKNVIID
                     DLRRGMWPFRYLLRLLDKYPFRVEVKGGTREFVATDIVITAPESPEQMFAYEGEELKQ
                     LLRRIHIVEYVGAREDLTDPPEDQHDLPSLPCPFGTPGGVASEVKCNSEPCWVAPSRD
                     SVDLTSILETAMGGTPIGLW*"
     CDS             complement(1276..2856)
                     /codon_start=1
                     /product="Putative CruV Capsid Protein"
                     /translation="MSSGKQAWLPYDEWKETAAGKAALAKKAAKPSKPKAKKTKARVS
                     RAMDIAKANKGDVLSSNYGARLGAVLGEGAQGLFKAITGYGQYKLALKPKKNSLISMG
                     QSPPVMMNAADGRKIMRHREFLQDIVTGPAGIFTNQSFRINPGDPTTFPWLSTIARCF
                     EEYQIRGMLFEFKSTSGNALTSTNTALGSVIMATQYDSAKNAFTTKSEMENHEYSASA
                     KQSESMMHAIECAPRLSVLPQLYVRSGATPAGTDPRLYDFGLFQIATDGQQGNNVTIG
                     ELWVTYEVEFYKAQLSPASFTSPAADHYRMSGDYDATHALSLSPSKQPGSNAGTEIVD
                     GNILAFDADKSNVDYMVMIYWIGTVAAAGWAGPSVVTTNCEQQQVWNGGTNFSGFAPS
                     AGVVTSMNCSIFYKFHVTAPNATLFFTGAPVFPTGTLAGDIWVIACNPAVVTLDTPME
                     KKLRELEANFERLQRQLTGADHESDEAEKRPPKPQKEPSEMTPEERDAKRKSLLAELT
                     GLMKSVSYPAPSGGSPAM*"
ORIGIN
        1 atgtctcgca taccccgtga accccggttc cgccggttca ggttcactat caacaacccc
       61 accgagctca acacagagat gtgtatcgct gctaagggat tctcctacct catcgttggt
      121 ctcgagatcg gtagtgagac tggaacgttc cacttccaag ggtacgcgga actgccagaa
      181 cagcggacac ggtccactat tctgaagatg ctcggcgctt cctcggagga aggccaccct
      241 gggtgggccc acattctccc gtgtaacgcg tcgcaacact cgaacgtcgt ctacgcatcc
      301 aaggactgcg atatcctcgt cgaggcgggt gaaccctcgt cacagggggc ccgcaacgac
      361 attgcggatg tccgacggac gatcctcaaa ggcggtagca tggaagatgt ctgcatgcaa
      421 gcgacatcct accaagctat gcgcacggga gagcttatga tgaagtatct taagcctccc
      481 tcgaaaccca gggttgaacc cctggtcacc tggctctggg gcccttcggg taccgggaag
      541 acacaccgtg ctacgcactt cttccctaag ccaggcttgc ccgacgcacc attcatatgg
      601 tccgacggca agtggtggga cgggtacgac cgtcacaaga acgtcatcat cgatgacctt
      661 cgccgcggca tgtggccatt ccgctacctc ctacgcctcc tggataaata tccattccgc
      721 gtagaggtta agggcggcac ccgcgagttc gtggcgaccg atatcgttat cactgcccct
      781 gagtcgcccg aacagatgtt cgcgtacgaa ggggaagagc tgaagcagct ccttcggcgt
      841 atccacatcg tggaatacgt cggcgcccgg gaagatctga cagatccccc cgaagatcaa
      901 catgatcttc cttctctgcc ttgcccattc ggcacacctg ggggtgttgc ctcagaagtc
      961 aagtgtaata gtgagccgtg ctgggttgca cccagccgcg actctgttga cttgacttct
     1021 atactcgaaa ccgctatggg cggaacaccc attggccttt ggtgagggcc atggtgttgt
     1081 ggtaccttcg taccaccgac tttttgactc atggtaggga cacccccctt cggggggtgc
     1141 cctagcacca ttcacaaaaa gctcgtgcca cgttaacaca ttgggtgttg tccgggaccg
     1201 catcaatctc gggcctcgcc ctctattgcg tctccgttaa caaccatagc gccacgggct
     1261 atatactctc cctcattaca ttgcgggaga ccctcccgac ggtgcgggat acgacactga
     1321 tttcatcagt ccggtgagct cggcgagcag gctcttgcgc ttcgcgtcgc gctcctcagg
     1381 agtcatctcc gatggctcct tttgaggctt tggaggccgc ttctcagcct cgtcgctctc
     1441 gtggtccgcg ccagtcaact ggcgctgaag acgttcgaaa ttcgcctcaa gttcgcgaag
     1501 tttcttctcc atcggggtgt cgagagtgac caccgctgga ttgcaagcaa tgacccaaat
     1561 gtctcctgca agagtacccg ttgggaacac aggtgcccct gtgaagaaga gggttgcgtt
     1621 aggcgcagtg acatggaact tgtagaagat cgagcagttc attgacgtca cgacgcctgc
     1681 cgaaggtgcg aacccggaga agttcgtacc accgttccag acctgctgct gctcgcaatt
     1741 ggtcgtcacg acactcggac ctgcccaccc tgcggctgcg acagtcccga tccagtagat
     1801 catgaccatg tagtccacat tgctcttgtc cgcgtcgaat gcgaggatgt taccatccac
     1861 gatctccgtt ccggcattac tgccgggttg cttcgatgga ctcaagctca acgcatgggt
     1921 cgcatcatag tcacccgaca tccgatagtg atcggctgcc ggcgacgtaa aggaggccgg
     1981 tgacagttga gccttgtaga actcaacctc gtatgtcacc cacagctctc cgatggtgac
     2041 gttgttgccc tgctgaccgt ctgtcgcgat ctggaacagc ccaaagtcat acaacctcgg
     2101 gtccgttcct gcgggggttg caccactgcg cacgtacagc tgcggcaata ccgacagccg
     2161 aggtgcgcac tcgatggcat gcatcataga ctcgctctgc ttcgcactcg ccgagtactc
     2221 gtgattttcc atctccgact tcgtcgtgaa ggcgttcttc gcactatcgt actgcgtcgc
     2281 catgataact gatccgagcg cggtgttcgt gctcgtcaaa gcgttgcctg atgtgctctt
     2341 gaactcgaag agcatccctc ggatctgata ctcctcgaaa caccgggcga tcgttgatag
     2401 ccacgggaac gtcgttggat ctcctgggtt gatcctgaac gactggttcg tgaagatccc
     2461 tgcgggaccc gtcacgatgt cttgcaagaa ctcgcgatgc cgcatgatct tgcggccgtc
     2521 agctgcgttc atcatcactg gaggcgactg ccccattgag atcaaactgt tcttcttggg
     2581 cttcaaggcc agcttatact ggccatagcc cgtgatggcc ttaaacaggc cctgagctcc
     2641 ttcacctagg actgcgccta ggcgggctcc gtagttcgac gacaagacgt ctcccttgtt
     2701 agctttcgcg atatccatcg ctcgcgaaac tcgtgccttc gttttcttgg ccttcggctt
     2761 ggacggcttc gccgccttct tcgccagcgc ggctttgcct gctgctgtct ccttccactc
     2821 gtcatacgga agccatgctt gcttccctga cgacatcctt cacgggttcg ctctcactca
     2881 ctaatcccag tcggtcctgt gggccggtca cagtcgcgtg acacactggt atcgatatgc
     2941 tgatgccttg ggacccgccc agtaatcagc gtatgtatgg cctcgagaac tcgggccacc
     3001 gaaaataatt catatgtacc cgtggcgata gatgcatatt gcccaatggc atattatcta
     3061 tcgcccgaca cgggtaccat atgccggcga tgcgcgcttc acgcttaccc cttcggctac
     3121 gcctcggtgc acggtctcaa taaccacgct aaccgcatgg ttattttcgc tcgcgggaga
     3181 ggatcgccgg cctcgatata cg
//
LOCUS       CruV-184                3206 bp    DNA     circular     24-FEB-2020
DEFINITION  Crucivirus-184 isolate CHIVOP3_758_1.
ACCESSION   CruV-184
KEYWORDS    .
SOURCE      Crucivirus-184
  ORGANISM  Crucivirus-184
            Unclassified.
REFERENCE   1  (bases 1 to 3206)
  AUTHORS   de la Higuera,I., Kasun,G.W., Torrance,E.L., Pratt,A.A.,
            Maluenda,A., Colombet,J., Bisseux,M., Ravet,V., Dayaram,A.,
            Stainton,D., Kraberger,S., Zawar-Reza,P., Goldstien,S., Briskie,J.,
            White,R., Taylor,H., Gomez,C., Ainley,D.G., Harding,J.S.,
            Fontenele,R.S., Schreck,J., Ribeiro,S.G., Oswald,S.A., Arnold,J.M.,
            Varsani,A., Enault,F. and Stedman,K.M.
  TITLE     Direct Submission
  JOURNAL   Submitted (24-FEB-2020) Biology, Portland State University, 1719 SW
            10th Ave, Portland, Oregon 97201, USA
COMMENT     Bankit Comment: ALT EMAIL:ide@pdx.edu
            Bankit Comment: TOTAL # OF SEQS:451
            Bankit Comment: GAP: est. length - alignment

            ##Assembly-Data-START##
            Assembly Method       :: MetaSPAdes v. 3.12; IDBA-UD v. 1.1.1;
                                     MEGAHIT v. 1.0.3
            Sequencing Technology :: 454; Illumina
            ##Assembly-Data-END##
FEATURES             Location/Qualifiers
     source          1..3206
                     /organism="Crucivirus-184"
                     /mol_type="genomic DNA"
                     /isolate="CHIVOP3_758_1"
                     /isolation_source="Plant"
                     /country="Brazil"
                     /collection_date=2015
     CDS             1..1179
                     /codon_start=1
                     /product="Putative Replication Associated Protein"
                     /translation="MPASNATKSRKWCFTMFFDIDKMGPRGPEAKYFEDWECEYLYVG
                     LESCPTSGRLHYQGYVRFANPRALNGVRSLFTHTITGDNGKQKYPGHWEACKGTEEQN
                     VKYCSKESDLIIEWGQSEADLKPDKNQGKRNDIKAVRQMISEGKGMLEVVSEVNSAQA
                     IKIAETMLKYLEPERSWMPEVYWIYGPTGVGKSKLAFSQCERPWVSGETGRWFEGYDA
                     HEDVIFDDFRGDFCKFHVLLRLLDRYPYRIEVKGASRQFLAKRIFITSYHPPHLVYKD
                     RGNEDLAQLGRRICKIIHLLSDSEARSMPGKKIGLEPSDKELIPELGPEQKVGGNTTE
                     TPLTPPVFQGAPPPTMFYEGPKRTECSKPHADNLYCKDCVNLALDDFFAEDDSEEPDG
                     *"
     misc_feature    985..1008
                     /note="stem-loop"
     misc_feature    990..998
                     /note="nonanucleotide"
     CDS             complement(1235..2743)
                     /codon_start=1
                     /product="Putative CruV Capsid Protein"
                     /translation="MPRAAAEQKAPARKAAVKRTYVRKATVARPAIARPMVRKAASSE
                     YTYDKPGPWGKAGRWLGGALGGAYAGAGGAAVGSKLGSYLHYIGKIFGSGDYVTAAQQ
                     VKGNTLVNSSQVPQFATGKHTMRIQHREFLGDIITSSTPGAFEIQDYPINPGIELTFP
                     WLSNVVGVNYQQYRIRGMVFEFRSMSADALTSTNTALGSVVMATDYDSTDLRFTSKSQ
                     MENTEFGVSCKPSACMIHAIECDRAQTTLTEQYVRFGAPPSGADLRFYDLGRFSIATV
                     GMQGASVNVGELWVSYDIEFFKPIKLPPESDALLAYYKLDLATGSLNNHPVSVVLPGV
                     FDNIGVTYDEPSPLGNIVYLPHTMRAGTVLSVQHQWMGTAASTQSPSISLANGLSFTG
                     GTDLQFLTMPSPAPASSEQIFTEIWIVYDGSGTAANLPSMTFSSCVGPTSPTQGWLLI
                     SEMNAVTGTFTVPRVKPGIRRVSEPETDMVHVVEEEKKTTHPAPPTYTTRKR*"
ORIGIN
        1 atgcctgcaa gcaatgctac aaaatcacga aaatggtgct tcactatgtt ctttgacata
       61 gataaaatgg gtcctcgtgg acctgaagcc aaatatttcg aagactggga atgcgaatat
      121 ttatacgttg gactcgaatc ttgcccaact agtggtcgtc tacactacca aggttatgtt
      181 cgtttcgcca acccgagagc tctcaatggg gttcgctctt tgttcacaca tactatcact
      241 ggtgataatg gtaaacaaaa gtaccctgga cactgggaag cgtgcaaagg taccgaagaa
      301 caaaatgtca aatattgttc taaagaatcc gatcttatca tcgaatgggg acagtctgaa
      361 gctgacctta aacctgataa gaatcaaggt aaacgcaatg atatcaaagc tgtacgccag
      421 atgatcagcg agggtaaagg tatgctcgaa gtcgtctctg aagtcaacag cgcacaagct
      481 ataaaaatcg ctgaaacgat gttgaagtac cttgaacccg aacggtcatg gatgcccgaa
      541 gtttattgga tctatggacc taccggagta ggtaaatcca agctagcatt ctctcaatgt
      601 gaacgaccat gggtgtctgg cgaaactggt cgatggttcg aaggctatga cgctcacgaa
      661 gacgtcatct ttgatgactt tcgcggtgac ttctgtaaat ttcatgtact cctgaggctg
      721 cttgatcgct acccttacag gatcgaagtc aaaggtgcaa gtcgccaatt tctggcgaaa
      781 cgtatattca tcacgtctta tcacccgccg cacttagtat acaaagatcg cggcaatgaa
      841 gatttggcgc aactgggtcg tcgcatatgc aaaatcattc atcttttatc cgattccgag
      901 gcacgaagca tgcccggaaa aaagataggg ctagagccct cggataaaga attgattccg
      961 gaacttggcc cggaacaaaa agtcgggggt aatactacgg agacccccct cacacctccg
     1021 gtgtttcagg gcgctccgcc cccgactatg ttttatgagg ggccaaaaag gacagaatgt
     1081 agcaaacccc atgctgataa cctttattgc aaagattgtg ttaatcttgc tctcgatgat
     1141 ttctttgcag aagatgattc cgaggaacct gacggttaat atttatttgc aataaacaca
     1201 aaaaattttt ttcgcttagc gttccgcttg cactctatcg cttacgcgta gtatacgttg
     1261 gaggtgcagg atgagtcgtc ttcttctcct cctccacaac atgcaccata tcagtttcag
     1321 gctcagatac ccgcctgatc ccaggcttca cccgaggcac cgtaaaggtt cctgtaactg
     1381 cattcatctc tgaaattaac aaccacccct gagtcgggga ggttggccca acgcaagacg
     1441 agaatgtcat agacggaaga ttagcagcag ttccactgcc atcataaaca atccaaatct
     1501 ccgtaaagat ctgctctgaa gaagcaggag ccggactcgg cattgtaaga aactgcaaat
     1561 cagttcctcc agtaaacgaa agcccattcg ctagcgaaat gctaggcgac tgtgtagacg
     1621 ctgcagtccc catccactga tgctggactg aaagaaccgt tccagcccgc attgtatgcg
     1681 gaaggtaaac aatgttacct aatggagacg gctcatcata cgtcacccca atattatcaa
     1741 aaactcctgg cagtacaact gacacaggat gattattgag gctcccagtt gccaaatcca
     1801 acttataata cgcaagaagc gcatctgact caggaggcaa tttaatgggc ttgaagaact
     1861 caatgtcata cgacacccaa agttctccaa cgttcacact agccccttgc atcccaactg
     1921 tagcgataga aaatcgccca agatcataaa atctcaggtc agcacctgac ggtggagcgc
     1981 caaagcgcac atactgctcc gtcaaagtcg tctgagcgcg atcgcactcg atcgcatgaa
     2041 tcatacaagc cgacggcttg caagacactc caaactcagt gttctccatc tgagacttag
     2101 aagtgaaacg aagatcagta ctgtcgtaat ccgtagccat cacgacagat cccaatgcag
     2161 tattcgtaga cgtcaatgca tccgcagaca tcgatcgaaa ctcaaacacc atcccgcgaa
     2221 tgcggtactg ctgataattc actccaacta cgttggagag ccacggaaac gtgagctcaa
     2281 ttccaggatt aataggataa tcctgaatct caaacgcacc tggcgtggaa gatgttataa
     2341 tatcccccag aaactcccga tgctgtatcc gcatcgtatg tttcccagtc gcaaactgag
     2401 gaacctgaga ggaattcaca agtgtattcc ccttcacttg ctgggctgcg gtgacatagt
     2461 cacccgagcc aaatatcttt ccgatataat gcaagtaaga tccgagctta ctacctacgg
     2521 cagcccctcc agctccggca taagctccgc cgagggcacc cccaagccaa cgtccagcct
     2581 ttccccaagg tccaggctta tcatacgtat actcagaaga agcagccttc cgcaccatag
     2641 gccgcgctat cgcaggccgt gctacagtag ctttacgtac atatgtacgc ttcactgcag
     2701 cttttcgcgc aggcgctttc tgctccgcag cagcacgagg catctcgacg ccggtatacc
     2761 ttgcacaaaa tatttcttca aatgaaaatt atttaatttc gcatatccaa aaagatatgc
     2821 aacatcacgc tcatgctgcg cctccacctc ttcgagagtc ggcatcgcaa ttattttata
     2881 caaatataaa aaatgttttc gcaaatactc gcactgaacc gatggagggt actgctatac
     2941 accacggtca tcgctcgtcc agatgatatg attaatgaga atattgcgtg accacgcaat
     3001 aaacgagtaa tattctagtg cacattaatc actaatcggg taggtgctac agcacatcgc
     3061 tgtgggtccg cgccctgtat cactagcgtg cagggccgcg gcccacagct gggtgccgca
     3121 gtactaccct cacacagtgc gggtcggcac ccatatattt attttcgttt tagaattttg
     3181 tgcaggcaca tcatactgtt ccagcc
//
LOCUS       CruV-185                3212 bp    DNA     circular     24-FEB-2020
DEFINITION  Crucivirus-185 isolate 120_1486_IR2_3297.
ACCESSION   CruV-185
KEYWORDS    .
SOURCE      Crucivirus-185
  ORGANISM  Crucivirus-185
            Unclassified.
REFERENCE   1  (bases 1 to 3212)
  AUTHORS   de la Higuera,I., Kasun,G.W., Torrance,E.L., Pratt,A.A.,
            Maluenda,A., Colombet,J., Bisseux,M., Ravet,V., Dayaram,A.,
            Stainton,D., Kraberger,S., Zawar-Reza,P., Goldstien,S., Briskie,J.,
            White,R., Taylor,H., Gomez,C., Ainley,D.G., Harding,J.S.,
            Fontenele,R.S., Schreck,J., Ribeiro,S.G., Oswald,S.A., Arnold,J.M.,
            Varsani,A., Enault,F. and Stedman,K.M.
  TITLE     Direct Submission
  JOURNAL   Submitted (24-FEB-2020) Biology, Portland State University, 1719 SW
            10th Ave, Portland, Oregon 97201, USA
COMMENT     Bankit Comment: ALT EMAIL:ide@pdx.edu
            Bankit Comment: TOTAL # OF SEQS:451
            Bankit Comment: GAP: est. length - alignment

            ##Assembly-Data-START##
            Assembly Method       :: MetaSPAdes v. 3.12; IDBA-UD v. 1.1.1;
                                     MEGAHIT v. 1.0.3
            Sequencing Technology :: 454; Illumina
            ##Assembly-Data-END##
FEATURES             Location/Qualifiers
     source          1..3212
                     /organism="Crucivirus-185"
                     /mol_type="genomic DNA"
                     /isolate="120_1486_IR2_3297"
                     /isolation_source="Water"
                     /country="Norway"
                     /collection_date=2011
     CDS             1..1107
                     /codon_start=1
                     /product="Putative Replication Associated Protein"
                     /translation="MPPGMVGGTPGCMEQVQQQPTSIATMPVIPGGEGEDVVMKKGAV
                     GGSPYDCKCRAYQLTVFDGSGVKSGNYLTVMEYLRGLKLLDYLITCWEVCPKTLKEHW
                     HIYCHFRNSVKLCQKKCLYSHLEVVRGSVDDNIHYIKKTGKHSDEWNRDPQHHLDLVE
                     WGEIPTDRGGILSGTALMQMSVRETIEYDPRCHSAYLKARNLLLNPVSIDIDDWAKEV
                     KVYFIQGPSGIGKTQRAKELVRDTYPVGARNVDIIKYENGFWSGVGGGANIAVYDEWR
                     SNQMKPSEFINLIDYNKQNMNIKGGSTRNNYELIIITSVEWFDEIYINVGGEPRLQWQ
                     RRIEVIDMYGDTPRREKKPLTGNETEFNGVKYGY*"
     CDS             complement(1500..3155)
                     /codon_start=1
                     /product="Putative CruV Capsid Protein"
                     /translation="MVPSTGVGFTIIIVFDRTIEIHRIDTRYSITRGSMLFRFAKIFL
                     KKKMVGEEDVPPSLLFFLFFFIFSLFRKREEHAMSAIPFASATRVPKDVWNQMTPAER
                     SALRASLGLTSRAPAKRTYKRKAGAKKTYTRRAPRGRRIAYRNTMTGYGDYTDDLAGL
                     AGPLLKHGLPLVIKGLTGFGDYNVEQNSMIPKLGGDPPIIRNTNTGGFILRHREYVSD
                     VYAQSSFTNNVLPINPGLLSTFPLASQVADTFEQYEIRGMVMEFKSMSSDAVLSTSAS
                     SALGTVIFATQYNALDDPFEDKRTMENYEFANSTKPSCSMLHPIECKRSQTPLDLLYV
                     RTGPVTTGDLRMYDLGNFNYAVQGCQNAAPGEVIGELWISFELEFFKPRLLPLGGSST
                     YSDHYSLLGNSLIAVGNPFYTQGGLDLQAGSNLGTYIQVAALAPYAARSIVFPPQITD
                     GIFKIDYYMVGSTASANSPAVGSTTLVNCTAHPYWGGGTVGSLNVDPEPANTTKNFHF
                     SQVININQVLPGIRAGINFDMTGVMPTGNQIFDLVITLLQPSA*"
ORIGIN
        1 atgccgccgg gcatggtagg tggtactcct gggtgtatgg aacaagtcca acagcaacca
       61 acttcaatag caactatgcc agtcattcct gggggagagg gggaagacgt tgttatgaag
      121 aagggagccg ttggaggaag tccatacgac tgcaagtgta gggcatacca gttaactgta
      181 tttgatggaa gtggtgtaaa gagtggaaac tacctaacag tgatggagta cctaagagga
      241 ctaaagctct tagactacct catcacatgt tgggaggtat gccctaaaac cttgaaggaa
      301 cactggcaca tctactgcca cttcagaaac agtgtgaagc tatgtcagaa gaagtgcctg
      361 tacagccatc ttgaagtagt aaggggaagt gttgatgaca acatccacta catcaagaaa
      421 actggcaaac actcagatga gtggaatagg gatcctcaac accatctaga tcttgtagaa
      481 tggggtgaga tccctactga taggggtggt atcctaagtg gtactgcctt aatgcagatg
      541 agtgtcaggg agacgataga gtacgacccc agatgtcact cagcctacct taaggccagg
      601 aacctactcc ttaacccagt cagtatagat attgatgatt gggctaagga agtgaaggtg
      661 tacttcatcc aaggaccatc tggtattggt aaaacgcaac gtgctaagga actggttaga
      721 gatacatacc cagtaggtgc cagaaacgtt gacattatca aatacgagaa tgggttttgg
      781 agtggtgtag ggggaggtgc taacatagct gtttacgacg aatggcgtag taaccagatg
      841 aagccttctg agttcattaa cctgattgac tacaataagc agaacatgaa cattaaggga
      901 ggtagtacac gcaacaacta tgaactcatc atcatcacaa gtgtggaatg gtttgatgag
      961 atttacatca atgttggtgg ggagcctaga cttcaatggc aacgcagaat tgaagtcata
     1021 gacatgtatg gagacacgcc cagacgtgag aaaaagcctc taactgggaa tgagaccgaa
     1081 tttaacggtg tgaaatacgg ctattagagg cctgcaggtt tgtgacttgc atgaaagaac
     1141 gctaggtcac gcaccttaat aaaaatacta gaactagtat tctaagaaga cttgtgtaag
     1201 tctatacctt gagatagaaa agcatgatgt atccttgaaa ggaattcaga ctgttgagtc
     1261 tgttcaagat gtacaccatg tagctcatta gtaatgcgct caagagctaa gttaagttta
     1321 actaactctt cctgaacgtc tgccattgca tcctccagat cgtcaatttt ctgacaaatt
     1381 gcggtgttca ttttatacga cgagaatcgt attaaaaata agttggccat tgtggtcgtg
     1441 ggcggccccc gccgcccgtt gctatgatcg atcgatcata attatgactc attggtcatt
     1501 taggcagatg gttgtagaag agtgattaca agatcaaaga tctggttacc tgtgggcata
     1561 actccagtca tatcgaagtt aataccagca cgaataccag gtagaacctg atttatgtta
     1621 atcacctgag agaagtgaaa attcttggtt gtgttagcag gttcaggatc aacattgagt
     1681 gatccaacag tgccaccacc ccaataagga tgtgcagtac agttcacaag tgtggttgaa
     1741 ccaactgcag gagaattggc tgaagcagtg cttcctacca tgtagtaatc tatcttgaag
     1801 atcccgtctg tgatctgagg tggaaataca atcgagcgag ctgcataagg agctagggca
     1861 gctacttgga tgtatgttcc aagattactt ccggcttgaa gatctaatcc accctgagta
     1921 tagaatggat taccaactgc gatcaaactg ttgcccaaca acgagtagtg atcggagtat
     1981 gtgctagatc caccaagtgg taggagtcta ggcttgaaga attcaagctc gaatgagatc
     2041 cataactcac ctataacctc acctggggct gcattttgac atccctggac tgcatagttg
     2101 aagttaccta gatcatacat tcgaagatca ccagtggtaa ctggccctgt tctcacgtag
     2161 agaagatcaa gaggtgtctg acttctctta cattcgattg gatgcagcat actgcaactg
     2221 ggcttagtag aattagcaaa ctcgtaattc tccatagtac gcttgtcttc aaagggatca
     2281 tccaaggcgt tgtactgagt tgcaaagata accgtaccta atgcagatga agcacttgta
     2341 gaaagaactg catctgaaga catggactta aactccatta ccattcccct gatctcatat
     2401 tgctcaaagg tatccgcaac ctgagatgca agaggaaagg tactgagaag tcctgggtta
     2461 atcggaagca cgttgtttgt gaaagacgac tgagcgtaca catcactaac atactcccga
     2521 tgacggagga taaacccacc agtattcgta ttacgaataa tgggaggatc acctccaagc
     2581 ttagggatca tggagttctg ctctacattg taatcaccga atccagtgag tcccttaata
     2641 accaatggta aaccatgttt aagaagagga ccagcaagac cggcaagatc gtcggtgtag
     2701 tctccgtagc cagtcattgt attgcgataa gcaatccttc tgcctctggg ggctcttctg
     2761 gtataagtct tcttagcacc tgcctttctc ttgtaagtcc tcttagcagg agctctagaa
     2821 gtaagaccaa gagaagctct caaggctgag cgctcagctg gggtcatctg gttccagaca
     2881 tccttaggaa cacgcgttgc agaagcaaat ggaatagcag acattgcgtg ctcttcccgt
     2941 tttctaaaaa gagaaaaaat aaaaaaaaat aaaaaaaaga ggagggaagg aggaacgtcc
     3001 tcctcaccca ccatcttttt ttttaaaaaa attttcgcga agcgaaaaag catcgaccct
     3061 cgggtaatac tatacctcgt gtcgattcta tggatttcaa tcgtgcgatc gaaaactatg
     3121 atgatggtaa aacctacgcc ggtagatgga accatacaac caagcaaatg gaatggtatg
     3181 taccagaaca accagccagg agaagaacac gc
//
LOCUS       CruV-186                3222 bp    DNA     circular     24-FEB-2020
DEFINITION  Crucivirus-186 isolate 120_173_SvL2_3298.
ACCESSION   CruV-186
KEYWORDS    .
SOURCE      Crucivirus-186
  ORGANISM  Crucivirus-186
            Unclassified.
REFERENCE   1  (bases 1 to 3222)
  AUTHORS   de la Higuera,I., Kasun,G.W., Torrance,E.L., Pratt,A.A.,
            Maluenda,A., Colombet,J., Bisseux,M., Ravet,V., Dayaram,A.,
            Stainton,D., Kraberger,S., Zawar-Reza,P., Goldstien,S., Briskie,J.,
            White,R., Taylor,H., Gomez,C., Ainley,D.G., Harding,J.S.,
            Fontenele,R.S., Schreck,J., Ribeiro,S.G., Oswald,S.A., Arnold,J.M.,
            Varsani,A., Enault,F. and Stedman,K.M.
  TITLE     Direct Submission
  JOURNAL   Submitted (24-FEB-2020) Biology, Portland State University, 1719 SW
            10th Ave, Portland, Oregon 97201, USA
COMMENT     Bankit Comment: ALT EMAIL:ide@pdx.edu
            Bankit Comment: TOTAL # OF SEQS:451
            Bankit Comment: GAP: est. length - alignment

            ##Assembly-Data-START##
            Assembly Method       :: MetaSPAdes v. 3.12; IDBA-UD v. 1.1.1;
                                     MEGAHIT v. 1.0.3
            Sequencing Technology :: 454; Illumina
            ##Assembly-Data-END##
FEATURES             Location/Qualifiers
     source          1..3222
                     /organism="Crucivirus-186"
                     /mol_type="genomic DNA"
                     /isolate="120_173_SvL2_3298"
                     /isolation_source="Water"
                     /country="Norway"
                     /collection_date=2012
     CDS             complement(1..1035)
                     /codon_start=1
                     /product="Putative Replication Associated Protein"
                     /translation="MSALETKDIKPSAAEDKKLKFTKARFCCFTAWDEKRPPTWRPSE
                     MDYLVYQAEICPETKKFHWQGYVEFKQDRMAKTVKKLLETGDATNIQKRLGTGLQASE
                     YCKKAETARSPYLHVEHGTLTNGGSTKSGSRTDLISAEHNIIKFAKAGKSFEEVVELV
                     PGIMLKYGRNFRELYNMNYEVKLIMPVEIKLYEWQTAIIKMIEIGYIKRQIIWIWSHN
                     SGTGKSTLIDYLVSTLGSKQVLTGTFKEADLMYAFNGQKVITFNIPRQQKLHETHIAI
                     LEKVSDGGCILSTKYESKPKIMQSVIIVFANIRAPHDLMPKRCIEINVDPKGYVHPMD
                     PLTEYMIRDD*"
     misc_feature    119..142
                     /note="stem-loop"
     misc_feature    126..134
                     /note="nonanucleotide"
     CDS             1418..3001
                     /codon_start=1
                     /product="Putative CruV Capsid Protein"
                     /translation="MLSKSGYAKLSKADKAKLKLSLARKSVRGSKGMGRVLKGHGDYW
                     TGPGSKRAESMFDSAGAKAGESIFGTPGKYIGGAMHGLFKRLTGKGDYKVSSNTIMMG
                     NDPPLFAKSGSRGMVVQHREYITDIITSATAGEFKIQTFPINAGQPSTFPWLYAIGNQ
                     YEQFVVRGMVWEFKSMSSDALNSVNTALGSVIMATQYNSVASAFTNKSQMENHEFSTS
                     FKSSIDGVHAIECARGETTVSALYTRAGPVPATADKRLYDLGETSIATVGFQGTSVNI
                     GELWVTYDIEMLKPQLNTDGNVTPIPDHYSIASGIPTSSAYFGVTNQLVSGSTGTTLY
                     SEADGYSQGMLQLPLSTNNQTYLVEYWVQSLTGPSPLSIVLAPVVNGVGVSTGGPWFL
                     TNTSPFIRQYTGAVSDVQFCACTVYVPANISCQYSFSPNATLPVNPADFDLIITRVPQ
                     ATGFVPPSFLSRKVITEVKEEKKSEEKDDDDFLEFLRYKKLMSMKREEESKDYHVVSE
                     PPTPSKSDPSSLMKVASRK*"
ORIGIN
        1 ctaatcatca cggatcatat attccgtgag tgggtccata ggatgtacgt aacctttagg
       61 atctacgtta atttcgatgc atcgcttcgg cattaaatca tgtggcgcac gtatgttagc
      121 aaatacaata ataacgcttt gcattatttt gggtttggat tcgtattttg tgcttaaaat
      181 acatccacca tcactaacct tttctagaat agcaatatga gtttcatgaa gtttttgctg
      241 acgaggaatg ttaaaggtta ttactttttg gccattaaat gcatacatta aatcggcttc
      301 tttaaaagta ccggttaaaa cttgttttga accgagtgta ctaacgaggt aatcaataag
      361 tgttgattta ccagtgccac tattgtggga ccaaatccat atgatttgtc gtttaatata
      421 tccaatttct atcattttta tgattgcagt ttgccattca taaagtttga tttcaacagg
      481 cattataagt tttacttcat aattcatgtt gtaaagttcc ctgaagttac ggccatactt
      541 cagcataatt ccaggaacta attcaacaac ttcctcaaag gatttgccag ctttggcaaa
      601 tttgatgata ttatgttctg ctgaaattag atcagttcgt gaaccagatt tggtcgaacc
      661 accattggta agggtgccat gttctacgtg taaatatggt gatcgtgctg tttcagcttt
      721 tttacaatat tcggatgctt gcaatccggt tcccaaacgt ttttggatat tggtggcatc
      781 accagtttct aaaagttttt tgacagtttt ggccatacga tcttgtttga attctacata
      841 gccctgccag tgaaactttt tagtttcagg acagatttcg gcttggtaga ccaaataatc
      901 catctctgat gggcgccatg ttggcggtcg tttttcatcc cacgcggtaa aacagcagaa
      961 acgagcttta gtgaatttca attttttatc ctcagcggcg gatggtttga tatctttggt
     1021 ttctagggcg gacatgtata tgtacctccc gccaaagttt aaggtaaata aatattaaat
     1081 atttatgagg ttgaggttaa gaaatttaat taaataattc caaatttcta ttattaccct
     1141 caacctcccg caaggtgcga attaattaat taattaagaa gcaggttttc aaggcgtagt
     1201 ccgttaagaa ataccgaggt aatgcccctt tggggctaca gaggtgatta ttcaggtgca
     1261 taccgataga ataatcgcgg atttttttga gcgaagcgaa aaaaaaccga ggcgaagccg
     1321 agggcgcaac cccctggggg gtatgcatcc tttgttatct ttggcttggc cacaataaat
     1381 atttatttat taaatattta tttaggagtc atatattatg ctctctaaaa gtggatatgc
     1441 aaagttaagt aaggctgata aagcgaaact taaactatct ttggcccgaa aaagtgttcg
     1501 tggctcaaaa ggcatgggtc gtgttctaaa gggacacgga gattactgga ctggtccagg
     1561 atctaaacgt gctgaatcca tgtttgattc cgcaggcgct aaagccggtg agtccatttt
     1621 tggtactcca ggaaaataca ttggtggtgc tatgcatggt ctgttcaagc gattgacagg
     1681 caagggtgac tataaagtct cctccaacac gattatgatg ggcaatgacc caccattgtt
     1741 tgcaaaatct ggctctcgag gcatggttgt gcaacatcgt gaatatatca ctgatatcat
     1801 tacatctgca acagctggag aatttaagat ccagactttc ccgattaatg caggtcaacc
     1861 ttcgaccttt ccatggttat atgccattgg aaatcagtat gaacaatttg ttgttcgtgg
     1921 catggtgtgg gaatttaaga gtatgtcttc agacgcactc aattccgtaa acactgcctt
     1981 gggttctgtt attatggcta cgcagtacaa ctctgttgca tcggccttta ctaacaagtc
     2041 tcaaatggag aatcatgagt tctccactag tttcaagagt tcgatcgatg gagttcacgc
     2101 aatcgagtgt gctcgtggtg aaactactgt tagtgcactt tatacgcgtg ctgggcctgt
     2161 accagcaacc gcagataaac gtttgtacga tcttggtgaa accagtattg caactgtagg
     2221 atttcaaggt acctcagtaa atatcggtga actttgggtt acgtatgata ttgagatgct
     2281 taagccgcaa ttgaatactg acggtaatgt cacacccatt ccggatcatt atagcattgc
     2341 atccggtatt ccaacttcat cggcgtattt cggtgttaca aatcagttag tatctggttc
     2401 tactggtact actttgtatt ccgaagctga tggttatagt cagggtatgc tgcagttacc
     2461 tttgagtaca aataatcaaa cttatttggt cgaatattgg gtacaaagtt taaccggacc
     2521 ttcacctctt agtattgtat tggcccctgt tgtaaacggt gtaggcgttt caactggtgg
     2581 tccatggttt ttaaccaata caagtccatt tattcgacaa tatacaggag cagtttctga
     2641 cgtacaattt tgcgcatgca cagtttatgt tcctgcgaat atttcttgtc agtattcgtt
     2701 tagtccaaac gcaacgttac cagtaaatcc ggctgatttt gatttgatta tcacgcgcgt
     2761 accgcaagcg actgggtttg ttccaccatc tttcttatct cgtaaagtta ttactgaggt
     2821 aaaggaagag aagaaatctg aagagaagga cgatgatgat ttcttagagt ttttgcgtta
     2881 caaaaaactt atgagtatga aacgtgagga ggaatctaag gactaccatg tagtcagtga
     2941 gcctccgacg ccttctaaga gtgacccgtc atctttgatg aaagtggcat ctcgaaagta
     3001 ggtactcaaa attctaaaaa agagaattta ttttttgctg gcacatatta ttaggccgac
     3061 taatcatata tgcgaccagg actcgtaagt catcgcacga gtatatgcga aaccgagctt
     3121 attttttgga gcgtagcgac aaaaaataaa ccgcgagctt tcgcatacat tagggtgatg
     3181 acacgaaacc ctgatcactt aagtgatcag ccaccctaaa at
//
LOCUS       CruV-187                3227 bp    DNA     circular     24-FEB-2020
DEFINITION  Crucivirus-187 isolate 001653_virusbaton.
ACCESSION   CruV-187
KEYWORDS    .
SOURCE      Crucivirus-187
  ORGANISM  Crucivirus-187
            Unclassified.
REFERENCE   1  (bases 1 to 3227)
  AUTHORS   de la Higuera,I., Kasun,G.W., Torrance,E.L., Pratt,A.A.,
            Maluenda,A., Colombet,J., Bisseux,M., Ravet,V., Dayaram,A.,
            Stainton,D., Kraberger,S., Zawar-Reza,P., Goldstien,S., Briskie,J.,
            White,R., Taylor,H., Gomez,C., Ainley,D.G., Harding,J.S.,
            Fontenele,R.S., Schreck,J., Ribeiro,S.G., Oswald,S.A., Arnold,J.M.,
            Varsani,A., Enault,F. and Stedman,K.M.
  TITLE     Direct Submission
  JOURNAL   Submitted (24-FEB-2020) Biology, Portland State University, 1719 SW
            10th Ave, Portland, Oregon 97201, USA
COMMENT     Bankit Comment: ALT EMAIL:ide@pdx.edu
            Bankit Comment: TOTAL # OF SEQS:451
            Bankit Comment: GAP: est. length - alignment

            ##Assembly-Data-START##
            Assembly Method       :: MetaSPAdes v. 3.12; IDBA-UD v. 1.1.1;
                                     MEGAHIT v. 1.0.3
            Sequencing Technology :: 454; Illumina
            ##Assembly-Data-END##
FEATURES             Location/Qualifiers
     source          1..3227
                     /organism="Crucivirus-187"
                     /mol_type="genomic DNA"
                     /isolate="001653_virusbaton"
                     /isolation_source="Water"
                     /country="France"
                     /collection_date=2013
     CDS             1..954
                     /codon_start=1
                     /product="Putative Replication Associated Protein"
                     /translation="MPKASASKARARRWCFTIHDRDTAVKMYHNIDFQDKLQKRFSYV
                     CFQLESAPSTTSLHVQGYLESKSLTSMKKIKEDFECDSMHLEASNGDAEQNKEYCSKS
                     RTKAGEFMEWGTPGPGQGKRTDLANMYIDVQAGMPINELIATYPANWFRYGRNIMQLY
                     SMRQPDIRPKLRIHYYYGPPRTGKSLLVRELLKETQGVFYANDTKEGWFDGYTGQSTV
                     VFDEFRGKFPINDMLKILDHYPHRQQLKGAFFPLSVTDVYFTSNFAPESFYTGDEQPQ
                     WLSRIRGPRFELHEVLCAAQVQEKLARLSNLYQATVPVPAV*"
     CDS             complement(1375..2793)
                     /codon_start=1
                     /product="Putative CruV Capsid Protein"
                     /translation="MPIYRRYGRRGRGRRTSSRRAPVKRRRTGGSRRPGYFSTMARRF
                     GGAGASTLGRMAAERAAAGVLPAVGAYLKDAAYDAAASLIPGGGVIRRVYKGVKRYWD
                     GNADMMDEEDKDPVGPIARVDGPGAYTIRKNSLMSGSQTPAMHSVEDGVRIRHREYVQ
                     DVKTATAFTNQTFVMNPGLAEVFPWLSAIAQNFEEYEWLGLMVHFKSTCSDAIASSTN
                     LALGTMILAAEYNVNQAAYVNKQQMENSYFAMSSKPSCDMLMPVECDPKDNPLAVHYI
                     RTGAVPSGQDARMYDMCNVQLASVGAQAAFNTGELWITYDVILRKPQLDSGLALAAAS
                     AHYSLVAPAVTTAYFGTSQVAKYDSIGLTVTGTAFSFPIGQAGVYLVSWSARGASTAS
                     LVAPTTTLTNCTALQIWANDAATSMNTLGSPTSTIVQLEFVISITDPNQLCTVTLSAG
                     TLCGTPTYGDLIVTQINGNLNS*"
ORIGIN
        1 atgcccaagg cttccgccag caaggcacga gctcgtcgat ggtgcttcac catccacgat
       61 cgagacacgg cagtcaagat gtaccacaac attgacttcc aagacaagct gcaaaaaaga
      121 ttcagctacg tctgttttca gcttgaaagt gcgccttcga caacttctct tcatgtccag
      181 ggctacctgg aatctaaatc tctaacgagc atgaagaaga tcaaagaaga cttcgagtgt
      241 gactcaatgc atctcgaagc gtcgaacggc gatgctgaac agaacaagga gtactgcagc
      301 aaaagcagga ccaaggctgg agaattcatg gaatggggta cgcctggtcc tggccaggga
      361 aagcggacgg atctagccaa catgtacatc gatgtacaag ctggcatgcc cattaacgag
      421 ctgatcgcga cttacccggc caactggttt cggtatggtc gcaacatcat gcagctctac
      481 tcgatgcgtc agcccgacat ccgtccaaaa ctgcgcatcc actactatta tggtccgccg
      541 cgtactggaa aatcgttact cgtccgagaa ctactcaaag aaacccaagg tgtcttctat
      601 gccaacgaca ccaaagaagg atggttcgac gggtataccg gacaatccac cgtcgtcttt
      661 gacgagttcc gtggcaaatt tccgattaac gacatgctca agatactcga ccattacccc
      721 catcgacaac agctcaaggg agcctttttc cccctgtccg tcaccgatgt ctacttcacc
      781 agtaacttcg ccccagagtc cttctacaca ggagacgaac aaccccagtg gctctcacgc
      841 atccggggcc cccgcttcga actacacgaa gtcctctgtg cagcacaagt gcaagaaaaa
      901 ctggcgagat tgagcaatct ttaccaagct acggtaccgg tcccagcagt gtgataaata
      961 aatcagcagc actggtcact gtcggagtac cagtaaacgt accgtatacc gtcatacttt
     1021 gacctggagc agtaaccttc acgatattat cgatgaaaca gggaaacaga agctgtccca
     1081 ctgttacggt attccaagcg tcttcctcat agaggttgta cgcagaacaa ccaccgttgt
     1141 aatccagagt ccagttgaca gagtcagact cggcctcaga gttcgtaagg atcagagaaa
     1201 tcctatacaa acccgcttgg taaaaagtaa tggaggatgg aaacacagcc acgatagaat
     1261 cgagctgtgc aatatccggg tcagacaact gtgagttgcc taaaggagcc cctttgaaat
     1321 cggatattgt ccaatgactt gttccagctt ggagagaggg cgtctcctcc ggggttacga
     1381 gtttaaattg ccgttgatct gagtcacgat caaatcacca tacgtcggcg ttccacacag
     1441 agttccggcc gacaacgtaa ccgtacagag ctggttcgga tccgtgatag aaatcacaaa
     1501 ctccaactgc acaatcgtgc tagtcggcga tccgagtgtg ttcatggagg tagcagcatc
     1561 attggcccaa atctgcaacg ccgtgcagtt ggtcaacgta gttgttggag caaccaatga
     1621 tgcagtcgaa gcacctcgag cagaccacga cacaagatac actcctgctt gcccaatagg
     1681 gaaagagaag gctgtgcccg taacggtcaa gccaatagag tcatacttgg caacttgaga
     1741 agttccaaag tacgccgtag taacagcagg agccacaaga gagtaatgag ccgacgcagc
     1801 agccaacgca agaccggaat caagttgcgg cttgcgaagg atgacgtcgt aagtgatcca
     1861 gagctctccc gtgttgaagg cggcttgcgc accaacacta gcaagttgaa cgttgcacat
     1921 atcgtacatc cgggcatcct gtccagaagg tacagctccc gtacgaatgt aatgaacggc
     1981 cagggggttg tccttaggat cacactccac aggcatcaac atgtcgcaag agggcttgct
     2041 agacatcgca aagtaggagt tctccatttg ctgcttgttg acgtaggcgg cttggttcac
     2101 gttgtactct gcagcaagga tcatcgtacc caaggccagg ttcgtagacg aagcaatggc
     2161 atccgagcaa gtgctcttga agtgaaccat caagcccaac cactcatact cttcaaagtt
     2221 ctgagcgatg gctgacagcc acgggaacac ctcggcaagg ccagggttca tgacgaacgt
     2281 ttgattggta aaagcggtag ccgtcttcac gtcttgcacg tattctcgat gacgaatacg
     2341 cacaccatcc tcaacggaat gcatagcggg agtctgagaa ccggacatca acgagttctt
     2401 ccgaatcgtg taagccccgg gcccatcgac acgagcaatg ggtcccaccg ggtccttgtc
     2461 ctcctcatcc atcatgtcag cgttaccgtc ccagtaccgc ttgacgccct tgtaaacgcg
     2521 acggataaca ccgccgccag gaatgagaga agcagcagca tcataagccg cgtccttaag
     2581 gtacgctccc acggcgggaa gcacccccgc agcagccctc tcagccgcca tgcgacccaa
     2641 agtcgaagcg cccgccccac caaaccggcg agccatggtg gagaagtatc cgggccttcg
     2701 gctaccaccg gtacggcggc gcttgacagg agcacggcga gaagaagtcc ggcgaccgcg
     2761 accccgacga ccatagcgtc ggtagatagg cattctggtt ttctaatacc agaataaggc
     2821 tttgactcaa atgaaacgcg cttagaaaaa ctattcaatt ttttaagttt gagcaaaggt
     2881 ggtgggtaat actactacag gggctcaaaa atttcgcccc ttccgtggcc cacccccgag
     2941 ttgctagagg agcggcttct acttcgtact tacaactcgc gcctatcagc acatagaatc
     3001 acgctttggt cctctatgcg ctcctcgcaa tattaaagta gagaagctcg gtgcgcgtcc
     3061 ggaccgcgat gattcattat atggggaggg ggaaaccccc tccccaaacc cctccccgtg
     3121 cgaaaaatcc cagtgtccgg taaatacaga actaggcccc tcaagaggtg cgtttcaaaa
     3181 cgacctccaa cactgatcag accaagaaaa aagagaactg atcaacc
//
LOCUS       CruV-188                3230 bp    DNA     circular     24-FEB-2020
DEFINITION  Crucivirus-188 isolate 001649_virusbaton.
ACCESSION   CruV-188
KEYWORDS    .
SOURCE      Crucivirus-188
  ORGANISM  Crucivirus-188
            Unclassified.
REFERENCE   1  (bases 1 to 3230)
  AUTHORS   de la Higuera,I., Kasun,G.W., Torrance,E.L., Pratt,A.A.,
            Maluenda,A., Colombet,J., Bisseux,M., Ravet,V., Dayaram,A.,
            Stainton,D., Kraberger,S., Zawar-Reza,P., Goldstien,S., Briskie,J.,
            White,R., Taylor,H., Gomez,C., Ainley,D.G., Harding,J.S.,
            Fontenele,R.S., Schreck,J., Ribeiro,S.G., Oswald,S.A., Arnold,J.M.,
            Varsani,A., Enault,F. and Stedman,K.M.
  TITLE     Direct Submission
  JOURNAL   Submitted (24-FEB-2020) Biology, Portland State University, 1719 SW
            10th Ave, Portland, Oregon 97201, USA
COMMENT     Bankit Comment: ALT EMAIL:ide@pdx.edu
            Bankit Comment: TOTAL # OF SEQS:451
            Bankit Comment: GAP: est. length - alignment

            ##Assembly-Data-START##
            Assembly Method       :: MetaSPAdes v. 3.12; IDBA-UD v. 1.1.1;
                                     MEGAHIT v. 1.0.3
            Sequencing Technology :: 454; Illumina
            ##Assembly-Data-END##
FEATURES             Location/Qualifiers
     source          1..3230
                     /organism="Crucivirus-188"
                     /mol_type="genomic DNA"
                     /isolate="001649_virusbaton"
                     /isolation_source="Water"
                     /country="France"
                     /collection_date=2013
     CDS             1..975
                     /codon_start=1
                     /product="Putative Replication Associated Protein"
                     /translation="MDKQKHLTTACTTFDFRISKEYKLNDDMMDLTVNDIRDMLNGLT
                     SAYVFQLEKGNETGYIHYQGRFRLIKKTTLNNLKNLMRARELPLLPNYLSPTVTKEHL
                     KRTFNYQLKEDTRLSGPYKDNDILESDDSNLSFSELSKKYDVLQHLTSENKLYPYQRS
                     ILESSSKHDLRAIDVIYDPVGNNGKSTIKDYLDITRKAIIIPPTGDSTKLMEASYGII
                     KSEMDRIELKPDEIYRPGMILVDIPRSIKTGELYTFYGVLEQLKGGIAYDFRYSFKRL
                     RFQPPRIWIFTNSLPPPDCLSPDRMRIWTINESKELVKYEPDDDDELI*"
     CDS             1504..2841
                     /codon_start=1
                     /product="Putative CruV Capsid Protein"
                     /translation="MYYTYMPARQKQPAKRQARKPARPYKRPTSSYGGKGAYYVQGGA
                     HIKGALGPFKGGASINAGYAKDMKLGGYGAYSLENVKHNTLIKPDPPQMMNSYYREGA
                     FVIRHREYIGDIISSSTANTFNIQTFPLNPAQASTFPWLASGIGPSFEEYKLNGCVFE
                     FKSTCSDAIASSTNLALGQVMMCVQYDPTDPVFTTSNQLLNYFWAQSGKVSDNIYHFV
                     ECDKQQSPLVHLYTRSGAASSDSDLRFSDFGRFSIATNGLQGTSINLGQLWVSYEFLM
                     YKPKVASLESVAGGWFHLGLSDGSVTNLNPFGSTPMTSGQVDSENNLNIILSDNVNYG
                     RITFPKLYQDSSYVISCVWRGDSTASVSTPTVIASDSAVSFIEHLDDNQIAAVLLPST
                     SSTSTMVSIQTWVTIPGDAVAHTVDFGVGTLPANTTVDVYVSQIPYLDPNIYA*"
ORIGIN
        1 atggacaaac aaaaacacct aacaacagca tgtacaacat ttgattttag gatcagtaag
       61 gaatacaaat taaatgatga catgatggat cttacagtga acgatataag agatatgtta
      121 aacggattaa catcagctta tgtctttcag ttagaaaagg gtaacgagac cggatatatt
      181 cattaccaag gcagatttag gttaataaag aagaccacac ttaataattt gaagaatctc
      241 atgagggcac gtgaactacc attattacct aattatttaa gtcctacagt tacaaaggaa
      301 cacttaaaac ggacatttaa ctatcaatta aaggaagaca ctagactatc tggaccgtat
      361 aaggataatg atattctaga aagtgatgat tcaaacttat ctttttcaga gttatctaaa
      421 aagtatgatg ttttacagca tctaacgtca gagaataaat tataccccta ccaacggtcc
      481 atattagagt cttcaagtaa gcacgattta agggcaattg atgttatata tgatccggta
      541 ggaaataacg gtaagagcac tattaaggat tatttagata ttacacgtaa agctataata
      601 atacccccta caggagatag tacaaaatta atggaggctt cttatggaat aattaaatct
      661 gagatggaca ggattgaatt aaagcctgat gaaatatata gacctggtat gatactggta
      721 gatataccaa ggtctattaa aacgggtgaa ttatatactt tttatggtgt attagaacaa
      781 ttaaaagggg gtatagctta tgattttaga tattcattta aacggttaag gttccaacca
      841 cctagaatat ggatatttac aaattcatta ccaccccctg attgtttatc acccgatagg
      901 atgagaatat ggacaattaa tgaatcaaaa gaattggtga aatatgaacc agatgatgat
      961 gatgaattaa tttgatgtcc agattatcta caaactggac aaatatgtcc agattacttt
     1021 acatggtttt cattatacaa acaccatata atgaaaatgt ccagatgtcc aaataataca
     1081 atactattaa taggaaaggt tttaaggaat aacatttatg ttatgttctt ccaagtgctt
     1141 tacgcactgt ccttaaaacc ttttacctta tgtgtgcaaa caaacaataa ggtagaagaa
     1201 tggagggctt tatgaagaaa gcccctggtt ttatggtacc atctcatggt ttttgtattt
     1261 atacaaaaaa cccatagaac gggcaaaccc gggtcacggt tggagcgggt agcgagcgat
     1321 aaataaaaaa agcgagcgga cacggaggtt cggaccccac atgacaacgg gcccctttct
     1381 atgtacgaag tagatgagat ggtcacccta aactagcgta ccagtaggcg gtaaacgggc
     1441 cctttacaac gggctttttt ttatggtata aaaaaaaatt agtttactct aggttttttt
     1501 tttctgtact atacatatat gccagccaga caaaaacaac cagccaagcg acaagctaga
     1561 aaacccgcta gaccctataa gaggcctaca tccagttatg gaggtaaagg ggcctattat
     1621 gttcaaggcg gtgcccacat taaaggagct ttaggtccat ttaaaggggg agccagtata
     1681 aatgccgggt acgctaaaga tatgaaatta gggggatacg gggcctattc attggagaat
     1741 gtgaaacaca acacgttaat taaacccgat ccaccccaga tgatgaattc ttattatagg
     1801 gaaggagctt ttgttatccg acatcgagaa tacatcggag atataataag ttcatctacg
     1861 gcaaacacat ttaacattca aacatttcca ttaaatccag cacaagctag tacatttcca
     1921 tggttagctt ctggtattgg tccgtcattt gaagaataca aattgaacgg gtgtgtattt
     1981 gaatttaaat ccacatgttc tgatgcaatt gcatcatcca ccaatctggc cctaggacag
     2041 gttatgatgt gtgtacaata cgatcctaca gatccagtgt ttactacatc taaccaattg
     2101 ttgaattatt tctgggcaca atcaggcaaa gtatctgata acatttacca ttttgtagaa
     2161 tgtgataaac aacaatcacc attagtccat ttatacactc ggtcaggagc agcatcttct
     2221 gattccgact taagatttag tgattttggt agattttcca tagcaacaaa tggattacaa
     2281 ggaacatcta taaatttagg tcaattatgg gtatcttatg aattcttaat gtacaaacct
     2341 aaagtagcat cattagaatc agttgctgga ggatggttcc acttaggatt atctgacgga
     2401 tcagtaacta atttgaatcc ttttggttcc acaccgatga catcaggaca agtagactct
     2461 gagaataatc ttaatattat tctttctgac aatgttaatt atggaagaat cacttttcct
     2521 aagttatacc aagattcatc ttacgttata agctgtgtat ggcgaggtga ttcaactgca
     2581 tcagtatcta caccaacagt tatagcatct gattcagccg tcagttttat agaacattta
     2641 gatgataatc aaatagcggc agtattatta ccgtccacat catcaacttc cacaatggta
     2701 tccatccaga catgggtaac tataccagga gacgccgtag cacacactgt agattttgga
     2761 gttggaacac taccagcaaa tactacagta gatgtatacg tgtcacagat accatattta
     2821 gatcctaata tttatgccta aagtttaaaa ttaatttata tatacatata tatatacatt
     2881 aatggatccg tattataatt attatttaac tagtaaatat tttggtttac tgtacacacg
     2941 gacatgtaag gaatgtaacg tccgtaaata taattataag tttctagatg aattatatct
     3001 agaggattac aacaagatta gataccataa gagatgtgaa ccatgtagga aatatagaca
     3061 aatggtcaga cgatttatgg atacatcaac cagtgacaac cctaggatgg aatcaaccag
     3121 tgacaatctt aggatgacac ataccagcga taactcttat ttgtaataca tttaaacggt
     3181 aaaaacattt agataattat atctagattt tttttatagt gtctacatat
//
LOCUS       CruV-189                3230 bp    DNA     circular     24-FEB-2020
DEFINITION  Crucivirus-189 isolate 120_1481_IR2_3297.
ACCESSION   CruV-189
KEYWORDS    .
SOURCE      Crucivirus-189
  ORGANISM  Crucivirus-189
            Unclassified.
REFERENCE   1  (bases 1 to 3230)
  AUTHORS   de la Higuera,I., Kasun,G.W., Torrance,E.L., Pratt,A.A.,
            Maluenda,A., Colombet,J., Bisseux,M., Ravet,V., Dayaram,A.,
            Stainton,D., Kraberger,S., Zawar-Reza,P., Goldstien,S., Briskie,J.,
            White,R., Taylor,H., Gomez,C., Ainley,D.G., Harding,J.S.,
            Fontenele,R.S., Schreck,J., Ribeiro,S.G., Oswald,S.A., Arnold,J.M.,
            Varsani,A., Enault,F. and Stedman,K.M.
  TITLE     Direct Submission
  JOURNAL   Submitted (24-FEB-2020) Biology, Portland State University, 1719 SW
            10th Ave, Portland, Oregon 97201, USA
COMMENT     Bankit Comment: ALT EMAIL:ide@pdx.edu
            Bankit Comment: TOTAL # OF SEQS:451
            Bankit Comment: GAP: est. length - alignment

            ##Assembly-Data-START##
            Assembly Method       :: MetaSPAdes v. 3.12; IDBA-UD v. 1.1.1;
                                     MEGAHIT v. 1.0.3
            Sequencing Technology :: 454; Illumina
            ##Assembly-Data-END##
FEATURES             Location/Qualifiers
     source          1..3230
                     /organism="Crucivirus-189"
                     /mol_type="genomic DNA"
                     /isolate="120_1481_IR2_3297"
                     /isolation_source="Water"
                     /country="Norway"
                     /collection_date=2011
     CDS             1..1047
                     /codon_start=1
                     /product="Putative Replication Associated Protein"
                     /translation="MAKQVSPVHNVMIRISRPDSCSKELWYAETSQKIIQSMFKSFSK
                     NYIYQYEFTKSHQYDENNEEIPDSERDNDHIQGFSKLERKQRPSALAKIIRTELEILM
                     KDFSISVQPAHDTNALSKYSMKSDTRVNNLVFSDHAIYLGVDLPKLENLYPYQKDLYQ
                     MCLSKPDDRLIYWIYDKIGNTGKSKFCKFMSYHIKTPKLDYGKTSDILNLVSKYPNSN
                     AYLFNLTRAKPMQIGEQDLYSTLESCKDGHFCNTKYNTEMVLQNPAHIIVFANHYPQI
                     NLMSLDRWRIMTITEEKKLVPYQKKEEEEEEVKKDFTPKLDYEQLRDIRMQKERLEKY
                     TSVSLLDRLANLKN*"
     CDS             1290..2834
                     /codon_start=1
                     /product="Putative CruV Capsid Protein"
                     /translation="MSAPRRARRVVEEEYVPVAAAAAPKKKKSWLKRAVKNTRNFIRK
                     AARPVLSAGVAMLQRKALNKISGMGDYTINANSFITPVDSVPQFGTLGAGVRVRHREY
                     IQDINSYTDFTNTSFSVNPGLATTFPWLSSIANSFEQWKALGIVFEFKSTSANALNST
                     NTALGVIVMASEYNASRPAFTSKQQMETAIFAGSTKPADSLMHAIECAPEQTPTTPQY
                     VRSGAPPANQDYRLYDLCNFQIAGVGSQAQAAVGELWISYDIVFYKPILNSSPGNNID
                     TAHYILANSGNAAFGSMGDSTLLVKSFDQIGGDITYDSPNGYQFPIGKSGNYLVVYHM
                     QQDNAALVVCGLPTIAVNNGTVVNQWVGQTPTPVPFSEGIGRDLLVPTPMSAQVFFVL
                     VNIPDPNKKCLLQITLGQVNFPGFSDFLITQVNGQLPTSLPASIMSAAKSAKLVKQKK
                     YIESAAAGDDNHDEDEDDEECHSLDTIIETINKLKTTYGFSDEQVKSFLDLRPSNKSA
                     KTSRKC*"
ORIGIN
        1 atggcaaaac aagtttcgcc tgttcataac gtaatgataa gaatcagtcg ccctgattca
       61 tgcagtaaag aactatggta tgcagaaact tcacaaaaaa tcatacagag tatgttcaaa
      121 tcatttagta agaattatat atatcagtat gaattcacta aatctcatca atatgatgaa
      181 aataacgaag aaatcccaga tagtgaaaga gataacgacc acattcaagg attctctaaa
      241 ctagaaagga agcaacgacc ttccgcacta gcaaaaataa taagaactga attagaaata
      301 ctaatgaagg acttctcaat aagcgttcaa ccagcacatg atacaaacgc attatcaaaa
      361 tattcaatga agtcagatac acgtgttaat aacttagtat tctctgacca tgcaatatat
      421 ctaggagtag atttacctaa attagaaaat ctctacccat atcaaaaaga cctatatcag
      481 atgtgtttat caaaacctga tgatagactt atttactgga tttatgataa aatcggaaat
      541 accggaaaaa gcaaattctg caaatttatg tcctaccata taaaaacgcc taagcttgac
      601 tatggtaaaa cttcagacat attaaattta gtcagcaaat atccaaatag caatgcctat
      661 ttattcaacc tcactcgagc aaaaccaatg caaataggcg aacaagacct atatagcaca
      721 ttagaaagtt gcaaggatgg acatttctgc aacacgaaat acaatactga aatggttcta
      781 cagaatcctg cacatattat agtattcgca aatcattacc cgcaaataaa tcttatgtca
      841 ttagacagat ggcgtattat gacaataaca gaagaaaaga agttagtccc ttatcaaaaa
      901 aaagaagaag aagaagaaga agtaaagaaa gactttactc ccaagcttga ttatgaacaa
      961 ttacgtgata taagaatgca aaaagaaaga ttagagaaat atacaagtgt atcattactc
     1021 gacagattgg ctaatttaaa aaattagttc caatagttcc aaaaaattat attatgtagt
     1081 gaaacgaaaa atataataaa taatggggtt gcataattcc aacgtagtgc aacaacccct
     1141 taggataaat attataaata atattatatc cgacttaatt ttaatctaaa attaaaaata
     1201 aaattttaaa taaaatttct aaaaattctg gtaagggtaa ttaattattt taaattaaaa
     1261 atataattac tacatatata acatccgaga tgtctgctcc acgtcgtgct cgtcgcgtag
     1321 tggaagaaga atatgttcct gtggctgctg ctgctgcacc taagaagaaa aaaagttggc
     1381 ttaaacgtgc tgtaaaaaac acacgcaact ttatacgcaa agccgctcgc cctgtgctaa
     1441 gtgctggcgt agcaatgcta caacgtaaag cattaaataa aatctctgga atgggcgatt
     1501 atactattaa tgccaattct tttattactc cagttgattc tgttccgcaa ttcggaactc
     1561 tcggcgctgg cgttcgagtt cgtcatcgtg aatacattca agatatcaac agttatactg
     1621 atttcacaaa tacttcattt agtgtaaatc ctggacttgc tacaacattc ccatggttgt
     1681 catccattgc aaatagtttt gaacaatgga aagctctcgg tattgtcttt gaatttaaaa
     1741 gcacatccgc caatgccctt aactcaacaa acacggctct cggcgttatt gtgatggcat
     1801 ctgagtataa tgcgtctcga cctgcattta cctctaagca acaaatggaa actgccattt
     1861 tcgcgggctc aactaaaccc gctgattcat taatgcacgc tatcgagtgt gctcctgaac
     1921 agacacctac cacgcctcag tatgtgcgtt ctggtgctcc tcctgcaaat caagactatc
     1981 gtctttatga cctttgcaac ttccaaattg ccggtgtagg ctcccaggcc caggccgcag
     2041 tcggagaact ttggatttcg tacgatatag tgttttacaa acctattctt aactcatctc
     2101 ccggcaataa tattgacact gctcattata ttcttgctaa ctcgggtaat gctgcctttg
     2161 gctctatggg agattctact ctccttgtca aatcatttga ccaaatcggt ggagatatca
     2221 cttatgattc tcctaacggt tatcagtttc ctattggaaa gagtggaaac tatttagtcg
     2281 tgtatcacat gcaacaagac aacgctgctt tagtagtgtg tggtcttcct acaattgctg
     2341 ttaataacgg cactgttgta aatcagtggg taggacaaac tcctacacct gttcctttct
     2401 cagaaggtat cggcagagat ttactagttc ctacaccaat gagtgcgcaa gtattctttg
     2461 tgctagttaa tattcctgac cctaataaaa aatgcctgct acaaattact cttggacaag
     2521 taaattttcc tggcttctca gattttctta ttacccaagt aaatggacaa cttccaactt
     2581 cgcttcctgc tagtataatg tccgctgcta aatcggccaa actagtgaag caaaaaaaat
     2641 acatcgaatc cgctgctgct ggtgatgata atcatgatga agatgaagat gatgaagagt
     2701 gtcatagtct tgacaccata attgaaacaa ttaacaaact gaaaactact tacggattca
     2761 gtgatgaaca agttaaatca ttccttgatt tacgcccatc aaataaatca gcaaaaactt
     2821 ctagaaaatg ctgaacaata aaaaaaaggt aatccttggc aacccccctg attaataaag
     2881 attctatggg gaagcgagtc ttcgcccata gagataacac atttaggtct aaaataataa
     2941 ttatattttt ttagtcttta tttacccgat tacccggaat atttttttaa aacaaccgcc
     3001 ttcgctctcc gtcgagtgtg ccgaataggt caaaaaaatt aaagagaaac ggcccgtcaa
     3061 gccgtcaaag aaacaaacgc tagataatat cattataaaa taagacctaa atttattata
     3121 aattagggcg aagatgaagc gtgtcataca aaattaatta taattatggt ttaacattaa
     3181 ttaattaatt aaaaataaaa tagttccata catatagaac tataacaacc
//
LOCUS       CruV-190                3241 bp    DNA     circular     24-FEB-2020
DEFINITION  Crucivirus-190 isolate H10_4124.
ACCESSION   CruV-190
KEYWORDS    .
SOURCE      Crucivirus-190
  ORGANISM  Crucivirus-190
            Unclassified.
REFERENCE   1  (bases 1 to 3241)
  AUTHORS   de la Higuera,I., Kasun,G.W., Torrance,E.L., Pratt,A.A.,
            Maluenda,A., Colombet,J., Bisseux,M., Ravet,V., Dayaram,A.,
            Stainton,D., Kraberger,S., Zawar-Reza,P., Goldstien,S., Briskie,J.,
            White,R., Taylor,H., Gomez,C., Ainley,D.G., Harding,J.S.,
            Fontenele,R.S., Schreck,J., Ribeiro,S.G., Oswald,S.A., Arnold,J.M.,
            Varsani,A., Enault,F. and Stedman,K.M.
  TITLE     Direct Submission
  JOURNAL   Submitted (24-FEB-2020) Biology, Portland State University, 1719 SW
            10th Ave, Portland, Oregon 97201, USA
COMMENT     Bankit Comment: ALT EMAIL:ide@pdx.edu
            Bankit Comment: TOTAL # OF SEQS:451
            Bankit Comment: GAP: est. length - alignment

            ##Assembly-Data-START##
            Assembly Method       :: MetaSPAdes v. 3.12; IDBA-UD v. 1.1.1;
                                     MEGAHIT v. 1.0.3
            Sequencing Technology :: 454; Illumina
            ##Assembly-Data-END##
FEATURES             Location/Qualifiers
     source          1..3241
                     /organism="Crucivirus-190"
                     /mol_type="genomic DNA"
                     /isolate="H10_4124"
                     /isolation_source="Water"
                     /country="New Zealand"
                     /collection_date=2012
     misc_feature    join(3229..3241,1..20)
                     /note="stem-loop"
     misc_feature    1..9
                     /note="nonanucleotide"
     CDS             296..1426
                     /codon_start=1
                     /product="Putative Replication Associated Protein"
                     /translation="MSISKQDFIKGEGEEDNGKKFRDFVFTYNNYNDEVETRIKAFGE
                     QYARYIVYGREIAPTTGTKHLQGYIYLKSSNTRKSLIKHIGKCYIAPRSRRATFEQAI
                     TYCKEDGDFYEFGKAPITKHATISTRWIILKEEMKKGTPIEELMASNLDLFIKYPSGC
                     KNAYNILRPKVNFDLLKEYPTLRPFQKDIIDYVQTQPEKRKILWICDNGRSGKSELVA
                     HLLTHYKFIYLENAKTQDIAHAWNGEHVLFDLPRVSEEHLNYSALESLKNGRIFSSKF
                     DSQSKCYNRPHIIIFANYTPNISSLTMDKWDIRFIDEKFNMQCIDAQKIKDEELTIEK
                     LEDIYNVKKTVKPKNKKVKKISDYIDNGILQENENSIILNFD*"
     CDS             1466..2740
                     /codon_start=1
                     /product="Putative CruV Capsid Protein"
                     /translation="MPPAKKAPAKKSVVKQFWQKAYKQKNSYIRRGLVAGGGAIGRAL
                     LPTGGDYLGRMAGTGIADLIGAGRYSLADIKYNSALSLLPENATLHSQVPSMHKTGNG
                     EILVSHREYMGEVISSSTADTFKVDKYPVNPAMRETFPWLSAIAQQFESYRIRGMVFE
                     FRSNSGNALNSTNTALGSVQMGVSYRPSSLSPTNKQQVLNLDFGSESVPSKDFITALE
                     CEPSTLINGGRYYTRGSDELPADDSINTYDMANFYISSSGVQGTNVVLGSVFCSYQIL
                     LQKPIDSADQGAWLHQAHYVGPARTITTTNGGNNVFNGLVEVHDGIGITFPASDTIQF
                     PRGTVGKYLLYIGIIGSAQTLGTWTPTLTNCVHVSGQYNTGGSDTNLLFCYAVNIIDP
                     AKVATVKLVGTGTPSDWTQHFLKVIQLSNTIF*"
ORIGIN
        1 tattattacc tatactacgg cgttccagtg ttccactgga acgacaaaag gcatatgcta
       61 acgaataaac tttcttattt tatggaacac cacgatatgc tctaaaaaaa taactttggt
      121 gttccaaaaa actttttctt atttgataat gaagatatct aaagactgat accgttttta
      181 gatatcgatt aatcaagggc gtaaacgccc tataacaact ttcaaattgt atctattagt
      241 cgttttacaa ataaacgcac ctaaaattat aaaatatcct ataactatat agagaatgtc
      301 aatttctaaa caagacttta taaagggtga aggtgaagaa gacaatggaa aaaaattccg
      361 agacttcgta ttcacataca acaattataa tgatgaagtt gagacgcgaa ttaaagcatt
      421 tggcgaacaa tacgcgcgat atatagttta tgggcgtgaa atagcaccaa ctacaggaac
      481 taaacattta caaggttata tataccttaa atcatcaaat accagaaaaa gtttaatcaa
      541 acatattgga aaatgttata tagctccacg aagtcggcgc gcaacatttg aacaagcgat
      601 aacttattgt aaagaagatg gagactttta cgaattcgga aaagctccta taactaaaca
      661 cgctactata tccacacgat ggattatatt aaaagaagag atgaaaaaag gcacaccaat
      721 agaagaatta atggcgtcta atttagattt atttataaaa tacccttcag ggtgtaagaa
      781 tgcatataac atattacgac ctaaagtaaa ctttgattta cttaaagaat atccaacttt
      841 aagaccattc caaaaagaca taatagatta tgttcaaaca caaccagaaa agcgaaagat
      901 tttatggata tgtgataatg gacgttctgg aaaatctgaa ttagtagcac atttattaac
      961 acattataaa tttatatatt tagaaaatgc aaaaacacaa gatatagcgc acgcatggaa
     1021 tggcgaacat gtattattcg accttccacg agtaagcgaa gaacatttaa actattcggc
     1081 actcgaaagt cttaaaaatg gccgtatatt ttcatctaag tttgatagtc aatcaaaatg
     1141 ttataataga ccacatataa ttatttttgc aaactataca cccaatatat catcattaac
     1201 aatggataaa tgggatatta gatttatcga cgagaaattt aatatgcagt gtatagatgc
     1261 acaaaaaatt aaagatgaag aattaacaat tgaaaaactc gaagatatat acaatgttaa
     1321 aaaaactgtc aaaccgaaaa ataagaaagt taaaaaaatt tccgattata tagataacgg
     1381 aattttacaa gaaaatgaaa attcaataat tttaaatttc gattaaaaaa tataatttct
     1441 aaactaatta tataaaacaa ataccatgcc tccagcaaag aaagcgccag cgaaaaaatc
     1501 agtagttaaa cagttttggc aaaaagcata caaacaaaag aattcgtaca ttcgccgtgg
     1561 attagtagca ggtggtggtg caattgggcg tgcactactt cctacaggtg gcgattatct
     1621 aggacgtatg gcgggcacag gaatcgcaga cctgattggt gcgggtagat attcattagc
     1681 tgatattaaa tataattcag cattatcatt gcttccagaa aatgcgacat tacattcaca
     1741 agtgccatca atgcacaaaa caggtaatgg tgaaattctt gtttcacacc gcgaatatat
     1801 gggtgaagtt atttcatcct ctacagcaga tacatttaaa gtggataaat atccagtaaa
     1861 tccagcaatg cgcgaaacct ttccttggtt aagtgcaata gctcaacaat ttgaaagtta
     1921 tagaattcgt ggtatggtat ttgaatttag aagcaatagc ggtaatgcgc taaatagcac
     1981 aaatactgct cttggttctg tacaaatggg cgtatcttat cgtccatcaa gtttatctcc
     2041 aaccaataag caacaggtac ttaacctaga ttttggttca gagagtgtac catctaaaga
     2101 ttttattaca gcgcttgaat gcgaaccttc tacacttatt aatggtggta gatattatac
     2161 tcgtggtagc gatgagctac cagctgatga ttcaattaat acttatgata tggctaattt
     2221 ttatattagt tcatcaggag tacaaggaac aaatgttgtt cttggaagcg tattttgttc
     2281 ttaccaaata cttcttcaga agccaattga tagcgcagat caaggagcat ggctacatca
     2341 agcacactat gtaggaccag cacgcactat taccacaaca aatgggggta ataacgtctt
     2401 taatggactt gtcgaagttc atgacggtat tggaattact tttcctgcgt cggacacaat
     2461 tcaattccca cgtggaacag ttggtaaata tctcctgtat attggcataa ttggcagtgc
     2521 tcagacttta ggaacttgga cacctacgct cacaaattgt gttcacgtgt caggacaata
     2581 taatacaggt ggaagcgata caaatctatt attttgttat gcggtgaata ttattgaccc
     2641 tgcaaaagta gcaactgtta aattagttgg aacaggtaca ccaagtgatt ggacacaaca
     2701 tttcctgaaa gtaattcaat tatcaaatac aattttttaa ctaaataaat tagtcgatta
     2761 gaacagacta aataaaaatc tttagatccg aaataatcat gaatttttca ctgatttata
     2821 ataaccagtg aattaacaaa taaataaaaa aaacattaat ttttgatggc atagggtttt
     2881 gccgacaact tagaataatt taaaaacatt ttatttttta tttattttaa tctcacgaag
     2941 tgagattaaa aaagaaaatt ttttaattta ttattagttt tcaaaaaacc cgatgtaaaa
     3001 aataatgtaa actaaatata taatgaataa gcaagtttat ttaaatggat tatatacacg
     3061 taaagaatta attatgatta taataaagtc tacaataaga cgaattttag gaaaaagaga
     3121 attatttata tcacataatc aatgatttta acaatcacat aaccaatgat tttaaaaaaa
     3181 attttttaaa atctacaaat atataaccta tatataaaat gccttttccc gtagtatagc
     3241 a
//
LOCUS       CruV-191                3243 bp    DNA     circular     24-FEB-2020
DEFINITION  Crucivirus-191 isolate 120_502_IR1_3296.
ACCESSION   CruV-191
KEYWORDS    .
SOURCE      Crucivirus-191
  ORGANISM  Crucivirus-191
            Unclassified.
REFERENCE   1  (bases 1 to 3243)
  AUTHORS   de la Higuera,I., Kasun,G.W., Torrance,E.L., Pratt,A.A.,
            Maluenda,A., Colombet,J., Bisseux,M., Ravet,V., Dayaram,A.,
            Stainton,D., Kraberger,S., Zawar-Reza,P., Goldstien,S., Briskie,J.,
            White,R., Taylor,H., Gomez,C., Ainley,D.G., Harding,J.S.,
            Fontenele,R.S., Schreck,J., Ribeiro,S.G., Oswald,S.A., Arnold,J.M.,
            Varsani,A., Enault,F. and Stedman,K.M.
  TITLE     Direct Submission
  JOURNAL   Submitted (24-FEB-2020) Biology, Portland State University, 1719 SW
            10th Ave, Portland, Oregon 97201, USA
COMMENT     Bankit Comment: ALT EMAIL:ide@pdx.edu
            Bankit Comment: TOTAL # OF SEQS:451
            Bankit Comment: GAP: est. length - alignment

            ##Assembly-Data-START##
            Assembly Method       :: MetaSPAdes v. 3.12; IDBA-UD v. 1.1.1;
                                     MEGAHIT v. 1.0.3
            Sequencing Technology :: 454; Illumina
            ##Assembly-Data-END##
FEATURES             Location/Qualifiers
     source          1..3243
                     /organism="Crucivirus-191"
                     /mol_type="genomic DNA"
                     /isolate="120_502_IR1_3296"
                     /isolation_source="Water"
                     /country="Norway"
                     /collection_date=2011
     CDS             1..999
                     /codon_start=1
                     /product="Putative Replication Associated Protein"
                     /translation="MPKITKPRKSGKHTSPKNQRARRWTWTIFDPIVIDRLTKDGDLF
                     EDLKQKLKYVCFQAERCPSSGKIHLQGYCETDYDTSGQQLQKTFECPSMHLAKSKGNA
                     EQNKVYCSKSETRIGEFKEAGKPGPGQGKRTDLVEMHQQIMLGAPLRTIMNEYSPNWY
                     RYGSNILKTYSYMDPPFRESIKVHYLYGPPNIGKSGLARVMRKDQDAYIAKDNANGWF
                     DGYMGQQVVVFGEFRGRFPIEDMLELLDHSETRQQVKNAYLPIHATEFYFLSNFPPEE
                     HYRGKEHPQWLSRIRGPRFDLHEVLNGDQIIKRQKELGLDFYLPKSEQKLKLKPTV*"
     CDS             complement(996..1370)
                     /codon_start=1
                     /product="putative ORF"
                     /translation="MGTSISVPENSLGLVIASGSGASLTIPANTFGAEETVRLLVNVS
                     WLGTLVLIPDVLYTSNVSSLSYYVSGTQTKKTCNASPAGATLMFWYQITDPRQDAVVT
                     LDDTGGFSSATDALLHVTASIY*"
     CDS             complement(1445..2857)
                     /codon_start=1
                     /product="Putative CruV Capsid Protein"
                     /translation="MPRRTFRRSFKRRPSYSRRRPVKRYRRSGGSGLASRFVGSAARA
                     AGALAGAAAANAAASQVSAMGWEGAKSLASRAADAAGRYLIPGYGIVSDAVAGVKRYR
                     DDGAAAMDIQGVNRMGGVIDGRGAYSIRKNSLMGDSSQVPMMHSTEDGVRVRHREYVG
                     DISTSVLFTNRTYPLNPGVSSTFPWLSAIAQNFEQYEWLGVVVQLKSISSDAIASSTN
                     LAMGTIIMAAEYNVNQPSYVNKSQMENSYFACSAKPSEDFIMPVECDPRDNPIATHYV
                     RIGDVSSNQDLRLYDMCNIQVATVGSQAAYPVCELWISYDVILRKPQLDSGLALSAKT
                     AHYSLVAPAVPSGYFGASRSEQFDSIGLVVTGTTVTFPVGLSGTWLVNYSARGSSTAC
                     VDPTVAVTNCAIQTFYAAGTTGSASNTGTTTSIYMLEFILKFTDPNLAAVISLTGGTL
                     PATPTTGDLVITQVNDALNS*"
ORIGIN
        1 atgccgaaaa taaccaagcc tcgcaagtct ggcaaacata cgtcgccaaa aaatcagcga
       61 gcccgacgtt ggacttggac cattttcgac cccattgtta tcgacaggct caccaaagat
      121 ggagatttgt tcgaggatct gaaacaaaag ctcaagtacg tttgcttcca agctgaacgt
      181 tgtccgtcaa gcggaaagat tcatttgcaa ggctactgtg agaccgacta cgatacctcg
      241 ggacaacaac tacaaaagac ctttgagtgc ccctcaatgc atctcgcaaa atcaaaggga
      301 aacgcagaac aaaacaaggt atactgctcg aagtcagaaa ccaggatcgg cgagttcaaa
      361 gaagccggga agcccggtcc tggtcaaggc aaacggacag atttggtaga gatgcatcaa
      421 caaatcatgc tcggtgcacc actgcggaca attatgaacg aatattcgcc caactggtat
      481 cggtatggat cgaacatcct aaagacgtat agctacatgg acccgccatt tagagaatcc
      541 atcaaggtgc attacttgta cggacctcca aacatcggaa aatccggact ggcccgcgta
      601 atgcgcaaag atcaagacgc atatattgcc aaagacaacg ctaacggatg gttcgatggc
      661 tatatgggcc aacaagtagt cgtcttcggc gagttcagag gccgatttcc aatcgaagac
      721 atgctggaac tgttggatca cagtgaaact cgccagcaag tcaagaacgc ttacctccca
      781 attcacgcaa cagagttcta cttcctatcg aacttccccc cggaagaaca ctaccgagga
      841 aaagaacatc cccaatggct ctctcgcatt cggggacccc gttttgatct acacgaggta
      901 ctcaatggcg atcaaatcat caaacgacaa aaggaactgg gccttgactt ctacctgcca
      961 aaaagtgaac aaaaactgaa actaaaacca acggtttagt aaatactcgc tgtaacatga
     1021 agtaatgcgt ctgtggcaga ggaaaaaccg ccagtgtcat caagagtaac aacagcatct
     1081 tgtctggggt cagtaatttg ataccaaaac atcaaagtcg ccccagcagg agatgcgtta
     1141 cacgtcttct tggtttgggt cccactgacg tagtacgaaa gcgaactcac gttcgaagta
     1201 tacaagacat caggaatgag gactagcgtc cccaaccaag agacattgac caaaagacga
     1261 accgtttcct ctgcaccgaa ggtgttggcc ggaatagtca agctagctcc ggagccagag
     1321 gcaatgacta gtccaaggga gttctctggc acagagatac tagtgcccaa aggattttgg
     1381 aagtcaatac cggtgacgga ctcaccgcga taaaccttgg tagaggcgcc cggtccatcc
     1441 gggcttacga gtttaaagca tcgttgacct gagtgatgac aagatcaccc gtagtaggcg
     1501 ttgcaggcaa cgtaccaccc gtcaacgaga tgacagcagc aagattcgga tcggtaaact
     1561 tgaggatgaa ctcaagcatg taaatggacg ttgtggttcc cgtattgctg gccgatcccg
     1621 ttgtgccagc agcgtaaaac gtctgaatgg cgcagttagt aacagcaact gtaggatcaa
     1681 cacaagcggt cgaagatccg cgtgccgaat agttgaccaa ccacgtgccg gacaatccca
     1741 ctgggaacgt aacagtggtg ccggtcacga ccaaaccgat cgaatcaaat tgctcagacc
     1801 gcgaagcccc aaagtagccc gacggaactg caggagcaac gagcgaatag tgggccgtct
     1861 tggcagacaa ggccagacca gagtcaagtt ggggcttgcg cagaatgaca tcgtacgaaa
     1921 tccaaagctc acacactggg taagccgctt gggatccgac agtcgcgact tgaatgttgc
     1981 acatgtcgta gagacgaaga tcctgattgg aagaaacatc tccaatacgg acatagtgcg
     2041 tagcaatcgg gttgtctcga ggatcacact cgacaggcat gatgaagtct tccgacggct
     2101 tggcactgca agcgaagtaa ctgttctcca tctgggactt gttgacgtag gacggctggt
     2161 tgacgttgta ctcggccgcc atgatgatgg tacccatcgc caagttcgta gacgaagcga
     2221 tagcgtccga agaaatactc ttcagttgaa caaccactcc aagccactcg tattgttcaa
     2281 agttctgcgc gatcgcagac aaccaaggga aggtagacga cacaccagga ttcaacggat
     2341 aggtacggtt cgtaaagagg actgaagtcg agatgtctcc gacatactca cgatgacgaa
     2401 cccgaacacc atcctccgtg gaatgcatca tgggcacttg agaagaatcg cccatcaacg
     2461 agttcttgcg aatcgaatac gcaccacggc cgtcgatgac accacccatt cggttcacac
     2521 cttgaatgtc catggcggct gcgccgtcat cacggtaacg cttgacacca gcgacagcat
     2581 cagacacgat gccatagcct gggatcaagt aacgaccagc agcatcggca gcacgcgatg
     2641 ctagagactt agcaccttcc caacccatag cgctgacttg actagcggca gcattcgccg
     2701 cagcagcgcc agcaagagca ccagccgctc gcgcggcaga accaacaaag cgtgaggcaa
     2761 gcccagatcc ccccgaccga cggtaccgct tcacgggtcg gcgacgactg tacgacggac
     2821 gacgtttgaa cgatcgccga aaagtccggc ggggcatgaa tcactttttt agacgaaaac
     2881 tgagaatttt ttttcagttt tccgtatacc acattttaaa aaggagctga gtaaaggtct
     2941 gagtaaaggt tacccatgta actcttacag accacttctt caaaatggtg aaaaacctct
     3001 tcatactcag acatacctaa aagtctgagt agaggtgact aggtaatact agggcccctt
     3061 cctacggagg gaccctgcct agtcacccct accttccaaa aagtttttaa tgtagggaat
     3121 tttcacaaag tttcatgggg gggcataccc ccccccaaac cccccgcttt acaaggagcg
     3181 ggggatcaag catctgtggt catccgtaat cccgaaaaaa aatccattct tccaataaga
     3241 aag
//
LOCUS       CruV-192                3244 bp    DNA     circular     24-FEB-2020
DEFINITION  Crucivirus-192 isolate 001637_virusbaton.
ACCESSION   CruV-192
KEYWORDS    .
SOURCE      Crucivirus-192
  ORGANISM  Crucivirus-192
            Unclassified.
REFERENCE   1  (bases 1 to 3244)
  AUTHORS   de la Higuera,I., Kasun,G.W., Torrance,E.L., Pratt,A.A.,
            Maluenda,A., Colombet,J., Bisseux,M., Ravet,V., Dayaram,A.,
            Stainton,D., Kraberger,S., Zawar-Reza,P., Goldstien,S., Briskie,J.,
            White,R., Taylor,H., Gomez,C., Ainley,D.G., Harding,J.S.,
            Fontenele,R.S., Schreck,J., Ribeiro,S.G., Oswald,S.A., Arnold,J.M.,
            Varsani,A., Enault,F. and Stedman,K.M.
  TITLE     Direct Submission
  JOURNAL   Submitted (24-FEB-2020) Biology, Portland State University, 1719 SW
            10th Ave, Portland, Oregon 97201, USA
COMMENT     Bankit Comment: ALT EMAIL:ide@pdx.edu
            Bankit Comment: TOTAL # OF SEQS:451
            Bankit Comment: GAP: est. length - alignment

            ##Assembly-Data-START##
            Assembly Method       :: MetaSPAdes v. 3.12; IDBA-UD v. 1.1.1;
                                     MEGAHIT v. 1.0.3
            Sequencing Technology :: 454; Illumina
            ##Assembly-Data-END##
FEATURES             Location/Qualifiers
     source          1..3244
                     /organism="Crucivirus-192"
                     /mol_type="genomic DNA"
                     /isolate="001637_virusbaton"
                     /isolation_source="Water"
                     /country="France"
                     /collection_date=2013
     CDS             1..1119
                     /codon_start=1
                     /product="Putative Replication Associated Protein"
                     /translation="MLLIGNANVLIKKLRRHVFVLPNYTQEDIDKLNMINPNLCDYII
                     WGYEICPTTRTKHLQGYVEWNKAQYGNVAKQILGNNRIHLEQVEKNRNANIAYCKKAE
                     TKDPDYNPNFIEIDNIANEQGIVVPNQFKAIYEFISKESDLGIIAQHFPELVIKHSMG
                     IKCIVECTKDTIMKEKLFNKYNNAILRPWQLCLRDELINCQPDDRSIIWYVDPEGNQG
                     KSWMCNYLMCVRNALVLGNAKSSDIAHIWNGEDIVVFDFSRSQESHINYGIIEDIKNG
                     RVFSPKYNGKVKIYMSPHIVCFSNFHPELSKMSQDRWDIRNPQDGIIQVDIVQDPISN
                     ELNYHDHDIIDVSHDDIPEDNIDDDELHTDELDEFLGL*"
     CDS             complement(1544..3052)
                     /codon_start=1
                     /product="Putative CruV Capsid Protein"
                     /translation="MVARRRQVRRAPARRVPARRAPPRQIVVKEAPMVPKEVTRYAKK
                     TASTGLGMTGRALMRGALGVAGAGIGSLLGPLGTKVGGVLGMMGGNLVDKVAPYAIPF
                     FERIGEITGLGDYAISADVNQNTLLSGRPPKVVNKAHRGETVISNQEFLTDIYTSNTA
                     GNFQIQGYPLNPGLLNSFPLLAAIAANYEEYRFEGLAYEFRSMSADALNSTNTALGSV
                     MMATQYNSVLPAFASKAEMENYEGGVSCKPSKNILHFVECEPAQNVLNELYVRSGSVP
                     SGQDQRFYDLGTMYIATTGFQGANVNIGELWVTYQVGLLKLKMFVSLGNFNSYWRQVN
                     SAYNATTGLLGTGTVTPGYQNMVCSVAVVGGSAGLATYTNGLYFPSSSIKQSYKVTLI
                     WAGAGSSAVRFPSYITVGCFNGAGLAEGTPSVAYDYATPAVGDTAFAMSCALTITTDG
                     LTGVRGFGGTSGGSIAGAYIAFQNSTTVLPTAGNEMEIIVQQVPNAGLAIAW*"
ORIGIN
        1 atgttgttaa ttggcaatgc aaatgtacta atcaagaaat tgcgtagaca tgtcttcgtc
       61 ttaccaaatt acacacaaga agatatagat aaattaaata tgattaatcc aaatctttgt
      121 gactatataa tttggggcta tgaaatttgt cctactacta ggacaaaaca tttacaagga
      181 tatgtagaat ggaataaagc ccaatatggc aatgttgcta aacaaatttt aggcaacaat
      241 cgtattcatc tagaacaagt cgagaagaat cgaaatgcca atattgcata ttgcaaaaaa
      301 gctgaaacta aagatccaga ttataatcct aatttcattg agattgataa tattgcaaat
      361 gaacaaggaa ttgttgtacc caatcaattc aaggctatat acgaattcat ttctaaagaa
      421 agtgatttag gaatcatcgc ccaacacttc cctgaactcg tgatcaaaca ttcaatggga
      481 atcaaatgta ttgttgaatg tactaaagat acaatcatga aagaaaaatt gtttaacaag
      541 tataacaatg caatcttacg accttggcaa ttatgtctta gagacgagct tataaattgc
      601 caaccagatg acagaagtat aatctggtac gtcgatccag aaggcaatca aggcaaatct
      661 tggatgtgca attacttgat gtgtgtgaga aacgcattag tcctaggaaa tgccaaatct
      721 agcgatattg ctcacatctg gaatggcgaa gatatagttg tctttgactt ctcgaggtct
      781 caagaatctc atatcaatta tggtataatt gaagacatta aaaatggtag agtattctct
      841 cccaaatata atggcaaagt caagatttat atgtccccac atattgtatg tttttcaaac
      901 ttccatccag aactatctaa gatgagtcag gacagatggg acataagaaa tccccaagac
      961 ggtattattc aagtagatat tgttcaggat cctatatcca atgaattgaa ttatcatgac
     1021 catgatatca tagatgtttc ccacgatgat atccctgaag acaatattga cgacgacgaa
     1081 ttacacacag atgaactgga tgaattctta ggtctttgaa taataatata agttatatat
     1141 attctataat aacttaaagt ctgtaatact ggcaatccat tggattgcta tactagactt
     1201 taagttttct tttttctcat tcctccagaa caaggagaca aaaaatatct aacactggcc
     1261 catccactca cagctagaaa cggcaatgac gtttgggtca agtaaaaaac attggctggg
     1321 acccggctct ccgattcctc tttaaaatga tttttcccct aattacattg gggcccctaa
     1381 ataattgttg aaggcggatt ctcgagacac cctagccaag tttgtttatc tttaggagaa
     1441 agggaatgga taagtcacgg cccttcccgc ttatgagcga agccgtccaa gtctgttaga
     1501 ccattatatt ttttttgtta tagaatatat attatattat tatttaccat gctattgcca
     1561 atcctgcatt aggtacttgt tgtactataa tctccatttc attaccagct gtcggaagaa
     1621 ctgttgtcga gttttgaaat gcaatatatg caccagcgat agatcctccg gatgtcccgc
     1681 caaaacctct tacacctgtc aatccatctg tcgtaatcgt cagagcacat gacatggcaa
     1741 acgcagtatc tccgaccgct ggcgtcgcat aatcatatgc aacactagga gtgccctcag
     1801 ccaatccggc accattaaaa caacccactg taatatacga tggaaatcga acagcggaag
     1861 atccggcacc agcccatata agagttacct tatatgattg tttaatagac gatgatggaa
     1921 aataaagacc attagtgtaa gttgccaaac cagcacttcc accgaccaca gccacagaac
     1981 aaaccatatt ctgatatcct ggcgttactg taccagtacc taataaccca gttgtggcat
     2041 tataggctga attaacttga cgccaataac tattaaaatt tccaagagat acaaacatct
     2101 taagtttaag aagtcctact tgataagtaa cccaaagttc tccaatattt acattggcac
     2161 cctggaaccc agtagttgca atatacatag ttccgagatc ataaaatctt tgatcttgtc
     2221 ccgaaggtac agatccacta cgaacatata gttcattcaa tacattttga gctggctcac
     2281 attctacaaa atgtaatata ttttttgacg gtttacaaga cactcctcct tcatagttct
     2341 ccatttcagc tttggatgca aaagctggta ataccgagtt atattgcgta gccatcataa
     2401 cagaaccaag ggcagtattt gttgagttca aagcgtccgc tgacattgat ctaaattcat
     2461 aagccaatcc ttcaaaacga tattcctcat aattggctgc tatcgcggca agcaatggaa
     2521 atgaatttaa taaacctgga ttcaacggat aaccttgaat ctggaaattt ccagctgtat
     2581 tcgaagtata aatatcagtt agaaattcct gattagaaat aacagtctca ccacgatgag
     2641 ctttatttac tactttgggt ggcctgccag ataacaaagt attttgattt acatctgcag
     2701 atattgcata atctccgaga ccagtaatct ctccgattct ttcaaaaaat gggatggcat
     2761 acggagctac cttgtcaact aaatttccac ccatcattcc taggacacct ccaacctttg
     2821 ttcctaatgg accaaggagt gatcctatcc ccgcgccagc cactccaagg gcgcccctca
     2881 ttaaagctct accagtcata cctaaacctg tactcgcggt tttctttgca tacctggtca
     2941 cctcttttgg taccatcggt gcttctttaa caacgatctg ccttgggggc gctctcctgg
     3001 caggtactcg gcgagctgga gctctcctca cttgtctacg tcttgctacc atttcaatat
     3061 atatattgaa atttaatatt atttaattat taaataatgg tacaatagta caattttggc
     3121 attatcactc gattagagga taacattgta caagtacaat gttagtacaa aacaataata
     3181 taatattaaa ataatattat attatacata tatatagtac atagtacaaa gtacaataat
     3241 ttat
//
LOCUS       CruV-193                3248 bp    DNA     circular     24-FEB-2020
DEFINITION  Crucivirus-193 isolate 001632_virusbaton.
ACCESSION   CruV-193
KEYWORDS    .
SOURCE      Crucivirus-193
  ORGANISM  Crucivirus-193
            Unclassified.
REFERENCE   1  (bases 1 to 3248)
  AUTHORS   de la Higuera,I., Kasun,G.W., Torrance,E.L., Pratt,A.A.,
            Maluenda,A., Colombet,J., Bisseux,M., Ravet,V., Dayaram,A.,
            Stainton,D., Kraberger,S., Zawar-Reza,P., Goldstien,S., Briskie,J.,
            White,R., Taylor,H., Gomez,C., Ainley,D.G., Harding,J.S.,
            Fontenele,R.S., Schreck,J., Ribeiro,S.G., Oswald,S.A., Arnold,J.M.,
            Varsani,A., Enault,F. and Stedman,K.M.
  TITLE     Direct Submission
  JOURNAL   Submitted (24-FEB-2020) Biology, Portland State University, 1719 SW
            10th Ave, Portland, Oregon 97201, USA
COMMENT     Bankit Comment: ALT EMAIL:ide@pdx.edu
            Bankit Comment: TOTAL # OF SEQS:451
            Bankit Comment: GAP: est. length - alignment

            ##Assembly-Data-START##
            Assembly Method       :: MetaSPAdes v. 3.12; IDBA-UD v. 1.1.1;
                                     MEGAHIT v. 1.0.3
            Sequencing Technology :: 454; Illumina
            ##Assembly-Data-END##
FEATURES             Location/Qualifiers
     source          1..3248
                     /organism="Crucivirus-193"
                     /mol_type="genomic DNA"
                     /isolate="001632_virusbaton"
                     /isolation_source="Water"
                     /country="France"
                     /collection_date=2013
     CDS             1..1269
                     /codon_start=1
                     /product="Putative Replication Associated Protein"
                     /translation="MTLQHRRKVRTLEDYGVLTKSESIHNLDFHYEGGGSLSSVPVGP
                     DDNGNTTTAPPDKPLDSDSKTFISESIEYNACKRKDGLYKEKGPQSKNWCFTNFALDD
                     IQWYDDPNIGFIAYGLETCPTTLKLHHQGFLQLRTKKTLGWIKNNLHASVRFAIMKGN
                     FKQNVDYCSKESRFTKRGEWTGQGQRTDLKDAIKKMVDQTFDIEDEENASLMIKYNKG
                     MLYFQNHLTEKRCKLASKEHFDKTFNVLNINQQFWKSILFYQNRRQILWIVDLTGNLG
                     KSFFSTYMYFYENAFEAENCAGKDLAYAYKGEPYAIFDFERCNEDVINYGALEKLKNG
                     KIFSSKYESTSKRFVPPSVCAMSNFKPNIKMFSIDRWMILTYVDGNLIKYQDGSYIKH
                     INEFDSDDEDNKHFPSENFSDEIEPRDDDE*"
     CDS             complement(1726..3141)
                     /codon_start=1
                     /product="Putative CruV Capsid Protein"
                     /translation="MPRGRSTVQKGLDAAQRDMYYNPTSVPRSRKARAPAKRRPARIS
                     KGGGSKATGNRIRSAGEKLGQFLGNGLGGPAGGIAGSMLGKAGGAMISKIFGHGDYAV
                     SNAESLKENNLVLSNAAQMPQFGTGKVIVNFTHREFLGDVFSSSTASTFKIDSYPINP
                     ALSVTFPWLSGVVGAKFQQYRINGMSFEFRSMSSDALNSTNTALGSVIMSTDYDSADA
                     TFTSKQEMENTEFGVSCKPSVNMIHGIECARAQTPVSELYLRAFDVPSGKDIRLYDMG
                     RFSIASTGCQGTNVNLGELWVSYDIDVFKAIENVPAYLTPFAQYAITGATASAPFGTS
                     RAVPSTVTNAADQIGLTFASANKVQWPVNTDVGATYMIVIDYVGAAAAAFTPPSVTCG
                     NGMYFNTYHDFFAPSSAAVSANIILTYVVQYKGGATPAALPYVNVGNFTWAPAGGIAE
                     ADIWMTQVSGLYGGVYTIQTA*"
ORIGIN
        1 atgactttgc agcatcgtag aaaagttcgt acgttggaag attatggtgt cttaactaaa
       61 agtgaatcca ttcataattt ggacttccac tacgaggggg gcggttcatt atcatctgtt
      121 cccgttggtc ccgatgataa tggtaatact acaaccgctc ctccggacaa gccccttgat
      181 tcagattcta aaacttttat ctctgaatct attgaatata atgcttgtaa gcgtaaagac
      241 ggcctttaca aagaaaaggg tcctcaatcg aaaaactggt gttttactaa tttcgcgcta
      301 gatgatattc aatggtacga tgacccaaat attggtttta ttgcttatgg gctcgaaact
      361 tgcccaacca ctttaaaact ccatcaccaa ggctttcttc aacttcgtac caaaaagact
      421 cttggatgga ttaaaaataa ccttcatgct tctgtacgct tcgcaatcat gaaaggcaat
      481 tttaaacaaa atgtggacta ttgttctaaa gaatcacggt tcactaaacg aggtgagtgg
      541 accggtcaag gtcaacgtac tgaccttaaa gacgccatca aaaagatggt agatcaaact
      601 tttgacattg aagatgaaga gaatgcttct cttatgatta aatacaataa aggcatgctt
      661 tatttccaaa atcacttaac tgaaaagcgc tgtaaacttg cgtctaaaga acactttgat
      721 aaaacattta atgtattaaa tattaatcaa caattttgga aaagtatact tttctatcaa
      781 aatcgacggc aaattctttg gattgttgat cttactggta atcttggtaa atcatttttc
      841 tctacctaca tgtattttta tgaaaacgca tttgaggccg aaaactgtgc aggcaaggat
      901 ttagcctacg cttataaagg cgaaccatac gcaatctttg attttgagcg ctgtaatgaa
      961 gatgtcatta actatggcgc tcttgaaaaa ttaaagaatg gtaagatatt tagttctaaa
     1021 tatgaatcta ctagtaagag attcgttcca ccttctgttt gtgctatgtc taacttcaaa
     1081 ccgaatatta aaatgttctc tattgaccgt tggatgatac tcacttatgt cgatggtaat
     1141 ctaataaagt atcaagacgg atcttacatc aaacacataa atgaatttga ctcagatgac
     1201 gaagacaata agcactttcc ttcagaaaac ttttctgatg aaatcgaacc acgagatgat
     1261 gatgagtaga gggatgggac gggccctaat tccgttcgct aacgctgcct ccatagatgc
     1321 cctgatcgtt ctactgctcg cgacgccgct cgctccgaga ttaaataata aagttttttt
     1381 gaattttatt attttttttg aatatttgct tttaataccc tctgggacgc gtacccttgc
     1441 ggctactgct tatttacaat taatatatct caaaacatct cgtcctttca tctcggtggt
     1501 tgatcctcgg cgcagctaaa aagtcaccac ttgatttcat taataacaag tattaatggc
     1561 atcttcgaca gattttacac aacttcttct tttgtgaatc ttttgtatct gtcttcgtca
     1621 attggctttt ttggccaatt ggatgatcag tttttttgac tgatctggtg acttttggtc
     1681 gatactacat atatttttat acatagtatc gttattttat gttctttagg ctgtttgtat
     1741 tgtatataca ccaccataaa gtccgctaac ttgggtcatc catatatcgg cttctgcgat
     1801 tcctcctgct ggtgcccatg tgaagttacc tacgttcaca tatggtagtg ctgccggtgt
     1861 tgcaccacct ttatattgaa caacataagt taatattata tttgctgaga ctgctgcgct
     1921 gcttggtgca aaaaaatcat gatatgtatt aaaatacatc ccgtttccgc atgttactga
     1981 cggtggtgta aatgctgcgg ctgccgctcc tacataatct attactatca tatacgtcgc
     2041 gcctacgtct gtattaactg gccattggac cttattagct gatgcaaatg tcaagcctat
     2101 ttgatcagcc gcgtttgtca ctgttgaagg tactgcacgc gatgttccaa atggcgctga
     2161 agctgtcgca ccagttatcg cgtattgcgc aaacggggtt agatatgctg ggacgttttc
     2221 aattgctttg aatacatcga tatcatacga gacccacaat tcaccaaggt tcacattggt
     2281 tccctgacac cccgttgacg caatactgaa tcgtcccata tcgtacaagc gtatatcttt
     2341 ccctgacggt acatcaaacg cccttaagta caattcggag acgggtgttt gtgctcgagc
     2401 gcattcaatt ccatggatca tgttcactga aggcttgcat gacacaccaa attcagtgtt
     2461 ttccatctcc tgtttagatg taaaagtcgc atcagcagaa tcatagtctg ttgacataat
     2521 tactgaccct aatgctgtat ttgtggagtt caatgcatca ctactcatgc ttcgaaattc
     2581 gaatgacatt ccgttgattc tgtattgttg aaactttgct ccaaccactc cactcaacca
     2641 cggaaacgtt acacttaatg ccgggttgat tggataactg tcgatcttga aggtagatgc
     2701 agtactcgaa ctgaatacgt ctcctaaaaa ttccctatgg gtaaagttta cgatgacctt
     2761 tcccgtacca aattgcggca tttgcgctgc attggacaac acaaggttgt tctctttcag
     2821 agattcggcg ttcgagactg cataatcacc atgtccaaaa atcttgctta tcattgctcc
     2881 accggctttg cctagcatgc tacctgcgat accacctgct ggacctccca gtccattgcc
     2941 aagaaattgc cctagtttct cacctgcgct ccggatacgg ttgcctgtgg ctttactacc
     3001 tcctcctttg ctgattcgag cgggccttct tttggctggt gcccgagctt ttctgcttct
     3061 tggcaccgat gtagggttat aatacatatc acgttgtgct gcatctaacc ctttttgaac
     3121 agtgcttcta cctctcggca ttttaagttt gtatgatata tatactatag ataataaaaa
     3181 ttccttaaaa tgtttacttt tccacccagt ttaaaactaa tatctatagc tagaatataa
     3241 acttaaag
//
LOCUS       CruV-194                3250 bp    DNA     circular     24-FEB-2020
DEFINITION  Crucivirus-194 isolate 001628_virusbaton.
ACCESSION   CruV-194
KEYWORDS    .
SOURCE      Crucivirus-194
  ORGANISM  Crucivirus-194
            Unclassified.
REFERENCE   1  (bases 1 to 3250)
  AUTHORS   de la Higuera,I., Kasun,G.W., Torrance,E.L., Pratt,A.A.,
            Maluenda,A., Colombet,J., Bisseux,M., Ravet,V., Dayaram,A.,
            Stainton,D., Kraberger,S., Zawar-Reza,P., Goldstien,S., Briskie,J.,
            White,R., Taylor,H., Gomez,C., Ainley,D.G., Harding,J.S.,
            Fontenele,R.S., Schreck,J., Ribeiro,S.G., Oswald,S.A., Arnold,J.M.,
            Varsani,A., Enault,F. and Stedman,K.M.
  TITLE     Direct Submission
  JOURNAL   Submitted (24-FEB-2020) Biology, Portland State University, 1719 SW
            10th Ave, Portland, Oregon 97201, USA
COMMENT     Bankit Comment: ALT EMAIL:ide@pdx.edu
            Bankit Comment: TOTAL # OF SEQS:451
            Bankit Comment: GAP: est. length - alignment

            ##Assembly-Data-START##
            Assembly Method       :: MetaSPAdes v. 3.12; IDBA-UD v. 1.1.1;
                                     MEGAHIT v. 1.0.3
            Sequencing Technology :: 454; Illumina
            ##Assembly-Data-END##
FEATURES             Location/Qualifiers
     source          1..3250
                     /organism="Crucivirus-194"
                     /mol_type="genomic DNA"
                     /isolate="001628_virusbaton"
                     /isolation_source="Water"
                     /country="France"
                     /collection_date=2013
     misc_feature    join(3241..3250,1..16)
                     /note="stem-loop"
     misc_feature    join(complement(1..8),3250)
                     /note="nonanucleotide"
     misc_feature    1..9
                     /note="nonanucleotide"
     CDS             215..1873
                     /codon_start=1
                     /product="Putative CruV Capsid Protein"
                     /translation="MTTYMLSKSDYAKLSATGKKKMKASMAKKVRSVGPRSMARKVKG
                     HGDYYVGPGEKRTSDYTQNLGSRIGKLALGPVGQYVGGAAHALFKKVTGFGDYKVSQN
                     TMLSGADPPQFASSGRGTLIRHREYIADITGSVAFAVTSYPINAGLNSSFPWLSQLAA
                     NFEQYIVRGMIFEFKSTSADALNSTNTALGAVVMATEYNSVNPNFSSKAQAENHEFST
                     SAKPSSSFMHPIECARSETSISALYIRTGTVPTGTDQRMYDLGNFQIATVGMQAAAVI
                     GELWVTYDVELLKPRLYDSVGLNVYEDHYRCTTVSAGTYFGTGRTLATGSNLGSSVSG
                     SVITFPADVSPQYYIINYLCLGNSTVLGTAIALSYGTNVIGVSYYSNDAFFQLNPAAA
                     STTNLQFFTAKVVVGGSSSITGNTLTISAGTLPTGITSTDLTIVGANPLVVTKSRTKS
                     RFLDDDEGDSSDEEELLVAMKRMKHAKMRKELAEWKASAHVVSEGKEEKKEKLSIDSE
                     DDDDSAMLAEFLASKAKRKVVVVGAGVPEPPVSVPTPSRVSSKK*"
     CDS             2143..3192
                     /codon_start=1
                     /product="Putative Replication Associated Protein"
                     /translation="MAAHIEKKKDKKLPSNDFVNGQYCVFTSWCLQAPVWNEEKMKYL
                     VFQREQCPETERKHWQGYVEMKSSTRHLALKNLLGITDPIEDPVTMNKIPGGWKALQR
                     RGNALQASSYCMKSESRVKPDVLPTEFGEAPSKTHKGSGHRTDLEAPSKMIIEGKSLK
                     EVALLHPVAIVKYARGFEALHRLTDDVKPVDPEIKLRPWQESLVKVIDAGFVKRQILW
                     VWSLKSATGKSTMRDYLLFKYGIDKVLPGSWTIADMLYAYQKHGLVIFDLPRKQELHN
                     THLAVLESMSDGGIKMSTKYESKPKLIRSVILVFANIPPPEAEMPERCVSIWIDTDEF
                     KAEYTAAKKALTDDD*"
ORIGIN
        1 tattattacc ccccacctca caacgacaac acgttgtgtc agaaatatta attaattaat
       61 gtttctgtgg aacattatat atgttcttgg ttgagcccgt agggcgaggc catgcccatg
      121 gcgcgtcgag cttgcgagat gtcgcagcca tgggcggtgt accgcaggtg ctgttttctg
      181 agattaatat ttattattat tttagaagaa tccgttgact acatatatgt tgtccaaatc
      241 ggattacgct aaactctctg ccactggcaa gaagaaaatg aaagccagta tggctaagaa
      301 agttcgctct gtcggacctc gttctatggc ccgtaaagtt aagggccatg gcgactatta
      361 tgtcggccct ggagaaaaac gaacttctga ttatactcag aatttgggtt cacgtattgg
      421 caagctggcc ttaggtcccg tcggccaata tgtcggcggt gccgcccatg cgttattcaa
      481 aaaagtaact ggttttggag actataaggt ctcgcaaaat accatgttat ccggtgcaga
      541 ccctcctcag tttgcatcat ctggtcgcgg tactcttatc cgacaccgtg aatatatcgc
      601 tgatattaca ggttctgttg catttgctgt tacctcttat cccattaatg ccggattaaa
      661 cagctcgttt ccgtggttat ctcaacttgc cgccaatttc gaacagtata ttgtgcgtgg
      721 aatgatcttt gagtttaagt ccacttcggc cgatgcactt aactctacaa ataccgccct
      781 tggtgcggtc gttatggcta ctgagtataa ctcagttaat cctaactttt cgtctaaagc
      841 ccaagcagaa aatcatgaat ttagcacttc tgctaagcct tcatcaagtt tcatgcatcc
      901 tattgagtgt gctcgttccg aaacctctat ttcagccttg tatatccgca caggaaccgt
      961 acctacaggc actgatcaac gtatgtatga tcttggtaat ttccaaattg ccactgttgg
     1021 tatgcaagca gccgctgtta ttggtgagct ttgggtcacc tacgatgtcg agttgctcaa
     1081 gcctcgcttg tacgattcag ttggtcttaa tgtgtatgaa gaccattatc gttgcactac
     1141 tgttagtgct ggtacttatt tcggcacagg ccgaaccctt gctactggtt ctaatctcgg
     1201 ttcttccgtt tctggtagcg ttattacatt cccagctgat gtatcaccac aatattatat
     1261 tattaattat ttgtgtcttg gcaatagcac tgttctcggt accgctattg ctttatctta
     1321 cggtacaaat gttattggag tttcatacta ttcgaatgat gcatttttcc agttgaatcc
     1381 agccgccgca tcaacaacta atttgcaatt tttcaccgca aaggttgtcg ttggcggttc
     1441 ttcaagtatt actggtaata ctcttactat tagcgctggt actttgccca caggcattac
     1501 cagtacagat cttactattg ttggcgctaa tccccttgtt gtaactaaat cacgaactaa
     1561 gtctcgattt cttgatgatg acgagggtga ttctagtgat gaggaagaac ttcttgtagc
     1621 tatgaagcgg atgaagcatg ccaaaatgcg aaaagaactg gcagaatgga aagcttctgc
     1681 tcatgttgta tctgagggca aagaagagaa aaaagagaaa ctctctattg actctgagga
     1741 tgacgatgat tccgctatgc ttgcagagtt cttagcctca aaggctaaac gtaaggttgt
     1801 tgttgttggc gctggtgttc cagagcctcc cgtatctgtt ccaactcctt ctcgtgtatc
     1861 ttcaaaaaag tgagtcctcc aggcgggtta gcctggtagt gtttttttta ttgttatttg
     1921 ttctgagaaa ccccgcgatg cccgaagggc tcaggggttt cgatcccaaa acccaaaaca
     1981 attcgttggc aagcaaggag caacgcagcc cgaagggcga gtatgcgacg cgcagtcctc
     2041 cggccaagcc cgaagggcgt tggcgggccg gagcgctaat gttctatatt tatttattat
     2101 ttattttttt agatgggtat gttatagatt gcatacaaca ctatggctgc ccatattgag
     2161 aaaaagaaag ataagaagtt accatcaaat gattttgtta acggtcaata ctgtgtattt
     2221 acttcttggt gtttacaggc tccagtttgg aacgaagaaa aaatgaagta tttagttttt
     2281 cagcgtgaac aatgtcctga aactgagcgt aagcattggc aaggttatgt agagatgaaa
     2341 tcatcgacac gtcatcttgc tctgaagaat ttgttaggaa ttaccgatcc tattgaggat
     2401 ccagtaacaa tgaacaaaat tcctggcggt tggaaggcac tgcagcgtcg cggaaatgct
     2461 ctccaagcca gtagttattg tatgaaatca gaatcccggg ttaaaccaga tgttttacct
     2521 actgagtttg gagaagctcc ttccaaaact cataagggat ctggtcatcg tacagattta
     2581 gaagctcctt ctaaaatgat tattgaaggt aaatcattaa aggaagttgc tttgcttcat
     2641 cccgtggcta ttgttaaata tgcgcgagga tttgaggcat tacatcgact tactgatgat
     2701 gttaaacctg ttgatcctga gattaagtta cgaccttggc aagaatctct tgttaaggtt
     2761 attgatgctg gttttgttaa gcgacaaatc ctttgggttt ggtctttaaa atctgctact
     2821 ggtaaatcta caatgcgtga ttatttgttg ttcaaatatg gcattgataa ggttttgcct
     2881 ggttcttgga ctattgctga tatgctgtat gcatatcaaa agcatggtct tgttatattt
     2941 gatttgcctc gtaagcagga gctccataat acgcatctcg ccgtgttgga atctatgtct
     3001 gatggcggca taaaaatgtc tactaagtat gaatcaaagc ctaagctgat tcgttctgtt
     3061 atattggtat ttgcaaatat accgcctcct gaagcagaaa tgccagagag gtgtgtgagt
     3121 atttggatag atactgatga gttcaaagct gaatatactg ctgctaaaaa agctcttact
     3181 gacgatgatt aggtgggggt tacagaactg taataacaga tccaattttt tggatgtggc
     3241 atggaaggtc
//
LOCUS       CruV-195                3256 bp    DNA     circular     24-FEB-2020
DEFINITION  Crucivirus-195 isolate LSCO_103359.
ACCESSION   CruV-195
KEYWORDS    .
SOURCE      Crucivirus-195
  ORGANISM  Crucivirus-195
            Unclassified.
REFERENCE   1  (bases 1 to 3256)
  AUTHORS   de la Higuera,I., Kasun,G.W., Torrance,E.L., Pratt,A.A.,
            Maluenda,A., Colombet,J., Bisseux,M., Ravet,V., Dayaram,A.,
            Stainton,D., Kraberger,S., Zawar-Reza,P., Goldstien,S., Briskie,J.,
            White,R., Taylor,H., Gomez,C., Ainley,D.G., Harding,J.S.,
            Fontenele,R.S., Schreck,J., Ribeiro,S.G., Oswald,S.A., Arnold,J.M.,
            Varsani,A., Enault,F. and Stedman,K.M.
  TITLE     Direct Submission
  JOURNAL   Submitted (24-FEB-2020) Biology, Portland State University, 1719 SW
            10th Ave, Portland, Oregon 97201, USA
COMMENT     Bankit Comment: ALT EMAIL:ide@pdx.edu
            Bankit Comment: TOTAL # OF SEQS:451
            Bankit Comment: GAP: est. length - alignment

            ##Assembly-Data-START##
            Assembly Method       :: MetaSPAdes v. 3.12; IDBA-UD v. 1.1.1;
                                     MEGAHIT v. 1.0.3
            Sequencing Technology :: 454; Illumina
            ##Assembly-Data-END##
FEATURES             Location/Qualifiers
     source          1..3256
                     /organism="Crucivirus-195"
                     /mol_type="genomic DNA"
                     /isolate="LSCO_103359"
                     /isolation_source="Chirominidae"
                     /country="New Zealand"
                     /collection_date=2013
     CDS             1..1140
                     /codon_start=1
                     /product="Putative Replication Associated Protein"
                     /translation="MEQTQLSKTTNQPAKHWVFTLNNYQQDDLSATEKLSEECEYLVY
                     GVELGESGTPHLQGYFCLKTKKRRTAIAKILTRAYLQPKSPKSTVKQAVDYCKKEGNF
                     KEFGRLPEEKHAAGNQANLDKWFTIKQLAQKGELESIEPEVFIKHYSTLKRIKADARP
                     IPADLDWTVEPPNEWIYGPTGTGKSRTARAENPSCYLKMANKWWENYEDEAVVLIEDV
                     GQSHIWMGDFLKIWADRYGFRAEIKHLSLVLRPKKIVITSNYHPSEIWSDPNVLEPIL
                     RRFKVRHLLTPAWGTTKSPLKRDVSQPNLPAKKRRAELEPKPVPKWRWDVETSTLLPA
                     TKQGVLPYACTAKDAITLSEDENDEMAWSDTGSTCCTESLEDIYE*"
     CDS             1584..3014
                     /codon_start=1
                     /product="Putative CruV Capsid Protein"
                     /translation="MDSYDIRSPGKVTGAYKRPRPGKTPYVPKTYKNPPIPYEVSAPG
                     RFWRISKKSKPAVYWKKRYWRRRITGRGAYVMDPGDSVGRRFGGFIGSKLGEFVGGYG
                     QALITGTGAYTIKKNVMMDGNLPQMVNMPSGGGTIIRFQEYLGDVISGGTLTAGSSVF
                     DVQSYLINAANPATFPFLSQIASNYEQYEIEGMIFQFRSTSANALNSTNTALGTVIMG
                     TQYDVADAPFASKSEMLNHQYSVSVKPSDSTLHMIECAPNQTTTSLLYTLSSEQTPAG
                     QDSRFYHLGRFSIATQGFQAANVVIGELHVTYQVRLLKPKLSDLLGNNFLSTYITTTG
                     YSSALPLGSAVQLTPNSFNNACTLDLPNRSLSFPNASVGYFRILITWNGTTGVGFQGP
                     GIQSCTNGAPQFNIGAIPLCYPQNGTGGSGITQCAIAFGLKTSGNGDACVLKLDGFGA
                     LPTGGIYPSNFCVIRVTQTNSATGAF*"
ORIGIN
        1 atggaacaaa cacagctctc aaaaaccacc aatcagcctg ccaaacactg ggtcttcacc
       61 ctgaacaact atcaacagga cgatctttcg gcaacggaga aactctccga agaatgtgag
      121 tacctcgtgt acggggtaga actgggggag tctgggaccc ctcatctcca aggttatttt
      181 tgtttgaaaa caaaaaaacg gcgaacggca attgcgaaga tattgacgcg agcctactta
      241 caacctaaat caccaaagtc gactgttaaa caggctgtag attactgcaa aaaagaaggt
      301 aacttcaagg aatttgggag attgccagag gagaagcacg ccgcaggaaa ccaggcgaac
      361 ctcgacaaat ggtttacaat taaacagtta gcccaaaaag gcgaacttga atcaatcgaa
      421 ccagaggtct tcatcaaaca ttactctact ctcaagcgta tcaaagcgga tgctaggcca
      481 attccagcag accttgattg gacggtagaa cctccaaacg aatggatcta tggaccgaca
      541 gggacaggca aatcgcgtac agcgcgcgca gagaaccctt cctgttacct taaaatggcc
      601 aacaaatggt gggaaaacta cgaggatgaa gcggtggtgc taatcgaaga cgtggggcag
      661 tcacacatct ggatgggcga ctttctaaaa atctgggctg atcgatacgg gtttcgagca
      721 gaaatcaagc atttatcttt ggtactacga ccaaaaaaaa ttgttataac aagcaattac
      781 cacccgtctg agatttggtc tgacccaaat gttcttgaac caattcttcg cagattcaag
      841 gtcagacacc tactcacccc agcatgggga acaaccaaat caccgttaaa aagagatgtc
      901 tcacaaccaa acttgccagc gaaaaaaaga agagctgaac tcgaaccaaa gccagtgcca
      961 aaatggcgat gggatgtgga gacaagcact ttattgccag caactaaaca aggtgtactt
     1021 ccttatgcgt gcactgctaa ggacgcaatc accttgtctg aagacgagaa tgacgaaatg
     1081 gcctggagcg acacagggag cacatgttgt actgaaagtc ttgaggacat ttacgaataa
     1141 ataaattgtg taaagaaaat ttgtattttt ttccggaata aggcacatcc taaaaattag
     1201 gaaataaaaa gcatgtccat taatcgccgt acatctccgg gcgaagcccc ctacacctgg
     1261 agggggtccg gacgaggacc cccgtcaaac gacgcgcgag gggccccacg gggacgcgcg
     1321 gacaacacta acataaacaa aaacaataaa tatatcatag gaaaaaaacg ctcgaagaga
     1381 atcaacacaa tagcgccggg ggttaaaccc gggaacagct gtgttgtcaa cggccctaga
     1441 ctttgaggaa atgtaatttc cattattacc tagggacgtt ggaacttaga acgttagaac
     1501 tcgttctcac gtgacatttt gttccagaac accgttatca ctataaaagg aataatggtg
     1561 gccgaaggcc attattattc cgcatggact cgtacgacat acgttcacca ggcaaagtaa
     1621 cgggagccta caaacggcca cgccccggta aaacaccgta tgttccaaaa acatacaaaa
     1681 atccaccgat cccttacgaa gtatccgcgc ctggaagatt ctggaggatt tcgaaaaagt
     1741 cgaaacccgc tgtttactgg aagaagcggt attggcgacg cagaattacc ggacgcggag
     1801 cgtacgtaat ggacccagga gactcagttg gtaggcgctt tggtgggttt attggttcaa
     1861 aacttggtga attcgttggt ggctacggcc aagctttaat tacaggaaca ggtgcgtaca
     1921 cgatcaaaaa aaacgttatg atggacggta acttacccca aatggtaaat atgccgtccg
     1981 gcggaggaac cattattcgc tttcaagaat atttaggtga cgttatttcc ggtgggactc
     2041 taacagcagg ctcatcggtt tttgacgtac agtcgtattt aatcaacgcc gcgaacccgg
     2101 caacgtttcc attcctgtcc caaatagcaa gtaattacga gcagtatgaa atcgaaggaa
     2161 tgattttcca attcagaagt acttccgcta acgctttaaa ctctacaaat acagcacttg
     2221 ggacagtcat catgggaacg caatacgacg tggcagatgc accttttgca agcaaaagtg
     2281 agatgctaaa tcatcaatac tcagtttcgg tgaagccgag cgacagcaca cttcatatga
     2341 tagaatgcgc acctaaccaa acaaccacct cattattata tactttgtct tcagaacaaa
     2401 cccctgcagg gcaagattct cgattttatc acctcgggag attctccatc gcgacccagg
     2461 ggtttcaggc cgccaatgtc gtaattggcg aactgcatgt gacataccag gtacgcttac
     2521 ttaaaccaaa attaagcgat ttacttggaa ataacttctt gtcaacatac ataaccacaa
     2581 cgggttattc aagtgcattg cctttaggga gtgcggttca attgacgcca aactcgttca
     2641 acaacgcttg tacactagat ttacccaacc gttcactttc attcccgaat gcttccgtag
     2701 gatattttcg aatattaatt acatggaatg ggactacagg tgtgggtttc cagggaccag
     2761 gaattcagag ttgtacgaac ggggcacccc aattcaatat tggcgcaatt ccattatgtt
     2821 atccccaaaa cggaactgga ggttcaggta tcacgcagtg tgcaattgct tttgggttaa
     2881 aaacaagtgg taacggagat gcttgtgtcc tcaaactcga tgggtttggg gcactaccga
     2941 caggtggtat ttatcccagt aacttttgtg taatacgagt tacacaaact aactctgcaa
     3001 caggagcatt ttaaataaaa tattgttctg gaaaggcgtt tagcacagca cgtatcatag
     3061 tcaataagta accgttaggt cttaagttca aatccgggag aagtcggttt ttttaatcaa
     3121 aaatagattt atcatcctgg tacacctacc gggaggacgg tctacacgtg gagcaaacgg
     3181 agtaaacgta gtgccacgta actcctttaa taaaactgac tccggaacat gtgattcaat
     3241 tataatcaag ctcaaa
//
LOCUS       CruV-196                3267 bp    DNA     circular     24-FEB-2020
DEFINITION  Crucivirus-196 isolate 120_168_SvL2_3298.
ACCESSION   CruV-196
KEYWORDS    .
SOURCE      Crucivirus-196
  ORGANISM  Crucivirus-196
            Unclassified.
REFERENCE   1  (bases 1 to 3267)
  AUTHORS   de la Higuera,I., Kasun,G.W., Torrance,E.L., Pratt,A.A.,
            Maluenda,A., Colombet,J., Bisseux,M., Ravet,V., Dayaram,A.,
            Stainton,D., Kraberger,S., Zawar-Reza,P., Goldstien,S., Briskie,J.,
            White,R., Taylor,H., Gomez,C., Ainley,D.G., Harding,J.S.,
            Fontenele,R.S., Schreck,J., Ribeiro,S.G., Oswald,S.A., Arnold,J.M.,
            Varsani,A., Enault,F. and Stedman,K.M.
  TITLE     Direct Submission
  JOURNAL   Submitted (24-FEB-2020) Biology, Portland State University, 1719 SW
            10th Ave, Portland, Oregon 97201, USA
COMMENT     Bankit Comment: ALT EMAIL:ide@pdx.edu
            Bankit Comment: TOTAL # OF SEQS:451
            Bankit Comment: GAP: est. length - alignment

            ##Assembly-Data-START##
            Assembly Method       :: MetaSPAdes v. 3.12; IDBA-UD v. 1.1.1;
                                     MEGAHIT v. 1.0.3
            Sequencing Technology :: 454; Illumina
            ##Assembly-Data-END##
FEATURES             Location/Qualifiers
     source          1..3267
                     /organism="Crucivirus-196"
                     /mol_type="genomic DNA"
                     /isolate="120_168_SvL2_3298"
                     /isolation_source="Water"
                     /country="Norway"
                     /collection_date=2012
     CDS             1..1344
                     /codon_start=1
                     /product="Putative CruV Capsid Protein"
                     /translation="MPAAPKKKYYKKKTTTMAAKPVTRKRAPNYVRQARRSYKGADTG
                     IQYDYPGVGARIGGGIGSIFGPAGTALGSLLGHGAQSLVKHVTGFGDYMIEQNSLMPG
                     ALSPPIINNSKKPSSGVIVRHREYITDITATTGFTTTVYPINAGILATFPWLSQIASS
                     FEEYCIHGMLFEFKSMSSDAVLSTAANTALGTVIMATQYNALNPNFTDKRQMENYEFC
                     NSAKPSESFIHPVECKGSETPVKCLYVRTAPVSTGDLRLYDLGNFTIATQGMQVAGGV
                     IGELWVTFEIEFYKPKLLEGQNLLTDHFILTAVSNTRPLGAALTTEPVPGSAIGTVID
                     VDGSGLTTLNFPFSIVDGTFMVDYSVRGTATAIVPPAVSFSTNCIVGPNLFENNTIPS
                     QLVRAGTTTSVFMQSFLVTITGPNARIIFGAAGILPAAVVAGDLFIYQINPEIEL*"
     CDS             complement(1790..2887)
                     /codon_start=1
                     /product="Putative Replication Associated Protein"
                     /translation="MNKKVIANLAQNGSKKMAVEGNTIPTAPAKRAYKKKDSNQVKEK
                     RVNPGKSWCFTWNNYKKEDIDKLISVFANTGSNSYTYIFGKEIGENSLIPHLQGFVVC
                     EKKIRPKEHFKLDKEIHWEPMKGSIESNMTYCTKEGEYYTSKHLVVPIELLSRHEREK
                     KLDLIKQSDFFNWQNELVNILKNKPSKRSLYWIYSKEGKTGKTEFAKWLSFHMNAIPL
                     NGKKNDILYCAAEFESNIYIYPLSRTMEDFVSYDSLESIKDGFYMCGKFESKPICRPN
                     PHVIIFANFRPDKSKMSKDRWNIWNINRRILNSELNKFNKMKNLNIEHCDDGDSGDIE
                     NNTNNSDISEGEIMNDDLISIGNDQIILPEF*"
     misc_feature    2808..2836
                     /note="stem-loop"
     misc_feature    2818..2826
                     /note="nonanucleotide"
ORIGIN
        1 atgcccgcgg ctccaaaaaa aaagtattat aaaaaaaaaa caacaactat ggctgctaaa
       61 cctgttactc gtaaaagggc acctaattat gtgagacagg ctagacggtc ttataaaggt
      121 gcagacactg gtattcagta tgattatcca ggtgttggtg cacgtattgg cggtggtatt
      181 ggttctattt ttggacctgc aggtactgca ttaggttcat tattaggaca tggggcacaa
      241 tcgttggtta agcatgtgac aggttttggt gattacatga ttgaacaaaa ttcattgatg
      301 ccaggtgcat tgtcacctcc tattattaat aattcaaaaa aaccatcatc tggtgtaatc
      361 gtacgtcata gggagtatat aacagatata acagccacta cgggttttac aactactgtt
      421 tatcctataa atgctggtat tttggcaaca tttccatggt tatctcaaat tgcttcatca
      481 tttgaagaat attgtattca tggtatgttg ttcgaattta agagcatgag ttctgatgct
      541 gttttatcaa cagcagcaaa tacagctctt ggtaccgtca ttatggctac acaatataat
      601 gctttaaatc ccaatttcac tgacaagcgt cagatggaaa attatgagtt ttgtaattct
      661 gcaaaacctt ctgaatcatt tatacatcct gtagagtgta aaggctcaga aacacctgta
      721 aagtgtttat acgttagaac tgctccagtt tctacgggtg atctgagatt atatgatcta
      781 gggaatttta caatagctac acaaggtatg caagtagctg gtggagttat aggtgagctt
      841 tgggtcacat ttgaaattga gttttataaa ccaaaattat tggaaggaca aaatctcctt
      901 acagatcatt tcatattaac tgctgtaagc aatacgagac ctttaggtgc tgcattgact
      961 acagaaccag ttccaggttc agctatagga actgttatag atgtagatgg atctggacta
     1021 acaacgttaa attttccatt ttctatagta gatggaacat ttatggttga ttattctgtg
     1081 agaggaacag cgactgcaat agttcctcct gctgtatcat tttctacaaa ttgtatagtg
     1141 ggaccgaact tatttgagaa taatacaatc ccaagtcaac ttgttagagc aggtacaact
     1201 acatctgttt ttatgcaatc atttttggtt acaattactg gacctaatgc acgaataata
     1261 tttggtgcag caggtatttt accagcagcg gtggtagcag gtgatttatt tatttatcaa
     1321 attaatcctg aaattgagct ataaattata tttaattgaa atagtgtagc gctacttatg
     1381 gtgcaaagag caacgataaa aataaaataa tctaaataaa tatgtaatat ttctttaggt
     1441 catttaaaaa ttaaatttaa atttaaattt ataaaataaa ttaaataatc caatataact
     1501 atatccgata tatatctaca aatctgaaag atttgccgaa atggaatttc ggaatttgag
     1561 atagaaacaa aaatactcct ttagagatga gcggtacgcg aatcgtgaga tttttgattt
     1621 cgtatatcga gaagagcttt agctcagaac atctaattat atcaattata tagttatatt
     1681 taatagaaat agtgtagcgc tacttatggt gcaaagagca acgataaaga taaatagttt
     1741 aattatttaa ttaatttata gtaattaatt aaaattgtaa aatataaaat taaaattctg
     1801 gaagaattat ttggtcattt cctatgctga ttagatcatc attcattatt tctccttcag
     1861 aaatatctga attatttgta ttattttcaa tatcaccact atcaccatca tcacaatgct
     1921 caatatttaa atttttcatt ttattgaatt tatttaattc actatttaaa attcttctat
     1981 ttatgttcca aatattccat ctatctttgg acattttaga tttatcaggt ctaaaatttg
     2041 caaagattat gacatgtgga tttggtctac aaattggttt tgattcaaat ttaccacaca
     2101 tataaaatcc atctttaatt gattcaagtg aatcataact aacgaaatct tccattgttc
     2161 tacttaatgg ataaatatat atattacttt caaattctgc agcacagtat aatatatcat
     2221 tttttttacc attcagtggt atagcgttca tgtgaaagct taaccattta gcaaattctg
     2281 ttttaccagt tttgccttct ttactatata tccagtataa tgatctttta ctgggtttat
     2341 tttttagtat atttactaat tcattttgcc aattgaaaaa atcagattgt tttatcagat
     2401 ctaatttttt ttctctttca tgtcttgata atagttcaat tggcactact aaatgtttac
     2461 ttgtataata ttcaccttct ttggtgcaat atgtcatatt gctttcaatt gaacctttca
     2521 ttggttccca atgtatttct ttatctaatt taaaatgttc ttttggtcta attttttttt
     2581 cacatacaac aaaaccttgc agatgtggta taagagaatt ttcgccaatt tctttgccaa
     2641 atatataggt ataagaattg gaaccagtat ttgcaaaaac agatattaac ttgtcgatat
     2701 cttctttttt ataattgttc caggtaaagc accaggattt acctggattt actctttttt
     2761 ctttaacttg gttactatct ttctttttgt atgccctttt cgcgggcgcc gttggaatag
     2821 tattaccttc aacggccatt ttttttgagc cattttgagc caaatttgcg ataacttttt
     2881 tattcatgat taaatatata ttgtataata tttctttaag ccatttaaaa tcgaaattaa
     2941 aattaaaaat atattttgta aaataaaatt taattgaaat agcgtagcgc tacttatggt
     3001 gcaaagagca acgataaaaa taaaatgacc caaataaata tgtaataatt ctttaggtca
     3061 ttttaaataa tattttacaa aataataatt taaaaatatg ttttatcaaa ttgcgagtgg
     3121 actttatgtc ccgaggagta taagttaaat tggtactatt tttggaacat ttttgtataa
     3181 caaaaatttg aacaaaattt ggaacaaata tataatattt ctttaggtca ttttaaattt
     3241 taatttaaat tgtaatatta taatatt
//
LOCUS       CruV-197                3273 bp    DNA     circular     24-FEB-2020
DEFINITION  Crucivirus-197 isolate 001611_virusbaton.
ACCESSION   CruV-197
KEYWORDS    .
SOURCE      Crucivirus-197
  ORGANISM  Crucivirus-197
            Unclassified.
REFERENCE   1  (bases 1 to 3273)
  AUTHORS   de la Higuera,I., Kasun,G.W., Torrance,E.L., Pratt,A.A.,
            Maluenda,A., Colombet,J., Bisseux,M., Ravet,V., Dayaram,A.,
            Stainton,D., Kraberger,S., Zawar-Reza,P., Goldstien,S., Briskie,J.,
            White,R., Taylor,H., Gomez,C., Ainley,D.G., Harding,J.S.,
            Fontenele,R.S., Schreck,J., Ribeiro,S.G., Oswald,S.A., Arnold,J.M.,
            Varsani,A., Enault,F. and Stedman,K.M.
  TITLE     Direct Submission
  JOURNAL   Submitted (24-FEB-2020) Biology, Portland State University, 1719 SW
            10th Ave, Portland, Oregon 97201, USA
COMMENT     Bankit Comment: ALT EMAIL:ide@pdx.edu
            Bankit Comment: TOTAL # OF SEQS:451
            Bankit Comment: GAP: est. length - alignment

            ##Assembly-Data-START##
            Assembly Method       :: MetaSPAdes v. 3.12; IDBA-UD v. 1.1.1;
                                     MEGAHIT v. 1.0.3
            Sequencing Technology :: 454; Illumina
            ##Assembly-Data-END##
FEATURES             Location/Qualifiers
     source          1..3273
                     /organism="Crucivirus-197"
                     /mol_type="genomic DNA"
                     /isolate="001611_virusbaton"
                     /isolation_source="Water"
                     /country="France"
                     /collection_date=2013
     CDS             1..1269
                     /codon_start=1
                     /product="Putative Replication Associated Protein"
                     /translation="MTLQHRRKVRTLEDYGVLTKGEEPPNASTKVFHYEGGGSLAPVP
                     DGPEANGNTTTAPPDKPLSDDIRIYDINEIIEYNSYKRKDGLYKEKGPQSKNWCFTNF
                     ALDDIQFYDDPNIGFIAYGQETCPTTLKLHHQGFLQLRAKKTLGWIKNNLSKTVRFAI
                     MKGNFKQNVEYCSKEGKLIKRGEYTGQGQNTPLLEAVKNIKENKFDFDNDENAVILLK
                     YNKGVQHLINYHKDKEDKKLNKLDFESMFACLNHRQSYWLKLLNQQSRRQILWIHDIT
                     GNLGKSFFSDYLCIFENAFEAENAANKDLAYAYKGQKYVVFDFERSNADVINYGVIEK
                     LKNGKIFSAKYESCSKRFVPPKVICLSNFAPNIKMFSLDRWYILSYRDGHLIKIRNGD
                     YVMHIDDDSDYSEHYPSDNDAAAEVECLEE*"
     CDS             complement(1707..3173)
                     /codon_start=1
                     /product="Putative CruV Capsid Protein"
                     /translation="MREQYIVPRELAAYRKVNRRKEDEYGASFGYVPGPPKRRAPARK
                     KAPARRAPAAKASKGRGSATGNRIRSAGESLGTFLGNGLGGPAGGVAGGMLGKLGGGM
                     ISKIFGHGDYAVSNAGSLKENNLVLSNAAQMPQFGTGKVAVNFTHREFLGDVFSSSTA
                     STFKIDSYPINPGQTLTFPWLSGVVGAKFQQYRINGCSFEFRSMSADALNSTNTALGS
                     VIMSTDYDSADATFTSKQEMENTEYGVSCKPSVNMIHGIECARSQTPVSELYIRAFDV
                     PSGKDIRLYDIGRFSIASTGCQGTNVNLGELWISYDIDVFKAIEQVPGYLTPFATYSL
                     VGMDATHPLGTSRITFGSTASPPFTNLGEDQIGLTFPSVNRVSWPITIQVGAIYLVNY
                     VLVASEAKAITNPVVTCGNGFKFNSTIDLTTPAGGVTTGTSQGVWLLQYQGGATPAAL
                     PFLNIANFTWGVASILEADLIISQVSGNFPGNYDITTA*"
ORIGIN
        1 atgaccttac agcatcgtag aaaagttcgt acgttggaag attatggggt cttaaccaaa
       61 ggtgaagagc ctccaaatgc aagcactaaa gtcttccact acgagggggg cggttcatta
      121 gctccagttc ccgatggtcc cgaagctaat ggtaatacta caaccgctcc tccggacaag
      181 cccctctctg atgatatacg tatatatgat attaatgaaa taattgaata taattcatac
      241 aagcgtaaag acggccttta taaggaaaag ggtcctcaat cgaaaaactg gtgttttacc
      301 aatttcgcac ttgatgatat tcaattctat gatgacccta acattggttt tattgcttat
      361 ggccaagaaa cttgtccaac cactttaaaa ctccaccatc aaggtttctt acagcttaga
      421 gctaagaaaa ctcttggttg gattaaaaat aacctttcta agacggtcag gtttgccatc
      481 atgaaaggca attttaaaca aaatgttgaa tactgctcga aagaaggcaa gcttattaaa
      541 cgaggcgaat acacgggtca aggtcaaaat acacctttat tagaagcagt taagaatatc
      601 aaagaaaata aatttgactt tgataatgat gaaaacgctg ttatccttct taaatataat
      661 aagggcgttc aacatcttat taactatcac aaggacaagg aagataaaaa actaaataaa
      721 ttagattttg aatctatgtt cgcttgtctt aaccatcgac agtcctattg gcttaagctg
      781 ttgaatcaac aatctagacg ccaaatcctt tggattcatg atattactgg taatcttggt
      841 aaatcctttt tttcggatta tctctgtatc ttcgagaatg ccttcgaggc tgaaaatgca
      901 gccaacaagg atcttgctta tgcttataaa ggtcaaaaat atgttgtttt tgattttgaa
      961 agatcgaatg cggacgtcat taactacggt gttattgaga aactcaaaaa tggtaagatc
     1021 ttttctgcta aatacgagtc ttgtagcaag agatttgtac cacctaaagt tatctgttta
     1081 tctaactttg cacctaatat taaaatgttc tcgcttgatc gttggtatat actatcctat
     1141 cgcgatggtc atctcattaa aattcgaaac ggcgattacg tcatgcatat tgacgacgat
     1201 agcgattatt ctgaacatta tccatctgat aatgacgctg ccgctgaagt tgaatgttta
     1261 gaagagtgat gacggccaat tattccgtat gctacgctac ctccataatg gccgtataaa
     1321 ttcataggtg ctcaccctac gccgtgcggg tagggcctcg ctttagatta atttccgagt
     1381 ggcgattttg ggcaaggcga ttttgcaaag tacgatttga caaagtacga tttgataaat
     1441 ttatgttttg aataccctct gggtctaccg cggctgctgt cgcaacgccg attctgacac
     1501 ataacctctt aaatacacac tccttcactg tctaaaaaaa aactcggtgc ggtactcggc
     1561 gaagcttata ctcggcactt aaatatcatc atcgcgtcca atgatggtat tcatgataaa
     1621 tcttaaattt tttatattca tcttcaccaa cggctttaga taaatactta taaaaatatg
     1681 ttataagtat ttctttacat gcaaccttaa gcggttgtta tatcataatt tccggggaaa
     1741 ttaccactga cttgtgatat aattaggtca gcttctaata tcgaagctac accccatgta
     1801 aaattggcta tatttaaaaa tggcagtgcg gctggcgtag cgcctccttg atattgtaaa
     1861 agccatacac cttgcgatgt acccgttgtt acacccccag caggcgtagt taaatcaata
     1921 gttgaattaa acttaaatcc atttccacat gttacaacag gatttgttat tgcctttgct
     1981 tcactggcta caagtacgta attaaccaag taaattgctc caacttgtat tgttatgggc
     2041 caagatactc gattcacgct cgggaaggtc aagcctatct gatcctcgcc taaattcgta
     2101 aatggcggtg atgcggtact tccaaacgtt attcgagatg tacctagggg gtgtgttgca
     2161 tccatcccaa ctaatgaata agtggcaaat ggagtcaagt atcccggtac ttgctcaatt
     2221 gccttaaaaa cgtctatgtc atacgaaatc caaagctctc ctagattcac attggttcct
     2281 tgacatcccg ttgacgctat actgaatctc cctatatcgt acaagcgtat atccttccct
     2341 gacggtacgt caaacgccct aatgtacaat tcggatactg gtgtttgtga tcttgcgcat
     2401 tcaattccat gtatcatgtt cactgaaggc ttacaagaaa caccatattc agtattctcc
     2461 atttcctgtt ttgaagtaaa tgtcgcatcg gcagaatcat agtctgttga catgattact
     2521 gagcctaacg ctgtattagt ggagtttaac gcatcggcgg acatacttcg aaattcaaag
     2581 ctacatccgt taatccgata ctgttggaac ttcgcaccaa ccactcctga caaccacgga
     2641 aacgttaacg tttgtccggg attgatggga tagctgtcga tcttaaaagt acttgcagta
     2701 ctcgaactaa acacgtcgcc taaaaactcc ctatgggtaa agtttacggc gactttgcct
     2761 gttccaaact gtggcatttg agccgcattt gatagcacta ggttattctc tttcagagat
     2821 cctgcgttcg agactgcata gtccccgtgc ccaaatattt ttgatatcat acctcctcca
     2881 agttttccga gcattccacc ggctacacct cccgctggtc ctcctaagcc gttccccaaa
     2941 aatgttccca agctctcacc agcacttcgg attctattac ctgtggctga tcctcgtcct
     3001 ttagacgctt tcgctgctgg tgctcttcta gctggcgctt ttttacgagc tggggctcta
     3061 cgcttggggg gacctggcac atatccaaag gatgcaccgt attcatcttc ctttcggcga
     3121 ttaaccttac ggtatgctgc tagttcccta ggcactatgt attgttctct catttcgaaa
     3181 ttactatagt atactatagg tttttaattt ctgagagaat acttttccac ccagtttgaa
     3241 attaatatcc catactagga tatatattca aag
//
LOCUS       CruV-198                3279 bp    DNA     circular     24-FEB-2020
DEFINITION  Crucivirus-198 isolate 001608_virusbaton.
ACCESSION   CruV-198
KEYWORDS    .
SOURCE      Crucivirus-198
  ORGANISM  Crucivirus-198
            Unclassified.
REFERENCE   1  (bases 1 to 3279)
  AUTHORS   de la Higuera,I., Kasun,G.W., Torrance,E.L., Pratt,A.A.,
            Maluenda,A., Colombet,J., Bisseux,M., Ravet,V., Dayaram,A.,
            Stainton,D., Kraberger,S., Zawar-Reza,P., Goldstien,S., Briskie,J.,
            White,R., Taylor,H., Gomez,C., Ainley,D.G., Harding,J.S.,
            Fontenele,R.S., Schreck,J., Ribeiro,S.G., Oswald,S.A., Arnold,J.M.,
            Varsani,A., Enault,F. and Stedman,K.M.
  TITLE     Direct Submission
  JOURNAL   Submitted (24-FEB-2020) Biology, Portland State University, 1719 SW
            10th Ave, Portland, Oregon 97201, USA
COMMENT     Bankit Comment: ALT EMAIL:ide@pdx.edu
            Bankit Comment: TOTAL # OF SEQS:451
            Bankit Comment: GAP: est. length - alignment

            ##Assembly-Data-START##
            Assembly Method       :: MetaSPAdes v. 3.12; IDBA-UD v. 1.1.1;
                                     MEGAHIT v. 1.0.3
            Sequencing Technology :: 454; Illumina
            ##Assembly-Data-END##
FEATURES             Location/Qualifiers
     source          1..3279
                     /organism="Crucivirus-198"
                     /mol_type="genomic DNA"
                     /isolate="001608_virusbaton"
                     /isolation_source="Water"
                     /country="France"
                     /collection_date=2013
     CDS             join(complement(1..241),complement(3146..3279))
                     /codon_start=1
                     /product="putative ORF"
                     /translation="MAFRRRKFRKSFGRRRTYGRRSFGFGKRRKIAKLYVTNPVTGRE
                     ILLKSLVAEARKNPSKYRSLRTKKKKVKQVTLDANTGQKVIVGEIARIGKKRKLGPLA
                     DIDPYVAPQVLPPLPNGDMPVD*"
     CDS             join(3210..3279,1..245)
                     /codon_start=1
                     /product="putative ORF"
                     /translation="MSAKGPSLRFFPIRAISPTITFWPVLASKVTCFTFFFFVRSERY
                     FEGFFLASATKDFNKISRPVTGFVTYNFAILRRFPNPKDRRPYVRRLPKLLRNLRLRK
                     AI*"
     misc_feature    join(3274..3279,1..17)
                     /note="stem-loop"
     misc_feature    1..9
                     /note="nonanucleotide"
     CDS             706..1722
                     /codon_start=1
                     /product="Putative Replication Associated Protein"
                     /translation="MTKKGFPWTLNNYTEDEVQNLSNTELHEERSVRFMLFVKQIGDE
                     KTPHLQGYTQYFTSVRWTALKQYSKRIHVEKKQMGSAQQNIDYIVLNEKKTNVAEPYS
                     WGTPCLAKGERTDLKVVAETIIKEQLDEKQVALEFPGVYMRYANGIRGLCRQVHKPKP
                     YVRATPLELHIIYGPSGIGKTYAAEQKFIDGNMSAAKISQLNLKGGWWDDYNGEDVIF
                     LDEFRDYCMYPENFLQLVDRMKKLPVKGQFIDCPAKAIWITTPDHPYEWWKKWMSKTS
                     NNWAQLSRRITKLYKMDSREEGLVEISPQDASHCPVIPEIPNVQELAALVPTGPILCK
                     KRKR*"
     CDS             complement(1826..3100)
                     /codon_start=1
                     /product="Putative CruV Capsid Protein"
                     /translation="MSKRKFYDFGGSISRGALSGAGRALGNLIPIPGFGDVGGYLGNL
                     AGSFFHNLTGIGDYKTMGLSPIKRNTLMGINVGGNVSGQGAYTFGSGDRQYVVKNEFI
                     QNISSSIGFQNQSFLIQPGNNVSYPWASNLAPSYNEWEPMGEVWYFKSTSGDALNSQN
                     TALGTVALTCEYNPTDPPYETMSDVLNNIWTVSTKPSCDVALPIECDPNERGVKQFFV
                     RTTQTVGADDSIKWYDLGNVQVCTEGQQTDGVIIGQLWHAYAFVFKKPQQARSVVQRP
                     IFSFGSNVIAPPLLFGSPSGDYTFVNVGMAGCTVDVPGNGIIFSRTCDGLYNVDFRYN
                     NVGTTAFPGLGMTATPGVDLNTFSGGELLTGGTARSYEYSAMISVDGSVPISKEVYFS
                     PNAGPGVNSNLLNASLQVYVTPFSSAILPVIS*"
     misc_feature    2679..2705
                     /note="stem-loop"
     misc_feature    2692..2700
                     /note="nonanucleotide"
ORIGIN
        1 cagtattagc atccaaagta acctgtttca ccttcttctt cttcgtacga agtgaacgat
       61 acttcgaagg attctttctt gcttccgcaa ccaaagactt caacaaaatc tctcgaccag
      121 tcacaggatt tgtaacgtac aacttcgcga tcttacgacg ctttccaaac ccaaaggatc
      181 gacgaccgta tgtacgacgt ctaccaaaac tcttacgaaa cttacgtctt cgaaaagcca
      241 tttaaaatga gaagagagaa aattaaattt caacctcttt ggattcttcc tcctcttcgg
      301 aatcaccacc acccaaacaa cattcacaaa tgatttggaa aggaccaact acttcgccac
      361 ttcgaagata gcacaaaatc gtctctgttt ctccacaatg tgagcaaaca aaaaggcact
      421 gcttaagcac ctttcgttcc atacccaatg taacaccaga accacaagga aaagtcatgg
      481 aagacattta cctataagaa agcaactttt ttttccaaag tccaaaaatt tgtctcgacg
      541 accaagaaaa tttgtctcta ggtacaaaga attgaccgag gaaaattttt ttcaacccag
      601 tacaattaag cttcgcattg tctctaggtt ggggggtaat actacgctcc ttcgtcgctt
      661 gcccccaaca caaaccgctt cgcattttta tcaaaccgct tcgcaatgac caagaaaggg
      721 tttccttgga cacttaacaa ctacacagag gatgaggtcc aaaacctctc caatacagaa
      781 ctacacgaag aacgtagtgt tcgatttatg ctattcgtga aacaaatagg agatgagaaa
      841 actcctcacc tccagggata cacacaatac ttcacgtccg tccgatggac agccctgaaa
      901 caatattcga aacgtataca cgtcgaaaag aagcagatgg gatccgctca acaaaacatc
      961 gactacatag tactcaatga aaagaaaacc aacgtagcgg aaccgtactc atggggaaca
     1021 ccttgtctag cgaaaggcga aagaacagac ctgaaggttg tagcagaaac catcatcaaa
     1081 gaacaattag acgagaaaca agtagcactt gaatttccag gagtctacat gcgctacgcg
     1141 aatggtatac gaggactttg cagacaagtc cacaaaccaa aaccttacgt acgtgcaact
     1201 cctttggaac tacacatcat ctacggacct tccggaatcg gaaagaccta cgccgcagaa
     1261 caaaagttca tcgacggaaa catgtcagca gcgaagatct cgcaactaaa cctaaaagga
     1321 ggatggtggg acgactacaa cggagaagat gtaatcttcc tcgacgaatt cagagactat
     1381 tgcatgtacc ctgaaaactt ccttcagctc gttgaccgaa tgaaaaaact accagtcaaa
     1441 ggacaattca ttgactgtcc agctaaagcg atatggatca ctacgccaga ccatccttac
     1501 gaatggtgga aaaagtggat gagcaaaaca tcgaacaact gggctcagct gtcgagacga
     1561 atcaccaaat tgtacaaaat ggattcaaga gaagaaggtc ttgtagaaat aagtccacaa
     1621 gacgcgtctc actgtccagt gataccagaa ataccaaacg tccaagaact tgcagctctc
     1681 gtacctacag gccctatttt atgtaaaaaa cggaaacgct aagctccctc gcagggcgga
     1741 cgacaaacca ttaggtgctc cgcacactac aatgggcgct ccgcgcatgg tcttgaagtg
     1801 acagaaattt gcaaaccatt gaaatttatg aaatcacagg aagaatagcc gaagaaaacg
     1861 gcgtcacata aacttgaaga gaagcgttaa gcaaattgct attaacgcca gggccagcat
     1921 tcggagagaa gtaaacttct ttcgaaatag gaacgcttcc gtcaacggaa atcattgcac
     1981 tgtactcgta agaccttgca gtaccaccag tgagaagctc accaccagaa aaggtattca
     2041 aatcaacgcc aggagtcgct gtcataccaa gaccaggaaa cgcagttgtc ccgacattgt
     2101 tgtaacgaaa atcgacattg taaaggccat cacaagtacg actgaaaatg ataccattcc
     2161 caggaacatc aacagtacat ccagccatcc caacattaac aaatgtatag tcgccactcg
     2221 gagaaccaaa cagcaatggc ggagcaatga cgtttgaacc aaaagagaaa ataggacgct
     2281 gaacaactga tcgagcttgt tgaggctttt tgaaaacaaa agcataagcg tgccacaatt
     2341 gaccaattat gacaccatcc gtttgctgac cttcggtaca aacctgtaca ttaccaagat
     2401 cgtaccactt gatactatca tccgctccaa ctgtttgagt tgtacgaaca aagaactgct
     2461 taacgccacg ttcattggga tcacattcaa tcggcaaagc aacatcacaa gatggcttcg
     2521 tagacacagt ccatatgtta ttgagaacat ccgacatagt ctcataagga ggatccgtag
     2581 gattgtactc acacgtcaac gcaacggtac ccaaagcagt gttctgcgaa ttcaacgcat
     2641 caccactggt agatttgaag taccaaacct cacccatagg ctcccattcg ttataactag
     2701 gagccaaatt ggaagcccaa ggataactaa cgttgttccc aggctgaatc aaaaaactct
     2761 gattctgaaa accaatactc gaagagatat tttgaataaa ttcatttttg accacatatt
     2821 gacggtcacc cgaaccaaat gtgtatgcac cttgtccact aacgtttcca cccacattga
     2881 tacccatcaa cgtattacgt ttgataggag aaagacccat ggttttgtag tcaccaatac
     2941 cagtaaggtt atggaaaaag gaccctgcca aatttccaag atatccaccc acatcaccaa
     3001 agccaggaat aggaatcaaa ttcccaagcg ctctaccagc accgctaagc gctccacgac
     3061 tgatagaacc gccaaaatca taaaactttc gtttagacat tttaaaatct tttaaaacgc
     3121 agaaagagaa aaaaaatttc tctgcctaat caactggcat atcgccattt ggaagaggag
     3181 gcaaaacctg aggagctaca taaggatcta tgtcagccaa aggtccgagc ttacgcttct
     3241 tcccaatacg agcaatctcg ccaacaataa ctttttggc
//
LOCUS       CruV-199                3279 bp    DNA     circular     24-FEB-2020
DEFINITION  Crucivirus-199 isolate 120_1503_IR2_3297.
ACCESSION   CruV-199
KEYWORDS    .
SOURCE      Crucivirus-199
  ORGANISM  Crucivirus-199
            Unclassified.
REFERENCE   1  (bases 1 to 3279)
  AUTHORS   de la Higuera,I., Kasun,G.W., Torrance,E.L., Pratt,A.A.,
            Maluenda,A., Colombet,J., Bisseux,M., Ravet,V., Dayaram,A.,
            Stainton,D., Kraberger,S., Zawar-Reza,P., Goldstien,S., Briskie,J.,
            White,R., Taylor,H., Gomez,C., Ainley,D.G., Harding,J.S.,
            Fontenele,R.S., Schreck,J., Ribeiro,S.G., Oswald,S.A., Arnold,J.M.,
            Varsani,A., Enault,F. and Stedman,K.M.
  TITLE     Direct Submission
  JOURNAL   Submitted (24-FEB-2020) Biology, Portland State University, 1719 SW
            10th Ave, Portland, Oregon 97201, USA
COMMENT     Bankit Comment: ALT EMAIL:ide@pdx.edu
            Bankit Comment: TOTAL # OF SEQS:451
            Bankit Comment: GAP: est. length - alignment

            ##Assembly-Data-START##
            Assembly Method       :: MetaSPAdes v. 3.12; IDBA-UD v. 1.1.1;
                                     MEGAHIT v. 1.0.3
            Sequencing Technology :: 454; Illumina
            ##Assembly-Data-END##
FEATURES             Location/Qualifiers
     source          1..3279
                     /organism="Crucivirus-199"
                     /mol_type="genomic DNA"
                     /isolate="120_1503_IR2_3297"
                     /isolation_source="Water"
                     /country="Norway"
                     /collection_date=2011
     CDS             1..1491
                     /codon_start=1
                     /product="Putative Replication Associated Protein"
                     /translation="MNFFDLLYKMSHSNNTISNTPGFGNWHTDPTVDLDAFIARAYLG
                     APPTRERSVYYDDNNDDGEHEDHTAERTASLYQLSECDSGETYSLASEDASSIDIDDI
                     EDLEFDDEIYEDRVFAPKPAPSHRFRNWFITYFAEEWQPLFHKAAGLTYALMTKELCP
                     TTQKIHFHCYAEYNLPVDLRRVQADLSAPNANCQPRKGTQRQAIAYILKERTRFPGSK
                     PIEYGRPKRQGQRSDLDWIAEAIMQGVSRREIQQIGGGNVLRYGNMISGAIRAMNLQD
                     DNDLMTLARRRMAAKNNVSYAETLKIPIAEMKAMFPEVRREYATDPALFEASEEAKRA
                     LHYIAPSDQAKEKKMAQRKDAKEQKAKAKEQEKREKYDKIMQSDPIEHIGELYKAYKK
                     PTQVFLSRVYGDHMPNIMNHWMHYLRHNYIEHFQDRSTMTQYKDVFATLFQNYMFKFA
                     CATFTDKPVHKACMEEFRHYEGAAQVAEWIENNKDWFYFPTLAKSA*"
     CDS             1493..2932
                     /codon_start=1
                     /product="Putative CruV Capsid Protein"
                     /translation="MYNIKYIIKMPAKRTYKRTTTRRPAARRTVPRDYVKYEGGGGAY
                     TSRVAPRLTRARAGYARISKHGSYAYDDAPFASAGEAAGRYIGQFAPRVGRFLGHNVG
                     RLFGSGSYGMQSTTMAPLVPRFVNDSGDDSVVITHREYLGDVITSNTIGATKIMTFPL
                     NPATTQTFPWLSNIVQPNYQQYKFDGLVFEFRSFSGNALNSTNTALGSLFAAINYDSS
                     DSDFTSREQIENSDWSKSCKPSDDVLIPVECATRQTAMGGLLYVVNGNNIPVGSDPKT
                     FLLGKLAIGTRGFQAANVNIGSLYVTYKIRLYKPLMTAPLSNANIVQYVRLGCTPALP
                     FGTSTQSTSPGGLACDTLGMTFTDTQTLQIRPDRLMNGQIYCLKCTWTGAATAGIASV
                     DFLTIVSNGIGGLQDYFGAGAVFTSPTSGGVNDLSCTSMIIFSITDDSVPVNIKVPLT
                     SNIPNPCSMLLNLWQISGDPPATTGFYTG*"
ORIGIN
        1 ctgaattttt ttgatttatt atacaaaatg tctcactcta acaatactat tagcaatacc
       61 cctggatttg gtaattggca cacggaccct accgtggacc tggatgcatt tattgcacga
      121 gcgtatctcg gagccccgcc gacgagagag cgtagcgtat attatgacga taataacgat
      181 gacggagagc acgaagatca cacagccgaa cggactgctt ctttatatca gctctctgaa
      241 tgcgatagtg gtgaaaccta ctcgctagca tcggaagatg cgtcttctat tgacatcgat
      301 gacatcgagg atttagaatt cgatgacgaa atttatgagg atagagtatt cgctccaaaa
      361 cctgcgccat cgcacaggtt tagaaattgg ttcataacct attttgcaga ggaatggcaa
      421 ccgctattcc ataaggccgc cggacttaca tacgcattaa tgacgaagga attatgcccg
      481 acaactcaga agattcattt ccactgctac gccgaatata atcttccagt agacttgcgc
      541 cgcgtgcaag cagaccttag cgcacccaat gcaaattgcc aaccccgcaa gggaacgcaa
      601 cgacaagcaa ttgcatacat cctgaaagag cgcacgcgat ttccaggatc gaaacccatc
      661 gagtatggcc gccccaagcg ccaaggacaa cgctccgacc tcgactggat tgcagaagcc
      721 attatgcaag gagtttcaag acgtgaaatc caacaaatcg gtggcggaaa cgttttacgc
      781 tacggtaata tgatttcagg tgctatacgc gctatgaatt tacaagacga taacgacctt
      841 atgactttag cccgtcgtcg tatggccgcc aagaataatg taagttatgc tgagactctc
      901 aagataccca tcgcagagat gaaggctatg tttcccgagg tccgtaggga atacgcgaca
      961 gacccggcat tgtttgaggc aagcgaggaa gcgaagcgtg ccttacacta catcgctccg
     1021 tccgaccagg caaaggagaa gaagatggct cagcgcaagg acgccaaaga gcagaaggcg
     1081 aaagccaagg aacaagagaa acgcgagaaa tacgataaga ttatgcaatc cgatcccatt
     1141 gagcatatcg gtgaattata caaggcatat aagaagccta cccaggtctt tctttcgcga
     1201 gtttacggcg accatatgcc taatattatg aatcattgga tgcactactt gcgacacaat
     1261 tatattgaac actttcaaga ccgaagtact atgactcaat acaaggacgt attcgctact
     1321 ctctttcaaa attatatgtt taagtttgct tgtgccacct ttacagacaa accggtacac
     1381 aaggcttgta tggaagagtt tcgccattac gagggagcag cacaagttgc tgaatggata
     1441 gagaataata aggattggtt ctatttccct accctcgcta agtccgccta agctgtataa
     1501 tataaaatat attatcaaaa tgcccgctaa acgtacttat aagagaacaa caacccgacg
     1561 acccgccgca cgccgtacag tcccccgcga ctacgtaaag tatgagggag gaggaggtgc
     1621 atatacatct agagtcgccc cgcggctcac gcgcgcccgc gcaggatatg ctaggattag
     1681 taaacacgga tcttacgctt atgacgatgc ccccttcgca tcagcaggcg aagccgcagg
     1741 aagatatatt ggacaattcg cgccccgtgt aggtcgtttc ttaggccaca acgtaggccg
     1801 tctttttggt tcagggtcat atggtatgca atctaccact atggcaccgc tagttccaag
     1861 attcgtgaat gattcgggag acgattcagt cgtgattaca caccgtgagt atcttggaga
     1921 cgttataaca tcgaacacaa tcggagcaac caagattatg acattcccac taaatcccgc
     1981 gaccacgcag acattcccat ggctgtcaaa tattgttcaa ccgaactatc aacaatataa
     2041 atttgacgga ttagtatttg aattccggtc tttttctggc aatgcattga acagtacaaa
     2101 taccgctctt ggttctttgt tcgccgcgat taactacgat tccagtgata gcgactttac
     2161 gtcccgcgaa cagattgaaa acagtgattg gtcgaaatcc tgtaaacctt ctgatgacgt
     2221 actcattccc gttgaatgtg ctactcggca gaccgccatg ggcgggttat tgtacgttgt
     2281 aaacggaaat aacattccgg tcggatccga tcctaaaaca tttttactcg gaaagctagc
     2341 tattggtact agaggcttcc aagcagcaaa cgtaaatatt ggttcattat acgttacgta
     2401 taagattagg ttgtataagc cgttaatgac cgccccgtta tctaacgcca atatcgtaca
     2461 gtatgtccgt ttaggatgca cacctgcact ccccttcggt acatctacac aatccacctc
     2521 gcctggtggt ttagcttgcg atactcttgg tatgactttc accgatacgc aaacgcttca
     2581 aattagaccc gaccgtctta tgaatggaca aatctattgt cttaagtgta cttggacagg
     2641 cgccgctacc gccggtattg catcagtcga tttcctcact atagtctcga acggaatcgg
     2701 tggtctgcaa gactatttcg gcgccggcgc cgtatttacc tcacccacat ctggtggtgt
     2761 aaatgacttg tcctgtacat caatgattat ctttagtatt acggatgatt cagtaccggt
     2821 taacatcaag gttccattaa cttctaatat ccctaaccct tgctctatgt tattgaactt
     2881 gtggcaaatc agtggcgacc ctccggcaac aactggtttc tataccggtt aactacaaat
     2941 gtataaaaat atacctatct cgatcaccta tctgaatgat tgatcgtaaa aacgggtaat
     3001 gatgacacac gcagtggggc atcaaacaaa catgtcatac ccgtctaaac tcagcactct
     3061 gctaagagtc gacgccagtc gaaatcagga cagcgaacaa aaaaaaaaaa tatttatatt
     3121 tttttgtagt attaccgctg tcttatgaac atattttaat aaaatatggt ctaagataac
     3181 ttctgtccca caaagttatt ttattttaga gagttcatgt gtttatcgat accatcgatg
     3241 aatgtacaaa agtttacctt ttttcacttc tacacataa
//
LOCUS       CruV-200                3282 bp    DNA     circular     24-FEB-2020
DEFINITION  Crucivirus-200 isolate 001606_virusbaton.
ACCESSION   CruV-200
KEYWORDS    .
SOURCE      Crucivirus-200
  ORGANISM  Crucivirus-200
            Unclassified.
REFERENCE   1  (bases 1 to 3282)
  AUTHORS   de la Higuera,I., Kasun,G.W., Torrance,E.L., Pratt,A.A.,
            Maluenda,A., Colombet,J., Bisseux,M., Ravet,V., Dayaram,A.,
            Stainton,D., Kraberger,S., Zawar-Reza,P., Goldstien,S., Briskie,J.,
            White,R., Taylor,H., Gomez,C., Ainley,D.G., Harding,J.S.,
            Fontenele,R.S., Schreck,J., Ribeiro,S.G., Oswald,S.A., Arnold,J.M.,
            Varsani,A., Enault,F. and Stedman,K.M.
  TITLE     Direct Submission
  JOURNAL   Submitted (24-FEB-2020) Biology, Portland State University, 1719 SW
            10th Ave, Portland, Oregon 97201, USA
COMMENT     Bankit Comment: ALT EMAIL:ide@pdx.edu
            Bankit Comment: TOTAL # OF SEQS:451
            Bankit Comment: GAP: est. length - alignment

            ##Assembly-Data-START##
            Assembly Method       :: MetaSPAdes v. 3.12; IDBA-UD v. 1.1.1;
                                     MEGAHIT v. 1.0.3
            Sequencing Technology :: 454; Illumina
            ##Assembly-Data-END##
FEATURES             Location/Qualifiers
     source          1..3282
                     /organism="Crucivirus-200"
                     /mol_type="genomic DNA"
                     /isolate="001606_virusbaton"
                     /isolation_source="Water"
                     /country="France"
                     /collection_date=2013
     CDS             1..1557
                     /codon_start=1
                     /product="Putative Replication Associated Protein"
                     /translation="MSKLSKKMIDSGNDELEFDYLLAGKTSAKVRYCCITINNIEPGQ
                     IDAIREAYAKGKITYYVIAFEYGKNMTLHIQGYLEFKCQVTFATVNKLLAYEDGSWPH
                     SEKRGMNSCQKQAIDYCKKGIQSKAEWKKLHQSGPNFGKEAKFEEDGIPKPGQGTRTD
                     IETAKCWMKEGLSMEEILERCNNYQVSKYVENFIHMFEPQKTWKPIVRWFWGESGCSK
                     SHTAKLQAGYIDSNGKPTGNKNVYIKPQMDRWWPKYDGQELVIIDELRANIIPCAKLL
                     SLLGSEPDMVENKGKYRQFRAKEIWITTSKCPEEFYKNYKDEIEDIKQLIRRINGEEK
                     DENGVLLGCSTHFNIPYETVNFELHTTSQEEEMAKAQKISDSKIKSEDLIESDNTING
                     SEVAGNSQTATQDYKCPLSDDARLAAKKRFDDFLVILKNKKLNELSNSISEIISESSS
                     SEETEIVTLYECLKCKKWEEKMSPNENLCIYCYKNTCKPKKEGRTPCYKCEHGTCWES
                     MDWKCNHCGK*"
     CDS             1839..3197
                     /codon_start=1
                     /product="Putative CruV Capsid Protein"
                     /translation="MSARKRIVRKKKYSDRDKAEYYKSLALSNDTRMIRRFPAYTGRG
                     AYKKRTRRTFREKVGNTAVRDVIQPIASAIGGPLAGYAVGGLGYLADKAIKFFTGRGD
                     YTIKENSLMSEGSDMPPIINRDPGGGFLIRRGEYIGDVISGAANSFTLNNYFVNPGQI
                     STFPWLAQVAVNFEEWVCEGMYFEFRTMSADALNSTNTALGQVIMAASYNSSNANFTN
                     KQVMENYEGGISCKPSCSMRYFVECARSKTVLDDLYVRPGAVPAGDDQRFYDLANFQI
                     ATNGLQGANVNVGELWVEYQISLRKPKMFSALGFNILMHDNGRTGCANNIPFGTTSSL
                     NTNLNNIPIVFTGTTMVFPPSSIPQAFYGTWKWTSSAVTTTVPALTFGPGATSFNLIQ
                     APNNGTLAVTQEAAAYTFYFPGNSSAADRTITAATNGTISVDGCRITIIQCSNAFDGF
                     PE*"
ORIGIN
        1 atgtcaaaac tttcaaaaaa aatgatagat tcgggtaacg atgaactcga atttgactac
       61 ttactagccg gtaaaacatc ggctaaagta agatactgtt gtataactat aaataatata
      121 gaacctggac aaattgatgc aattagagaa gcatacgcta aaggaaagat tacatactat
      181 gtaatagctt tcgaatatgg aaaaaatatg actctacata tccaaggata cttagagttt
      241 aaatgtcaag taacattcgc tacagtgaat aaattacttg catatgaaga tgggtcatgg
      301 ccccatagtg aaaaaagagg aatgaattcc tgtcaaaagc aagctattga ctattgtaaa
      361 aaaggtattc agtctaaagc tgaatggaaa aaacttcatc aaagcggtcc taactttggt
      421 aaggaagcta aatttgaaga agatggaatt cctaaacctg gtcaaggaac caggacggat
      481 atagaaacag ctaaatgctg gatgaaagaa ggattatcta tggaagaaat acttgaacgt
      541 tgtaacaact atcaagtttc aaaatatgtt gaaaacttca tacatatgtt tgaaccacaa
      601 aaaacatgga aaccaatagt tcgttggttt tggggagaat ctggatgttc aaaatcacat
      661 acagctaaac tacaagcagg ttatattgac tctaacggaa aacctacagg caataaaaac
      721 gtttatataa aaccacaaat ggatagatgg tggcctaaat atgatggcca agaattagtc
      781 ataattgacg aattaagagc taacataatc ccttgtgcta aattattatc tttacttggt
      841 tcagaaccag atatggtaga gaacaaaggt aaatatagac aattcagagc taaagaaata
      901 tggatcacga catcaaaatg tcccgaagaa ttttacaaaa attataaaga tgaaattgaa
      961 gacatcaaac agttaattag aagaattaat ggcgaagaga aagatgaaaa cggtgtttta
     1021 ctcggatgtt caacacattt taacattcct tacgaaacag taaattttga acttcacacc
     1081 acttctcaag aagaggaaat ggctaaagct caaaaaatat cggattctaa aataaaatct
     1141 gaagatttaa tagaaagcga taataccata aatggctcag aagtcgcggg taatagtcaa
     1201 accgcgacac aggactataa atgtccacta tcggacgatg cccgattggc agcaaaaaaa
     1261 agatttgatg attttctagt gatactaaaa aataaaaaat taaatgaatt atcaaattca
     1321 atttcagaaa ttatttctga atcttcatca agtgaagaaa cagaaatagt aacactttac
     1381 gaatgcttaa agtgtaaaaa atgggaagaa aaaatgtcac caaatgaaaa cttatgtata
     1441 tattgttaca aaaatacatg taaacctaaa aaagaaggaa gaactccatg ttacaagtgt
     1501 gaacatggga catgctggga aagcatggat tggaaatgta atcactgcgg aaaataagtt
     1561 tgatacaaca ttcacgaaaa gcgaattagt ttcataagtt ctcatcagcc tttttcatgc
     1621 ctaacggcaa gaaaaaaaag gcttcttcgg ccttatgaaa caacattcac ttttctgcgc
     1681 accattatat atatatacgc gtgcaagcac gcaacaccta atgggaatac gtttggttgt
     1741 ttaagaaaac aacactaaag cgtatatagg ccagttttgg atcaatccga attatatttt
     1801 tttattttag aaaaatattc ggattaatat agaccaaaat gtcagcaaga aaaagaatag
     1861 taagaaagaa gaaatattct gacagagaca aagcagaata ctataaatca cttgcactaa
     1921 gcaatgatac aagaatgata agaagatttc cagcttacac tggaagaggg gcctataaaa
     1981 aaaggacaag aagaactttc agagaaaaag taggaaatac tgcagtaaga gatgtaatac
     2041 aacctatcgc atcagcaata ggaggtccat tagctggata tgcagtagga ggattaggat
     2101 acttagcaga taaagcaata aaattcttta caggaagggg agattacaca ataaaagaaa
     2161 actcattaat gagtgaagga agtgacatgc cacccataat aaatagggat ccaggaggag
     2221 gattcttaat aagaagagga gaatatatag gagatgtaat atcaggagcg gctaattcat
     2281 tcacactgaa taactacttt gtaaatcctg gacaaatatc aacatttcca tggctggcac
     2341 aagtggcagt aaattttgaa gaatgggtat gcgaaggaat gtatttcgag ttcagaacta
     2401 tgtcagcaga cgctttaaat agtacgaaca ctgcattagg tcaagttata atggcagcca
     2461 gttataactc atctaatgcg aactttacga ataaacaagt tatggaaaac tatgaaggag
     2521 gaatttcgtg taaaccctca tgttccatga gatacttcgt agaatgtgca aggtcaaaaa
     2581 ctgtattaga tgacctttat gtaagaccag gagctgtacc agcaggagat gatcaaagat
     2641 tctatgatct tgctaatttt caaatagcaa cgaatggatt acaaggagct aacgtaaacg
     2701 taggtgaatt atgggtagaa tatcaaataa gtttaagaaa acccaaaatg ttttcagcac
     2761 ttggatttaa tattttaatg catgacaatg gtagaacagg atgtgcaaac aacatacctt
     2821 ttggtacaac aagtagttta aatactaatt taaacaatat acctattgta tttacgggta
     2881 cgacaatggt gtttccgcca tcatcaatac ctcaagcatt ttatggtact tggaaatgga
     2941 ctagctcagc agtaacaaca actgttccag ctctcacatt tggtcctgga gctacatcat
     3001 ttaaccttat acaagcacca aataatggaa cattagcagt aacacaagaa gcggcagcat
     3061 atactttcta ctttccagga aattcaagtg cagcagatag aactataact gcagcaacaa
     3121 atggaacgat ttcagtagat ggatgtagaa taaccataat ccaatgctct aacgcattcg
     3181 atggattccc tgaataaact ccaaaaatag aatgacctaa atttaattta ttttttttaa
     3241 aaaaataagt taactaatat aggtcggaaa aaataatttt ca
//
LOCUS       CruV-201                3283 bp    DNA     circular     24-FEB-2020
DEFINITION  Crucivirus-201 isolate 120_1443_IR2_3297.
ACCESSION   CruV-201
KEYWORDS    .
SOURCE      Crucivirus-201
  ORGANISM  Crucivirus-201
            Unclassified.
REFERENCE   1  (bases 1 to 3283)
  AUTHORS   de la Higuera,I., Kasun,G.W., Torrance,E.L., Pratt,A.A.,
            Maluenda,A., Colombet,J., Bisseux,M., Ravet,V., Dayaram,A.,
            Stainton,D., Kraberger,S., Zawar-Reza,P., Goldstien,S., Briskie,J.,
            White,R., Taylor,H., Gomez,C., Ainley,D.G., Harding,J.S.,
            Fontenele,R.S., Schreck,J., Ribeiro,S.G., Oswald,S.A., Arnold,J.M.,
            Varsani,A., Enault,F. and Stedman,K.M.
  TITLE     Direct Submission
  JOURNAL   Submitted (24-FEB-2020) Biology, Portland State University, 1719 SW
            10th Ave, Portland, Oregon 97201, USA
COMMENT     Bankit Comment: ALT EMAIL:ide@pdx.edu
            Bankit Comment: TOTAL # OF SEQS:451
            Bankit Comment: GAP: est. length - alignment

            ##Assembly-Data-START##
            Assembly Method       :: MetaSPAdes v. 3.12; IDBA-UD v. 1.1.1;
                                     MEGAHIT v. 1.0.3
            Sequencing Technology :: 454; Illumina
            ##Assembly-Data-END##
FEATURES             Location/Qualifiers
     source          1..3283
                     /organism="Crucivirus-201"
                     /mol_type="genomic DNA"
                     /isolate="120_1443_IR2_3297"
                     /isolation_source="Water"
                     /country="Norway"
                     /collection_date=2011
     CDS             1..1356
                     /codon_start=1
                     /product="Putative Replication Associated Protein"
                     /translation="MSKEEIIEETNEALEYAPVLDELLHDKADSTGQSILWLLTYKTH
                     VEKSTLAKFLQKIFLATPKGFSRAAATQYREMDQVINKLAIAHEYGDKTNNYEHTHVA
                     IQLQVPIKFSNMRTLDYNGLHPHIKAIPSKNKDKAIRDTFFYLTKEDEEAKEEIENLF
                     DLSRSQYIAKLRGSAKTVLENVEEARGDTRALLGLAKKPSDVPGLIAANNLLQSDQIN
                     IVHYPLKYKWQHDIIELLLMPTHEGTRDVIWVYDDVGGSGKTNLSCYAASVYPERVIT
                     LSSLTGDVNYPIAMNKKFTGGAVMIDIPRDFDAKVIPWGKIEQIQNGFFQSNKYAGAT
                     KGVGKVRVIILANFIPEINRRVISHDKPEMWVVEKEWEDERMCVDVAGVIKWDRWYKP
                     YDKLTGKQEDWVNQTFHQKEALEECGLRSELTIQIENMSNRQLEQLAKLIERKTGKKF
                     S*"
     CDS             complement(1564..2979)
                     /codon_start=1
                     /product="Putative CruV Capsid Protein"
                     /translation="MSTGYYRRYAPSTGYQKRTYAPRYNNRTYTPRTYIKGRGAYKTG
                     TVKRRQAPRAAVSNGSSNSNSGGFWRKAAGYAPAAGSLLGNYISPGIGGAIGSAAGGL
                     LASIAGHGAYAIKRNTVFFPDQVPLFTTKQDGSIRMQHREFITDVVSSSTAGAFNIDT
                     YPIDVANPTSFPFLSQIASNFEEWRMEGLVFEYKTTSGSISATGQLGTAVLATQYNSN
                     ALPFTNKQAMEAYTFASSTVVSASVIHPVECDPMQTPSNGLFYTNVPGTGNGTEDKRW
                     QQLGNFNIATQGCAVSENIGELWVSYDVWLCKPRLISDTSALSDHWHLTTGINFGTPE
                     YFGTGVTLSNGSDQYTLLQANTIYFDSSFNGNVSITYYVTGGVGAWITPDIGVNVADR
                     IGAVPLNLLSGTNQVVSFYDALEVDIQITAYFTITSTPNSVGLLPGYGAKIVFGATTA
                     GASFGAVLGDVIISQLPSDFN*"
ORIGIN
        1 atgagcaaag aagaaataat agaagaaacc aatgaagctc ttgaatatgc gccagtttta
       61 gatgaactgc ttcatgataa agcagattca actggtcaat cgatattgtg gcttttgaca
      121 tacaaaacac acgtcgagaa aagtactctt gcaaagttct tgcaaaagat atttcttgca
      181 acacccaagg ggttttcaag agcagccgca actcaatata gagaaatgga tcaagtcata
      241 aacaagcttg caattgctca cgaatatggt gataaaacaa acaactatga gcatactcat
      301 gttgctatcc aattgcaagt tcctataaag ttcagtaata tgcgtactct agattataat
      361 ggattacatc ctcacataaa agctatacca tcgaaaaaca aagataaagc aatacgggat
      421 accttcttct acctcacaaa ggaggatgaa gaagcgaaag aagaaataga aaatctcttc
      481 gacttaagta gatcgcaata tattgcaaaa ttacgaggat cagctaaaac tgttctagaa
      541 aacgtcgaag aggccagagg tgacacaagg gcacttctgg ggttagcgaa aaagccatca
      601 gacgtacctg gtctgattgc tgctaacaac ctcctacaga gtgatcaaat aaacatagtt
      661 cattatccac tcaagtacaa atggcagcac gatatcatag aactcttgct tatgccaacc
      721 catgaaggaa caagagatgt tatatgggtg tacgatgatg taggaggaag cggtaaaaca
      781 aacttgtcat gctatgcagc aagtgtctat cctgaaagag ttataactct ttcgagcctt
      841 acaggagatg tcaactatcc tatagccatg aataagaaat tcacgggagg agctgtcatg
      901 atagacatac cgcgagattt cgacgctaaa gtaatacctt ggggtaagat tgaacaaatc
      961 caaaatggat tcttccaatc taacaaatac gcaggagcta ccaagggagt tggcaaagta
     1021 cgagtaatca tacttgccaa cttcattccc gagataaaca gacgtgtcat atcacatgac
     1081 aaaccggaaa tgtgggtcgt cgaaaaagaa tgggaagacg agaggatgtg tgtcgacgtg
     1141 gcaggcgtga taaaatggga tcgatggtat aaaccatacg acaagcttac tggtaagcag
     1201 gaagattggg taaaccaaac attccatcaa aaagaagcac tcgaggagtg cggtctacga
     1261 tcagaactaa ccattcagat cgagaacatg tccaatagac aattggaaca attagcaaag
     1321 ctaatagaac gaaaaacagg aaaaaagttt tcctaatgtt ttctgttctt ggcatttatt
     1381 aatttaaaaa aattaaaaat gcaccacgtc gaaaattata tctctaagag agacataatt
     1441 acaacagact actacatagt cgttgaaaag gggctaacat taattaaagc tacgctcgta
     1501 aactcgctcc gcttttgccc cttttctgct tgctcgccta cggctcgcgg cgcgggaagt
     1561 aacttagtta aaatcggaag gaagttgaga aatgataaca tctcccaaca cagcaccaaa
     1621 ggatgctcca gcagtagtag caccaaagac aatcttagct ccatatccag ggagaagacc
     1681 gactgaattt ggtgtactag taattgtaaa atatgccgta atttggatgt caacctccaa
     1741 agcatcataa aaactaacaa cctgattagt tccagagagc aaattcaaag gaacagctcc
     1801 aatacggtcg gcgacgttca ctccaatatc aggagtaatc cacgcaccta cacctcctgt
     1861 aacataataa gtaatagaaa cattgccatt aaacgaagaa tcaaaataaa tggtgtttgc
     1921 ttgaagcaaa gtatattggt cagatccatt ggacaaggtt actccagtac caaaatactc
     1981 tggagtacca aaattaattc cagtggttaa atgccaatga tctgacaaag cggaggtatc
     2041 agaaatcaag cgtggtttgc aaagccaaac atcgtaagaa acccagagtt caccgatgtt
     2101 ttcagagaca gcacatcctt gagtagcaat gttgaagttt ccaagctgct gccatcgttt
     2161 atcttcagtt ccatttccag ttccgggtac attagtgtag aagagaccat ttgaaggagt
     2221 ctgcattgga tcacattcaa ctggatggat aacgcttgca gaaacgacag ttgaggaagc
     2281 aaaggtataa gcttccattg cctgtttatt tgtaaatggc agggcgttgg aattatattg
     2341 agttgcgaga actgctgttc caagttggcc agttgcactg atacttcctg aagttgtttt
     2401 gtactcaaaa acaagtcctt ccattctcca ttcttcaaaa ttggaagcaa tctgggagag
     2461 gaagggaaag gaagtgggat ttgccacgtc gatggggtag gtatcaatat tgaaagctcc
     2521 tgctgtagag ctggagacga cgtctgtgat aaattctctg tgttgcattc gaattgaccc
     2581 atcttgtttt gtggtaaaca gtggcacttg atctgggaaa aagactgtgt tccgtttaat
     2641 ggcataagcc ccgtgacctg caatcgaggc aaggagacct cccgctgcac ttccgattgc
     2701 acctccaata ccgggagaga tgtaattacc gaggagagat cctgctgctg gagcatatcc
     2761 tgctgctttt ctccaaaacc caccggagtt ggagtttgaa ctaccgttgg agacagctgc
     2821 tcttggtgct tgacgtcttt tgacagttcc tgtcttgtac gcaccacgcc ctttaatata
     2881 agttcttggg gtgtacgttc tattgttata tctcggtgcg tatgtccttt tctgataccc
     2941 cgtcgatggt gcataacgcc tgtaataacc tgttgacatt ttctattttt tctttccgtg
     3001 gcgttagaat atttttatag aaaatatttt ttaaaaataa tttttggatt taatccaaaa
     3061 atgtagcgca ggggcggacg cctcgtttcc tcggcggcct gcctgcggaa acaccacggg
     3121 atctttttcc cattttatca cggatcgttt cttccaaggc gctacgcgcc acacaactaa
     3181 agctattaat tatattattt cgcattttcc ctaaagaaaa ttatttttgt aataaaatac
     3241 atacatacat acatacatac atattttaat tctatataaa aga
//
LOCUS       CruV-202                3285 bp    DNA     circular     24-FEB-2020
DEFINITION  Crucivirus-202 isolate 001603_virusbaton.
ACCESSION   CruV-202
KEYWORDS    .
SOURCE      Crucivirus-202
  ORGANISM  Crucivirus-202
            Unclassified.
REFERENCE   1  (bases 1 to 3285)
  AUTHORS   de la Higuera,I., Kasun,G.W., Torrance,E.L., Pratt,A.A.,
            Maluenda,A., Colombet,J., Bisseux,M., Ravet,V., Dayaram,A.,
            Stainton,D., Kraberger,S., Zawar-Reza,P., Goldstien,S., Briskie,J.,
            White,R., Taylor,H., Gomez,C., Ainley,D.G., Harding,J.S.,
            Fontenele,R.S., Schreck,J., Ribeiro,S.G., Oswald,S.A., Arnold,J.M.,
            Varsani,A., Enault,F. and Stedman,K.M.
  TITLE     Direct Submission
  JOURNAL   Submitted (24-FEB-2020) Biology, Portland State University, 1719 SW
            10th Ave, Portland, Oregon 97201, USA
COMMENT     Bankit Comment: ALT EMAIL:ide@pdx.edu
            Bankit Comment: TOTAL # OF SEQS:451
            Bankit Comment: GAP: est. length - alignment

            ##Assembly-Data-START##
            Assembly Method       :: MetaSPAdes v. 3.12; IDBA-UD v. 1.1.1;
                                     MEGAHIT v. 1.0.3
            Sequencing Technology :: 454; Illumina
            ##Assembly-Data-END##
FEATURES             Location/Qualifiers
     source          1..3285
                     /organism="Crucivirus-202"
                     /mol_type="genomic DNA"
                     /isolate="001603_virusbaton"
                     /isolation_source="Water"
                     /country="France"
                     /collection_date=2013
     CDS             1..1347
                     /codon_start=1
                     /product="Putative Replication Associated Protein"
                     /translation="MSRTREVLTNKDVSKALREIYVSKHGPIDKRSSASDSEPTLKKS
                     PLQKQRVIVRPLDTKGKRPEASRPVAIQKVDVISASLEVENELLEINHPGGYESVTRG
                     AGHWMSLLEDYESSCETYNRRSFDFVSTLSTTEDESCKIKPDDWKSPIKHQRLRGVCF
                     TLNNYTDKDQQDLRDQYDAGLYTYLVFGREVGKLGTPHLQGYAHFTKQNSFEQIRKIF
                     GRKFHFEYARATGSKFAERYLYCMKDGDFEEFGVRPVHGTNEQKKLKITELIKEGKNQ
                     TEIREICPEGYFHHGVKVENWFEDERKINPPKLKTKFYYIERSEDINIYEAIEKLFPN
                     EEFAHIFNLSELISYRNNDGIVKNICYHNPDNISQVESWSYGSKILCKNGYKQLPIYC
                     ERMIVYCPWFGLNNKLKKYVKLENLHEDFMLVPLKETREQEDPLDCVQSKGPAQRR*"
     CDS             1391..2746
                     /codon_start=1
                     /product="Putative CruV Capsid Protein"
                     /translation="MSKSKNSVKRQPRRPVSTKQLVVIEPERKNRKVARKTYKSYARA
                     EKAPGALSGLGRFAGGALGSTFGPAGTGVGSFLGGKLGHLIETVTGFGDYKVEQNSIM
                     KGGMTVPQIVNSAETGGVIIRHREFIADIPATTAFTVQTFTIQPGLAHTFPWLSQIAN
                     SFEQYRMRGMLFEFNSTSSDAVLSTATSTALGSVIMMTDYDVADAPPTGKRQMLNSEY
                     ASSSKPSCTFIHPIECKKSMTPQTMMYTRGSIAPPPGFDPRLYDFANFHIATEGMQIP
                     PATPAGIIGELWVTYELEFFKPQFSFYGPTDHFRFNTITAARPFGTVVGSNIKNGGTI
                     GGSISGSALDYSFPPEVSSGKYLVVYSVLGAATALAVAPDLTIVNGVLDAYWANDVLA
                     WLNAPEPAATSATTAIYGTVEVRAQNCSIHLDVSGNPPTANNGDWWVVRISDSISFNI
                     N*"
ORIGIN
        1 atgtcacgaa ctcgcgaagt ccttacaaac aaagatgtat cgaaagcatt aagagaaata
       61 tatgttagta agcatggtcc gattgacaag cggagcagcg cgagcgatag cgagcctact
      121 ttgaagaagt caccacttca aaaacagcgc gtgattgtcc gtcctttgga tacgaagggt
      181 aagaggccag aggcctcgag acccgtagct attcaaaagg tggacgtgat aagcgcgtct
      241 ctagaagttg agaatgaact tttagagatt aatcatccag gcggttacga gagtgtaacg
      301 agaggggccg gtcattggat gagtttatta gaagattatg aatcttcgtg cgagacgtat
      361 aatagaaggt cttttgactt tgtatcaaca ttgtcaacga ccgaagatga gtcttgtaaa
      421 ataaagcctg acgattggaa gtctcctata aagcatcagc gattacgagg tgtatgtttt
      481 actctaaata attacaccga taaagaccaa caagacttaa gagaccaata cgacgcaggt
      541 ttgtatacat acctcgtctt tggtcgcgaa gtcggcaagt tgggaactcc gcatcttcaa
      601 ggttacgcgc attttacaaa acaaaattct ttcgaacaaa taagaaaaat atttggaaga
      661 aagtttcatt ttgaatatgc aagagcaacc ggttcgaagt ttgcggagcg atatctatat
      721 tgtatgaagg acggagattt tgaagaattc ggagtccggc ccgttcatgg tacgaatgaa
      781 caaaaaaaac ttaagatcac cgaacttata aaagagggaa aaaatcaaac ggaaattcgt
      841 gagatttgtc ccgaaggtta ttttcatcac ggtgtaaaag tagagaattg gtttgaagac
      901 gagagaaaaa taaacccacc aaaactaaaa actaaatttt attatataga gcgatcggaa
      961 gatatcaata tctacgaagc gatagagaag ctcttcccaa atgaagagtt cgcccacatc
     1021 tttaatttat ccgaattaat ctcatatcga aacaatgatg gaattgttaa aaacatttgc
     1081 tatcacaatc cagataacat aagtcaggta gaatcctggt cttatggatc taagattttg
     1141 tgcaaaaatg gttataaaca acttccaatc tattgtgaac gtatgatagt ttactgtcca
     1201 tggtttggac taaacaacaa actaaaaaag tacgtaaaac ttgaaaatct gcatgaagat
     1261 tttatgttag tacccttaaa ggaaacgcgc gagcaagagg atcctttaga ttgtgtacaa
     1321 tcaaaaggtc cggcccagcg caggtaactt taagtctata aaaatataaa aatattctta
     1381 tagattataa atgtcaaaat caaaaaactc cgtaaaaagg caaccaagac gtcctgtatc
     1441 aactaaacag ttggtagtca tagaaccgga acgaaaaaat cgtaaagtag ctcgaaagac
     1501 gtacaaatct tacgcaagag cagaaaaagc accaggcgca ctttctggat taggaagatt
     1561 tgcaggcgga gcgttaggat caactttcgg tccagcagga actggggtag gttcattttt
     1621 aggaggcaaa ttaggtcatc tgatcgaaac agtaacaggt tttggagact acaaagtcga
     1681 acaaaactct attatgaagg gaggaatgac cgtcccacaa atagtaaatt ctgcagaaac
     1741 cggaggtgtc ataataagac accgagaatt catagcagat atcccagcca ccacggcgtt
     1801 caccgttcaa acatttacga tccagcctgg attagcacac acttttccat ggttatcgca
     1861 gatcgcaaat agttttgaac aatacagaat gcgaggaatg ttgtttgaat ttaattcaac
     1921 ctcttccgac gcagtcctat ctacagcaac tagtactgca ctagggtctg tcatcatgat
     1981 gacagactac gacgtagcag acgcaccacc aacaggtaaa agacaaatgt tgaactcaga
     2041 atacgcaagt tcgagtaaac catcttgtac ttttatccat ccaatagaat gtaagaaatc
     2101 aatgacgccg cagacgatga tgtacacgcg ggggtctata gcaccaccgc caggcttcga
     2161 tccaaggctt tatgatttcg caaactttca catagcaact gaaggaatgc aaattcctcc
     2221 agccacccct gctggcataa taggagaatt gtgggtgact tatgaattag aattctttaa
     2281 gccacaattt tccttttatg gtccaacaga tcactttaga ttcaacacta taacagcggc
     2341 aaggccgttt ggtacagtag ttggatcaaa tataaaaaac ggtggtacaa ttggagggtc
     2401 aataagcggc agcgcattag actacagttt tccaccggaa gtatcatccg gtaagtatct
     2461 agtagtttat tccgtgttgg gagcagcaac agctttagcg gttgcgcccg accttacaat
     2521 agtgaatgga gtattagatg cttattgggc caatgatgta ctagcgtggt tgaatgcgcc
     2581 agaaccagca gcaacctcag cgacaactgc aatttatgga accgtagaag tacgagccca
     2641 aaattgctca atccacttag atgtaagtgg caatccacca accgctaata atggagattg
     2701 gtgggttgtg agaatctctg attcgattag ctttaacatt aattaaaatt ttgataaaat
     2761 cttaattatt gcagaaagcc gtaataacac ataaatgtat gactgcccag cgggagtgag
     2821 cggaagcgag cgggcagcgg tggtattgag cgtagcgaaa tgccacatcg tatatgaaga
     2881 tagaaattga ttgattttcc aggcttggaa aataattata aatttttatc gcaacaaaaa
     2941 aaatctatac ttttttgact aaaatattgg gctttcaaaa ttgcctaaat tattgaatgg
     3001 tcaaaatatt tggtcaaatt aaacgtagct aagtggcctg gccaatagtc cagccacgtg
     3061 cgtagagccg gcaacgacta cgcagtgtcc cggccggagg caaggagtat aaaatacgaa
     3121 ttagcacggc cgaacttgtt gaatgatata aatatttggt cacattacgt gaagaaatag
     3181 tataaattca acccgttata gggcaggccc ccggccgtag tattacccta taacgtcctg
     3241 agtgagtgag taattttaaa aaataatcta tagttatttt ataga
//
LOCUS       CruV-203                3299 bp    DNA     circular     24-FEB-2020
DEFINITION  Crucivirus-203 isolate 120_1438_IR2_3297.
ACCESSION   CruV-203
KEYWORDS    .
SOURCE      Crucivirus-203
  ORGANISM  Crucivirus-203
            Unclassified.
REFERENCE   1  (bases 1 to 3299)
  AUTHORS   de la Higuera,I., Kasun,G.W., Torrance,E.L., Pratt,A.A.,
            Maluenda,A., Colombet,J., Bisseux,M., Ravet,V., Dayaram,A.,
            Stainton,D., Kraberger,S., Zawar-Reza,P., Goldstien,S., Briskie,J.,
            White,R., Taylor,H., Gomez,C., Ainley,D.G., Harding,J.S.,
            Fontenele,R.S., Schreck,J., Ribeiro,S.G., Oswald,S.A., Arnold,J.M.,
            Varsani,A., Enault,F. and Stedman,K.M.
  TITLE     Direct Submission
  JOURNAL   Submitted (24-FEB-2020) Biology, Portland State University, 1719 SW
            10th Ave, Portland, Oregon 97201, USA
COMMENT     Bankit Comment: ALT EMAIL:ide@pdx.edu
            Bankit Comment: TOTAL # OF SEQS:451
            Bankit Comment: GAP: est. length - alignment

            ##Assembly-Data-START##
            Assembly Method       :: MetaSPAdes v. 3.12; IDBA-UD v. 1.1.1;
                                     MEGAHIT v. 1.0.3
            Sequencing Technology :: 454; Illumina
            ##Assembly-Data-END##
FEATURES             Location/Qualifiers
     source          1..3299
                     /organism="Crucivirus-203"
                     /mol_type="genomic DNA"
                     /isolate="120_1438_IR2_3297"
                     /isolation_source="Water"
                     /country="Norway"
                     /collection_date=2011
     misc_feature    join(3288..3299,1..19)
                     /note="stem-loop"
     misc_feature    1..9
                     /note="nonanucleotide"
     CDS             264..1148
                     /codon_start=1
                     /product="Putative Replication Associated Protein"
                     /translation="MYKNTVSSSSGMSRIRRVVFTFNNYDPLYNDDGALDTWLGGLGA
                     TYACAGRELAPGTGTPHLQGYATFKHAKSLSAIRRLLVGAHVEPARGTGSQCRTYCSK
                     DGSFREVGDPPADDEDSGSREKQRWENARALAKSGSFEEIPADIYVRYIGNLHRIYRD
                     TLPPLESLPATCGRWLFGPTGSGKSRGVRSNFPLVYPKPLNKWWDGYDAQTHVLLDDV
                     DMNQASWIGNFLKIWADHYPFIAEKKGGSRLIRPAMLVVTSQYSIRTLFNDDELVAAL
                     ERRFIVINVNKDVPIEWV*"
     CDS             1337..1921
                     /codon_start=1
                     /product="putative ORF"
                     /translation="MHALKMPASKKKVVVRIPKKVLPRDVPRPSSQLRREQRKKLLKE
                     VDLLEEASLIMQTNPPGDMGSANLERLAVIADELDDPSIYPLVHNFSKGKKRAVPDDY
                     APDDVVRVTNKPAVVPVITGRKRLRDEGVFVLPNRGGNYPAVASSSKLPIGGTSTRSI
                     GTQTDPPERDPSVTLWSAELLHKTGIKRIVCYLN*"
     CDS             complement(1936..3219)
                     /codon_start=1
                     /product="Putative CruV Capsid Protein"
                     /translation="MPKRIMRSSRKSRPAARRAPAATRGRLGNFLRYAGQGLGAYGGA
                     LLGNSAAGATFGRSAGANISKWLGAGSYKISKNTLAAGEIPAMHNTNEGVIIRHREYL
                     GDIFSSGTARAFNIQTFSLNPGLQRTFPWLSSLANNFTEYDIKGLVFHFKSNSGDAFT
                     STDASTGTVLMCTQYRASQAAPSNKQEILQQYWSQSAAPQQDFVHAVECDPKENPLKV
                     HYIRTGTKPTTDDQMFYDLGSTYIASVGLQGTNVNMGELWVTYEIELRKPQLRDLRNP
                     SMEAFHAWGTGTGITNTQWFTTIINNAGQTINNQSTFTPTIGDTAVTLPRGLAGRYFV
                     SYHLQGSSTATAAPTITVGTNGALTNSFTNSSTSQFNSGGTSTRQIIEFAFEIQDTNQ
                     ETTFFFQSGTGTPVTTPTGVDLYIWQMPYYGTSFN*"
ORIGIN
        1 tattattacc tagaccacct gtgtaagtgc aacgtaacac aaacttcagg gggccctgtg
       61 ccccctcttc caacgttaca caaaacaacc acaatctggg ggccctgtgc cccccattgc
      121 ataaaaaaaa ttaaaacaca gcctctggat agtgtctgtg ttcgcccttg aattacccct
      181 aaatttgcgg aattattttt ccccggctta aaactttaag cctcacgcag cctctttttt
      241 ctgccccgcg gcagcctcct cctctgtata aaaacaccgt tagttcctcg tccggcatga
      301 gtcgtattcg ccgagtcgta tttacgttta ataattatga ccccctctat aacgacgacg
      361 gggctttgga cacctggctc ggaggacttg gagctacgta tgcttgtgcc ggccgagaac
      421 tcgctcccgg aaccgggaca ccccaccttc agggatacgc cacctttaag catgccaagt
      481 ctctctcagc cattcgacga ttattggtgg gagcccacgt ggagcccgcc cgagggaccg
      541 gatcccaatg ccgaacttac tgctctaagg atggaagctt tcgggaggtc ggggaccccc
      601 cagcagacga cgaggactcc gggagtaggg agaagcagcg ttgggagaac gcccgtgcct
      661 tggctaaatc gggctcgttc gaggagatcc ccgcagacat ttacgtccga tacattggaa
      721 acttacatcg gatctatcgc gacacgctcc cacccctcga gtccttgccc gcgacctgcg
      781 gacggtggtt atttggcccc actggttctg gcaaatcaag aggagttaga agtaatttcc
      841 cgttagttta tcctaagccg cttaataaat ggtgggatgg gtatgatgcc cagacccatg
      901 tacttttgga tgatgtagat atgaatcaag ctagctggat tggaaacttt cttaaaattt
      961 gggcggacca ctacccgttc attgctgaga aaaaaggagg ttcccgacta attagaccag
     1021 ccatgttagt agtgactagc cagtatagta tacgtacgtt atttaatgac gatgagcttg
     1081 tcgcagctct tgagcggaga tttattgtaa ttaatgttaa taaagatgta cccattgagt
     1141 gggtctaagg ttagggtttc aagatgtttt agggttttag ggttttaggg ttttacattt
     1201 gtttgcgtta tcccggttta actctgtatt aaccaacatt tttagccaca gcggctagcg
     1261 taggccgtta ggccgtttca ctcagcgcgc gagtttcgag cgtcgcctct gaaagtataa
     1321 atagcttcat ctccccttgc atgcattaaa aatgcctgct tccaaaaaga aagtcgttgt
     1381 tcgtattcct aagaaggtcc tcccgaggga tgttccccga ccttcgtctc agttacgccg
     1441 tgaacaacga aagaaactac tgaaagaagt tgatctcctc gaagaggcga gtttgataat
     1501 gcagactaat ccccctgggg atatgggttc tgccaacctc gaaagattgg cggtaatcgc
     1561 cgacgaactt gacgatcctt ccatttatcc acttgtgcac aacttttcaa aaggaaagaa
     1621 gagggcggtc cccgatgact atgcacccga tgacgttgtt cgggtgacta ataaaccagc
     1681 agtggttcca gtaattacag gtaggaaaag gttacgtgac gaaggtgttt ttgttcttcc
     1741 taaccgtggt ggtaattacc ctgctgtggc ctcgtcctcc aagttaccta ttggaggtac
     1801 tagcacgagg tccataggga ctcaaaccga tcctcccgaa agggaccctt cggttaccct
     1861 ttggagcgct gaattacttc ataagactgg cataaaaaga atagtttgtt atttgaacta
     1921 ataaaactat aaacactaat tgaaacttgt tccataataa ggcatttgcc aaatatacaa
     1981 atccacccca gtcggagtag tgactggggt acctgttccc gactgaaaga agaaggtagt
     2041 ttcctgattt gtatcctgaa tttcgaatgc aaattcgata atttgacgag ttgaagttcc
     2101 cccgctattg aactgacttg tgctcgaatt cgtgaatgaa ttagtaagag ccccatttgt
     2161 tccgacggtg attgtcggtg ctgcagtcgc cgttgaggaa ccctgtaaat ggtaactcac
     2221 gaagtacctt cctgccagtc ctcttggcag ggttactgcc gtgtccccga ttgttggggt
     2281 gaaagtactc tggttgttaa tcgtttgccc tgcgttgttt atgatggttg tgaaccactg
     2341 agtgttagtt attcccgtcc ctgtccccca ggcgtggaat gcttccatcg atggatttct
     2401 taaatcccgg agttgcggtt ttcgcaattc tatttcgtat gtaacccaca attcacccat
     2461 gttgacgtta gtcccctgca agccgactga agctatgtaa gtagaaccca agtcgtagaa
     2521 catttgatcg tccgtagtgg gtttggtgcc tgttctaatg tagtgcactt ttaacggatt
     2581 ttcctttgga tcacactcca ctgcgtgtac gaagtcctgc tgtggggcag cgctttgact
     2641 ccagtattgc tgtagtattt cctgtttgtt cgatggggct gcttgcgatg ccctgtactg
     2701 tgtacacatt aggaccgttc ctgtgcttgc gtctgtcgaa gtgaatgcat ctccgctatt
     2761 cgatttgaag tggaagacca gacccttgat gtcgtattcg gtgaagttat ttgccaacga
     2821 gcttaaccag ggaaaggtcc tttggagccc tggattcaag ctgaaagtct gaatgttgaa
     2881 tgcccttgca gttcctgagc tgaaaatgtc accgaggtat tctctgtgcc tgattatgac
     2941 accctcattt gtgttgtgca ttgctggaat ctctcctgca gcaagtgtgt ttttgcttat
     3001 tttgtacgaa ccagctccta accacttgct tatatttgca ccagcggatc ttccaaatgt
     3061 tgccccagct gcgctgttcc ctaggagagc tccgccgtac gcgcctagcc cttgcccggc
     3121 gtagcgtagg aaattcccga ggcgtcctct cgtcgctgcc ggagctcgtc tcgcagccgg
     3181 cctcgatttc cgtgagctcc tcataatacg tttcggcatt taactgacta atgattattt
     3241 taataattta cttgaagtgc gatattgagt ggcgcagcta cacaaatggt ggtctagca
//
LOCUS       CruV-204                3303 bp    DNA     circular     24-FEB-2020
DEFINITION  Crucivirus-204 isolate SR5_34549.
ACCESSION   CruV-204
KEYWORDS    .
SOURCE      Crucivirus-204
  ORGANISM  Crucivirus-204
            Unclassified.
REFERENCE   1  (bases 1 to 3303)
  AUTHORS   de la Higuera,I., Kasun,G.W., Torrance,E.L., Pratt,A.A.,
            Maluenda,A., Colombet,J., Bisseux,M., Ravet,V., Dayaram,A.,
            Stainton,D., Kraberger,S., Zawar-Reza,P., Goldstien,S., Briskie,J.,
            White,R., Taylor,H., Gomez,C., Ainley,D.G., Harding,J.S.,
            Fontenele,R.S., Schreck,J., Ribeiro,S.G., Oswald,S.A., Arnold,J.M.,
            Varsani,A., Enault,F. and Stedman,K.M.
  TITLE     Direct Submission
  JOURNAL   Submitted (24-FEB-2020) Biology, Portland State University, 1719 SW
            10th Ave, Portland, Oregon 97201, USA
COMMENT     Bankit Comment: ALT EMAIL:ide@pdx.edu
            Bankit Comment: TOTAL # OF SEQS:451
            Bankit Comment: GAP: est. length - alignment

            ##Assembly-Data-START##
            Assembly Method       :: MetaSPAdes v. 3.12; IDBA-UD v. 1.1.1;
                                     MEGAHIT v. 1.0.3
            Sequencing Technology :: 454; Illumina
            ##Assembly-Data-END##
FEATURES             Location/Qualifiers
     source          1..3303
                     /organism="Crucivirus-204"
                     /mol_type="genomic DNA"
                     /isolate="SR5_34549"
                     /isolation_source="Water"
                     /country="New Zealand"
                     /collection_date=2012
     misc_feature    join(3293..3303,1..17)
                     /note="stem-loop"
     misc_feature    1..9
                     /note="nonanucleotide"
     CDS             109..936
                     /codon_start=1
                     /product="Putative Replication Associated Protein"
                     /translation="MSRHKNFCFTFNNYENTDLVDNIDCRYIAYGKEVSSTGTPHLQG
                     WISFRNAKTKTAVCKLMPGCHISVMNGSVEQNTAYCSKEGELIERGIKPMSNAKIGEV
                     EIARWQNARELAKLGKFDEIDADIALRCYSTLKTIFKDNQVKPEPVEVKCFWIYGETG
                     TGKSHCVETNFPNCYKKAMDDMKWFCGYNNEEVIYLEDFDIYQVKWGGLLKRLADKWP
                     MQASVKGSMLYIRPKVVIVTSNYRPDQIWRDEQTLQPLERRFKIIEKTSQEQEIEFN*
                     "
     CDS             complement(1007..1360)
                     /codon_start=1
                     /product="putative ORF"
                     /translation="MSLRQSISNFFWCRRPSGSTAIHNFTGRAFGASLGPFSISCSFS
                     ISSLISTFLNATTLLLVTIGTTLLTVRWISRCISSSSWFLWHHHYIHWHLWHHSITHL
                     HFFWLSFYIFFRYKN*"
     CDS             1052..1426
                     /codon_start=1
                     /product="putative ORF"
                     /translation="MQMCNGMMPQMPMYVMMMPQKPTRRRYTPGYPTDSKKRCANGYQ
                     KKCRCVKKSGYKRTYRKRATNTKWSQTGSKSSSGKVMYGRGSRWSSAPKEVTDALTQT
                     QSVDFGTSMDMSNVSKVTNLVI*"
     CDS             complement(1539..2495)
                     /codon_start=1
                     /product="Putative CruV Capsid Protein"
                     /translation="MTTIVHREFIRDIVSSSSVGAFEVQSFVVNPGSTNTFPWLSVIS
                     QNFEQYYIHGLQFFFRSGAGDNSTTGQQGNVILAAEYNVNSPPYFNKQVMENSAGARS
                     VKQSNSVEFNLKSVNRTLFTRSGQIPAGDSLKFYDHAIFQIATVGFPTASFNAGELWV
                     AYKISFIKPQIPANFGGVIQSGLISRSGASSANLLGSATVIAPIGPIAISVPSLNNVR
                     FDNLSIGNSYTIMIHYNGTAALIAALSVGALSGFVGKNLFNAKTTTNANGGTGGTSGN
                     YTYETCFTATSTTGTISFNGGTPPSAAIVDILVRSIDNTLVA*"
ORIGIN
        1 tattattacc tagaccctcc gtgcagtgta atttgtggct gtgtggtttt aaaaaaaaaa
       61 tcggtcgcaa attatataaa agcacgatga ttttgaaagt cgaaaattat gtcaagacat
      121 aaaaattttt gctttacttt caacaattat gaaaatacgg atttagtcga caatatcgat
      181 tgtcgctata ttgcgtatgg aaaagaagtg tcatccaccg gtacaccaca tttgcaaggt
      241 tggattagtt ttaggaacgc taaaaccaaa acagcagttt gcaaattgat gccgggatgt
      301 cacattagtg taatgaatgg ttctgtggaa cagaacacag catattgcag taaagaggga
      361 gaattaattg aaagaggaat taaacctatg tcgaatgcga aaattggaga agttgagatt
      421 gctcgctggc agaatgctcg tgagttagct aaacttggta aatttgatga aattgatgct
      481 gatattgcac taagatgtta ttcaactctg aagacaatat tcaaagacaa tcaagtgaaa
      541 cccgaaccag ttgaagttaa atgtttttgg atatacggag aaacaggaac agggaagtca
      601 cattgtgttg aaaccaattt tccaaattgt tataaaaaag caatggacga tatgaagtgg
      661 ttttgcggat acaataatga agaggtgata tatttggaag actttgacat ttaccaagtg
      721 aagtggggtg gtttgttaaa acgactcgct gataaatggc caatgcaagc gtctgtaaaa
      781 ggatctatgt tgtacataag accaaaagta gtaattgtga cttccaatta tcgacccgac
      841 caaatttggc gggatgaaca gacgttacaa ccgttggaac gacggtttaa gataattgag
      901 aaaacgtcac aagaacaaga aattgagttt aactaacaaa atgtggataa taatatatta
      961 cataatatta attctgtgga tcgcagcctt tttaattggt tgttacttag tttttgtacc
     1021 tgaaaaatat atagaaactt aaccaaaaaa aatgcaaatg tgtaatggaa tgatgccaca
     1081 gatgccaatg tatgtaatga tgatgccaca aaaaccaact cgaagacgat atacacctgg
     1141 atatccaacg gacagtaaga agcgttgtgc caatggttac caaaagaagt gtcgttgcgt
     1201 taagaaaagt ggatataaga gaacttatcg aaaacgagca actaatacta aatggtccca
     1261 aactggctcc aaaagctctt ccggtaaagt tatgtatggc cgtggatcca gatggtcgtc
     1321 tgcaccaaaa gaagttactg atgctttgac gcaaactcaa agtgttgatt ttggtacatc
     1381 aatggatatg tcaaatgtat ctaaagtaac taatttagta atttagcgtc aaacagagga
     1441 agatgaagta caatttgtaa cccctaaaaa ggtacgcatt cttgctcaag aaactcctca
     1501 atttgattcc acctacgaac taaatttatt tcaataaatt aagcgaccaa agtattgtca
     1561 attgatctga ctaagatatc tacgattgca gcacttggtg gcgtacctcc attgaaagaa
     1621 atagttccag tagtacttgt tgcagtgaaa caagtctcgt aagtgtaatt ccctgatgta
     1681 cctccagtac caccattagc gttagttgta gtctttgcat tgaaaaggtt ctttccaaca
     1741 aaaccagata atgctccaac agataatgca gcaattaatg ctgcagttcc gttgtagtga
     1801 atcataatgg tgtaagagtt accaattgat agattgtcaa atctgacatt gtttagagaa
     1861 ggtactgaga ttgcaatagg accaatagga gcaataacag tagcgcttcc caacaagtta
     1921 gcagaacttg cccctgatcg ggagattaaa ccagattgaa tgacaccacc gaaatttgcc
     1981 ggaatttgtg gtttaatgaa actgatttta taagctaccc ataattcacc agcattgaaa
     2041 cttgcagttg gaaatcctac ggttgcaatt tgaaaaattg catgatcgta gaattttaaa
     2101 gaatctccag ctggaatttg accagatcgt gtaaataaag ttctgttaac agatttaaga
     2161 ttgaattcta cagaatttga ttgttttaca gatcttgcac cagcagaatt ctccataact
     2221 tgtttgttaa aataaggagg agaattgaca ttgtattctg cagccagaat aacattgccc
     2281 tgttgaccag tagtggagtt gtccccagca ccacttcgaa agaaaaactg tagtccatgg
     2341 atgtagtatt gttcaaaatt ttgagaaata acagacagcc atggaaatgt gttagtggaa
     2401 ccagggttaa caacaaaact ttgaacttca aaagctccaa ctgaagatga agaaacaata
     2461 tcacgaatga attcacgatg aacaatagta gtcattttat ttaggaaaga aaattaatcg
     2521 aatttaaaat tggtttgcgg ggcagagcca accgtaacat ttttcaattt ttctttaatt
     2581 tgctttctgt ttggatgttt agagtcataa atatcaccac ctaacttcca accagttgtg
     2641 gcaccaggta catctccaaa gatgaaacct aaggcggcac cactggcttg tattggtttt
     2701 tttactaaga agtctgcaaa tttttcagct tcgtaaaacc aaggatcagt catattttgt
     2761 ttgcaaaact aattatatat taaatgatct aaaacagctt gtagcattgc aattttctcc
     2821 atgttataat gaattagtag ttgattccca tattggtcat cagcgactaa ttcgtctact
     2881 attgaccaat agtagtttaa cttgcgacga acaatttcgt acaaatctat agaagagtca
     2941 tccaatgaag tgttaatagt tcgagccatt gaagaattaa aacggggaag ttggttctta
     3001 tataggcttt gtggctgtga taataaaaaa atttaatata tgcggctaac ggctcgtcat
     3061 ttaattttaa ccggaaatgg catggggtga cgctatggcg gtgaggaggg aaccgacgaa
     3121 tccccagata gtggcgaacc ccaatggcac agtcatggcc tcccggctaa gggttattac
     3181 aatcaatctg actagcatta tttgtagtaa aaagggggtt cgggtggaaa gcccgatttg
     3241 ggttgcggta acgcaacttt ttttggaaaa aaaaattgaa aaactgcacg gcgggtctag
     3301 tca
//
LOCUS       CruV-205                3303 bp    DNA     circular     24-FEB-2020
DEFINITION  Crucivirus-205 isolate Sty_13.
ACCESSION   CruV-205
KEYWORDS    .
SOURCE      Crucivirus-205
  ORGANISM  Crucivirus-205
            Unclassified.
REFERENCE   1  (bases 1 to 3303)
  AUTHORS   de la Higuera,I., Kasun,G.W., Torrance,E.L., Pratt,A.A.,
            Maluenda,A., Colombet,J., Bisseux,M., Ravet,V., Dayaram,A.,
            Stainton,D., Kraberger,S., Zawar-Reza,P., Goldstien,S., Briskie,J.,
            White,R., Taylor,H., Gomez,C., Ainley,D.G., Harding,J.S.,
            Fontenele,R.S., Schreck,J., Ribeiro,S.G., Oswald,S.A., Arnold,J.M.,
            Varsani,A., Enault,F. and Stedman,K.M.
  TITLE     Direct Submission
  JOURNAL   Submitted (24-FEB-2020) Biology, Portland State University, 1719 SW
            10th Ave, Portland, Oregon 97201, USA
COMMENT     Bankit Comment: ALT EMAIL:ide@pdx.edu
            Bankit Comment: TOTAL # OF SEQS:451
            Bankit Comment: GAP: est. length - alignment

            ##Assembly-Data-START##
            Assembly Method       :: MetaSPAdes v. 3.12; IDBA-UD v. 1.1.1;
                                     MEGAHIT v. 1.0.3
            Sequencing Technology :: 454; Illumina
            ##Assembly-Data-END##
FEATURES             Location/Qualifiers
     source          1..3303
                     /organism="Crucivirus-205"
                     /mol_type="genomic DNA"
                     /isolate="Sty_13"
                     /isolation_source="Water"
                     /country="New Zealand"
                     /collection_date=2012
     misc_feature    join(3293..3303,1..17)
                     /note="stem-loop"
     misc_feature    1..9
                     /note="nonanucleotide"
     CDS             109..936
                     /codon_start=1
                     /product="Putative Replication Associated Protein"
                     /translation="MSRHKNFCFTFNNYENTDLVDNIDCRYIAYGKEVSSTGTPHLQG
                     WISFRNAKTKTAVCKLMPGCHISVMNGSVEQNTAYCSKEGELIERGIKPMSNAKIGEV
                     EIARWQNARELAKLGKFDEIDADIALRCYSTLKTIFKDNQVKPEPVEVKCFWIYGETG
                     TGKSHCVETNFPNCYKKAMDDMKWFCGYNNEEVIYLEDFDIYQVKWGGLLKRLADKWP
                     MQASVKGSMLYIRPKVVIVTSNYRPDQIWRDEQTLQPLERRFKIIEKTSQEQEIEFN*
                     "
     CDS             complement(1007..1360)
                     /codon_start=1
                     /product="putative ORF"
                     /translation="MSLRQSISNFFWCRRPSGSTAIHNFTGRAFGASLGPFSISCSFS
                     ISSLISTFLNATTLLLVTIGTTLLTVRWISRCISSSSWFLWHHHYIHWHLWHHSITHL
                     HFFWLSFYIFFRYKN*"
     CDS             1052..1426
                     /codon_start=1
                     /product="putative ORF"
                     /translation="MQMCNGMMPQMPMYVMMMPQKPTRRRYTPGYPTDSKKRCANGYQ
                     KKCRCVKKSGYKRTYRKRATNTKWSQTGSKSSSGKVMYGRGSRWSSAPKEVTDALTQT
                     QSVDFGTSMDMSNVSKVTNLVI*"
     CDS             complement(1539..2495)
                     /codon_start=1
                     /product="Putative CruV Capsid Protein"
                     /translation="MTTIVHREFIRDIVSSSSVGAFEVQSFVVNPGSTNTFPWLSVIA
                     QNYEQYYIHGLQFFFRSGAGDNSTTGQQGNVILAAEYNVNSPPYFNKQVMENSAGARS
                     VKQSNSVEFNLKSVDRTLFTRSGQIPAGDSLKFYDHAIFQIATVGFPTASFNAGELWV
                     AYKISFIKPQIPANFGGVIQSGLISRSGASSANLLGSATVIAPIGPIAISVPSLNNVR
                     FDNLSIGNSYTIMIHYNGTAALIAALSVGALSGFVGKNLFNAKTTTNANGGTGGTSGN
                     YTYETCFTATSTTGTISFNGGTPPSAAIVDILVRSIDNTLVA*"
ORIGIN
        1 tattattacc tagaccctcc gtgcagtgta atttgtggct gtgtggtttt aaaaaaaaaa
       61 tcggtcgcaa attatataaa agcacgatga ttttgaaagt cgaaaattat gtcaagacat
      121 aaaaattttt gctttacttt caacaattat gaaaatacgg atttagtcga caatatcgat
      181 tgtcgctata ttgcgtatgg aaaagaagtg tcatccaccg gtacaccaca tttgcaaggt
      241 tggattagtt ttaggaacgc taaaaccaaa acagcagttt gcaaattgat gccgggatgt
      301 cacattagtg taatgaatgg ttctgtggaa cagaacacag catattgcag taaagaggga
      361 gaattaattg aaagaggaat taaacctatg tcgaatgcga aaattggaga agttgagatt
      421 gctcgctggc agaatgctcg tgagttagct aaacttggta aatttgatga aattgatgct
      481 gatattgcac taagatgtta ttcaactctg aagacaatat tcaaagacaa tcaagtgaaa
      541 cccgaaccag ttgaagttaa atgtttttgg atatacggag aaacaggaac agggaagtca
      601 cattgtgttg aaaccaattt tccaaattgt tataaaaaag caatggacga tatgaagtgg
      661 ttttgcggat acaataatga agaggtgata tatttggaag actttgacat ttaccaagtg
      721 aagtggggtg gtttgttaaa acgactcgct gataaatggc caatgcaagc gtctgtaaaa
      781 ggatctatgt tgtacataag accaaaagta gtaattgtga cttccaatta tcgacccgac
      841 caaatttggc gggatgaaca gacgttacaa ccgttggaac gacggtttaa gataattgag
      901 aaaacgtcac aagaacaaga aattgagttt aactaacaaa atgtggataa taatatatta
      961 cataatatta attctgtgga tcgcagcctt tttaattggt tgttacttag tttttgtacc
     1021 tgaaaaatat atagaaactt aaccaaaaaa aatgcaaatg tgtaatggaa tgatgccaca
     1081 gatgccaatg tatgtaatga tgatgccaca aaaaccaact cgaagacgat atacacctgg
     1141 atatccaacg gacagtaaga agcgttgtgc caatggttac caaaagaagt gtcgttgcgt
     1201 taagaaaagt ggatataaga gaacttatcg aaaacgagca actaatacta aatggtccca
     1261 aactggctcc aaaagctctt ccggtaaagt tatgtatggc cgtggatcca gatggtcgtc
     1321 tgcaccaaaa gaagttactg atgctttgac gcaaactcaa agtgttgatt ttggtacatc
     1381 aatggatatg tcaaatgtat ctaaagtaac taatttagta atttagcgtc aaacagagga
     1441 agatgaagta caatttgtaa cccctaaaaa ggtacgcatt cttgctcaag aaactcctca
     1501 atttgattcc acctacgaac taaatttatt tcaataaatt aagcgaccaa agtattgtca
     1561 attgatctga ctaagatatc tacgattgca gcacttggtg gcgtacctcc attgaaagaa
     1621 atagttccag tagtacttgt tgcagtgaaa caagtctcgt aagtgtaatt ccctgatgta
     1681 cctccagtac caccattagc gttagttgta gtctttgcat tgaaaaggtt ctttccaaca
     1741 aaaccagata atgctccaac agataatgca gcaattaatg ctgcagttcc gttgtagtga
     1801 atcataatgg tgtaagagtt accaattgat agattgtcaa atctgacatt gtttagagaa
     1861 ggtactgaga ttgcaatagg accaatagga gcaataacag tagcgcttcc caacaagtta
     1921 gcagaacttg cccctgatcg ggagattaaa ccagattgaa tgacaccacc gaaatttgcc
     1981 ggaatttgtg gtttaatgaa actgatttta taagctaccc ataattcacc agcattgaaa
     2041 cttgcagttg gaaatcctac agttgcaatt tgaaaaattg catgatcgta gaatttcaaa
     2101 gaatcaccag ctggaatttg accagatctg gtaaataaag ttctgtcaac agatttaaga
     2161 ttgaattcaa cagaatttga ttgttttaca gatcttgcac cagcagaatt ctccataact
     2221 tgtttgttaa aataaggagg agaattgaca ttgtattctg cagctagaat aacattccct
     2281 tgttgaccag tagtggagtt gtccccagca ccactgcgaa aaaaaaattg tagtccatgg
     2341 atgtagtatt gttcataatt ttgagcaata acggatagcc atggaaatgt gttagtggaa
     2401 ccagggttaa caacaaaact ttgaacttca aaggctccaa ctgaagatga agaaacaata
     2461 tcacgaatga attcacgatg aacaatagta gtcattttat ttaggaaaga aaattaatcg
     2521 aatttaaaat tggtttgcgg ggcagagcca accgtaacat ttttcaattt ttctttaatt
     2581 tgctttctgt ttggatgttt agagtcataa atatcaccac ctaacttcca accagttgtg
     2641 gcaccaggta catctccaaa gatgaaacct aaggcggcac cactggcttg tattggtttt
     2701 tttactaaga agtctgcaaa tttttcagct tcgtaaaacc aaggatcagt catattttgt
     2761 ttgcaaaact aattatatat taaatgatct aaaacagctt gtagcattgc aattttctcc
     2821 atgttataat gaattagtag ttgattccca tattggtcat cagcgactaa ttcgtctact
     2881 attgaccaat agtagtttaa cttgcgacga acaatttcgt acaaatctat agaagagtca
     2941 tccaatgaag tgttaatagt tcgagccatt gaagaattaa aacggggaag ttggttctta
     3001 tataggcttt gtggctgtga taataaaaaa atttaatata tgcggctaac ggctcgtcat
     3061 ttaattttaa ccggaaatgg catggggtga cgctatggcg gtgaggaggg aaccgacgaa
     3121 tccccagata gtggcgaacc ccaatggcac agtcatggcc tcccggctaa gggttattac
     3181 aatcaatctg actagcatta tttgtagtaa aaagggggtt cgggtggaaa gcccgatttg
     3241 ggttgcggta acgcaacttt ttttggaaaa aaaaattgaa aaactgcacg gcgggtctag
     3301 tca
//
LOCUS       CruV-206                3304 bp    DNA     circular     24-FEB-2020
DEFINITION  Crucivirus-206 isolate 001597_virusbaton.
ACCESSION   CruV-206
KEYWORDS    .
SOURCE      Crucivirus-206
  ORGANISM  Crucivirus-206
            Unclassified.
REFERENCE   1  (bases 1 to 3304)
  AUTHORS   de la Higuera,I., Kasun,G.W., Torrance,E.L., Pratt,A.A.,
            Maluenda,A., Colombet,J., Bisseux,M., Ravet,V., Dayaram,A.,
            Stainton,D., Kraberger,S., Zawar-Reza,P., Goldstien,S., Briskie,J.,
            White,R., Taylor,H., Gomez,C., Ainley,D.G., Harding,J.S.,
            Fontenele,R.S., Schreck,J., Ribeiro,S.G., Oswald,S.A., Arnold,J.M.,
            Varsani,A., Enault,F. and Stedman,K.M.
  TITLE     Direct Submission
  JOURNAL   Submitted (24-FEB-2020) Biology, Portland State University, 1719 SW
            10th Ave, Portland, Oregon 97201, USA
COMMENT     Bankit Comment: ALT EMAIL:ide@pdx.edu
            Bankit Comment: TOTAL # OF SEQS:451
            Bankit Comment: GAP: est. length - alignment

            ##Assembly-Data-START##
            Assembly Method       :: MetaSPAdes v. 3.12; IDBA-UD v. 1.1.1;
                                     MEGAHIT v. 1.0.3
            Sequencing Technology :: 454; Illumina
            ##Assembly-Data-END##
FEATURES             Location/Qualifiers
     source          1..3304
                     /organism="Crucivirus-206"
                     /mol_type="genomic DNA"
                     /isolate="001597_virusbaton"
                     /isolation_source="Water"
                     /country="France"
                     /collection_date=2013
     misc_feature    join(3294..3304,1..17)
                     /note="stem-loop"
     misc_feature    1..9
                     /note="nonanucleotide"
     CDS             complement(200..1240)
                     /codon_start=1
                     /product="Putative Replication Associated Protein"
                     /translation="MGERREIILSEAEAVDDKSRKKNWVFTWNNYTVEDLKTVNEEWL
                     KLSDMRCVVYEPEIGKQGTPHLQGFFCFDKLKSFKQVKEMIDKRVWFAQMRKPIEASL
                     KYCSKEGGTIYLGEVPKTQKEKGALGKEYVSKYGHVWSHLIDDIKGGMTFKTAAEKYP
                     DAHGMYPRGFKEKFDLFAPKPVFNLPERVEKLYDWQTECIRILGSDPDDRSVYWIWSK
                     EGNTGKSDMLKHLVSVNGFQPMQNAPTRDLACAWKGDNVVFDYSRDEQAGGQINYAVL
                     EHIKNRLLFSPKYESQTKVSEDFKSVHVVCFSNAPPDVSKLSQDRWNVYRIENIENKP
                     WLKQTVCENALF*"
     CDS             1499..2875
                     /codon_start=1
                     /product="Putative CruV Capsid Protein"
                     /translation="MPQVRAAPRRRLAVAPKRRAPIRKASYAAPVKQPSMRAAKPRSQ
                     AGNAFRSAGSAGGTLLGTALGGPAGGALGGMLGRGAGAMLSKIFGHGDYQVSNIGQIK
                     ENSLVLSNAANIPQFGTGRVAVNFKHREFLGDVISSNTAGAFKIDSYPLNPGLVGTFP
                     WLSQVVGDSFQQHRINGMTFEYRPMSGDALNSTNTALGSVIMATDYDSPDAIFATKQQ
                     MENTEYGVSCKPSVGMMHAIECARGQTPVNELYIRAFANPSNTDLRLYDLGRFSIATT
                     GCQAAHVNLGELWVTYDIDAFKAIQQPPGYIAPVSVYSLVAVDATHSLGLTPSTNLGV
                     LNQIGTTLDLVAGVSHLNIPLLGLPTGIIYQLQYKVLGDSTASVALVTPVPSGGMVEL
                     GNAGFSSPAATGTSTTRAWSAYYTYDGTGTLALPPRIAIPGGVVPANISLAELSLTQV
                     GAGIINVP*"
ORIGIN
        1 tagtattaca gaccccctag atatcatatc attagatata ggtttagcgc ttcagcgcgg
       61 ttattatgga ctttaggcca gaaaatacgg ggtgtaccta ggtatcacca tcgtatcatt
      121 ttgctctacc ctagatatca tcgtcaggtg agctctaaat gcaaaaaaaa tgagatttgt
      181 tattcaccaa atttggtgat tagaaaagag cattttcaca cactgtttgc tttagccaag
      241 gtttgttttc gatattctca atacgataga cgttccaacg atcttgtgac aatttcgaga
      301 catccggagg agcgttcgag aaacacacaa cgtgaacgga tttgaagtct tcagatactt
      361 tagtctgtga ctcgtattta ggtgagaaga gtagacggtt cttgatgtgc tctagcacag
      421 catagtttat ttgacctcca gcttgctcgt cacgactgta gtcaaataca acgttatctc
      481 ctttccaagc acatgctaga tctctagttg gtgcattctg cattggttga aaaccattga
      541 cagatacaag gtgtttaagc atatcggatt tacccgtatt gccttctttg gaccagatcc
      601 agtaaacgga tctatcatca ggatcgcttc caagtatcct tatgcattct gtttgccagt
      661 cgtagagttt ctctacacgt tctggtagat taaagacagg tttaggagcg aatagatcaa
      721 acttttcttt gaatccgcga gggtacatac cgtgggcatc agggtatttt tccgctgctg
      781 tcttgaaggt catgcctcct ttgatgtcat cgattaggtg actccaaaca tgtccgtatt
      841 tagacacata ctctttaccg agtgctcctt tctctttctg agttttgggc acttctccaa
      901 ggtaaatagt tcctccttct ttggagcaat actttagtga tgcttcaatg ggtttgcgca
      961 tttgtgcgaa ccatactctt ttatcaatca tctccttcac ttgtttgaag gatttgagtt
     1021 tatcaaagca aaagaatcct tgaagatgag gtgtgccttg cttgcctatc tcaggttcgt
     1081 aaacaacaca tcgcatgtct gacagcttga gccattcctc attgactgtt ttcagatctt
     1141 ctactgtgta gttgttccac gtgaagaccc agttcttctt tcgtgatttg tcatcgacag
     1201 cttcagcttc ggacagaata atctctcggc gctcccccat ccctaaagga gcaacgggtg
     1261 attcttttgt ctcgactgaa tgcttttctt cgacttgttc cagtggcata ctgccgcgca
     1321 gaggagccaa gttcgcagat agatgttcaa atgcatcaga catgattcgg tgtaggtata
     1381 ttctacctag atatcatttc taataacatt aattaattat taattattta attttctttt
     1441 gagaaagttg cggtgcatag ggtataccca tctgctaagt caaaacttaa ctcaaggtat
     1501 gcctcaagtg cgcgctgccc ctcgtcgacg gcttgccgtt gctccaaagc gtcgtgctcc
     1561 tattcgtaag gcatcttatg cggctcccgt aaagcaacct tctatgcgag ctgccaaacc
     1621 tcgttctcag gctgggaacg ccttccgatc agctggttct gctggaggta ctttactcgg
     1681 tacggctctt ggaggccctg caggaggagc tcttggcggt atgcttggcc gtggagctgg
     1741 tgctatgctg agtaagattt ttggccatgg tgactatcaa gtctccaata ttggccaaat
     1801 taaagagaac agcttggttt tgtccaatgc tgcaaacata cctcagtttg gaactggacg
     1861 tgttgcggtg aattttaagc atcgcgagtt ccttggggat gttatcagtt ctaatactgc
     1921 tggcgcattt aaaatcgaca gttacccgct gaatcctggt ttggtgggta catttccatg
     1981 gctttcgcag gttgtgggcg attcgttcca gcaacatcga atcaatggta tgacgtttga
     2041 gtatcgaccg atgtccggtg atgcactcaa ctctacaaac accgcacttg gttcggtgat
     2101 tatggctacg gattacgaca gtcccgatgc tatcttcgct acgaagcagc agatggagaa
     2161 tacggagtat ggtgtgtcat gcaagccttc agtgggcatg atgcatgcta ttgagtgtgc
     2221 tcgtgggcaa actccagtca atgagctcta tattagagcg tttgctaatc ccagcaatac
     2281 tgacttgcgt ttgtatgatt tgggacgatt ttccattgca actactggtt gccaggctgc
     2341 acacgtgaac cttggtgaat tgtgggtgac gtatgacatt gatgccttta aggctattca
     2401 acagccacct ggatatatcg cacctgtgtc agtgtattct ttggtcgcag tggatgctac
     2461 gcattcatta ggccttacac cttcaacaaa tcttggtgtg ttgaatcaga ttggtacaac
     2521 cctcgattta gtcgcagggg ttagccatct gaatattcct ttgcttggtt taccaacagg
     2581 tatcatttat cagctccaat ataaggtatt gggtgattct actgcatccg ttgctcttgt
     2641 tactccggtt ccgtccgggg gaatggttga gctaggaaat gccgggtttt cgtctccagc
     2701 tgcgactgga acttcgacaa cccgagcatg gtcagcttat tatacgtatg atgggacagg
     2761 cactttggcg cttcctccac ggattgcaat tccgggtgga gtcgttcctg caaacatatc
     2821 tctagcagag ttatcgctaa cgcaggttgg tgctgggatc atcaatgttc catagtttgt
     2881 ctatggtttt gaggtattaa aaaaaacaaa taaacaaagc cttttaggct atattttttg
     2941 tcaatttgaa gaacatgatc ggaaacgatg ttctgtgtca aataataaat aaataattca
     3001 acatagttgt aattgttccc aaatgttcat ctggtcgctt gcgatccagg atttgaagaa
     3061 catgatcgga aacgatggcc cgtgtacata aaatcaacaa aaaaaggacg cgcgtgtttg
     3121 gtgtaacctt tatgtatgtc tgagggacgc cgcgcggacg tcgaggggcg tgggcgccgg
     3181 agcgccccca cacttgtttg gggcgctctg tcgcccacgc ctcttgccgt ctgtgtggcg
     3241 tgagggtaat acatgtaatg tcttacttgg gggagatttg tgtaaacatg atcgggggtc
     3301 tcta
//
LOCUS       CruV-207                3304 bp    DNA     circular     24-FEB-2020
DEFINITION  Crucivirus-207 isolate GP2_73290.
ACCESSION   CruV-207
KEYWORDS    .
SOURCE      Crucivirus-207
  ORGANISM  Crucivirus-207
            Unclassified.
REFERENCE   1  (bases 1 to 3304)
  AUTHORS   de la Higuera,I., Kasun,G.W., Torrance,E.L., Pratt,A.A.,
            Maluenda,A., Colombet,J., Bisseux,M., Ravet,V., Dayaram,A.,
            Stainton,D., Kraberger,S., Zawar-Reza,P., Goldstien,S., Briskie,J.,
            White,R., Taylor,H., Gomez,C., Ainley,D.G., Harding,J.S.,
            Fontenele,R.S., Schreck,J., Ribeiro,S.G., Oswald,S.A., Arnold,J.M.,
            Varsani,A., Enault,F. and Stedman,K.M.
  TITLE     Direct Submission
  JOURNAL   Submitted (24-FEB-2020) Biology, Portland State University, 1719 SW
            10th Ave, Portland, Oregon 97201, USA
COMMENT     Bankit Comment: ALT EMAIL:ide@pdx.edu
            Bankit Comment: TOTAL # OF SEQS:451
            Bankit Comment: GAP: est. length - alignment

            ##Assembly-Data-START##
            Assembly Method       :: MetaSPAdes v. 3.12; IDBA-UD v. 1.1.1;
                                     MEGAHIT v. 1.0.3
            Sequencing Technology :: 454; Illumina
            ##Assembly-Data-END##
FEATURES             Location/Qualifiers
     source          1..3304
                     /organism="Crucivirus-207"
                     /mol_type="genomic DNA"
                     /isolate="GP2_73290"
                     /isolation_source="Sediments"
                     /country="New Zealand"
                     /collection_date=2014
     CDS             complement(1..1200)
                     /codon_start=1
                     /product="Putative Replication Associated Protein"
                     /translation="MPAPKVTIPQYTNWCFTFNNYEDEDIEKLKNFEIIKYCVFGYEK
                     GESGTKHLQGYFEIATKRTIKGLKKLLLDHSPRFKFHFEPRFGSQKQAIAYCKKGSQS
                     HEEWTKYGVDGENYGKDYDGLEWGIKKKQGGRTDLDFARQLATDYGMSEVSAKCNLQA
                     IKTAQLYLSYNEAPRELETNPIVIYIYGEPEAGKSFYARQLTKELTPYQWDPAEMGKW
                     WDGYDGHECVILDDIRLQHTSEAMLLKLLDRYSCQVQVKGGARQIQASVFVITTLKRP
                     EEFWTSQENCKQFTRRITITLRKESWDTYSITNVNKNFDEVTARFETDNLFLKKLLAD
                     SKKWGGNTDPPTILNILNDELSLEKCLEVEDIFDDIDDIQLNQSIMINYEKNDNIEFW
                     EDEWQAN*"
     misc_feature    164..190
                     /note="stem-loop"
     misc_feature    174..182
                     /note="nonanucleotide"
     CDS             complement(1697..3130)
                     /codon_start=1
                     /product="Putative CruV Capsid Protein"
                     /translation="MNGFLLDSKKSTINYFFLFFYVWVYIYYTMSGKKYTQAQKLAYY
                     KKKSQRTYRGKGAYFRGPNPRGAPAPKSRAYGEMVGTGLGSMVKYIPGGAAFAAPAAL
                     LGQKLGGYLGNKLGSYMGWGEYRVKKNSLVVPEGNSPAQMHGDGRTTRICHREYITDI
                     ISSGTAGAFKLQSFQMQPGNNLIFPWLSSVALSYQKYKIHGAIVEFKSGSGDAINGTN
                     TSLGECIISSNYNCADSNFVNRMQMENTQYCSSAKPSVSFVHIIECDPALQAQETLYV
                     SPTTVPQAGLGINDINFVNVQVATVGVQGTNVNLGSLYITYDIELIQPLSDYIDKYPK
                     SDWFAPTLATVSAANPCGTANVADKGNSIGGILIGNTYIFPPLLQEGLFKFQWYAIGT
                     AALYTAPGVTLTNCVIRATMGGDVVGALAFPQNGVNATNVMLIYVIEITGQNASFVIN
                     SGGTIPTAVTGCALMVDQIDRDLVGPG*"
ORIGIN
        1 ttaattagct tgccattcat cttcccaaaa ttcaatatta tcattttttt cataattaat
       61 cataatactt tgatttaatt gaatatcatc aatatcatcg aaaatatctt caacctctaa
      121 gcatttttct aaacttagtt catcattcaa aatatttaat attgtggggg ggtcagtatt
      181 acccccccac ttcttgctat ctgctagcaa tttttttaaa aataaattat cagtctcgaa
      241 acgagctgta acttcatcga agtttttgtt aacgttggtt atactgtaag tatcccaact
      301 ttctttacgg agagttatgg taatacgtct ggtaaactgt ttacaatttt cctgactagt
      361 ccaaaactct tcaggtcgtt taagcgtggt aatgacaaat acagaagcct gtatctggcg
      421 tgcaccacct ttaacttgga cctgacaact atagcgatca agtaacttta gtaacattgc
      481 ttcgctagta tgttgcaaac gaatgtcgtc tagaataacg cattcatgtc cgtcatatcc
      541 atcccaccac ttacccattt cagcgggatc ccactgatat ggagtaagtt ctttcgtaag
      601 ctgacgagcg tagaagcttt taccagcctc aggctcaccg tatatatata taacgattgg
      661 attggtttcg agctcacgtg gagcctcgtt gtatgataaa tataattgtg ctgttttaat
      721 agcttgtaaa ttacattttg cagatacttc actcattcca taatctgtag caagttgtct
      781 ggcaaagtcc agatcggtac gaccaccttg ttttttcttg atgccccatt caagaccatc
      841 ataatcttta ccgtagtttt caccatctac gccatatttt gtccattctt catgtgattg
      901 agaacccttt ttgcagtatg caatagcttg tttttgagag ccgaagcgag gttcaaagtg
      961 aaacttgaaa cgaggtgaat gatccaataa caacttcttc aatcctttaa ttgtacgttt
     1021 agtagcaatt tcaaagtaac cttgtaagtg ttttgtacca ctttcacctt tttcataacc
     1081 aaatacgcag tatttaatta tttcaaaatt tttcaatttt tcaatatctt catcttcata
     1141 attgttgaat gtaaaacacc aattagtgta ttgcggtatc gtgactttag gagcaggcat
     1201 gttgtatata aatagcaaga agttaatttt ttaaaaaaat aaaaaattaa ttaattaaat
     1261 atatagatgg agacagaaga ggttagacaa cgtataaact ctgaatgtgt caagataata
     1321 tatttaaaaa ataataaata tgtaatgtca tatttttcat tcgcaccact catttttgat
     1381 actttcagtg cttcagatga tgaagatgaa aggcaagata attaacaaaa actcctatca
     1441 tcacccccct tgagtctaat gtactatgtt aaggtgaaag gcaactttct catagtacat
     1501 tacaattata cgtataattg taaaactcat taaaaaaaat taaaataatt aattgttttt
     1561 ttccgcgaat aaaggaaaca aataattagt tcatgacact cgcgaagcgg gaatgaacgc
     1621 ttttttatgg cgtagcaata aaaaaaatta tttttttaat ttatgaagcg acaacgagtg
     1681 atgttctcac gagtgcttat ccgggaccga caagatcacg atcgatttga tcgaccatga
     1741 gagcacatcc cgtaacagct gtagggattg taccacctga attaataaca aaactggcat
     1801 tttgaccagt tatctcaatt acataaatta acattacgtt agtagcatta acaccatttt
     1861 gagggaacgc aagagcaccg acaacatcac cacccatagt agctcgaata acgcagttag
     1921 tgagcgttac accaggtgca gtatataaag cagctgtacc aattgcatac cattggaact
     1981 taaacaaacc ctcttgtaga agcggtggaa atatataagt atttccaatt aaaataccac
     2041 caattgaatt acctttatct gcaacattag cagtaccgca aggattagca gcagaaacag
     2101 tagcgagagt aggagcaaac caatcgctct taggatattt atcaatataa tcacttagag
     2161 gttgaattaa ttcaatatca taggtaatat acaaactacc caaattaacg tttgtacctt
     2221 gaacaccaac agtggcaact tgcacattta caaaattgat atcgttaata ccaagcccag
     2281 cttgaggaac tgttgtagga gaaacatata atgtttcctg agcttgcaat gcgggatcac
     2341 attcaataat gtgaacgaag ctaactgaag gtttagcgct agaacaatat tgagtgttct
     2401 ccatttgcat gcgattaaca aaattactat cagcacaatt atagttagaa cttataatgc
     2461 actccccgag actggtgttc gtgccattaa tggcgtcgcc ggagccactt ttaaactcaa
     2521 caatagcacc atgaatttta tacttttggt aactcaaagc aacagatgaa agccaaggga
     2581 atataagatt atttccgggc tgcatttgga agctttgaag tttaaacgca ccagcggtac
     2641 cagaacttat aatatctgta atatattcac gatggcaaat acgtgtagta cgtccatcac
     2701 catgcatttg agccggagaa ttgccttccg ggaccactaa actattcttt ttaacgcgat
     2761 attcacccca tcccatatag cttcctaact tgttgccaag gtatccacca agtttttgac
     2821 caagcaaagc agcaggcgca gcgaaggcag caccaccagg aatgtactta accatacttc
     2881 ctagaccagt accaaccatt tcaccataag ctctactttt aggagcagga gcaccgcgag
     2941 gattaggacc cctaaaataa gcgcctttac ctcgataggt tctttgagat ttctttttgt
     3001 agtaagctaa cttttgagct tgcgtatact tttttccaga cattgtgtag tatatgtaga
     3061 cccaaacata aaaaaataaa aaaaaataat taattgttga ctttttgcta tccagtaaaa
     3121 aaccattcat tacacttcct acttgacata tttaatataa attttcattc agtcgccgaa
     3181 aaaaataatt aattatttta attttattta atgattttat aattttacgt ataattgtaa
     3241 tgcactatga gaaagatgac gagagcctta aacatagaca ttacggctca cacccccctt
     3301 tccg
//
LOCUS       CruV-208                3306 bp    DNA     circular     24-FEB-2020
DEFINITION  Crucivirus-208 isolate 120_482_IR1_3296.
ACCESSION   CruV-208
KEYWORDS    .
SOURCE      Crucivirus-208
  ORGANISM  Crucivirus-208
            Unclassified.
REFERENCE   1  (bases 1 to 3306)
  AUTHORS   de la Higuera,I., Kasun,G.W., Torrance,E.L., Pratt,A.A.,
            Maluenda,A., Colombet,J., Bisseux,M., Ravet,V., Dayaram,A.,
            Stainton,D., Kraberger,S., Zawar-Reza,P., Goldstien,S., Briskie,J.,
            White,R., Taylor,H., Gomez,C., Ainley,D.G., Harding,J.S.,
            Fontenele,R.S., Schreck,J., Ribeiro,S.G., Oswald,S.A., Arnold,J.M.,
            Varsani,A., Enault,F. and Stedman,K.M.
  TITLE     Direct Submission
  JOURNAL   Submitted (24-FEB-2020) Biology, Portland State University, 1719 SW
            10th Ave, Portland, Oregon 97201, USA
COMMENT     Bankit Comment: ALT EMAIL:ide@pdx.edu
            Bankit Comment: TOTAL # OF SEQS:451
            Bankit Comment: GAP: est. length - alignment

            ##Assembly-Data-START##
            Assembly Method       :: MetaSPAdes v. 3.12; IDBA-UD v. 1.1.1;
                                     MEGAHIT v. 1.0.3
            Sequencing Technology :: 454; Illumina
            ##Assembly-Data-END##
FEATURES             Location/Qualifiers
     source          1..3306
                     /organism="Crucivirus-208"
                     /mol_type="genomic DNA"
                     /isolate="120_482_IR1_3296"
                     /isolation_source="Water"
                     /country="Norway"
                     /collection_date=2011
     CDS             1..474
                     /codon_start=1
                     /product="putative ORF"
                     /translation="MDETSRSLKRIEDTPDSFSFDDYNESRISDIYHQSGEGSRVSSQ
                     YISEVIRQDQSNMAHNLDAEEARRENRVNELVMDIGSEIREDLQRTLLPAGQQELLRA
                     VIEHKANLGWRDAEDQESHERESKYVKQKVYQTLLAPNKKGKYWKMNQNNFFGWQ*"
     CDS             complement(478..879)
                     /codon_start=1
                     /product="putative ORF"
                     /translation="MAKIKLVSRFFSVVRLDAHRTLRDYHMTQKKQKKKNPLTLRDYH
                     MTGCDYVSKHYIKNRFSLGLYMTDYKLFSTMDAATQYDEDDYGVPDYYPFSSQAQFEN
                     YMQLQENLQEVVYHQMVDDISTVCTDSEVEQ*"
     CDS             995..1927
                     /codon_start=1
                     /product="Putative Replication Associated Protein"
                     /translation="MYYLLHRVLSSQSPTPTPLKSETPLKSETPKMHKRRRIPFVYVM
                     ARRQGIYWLGTIPRDDFFPYGPLRDGVQYIKGQLEVGADTGYEHWQILVVFSRKVSCH
                     FVYSCFGNFHFELTRSEAAEDYVWKEETRVAGSQFELGERKMRRNVAADWENIRSLAK
                     SGQLASIPADIYVRCYNQLSRIRVDHLVPVAIERTCFVFWGKTGTGKSRRAWEEGGME
                     AYAKDPCTKWWCGYQGQKNVIIDEFRGLIGISHLLRWLDRYPVFVETKGAAQPFLGER
                     IWITSNVSPGAWYPELDSSTSDALMRRLQVIEFE*"
     CDS             1944..3275
                     /codon_start=1
                     /product="Putative CruV Capsid Protein"
                     /translation="MAKGAPVVIAGTAYAGRGRKRISRKRKPSRPLASLGVKRRRISG
                     SGDYTLSSGGRAESTPFGSVGGHIGGMIGGAYGKGVGWAGGHLLGRILGSGDYTMGQM
                     CRNNLITNGTEVPQFISGGRNNIVVHREYIQDITTSSVAGAFKLDSFSINPGLVGTFP
                     WLASLAPNYEQYVVHGMIFEFKSTSADALNSTNTALGTVIMSTDYNVNAANFTSKVQM
                     ENAEFAQSARPSQSQIHGIECAPRENQSKCYFIRNGSINSQDRRLSDFGNFQIATIGM
                     QGTSVNIGELWCSYVIEFLKPQIIAWPQEQVIRMRNATGITTVASLGTAANQVVAGPL
                     LGTFTSTGFTITNCIVGVNYRAQYAVSCAAATGSTGFVITSGATAVNLLANNTASAYN
                     SATMTTSGAWMAALTFTATSISVVLVAPTLSVLTTPTSVDAAVYQLDQTNI*"
     misc_feature    3264..3284
                     /note="stem-loop"
     misc_feature    3269..3277
                     /note="nonanucleotide"
ORIGIN
        1 atggacgaaa cgtctcgtag tttaaagcgt attgaagata ctccggattc tttttccttt
       61 gatgattata atgaatcaag gatatccgat atatatcacc agtctgggga agggtctcgg
      121 gttagttcac aatatatttc tgaagtaatt aggcaagacc aatcgaatat ggcccataat
      181 ctagatgctg aggaggctag aagagaaaat agggtgaacg aattagttat ggatattgga
      241 tctgaaatta gggaagattt gcaaagaact ttacttcctg ccggtcagca agagctcttg
      301 cgtgctgtta ttgaacataa agcaaattta ggttggcgtg atgccgagga tcaagaaagt
      361 catgaaaggg aaagtaaata tgttaaacag aaagtttatc agactctttt ggcacctaat
      421 aagaaaggta aatattggaa gatgaaccaa aataattttt ttggttggca ataaacttta
      481 ttgttctact tctgaatcag tgcagacagt tgaaatatcg tctaccattt gatgatatac
      541 cacttcttgt agattttctt gtagttgcat ataattttca aattgtgctt gtgagctgaa
      601 cgggtaataa tcgggaacac catagtcatc ttcatcatat tgagtagcag cgtccattgt
      661 agaaaacagt ttataatctg tcatatataa ccctagggaa aaacggttct ttatatagtg
      721 tttgcttacg taatcacacc ctgtcatgtg atagtcccgt agggtcaggg gattcttttt
      781 tttttgtttt ttttgtgtca tgtgatagtc ccgtagggtc ctgtgagcgt caagccgaac
      841 aacactgaaa aaacggctta ctagcttgat cttagccaat atggtgtgag aagattgtag
      901 ggaggggact ggggcccccc cgtagctatg ccgcccggct agggttgccc tgaactcgga
      961 gatgtagtaa gcacgtgggt gagaacggac tcacctgtat tacttactac atcgagttct
     1021 tagttctcag agtccgactc cgactccgct aaagtccgaa actccgctaa agtccgaaac
     1081 tccgaaaatg cataaaaggc gccggatccc ttttgtgtat gtcatggcta gacgacaggg
     1141 aatttattgg ttgggaacca tccctagaga cgactttttt ccctatggac cccttcgaga
     1201 tggagtgcag tacatcaaag gacagcttga agtcggggct gacaccggat acgaacattg
     1261 gcaaatcttg gtggtcttca gccgaaaagt atcttgccac tttgtctact cctgtttcgg
     1321 aaacttccac tttgaactta caagatctga agccgccgaa gattatgttt ggaaagaaga
     1381 aactcgggtt gctggatccc agtttgagct tggagaaaga aagatgcgaa gaaatgttgc
     1441 tgcagactgg gaaaatatcc gaagtttggc caaatcagga cagctggcgt ctattccagc
     1501 ggatatatac gttcgatgtt ataaccaatt gtcacgaatc cgcgtcgatc atcttgtccc
     1561 agttgctatt gagcgaacgt gttttgtttt ctggggtaaa actggaacgg gaaagtctcg
     1621 ccgtgcatgg gaggaaggag gaatggaagc ttacgctaag gatccgtgca ccaagtggtg
     1681 gtgtggttat caaggtcaga aaaacgttat catcgatgaa tttcgagggc ttattggaat
     1741 ctcccatttg cttagatggt tggatcgtta tccggtcttt gtggaaacga aaggggcagc
     1801 acaaccattc ctcggtgaac gaatttggat cacctcaaat gtatcccctg gagcttggta
     1861 ccctgagttg gactcctcaa caagtgatgc tttgatgcgg agattacaag ttatagaatt
     1921 tgaataaaat tctttactta tttatggcaa agggtgcgcc agtcgttatt gcaggaacag
     1981 cttatgcagg tcgtggaaga aaacgcatca gtcgaaaacg gaagccaagt cgtccacttg
     2041 cgtcgctcgg tgttaaacgc cggcgcatta gtggcagcgg tgattatact cttagtagcg
     2101 gggggagggc tgaaagcact ccttttggct ctgtaggtgg ccatattggt ggaatgattg
     2161 gtggagctta tggtaaagga gtaggctggg ccggaggtca tttgcttggt agaatattag
     2221 gaagtggtga ttatactatg ggtcagatgt gtcgtaacaa tttgattaca aatggaacgg
     2281 aagttcctca gtttatatcc ggtggacgca ataatattgt tgttcatcgt gagtatattc
     2341 aggatattac tacatccagt gttgctggag cgtttaaatt ggatagtttt agtatcaatc
     2401 caggtttggt tggcaccttt ccttggttgg catctttggc tccaaattat gaacagtatg
     2461 ttgttcatgg aatgatattt gagtttaaaa gtacttcagc agacgctttg aacagtacca
     2521 atactgcgtt gggtactgtg attatgtcca ctgattataa tgtgaacgct gctaatttta
     2581 catcgaaggt ccaaatggaa aatgctgagt ttgctcagtc tgcgcgtcca agtcaatcgc
     2641 agattcacgg tattgaatgt gctcctagag agaatcagtc taagtgttat tttattcgca
     2701 atggtagtat taacagccaa gaccgacgtt tgtccgattt tggcaatttt cagattgcaa
     2761 caattggtat gcaaggaact agtgttaaca ttggtgaatt atggtgttcg tacgttattg
     2821 aatttttgaa gcctcaaatt attgcttggc cacaggagca ggttattcgt atgagaaatg
     2881 ctactggtat tactactgtt gcttcgttgg gaactgctgc caaccaagtt gtagctggtc
     2941 ctctacttgg gacttttact tctaccgggt tcactataac caattgcata gttggggtta
     3001 attaccgtgc ccaatatgct gttagttgtg ctgcagcaac tggttcaaca ggtttcgtta
     3061 ttactagtgg tgctactgca gttaatttgt tggctaacaa tacagctagt gcttataatt
     3121 ctgctactat gactacgtct ggtgcatgga tggctgcttt aacgtttaca gctacctcta
     3181 tttctgttgt tttagtagct cctactttga gtgttttgac tactccaact tctgttgacg
     3241 cagcggttta ccagttagat caaactaata tttaaatggt tagtgaataa aaattcttta
     3301 cttaat
//
LOCUS       CruV-209                3309 bp    DNA     circular     24-FEB-2020
DEFINITION  Crucivirus-209 isolate 120_158_SvL2_3298.
ACCESSION   CruV-209
KEYWORDS    .
SOURCE      Crucivirus-209
  ORGANISM  Crucivirus-209
            Unclassified.
REFERENCE   1  (bases 1 to 3309)
  AUTHORS   de la Higuera,I., Kasun,G.W., Torrance,E.L., Pratt,A.A.,
            Maluenda,A., Colombet,J., Bisseux,M., Ravet,V., Dayaram,A.,
            Stainton,D., Kraberger,S., Zawar-Reza,P., Goldstien,S., Briskie,J.,
            White,R., Taylor,H., Gomez,C., Ainley,D.G., Harding,J.S.,
            Fontenele,R.S., Schreck,J., Ribeiro,S.G., Oswald,S.A., Arnold,J.M.,
            Varsani,A., Enault,F. and Stedman,K.M.
  TITLE     Direct Submission
  JOURNAL   Submitted (24-FEB-2020) Biology, Portland State University, 1719 SW
            10th Ave, Portland, Oregon 97201, USA
COMMENT     Bankit Comment: ALT EMAIL:ide@pdx.edu
            Bankit Comment: TOTAL # OF SEQS:451
            Bankit Comment: GAP: est. length - alignment

            ##Assembly-Data-START##
            Assembly Method       :: MetaSPAdes v. 3.12; IDBA-UD v. 1.1.1;
                                     MEGAHIT v. 1.0.3
            Sequencing Technology :: 454; Illumina
            ##Assembly-Data-END##
FEATURES             Location/Qualifiers
     source          1..3309
                     /organism="Crucivirus-209"
                     /mol_type="genomic DNA"
                     /isolate="120_158_SvL2_3298"
                     /isolation_source="Water"
                     /country="Norway"
                     /collection_date=2012
     CDS             1..1284
                     /codon_start=1
                     /product="Putative Replication Associated Protein"
                     /translation="MLCAEMILAKSEDEALTETIEPMTMMDGRLTLDLDAKLGHSPTY
                     WADDKKFRLQATCVMFTYATHIDKKMLIKFLSGKLSRGVEDSLVAHELGKKKKYLHTH
                     AIIKLDRRCDTKKPDTFDWECEDGTTIHPHILPFQPNLWKQKVLYLCKEDPENKERGG
                     TTSLTLAQLTWAKKDQAGALSLATSFQEITGILALYALKPAVAKNQKCILKRWQNRLF
                     DRIQYNEEYEMGGFTGRNVDDSDDSGSFEFISGGKRKIIWVWDIKGCCGKTIFAQELI
                     RRDPVRYTGLTSIPQESHLATIIEGSISRGWDGSVVLICLSRQAEDYKIYKGLEDIRD
                     GHMTSTKYHGKYLSWTSQHVVVLANWLPNFSALTWDKWELYTINKKTKQLETMTLDEG
                     IKARNEYIAAKEEENSLNEAYMRHVAIDIYKHKGF*"
     CDS             complement(1614..3008)
                     /codon_start=1
                     /product="Putative CruV Capsid Protein"
                     /translation="MSRYSDAEKAAYWRSKALAKTPSGRTKKYSSGGKKYRPKESYEG
                     KLEYAKYRDDSRRKGKGYNAAPGIISSIGSSLGGVGGGMLGGPAGALTGNFLGGKLGH
                     LIENITGFGDYKVHSNTIMTGGMSPLQVVNSINRDNGVILRHREYLGDIPATVVFTVQ
                     SFLINPGLSSTFPWLSQIANSYEQYRIRGMLFEFNSTSSDALLSAATSTALGSVVMQT
                     DYDVADLPPTSKRQMLNSEWSCSSKPSCTFIHPIECKKNQSTPSLLYTRGAIAVPAGF
                     DQRLYDFARFNIATEGMQAAGGVLGELWITYELELFKQQFSFVGLTDHFRLGAVTNVA
                     NLGTAAQSSVAAGGTIGGAINVAGDTYSFPPQLASGKYLVTYWVLGGAQAVTIPTVTY
                     INCTGLNYFANDTVSIVGSPTPPLGILTTVTVEWLINLTQQNAGFKVSAGLLPQPFTS
                     GDLVVTRIADSIVA*"
ORIGIN
        1 atgctatgtg ctgagatgat tctagcgaaa agtgaggatg aagcactcac agaaacaata
       61 gaacccatga cgatgatgga tggaaggtta acactagacc tagacgcaaa gctaggtcac
      121 tcaccaacat actgggcaga cgacaagaag ttcagactgc aagcaacatg tgtgatgttc
      181 acatacgcga cacacataga caagaagatg ctaatcaagt tcctatctgg aaaactaagc
      241 agaggtgtgg aagactctct tgtagcacac gagctaggca agaagaagaa ataccttcac
      301 acacacgcaa taatcaagtt ggacagaagg tgtgatacaa agaaaccaga cacattcgat
      361 tgggagtgtg aggacggtac aacgatacac ccacacatcc tgccgttcca acccaactta
      421 tggaagcaaa aagttttata cctgtgcaag gaggacccag agaacaaaga gagaggtgga
      481 acaacatctc taactctagc ccaactaaca tgggcaaaga aagaccaagc aggagcgcta
      541 tcgttagcaa caagcttcca agagataact ggaatacttg cactatacgc cctaaagcca
      601 gcagtagcca agaatcagaa atgtatactg aaaagatggc agaacaggct cttcgacaga
      661 atccaataca atgaagaata tgaaatgggt ggattcacag gaaggaacgt agatgactcg
      721 gatgactcag gatcattcga attcatctca ggcggcaagc gcaagataat atgggtgtgg
      781 gacataaaag gatgctgtgg caagacaata ttcgcacaag aactcataag aagagaccca
      841 gtaagataca caggtctaac atccatacca caagagagcc acctagcaac gataatagaa
      901 ggatcaatat caagaggatg ggatggttca gtagtactaa tctgcttatc aagacaggca
      961 gaagactaca agatctacaa aggacttgaa gatataagag acggacacat gacatcaacc
     1021 aagtaccacg gtaagtacct atcatggaca tcacaacacg tagtagtgct agccaactgg
     1081 ttacccaact tctcagcact aacatgggac aaatgggaac tatacaccat aaacaagaag
     1141 accaagcaac tcgaaacgat gaccttagac gaaggcataa aagcacgtaa cgaatacata
     1201 gcggctaaag aagaggaaaa ctcacttaat gaagcttaca tgagacacgt agccatagac
     1261 atatacaaac ataaaggatt ctaagacatt cactgtaaag tgggtgcggg gacaagcccc
     1321 tccccttcca taaacaatta tgtacacatg ataaatggag gacaagaagc aacccaacaa
     1381 ggaacaggta gccaagatcg tcagcattga caagtacatt gcaatggaac gtatccaaga
     1441 agttctagaa gaactaatga acgaactgga catcactgaa tgtgaactag taggactact
     1501 tcactaccta tacagtgacg gatgcagtaa acacaaagac gatgatgtac ctccaccggt
     1561 taatcaaccg cgctgattaa caacttagag tcgatcgact ctaatataca agattaagct
     1621 acgatgctat ccgctattct agtgacaaca agatcaccag atgtgaaagg ctgaggaagt
     1681 aacccagccg ataccttgaa cccagcgttc tgctgtgtaa ggttaatcaa ccactcaacg
     1741 gtaactgttg taagtatacc aagtggtggt gttggagacc ctactatgct aacagtatcg
     1801 ttagcaaagt agttaagacc cgtacaatta atgtacgtaa ctgttggaat ggtgactgct
     1861 tgagcaccac ccaagaccca gtaagtaaca agatacttac cagatgccaa ctgtgggggg
     1921 aaagaataag tatccccagc cacattaatg gcaccaccaa tagtacctcc agcggcaaca
     1981 gaagactgag ccgcggtacc taaattggcg acgttggtaa cagctcccaa cctgaagtgg
     2041 tcggttaatc caacaaaact gaactgctgc ttaaagagtt ctaactcata ggtgatccat
     2101 agctcaccaa gaactccacc ggcagcttgc atgccctcgg ttgcaatgtt gaaccgggca
     2161 aaatcataca ggcgttgatc gaagcctgct ggaactgcaa tagcacccct tgtatagagc
     2221 aaggacgggg tactttgatt cttcttacac tcaattggat gaatgaaggt acaagatggc
     2281 ttacttgaac atgaccactc actgtttaac atctgacgct tagatgtggg tggtaagtca
     2341 gcaacgtcgt aatcagtctg cattacgaca ctacccaagg cggtacttgt tgctgcggag
     2401 agtaaggcat cagaggacgt cgagttaaac tcgaacaaca tacccctgat cctatactgc
     2461 tcataagagt tcgctatctg agatagccac gggaacgtgg aactcaagcc aggattgatg
     2521 agaaagctct gtaccgtgaa cacaacggtc gctggaatat cacctagata ctccctatga
     2581 cgaaggataa ctccattgtc tctattaata gaattaacaa cctggagagg cgacattcca
     2641 ccagtcataa tggtattgga atgcaccttg taatctccga accccgtaat gttctcgatt
     2701 aagtggccta gtttgccacc aaggaagttg cctgttagtg ctcccgctgg tccgcctaac
     2761 atgccacctc ccactcctcc taggctagaa cctattgagg agatgatacc gggcgcggca
     2821 ttataaccct tgcccttacg acgggaatca tcacgatact tagcgtattc tagcttcccc
     2881 tcgtacgact cttttggacg gtacttctta ccgccactag agtacttctt cgtacgtccg
     2941 ctgggggtct tggctaaagc cttcgacctc caatacgcgg ccttctcagc gtcactatac
     3001 ctactcatgt ttagtgacgg gtatatttac tctaaataca ttggtggggg aatgtatcgt
     3061 cttcccccta taccccctta actccctcta atttccggcc taatattata taccctgact
     3121 cagggtagcg gccggaatta ggggtgggga ctcgctacgc tcgccccacc ccacaaataa
     3181 aaaaatatcc cactggggca tatgctccag gacacttaaa aaaatacccc actgggacac
     3241 cacaggatac ttaagaaaag tactccagta taaaataaga gaacactcac tcaactcact
     3301 cactcaaat
//
LOCUS       CruV-210                3310 bp    DNA     circular     24-FEB-2020
DEFINITION  Crucivirus-210 isolate 001650_virusbaton.
ACCESSION   CruV-210
KEYWORDS    .
SOURCE      Crucivirus-210
  ORGANISM  Crucivirus-210
            Unclassified.
REFERENCE   1  (bases 1 to 3310)
  AUTHORS   de la Higuera,I., Kasun,G.W., Torrance,E.L., Pratt,A.A.,
            Maluenda,A., Colombet,J., Bisseux,M., Ravet,V., Dayaram,A.,
            Stainton,D., Kraberger,S., Zawar-Reza,P., Goldstien,S., Briskie,J.,
            White,R., Taylor,H., Gomez,C., Ainley,D.G., Harding,J.S.,
            Fontenele,R.S., Schreck,J., Ribeiro,S.G., Oswald,S.A., Arnold,J.M.,
            Varsani,A., Enault,F. and Stedman,K.M.
  TITLE     Direct Submission
  JOURNAL   Submitted (24-FEB-2020) Biology, Portland State University, 1719 SW
            10th Ave, Portland, Oregon 97201, USA
COMMENT     Bankit Comment: ALT EMAIL:ide@pdx.edu
            Bankit Comment: TOTAL # OF SEQS:451
            Bankit Comment: GAP: est. length - alignment

            ##Assembly-Data-START##
            Assembly Method       :: MetaSPAdes v. 3.12; IDBA-UD v. 1.1.1;
                                     MEGAHIT v. 1.0.3
            Sequencing Technology :: 454; Illumina
            ##Assembly-Data-END##
FEATURES             Location/Qualifiers
     source          1..3310
                     /organism="Crucivirus-210"
                     /mol_type="genomic DNA"
                     /isolate="001650_virusbaton"
                     /isolation_source="Water"
                     /country="France"
                     /collection_date=2013
     CDS             1..1452
                     /codon_start=1
                     /product="Putative Replication Associated Protein"
                     /translation="MMWIKWYKRKDLSRKPQTKMSASPKFRFQNQRALLTYRTHLNKE
                     EYCHHIARITRIEPAFIRLAHETGDEECQYLHTHVVVDFGKNFQTTNERFFDYTLTDG
                     SVIHPHIKVLKNRTALKDASKYIAKEDPENSDLANTTNVIDVIQGANSLNEAFQLTIG
                     EGAYKISSAMAIKTVFDAKKPDLLRRPPYSPTLEWQVDFINEMMHEPDFRSIIWYYDT
                     IGNNHKTQLAKYMMRMDPIKWYVVKDMGTSRDSSTIIANAIVAGWQGYGIIIDLPRTA
                     ENHTRMYTYIEEIKDGFITAQKYSGRTIIFDNPHVVVMANWLPLTECLSLDRWDIRHL
                     NSGVCTSRTVAGVQVSSGTHDDPHRLELKETEEQPTNDYVVIPTVEPPNSVIRPWTPV
                     IHIPTPKIEFSNKSMNSPNHASGHSDIASNISGPSILGTHTRSLVPETNINTSLSPVK
                     SPPKTALLTSPKNTKYPNILPVRPSLQLSKKKK*"
     CDS             complement(1538..2881)
                     /codon_start=1
                     /product="Putative CruV Capsid Protein"
                     /translation="MPAQRKSQQQKKPQKKSVPYYRSLAPKRSYRSGKGAYYISGGAY
                     ARGKIPYLGNVGAAMNAGYSNGKVSAPTLSGLGAYAISNIKHNVLIKPDVPQMVNARY
                     AEGGTIVRHREYLGPVVTSATAGSFKIESYPLNPAQDVTFPWLSSIASNYEEYKPNGL
                     LFEFRSTASDAIASSTNLALGQIMMCTQYDPTDPEFASDVELLNYSWAQSGKVSDCVQ
                     HYVECDPRQSPLAHLYTRQGATTSDSDLRFSDFGRFSIASSGLQGTSVQVGQLWVTYE
                     FIMYKPKLGSAGADPGGWYHYSNDVGISATQPLGTISVGVVDAENNIAVNPVDATNDV
                     NLFLPASFKPTSYMVKIAYIGNSTALVTPPTFGNTTDPSIQFVQHSDNNNSAQIFTPN
                     GYPATSEEATCTTWISVLGDGASHALKLSSGVQPTTAKVDLYISQIPYLDPHLYG*"
ORIGIN
        1 ttgatgtgga taaaatggta taaaaggaag gatttgagta gaaaaccaca aaccaaaatg
       61 tcagcttcac cgaaattccg cttccagaac caacgtgccc tacttactta taggacacac
      121 ctgaataaag aggagtactg tcaccacatt gcacgtatca cacgaattga acctgctttc
      181 atcagattag cacacgaaac tggtgacgaa gaatgtcaat acctacacac tcacgtggta
      241 gtagactttg gcaaaaactt tcagactacc aatgaaagat tcttcgacta cacattgacg
      301 gatggatctg tgattcaccc tcatattaag gttcttaaaa atcgcactgc tcttaaagac
      361 gcttcgaaat atatagctaa agaggaccct gaaaatagtg atcttgcgaa tacaacgaat
      421 gttatcgatg taattcaggg agctaattct cttaatgaag ctttccagct tactatcggg
      481 gaaggagcgt acaagatatc tagcgctatg gctattaaaa ctgtttttga tgccaaaaaa
      541 ccagaccttc taagaagacc gccatactct cctacacttg aatggcaagt ggatttcatt
      601 aatgaaatga tgcacgaacc cgattttaga agtatcatct ggtattatga taccatcggt
      661 aataatcata agacccaatt agctaaatat atgatgagga tggaccctat taaatggtac
      721 gtagtaaaag atatgggaac atcacgtgat agctccacta tcatcgccaa tgctatcgtt
      781 gctggatggc aagggtacgg tattatcatt gatctaccta gaactgctga gaatcataca
      841 agaatgtata cgtacatcga agaaataaag gatggattca ttactgctca gaaatactct
      901 ggaagaacca tcatcttcga taatcctcat gttgttgtaa tggctaactg gttaccattg
      961 acagaatgcc tgtcgttaga tagatgggat attcggcacc ttaactcagg tgtttgtact
     1021 tcaagaaccg ttgctggagt acaagtatcc agtggcactc acgacgatcc tcatcgcctt
     1081 gaattaaaag aaaccgaaga acaaccaact aacgactatg tagtcattcc aactgttgaa
     1141 cccccaaaca gcgtgattcg accatggaca ccagtaattc acattcctac accgaaaatc
     1201 gagttttcta ataagtccat gaactcccca aatcacgcct caggtcactc cgatatagca
     1261 agcaatatct ccggaccttc gattctcggg acccacacca ggtccctcgt accggagact
     1321 aacattaata cttcgttgtc tccggtaaaa tccccaccca aaacagctct tctaacgagc
     1381 cccaaaaata ccaaatatcc aaatatccta ccagttagac cttctcttca actatcaaag
     1441 aagaaaaagt aataactaac taactaacta actaactaaa taatatatat ttgcaacgtt
     1501 actaactaaa taatatatgt aattaacata tatgatttta accatagaga tgaggatcaa
     1561 gatatggaat ctgagaaata taaagatcaa ctttagctgt tgtaggttga acacccgagc
     1621 taagtttcaa agcatgtgat gcaccatcac caagaacaga gatccacgtt gtacatgtag
     1681 cctcttctga agttgcagga taaccatttg gagtaaaaat ttgggcagaa ttattattat
     1741 cagaatgctg aacaaactga attgaaggat cagtagtatt cccaaaagtt ggtggtgtaa
     1801 caagagcagt agaattgccg atatatgcaa ttttaaccat atacgacgta ggtttaaagg
     1861 atgctggtag aaaaagatta acatcgtttg tagcatcaac tggattaact gctatattat
     1921 tctcggcatc tactactcca acagaaatag taccaagagg ttgagttgca gatataccaa
     1981 catcattact ataatgatac caacctccag gatcagcacc cgcagagcca agctttggct
     2041 tatacattat gaactcgtag gtaacccata gctgacctac ttgcacacta gtaccttgaa
     2101 gtccactaga tgcaattgaa aatcgtccaa aatctgagaa gcggagatct gaatcgctag
     2161 ttgtagcacc ttgacgtgta tacaaatgag ctaaaggaga ttgtctggga tcacattcaa
     2221 cataatgttg aacacaatcg gaaaccttac ccgattgagc ccatgaataa ttaagtaatt
     2281 caacatctga agcgaattct ggatcagtag gatcatactg agtacacatc ataatttgac
     2341 caagagctaa attagtagac gatgcaatag catctgatgc agtagatcta aattcaaaca
     2401 aaagaccatt gggtttatac tcttcgtaat tggaagcaat agaactcagc catggaaaag
     2461 taacatcctg tgccggattg agtggataag actcaatctt aaaagatcca gcagttgctg
     2521 aagtaacaac cggtcctaaa tactcacgat gtctaacaat tgtccctcct tcagcataac
     2581 gagcgttaac catttgaggt acatcaggtt tgattaaaac attgtgcttg atattagata
     2641 tagcatatgc acctaaacct gacaaggtag gtgcggaaac tttaccatta gaatatcctg
     2701 cattcattgc agcgcctaca ttaccaagat atggtatttt ccctcgagcg tacgctcctc
     2761 ctgaaatata gtaggctcct tttccagacc tataactcct tttgggagca agtgatcgat
     2821 aataaggaac tgatttcttc tggggtttct tttgttgctg tgacttacgt tgtgcgggca
     2881 ttttagatag atattattgc tttagaccat tttaaaaata aataaaataa taaaggggtc
     2941 taaacatatg agttgccgtt atagttagat cgggagcgat acgttccgct ccgctccact
     3001 gctctgcgca ggcctgtggt gcccatccgg gcactatgtt agattgatca gattgggggt
     3061 caaaaagttg ctttagacca ttttaacgat agataaaata ataaaggggt ctaaacatat
     3121 gagttgccga tatagtatct gattgtattg attgattaga tatgggggtc aaaaagttgc
     3181 tttagaccat tttaacgata gataaaataa taaaggggtc taaacatatg agttgccgat
     3241 atagtatctg attgtattga ttgattagat atgggggtca aaaagttgct ttagaccatt
     3301 ttaacgataa
//
LOCUS       CruV-211                3310 bp    DNA     circular     24-FEB-2020
DEFINITION  Crucivirus-211 isolate SR1_25609.
ACCESSION   CruV-211
KEYWORDS    .
SOURCE      Crucivirus-211
  ORGANISM  Crucivirus-211
            Unclassified.
REFERENCE   1  (bases 1 to 3310)
  AUTHORS   de la Higuera,I., Kasun,G.W., Torrance,E.L., Pratt,A.A.,
            Maluenda,A., Colombet,J., Bisseux,M., Ravet,V., Dayaram,A.,
            Stainton,D., Kraberger,S., Zawar-Reza,P., Goldstien,S., Briskie,J.,
            White,R., Taylor,H., Gomez,C., Ainley,D.G., Harding,J.S.,
            Fontenele,R.S., Schreck,J., Ribeiro,S.G., Oswald,S.A., Arnold,J.M.,
            Varsani,A., Enault,F. and Stedman,K.M.
  TITLE     Direct Submission
  JOURNAL   Submitted (24-FEB-2020) Biology, Portland State University, 1719 SW
            10th Ave, Portland, Oregon 97201, USA
COMMENT     Bankit Comment: ALT EMAIL:ide@pdx.edu
            Bankit Comment: TOTAL # OF SEQS:451
            Bankit Comment: GAP: est. length - alignment

            ##Assembly-Data-START##
            Assembly Method       :: MetaSPAdes v. 3.12; IDBA-UD v. 1.1.1;
                                     MEGAHIT v. 1.0.3
            Sequencing Technology :: 454; Illumina
            ##Assembly-Data-END##
FEATURES             Location/Qualifiers
     source          1..3310
                     /organism="Crucivirus-211"
                     /mol_type="genomic DNA"
                     /isolate="SR1_25609"
                     /isolation_source="Water"
                     /country="New Zealand"
                     /collection_date=2012
     CDS             1..897
                     /codon_start=1
                     /product="Putative Replication Associated Protein"
                     /translation="MSRSRNWCYTINNPTNEEIETCDVIECSYNVFGFEVGNEGTPHL
                     QGYIEFKDAKTLRSAKKSLGGRAHLEQRRGTATEASDYCKKDNLFIENGSISNQGKRN
                     DLDTLAELVKTKGIKGVIEEAPGMFIKFGKNIERLSEFYMKPRTTAPKVIWLWGKAGV
                     GKTKLAHENIDNEDIYIWNQTKWWTGYRQQSRVIIDDYSFDNSDHHYRYLLQLIDRYS
                     IQVETKGGMVYFNSPEIYITCEFPPTEFWQVGNKLDQITRRISEIVEVKDVICKESND
                     IITLVPPELEPSDNIDEDWLDI*"
     misc_feature    435..461
                     /note="stem-loop"
     misc_feature    447..455
                     /note="nonanucleotide"
     CDS             complement(1865..3208)
                     /codon_start=1
                     /product="Putative CruV Capsid Protein"
                     /translation="MPRPAAKAPKGTKKDTKASEYYRKQKKSKYTRKTPRAPLAAASS
                     TESFGESAGKLLGGLAQKAIKALTGFGEYTTPGFGIKQNVLIEHGDPPTVQNAGKEFI
                     VRHREYLGDIYSGVAGAVGAPAASPFTIQAFPINPGLQLSFPWLANLAARFEEYELQG
                     MLYEYKSMYSDAAVQTGGSLGTVIMATSYNAAKPNFPSKIEMENYEFAQSAKPSVSMI
                     HPIECQPGQNVFNSQYIRLGPLLDGEDIKTYDAGKFQIASQGIPVSGSALSLGELWST
                     YQICFRKPKVTNYIDSGYARLSQTAAVQPAGGSPFAPLISWSTITDNIGIKLVNERTF
                     TLPLFNNTRTYQINLICGDPNNLNTQAAAVGYFPTTATNVTNGVISIMPQIPSPAFTQ
                     ATGSLNISSSAFLLTITTNPISPGQTLCTVQLPNGCGATLSYIKNLIINAVPTSS*"
ORIGIN
        1 atgtctagat ctagaaattg gtgttacact atcaataatc caacaaacga agaaattgaa
       61 acatgcgatg ttatcgaatg ttcgtacaat gtattcggtt ttgaagttgg taacgagggt
      121 accccccacc ttcaaggata tatagagttt aaggacgcaa aaactcttag gtcagctaaa
      181 aaatcgctag gaggccgagc tcatcttgaa caacgaagag gaacagctac tgaagcatct
      241 gattattgca aaaaagataa tctttttatt gaaaatggtt ccatttccaa tcaaggtaag
      301 cgaaatgacc ttgataccct agctgaacta gttaagacca aaggtattaa aggtgtcatc
      361 gaagaagcac ctggtatgtt cataaaattc ggcaaaaaca tcgaacgttt atccgaattt
      421 tatatgaagc ccagaaccac agcgcctaaa gttatatggt tatggggtaa ggcgggtgtt
      481 ggtaagacca aactcgctca tgaaaatata gataacgaag atatatatat ttggaatcaa
      541 actaaatggt ggacagggta ccgtcaacaa tctagagtca taatcgatga ttattctttt
      601 gataatagtg atcatcacta taggtattta ttgcaactta tagatagata ctctatccaa
      661 gttgaaacaa aaggtggtat ggtctatttt aatagtcctg aaatttacat tacatgtgaa
      721 tttccgccca ccgaattttg gcaagttgga aacaaacttg atcaaattac tcgacgaatt
      781 tctgaaattg tcgaggttaa agatgtaata tgtaaagaat ctaatgatat tatcactctc
      841 gtcccccccg aactcgagcc cagtgataat attgatgagg attggcttga catttgaaat
      901 atttaactat cttatgtgga aaactataaa aaacaaatga taataaatga tctttttttc
      961 tgattacttt ggtatcattt ttggtacaat gcgtggacaa agggtcggtc cccccctccg
     1021 ctgagtctag ccagtaaacc accccgcagg ggggttgttt tactgtcgtc acctcccaca
     1081 gggaaccccc tcggcgtaaa cgcctacagc ccgtaggtct gaaaggggac agctttgcac
     1141 atcttgcacg catggcaaaa aaattacaca aaagttttta cacagaagtg gcaggtaata
     1201 ctaggaaaat ttgtattttc ctttctgcca cctacaaggg tgtaaaaaat aattaagtaa
     1261 ttatagcaca taaatgcagc gagtcaccct ataagaaaat ccttaatcat tgatctggac
     1321 tagtacaatg gacgccttcg caggcgccca tccatctacc gctgatgaag gcctaaaaag
     1381 tgttgtattt tttcttatgt gcaacacgat gaatttattc agtcgtactt tttgtatctc
     1441 gcatgagagt tagttcgata aagtcgagtg cttcttgtcg agtaagttgt ctttctagac
     1501 cgttatcttt gacggtaacg ttttcaatgc accaagttat tgatgcttcg aagcgtttga
     1561 ttgctaaaga tattcttaaa ttttgacgta tgtttggtcg acggtaacga ccttcaggcg
     1621 tctttactaa aatttgattt cgtttgtttg attgcatgat tgacataata taatatcgaa
     1681 aaaaaataaa ataggcaatt aattaccaga tttggtacca tgttttttgt gaagaatttt
     1741 cgtgagaagt atcaggaatt tcttgtaaat ataattcatt acatgaagta ttttttggtg
     1801 atagagtgtt ggtgtagtta tcaattatat tatcaatgat gtttttcttg acttcgtctt
     1861 cgattcatga tgatgtaggt accgcgttga tgattaaatt tttaatgtat gacaatgttg
     1921 ctccgcaacc attaggtaac tgaacggtac aaagtgtttg tcctggtgat atagggttag
     1981 tggtaatggt caataagaac gctgaactag aaatattgag tgacccagtt gcttgggtaa
     2041 atgcagggga gggtatttgc ggcataatac taatgacacc attagtgaca ttagtggctg
     2101 tggtagggaa atatcctacc gcagcggctt gagtattaag attatttggg tctccgcata
     2161 tcaaatttat ttgataggtg cgagtattat tgaaaagtgg tagggtaaat gttcgctcat
     2221 tgaccaattt gataccgata ttgtcagtaa ttgttgacca actaatgagt ggtgcgaagg
     2281 gtgaacctcc ggcaggttga actgcggctg tttgggacag tctcgcgtat ccagaatcaa
     2341 tataatttgt taccttgggt ttacggaagc aaatttgata agtagaccat agttcaccta
     2401 gtgataaagc actacctgac acaggtatac cttgagatgc aatttggaat tttccagcat
     2461 catatgtttt gatatcttcg ccatcaagga ggggtcctag tctaatatat tggctattaa
     2521 aaacattttg tccaggttga cattcgatag ggtggatcat gctcaccgat ggcttcgctg
     2581 attgggcaaa ttcataattt tccatctcaa tttttgatgg aaagttaggt ttcgcagcgt
     2641 tataacttgt ggccatgata actgtaccta gtgaacctcc ggtttgcacc gcggcatctg
     2701 agtacatact tttatactca tataacatac cttgtaactc atattcttcg aatctggcgg
     2761 ctaaattagc gagccatggg aacgataatt ggagtccagg attgattgga aatgcttgaa
     2821 tagtaaaggg tgatgcggca ggggcaccta ctgctccagc tacgccggaa taaatatcac
     2881 ctaaatattc acgatgtcgg acaataaatt ctttgccggc attttgaaca gtgggcgggt
     2941 cgccatgttc aattagtaca ttttgcttga taccaaaacc gggtgttgtg tattctccga
     3001 atccagtgag ggccttgatt gctttttgag ccaatccacc tagcaacttg cctgcacttt
     3061 ctccgaaact ttcggtagat gaggccgctg cgagtggtgc ccgaggtgtc ttacgagtat
     3121 atttagattt cttttgcttt cgatagtact cggatgcttt cgtatccttc ttagttcctt
     3181 tgggagcttt agctgctggt cgaggcataa tattattagg tagctaacaa tacttagggg
     3241 aaatataaat tttaattagt taataattaa attaagtaaa acttacgtgt aagttatgtt
     3301 ggtgtaaaaa
//
LOCUS       CruV-212                3311 bp    DNA     circular     24-FEB-2020
DEFINITION  Crucivirus-212 isolate SR1_25204.
ACCESSION   CruV-212
KEYWORDS    .
SOURCE      Crucivirus-212
  ORGANISM  Crucivirus-212
            Unclassified.
REFERENCE   1  (bases 1 to 3311)
  AUTHORS   de la Higuera,I., Kasun,G.W., Torrance,E.L., Pratt,A.A.,
            Maluenda,A., Colombet,J., Bisseux,M., Ravet,V., Dayaram,A.,
            Stainton,D., Kraberger,S., Zawar-Reza,P., Goldstien,S., Briskie,J.,
            White,R., Taylor,H., Gomez,C., Ainley,D.G., Harding,J.S.,
            Fontenele,R.S., Schreck,J., Ribeiro,S.G., Oswald,S.A., Arnold,J.M.,
            Varsani,A., Enault,F. and Stedman,K.M.
  TITLE     Direct Submission
  JOURNAL   Submitted (24-FEB-2020) Biology, Portland State University, 1719 SW
            10th Ave, Portland, Oregon 97201, USA
COMMENT     Bankit Comment: ALT EMAIL:ide@pdx.edu
            Bankit Comment: TOTAL # OF SEQS:451
            Bankit Comment: GAP: est. length - alignment

            ##Assembly-Data-START##
            Assembly Method       :: MetaSPAdes v. 3.12; IDBA-UD v. 1.1.1;
                                     MEGAHIT v. 1.0.3
            Sequencing Technology :: 454; Illumina
            ##Assembly-Data-END##
FEATURES             Location/Qualifiers
     source          1..3311
                     /organism="Crucivirus-212"
                     /mol_type="genomic DNA"
                     /isolate="SR1_25204"
                     /isolation_source="Water"
                     /country="New Zealand"
                     /collection_date=2012
     misc_feature    join(3307..3311,1..15)
                     /note="stem-loop"
     misc_feature    1..9
                     /note="nonanucleotide"
     CDS             complement(122..472)
                     /codon_start=1
                     /product="putative ORF"
                     /translation="MSDKKTKDDYLFEKLMKRYIERENKYNRLKEEIKPILKKEPRYN
                     SYSRPYDNPKSYKVKPLPSYNNYSAEKEYFRTPKPRSYKTRKVDNNAPLDNNNETMLF
                     VDEGMGIRNIGEIV*"
     CDS             complement(523..1842)
                     /codon_start=1
                     /product="Putative CruV Capsid Protein"
                     /translation="MSFLRKRSSRKASRKASRKVARILGVRRLKGRGDYIPESYKNTA
                     KNYGHKAVDYVVDTASSGVDKLIGKLIGSGDYASNQVKANTLMNIAQQVPEMHSNKAS
                     NTARICHREYLGDVYTHPTTPGAFNLQSYQLNPGIKSSFPWLANIANTSYQKWRPLGI
                     VYEFRSMSADALNSTNTALGSVIMACNYSSVDPLFTTKQQMENSQWGMSSKPSQSMLF
                     GIECAKNQTPLGEMYVNNGSENTIADPRFDNLGVFQIATAGGQKENVNLGELWVSYDI
                     ELIAPVQNKALTNAGMAHYRIDVGTVTPTSLFGSASRIKVFDNYGITLNANTLTIPRS
                     KLDPKGLYCLYYQLRGATTTNVGAIIVVYQGSAKGVDLLQSGGSNQEPYPRGPANNAE
                     MGITAFFSFDPTVAGDVILVFANCIAVPASLVVCDLLVQEVNSAIVA*"
     CDS             complement(2177..3226)
                     /codon_start=1
                     /product="Putative Replication Associated Protein"
                     /translation="MKKIVPQNTSIVPVNYGNTIIDTIEKSSIVKTKKSKNIVLDSQT
                     PIKSKESKQPKKRKSWFFTFNNYTIDDINTLKEKFNSVCEKYIFQEEKGKGTFNASWN
                     EDGNCIKTDEKEGTTHLQGVIFLHDDMRPTELNLSDRIHWEGTRNKKASINYCSKLES
                     SNGKTYSKNIMVNKLSYIKPTDFYDWQKEIYNIILDKPNDRTVHYIYGNGNDGKSLGL
                     CRTLGILHSNECIQIDDGDDSDVKYLISNFICDEDGNQREDKNLKCVILDLPREFNIE
                     KLNSKTIEKIKNGCCVNLKYKGKTNYFNPPHLIIFTNTEPSSSVLKKLTLDRWKIGEI
                     KEKKIDWKNTNDFLI*"
ORIGIN
        1 tagtattacc ataatacacg gtctctccca cgaagtgcga atgtggggat gtcgtgatca
       61 tgtaatgatc atgacagggg cgccacacca ctatattata acattttata gtgcgttttt
      121 attagacaat ttcgccgatg ttacggattc ccatcccttc gtcaacgaat aacatcgtct
      181 cattattatt atctaatggt gcgttattgt ctaccttacg agtcttataa cttctaggtt
      241 taggagtcct aaaatattct ttttctgctg aatagttatt ataagacggt aaaggtttaa
      301 ccttataact tttagggtta tcataaggtc tactataact attatatctc ggttcttttt
      361 tgagaatagg ctttatttct tctttaagcc tgttgtattt attttctcgt tctatgtatc
      421 ttttcattag tttttcaaat aaataatcgt cctttgtttt tttatcactc attatatgtt
      481 agggatagaa aaaataaaaa taataaatac atctataatt ctttaagcca ctatggcgga
      541 atttacctct tgaaccaata aatcacatac tactagtgaa gcggggactg ctatacaatt
      601 tgcaaacacc aaaatgacat cgcctgccac cgttgggtca aaagagaaaa aagccgttat
      661 tcccatttct gcattattag ctggtccacg gggataaggt tcttggttcg aacctcctga
      721 ttgtaggagg tcgacacctt tcgccgaacc ttgatatact actataatag ctccgacatt
      781 tgtcgttgta gccccccgta attgatagta taaacaatat aggccttttg ggtctagttt
      841 gcttctcggt atagtcaaag tatttgcatt taatgtaata ccataattgt caaatacttt
      901 aatacgtgaa gcgctaccaa aaagggatgt aggagtaacc gttccgacat ctatcctata
      961 atgtgccata cctgcatttg ttagtgcttt attttgaact ggtgcaatta gttcaatgtc
     1021 atagcttacc catagttctc ctaaattcac attttctttt tgtcctcctg ctgtagctat
     1081 ttggaaaact ccgaggttat cgaaacgtgg gtcggctatt gtgttttctg aaccattgtt
     1141 gacatacatt tcgcctaatg gagtttgatt ttttgcgcat tcaataccga ataacataga
     1201 ttgcgaaggt ttggagctca taccccattg tgaattttcc atttgttgtt ttgttgtaaa
     1261 aagtgggtca acacttgaat aattacatgc cataataaca gaacctaaag ctgtatttgt
     1321 agagtttaaa gcatctgcac tcatacttct aaattcatat acaattccta aaggtctcca
     1381 cttttggtat gaagtattag caatatttgc caaccatggg aaagagctct taatacctgg
     1441 gttcaattgg taagattgaa ggttaaaagc tcccggtgta gttgggtgtg tatagacatc
     1501 gcccaaatat tcacgatggc aaattctcgc cgtattactt gctttgttgg agtgcatctc
     1561 tggaacttgt tgagcgatat tcataagtgt atttgctttg acttggttac tagcataatc
     1621 accgctacca attaatttac cgattaattt atcaactcca ctagatgctg tatcaacaac
     1681 gtaatcaact gctttatgtc cgtaattttt agctgtgttt ttgtaggatt caggaatgta
     1741 atccccacga cctttcagac gtctaacacc taaaatacgg gcaactttac ggctagcttt
     1801 gcgactcgct ttacgggagc ttcgttttct caaaaaagac atttttctat atatgtttag
     1861 aaatagaaaa taaaataata aattatatgc ataaaattat atctttaagt agtttataca
     1921 aatgtttgaa aaattgttta agtctatgtt tcattgtata aataagtaag atatatttta
     1981 taccgtttcg gtataataaa tttatgatcc gaaggatcat aaatttagta tccgaatccg
     2041 aggaggcgta gacgacgaac gagcctagaa gcttggagca attttttcag gcggagagcc
     2101 ggtgagcgaa gcgaagaaaa aacgagggtt cgcttgcgaa ccttgcggcg ctggctggcg
     2161 agcatagatt ggatggttaa attagaaagt cgtttgtatt tttccaatct atttttttct
     2221 ctttgatttc tcctattttc catcggtcta atgtaagttt ttttaaaact gaactagatg
     2281 gttctgtgtt tgtaaatatg attaaatgtg gaggattaaa atagtttgtt ttacctttat
     2341 atttaagatt tacacaacat ccattcttta ttttttctat tgttttgcta tttaactttt
     2401 caatgttaaa ctctcttggt aaatccagga tgacacattt taaattttta tcttcacgtt
     2461 gatttccatc ttcgtcacat ataaaattac tgatgagata tttaacgtcg ctatcatctc
     2521 cgtcatcaat ttggatacat tcattactat gtaatatacc gagagtacgg cataaaccta
     2581 aactttttcc atcattacca tttccgtata tataatggac agttctgtca ttcggtttat
     2641 ctagaatgat attatatatt tctttttgcc aatcataaaa gtcggttggt ttaatatatg
     2701 ataatttatt aaccattatg ttcttactat acgttttacc gttagagctt tccaatttag
     2761 aacaataatt gatggatgct tttttattcc tcgttccttc ccagtgaatt ctatcgctta
     2821 aatttaattc ggtaggtctc atatcatcat gtagaaatat aacaccctgt aagtgtgtgg
     2881 ttccctcttt ttcatctgtt tttatacagt taccatcttc attccatgaa gcgttgaaag
     2941 ttccttttcc tttttcttct tgaaaaatat atttctcaca tactgaatta aacttttctt
     3001 taagtgtgtt tatatcatct attgtatagt tattgaaagt aaaaaaccat gattttcttt
     3061 tttttggttg tttagactcc ttggacttta tgggggtctg ggagtctaaa acaatatttt
     3121 tacttttttt tgttttgact atactacttt tttctattgt atcaataata gtgttaccat
     3181 aattcacggg tactattgag gtattttggg gtactatttt tttcatttat aactatatta
     3241 gagaaaataa aatgtaaata tttgaacgca tatagtgcgt tcaaaatacc gaaatagaga
     3301 ccgtggatta t
//
LOCUS       CruV-213                3314 bp    DNA     circular     24-FEB-2020
DEFINITION  Crucivirus-213 isolate 120_1420_IR2_3297.
ACCESSION   CruV-213
KEYWORDS    .
SOURCE      Crucivirus-213
  ORGANISM  Crucivirus-213
            Unclassified.
REFERENCE   1  (bases 1 to 3314)
  AUTHORS   de la Higuera,I., Kasun,G.W., Torrance,E.L., Pratt,A.A.,
            Maluenda,A., Colombet,J., Bisseux,M., Ravet,V., Dayaram,A.,
            Stainton,D., Kraberger,S., Zawar-Reza,P., Goldstien,S., Briskie,J.,
            White,R., Taylor,H., Gomez,C., Ainley,D.G., Harding,J.S.,
            Fontenele,R.S., Schreck,J., Ribeiro,S.G., Oswald,S.A., Arnold,J.M.,
            Varsani,A., Enault,F. and Stedman,K.M.
  TITLE     Direct Submission
  JOURNAL   Submitted (24-FEB-2020) Biology, Portland State University, 1719 SW
            10th Ave, Portland, Oregon 97201, USA
COMMENT     Bankit Comment: ALT EMAIL:ide@pdx.edu
            Bankit Comment: TOTAL # OF SEQS:451
            Bankit Comment: GAP: est. length - alignment

            ##Assembly-Data-START##
            Assembly Method       :: MetaSPAdes v. 3.12; IDBA-UD v. 1.1.1;
                                     MEGAHIT v. 1.0.3
            Sequencing Technology :: 454; Illumina
            ##Assembly-Data-END##
FEATURES             Location/Qualifiers
     source          1..3314
                     /organism="Crucivirus-213"
                     /mol_type="genomic DNA"
                     /isolate="120_1420_IR2_3297"
                     /isolation_source="Water"
                     /country="Norway"
                     /collection_date=2011
     CDS             1..873
                     /codon_start=1
                     /product="Putative Replication Associated Protein"
                     /translation="MVVQRSRAFTFTLNNYADDVLDKLEELVCVYMIAGAEVAPSTGT
                     KHVQGYVYFANARSMKSVIKLVHAIHPGGHVEMARGTVDQNYEYCSKEGDFIEFGIKP
                     LSSEEKGELEVDRWDEARAFAKEGRFDEISSDLFVKYRNAFKGIRQDFQEAMIHPEND
                     VLENYWFCGKSGSGKSREARKRWPGAYLKMKNKWWNGFKIGEHETVIIDDVDPTHQNW
                     IGAFLKEWSDHYPFQAEIKNGTFVIRPKRIVVTSQYTIEQIFTDPETVSALKRRFATK
                     HFGRDAVLHRELVE*"
     CDS             complement(1595..3118)
                     /codon_start=1
                     /product="Putative CruV Capsid Protein"
                     /translation="MSTMSYSVNNNKRPRAAYDMDSLAKAMSSAKALNRFEQRAAAME
                     AENNYNVGKVAIPKSYARGAAVAGGAALAGALARKAYNMATQKKPRRRRKRAAIGGIR
                     GQGAYFTDGGALLGAGLGAGFGSLGGGVGAFGGAGLGYQLGSSAGSLIDQLTGMGEYS
                     IKANSILAKVDEGVQIPSFGNLTNATIVQHRSFVQDIVTPANPLVFNVQQFRINPANQ
                     QLFPWLSTLANSFEQYQILGMVFQYKSLSSDSATTLALGSVTMGTKYEANDPPFRNKI
                     EMENAQYTCSGKPCDTFIHALECDPTVMSDPIKYNLQSGIYPIGKDPRGYDVGTFEIA
                     TTGLPSTAGEILGELWITYEVALFRPLLNTISASDRYTITAPTSPNTLIGPAATVRLP
                     DAGSTLGTTVVGSNLNFPSGLTRGTYNVRLAYQGGATALTNTLAPTFVNCARSPALDT
                     YFVSAGATSSYQIWDAIIIVTAAGASIAFGAGTPTLPTAINRATLLVNQIPDNWNIV*
                     "
ORIGIN
        1 atggttgtgc agcgctctcg tgccttcacg ttcacgttga acaattacgc cgatgatgtt
       61 ttggacaagc tggaagagct tgtttgtgtg tacatgatcg ctggtgccga agttgccccg
      121 tcgaccggca ccaaacatgt acaagggtat gtatatttcg cgaacgctcg ttcgatgaaa
      181 tcggtgatca agttggtgca cgctatccat cctggtggcc acgtggaaat ggcgcgcggc
      241 actgtcgatc aaaactacga gtattgttcc aaagaagggg atttcattga atttggaatc
      301 aagcctttgt cttctgaaga gaagggtgaa ctcgaagtcg atcgctggga tgaagcccgt
      361 gcatttgcga aagaaggccg atttgatgag atttcgtctg atttgttcgt caaatatcgt
      421 aatgccttta agggaatccg tcaggatttc caagaggcta tgattcatcc ggaaaacgat
      481 gtgttggaga attattggtt ctgtggcaaa agtggttccg gaaaatcgcg cgaagcgcga
      541 aagcgttggc cgggcgcgta tttgaagatg aagaacaaat ggtggaacgg tttcaagatc
      601 ggtgaacatg aaactgtgat cattgatgac gtcgacccga ctcatcaaaa ttggattggt
      661 gcctttctga aagaatggtc cgatcactac ccgttccagg ccgaaattaa gaacggaacg
      721 ttcgtaattc gcccaaaacg gattgtcgtt acgtcccagt acacgatcga gcaaatcttt
      781 acggaccctg aaacggtgtc cgcattgaag cgtcgttttg cgactaaaca ttttggtcgc
      841 gacgctgtcc ttcatcgcga gttggtggag tgatggaggg gtcccatctc gaacatttgg
      901 ccgacggaca aatatcagta cagcttcatg tgtactgttt atctcaatta tgtccccttc
      961 atttgttccg aagcgaatca gtgtggctga gctgacacgg gagatgattc tggatttagc
     1021 cgctgaatat ttggcggaag acgaggcgga gcctcctcgt ctaacgcgaa cgcgtcgtat
     1081 gtcgccacga tttcaccgcg gttatcctgt gatcaatttg acgttcgcct tcgacgaacc
     1141 cgacctgaac ctttaaaata aaatcccatt aaagtttgaa tgatggtggc ccagagggtt
     1201 ttttagatta agcgcgaagc gcttaatcta aaaaacccgc ggccaccaga atgggcttag
     1261 attgttatgc cctttagggt gcaagctttg gctttttagg gattttaggt ttttttttgg
     1321 gttcatggaa ggggggtccc ataagcttct atattattat cttaacataa agcttccgtt
     1381 gtggaacgga tgctgcaatg ttatttaatg cgtttccttt ggaaatagct gtacatatat
     1441 tggacttcct caattggttt gaggaagttg agatgttcgg ttggaacggg ttgggattga
     1501 gtcctcgaat tgaattccaa attcgttaca ataaagattt aaattggcgt aattaaggca
     1561 taaaaaccca gtgggaaaag ccgttttatt aatattaaac aatattccag ttatctggga
     1621 tttggttaac gagcaatgta gctcggttaa tcgcggttgg caaggttggc gttcctgccc
     1681 caaatgcgat gctcgctcct gcagcagtaa caataataat ggcgtcccaa atctggtagg
     1741 acgacgtagc gccagctgaa acgaagtacg tgtcgagagc gggggatcgc gcgcaattaa
     1801 cgaatgtagg cgccagtgta ttagtcaggg cggtagcacc gccctggtag gctaggcgaa
     1861 cgttgtatgt accccgagta agaccggaag ggaagttaag attagaacca accaccgtcg
     1921 ttcccaatgt ggaacccgca tcgggtagcc gcaccgttgc ggcaggaccg atcagtgtgt
     1981 ttggagaagt tggggcagta atcgtgtaac gatccgaagc cgaaatcgtg ttgagcaaag
     2041 ggcgaaacag tgcaacttcg tacgtaatcc acaattcgcc caaaatttcc ccagctgtgc
     2101 tgggtagccc cgtagtggca atttcgaatg tgccgacgtc gtatcctcga ggatctttac
     2161 caatggggta aatgcccgac tgcaggttgt atttaatggg atccgacata actgtcgggt
     2221 cgcattctag ggcgtgaatg aatgtatcgc atggtttacc ggaacacgtg tattgcgcgt
     2281 tttccatttc aattttgttg cggaacggcg gatcgtttgc ttcgtatttc gtccccatgg
     2341 taacgcttcc gagagccaat gtggtcgctg aatcggaaga cagcgatttg tattggaaca
     2401 ccatgcccag aatctggtat tgttcgaaac tgtttgctag tgtggacaac cacgggaaaa
     2461 gctgctggtt tgctgggttg atgcggaatt gctgaacgtt gaagactagg gggttcgccg
     2521 gagttacgat gtcttgcacg aaagaccgat gttgtacgat cgttgcattg gtaaggttgc
     2581 cgaaagatgg aatttggact ccttcgtcca cttttgccaa aattgaatta gccttaatac
     2641 tgtactctcc catccctgtc agctggtcga ttaacgatcc agcagacgat cccagctggt
     2701 accccaggcc tgcacctccg aaagcaccca cgcctccacc gagtgaacca aaacccgcgc
     2761 cgagtcctgc cccaaggagt gctccgccgt cggtgaagta ggctccctgg cctctaattc
     2821 caccgatcgc cgcgcgcttg cggcggcggc gcggcttttt ttgcgtagcc atgttataag
     2881 cttttcgcgc cagcgctcca gcaagcgccg cgccgcccgc gacagcggcg cctctcgcgt
     2941 acgattttgg aatggcgact ttgccgacat tgtagttgtt ctcggcttcc attgccgctg
     3001 ctcgctgttc aaagcgattg agcgcttttg ccgagctcat agctttcgca agggagtcca
     3061 tatcgtatgc cgctcgcggc cgcttgttat tgttaacgct gtatgacatc gtactcatag
     3121 cgaatgaaac aaaaacaccg tgacacttga taaagactgg ctgcggacga agtccgtggc
     3181 cagtcagaaa tgacacacgg ggccttgggg aagcgcagcg tagtattacc ccaaggctag
     3241 tgtgtcattg tgtcagtttt tgaaaagccc gctctttttg tgaagtccgg aggagtggca
     3301 gcccccaatt caaa
//
LOCUS       CruV-214                3316 bp    DNA     circular     24-FEB-2020
DEFINITION  Crucivirus-214 isolate 120_904_SvL1_3295.
ACCESSION   CruV-214
KEYWORDS    .
SOURCE      Crucivirus-214
  ORGANISM  Crucivirus-214
            Unclassified.
REFERENCE   1  (bases 1 to 3316)
  AUTHORS   de la Higuera,I., Kasun,G.W., Torrance,E.L., Pratt,A.A.,
            Maluenda,A., Colombet,J., Bisseux,M., Ravet,V., Dayaram,A.,
            Stainton,D., Kraberger,S., Zawar-Reza,P., Goldstien,S., Briskie,J.,
            White,R., Taylor,H., Gomez,C., Ainley,D.G., Harding,J.S.,
            Fontenele,R.S., Schreck,J., Ribeiro,S.G., Oswald,S.A., Arnold,J.M.,
            Varsani,A., Enault,F. and Stedman,K.M.
  TITLE     Direct Submission
  JOURNAL   Submitted (24-FEB-2020) Biology, Portland State University, 1719 SW
            10th Ave, Portland, Oregon 97201, USA
COMMENT     Bankit Comment: ALT EMAIL:ide@pdx.edu
            Bankit Comment: TOTAL # OF SEQS:451
            Bankit Comment: GAP: est. length - alignment

            ##Assembly-Data-START##
            Assembly Method       :: MetaSPAdes v. 3.12; IDBA-UD v. 1.1.1;
                                     MEGAHIT v. 1.0.3
            Sequencing Technology :: 454; Illumina
            ##Assembly-Data-END##
FEATURES             Location/Qualifiers
     source          1..3316
                     /organism="Crucivirus-214"
                     /mol_type="genomic DNA"
                     /isolate="120_904_SvL1_3295"
                     /isolation_source="Water"
                     /country="Norway"
                     /collection_date=2012
     CDS             1..1098
                     /codon_start=1
                     /product="Putative Replication Associated Protein"
                     /translation="MSKTKNDKKFRGFCCTDYVCDNLFWGKQFEDGMIEYLIVGQEFC
                     PKTGREHYQVFLYSKNPRGYGAMKKNLSYLGVDGNTQYRWCAPTKGSAAQNKRYCSKE
                     GRFWEMGICPAQGKRRDLDRMREEGKKKVLDGMRVNKVMIDLAEQNMQVYCQYSKPFE
                     KYLLSQEPVRDWIPEVILIFGPPGTCKSRTAIEDGAQKVYVSGVPANPFISGYEGSDI
                     VLFDDFDQRQIERSAMLCLLDRYEHRVNTKGSDRNWKPKKFYFTSNDDPLKWYTGDYA
                     WIRRLTKAIDLSSGFREYYVRVPGTARWEPVDKATFEAARPVEEGHLRALPELSAEAL
                     EKKRAAFKAAEVFQNFIPAAAPAAAAPVYFG*"
     CDS             complement(1416..3110)
                     /codon_start=1
                     /product="Putative CruV Capsid Protein"
                     /translation="MSTRIRNPQAYAAAVARNGPLPPRAGPPPLRPPVYKPYRAPASA
                     GAGYRRPYVPRAVAAAPRRTYTRTAPRRVPRAVSAMSGAGGSVLATLMGRGTYVVNKN
                     TLVHPTQSEQVPAFGNVSGGTRIRHREFIQDIVGSTVFTNRNYPINPGNAEVFPWLAS
                     TAKNYEQYKINGMIFEFVSTSATALNSTNTALGTVILSTQYNSLAPSFTNKQQMENNV
                     FTVSCKPAESVMHPIECEPSQTSVPILYTYSGTLPPTGDIRLYNAGNFQLATVGMQAD
                     AVIGELHVTYDVTFYKPQLDQGLSLDSSTALYNLYGIGVTGAGITAAFPFGGEAGQNP
                     IKWFDSIGLTIDQSTGNVFLPTGSSGTYLFQYGVIGQYTAMQDKPTLTYFNIDETESF
                     PYFGSANPFSVSAVAAAEMAVSVIFKILDPNFPAYINVDFAGAAVPPGVSNAGFIFVQ
                     QMNGDFPATSQEPFPTLYESAAAATSSSHAFGPPKKSSLKQKQPEAAAAHSATASSSS
                     IPQVASEDEEKETEYMDIPRSPPASIPVQLQNPGPRRTSIPKNPSTSLLGRAFGRP*"
ORIGIN
        1 atgtccaaga ctaagaatga caaaaagttt cgtggatttt gctgtactga ttatgtatgc
       61 gacaatttat tttggggcaa acaatttgag gatggaatga ttgaatatct catcgtggga
      121 caagaatttt gtccaaaaac tggaagagag cattatcaag tttttcttta ttcaaagaat
      181 ccgagaggat atggtgcgat gaagaaaaat ttatcgtatt tgggtgttga tggaaatact
      241 caatatcgct ggtgtgcgcc aactaaagga tcggcagctc agaataagag gtactgttca
      301 aaagaaggga gattttggga gatgggtatt tgtccagctc aaggaaaaag aagagattta
      361 gatagaatga gagaagaagg aaaaaaaaag gttttggatg gtatgagggt taataaagtg
      421 atgattgatc ttgcggaaca aaacatgcaa gtatattgtc agtattcgaa gccatttgaa
      481 aaatatctat tatcgcaaga accggttaga gattggatac cggaagtaat tttaattttt
      541 ggaccgcctg gaacgtgtaa atcgagaact gcgattgaag acggggctca aaaagtttat
      601 gtaagcggag ttcccgctaa tccgttcatt tcgggatatg aaggaagtga tatagttttg
      661 tttgatgatt ttgatcaaag acaaattgag agatcagcga tgctgtgttt attagatcgg
      721 tatgaacacc gtgtgaatac aaaaggttcg gatagaaact ggaagccgaa gaaattttat
      781 ttcactagta atgatgatcc actaaagtgg tatacgggag attatgcttg gattagaaga
      841 ttaaccaaag cgattgatct ctcaagtggc tttcgagaat attacgtaag agtgcctgga
      901 actgctagat gggagccagt tgataaggca acgtttgagg cggctaggcc tgttgaagaa
      961 ggtcacctga gggctttacc tgagttgagt gctgaagcat tagagaagaa aagggctgct
     1021 ttcaaggcag ctgaagtatt ccagaatttc attccggctg cggcaccggc tgcggcggcg
     1081 ccagtctact ttggttaaaa tatacactca aaatcgtgta taaaattaga atttttggaa
     1141 ctaaagttaa aattaggtgt tccatttttg gtttttggtt ttggaacaga agtactctgt
     1201 gggtaataaa tagccacaga gtacgagatt tttgattttt aattgctttt ggaaaaactc
     1261 gcttcgctcg tttttctttc taggcgctcc gcgcaagaac tcgcttcgct cgttttcacg
     1321 ccttcggcgt aattacactt ggtggatgaa gtgttttata atgttttcga tgatatacag
     1381 tttatgtata ttattatgaa gagatgaaaa tatttttaag ggcgaccaaa tgctctgcct
     1441 agaagtgagg tgctaggatt tttgggaatg gaagttcgac gagggcccgg attttggagt
     1501 tggactggaa ttgaggctgg aggggatcga gggatgtcca tgtattcggt ctctttctct
     1561 tcgtcttcgg aagcgacttg aggaatcgag gagcttgacg cagtggcaga atgggcagct
     1621 gcagcctctg gttgtttttg cttgagtgat gatttttttg ggggaccaaa tgcgtgagaa
     1681 gaagaagttg cagcggcggc agattcatat agagtgggaa atggctcttg agatgttgcg
     1741 gggaaatcac cattcatttg ttgaacaaag ataaaaccgg cattgctcac accggggggt
     1801 acggcagcac ctgcgaagtc aacattgatg taggcaggga agttaggatc tagaattttg
     1861 aaaattacag aaacggccat ttcggcggct gcaacggcac taacggagaa tggatttgca
     1921 ctaccaaaat atggaaaact ttcagtttcg tcaatgttaa aataagtaag agtgggttta
     1981 tcttgcattg cagtatattg accgataacg ccatattgaa aaagataagt gccgcttgat
     2041 ccagttggaa gaaatacatt tccagttgac tgatcaattg tgagaccaat actatcaaac
     2101 catttgattg gattttgacc ggcttctcct ccgaaaggaa atgcggcagt aatgccagca
     2161 ccagtgacac caatgccata aagattgtag agagcagtgc tagaatcgag agaaaggcct
     2221 tgatcaagtt gaggtttgta aaaagtaacg tcataagtta cgtgaagttc tccaataact
     2281 gcgtctgctt gcattccgac agttgcaagt tgaaaattgc cggcattgta aagacgaata
     2341 tctccagtag gaggtagagt tccagaataa gtgtaaagaa taggaactga agtttgcgaa
     2401 ggctcacact cgataggatg catgacagat tctgcaggtt tgcaagaaac agtaaacaca
     2461 ttattctcca tttgctgctt gttagtaaaa gatggagcga gggaattata ttgtgttgac
     2521 aaaataactg ttcccaaagc tgtgtttgtg gaattcaaag cagttgctga agttgaaacg
     2581 aattcaaaaa tcataccgtt gattttgtac tgttcgtaat ttttagcagt tgaagctaac
     2641 caaggaaata cctccgcatt gcctggattg ataggataat tgcgatttgt aaaaacagta
     2701 gatccaacaa tatcttgaat aaattctcgg tgtcgaattc gagttcctcc ggatacgttt
     2761 ccaaatgctg ggacttgttc actttgagtt ggatggacta acgtattttt attcacaaca
     2821 taggttcctc gacccataag tgttgcgaga acagaaccgc cagctccgct catggcagaa
     2881 accgcgcgag gcacacgtct aggtgcagtt ctagtataag ttcgacgagg agctgcggct
     2941 acagctctgg ggacataagg tcttcgatat ccggctccag cagaagcggg ggctcgataa
     3001 ggtttataga caggaggtct aagcggagga ggtccagcgc gaggaggaag agggccattg
     3061 cgagcaacgg cggcagcata agcttgcgga tttcgaattc gagtcgacat gatttaatat
     3121 gtcagtaaaa ttttaaaaaa tcttcgattt cagaaaatga aatggggtgg aacagaagtt
     3181 gatttttatt tttatttttg gaacagaagt aaacatggcc tgtgctgttg aaaaagaaaa
     3241 agttgatgaa actttagaag ttgaagaaat cgaagaagat ttcacaaaag aagttttcga
     3301 agaaggattt cacgaa
//
LOCUS       CruV-215                3326 bp    DNA     circular     24-FEB-2020
DEFINITION  Crucivirus-215 isolate 120_155_SvL2_3298.
ACCESSION   CruV-215
KEYWORDS    .
SOURCE      Crucivirus-215
  ORGANISM  Crucivirus-215
            Unclassified.
REFERENCE   1  (bases 1 to 3326)
  AUTHORS   de la Higuera,I., Kasun,G.W., Torrance,E.L., Pratt,A.A.,
            Maluenda,A., Colombet,J., Bisseux,M., Ravet,V., Dayaram,A.,
            Stainton,D., Kraberger,S., Zawar-Reza,P., Goldstien,S., Briskie,J.,
            White,R., Taylor,H., Gomez,C., Ainley,D.G., Harding,J.S.,
            Fontenele,R.S., Schreck,J., Ribeiro,S.G., Oswald,S.A., Arnold,J.M.,
            Varsani,A., Enault,F. and Stedman,K.M.
  TITLE     Direct Submission
  JOURNAL   Submitted (24-FEB-2020) Biology, Portland State University, 1719 SW
            10th Ave, Portland, Oregon 97201, USA
COMMENT     Bankit Comment: ALT EMAIL:ide@pdx.edu
            Bankit Comment: TOTAL # OF SEQS:451
            Bankit Comment: GAP: est. length - alignment

            ##Assembly-Data-START##
            Assembly Method       :: MetaSPAdes v. 3.12; IDBA-UD v. 1.1.1;
                                     MEGAHIT v. 1.0.3
            Sequencing Technology :: 454; Illumina
            ##Assembly-Data-END##
FEATURES             Location/Qualifiers
     source          1..3326
                     /organism="Crucivirus-215"
                     /mol_type="genomic DNA"
                     /isolate="120_155_SvL2_3298"
                     /isolation_source="Water"
                     /country="Norway"
                     /collection_date=2012
     CDS             1..1149
                     /codon_start=1
                     /product="Putative Replication Associated Protein"
                     /translation="MNGCARAWPLIVFLVEDSLDHHIATRMADIKLEYVADADSEVGR
                     IIYTGDFLKQRARNWCFTHNNYTQEDILVVKAWATTLAGFMGVMFEEEAGEAKATPHL
                     QGFVCFSTMKSGKQMNEIGPRTHWSVMKGSIESNKTYCSKDDSGVVVIGQFPMSARDK
                     GIAGAAHGVKGKQFGQLAPASWDELIKAIKAGKTRRELMEAFPKLAGQYSKGFDNYIA
                     EFTPKHTFSILERYGSYLPWQEQLIDIIALGAGPREVIWIYSPEGNVGKSDMAKHLIY
                     NQGFQPLQNATSRDLSCAWQCGSVVIDLARDQGGSPINYGFMECVKDGRSFSSKYEST
                     CKLADPKRNRFVICFANERPDPSKLSADRWAIFRVTGGILQWEEPGLV*"
     CDS             complement(1344..1652)
                     /codon_start=1
                     /product="putative ORF"
                     /translation="MAQMVSATDQAKWTMQPKAITLTPSPLPSRAKRAEGARPSPCSH
                     AKRVEGRRPRRGFGPEIPSCSVRRNRRRAARMRGAGAKRRHRQPCAYPPPKSRGKKCK
                     *"
     CDS             1913..3289
                     /codon_start=1
                     /product="Putative CruV Capsid Protein"
                     /translation="MLYIATCHRSKMPQAKAAPVRRNRGKKAAPKARKAPVRAAGPSR
                     RNLSAAQAFRGAGGGMGTALGSFLGGPAGGAMGNFVGRGAGALLSKIFGHGDYQVSNV
                     QGIKENSLVVANAANIPEFGTGRVAVKFRHREFLGDVISSATVGEFKIDSYSINPGVS
                     RSFPWLSQVVGSSFQQHRINGLTYEFRSMSSDALNSTNTALGSVIMATDYDSPDAVFA
                     SKQQMENTEYGVSCKPSVNMMHAIECARGQTPVSELYIRAYDNPPNTDLRLYDLGRFS
                     IATVGCQGANVNLGELWATYDIDAFKAIEQPPGYSIPQADYIITADGKVSLDAHPFGN
                     VVADVGRKVNIDQIGLTVTGQTVIFPYSIAPQTAYNVSWQAVGDSTALTLGTLVGSNG
                     MDCGTRFIMANDTSTKAIWTCNATYFGGASPSAPPTITFQGTTLPANVTFAELSVSQI
                     GSQQTGPG*"
ORIGIN
        1 atgaatggat gcgcacgggc atggccatta attgtttttt tggttgaaga ttctcttgac
       61 caccatatag ctacgcgaat ggccgatatc aagctagagt acgtagccga tgcagatagt
      121 gaagtaggac gcattatcta cactggcgac ttcctcaagc aaagagctcg gaactggtgt
      181 tttacacaca acaactacac gcaagaagac atcctggtcg ttaaggcctg ggccacgaca
      241 ttagctggct tcatgggtgt gatgttcgaa gaggaggcgg gagaggctaa agcaacccca
      301 cacctccaag gcttcgtgtg cttctctacg atgaagtctg gcaagcagat gaacgagata
      361 ggacctcgta cgcactggtc ggtcatgaaa ggatctatcg agtccaacaa gacctactgc
      421 agcaaggacg actccggggt cgtggtcatt gggcagttcc ccatgtccgc aagggacaag
      481 ggcatagctg gagcagctca cggagtcaag ggcaaacagt tcggacagtt agcccctgcg
      541 tcctgggatg agctgatcaa ggctattaaa gcgggcaaaa ctaggcgaga gttaatggaa
      601 gcgttcccga agctggcagg gcagtattcg aaagggtttg acaattatat cgctgagttt
      661 acccctaaac atacgttctc catcctggag cgatacggct cgtatctccc gtggcaagag
      721 cagctaatcg acattatcgc tctcggcgcg ggacctcggg aagtgatctg gatctactcc
      781 cctgagggta acgtaggcaa gtccgacatg gctaagcacc tgatttacaa tcaaggcttc
      841 caaccgctgc aaaacgccac aagcagggac ctgtcgtgcg cttggcagtg cggttctgtc
      901 gttattgatc ttgcgcgaga ccagggaggt tcgccgatca actatggctt tatggagtgc
      961 gtgaaggacg gcaggtcctt ctcatccaaa tacgagtcta cctgcaagct ggcagaccct
     1021 aaacgcaata ggttcgttat atgcttcgca aacgaacgcc ccgatcccag taagctatca
     1081 gctgaccgct gggctatctt ccgtgtcacg gggggtatcc tccaatggga ggagcccggt
     1141 ttggtctgag attgcggagt acctgtgttt cccctgcggg gggctgggtg cgccgcctgc
     1201 ggcgggcggt tcgtgtgttc cgcatctcga cgaccattta gcgttccggg cgttgccctt
     1261 cacttgaccc ctctgagctg agcgcgaggg gggggtctag gtaatagtag gctacgccac
     1321 tagacccccc tcgagatcac acctcattta cattttttgc ccctagattt ggggggaggg
     1381 tatgcgcatg gctggcggtg cctccgcttc gctccggctc ccctcatgcg tgctgccctt
     1441 cgcctattcc tgcgcacgct gcagctaggg atctcgggcc cgaaacccct gcggggtctt
     1501 cggccctcaa cccgcttcgc gtgactgcag gggctcgggc gcgcgccttc ggcccgcttc
     1561 gccctgctgg gaaggggtga tggtgttaat gttatagcct tcggctgcat tgtccatttg
     1621 gcctgatcag tggccgagac catctgggcc aaccctattt aggacctaaa ggccctttgg
     1681 atgactgcct gatgttgtta gccaggctag attatcggtc ttccgatcgt tgctatgcaa
     1741 ctcgcggccg aatcctacgg atcccctccc ggggccgaat tcccgccttc tacaacaaga
     1801 ggcatttaat tatttatttg ggagaagaaa gctccaaaac aggggggttt cggagagttc
     1861 ttggcgaaat caattaatta tttaattaat tattctcgtc aaagattgtc ggatgctata
     1921 tatagcgacc tgtcatcgtt caaagatgcc tcaagctaaa gcggcacctg tgagacgcaa
     1981 cagaggaaag aaagcagcac caaaagcacg taaagcccca gtccgtgcgg ctggcccatc
     2041 acgacgcaat ctgagcgcgg ctcaggcatt ccgtggtgct ggtggtggca tgggtacggc
     2101 attgggaagc ttccttggag gacccgcagg cggggcgatg ggtaactttg ttggaagagg
     2161 agcaggggcg ctcctgagta agattttcgg acatggagac taccaagtat ccaacgtcca
     2221 agggatcaag gaaaacagct tggtggtggc aaacgcagct aacatcccag agttcggaac
     2281 aggacgagtg gcggtcaaat tccgccatcg cgagttcctc ggggacgtga tctcgtcagc
     2341 aaccgtagga gagtttaaga ttgatagcta ctcgattaat cctggtgtca gtagatcatt
     2401 cccgtggctt tcccaagtgg tcggatcttc gtttcaacaa cataggatca acgggctgac
     2461 ctacgagttt aggagcatgt catcggacgc tctgaactct acaaacaccg ctttgggttc
     2521 tgtcattatg gcgactgact acgacagtcc ggacgctgtt tttgcctcaa aacagcaaat
     2581 ggagaacact gagtatgggg tgtcatgtaa accatcggtt aacatgatgc acgctatcga
     2641 gtgtgccagg gggcaaactc cggttagcga gctgtacatt agagcttacg ataacccgcc
     2701 taacactgat cttaggctgt acgatttggg gcgattcagt atcgcgactg ttgggtgcca
     2761 aggggcaaat gtcaatttag gcgagctgtg ggctacctat gacattgatg cgttcaaggc
     2821 gatcgaacaa ccgcctgggt acagcatccc acaggccgac tacatcatca ctgcggacgg
     2881 taaagtaagc ttagacgctc acccgtttgg caacgtggtc gcggacgtag gaaggaaagt
     2941 aaacatagat cagatcggtt tgacggtgac gggacaaacg gtgatctttc catactcgat
     3001 tgcgccccag acagcctaca acgtgagttg gcaggcagtg ggtgattcaa ccgcattaac
     3061 gcttggaacg ctcgtcggct ccaacgggat ggattgcgga acacggttta tcatggcgaa
     3121 cgacacatcg actaaggcga tttggacctg caatgcgaca tacttcgggg gtgcctcacc
     3181 atcggcacca ccgactatca cctttcaggg aacgacgctg ccggcaaatg tgacctttgc
     3241 tgagctatcc gttagtcaga tcggatcaca gcagactggc ccagggtagg gcctgcgcga
     3301 tgatcatctc gatcgacttt tagtta
//
LOCUS       CruV-216                3330 bp    DNA     circular     24-FEB-2020
DEFINITION  Crucivirus-216 isolate 120_1417_IR2_3297.
ACCESSION   CruV-216
KEYWORDS    .
SOURCE      Crucivirus-216
  ORGANISM  Crucivirus-216
            Unclassified.
REFERENCE   1  (bases 1 to 3330)
  AUTHORS   de la Higuera,I., Kasun,G.W., Torrance,E.L., Pratt,A.A.,
            Maluenda,A., Colombet,J., Bisseux,M., Ravet,V., Dayaram,A.,
            Stainton,D., Kraberger,S., Zawar-Reza,P., Goldstien,S., Briskie,J.,
            White,R., Taylor,H., Gomez,C., Ainley,D.G., Harding,J.S.,
            Fontenele,R.S., Schreck,J., Ribeiro,S.G., Oswald,S.A., Arnold,J.M.,
            Varsani,A., Enault,F. and Stedman,K.M.
  TITLE     Direct Submission
  JOURNAL   Submitted (24-FEB-2020) Biology, Portland State University, 1719 SW
            10th Ave, Portland, Oregon 97201, USA
COMMENT     Bankit Comment: ALT EMAIL:ide@pdx.edu
            Bankit Comment: TOTAL # OF SEQS:451
            Bankit Comment: GAP: est. length - alignment

            ##Assembly-Data-START##
            Assembly Method       :: MetaSPAdes v. 3.12; IDBA-UD v. 1.1.1;
                                     MEGAHIT v. 1.0.3
            Sequencing Technology :: 454; Illumina
            ##Assembly-Data-END##
FEATURES             Location/Qualifiers
     source          1..3330
                     /organism="Crucivirus-216"
                     /mol_type="genomic DNA"
                     /isolate="120_1417_IR2_3297"
                     /isolation_source="Water"
                     /country="Norway"
                     /collection_date=2011
     CDS             1..957
                     /codon_start=1
                     /product="Putative Replication Associated Protein"
                     /translation="MSKESPCIGYDLSYNKIRVDGVVLTHLVIGKALLKVAKKFVFQL
                     EKGEVKGIEHYQIRCHYFSKTTKKHALDLFMEAIGLDSKDYKNAPHISITTVGVHGHK
                     NFNYVMKLDTHVEGPWTDKDFVSDILIEPEYIQKRFRHNPLDTLTLLQKDIIFNCSDY
                     NDRIIDIIYDKNGNNGKSYLSEVLTTFKNACTLPTTTNMKELIQYAYGYCNNKIVNKA
                     KDCREISPFIMDMPRAIEAYEIRTIIAALESIKKGRLTDLRYVNKEVIIEPPRIWVFC
                     NTVPPLEYLSKDRMKIWDIKEIDGVNRLVPYIPLKQVIKKLV*"
     CDS             1008..2447
                     /codon_start=1
                     /product="Putative CruV Capsid Protein"
                     /translation="MSAKTYTRKTYVPKKRTYAKKRYVPKSRPYTNYVRGASSVSKRF
                     VNNVSAVKADPRLSSLIKMSKNYISGMGDYDVSGITHNSILGKLSPIVPEFTNHNSGG
                     TIRICHREFIQDLKSSILFQANNFNINPADPKTFPYLSRIASQFEQYRLDGIVFEFKS
                     GSSDALNSINTALGYVIGAVEYNSLSAPFANKLQMENAIFASSTKPSLSMITAVECAP
                     DQTTIPLLYTRVDDAILAPSDLRLYDLGRFTIATVGMQQADVNIGEIWVSYSCSLLKP
                     RLSLIPSIPSAVPLWVANFTDIALYNNMLGDFSLINIPTFDNMGVTVDYSTVGTVTTN
                     RFNFPAGTTGNFILSYSVNGNDAPDPLSNTRPLFSDPFTASSNITSVSAFPANGQGGL
                     SEWTQTGLTLVGNNQISVSKNYQMQYCFSIDDVNLPSYLDMVVDLSYIVDPVAPGMPG
                     AIVFTGLVGFPSLTAGTCYITQVNARILG*"
ORIGIN
        1 atgagtaaag aaagtccttg tattggttat gatttatctt ataacaaaat ccgagtggat
       61 ggtgttgtat taacacattt ggttatagga aaggctttat tgaaagttgc aaaaaaattt
      121 gtttttcaac ttgaaaaggg tgaagtgaaa ggtattgaac attatcaaat tagatgtcat
      181 tactttagta aaacaactaa aaagcatgca ttagatttat ttatggaagc gataggttta
      241 gattcaaaag attataaaaa tgcgccacat ataagtataa caactgttgg tgttcatggt
      301 cataagaatt ttaattatgt catgaaatta gatacacatg tggaaggacc ttggactgat
      361 aaagattttg ttagtgatat tttaattgaa cctgaatata tacaaaaacg ttttagacat
      421 aatccgttag atacattaac acttttacaa aaagatatta tctttaattg ttctgattat
      481 aatgatagga ttattgatat tatatatgat aagaatggta ataatggtaa aagttattta
      541 tctgaggttt taacaacttt taaaaatgca tgtactcttc ctacaacaac aaatatgaaa
      601 gaacttatac aatatgctta tgggtattgt aataataaga ttgtgaataa ggctaaagat
      661 tgtcgtgaaa ttagtccttt tataatggat atgccacgtg ctattgaggc gtatgaaata
      721 agaacaatta ttgctgcttt agagtcaatt aaaaaaggtc gtttgactga tttaagatat
      781 gttaataaag aggttattat agaaccgcca cggatttggg tattttgtaa tactgtgccg
      841 cctttagagt atttatctaa ggataggatg aaaatttggg atataaaaga gattgatggt
      901 gtcaatagat tagtgcctta tatcccttta aaacaagtaa ttaaaaaatt agtttaaatt
      961 tattttataa aatatagaaa aaaatatttt ctttatttta atttataatg tctgcaaaaa
     1021 cttatacaag aaaaacttat gttccaaaaa agagaacata tgcaaaaaag cgatatgtgc
     1081 ctaaaagtcg cccttatact aattatgtta ggggggcttc ttctgtttca aaacgttttg
     1141 ttaataatgt atctgcggtt aaagcggatc cacgattatc aagtttaatc aaaatgtcaa
     1201 aaaattatat atctggaatg ggtgattacg atgttagtgg tatcactcat aattcaattt
     1261 tgggtaagtt atctccgatt gttcctgaat ttactaatca taattctggt ggaactatac
     1321 gaatttgcca ccgtgaattt attcaagatc ttaaaagttc aatactattt caagcgaata
     1381 attttaatat taatcctgcg gatccaaaaa cttttccata tttatctcgt atagcttcac
     1441 aatttgaaca atatcgtttg gatggtattg tttttgagtt taaaagtggt tcaagtgatg
     1501 ctttaaatag cattaatact gctttaggtt atgttattgg tgcggttgaa tataatagtt
     1561 tatctgcgcc ttttgctaat aaattacaaa tggaaaatgc tatctttgcg agttcaacaa
     1621 aaccaagttt aagcatgatt actgcggttg aatgtgcccc tgatcaaaca actattccgt
     1681 tattatatac gcgtgtggat gatgctatat tagcgccttc tgatttacgt ttgtatgatt
     1741 taggacgatt tacgattgcg acggttggga tgcaacaagc ggatgtaaat attggtgaaa
     1801 tttgggttag ttattcatgt agtctcttaa agccccgttt gtctttaatt ccatcaattc
     1861 caagtgctgt gcctttgtgg gttgcgaatt ttacggatat tgcgttatat aacaatatgt
     1921 taggtgattt tagtcttata aatattccaa catttgataa tatgggggtt actgttgatt
     1981 attctacggt gggtactgtt acaactaatc gttttaattt tcctgctggg acaactggta
     2041 attttatatt aagttattct gtgaatggta atgatgctcc tgatccatta agtaatacac
     2101 gccctttgtt tagtgatcca tttactgcat cttctaatat tacctctgtt tctgcatttc
     2161 ctgctaatgg tcaaggtggt ttatctgaat ggacacaaac tggtttaact ttagttggta
     2221 ataaccaaat tagcgtttca aaaaattatc aaatgcaata ctgttttagt attgatgatg
     2281 ttaatcttcc atcttattta gatatggttg ttgatttgtc atatattgtt gatcctgtcg
     2341 cccctggtat gcctggtgcc atcgtattta ctggactcgt aggttttccc tcattaactg
     2401 ctggtacatg ttatattaca caagttaatg cccgtatttt aggttaaaca taacctttta
     2461 ctatataaat catctttagt cgcgggctcg caacgcgagt gttagcgatg tgtgttcttt
     2521 ttaaattgat cctatattat tatataggat taaaataaga tgctattaat atgttattta
     2581 ttgatagttt aatgcttatt ataagcttaa tttatgcaat atttgactct tataacgcac
     2641 gggtaggcgt aagcccgcgg gctcgcgtaa gcgagaaccg aagaaataat atagctataa
     2701 tattatgtga tttcctaata attatttatt tagaaatatt tctaaataga ttccaattaa
     2761 tatattagat atatatcctt tttcttatat ttccaaatag ttaaactata tatataagga
     2821 ttattttaat aaacattttt taataacgaa tatgtaatat gagttatgta gcgacaacga
     2881 atgaaatgag tgcctaaaaa atgtcttgcc ttatgtttat cttatggctt ttttaaaaat
     2941 attgtcgcgt aagcggcgct ttagcgcttt ttagggatac aatatgagcg agatgtgtgt
     3001 atgaaataca cacatatcac tcggggttaa gcgacggctg tcctaaggta tgaagcgtaa
     3061 gcgaatacct taggactaag ccgtataagc gacccctgtc ttatatatgc cttgtgcctt
     3121 tttaaaaata tatatatgta gagtgatgaa atcacgagaa catatgcctt gtgccttttt
     3181 aaaaatatat atatgtctta tgccttttta aaaatatatt ttaatgtata atttttggtt
     3241 ggtaaaaaaa atataaaata tatagaattt cttatttttc taattgagtg cgtttaaatt
     3301 ggaaaaatta attatctata gtatagtata
//
LOCUS       CruV-217                3333 bp    DNA     circular     24-FEB-2020
DEFINITION  Crucivirus-217 isolate 001577_virusbaton.
ACCESSION   CruV-217
KEYWORDS    .
SOURCE      Crucivirus-217
  ORGANISM  Crucivirus-217
            Unclassified.
REFERENCE   1  (bases 1 to 3333)
  AUTHORS   de la Higuera,I., Kasun,G.W., Torrance,E.L., Pratt,A.A.,
            Maluenda,A., Colombet,J., Bisseux,M., Ravet,V., Dayaram,A.,
            Stainton,D., Kraberger,S., Zawar-Reza,P., Goldstien,S., Briskie,J.,
            White,R., Taylor,H., Gomez,C., Ainley,D.G., Harding,J.S.,
            Fontenele,R.S., Schreck,J., Ribeiro,S.G., Oswald,S.A., Arnold,J.M.,
            Varsani,A., Enault,F. and Stedman,K.M.
  TITLE     Direct Submission
  JOURNAL   Submitted (24-FEB-2020) Biology, Portland State University, 1719 SW
            10th Ave, Portland, Oregon 97201, USA
COMMENT     Bankit Comment: ALT EMAIL:ide@pdx.edu
            Bankit Comment: TOTAL # OF SEQS:451
            Bankit Comment: GAP: est. length - alignment

            ##Assembly-Data-START##
            Assembly Method       :: MetaSPAdes v. 3.12; IDBA-UD v. 1.1.1;
                                     MEGAHIT v. 1.0.3
            Sequencing Technology :: 454; Illumina
            ##Assembly-Data-END##
FEATURES             Location/Qualifiers
     source          1..3333
                     /organism="Crucivirus-217"
                     /mol_type="genomic DNA"
                     /isolate="001577_virusbaton"
                     /isolation_source="Water"
                     /country="France"
                     /collection_date=2013
     CDS             1..1158
                     /codon_start=1
                     /product="Putative Replication Associated Protein"
                     /translation="MPASKAEKSRKWCFTLNNPKGGAEIYGDDWVVEYLIVGHEIAPT
                     TGTPHHQGYVRFKNPIGLGGKGPNPRGGVRKLLPTAAWFACKGTEEDNYDYCSKDRVT
                     VIEYGEREAPDPNADPDEPAKGQGKRNDIRYARRLVKAGAGMAEICDKIDSYQGIRAA
                     ETFLKYMEIKRSWIPEVIWIYGPTGVGKTHLAHELCETEPWTSLDTGRWFEGYDAHED
                     VIFDDIRGDFCKFHVLLRLLDRYEYRIEVKGASRQFLARRIFVTTCKPPHELYKDRAG
                     EDLMQLGRRITTIIWMEKTGTSWQAPGRDIGRLPTVPYLVPSGSDLEATHPALEPEQK
                     SGVILANGLPASKCVGQNSLPSPCSGPDLRDDNVADEQELDDEFMSQFELD*"
     CDS             complement(1205..2533)
                     /codon_start=1
                     /product="Putative CruV Capsid Protein"
                     /translation="MPRTAAAKKPAAKARRAPAKKAYAPRARAAPRAAPARQPRSKFD
                     YSRAGGGIGGILGSVLGGPGGGAIGSALGTLGGAALTKIMGTGDYQVSNIGRVSHNSI
                     VLSNASNIPMFGGGKVAVNIKHREFLGDVISSSSAGAFSIKSYSINPGLPQSFPWLAN
                     VCGASFQQYRINGAVYEFRSMSSDALNSTNTALGSVVMATDYDSKDTVFGTKQQMENT
                     EFGVSCKPSCNMLHALECARNQTSVSELYIRAGAVPSGADVRLYDLGRFSIATVGCQG
                     TSVNLGELWVSYDIDLFKAIEQPPGYLVLSAEYSNTPAGGSFDTHPISTTALGYGDNI
                     GLTFTSNKIQWPYDIQNGSAWMVAMTWAGNSTACTPGAVTAGNGMSVTDNFNQAANST
                     SPKLITVVFLLYDGTGTPAALPYVNFANGTYPATAVVSEINVTQVNPNIL*"
     CDS             2656..2958
                     /codon_start=1
                     /product="putative ORF"
                     /translation="MLSADIPAPFGGGHPVRILSYFRTLRASRLARACVSHYAASARP
                     GRNNIFAREYIRVATYANGPAPPMITGYVTSAIKMVYQSWCDMIWCARHWRHLQKL*"
ORIGIN
        1 atgcccgctt ctaaagcaga aaaatcacga aagtggtgct tcaccctcaa taatcccaaa
       61 ggcggagccg aaatttatgg agatgattgg gtagttgagt acctaatcgt cggacacgaa
      121 atcgcgccga ctaccggcac accccaccac caaggttacg ttcgattcaa gaaccctatc
      181 ggccttggcg gcaagggccc caacccacga ggtggggtac gcaagctgct accgacagct
      241 gcgtggtttg catgcaaagg taccgaagaa gataactacg actattgcag caaagatcgt
      301 gttactgtca tcgaatacgg agagcgcgaa gcgcctgacc ccaacgccga ccctgacgag
      361 ccggctaaag gacaaggcaa gcgcaacgac atacgatacg cccgccgcct tgtcaaagca
      421 ggtgccggaa tggcagagat ctgcgataaa atcgacagct atcaaggtat ccgcgcagca
      481 gaaaccttcc taaaatacat ggaaatcaag cgtagctgga ttcctgaagt gatatggatt
      541 tacggcccca ctggggtggg taaaacccat ctcgctcacg aactttgcga gaccgaaccc
      601 tggacgtccc ttgatacggg ccgatggttc gagggctatg acgcccacga agatgtaatc
      661 ttcgatgaca tccgcggaga tttctgcaaa ttccacgttc tacttcggct cctcgaccgt
      721 tacgagtatc gcatcgaagt caaaggcgcc tcgcgacaat ttcttgcgcg acgaatattt
      781 gtcactacgt gcaaaccgcc tcatgagctc tacaaagata gagctggcga ggatctaatg
      841 cagctaggcc gccgcatcac tacgatcatc tggatggaaa agacgggtac ctcgtggcaa
      901 gccccgggcc gcgacattgg ccgcttaccc actgttccat atttggttcc ctctggttcc
      961 gatttggagg ctacgcatcc ggcactggaa ccggaacaaa agtctggggt aatattagcg
     1021 aatggcctac ccgcatcaaa atgcgtaggc caaaattcgc ttccctcgcc atgctcgggc
     1081 ccagacttac gcgacgacaa tgtcgccgac gaacaagaac tcgatgatga atttatgtca
     1141 caattcgagc ttgattaacc gatcgtaacc gatcgcaaaa aacttttttc tcgtcctaac
     1201 ggactcaaag aatgttagga ttgacttgag tcacattgat ttcgctcacg accgcagtag
     1261 cgggataagt cccgttcgca aagttgacgt aaggcaaagc cgcaggagtg cccgtaccgt
     1321 catacagaag gaagaccaca gtaatcaact tcggagaagt tgagttcgcc gcctgattga
     1381 aattatcagt aactgacata ccatttcccg cagtaactgc accaggggtg cacgcggtcg
     1441 aattgccagc ccaagtcatg gcaaccatcc aggcggaccc attctgaata tcatagggcc
     1501 attgaatctt gttcgaggta aacgtcagac caatattatc gccatagcca agagcagtag
     1561 tgctgatagg atgagtatcg aaactaccgc cagcaggagt attcgaatac tcagcactca
     1621 gaacgagata accaggaggc tgttcgatcg ccttaaagag atcgatatca tagctcaccc
     1681 aaagttcgcc aagattaaca ctcgttcctt gacaaccaac ggttgcaatt gagaagcgac
     1741 cgagatcgta aagacgaaca tcggcgcccg aaggaacagc acccgcccga atatacaatt
     1801 cggaaacgga agtttgattc ctcgcacact caagagcgtg aagcatgttg caagaaggct
     1861 tgcaactcac cccgaactcg gtgttctcca tctgctgctt tgtcccaaag acagtatcct
     1921 tactatcgta atcagtagcc ataaccaccg acccaagggc tgtgtttgtc gaattcaatg
     1981 cgtccgaaga catacttcga aactcgtaca cagcaccgtt tatacggtac tgctggaaag
     2041 acgcaccgca cacattggca agccaaggga aagattgcgg caatcctgga ttgatcgaat
     2101 aactcttgat gctaaacgca ccagcagaag aagaactgat cacatcaccg agaaattcac
     2161 ggtgcttaat gttcacagcc acctttccac cgccgaacat aggaatgttc gaagcgttcg
     2221 aaagcacaat agagttgtgt gacacgcgtc caatattgga cacttgataa tcccccgtgc
     2281 ccatgatctt agtcaaggca gcacccccaa gggtacccaa agcggagcca atagcaccgc
     2341 cacccggacc gccaagaacg gatccaagta ttcctccaat cccgcccccg gccctcgaat
     2401 aatcgaattt agaccgaggc tgacgggctg gagccgcacg cggagccgca cgcgcacgag
     2461 gcgcatacgc ctttttagcc ggagcccgac gcgctttcgc ggccggcttc ttagcggcag
     2521 cagtacgtgg cataatgcaa cggacagcaa gataatacca taaataattt tctaaaatcg
     2581 aaaaataatt gtcgatgtca gccactggtg gggcactacg cccgctggac ggtcgtataa
     2641 tccagaaaat tattattgtt gagtgcggat atccctgccc cctttggggg cgggcacccc
     2701 gtccgcatcc ttagttattt ccggaccctt cgggcttccc gtctagcgcg ggcgtgcgtg
     2761 tcacactacg cggcttccgc gcgacccggc cggaataata tttttgcgcg cgaatatatc
     2821 cgcgtcgcca catacgcaaa cggacctgcg cccccgatga taacagggta cgtcacgtcc
     2881 gccatcaaaa tggtatacca atcctggtgc gatatgatat ggtgcgcccg acactggcga
     2941 cacctccaaa agctatagca tttggagata gctccgaaag ctatattata tagctccgaa
     3001 agctatcaca tttggagatt actccaaaag ctatctccga aagctaccaa aaaatttatt
     3061 aattatatac gggccgtaac atacggggcc cggggaccct gtgccatacc ggcaccaccc
     3121 catatatttt ttttctcacc cgattatccg cgcatatacg cacgctgacg ggagggggtc
     3181 acccccttac ccctatatat atttattttt cggacaagcc aaggggacac cccccctcac
     3241 cccctatata tttatttttt cgcggccgca tttcgccgcc gctccatacc cccgagctac
     3301 catatacgga acaagctcgt gtgcactttc gtc
//
LOCUS       CruV-218                3344 bp    DNA     circular     24-FEB-2020
DEFINITION  Crucivirus-218 isolate 001565_virusbaton.
ACCESSION   CruV-218
KEYWORDS    .
SOURCE      Crucivirus-218
  ORGANISM  Crucivirus-218
            Unclassified.
REFERENCE   1  (bases 1 to 3344)
  AUTHORS   de la Higuera,I., Kasun,G.W., Torrance,E.L., Pratt,A.A.,
            Maluenda,A., Colombet,J., Bisseux,M., Ravet,V., Dayaram,A.,
            Stainton,D., Kraberger,S., Zawar-Reza,P., Goldstien,S., Briskie,J.,
            White,R., Taylor,H., Gomez,C., Ainley,D.G., Harding,J.S.,
            Fontenele,R.S., Schreck,J., Ribeiro,S.G., Oswald,S.A., Arnold,J.M.,
            Varsani,A., Enault,F. and Stedman,K.M.
  TITLE     Direct Submission
  JOURNAL   Submitted (24-FEB-2020) Biology, Portland State University, 1719 SW
            10th Ave, Portland, Oregon 97201, USA
COMMENT     Bankit Comment: ALT EMAIL:ide@pdx.edu
            Bankit Comment: TOTAL # OF SEQS:451
            Bankit Comment: GAP: est. length - alignment

            ##Assembly-Data-START##
            Assembly Method       :: MetaSPAdes v. 3.12; IDBA-UD v. 1.1.1;
                                     MEGAHIT v. 1.0.3
            Sequencing Technology :: 454; Illumina
            ##Assembly-Data-END##
FEATURES             Location/Qualifiers
     source          1..3344
                     /organism="Crucivirus-218"
                     /mol_type="genomic DNA"
                     /isolate="001565_virusbaton"
                     /isolation_source="Water"
                     /country="France"
                     /collection_date=2013
     misc_feature    join(3335..3344,1..17)
                     /note="stem-loop"
     misc_feature    1..9
                     /note="nonanucleotide"
     CDS             complement(224..595)
                     /codon_start=1
                     /product="putative ORF"
                     /translation="MEDYGWVSDFEPLDQATDLIAEAFLILHEYKKELEKRDTFPKLK
                     NIYNKIGDILKDLDVYDPNRDLSDAFLIKIKEIDGEPNLTKSQKQNLIMDKFEKSFGN
                     NDIETHLDKMCLEDNENEMQS*"
     CDS             643..2022
                     /codon_start=1
                     /product="Putative CruV Capsid Protein"
                     /translation="MPQRRRPAPAYPSLSNARRQQIRRAATRAASAPAPAPAPASTKR
                     RIPKYGQYLGALAGTAVAPFLAPYAPMLAAGAPAAGAYLGNQAQKLVKYVTGWGDYKI
                     GVNSLVKSAEQVPEFRNTGSSRCTMIRHREFIGDVVGGALVSSASAFTINTLPVQPGN
                     STTFPWLSKIAQNYEQYRFHGLLFEFKTTSGNTSTTPMLGTLIMASQYSSLSPIFSNK
                     QEMENYEFAGSTVPSRNLIHPLECDPKQTQCNGIFNVQSSSNAYGGDKRLYDLADFSI
                     ATIGLPQASEVVGELWVTYDICLMKPRLNASGGSNADHWYGTGIGVNNSTNWLGTDFQ
                     LTINSDAFTSSDNVSKIFFNKNFYGIVSVTYAVISATTTTPVCPGITGTNGITDYNML
                     HVINGFNTQASYNSIFNNSQTAFYTTFWFKVQPLSNGTIPTLTFSTGTTGTLSSIDVI
                     IVQQPSDFS*"
     CDS             complement(2069..3169)
                     /codon_start=1
                     /product="Putative Replication Associated Protein"
                     /translation="MSSVWSDIKQVRRSPKYEKINIFVPFDEKEKDNGKRTRRFCFTI
                     NNYTDDDWTDIDELKVRYLCYECEIGESGTPHIQGYVIFHDPHSIKNVAKMLKRAAIF
                     VAKGTTEQNRTYCSKEDNGTFEEFGEIPKQGQRNDKLKMIDTINMPEMQIRELFPYQA
                     DKLMDNKKKLAILRTPHQRYDSPRLTLYITGGARLGKSKLAHDLFKRENKEDIVFDKI
                     AANGRFIAGYTQDRKPLLWDEFREECLSLRDFLQITDPWYNNIQDIKNGSAKIDTDVI
                     VITSIIDPEHLYDKDKGKEDVLGQIMGRINVIDINKIYIPDPYSSDAVNKFIADRENL
                     KIKEFKNKIDKIYENQLSEEEKYITVTIETFD*"
ORIGIN
        1 caatattacc tttagctcaa ttattagtgc aataatcaca gattgttatt atttgcagac
       61 tccatcctta attttaagga ttgactaata atttagacaa attttagcat atatttactt
      121 ctgtacagat atgtacaatt tgtaaaaatc atcattttat tattattttt gagtatgagt
      181 aatattattt tataatttta ctacttctgt gcaaaatgtg caattatgat tgcatttcat
      241 tttcattatc ttctaaacac atcttatcta agtgagtttc gatatcatta tttccaaaag
      301 atttttcaaa tttgtccatg atcaaatttt gcttttgaga tttcgttaaa tttggttcac
      361 catcaatttc cttaatttta attaagaacg catcactaag atctctattt ggatcatata
      421 catctaaatc tttcaatata tctccgattt tattgtatat atttttaagt ttaggaaaag
      481 tatctctttt ctctaactct tttttgtatt cgtgtaggat taaaaaagct tcagcaatca
      541 gatcagttgc ttgatctaat ggttcgaagt cagacaccca tccgtagtct tccatatgtc
      601 ttttttagat atatggcaat ttttttatta tattaaataa aaatgccaca acgtagaaga
      661 ccagctcctg cgtatccatc gttgtctaat gcaagacgtc aacaaattag acgtgctgca
      721 actcgtgctg cttcagctcc agctccagct ccagctccag cttctaccaa acgtagaatt
      781 ccaaaatatg gtcaatattt aggagcattg gctggaaccg cagttgcacc attccttgcc
      841 ccatatgccc ctatgcttgc tgcaggtgct ccagctgcag gagcttattt ggggaatcaa
      901 gcccaaaaac tagttaaata tgtaactggt tggggagact acaagatagg agttaattct
      961 ttagttaaat ccgctgaaca agttccagag tttagaaata ctggatcttc tagatgcact
     1021 atgattcgtc atagagaatt tattggagat gttgtaggtg gtgctttagt atcatctgca
     1081 tcagctttca caattaatac tttgcctgtt caaccaggaa actcaactac gtttccatgg
     1141 ttatctaaaa ttgctcaaaa ttatgagcaa tacaggtttc acggactgtt atttgaattt
     1201 aaaacaactt ccggaaatac ttctacaact cctatgcttg gcacattgat aatggccagt
     1261 cagtactcat cactttcacc aattttttcc aacaaacagg aaatggaaaa ttatgaattt
     1321 gcaggctcga ctgtcccttc aagaaatcta attcatcctc ttgaatgtga tccaaaacaa
     1381 actcaatgta atggaatatt taatgttcaa tcatcttcaa atgcttatgg aggtgacaag
     1441 agactttatg atctcgcaga cttttctatt gcaacaatag gcctccctca agcttctgaa
     1501 gttgtcggtg aattatgggt tacttacgat atttgtctta tgaaacccag acttaatgca
     1561 tctggtggtt ctaatgctga tcattggtat ggaactggaa tcggagtcaa caattcaact
     1621 aattggttag gcacagattt tcaattaact attaattcag atgcatttac atcatctgat
     1681 aatgttagta aaatattctt taataagaat ttctacggaa ttgtttcagt tacatatgct
     1741 gtcatttcag ctactacaac tactccagta tgtccaggta ttacgggaac taacggaatc
     1801 acggattata acatgcttca cgttataaac ggttttaata ctcaggcttc gtataattct
     1861 atatttaata attcgcagac tgctttttat actacatttt ggtttaaagt tcaaccttta
     1921 tccaatggaa caatccctac ccttacgttc tctactggaa ctactggtac tctttcatcc
     1981 attgatgtta taattgttca acaaccatca gatttttctt aaatttaatt tattcaattt
     2041 tatgtattat tatattatta atacatattt aatcgaaggt ttcaatagtt actgtgatat
     2101 acttttcttc ttctgataat tgattttcat atattttatc aattttattt ttaaattctt
     2161 taatttttaa attttctcta tctgcaataa atttatttac tgcatcagaa gaatatggat
     2221 ctggaatata tattttgtta atatcaatca catttattct tcccattatt tgtccgagga
     2281 catcttcttt tcctttatct ttatcatata gatgttccgg atctatgatt gatgttataa
     2341 ctattacatc tgtatctatt tttgcagatc catttttgat atcttgtata ttattatacc
     2401 aaggatcagt aatttgaaga aaatctctaa gagataagca ttcttctcta aattcatccc
     2461 ataatagagg ttttctatct tgagtatatc cagcaataaa tctaccattt gcggctattt
     2521 tgtcaaaaac tatatcttct ttattttctc ttttaaagag atcatgcgct aatttagatt
     2581 tacctaatcg tgctcctcct gtaatgtata gagttaatct cggactatca tatctttgat
     2641 gtggtgttct aagaatagct aattttttct tattatccat tagtttatct gcttgataag
     2701 gaaataattc tcgtatttgc atttcaggca tattaatggt atcaatcatt ttcaatttat
     2761 catttcgttg accttgcttt ggaatttctc caaattcttc aaaagtacca ttatcttctt
     2821 tagaacaata agttctattt tgttctgtag ttcctttggc tacgaaaatt gcagcacgtt
     2881 ttaacatctt cgcaacattt ttgatactat gaggatcatg aaaaataaca tatccttgta
     2941 tgtgaggagt accagattca ccaatctcac actcataaca taagtatcta acttttaatt
     3001 catcaatgtc ggtccagtcg tcatctgtat aattattaat agtgaaacaa aaacgtctag
     3061 ttctttttcc attatctttt tccttttcat caaaagggac aaatatattt attttttcat
     3121 atttgggtga tcttctaact tgtttgatgt cactccacac actactcatt tgcacagaag
     3181 ttgatttctt tttaacttct gtacaaatta tttttatata aaaatttatt tgagtctatt
     3241 tttagactat caaatcaaaa taagatcaaa cgccccgtgt tatgatccat acatactaga
     3301 gcgagcgata gcgagcgata ttcgatgact agaaagctaa agtc
//
LOCUS       CruV-219                3344 bp    DNA     circular     24-FEB-2020
DEFINITION  Crucivirus-219 isolate 001566_virusbaton.
ACCESSION   CruV-219
KEYWORDS    .
SOURCE      Crucivirus-219
  ORGANISM  Crucivirus-219
            Unclassified.
REFERENCE   1  (bases 1 to 3344)
  AUTHORS   de la Higuera,I., Kasun,G.W., Torrance,E.L., Pratt,A.A.,
            Maluenda,A., Colombet,J., Bisseux,M., Ravet,V., Dayaram,A.,
            Stainton,D., Kraberger,S., Zawar-Reza,P., Goldstien,S., Briskie,J.,
            White,R., Taylor,H., Gomez,C., Ainley,D.G., Harding,J.S.,
            Fontenele,R.S., Schreck,J., Ribeiro,S.G., Oswald,S.A., Arnold,J.M.,
            Varsani,A., Enault,F. and Stedman,K.M.
  TITLE     Direct Submission
  JOURNAL   Submitted (24-FEB-2020) Biology, Portland State University, 1719 SW
            10th Ave, Portland, Oregon 97201, USA
COMMENT     Bankit Comment: ALT EMAIL:ide@pdx.edu
            Bankit Comment: TOTAL # OF SEQS:451
            Bankit Comment: GAP: est. length - alignment

            ##Assembly-Data-START##
            Assembly Method       :: MetaSPAdes v. 3.12; IDBA-UD v. 1.1.1;
                                     MEGAHIT v. 1.0.3
            Sequencing Technology :: 454; Illumina
            ##Assembly-Data-END##
FEATURES             Location/Qualifiers
     source          1..3344
                     /organism="Crucivirus-219"
                     /mol_type="genomic DNA"
                     /isolate="001566_virusbaton"
                     /isolation_source="Water"
                     /country="France"
                     /collection_date=2013
     misc_feature    join(3340..3344,1..14)
                     /note="stem-loop"
     misc_feature    1..9
                     /note="nonanucleotide"
     CDS             complement(25..1704)
                     /codon_start=1
                     /product="Putative CruV Capsid Protein"
                     /translation="MLSKSDYSKLSAAGKKKVKGSSSRTRLPGPRATRTRLRGKGDYW
                     SFAPERILKGSSPFAKVKSAGSGIGSQLGGMLGGAPGRALGGAAHSLFKKITGFGDYK
                     VASNTIMMGTDPPSFATSGRGTIVRHREYIGDVAGSIAFTTTSYPINAGINRSFPWLS
                     QIASNFEQYIVRGMVFEFKSTSADALNSTNTALGAVIMATEYNSVNPNFSSKAQAENH
                     EFCTSAKPSESFLHPIECARGETSISALYTRTGTVPSNTDQRMYDLGNFQIMTVGMQA
                     VAVIGELWVTYDVELLKPRLYDTAGFSFYEDHFRCSTVSATGNYFGTSQVLATGSNLG
                     ASVSGSTITFPADSAVNYYFVYYCCVGVSTILANGIALSLGTNVIGVTYFLNDAASVV
                     APAAGATSNLQTFKIKVVVGASTSITGNTITVTAGTLPTGIVGTDLVISSANPLVVTA
                     PRKSRFSSESDDESASEDEEDLLKQMKKMKKEKMMKELAEWKASKSDSKEEKKVESLS
                     LTPESDEDSDGLDMLIMRREQKLLARSCKTPAPGAGAPIEPPHPGKVSSKK*"
     CDS             complement(1939..3009)
                     /codon_start=1
                     /product="Putative Replication Associated Protein"
                     /translation="MCIQHMAAHIETKEEKKTPAKKLPSVDFSNGLYCVFTSWCSQKP
                     TWNPDKMKYLVFQREQCPETKKKHWQGYVEMKSSTRHLALKKMLEITDPEEDAETGIK
                     GPGGWKALIRRGSAIQAAQYCKKIETRINPETLPTEYGEKPVKTSQGHRSDLEAPALM
                     IREGKSIEEIADACPIAYMRYTNGITAYHQIHNKFVEPDPGITLRPWQEAVCQLIDLG
                     FKKRQILWIWSQKSGTGKSTMRDYIQFKYGNIRAMDGVWNMTELLYLYKKHDIIVFDL
                     PRKTPLNDSRQAILENLSDGGTRTSTKYQTVQKLLRAVIIVFANIPPPEADMPMRCEE
                     IWIDSDEDKAEYEKNREKPEED*"
ORIGIN
        1 tattattaat ttttgttagt atttttattt tttggatgag accttacctg ggtgaggcgg
       61 ctcaatagga gcacctgcac caggagcggg agttttacaa ctacgcgcta gtaatttttg
      121 ttctcgtcgc ataattaaca tatccagacc gtctgagtcc tcatcggatt ctggagttaa
      181 gcttagagat tcaactttct tttcttcttt agaatcagac ttggaagctt tccattcggc
      241 cagttccttc atcatttttt cttttttcat ctttttcatt tgtttcaaaa gatcttcttc
      301 atcttcgcta gccgattcat catcagactc agacgagaaa cgtgattttc gtggtgcagt
      361 tacaacaagt gggtttgctg aactaataac taaatcagtt ccaacgatgc ctgtaggcaa
      421 agttcctgcg gtaactgtaa tcgtattgcc ggtgatacta gtcgatgcac ctactacaac
      481 tttaatctta aaagtttgta aattgctagt ggcaccagct gctggagcaa caacgcttgc
      541 agcatcattt aagaaatatg ttacaccgat aacattagtg ccaagactta acgcgatacc
      601 attcgctaat atagttgaaa cacctacaca gcagtagtat acgaaataat aatttacggc
      661 actatcagct ggaaatgtaa ttgtgcttcc ggatactgat gcgccaaggt tagaacctgt
      721 cgctagaact tgtgatgtac caaagtaatt tccagtagct gaaacagtac tgcaccggaa
      781 gtgatcttca tagaatgaaa aaccggctgt atcatacaaa cgtggtttga gaagttcaac
      841 atcataagtg acccaaagct caccaatgac tgctacagct tgcataccaa cggtcataat
      901 ttggaagttt ccaagatcat acatgcgttg atctgtgttt gaaggcactg taccagtgcg
      961 agtataaagt gctgagatcg aagtttcgcc acgagcacat tcaataggat gcaaaaatga
     1021 ttctgatggc ttcgctgaag tacagaattc atgattttct gcttgagctt tgctactgaa
     1081 attaggatta actgagttat attcagtagc cataataacc gcaccgagcg cagtattcgt
     1141 agaattcaat gcgtccgctg acgtagactt gaactcaaac accataccac gaacaatata
     1201 ttgctcgaag ttggatgcga tttgagataa ccaaggaaaa gaacggttta tgcctgcgtt
     1261 aataggataa ctagtagtcg tgaatgcaat agagccggct acatcgccga tgtactcacg
     1321 atgacgaaca atagttccac gacctgatgt cgcgaaagat ggtgggtctg tgcccatcat
     1381 aatagtattg gaggctactt tgtagtcacc aaaaccagtt atttttttaa ataaactgtg
     1441 ggctgcgcct ccaagagcac gaccaggtgc tcctccgagc atacctccga gttgtgatcc
     1501 gataccggat cctgcgctct taactttagc gaaaggagat gaacccttca atatacgttc
     1561 gggagcgaac gaccaataat cacccttgcc acgtaaacgt gtacgggttg cacgagggcc
     1621 aggaagtcgc gttcgcgagc ttgagccctt aacttttttc ttacctgcag ctgaaagctt
     1681 agaataatca gattttgata gcatacccta tcaacaaaag ttttctaaat taaattaatt
     1741 aatttcaccc agggactatg ccgctgggtc cagtgcattg cttcgcgcgc actggttcaa
     1801 cgcaaaacac ataggttttt tgtgaaagtt tcacgaaacc gttgcacaac gtttccgttg
     1861 tggaagtggg tggtaataat agaacatctc acttctctca aaaaattgag agcgcgttcg
     1921 cttttcttag cacccacttt aatcctcttc aggtttttct ctattttttt catactcagc
     1981 tttgtcttcg tctgaatcta tccatatttc ttcacacctc atcggcatat ctgcctctgg
     2041 tggtggaata tttgcaaata caataataac tgctcgcaat aatttttgta cagtttgata
     2101 tttagtcgat gtacgtgtac cgccatcaga caaattttca agtattgctt gacgactgtc
     2161 atttaagggc gttttacgag gcaaatcgaa tacgattatg tcatgttttt tatacaaata
     2221 aagcagttct gtcatattcc atactccatc catagctcga atatttccat atttgaattg
     2281 aatataatca cgcattgtag atttacctgt tccagatttt tgagaccaaa tccatagaat
     2341 ttgtcgtttt ttaaatccta gatcaattaa ttgacatact gcttcttgcc aaggtcgaag
     2401 agtaattcct ggatcaggtt ctacaaattt gttatgaatt tgatggtaag ctgtaatacc
     2461 atttgtatat cgcatatatg ctataggaca tgcgtctgca atttcttcta ttgattttcc
     2521 ttcacgaatc ataagtgcgg gagcttcaag atcggaacga tgaccttgtg aagtcttcac
     2581 cggtttttct ccatattctg ttggaagtgt ttctggatta atacgtgttt caattttttt
     2641 acaatattgt gctgcttgaa tagctgaacc gcgacgtatt aatgctttcc agccgcctgg
     2701 gcctttgatg ccggtttctg catcttcttc tggatctgtt atttctaaca tttttttgag
     2761 agctagatgc ctagtagatg atttcatttc tacatagcct tgccaatgct tcttcttagt
     2821 ttcagggcat tgctctcgtt ggaacaccaa atatttcatt ttgtcaggat tccaagtagg
     2881 tttttgtgaa caccaagaag tgaaaacaca atatagacca tttgaaaaat ctacagaagg
     2941 tagttttttt gctggagtct ttttttcttc ttttgtctca atatgtgctg ccatgtgttg
     3001 tatacacata acacatactt tgtttaaata ataattaatt catctttttg aaaatgtggt
     3061 gcttcgcact gtttttcttt tgtgaacatt gcaacttcgc ttcgctcagt tggtgtgaat
     3121 gtggaaactt cgcttcgctc agtttcgccc ccttcggtgg ctaacccgcc cattcatcct
     3181 gctaatctat caagatatat ggtcatatgt ttattatacc gaaaactcgc tgcgctcggt
     3241 ttcggtgtag taggcataag atctcatata tcttaataga tctacagaat gactgtacga
     3301 tcttgccttc ctgattttgg ggacttgtag acatttataa aaaa
//
LOCUS       CruV-220                3344 bp    DNA     circular     24-FEB-2020
DEFINITION  Crucivirus-220 isolate 120_896_SvL1_3295.
ACCESSION   CruV-220
KEYWORDS    .
SOURCE      Crucivirus-220
  ORGANISM  Crucivirus-220
            Unclassified.
REFERENCE   1  (bases 1 to 3344)
  AUTHORS   de la Higuera,I., Kasun,G.W., Torrance,E.L., Pratt,A.A.,
            Maluenda,A., Colombet,J., Bisseux,M., Ravet,V., Dayaram,A.,
            Stainton,D., Kraberger,S., Zawar-Reza,P., Goldstien,S., Briskie,J.,
            White,R., Taylor,H., Gomez,C., Ainley,D.G., Harding,J.S.,
            Fontenele,R.S., Schreck,J., Ribeiro,S.G., Oswald,S.A., Arnold,J.M.,
            Varsani,A., Enault,F. and Stedman,K.M.
  TITLE     Direct Submission
  JOURNAL   Submitted (24-FEB-2020) Biology, Portland State University, 1719 SW
            10th Ave, Portland, Oregon 97201, USA
COMMENT     Bankit Comment: ALT EMAIL:ide@pdx.edu
            Bankit Comment: TOTAL # OF SEQS:451
            Bankit Comment: GAP: est. length - alignment

            ##Assembly-Data-START##
            Assembly Method       :: MetaSPAdes v. 3.12; IDBA-UD v. 1.1.1;
                                     MEGAHIT v. 1.0.3
            Sequencing Technology :: 454; Illumina
            ##Assembly-Data-END##
FEATURES             Location/Qualifiers
     source          1..3344
                     /organism="Crucivirus-220"
                     /mol_type="genomic DNA"
                     /isolate="120_896_SvL1_3295"
                     /isolation_source="Water"
                     /country="Norway"
                     /collection_date=2012
     CDS             complement(1..1101)
                     /codon_start=1
                     /product="Putative Replication Associated Protein"
                     /translation="MNGVVDNTPLDNNLVNKEITKSNPIDDKSKCRNWCFTINNPVTS
                     KEEWLEILKNKNVRYVVMGDEIGANGTPHIQGYVDFVNPHSFSGVTKVFNKLAHIEKR
                     KGSWKQASDYCKEDGVFVEFGKPPRQGARGDIDDVKDCIMENMGIVEMIDEGYINNHQ
                     QLRFAEGLKKYKELKRDPSNPPHILWFWGPTGTGKTRTAMELYPNAWLSGKTLKWWEN
                     YDGQKHVIIDDFRKDFCTFHELLRILDRYPFRVECKGGSRELVAEVIVITCPYRPEEL
                     YEGRTSEDVCQLTRRITEIRKFNNIIINNEIGTEQRSAGNTISAESPAIIINPQINNN
                     AGPSVPPPTFIDVVRVPRVPTPRRPGYEFNVY*"
     misc_feature    140..165
                     /note="stem-loop"
     misc_feature    150..158
                     /note="nonanucleotide"
     CDS             1350..3083
                     /codon_start=1
                     /product="Putative CruV Capsid Protein"
                     /translation="MVYTAAQKKAYGVKMAKARANALAGKAKRASPKVVSGRGAYFKG
                     TAGMRTAKGFGDYDLGEGSSIGSHVGAWLGDKAQNLIKSVAGFGDYVKPSWNPKSNSL
                     LSMGQDPPIVKNSPEGNFIIRHREYLQDIITGTPGQFVVNSYKIQPGLPDTFPWMSIT
                     AQCFEQYQLRGMIFEFKSTSADSLNSTNTALGEVIMATEYDSKKPNFASKVDMQNHQY
                     AVAARQSSSMLHPIECAHPLSTLDVLYVRSGQVPEGADSRMYDFGNFQIATVGQQGSN
                     INIGELWVTYEVELLKARLPGEGNIVFSSAYYQSGSVCTSSNRTAAVSNSGTFLVQPD
                     NNLPILWSNNAMTFPNDVGNGTYQVTVEWVGTTTLISGVPVLEPEVGSGIIVGPDSGP
                     FGVMVGPGTGVNSERLCLVYFVYLGTPNPGVARTATFQGGVIPTNSQFNCVITSILTG
                     FNTISFTDIYNPPVFSVAAKKPLRQSPLQARSEEFAIKAEIERLTQLYEAKLARETLA
                     PLSDDEPSAYKPFLHGNYTPNELEAISKEITVKVDDDWHDMTESTAVNIQAIVEKARQ
                     KRLAKEQQHKQ*"
ORIGIN
        1 ttaataaaca ttaaactcat aaccagggcg ccgaggagtg ggaactctag gcaccctaac
       61 aacatcaatg aaagtaggag gaggaacaga aggtccagca ttattattaa tctgtggatt
      121 aataataata gctggagact cagcagatat agtattacct gctgacctct gttccgttcc
      181 tatttcatta tttataataa tattattaaa tttacgtatc tcagtaatgc gacgcgttaa
      241 ctgacatacg tcctcacttg tacgaccctc gtacaattct tcagggcgat agggacaagt
      301 tatgacaatg acttcggcta ctagctccct gctaccaccc ttacattcca ctctgaaggg
      361 atatctatca agaattcgta gtagctcatg gaatgtacaa aaatccttcc taaaatcatc
      421 aataattaca tgcttttggc catcgtaatt ttcccaccat tttaaggttt ttcctgatag
      481 ccacgcgtta gggtacaatt ccatagcggt acgtgttttg ccagtcccag tgggacccca
      541 aaaccaaaga atatgagggg gattcgaagg atcacgcttg agttctttgt atttcttcaa
      601 cccctcagca aagcgaagct gctgatggtt gttgatatac ccttcatcaa tcatttcaac
      661 aatacccata ttttccataa tacaatcttt aacatcatca atatcaccac gtgcgccctg
      721 ccgaggaggt ttaccaaatt caacaaacac accatcttcc ttacagtagt cagaagcttg
      781 cttccaacta cctttacgtt tttcaatatg tgccaactta ttaaaaactt tggtaacacc
      841 gctgaaggaa tgaggattga caaagtcaac gtatccttgg atatgcggag tcccattcgc
      901 accaatttca tcacccataa caacatatct aacattttta ttcttcaata tttctaacca
      961 ttcttcctta ctagtaacag gattgttaat agtaaaacac caattacgac atttagattt
     1021 atcatcaata ggattactct tagtaatttc tttgttgact aaattattat caagtggagt
     1081 attatctaca acaccattca ttacattaac actatctcta acacttttaa taagtttagg
     1141 gtcagggcga ataatgagac gagtcgattt agttgccatg gtatttatta ttattttgga
     1201 acggaacgga agtctataga ccacttaata gcactattaa acagtataaa gacttccaaa
     1261 ttaataatat tattgagata tcaataatag cataaaaaca accaaactta tataaacaca
     1321 ctcaacacac accatcaaaa caaacaataa tggtctacac cgcagcgcaa aagaaggctt
     1381 acggagtcaa gatggccaag gccagagcaa acgcattggc tggaaaagct aaacgtgcat
     1441 cacccaaagt ggtctccggg aggggagcat attttaaagg aactgcagga atgagaactg
     1501 ctaagggatt tggtgattac gatttaggag agggctcctc aatcggcagt cacgttggcg
     1561 catggctagg agacaaagca caaaacctca tcaaatccgt agcaggcttt ggtgattacg
     1621 ttaagccatc gtggaatcct aaaagcaact ctcttttgtc gatgggccag gatccaccta
     1681 ttgtaaagaa ctcaccagaa ggtaatttta ttatccgtca cagggagtat ttacaagaca
     1741 ttatcactgg caccccgggc caattcgtgg taaacagtta taagattcaa ccaggacttc
     1801 cggacacatt tccttggatg tcaatcactg cacagtgttt cgaacaatac caactcagag
     1861 gcatgatatt tgagtttaaa tcgacatcgg cagattctct caacagtacc aacaccgccc
     1921 taggtgaggt tatcatggcc actgagtacg attcgaagaa acctaatttt gcttccaaag
     1981 tggatatgca aaatcatcaa tatgctgtag cagctcgcca gagttcctca atgttgcatc
     2041 caattgaatg tgcacaccca ctcagtacat tagacgtgct ctatgtacgc agtggacaag
     2101 ttccggaagg tgcagacagt cgcatgtacg attttggtaa ttttcaaatc gctaccgttg
     2161 gccaacaagg ctcaaacatt aatattggcg aactttgggt aacatacgaa gtagaattgc
     2221 tgaaggcaag gttgcccgga gaaggtaata tagtctttag tagtgcatac taccaaagtg
     2281 gtagcgtttg tacctcatca aatcgtaccg cggcagtgtc aaactctggt actttcttgg
     2341 tacaaccaga taataactta cctatacttt ggtcaaataa tgcaatgaca tttcccaatg
     2401 atgtaggtaa tggtacttat caagtaacag tagaatgggt aggaactact actcttataa
     2461 gtggtgtacc cgtattggaa ccggaggttg gttcaggcat tattgtaggg ccggattctg
     2521 gaccgtttgg tgtgatggtg gggccaggta ctggcgtcaa ctcagagcga ttatgtcttg
     2581 tttattttgt ttatcttggt actcctaacc caggagtagc tcgcactgct acatttcaag
     2641 gaggtgtaat ccctactaac tcccagttta attgtgtaat tacttccata ttgactggat
     2701 ttaatactat tagttttaca gacatataca atccacctgt attttctgta gctgcgaaga
     2761 agcccctaag acaatcacct cttcaagctc gctcagagga gtttgctatt aaggcagaaa
     2821 ttgaaagatt aacccagcta tatgaagcta aattagcacg agaaacactt gcgccattgt
     2881 cagacgatga gccaagtgct tataaaccat tcctacatgg gaattacact cctaacgaat
     2941 tagaggcaat ttctaaagag attactgtca aagtagatga cgactggcat gatatgaccg
     3001 agtcaactgc tgtaaacatt caagctattg tagaaaaagc ccgacagaag agacttgcca
     3061 aagaacaaca acacaaacaa taataatcga taacaacata tatatctaca catagatata
     3121 taaaacacaa ccaatctgta tatttactta tatgcaagtg cgcgctcacc ggacaaaagt
     3181 ggaacgggac attattggac tgtcaaaagc agcttgctgc tttttagaca ggccgataat
     3241 ggagcctcca cgcgcataca tagtagatat acatactaca tcaatcgggg gcttgtcccc
     3301 cttattaata taataagtac attattatat tactaacaac aaca
//
LOCUS       CruV-221                3350 bp    DNA     circular     24-FEB-2020
DEFINITION  Crucivirus-221 isolate 120_1405_IR2_3297.
ACCESSION   CruV-221
KEYWORDS    .
SOURCE      Crucivirus-221
  ORGANISM  Crucivirus-221
            Unclassified.
REFERENCE   1  (bases 1 to 3350)
  AUTHORS   de la Higuera,I., Kasun,G.W., Torrance,E.L., Pratt,A.A.,
            Maluenda,A., Colombet,J., Bisseux,M., Ravet,V., Dayaram,A.,
            Stainton,D., Kraberger,S., Zawar-Reza,P., Goldstien,S., Briskie,J.,
            White,R., Taylor,H., Gomez,C., Ainley,D.G., Harding,J.S.,
            Fontenele,R.S., Schreck,J., Ribeiro,S.G., Oswald,S.A., Arnold,J.M.,
            Varsani,A., Enault,F. and Stedman,K.M.
  TITLE     Direct Submission
  JOURNAL   Submitted (24-FEB-2020) Biology, Portland State University, 1719 SW
            10th Ave, Portland, Oregon 97201, USA
COMMENT     Bankit Comment: ALT EMAIL:ide@pdx.edu
            Bankit Comment: TOTAL # OF SEQS:451
            Bankit Comment: GAP: est. length - alignment

            ##Assembly-Data-START##
            Assembly Method       :: MetaSPAdes v. 3.12; IDBA-UD v. 1.1.1;
                                     MEGAHIT v. 1.0.3
            Sequencing Technology :: 454; Illumina
            ##Assembly-Data-END##
FEATURES             Location/Qualifiers
     source          1..3350
                     /organism="Crucivirus-221"
                     /mol_type="genomic DNA"
                     /isolate="120_1405_IR2_3297"
                     /isolation_source="Water"
                     /country="Norway"
                     /collection_date=2011
     misc_feature    join(3338..3350,1..19)
                     /note="stem-loop"
     misc_feature    1..9
                     /note="nonanucleotide"
     CDS             8..1342
                     /codon_start=1
                     /product="Putative CruV Capsid Protein"
                     /translation="MPRKRKSTARRASARKVSFKKSVRRSDRSKASNVTGQGNYKYTK
                     NVGPFERLGTYLGNKAGQMVGNVTGMGDYALHGTEVLSANPPRMFTDKEGTYIEHREY
                     LGDLISSSSASTFKVQRFGICPSDSNSFPWLSGIAQPNYQQYKFEQLIFEYKSYSGDA
                     ITGTNTALGAVFACINYDYSDPAVSSRSEVENTDWSNSCVPSQSMLIPVECKPQLTGL
                     NGGLLYVVNGNNVPVGNDPKTYYLGNLFIGTTGCQGTSVLLGSIFVNYRIRLYKPLMT
                     RPLSNALIYTSSRTDADGNFLWGEAEATSVYNADSIGVSIGGTNGAVLTLDQSRLQVG
                     QRYVLVHTITGSSTASVIYGTVTMSSGISGYTYFTKAAPYDANSYGSPLAAATSTSLS
                     MVIVFVVSNNTTNQTITLSGSPVYPTNNLGTIMIAQICGVPIENIGDYDPSV*"
     CDS             2163..3188
                     /codon_start=1
                     /product="Putative Replication Associated Protein"
                     /translation="MENNSFIGITESVNDESQRRIKLIREDVSGTGVRVKFCDLDTLQ
                     NEGSSSICAVWDFRCNEDMYDAETLWQFMKSNCKKYIFQKEKSDSGYIHWQGRFSLIK
                     KRNKSSLMKLMNVVPNYLMPTAKVNHQDLFFYCMKEDTRIGETYMDEYHRKLLGLEEV
                     FMPLQYQDIKPYKWQQLVLDSADQFDKRCIDLIFDPTGGKGKSTIAAIAEIKYKAIDM
                     PIMNDYRDLVSLACNICMDTQNRSPKLMFFDMPRSMNKSSLDGFYSAIEQIKKGKLYD
                     VRYHYKAYWINSPRVWVFSNELPDVNLLSRDRWRIWQINDKNELVPYKDNSDSYIKKK
                     KDPKPKA*"
ORIGIN
        1 gattattatg ccaagaaaac gtaagtctac agctcgaaga gctagtgcaa gaaaagtttc
       61 atttaaaaag tctgttcgta gatctgatag atctaaagct tcaaatgtca ccggacaagg
      121 aaattataag tatacgaaaa atgtaggacc ttttgagaga ctagggactt atttaggaaa
      181 caaagctggt cagatggtag gtaatgttac agggatgggg gattatgctc ttcacggcac
      241 tgaagtatta tcggcgaatc caccacgtat gtttactgat aaagaaggca cgtacataga
      301 acatagagag tatctcgggg atctgatcag ttcttcttcg gcctctacat ttaaagtaca
      361 gcgcttcggg atttgtccgt cagacagcaa tagttttcct tggttgagtg gaattgctca
      421 gccgaactac caacagtata aatttgaaca gctgattttt gagtataaaa gttactcagg
      481 tgacgcaatc acagggacaa atacagcgct tggtgccgtt ttcgcctgta taaattatga
      541 ttattcagat cccgcggtgt cgtctcgatc agaagtcgaa aacactgatt ggtcaaattc
      601 ttgcgttcca tcgcaatcaa tgttaattcc tgtggaatgc aaacctcaac ttacgggttt
      661 gaatggagga cttttgtacg ttgtaaatgg taataatgta ccggttggga atgatcctaa
      721 aacttattat ttaggtaatt tattcatagg tacaacagga tgtcagggaa cgtcagtcct
      781 attaggttca attttcgtga attatcgtat acggctttat aagcccttaa tgacacgtcc
      841 attgtctaat gctttgatat atacttcatc gcgtaccgat gccgatggta atttcttgtg
      901 gggtgaagcg gaagctacta gcgtatataa tgctgattcc attggtgtat caattggtgg
      961 aaccaatggt gcagtgttaa ctctagatca atcaagatta caggtcggac aaaggtacgt
     1021 gttagtacat acgattacag gctcaagtac agcaagtgta atttatggga cggtgacaat
     1081 gtcatctggt atatccggtt atacttattt tactaaggct gcaccatacg acgctaattc
     1141 gtatggcagc ccactcgctg cagccacttc tacatcacta agtatggtaa ttgtatttgt
     1201 agtaagtaac aatactacaa atcaaacaat tactttatct ggttctcctg tatatccaac
     1261 aaataatttg ggtactataa tgattgcgca gatatgtggt gttccaatag aaaatattgg
     1321 tgattatgat ccttcagtct aaaactaatt cttaatgcct taaattatta tttcatttta
     1381 ttaatgatat aatatatata tgcagtcggc agacccgaaa atcagcgaag cagatttatc
     1441 ggctggaggc tagaggagcg aggacttgac gagaagcgga cggtaggagc atcgcagccc
     1501 gaggcgtagc cgaagggtcc gagtctccgg ctgacgtaaa gccgccatat tatacggtat
     1561 acctttgagc gattattatg agcgattatt ataagcgatt attaagagcg attaaaaaac
     1621 aaaattattt aaggtggtaa ttattttttt aagcttaaaa caaaattctt taagtgtatt
     1681 tatcatttaa agagaaagca ccgcgatttt tatctttaag caattttaag tgtatttaga
     1741 aatttactta aaattagatt aaattgtaaa atcgtggtgc ccctgcagtg gcaaattatt
     1801 ccaatcattt ccagttataa taaaaaaaaa tgtattacag cttaattttt attccaacaa
     1861 tccaacaatg ttaaggtatg tttataggat ataatattat aatggaatga taatgaaatg
     1921 ttaaaatgat atgtatggca ttaaggggtt tttccaggga gcgtagcgag cgcccttaat
     1981 gccagagtgc gattatttga ttaatttctt atttcaagtc acatcaaatt caacatcaaa
     2041 agcatcacaa catctatttt ttgattcgtt ttatttttaa ttttatttga ttttaattgt
     2101 gaatttaatt aaaaaaacgc gcgataattt tgtaaaaatt tatttccaag tatagattat
     2161 aaatggaaaa taattctttt attggaatta ctgagagtgt caatgatgaa agtcaaagac
     2221 gtatcaagtt gatacgcgaa gatgtttcag gaacaggtgt aagggtcaaa ttttgtgacc
     2281 ttgatacttt acaaaatgaa ggttcttcat caatatgtgc cgtttgggac tttagatgca
     2341 atgaggatat gtatgatgca gaaacgttat ggcaatttat gaaatcaaat tgcaaaaagt
     2401 acatttttca gaaggaaaag agtgatagtg gatatattca ctggcagggt cgattttctc
     2461 ttatcaaaaa aaggaataag tcttcattga tgaagcttat gaatgttgta cccaattatt
     2521 taatgccaac tgcaaaggtt aatcatcagg atctattttt ctattgtatg aaggaagata
     2581 ctcgaatagg tgagacgtat atggatgaat accatagaaa gttattaggt cttgaggaag
     2641 tttttatgcc tttgcagtat caagatataa agccatataa atggcagcag cttgttttag
     2701 attctgctga tcaatttgat aagcgatgta ttgatttaat ttttgatccg acaggaggaa
     2761 agggtaaatc gaccatagct gccatagctg aaataaaata caaagctatt gatatgccta
     2821 ttatgaatga ttaccgtgat cttgttagtt tagcctgtaa tatttgtatg gatacgcaaa
     2881 ataggagtcc taagcttatg ttttttgata tgcctcgttc aatgaataaa tcgtccttgg
     2941 atggttttta cagtgcaata gagcagatta aaaaaggtaa gttgtacgac gtgcgttatc
     3001 attacaaagc ttattggatt aattcaccaa gagtatgggt attctctaat gagttacctg
     3061 atgtaaattt actatcacgt gatcgttgga gaatatggca gataaatgat aaaaatgaac
     3121 ttgtgccata taaagacaat tctgattctt acattaaaaa aaagaaagat cccaagccaa
     3181 aggcttaggg cgcttttaaa aaaaatgcgt aacaagctgg taggcttaaa gaaataatat
     3241 ttctttaagc ctatttttta aaagtgttga atttcttttt aagtgtaaaa ttatttcttt
     3301 aagccaagaa aacgcacgtc aattattata aaatttattt tcttagtata
//
LOCUS       CruV-222                3352 bp    DNA     circular     24-FEB-2020
DEFINITION  Crucivirus-222 isolate 001557_virusbaton.
ACCESSION   CruV-222
KEYWORDS    .
SOURCE      Crucivirus-222
  ORGANISM  Crucivirus-222
            Unclassified.
REFERENCE   1  (bases 1 to 3352)
  AUTHORS   de la Higuera,I., Kasun,G.W., Torrance,E.L., Pratt,A.A.,
            Maluenda,A., Colombet,J., Bisseux,M., Ravet,V., Dayaram,A.,
            Stainton,D., Kraberger,S., Zawar-Reza,P., Goldstien,S., Briskie,J.,
            White,R., Taylor,H., Gomez,C., Ainley,D.G., Harding,J.S.,
            Fontenele,R.S., Schreck,J., Ribeiro,S.G., Oswald,S.A., Arnold,J.M.,
            Varsani,A., Enault,F. and Stedman,K.M.
  TITLE     Direct Submission
  JOURNAL   Submitted (24-FEB-2020) Biology, Portland State University, 1719 SW
            10th Ave, Portland, Oregon 97201, USA
COMMENT     Bankit Comment: ALT EMAIL:ide@pdx.edu
            Bankit Comment: TOTAL # OF SEQS:451
            Bankit Comment: GAP: est. length - alignment

            ##Assembly-Data-START##
            Assembly Method       :: MetaSPAdes v. 3.12; IDBA-UD v. 1.1.1;
                                     MEGAHIT v. 1.0.3
            Sequencing Technology :: 454; Illumina
            ##Assembly-Data-END##
FEATURES             Location/Qualifiers
     source          1..3352
                     /organism="Crucivirus-222"
                     /mol_type="genomic DNA"
                     /isolate="001557_virusbaton"
                     /isolation_source="Water"
                     /country="France"
                     /collection_date=2013
     CDS             1..900
                     /codon_start=1
                     /product="Putative Replication Associated Protein"
                     /translation="MSIDNKRARGWIFVYNNWQEKDVDFFKAFAKESRYLVFGKEIGE
                     NKTPHLQGYVEFANQRSGAAMRKQHDGKTHWEMRIGTPEEAAAYCKKGEQPKDEWEEQ
                     GTKGPNYGKNAIIFEHGKVPKQGQRSDLDEIAELVVDGASITEIASTYPKEYIKFHKG
                     ISALKSALYEHRKTKPYVCWRWGETGVGKTRGAISEHKSVYIKDGTQWWDGYEQQTAI
                     IIDDFDGKWPFRDLLRLLDYNPYQGQYKGGYVPINSPVIYITCDRPPALMGYSEKELS
                     QLTRRIDAIIEIKADMTTQTPTA*"
     CDS             1128..1430
                     /codon_start=1
                     /product="putative ORF"
                     /translation="MFLERFVIIVYSDTMSVTSPFQVPGLSAIIAEYATDTKPFEMCM
                     DEISMIAECFNSLLKRWDDLSFSWIAFAISNLREQKALGVRWKRHRDGRPTLILTL*"
     CDS             complement(1777..3123)
                     /codon_start=1
                     /product="Putative CruV Capsid Protein"
                     /translation="MPRVAAAKPKKKKAAPRAKASVRGHGDYGISPSMRYGSTYSNPG
                     PVGKAGRYLGGLAGKAVAGPVGQFLGEHVGGLAHYIGKIFGSGDYVASAGVKVNSIVN
                     PSYQPQALAFNSGKTQVRIRHREFLGDVLSASVANTFQIKSYSLNPGLGTTFPWLAQV
                     CGATFQQYRINGMVFEFRSMSADALNSTNTALGSVIMATDYDSADAAFNSKQQMENTE
                     FGVSCKPSSNMIHAIECARSATSITEQYIRAFANPPNTDIRVYDLGKFYIATSGFQGT
                     NVNCGELWVSYDITLIKAIEQPPGYIIPASLYTITNGGTVNLNAHPFGNSPADVGRNV
                     GVDQIGLTVSGTTIKFPSDMMVGSQFNVSYYCAGAATACTLGTIVGTNGLDCSVQYLP
                     ANDTLTKVIWSIGAIYRGGGTVASPPTLTFQGTTLPANVTYAEVAITQMGYAQTGP*"
ORIGIN
        1 atgagcatcg ataacaaaag agccagaggc tggatctttg tttacaacaa ctggcaagag
       61 aaagacgttg acttctttaa ggctttcgcc aaagaatcac gatatctcgt ttttggcaag
      121 gaaatcggcg aaaataagac gcctcacctt caaggatacg tggagttcgc gaatcagcga
      181 tctggcgcag cgatgcgcaa acaacacgat ggaaaaactc actgggagat gcgcataggc
      241 actccagaag aggccgcagc ctattgcaag aaaggcgagc agccaaagga tgaatgggaa
      301 gaacagggca ctaaaggccc aaattatggt aagaacgcaa tcatttttga gcacggtaaa
      361 gtgccaaaac aaggacagcg atctgaccta gatgaaattg cagagctcgt tgtagatgga
      421 gcttccatca cagaaatagc gagcacgtac cctaaagagt acatcaaatt tcataaaggc
      481 atcagtgcat taaaatctgc actgtatgaa caccgcaaga caaaaccata cgtttgctgg
      541 cgatggggag agaccggcgt cggtaaaaca cgaggcgcga tctctgagca caaatccgtc
      601 tatatcaaag acggaactca atggtgggac ggctatgaac aacagactgc gatcatcatt
      661 gatgatttcg acggaaaatg gcctttccgc gatctgttac gcctattgga ttacaaccct
      721 taccagggtc aatacaaagg cggctacgtc cccatcaaca gtccggtgat ctacattact
      781 tgcgatcgcc cgcctgcatt gatgggctat tctgagaaag agttgtccca actcacacgc
      841 agaatcgacg ccatcattga gatcaaagct gatatgacga cgcaaacacc tacggcgtga
      901 gcgtctgtca gataagctct gaatcaactt atgagtcatt ttttaatact aaaaaatttt
      961 gactcagaag ttagtcacag cgttaaagct tcccatacgg ttttcgtggg ggaaaaacct
     1021 caatggttgc tttgacacgc ttgactcaga agtggcgagg taatactagc ccaacatctg
     1081 agtcagctgt tggtcggctc gccatgcggc cttcggccta attaattttg tttttggaaa
     1141 gatttgtgat catcgtatac tcagacacaa tgtctgttac gagtcccttt caggtccccg
     1201 gcttatccgc aattattgcg gaatacgcaa cagacaccaa accctttgaa atgtgcatgg
     1261 atgaaatatc catgatagct gaatgcttca actcattact caaacgatgg gacgatttga
     1321 gctttagttg gatagcattt gctataagca atctaagaga acaaaaagca cttggagtaa
     1381 gatggaaacg tcatagagat ggcagaccaa cccttatatt aaccttatga agtttgcatg
     1441 ccggcctgcg gccgggggtt atcccgcaca cacgccttgg gaacgcgcgc ctaatgggcc
     1501 cccccccccc ccccgcctcc aatcctggta tatggtacgc tatcgcgtac catggatttg
     1561 aagcctccgg cggggcctcc catagaccgc acgcccccaa ggcgtgcgta cggcccccgg
     1621 ggaagggata cctatgtttg tagcatcaaa cttgaacggg ggtgtgttct gggcggcaaa
     1681 aaattttttg ctgtatgaga ctgtcttccc ctcccgaccc catctcaagg ggtgcctccc
     1741 tctctcttgt ttcgaggtgg agtgaggttt tgatcattat gggcccgtct gcgcgtatcc
     1801 catctgtgtt atcgcaacct cagcataagt gacattcgca ggcaatgtcg ttccttggaa
     1861 cgtcagtgta ggtggcgatg ccactgtacc tccacctcga tagattgcac caatggacca
     1921 aataacctta gttaaggtat cattggcagg taagtactgt acgctacaat cgaggccatt
     1981 cgtacctacg attgtaccta gagtacatgc ggtagctgcg ccagcgcaat agtaagatac
     2041 attaaactga gaaccaacca tcatatccga tgggaacttg atagtggttc cggacactgt
     2101 aagtccgatc tgatcaacac ccacattacg gccaacgtca gctggactgt taccgaaagg
     2161 atgagcattg agattgactg taccaccatt agtgatggtg tacagagaag ctggaataat
     2221 atatcccgga ggctgctcaa tcgccttgat aagtgtgata tcatacgaaa cccaaagctc
     2281 accgcagttc acgttggttc cttgaaatcc agatgtagcg atgtagaact ttccaagatc
     2341 atacacgcga atatccgtat ttgggggatt ggcgaacgcg cgaatatact gttcagtaat
     2401 tgacgttgcg gaacgcgcac attcgatagc atggatcatg ttagacgaag gcttacatga
     2461 cacaccaaat tcggtattct ccatctgttg ctttgaattg aatgcagcat ccgcgctatc
     2521 ataatcagtg gccataatca ctgaacccaa agcagtattc gttgaattca acgcatcggc
     2581 actcatgctc cggaactcaa acaccatacc gttgatgcga tactgctgaa aagtagcacc
     2641 gcacacctgg gcaagccacg gaaacgtggt gcctaatcca ggattaagac tgtaactctt
     2701 aatctggaaa gtgttagcga ccgaagcact cagaacatct ccgaggaatt ctcgatgtct
     2761 gatccgtacc tgcgttttgc cagagttaaa cgccaatgct tgaggctgat aagaagggtt
     2821 aacgatactg ttgaccttca cgcctgccga cgcgacatag tcaccagaac cgaagatctt
     2881 gccgatataa tgcgccaagc ctccaacatg ctcaccaagg aactgaccga caggtccagc
     2941 aacagctttc cctgcaagac cgcctaaata acggccggct ttgccaacgg gacccggatt
     3001 actataagtg gatccgtaac gcattgaagg gctgattcca taatccccat gaccacggac
     3061 agaggcttta gcacgaggcg cagccttctt tttctttggt ttcgcagcag caacacgggg
     3121 catcttcgat gagacacttg ttgcgagaca gtgatgcttc actggtatac agtatccgcg
     3181 atccttgcct aaaaataaaa taaataatca attaattatt tgatttcaga gccgctagcg
     3241 agactttgcc aggaatcaaa taattaattg attatttatt cctgcaaaag ctttgtgcat
     3301 gtatacatat aacgcttcta tcttgcgtga tcgacgttaa tcttgtgggg ag
//
LOCUS       CruV-223                3354 bp    DNA     circular     24-FEB-2020
DEFINITION  Crucivirus-223 isolate 120_1399_IR2_3297.
ACCESSION   CruV-223
KEYWORDS    .
SOURCE      Crucivirus-223
  ORGANISM  Crucivirus-223
            Unclassified.
REFERENCE   1  (bases 1 to 3354)
  AUTHORS   de la Higuera,I., Kasun,G.W., Torrance,E.L., Pratt,A.A.,
            Maluenda,A., Colombet,J., Bisseux,M., Ravet,V., Dayaram,A.,
            Stainton,D., Kraberger,S., Zawar-Reza,P., Goldstien,S., Briskie,J.,
            White,R., Taylor,H., Gomez,C., Ainley,D.G., Harding,J.S.,
            Fontenele,R.S., Schreck,J., Ribeiro,S.G., Oswald,S.A., Arnold,J.M.,
            Varsani,A., Enault,F. and Stedman,K.M.
  TITLE     Direct Submission
  JOURNAL   Submitted (24-FEB-2020) Biology, Portland State University, 1719 SW
            10th Ave, Portland, Oregon 97201, USA
COMMENT     Bankit Comment: ALT EMAIL:ide@pdx.edu
            Bankit Comment: TOTAL # OF SEQS:451
            Bankit Comment: GAP: est. length - alignment

            ##Assembly-Data-START##
            Assembly Method       :: MetaSPAdes v. 3.12; IDBA-UD v. 1.1.1;
                                     MEGAHIT v. 1.0.3
            Sequencing Technology :: 454; Illumina
            ##Assembly-Data-END##
FEATURES             Location/Qualifiers
     source          1..3354
                     /organism="Crucivirus-223"
                     /mol_type="genomic DNA"
                     /isolate="120_1399_IR2_3297"
                     /isolation_source="Water"
                     /country="Norway"
                     /collection_date=2011
     CDS             1..1134
                     /codon_start=1
                     /product="Putative Replication Associated Protein"
                     /translation="MTNKLTRAEQVMQMKKKEQYDEYIEYCKRMSGYDDEENERKRMN
                     RRFVGDDEEPSDCDNDDAEDTRLRQERLAQLKNVAPVNPPLSTQARSAKSSIDAPPTE
                     AAIDYELLDEFKDSGIRWYNWFLTSYADEGITLKCVHKDVIFCVSKREICPTTARPHS
                     HTYVEFRQRKSMRWLKNFFKNPALNCQPRLGTAKQAIEYVVKEATTYPGAKPEFYGEP
                     KKQGNRSDLDAMKEAIVQGGAGIDLLMKFNGNALRHMGMIERAQKVRWGYSAMENAII
                     GMRLGRPMPDKLIDGIRADDEILAEIEIIIEEAEAIDEEREKVDELNENIREEDDEEK
                     IKGNKKERTQMQALRRIGLEELNEKVGEENRNIEAKAHAARAE*"
     CDS             1326..1658
                     /codon_start=1
                     /product="putative ORF"
                     /translation="MWYSNFCEKFESSYQHLLGICAKMPKSPILKLKKKIMKMASGEL
                     YWYYDAILKIYNQAPGEEYEIELNKENYSRGCTEAVYDFMFLSMNRMGFIVELVRNRL
                     FELRENYV*"
     CDS             complement(1693..3072)
                     /codon_start=1
                     /product="Putative CruV Capsid Protein"
                     /translation="MPYVYVKSSQPRQQSYSYGAHPRGAYPRPAYSRPYVPRGAYGGR
                     GSYWSDFGTKYGAIAGNYLGRSVPTWLGGGAYRPRATARKPRGAYPARGTHRGRGSYE
                     EAQQGQLAPIPPSFNTSKKDDYVEIVKREYIGDIISSGTASTFLTQSFYVNPSDSITF
                     PWLANLCATSFQQYRIISCMFEFKSTSADALNSTNTALGGVVASINYDSTDAPPLTRA
                     QMENTDWSQCVKPSQGMLIPLECKESTTGLQGLLYTRQSQTLPTGSDRKLFDMAQLTI
                     GTFGIQGTSVILGSLYVSYRIRLYKTVQFAPLINAGYANYTGAGATTSAYFGTTPTGD
                     DNMGLTLTGTVITIPRAYCIPGACYVLLVAYTGSSAALTNPTVTYGGSASALNMYSIN
                     NGSTYTARLSSTAGATQNQLVLLEAFSISNTPTAAATITFSAGTLPGGSAYNSFTIIQ
                     RNADLATFI*"
ORIGIN
        1 atgacaaaca aactgactcg tgccgagcaa gtgatgcaaa tgaagaagaa agagcaatac
       61 gatgaatata ttgaatattg taagagaatg agtggttatg atgatgaaga gaatgagagg
      121 aaaagaatga ataggagatt tgttggtgat gacgaagagc catccgattg tgataatgac
      181 gacgccgagg atactcgcct tcgtcaagaa cgtctggctc aattgaaaaa tgtggctcct
      241 gtcaatcctc cgctttctac gcaagctcgg tctgccaaga gtagtattga tgctcctcct
      301 acggaagccg ccattgacta cgaattgtta gatgaattta aagatagtgg tattagatgg
      361 tataattggt tcctcactag ctacgccgat gagggaatca cccttaaatg cgtgcataaa
      421 gatgtgattt tttgtgtttc taagcgcgaa atctgcccta ctaccgcacg tcctcattcg
      481 cacacctacg tcgaatttag acagagaaaa agcatgagat ggctcaagaa ctttttcaaa
      541 aatcctgccc taaattgcca gcctcgttta ggaacagcaa aacaagctat tgagtatgtg
      601 gttaaagaag ctactacgta tcctggagct aaacctgagt tttatggtga gcctaagaaa
      661 cagggcaatc gcagcgacct tgacgcaatg aaagaagcca tcgtccaagg cggcgccgga
      721 attgacctgt tgatgaaatt taacggtaat gcccttaggc acatgggtat gattgagcga
      781 gctcaaaaag ttagatgggg ttatagtgct atggagaacg ccatcatcgg aatgagactt
      841 ggccggccga tgcccgacaa gctcatcgat ggcattcgcg ccgacgatga aatcttggcg
      901 gagattgaaa taattattga agaagctgaa gctattgatg aagaaagaga gaaagttgat
      961 gaactaaatg aaaatattag agaagaagat gatgaagaga aaattaaggg taataagaag
     1021 gaaaggactc agatgcaagc ccttcgccga attgggttag aagaattgaa cgaaaaggtt
     1081 ggtgaagaga atagaaatat tgaggctaaa gctcacgccg ctagggcgga ataatgttcg
     1141 agcgcagagg ctcaacatct gaaaaaaatt gacccctata tacgaatata tggataccat
     1201 atttttttgg accaaaaagc gccttgcctt tatgtgtccg gtaatattac gaaatccttc
     1261 agattccttc ggacacataa tcccggaagg tcgtttctgg caattgttaa gtattttagc
     1321 ttggtttgtg gtattctaat ttttgcgaaa aattcgagag ctcctatcag catcttttag
     1381 gtatttgtgc gaaaatgcct aaatccccta tcttgaagct caagaagaaa attatgaaga
     1441 tggcctccgg cgagctctat tggtattatg acgctattct taaaatctat aatcaagccc
     1501 ctggtgagga gtatgaaata gaacttaata aagagaacta cagtcgtggc tgtactgaag
     1561 ctgtttacga cttcatgttt ttaagtatga atagaatggg ttttatcgta gagctcgtaa
     1621 gaaacagact cttcgaactc cgagaaaact atgtttgaat gtgttgtgtg tttacatata
     1681 tattatatgc ctttaaatga atgtcgccag gtcagcgttg cgctgaataa tggtgaagct
     1741 attataggcc gagcctcctg gcaatgtgcc tgcgctaaat gtgattgtcg ctgcggcggt
     1801 tggtgtgttg gaaatactga atgcttcaag gagcacaagt tggttttgtg ttgctcctgc
     1861 ggttgaagac aaccttgctg tataagttga accattgttg attgagtaca tattgagagc
     1921 acttgcgctt cctccatacg tcactgttgg atttgtgagc gctgccgatg aacctgtgta
     1981 agctacgagc aagacataac acgctcctgg aatacaatac gccctcggaa tagtgatgac
     2041 tgttcctgtc agggttaatc ccatattgtc atctcccgtt ggagtcgtgc caaaataggc
     2101 agatgtagtc gctcctgccc cagtgtaatt ggcataccct gcgttgataa gcggcgcaaa
     2161 ctggactgtc ttatacaatc ggatgcgata tgatacatac aaggaaccta agatgactga
     2221 tgtcccctga atgccgaaag tcccaatggt gagttgcgcc atatcaaaga gcttcctatc
     2281 tgaccccgtt gggagcgttt gtgattgtct ggtgtatagc aagccttgca atccggtcgt
     2341 actctccttg cactccagcg gaataagcat tccctgggac ggctttacac actgcgacca
     2401 gtcggtgttc tccatctgcg ctctcgttaa aggcggtgca tccgtgctgt cgtagttgat
     2461 agacgcaacc actccaccca gcgctgtgtt tgttgagttg agtgcatcag ccgatgtaga
     2521 tttgaattca aacatacacg aaatgatgcg atattgttga aaactggtgg cgcacaagtt
     2581 tgccaaccag ggaaatgtaa ttgagtctga tgggttgacg taaaaagact gcgtcagaaa
     2641 tgttgaagct gtcccagacg aaataatatc tccgatatat tccctcttca caatttccac
     2701 gtagtcatcc tttttactag tgttaaatga tggtggaatt ggggcaagtt gcccttgctg
     2761 tgcctcctcg taagaacctc ggcctcggtg tgtccctcgg gcaggataag ctccccttgg
     2821 cttgcgtgct gttgctcggg gtcggtatgc acctcccccc agccaggtag gaactgacct
     2881 acccagatag ttccctgcaa tagctccata tttcgttccg aagtcactcc aatatgaacc
     2941 tcttccccca tatgcacctc tcggcacata aggtcgggaa tatgctggcc tcggatacgc
     3001 tccccgtggg tgagctccat acgagtatga ttgttgtctt ggttgtgatg atttcacgta
     3061 gacatagggc attttatccc ctaaatcgtc tccgtggtga taagcgttac ttttggggta
     3121 cttgtatcaa attttaggat atttatagcc ttgtgggcag ctcaaacatc gctaatattt
     3181 gatattcttt cccgctgtgc cagatggaaa gaaatgtgtt actcccccgc tggcgctatt
     3241 gggcttacgg tcatcctgcc cctccagcag gctgcccgac gatacactgg aaggaacgtt
     3301 gatgtttttt ttgccttttt tcttattgcc ttgtttggaa gtgtatggta gaaa
//
LOCUS       CruV-224                3359 bp    DNA     circular     24-FEB-2020
DEFINITION  Crucivirus-224 isolate PCD_184.
ACCESSION   CruV-224
KEYWORDS    .
SOURCE      Crucivirus-224
  ORGANISM  Crucivirus-224
            Unclassified.
REFERENCE   1  (bases 1 to 3359)
  AUTHORS   de la Higuera,I., Kasun,G.W., Torrance,E.L., Pratt,A.A.,
            Maluenda,A., Colombet,J., Bisseux,M., Ravet,V., Dayaram,A.,
            Stainton,D., Kraberger,S., Zawar-Reza,P., Goldstien,S., Briskie,J.,
            White,R., Taylor,H., Gomez,C., Ainley,D.G., Harding,J.S.,
            Fontenele,R.S., Schreck,J., Ribeiro,S.G., Oswald,S.A., Arnold,J.M.,
            Varsani,A., Enault,F. and Stedman,K.M.
  TITLE     Direct Submission
  JOURNAL   Submitted (24-FEB-2020) Biology, Portland State University, 1719 SW
            10th Ave, Portland, Oregon 97201, USA
COMMENT     Bankit Comment: ALT EMAIL:ide@pdx.edu
            Bankit Comment: TOTAL # OF SEQS:451
            Bankit Comment: GAP: est. length - alignment

            ##Assembly-Data-START##
            Assembly Method       :: MetaSPAdes v. 3.12; IDBA-UD v. 1.1.1;
                                     MEGAHIT v. 1.0.3
            Sequencing Technology :: 454; Illumina
            ##Assembly-Data-END##
FEATURES             Location/Qualifiers
     source          1..3359
                     /organism="Crucivirus-224"
                     /mol_type="genomic DNA"
                     /isolate="PCD_184"
                     /isolation_source="Soil"
                     /country="New Zealand"
                     /collection_date=2012
     CDS             complement(1..1554)
                     /codon_start=1
                     /product="Putative Replication Associated Protein"
                     /translation="MTTSNTKNNIETLERDIPDETRVELTTRKQARNYCWTLNNYTAE
                     ELESISLNKFRYLIYGKEIAPTTGTPHLQGYLESSKPITFMAIKEACPGLQRASFFVR
                     KGTRDQARDYCLKDGQTTEYGNWNAGGQGTRTDWQQIWTTIKDGASFHDIASEYPEQA
                     IKYNSGIKACIASADAELGKQKLAQEFASFKPNKWQHDLLMDIAKKPDHRKVIWIVDT
                     KGGRGKSYLAKHLLANMNACYLTNCKTTDAAHAYSGQNIVVFDYSRSNEDRINYGIIE
                     QIKNGLIFSPKYDSRTKVHAIPHVVCFSNFAPERSMLSADRWDIRELNDNSVAPIENT
                     DNIPHQKDSVAKSPSINNELEQLIEDAADELELYGVDLSKDVQEMYTPDEDNWEEFNQ
                     ATTPPCRSQVAIPLPEWYNMHRYFGLPKDLPTVSNVHISPAVQAAIEQLSVNYQGSSS
                     TEDHLDTREAGNTGTASLTCPNNSPMSIEYQDYLAEKKLRYLIQHQNDDTDDEFTDCE
                     DIYTSSDDI*"
     misc_feature    153..182
                     /note="stem-loop"
     misc_feature    165..173
                     /note="nonanucleotide"
     CDS             1676..2989
                     /codon_start=1
                     /product="Putative CruV Capsid Protein"
                     /translation="MSKQIVLATKTRRRRRQVPRKYPVRFRALPVLTGRGAYRKKYPR
                     STSSYSTPYGAMIGGGIGTALGGPVGGMIGGGVGHLAQSLIKHITGFGDYTVEVNSLM
                     GNRFNPPELINQSHRSVCLRHREFITDITASSAFTINAFDINPGLSDSFPWLSGVANN
                     FEEYMIAGMVYEYKSLSADYTTASSAALGYVAMATQYNPLLPEFTDKVHLENYEFANS
                     AKPSENFIHPIECKKSLNPVNQLFVRTGAVETGADQRLYDLGKFQIATAGNSGSGILG
                     ELWCTYEIHFFKPKLVDAEDILMAGHWQLNSVTNASPLGSAAATEVTGTNLPVTLTGG
                     SIEFPSTISDGRYLITYNVVGSSTATTMFTLTATSNCSIPNKYAGNTVANFKTQNGVT
                     TTGGISMWTVTITGPSAIVVVNDDGTLPASATSGDLWIVQLNSEL*"
ORIGIN
        1 ttatatgtca tcggatgatg tgtatatatc ttcgcagtca gtgaattcgt cgtctgtgtc
       61 atcattctga tgttgtatta gatatctgag ctttttttca gctaagtagt cttggtattc
      121 aatggacata ggactattgt taggacacgt aagagaggcg gtaccagtat tacccgcctc
      181 tcgtgtatcg aggtggtctt cagtagagga agacccttga tagttcacac tcagttgttc
      241 aatggcagct tgtacagctg ggcttatatg cacgttagat actgttggta ggtctttggg
      301 taatccaaag tatctatgca tgttgtacca ttcgggcaat ggtatagcca cctgacttcg
      361 gcagggtggc gttgttgctt gattgaactc ttcccagttg tcttcatcag gcgtgtacat
      421 ttcttgtacg tcctttgata agtcaactcc atagagttct aattcgtctg cagcgtcttc
      481 tatcaattgt tcgagttcat tattgataga cgggcttttg gctacactat ctttttggtg
      541 tggtatgttg tccgtattct caattggcgc tacagagttg tcgtttagtt cgcgtatgtc
      601 ccagcggtct gcacttagca tcgagcgttc tggtgcgaag ttactgaagc agactacatg
      661 ggggatagcg tgtactttgg ttcgactgtc atatttggga gagaatatga gtccgttctt
      721 tatttgttct atgatgccgt agtttatgcg gtcttcatta gagcggctgt agtcgaatac
      781 tactatattt tgtcccgaat aggcgtgagc ggcatctgtt gtcttacagt ttgtaagata
      841 gcatgcgttc atattcgcta gtaagtgttt ggcgagatat gacttaccac gtccgccttt
      901 tgtgtcgact atccagataa ctttacggtg atctggcttc ttggctatgt ccattagaag
      961 gtcgtgctgc catttgttag gcttaaacga agcgaattct tgtgctagct tctgtttgcc
     1021 gagttcagca tcagcacttg ctatgcatgc tttgatgcct gaattgtatt ttatggcttg
     1081 ttctgggtat tcgctggcaa tgtcatggaa ggaagcgccg tctttgattg ttgtccagat
     1141 ctgttgccag tctgtgcgcg ttccttgtcc accagcgttc cagttaccat attcagttgt
     1201 ttggccatct ttgaggcaat agtcacgtgc ttggtcacgt gtgccttttc taacgaagaa
     1261 tgatgcccgt tgaagtcctg ggcatgcttc tttgatagcc ataaatgtta tcggtttaga
     1321 tgattctaga taaccttgta agtgaggagt tcctgttgta ggagcgatct ctttaccgta
     1381 tattagatat cggaacttgt tgagtgatat actctcgagt tcttctgctg tgtagttgtt
     1441 gagtgtccag cagtagttac gggcttgctt acgtgtagta agctcaactc gtgtctcgtc
     1501 gggtatgtct ctctcgagtg tctcgatgtt gttttttgtg tttgatgttg tcattgtata
     1561 cgctgtgtat ttgcgggtat atatacttcg tatactatct tttttttggt aattaattaa
     1621 ttggtaatta tttatttttg tgggaaaaga tgtccccatg tatataccca catttatgtc
     1681 taagcaaata gtgttagcaa caaaaacacg tcgtcgtaga cgtcaagtgc ctcgtaagta
     1741 tcctgtaaga tttagggctt taccggtgtt gactggtaga ggagcttata ggaagaagta
     1801 tccacgatca actagttcgt attcaacgcc ttatggcgct atgatcggtg gaggtattgg
     1861 gactgcttta ggtggtcctg taggtggtat gatcggcggt ggagtcggtc atcttgcaca
     1921 aagccttatt aagcatatta ctggctttgg agactatact gtagaagtca attcattgat
     1981 gggcaatagg tttaatcctc ctgagttgat taatcagtct catcgtagtg tatgcctgcg
     2041 acacagggag tttataacgg atattactgc gtcttcagcg tttacaatta acgcgtttga
     2101 tataaatcca gggttatccg attctttccc ttggttatca ggtgttgcaa ataattttga
     2161 ggaatacatg atagctggca tggtatatga gtataagtct ttatctgctg attatactac
     2221 tgcatcgtct gcagctcttg gatatgtggc tatggctact caatataatc ctttattgcc
     2281 cgagtttaca gataaggtcc atttagagaa ctatgagttc gctaatagtg ctaagccttc
     2341 agagaatttt atacatccaa ttgagtgcaa gaagtcgctc aatcccgtta atcagttgtt
     2401 tgttcgtact ggagcggtag agactggtgc tgaccagcgt ctgtacgatc ttggtaagtt
     2461 tcagatagca actgcaggta attcaggctc cggtattctc ggagagttgt ggtgtactta
     2521 tgagatacac ttcttcaagc ctaagcttgt tgatgcggaa gatatcttaa tggcaggcca
     2581 ttggcagctt aatagcgtta ctaatgcgtc tcctcttggt tcagctgctg ctactgaagt
     2641 tactggtact aatcttcctg taactcttac tggtggttct attgaattcc cgtcgactat
     2701 ctctgatggt cgttatttga ttacttataa tgttgttggt tcgagtactg ctactactat
     2761 gtttacgttg acagctacgt ctaattgtag cattcctaat aagtatgctg gtaatacagt
     2821 agctaatttt aaaacgcaga atggtgtgac caccactggt ggtatttcta tgtggacagt
     2881 aactatcact ggtccaagtg ctatagtagt tgttaatgat gatggaactc ttccagcgtc
     2941 tgctacttca ggtgatctgt ggatagtcca attgaattct gagttgtaaa ggtctttagg
     3001 acaaaaaagg gccttattgc cctctctttt tttgatgccg ggctaggtca tcaggtcagc
     3061 atattggtta gatagttaga atagttgtca gtacagatga gtgcgtgggg cgatggggcc
     3121 ccactgcact gagtgcttcc ctgacagagg cgcaccgcac gaagtgtgga gcgaagccgt
     3181 taggctgagc tgcacacata ggaggagcaa gtattatcaa aatagtctat tctcgggagc
     3241 gagcgcgaag cgcgatgtgc ccgtattgaa tcggtagatc agcacaccgc aggtgtggtg
     3301 ggggcgtccc gcgcgtggga cccaccacac ccggtgtgcc attaactaat ggctattgt
//
LOCUS       CruV-225                3362 bp    DNA     circular     24-FEB-2020
DEFINITION  Crucivirus-225 isolate 000707_ADNa22.
ACCESSION   CruV-225
KEYWORDS    .
SOURCE      Crucivirus-225
  ORGANISM  Crucivirus-225
            Unclassified.
REFERENCE   1  (bases 1 to 3362)
  AUTHORS   de la Higuera,I., Kasun,G.W., Torrance,E.L., Pratt,A.A.,
            Maluenda,A., Colombet,J., Bisseux,M., Ravet,V., Dayaram,A.,
            Stainton,D., Kraberger,S., Zawar-Reza,P., Goldstien,S., Briskie,J.,
            White,R., Taylor,H., Gomez,C., Ainley,D.G., Harding,J.S.,
            Fontenele,R.S., Schreck,J., Ribeiro,S.G., Oswald,S.A., Arnold,J.M.,
            Varsani,A., Enault,F. and Stedman,K.M.
  TITLE     Direct Submission
  JOURNAL   Submitted (24-FEB-2020) Biology, Portland State University, 1719 SW
            10th Ave, Portland, Oregon 97201, USA
COMMENT     Bankit Comment: ALT EMAIL:ide@pdx.edu
            Bankit Comment: TOTAL # OF SEQS:451
            Bankit Comment: GAP: est. length - alignment

            ##Assembly-Data-START##
            Assembly Method       :: MetaSPAdes v. 3.12; IDBA-UD v. 1.1.1;
                                     MEGAHIT v. 1.0.3
            Sequencing Technology :: 454; Illumina
            ##Assembly-Data-END##
FEATURES             Location/Qualifiers
     source          1..3362
                     /organism="Crucivirus-225"
                     /mol_type="genomic DNA"
                     /isolate="000707_ADNa22"
                     /isolation_source="Water"
                     /country="France"
                     /collection_date=2013
     CDS             1..996
                     /codon_start=1
                     /product="Putative Replication Associated Protein"
                     /translation="MTDQHDDEPEQKNECRGFDVTIWETMGDKVFTHTKIGEVLNEIA
                     NKYAFQLEEGEEKKRRHFQIRLNTIKKTRWRQLTKLFVIALDVKGIKGHIHIRPTSKG
                     VHSKRNFNYVLKADTRILGPWTEHDFNEDNKQYIPIPLRGITRDKLRPFQQSLLDMTN
                     IYDDRAIDFIYDPTGNYGKGFAADYLEIYNDCYILPSIDDYKLIIQDAHNFITSRINR
                     FGEKGRNVKAFILDMPRVMKMDDMHNLIGAIETIKGGRFVDYRHKSSAIVRIEKPRVF
                     VFTNKGIDLTLYSKDRFKIWRITEKLELKPYTFPTDDKAYNLEFKPKEKKIIRLI*"
     CDS             1081..2493
                     /codon_start=1
                     /product="Putative CruV Capsid Protein"
                     /translation="MPARAKTARPRRVYKQDMKSARRANRVARVVAQPQRAQARRNMG
                     NEFLRGVGRTLAPVASSVLSSLGKRAMTMISGRGDYDISGITQNSLIGKMSPTVPQFT
                     NGANGSVHITHREYLTDILSTTDFNRLSFPINPALPSTFPWLSKIAANFEQYQIDGLI
                     FEFKSGSSDALNSTNTALGYVVMATEYNSLAQGYQNKLEMENAIFAVSTKPSLSCIHA
                     IECAPNQSPLNILYTRTGFESISDSDKRLYDLGVFNLATVGMQAIGANIGELWVSYSV
                     KMYKSRFLAPGLTIPTYSLQFGDVNNARALLGSVASQVVKINTLNVNVNQTFNATTNI
                     ISAIFTLPPGSAGSYVLNYELVGDLSNVLLGTRPFQPNPFSSLVNVSERNIYPSNDAK
                     IGLLDNSKPYNSNIIATLQTPGSVSCLLTYSFAVVNNNLSASFTFSIEQGTLSTIVRT
                     TTTAPFVLGVCYITQVNAQL*"
ORIGIN
        1 atgaccgacc agcacgacga cgaaccagag cagaagaacg aatgcagagg atttgacgtg
       61 actatatggg agactatggg ggataaggta ttcacacata caaagatagg cgaggtgctt
      121 aatgaaatag caaataaata tgcttttcag ttagaagagg gcgaagagaa aaagcgccga
      181 cactttcaaa ttagattgaa cacaataaaa aagacaagat ggcgacaatt aacaaaatta
      241 tttgttatcg ctttagatgt aaaagggatt aaaggacata tacatatacg ccctacaagt
      301 aaaggggtgc atagtaaaag aaattttaac tatgtattaa aggcagacac ccgaatatta
      361 ggaccatgga cagagcatga ttttaacgaa gataataagc aatatatccc cataccttta
      421 cgaggtataa cacgtgataa attgcgaccg ttccagcagt cccttttaga tatgactaat
      481 atatatgacg atagggctat cgactttata tatgacccta cgggcaatta cggtaaaggg
      541 ttcgcggccg attatttaga gatatataac gattgctata ttctccccag catagatgat
      601 tataaactta taatacaaga tgcacataat tttataacgt ctagaattaa tagattcgga
      661 gaaaaggggc gaaatgtaaa agcgtttata ttggatatgc cccgagttat gaaaatggac
      721 gatatgcata atttaatagg agctatagaa acaattaaag ggggccgttt tgttgattat
      781 aggcataaaa gtagcgcaat tgttagaatt gaaaagcccc gagtatttgt atttactaat
      841 aaagggatag atttaacttt atacagtaaa gatagattta aaatttggag gataaccgaa
      901 aaattagaac ttaagcctta tacatttcct acagatgata aggcctataa tttagaattt
      961 aagcctaaag aaaaaaaaat tatacgatta atataaaaaa tctttttata atatagaaaa
     1021 aatataatat ttaatttaaa tataaattaa attttatttt ctaaatatag attatcaaca
     1081 atgccagcca gagcaaaaac agcacgacca cgcagagtgt acaaacaaga tatgaaaagt
     1141 gcaagacgcg caaacagagt cgcccgagtg gtagcacagc cacaacgagc acaagcacgg
     1201 cgtaatatgg gtaatgaatt tttgcgagga gtagggagaa cccttgcgcc cgttgcttct
     1261 tccgttttaa gttctttagg caaaagagct atgactatga tttcagggcg tggagattat
     1321 gatatatcag gtatcacgca aaacagcttg ataggcaaaa tgagtcctac agttccacaa
     1381 tttacgaatg gagctaatgg ttcagttcat ataacacaca gagaatatct tacagatata
     1441 ttaagcacta cggatttcaa ccgtttatcg tttcctatta atccagcgct tccttccacc
     1501 tttccgtggt tgtctaagat tgcggcgaat tttgagcagt atcagataga cggattaata
     1561 tttgagttta aaagtggtag cagtgatgct ttaaattcta ctaatacggc gttggggtat
     1621 gtggtgatgg ctacggagta taactcttta gctcagggtt atcagaataa attagaaatg
     1681 gagaatgcta tatttgccgt ttccactaag ccttccttaa gttgtatcca tgcgatagaa
     1741 tgcgcaccga atcaaagccc cttgaatatt ttatatacac gtacaggctt tgagagcata
     1801 agtgatagtg ataaacgact ttatgacctt ggagtattta atctcgcaac agtagggatg
     1861 caagcaatcg gtgcgaacat aggggagcta tgggtgtcat attcagtgaa aatgtataag
     1921 agtagatttt tagccccagg tttaacaatt cctacatata gcctacaatt tggcgatgtt
     1981 aataacgcta gagccctttt gggttcggtt gcgtcgcagg tagtaaaaat taacacttta
     2041 aatgtaaatg ttaatcaaac gtttaacgct actactaata taataagtgc tatatttaca
     2101 ttgcccccag gttcggcggg ttcatatgtt cttaattatg aattggtggg ggacttatca
     2161 aatgttttat taggcacacg accatttcaa ccgaacccct tctcgagtct tgtaaatgtt
     2221 tcagaacgta atatataccc ttctaatgat gctaagattg gccttttaga taattctaag
     2281 ccttataatt caaatattat tgctacttta caaacaccag gcagtgttag ttgcctatta
     2341 acatattcct ttgcagttgt gaataacaac ctctcagcgt cctttacttt ttcaattgaa
     2401 cagggtacat tgtctacaat tgtaagaact acaactaccg cgccgtttgt tcttggtgta
     2461 tgttatatta cacaggttaa cgcacaatta taataaaaga aaagcgtaat attccatagg
     2521 atatatggag tgggctttag cctttcgcac actagataaa ttatttaata agattgaaaa
     2581 caaccctatt aaattaaaaa atgcgccatt ttaaataaaa agcgccatta attgccatta
     2641 aaagccatta taatttatgt taaaagtttt aaacatatac acttttttaa cgttttgggc
     2701 cattgcgcca ttatccacaa agctatatat aagggagtac acatttcctt tatagataaa
     2761 gaaaataaca ttcataaagc gaatatgcaa tatgagcggt tgcaactatg gacctgcaag
     2821 ggctatagcc tgaatgttat tttccccttt agtaagttta ataagttagt tttactaaat
     2881 gggataaacg cgatagcgtg cgcgtagcgt ataaaagccc gtctggggct ctgttgcttg
     2941 ccccctgcgg gggcatatga taaaagcccg tctggggctc tgttgcttgc cccctgcggg
     3001 ggcatatggt tttagcccgc ctggggctca accaccgaaa aaacacacaa cacccgcgaa
     3061 agcgtagctc gcgggggtta gggtggcttt taaggcatac ccgataattt atttaaaaag
     3121 ggtatatatg gtggatacat ataataaaag ggacctgcaa ggatcccgcc acatattata
     3181 taatgtagtc atacaccccc gagggcctac tgttcattat gcttttttaa ttttcttttt
     3241 aaatatagaa aaaaaatatt aaatatatag aaaaataaat tttttaaata tatagaaaaa
     3301 agcgccattt aaaatttaat taattggctt aaataaaaat tattatctaa atattaatta
     3361 ta
//
LOCUS       CruV-226                3362 bp    DNA     circular     24-FEB-2020
DEFINITION  Crucivirus-226 isolate 120_1402_IR2_3297.
ACCESSION   CruV-226
KEYWORDS    .
SOURCE      Crucivirus-226
  ORGANISM  Crucivirus-226
            Unclassified.
REFERENCE   1  (bases 1 to 3362)
  AUTHORS   de la Higuera,I., Kasun,G.W., Torrance,E.L., Pratt,A.A.,
            Maluenda,A., Colombet,J., Bisseux,M., Ravet,V., Dayaram,A.,
            Stainton,D., Kraberger,S., Zawar-Reza,P., Goldstien,S., Briskie,J.,
            White,R., Taylor,H., Gomez,C., Ainley,D.G., Harding,J.S.,
            Fontenele,R.S., Schreck,J., Ribeiro,S.G., Oswald,S.A., Arnold,J.M.,
            Varsani,A., Enault,F. and Stedman,K.M.
  TITLE     Direct Submission
  JOURNAL   Submitted (24-FEB-2020) Biology, Portland State University, 1719 SW
            10th Ave, Portland, Oregon 97201, USA
COMMENT     Bankit Comment: ALT EMAIL:ide@pdx.edu
            Bankit Comment: TOTAL # OF SEQS:451
            Bankit Comment: GAP: est. length - alignment

            ##Assembly-Data-START##
            Assembly Method       :: MetaSPAdes v. 3.12; IDBA-UD v. 1.1.1;
                                     MEGAHIT v. 1.0.3
            Sequencing Technology :: 454; Illumina
            ##Assembly-Data-END##
FEATURES             Location/Qualifiers
     source          1..3362
                     /organism="Crucivirus-226"
                     /mol_type="genomic DNA"
                     /isolate="120_1402_IR2_3297"
                     /isolation_source="Water"
                     /country="Norway"
                     /collection_date=2011
     CDS             1..1380
                     /codon_start=1
                     /product="Putative CruV Capsid Protein"
                     /translation="MVNYSDKQKAAYYKRKSASVANPRKRAPRTYQKRKNTKYQYPGV
                     GRYLGGMAGGAIGNAITPGIGGAIGSALGGVVGHGAQALVKNITGFGDYHVSKNALVY
                     NRDAVPEFSTNNERCTMVTHREFIQDIIGSTGFNLQQFRINPGILTTFPWLANIAENY
                     EQYVIQGMVYEFKTTCATAVSSTNTALGTVIMASQYNSLSPPFTNKQQMENYEFAQSG
                     VPSASILHPIECDPQQTQCGGVFNVAESDLFNPGDIRLYDIARFNIATVGMQAAATIG
                     ELWVTYKICFLKPKLHAIAAGGDKYFQNAPGDVDVLAPIGPHTLLVPNPANSRIVLPI
                     SDKVFSVDLSFTGILQCMVIWYLEASVTSFNGSTWSATGGSATSDVSQYFTGGVASAI
                     GNTLLPATGTGYGYAVGYFKFGGGPSGAGVLPTVQLSNLVALVGGAMNIVHVNFFTVP
                     HQYDSLPLV*"
     misc_feature    603..629
                     /note="stem-loop"
     misc_feature    complement(608..616)
                     /note="nonanucleotide"
     misc_feature    609..617
                     /note="nonanucleotide"
     CDS             complement(1673..1972)
                     /codon_start=1
                     /product="putative ORF"
                     /translation="MDHQDFHPYYTQEFSQSVHPKCNHSLFRNLNHRINHRINHMIIH
                     SLFHPKCFTSLFTQIYNLGFYSKELLPKRFTQRDFFWALATLPVTLAYGLPKKIY*"
     CDS             complement(2368..3243)
                     /codon_start=1
                     /product="Putative Replication Associated Protein"
                     /translation="MEKTGEITKKVTKETSVCVFDFTLFDDIPYEVIKVALAKICKKY
                     TFQQEKGEETGTIHFQGRFSLKNKKRKCDVIRVCSKDLGWLKFHISITSKENRTNDFY
                     VTKDETRIAGPFSDKEVIEDIPPDVLEMKTLYPWQQQMCDLLHEDSNRAIDIVYDPEG
                     CKGKTALTRYMRHYHGAQLIPLLNDAKDIMRIAYNSITKKGIKSIYMIDMPRAVAKDK
                     LQQFFSGIELLKSGYAYDDRYEFNDITFKKPRVCVFTNTYPTMNFMSKDMWNIWKIED
                     NQLISLVKNGVDVNF*"
ORIGIN
        1 atggttaatt attcggacaa acaaaaagct gcctattata aaagaaagtc ggcgtctgtt
       61 gctaatccta gaaaaagagc acctaggact tatcaaaaaa gaaaaaatac taaataccaa
      121 tatccaggtg tgggtaggta tttaggagga atggcgggtg gagccatagg aaatgctata
      181 acaccaggta ttggaggtgc tataggaagt gcattaggtg gtgttgtagg tcatggtgct
      241 caagcattgg ttaaaaatat tacaggattc ggtgattatc atgtatcaaa aaatgcatta
      301 gtgtataata gagatgctgt tccagagttc tctactaata acgaaaggtg tactatggtt
      361 acacatagag agttcataca ggatattata gggtctactg gttttaatct tcaacaattt
      421 agaattaatc ctggtattct aacaactttt ccttggttag caaatattgc tgaaaattat
      481 gaacaatatg tcatacaagg aatggtatat gaatttaaga ctacttgtgc aactgcagta
      541 tcttctacaa atactgcact aggtactgtt ataatggcat cacaatataa tagtttatct
      601 cctccattta ctaataaaca acaaatggaa aattatgagt ttgctcaatc aggagtacct
      661 agtgcttcta ttttacatcc aattgagtgt gatcctcaac aaacacagtg tggtggtgta
      721 tttaatgttg ctgagtctga cttatttaat cctggagata tcagattata tgatatagct
      781 cgttttaata ttgctactgt aggtatgcaa gcagcagcaa cgatcggtga actttgggta
      841 acttataaaa tatgtttctt aaagcctaaa ttgcacgcta tagcagctgg tggtgataaa
      901 tactttcaga atgctcctgg tgacgttgat gtattggctc ctattggtcc acatacatta
      961 cttgttccaa atcctgctaa ttcgagaatt gtattaccta ttagtgataa agtatttagt
     1021 gttgatctta gttttactgg tatattacag tgtatggtta tttggtattt agaggcgtca
     1081 gttacatctt ttaatggttc tacttggtca gctactggag gatcagcaac ttctgacgta
     1141 agtcaatatt ttacaggtgg cgtagcaagt gcaattggta atactttgtt accagctact
     1201 ggtacaggtt atgggtatgc agtaggttac tttaaatttg gtggtggtcc aagtggagca
     1261 ggagtgttac caacagttca attaagtaat ttagtagctc ttgttggagg tgctatgaat
     1321 atagtacatg taaatttctt tacggttcct catcagtatg acagtttacc acttgtataa
     1381 tttatataat aactctcatt gagcattatt ataaacatta gacttgcgat tccaacgaag
     1441 ttggctgatt ttaagggtag tcgcactggc aacgccatgt gcagcgaacc gtccgtagcc
     1501 cttggggcga ggattacgca agatagtcat cataatcgtt tctccgttta tttctctgca
     1561 ttctccgggt gtctccgttg attctccgat agagaaagtc agctgttaag cactttctcc
     1621 gtttctccga tctccgtaat aagtaataat aaggaaggat taaaggaaga aactaataga
     1681 ttttttttgg gaggccgtag gccagtgtta cagggagcgt agcgagcgcc caaaaaaaat
     1741 ccctttgggt aaatctcttt ggtaaaagct ctttagagta aaaacctaag ttatagattt
     1801 gggtgaacag gctggtgaaa cactttgggt gaaacagact gtgaattatc atgtgattaa
     1861 tcctgtgatt aatcctatga tttagattgc gaaataggct gtgattacac tttgggtgaa
     1921 cagactgtga aaactcctgt gtatagtatg ggtgaaaatc ctggtggtcc atctggtgag
     1981 ttttctgtta aatattactt aagtcatgtt gtgtacgacg catgaggttt tgcttgcaaa
     2041 ccgaaataag tctcgagtga aaggccgaac tttagtccag gccgagcgag ctcttactag
     2101 acatgaataa aagtaatttt gaagttaata actgcgatta ctaacttcga aacgatatgt
     2161 tcctaatagc gtcaaaaggc gaccatcggg agccccccgt aggggtcgag cgtagcgaga
     2221 ttttgtaagc gtttgtgggc cgactcgaat taatcctcgc agtcccctgg tgttcgtttc
     2281 ttgcggtgga gagcgaagcc gtaggctgcg cgagcatttt atcaaatatt ttattaaaca
     2341 tttttaagtt ataactatta ttatacttta aaaattaacg tcgacaccat ttttaactaa
     2401 acttattagt tggttatctt ctattttcca aatattccac atgtctttag acataaaatt
     2461 cattgttggg taagtgtttg taaaaacaca aactctaggt tttttaaaag tgatatcgtt
     2521 aaattcatat ctatcatcat atgcataacc actttttaat agttcaatac ctgaaaagaa
     2581 ctgttgaagc ttgtctttag ctacagctct aggcatatca atcatgtata tgcttttaat
     2641 acctttttta gttatgctat tgtaagctat tctcatgatg tctttagcat catttaaaag
     2701 gggaattaat tgagcaccat ggtagtgtct catatatcta gttaaagcag tcttgccttt
     2761 acaaccttca ggatcatata ctatgtcaat tgctctgttt gaatcttcat gtaataaatc
     2821 gcacatttgt tgttgccatg gatataatgt tttcatttct aatacatctg gtggtatgtc
     2881 ttcaatcact tctttgtcac tgaatggacc agctattctg gtttcatctt tagtaacata
     2941 aaagtcattt gtacggttct cttttgaagt aatagatatg tgaaatttca accaaccaag
     3001 atctttagag cagactctta ttacatcaca ttttctcttt ttattcttta gtgagaatct
     3061 tccttgaaaa tgtattgtgc cagtctcttc tcctttctct tgttggaagg tatatttctt
     3121 acatatcttt gctagtgcaa ccttaattac ttcataaggt atatcatcaa aaagtgtgaa
     3181 gtcaaaaacg catacagatg tttctttagt aactttttta gttatttctc cagttttctc
     3241 catagaaatt attaatacta tattcaaaga tttttatttt taagtaaaaa gtcgtattta
     3301 tggggtgaac ataatacatt agaaattaat tttctagact gaacttatag aaataaaaaa
     3361 aa
//
LOCUS       CruV-227                3365 bp    DNA     circular     24-FEB-2020
DEFINITION  Crucivirus-227 isolate 120_1395_IR2_3297.
ACCESSION   CruV-227
KEYWORDS    .
SOURCE      Crucivirus-227
  ORGANISM  Crucivirus-227
            Unclassified.
REFERENCE   1  (bases 1 to 3365)
  AUTHORS   de la Higuera,I., Kasun,G.W., Torrance,E.L., Pratt,A.A.,
            Maluenda,A., Colombet,J., Bisseux,M., Ravet,V., Dayaram,A.,
            Stainton,D., Kraberger,S., Zawar-Reza,P., Goldstien,S., Briskie,J.,
            White,R., Taylor,H., Gomez,C., Ainley,D.G., Harding,J.S.,
            Fontenele,R.S., Schreck,J., Ribeiro,S.G., Oswald,S.A., Arnold,J.M.,
            Varsani,A., Enault,F. and Stedman,K.M.
  TITLE     Direct Submission
  JOURNAL   Submitted (24-FEB-2020) Biology, Portland State University, 1719 SW
            10th Ave, Portland, Oregon 97201, USA
COMMENT     Bankit Comment: ALT EMAIL:ide@pdx.edu
            Bankit Comment: TOTAL # OF SEQS:451
            Bankit Comment: GAP: est. length - alignment

            ##Assembly-Data-START##
            Assembly Method       :: MetaSPAdes v. 3.12; IDBA-UD v. 1.1.1;
                                     MEGAHIT v. 1.0.3
            Sequencing Technology :: 454; Illumina
            ##Assembly-Data-END##
FEATURES             Location/Qualifiers
     source          1..3365
                     /organism="Crucivirus-227"
                     /mol_type="genomic DNA"
                     /isolate="120_1395_IR2_3297"
                     /isolation_source="Water"
                     /country="Norway"
                     /collection_date=2011
     misc_feature    join(3356..3365,1..17)
                     /note="stem-loop"
     misc_feature    1..9
                     /note="nonanucleotide"
     CDS             75..929
                     /codon_start=1
                     /product="Putative Replication Associated Protein"
                     /translation="MPNKMSRHKNYCFTFNNYPDTLLVDSLVDEGVCKYIAYSKEVAP
                     TTGTRHLQGYVTFKNAKTLTAARAVLPGCHLSVMLGSIAQNDDYINKVAVMTEWGEKP
                     ISNDNRGRAEALRWQRARDLAKEGKIDEIDADIFIRYYGTLKRIRSDYATKPDPVPTR
                     CYWIYGNTGTGKSHAVESAPFTIYKKAMDDLKWFDGYQGEECVYLEDIDKYQVKWGGL
                     IKRLADKWPMQASIKGSMQYIRPAIVCVTSNYSIGEIWTDPQTYEPLQRRFKEIEKLS
                     QEQVIDFS*"
     CDS             1027..1362
                     /codon_start=1
                     /product="putative ORF"
                     /translation="MGNQVVRLVLMLYPNKMYQKTIQGGVKTFPVEITKVFKEWQYNG
                     YTYLSCIARQRKMMVVTKKVAVPARNPRKRSLEDVYVEQYDHRKKRRMSGGGSRRGSG
                     MSLSGVDMS*"
     CDS             1378..2703
                     /codon_start=1
                     /product="Putative CruV Capsid Protein"
                     /translation="MKRSVLFVLKTRTVTLTLTLTLNLTLALTLTLNLTRFTFSQIKI
                     MPYPKYRRKYRLKGRGDYQSGYMNNKGSTNFSLISWGQKNKIYGSGDYKMGTTPTSNS
                     IWNAAGTPKFGSATRVNVVTNREYIQDIVTSSTVGAFKNDVFPINPGLSSTFPWLSVL
                     AQNYEQYRVMGIIFEFKSTVGDNSGVQNVGTVVLGAEYNVSSPPFVNKQVMENSEFAQ
                     SAKSIESQIHGIECAYKEGPLDILFVRTGAITNPNDSIKWYDKANFQIATTGFSTASQ
                     NIGELWVSYHIEFYKPQIPPNIGGVIQTGHFVKEAAGGGGGVNNHFGSGVVRQSGNLP
                     LSSIASTLTINGLYIGNTYTMTAYWAGLVVTFSEPTLAAAVGYTLKGFYGSSLDTRIS
                     GGGSSCNFATFEMCFVATSATAVIEFTGGTVPSDSEVDVLVRGIDNLTL*"
ORIGIN
        1 aattattacc tggacctgcc gtgcagtgta gtgtggcgct atactgcacg agcccccccg
       61 gcgactgagg gggattgcct aacaaaatgt cgcgacataa aaattattgt tttacattta
      121 acaattaccc tgatactctg ttggttgata gcttggttga cgaaggtgtg tgtaagtata
      181 tagcgtattc caaagaagta gctccaacca ccggtacgcg tcacttgcaa gggtatgtca
      241 cgttcaaaaa cgctaaaacc ctgaccgctg ccagagctgt tttgccagga tgtcatttat
      301 ctgttatgct tggttccatt gctcaaaatg atgactatat caacaaagtt gctgttatga
      361 cggagtgggg agaaaagcct atatcaaatg acaatcgggg acgtgcggag gcactgcgat
      421 ggcaacgcgc tcgggactta gctaaagaag gaaaaatcga tgaaattgac gcggatatct
      481 tcattcgtta ttatggaact cttaaacgaa ttcgttcaga ttatgcaact aaaccagatc
      541 ctgtacccac aagatgttat tggatttatg gaaataccgg cactgggaag agtcatgctg
      601 tcgaaagcgc gccttttaca atttacaaga aagctatgga tgatctcaaa tggttcgatg
      661 gttaccaggg cgaagaatgc gtttatcttg aagatattga caagtatcaa gttaagtggg
      721 gcgggctcat caaacgactt gctgataagt ggcctatgca agctagtatt aaaggatcga
      781 tgcaatacat tcgaccggcc atcgtttgtg ttacttctaa ttacagcatt ggagaaatct
      841 ggactgatcc ccaaacctat gagcccctgc agcgacgttt taaagaaata gaaaaactca
      901 gtcaagaaca agttattgac ttttcctaat aaataatgtt cgatgtacag agtctacttg
      961 cagcttgtat aatgggagct ggtgttatgt taggtggagt ttgtattata gtggttaagc
     1021 atatgtatgg gaaatcaagt tgtcaggtta gttctgatgc tttaccctaa taaaatgtat
     1081 caaaagacta ttcaaggagg tgttaaaacg tttcccgttg agattactaa agtatttaaa
     1141 gaatggcaat ataatggtta tacgtatttg tcttgtattg caagacagag aaaaatgatg
     1201 gttgttacaa aaaaagtagc agtaccagct agaaatccac gtaagaggtc cctagaagat
     1261 gtgtatgtgg agcagtatga tcacagaaaa aaaagaagaa tgtccggtgg gggctctagg
     1321 aggggttctg gtatgtctct ttcaggagtt gacatgagtt aatgaacagg gaagaccttg
     1381 aaaaggtctg tactgtttgt gttaaaaacg cgaacggtaa ccctaaccct aaccctaacc
     1441 ctaaacctaa ccctagccct aaccctaacc ctaaacctaa ccaggtttac cttttcccaa
     1501 ataaaaatta tgccttaccc taagtatcgt agaaagtatc gtttaaaagg tcggggagat
     1561 taccaatcgg gttatatgaa taataagggc tctactaact tttcgttgat tagttggggt
     1621 cagaaaaaca aaatttacgg aagcggagac tataagatgg gtactacacc tacctctaat
     1681 tccatttgga atgccgctgg taccccgaaa tttgggtctg ccactagagt caacgttgtt
     1741 accaataggg aatacattca agatatcgtt acgtctagta ccgttggagc cttcaaaaac
     1801 gatgtctttc ctattaatcc tgggctttca tctacctttc cgtggttatc agtattggct
     1861 caaaattacg agcagtatcg tgttatgggt attatatttg agttcaaatc caccgttggt
     1921 gataactctg gagttcaaaa cgtaggtact gtagtgttag gcgcggagta caatgtgagt
     1981 tcacctccgt ttgttaataa gcaagttatg gaaaactcag aatttgccca gagtgccaag
     2041 tctattgagt ctcaaatcca tgggattgag tgcgcctaca aggaagggcc gcttgatatt
     2101 ctgtttgtcc gaaccggggc aattactaac ccaaatgaca gtattaaatg gtacgacaaa
     2161 gccaattttc aaattgctac tacaggcttc tcgactgcat cccaaaacat tggggaacta
     2221 tgggtgtcct atcacatcga attttataag cctcaaattc ctcctaatat tgggggtgtt
     2281 attcaaactg ggcattttgt aaaagaagca gccggtggtg gcggtggggt taataaccat
     2341 ttcggttccg gggttgttcg gcaaagtgga aatctgcccc tgtctagtat tgctagtacc
     2401 ttgactatca atggattata cattggaaac acttatacta tgactgccta ttgggctgga
     2461 ttggttgtta cctttagtga accgactttg gctgcagcag ttgggtacac attgaaaggg
     2521 ttttatggat cttctcttga tacaaggatt agtggtggtg gcagttcgtg taattttgcc
     2581 acctttgaaa tgtgttttgt ggcaacctca gctacagcag ttattgagtt tacaggggga
     2641 actgtaccgt ctgattccga agtggatgtc cttgtacgtg ggattgacaa tttaacactc
     2701 taatatataa aggagtactt tattaaacta aaactttcgt acttttctaa caacgtcaga
     2761 cagtaggttg atgtacttca tgtagtagtc ttgcatgtct tcatcttcga acttgcaagc
     2821 tgcttcccaa ccattccatg cagcgttgat tccgtccatc aaattaccaa tagtgacagt
     2881 cttcaaatca tcagttacaa ctccgttgga agcgcgagta gctaaagttg gggtgaggag
     2941 acctagagac tgagaaggag ttgccatttg agtttttttt tttgggggga gtgggggagt
     3001 cgccctttat atactagttg cttatgtaag caataaaaaa aaaataaaaa aaaaaccgat
     3061 ccttgcagtt tggatcgacg tccgatagtg tcggacattt atgtccgata ttcggacaac
     3121 ctatttaacc gagactgaga tcggatacaa tcagaccttt aggtctgatg tattcgaccg
     3181 acacctctga tactatccag acctttaggt ctgatagtat tagaccaacc gctcattctg
     3241 aagacagagt ctgaagaagg aagggggggg ggcggccctt aaccatatta ctttctgtca
     3301 agcacgaatg cagaatgggc gagcctccgc ggcgactgag cgggagatgc acgggaggtc
     3361 cagca
//
LOCUS       CruV-228                3369 bp    DNA     circular     24-FEB-2020
DEFINITION  Crucivirus-228 isolate 120_468_IR1_3296.
ACCESSION   CruV-228
KEYWORDS    .
SOURCE      Crucivirus-228
  ORGANISM  Crucivirus-228
            Unclassified.
REFERENCE   1  (bases 1 to 3369)
  AUTHORS   de la Higuera,I., Kasun,G.W., Torrance,E.L., Pratt,A.A.,
            Maluenda,A., Colombet,J., Bisseux,M., Ravet,V., Dayaram,A.,
            Stainton,D., Kraberger,S., Zawar-Reza,P., Goldstien,S., Briskie,J.,
            White,R., Taylor,H., Gomez,C., Ainley,D.G., Harding,J.S.,
            Fontenele,R.S., Schreck,J., Ribeiro,S.G., Oswald,S.A., Arnold,J.M.,
            Varsani,A., Enault,F. and Stedman,K.M.
  TITLE     Direct Submission
  JOURNAL   Submitted (24-FEB-2020) Biology, Portland State University, 1719 SW
            10th Ave, Portland, Oregon 97201, USA
COMMENT     Bankit Comment: ALT EMAIL:ide@pdx.edu
            Bankit Comment: TOTAL # OF SEQS:451
            Bankit Comment: GAP: est. length - alignment

            ##Assembly-Data-START##
            Assembly Method       :: MetaSPAdes v. 3.12; IDBA-UD v. 1.1.1;
                                     MEGAHIT v. 1.0.3
            Sequencing Technology :: 454; Illumina
            ##Assembly-Data-END##
FEATURES             Location/Qualifiers
     source          1..3369
                     /organism="Crucivirus-228"
                     /mol_type="genomic DNA"
                     /isolate="120_468_IR1_3296"
                     /isolation_source="Water"
                     /country="Norway"
                     /collection_date=2011
     CDS             1..483
                     /codon_start=1
                     /product="putative ORF"
                     /translation="MDREQRLQQIAAQQLTAMSSSTQGVLSPGSISASSNGLNPLQMV
                     HPTTPSSFPSPLMNLPNISGQNASFMQRILESLHNETQIMDQLNEVFEDIFNMISDIE
                     EQTGMLYYGQLPPQAFAQVLRRLIDFKLLLKHSGFQSITDADVNQVVSQLEKMSLSPF
                     *"
     CDS             543..1952
                     /codon_start=1
                     /product="Putative CruV Capsid Protein"
                     /translation="MPRRFQSGTQRRVPYARRYQARYAARSYVPRVAGRGAYTTSSRA
                     PYTRRRRPSTSPWSQVMRYAAKAAPFAGGMLGNYIAPGIGGVVGTAAGSLIAKIAGHG
                     AYNVKHNTVMFPDQVPLFATKEDGCIRMQHREFICDIVSSNAGTPTAFEALRFNIDPA
                     DPVTFPFLSQIACNFEEWRMEGLIFEFKTTSGNTTVNQALGTVVMATQYNSNANAFAN
                     KQAMEAYTFASSTVPSASVIHPVECDPSQTPSNGIFYTDCPGVQSGVQDLRWSQMGVF
                     NIATVGCPTNLTTLGELWCSYDIWLCKCRLITDTASRADHYLLTGTFTPVSPLGTAST
                     YSPSSDSFGSTTPFSSFNGTNNTLYISSSFSGNIAVTLSYFQSAVSINVDPTPAPGSG
                     ATPLNLLSNNTTNSSGDKQYGAGNGTMQRTFYYAISPDPVGVGVAGFGAEIVFAGGLY
                     ANVTHGDLIVNSIPADLIN*"
     CDS             complement(2055..3269)
                     /codon_start=1
                     /product="Putative Replication Associated Protein"
                     /translation="MEIEIESVKTEVTTEVDSNTKISTSIENKIREGKTSASRSKRLE
                     ARTLEKQCELLEADRKLNEYIALNIVQNGKTSKNVEDESAHRSYILTINNATLEEEKE
                     MIADNYQYIAYQWECESTVHIQAFLYYASPRKFRVMKNKYPRAHIEVVRNNKAAAAYC
                     IKEKTRIFGRGPFEWGTKPNQGARTDLDTLARDLINGKKMSEIREEEPGLYVKYFNGL
                     KNLAAELAPKRTEKPEVTWIWGDGGVGKTKYVIDKHGADNVYRKDMSKWWDKYKQQEA
                     ICIDDFNNSWDYTYFLNLLDRYQVNYEVKNGHAEINSKYIYITCEFHPKHFWGNQKIE
                     DEKNNKLVQVMRRIDLIKHITRTEETKIKWPREEGLIFEYPKEEKGICNKIVPNRKDK
                     NENKILENKRVV*"
     misc_feature    3196..3227
                     /note="stem-loop"
     misc_feature    3209..3217
                     /note="nonanucleotide"
ORIGIN
        1 atggatcgcg aacaacgtct tcaacagatt gctgctcagc aattaacggc gatgtcatca
       61 agtactcaag gcgtcttgtc tccaggatcg ataagcgcgt cgagcaatgg gctcaatccc
      121 ttgcaaatgg tacaccctac gaccccctca tcattcccat cacctttgat gaatttgccg
      181 aatatttcag ggcaaaacgc atccttcatg cagcgtatcc tagagtctct tcataatgag
      241 actcagatta tggatcaatt aaacgaagtt tttgaagata tcttcaatat gatctctgat
      301 attgaagaac aaaccggaat gttatattat ggtcaattac cccctcaagc ctttgctcaa
      361 gtcttaaggc gcctaattga ctttaaactc cttctcaaac attctggttt tcagtccatt
      421 actgacgctg acgttaatca agttgtctct caacttgaga aaatgtctct ctctcctttt
      481 taaaaaagat tttgcaacaa aatctcttaa agcttttttt tctattaata gaaaaaagaa
      541 aaatgcctcg tcgttttcaa tctggtactc aacgacgtgt tccctacgca cgtaggtatc
      601 aagctcgtta tgccgctagg tcatatgttc ctcgagttgc tggaagagga gcttacacca
      661 cttcatcaag agctccttat actcgtagaa ggagaccatc tacctctcct tggtctcaag
      721 ttatgagata tgcagcaaag gctgctcctt ttgctggagg aatgcttgga aattatattg
      781 ctccaggtat tggcggagtt gtaggcacag cagctggatc tcttattgct aagatagctg
      841 gacatggtgc ctacaatgtt aaacataata ctgttatgtt ccctgatcaa gtgcctcttt
      901 ttgcaacaaa agaggatggt tgtatcagaa tgcaacaccg agaatttatc tgtgatattg
      961 tttcatctaa cgcaggaact ccaacggctt ttgaagccct gagatttaat attgatcctg
     1021 ctgatcctgt tacattccct ttcctttctc aaattgcttg caattttgag gaatggagaa
     1081 tggaaggtct tatttttgaa ttcaagacta cttcaggaaa tacaactgta aatcaagcat
     1141 taggtactgt tgtcatggct actcagtaca acagtaatgc taatgctttt gctaacaagc
     1201 aggcaatgga agcatacaca tttgcttcgt caactgtgcc atctgcttct gttattcatc
     1261 ctgttgaatg cgatcctagt caaactccat caaatggaat tttctatact gattgcccag
     1321 gagtgcaatc tggtgttcag gatttgcgtt ggtcgcaaat gggagttttt aatattgcta
     1381 ccgtcggctg ccccaccaac cttactaccc taggtgagct gtggtgttcg tacgatatct
     1441 ggctctgcaa atgtcgtctt atcactgaca cggcgtctag ggctgatcat tatttgctaa
     1501 ctggaacttt cactccagtt agtcctcttg ggactgcatc cacttattct ccttcttctg
     1561 attcctttgg atcaacaact ccattttcat cattcaatgg aacaaataat acattgtata
     1621 tttcttcatc tttctccggt aacattgctg ttaccctatc gtatttccag tcagccgtga
     1681 gcattaatgt cgatcctact cctgcccctg gttcaggagc cactccttta aatcttctta
     1741 gcaataatac tactaacagc tctggtgata agcagtatgg tgctggaaat ggcactatgc
     1801 aaagaacttt ctactacgca atcagtcctg atcctgttgg agttggagtt gcgggattcg
     1861 gagcagaaat agtctttgca ggtggccttt atgccaacgt cactcatggt gatctgattg
     1921 ttaacagcat tcctgctgat cttattaatt aatgaactac cacgttcaac gaattgcgat
     1981 ggaggacgcg aagcggccga ctgagtgatt cactaaaaat cacattaaaa aaatatattg
     2041 catcaatata ttttttatac aactctttta ttttctaata ttttattttc atttttatct
     2101 tttctattag gaacaatttt gttgcaaatt cctttttcct cttttggata ttcgaaaata
     2161 agtccttctt ctcttggcca ctttattttc gtttcttctg ttcgagtgat gtgttttatt
     2221 aaatcaatac gacgcatcac ttgaactaat ttattatttt tctcgtcttc tatcttctgg
     2281 tttccccaaa aatgttttgg gtgaaattca cacgttatat atatatactt actgttaatt
     2341 tcagcatgtc catttttcac ttcataattt acttgatatc tgtctagtaa atttaggaag
     2401 taagtatagt cccagctatt attaaaatca tctatacaga tagcttcttg ttgtttgtat
     2461 ttatcccacc attttgacat atcttttcta tatacgttat ctgccccatg tttatctatt
     2521 acgtatttag tttttcctac accaccatct ccccatatcc atgtaacttc aggtttttct
     2581 gttctttttg gtgctaattc agctgctaga tttttcaatc cgttgaagta tttcacgtat
     2641 agccctggtt cttcttctct tatctcgctc attttttttc cattaatgag atcacgagca
     2701 agagtatcaa gatcagtcct tgcaccttga tttggttttg taccccattc aaaaggacct
     2761 cttccaaaga tccttgtctt ttcttttata caataagcag cagcagcctt attgtttcta
     2821 accacttcaa tatgtgctcg cggatattta tttttcataa ctctaaattt cctcggagat
     2881 gcatagtaga ggaaagcctg aatatgaact gtagattcac attcccactg ataagcaatg
     2941 tactggtaat tatctgcaat catttctttt tcttcttcta gtgttgcatt gtttatcgtg
     3001 agaatatatg atctatgtgc tgattcatcc tctacatttt tagatgtttt tccattttga
     3061 actatgttca atgcaatgta ttcattgagt tttctatctg cttctagcag ttcacattgt
     3121 ttttcaagtg ttcttgcttc gagtctttta ctccttgaag cagacgtctt tccttctcta
     3181 attttatttt cgattgaagt tgatatttta gtattactat caacttctgt cgtcacttct
     3241 gttttcacag attcgatttc gatttccatt ttttactcta aaaaatattt ttttactcta
     3301 aaataaatac aaaagttaaa acagaagtat aaaacagaaa attccctcac ttaaaattta
     3361 tgccaaaga
//
LOCUS       CruV-229                3372 bp    DNA     circular     24-FEB-2020
DEFINITION  Crucivirus-229 isolate 001541_virusbaton.
ACCESSION   CruV-229
KEYWORDS    .
SOURCE      Crucivirus-229
  ORGANISM  Crucivirus-229
            Unclassified.
REFERENCE   1  (bases 1 to 3372)
  AUTHORS   de la Higuera,I., Kasun,G.W., Torrance,E.L., Pratt,A.A.,
            Maluenda,A., Colombet,J., Bisseux,M., Ravet,V., Dayaram,A.,
            Stainton,D., Kraberger,S., Zawar-Reza,P., Goldstien,S., Briskie,J.,
            White,R., Taylor,H., Gomez,C., Ainley,D.G., Harding,J.S.,
            Fontenele,R.S., Schreck,J., Ribeiro,S.G., Oswald,S.A., Arnold,J.M.,
            Varsani,A., Enault,F. and Stedman,K.M.
  TITLE     Direct Submission
  JOURNAL   Submitted (24-FEB-2020) Biology, Portland State University, 1719 SW
            10th Ave, Portland, Oregon 97201, USA
COMMENT     Bankit Comment: ALT EMAIL:ide@pdx.edu
            Bankit Comment: TOTAL # OF SEQS:451
            Bankit Comment: GAP: est. length - alignment

            ##Assembly-Data-START##
            Assembly Method       :: MetaSPAdes v. 3.12; IDBA-UD v. 1.1.1;
                                     MEGAHIT v. 1.0.3
            Sequencing Technology :: 454; Illumina
            ##Assembly-Data-END##
FEATURES             Location/Qualifiers
     source          1..3372
                     /organism="Crucivirus-229"
                     /mol_type="genomic DNA"
                     /isolate="001541_virusbaton"
                     /isolation_source="Water"
                     /country="France"
                     /collection_date=2013
     CDS             1..1467
                     /codon_start=1
                     /product="Putative Replication Associated Protein"
                     /translation="MKFRNFVFTINNPTENDITLIKNCYEDGYIRYVHGGYEVGEKTK
                     TLHIQGHCNLHDQMRRKAIKKYFPRTEPQHMKADNVYDSINYTKKGEMLKSEWKKKGP
                     DGKPLREKGKDFGKNYQCAIELGKPPKQGERKDLDNIQKELLDGVPLHDIIWRCKNAQ
                     QQKYAENLYRCPQVPKPHRTWRTEAHVYWGKANTGKTYKARESAQKYADKHGYRVWYS
                     GENGKWFSDYNGQEVVVMEDFDDSQYTLGVLLRLLDNVPYRVGGKYQPEEEWLAKRVY
                     FSTNFDPQGWYGNCDPKRREALLGNENCENGRITFSRCFYNQRKDGRSTTIHLTEDDP
                     YEEDEISENSIELESFSEETPPDDYEIEIRDEDLNLEIDSKSVSEVGRVIYEPAETKK
                     IIVKNKSYIDPIEIDFRKKLNKNHSTDVDKILEKKNEILIKNEILYENNNKFCKNCGE
                     EKPEKYRIHDKNMNEIIQYKKLCEECNSEKNYEKQYRR*"
     CDS             1884..3299
                     /codon_start=1
                     /product="Putative CruV Capsid Protein"
                     /translation="MDKYMPATRRRYTTRKRYTGRTTSAYTRPTYKRRAPARRTYTKR
                     SSSTLSKIASTVGKPLVSGAMGTIGRAAGATLAGLPGAIAGGFIGDTLLPKLGQWTTN
                     ALSSVGDLGRSIIAKFGDYEIKQNSFLGKGPADMPDIVNPDPGGGFLIRRHEYLGDVI
                     SGTAGTFNVNNYFINPGQASTFPWLSQIAQNFEEWVCEGMYFEFRTMSSDALNSTNTA
                     LGQVIMAANYNATNANFTTKQAMENYEGGMSCKPSVNMMYFVECARNQTVLDDLYVRP
                     GPVPAGNDQRFYDLANFQIATNGLQASNVNLGELWVCYQISLRKPKMFSAFGFGNLFH
                     DNLRGSCSSAVPLGTSSALNSNVVNIPIVYTGTTMVFPISAVGQAFIGFWKWSNSAVT
                     SVMPTITFGAGATSFAIASTPNDGIAGIKEETLIYTFYFPGNSSVADRTITFGVAGTI
                     SVSQCRININQCTNAFDGFPE*"
     misc_feature    3149..3171
                     /note="stem-loop"
     misc_feature    3154..3162
                     /note="nonanucleotide"
ORIGIN
        1 atgaaattta gaaattttgt atttacaata aataacccaa cagaaaatga tatcacccta
       61 ataaaaaatt gttatgagga tggatacatt agatacgtac acggaggata cgaagttggt
      121 gaaaagacaa aaactctaca cattcaaggt cattgtaatc tacatgacca aatgagaaga
      181 aaggctataa aaaaatattt tccaagaaca gaacctcaac atatgaaagc tgataacgta
      241 tacgacagca ttaattacac caaaaaaggt gaaatgttga aatctgaatg gaaaaagaaa
      301 ggtcccgatg ggaaaccttt aagggaaaag ggtaaagatt ttggaaaaaa ttaccaatgt
      361 gctatcgaac taggtaaacc accaaagcaa ggtgaaagaa aagaccttga caatattcaa
      421 aaagaattat tagatggtgt acctcttcat gatataatat ggagatgtaa aaatgctcag
      481 caacaaaagt atgctgagaa tttatacaga tgtcctcaag ttcctaaacc ccatagaaca
      541 tggagaactg aagctcatgt ttactgggga aaagccaata caggaaaaac ttataaagcg
      601 agagaatccg cacaaaaata tgcggacaaa cacggatacc gggtttggta ttctggagaa
      661 aacggaaaat ggtttagcga ttataacgga caagaagtcg tagtcatgga agacttcgac
      721 gattcgcaat acacactagg agtgttatta agactcctag ataatgtacc atacagagta
      781 gggggtaaat atcaaccaga agaggaatgg ttagcaaaaa gagtgtactt ttcaactaat
      841 tttgaccccc aaggatggta tggtaactgt gacccaaaaa gaagagaagc tcttttaggt
      901 aacgaaaatt gtgaaaatgg tcgcataaca ttttcaagat gcttctataa ccaaagaaaa
      961 gatggtagaa gtactaccat tcatctaaca gaagatgatc cttatgaaga agatgaaatt
     1021 tctgaaaatt ctatagaatt agaaagtttc agtgaagaaa ctccaccaga tgactatgaa
     1081 attgaaattc gagacgaaga tttaaattta gaaatcgatt ctaaaagtgt ctcagaagtc
     1141 ggcagggtaa tatacgaacc tgccgagaca aaaaaaataa tagtaaaaaa caaaagttat
     1201 atagacccta tagaaataga ttttagaaaa aaattaaata aaaatcattc aacagatgtt
     1261 gataaaattt tagaaaaaaa gaatgaaatt ttaataaaaa atgaaatttt atatgaaaat
     1321 aataacaaat tttgtaaaaa ttgtggtgaa gaaaaacctg aaaaatacag aatacacgat
     1381 aaaaatatga atgaaattat tcaatacaaa aaactttgtg aagaatgtaa ttcagaaaaa
     1441 aattatgaaa aacaatatcg aagataacga aattaaaaaa tctgaactta ctaccgttaa
     1501 aatttcctac gatcgtcttc tcatctaggt aaacctatct tcaaatcctc tttacaaaaa
     1561 ttcacttacg taaattcatt tttatattcc gttcctatct gtaatgtttg atgggcgtgt
     1621 aatctgaaga gctagctatg cccatggtta gaaaaaccac ttggccgctt cattacgatg
     1681 aaaattaagg taatttcccg tcttgcgccg agaaaaaatt accttaattt taatcgtaat
     1741 tcattgccaa tggttttttc cgagtatggc cttttactgc ttacgcagag cgcctacggc
     1801 ctactatttc ttattctgaa atattaaacc aggggctatc taggccagtt ttgaaatatg
     1861 atttttattt tttgtaatct tagctggata aatatatgcc agcaacaaga agaagatata
     1921 caacaagaaa aaggtataca ggcagaacaa cgtctgctta tactagacca acatataaaa
     1981 gaagagctcc agctagaaga acttatacca aaaggtcaag ttcgacactg tcaaaaatag
     2041 catcaacggt aggaaaaccg ttagtatcag gagctatggg aaccatagga agagcagcag
     2101 gagcaacatt agctggtcta cctggggcaa ttgctggagg atttatagga gatactttat
     2161 taccaaaact aggacaatgg acgacaaatg cactgagttc tgtaggtgac ttaggaagat
     2221 caataattgc aaaattcgga gattatgaaa taaaacaaaa ctcatttcta ggaaaaggtc
     2281 cagcagatat gccagatata gtaaatcctg acccaggcgg tggatttcta ataagaagac
     2341 acgaatatct aggagatgtt atatcaggaa cagctggaac atttaatgta aataattact
     2401 ttattaatcc tggtcaagcc tcaacgtttc catggttatc acaaattgct caaaattttg
     2461 aagaatgggt atgcgaggga atgtacttcg aattcagaac aatgtcaagt gacgcactaa
     2521 acagtacaaa tactgcttta ggtcaagtaa taatggctgc aaattataat gcaaccaacg
     2581 caaacttcac tacaaaacaa gcaatggaaa actacgaggg aggcatgtca tgcaaaccat
     2641 cagtaaatat gatgtacttt gtagagtgtg caagaaacca gacggtacta gatgaccttt
     2701 atgtaagacc aggaccagtt ccagcaggaa atgatcaaag attctatgat ttagctaatt
     2761 ttcagatagc aactaacggt ttacaagcaa gtaatgtaaa cttaggtgaa ttatgggttt
     2821 gctatcaaat aagcttaaga aaaccaaaaa tgttttcagc ttttggattt ggcaacctat
     2881 ttcacgacaa tttgagagga agctgtagca gtgcagttcc attaggaaca tcaagcgcac
     2941 tcaactcaaa tgtagtgaat ataccaatag tatacacagg aacaacaatg gtatttccaa
     3001 tttctgctgt tggacaagca ttcataggat tttggaaatg gtcaaactca gctgtaacgt
     3061 cagttatgcc aacgataaca tttggggcag gagcaacgtc attcgctata gcttcgacgc
     3121 caaatgatgg aatagctgga ataaaagaag aaacactaat atatactttc tactttcctg
     3181 gaaattcaag tgttgcagat agaacaataa cttttggtgt agcagggact atttcggtct
     3241 ctcaatgtag aataaatatc aatcaatgca ctaatgcatt cgatggattc cctgaataaa
     3301 accaaaaatc acaaaaactt gaatttaatt attttttttt aaaaatttaa tgaccttaat
     3361 ataggtcaaa aa
//
LOCUS       CruV-230                3373 bp    DNA     circular     24-FEB-2020
DEFINITION  Crucivirus-230 isolate 001540_virusbaton.
ACCESSION   CruV-230
KEYWORDS    .
SOURCE      Crucivirus-230
  ORGANISM  Crucivirus-230
            Unclassified.
REFERENCE   1  (bases 1 to 3373)
  AUTHORS   de la Higuera,I., Kasun,G.W., Torrance,E.L., Pratt,A.A.,
            Maluenda,A., Colombet,J., Bisseux,M., Ravet,V., Dayaram,A.,
            Stainton,D., Kraberger,S., Zawar-Reza,P., Goldstien,S., Briskie,J.,
            White,R., Taylor,H., Gomez,C., Ainley,D.G., Harding,J.S.,
            Fontenele,R.S., Schreck,J., Ribeiro,S.G., Oswald,S.A., Arnold,J.M.,
            Varsani,A., Enault,F. and Stedman,K.M.
  TITLE     Direct Submission
  JOURNAL   Submitted (24-FEB-2020) Biology, Portland State University, 1719 SW
            10th Ave, Portland, Oregon 97201, USA
COMMENT     Bankit Comment: ALT EMAIL:ide@pdx.edu
            Bankit Comment: TOTAL # OF SEQS:451
            Bankit Comment: GAP: est. length - alignment

            ##Assembly-Data-START##
            Assembly Method       :: MetaSPAdes v. 3.12; IDBA-UD v. 1.1.1;
                                     MEGAHIT v. 1.0.3
            Sequencing Technology :: 454; Illumina
            ##Assembly-Data-END##
FEATURES             Location/Qualifiers
     source          1..3373
                     /organism="Crucivirus-230"
                     /mol_type="genomic DNA"
                     /isolate="001540_virusbaton"
                     /isolation_source="Water"
                     /country="France"
                     /collection_date=2013
     CDS             1..1530
                     /codon_start=1
                     /product="Putative Replication Associated Protein"
                     /translation="MDVKTKPLTREQREARDAALIAHAFDNAYATAALPAEASVRKKK
                     GSTVIHDAAVPEDTDTSPYKTGLRSRGWVFTWNNPVVPPGYTDFVDYYKAWEPQVSYL
                     VVGNEHAPTTGTPHHQGAVWFKNQRTWSACRSTIKCFWAPAKGSGEQNFNYSNKESIL
                     IEYGERKQQGNRSDLEDVRNLIQTGGQMKEVTERASNLQAIKIGQLYLQHHPELHVRR
                     NWEMEIYWIKGPSGAGKTRWCYEQAPMAHFSSRNLQWWDGYSDQEDVIVDDFRGDFCT
                     YHELLRICDRYPYRVQVKGSSCELLAKRIFFTSCMLPTEVYNTREDIWQLLRRITAVY
                     EFCSEDGSANVYKHVNKVDSTKVEQLGKLPPRTLELEVKSVPKVLPGFGASVPPTDVE
                     LIIKNQPASSQKSGGNKSRVGSNIILPSPASPDNLPKGMCPLCRDNYCNGRCGTVIFH
                     CNGCGSAEEEHKCPAYDIECPHEHIFADDFGNETCEQCGAFLENDESVELIEDDGFNP
                     L*"
     CDS             complement(1567..2982)
                     /codon_start=1
                     /product="Putative CruV Capsid Protein"
                     /translation="MPRPAAAKVVYVQAPPKKKRVVRRAGGVGMIRGSGDYYQYGPFG
                     GKRYSGSPTYNNPGIWGSAGRLAGGLAGKALAGPAGAAIGSKIGSYAHYIGKIFGSGD
                     YVTAADNVKSNVLVNESQIPRFSTQSGKNTVHIRHREFLTDIISSGTAGAFNLQSFAI
                     QPGLPSSLPWLSNVCGTTFQQYRINGMVFEFRSMSSDALNSTNTALGSVVMATDYDSK
                     DASFSTKAQMENTEFGVSCKPSSCMIHAIECARSQTAVSELYIRNAAVPSGADPRLYD
                     MGNFQIATVGFQGTSVNAGELWVSYDITLFKAIQLKPGAGLLQYHREIALSAGTTPLL
                     ADTSVHAQPQFDNIGCTVDATNKILSLPVGLTIGTRFLITYAARGASTASVTVPAVAF
                     SQGISSHNGFQNGLSGGYSGPQAVNTSTTNFLLGAFEVTAASSVSAPPIITFSTITTP
                     PGATCLADLYIVQMNAADGLA*"
ORIGIN
        1 atggacgtca agactaaacc tttgactcgg gagcaacgtg aagcaagaga tgctgcacta
       61 attgctcatg cctttgataa cgcatatgct acggccgccc taccggctga agcctccgta
      121 cgtaaaaaga aaggttctac cgtcatccat gatgctgccg ttccggaaga cacagacact
      181 agcccttata aaacggggct acgctcccga ggttgggtat tcacctggaa taatccagtc
      241 gtacctcctg gatacaccga cttcgtcgat tattacaaag cttgggaacc ccaagtttcc
      301 taccttgttg taggaaatga acacgcgccc actactggga cgcctcatca ccaaggtgct
      361 gtttggttca aaaaccagcg tacatggtcc gcttgccgat ctacgatcaa atgcttttgg
      421 gcacctgcta aaggttctgg agagcaaaac tttaactact ccaataagga gtccatactc
      481 attgagtacg gtgagcggaa gcaacaaggc aaccgctctg acctcgaaga tgtcagaaat
      541 ctaatccaaa caggtggaca gatgaaagaa gttactgaac gagctagtaa ccttcaagca
      601 ataaagattg gtcaacttta cttgcaacac cacccagaac tccatgtccg cagaaattgg
      661 gaaatggaga tctattggat taaaggccct tccggtgccg gtaaaaccag atggtgttat
      721 gagcaagctc cgatggctca cttctcatct cgcaatctac aatggtggga cggatactcc
      781 gaccaagaag acgtcattgt tgatgacttc cgtggagact tctgcaccta tcatgaactg
      841 ctccgcatct gcgatcgata tccctatcga gttcaagtaa aaggctcatc atgcgagctt
      901 ttagccaaac gtatattctt tacgagctgt atgcttccta ccgaggtgta taacacccgc
      961 gaggatattt ggcaactcct acgacgcatt actgccgtct acgagttctg ttccgaggat
     1021 ggtagtgcaa atgtttacaa acatgtgaac aaagtggatt caactaaagt ggaacaactt
     1081 ggcaagctgc cacctcggac tttggagctg gaggtaaagt cggtacccaa ggtcctcccg
     1141 ggatttggtg caagtgtgcc accaactgat gttgaattaa ttattaaaaa tcagcccgcc
     1201 tcatctcaaa agtcgggggg taataaaagc cgggtcgggt cgaatattat tctcccctcg
     1261 ccggcatccc ccgacaattt acccaagggt atgtgcccgc tttgccggga taattattgc
     1321 aatggccgtt gcggtacggt catatttcac tgcaatggtt gcgggtccgc agaagaagaa
     1381 cataaatgtc ctgcttatga tatcgaatgt cctcacgaac atatcttcgc agatgatttc
     1441 ggcaacgaaa catgcgaaca atgtggagct ttcctcgaaa acgacgaaag tgttgagtta
     1501 atcgaagatg atggatttaa tccactttga taaacacaaa aagatttttt tcgttccttt
     1561 aagaactcaa gcaagaccat cagcggcatt catctgtact atatacagat ccgcaagaca
     1621 agtagctcca ggaggagttg tgatggtaga gaatgtaatg attggaggtg cagacacgga
     1681 cgatgccgcg gtcacctcaa atgcacccaa caagaaattt gttgttgatg tattcacggc
     1741 ctgaggccca gaatatccac cactgagccc attctggaac ccattatgtg atgatattcc
     1801 ttgagagaaa gccacagctg gtactgtcac tgatgccgtt gacgcgcctc gcgctgcata
     1861 agtaatcaga aatcttgttc caattgttaa accaactgga agactcaaga ttttattggt
     1921 tgcatctact gtgcatccaa tattatcaaa ctgcggctgt gcatgaacac tcgtatctgc
     1981 cagcagagga gttgttcctg cagaaagagc aatttcacga tgatactgaa gcaggcctgc
     2041 gccaggcttc aactgaattg ctttaaacag ggtaatgtca taagacaccc agagctcacc
     2101 tgcgtttaca ctagtaccct gaaatccaac ggtcgcgatc tggaaatttc ccatatcgta
     2161 gagccttgga tctgcaccgg atggcacggc ggcatttcga atatacaatt cgctgactgc
     2221 tgtctgactg cgcgcgcact caattgcatg aatcatacag gaagaaggct tgcacgacac
     2281 accaaactcg gtgttttcca tctgggcttt tgttgaaaag ctggcatctt tgctatcgta
     2341 gtcagtagcc atcactacag atcctaatgc cgtattcgtg ctattaagcg cgtcagaaga
     2401 catcgaacga aattcgaaaa ccataccgtt aatccggtac tgctgaaatg ttgttccaca
     2461 aacatttgac aaccaaggaa gagaagatgg caaacctggc tgaatcgcaa acgattgcag
     2521 attaaatgcc ccagctgtgc cagaactaat aatgtcagta agaaattcgc gatgacgaat
     2581 atgtactgta ttcttaccac tttgggtaga gaaacgtgga atctgtgatt cattcacgag
     2641 tacatttgac ttgacattat ctgcagcagt gacatagtca cctgacccaa atatctttcc
     2701 gatataatgg gcataggatc caattttgga tcctatagct gcacctgcag gccctgctaa
     2761 tgctttaccg gcaagtccac cggccaagcg tccagcactt ccccaaatgc ctggattatt
     2821 gtaagtcgga ctcccagagt accgcttacc accaaaagga ccatattgat aataatctcc
     2881 gcttccgcga atcattccaa cacctcctgc gcgtctcacg actcgctttt tctttggcgg
     2941 ggcctgcaca tataccacct tcgctgctgc gggtcggggc atgatgtcgc aatgttagtg
     3001 gatatatttt tctctaatat aaataatatg ggtcagcaat gctgcccctc cacaaagtat
     3061 gtaatcacat actaaggcca ggccttcaag taggtctgct cgctacagaa cacgtctgat
     3121 ccgtagtttc tcgcatcgac aacatcttct tgagatgctg ctatcgcagt agtcttgatt
     3181 agcctaggct tgcatgcgcg ccgtggctgc gcaagatcat tgatagcagg ggcaagccct
     3241 tgtttcaact cgaaaccaag ctgcatacgg gacttgttcc gtaggtcgga cccattatcc
     3301 gacctgtccg tgttagatca atgattaatt tttactttta gggattttac acccttgtta
     3361 tacttgagta aac
//
LOCUS       CruV-231                3375 bp    DNA     circular     24-FEB-2020
DEFINITION  Crucivirus-231 isolate 001539_virusbaton.
ACCESSION   CruV-231
KEYWORDS    .
SOURCE      Crucivirus-231
  ORGANISM  Crucivirus-231
            Unclassified.
REFERENCE   1  (bases 1 to 3375)
  AUTHORS   de la Higuera,I., Kasun,G.W., Torrance,E.L., Pratt,A.A.,
            Maluenda,A., Colombet,J., Bisseux,M., Ravet,V., Dayaram,A.,
            Stainton,D., Kraberger,S., Zawar-Reza,P., Goldstien,S., Briskie,J.,
            White,R., Taylor,H., Gomez,C., Ainley,D.G., Harding,J.S.,
            Fontenele,R.S., Schreck,J., Ribeiro,S.G., Oswald,S.A., Arnold,J.M.,
            Varsani,A., Enault,F. and Stedman,K.M.
  TITLE     Direct Submission
  JOURNAL   Submitted (24-FEB-2020) Biology, Portland State University, 1719 SW
            10th Ave, Portland, Oregon 97201, USA
COMMENT     Bankit Comment: ALT EMAIL:ide@pdx.edu
            Bankit Comment: TOTAL # OF SEQS:451
            Bankit Comment: GAP: est. length - alignment

            ##Assembly-Data-START##
            Assembly Method       :: MetaSPAdes v. 3.12; IDBA-UD v. 1.1.1;
                                     MEGAHIT v. 1.0.3
            Sequencing Technology :: 454; Illumina
            ##Assembly-Data-END##
FEATURES             Location/Qualifiers
     source          1..3375
                     /organism="Crucivirus-231"
                     /mol_type="genomic DNA"
                     /isolate="001539_virusbaton"
                     /isolation_source="Water"
                     /country="France"
                     /collection_date=2013
     CDS             1..1371
                     /codon_start=1
                     /product="Putative Replication Associated Protein"
                     /translation="MPPSSATKSRKWCFTMFWDGVAPGAAYFDDWEVEYLYVGKEICP
                     ESKREHYQGYVRFGNARALGGVTKLFIHKLKLPSGRPGYPGHWEACKGTEAQNVKYCA
                     KEDDLVIEFGESETDLAPDKHQGKRNDIIAVRNMIIDGARMRDVVLHSASYQGIKIAE
                     TMLKYIEPSRSWKPEVYWIYGPTGVFKTRYASEACADPWISGIDGKWFEGYDAHEDVI
                     FDDFREDFCKFHILLRLLDRYPYRVEVKGASRQFLAKRIFITSCHPPESTFRGAPEED
                     ILQLGRRIDHVVYMPTAGVAYSRPGSVIGLEPSVRFVFPALRPAQKVWGNTTDAPHPT
                     EAERPALTGPQTGLICDTKCFEPHGPDFVCDDCENDCSYPEPSESGDHARSLDEQWIC
                     PCGFSESECSIVCLEEACPCFMEKPIKEEAPQLPLSLPPPLIIPQSPIPGRPPAIIYT
                     RPRRAI*"
     misc_feature    970..995
                     /note="stem-loop"
     misc_feature    975..983
                     /note="nonanucleotide"
     CDS             complement(1474..2865)
                     /codon_start=1
                     /product="Putative CruV Capsid Protein"
                     /translation="MLMPRLAVAPRRKLAVAPRKRVPVKVTKAVYVKPARSLKAAAPK
                     VYKNASKSGAFGTAGGLLGTLLGTALGGPGGGMMGGTLGKLGGAALSSIFGHGDYQVT
                     NGPAIKENNLLSNGGNAANIPQFGTGKVACNFKHREYIGDIISSATAGAFKIDSFNVN
                     PGLPTTFPWLSGVVGANFQQYRLNGMAFEFRSMSGDALNSVNTALGSVIMSSDYDSAD
                     AAFTSKQQMENTEYGVSCKPSVNMLHAIECARSQTSISELYVRNFGVPTGTDIRLYDM
                     CKFYIASVGCQGTSVNLGELWVTYDVDAFKAIEQAPGYSNPVSEYTLTTVDATHALGA
                     SQTTVVEQIGLVLAYVAGTSYINFPLQSMQVGAKYLVTYKIYGSSTASVATCTYVPTG
                     GMVALNANASLLQNPDTATATSAVRAVSEVFTYNGTGTVAVPPQIAITGGVVPTAIIV
                     ASLIVVQIGAAFV*"
ORIGIN
        1 atgcctccct cgtcggccac taaatcgcga aaatggtgtt tcaccatgtt ttgggatgga
       61 gtcgcacccg gcgctgccta ctttgacgac tgggaggtcg aatatcttta cgttggtaaa
      121 gaaatttgcc ctgaatctaa acgcgaacat taccaaggtt atgtccgatt tggtaacgcc
      181 cgcgcccttg gtggggttac taaattgttt attcacaagc ttaaactccc ctctggccgc
      241 cccggctatc caggtcattg ggaagcttgt aaaggcactg aagcgcaaaa cgtaaagtat
      301 tgcgccaaag aagatgacct tgtcatcgaa tttggtgaat ctgaaactga tctggctcca
      361 gataagcatc aaggtaagcg caacgatatt attgctgttc gcaatatgat tatcgatggc
      421 gctcgtatga gagacgttgt ccttcatagc gctagttatc agggaatcaa gattgctgaa
      481 actatgttaa aatacatcga accttcccgt tcctggaagc ccgaagtgta ttggatttac
      541 ggtccgactg gcgtcttcaa gactcgttac gcgtccgagg cctgcgccga cccttggatc
      601 tctggaatcg acggtaaatg gttcgagggc tacgacgccc acgaagacgt catcttcgac
      661 gactttcgtg aagacttttg caagtttcac atcttgttaa gattgcttga tcgttacccg
      721 taccgggtcg aagtcaaggg agcgtcacgc cagttcctgg ctaaacgcat cttcatcacg
      781 agttgccacc cgccggagtc cacgttccgc ggagctcctg aagaagatat cctccagctc
      841 ggccgtcgga ttgaccatgt ggtctatatg cctaccgctg gggtagcata ctcacggccc
      901 gggtcggtga tagggttaga accctccgtt cgctttgtgt ttccggcact tcggccggca
      961 caaaaagtct ggggtaatac tacggacgcc ccccatccta cggaagcgga acgccctgcc
     1021 ctaacgggcc cccagactgg tttaatttgt gatacaaagt gtttcgaacc acatggtccg
     1081 gattttgttt gtgatgattg cgagaacgac tgctcttatc ccgaaccatc tgaaagtggc
     1141 gaccatgctc gctcacttga cgaacaatgg atatgccctt gcggattctc cgaaagtgaa
     1201 tgctcaattg tttgtctaga agaagcatgc ccatgcttca tggaaaaacc gataaaggaa
     1261 gaagctccgc aactaccttt atcactaccg ccgcccctaa ttatccctca atctccgatt
     1321 ccgggacgac cgccggctat tatttataca cgaccacggc gagctatcta aagatttgct
     1381 cttgtttccg cggcgagcca tctaaagatc tgctcttatt cccgcgttag tgttataaaa
     1441 cacaaaaagt tttttgtgaa catacaaagc tacttacacg aaagcagctc cgatttgcac
     1501 aacaatcagc gaagccacga tgatggctgt aggcacaaca cctccggtga ttgcaatctg
     1561 aggaggcact gcaacggtac cagtaccatt gtatgtaaac acctcactca ccgcacgaac
     1621 tgcagaagtt gcagttgcag tgtctgggtt ctggagcaaa gatgcattcg cattgagcgc
     1681 gaccattcct cccgtaggta cgtatgtaca tgttgctaca cttgcagtag aagatccata
     1741 gatcttatac gtaaccaagt acttagctcc cacctgcatt gattgaagcg ggaaattgat
     1801 gtatgaggtc cctgccacat acgccaatac aagaccaatt tgctcaacaa ctgtggtttg
     1861 cgatgcccct aacgcgtgag tggcatcaac agtggtcaac gtgtattctg aaacaggatt
     1921 tgaataccct ggagcttgct caatagcctt gaacgcatct acatcatacg tgacccacag
     1981 ctcaccaagg ttcactgagg ttccctggca tcctacggac gcgatgtaga acttgcacat
     2041 gtcgtacagc cgtatgtcag taccagttgg cacaccaaag tttctgacat acaactccga
     2101 aattgacgtt tgcgaccttg cgcactcaat tgcatgcaac atgttaacag atggcttgca
     2161 cgacactccg tactccgtgt tctccatctg ttgttttgaa gtgaacgcag catcggcaga
     2221 atcataatcg gacgacatga taactgatcc taatgcagtg ttcactgagt tcaacgcgtc
     2281 tcccgacatc gatctgaact caaacgccat tccattgagt cgatattgct gaaagtttgc
     2341 tcccacaaca cctgacaacc aagggaatgt tgttggtaaa cctgggttta cattaaaaga
     2401 atcaatcttg aaagcccccg ctgttgcgga cgagataata tctccgatat actctctgtg
     2461 cttaaagttg caagccacct tgcctgtacc aaattgagga atattggcag cattcccacc
     2521 gtttgacaac agattgtttt ctttaatggc tgggccattc gttacctgat aatctccgtg
     2581 cccgaagata ctagataacg cagccccccc gagtttgcct aatgtgcctc ccatcatgcc
     2641 tcctccgggt ccacccaaag ctgttcctag aagagttcct aggagccccc ctgccgttcc
     2701 gaatgctccg ctcttgctcg cattcttgta caccttcgga gccgctgcct ttagcgagcg
     2761 tgcaggtttg acatacacag cttttgttac tttcaccggt acccgctttc gcggtgctac
     2821 tgccagcttt cgccgaggcg ctactgctaa tctgggcatc agcaatcggg tatactatcc
     2881 gatttttttc ctctaaaata ttattatatc catgaagtgc tgcgtaactt tccagccaac
     2941 gcatagctac ttcatgttgc atctcgagag tatcccaagc tgacatagcg aattatttac
     3001 tgctcgcaca aaatatttac aaatattttt gcccttttaa tagacagtta tggcttcggg
     3061 attcatgaac aggtctactc gctatacacc ggatgctggg gcattagtgt atacgatcat
     3121 atttccccca cggggaacta agtggagcac tcagggttga ggttgccccc ttcgggggca
     3181 gtgcaaccta agagtttgca cttggcctag tcaggcatat gaccctttag tatccaccac
     3241 cgttccacta tgcgcataca gtgcataggt cgggccatgt attcaccctt gcccctataa
     3301 ctggtcatta atttcgattt gaactttttg ttccgctatg attatactgt ccgctctcgc
     3361 caacagctac tcctc
//
LOCUS       CruV-232                3383 bp    DNA     circular     24-FEB-2020
DEFINITION  Crucivirus-232 isolate 120_147_SvL2_3298.
ACCESSION   CruV-232
KEYWORDS    .
SOURCE      Crucivirus-232
  ORGANISM  Crucivirus-232
            Unclassified.
REFERENCE   1  (bases 1 to 3383)
  AUTHORS   de la Higuera,I., Kasun,G.W., Torrance,E.L., Pratt,A.A.,
            Maluenda,A., Colombet,J., Bisseux,M., Ravet,V., Dayaram,A.,
            Stainton,D., Kraberger,S., Zawar-Reza,P., Goldstien,S., Briskie,J.,
            White,R., Taylor,H., Gomez,C., Ainley,D.G., Harding,J.S.,
            Fontenele,R.S., Schreck,J., Ribeiro,S.G., Oswald,S.A., Arnold,J.M.,
            Varsani,A., Enault,F. and Stedman,K.M.
  TITLE     Direct Submission
  JOURNAL   Submitted (24-FEB-2020) Biology, Portland State University, 1719 SW
            10th Ave, Portland, Oregon 97201, USA
COMMENT     Bankit Comment: ALT EMAIL:ide@pdx.edu
            Bankit Comment: TOTAL # OF SEQS:451
            Bankit Comment: GAP: est. length - alignment

            ##Assembly-Data-START##
            Assembly Method       :: MetaSPAdes v. 3.12; IDBA-UD v. 1.1.1;
                                     MEGAHIT v. 1.0.3
            Sequencing Technology :: 454; Illumina
            ##Assembly-Data-END##
FEATURES             Location/Qualifiers
     source          1..3383
                     /organism="Crucivirus-232"
                     /mol_type="genomic DNA"
                     /isolate="120_147_SvL2_3298"
                     /isolation_source="Water"
                     /country="Norway"
                     /collection_date=2012
     misc_feature    join(3368..3383,1..22)
                     /note="stem-loop"
     misc_feature    1..9
                     /note="nonanucleotide"
     CDS             367..2172
                     /codon_start=1
                     /product="Putative CruV Capsid Protein"
                     /translation="MSLMPKAQYMKLSPAAKRKLKARLARLGVGAKVGRSTASDMYGK
                     MRGQGDYYRAGGSTGFRKVKDASRGIGAQLGRNIGGRFGFGDEGAKLGGYAHSLFKQL
                     TGFGDYKIKKNSIMMGTDPPSFGTLGRGTIIRHREFLGDVISSPTAGAFQLASFPIQP
                     AYTNTFPWLGQIACCYEQYQLRGMVFEFKSTSSDALNSVNTALGTVVMVTEYNVTNPV
                     FTNKAAMENHEYCTSGKPSGNILHPIECDPSQTPNNCYFTRQAIPLSVTAPTIGSILP
                     VAGQDARQSEVGLFQIATVGCQGTNVNLGELWVTYDVELLKAQVRSNGSAGGPFSDHY
                     FMPTGSITTAAPFGTTAPVLRVGSNCGSSINTATNSIILPQDVFPNRYMIQYVVFGTT
                     ATVIATQMTIAYGANVAPSLIWNGDLLNDFQTTAGDTSHFHTVNIVVQVGASATSTTN
                     NVTFSLGTMPTGVVSGDVWVTYLPDNILTAPKWSVVRPPSHCDRKEDKTEEADDSEDD
                     EKSDIDYEKLFKMLLKQQSKSSPEGKVEKKVDEDEDEYEAMLELVKLRKAAASSSASP
                     LPPPPTSLPPTTPTTASAASVPPMPVMPPRSSSRK*"
     CDS             complement(2203..3075)
                     /codon_start=1
                     /product="Putative Replication Associated Protein"
                     /translation="MSDELFDSADKNDFQNRYWIPTIWIENLPKELKIASAEDLVQYT
                     INHVKLKKKVEACCTRFERCPTTGKLHAHLFIKLKKSDRLWAALIAAFGPTAIKVLYA
                     RRDGENAKSYSVKDETSEGEPAFHNIDPESIKPWGILGGKAGTVQKGTKPKDTKTPEE
                     KAISFAEAKMMKEAAEQKLIRAACSSKNWAKFDFSFFIRKRAAEHEAAVEEAEMRLKH
                     LSAEIDSRLIIEYGKRYGYDPISAAELYYDELKHYERPKVEGVIRPSELPAGVPRLPS
                     ELAAGVPRLPLPMA*"
ORIGIN
        1 tagtattacc aaggacacac acgcttgtcg tgtgtaaatg tgtcatctag ggtttcccta
       61 gtgtaccatt tgctagcttc tgctagcaca ttctgataca cgtgtagcag atgggacgta
      121 ccataagatt ggtacgtggt cttattgtag ctccggtgag cgcatcaacg cgtgcgtttg
      181 tgcctatgtg tgtaccttta tggtaccgct tatcccgtga agttatgata cgtgcaataa
      241 gaggttgagt gcccctgcag agaccttcta cccacttatg agtgcccttt atggtctatg
      301 tcatcgtaca gagcaattaa atgagctttg atttactata atcaaacctg ctcaaatacg
      361 cccatcatga gtcttatgcc caaagctcag tatatgaagt tatctcctgc cgctaagcgt
      421 aagctgaagg cacgcctagc tcgtctaggt gttggagcga aagttggccg ctcaactgca
      481 tctgacatgt atggtaagat gcgaggccaa ggcgattatt atcgcgccgg tggttctacc
      541 ggctttcgta aagtcaaaga tgcttctcgt ggcatcggtg ctcagctagg ccgcaatatt
      601 ggcggtcgct ttggctttgg cgatgaagga gctaagttgg gtggttatgc ccactctctc
      661 ttcaagcagc ttactggctt cggtgattat aagatcaaga agaactccat catgatgggt
      721 actgacccgc cgtctttcgg cactttgggt cgcggtacga ttattcgtca ccgtgagttc
      781 ttgggtgatg tgatttcgtc gcccaccgct ggagcctttc agctcgccag ctttcctatc
      841 cagcctgcgt ataccaatac gtttccatgg ttgggtcaga tcgcatgctg ttacgaacag
      901 tatcagctgc gtggtatggt ctttgagttc aagtctacat cttcggatgc tctgaactca
      961 gtcaacactg ctcttggtac tgtggtgatg gtcactgaat acaatgtgac caaccctgtg
     1021 ttcactaaca aggctgccat ggagaaccat gagtactgta cctctggcaa gccctctggc
     1081 aacattctcc atcccattga gtgtgacccc agtcagactc cgaacaattg ttacttcact
     1141 cggcaggcaa ttcctctgag tgtaacagct cccaccattg gttctatcct tccggtggcg
     1201 ggtcaggatg ctcgtcagtc tgaggtcggt ctgtttcaga tcgctaccgt tggttgtcaa
     1261 ggaaccaacg ttaatctggg tgaactatgg gttacctatg atgttgagct tctcaaggct
     1321 caggttcgct cgaacggttc ggcaggtggt cctttctcag accattattt catgccaacc
     1381 ggcagcatca caactgctgc cccttttggg acgactgctc ctgttttgcg agttggttct
     1441 aattgtggct cgagtatcaa cacggccacc aactcgatca tcctacctca ggatgtcttt
     1501 ccgaaccggt atatgattca gtatgtcgtg ttcggaacaa cggctaccgt tattgcaacg
     1561 cagatgacta tcgcgtatgg tgctaatgtt gcgccatccc ttatctggaa cggtgatcta
     1621 ctcaacgatt tccagaccac cgcaggtgat acgtctcatt tccatactgt gaatattgtg
     1681 gttcaggtcg gagcttctgc gacgtctacg actaacaatg tgacgttctc tctgggtact
     1741 atgcctactg gcgtagtcag tggtgatgtc tgggtaacat atctcccaga caacattctc
     1801 accgcaccta agtggtcggt ggttcgtcct ccttctcatt gcgatcgcaa ggaggacaag
     1861 accgaagagg ccgatgattc agaggacgat gagaagtccg atatcgacta tgagaagctc
     1921 ttcaagatgc tccttaagca acagtctaag tcttctcccg aaggtaaggt ggagaagaaa
     1981 gtcgatgagg acgaggatga atacgaggcc atgttggaac tcgtgaagct ccggaaggca
     2041 gcagcctcaa gctccgcgtc tccactccct ccccctccta cctccctccc tcctactact
     2101 cctactactg cgtctgctgc ctctgtgccg ccaatgcctg ttatgccgcc ccggtcttct
     2161 tcacggaagt gaaatctata tgtctcatac gagacatatg tcttatgcca ttggtaatgg
     2221 cagtcggggt actcccgctg ctaactctga tggcagtcgg ggtactcctg ctggtaactc
     2281 tgatggccta atcactcctt cgactttagg cctttcatag tgcttgagct catcgtagta
     2341 gagctcagct gctgatatgg gatcatagcc atatcttttc ccatattcta tgattagacg
     2401 actgtctatc tcggctgata ggtgcttaag tctcatctca gcttcttcca ctgcagcctc
     2461 gtgctcagcg gctcgtttcc gaataaagaa cgagaagtcg aacttggccc agttcttgga
     2521 cgaacatgca gctcgaatga gcttctgctc tgcggcttcc ttcatcatct ttgcctcggc
     2581 aaaggatatg gctttctcct ccggagtctt ggtatcctta ggcttcgtac ccttttgtac
     2641 ggtgccagcc ttcccaccta ggatacccca cggcttgatc gattctggat caatgttgtg
     2701 aaacgcgggc tctccctctg aggtctcatc cttcacagag tagctcttag cgttttcccc
     2761 gtccctacgt gcatacaaga ctttgattgc agtaggtccg aaggccgcga taagagccgc
     2821 ccatagacgg tcggacttct tgagtttgat gaagaggtga gcatgaagct tacctgtagt
     2881 ggggcagcgc tcgaaacgag tgcaacaggc ctccaccttc ttcttgagtt tcacgtgatt
     2941 gatggtgtat tgcaccagat cctcagctga ggcaatcttg agttccttag gcaggttctc
     3001 tatccaaatg gtagggatcc aatacctgtt ctggaagtcg ttcttgtcgg ctgaatcgaa
     3061 gagttcatcg ctcattgctg tttgctattt gatgctgagc tcagtgaatc aagcgatgaa
     3121 tcaattttgg tcgggagcct tagcttaacc gtaccataag agctcggctc gtcttatgtc
     3181 atcgtacata agatttcata gatcatgtat ccatgtcgat gtcgtcgcgc agcgacccat
     3241 actaatctca tatcccaatt tatgtggtat gagtatgaga tgtagcgatt tattgctact
     3301 tcggcatttc tgccccagag attgctgctc tggaggtata catagcaatc ttgcaatgtt
     3361 ctaacgcgtg tgtgtccttg att
//
LOCUS       CruV-233                3384 bp    DNA     circular     24-FEB-2020
DEFINITION  Crucivirus-233 isolate 001576_virusbaton.
ACCESSION   CruV-233
KEYWORDS    .
SOURCE      Crucivirus-233
  ORGANISM  Crucivirus-233
            Unclassified.
REFERENCE   1  (bases 1 to 3384)
  AUTHORS   de la Higuera,I., Kasun,G.W., Torrance,E.L., Pratt,A.A.,
            Maluenda,A., Colombet,J., Bisseux,M., Ravet,V., Dayaram,A.,
            Stainton,D., Kraberger,S., Zawar-Reza,P., Goldstien,S., Briskie,J.,
            White,R., Taylor,H., Gomez,C., Ainley,D.G., Harding,J.S.,
            Fontenele,R.S., Schreck,J., Ribeiro,S.G., Oswald,S.A., Arnold,J.M.,
            Varsani,A., Enault,F. and Stedman,K.M.
  TITLE     Direct Submission
  JOURNAL   Submitted (24-FEB-2020) Biology, Portland State University, 1719 SW
            10th Ave, Portland, Oregon 97201, USA
COMMENT     Bankit Comment: ALT EMAIL:ide@pdx.edu
            Bankit Comment: TOTAL # OF SEQS:451
            Bankit Comment: GAP: est. length - alignment

            ##Assembly-Data-START##
            Assembly Method       :: MetaSPAdes v. 3.12; IDBA-UD v. 1.1.1;
                                     MEGAHIT v. 1.0.3
            Sequencing Technology :: 454; Illumina
            ##Assembly-Data-END##
FEATURES             Location/Qualifiers
     source          1..3384
                     /organism="Crucivirus-233"
                     /mol_type="genomic DNA"
                     /isolate="001576_virusbaton"
                     /isolation_source="Water"
                     /country="France"
                     /collection_date=2013
     CDS             1..1176
                     /codon_start=1
                     /product="Putative Replication Associated Protein"
                     /translation="MSDAFEHLSANLVPLRGSMPLEQVEEKLSVETKESPVTPLGMEE
                     KREIIASEAEAVDDKSRKKNWVFTWNNYTVEDVKTVNEDWLKLSDMRCVVYEPEIGKQ
                     GTPHLQGFFCFDKLKSFKQVKEMINKRVWFSQMKKPIEASLKYCSKDKGTIYLGEVPM
                     SQKEKGTHGIKGKESGKDAPDIWADILTDIKKGISRKELMEKYMSIYAVYHAGIEKYI
                     EMFTPRVPFNIEKTHESFYPWQRQLLDIIDLKADARSVIWIYSKRGNTGKTAMFKHLL
                     CMNETFEPFMNATSRDLSCAWKGGDAIFDLARDDGGVPMNYAFMEMIKNGYAFSAKYD
                     SKCKKALDMTKERFVICFSNSRPDVSKLSIDRWAIFRIKMDLTLHLEDPYCFDENDI*
                     "
     CDS             complement(1847..3244)
                     /codon_start=1
                     /product="Putative CruV Capsid Protein"
                     /translation="MPQVRAAPRRRLAPAPRVARRPAVTRSRVAPLPARSAPVARRVV
                     PKQRSQAGNAFRSGGSTLGGMLGSALGGPAGGAVGGMLGRGAGAMFSKLFGHGDYAVT
                     NADSLKENNIVLSNAAQAPMFGSGRVACKFVHREFLGDVFSSSTANTFKIDSYPINPG
                     QGTTFPWLSGVVGAKFQQYRINGMTFEFRSMSSDALNSTNTALGSVIMSTDYDSADDT
                     FASKQEMENTEYGISCKPSINMLHGIECARSQTPVSELYIRAFDVPTAKDIRLYDLGR
                     FSIASVGCQGTNVNLGELWVSYDIDAFKAIEQVPGYLIPFSQYSLTGALATVPLGTAH
                     PQLVAGGSPNSIDLTLTANKVSWPIDIEVGSIWRLSLQYVGSTSAALTTPVLTSGNGM
                     FFNGTTIFKTPSSTTTQVAVEADLQYQGGATPNALPYFNIANFVWTTAVVQSAILMST
                     QVSGLYPGVYTSQVE*"
ORIGIN
        1 atgtctgatg catttgaaca tctatctgcg aacttggttc ctctgcgcgg cagtatgcca
       61 ctggaacaag tcgaagaaaa gctttcagtc gagacaaaag aatcacccgt tactccttta
      121 gggatggaag agaaacgaga gattattgcg tccgaagctg aagctgtcga tgacaaatca
      181 agaaagaaga actgggtctt cacttggaac aactacaccg tcgaagatgt aaaaacagtc
      241 aatgaagatt ggctcaagct gtctgacatg cgatgtgttg tttacgaacc tgagataggc
      301 aagcaaggca cgcctcatct tcaagggttc ttttgctttg ataagctcaa atccttcaaa
      361 caagtgaagg aaatgatcaa taaaagagta tggttttctc agatgaagaa acccattgaa
      421 gctagcctta aatactgctc aaaggataaa ggaacaattt atcttggaga agtgccaatg
      481 tctcagaaag agaaaggaac acatggtatt aagggcaaag agtctggaaa agatgcgccc
      541 gatatatggg cagatatttt aacagacatt aagaaaggaa taagccgtaa agagcttatg
      601 gagaagtaca tgtccatata tgcagtttat catgcaggca tagaaaaata catcgaaatg
      661 ttcactccac gagtaccttt caatatcgaa aagactcatg agtctttcta tccgtggcaa
      721 cgccaactac tcgatattat cgatctcaaa gcagatgcaa gatctgtcat ttggatctat
      781 tcaaaacgag gaaatactgg taaaacagct atgtttaagc atttactctg tatgaatgaa
      841 acctttgaac cattcatgaa cgcaacctct cgcgacctat cttgcgcctg gaaaggtggc
      901 gatgccatct ttgatctagc aagagatgac ggaggcgtcc ccatgaacta cgccttcatg
      961 gaaatgatta agaatggata cgcatttagt gctaaatacg attcaaaatg taaaaaagct
     1021 cttgatatga ctaaagagcg ttttgtaatt tgcttctcaa attcacgtcc agacgtgtca
     1081 aaattgtcga tagatcgatg ggcaattttt agaatcaaga tggaccttac attacattta
     1141 gaggatccct attgctttga cgaaaatgat atctaggtct agaataattt tagttgaaat
     1201 ttaagctctt tttttgatat agagctgaaa taaaccccat agagataaat gatattgata
     1261 tctagggggg gtcctgtaat actatagcgg accccccgat atcatgcatg tttgcacaaa
     1321 tctccaccag ataagacatt acatgtatta ccctcacgcc ccacggacga ctagaggcat
     1381 ggacgacaaa gcgccccaaa caagtgtggg ggcgcttcgg cgcccacgcc cctcgacgtc
     1441 cgcgcggcgt ccctgagaac atacataaag gttacaccaa acacgcgcgc cctttttttg
     1501 ttgattttat gtacacgggc catcgtttcc gatcgtgttc ttcaaatccc ggatcgcaag
     1561 cggccagatg aacacttggg agcaattaca actatgttga attatttatt tattatttac
     1621 atataaacat cgtttccgat cgtgttcttc aaatcctgga tcgcaaacga ccagatgaac
     1681 acttaaaacg aaaacttata aaacattgtt tcacatcatg tttcattttt tgatcaaaaa
     1741 atatagcctt taggctcaat ttgtttgttt catgcacctt ttactgtgca tcacctatca
     1801 ggacttcata ccttctaggt tcattctttt cagaaacata acaaaattac tcaacctgtg
     1861 atgtatacac accaggatac aatccagaaa cctgggttga catcaatata gcagattgca
     1921 ctacagcagt agtccacaca aaattagcaa tattgaaata agggagtgca ttaggagtag
     1981 caccaccctg atactgaaga tcagcttcaa cagcaacttg cgttgtcgta gaactcggag
     2041 tcttaaaaat ggtagtacca ttgaaaaaca tgccattacc agaggtaagg acaggtgttg
     2101 taagtgcagc tgaagtagaa cctacatact gcaaagaaag ccgccaaata gaaccaactt
     2161 cgatatctat tggccaacta accttattcg ctgtcaatgt caaatcaatt gaattcggcg
     2221 aaccaccagc aaccaactga gggtgtgctg taccaagagg cactgttgcg agagcaccag
     2281 taagagaata ttgagaaaac gggataagat acccaggtac ctgttcgata gccttaaacg
     2341 catcgatatc atatgaaacc cataactcac caagattcac attcgttcct tgacatccta
     2401 cagaagcgat cgaaaatcga ccgagatcat acaacctgat atccttcgct gtaggaacat
     2461 caaaagcccg aatatagagc tcagacacag gagtctgaga tctcgcacat tcgatcccat
     2521 ggagcatatt gatcgatggc ttacaagaaa taccatactc ggtattctcc atttcctgct
     2581 ttgaagcaaa ggtatcatca gctgaatcat agtctgtaga cataatcact gaaccaagag
     2641 ctgtgttggt agaattcaac gcatcggacg acatacttcg aaattcgaat gtcataccat
     2701 taatacgata ctgttgaaac ttagccccaa ccacacccga taaccacgga aaagtagttc
     2761 cttgaccagg attgatagga taactatcaa tcttaaatgt attggcagta gatgagctaa
     2821 acacatcacc caggaattcg cgatgcacaa acttacatgc aacacgtcct gaaccaaaca
     2881 tcggagcctg agcggcatta gaaagaacaa tattgttctc cttaagtgaa tcagcattag
     2941 taactgcata atcaccatga ccgaatagct tactaaacat ggcaccagca ccacgtccaa
     3001 gcattccgcc aacagctccc ccggcagggc ctcccagagc agaacctaac attcctccaa
     3061 gtgtagaacc accagatcga aatgcattac cagcctgtga ccgttgcttc ggcacaacac
     3121 gtcgagcaac aggagcagaa cgagcaggta aaggagccac acgagaacgt gtaacagcag
     3181 gacgtcgagc tactcgagga gcaggcgcga gacgacgcct tggagcggct cgaacttgag
     3241 gcataaacct tgagttaagt tttgacttag cagacgggta taccctatgc acaacaactt
     3301 cctcaaaata atattaatta attagctacc aattaattaa attcataaga atgatatgta
     3361 ggtacaatat acctgcacta aatc
//
LOCUS       CruV-234                3385 bp    DNA     circular     24-FEB-2020
DEFINITION  Crucivirus-234 isolate SR1_25544.
ACCESSION   CruV-234
KEYWORDS    .
SOURCE      Crucivirus-234
  ORGANISM  Crucivirus-234
            Unclassified.
REFERENCE   1  (bases 1 to 3385)
  AUTHORS   de la Higuera,I., Kasun,G.W., Torrance,E.L., Pratt,A.A.,
            Maluenda,A., Colombet,J., Bisseux,M., Ravet,V., Dayaram,A.,
            Stainton,D., Kraberger,S., Zawar-Reza,P., Goldstien,S., Briskie,J.,
            White,R., Taylor,H., Gomez,C., Ainley,D.G., Harding,J.S.,
            Fontenele,R.S., Schreck,J., Ribeiro,S.G., Oswald,S.A., Arnold,J.M.,
            Varsani,A., Enault,F. and Stedman,K.M.
  TITLE     Direct Submission
  JOURNAL   Submitted (24-FEB-2020) Biology, Portland State University, 1719 SW
            10th Ave, Portland, Oregon 97201, USA
COMMENT     Bankit Comment: ALT EMAIL:ide@pdx.edu
            Bankit Comment: TOTAL # OF SEQS:451
            Bankit Comment: GAP: est. length - alignment

            ##Assembly-Data-START##
            Assembly Method       :: MetaSPAdes v. 3.12; IDBA-UD v. 1.1.1;
                                     MEGAHIT v. 1.0.3
            Sequencing Technology :: 454; Illumina
            ##Assembly-Data-END##
FEATURES             Location/Qualifiers
     source          1..3385
                     /organism="Crucivirus-234"
                     /mol_type="genomic DNA"
                     /isolate="SR1_25544"
                     /isolation_source="Water"
                     /country="New Zealand"
                     /collection_date=2012
     CDS             1..897
                     /codon_start=1
                     /product="Putative Replication Associated Protein"
                     /translation="MSRSRNWCYTINNPTNEEIETCDVIECSYNVFGFEVGNEGTPHL
                     QGYIEFKDAKTLRSAKKSLGGRAHLEQRRGTATEASDYCKKDNLFIENGSISNQGKRN
                     DLDTLAELVKTKGIKGVIEEAPGMFIKFGKNIERLSEFYMKPRTTAPKVIWLWGKAGV
                     GKTKLAHENIDNEDIYIWNQTKWWTGYRQQSRVIIDDYSFDNSDHHYRYLLQLIDRYS
                     IQVETKGGMVYFNSPEIYITCEFPPTEFWQVGNKLDQITRRISEIVEVKDVICKESND
                     IITLVPPELEPSDNIDEDWLDI*"
     CDS             complement(1940..3283)
                     /codon_start=1
                     /product="Putative CruV Capsid Protein"
                     /translation="MPRPAAKAPKGTKKDTKASEYYRKQKKSKYTRKTPRAPLAAASS
                     TESFGESAGKLLGGLAQKAIKALTGFGEYTTPGFGIKQNVLIEHGDPPTVQNAGKEFI
                     VRHREYLGDIYSGVAGAVGAPAASPFTIQAFPINPGLQLSFPWLANLAARFEEYELQG
                     MLYEYKSMYSDAAVQTGGSLGTVIMATSYNAAKPNFPSKIEMENYEFAQSAKPSVSMI
                     HPIECQPGQNVFNSQYIRLGPLLDGEDIKTYDAGKFQIASQGIPVSGSALSLGELWST
                     YQICFRKPKVTNYIDSGYARLSQTAAVQPAGGSPFAPLISWSTITDNIGIKLVNERTF
                     TLPLFNNTRTYQINLICGDPNNLNTQAAAVGYFPTTATNVTNGVISIMPQIPSPAFTQ
                     ATGSLNISSSAFLLTITTNPISPGQTLCTVQLPNGCGATLSYIKNLIINAVPTSS*"
ORIGIN
        1 atgtctagat ctagaaattg gtgttacact atcaataatc caacaaacga agaaattgaa
       61 acatgcgatg ttatcgaatg ttcgtacaat gtattcggtt ttgaagttgg taacgagggt
      121 accccccacc ttcaaggata tatagagttt aaggacgcaa aaactcttag gtcagctaaa
      181 aaatcgctag gaggccgagc tcatcttgaa caacgaagag gaacagctac tgaagcatct
      241 gattattgca aaaaagataa tctttttatt gaaaatggtt ccatttccaa tcaaggtaag
      301 cgaaatgacc ttgataccct agctgaacta gttaagacca aaggtattaa aggtgtcatc
      361 gaagaagcac ctggtatgtt cataaaattc ggcaaaaaca tcgaacgttt atccgaattt
      421 tatatgaagc ccagaaccac agcgcctaaa gttatatggt tatggggtaa ggcgggtgtt
      481 ggtaagacca aactcgctca tgaaaatata gataacgaag atatatatat ttggaatcaa
      541 actaaatggt ggacagggta ccgtcaacaa tctagagtca taatcgatga ttattctttt
      601 gataatagtg atcatcacta taggtattta ttgcaactta tagatagata ctctatccaa
      661 gttgaaacaa aaggtggtat ggtctatttt aatagtcctg aaatttacat tacatgtgaa
      721 tttccgccca ccgaattttg gcaagttgga aacaaacttg atcaaattac tcgacgaatt
      781 tctgaaattg tcgaggttaa agatgtaata tgtaaagaat ctaatgatat tatcactctc
      841 gtcccccccg aactcgagcc cagtgataat attgatgagg attggcttga catttgaaat
      901 atttaactat cttatgtgga aaatcataaa aaacaaaaga taataaatga tctttttttc
      961 tgattactta tatgaatgtt ttgaacgcgt atagacaggt gttcctcccc ccctgttcct
     1021 gcaatcggtc gtaaactccc tcaattggac ggggcccccc ctgaattgta aacaattctt
     1081 cacggaggct gtcacgccgt aaacggtcga atagcgcgtg gtaaaaaaat gcacagaagt
     1141 ttttacacag aagtggcagg taatactaga aaaaattaat tttttctttt tctgccacca
     1201 aaacacaagt gtaaaaaata attttaccag gcttggctcc caagactgaa attatatatt
     1261 ccgtgagtgt gttaattgat atataaccat acttagtgat atatagtttt tgtcgattcc
     1321 gtaattgggg tatatgaacg aggcctaaag gcgctcgccc caatctacga attaaaaaaa
     1381 ttatggatca aaaagtatgt ttataaaatc atataaacat cctcccaaaa atataatttt
     1441 gagtcgtatg gtagatgata gtcacaaata attacattat ttatacgtta tttatacata
     1501 ctttttgttg atcttgcatg agcgttagtt cgataaagtc aagcgcttct tgtcgagtaa
     1561 tttgtctttc tagaccgtta tctttgacgg taacgttttc aatgcaccaa gttattgatg
     1621 cttcgaagcg tttgattgct aaagatattc ttaaattttg acgtatgttt ggtcgacggt
     1681 aacgaccttc aggcgtcttt actaaaattt gatttcgttt gtttgattgc atgattgaca
     1741 taatataata tcgaaaaaaa ataaaatagg caattaatta ccagatttgg taccatgttt
     1801 tttgtgaaga attttcgtga gaagtatcag gaatttcttg taaatataat tcattacatg
     1861 aagtattttt tggtgataga gtgttggtgt agttatcaat tatattatca atgatgtttt
     1921 tcttgacttc gtcttcgatt catgatgatg taggtaccgc gttgatgatt aaatttttaa
     1981 tgtatgacaa tgttgctccg caaccattag gtaactgaac ggtacaaagt gtttgtcctg
     2041 gtgatatagg gttagtggta atggtcaata agaacgctga actagaaata ttgagtgacc
     2101 cagttgcttg ggtaaatgca ggggagggta tttgcggcat aatactaatg acaccattag
     2161 tgacattagt ggctgtggta gggaaatatc ctaccgcagc ggcttgagta ttaagattat
     2221 ttgggtctcc gcatatcaaa tttatttgat aggtgcgagt attattgaaa agtggtaggg
     2281 taaatgttcg ctcattgacc aatttgatac cgatattgtc agtaattgtt gaccaactaa
     2341 tgagtggtgc gaagggtgaa cctccggcag gttgaactgc ggctgtttgg gacagtctcg
     2401 cgtatccaga atcaatataa tttgttacct tgggtttacg gaagcaaatt tgataagtag
     2461 accatagttc acctagtgat aaagcactac ctgacacagg tataccttga gatgcaattt
     2521 ggaattttcc agcatcatat gttttgatat cttcgccatc aaggaggggt cctagtctaa
     2581 tatattggct attaaaaaca ttttgtccag gttgacattc gatagggtgg atcatgctca
     2641 ccgatggctt cgctgattgg gcaaattcat aattttccat ctcaattttt gatggaaagt
     2701 taggtttcgc agcgttataa cttgtggcca tgataactgt acctagtgaa cctccggttt
     2761 gcaccgcggc atctgagtac atacttttat actcatataa cataccttgt aactcatatt
     2821 cttcgaatct ggcggctaaa ttagcgagcc atgggaacga taattggagt ccaggattga
     2881 ttggaaatgc ttgaatagta aagggtgatg cggcaggggc acctactgct ccagctacgc
     2941 cggaataaat atcacctaaa tattcacgat gtcggacaat aaattctttg ccggcatttt
     3001 gaacagtggg cgggtcgcca tgttcaatta gtacattttg cttgatacca aaaccgggtg
     3061 ttgtgtattc tccgaatcca gtgagggcct tgattgcttt ttgagccaat ccacctagca
     3121 acttgcctgc actttctccg aaactttcgg tagatgaggc cgctgcgagt ggtgcccgag
     3181 gtgtcttacg agtatattta gatttctttt gctttcgata gtactcggat gctttcgtat
     3241 ccttcttagt tcctttggga gctttagctg ctggtcgagg cataatatta ttaggtagct
     3301 aacaatactt aggggaaata taaattttaa ttagttaata attaaattaa gtaaaactta
     3361 cgtgtaagtt atgttggtgt aaaaa
//
LOCUS       CruV-235                3386 bp    DNA     circular     24-FEB-2020
DEFINITION  Crucivirus-235 isolate 001534_virusbaton.
ACCESSION   CruV-235
KEYWORDS    .
SOURCE      Crucivirus-235
  ORGANISM  Crucivirus-235
            Unclassified.
REFERENCE   1  (bases 1 to 3386)
  AUTHORS   de la Higuera,I., Kasun,G.W., Torrance,E.L., Pratt,A.A.,
            Maluenda,A., Colombet,J., Bisseux,M., Ravet,V., Dayaram,A.,
            Stainton,D., Kraberger,S., Zawar-Reza,P., Goldstien,S., Briskie,J.,
            White,R., Taylor,H., Gomez,C., Ainley,D.G., Harding,J.S.,
            Fontenele,R.S., Schreck,J., Ribeiro,S.G., Oswald,S.A., Arnold,J.M.,
            Varsani,A., Enault,F. and Stedman,K.M.
  TITLE     Direct Submission
  JOURNAL   Submitted (24-FEB-2020) Biology, Portland State University, 1719 SW
            10th Ave, Portland, Oregon 97201, USA
COMMENT     Bankit Comment: ALT EMAIL:ide@pdx.edu
            Bankit Comment: TOTAL # OF SEQS:451
            Bankit Comment: GAP: est. length - alignment

            ##Assembly-Data-START##
            Assembly Method       :: MetaSPAdes v. 3.12; IDBA-UD v. 1.1.1;
                                     MEGAHIT v. 1.0.3
            Sequencing Technology :: 454; Illumina
            ##Assembly-Data-END##
FEATURES             Location/Qualifiers
     source          1..3386
                     /organism="Crucivirus-235"
                     /mol_type="genomic DNA"
                     /isolate="001534_virusbaton"
                     /isolation_source="Water"
                     /country="France"
                     /collection_date=2013
     CDS             1..1266
                     /codon_start=1
                     /product="Putative Replication Associated Protein"
                     /translation="MPASLASKSRKWCFTMYWDGKEPGKDYFDDWECDYMYVGKEICP
                     ESRREHYQGYIRFTNPRALAGVRKLFTHTVLTPAGRPGYPGHWEPCKGTEEQNVHYCA
                     KDEDLVIEWGDREADLAIDKHQGKRNDLIAVREMIRDGANMRDVIENCNSYQGIKIAE
                     TMLKHMEAPRCWVPEVYWLYGPTGTGKSKMAFDQCVDPWVSAKDGKWFDGYDGHEDVI
                     FDDFRHDFCPFHILLRLLDRYPFRIEHKGGCRQFLAKRIFITSCHSPDKVYPLLGEDL
                     AQLGRRITNIIFVPRLGEYLGIPGSEIGLEPSAGFVFPERGTEQKVGGNTTEVTDDKS
                     PLAPALTRPPPGLIESKCVVQHGADMVCDGCENDELLAAFAEEEASSPVVLHTPEALG
                     EVQVVAPRTMPPAFHPAPPPRRKGGPLTW*"
     CDS             complement(1368..2924)
                     /codon_start=1
                     /product="Putative CruV Capsid Protein"
                     /translation="MPRKAVANYTRPGYVRPPSYTPAQVAKVKAAAAFARANPRVQAL
                     GGYARARPRAVARPRAAARSSGNSALGTAIGSALGMALGGPAGGAMGGMIGNAGGGFL
                     SKIFGHGDYAVSNASSLKQNNLVLSNSANIPQFGTGKVATKFVHREFLGDVFSSSTAN
                     TFKIDSYAINPGQSETFPWLAGVVGAKYQQYRINGMTFEFRSMSADALNSTNTALGSV
                     IMSTDYDSADVVFSSKQQMENTENGVSCKPSVNMMHGIECARAQTPVNELYIRAFDVP
                     SGKDIRLYDLGRFSIASTGCQGTNVNLGELWVSYDIDAFKAIEQVPAYLTPFSTFALQ
                     GMTATAPLGTSRTAFPTDLEDYDQIGLTFVSENKVTWPLDTQVGANFFINYVLVGAAA
                     AALTQPVITTGGGMKISTVVDFIAPASAVTTAYIMGQWAIQYQGGGTPAAPPYFNIAD
                     FTWAPAGGVLEADFLVTQVSGNFASGTYTLTTGGAPPVGVEDPEDTDYVRPPEEQKKA
                     LSVSHSVRRR*"
ORIGIN
        1 atgcctgcgt cactcgcttc gaagtcacgt aaatggtgct ttactatgta ttgggacggc
       61 aaagaacctg gtaaggatta ctttgatgat tgggaatgcg attatatgta cgttggtaaa
      121 gaaatctgcc ctgaatctag gcgcgaacac taccaaggat acatccgttt cactaatcct
      181 cgggctctcg ctggtgttcg aaaactcttc acacatactg tgctgactcc tgccggacgc
      241 cctggctatc ccggacattg ggaaccttgc aaaggtacag aagagcaaaa cgttcactat
      301 tgtgccaaag acgaagatct cgttattgaa tggggtgacc gtgaagctga tctagctata
      361 gataagcatc aaggtaaacg taacgacctt atcgcggttc gcgaaatgat tcgtgacggt
      421 gctaacatgc gagatgttat tgaaaactgc aactcttacc aggggataaa gattgctgaa
      481 actatgctaa agcatatgga agcaccacga tgttgggtgc ctgaagttta ctggttatat
      541 ggccctacgg gaactggtaa aagcaaaatg gcttttgatc aatgtgttga tccttgggtt
      601 tccgctaaag acggtaaatg gtttgatggt tatgatggtc acgaagacgt gatctttgat
      661 gactttcgtc atgatttctg cccctttcac attctgctca ggttgctaga tagatatcct
      721 ttcaggattg aacataaagg tggctgccgc cagtttcttg cgaaacgcat ctttattacg
      781 agctgccact cgcccgataa ggtataccca cttctcggtg aagaccttgc ccaacttggg
      841 cgaaggatta caaatattat atttgttccg cggctcggtg aatacttagg tattcccgga
      901 tcggagatag ggctagagcc ctccgctggc tttgtgtttc cggaacgtgg cacggaacaa
      961 aaagtcgggg gtaatactac ggaggtcacg gatgataaat caccgctcgc tcctgcgcta
     1021 acgcgccccc ctcctggttt gattgaaagc aaatgtgtgg tacaacatgg tgctgatatg
     1081 gtctgcgacg gctgtgaaaa tgatgagcta ctcgctgcat ttgcagagga agaagcttct
     1141 tccccggttg tattacatac accggaagcc cttggtgaag tgcaagttgt cgctccgcgc
     1201 actatgccgc cggcatttca tccggctcca ccacctaggc gtaaaggagg acctcttact
     1261 tggtaagaat ttgctatcgc cacgcttgtg gctcccgcat gggttaaaaa gtccacaaaa
     1321 aattttttgg tggcttagcc agctatgttg ttacacaacc gctttggcta tcgtcttcgg
     1381 acagaatggg agacagaaag agcctttttt tgctcttctg gagggcgaac ataatcagta
     1441 tcctccggat cttcaacgcc tactggaggt gctcctcccg ttgtaagagt ataagtacca
     1501 ctggcaaaat ttccactaac ttgcgttact aagaaatctg cttccaacac gcctccggct
     1561 ggcgcccaag taaaatcggc gatgttgaaa tatggcggtg ccgcgggcgt ccctccgcct
     1621 tgatactgga ttgcccactg acccataata taggcagttg tcacggctga tgccggagca
     1681 ataaagtcca caactgtgct gatcttcatg cctcctccgg tcgtgatcac tggttgtgtc
     1741 aacgcagctg ccgcagcgcc tactagcaca tagttgataa agaaattggc acctacttgc
     1801 gtatccaaag gccaagtcac tttgttttct gacacaaatg taagacctat ctgatcgtaa
     1861 tcctctaggt ctgttggaaa tgcagtccga gacgttccta acggagccgt tgctgtcatt
     1921 ccttgaaggg caaatgttga gaaaggagtt aggtacgcag gtacctgctc aattgctttg
     1981 aatgcatcga tatcataaga tacccacagc tctccaaggt tcacattcgt tccctggcag
     2041 cctgtcgaag cgattgaaaa ccgtcccaaa tcatagaggc gaatatcctt gcctgacggc
     2101 acgtcaaacg ctcgaatata cagctcattc actggcgttt gagctcgtgc acattcgatg
     2161 ccatgcatca tgttgactga aggtttgcac gacaccccat tttcggtgtt ctccatctgt
     2221 tgttttgacg agaaaaccac atcagctgaa tcatagtctg ttgacatgat cactgaacca
     2281 agagctgtat ttgttgaatt taacgcatcc gctgacattg atcggaactc aaaggtcata
     2341 ccattgattc gatactgctg gtacttagca ccgacaacgc cggctaacca agggaaagtc
     2401 tctgactgtc ctggattgat cgcataactg tcgatcttaa atgtgttagc tgtagacgag
     2461 gaaaacacat caccaagaaa ctcgcgatgc acgaatttgg tggccacctt gccagttccg
     2521 aactgcggta tgtttgctga atttgataat acaaggttgt tttgtttcaa cgaacttgcg
     2581 ttactaacag cataatctcc atgtccgaag atttttgata gaaacccacc tccggcgttc
     2641 cctatcattc cacccatcgc acctcctgca ggtcctccta aggccatccc aagggctgac
     2701 ccgatggctg tacccagcgc tgaattaccg cttgatcggg cagcagctct aggacgagct
     2761 acagccctcg gtctagcacg agcataacca cctaaagctt gtactctagg attggctcga
     2821 gcaaatgcag ctgcagcttt aacctttgcc acctgcgccg gcgtatatga cggcggacgt
     2881 acataccctg gtcgggtata attggcaact gctttgcgag gcatcgcgtt gatgggtata
     2941 ctacccgatt atattcttct aatacaatat tatatggcgc atggctatat agccagcgca
     3001 tgtcttcctc atgctgtcgc tctcgagtgt cccacactga catcttatgt tatatttttt
     3061 tataactttt ttatattatg caaggtgcgt cgctgccaat agggtctgct cgctacctgc
     3121 ccggtctggc gttcttcgaa agatacgacg aacaccccct tggggtactg ctatcgcagt
     3181 agtttcactt agccttgctt gctttgcgcg cgtggctgcg caaaatcatt gatagcaggg
     3241 ggcaagccct tgtcgtccta ttctcggcca aggcatactt gacacgagtc aggtaggtcg
     3301 gaccacactg tttgctccta cctcttgctg ctattatata ttttatttta gacttcttgt
     3361 tccgggcact ctataccctg ctcgtt
//
LOCUS       CruV-236                3391 bp    DNA     circular     24-FEB-2020
DEFINITION  Crucivirus-236 isolate 120_1377_IR2_3297.
ACCESSION   CruV-236
KEYWORDS    .
SOURCE      Crucivirus-236
  ORGANISM  Crucivirus-236
            Unclassified.
REFERENCE   1  (bases 1 to 3391)
  AUTHORS   de la Higuera,I., Kasun,G.W., Torrance,E.L., Pratt,A.A.,
            Maluenda,A., Colombet,J., Bisseux,M., Ravet,V., Dayaram,A.,
            Stainton,D., Kraberger,S., Zawar-Reza,P., Goldstien,S., Briskie,J.,
            White,R., Taylor,H., Gomez,C., Ainley,D.G., Harding,J.S.,
            Fontenele,R.S., Schreck,J., Ribeiro,S.G., Oswald,S.A., Arnold,J.M.,
            Varsani,A., Enault,F. and Stedman,K.M.
  TITLE     Direct Submission
  JOURNAL   Submitted (24-FEB-2020) Biology, Portland State University, 1719 SW
            10th Ave, Portland, Oregon 97201, USA
COMMENT     Bankit Comment: ALT EMAIL:ide@pdx.edu
            Bankit Comment: TOTAL # OF SEQS:451
            Bankit Comment: GAP: est. length - alignment

            ##Assembly-Data-START##
            Assembly Method       :: MetaSPAdes v. 3.12; IDBA-UD v. 1.1.1;
                                     MEGAHIT v. 1.0.3
            Sequencing Technology :: 454; Illumina
            ##Assembly-Data-END##
FEATURES             Location/Qualifiers
     source          1..3391
                     /organism="Crucivirus-236"
                     /mol_type="genomic DNA"
                     /isolate="120_1377_IR2_3297"
                     /isolation_source="Water"
                     /country="Norway"
                     /collection_date=2011
     CDS             1..873
                     /codon_start=1
                     /product="Putative Replication Associated Protein"
                     /translation="MKNKKLALSGSTIDGNTNPIVETPLLKKKKEKRCNAVKNWCFTY
                     NNYCVENIQSLLALCKEFTEKYFFAEEEGENKTPHLQGFISLLKKGRPCEIFKAILNE
                     EGKHPIHWESMKGSMPENIKYCSKEGGKFYKSSNVRLIPKLNIITNLHPWQIFVESLV
                     KEPCTDDRVIHWFYDLVGNKGKTAMTKYLCYHYGAMLVEGKKKDILFGIQNAEDTDIF
                     IFDFSRSIENYVSYDAIESVKNGIFFSTKYESKMILRNSPHIIIFANFLPEEEKLSKD
                     RWRIYDLSKSENLI*"
     CDS             complement(1329..2720)
                     /codon_start=1
                     /product="Putative CruV Capsid Protein"
                     /translation="MPARKRASSKKKRVTVRAPRSTVKRYAPKASGGGAYTLENPGPF
                     GKAGQALGGIVGSHLGGRALGSALSTGGRYLGHKIGKFFGSGAYGYNSSTMSPADQLP
                     KFSNDHGDDSICITHREYLGDLITSNTIGGFKLTTFPINPASTQSFPWLCNIAQPNFQ
                     QYRFEGLIFSFQSFSADALNSTNTALGSVFAAVNYDYTDENFTNRNQIENSDWSMSCK
                     PSESMLIPVECAPRQTGMGGLLYVVNDNNIPVNADPKMYFLGKLSVGTTGFQASNINI
                     GSLYVTYKIRLYKKIMTPPLSNALIATFVREGSTAGANVCGTALYGGLIVNNNCDSIG
                     ITFPTSNKIRMNKNRLIQGQRYLFVHQWSGVNTVGIKPPDFSLNTSSGCIGSPNTMNN
                     YQTSYNNAPDVASTGATLIAVQTVEITDDSIDSDIEYSPTGFVPPGACLYNIEIYQIC
                     GIALNRIGFYDGN*"
     CDS             2933..3319
                     /codon_start=1
                     /product="putative ORF"
                     /translation="MDKLELQGKIDYCNIKMIDVHDVIDKIEDKIMICATPESFSLLS
                     SKLRDMILDLRLRLMHFEIMFDSIIDEVKEGDMITYCTSSETESPTACGDIEGYNFTV
                     HGADSEYRDTTDDYVRKNKKSKPFKN*"
ORIGIN
        1 atgaaaaata aaaagttggc tctgtctggc tctactattg atggtaatac taacccaata
       61 gtagagaccc ctcttcttaa aaagaagaag gaaaaaagat gtaatgccgt caagaactgg
      121 tgttttacat acaataatta ttgtgtggaa aacattcagt ctctcttggc tctctgtaaa
      181 gagtttacag agaaatattt tttcgccgaa gaagaaggag aaaataaaac gccacattta
      241 caaggtttca tttctttatt aaagaaagga agaccctgcg aaatttttaa agcaatactt
      301 aatgaagagg ggaagcatcc aattcattgg gagtcaatga aaggaagcat gcccgaaaac
      361 attaagtatt gctctaaaga aggcgggaag ttttacaagt cttctaacgt tagattaata
      421 ccaaagttga atattattac taatcttcac ccttggcaaa tcttcgtaga gagtcttgta
      481 aaagaaccct gcacggatga cagagtcatc cattggtttt atgaccttgt cggtaacaag
      541 ggcaaaacag ctatgactaa atatttatgt taccattacg gcgctatgct tgtagaaggt
      601 aagaaaaaag acattttgtt tggtatccaa aatgccgaag acaccgatat ttttatattt
      661 gatttctctc gatctataga aaactacgtt tcctatgatg cgatagaatc agttaaaaat
      721 ggtatctttt tttcaactaa atatgagtca aagatgattt tgcgaaattc accacatata
      781 attattttcg ctaattttct tcctgaagaa gaaaagctca gtaaagatag atggcgaatt
      841 tatgatttaa gtaaaagtga aaatttaatt taataagata tattatatat atgttattaa
      901 tggtgcgtac gcttaatccc ttaagttccc ttaaaaaatg acgcccttgc ggttcgtgtg
      961 gttttttttt tgaattcgct cgaacttttg atttgacctc ccttcggtcg ctcaaaaaaa
     1021 gcattctcgt tcattcaaaa aaaagcaccc cactcccttg ggctattttt taagagaact
     1081 taagagatta agcggtcttt gcgcagaccg ctttccgtgt cccttcggtc ccgctcccgc
     1141 accattgtaa attttatttt ttacttaaat ggttgattgg gttttttttg ggggttcggg
     1201 gtggaaaagg gcgggaaggc tgaagccttc cctttttacc attattattt atatctttat
     1261 ttataatata agatacgaaa tattaaaact gggcgaacaa cgaagttgtg agcccctaat
     1321 taaattattt aattaccgtc ataaaaccca atacgattga gagcaattcc acatatttga
     1381 tatatctcga tattgtacaa acatgctcca ggtggaacaa atccagttgg actgtattcg
     1441 atatcagaat cgatgctatc gtctgtgatt tccactgtct gaacggctat taacgttgca
     1501 cctgtcgatg ctacatcggg tgcattgtta tacgacgttt ggtaattgtt catcgtgttt
     1561 ggcgaaccta tgcatcctga gctagtattt agcgaaaaat cgggcggttt gatgccaact
     1621 gtgttgacac cactccactg atggacgaac aaatatcgtt gaccttggat caagcgattt
     1681 ttattcatcc tgattttatt ggatgtaggg aacgtaatgc ctatagagtc acaattgtta
     1741 ttaacaatta gtcccccgta tagtgccgtc ccgcacacgt tcgcccccgc tgtactgcct
     1801 tctctaacaa aagtagcaat tagtgcattg ctaagaggtg gtgtcattat ctttttatac
     1861 aaacgtattt tatacgttac gtataatgaa ccaatgttta tatttgaagc ttgaaatccc
     1921 gttgtgccca cagacaattt acccaaaaaa tacatttttg gatcggcatt aactggaatg
     1981 ttattgtcgt tgacgacata taacaatcca cccatgcctg tttgtcttgg tgcgcattca
     2041 acgggaatca acatcgattc agacggttta caactcatag accagtctga gttctcaatc
     2101 tggttacgat ttgtaaagtt ttcgtctgta taatcatagt tgactgcggc aaatacgcta
     2161 ccgagtgcgg tattggtgct gttgagggca tcagcactaa aactttggaa agagaatatc
     2221 aacccctcga acctatactg ttgaaagtta ggttgagcaa tgttacacag ccatggaaat
     2281 gactgtgtag aagcgggatt aatagggaaa gtagtaagtt taaatcctcc tatcgtattt
     2341 gatgtgataa gatcacccaa atactcacga tgagttatac atatactgtc gtctccatga
     2401 tcgtttgaaa atttaggtag ttggtccgct ggtgacatcg tagacgagtt gtaaccatac
     2461 gcaccactgc cgaaaaattt tcctattttg tgtccgagat atcgtccacc tgttgataat
     2521 gctgacccta acgcccgtcc gcctaaatgc gaaccgacga tcccgcctaa tgcttgtcct
     2581 gctttaccaa acggacctgg gttctcaagt gtgtaagcac caccgcccga ggctttgggt
     2641 gcgtaacgct tgaccgttga gcggggcgct ctaactgtaa cccgcttttt tttacttgat
     2701 gctcgttttc ttgctggcat tttctattta taattaatat atagaaaata aattttctat
     2761 atataattat aaattatgtt taattcttgt tgtgattgca actgcgaaaa actggaaaat
     2821 gtcaacaact taattaattt gtataaatcc aaattagata agcttgaaaa cgcgcgcaat
     2881 aaaattattt tagctctgca aaaataatta atttatcata ctataatata gtatggataa
     2941 attggaattg caaggaaaaa tagattattg taatatcaaa atgatagatg ttcacgatgt
     3001 aattgataaa atagaagata aaataatgat ttgtgctacg cccgaatcat tttctcttct
     3061 atcgagtaaa ttacgtgaca tgattcttga cctacggcta agactcatgc attttgaaat
     3121 aatgttcgat tctattattg atgaggtaaa agaaggtgat atgatcactt attgtacatc
     3181 atcggaaaca gaatcgccca cagcttgcgg ggatattgaa gggtataatt ttactgttca
     3241 tggagcagat agcgagtacc gtgacaccac tgacgattac gtcagaaaaa acaaaaaaag
     3301 taaacccttc aaaaattaaa ttatttaatg cgttttaaac gcactaaata tttgaatttc
     3361 agagcaaaat ttttatgtgt catatagtta t
//
LOCUS       CruV-237                3394 bp    DNA     circular     24-FEB-2020
DEFINITION  Crucivirus-237 isolate 120_146_SvL2_3298.
ACCESSION   CruV-237
KEYWORDS    .
SOURCE      Crucivirus-237
  ORGANISM  Crucivirus-237
            Unclassified.
REFERENCE   1  (bases 1 to 3394)
  AUTHORS   de la Higuera,I., Kasun,G.W., Torrance,E.L., Pratt,A.A.,
            Maluenda,A., Colombet,J., Bisseux,M., Ravet,V., Dayaram,A.,
            Stainton,D., Kraberger,S., Zawar-Reza,P., Goldstien,S., Briskie,J.,
            White,R., Taylor,H., Gomez,C., Ainley,D.G., Harding,J.S.,
            Fontenele,R.S., Schreck,J., Ribeiro,S.G., Oswald,S.A., Arnold,J.M.,
            Varsani,A., Enault,F. and Stedman,K.M.
  TITLE     Direct Submission
  JOURNAL   Submitted (24-FEB-2020) Biology, Portland State University, 1719 SW
            10th Ave, Portland, Oregon 97201, USA
COMMENT     Bankit Comment: ALT EMAIL:ide@pdx.edu
            Bankit Comment: TOTAL # OF SEQS:451
            Bankit Comment: GAP: est. length - alignment

            ##Assembly-Data-START##
            Assembly Method       :: MetaSPAdes v. 3.12; IDBA-UD v. 1.1.1;
                                     MEGAHIT v. 1.0.3
            Sequencing Technology :: 454; Illumina
            ##Assembly-Data-END##
FEATURES             Location/Qualifiers
     source          1..3394
                     /organism="Crucivirus-237"
                     /mol_type="genomic DNA"
                     /isolate="120_146_SvL2_3298"
                     /isolation_source="Water"
                     /country="Norway"
                     /collection_date=2012
     CDS             1..1194
                     /codon_start=1
                     /product="Putative Replication Associated Protein"
                     /translation="MPRVPRVTTNGRRATSRKLPRKTGRKDDPYVRVYDKKFKYWCFT
                     HHIRDADDDFLDFIKGIPSTYLVIGFETTKNGRPHYQGYIEFVTRKTKSAARAALPGC
                     HVEMRRGTAIEASEYCKEDGDFEEIGVLSKPVGAAGGQTTQAKYRRAIELSKTGRLDQ
                     LAEEDPSAYFLHYHTCKKLEADFTPQPTNLDAVCGEWWWGVMDAGKTTIIREMYPNHY
                     EKSFDKWWCAYQNEDVVFMDEVDQSHAGFARNFKVWADKFPFKGAAKNSHVYIRPKKF
                     IVASNYSIDEVFAKSNRVMREAIKKRFKVIYFPYAWYDSMAHTNPYHSKQQVSIEPLR
                     IGWRDDSELALTVPNTPLSGSYELDNLDRSLDEIQNSLEEIYNSPEWIPYPAGGPIVN
                     GPGSF*"
     CDS             complement(1221..3023)
                     /codon_start=1
                     /product="Putative CruV Capsid Protein"
                     /translation="MSKRPRIETLDSDDEGEPQLKILRRGGEGINPAREMGGWDDERQ
                     AEESSDIVNISDNPEVITEARGKNRLSLTERRALAKRPKGAKLKTIEYNNPVVSIPST
                     LPMTEMQVVTGGTGKASAAQIKAAEAEIAAGRKVDYGLWLQTKHGKKKTTKRKRKSTG
                     RSGYRRRPYNGGQPLIVSAGTARSLSGYGAYGLQGNVEGTLMGQNYRLRGFYDSSTDG
                     TAVSGYGAYEVKNNSLMNLIDTGTNPPRVMNTNRGEATIINHREYLGDLLSGSGSPSA
                     FNLQTFALNAGNSSLFPFLGTIAHKFQEWEPRGVLFELKTLSSDYAAALSMGSMFMAA
                     DYNSLSPAPNSKIALENMEYADSCKPSRSLIMPIECDPRISTQTHLNVADDLDYEGGD
                     KRLFDLCNVYIGSQGIPTADTPIAEIWVTYEIALFKPIIQNGMPDTSSPSARFLFHNV
                     QDALMFGNFTETAPGSSAGFTVDGTNPADCRLFFPAYDTTYIVVYQITNDNSGTIHGF
                     QIPAVTAVGTITTLSMWTTKNNIQNASQLSSGVLNGDGVHVCDTLLLTVVVKQTGGTI
                     NSGSLSFSGSDLVVAGVTKADLLVTGYNPLLQQF*"
     misc_feature    2732..2761
                     /note="stem-loop"
     misc_feature    2741..2749
                     /note="nonanucleotide"
ORIGIN
        1 atgccaagag taccgagagt gactacgaac ggtcgacgag cgacctcgcg aaaacttcca
       61 cggaagaccg gccggaagga cgatccttac gttcgtgtat acgacaagaa gttcaagtat
      121 tggtgtttta cgcaccatat acgtgacgcc gacgatgatt tcctggattt catcaaggga
      181 attccgtcga cgtatttggt catcggtttt gagactacga aaaacggacg accgcattac
      241 cagggttata tcgaattcgt tacacgaaag acgaagagcg ccgctcgcgc cgctctccct
      301 ggttgccacg tcgaaatgcg acgtggaacg gcaatcgagg cttccgagta ttgcaaagag
      361 gacggagatt tcgaagaaat cggagtactc tcgaagcccg taggcgcagc cggcgggcaa
      421 acgacgcaag cgaagtatcg tcgcgcgatc gagctgagta agacgggccg actcgaccaa
      481 ttggccgagg aagatccgtc cgcttatttt ttgcattacc atacatgcaa aaaacttgag
      541 gcggatttta cgcctcaacc taccaacctc gatgcagtat gcggagagtg gtggtggggc
      601 gtgatggacg ctggtaaaac cacgatcatc cgcgagatgt accccaatca ctatgagaaa
      661 tctttcgata agtggtggtg tgcatatcaa aatgaagacg tcgtatttat ggacgaagtc
      721 gatcaatccc acgctggttt cgctagaaat tttaaagtgt gggctgataa atttcctttc
      781 aaaggagcgg cgaagaatag ccacgtctat attcgcccga agaaatttat cgtcgcgagc
      841 aattattcga tcgatgaggt ttttgcgaaa tcgaatcgag ttatgcgaga ggcaataaaa
      901 aaacgattta aggttattta ttttccgtac gcgtggtacg acagcatggc gcacacgaat
      961 ccgtaccatt cgaaacagca agtttcgatc gagccgctcc gaattggatg gcgagatgat
     1021 tcggaactgg ctctgacggt cccgaataca cctttatcgg gttcttacga actcgataat
     1081 ctcgatcgtt ccttagacga gattcagaat tcactcgaag aaatttataa ttcgccagag
     1141 tggattcctt accctgctgg agggcctatt gtaaatggcc cgggaagttt ttaaataaat
     1201 catcgttaac gtaattttat ttagaattgt tgtaacaacg ggttatatcc cgtgacaagc
     1261 agatccgctt tcgtaactcc tgctacgacg agatctgaac cggagaacga tagagaaccc
     1321 gagtttatcg tccccccggt ttgtttgacg actacagtca acaataacgt gtcgcagacg
     1381 tgtacgccat ctccgttaag tacaccgctg cttaattgag aagcattttg gatgttattt
     1441 tttgtcgtcc acatgctgag cgttgttatc gttccaacgg ctgttaccgc aggtatttgg
     1501 aagccatgga tcgtccccga attatcgttg gttatttgat aaacaacgat ataagtcgtg
     1561 tcgtaagcgg ggaagaagag acggcaatcc gcaggatttg taccgtctac tgtaaaaccg
     1621 gcgctcgatc caggcgcggt ttcggtaaaa ttgccgaaca taagtgcatc ttgcacgttg
     1681 tggaagagga atctcgctga aggcgatgat gtatccggca taccgttttg tataatcggt
     1741 ttgaataaag cgatttcata cgttacccag atctccgcaa taggagtatc tgcggtcgga
     1801 attccttgac ttccgatata gacgttacag agatcgaaga gacgtttatc gcctccttcg
     1861 taatctaaat cgtctgccac attgagatgc gtctgagtac ttatacgagg atcgcactcg
     1921 attggcatta ttaacgatct actcggttta cacgagtcgg cgtattccat gttttccaat
     1981 gcgattttcg aatttggagc aggactcaac gagttgtagt ccgcagccat gaacatggaa
     2041 cccatactga gagctgcagc gtaatcgcta gataacgttt taagctcgaa tagtacaccc
     2101 ctaggttccc attcttggaa cttatgggcg atcgttccca aaaagggaaa gagagaggag
     2161 tttccagcgt ttaacgcgaa agtctgcagg ttgaaagccg aaggcgaacc gctaccggac
     2221 aagagatcac cgagatattc tcgatggttg atgattgttg cttcaccccg attcgtgttc
     2281 attacgcgag gagggttggt tccggtatcg atgaggttca taagagagtt atttttaact
     2341 tcgtatgcac cgtaaccgct tacggcggtt ccatcggtag aactatcgta aaatccacgt
     2401 aatcgatagt tctgccccat caaggtgcct tcgacgttac cttgtaaccc gtaagcaccg
     2461 taaccgctta gagatcgagc tgttccagcg ctgacgatta acggttgccc accgttgtaa
     2521 gggcggcgtc tatagccgct tcgaccggtc gacttacgtt tacgtttagt cgttttcttt
     2581 ttgccatgct ttgtctgcaa ccaaagaccg taatcgactt tgcgtccagc agcgatttca
     2641 gcttcggcag ctttaatttg agcagcagaa gcttttccgg taccaccggt aacaacttgc
     2701 atttccgtca tcggtaacgt tgatggaatg gaaacgaccg gattattata ttcgatcgtt
     2761 tttaatttcg ctccctttgg tctttttgcc aaagcacgtc tttcggtgag cgacaagcga
     2821 ttttttcctc gcgcttccgt aatcacttcg ggattatcgg aaatattgac gatgtctgac
     2881 gattcttcgg cttgacgttc gtcatcccaa cctcccattt ctctcgcggg gttaattcct
     2941 tcacctccgc gacgcaagat tttgagttga ggttcaccct catcatcgga atcgagcgtt
     3001 tctattcttg gacgtttgct catgacgtcg aagatacccg gaaataatta ttgtacttac
     3061 aataaattat aaatccgggg tggggaggag cgaagcgacg acgcagatgg gtaaaaacct
     3121 agcgccgcag cgcgaagcgc ggaggcaaga ctaccctagc ggcagggagc caagcacgcg
     3181 cagcgtgcga tggcgaccgt gagcgtattt ttgggagtac gaaagtcgac caatagaaaa
     3241 ctccgggtcc gactgtcgga ccggaatttt aattccggag tgttttcact gaaaccggag
     3301 tttcgggaat ttaaaaaacg ggacacttgt gcggagtaac cattattacc tttagagcac
     3361 aagtgtccct gtccccagag gaacgatagt gact
//
LOCUS       CruV-238                3396 bp    DNA     circular     26-FEB-2020
DEFINITION  Crucivirus-238 isolate 120_1371_IR2_3297.
ACCESSION   CruV-238
KEYWORDS    .
SOURCE      Crucivirus-238
  ORGANISM  Crucivirus-238
            Unclassified.
REFERENCE   1  (bases 1 to 3396)
  AUTHORS   de la Higuera,I., Kasun,G.W., Torrance,E.L., Pratt,A.A.,
            Maluenda,A., Colombet,J., Bisseux,M., Ravet,V., Dayaram,A.,
            Stainton,D., Kraberger,S., Zawar-Reza,P., Goldstien,S., Briskie,J.,
            White,R., Taylor,H., Gomez,C., Ainley,D.G., Harding,J.S.,
            Fontenele,R.S., Schreck,J., Ribeiro,S.G., Oswald,S.A., Arnold,J.M.,
            Varsani,A., Enault,F. and Stedman,K.M.
  TITLE     Direct Submission
  JOURNAL   Submitted (24-FEB-2020) Biology, Portland State University, 1719 SW
            10th Ave, Portland, Oregon 97201, USA
COMMENT     Bankit Comment: ALT EMAIL:ide@pdx.edu
            Bankit Comment: TOTAL # OF SEQS:451
            Bankit Comment: GAP: est. length - alignment

            ##Assembly-Data-START##
            Assembly Method       :: MetaSPAdes v. 3.12; IDBA-UD v. 1.1.1;
                                     MEGAHIT v. 1.0.3
            Sequencing Technology :: 454; Illumina
            ##Assembly-Data-END##
FEATURES             Location/Qualifiers
     source          1..3396
                     /organism="Crucivirus-238"
                     /mol_type="genomic DNA"
                     /isolate="120_1371_IR2_3297"
                     /isolation_source="Water"
                     /country="Norway"
                     /collection_date=2011
     CDS             1..1359
                     /codon_start=1
                     /product="Putative CruV Capsid Protein"
                     /translation="MLYTINNMSKKYTAEEKIAYYKNLANAKSAYKYKKRPKKEGESK
                     RGRSRKKNNKYYSKYGAVAGGAIGTAMYGPVGGVIGTGLGGLAGAALSSIMGHGAYTV
                     NRNTIVKNTVPQFVSRMSDGCIRMKHKEFISDVVTSATPGAFSNRVYKLSATSNSTFP
                     YLSQIAVNFEEFAFEGLVFSYVPSSGSISTTGQLGTVIMATQYNALSQPFTNKQQAEA
                     STYAVSQVVSQPVIHPIECDAKQTPSNGLFYCQRPSLQLNNQDPRWQQLGQFNMMTQG
                     APNASENIGELWVSYDVVLMKPILIQDDGEQADHWSSATGIVVGTTPFGSDFAISDFS
                     DGFTQLVAPNSVEIDASFNGNIAISYGVHHGAGTTGTIGTDPTFTAGVGVTPLNILHG
                     SITNQYVKSYFDQTNLVLWTTGYFTIASTGVPSVITLVGGAALASTSMDLIVEQVPAD
                     FD*"
     misc_feature    join(3396,1..24)
                     /note="stem-loop"
                     /modified_by="nacho"
     misc_feature    complement(8..16)
                     /note="nonanucleotide"
     misc_feature    9..17
                     /note="nonanucleotide"
     CDS             1797..2933
                     /codon_start=1
                     /product="Putative Replication Associated Protein"
                     /translation="MQLNIVQPASQEVPIITYDFTVWPEYEGITGCSGITGHFTEQAY
                     QLSKKLRTFCRAFCFQLEIAPTTGRLHFQGRLKLKEKKRKTQFISFFGDQVTKICHIS
                     PTAKENSKNFFYVMKESTKLLGPWADTDNGGKEMPWHLAMFPDLLAWQETIKRLIQIR
                     ELRILNCVINGVGNNGKTICCQKLVWEGLACYLPFSDSHKELMQAVLDCGDKPAFIFD
                     FPRALKKSQMRPLFSAIEDIKSGWSFNHKYGYRSDLRGQPNIWLFMNKWPNIRYLSAD
                     RWKFWNITDSKELVQYTPEEIDAAQKALSAKAYAKKHPGQKYRRAKEPAILNVPPITI
                     ATMATEAIKVPSIPTPPLRELPMTPRRHSLLNGLPLPPIGERVI*"
ORIGIN
        1 atgttatata ctattaataa catgtcaaag aaatacacag cagaagaaaa aatcgcatat
       61 tacaagaacc ttgcaaatgc gaaaagcgct tacaaatata agaaaagacc aaagaaagaa
      121 ggtgaatcta aaaggggtag atccagaaaa aagaacaaca aatattatag caaatatggt
      181 gccgtagctg gaggagcaat cggcacagcc atgtatggtc ctgtcggagg tgtcattgga
      241 acaggtctcg gaggcttagc tggtgcagct ttaagctcca tcatgggaca cggtgcttac
      301 actgtaaaca gaaacaccat cgtgaaaaat actgttccac aattcgtctc ccgtatgtcc
      361 gacggttgta tcagaatgaa acataaagag ttcatttctg acgtcgttac ttctgcgaca
      421 cccggtgcgt tcagtaacag agtttataag ttaagtgcta ctagcaactc aactttcccg
      481 tacctaagtc aaatagcagt aaatttcgaa gaatttgctt ttgaaggact agtattctcc
      541 tacgttcctt ccagcggatc tatttcgacc actggacaat taggtactgt tatcatggca
      601 actcaataca acgcattgag tcaaccattc acaaacaaac aacaagctga agcttcgaca
      661 tatgctgtct ctcaagttgt ttcccaacct gtcattcacc ctattgagtg cgatgccaaa
      721 caaactcctt caaacggact tttctactgt caacgaccaa gtctccaact taacaaccaa
      781 gatcctcgtt ggcaacaact tggtcaattc aatatgatga ctcaaggtgc acccaatgct
      841 tcggagaaca ttggtgaatt atgggtatct tacgatgtgg ttttaatgaa accaatccta
      901 attcaagatg atggtgaaca agctgatcac tggtcaagtg ctacaggcat tgtcgtgggt
      961 accactcctt tcggttctga cttcgctatc tccgacttct cagatggatt tacgcaacta
     1021 gttgctccaa atagtgttga gatcgacgca tctttcaatg gaaatatagc tataagttat
     1081 ggtgtacatc atggagctgg tactactggt accataggaa ctgatcctac gttcaccgcc
     1141 ggtgtcggtg taacgccgct taacatatta cacggtagca taacaaatca atatgttaaa
     1201 tcatatttcg accaaaccaa tcttgtcctt tggactacag gttacttcac catcgcctcc
     1261 actggcgttc cgtctgttat taccctagtg ggcggtgcag ctttagcttc aactagtatg
     1321 gatcttatag tggaacaagt tccagccgac ttcgattaat tatatatttt tacaaaatat
     1381 ataaattaga gcgggatacc cgacacgtca ttcgcttcgc gcttgcaaac tttatatata
     1441 gtataaagct tgttttaaat aaaacgtcta aagacactta tttcttcgaa aaggtctaaa
     1501 tgcaacatta ccatgacgat tcttcatctg aactagatac cgacgaagaa atagaattat
     1561 taaacgaatt aaaagattta aacatcgatg attctcccat tactaggctc gattcatcat
     1621 taattatata tactcctgag gaggtatacg aactagaaat taacaaagaa attatcaata
     1681 aactaatcaa taacaacata aaaagtattc tattagacct ttattctatt cccagaatag
     1741 ataataaaaa taattaaacg gtctaaatgt tccaacatgt tctataacac aagaacatgc
     1801 aactaaacat cgttcagcca gcttcacaag aagtacctat tatcacatac gatttcactg
     1861 tatggcccga atacgaagga attaccggtt gttccggtat taccggacac tttacagaac
     1921 aagcttatca gctatccaaa aagttaagaa ctttttgtag agcgttctgt tttcaactag
     1981 aaatagcacc taccacaggt agattgcact tccaaggaag attaaaatta aaagaaaaga
     2041 aacgaaaaac tcaatttata tcctttttcg gagaccaagt gacaaaaata tgccacataa
     2101 gtccaaccgc aaaagaaaat tcaaagaatt tcttttacgt catgaaagaa tcaactaaac
     2161 ttctcggccc atgggccgat acagataacg gagggaaaga aatgccttgg catttagcaa
     2221 tgttcccaga cttactagct tggcaagaaa caatcaaaag actgatacaa ataagagaat
     2281 taagaattct caactgcgtc ataaacggag taggcaacaa tggtaaaaca atatgctgcc
     2341 aaaaactcgt atgggaagga ttagcttgct acctcccatt ctcagacagt cacaaagaat
     2401 taatgcaagc ggttctcgac tgtggcgata aacctgcttt tatcttcgac ttcccaagag
     2461 cactcaaaaa gagtcaaatg agacctcttt tttcggccat cgaagatatc aaaagtggat
     2521 ggagcttcaa ccacaaatat ggttacagaa gcgacctaag aggtcaaccc aatatttggc
     2581 tattcatgaa taaatggccc aatattaggt atctttccgc tgaccgctgg aaattctgga
     2641 atatcactga ttctaaagaa ctagttcaat acactccaga agaaatagat gccgctcaaa
     2701 aggcactatc agcaaaagct tacgctaaga aacatcccgg acaaaagtac cgcagagcta
     2761 aagagcctgc tatcctgaat gtgccaccca ttactatcgc aacgatggcc acggaggcga
     2821 ttaaggtccc ttcgatccct acacctccac tccgtgagtt accaatgacg cccagaaggc
     2881 attcattact caacggatta ccactccccc ctatcggaga aagagtgata taaaataatt
     2941 cgtgattcca caaattatta tccagcctcg accttacggt ctcggaccct ggcggggcgg
     3001 cgcttcgctg taatactaca agccccgcca ttaactctat cgcgacttgt cgctcgagta
     3061 aatgtatata ttatatacgc aaaagtacat gaagtacatt cttatttatt tatttattta
     3121 acatatagtc taaagataaa cttttactag aaataaatga agaaaccagc tggattaaca
     3181 tggcctcaat ggcaattaac agaaaaactt ctcaaaacaa actgggcaga cggaacccaa
     3241 gaacacagct tcctcacaga tatgaacgac agactttcaa ttgcagaaag aaatcaatta
     3301 tatagagatc ctcaaaacaa atggtttaaa ggaggtggcg atgttgtttg gaaaactaac
     3361 acagcattta ttccaagata aatagtctaa agcaac
//
LOCUS       CruV-239                3398 bp    DNA     circular     26-FEB-2020
DEFINITION  Crucivirus-239 isolate 120_461_IR1_3296.
ACCESSION   CruV-239
KEYWORDS    .
SOURCE      Crucivirus-239
  ORGANISM  Crucivirus-239
            Unclassified.
REFERENCE   1  (bases 1 to 3398)
  AUTHORS   de la Higuera,I., Kasun,G.W., Torrance,E.L., Pratt,A.A.,
            Maluenda,A., Colombet,J., Bisseux,M., Ravet,V., Dayaram,A.,
            Stainton,D., Kraberger,S., Zawar-Reza,P., Goldstien,S., Briskie,J.,
            White,R., Taylor,H., Gomez,C., Ainley,D.G., Harding,J.S.,
            Fontenele,R.S., Schreck,J., Ribeiro,S.G., Oswald,S.A., Arnold,J.M.,
            Varsani,A., Enault,F. and Stedman,K.M.
  TITLE     Direct Submission
  JOURNAL   Submitted (24-FEB-2020) Biology, Portland State University, 1719 SW
            10th Ave, Portland, Oregon 97201, USA
COMMENT     Bankit Comment: ALT EMAIL:ide@pdx.edu
            Bankit Comment: TOTAL # OF SEQS:451
            Bankit Comment: GAP: est. length - alignment

            ##Assembly-Data-START##
            Assembly Method       :: MetaSPAdes v. 3.12; IDBA-UD v. 1.1.1;
                                     MEGAHIT v. 1.0.3
            Sequencing Technology :: 454; Illumina
            ##Assembly-Data-END##
FEATURES             Location/Qualifiers
     source          1..3398
                     /organism="Crucivirus-239"
                     /mol_type="genomic DNA"
                     /isolate="120_461_IR1_3296"
                     /isolation_source="Water"
                     /country="Norway"
                     /collection_date=2011
     CDS             1..1359
                     /codon_start=1
                     /product="Putative CruV Capsid Protein"
                     /translation="MLYTINNMSKKYTAEEKIAYYKNLANAKSAYKYKKRPKKEGESK
                     RGRSRKKNNKYYSKYGAVAGGAIGTAMYGPVGGVIGTGLGGLAGAALSSIMGHGAYTV
                     NRNTIVKNTVPQFVSRMSDGCIRMKHKEFISDVVTSATPGAFSNRVYKLSATSNSTFP
                     YLSQIAVNFEEFAFEGLVFSYVPSSGSISTTGQLGTVIMATQYNALSQPFTNKQQAEA
                     STYAVSQVVSQPVIHPIECDAKQTPSNGLFYCQRPSLQLNNQDPRWQQLGQFNMMTQG
                     APNASENIGELWVSYDVVLMKPILIQDDGEQADHWSSATGIVVGTTPFGSDFAISDFS
                     DGFTQLVAPDTVEIDASFNGNIAISYGVHHGAGTTGTIGTDPTFTAGVGVTPLNILHG
                     SITNQYVKSYFDQTNLVLWTTGYFTIASTGVPSVITLVGGAALASTSMDLIVEQVPAD
                     FD*"
     misc_feature    join(3398,1..24)
                     /note="stem-loop"
                     /modified_by="nacho"
     misc_feature    complement(8..16)
                     /note="nonanucleotide"
     misc_feature    9..17
                     /note="nonanucleotide"
     CDS             1797..2933
                     /codon_start=1
                     /product="Putative Replication Associated Protein"
                     /translation="MQLNIVQPASQEVPIITYDFTVWPEYEGITGCSGITGHFTEQAY
                     QLSKKLRTFCRAFCFQLEIAPTTGRLHFQGRLKLKEKKRKTQFISFFGDQVTKICHIS
                     PTAKENSKNFFYVMKESTKLLGPWADTDNGGKEMPWHLAMFPDLLAWQETIKRLIQIR
                     ELRILNCVINGVGNNGKTICCQKLVWEGLACYLPFSDSHKELMQAVLDCGDKPAFIFD
                     FPRALKKSQMRPLFSAIEDIKSGWSFNHKYGYRSDLRGQPNIWLFMNKWPNIRYLSAD
                     RWKFWNITDSKELVQYTPEEIDAAQKALSAKAYAKKHPGQKYRRAKEPAILNVPPITI
                     ATMATEAIKVPSIPTPPLRELPMTPRRHSLLNGLPLPPIGERVI*"
ORIGIN
        1 atgttatata ctattaataa catgtcaaag aaatacacag cagaagaaaa aatcgcatat
       61 tacaagaacc ttgcaaatgc gaaaagcgct tacaaatata agaaaagacc aaagaaagaa
      121 ggtgaatcta aaaggggtag atccagaaaa aagaacaaca aatattatag caaatatggt
      181 gccgtagctg gaggagcaat cggcacagcc atgtatggtc ctgtcggagg tgtcattgga
      241 acaggtctcg gaggcttagc tggtgcagct ttaagctcca tcatgggaca cggtgcttac
      301 actgtaaaca gaaacaccat cgtgaaaaat actgttccac aattcgtctc ccgtatgtcc
      361 gacggttgta tcagaatgaa acataaagag ttcatttctg acgtcgttac ttctgcgaca
      421 cccggtgcgt tcagtaacag agtttataag ttaagtgcta ctagcaactc aactttcccg
      481 tacctaagtc aaatagcagt aaatttcgaa gaatttgctt ttgaaggact agtattctcc
      541 tacgttcctt ccagcggatc tatttcgacc actggacaat taggtactgt tatcatggca
      601 actcaataca acgcattgag tcaaccattc acaaacaaac aacaagctga agcttcgaca
      661 tatgctgtct ctcaagttgt ttcccaacct gtcattcacc ctattgagtg cgatgccaaa
      721 caaactcctt caaacggact tttctactgt caacgaccaa gtctccaact taacaaccaa
      781 gatcctcgtt ggcaacaact tggtcaattc aatatgatga ctcaaggtgc acccaatgct
      841 tcggagaaca ttggtgaatt atgggtatct tacgatgtgg ttttaatgaa accaatccta
      901 attcaagatg atggtgaaca agctgatcac tggtcaagtg ctacaggcat tgtcgtgggt
      961 accactcctt tcggttctga cttcgctatc tccgacttct cagatggatt tacgcaacta
     1021 gttgctccag atactgttga gatcgacgca tctttcaatg gaaatatagc tataagttat
     1081 ggtgtacatc atggagctgg tactactggt accataggaa ctgatcctac gttcaccgcc
     1141 ggtgtcggtg taacgccgct taacatatta cacggtagca taacaaatca atatgttaaa
     1201 tcatatttcg accaaaccaa tcttgtcctt tggactacag gttacttcac catcgcctcc
     1261 actggcgttc cgtctgttat taccctagtg ggcggtgcag ctttagcttc aactagtatg
     1321 gatcttatag tggaacaagt tccagccgac ttcgattaat tatatatttt tacaaaatat
     1381 ataaattaga gcgggatacc cgacacgtca ttcgcttcgc gcttgcaaac tttatatata
     1441 gtataaagct tgttttaaat aaaacgtcta aagacactta tttcttcgaa aaggtctaaa
     1501 tgcaacatta ccatgacgat tcttcatctg aactagatac cgacgaagaa atagaattat
     1561 taaacgaatt aaaagattta aacatcgatg attctcccat tactaggctc gattcatcat
     1621 taattatata tactcctgag gaggtatacg aactagaaat taacaaagaa attatcaata
     1681 aactaatcaa taacaacata aaaagtattc tattagacct ttattctatt cccagaatag
     1741 ataataaaaa taattaaacg gtctaaatgt tccaacatgt tctataacac aagaacatgc
     1801 aactaaacat cgttcagcca gcttcacaag aagtacctat tatcacatac gatttcactg
     1861 tatggcccga atacgaagga attaccggtt gttccggtat taccggacac tttacagaac
     1921 aagcttatca gctatccaaa aagttaagaa ctttttgtag agcgttctgt tttcaactag
     1981 aaatagcacc taccacaggt agattgcact tccaaggaag attaaaatta aaagaaaaga
     2041 aacgaaaaac tcaatttata tcctttttcg gagaccaagt gacaaaaata tgccacataa
     2101 gtccaaccgc aaaagaaaat tcaaagaatt tcttttacgt catgaaagaa tcaactaaac
     2161 ttctcggccc atgggccgat acagataacg gagggaaaga aatgccttgg catttagcaa
     2221 tgttcccaga cttactagct tggcaagaaa caatcaaaag actgatacaa ataagagaat
     2281 taagaattct caactgcgtc ataaacggag taggcaacaa tggtaaaaca atatgctgcc
     2341 aaaaactcgt atgggaagga ttagcttgct acctcccatt ctcagacagt cacaaagaat
     2401 taatgcaagc ggttctcgac tgtggcgata aacctgcttt tatcttcgac ttcccaagag
     2461 cactcaaaaa gagtcaaatg agacctcttt tttcggccat cgaagatatc aaaagtggat
     2521 ggagcttcaa ccacaaatat ggttacagaa gcgacctaag aggtcaaccc aatatttggc
     2581 tattcatgaa taaatggccc aatattaggt atctttccgc tgaccgctgg aaattctgga
     2641 atatcactga ttctaaagaa ctagttcaat acactccaga agaaatagat gccgctcaaa
     2701 aggcactatc agcaaaagct tacgctaaga aacatcccgg acaaaagtac cgcagagcta
     2761 aagagcctgc tatcctgaat gtgccaccca ttactatcgc aacgatggcc acggaggcga
     2821 ttaaggtccc ttcgatccct acacctccac tccgtgagtt accaatgacg cccagaaggc
     2881 attcattact caacggatta ccactccccc ctatcggaga aagagtgata taaaataatt
     2941 cgtgattcca caaattatta tccagcctcg accttacggt ctcggaccct ggcggggctt
     3001 ggcgcttcgc tgtaatacta caagccccgc cattaactct atcgcgactt gtcgctcgag
     3061 taaatgtata tattatatac gcaaaagtac atgaagtaca ttcttattta tttatttatt
     3121 taacatatag tctaaagata aacttttact agaaataaat gaagaaacca gctggattaa
     3181 catggcctca atggcaatta acagaaaaac ttctcaaaac aaactgggca gacggaaccc
     3241 aagaacacag cttcctcaca gatatgaacg acagactttc aattgcagaa agaaatcaat
     3301 tatatagaga tcctcaaaac aaatggttta aaggaggtgg cgatgttgtt tggaaaacta
     3361 acacagcatt tattccaaga taaatagtct aaagcaac
//
LOCUS       CruV-240                3400 bp    DNA     circular     24-FEB-2020
DEFINITION  Crucivirus-240 isolate Ga0132256_100061645.
ACCESSION   CruV-240
KEYWORDS    .
SOURCE      Crucivirus-240
  ORGANISM  Crucivirus-240
            Unclassified.
REFERENCE   1  (bases 1 to 3400)
  AUTHORS   de la Higuera,I., Kasun,G.W., Torrance,E.L., Pratt,A.A.,
            Maluenda,A., Colombet,J., Bisseux,M., Ravet,V., Dayaram,A.,
            Stainton,D., Kraberger,S., Zawar-Reza,P., Goldstien,S., Briskie,J.,
            White,R., Taylor,H., Gomez,C., Ainley,D.G., Harding,J.S.,
            Fontenele,R.S., Schreck,J., Ribeiro,S.G., Oswald,S.A., Arnold,J.M.,
            Varsani,A., Enault,F. and Stedman,K.M.
  TITLE     Direct Submission
  JOURNAL   Submitted (24-FEB-2020) Biology, Portland State University, 1719 SW
            10th Ave, Portland, Oregon 97201, USA
COMMENT     Bankit Comment: ALT EMAIL:ide@pdx.edu
            Bankit Comment: TOTAL # OF SEQS:451
            Bankit Comment: GAP: est. length - alignment

            ##Assembly-Data-START##
            Assembly Method       :: MetaSPAdes v. 3.12; IDBA-UD v. 1.1.1;
                                     MEGAHIT v. 1.0.3
            Sequencing Technology :: 454; Illumina
            ##Assembly-Data-END##
FEATURES             Location/Qualifiers
     source          1..3400
                     /organism="Crucivirus-240"
                     /mol_type="genomic DNA"
                     /isolate="Ga0132256_100061645"
                     /isolation_source="Soil"
                     /country="USA"
                     /collection_date=2015
     CDS             1..1056
                     /codon_start=1
                     /product="Putative Replication Associated Protein"
                     /translation="MADNVQLYCFDFTLDVVASEKTKTPGVTVSVEQLKNWLRERAKK
                     WVFQIEKGAESGILHYQGRLTLISKQRLYTLAKECEWQWIHLSPTASVNKNSFIYVMK
                     ADTKVQGPWTDKDVPLPPRFQDIVLKPFQKQIQIIMKIFDMRGINVVIAQPNLGKTHA
                     TQYFTLTDNAIIVPPYPDFEKVLGFVHGMMEDSAPYGTPNYQKLWTIFIDMPRAMPKL
                     KLQGMYSALETIKSGMVYDWRNKAKKYIFHHNPIIVVFTNEKPDLSLLGDDRWKLWTI
                     SDNWLEEYDDTPNIPVVPDIPSFNLGKTYEQIELEKARFEVEALKKQIEDLKNPPKPE
                     TNYKLAFPALITPPRKS*"
     CDS             complement(1535..3154)
                     /codon_start=1
                     /product="Putative CruV Capsid Protein"
                     /translation="MSKRQKEIAAAYAKKAGLKVIGAPKKVSGYGTYYPKPAYKKRAV
                     RGYGGYGQDIGSRLGGFVGNAAEDLLGGFGKALGFGDYAKAGFAVKKNSLLNGGNDPP
                     VVRNAKDGRFILRHREYIQDIITGAAGAFSVQNLPINPGIQRTFPWLAEVAQAFEQYR
                     LRGMLFEFKSTSGDALNSTNTALGSIIMATEYDSSRPLFTSKQQMENHQFASSARQSC
                     SMLHPIECDRSQTPITDLYIRTGLPSDSSDNDTDLRMSDWGNFQIATTGQQAASVNIG
                     ELWVTYEIELLKPCINEVGPVQLSSFYFSNDGTFTAGNYFGTPGTSIKSNTGNAIQMD
                     LSVGNIIRFPKGTYGTYLLAMEWVGTANALSVLPTFAGSAITEGVTLGTAISPFTTIF
                     APANGETCTKLVAVLLVSCFGQTNTGAGPGIVVSGGVLPNTPTTAKLYVTSVMDGLIN
                     ANNPPLPEEIDVNEITDDDFKEFIQSNLQDLRFKFLQEKDEKDKAKALEETSKPSNAV
                     EEKKKLDNMSQEELLELYLKMKAKREGKNPA*"
     misc_feature    2777..2796
                     /note="stem-loop"
     misc_feature    2782..2790
                     /note="nonanucleotide"
ORIGIN
        1 atggcagaca acgtacagtt atactgtttt gacttcacac ttgatgtagt agcatcagaa
       61 aaaacaaaaa caccaggggt aacagtatct gtagagcagc tcaagaattg gttaagagag
      121 cgagccaaga agtgggtatt tcagattgag aagggagcag agtcaggtat tcttcattat
      181 caaggtagac ttacattaat atcaaaacaa aggttatata cattagcgaa ggaatgtgag
      241 tggcaatgga tacatttatc accaacggca tcagttaaca aaaacagctt catttatgtt
      301 atgaaggcag atacaaaggt tcaagggccg tggacagata aggatgttcc gttaccaccc
      361 aggtttcaag atatagtgtt aaagcctttc caaaaacaaa tacaaataat aatgaagata
      421 tttgatatgc gagggatcaa tgttgtaata gcccagccta atttaggcaa gactcacgca
      481 acacaatact ttactctcac agataatgca attatagtac caccatatcc tgattttgag
      541 aaagttttag gatttgtaca cggtatgatg gaggatagtg ctccatatgg tacacctaat
      601 taccaaaaac tttggacaat attcatagat atgccaagag caatgcccaa acttaaatta
      661 cagggtatgt attcagcttt agagactatt aagagtggga tggtttatga ttggagaaat
      721 aaagctaaga aatatatatt tcatcataat ccgataatag ttgtcttcac taatgagaag
      781 ccagatttgt ctttacttgg tgatgataga tggaaactct ggacaatttc agataattgg
      841 ttggaggagt atgacgatac tcctaatatt ccagtagttc cagatattcc atcattcaat
      901 ttaggtaaga catatgagca gatagaatta gagaaggcaa gatttgaggt agaagcatta
      961 aaaaaacaga tagaggatct taaaaatcca ccaaaaccag aaactaatta taaattagct
     1021 tttcctgcac tcataacacc tcctaggaaa tcatagtctc aaaacttata ctacactaaa
     1081 tatagtataa attaacaaca aacatgcatc tttgatgcta ctgcttaagt agaactcagc
     1141 gcagagcgct ggagttcgta acctttcaag tcacaaacta gaactactgc tagttgtcta
     1201 tacatatttt gatccatgct gatccaacgg tgagccatac aaacacatca aacatatcac
     1261 ttactacaaa ccgagttgtt ctattctgat ccaactgatc caactcgtct ataaaaaaat
     1321 atatatagga tatatatttt atttaattga gtgagtgagc gcaaagcgcg aacgaacgaa
     1381 caatttaatt ttttttcagt gttacaggca tacctgccgc ctaaaaaaaa ttaaatattt
     1441 tatatacata taaaaattta cagtttaaaa tatattcatc taggatgatt aaatttagtt
     1501 tttataaaaa acccccgcga ggtaaacccc gcatttatgc ggggttttta ccttcgcgtt
     1561 tagctttcat ttttaaataa agttctaaca attcttcttg actcatattg tcaagtttct
     1621 tcttttcttc gacggcgttc gaaggtttag aagtttcttc tagtgcttta gccttatctt
     1681 tctcatcttt ttcttgtaag aatttaaagc gtaagtcttg taagttggat tgtataaact
     1741 ctttaaaatc atcatcagtg atttcgttaa catcaatctc ctcaggaaga ggaggattat
     1801 tagcattaat caaaccatcc ataacagaag taacatacaa cttcgcagtg gtaggagtat
     1861 taggtaatac accaccactt acaacaatac caggaccagc accagtatta gtttgaccaa
     1921 aacaacttac aagtaataca gcgactaact tagtacaagt ttcaccatta gccggagcaa
     1981 agatagttgt aaacggacta atggcagttc caagagtaac accttcagtg atagcggaac
     2041 cagcaaaagt gggaagaacg gataaagcat tagcagttcc aacccattcc atagccaata
     2101 aataagtacc ataagtacct ttaggaaatc taataatatt accaacagat aaatccattt
     2161 gaatagcatt tcctgtatta gatttaatag aggtaccagg agtaccaaaa taattaccag
     2221 cagtaaaagt accatcatta ctaaaataga atgaagacaa ctgaacaggg ccgacttcat
     2281 taatacaagg tttcaataat tcaatctcat aagtaaccca aagctcgcca atgtttacac
     2341 tagcagcttg ctgaccagta gtagcaattt ggaaattacc ccaatctgac atacgaagat
     2401 cagtatcatt atcagatgaa tctgaaggca atccagttcg aatatacaaa tcagttatag
     2461 gagtctgact acgatcacac tcaatagggt ggagcatcga acatgattga cgggctgagg
     2521 atgcaaattg gtgattttcc atttgctgtt tactggtaaa cagagggcga gaagaatcat
     2581 attcagtagc cataataata gaaccaaggg cagtattagt agagttcaaa gcatcaccag
     2641 atgtactctt aaattcaaat aacattccac ggagtctgta ttgttcgaat gcttgagcaa
     2701 cttcagctaa ccaagggaat gttctttgaa taccaggatt tattggtaga ttctggactg
     2761 agaaagctcc agcagctcca gtaataatat cctggatata ctcacgatgg cgtaggataa
     2821 agcgtccgtc tttagcatta cgaacaacgg gaggatcatt accaccgttg aggagagaat
     2881 tcttcttaac ggcaaatcca gctttagcat aatcaccaaa gccaagagct ttgccaaagc
     2941 cacctaacaa gtcttcagca gcattaccaa caaaaccacc gaggcgagat ccaatatctt
     3001 gcccgtaacc gccatatcct cgaacagctc ttttcttata agctggttta ggatagtagg
     3061 taccatagcc gctcactttc ttaggagcgc caatcacttt caatccagct ttcttagcat
     3121 aagcggcagc tatctctttc tgtcgtttag acatcttaca atctcttaca atctcttaca
     3181 atctcttaca aaagtctttt ataagaaata aatttatatt ttaaacttca attttattaa
     3241 taccaataaa aatcatcata ttctaaataa tatacaccat atccatggta atagtaatca
     3301 ttcatatata atctattatc catctttcaa cttttattat aaaaataaaa aataaaaaaa
     3361 aatcttagat ccaaaaaaac ttggatcaat tggatcaaaa
//
LOCUS       CruV-241                3402 bp    DNA     circular     24-FEB-2020
DEFINITION  Crucivirus-241 isolate SR5_34666.
ACCESSION   CruV-241
KEYWORDS    .
SOURCE      Crucivirus-241
  ORGANISM  Crucivirus-241
            Unclassified.
REFERENCE   1  (bases 1 to 3402)
  AUTHORS   de la Higuera,I., Kasun,G.W., Torrance,E.L., Pratt,A.A.,
            Maluenda,A., Colombet,J., Bisseux,M., Ravet,V., Dayaram,A.,
            Stainton,D., Kraberger,S., Zawar-Reza,P., Goldstien,S., Briskie,J.,
            White,R., Taylor,H., Gomez,C., Ainley,D.G., Harding,J.S.,
            Fontenele,R.S., Schreck,J., Ribeiro,S.G., Oswald,S.A., Arnold,J.M.,
            Varsani,A., Enault,F. and Stedman,K.M.
  TITLE     Direct Submission
  JOURNAL   Submitted (24-FEB-2020) Biology, Portland State University, 1719 SW
            10th Ave, Portland, Oregon 97201, USA
COMMENT     Bankit Comment: ALT EMAIL:ide@pdx.edu
            Bankit Comment: TOTAL # OF SEQS:451
            Bankit Comment: GAP: est. length - alignment

            ##Assembly-Data-START##
            Assembly Method       :: MetaSPAdes v. 3.12; IDBA-UD v. 1.1.1;
                                     MEGAHIT v. 1.0.3
            Sequencing Technology :: 454; Illumina
            ##Assembly-Data-END##
FEATURES             Location/Qualifiers
     source          1..3402
                     /organism="Crucivirus-241"
                     /mol_type="genomic DNA"
                     /isolate="SR5_34666"
                     /isolation_source="Water"
                     /country="New Zealand"
                     /collection_date=2012
     CDS             1..1392
                     /codon_start=1
                     /product="Putative CruV Capsid Protein"
                     /translation="MSKVARRRTYTKAEKAAFAKKMARARASRGRAVSFRGNGDYSLY
                     KAPRAIRASKSTTKSLGGTVGAHIGNGIHNLVKALTGFGDYNIAENSLMSGTIGGDPP
                     VMRNTANNSFIVHHREYIGDVYATNSFTAISYSLNPGLLSTFPWLHQVADSFEQYRFR
                     GLVFEYKTMSADSVLSTPASTALGTVIMSTQYNALDQPFTDKRTMENYEYANSSKPSC
                     SFFHPIECKLSQTSVAEMYVRTGPVTTGDLRLYDLGTFTIATQGMQNAAAGSVIGELW
                     LTTEVEFYKPKLLIGGGDLLTDHFQGISPVGFTTAAPFATAGILAPKSGNNLGCTLQI
                     PSVAPYGANTILFPQFVVDGTYQITYSYIGSGATANMPAYSATGTTINIDVVSTSVKV
                     LGSVASPNSYIQAPQAAVTGSNQFIVIQIVKIIQTAPGLQAGISFAFTGGTYPAGSGV
                     VDLIITQVSPTFN*"
     CDS             complement(2432..3346)
                     /codon_start=1
                     /product="Putative Replication Associated Protein"
                     /translation="MPRSAKKGKEGGTGQNACCVYDFTYFGVDVMSVDNMRDTLSKIS
                     KKYCFQLEKGNNTDKLHYQGRFSLKEKLRQSQLIAKLTTYDWEKFHVSVTSTENRDNN
                     FYVCKLDTRVAGPFTEENHRPIPEQVEYMEKMGLYPWQSSIIETLQVYDRRCIDIIYD
                     KLGNKGKSYLSFYMMSKYDCEMLPPLNDVKDLMRMAYDVGPKKVYIFDMPRAMNKEKL
                     FQLYSTIETLKSGFCYDDRYTYKRRLMPHPNLCIFTNVLPNRELLSEDRWRIWNIDKE
                     MKLVKYKEEISEEDLDILCDQFTEEMWD*"
     misc_feature    3035..3054
                     /note="stem-loop"
     misc_feature    3040..3048
                     /note="nonanucleotide"
ORIGIN
        1 atgtcaaaag tagcaagaag acgtacctac acaaaagccg aaaaagcagc atttgccaag
       61 aaaatggcta gagcacgtgc ctcaaggggc cgtgcggtgt catttcgagg caatggtgat
      121 tattcgttat acaaagctcc aagagcgatt agagcgtcta aaagtacgac taagagtctt
      181 ggagggacag tgggagcgca tatagggaac ggtatccaca atttggtcaa ggcgttaaca
      241 ggatttggag actataatat agcggaaaat tcgttaatgt caggaacaat tggtggagac
      301 ccgcctgtta tgcggaacac tgcaaacaat tcatttatag tacatcatcg cgagtacatc
      361 ggggatgtat acgcaacaaa ttcattcaca gccatatcat actcattaaa tccgggattg
      421 ttatcgacat tcccgtggtt acatcaagtt gccgactcgt tcgaacaata cagattcaga
      481 ggacttgttt ttgagtacaa aacaatgtca gctgatagtg tattatcgac accagcttca
      541 acagccctag gtacagttat aatgtcaaca caatataacg ccctagatca gccattcaca
      601 gataaacgga cgatggagaa ttacgagtac gctaattctt caaagcctag ttgttctttt
      661 ttccatccaa tagaatgtaa actttcacaa acatcggtag cggagatgta cgttcgtaca
      721 ggcccggtaa cgactggtga cttgcgacta tatgatttag gcacattcac aatcgccact
      781 caaggcatgc aaaatgccgc agcagggagc gtaattggtg aattgtggct aacaacagaa
      841 gtcgaatttt acaaaccaaa gctgttaata gggggaggcg acctattaac agatcatttc
      901 caaggaatta gtcctgtcgg tttcacaaca gcagcgccgt ttgcaaccgc aggaatactt
      961 gcgccaaaat cagggaataa cttgggttgc accttacaga taccatcggt agcgccatat
     1021 ggtgcaaaca caatattatt tccacaattt gtagtcgacg gaacatatca aataacatac
     1081 tcatacatag gttcaggtgc cactgcaaat atgccagctt attctgcaac tggtactaca
     1141 ataaacatag acgtggtcag tacttcagta aaagtacttg gctcagtcgc atcaccaaac
     1201 tcatatatac aggctcctca agccgcagta acaggttcaa atcaattcat tgtcatacag
     1261 attgttaaga ttatacagac agctccagga cttcaagcag ggataagttt cgccttcaca
     1321 gggggaactt atccagcggg ttccggagtt gtcgatttaa tcattacaca agtctcccca
     1381 acatttaatt aaaatattac aaatacttta attaaaccta aaactatctc atattcccag
     1441 agaaccctgg ccccacgagg atgggagtga gacaagtgag cgtagcgagc gctcgtcgat
     1501 cggaggccga ggctttgata ctagtatcaa aggctttgat actagtatca aagagagcaa
     1561 atcccaccgg gggatttgcg gcgagcaccg taggatgcga gagttaccaa taaactattc
     1621 ttatatttta acgttccagg ctcatggcac agtcgagcct tatccgattt atgtttacaa
     1681 aaaacaggat aaggctcgac tgtgccagtt gtgccaggta gttaaaaata tatatacaaa
     1741 ctttataaaa ccattataaa accctttaaa aggcattttt gaagctctta tcgagagcgt
     1801 agtgttacag gacatttatg tccgccaaaa atgcccattt caaagccttt ttaaattcta
     1861 ccaaacgcag taatgtgggt ccgtgagcat gataagtgag tgtaacgagt gttcatgcgt
     1921 gcgaaccgta ggtgagtcta ggaccccagc atttcaaaga ccgaagctga ccagcggagg
     1981 caccaagcgg aggatttttc taaatccgca gtaaaagcga aggcacatgg agcgaggagc
     2041 gaggcgaacg tagtgaagcc ccagtgccgt agtaaacaaa ccctttttca tacaattatt
     2101 ctcgcagata ataattttat taaatacact aaaacaaaca ccgaagccga ctggcggagg
     2161 tatcaagcgg agaatttttc taaattcgta gtgcaagcgg aggtacatgg agcgaggagc
     2221 gaggcgaacg cagtgaagcc ccagtaccgt agtaaacact caacaaacac cgaagctgac
     2281 cagcggaggc accaagcgga ggatttttct aaatccgcag taaaagcgaa ggcacatgga
     2341 gcgaggagcg aggcgaacgt agtgaagccc cagtgccgta gtaaacaaac ccatttttaa
     2401 ttcattaaat aaaatttaat taattaacaa ttcaatccca catctcttca gtaaattggt
     2461 cacataatat atctaaatcc tcttcactta tttcttcctt atatttaacc aatttcattt
     2521 ctttatcaat attccatatt ctccatctat cttcacttaa taattctcta tttggtaaga
     2581 cattggtaaa aatacataag ttaggatgag gcatcagccg tcttttgtat gtatacctat
     2641 cgtcgtagca aaaaccactc tttagcgttt cgattgtgct gtagagctga aatagttttt
     2701 ctttgttcat ggccctaggc atgtcaaaga tataaacttt tttaggtcct acatcataag
     2761 ccatacgcat taaatcctta acatcattaa gaggaggcaa catttcacaa tcatatttgc
     2821 tcatcatata gaatgacaaa taggatttac ctttatttcc caacttatca taaattatat
     2881 caatacaacg cctatcataa acttgtaaag tttcaatgat agagctttgc caaggatata
     2941 aacccatctt ttccatgtat tcaacttgtt caggtatagg gcggtgattt tcttcagtaa
     3001 aaggacccgc tacacgtgta tcaagcttgc atacatagaa attattatct ctattttccg
     3061 tcgacgttac agacacatga aatttttccc aatcatacgt agtaagtttg gcgattaatt
     3121 gactttgtct taacttttcc tttaacgaga aacgcccttg atagtggagt ttatcggtat
     3181 tattaccttt ttctaattga aaacaatact tcttacttat tttacttaat gtatctctca
     3241 tattatctac actcatcaca tctacaccaa aataagtaaa atcatacaca caacatgcgt
     3301 tctgacctgt tccaccttct ttccctttct tggcacttct tggcatctat tatacccggg
     3361 agaaaataaa atgtaaattt attttctacc gttataatag ca
//
LOCUS       CruV-242                3403 bp    DNA     circular     24-FEB-2020
DEFINITION  Crucivirus-242 isolate 120_869_SvL1_3295.
ACCESSION   CruV-242
KEYWORDS    .
SOURCE      Crucivirus-242
  ORGANISM  Crucivirus-242
            Unclassified.
REFERENCE   1  (bases 1 to 3403)
  AUTHORS   de la Higuera,I., Kasun,G.W., Torrance,E.L., Pratt,A.A.,
            Maluenda,A., Colombet,J., Bisseux,M., Ravet,V., Dayaram,A.,
            Stainton,D., Kraberger,S., Zawar-Reza,P., Goldstien,S., Briskie,J.,
            White,R., Taylor,H., Gomez,C., Ainley,D.G., Harding,J.S.,
            Fontenele,R.S., Schreck,J., Ribeiro,S.G., Oswald,S.A., Arnold,J.M.,
            Varsani,A., Enault,F. and Stedman,K.M.
  TITLE     Direct Submission
  JOURNAL   Submitted (24-FEB-2020) Biology, Portland State University, 1719 SW
            10th Ave, Portland, Oregon 97201, USA
COMMENT     Bankit Comment: ALT EMAIL:ide@pdx.edu
            Bankit Comment: TOTAL # OF SEQS:451
            Bankit Comment: GAP: est. length - alignment

            ##Assembly-Data-START##
            Assembly Method       :: MetaSPAdes v. 3.12; IDBA-UD v. 1.1.1;
                                     MEGAHIT v. 1.0.3
            Sequencing Technology :: 454; Illumina
            ##Assembly-Data-END##
FEATURES             Location/Qualifiers
     source          1..3403
                     /organism="Crucivirus-242"
                     /mol_type="genomic DNA"
                     /isolate="120_869_SvL1_3295"
                     /isolation_source="Water"
                     /country="Norway"
                     /collection_date=2012
     CDS             1..1188
                     /codon_start=1
                     /product="Putative Replication Associated Protein"
                     /translation="MTVGMSRTPLKHYAITISRRVPPGTKVQNEVPLRSHETVIQWLI
                     KVAKHYIFQGEEGGTGYPHFQGYIELEARKRIQTLAKETKALTDLPVHFSPASSEGIE
                     ALKKYCMKKDTRTEGPWQDGKPIYEGEDLYTEPYPWQKTLEELIDTTPDKRSIIWVYD
                     QEGNQGKSEFVKKMFILKKAGVLSYGKTSDMLNIVKKHMNCKAFLVDLTRAKPLDIGK
                     DDLYATIEQIKNGMVIASKYDGDSFAFRSPHVIVMANHLPNFHTLSNDRWKVKTIKEK
                     ALITLSAEEIEKELQRKTPPAIPKKATSRPVAYAPWRAARFQEPSASIPQVVVAGSSS
                     TRFARKKNAPLGLEHIPKLTPINPSLNAFYEHLRQKREPKNEAEKALEMQENYECFDY
                     ACA*"
     CDS             complement(1222..1599)
                     /codon_start=1
                     /product="putative ORF"
                     /translation="MLLTSGKISFPSFSEDKVFYVSFNYKWVVPSVNITTELTITPVG
                     CSSLPFFLNGATGQSSTLSALTTKADIINHVFSISAGSEAELTCSTDSATWTSNPDYF
                     DLVVAELDPAISSSLYESDLVIV*"
     CDS             complement(1717..3054)
                     /codon_start=1
                     /product="Putative CruV Capsid Protein"
                     /translation="MSSASSLRRSYLRRRRVPAYPNPVRPRYGRIRGSGAYSGSGLKK
                     LVEKYVRPYTKGFLGKAGGRVGAALGRTLLGDTRANGLAGGLAGKTMGDWLGKITGLG
                     DYKMQRNSLMSNPNVPFMHSSQDGVRIRHREFIQDVSSSTAFTNTSFAMNPGLDTTFP
                     WLSAIAQNFEEYRFEGLAFEFNSTSSDALNSTNTALGTLILAAEYNSAQSAYINKQQM
                     ENSMWSVSTKPSCNLLMPIECAPSLNPIANQYIRTGAVPSGQDIRLYDLCNVQVATVG
                     SQASAVIGELWITYDVVLLKPQLDSGLALSADTAHYRLTAPVVTTAYFGTARTAMFDS
                     IGLTLSGTVLTFPIGSNGVYLITYSVAGDSTAVVRPTITLANATNAPSLWTVGADEFI
                     DNSGSTSTRYLLSYAITISDPGLIATATFSIGTLPANPTSGDLIVTQLNGNVA*"
ORIGIN
        1 ttgaccgttg gaatgagtag aacaccactc aaacactatg caatcacaat atcgcgacgc
       61 gtaccaccgg gtaccaaagt acaaaacgaa gtacctctac gaagccacga aaccgtaatc
      121 caatggctta taaaggtggc taaacactac atttttcagg gtgaggaagg tggtaccgga
      181 tatcctcact ttcaagggta cattgaactt gaagccagaa aacgtatcca aacactagca
      241 aaagagacta aagcccttac agaccttcca gtacatttta gtccagcatc gtctgaaggc
      301 atcgaggcac taaagaagta ctgcatgaag aaggatacta gaacagaagg accctggcaa
      361 gatgggaaac ccatttacga gggtgaagac ctctacactg agccctatcc atggcaaaag
      421 actctagaag agttaatcga tacaacacca gacaaacgaa gcatcatctg ggtttacgac
      481 caggaaggaa accagggtaa gtctgaattc gtcaaaaaga tgttcatcct caagaaggct
      541 ggagttcttt cgtatggaaa gacgtcagac atgcttaaca ttgtaaagaa acacatgaac
      601 tgtaaagcgt ttcttgtaga cttgacaagg gcaaagcctc tggacattgg aaaagatgac
      661 ctttacgcaa cgatagaaca gatcaagaac ggaatggtta ttgcttcaaa gtacgatggc
      721 gactcatttg cctttagaag cccacacgta atagtgatgg cgaaccactt accgaacttc
      781 catacactct caaatgatag atggaaagtt aaaacaatca aggaaaaagc cctaataacc
      841 ctttctgctg aagaaattga aaaagaattg caaagaaaga cacctccggc gatcccgaaa
      901 aaggctacat cacgccccgt cgcttacgct ccgtggcgcg ccgcacgttt tcaagaaccc
      961 tctgcctcaa taccacaagt cgtcgtcgca ggctcctcct ccactcgctt cgctcggaag
     1021 aagaacgctc cgctggggtt ggagcacatc cccaaactaa cccctataaa ccctagtcta
     1081 aacgcgttct acgaacactt aagacaaaaa agggaaccca aaaatgaggc agaaaaagca
     1141 ttagaaatgc aagaaaacta cgaatgtttt gattatgctt gcgcatgatt tattaataaa
     1201 ctaaacccta atagggaacc cctaaactat aaccaaatca ctctcataga gagaactcga
     1261 gatagcagga tccaattcag caacgaccaa gtcgaaataa tcaggattgg atgtccaagt
     1321 cgcagagtcc gtagaacacg tcaactctgc ctcagacccg gcagaaatgc taaagacatg
     1381 gttgataata tccgcctttg tagtcaaggc agacaacgtc gaagactgtc cggtagcccc
     1441 attcaagaag aacgggaggg aactacaacc cactggggta atggtcaatt cagttgtgat
     1501 attgactgaa ggtacaaccc atttgtagtt gaaagataca tagaatacct tatcctcaga
     1561 gaaggatggg aaggagatct ttccagatgt gagaagcaac ccaatactat cgtatcgttg
     1621 tgttctcgta gcacctaacg gaaaagtagg ttcggcactt gtagaagagt aatgtgcgga
     1681 ttgaacaaca cagtcgtcac ccacatccga atcttcctaa gcaacattgc catttaactg
     1741 agtcacgatc aagtcgccgg aagtaggatt ggcaggaagg gtacctattg aaaaggtagc
     1801 cgttgcaatc aatccgggat cagaaatggt tatggcataa ctcaagagat aacgagtaga
     1861 cgtactcccc gaattatcaa taaactcatc ggctccaaca gtccacagag atggagcatt
     1921 cgtcgcatta gccaaagtaa tggttgggcg aacgactgca gtggagtcac ctgcaacaga
     1981 gtaggtgatg agataaacac catttgaacc aattggaaac gtcaagacgg tacctgaaag
     2041 agtcagtcca atagagtcaa acattgccgt acgagcagtc ccaaaatagg cggtcgtaac
     2101 cacaggtgcg gtcaaacggt aatgtgcagt gtctgcactt agtgcaagac ccgagtcgag
     2161 ttgaggcttc aacagaacga catcgtaggt aatccaaagt tccccaataa cggcggatgc
     2221 ctgggacccc acggtagcaa cctgaacatt gcacaagtca tacaagcgga tgtcctgacc
     2281 ggaaggcacc gcacccgttc ggatgtactg atttgcaata ggattaagcg aaggagcgca
     2341 ctcaataggc atcaacaagt tacaagaagg cttggttgac acagaccaca tggaattctc
     2401 catctgttgc ttattgatgt aggctgactg agcagaattg tactcagcag caagaatgag
     2461 ggtacccaac gctgtgttag ttgagttcag agcatcagaa gacgtcgaat tgaactcaaa
     2521 agcaagcccc tcaaaacgat actcctcaaa gttctgcgcg atagcagaca accatggaaa
     2581 ggtagtatca agaccaggat tcatagcaaa cgaagtgttt gtaaacgccg tacttgaaga
     2641 aacatcctga ataaattcac gatgacggat acggacacca tcctgagaac tgtgcatgaa
     2701 cggcacattg ggattggaca taagtgaatt acgttgcatc ttgtaatcac ccaaaccagt
     2761 gatcttacca agccaatccc ccattgtctt tccggctaaa cctccagcca aaccattcgc
     2821 cctagtatca ccaagcagcg tacgccctaa agcggcaccc acacgaccac cagccttacc
     2881 aaggaaaccc ttggtgtagg ggcgaacata cttctctacc agtttcttaa gaccagaacc
     2941 agagtaggcg ccagaaccac ggatcctacc ataacgagga cgtactggat taggatatgc
     3001 cgggactcta cggcgacgaa gataagaacg acggagactt gatgctgaag acatgacaat
     3061 atttatttaa aataataaaa ttcaagatca tccgagcacc ctaataaaat tatggaaccc
     3121 tctaaagacc tctaaaagtg ggaataaccc cttcaaaacc ccacaggagg agataccccc
     3181 ataacccccc ataaattaat ggatacattt attcatacta aataaatatt ccataattcc
     3241 accactcggg ttttggtcaa aaccctcagg aaccccctaa aggggacccc caaggaggca
     3301 ctacgcttcg ctccgttgta acagtcttgg gggtccctgc tcggacgatg tcctctctgg
     3361 gttttataat ataaataagt actattattt aattatttgt ttt
//
LOCUS       CruV-243                3405 bp    DNA     circular     24-FEB-2020
DEFINITION  Crucivirus-243 isolate SR3_42497.
ACCESSION   CruV-243
KEYWORDS    .
SOURCE      Crucivirus-243
  ORGANISM  Crucivirus-243
            Unclassified.
REFERENCE   1  (bases 1 to 3405)
  AUTHORS   de la Higuera,I., Kasun,G.W., Torrance,E.L., Pratt,A.A.,
            Maluenda,A., Colombet,J., Bisseux,M., Ravet,V., Dayaram,A.,
            Stainton,D., Kraberger,S., Zawar-Reza,P., Goldstien,S., Briskie,J.,
            White,R., Taylor,H., Gomez,C., Ainley,D.G., Harding,J.S.,
            Fontenele,R.S., Schreck,J., Ribeiro,S.G., Oswald,S.A., Arnold,J.M.,
            Varsani,A., Enault,F. and Stedman,K.M.
  TITLE     Direct Submission
  JOURNAL   Submitted (24-FEB-2020) Biology, Portland State University, 1719 SW
            10th Ave, Portland, Oregon 97201, USA
COMMENT     Bankit Comment: ALT EMAIL:ide@pdx.edu
            Bankit Comment: TOTAL # OF SEQS:451
            Bankit Comment: GAP: est. length - alignment

            ##Assembly-Data-START##
            Assembly Method       :: MetaSPAdes v. 3.12; IDBA-UD v. 1.1.1;
                                     MEGAHIT v. 1.0.3
            Sequencing Technology :: 454; Illumina
            ##Assembly-Data-END##
FEATURES             Location/Qualifiers
     source          1..3405
                     /organism="Crucivirus-243"
                     /mol_type="genomic DNA"
                     /isolate="SR3_42497"
                     /isolation_source="Water"
                     /country="New Zealand"
                     /collection_date=2012
     CDS             join(3176..3405,1..196)
                     /codon_start=1
                     /product="putative ORF"
                     /translation="MFFLTQILARAPRRSVLARSWGLGPPTQCAPMPTPYNTTQANLS
                     KIPHSTLSRLVVAAGRPPGWLFLAGWCRLCPGYIVTPGLPGAGATTCLAGGGWLLFWL
                     AGGGWLHRPASSGGGCWPAPIPGWPRAPWGGRGPGQARL*"
     CDS             join(complement(1..237),complement(3280..3405))
                     /codon_start=1
                     /product="putative ORF"
                     /translation="MTTRAFLEKHVPWPHSLAWPGPRPPQGARGQPGMGAGQQPPPEE
                     AGRWSQPPPASQKRSQPPPAKHVVAPAPGSPGVTIYPGQSRHQPAKKSQPGGRPAATT
                     SLLNVEWGILLKFACVVL*"
     misc_feature    join(3396..3405,1..17)
                     /note="stem-loop"
     misc_feature    1..9
                     /note="nonanucleotide"
     CDS             238..1755
                     /codon_start=1
                     /product="Putative CruV Capsid Protein"
                     /translation="MKRQREETQLVPYQPVTKRIKMDVQRAPYQRGPGFYRGKPLGPL
                     KAPKYTDRMWRVRRPRRKTPKAMAQYWRKRYRRRVLTGRGDYSYSDSRSTGANLGGWL
                     GSKAGEYLGGMAQTAGMALISGLGDYTVNKNVLLSGNMPSIQNMTSGAGITISFQEYL
                     GDVVTPLQVGNSSPFDVTTYVINASNPRTFPWLSQIAQNFEQYEMEGMLFGFRTTSAD
                     LVTNGTNNLQLGTVIMATQYDVADPNFASKGEMLNYQFSTSVKPSQPAIHMIECDPRQ
                     TSVNLLYTGTAAAGTDPRLYNLGRFSIATQGFQGSGIVIGELHVTYQVRLLKPKLWTT
                     LGENIPWSSGIYTMSSGSTKSWLGVSGTIATQTINNSNQTVSIDNNLDITQVQSKVIF
                     QRQRFPQRIYILIAWSAQSGATGGFFNTSDGNPVFSAGGGATLISQQIYPLSATNDSI
                     TGSFMGIVDIAPDQSAPYFLIKSFTSTFQPDYPNGQIIARLTLAPTDPTGNGNLN*"
     CDS             complement(1871..2881)
                     /codon_start=1
                     /product="Putative Replication Associated Protein"
                     /translation="MKSSSSSSSSSEQVEDPIEHETELVISPYAGKPPARGERGNAQV
                     CWLYTWFNYDPEKLPVVPDTECKYRIEGKELTPTKKTPHVQGFVIFKTKQRWSTVTTN
                     YPGINWLPMCKKATPWNNFEYCSKDGDFTEFGERPKQPKASGRGNDQQAFAEAFAADS
                     VGAGIQIIKEKRPRDLALHGEAIERNLKKAKTEQWSHKYRENSFLVPLQDTGSNLLVH
                     GPSNTGKTHYAAAHFENPLVCSHVDDLKRLGPDNDGIIFDDMSFKHWPVEAIIHLLDR
                     EFGRSINVRYGTVQIRAGIPKIFTHNTRNPFYEEATIEEEQKIAIERRFRRVNVLNKL
                     YA*"
ORIGIN
        1 tatagtaacc ccagggctcc ccggagccgg agccaccacg tgcttggctg gtggtggctg
       61 gctcctcttc tggctggctg gtggtggttg gctccaccgg ccggcctctt ctggtggtgg
      121 ctgctggccg gctcccatcc ctggctggcc ccgcgccccc tggggggggc gcggccccgg
      181 ccaggccagg ctgtgaggcc aaggcacgtg tttttccaag aacgcacgtg ttgtcatatg
      241 aagcggcaac gtgaagagac ccaactcgtc ccttatcaac ctgttaccaa acgaatcaaa
      301 atggacgtac aacgagcacc ctatcaacga ggacctggat tttaccgtgg aaaacctctt
      361 ggcccgctta aagcacctaa atacacagat cgaatgtggc gagttcgccg accaaggcga
      421 aaaactccta aagcaatggc tcaatactgg cgaaagcgat accgaagacg agtcctcact
      481 ggacgaggag attatagtta ttcagatagt cgatctactg gtgcgaatct tggaggatgg
      541 cttggaagca aagcgggtga atacctcgga ggtatggccc aaaccgcagg aatggcctta
      601 atatccggtt tgggtgatta tactgttaac aaaaacgtgc tactatctgg aaacatgcct
      661 tcaattcaaa atatgacctc tggtgctgga atcactattt cctttcaaga atatcttggt
      721 gacgttgtca ctccactaca agtgggaaat tcttcacctt tcgatgttac aacatatgtt
      781 attaatgctt caaatcctcg aacattccct tggttatcac aaattgcaca aaattttgaa
      841 caatatgaaa tggaaggaat gctctttggt tttaggacaa cttcagctga cttagttact
      901 aatggcacaa ataacttaca acttggaact gttattatgg ctactcaata tgatgtagct
      961 gatccaaact ttgcatctaa aggtgaaatg ctcaattacc aattttcaac atctgttaaa
     1021 ccctcacaac ctgcaataca catgatagaa tgtgacccta gacaaacctc agtcaatctc
     1081 ctttatactg gtaccgcggc tgctggtacc gatcctcgac tttacaatct tgggagattt
     1141 tcaatcgcta ctcaaggttt tcaaggttct ggtattgtaa ttggtgaact acacgttacc
     1201 tatcaagtca gacttcttaa accaaaatta tggactacac ttggagaaaa tatcccttgg
     1261 agttcaggca tttatactat gagttcagga tcaactaaaa gttggcttgg tgtttctggt
     1321 actattgcta cacagactat taataattct aatcaaacgg tatcgataga taacaatctt
     1381 gatattaccc aggtacaatc aaaagttata ttccaacgtc aacgattccc tcaacgtata
     1441 tacatactta tagcatggtc agctcaatct ggagcaacag gaggattttt taatacctct
     1501 gatggtaatc ccgttttttc tgctggtggc ggtgcaacac tcatatcaca acaaatctac
     1561 cccctctctg ctactaatga ctctattact ggatctttta tgggaattgt agatattgcc
     1621 cctgatcagt cagcacctta ctttttgatt aaatcattca catctacatt ccaacccgat
     1681 tacccaaatg gtcaaattat tgcgagatta actcttgcac ctacagaccc aactggtaat
     1741 ggcaatctta attgatccag caaccacatt accgcggctg cggacttata tattttttgg
     1801 gttaaaatgt caaacaaagt aataaacaac agtttagttc aaatataatt tattaaaatc
     1861 aacaatatac ttaagcgtat aatttattta agacatttac tcgtcgaaat cgacgctcaa
     1921 tagctatctt ttgctcctct tcaatagtgg cttcttcata gaacgggttt cttgtattgt
     1981 gggtaaagat ctttgggatt ccagctctga tttggactgt tccatatctg acattgattg
     2041 atcttccgaa ctccctatct agcaaatgaa taatggcttc aactggccag tgtttgaatg
     2101 acatatcatc gaagattatt ccatcattat caggccccaa cctctttaaa tcgtcaacat
     2161 gtgaacatac taagggattt tcgaaatggg ctgcagcata atgcgtcttt cctgtatttg
     2221 agggtccatg taccaataga tttgatcccg tgtcttgtag tggtacaagg aagctattct
     2281 ctctgtattt gtgagaccac tgttcggtct tggctttttt aaggtttctt tcaatggctt
     2341 ccccgtgtag agccaagtca cgtggtcttt tctccttgat gatttgtatt ccggctccaa
     2401 cactatcagc ggcaaaggct tcagcgaagg cttgttgatc gttaccgcgg ccgctggcct
     2461 taggttgttt gggtctttct ccaaactcag tgaaatctcc gtccttacta cagtactcga
     2521 agttgttcca aggtgtggct tttttgcaca tgggcaacca gttgattcct ggataattgg
     2581 ttgtaactgt tgaccatctc tgcttggtct taaaaatgac aaatccttgc acgtgtggag
     2641 tcttttttgt tggtgtaagt tcctttcctt caattctgta cttgcattcc gtgtcgggca
     2701 cgactggtaa cttttcggga tcataattga accacgtgta aagccaacag acttgtgcgt
     2761 ttcctctctc cccacgtgca ggtggctttc ctgcgtatgg actaatgacc agttccgttt
     2821 catgttcaat aggatcctca acctgttcag atgatgatga tgatgatgat gatgatttca
     2881 tatatgatta ccgaaagagt agacgctgat gggtataatc cttatcggta ccttacagtc
     2941 gagaactcaa tgggtatgat ttataacccc ccctatataa gtatagaatt cgactcgaac
     3001 tcttttcaga tttgtactta tactatatga gggctcccca tttgggaaag ttttcaaata
     3061 ttttttttag aaattttggc tatttgtttt tttatactgt aaaaacatta gaaaccgttt
     3121 tttggctatt tgttttttta tactgtaaaa acattagaaa ccgttttttg gctatttgtt
     3181 ttttttaacc caaatcttag ccagagcgcc gagaaggtcg gtgctagcac ggtcgtgggg
     3241 gctggggccc ccgacccagt gcgcgcctat gcccacaccc tacaacacca cacaagcgaa
     3301 tttaagcaaa attccccact ctacattaag caggctggtg gtggctgctg gccggcctcc
     3361 tggctggctc ttcttggctg gctggtgccg gctctgccct gggta
//
LOCUS       CruV-244                3405 bp    DNA     circular     24-FEB-2020
DEFINITION  Crucivirus-244 isolate 120_1360_IR2_3297.
ACCESSION   CruV-244
KEYWORDS    .
SOURCE      Crucivirus-244
  ORGANISM  Crucivirus-244
            Unclassified.
REFERENCE   1  (bases 1 to 3405)
  AUTHORS   de la Higuera,I., Kasun,G.W., Torrance,E.L., Pratt,A.A.,
            Maluenda,A., Colombet,J., Bisseux,M., Ravet,V., Dayaram,A.,
            Stainton,D., Kraberger,S., Zawar-Reza,P., Goldstien,S., Briskie,J.,
            White,R., Taylor,H., Gomez,C., Ainley,D.G., Harding,J.S.,
            Fontenele,R.S., Schreck,J., Ribeiro,S.G., Oswald,S.A., Arnold,J.M.,
            Varsani,A., Enault,F. and Stedman,K.M.
  TITLE     Direct Submission
  JOURNAL   Submitted (24-FEB-2020) Biology, Portland State University, 1719 SW
            10th Ave, Portland, Oregon 97201, USA
COMMENT     Bankit Comment: ALT EMAIL:ide@pdx.edu
            Bankit Comment: TOTAL # OF SEQS:451
            Bankit Comment: GAP: est. length - alignment

            ##Assembly-Data-START##
            Assembly Method       :: MetaSPAdes v. 3.12; IDBA-UD v. 1.1.1;
                                     MEGAHIT v. 1.0.3
            Sequencing Technology :: 454; Illumina
            ##Assembly-Data-END##
FEATURES             Location/Qualifiers
     source          1..3405
                     /organism="Crucivirus-244"
                     /mol_type="genomic DNA"
                     /isolate="120_1360_IR2_3297"
                     /isolation_source="Water"
                     /country="Norway"
                     /collection_date=2011
     CDS             1..945
                     /codon_start=1
                     /product="Putative Replication Associated Protein"
                     /translation="MEMSISARRWCFTSFNVDGVVPDFKYESYCVYQKERAPDTGREH
                     FQGYVQFTRPVRRSALVKVLPGAHFEPQSSQSSNEQARVYCMKEETRVSAFVERGDFK
                     EEQPGKRSDIEAVVALIEAGASEKEIRREMPMMAAKYGRFIRDRRIDFLEEKSVSLGQ
                     VVLREWQSYIMSVIDGPVNERYIYWIYDDAGNNGKTFLSKYLVENYKAFYCRGGKTAD
                     IAYAYEYQSVVICDYARESAEFVQYNVLEAFKDGMLFSSKYESRMKKFASPHVFVMAN
                     FQPDYTKLSVDRIQLLSLSNGRATLRNGASLVKTIIVN*"
     CDS             1022..2569
                     /codon_start=1
                     /product="Putative CruV Capsid Protein"
                     /translation="MEEKDIPRSNKKRRADGVARGAYYPNIKPAGQLRNFMSQKYPKG
                     SPASQALWGATYKEADQQQKLTRLALRYKGDGDYKKFLRGASRAVGGAAGAVLGGIGK
                     SELGLGGMAMGALAGGRSGYDQGANFSKYMGWGDYSGGSVSGNQLIDGTGSQQRIEVN
                     PMNNTGDIFISHSEFVQNVTCSSAGATPSLFQVTDYPVNPGLSQTFPFLSQLAQCFVL
                     YELQGLMFHYKPNSGESGSASNSLGKVILATNYDPDALPYVNSIQMENYDYANSSKPS
                     LGMVHGVETAVHQGVTNMLYVRNGSVTKSKVFTDIGVFSVATEGIPFAGAGTAILGEL
                     WVTYKIRLSRANLYGSLLGLNIKQDVFSGTASAAALILTQTIKTSNQIGCVIANVSAT
                     AMSITFPQNIALGAYSVSVQYEDSVTPLGAKAFATPTLLTNLTMFVPARQLALSTGNS
                     GFSAPRTPLGAASNLAIAGTWFVTVNAPGTSQASFQVNVAAALTAATTFTIFISQVNA
                     NVGLTLA*"
     CDS             complement(2625..2927)
                     /codon_start=1
                     /product="putative ORF"
                     /translation="MECHICSIERTVMVFCKTCHNPVCLGCHGQMQRCPYCRSSYIPP
                     RRVFSSRPIPIPTAPRLVRTNTGRAMRIYPAPPTPPSDNSFDEWGSSDEDTFSITR*"
ORIGIN
        1 ctggaaatgt caatctcggc aagaaggtgg tgttttacgt cgttcaatgt ggatggtgtt
       61 gtgcctgact tcaagtatga gagttattgt gtttaccaaa aggaacgggc ccctgatact
      121 ggacgtgaac atttccaggg ttatgtccag tttactcggc ccgttcgacg atcagctctt
      181 gtaaaggtac ttcctggagc acattttgaa ccgcaatcca gccagtctag caatgagcaa
      241 gcacgagtgt attgtatgaa agaagaaaca cgcgtgtctg cgtttgttga acgaggcgat
      301 tttaaggagg agcaaccggg taaacggtct gacattgaag cggttgttgc tcttatagaa
      361 gccggggcgt cggagaagga gataagacga gaaatgccaa tgatggccgc gaagtatggg
      421 aggtttattc gagatagacg tattgatttt ctcgaagaaa aatccgttag tttgggccaa
      481 gttgttttac gcgaatggca atcttatatt atgtcggtta ttgatgggcc tgttaatgaa
      541 cgatatattt attggatata tgatgatgcg ggtaacaacg ggaagacatt cttgtctaag
      601 tatctagtgg agaattataa agcattttat tgtcgggggg gtaaaacggc tgatatcgct
      661 tatgcatatg agtatcaatc tgttgttata tgcgattatg ctagagagag tgctgagttc
      721 gtgcagtata acgttttaga agcatttaaa gacggtatgc tatttagttc gaagtacgaa
      781 tcgcggatga agaagtttgc ctctccccac gtctttgtta tggcgaattt tcaaccagac
      841 tatacgaagt tatcagttga tagaattcag ctactgtcgt taagtaacgg tcgtgcaaca
      901 ttgcgtaacg gcgcgtctct tgtgaaaact attatagtaa attaaagcat ttgtgcattc
      961 atttacacta ttttagtgaa acgcgtgtat ctatatatta tacttcgcat acgttgttgc
     1021 tatggaagag aaagatatac ctagaagtaa taagaaacgt cgtgccgatg gagtagctag
     1081 gggggcttat tatcctaata ttaagcccgc gggacaattg aggaacttta tgtctcagaa
     1141 atatccgaaa ggttctcccg ctagtcaggc attgtggggt gcaacctaca aagaggccga
     1201 tcaacaacaa aaacttactc gtcttgcgtt gcgctataaa ggggatggcg actataaaaa
     1261 atttttaaga ggagcctcta gggctgtagg cggtgctgcc ggcgctgttt taggaggtat
     1321 aggtaaatca gagctgggat taggaggtat ggccatgggt gctttggcgg gaggtcgttc
     1381 gggttatgac cagggagcta atttctccaa atatatgggt tggggtgatt attcaggtgg
     1441 atctgtatca ggtaatcaac ttattgatgg aaccggatct cagcagcgta tagaagtcaa
     1501 cccaatgaat aatactggag atattttcat ctctcattca gaattcgttc agaacgtcac
     1561 gtgttcaagc gcaggtgcta cgccttcgtt attccaagtc actgactacc cggttaaccc
     1621 tgggctttcg cagacttttc cgtttttgtc gcagttggca cagtgctttg tgctttatga
     1681 actgcagggt ttgatgtttc attataaacc caacagtgga gaaagtggat ctgctagtaa
     1741 ttcattaggg aaggttatat tggcaactaa ttacgaccct gatgctcttc cttatgtcaa
     1801 tagtattcag atggagaatt atgattacgc taattcgtcc aagccgtctt taggtatggt
     1861 gcacggtgtg gaaacggcag ttcatcaagg agtaactaat atgctgtatg ttcgtaacgg
     1921 atctgttact aagagtaaag tatttacaga tatcggtgta ttttctgttg ctacagaggg
     1981 tattccattt gcgggtgcag gtactgctat tttaggtgaa ttgtgggtta catataagat
     2041 tcgactgtca agagctaact tatatggttc attactggga ctcaatatta agcaagacgt
     2101 ttttagtggg actgcgtcag ctgctgcatt aattttaact cagacaatta aaacctcgaa
     2161 ccaaattgga tgtgttattg ctaatgttag tgcgacggcc atgtcgatta cctttcctca
     2221 gaatattgcg ctaggggcat attccgtatc agttcagtat gaagattcgg ttacgccttt
     2281 aggtgccaaa gccttcgcaa cgcctacact tctaacgaac ttgactatgt ttgtccctgc
     2341 gcgtcaatta gccttgagta caggtaattc gggttttagt gcgccgcgta ctccacttgg
     2401 cgctgcctct aatttagcga ttgcgggtac ttggtttgtc acagttaatg ctccgggcac
     2461 gtctcaagca agttttcaag ttaacgtagc agctgctttg actgccgcca ctacattcac
     2521 tatttttatt agtcaggtta atgcgaatgt aggtttaacc cttgcataag ctttaaacac
     2581 ggggttttta gtccaatgag gttttcccca ataaacatat tttattatct tgtaattgaa
     2641 aatgtatctt catctgacga cccccattcg tcgaacgagt tatctgaagg gggtgtagga
     2701 ggtgctggat aaatcctcat ggctcgaccc gtgttagttc ttaccaatct tggagccgtc
     2761 ggaattggta ttggtcttga agaaaatact cttcttggtg gaatatagct agatctacag
     2821 taaggacatc tttgcatttg tccatggcat cctaaacaca ctgggttatg acatgtcttg
     2881 caaaatacca ttactgttct ttcaattgaa catatatgac actccattta tttgctgaat
     2941 gaccttgtgc gcacattaac agcatgccaa tgctgtgaat atttttttat accattaggc
     3001 gtagtccata taattatttg tataagaagt gcgcactgga aaaataaaag ttgtctgtgc
     3061 gtggaatttt ctggaatttt ctgtccttaa tgcagcgggg cccttgccct caatttatag
     3121 agcagtccaa agcttgcgac gacccgatat agggcactga tcccgcgtcg gcttttgtct
     3181 ttgggctctt caatgagggt ctattaaccg caaagtacaa gtggcgacaa atagcaacgt
     3241 agtaagcaac ccattatcac gaagtgatac aaggttgatt acggagttgg tatagtatta
     3301 ccgccacctt gtatcatgta tcaaaacaaa aaaagtaacg tgatgcaaca tttgaaacgg
     3361 tttcatgctt gcataaaaaa tcgttgaata gttgcatcat tcatt
//
LOCUS       CruV-245                3406 bp    DNA     circular     24-FEB-2020
DEFINITION  Crucivirus-245 isolate H7_22978.
ACCESSION   CruV-245
KEYWORDS    .
SOURCE      Crucivirus-245
  ORGANISM  Crucivirus-245
            Unclassified.
REFERENCE   1  (bases 1 to 3406)
  AUTHORS   de la Higuera,I., Kasun,G.W., Torrance,E.L., Pratt,A.A.,
            Maluenda,A., Colombet,J., Bisseux,M., Ravet,V., Dayaram,A.,
            Stainton,D., Kraberger,S., Zawar-Reza,P., Goldstien,S., Briskie,J.,
            White,R., Taylor,H., Gomez,C., Ainley,D.G., Harding,J.S.,
            Fontenele,R.S., Schreck,J., Ribeiro,S.G., Oswald,S.A., Arnold,J.M.,
            Varsani,A., Enault,F. and Stedman,K.M.
  TITLE     Direct Submission
  JOURNAL   Submitted (24-FEB-2020) Biology, Portland State University, 1719 SW
            10th Ave, Portland, Oregon 97201, USA
COMMENT     Bankit Comment: ALT EMAIL:ide@pdx.edu
            Bankit Comment: TOTAL # OF SEQS:451
            Bankit Comment: GAP: est. length - alignment

            ##Assembly-Data-START##
            Assembly Method       :: MetaSPAdes v. 3.12; IDBA-UD v. 1.1.1;
                                     MEGAHIT v. 1.0.3
            Sequencing Technology :: 454; Illumina
            ##Assembly-Data-END##
FEATURES             Location/Qualifiers
     source          1..3406
                     /organism="Crucivirus-245"
                     /mol_type="genomic DNA"
                     /isolate="H7_22978"
                     /isolation_source="Water"
                     /country="New Zealand"
                     /collection_date=2012
     misc_feature    join(3397..3406,1..17)
                     /note="stem-loop"
     misc_feature    1..9
                     /note="nonanucleotide"
     CDS             239..1756
                     /codon_start=1
                     /product="Putative CruV Capsid Protein"
                     /translation="MKRQREETQLVPYQPVTKRIKMDVQRAPYQRGPGFYRGKPLGPL
                     KAPKYTDRMWRVRRPRRKTPKAMAQYWRKRYRRRVLTGRGDYSYSDSRSTGANLGGWL
                     GSKAGEYLGGMAQTAGMALISGLGDYTVNKNVLLSGNMPSIQNMTSGAGITISFQEYL
                     GDVVTPLQVGNSSPFDVTTYVINASNPRTFPWLSQIAQNFEQYEMEGMLFGFRTTSAD
                     LVTNGTNNLQLGTVIMATQYDVADPNFASKGEMLNYQFSTSVKPSQPAIHMIECDPRQ
                     TSVNLLYTGTAAAGTDPRLYNLGRFSIATQGFQGSGIVIGELHVTYQVRLLKPKLWTT
                     LGENIPWSSGIYTMSSGSTKSWLGVSGTIATQTINNSNQTVSIDNNLDITQVQSKVIF
                     QRQRFPQRIYILIAWSAQSGATGGFFNTSDGNPVFSAGGGATLISQQIYPLSATNDSI
                     TGSFMGIVDIAPDQSAPYFLIKSFTSTFQPDYPNGQIIARLTLAPTDPTGNGNLN*"
     CDS             complement(1872..2882)
                     /codon_start=1
                     /product="Putative Replication Associated Protein"
                     /translation="MKSSSSSSSSSEQVEDPIEHETELVISPYAGKPPARGERGNAQV
                     CWLYTWFNYDPEKLPVVPDTECKYRIEGKELTPTKKTPHVQGFVIFKTKQRWSTVTTN
                     YPGINWLPMCKKATPWNNFEYCSKDGDFTEFGERPKQPKASGRGNDQQAFAEAFAADS
                     VGAGIQIIKEKRPRDLALHGEAIERNLKKAKTEQWSHKYRENSFLVPLQDTGSNLLVH
                     GPSNTGKTHYAAAHFENPLVCSHVDDLKRLGPDNDGIIFDDMSFKHWPVEAIIHLLDR
                     EFGRSINVRYGTVQIRAGIPKIFTHNTRNPFYEEATIEEEQKIAIERRFRRVNVLNKL
                     YA*"
ORIGIN
        1 tatagtaacc ccagggctcc ccggagccgg agccaccacg tgcttggctg gtggtggctg
       61 gctcctcttc tggctggctg gtggtggttg gctccaccgg ccggcctctt ctggtggtgg
      121 ctgctggccg gctcccatcc ctggctggcc ccgcgccccc tggggggggg cgcggccccg
      181 gccaggccag gctgtgaggc caaggcacgt gtttttccaa gaacgcacgt gttgtcatat
      241 gaagcggcaa cgtgaagaga cccaactcgt cccttatcaa cctgttacca aacgaatcaa
      301 aatggacgta caacgagcac cctatcaacg aggacctgga ttttaccgtg gaaaacctct
      361 tggcccgctt aaagcaccta aatacacaga tcgaatgtgg cgagttcgcc gaccaaggcg
      421 aaaaactcct aaagcaatgg ctcaatactg gcgaaagcga taccgaagac gagtcctcac
      481 tggacgagga gattatagtt attcagatag tcgatctact ggtgcgaatc ttggaggatg
      541 gcttggaagc aaagcgggtg aatacctcgg aggtatggcc caaaccgcag gaatggcctt
      601 aatatccggt ttgggtgatt atactgttaa caaaaacgtg ctactatctg gaaacatgcc
      661 ttcaattcaa aatatgacct ctggtgctgg aatcactatt tcctttcaag aatatcttgg
      721 tgacgttgtc actccactac aagtgggaaa ttcttcacct ttcgatgtta caacatatgt
      781 tattaatgct tcaaatcctc gaacattccc ttggttatca caaattgcac aaaattttga
      841 acaatatgaa atggaaggaa tgctctttgg ttttaggaca acttcagctg acttagttac
      901 taatggcaca aataacttac aacttggaac tgttattatg gctactcaat atgatgtagc
      961 tgatccaaac tttgcatcta aaggtgaaat gctcaattac caattttcaa catctgttaa
     1021 accctcacaa cctgcaatac acatgataga atgtgaccct agacaaacct cagtcaatct
     1081 cctttatact ggtaccgcgg ctgctggtac cgatcctcga ctttacaatc ttgggagatt
     1141 ttcaatcgct actcaaggtt ttcaaggttc tggtattgta attggtgaac tacacgttac
     1201 ctatcaagtc agacttctta aaccaaaatt atggactaca cttggagaaa atatcccttg
     1261 gagttcaggc atttatacta tgagttcagg atcaactaaa agttggcttg gtgtttctgg
     1321 tactattgct acacagacta ttaataattc taatcaaacg gtatcgatag ataacaatct
     1381 tgatattacc caggtacaat caaaagttat attccaacgt caacgattcc ctcaacgtat
     1441 atacatactt atagcatggt cagctcaatc tggagcaaca ggaggatttt ttaatacctc
     1501 tgatggtaat cccgtttttt ctgctggtgg tggtgcaaca ctcatatcac aacaaatcta
     1561 ccccctctct gctactaatg actctattac tggatctttt atgggaattg tagatattgc
     1621 ccctgatcag tcagcacctt actttttgat taaatcattc acatctacat tccaacccga
     1681 ttacccaaat ggtcaaatta ttgcgagatt aactcttgca cctacagacc caactggtaa
     1741 tggcaatctt aattgatcca gcaaccacat taccgcggct gcggacttat atattttttg
     1801 ggttaaaatg tcaaacaaag taataaacaa cagtttagtt caaatataat ttattaaaat
     1861 caacaatata cttaagcgta taatttattt aagacattta ctcgtcgaaa tcgacgctca
     1921 atagctatct tttgctcctc ttcaatagtg gcttcttcat agaacgggtt tcttgtattg
     1981 tgggtaaaga tctttgggat tccagctctg atttggactg ttccatatct gacattgatt
     2041 gatcttccga actccctatc tagcaaatga ataatggctt caactggcca gtgtttgaat
     2101 gacatatcat cgaagattat tccatcatta tcaggcccca acctctttaa atcgtcaaca
     2161 tgtgaacata ctaagggatt ttcgaaatgg gctgcagcat aatgcgtctt tcctgtattt
     2221 gagggtccat gtaccaatag atttgatccc gtgtcttgta gtggtacaag gaagctattc
     2281 tctctgtatt tgtgagacca ctgttcggtc ttggcttttt taaggtttct ttcaatggct
     2341 tccccgtgta gagccaagtc acgtggtctt ttctccttga tgatttgtat tccggctcca
     2401 acactatcag cggcaaaggc ttcagcgaag gcttgttgat cgttaccgcg gccgctggcc
     2461 ttaggttgtt tgggtctttc tccaaactca gtgaaatctc cgtccttact acagtactcg
     2521 aagttgttcc aaggtgtggc ttttttgcac atgggcaacc agttgattcc tggataattg
     2581 gttgtaactg ttgaccatct ctgcttggtc ttaaaaatga caaatccttg cacgtgtgga
     2641 gtcttttttg ttggtgtaag ttcctttcct tcaattctgt acttgcattc cgtgtcgggc
     2701 acgactggta acttttcggg atcataattg aaccacgtgt aaagccaaca gacttgtgcg
     2761 tttcctctct ccccacgtgc aggtggcttt cctgcgtatg gactaatgac cagttccgtt
     2821 tcatgttcaa taggatcctc aacctgttca gatgatgatg atgatgatga tgatgatttc
     2881 atatatgatt accgaaagag tagacgctga tgggtataat ccttatcggt accttacagt
     2941 cgagaactca atgggtatga tttataaccc cccctatata agtatagaat tcgactcgaa
     3001 ctcttttcag atttgtactt atactatatg agggctcccc atttgggaaa gttttcaaat
     3061 atttttttta gaaattttgg ctatttgttt ttttatactg taaaaacatt agaaaccgtt
     3121 ttttggctat ttgttttttt atactgtaaa aacattagaa accgtttttt ggctatttgt
     3181 tttttttaac ccaaatctta gccagagcgc cgagaaggtc ggtgctagca cggtcgtggg
     3241 ggctggggcc cccgacccag tgcgcgccta tgcccacacc ctacaacacc acacaagcga
     3301 atttaagcaa aattccccac tctacattaa gcaggctggt ggtggctgct ggccggcctc
     3361 ctggctggct cttcttggct ggctggtgcc ggctctgccc tgggta
//
LOCUS       CruV-246                3408 bp    DNA     circular     24-FEB-2020
DEFINITION  Crucivirus-246 isolate 120_144_SvL2_3298.
ACCESSION   CruV-246
KEYWORDS    .
SOURCE      Crucivirus-246
  ORGANISM  Crucivirus-246
            Unclassified.
REFERENCE   1  (bases 1 to 3408)
  AUTHORS   de la Higuera,I., Kasun,G.W., Torrance,E.L., Pratt,A.A.,
            Maluenda,A., Colombet,J., Bisseux,M., Ravet,V., Dayaram,A.,
            Stainton,D., Kraberger,S., Zawar-Reza,P., Goldstien,S., Briskie,J.,
            White,R., Taylor,H., Gomez,C., Ainley,D.G., Harding,J.S.,
            Fontenele,R.S., Schreck,J., Ribeiro,S.G., Oswald,S.A., Arnold,J.M.,
            Varsani,A., Enault,F. and Stedman,K.M.
  TITLE     Direct Submission
  JOURNAL   Submitted (24-FEB-2020) Biology, Portland State University, 1719 SW
            10th Ave, Portland, Oregon 97201, USA
COMMENT     Bankit Comment: ALT EMAIL:ide@pdx.edu
            Bankit Comment: TOTAL # OF SEQS:451
            Bankit Comment: GAP: est. length - alignment

            ##Assembly-Data-START##
            Assembly Method       :: MetaSPAdes v. 3.12; IDBA-UD v. 1.1.1;
                                     MEGAHIT v. 1.0.3
            Sequencing Technology :: 454; Illumina
            ##Assembly-Data-END##
FEATURES             Location/Qualifiers
     source          1..3408
                     /organism="Crucivirus-246"
                     /mol_type="genomic DNA"
                     /isolate="120_144_SvL2_3298"
                     /isolation_source="Water"
                     /country="Norway"
                     /collection_date=2012
     CDS             1..1383
                     /codon_start=1
                     /product="Putative Replication Associated Protein"
                     /translation="MKQNSYYKQKLAEYIEQEKKIGWVQPDKYETPAAKAKEAYLLHK
                     AENKDEMNFISKKPDGAKFRIDAKRIFFTYKTHIDMDEFIKWLDAKHPIETWIICHEK
                     SDKTNAYEHTHASIEFKTAVASTNCRHFDYGIDPADGNPIHPNFKAVTKWSGSIAYCI
                     KQKPEAIYKSNFDVVAFLKKNKRNTELSPEEWKELVTKIVSQKNAYEAILNCAKSLKD
                     VSAVNTIYGARVRGMDPLLIEEFKNFIPRPWQDDLVKILRSPQNNDDIYWIIDKAGGK
                     GKSTFLDWWELQPEGNSTVFMTDIGKPSDMNDYMKNWMDQDASRWPRVILINLPREFN
                     VFNEIYKVIEVLKDGRITCTKYKACPLKYKRPHVVIFANAEPKVTGMSLRKWKLLGFD
                     KIDGEERLVSVNTETLATKQKAKAKATKVTGGRNIMDYVLPPSTINFKDIDPFIESSE
                     SDNEAPLDEI*"
     CDS             complement(1742..3193)
                     /codon_start=1
                     /product="Putative CruV Capsid Protein"
                     /translation="MGRFNWTVCRCYLLPNKNKNIVYNYIMPALVKRTYRPRRITAPY
                     RRRRVSAYRTPLYRSPRPAPRRRTIGSRSYAQNTDAMKSVSSNAGANIGGAIGSLFGP
                     AGSALGSLLGHGAQSLVKMVTGFGDYNVNRNSLMPGALEPPMIVNQLTGKGIVVRHRE
                     YIADITSSIIFTTQQYAINAGLVGTFPWLSQIAESFEEYCIHGMLFEFKSMSSDAVLS
                     TAANSALGTVIMATQYNALNPGFVDKREMENYEFSNSAKPSESFIHPIECKGTETPVK
                     CLYVRIGSVPTNADQRLYDLGVFTIATQGMQSAVGTIGELWVTFEIEFFKPKLISGLG
                     LQVLTDHYRLVGVTNAFPLGSLDRAPEKGSQIGTEIFITGSGATQVFFPFEIVDGTYL
                     VEYSVQGTATAVTSPTFTPLPNLTFAPPLYQNDTNASQFIRTGTVTSVYWQSSLVTIS
                     GPQAGFTIGIGVLPTAVTAGDLWITQINSGISA*"
ORIGIN
        1 atgaaacaga acagttacta taaacagaaa cttgctgaat acattgaaca agaaaaaaaa
       61 atcgggtggg tgcaacctga caaatatgaa acacctgcag caaaagctaa agaagcatac
      121 cttcttcaca aagctgaaaa taaagacgaa atgaacttta ttagcaaaaa acctgacgga
      181 gcaaaattca gaatagacgc aaagcgaata ttcttcacat acaaaactca catagacatg
      241 gatgagttta tcaaatggct agatgcaaag catccaatag agacatggat aatctgtcac
      301 gaaaaaagtg acaaaacaaa tgcatatgaa catacacatg catcaataga attcaaaact
      361 gccgtcgcat caaccaactg cagacatttt gattatggaa tcgatccagc agatggaaat
      421 ccaatccatc ctaacttcaa agcagttacc aaatggagtg gatctatcgc ttactgcata
      481 aaacagaaac ctgaagcaat ctacaaatcg aacttcgatg tagttgcatt cttaaaaaag
      541 aataaaagaa acactgaact ttcacctgaa gagtggaaag aattagtcac caaaattgtt
      601 tctcaaaaaa acgcatacga agcgattcta aactgcgcca aatcgcttaa agacgtgagc
      661 gcagtgaata caatttatgg agccagagta cgtggaatgg atcctctatt aatagaggaa
      721 ttcaaaaact tcataccacg accatggcaa gatgatctag taaaaatact aagatcacct
      781 caaaacaacg atgacatcta ttggataata gataaagccg gtggaaaagg caaaagtacc
      841 ttcctagatt ggtgggaact ccaacctgaa ggtaacagca cagttttcat gacagatatc
      901 ggaaaaccat ccgatatgaa cgactacatg aaaaactgga tggatcaaga cgcatccaga
      961 tggccaagag taattctcat aaacttacca agagaattca atgtatttaa cgaaatatac
     1021 aaagttattg aagtcctaaa agacggacga ataacttgta ctaaatacaa agcctgtcct
     1081 ttgaaataca aaagaccaca cgttgttatt ttcgcaaacg cagagcctaa agtgacagga
     1141 atgtcactac gaaaatggaa acttctcggc ttcgataaaa tcgacggaga agaacgctta
     1201 gtctccgtta acactgagac tttagctacc aaacaaaagg ctaaagctaa agccactaaa
     1261 gtgaccgggg ggcgtaacat tatggactac gtcctgcccc cctctaccat caatttcaaa
     1321 gacattgatc ctttcataga aagtagtgaa agtgataatg aagcaccatt agatgaaatt
     1381 taattcttat atataaaaat taaattttat aaaccactat aatagaatcc tatagtcgct
     1441 gacgcgacag tcccgctgcc gcggggccgg ctcgctacgc tcgaacaaca tctacaagtt
     1501 ggttgcgagt cgcttcgctc ccgcaaacaa cttatagaca ttgtagaaag gacctgcgcg
     1561 gtccttaaac cttgcgccca cgctggggcg agttctccaa aaaagcgcta gcgcgctttt
     1621 tttacgaacc gggaattaaa tagcaatcat aatttatttt tatttagaaa tactatcatc
     1681 atagcatttt taattaaaac aataatcata ggtatcaaca taaagattaa atcaataatc
     1741 attatgcact tataccagaa ttaatttgcg taatccaaag atcaccagca gtaactgcag
     1801 ttggcaaaac accaatacca atagtaaatc cagcttgagg accacttata gtaacaagtg
     1861 aagactgcca atatacagaa gtaactgtac ctgtcctaat aaactgagac gcattagtat
     1921 cattctgata taatggcgga gcaaaagtca aattaggtaa aggcgtaaaa gtaggacttg
     1981 ttacagcagt agcagtacct tgaacagaat actctactaa ataagtccca tcaacaatct
     2041 caaatggaaa aaacacttgc gtcgcaccag aaccagtaat aaatatctca gtacctatct
     2101 gagaaccttt ctcaggtgct ctatcaagag aacccaatgg aaaagcatta gtaacaccta
     2161 ctaatctata atgatcagtc aatacttgta atcccaaacc agaaataagt ttcggcttaa
     2221 agaactcaat ctcaaaagtg acccaaagct cacctatagt tccaacagca gactgcatac
     2281 cttgagtcgc aattgtaaaa actccaagat catataaacg ttgatcagca tttgtaggaa
     2341 ccgaacctat acggacatac aaacacttaa caggagtctc agttccctta cattcaatag
     2401 gatgaatgaa agattcagat ggcttagcag aattagaaaa ctcataattc tccatctcac
     2461 gtttatcaac aaaacctgga ttaagagcat tatactgagt agccataata acagtaccta
     2521 aagcactatt agctgccgta gacaacacgg catcagaact catactctta aactcaaata
     2581 acattccatg tatacaatat tcctcaaaag actcagctat ctgagaaagc catggaaaag
     2641 tacctactaa tcccgcatta atagcatact gttgagtagt aaaaataata cttgatgtaa
     2701 tatcagctat atactcacga tgacgaacaa caattccctt accagttaat tgattaacaa
     2761 tcattggtgg ctcaagagca ccgggcatca aagaatttcg attaacatta taatcaccaa
     2821 aaccagtgac catcttaacc aaactttgag caccatgccc taaaagggaa cccaatgcac
     2881 ttcctgcagg tccaaataaa gagccaatag ctccaccaat attcgcaccc gcatttgatg
     2941 aaacagactt catagcatct gtattctgag cataacttcg tgaacctatc gtacgtctcc
     3001 taggagcagg cctaggagaa cgatacaatg gagtacgata agcagacact ctcctccgac
     3061 gataaggagc agtaattcgt cgcggacgat aagttcgttt tactaaagca ggcataatat
     3121 aattatacac tatattttta tttttatttg gtaataaata acatctgcaa acggtccaat
     3181 tgaaacggcc caattgaaat tatacggcaa tttagcaatt gataatacgg caattttgta
     3241 accaaaaaac aggataagca tcctactata cggccaaatt gctgaaaaaa cggtgaataa
     3301 acatatactc ttatagagaa atatttttat attttccaac cagtttccca gtcagtttgc
     3361 cacccatatg aaatttattt tctaaccata taagtatata cggtcgca
//
LOCUS       CruV-247                3413 bp    DNA     circular     24-FEB-2020
DEFINITION  Crucivirus-247 isolate 001518_virusbaton.
ACCESSION   CruV-247
KEYWORDS    .
SOURCE      Crucivirus-247
  ORGANISM  Crucivirus-247
            Unclassified.
REFERENCE   1  (bases 1 to 3413)
  AUTHORS   de la Higuera,I., Kasun,G.W., Torrance,E.L., Pratt,A.A.,
            Maluenda,A., Colombet,J., Bisseux,M., Ravet,V., Dayaram,A.,
            Stainton,D., Kraberger,S., Zawar-Reza,P., Goldstien,S., Briskie,J.,
            White,R., Taylor,H., Gomez,C., Ainley,D.G., Harding,J.S.,
            Fontenele,R.S., Schreck,J., Ribeiro,S.G., Oswald,S.A., Arnold,J.M.,
            Varsani,A., Enault,F. and Stedman,K.M.
  TITLE     Direct Submission
  JOURNAL   Submitted (24-FEB-2020) Biology, Portland State University, 1719 SW
            10th Ave, Portland, Oregon 97201, USA
COMMENT     Bankit Comment: ALT EMAIL:ide@pdx.edu
            Bankit Comment: TOTAL # OF SEQS:451
            Bankit Comment: GAP: est. length - alignment

            ##Assembly-Data-START##
            Assembly Method       :: MetaSPAdes v. 3.12; IDBA-UD v. 1.1.1;
                                     MEGAHIT v. 1.0.3
            Sequencing Technology :: 454; Illumina
            ##Assembly-Data-END##
FEATURES             Location/Qualifiers
     source          1..3413
                     /organism="Crucivirus-247"
                     /mol_type="genomic DNA"
                     /isolate="001518_virusbaton"
                     /isolation_source="Water"
                     /country="France"
                     /collection_date=2013
     misc_feature    join(3399..3413,1..22)
                     /note="stem-loop"
     misc_feature    1..9
                     /note="nonanucleotide"
     CDS             377..1915
                     /codon_start=1
                     /product="Putative CruV Capsid Protein"
                     /translation="MPPTKRFKGNELVLYQPPEPKIEPEYQGEVHEPDRGIFPGLRRP
                     SSRPYRMPRQSYKRKRYTRARSSYRGYSYGRRRPYNRFRRPYYGYRGRGAYYLQGSGA
                     MSGGIPGIGKFALNGRGGLSNTVSGLGAYDMSSIMHNTIIKPDVPEVRNAAFVEGATI
                     IRHREYLGPIVTSATAGAFKIESFPLNPAQDMTFPWLSQIAQNYEEYKPNGLLFEFRS
                     TASDAIASSTNLALGQVMMATQYDPTDPEFDSDIDLLNYSWAQSGKVSECVQHFVECD
                     PKQSPMSHLYTREGSEASSSDLRFSDFGRFSIATQGLQGTSVQIGQLWVSYEFLLYKP
                     KVGDSQSTSGGIFMYFQQDGSVSNNQILGVIAAGVRFPDNNIDMKLSYDDNGYLRMIF
                     PARNVQCVYDVSFTVIGDGDGVTFINAAPVTTTQGTGIKYPKIYNEHTQGFVESPTTG
                     VANQSRWLVRYCVHVDAADNTNQNWILMRTNLATIAANSDTTFLVQQMAYIDPETYGY
                     TTGT*"
     CDS             complement(1972..2994)
                     /codon_start=1
                     /product="Putative Replication Associated Protein"
                     /translation="MVQFPNKYFKIVNYLRTFEMPRRARQRRPVDLTDDGKRHRNYCF
                     TINNPKEEDCNNLRDLKLESKYLLMGNENNWKYGILEFATSRPNSTHHFQCFVILKDG
                     KTKQAFKRIVPRAHFQPCNGSPESNIVYCTKDGEWIEYGPRPMSQEQKGEAGGEAEKQ
                     RWTHMRDLAKVRDWETFETEYPRELTLYEGSFQRIAIQHGPTLDLEDNSVVGIWIYGP
                     TGTGKTYHVMHNMVPDRSMVYSKNISKWWDGYKQEQHSLVLIDDLSKFHVKIADDLKV
                     WVQEYEFNAEFKGGSFRIRPKQIVVTSNYRIADIWEDAITRETLWRRFKCFKKMTKDG
                     HLETDD*"
ORIGIN
        1 cagtattacc tccaccccta gtgtgtagtg tgtagttttt gttttctcta taattctcta
       61 aacctaatat ttctgcgccg cggaaaatcg gattattgta gcgtacggca gtgagcgctc
      121 aatatgagga tgcagcggca ttctatatac ttgtttcttt acccctgaga ctacatctcc
      181 gcgagcgaag cgagaggaaa aaatttttag cctttctatt ttggcctaaa aagcttaatt
      241 taaccctacc ctagtgtgca gtgtgtaatc actgaacgtc aattcactgc actggggaat
      301 tcgctaatcg taatcatttc tttccaccac ggctaaacga cacggaaccg acataactga
      361 cgcagatttt tttgatttgc cccctactaa gagatttaaa ggcaacgaat tagtcttata
      421 ccaaccccca gaaccgaaaa tagagcccga atatcaagga gaagttcacg aacctgaccg
      481 tggaatattt cctggactta gaagaccttc ttcacgacct tatcgtatgc ctaggcaatc
      541 ttacaagcgt aagagatata cgcgcgcccg gtcctcatat agaggatatt catatggaag
      601 acgaagacct tataatcgct ttcggcggcc ttattatggc tatcgtggtc gtggtgctta
      661 ttatcttcag ggttctggag ctatgtccgg aggtattcct ggtattggaa agtttgcact
      721 taacggcaga ggtggactct cgaataccgt ctctggtcta ggcgcatacg atatgagcag
      781 cattatgcat aatacaatta ttaagccgga tgttccggaa gtgcgtaacg cagcttttgt
      841 ggaaggagcc actattattc gtcatcggga atatttagga ccaatagtaa cgtcggctac
      901 tgctggcgct tttaagattg aatcctttcc tcttaatcct gcgcaagata tgacttttcc
      961 ttggttatcg caaatcgctc aaaattatga agagtataag cctaatggtt tattgtttga
     1021 atttcgctct actgcttctg atgctatagc ttcatctact aatttagcat tgggacaagt
     1081 aatgatggcc actcaatatg atcctactga tccagagttt gatagtgata tagatctatt
     1141 aaactattca tgggcgcaaa gcggtaaggt ttctgaatgt gttcaacatt ttgttgaatg
     1201 cgatcccaag cagagtccaa tgtcgcattt gtatactcgt gaagggtctg aagcgtcttc
     1261 ttcagatctc cgattttccg attttggtcg tttttcaata gctactcaag gtctgcaagg
     1321 aaccagtgta caaattggcc agttgtgggt gtcctatgag tttttattgt ataaacctaa
     1381 ggttggtgat tctcaatcaa cttctggcgg tatctttatg tactttcaac aagacggtag
     1441 cgttagtaat aaccagattc ttggtgttat tgctgctggt gtcaggtttc ctgacaataa
     1501 cattgatatg aagttaagct atgatgacaa tgggtatttg cgcatgatct ttcctgctcg
     1561 taatgtccaa tgcgtttatg acgtttcctt tactgttatt ggagacggcg atggtgttac
     1621 ttttattaat gctgctcctg tgacgactac gcaaggtact ggtattaagt atcctaagat
     1681 ttataatgag cacactcaag gctttgtaga aagcccgacg actggtgtcg ctaatcaatc
     1741 gcgttggttg gttcgttatt gtgttcacgt ggatgctgcc gataacacta atcagaattg
     1801 gattttaatg aggaccaatt tagctactat tgcggccaat tccgatacta catttctagt
     1861 tcagcagatg gcttatattg atcctgagac ttatggatac actacgggta cttaaacatt
     1921 tagtaataaa aatgcaactt aaaaaaaatt gtctttattt tttaaaaaaa attagtcatc
     1981 agtttcaagg tgaccatctt tagtcatctt tttaaaacac ttgaatcgcc gccagagagt
     2041 ctctcgggta atcgcgtctt cccagatatc cgcgatcctg tagttagaag taactactat
     2101 ctgtttcggt ctgatgcgaa aagaaccgcc tttaaattcg gcgttgaatt cgtactcctg
     2161 aacccagact ttaagatcgt cggcaatctt aacatgaaac ttactaagat catcgattaa
     2221 tactaaagag tgttgctctt gcttatatcc atcccaccac ttcgagatgt ttttcgagta
     2281 aaccatgctg cgatccggaa ccatgttatg catgacatga taagtcttcc cagtcccagt
     2341 cggcccgtaa atccaaatcc cgacaactga attatcttct aagtccaaag tcggcccgtg
     2401 ctgaatcgca atgcgctgga acgatccttc gtagagagtc aattcgcgag gatattcggt
     2461 ctcgaaagtc tcccagtctc gcactttcgc taagtctcgc atatgagtcc atcgctgttt
     2521 ttcggcttct ccccccgctt ctcccttctg ctcttgagac attggtcgcg gtccgtactc
     2581 gatccattcg ccgtctttcg tgcagtagac gatgttactt tcgggggatc cattgcaggg
     2641 ttggaaatgg gctctcggca cgatcctttt gaaagcttgc ttagtctttc cgtctttaag
     2701 gataacaaag cactggaaat gatgcgtact attcggtcga gaggtcgcga actccaggat
     2761 cccgtacttc cagttgtttt cgttccccat cagaaggtac ttcgattcca gcttgaggtc
     2821 gcgcagattg ttgcagtcct cctccttcgg gttgttgatc gtgaagcagt aatttcggtg
     2881 gcgctttccg tcatcagtca gatccactgg tcttcgttgg cgagctcggc gaggcatttc
     2941 gaaagtacga aggtagttca cgattttaaa atatttattt ggaaactgaa ccatatccca
     3001 ctaccttctg tgtcaaaagt gccctctaat cattggttcg ttaatacatg gtttcaaaat
     3061 cctgagattt tgttttttaa ccaatcaaaa actgacacgt cattgactat aaaaggctat
     3121 tctaaataag gcagtcgacg tcatcattct aatttacatc aagtgatgtc ttgtgaaaga
     3181 cgtcataagg cgtttctttg gggatgtcaa acgacatttc tcgggggcgg cttaaagggg
     3241 gccacttaaa aggggaatgt cgcgacggac tgatactaat gaaataaacc cacgcaagcg
     3301 aagcgcggcg tggccattac acggagtgtt gtcgagcaag cttgcgagcc gccgaaggct
     3361 aagtacatgc ctattttttt gatttttttt ttttgcacac taggggtgga gta
//
LOCUS       CruV-248                3413 bp    DNA     circular     24-FEB-2020
DEFINITION  Crucivirus-248 isolate 120_189_Lv1_3299.
ACCESSION   CruV-248
KEYWORDS    .
SOURCE      Crucivirus-248
  ORGANISM  Crucivirus-248
            Unclassified.
REFERENCE   1  (bases 1 to 3413)
  AUTHORS   de la Higuera,I., Kasun,G.W., Torrance,E.L., Pratt,A.A.,
            Maluenda,A., Colombet,J., Bisseux,M., Ravet,V., Dayaram,A.,
            Stainton,D., Kraberger,S., Zawar-Reza,P., Goldstien,S., Briskie,J.,
            White,R., Taylor,H., Gomez,C., Ainley,D.G., Harding,J.S.,
            Fontenele,R.S., Schreck,J., Ribeiro,S.G., Oswald,S.A., Arnold,J.M.,
            Varsani,A., Enault,F. and Stedman,K.M.
  TITLE     Direct Submission
  JOURNAL   Submitted (24-FEB-2020) Biology, Portland State University, 1719 SW
            10th Ave, Portland, Oregon 97201, USA
COMMENT     Bankit Comment: ALT EMAIL:ide@pdx.edu
            Bankit Comment: TOTAL # OF SEQS:451
            Bankit Comment: GAP: est. length - alignment

            ##Assembly-Data-START##
            Assembly Method       :: MetaSPAdes v. 3.12; IDBA-UD v. 1.1.1;
                                     MEGAHIT v. 1.0.3
            Sequencing Technology :: 454; Illumina
            ##Assembly-Data-END##
FEATURES             Location/Qualifiers
     source          1..3413
                     /organism="Crucivirus-248"
                     /mol_type="genomic DNA"
                     /isolate="120_189_Lv1_3299"
                     /isolation_source="Water"
                     /country="Norway"
                     /collection_date=2010
     CDS             1..888
                     /codon_start=1
                     /product="Putative Replication Associated Protein"
                     /translation="MKNKKLALSGSTIDGNTNPIVETPLLKKKKEKRCNAVKNWCFTY
                     NNYCMENIQSLLALCKEFTEKYFFAEEEGENKTPHLQGFISLLKKGRPCEIFKAILNE
                     EGKHPIHWESMKGSMPENIKYCSKEGGKFYKSSNVRLIPKLNIITNLHPWQIFVESLI
                     KEPCTDDRVIHWFYDLVGNKGKTAMTKYLCYHYGAMLVEGKKKDILFGIQNAEDTDIF
                     IFDFSRSIENYVSYDAIESVKNGIFFSTKYESKMILRNSPHIIIFANFLPEEEKLSKD
                     RWRIYDLSKFNNGAEFLNP*"
     CDS             complement(1352..2743)
                     /codon_start=1
                     /product="Putative CruV Capsid Protein"
                     /translation="MPARKRASSKKKRVTVRAPRSTVKRYAPKASGSGAYTLDNPGPF
                     GKAGQALGGIVGSHLGGRSLGSALSTGGRYLGHKIGKFFGSGAYGYNSSTMSPADQLP
                     KFSNDHGDDSICITHREYLGDLITSNTIGGFKLTTFPINPASTQSFPWLCNIAQPNFQ
                     QYRFEGLIFSFQSFSADALNSTNTALGSVFAAVNYDYTDENFTNRNQIENSDWSMSCK
                     PSESMLIPVECAPRQTGMGGLLYVVNDNNIPANADPKMYFLGKLSVGTTGFQASNINI
                     GSLYVTYKIRLYKKIMTPPLSNALIATLVREGSTAGANVCGTALYGGLIINNNCDSIG
                     ITFPTSNKIRMNKNRLIQGQRYLFVHQWSGVSTAGIKPPDFSLNISSGCIGSPNTMNN
                     YQTSYNFAPDVPATGATLIAVQTVEIIDDSIDSDIEYSTTGFVPPGSCLYNIEIYQIC
                     GIALNRIGFYDGN*"
     CDS             2955..3341
                     /codon_start=1
                     /product="putative ORF"
                     /translation="MDKLELQGKIDYCNIKMIDVHDVIDKIEDKIMICATPESFSLLS
                     SKLRDMILDLRLRLMHFEIMFDSIIDEVKEGDMITYCTSSETESPTACGDIEGYNFTV
                     DGAGSEYRDTTDDYVRKNKKSKPFKN*"
ORIGIN
        1 atgaaaaata aaaagttggc tctgtctggc tctactattg atggtaatac taacccaata
       61 gtagagaccc cccttcttaa aaagaagaag gaaaaaagat gtaatgccgt aaagaactgg
      121 tgttttacat acaataatta ttgtatggaa aacattcagt ctctcttggc tctctgtaaa
      181 gagtttacag agaaatattt tttcgccgaa gaagaaggag aaaataaaac gccacattta
      241 caaggtttca tttctttatt aaagaaagga agaccctgcg aaatttttaa agcaatactt
      301 aatgaagaag ggaagcatcc aattcattgg gagtcaatga aaggaagcat gcccgaaaac
      361 attaagtatt gctctaaaga aggcgggaag ttttacaagt cttctaacgt tagattaata
      421 ccaaagttga atattattac taatcttcac ccttggcaaa tcttcgtaga aagtcttata
      481 aaagaaccct gcacggatga ccgagtcatc cattggtttt atgaccttgt cggtaacaag
      541 ggcaaaacag ctatgactaa atatttatgt taccattacg gcgctatgct tgtagaaggt
      601 aagaaaaaag acattttgtt tggtatccaa aatgccgaag acaccgatat ttttatattt
      661 gatttctctc gatctataga aaactacgtt tcctatgatg cgatagaatc agttaaaaat
      721 ggtatctttt tttcaactaa atatgagtca aagatgattt tgcgaaattc accacatata
      781 attattttcg ctaattttct tcctgaagaa gaaaagctca gtaaagatag atggcgaatt
      841 tatgatttaa gtaaatttaa taacggtgcc gaattcttaa acccttaagt ttacttaaac
      901 caaaaaaaca cgttttcaac gtgttttttt aggtttaaga aaacttaaga gtttaagaag
      961 tccttgcgcg gaccgctttc cgtgaaaacc caaaagggtt ttccgctccc agcaccctta
     1021 ttaaattttt taagggcaag tattcttaat cccttaagtt cccttaaaaa ttattacatg
     1081 gattgatatt tgcaaatcta aatcctagta gaatttttaa gagaacttaa gagattaaga
     1141 agtcctgagc ggaccgcttg atgccattga aaaattgtat ttttacttaa atcgattgat
     1201 tggttttttt ttgagggttc ggggtggaaa agggcgggaa ggctgaagcc ttcccttttt
     1261 accattatta tttatatctt tatttataat ataagataca aaatattaaa actgggcgaa
     1321 caacgaagtt gtgagcccct aattaaatta tttaattacc gtcataaaaa ccaatacgat
     1381 tgagagcaat tccacatatt tgatatatct cgatattgta caaacatgat ccaggtggaa
     1441 caaatccagt tgtactgtat tcgatatcag aatcgatact atcgtctatg atttccactg
     1501 tctgaacggc tattaacgtt gcacctgtcg ctggtacatc gggtgcaaag ttatacgacg
     1561 tttgataatt gttcatcgtg tttggcgaac ctatgcatcc tgagctaata tttagcgaaa
     1621 aatcgggcgg tttgatgcca gctgtactga caccactcca ctgatggaca aacaaatacc
     1681 gttgaccttg gatcaagcga tttttattca tcctgatttt attggatgta gggaacgtaa
     1741 tgcctataga gtcacaatta ttattaataa ttagtccccc gtatagtgcc gtcccgcaca
     1801 cgttcgcccc cgctgtactg ccttctctaa caagagtagc aattaatgca ttgctaagag
     1861 gtggtgtcat tatcttttta tacaaacgta ttttatacgt tacgtataat gaaccaatgt
     1921 ttatatttga agcttgaaat cccgttgtgc ccacagacaa tttacccaaa aaatacattt
     1981 tcgggtcggc attagctgga atattattgt cgttgacgac atataacaat ccacccatgc
     2041 ctgtttgtct tggtgcgcat tcaacgggaa tcaacattga ttcagacggt ttacaactca
     2101 tagaccagtc tgagttctca atctggttac gatttgtaaa gttttcgtct gtgtaatcat
     2161 agttgactgc ggcaaatacg ctaccgagtg cggtattggt gctgttgagg gcatcagcac
     2221 taaaactttg gaaagagaat atcaacccct cgaacctata ctgttggaag ttaggttgag
     2281 caatgttaca cagccatgga aatgactgtg tagaagcggg attaataggg aaagtagtaa
     2341 gtttaaatcc tcctatagta tttgatgtga taagatcacc caaatactca cgatgagtta
     2401 tacatatact gtcgtctcca tgatcgtttg aaaatttagg tagttggtcc gctggtgaca
     2461 tcgtagacga gttgtaacca tacgcaccac tgccaaaaaa ttttcctatt ttgtgtccga
     2521 gatatcgtcc acctgttgat aatgctgacc ctaacgaccg tccgcctaaa tgcgaaccga
     2581 cgatcccgcc taatgcttgt cctgctttac caaacggacc tgggttatca agtgtataag
     2641 caccactgcc cgaggcttta ggtgcgtaac gcttgaccgt tgagcggggt gctctaactg
     2701 taacccgctt ttttttactt gatgctcgtt ttcttgctgg cattttctat ttataattat
     2761 atatagaaaa taaattttct atatataatt ataaattatg tttaattctt gttgtgattg
     2821 caactgcgaa aaactagaaa atgtcaacaa cttaattaat ttgtataaat ccaaattaaa
     2881 taagcttgaa aacgcgcgca ataaaattat tttagctctg caaaaataat taatttatca
     2941 tactataata tagtatggat aaattggaat tgcaaggaaa aatagattat tgtaatatca
     3001 aaatgataga tgttcacgat gtaattgata aaatagaaga taaaataatg atttgtgcta
     3061 cgcccgaatc attttctctt ctatcgagta aattacgtga catgattctt gacctacggc
     3121 taagactcat gcattttgaa ataatgttcg attctattat tgatgaggta aaagaaggtg
     3181 atatgatcac ttattgtaca tcatcggaaa cagaatcgcc cacagcttgc ggggatattg
     3241 aagggtataa ttttactgtt gatggagcag gtagcgagta ccgtgacacc actgacgatt
     3301 acgtcagaaa aaacaagaaa agtaaaccct tcaaaaatta aattatttaa tgcgttttaa
     3361 acgcactaaa tatttgaatt tcagagcaaa atttttatgt tttatatagt tat
//
LOCUS       CruV-249                3419 bp    DNA     circular     24-FEB-2020
DEFINITION  Crucivirus-249 isolate 120_142_SvL2_3298.
ACCESSION   CruV-249
KEYWORDS    .
SOURCE      Crucivirus-249
  ORGANISM  Crucivirus-249
            Unclassified.
REFERENCE   1  (bases 1 to 3419)
  AUTHORS   de la Higuera,I., Kasun,G.W., Torrance,E.L., Pratt,A.A.,
            Maluenda,A., Colombet,J., Bisseux,M., Ravet,V., Dayaram,A.,
            Stainton,D., Kraberger,S., Zawar-Reza,P., Goldstien,S., Briskie,J.,
            White,R., Taylor,H., Gomez,C., Ainley,D.G., Harding,J.S.,
            Fontenele,R.S., Schreck,J., Ribeiro,S.G., Oswald,S.A., Arnold,J.M.,
            Varsani,A., Enault,F. and Stedman,K.M.
  TITLE     Direct Submission
  JOURNAL   Submitted (24-FEB-2020) Biology, Portland State University, 1719 SW
            10th Ave, Portland, Oregon 97201, USA
COMMENT     Bankit Comment: ALT EMAIL:ide@pdx.edu
            Bankit Comment: TOTAL # OF SEQS:451
            Bankit Comment: GAP: est. length - alignment

            ##Assembly-Data-START##
            Assembly Method       :: MetaSPAdes v. 3.12; IDBA-UD v. 1.1.1;
                                     MEGAHIT v. 1.0.3
            Sequencing Technology :: 454; Illumina
            ##Assembly-Data-END##
FEATURES             Location/Qualifiers
     source          1..3419
                     /organism="Crucivirus-249"
                     /mol_type="genomic DNA"
                     /isolate="120_142_SvL2_3298"
                     /isolation_source="Water"
                     /country="Norway"
                     /collection_date=2012
     misc_feature    join(3409..3419,1..19)
                     /note="stem-loop"
     misc_feature    1..9
                     /note="nonanucleotide"
     CDS             219..1499
                     /codon_start=1
                     /product="Putative Replication Associated Protein"
                     /translation="MREIFYYRILYSTMTKTVKTVSGKKVRVKKVLSDAKLAELKLKE
                     DEQPEKVMNATTGEGAVCKKTFESRRFVFTHHCKPKEHDFNVLNLFGCDKKVDENFSF
                     KDEKIKLGNFYFTFLAGQQEISATNKAAGIDKRHFQGYFELDRKVTWSSVKSSLSKFY
                     NIQPWFAHAKGSLDDCLNYVSKRDTSVEDTFVRYGDPMDERENSDIWNDFKLDIIAGK
                     YPKLVDFQRAVPPKILAQFSKGLQVVYETHRPKYVYSLEAEYGSCLPFQTSVIALANA
                     PVSKRHVIWICGDGDLGKSDLCKHLASQNGFLLLGEGKNTDLFDTWAGDHVIFDFPKD
                     YPDKEICYSAIERLKNGHVYSPKYVGVSKLYPHPHVICLANQLPRWGAYTSNRWDFVY
                     RIDSTSKELIHIPDPGKNPPLAIAPCFLPPKSTI*"
     CDS             1597..3009
                     /codon_start=1
                     /product="Putative CruV Capsid Protein"
                     /translation="MPNYAATKHVRKAMRGVRREFKSVAKKGLKKAGRALGNYVRSGD
                     LTRDAGLVGGSNAEAAVRAFTGHGDYKTVRGRGFKEYGTMGSHKRTGRHGHDAHVNME
                     KGEMVISHSEYLGELVTGTAAGFVSQSYGINPGNFGTFPWLSTVANNFQDYEFEKLIF
                     EYKPLTSEAMSSSSAQVVSMGSVVMATQYDSVQGPYPSKYLILKVDFVFNNYNFIDKA
                     QMENSDFSTSCKPSEHAQMAIECAPRYNPLGVLYVSPNISAATSGQPNSDIRFQNLAL
                     FQIASVGVPASTATDLGEIWVHYKIKLMKPQLGGSLTSLISGHYINPLANGAATGGAP
                     FGVVTATNQPQVATGTLLPLAFTSNSFSFPLAVTEGNYLCSYVVEGNAATIAGVGVAC
                     AAGAILQVWSNATTPDAQLVAQGPQNAATGIVSLSVNFLVQVNAPGSALCTVTLTSAS
                     PPTAGRWDLFVTPINSNIIS*"
ORIGIN
        1 tagtattacc cgaccctggt tgcgtagcag cgggtcgcga gggacgagca ggccgacgaa
       61 ggaggccgtt ttgaaaattt taggctctac atttacattg ttttaaaact tgtttttaaa
      121 atttagattg cttgataaaa ttttcaaagt ttttttcatt tcctttaggc gtttttgaac
      181 atcctggttg cgaggatttt tgaaaagttg atttccagat gagagaaatc ttctactata
      241 gaatattata tagtacgatg acaaaaacag taaaaacggt ttctggtaag aaagttaggg
      301 tgaagaaggt tctttctgat gcgaagttag ctgagctgaa attgaaggag gatgaacagc
      361 ctgaaaaagt tatgaatgct actactggag agggagcagt ttgtaaaaag acttttgaat
      421 ctaggcgttt tgtttttacg catcattgta aaccgaagga acatgatttc aatgtgctca
      481 atctttttgg atgtgataaa aaagttgatg aaaatttctc ttttaaggac gagaaaatta
      541 agttgggaaa cttttatttt acatttttgg ccggtcaaca ggaaatatct gctactaata
      601 aggcagccgg gattgataaa agacattttc agggatattt tgaactcgac cgtaaggtga
      661 cttggagttc agttaaatcg tctctgtcca agttttataa tattcaacct tggtttgctc
      721 atgcaaaagg tagtttggac gattgtttaa attatgtttc aaagagagat acctctgttg
      781 aagatacatt tgttcgttat ggggatccta tggacgaacg cgaaaattct gacatttgga
      841 atgatttcaa gcttgacatc atcgccggta aatatcctaa gcttgtagat tttcaacgtg
      901 cagttccacc taaaatctta gctcagtttt caaaaggtct tcaggtcgtt tatgaaactc
      961 atcggccgaa atatgtttat tctttggagg ctgaatatgg atcttgtctt ccatttcaaa
     1021 ccagtgttat tgctttagca aacgcccctg tttctaagcg tcacgttatc tggatttgtg
     1081 gtgatggaga cttaggtaaa tctgatcttt gcaagcatct tgcaagtcaa aatggatttt
     1141 tattacttgg tgaagggaaa aatactgatt tatttgatac ttgggctggt gatcatgtta
     1201 tatttgattt tccgaaagat tatcctgata aggaaatctg ttattcggca atcgaaagat
     1261 tgaaaaatgg acatgtttat tctcccaagt atgtaggggt atctaaatta taccctcacc
     1321 cacatgttat ttgtttggca aatcaacttc ctaggtgggg cgcatatacg tcaaatcgct
     1381 gggattttgt atatcgtatt gattcaacaa gtaaagagtt gattcatatt ccagatccag
     1441 gtaaaaatcc accattggcg atagcaccat gttttttgcc accgaaatct acaatttagt
     1501 aattaaaagt gtttaggagt tttttgtaaa taaaaagtgc gtttagaagt tgagagtaat
     1561 tttcttagat aatatataga tcatctatta tctaaaatgc ccaattacgc tgcaacaaaa
     1621 cacgttagaa aagccatgcg tggcgttaga agagaattca agtccgtcgc taagaaaggc
     1681 cttaaaaaag ccgggagagc tttggggaat tatgttcgga gcggtgatct cactcgcgac
     1741 gccggccttg tcggaggctc aaatgccgag gctgctgtgc gagcctttac tgggcatgga
     1801 gactacaaga ctgttcgagg ccgtgggttt aaggaatatg ggacgatggg ttcccataaa
     1861 cgtactggaa gacatgggca tgatgctcat gttaatatgg agaagggtga aatggttatt
     1921 tcacattctg agtaccttgg tgaacttgtc actggtacgg ctgctggctt tgtcagtcag
     1981 tcatatggaa ttaatccagg caattttggt acatttcctt ggttatctac ggtcgcaaat
     2041 aattttcaag attacgagtt tgaaaagctt attttcgagt acaagccgct tacttctgag
     2101 gctatgtcct caagttcggc tcaggtggtt tctatgggct cagttgttat ggcaacccag
     2161 tatgattcag tacagggacc atatccaagt aagtatttaa ttttaaaagt tgatttcgtt
     2221 tttaataatt ataattttat agacaaagcg cagatggaaa attctgattt ttccacgagt
     2281 tgtaaaccat cggaacatgc tcaaatggca attgaatgcg ctcctcgcta caatcctttg
     2341 ggcgtcctgt atgtttcgcc aaatatttct gctgcgacgt ctggtcaacc gaattcggac
     2401 attcgttttc agaatctcgc gttgtttcag atagcgtcgg ttggtgttcc tgcttcgaca
     2461 gcaactgacc ttggagaaat ttgggttcat tataaaatta agctaatgaa acctcagttg
     2521 ggaggttctt tgacttcttt gatttctgga cattacataa atccattagc taatggagct
     2581 gccacgggtg gtgctccttt tggagtggtt actgcgacaa atcagcctca agtagcaact
     2641 ggtactctgt tgcctttagc tttcacttcg aattcattta gttttccttt ggctgtgact
     2701 gaaggaaatt atctgtgttc ttatgttgtg gaaggaaatg cagcaactat agctggtgtt
     2761 ggcgtcgctt gtgctgctgg tgcaatttta caagtttggt caaacgcgac aactccagat
     2821 gctcaattgg tggctcaagg tccacaaaat gcagctaccg gtattgtttc attatcggtc
     2881 aattttctgg tccaggtcaa tgctcctggt tcagctcttt gtactgtgac tctgacttca
     2941 gcaagtccac caacagctgg aagatgggat cttttcgtga ctcccatcaa ttctaatatt
     3001 attagttaat gtcttgttaa gtttaattaa tttaagtttt ttaaaatctt tctgaattat
     3061 ttagttttat aatctaataa aaaaatgcaa ttaaaaacct gccaatgcaa tccaagcaca
     3121 gattaaaatt atttatcacc tggttaataa ttttgagatg ctttagtttt tttattagaa
     3181 cattaaagtt gtgccaactg cgaagcgaaa gcgagctatt tcaaatgatg ttaatcatta
     3241 ttattggtgc gtagcccaat cgaacacaaa tctataaaaa gaagcgccgt ggcgtgagcc
     3301 atggcaagac aaaagcgaag cggccatttt tacattagtt gatttgctat tttcttttca
     3361 aataaattgt agaatttttt ggattaaaat cttccgtagg aagattttcc agggtcgga
//
LOCUS       CruV-250                3420 bp    DNA     circular     24-FEB-2020
DEFINITION  Crucivirus-250 isolate H7_15845.
ACCESSION   CruV-250
KEYWORDS    .
SOURCE      Crucivirus-250
  ORGANISM  Crucivirus-250
            Unclassified.
REFERENCE   1  (bases 1 to 3420)
  AUTHORS   de la Higuera,I., Kasun,G.W., Torrance,E.L., Pratt,A.A.,
            Maluenda,A., Colombet,J., Bisseux,M., Ravet,V., Dayaram,A.,
            Stainton,D., Kraberger,S., Zawar-Reza,P., Goldstien,S., Briskie,J.,
            White,R., Taylor,H., Gomez,C., Ainley,D.G., Harding,J.S.,
            Fontenele,R.S., Schreck,J., Ribeiro,S.G., Oswald,S.A., Arnold,J.M.,
            Varsani,A., Enault,F. and Stedman,K.M.
  TITLE     Direct Submission
  JOURNAL   Submitted (24-FEB-2020) Biology, Portland State University, 1719 SW
            10th Ave, Portland, Oregon 97201, USA
COMMENT     Bankit Comment: ALT EMAIL:ide@pdx.edu
            Bankit Comment: TOTAL # OF SEQS:451
            Bankit Comment: GAP: est. length - alignment

            ##Assembly-Data-START##
            Assembly Method       :: MetaSPAdes v. 3.12; IDBA-UD v. 1.1.1;
                                     MEGAHIT v. 1.0.3
            Sequencing Technology :: 454; Illumina
            ##Assembly-Data-END##
FEATURES             Location/Qualifiers
     source          1..3420
                     /organism="Crucivirus-250"
                     /mol_type="genomic DNA"
                     /isolate="H7_15845"
                     /isolation_source="Water"
                     /country="New Zealand"
                     /collection_date=2012
     misc_feature    join(3410..3420,1..18)
                     /note="stem-loop"
     misc_feature    1..9
                     /note="nonanucleotide"
     CDS             86..703
                     /codon_start=1
                     /product="putative ORF"
                     /translation="MKKLAGKEFLFFRFSLEPEPPPGKRRRLEEKMQSAAKPTYANFD
                     KALWSEYKELKDSIANIKNPDRGAQNAERYLAAVAKLKAMNANPYEYKMLLKPVKYQI
                     AHLLKHIPMEHFLKEQGWGNWVALKHFMVGKRAHHKGRRARFGISAKRWKGYRGGSHG
                     PGLRRQWKSQQHRKYYGRRGKSYSAPYVVGRYRRPWRRFPRYSMY*"
     CDS             complement(167..598)
                     /codon_start=1
                     /product="putative ORF"
                     /translation="MLRLPLSTQPRSMTSSAVSFPSLRGDPEACSSSFVMSSLSNHKM
                     FKGNPISPSLFLQKVFHRDVLQKMGNLVLNRLQKHLVLVRIGIHGFELRDRGKVTLGI
                     LSSAVRVLNIGYRVLEFLVLTPKGFVEVGVGGLSGRLHLLL*"
     CDS             717..2051
                     /codon_start=1
                     /product="Putative CruV Capsid Protein"
                     /translation="MAAPSRKRSRWLGPALSAGFGAAATSSGYGAFAGAASALGGAIG
                     QKFFDITGYGDYNVNKNSLLSGTVPSVGNSSTHPDGLTISHREYIGDVITSATAKTFA
                     ISDFDLNVNNPQTWEWLTQIACNYEEWVPEGILFYFKSTSGEAFTSNDASLGTVIMAT
                     NYNPYNKPFETKAEMESYEFCTNGPPTNDLIHMIECDPHEGSISTYYVSSVNSSANVG
                     LMDKRFNTLGTFYIATTGFQGTNVNIGELWVTYQVTLLKPKLYESLGLASDSIRAVLS
                     GTLEQDTSLYGLWPLTNASHDAVIQQRTIPYTIRTPSDSLWYEYASGNVTLHWPVYAY
                     PTYYEVSVRVSKSVNFTAPGFSLSQSGGTFGATYAPVLITGEGQTPETGTTSIRSTCY
                     NAVIKVPGGFICHDPSGTPSTRFSTSTALVVHLQWQIRQIPNLSLSSGYIDP*"
     CDS             complement(2048..3136)
                     /codon_start=1
                     /product="Putative Replication Associated Protein"
                     /translation="MFLMQRSRGWCFTLNNWTHDEVLKIKENLAECRYWIMGREVAPD
                     TSTPHVQGYLYCKNAVSFNTVRLWLLGRAHVEKAKATPAQNYEYCSKDKDFEEYGSRP
                     MSQEEKGEVGGDAQKEKWSTIWNLAKQGNIDEIAEQFPKECFVHYRNLLLIRQSGSGS
                     SGDLDNVCGLWLYGESGAGKSHKSRHMDTVENTYIKGINKWWCNYHGQRTVILEDVGK
                     DHGFLGDFLKIWADKWAFTAEFKGGTMARARPTRFVVTSQYRIDDIWQDKETRDALHR
                     RFEEQEVVKGPLDVLIRSLPDPVAVFPGVGAQEVTVSPIAYDVVPSDDDMALSLSPID
                     SEEMSDYEKRKRKRVKCPFIEDECGESD*"
ORIGIN
        1 tagtattacc ctagacccct agtgtgtgtg tgggacgccc ctattctcgc caaatggtcc
       61 ctattaggat agcctacact tttcgctgaa aaagttagcg ggtaaagaat ttttattttt
      121 tcgtttttcg ctggagccgg aaccacctcc aggtaaacgc aggagattag aggagaagat
      181 gcagtctgcc gctaagccca cctacgccaa cttcgacaaa gccctttgga gtgagtacaa
      241 ggaactcaag gactcgatag ccaatattaa gaacccggac cgcggagctc aaaatgccga
      301 gcgttacctt gccgcggtcg cgaagctcaa agccatgaat gccaatcctt acgagtacaa
      361 gatgcttttg aagcctgtta agtaccagat tgcccatctt ttgaagcaca tcccgatgga
      421 acactttttg aaggaacaag gatggggaaa ttgggttgcc cttaaacatt ttatggttgg
      481 aaagcgagct catcacaaag gaagaagagc acgcttcggg atctccgcga agagatggaa
      541 aggataccgc ggaggaagtc atggacctgg gctgcgtaga caatggaagt cgcagcagca
      601 cagaaagtat tatggtcgcc gagggaaaag ctattcggcg ccctatgttg ttggaagata
      661 tcgaagacct tggagacgat ttccaagata ttcaatgtat taataaattt acttagatgg
      721 cagctccttc tcggaaacga agtcgctggc ttggacctgc tttatcagcc ggatttggcg
      781 ctgctgcaac gtcttcgggt tatggtgcct ttgcaggagc tgcctcagct cttggcggag
      841 ctattgggca gaagttcttt gacatcacag gttatggtga ctacaacgtt aataagaact
      901 ctctactgtc aggtactgtg ccgtcggtag ggaattcttc tacacatcct gacggattga
      961 caatatcaca tcgtgagtac attggagatg tgattacgtc tgctactgct aagacgtttg
     1021 ctatatctga ttttgatctt aatgtcaaca atccacaaac atgggaatgg ttgacacaaa
     1081 ttgcatgtaa ctacgaagag tgggttcccg aagggatact cttttatttt aagagcacaa
     1141 gcggcgaggc ctttacgtca aacgacgcgt cgcttggtac agtcattatg gctacgaatt
     1201 acaatcctta caacaagcct ttcgagacga aagctgagat ggaaagctat gaattttgca
     1261 cgaacggccc cccaacgaat gatttgattc atatgattga gtgcgatccg cacgaaggaa
     1321 gtatatccac ttactatgtg tcaagtgtga actcttctgc caatgttgga ttgatggata
     1381 aaaggttcaa tacgcttggt accttttata ttgctactac tggttttcaa ggaaccaatg
     1441 tgaacatcgg tgaactttgg gtaacttacc aggttactct tctgaagcca aagctgtatg
     1501 aatctcttgg ccttgcttct gattctatta gagctgtgct ttcgggcact ttagaacaag
     1561 ataccagttt atatggtttg tggcccttga cgaatgcatc ccacgatgca gttattcagc
     1621 aaagaaccat tccctatact atacgtacgc cttccgatag cctctggtat gaatatgcca
     1681 gtggaaatgt cactttgcat tggccagtgt atgcgtatcc aacgtactat gaggtatcag
     1741 tacgagtctc caaaagtgtt aattttacag caccaggctt ttctctttct cagagcggag
     1801 gtacttttgg agcgacctat gctccagttc ttattacagg agaaggacaa actccagaga
     1861 ccggtaccac ctcgataagg tcgacgtgtt acaatgctgt tattaaagtt cctggaggat
     1921 ttatatgtca cgatccatca ggaacaccaa gtactagatt ctctacaagt accgcccttg
     1981 tagtgcatct tcaatggcaa atcagacaaa taccaaattt atcattgagt tcaggctata
     2041 ttgatcctta atcactctct ccgcactcat cctcaataaa aggacatttt actctctttc
     2101 tctttctctt ctcatagtcg ctcatctcct cagaatcaat aggagaaagc gacaaagcca
     2161 tatcatcgtc ggacggcaca acgtcgtagg cgatgggact tacagtaact tcctgtgcgc
     2221 ccacccctgg gaacacggcg acaggatccg gtaacgaacg tatgagcacg tcgagtggcc
     2281 ctttgaccac ctcttgttct tcgaaccttc tgtgaagggc gtcacgcgtc tctttgtctt
     2341 gccagatgtc atcgatgcgg tactgcgagg tgacaacgaa acgggtggga cgagctcttg
     2401 ccatggtccc gcctttaaac tcagcagtaa acgcccactt gtcagcccag atcttgagga
     2461 agtctccgag aaacccgtgg tctttcccaa cgtcttcgag gatgacagtt ctttgtccgt
     2521 ggtaattgca ccaccacttg ttgatgccct tgatgtaagt attttccact gtgtccatat
     2581 gccgcgactt gtgggatttt ccagcaccag actcgccgta gagccacaag ccgcacacgt
     2641 tatcgagatc accagaagac ccagaacctg actgtcgtat aagcaaaaga tttctgtagt
     2701 gtacgaagca ttcttttgga aattgttctg cgatctcgtc gatattccct tgcttcgcta
     2761 agttccagat agtactccac ttctctttct gcgcatctcc accaacctca cccttttctt
     2821 cttgagacat cggacgagaa ccgtactctt cgaagtcctt gtctttggaa cagtactcgt
     2881 agttttgcgc aggagttgcc ttcgcttttt cgacgtgagc cctgccaagc aaccaaaggc
     2941 gaacggtatt gaaagacaca gcgtttttac agtacagata accttgaacg tgaggagtag
     3001 aagtatccgg agcgacctca cgacccataa tccaataacg gcactcagcc aagttctcct
     3061 taatttttag gacttcgtca tgcgtccagt tgtttagggt aaagcaccag cctctcgatc
     3121 gctgcattag aaacaaacgt tcactcgaat tagggtttat ttagggttga attgttttca
     3181 gctaaatgcc aataaaaggg atcactatag aaaagaaatg tgcccccctt ggggggttgg
     3241 gggggaaaag gctcccgcag ggttaattgg cccccgcagg gcagtctgac cccattgctt
     3301 tcccgggaga tttgccatgt cgcctaatct ccccgtaggg gaaagcgggg tctgggggcg
     3361 cagcccccag gttttttttt ggggaatgtt ttttgacgtc ttgcacacag ggtctaggcc
//
LOCUS       CruV-251                3420 bp    DNA     circular     24-FEB-2020
DEFINITION  Crucivirus-251 isolate PCD_232.
ACCESSION   CruV-251
KEYWORDS    .
SOURCE      Crucivirus-251
  ORGANISM  Crucivirus-251
            Unclassified.
REFERENCE   1  (bases 1 to 3420)
  AUTHORS   de la Higuera,I., Kasun,G.W., Torrance,E.L., Pratt,A.A.,
            Maluenda,A., Colombet,J., Bisseux,M., Ravet,V., Dayaram,A.,
            Stainton,D., Kraberger,S., Zawar-Reza,P., Goldstien,S., Briskie,J.,
            White,R., Taylor,H., Gomez,C., Ainley,D.G., Harding,J.S.,
            Fontenele,R.S., Schreck,J., Ribeiro,S.G., Oswald,S.A., Arnold,J.M.,
            Varsani,A., Enault,F. and Stedman,K.M.
  TITLE     Direct Submission
  JOURNAL   Submitted (24-FEB-2020) Biology, Portland State University, 1719 SW
            10th Ave, Portland, Oregon 97201, USA
COMMENT     Bankit Comment: ALT EMAIL:ide@pdx.edu
            Bankit Comment: TOTAL # OF SEQS:451
            Bankit Comment: GAP: est. length - alignment

            ##Assembly-Data-START##
            Assembly Method       :: MetaSPAdes v. 3.12; IDBA-UD v. 1.1.1;
                                     MEGAHIT v. 1.0.3
            Sequencing Technology :: 454; Illumina
            ##Assembly-Data-END##
FEATURES             Location/Qualifiers
     source          1..3420
                     /organism="Crucivirus-251"
                     /mol_type="genomic DNA"
                     /isolate="PCD_232"
                     /isolation_source="Soil"
                     /country="New Zealand"
                     /collection_date=2012
     misc_feature    join(3410..3420,1..18)
                     /note="stem-loop"
     misc_feature    1..9
                     /note="nonanucleotide"
     CDS             86..703
                     /codon_start=1
                     /product="putative ORF"
                     /translation="MKKLAGKEFLFFRFSLEPEPPPGKRRRLEEKMQSAAKPTYANFD
                     KALWSEYKELKDSIANIKNPDRGAQNAERYLAAVAKLKAMNANPYEYKMLLKPVKYQI
                     AHLLKHIPMEHFLKEQGWGNWVALKHFMVGKRAHHKGRRARFGISAKRWKGYRGGSHG
                     PGLRRQWKSQQHRKYYGRRGKSYSAPYVVGRYRRPWRRFPRYSMY*"
     CDS             complement(167..598)
                     /codon_start=1
                     /product="putative ORF"
                     /translation="MLRLPLSTQPRSMTSSAVSFPSLRGDPEACSSSFVMSSLSNHKM
                     FKGNPISPSLFLQKVFHRDVLQKMGNLVLNRLQKHLVLVRIGIHGFELRDRGKVTLGI
                     LSSAVRVLNIGYRVLEFLVLTPKGFVEVGVGGLSGRLHLLL*"
     CDS             717..2051
                     /codon_start=1
                     /product="Putative CruV Capsid Protein"
                     /translation="MAAPSRKRSRWLGPALSAGFGAAATSSGYGAFAGAASALGGAIG
                     QKFFDITGYGDYNVNKNSLLSGTVPSVGNSSTHPDGLTISHREYIGDVITSATAKTFA
                     ISDFDLNVNNPQTWEWLTQIACNYEEWVPEGILFYFKSTSGEAFTSNDASLGTVIMAT
                     NYNPYNKPFETKAEMESYEFCTNGPPTNDLIHMIECDPHEGSISTYYVSSVNSSANVG
                     LMDKRFNTLGTFYIATTGFQGTNVNIGELWVTYQVTLLKPKLYESLGLASDSIRAVLS
                     GTLEQDTSLYGLWPLTNASHDAVIQQRTIPYTIRTPSDSLWYEYASGNVTLHWPVYAY
                     PTYYEVSVRVSKSVNFTAPGFSLSQSGGTFGATYAPVLITGEGQTPETGTTSIRSTCY
                     NAVIKVPGGFICHDPSGTPSTRFSTSTALVVHLQWQIRQIPNLSLSSGYIDP*"
     CDS             complement(2048..3136)
                     /codon_start=1
                     /product="Putative Replication Associated Protein"
                     /translation="MFLMQRSRGWCFTLNNWTHDEVLKIKENLAECRYWIMGREVAPD
                     TSTPHVQGYLYCKNAVSFNTVRLWLLGRAHVEKAKATPAQNYEYCSKDKDFEEYGSRP
                     MSQEEKGEVGGDAQKEKWSTIWNLAKQGNIDEIAEQFPKECFVHYRNLLLIRQSGSGS
                     SGDLDNVCGLWLYGESGAGKSHKSRHMDTVENTYIKGINKWWCNYHGQRTVILEDVGK
                     DHGFLGDFLKIWADKWAFTAEFKGGTMARARPTRFIVTSQYRIDDIWQDKETRDALHR
                     RFEEQEVVKGPLDVLIRSLPDPVAVFPGVGAQEVTVSPIAYDVVPSDDDMALSLSPID
                     SEEMSDYEKRKRKRVKCPFIEDECGESD*"
ORIGIN
        1 tagtattacc ctagacccct agtgtgtgtg tgggacgccc ctattctcgc caaatggtcc
       61 ctattaggat agcctacact tttcgctgaa aaagttagcg ggtaaagaat ttttattttt
      121 tcgtttttcg ctggagccgg aaccacctcc aggtaaacgc aggagattag aggagaagat
      181 gcagtctgcc gctaagccca cctacgccaa cttcgacaaa gccctttgga gtgagtacaa
      241 ggaactcaag gactcgatag ccaatattaa gaacccggac cgcggagctc aaaatgccga
      301 gcgttacctt gccgcggtcg cgaagctcaa agccatgaat gccaatcctt acgagtacaa
      361 gatgcttttg aagcctgtta agtaccagat tgcccatctt ttgaagcaca tcccgatgga
      421 acactttttg aaggaacaag gatggggaaa ttgggttgcc cttaaacatt ttatggttgg
      481 aaagcgagct catcacaaag gaagaagagc acgcttcggg atctccgcga agagatggaa
      541 aggataccgc ggaggaagtc atggacctgg gctgcgtaga caatggaagt cgcagcagca
      601 cagaaagtat tatggtcgcc gagggaaaag ctattcggcg ccctatgttg ttggaagata
      661 tcgaagacct tggagacgat ttccaagata ttcaatgtat taataaattt acttagatgg
      721 cagctccttc tcggaaacga agtcgctggc ttggacctgc tttatcagcc ggatttggcg
      781 ctgctgcaac gtcttcgggt tatggtgcct ttgcaggagc tgcctcagct cttggcggag
      841 ctattgggca gaagttcttt gacatcacag gttatggtga ctacaacgtt aataagaact
      901 ctctactgtc aggtactgtg ccgtcggtag ggaattcttc tacacatcct gacggattga
      961 caatatcaca tcgtgagtac attggagatg tgattacgtc tgctactgct aagacgtttg
     1021 ctatatctga ttttgatctt aatgtcaaca atccacaaac atgggaatgg ttgacacaaa
     1081 ttgcatgtaa ctacgaagag tgggttcccg aagggatact cttttatttt aagagcacaa
     1141 gcggcgaggc ctttacgtca aacgacgcgt cgcttggtac agtcattatg gctacgaatt
     1201 acaatcctta caacaagcct ttcgagacga aagctgagat ggaaagctat gaattttgca
     1261 cgaacggccc cccaacgaat gatttgattc atatgattga gtgcgatccg cacgaaggaa
     1321 gtatatccac ttactatgtg tcaagtgtga actcttctgc caatgttgga ttgatggata
     1381 aaaggttcaa tacgcttggt accttttata ttgctactac tggttttcaa ggaaccaatg
     1441 tgaacatcgg tgaactttgg gtaacttacc aggttactct tctgaagcca aagctgtatg
     1501 aatctcttgg ccttgcttct gattctatta gagctgtgct ttcgggcact ttagaacaag
     1561 ataccagttt atatggtttg tggcccttga cgaatgcatc ccacgatgca gttattcagc
     1621 aaagaaccat tccctatact atacgtacgc cttccgatag cctctggtat gaatatgcca
     1681 gtggaaatgt cactttgcat tggccagtgt atgcgtatcc aacgtactat gaggtatcag
     1741 tacgagtctc caaaagtgtt aattttacag caccaggctt ttctctttct cagagcggag
     1801 gtacttttgg agcgacctat gctccagttc ttattacagg agaaggacaa actccagaga
     1861 ccggtaccac ctcgataagg tcgacgtgtt acaatgctgt tattaaagtt cctggaggat
     1921 ttatatgtca cgatccatca ggaacaccaa gtactagatt ctctacaagt accgcccttg
     1981 tagtgcatct tcaatggcaa atcagacaaa taccaaattt atcattgagt tcaggctata
     2041 ttgatcctta atcactctct ccgcactcat cctcaataaa aggacatttt actctctttc
     2101 tctttctctt ctcatagtcg ctcatctcct cagaatcaat aggagaaagc gacaaagcca
     2161 tatcatcgtc ggacggcaca acgtcgtagg cgatgggact tacagtaact tcctgtgcgc
     2221 ccacccctgg gaacacggcg acaggatccg gtaacgaacg tatgagcacg tcgagtggcc
     2281 ctttgaccac ctcttgttct tcgaaccttc tgtgaagggc gtcacgcgtc tctttgtctt
     2341 gccagatgtc atcgatgcgg tactgcgagg tgacaatgaa acgggtggga cgagctcttg
     2401 ccatggtccc gcctttaaac tcagcagtaa acgcccactt gtcagcccag atcttgagga
     2461 agtctccgag aaacccgtgg tctttcccaa cgtcttcgag gatgacagtt ctttgtccgt
     2521 ggtaattgca ccaccacttg ttgatgccct tgatgtaagt attttccact gtgtccatat
     2581 gccgcgactt gtgggatttt ccagcaccag actcgccgta gagccacaag ccgcacacgt
     2641 tatcgagatc accagaagac ccagaacctg actgtcgtat aagcaaaaga tttctgtagt
     2701 gtacgaagca ttcttttgga aattgttctg cgatctcgtc gatattccct tgcttcgcta
     2761 agttccagat agtactccac ttctctttct gcgcatctcc accaacctca cccttttctt
     2821 cttgagacat cggacgagaa ccgtactctt cgaagtcctt gtctttggaa cagtactcgt
     2881 agttttgcgc aggagttgcc ttcgcttttt cgacgtgagc cctgccaagc aaccaaaggc
     2941 gaacggtatt gaaagacaca gcgtttttac agtacagata accttgaacg tgaggagtag
     3001 aagtatccgg agcgacctca cgacccataa tccaataacg gcactcagcc aagttctcct
     3061 taatttttag gacttcgtca tgcgtccagt tgtttagggt aaagcaccag cctctcgatc
     3121 gctgcattag aaacaaacgt tcactcgaat tagggtttat ttagggttga attgttttca
     3181 gctaaatgcc aataaaaggg atcactatag aaaagaaatg tgcccccctt ggggggttgg
     3241 gggggaaaag gctcccgcag ggttaattgg cccccgcagg gcagtctgac cccattgctt
     3301 tcccgggaga tttgccatgt cgcctaatct ccccgtaggg gaaagcgggg tctgggggcg
     3361 cagcccccag gttttttttt ggggaatgtt ttttgacgtc ttgcacacag ggtctaggcc
//
LOCUS       CruV-252                3423 bp    DNA     circular     24-FEB-2020
DEFINITION  Crucivirus-252 isolate 120_1349_IR2_3297.
ACCESSION   CruV-252
KEYWORDS    .
SOURCE      Crucivirus-252
  ORGANISM  Crucivirus-252
            Unclassified.
REFERENCE   1  (bases 1 to 3423)
  AUTHORS   de la Higuera,I., Kasun,G.W., Torrance,E.L., Pratt,A.A.,
            Maluenda,A., Colombet,J., Bisseux,M., Ravet,V., Dayaram,A.,
            Stainton,D., Kraberger,S., Zawar-Reza,P., Goldstien,S., Briskie,J.,
            White,R., Taylor,H., Gomez,C., Ainley,D.G., Harding,J.S.,
            Fontenele,R.S., Schreck,J., Ribeiro,S.G., Oswald,S.A., Arnold,J.M.,
            Varsani,A., Enault,F. and Stedman,K.M.
  TITLE     Direct Submission
  JOURNAL   Submitted (24-FEB-2020) Biology, Portland State University, 1719 SW
            10th Ave, Portland, Oregon 97201, USA
COMMENT     Bankit Comment: ALT EMAIL:ide@pdx.edu
            Bankit Comment: TOTAL # OF SEQS:451
            Bankit Comment: GAP: est. length - alignment

            ##Assembly-Data-START##
            Assembly Method       :: MetaSPAdes v. 3.12; IDBA-UD v. 1.1.1;
                                     MEGAHIT v. 1.0.3
            Sequencing Technology :: 454; Illumina
            ##Assembly-Data-END##
FEATURES             Location/Qualifiers
     source          1..3423
                     /organism="Crucivirus-252"
                     /mol_type="genomic DNA"
                     /isolate="120_1349_IR2_3297"
                     /isolation_source="Water"
                     /country="Norway"
                     /collection_date=2011
     CDS             1..1329
                     /codon_start=1
                     /product="Putative Replication Associated Protein"
                     /translation="MPASLASKSRKWCFTMFWDGKEPGKDYFDDWECDYMYVGKEICP
                     ESRREHYQGYIRFTNPRALAGVRKLFTYTVLTPAGRPGYPGHWEPCKGTEEQNVRYCA
                     KDEDLVIEWGDREADLAIDKTQGKRNDLIAVREMVRDGANMRDVIENCNSYQGIKIAE
                     TMLKHMEAPRSWAPEVYWIYGPTGTGKSKIAFDLCENPWTSAKDGKWFDGYDGHEDVI
                     FDDFRHDFCPFHILLRLLDRYPYRIENKGGCRQFLAKRIFITSCHSPDKVYPLLGEDL
                     AQLGRRLTHIIYAPRLGEYFRMPGSEIGLEPSASFVFPELGTEQKVGGNTTEVTDDKS
                     PLTPALTRPPPGFSICNKCKMINPPVNHHMTCGNVESKCVVTHSADMVCDGCENDELL
                     AAFNEEEASSAVILHTPEALGDVQVVAPRNIPPVFHPAPPPRHKGGPLTW*"
     CDS             complement(1431..2951)
                     /codon_start=1
                     /product="Putative CruV Capsid Protein"
                     /translation="MPRKAVANYTRPGYVRPPSYTPAQVAKVKAAAAFARANPRVQAL
                     GGYARVRPRAVARPRAAARSSGGSSLGTAIGSALGLALGGPAGGAMGGMIGNAGGGFL
                     SKIFGHGDYAVSNASTLKENNIALSNSANIPQFGTGKVATKFRHREFLGDVFSSSTAN
                     TFKIDSYEINPGMPETFPWLAGVVGAKYQQYRINGMTFEFRSMSSDALNSTNTALGSV
                     IMSTDYDSADTVFSSKQQMENTENGVSCKPSVNMMHGIECARAQTPVNELYIRAFDVP
                     DGKDIRLYDLGRFSIASTGCQGTNVNLGELWVSYDIDCFKAIEQVPLYLQPFAQYGLT
                     AASATAPLGTSRAQLLTTGADNSLALTLTENKISWTDIPQVGSVWRVDYHYLGSSAAL
                     TTPVITTGNGMFININTLWKTSSITTTNLNLSCDIQYQGGATPAALPYLNIADFTWAT
                     SLVQADLYVYQMTGLMPGTFNDQSEISLAELDIDTDAPEYVHCLEEQKKPQLARRR*"
     CDS             3027..3326
                     /codon_start=1
                     /product="putative ORF"
                     /translation="MSSSCCRSRVSHADILCFVIFIIFYTLQERRFHKGSARYMPSLA
                     FFKRFDEHTLGCCYRSRFVFPLFAGRFAGSRGSANSLIAGASPCRPIFRPKAYLT*"
ORIGIN
        1 atgcctgcgt cactcgcttc gaagtcacgt aaatggtgct ttactatgtt ttgggacggc
       61 aaagaacccg gtaaggatta ctttgatgat tgggagtgcg attatatgta cgttggtaaa
      121 gaaatctgcc ctgaatctag gcgcgaacac taccaaggat acattcgttt cactaatcct
      181 cgggctctcg ctggtgttcg aaaactcttt acatatactg tgctgacccc tgccggacgc
      241 cctggctatc ccggacattg ggaaccttgt aaaggtactg aagagcaaaa cgttcgctat
      301 tgtgccaaag acgaagatct cgttattgaa tggggtgacc gtgaagctga tctcgctata
      361 gataagactc aaggtaaacg taacgacctc attgcagttc gcgaaatggt tcgggacggt
      421 gctaacatgc gagatgttat cgaaaactgc aactcttacc aagggataaa gattgctgaa
      481 acaatgctaa agcatatgga agcaccacga agttgggcgc ctgaagttta ttggatttat
      541 ggccctactg ggacaggtaa aagcaagatt gctttcgatc tctgcgagaa tccttggact
      601 tccgctaaag acggtaagtg gtttgatgga tatgatggac acgaagacgt gatctttgat
      661 gactttcgtc acgatttctg cccctttcac attctgctta ggttgcttga tagatatcct
      721 tacaggattg aaaataaagg tggctgccgc cagtttcttg caaaacgtat ctttattacg
      781 agttgccact cgcccgacaa agtgtaccca cttcttgggg aagaccttgc ccagttgggg
      841 cgaaggttga cgcatattat ttatgcgccg cggctcggtg aatactttcg tatgcccgga
      901 tcggagatag ggctagagcc ctccgctagc tttgtgtttc cggaacttgg aacggaacaa
      961 aaagtcgggg gtaatactac ggaggtcacg gatgataaat caccgctcac tcctgcgcta
     1021 acgcgccccc ctcctggttt tagtatatgc aacaagtgca aaatgattaa tcctccggtg
     1081 aatcatcaca tgacctgtgg taatgttgag agcaaatgtg tggtgacaca tagtgctgat
     1141 atggtctgcg acggctgtga aaatgatgag ctactcgctg catttaatga ggaagaagcc
     1201 tcttccgcgg ttatattaca tacaccagaa gcccttggtg atgtacaagt tgtcgctccg
     1261 cgcaacatac cgccggtatt tcatccggct ccgccaccta ggcataaagg aggaccactt
     1321 acttggtaag aatttgctat cgccacgctt gtgactcccg caagggttaa gaacctcaca
     1381 aaaaattttt tggtgactta gtcagctacg ttgttacaca accgctttga ctatcgcctt
     1441 cgggcaagct gaggcttttt ttgctcctca agacaatgca cgtactcagg tgcgtccgtg
     1501 tcaatatcca gctctgcgag actgatctct gattgatcgt tgaaggttcc tggcataaga
     1561 cctgtcatct ggtaaacata cagatctgct tgaactaggg atgtcgccca tgtaaaatct
     1621 gcgatattca gatagggtaa agcggcaggt gtggcgcctc cttgatactg aatatcacac
     1681 gaaagattca agtttgttgt tgtaatcgaa gacgttttcc aaagagtgtt gatattaata
     1741 aacatcccgt tacccgttgt gatgacggga gttgttagcg cagcagatga accaagataa
     1801 tgataatcta cgcgccatac agaccctacc tgcggaatgt ctgtccaaga aatcttgttt
     1861 tctgttaatg taagagctaa agaattgtcg gcacctgttg ttaacagttg tgctctagat
     1921 gtacccaatg gggctgtagc agatgcagct gttaacccat attgggcgaa aggctgcaaa
     1981 tacaatggta cttgctcaat agccttgaaa caatcaatgt catatgacac ccacaactct
     2041 ccaagattca cattcgttcc ctggcagcca gttgatgcga ttgaaaaccg tcccaaatca
     2101 taaagacgaa tatccttgcc atctggcaca tcaaacgctc ggatatacaa ctcgttcact
     2161 ggcgtctgag ctcgtgcaca ttcgatgcca tgcatcatgt tgactgatgg tttacaagac
     2221 actccatttt ctgtgttttc catctgttgt ttggaagaga agactgtgtc tgctgaatca
     2281 tagtctgttg acatgataac tgaaccgaga gcggtatttg ttgaatttaa cgcatcagac
     2341 gacattgatc gaaactcaaa ggtcataccg ttgattcgat attgctgata cttagcaccg
     2401 actacgccgg ctaaccatgg aaatgtttct ggcattcctg gattgatttc ataactgtca
     2461 atcttaaacg tgttcgctgt agatgaagag aacacatcac caagaaattc acgatgcctg
     2521 aacttggtgg ctaccttgcc cgttccgaac tgtggtatat ttgctgaatt cgataacgca
     2581 atgttgtttt ctttcaacgt tgatgcgtta ctaacagcat aatctccgtg cccgaagatt
     2641 ttggataaga acccacctcc ggcgtttcct atcattccac ccatcgcacc tccagcaggt
     2701 cctcctaagg ccaacccaag ggctgacccg atggctgtac ccagcgaaga acctccgctt
     2761 gatcgggcag cagctctagg tcgagctaca gccctcggtc taacacgagc ataaccacct
     2821 aaagcttgta ctctaggatt ggctcgagca aatgcagccg ctgctttgac ttttgccacc
     2881 tgcgccggcg tatatgacgg cggacgcaca taccctggtc gggtataatt ggcaactgct
     2941 ttgcggggca tcgcgttgac gggtatacta cccgattata ttcttctaat acaatattat
     3001 atggtgcatg gctatatagc cagcgcatgt cttcctcatg ctgtcgctct cgggtatccc
     3061 acgctgacat cttatgtttt gttattttta taatttttta tactttgcaa gagcgtcgct
     3121 tccataaagg gtctgctcgc tacatgccca gtctggcgtt cttcaaaaga ttcgacgaac
     3181 acacccttgg gtgctgctat cgcagtaggt ttgtctttcc cttgttcgcg gggcgcttcg
     3241 ctggctcacg tgggagcgca aactcattga tagcaggggc aagcccttgt cgccctattt
     3301 ttcggccaaa ggcatacttg acgtgatcat gtaggtcgga ccaccttgtt tgctccttgc
     3361 ctcttgctgc tataaatatt ttattttaga cttcttgttc cgggcactta tacccagctc
     3421 gtc
//
LOCUS       CruV-253                3424 bp    DNA     circular     24-FEB-2020
DEFINITION  Crucivirus-253 isolate 001510_virusbaton.
ACCESSION   CruV-253
KEYWORDS    .
SOURCE      Crucivirus-253
  ORGANISM  Crucivirus-253
            Unclassified.
REFERENCE   1  (bases 1 to 3424)
  AUTHORS   de la Higuera,I., Kasun,G.W., Torrance,E.L., Pratt,A.A.,
            Maluenda,A., Colombet,J., Bisseux,M., Ravet,V., Dayaram,A.,
            Stainton,D., Kraberger,S., Zawar-Reza,P., Goldstien,S., Briskie,J.,
            White,R., Taylor,H., Gomez,C., Ainley,D.G., Harding,J.S.,
            Fontenele,R.S., Schreck,J., Ribeiro,S.G., Oswald,S.A., Arnold,J.M.,
            Varsani,A., Enault,F. and Stedman,K.M.
  TITLE     Direct Submission
  JOURNAL   Submitted (24-FEB-2020) Biology, Portland State University, 1719 SW
            10th Ave, Portland, Oregon 97201, USA
COMMENT     Bankit Comment: ALT EMAIL:ide@pdx.edu
            Bankit Comment: TOTAL # OF SEQS:451
            Bankit Comment: GAP: est. length - alignment

            ##Assembly-Data-START##
            Assembly Method       :: MetaSPAdes v. 3.12; IDBA-UD v. 1.1.1;
                                     MEGAHIT v. 1.0.3
            Sequencing Technology :: 454; Illumina
            ##Assembly-Data-END##
FEATURES             Location/Qualifiers
     source          1..3424
                     /organism="Crucivirus-253"
                     /mol_type="genomic DNA"
                     /isolate="001510_virusbaton"
                     /isolation_source="Water"
                     /country="France"
                     /collection_date=2013
     CDS             1..921
                     /codon_start=1
                     /product="Putative Replication Associated Protein"
                     /translation="MSIDNCRARNWCFTLNNYTQEEFDEAEKLGTKTNYIIVGKEVGS
                     NSTPHLQGYLEYPNAVRGSAIRKLFGGRAHWEVRKGTPKQASEYCKKEKDFFEIGTLS
                     AQGTRTDLDEISSAVLAGASFQEIATTFPGTYIKYHKGIAALKDAAEGRLDLKPRDPN
                     KPPCVIWRWGLAGVGKTRHIYDKHKSIFLQKGTKWWDGYSQQEAIIIDDFDGRWPYRD
                     LLKVLDRYPLRVEIKGGTMELNSPFIYITCEHPPSWFWGLTAKYRNNNDAPPSENELE
                     QIMRRIVQGGGSVTEVVTQVWVVAEETKTG*"
     CDS             complement(1760..3259)
                     /codon_start=1
                     /product="Putative CruV Capsid Protein"
                     /translation="MPQARRSAARKPAGAPVRRAPARASARAPARAPARAPKPRSQAG
                     SAYRTGGATLGGMLGTALGGPAGTAVGSMVGRSAGAMFSKLLGHGDYQVSNVSQLKQN
                     NLVLSNAAQIPQFGTGKVAVKFKHREFLGDVFSSSTANTFKIDSYPLNPGQAITFPWL
                     SGVVGSKFQQYRINGMVFEFRSMSSDALNSTNTALGNVIMSTDYDSADVVFASKQEME
                     NTEYGISCKPSVNMLHGIECERFQTPVSELYIRAFDVPSGKDIRLYDLGRFSIASTGC
                     QGTNVNLGELWVSYDIDAFKAIEQVPGYLMPYAGYDCTGMSATVPFGTTRAIYDVTNG
                     ADTIGLTVGENKIEWPINIQVGATYMVIVQAVASESKSLTTPAVTSGNGMYIDHDMHL
                     TAPATTTTTGSWQLVYPVQYKGGATPAALPYLNVADYSWAVASITATRLFVVQVSGFF
                     PGVYTDVVGALPPDRALRDESEEEDTVKVVLRESVLKSMGGEQKNANRR*"
ORIGIN
        1 atgagtatcg ataactgtcg tgcacgtaac tggtgcttca cacttaacaa ctatacacaa
       61 gaagagttcg acgaagctga aaagctcgga acaaaaacga actacattat tgtgggaaaa
      121 gaagtaggtt caaacagtac acctcaccta caagggtacc tagagtaccc aaacgcagta
      181 agaggatctg cgatacgcaa actattcgga ggaagagcac attgggaagt acgcaaagga
      241 accccaaaac aagcatccga atattgcaaa aaagaaaaag atttctttga aatcggaaca
      301 ctttctgctc aaggtacgcg taccgacctt gacgagatta gctctgcagt acttgcagga
      361 gcatcgttcc aagaaattgc gaccactttc ccaggaacat acatcaaata tcacaaagga
      421 attgcagctc ttaaagatgc tgcagaagga cgcttggatc tcaaaccaag agatcctaac
      481 aagcctccgt gtgtgatatg gagatgggga ctagctggag taggaaaaac acgtcacatt
      541 tatgacaaac ataaatccat tttcttacaa aaaggaacaa aatggtggga cggttactca
      601 cagcaagaag cgataatcat cgacgacttc gacggacgat ggccttatcg tgacctattg
      661 aaagtgcttg atcgttaccc tcttcgagtt gaaatcaaag gcggaacgat ggaacttaat
      721 agtccattta tatacatcac ctgcgaacac cccccatcct ggttctgggg gttgacggcg
      781 aagtatagaa acaacaacga cgcgccacct tcagagaatg aacttgagca aatcatgaga
      841 cgaattgttc aaggtggcgg ttcagttact gaagttgtta cccaagtttg ggtagtggca
      901 gaagagacta aaaccggtta aataacctaa ataacagggt agtcaaaata gataccctta
      961 ttttttgatt ctattctcaa aaatacctat gttagccact ttaaaaaagt tggcacagat
     1021 gtatttccaa ttctgttaac acctgagcat tattttcaat aatagacagc acaggacgtt
     1081 ttggcacaga agtggtgggg taatactagt gtgccaaggc acacttaccc accactacgg
     1141 tctttgacct attctgacca ataaattcta tataaaaatg tttcggcata gatataactg
     1201 acagttacat ctcatgacga cactcccaaa agttctgata gacataatat gtgagtatgc
     1261 tttccctccg agagaatgca taaacacatt gaatgaactt cgttcactac atgaacattg
     1321 gctcccattt ggaagaacgg gaagactatc cttcactaaa tggtggtgga tagaaagaca
     1381 tataacttat gctgattaca aacgtaacat gctaatggga tacgacgaga cagaagtata
     1441 tcattcaaca tatgaatgat atgatacaac ggacgctcag cttcgctggc gtcctgtgtg
     1501 tacaaatatg gtgcacgacc tccggtcgtg gctcggggcg caaggcccct cgccttcctg
     1561 cacaaacatg tgtgtctagc gggccgctga gtcgcggccc ttgacgcctg aggcgctcgc
     1621 cgcgcggcgt caccttcggt gactaagggc aggatggtta gatatggccg actccgcttc
     1681 gctacgcggc caagaccaaa gcctgtaaaa aaaaatataa aattttgttc attttttgac
     1741 taagaaacca tacaaacact caccgacggt tcgcattttt ttgctctcca cccatagatt
     1801 tcagtacgga ctctcgcaag acaaccttca cggtatcttc ctcctcggac tcatcgcgca
     1861 aagcacgatc gggagggaga gctcctacaa catcggtata aactcctgga aagaagccac
     1921 tcacttgaac gacaaagagt cgtgtagcgg tgatactagc aacagcccaa gaataatccg
     1981 cgacattgag atagggaagg gctgcaggtg tggcacctcc cttatattga acgggataga
     2041 ccaactgcca tgagccagtt gttgtggttg tagctggagc tgtaagatgc atatcatgat
     2101 cgatatacat accattacca gaggtaacgg ctggagtagt gagagacttt gactcacttg
     2161 caacggcctg aacgatgacc atgtatgtag cgccgacctg gatattgatt ggccactcaa
     2221 ttttgttctc accgacagtg agaccgatag tatcggcacc attggtcaca tcgtagatag
     2281 cacgtgtagt accaaaagga acagtagcag acattccagt acaatcgtac ccagcatatg
     2341 gcataagata cccagggact tgctcgatag ccttaaaggc atcgatatca tatgagaccc
     2401 aaagttcacc aaggttcaca ttggttcctt gacatcctgt agatgcaata gagaatcgcc
     2461 ctagatcata gaggcgaata tccttgccac tcggcacatc aaatgcacga atatagagct
     2521 ccgacactgg agtctgaaac cgttcgcact cgatgccatg aagcatattc accgaaggtt
     2581 tgcacgaaat accatattca gtattctcca tttcttgctt tgaagcaaaa accacgtcag
     2641 ccgaatcata atcagtagac atgataacgt ttccgagggc tgtgttggtt gagttgagag
     2701 catcagaaga catgcttctg aactcaaaaa ccataccgtt gatacggtac tgttgaaatt
     2761 tcgaaccaac aacacccgac aaccacggaa aagtgatagc ctgaccagga ttgagaggat
     2821 agctgtcaat cttgaatgtg ttagcagtag aagaactgaa cacatcaccg agaaactcac
     2881 ggtgcttgaa cttgaccgcc accttcccag ttccgaactg aggaatttga gcggcattag
     2941 aaagaacaag attgttctgt ttgagttgac tgacattgga tacttgatag tctccatgtc
     3001 cgagcaattt actgaacata gctccggcgc tacgtccaac catactaccg acggcggttc
     3061 cagcaggacc accgagagca gtacccaaca taccgccaag ggttgcacct ccggtacgat
     3121 acgcactacc tgcttgagag cgaggcttag gcgcacgagc aggagcacga gcaggagcac
     3181 gagcagaagc acgagcagga gcacgacgaa caggagcacc tgctggcttt cgagcagcag
     3241 aacgacgagc ctgaggcatg ttggtattaa agttagtacg gagtcagagt atatatagta
     3301 agtagatact ctttaaaaga tttattaatt aaataagtta attaatttta ttctgagcat
     3361 tggcacagaa gttatatagt cagtgagttc actaagtgat agattctcag agctttggac
     3421 gggg
//
LOCUS       CruV-254                3425 bp    DNA     circular     24-FEB-2020
DEFINITION  Crucivirus-254 isolate 120_141_SvL2_3298.
ACCESSION   CruV-254
KEYWORDS    .
SOURCE      Crucivirus-254
  ORGANISM  Crucivirus-254
            Unclassified.
REFERENCE   1  (bases 1 to 3425)
  AUTHORS   de la Higuera,I., Kasun,G.W., Torrance,E.L., Pratt,A.A.,
            Maluenda,A., Colombet,J., Bisseux,M., Ravet,V., Dayaram,A.,
            Stainton,D., Kraberger,S., Zawar-Reza,P., Goldstien,S., Briskie,J.,
            White,R., Taylor,H., Gomez,C., Ainley,D.G., Harding,J.S.,
            Fontenele,R.S., Schreck,J., Ribeiro,S.G., Oswald,S.A., Arnold,J.M.,
            Varsani,A., Enault,F. and Stedman,K.M.
  TITLE     Direct Submission
  JOURNAL   Submitted (24-FEB-2020) Biology, Portland State University, 1719 SW
            10th Ave, Portland, Oregon 97201, USA
COMMENT     Bankit Comment: ALT EMAIL:ide@pdx.edu
            Bankit Comment: TOTAL # OF SEQS:451
            Bankit Comment: GAP: est. length - alignment

            ##Assembly-Data-START##
            Assembly Method       :: MetaSPAdes v. 3.12; IDBA-UD v. 1.1.1;
                                     MEGAHIT v. 1.0.3
            Sequencing Technology :: 454; Illumina
            ##Assembly-Data-END##
FEATURES             Location/Qualifiers
     source          1..3425
                     /organism="Crucivirus-254"
                     /mol_type="genomic DNA"
                     /isolate="120_141_SvL2_3298"
                     /isolation_source="Water"
                     /country="Norway"
                     /collection_date=2012
     CDS             1..1281
                     /codon_start=1
                     /product="Putative Replication Associated Protein"
                     /translation="MPASIASKSRKWCFTMYWDGKEPGKDYFDDWECDYMYIGKEICP
                     ESRREHYQGYIRFTNPRALAGVRKLFTHTVLTPAGRPGYPGHWEACKGTETQNVRYCA
                     KDEDLVIEWGDREADLTEDKAQGKRNDIIAVREMIRNGANMRDVIENCNSYQGIKIAE
                     TMLKHMEAPRDWVPEVYWIYGPTGTGKSKLAFDLCENPWTSAKDGKWFDGYDGHEDVI
                     FDDFRHDFCPFHILLRLLDRYPYRIEHKGGCRQFLAKRIFITSCHSPDKVYPLLREDL
                     AQLGRRLTHIIYAPRLGEYYGMPGSEIGMEPSASFVIPELGTEQKVGGNTTEVSDDKS
                     LLAPALTRPPPGLIESKCVVTHGADMVCDGCENDELLAAFAEEEASSTVILHTPEALG
                     EVQVVAPRNNMPKSIPPVFQPGPPPRRKGGPLTW*"
     CDS             complement(1381..2898)
                     /codon_start=1
                     /product="Putative CruV Capsid Protein"
                     /translation="MPRKAVANYTRPGYKRPSAYTAAMQAKAKAGLAYARANPRTQLL
                     GGYARARPRAVARPRAAKSSGGSALGSAIGSALGLALGGPAGGAMGGMIGNAGGGFLS
                     KIFGHGDYAVTNAGTLKENNIALSNSANIPQFGTGKVATKFKHREFLGDVFSSSAANT
                     FKINSFSINPGQEETFPWLAGVVAAKYQQYRINGLTFEFRSMSADALNSTNTALGSVI
                     MSTDYDSADTVFTTKQQMENTENGVSCKPSVNMMHGIECERAQTPVNELYIRAFDVPS
                     GKDIRLYDLGRFSIATTGFQGTNVNCGELWVSYDIDCFKAIEQVPGYLIPFSQYGLTA
                     ASATAPLGTSRAQLLTTGAANSIDLTLTENRISWPISTPVGSRFHLQWDMLADSKALT
                     PPAITAGNGMFINANTIWKSSVATTPDCFLQCDVEYQGGATPAALPYLNIADFTWTGI
                     TLQQADLYVTQTSGLLPGAYTSQSEITVSDLDPDDTEYVQQLDEEKRPRARVMKR*"
ORIGIN
        1 atgcctgcct ctattgcttc gaagtctcgt aaatggtgtt ttactatgta ttgggatggc
       61 aaagaacctg gaaaagatta ctttgatgat tgggaatgcg attatatgta cattggtaaa
      121 gaaatctgcc ctgaatctag gcgcgaacac tatcaaggat acatccgttt cactaatcct
      181 cgggctctcg ctggtgttcg aaaactattc acacatactg tgttaacccc tgccggtcgc
      241 cctggctatc ccggacattg ggaggcttgt aagggtactg aaactcaaaa cgttagatat
      301 tgcgctaaag acgaagatct cgttattgaa tggggtgacc gtgaagctga tcttactgaa
      361 gataaggctc aaggtaaaag gaacgacatt atcgcagttc gcgaaatgat tcgtaatggt
      421 gctaacatga gagatgttat tgaaaattgc aactcttacc aagggataaa gattgctgaa
      481 actatgctaa agcatatgga agccccacgt gattgggtgc ctgaggttta ttggatttat
      541 ggtcctactg ggactggtaa aagcaagctt gctttcgatc tctgcgagaa cccatggact
      601 tccgctaagg acggtaagtg gtttgatggt tatgatggac acgaagacgt gatctttgat
      661 gactttcgtc atgatttctg cccctttcac attctgctca ggttgctaga tagatatccc
      721 tacaggattg aacataaagg tggctgtcgc cagtttcttg cgaaacggat ctttattacg
      781 agttgtcact cgcccgacaa agtgtaccca cttcttaggg aagaccttgc ccagctgggg
      841 cgaaggttga cgcatattat ttatgcgccg cggctcggtg aatactatgg tatgcccgga
      901 tcggagatag ggatggaacc ctccgctagc tttgtgattc cggaactcgg aacggaacaa
      961 aaagtcgggg gtaatactac ggaggtctca gatgataaat cactgctcgc tcctgcgcta
     1021 acgcgccccc ctcctggttt gattgagagc aaatgtgtag tgacacatgg tgctgacatg
     1081 gtctgcgacg gctgtgaaaa tgatgagcta ctcgctgcat ttgcagagga agaagcctct
     1141 tccacggtga tattacatac accggaagcc cttggtgaag tgcaagttgt cgctccgcga
     1201 aacaatatgc caaaaagtat accaccggta tttcaaccgg gaccgccacc taggcgtaaa
     1261 ggaggacctc ttacttggta agaatttgct atcgccacgc tcgtgactcc cgcaagggtt
     1321 ataaagtccg taaaaaattt tttggtgacc tctatgatat tgcacatgga gcgatctcct
     1381 ctaccgtttc atcactcgcg cacgaggtct tttttcctca tccagctgct gcacatactc
     1441 tgtgtcatct ggatccaaat cactgacggt gatttctgac tgagaagtat acgcgcctgg
     1501 tagaagacct gaggtctgtg tcacgtacag atctgcctgc tgcagggtta tacctgtcca
     1561 tgtaaaatct gctatattca gatatggtaa cgctgctggt gttgcacctc cctggtactc
     1621 cacatcacac tggagaaaac aatctggcgt tgttgccact gatgatttcc aaatagtgtt
     1681 cgcattaata aacatcccat ttccggctgt gatagctgga ggagtcagag ccttcgaatc
     1741 tgcaagcata tcccactgga gatgaaatcg agaacccaca ggtgtggaaa taggccaact
     1801 gatcctattt tctgttaatg ttaaatcgat agaattagct gctcctgttg ttaacagttg
     1861 agctcgagac gttcctaacg gagcggttgc tgatgctgct gtaagcccat actgagagaa
     1921 aggaataaga tatcctggca cctgttcgat tgctttgaaa caatcgatgt catacgacac
     1981 ccagagttca ccacagttca cattagtacc ctgaaagcct gtggtcgcga tcgaaaatcg
     2041 gcccaaatca tacaaacgaa tatccttacc ggatgggacg tcgaaggctc gaatatatag
     2101 ctcattcact ggcgtttgag ctcgttcaca ttcaatgcca tgcatcatgt tcaccgacgg
     2161 cttacacgag actccgtttt cggtgttctc catctgctgt tttgttgtga agacggtatc
     2221 tgctgaatca taatctgttg acatgatcac ggaaccgaga gcggtatttg ttgagtttaa
     2281 cgcatccgca gacatcgatc gaaactcaaa ggtaagacca ttgattcgat actgctgata
     2341 cttggcagcg actacgccgg ctaaccatgg aaaggtctct tcttgacctg gattaataga
     2401 aaagctattg atcttgaaag tgttcgccgc agacgacgag aacacatcac caagaaattc
     2461 acgatgtttg aatttagtag ctaccttgcc cgttccgaac tgcggtatgt ttgccgaatt
     2521 tgataacgca atgttgtttt ccttcaacgt tcctgcgttt gttacggcat aatctccgtg
     2581 tccgaagatc tttgataaga acccacctcc ggcgttccct atcattccac ccatcgcacc
     2641 tcctgccggt cctcctaagg ccaacccaag ggctgacccg atggctgaac caagggctga
     2701 ccccccgctg ctcttagctg ctctaggacg agctactgct ctgggtctag cacgcgcata
     2761 tccacctaac aattgtgttc ttggattggc tcgagcatat gcaagaccgg ctttcgcctt
     2821 tgcctgcatc gccgctgtgt acgctgacgg acgcttatac cctggtcggg tatagttagc
     2881 aactgcttta cgaggcatgt tggcaacggg tatactaccc tattatattt ttctaatata
     2941 atattatatg ctgcataact ctgcagccat aacatatcta tctcatgctg tcgctctcga
     3001 gtatcccaca ctgacatgat ggttatttat tatttttata ttttattttt tacagtatgg
     3061 gcggcgctac gcaccaccct cctacatgac tgtaatcgac ttccaagtcc aggccttcaa
     3121 gtaggtctgc tcgctacaga acacgtctga tcctagatcc gtcggggcaa caacaccccc
     3181 cttgggtaca tgctatcgca gtagtcttga ctcaccacgc gagtcttggg gcttcgcccc
     3241 ttgcgcctct gtggtgtcgc aagatcattg atagcatggg gcaagccctt gttttcctcg
     3301 ggaccaagcg ttgcatacgg gacatgttcc gtaggtcgga cctattatct gacctgtccg
     3361 gtttggagca atgattaatt attttgtttt agacttctta tcccggatat acatacccgg
     3421 tagtc
//
LOCUS       CruV-255                3426 bp    DNA     circular     24-FEB-2020
DEFINITION  Crucivirus-255 isolate BS_297.
ACCESSION   CruV-255
KEYWORDS    .
SOURCE      Crucivirus-255
  ORGANISM  Crucivirus-255
            Unclassified.
REFERENCE   1  (bases 1 to 3426)
  AUTHORS   de la Higuera,I., Kasun,G.W., Torrance,E.L., Pratt,A.A.,
            Maluenda,A., Colombet,J., Bisseux,M., Ravet,V., Dayaram,A.,
            Stainton,D., Kraberger,S., Zawar-Reza,P., Goldstien,S., Briskie,J.,
            White,R., Taylor,H., Gomez,C., Ainley,D.G., Harding,J.S.,
            Fontenele,R.S., Schreck,J., Ribeiro,S.G., Oswald,S.A., Arnold,J.M.,
            Varsani,A., Enault,F. and Stedman,K.M.
  TITLE     Direct Submission
  JOURNAL   Submitted (24-FEB-2020) Biology, Portland State University, 1719 SW
            10th Ave, Portland, Oregon 97201, USA
COMMENT     Bankit Comment: ALT EMAIL:ide@pdx.edu
            Bankit Comment: TOTAL # OF SEQS:451
            Bankit Comment: GAP: est. length - alignment

            ##Assembly-Data-START##
            Assembly Method       :: MetaSPAdes v. 3.12; IDBA-UD v. 1.1.1;
                                     MEGAHIT v. 1.0.3
            Sequencing Technology :: 454; Illumina
            ##Assembly-Data-END##
FEATURES             Location/Qualifiers
     source          1..3426
                     /organism="Crucivirus-255"
                     /mol_type="genomic DNA"
                     /isolate="BS_297"
                     /isolation_source="Water"
                     /country="New Zealand"
                     /collection_date=2012
     CDS             1..435
                     /codon_start=1
                     /product="putative ORF"
                     /translation="MQSAADMSDDLVIDASQLPYESEMHISYDDFLDDMRALARTFVT
                     LNPEQDPDDPEKVYHQIDDFLERDLGFFTFDENELWMPDDLYYHVFDYIIGNIKGFDE
                     YVDRYMAADTVTALPPIPRNCACEEYFALPDDDRCARCRGYY*"
     CDS             534..1904
                     /codon_start=1
                     /product="Putative CruV Capsid Protein"
                     /translation="MVYKKYSDKQKAAYYKRKASGAAPTYTVRGRGDYKTKASAYYKK
                     AVSNYRKPYRYPGAGAAIGGAIGGAFGHPGIGSAIGAGAHSLMKTVTGYGDYNVAQNS
                     LVYNRDAVPEFTVNNKRCTMISHREFITDISGSIDFEKRVFRINPGVDTTFPWLASVA
                     ENYEQYVVQGMIFEFKTTCATAIGSTNTALGTVVLATQYNSLSPEFQSKQQMENYEFA
                     ISSVPSQSVLHPIECDPTQTQCGGIFNMADPADADGDRRLYDVGRFTIATVGMQAESV
                     IGELWVTYKICFLKPRLRSSDEDAVFLYQNVPGSISTNNPFGNEGDWKFMKPEWDSDL
                     AFVLNSNTIGIRPGFVGVVALNIYWDCTNAVTAANTATTVTGINCTNVTTSVITTVSG
                     QLANYLLPVGSGGNGVLYQTVYIKCIGHDFPHVAFTGFSIAGGACNIVRIQAITLPSE
                     FIDYPY*"
     CDS             complement(2095..3282)
                     /codon_start=1
                     /product="Putative Replication Associated Protein"
                     /translation="MQRNTEVNTELIGNTNTISSIIVSGVNSNSNITSHITNQIQLIN
                     SKYIPIEGPKPIGHIPLTLNIIRQNTTNITNIPTETILETKQETIPEKENTRVNKNFD
                     CKGVRSRSWFLTINNFTEQDVAMIKAEDTVYKVWQFEIGKSGTKHLHCLLYFKNARTW
                     PKKRYPTARIEYPRSLNDCIVYCQKERTRVDGPWSEGTCPCQGRRTDLEELANAIREG
                     TSLRDIATTNGAEYIRYHHGLKALREIYLKPRNWRKKPHVTWIYGASGTGKTRCIFEE
                     FDTGDITTDDIYIKENNKWFDGYQQQKVFLWDEISATGCPLVDFLKIFDRYPCQRETK
                     GGHVHINSGRMFITSSKHPLDMYSSSDNNDVEIMRRLDVIIKINNDESIEIEKDSDYE
                     WED*"
     misc_feature    3223..3256
                     /note="stem-loop"
     misc_feature    3237..3245
                     /note="nonanucleotide"
ORIGIN
        1 atgcagtcgg cagcagacat gtccgacgac ttagtaattg acgctagtca attaccatat
       61 gaatcagaaa tgcatatttc ctacgatgat tttttagatg atatgagggc tcttgcccgt
      121 acatttgtta ctttgaaccc tgaacaggat cctgatgatc ctgagaaggt ctatcaccag
      181 atagatgatt ttcttgaacg tgatttagga ttttttacat tcgatgagaa tgaattgtgg
      241 atgcctgatg atttgtatta tcatgtgttt gattacataa ttggtaatat taagggattc
      301 gacgaatatg tcgataggta catggctgct gatactgtta ctgctctacc acctataccg
      361 cgaaattgcg cgtgcgaaga atatttcgca ttacccgatg acgatagatg tgctagatgt
      421 cgaggatact actagataca gaagttaaca atattaatat ttactattaa tatcattata
      481 ataattaata aatggtctaa acaaaaggtt tactataaaa aacaacacta cgaatggtct
      541 ataagaagta ttcagataaa caaaaagctg catattacaa gcgtaaagct agcggagctg
      601 caccaacgta tactgtacgt ggacgtggtg actataaaac aaaggcaagt gcctattaca
      661 agaaggctgt aagtaattac agaaaaccat acagatatcc tggagccgga gccgcaattg
      721 gcggtgctat cggcggagca tttggacatc caggtattgg atcagctatt ggagctggag
      781 ctcactcatt aatgaagact gtaaccggat acggagatta caacgtcgct caaaactcat
      841 tggtatacaa tcgggacgca gtacctgaat ttacggtgaa caacaagaga tgcactatga
      901 tttcccatcg agagtttata actgacatta gtggttcaat tgatttcgag aagagagtat
      961 tcagaatcaa tccaggagta gatactacat tcccatggtt agcaagcgta gcagaaaatt
     1021 acgaacaata tgtcgtacaa ggaatgatct ttgagtttaa aactacatgc gcaacagcaa
     1081 taggatcaac aaatactgca ttaggaacag ttgtactagc aactcaatat aacagtttat
     1141 cacctgaatt ccaatctaaa cagcaaatgg agaactacga attcgcaatc tcatcagtac
     1201 caagtcagtc tgtattgcac cctatcgaat gtgacccgac acaaacacaa tgtggaggaa
     1261 tctttaatat ggcagatcca gcagatgctg atggtgatcg tagattgtac gatgtaggaa
     1321 ggtttacaat tgcaacagtt ggaatgcaag cagagtctgt tataggtgag ttgtgggtta
     1381 cttacaagat ttgtttctta aagccaagat taagatcatc cgacgaagac gcagttttct
     1441 tatatcaaaa tgtacctggt tctatttcga ctaataatcc atttggtaat gaaggcgact
     1501 ggaaatttat gaaaccagaa tgggattctg atctcgcatt tgtattaaat agtaatacga
     1561 ttggtattcg accaggtttt gtaggcgttg ttgctcttaa tatatactgg gattgtacaa
     1621 atgctgttac agctgcaaat actgctacta ctgtaacagg tataaattgt actaatgtta
     1681 ctacaagtgt tatcactact gtatctggcc aattggccaa ttacctttta ccagttggtt
     1741 caggaggaaa tggggtatta tatcagactg tatatattaa atgtattggg catgatttcc
     1801 ctcacgtagc tttcactggt ttctctatcg caggtggagc ttgtaatatt gttagaattc
     1861 aagcaataac tctcccatca gagtttattg attacccata ttaaataata actatatatt
     1921 agtacaatgt actaatatat ctactaaccg agcgcagctc ggccgtgtcc ggaggagaga
     1981 gcgcagctct ctccgatact gttctagtct tcccattcgt aatcactata tctactaacc
     2041 gagcgcagct cggccgtgtc cggaggagag agcgcagctc tctccgatac tgttctagtc
     2101 ttcccattcg taatcactat ctttttctat ttcgatagat tcatcgttat taattttaat
     2161 gatgacatct aatctacgca ttatctccac gtcgttatta tcactactag agtacatatc
     2221 taatggatgt ttagagctag taataaacat tctaccacta ttaatatgta cgtgaccacc
     2281 ttttgtttct ctttggcatg gatacctatc aaagatttta aggaaatcta ctagcggaca
     2341 acctgtagcg cttatttcat cccacaaaaa taccttttgt tgttgatatc catcaaacca
     2401 tttattattt tctttaatat aaatgtcgtc agtagtaata tcacctgtgt caaattcttc
     2461 aaagatacat ctggttttac cagtacccga agcaccataa atccaagtta catgtggctt
     2521 ctttctccaa ttacgaggtt ttaagtaaat ctcgcgaagc gctttaagtc cgtgatgata
     2581 cctaatgtat tcagcaccgt tagtagtggc aatatcacgt aaactagttc cttctctaat
     2641 agcattggct agctcttcaa gatcagtgcg tcgaccttga caaggacagg ttccttcaga
     2701 ccatggacca tctacacgag ttctctcttt ctggcaatat acaatgcagt catttagtga
     2761 tcgagggtat tcgattctgg cggttggata ccgtttcttg ggccaagttc tagcattctt
     2821 gaagtaaagc aaacaatgta agtgtttagt accactttta cctatttcaa attgccatac
     2881 tttatatacc gtatcttcag ctttaatcat agccacatcc tgttcagtaa aattattaat
     2941 ggtcaaaaac catgatctag atcttacacc cttacagtcg aaatttttat taactctagt
     3001 attctctttt tctggtatag tttcttgttt ggtttcaagt atagtttcag taggaatatt
     3061 agtaatatta gtagtatttt gtctaattat attgagtgta agtggtatat gaccgattgg
     3121 ttttggtcct tcaatgggga tgtatttgct gttaataagt tgtatctgat tagtgatatg
     3181 actagtaata ttgctattac tattaactcc tgacacaatt atagaactta tcgtattagt
     3241 attaccgata agttctgtgt taacttctgt gtttctttgc attttactgc taaatatgca
     3301 acttttagac caaaatacat gttattttgt ctgcattata gcgatagcgt atatattatt
     3361 tatgtgttta gacccatctg taaagatgta taataaatgg tctaaacaga agcattttat
     3421 gttaaa
//
LOCUS       CruV-256                3426 bp    DNA     circular     24-FEB-2020
DEFINITION  Crucivirus-256 isolate 001509_virusbaton.
ACCESSION   CruV-256
KEYWORDS    .
SOURCE      Crucivirus-256
  ORGANISM  Crucivirus-256
            Unclassified.
REFERENCE   1  (bases 1 to 3426)
  AUTHORS   de la Higuera,I., Kasun,G.W., Torrance,E.L., Pratt,A.A.,
            Maluenda,A., Colombet,J., Bisseux,M., Ravet,V., Dayaram,A.,
            Stainton,D., Kraberger,S., Zawar-Reza,P., Goldstien,S., Briskie,J.,
            White,R., Taylor,H., Gomez,C., Ainley,D.G., Harding,J.S.,
            Fontenele,R.S., Schreck,J., Ribeiro,S.G., Oswald,S.A., Arnold,J.M.,
            Varsani,A., Enault,F. and Stedman,K.M.
  TITLE     Direct Submission
  JOURNAL   Submitted (24-FEB-2020) Biology, Portland State University, 1719 SW
            10th Ave, Portland, Oregon 97201, USA
COMMENT     Bankit Comment: ALT EMAIL:ide@pdx.edu
            Bankit Comment: TOTAL # OF SEQS:451
            Bankit Comment: GAP: est. length - alignment

            ##Assembly-Data-START##
            Assembly Method       :: MetaSPAdes v. 3.12; IDBA-UD v. 1.1.1;
                                     MEGAHIT v. 1.0.3
            Sequencing Technology :: 454; Illumina
            ##Assembly-Data-END##
FEATURES             Location/Qualifiers
     source          1..3426
                     /organism="Crucivirus-256"
                     /mol_type="genomic DNA"
                     /isolate="001509_virusbaton"
                     /isolation_source="Water"
                     /country="France"
                     /collection_date=2013
     misc_feature    join(3421..3426,1..17)
                     /note="stem-loop"
     misc_feature    1..9
                     /note="nonanucleotide"
     misc_feature    complement(3..11)
                     /note="nonanucleotide"
     CDS             274..1902
                     /codon_start=1
                     /product="Putative CruV Capsid Protein"
                     /translation="MSGRRKSYARKSTGGGKRFAAPASASRRRTMDRDEPAPRRRRHT
                     VGKTVGGAIGGKLGGPLGRVIGSGLGKAASSVFGKIFGRGAYTVKTNTITNPGSYVID
                     GVDQVPLMHNDMGTTRIRHREYITDIFSTEDFTNLTFAIQPTNSQLFPWLAAAAQNYE
                     QHKWLGVMFEFKSLSANALTSANTGLGSVSLATQYNALNAPFVNKQELLNYQFASSCK
                     PADSMLHPVECDPGQTPNQPLYVRIGSQAAGDARLYDIGVLNLVTQGMQSDGATIGEL
                     WVTYDILLIKPRISSGLGLALRSAFYTYRGIGGGGWVINDDSPMGTEAISSFDSIGLE
                     FEFEEVAGPGIDCTITFPLGCEGLYLVQNTDFGNGTASVGNQTIGTVVYTNCTARSGL
                     YNVTSANTTGSIDVGLNGGVADCIAMSNDNIISIPNPNLRASIKYVSQTGWIMPPSVT
                     GISVGNLVVCQLNNQVAPPAAANTPGHFGLRKRIHEEEKIEREPAPGGRRIVVQDDDD
                     TYEGVGPVPVHVVREDLDEKRVRRIAAPTRVSKS*"
     CDS             1983..3206
                     /codon_start=1
                     /product="Putative Replication Associated Protein"
                     /translation="MYSTMPRVVDSRHRSYCFTAYPEIERPKKSGRFIEIFDWDSADA
                     YLGLEDKVKYLIVGKETCPKTGRQHHQGYVIWENAKTLSACCKLLPEAAHVEPQKARD
                     DFDAAQYCWKDDDLVVEFGVRPEQGKRNDLHGVRKMIEDGHGMRDIVEVCDSYQAIRG
                     GELLMKYKEAQRDWLPEVFWYWGKAGTGKTRLALEKAGPEPWISGRTLKWWYDYDAHE
                     DVIIDDFRGDFCTFHELLRILDRYPYVLETKGGHRQLLARRIWITSCYPPEKVYQSRE
                     DVVQLLRRITRVMEFKDTSSNVPRLPERWEFVDGREVLVSDPNPPGQHDVQPDVDDVK
                     IDAIDDNRERLERLQSKIGALLASGNSAQKLLHTPQRSGVILAPTSPAHLDEPKKKVV
                     RFDWDNVLEDLLDDL*"
ORIGIN
        1 aattaatatt tgtgtgaaaa aagtttatat gccctggcga ttcggcgagc gagatgcgag
       61 ggcatgtatt tatttaatac gccagcacat tatttatact gtgccggcgt ggtgggcggc
      121 acggtgtgta gtaaaaatag tagatattta tttagaatgg aaaaatgttt gcaggtgtga
      181 tgaatgccga gccggcaact atacacccgc attgattaat attattttat attagagaat
      241 atctggctac gtatatccct ttgtcgcgct gacatgtcag gtcgtcgaaa gagctacgcc
      301 aggaaatcca cgggtggggg taagcggttc gctgctcctg cttcggcatc tcgccgacga
      361 accatggacc gcgatgagcc tgcacctaga cgcagacggc ataccgtcgg taagacggtg
      421 ggcggcgcca tcggcggtaa acttggtgga ccactcggac gagtgatcgg aagtggtctt
      481 ggtaaggctg catcctcagt cttcgggaag atcttcgggc gcggcgccta cacggtcaag
      541 accaacacga tcacgaaccc aggttcgtat gtgatcgatg gtgtggatca agtacccttg
      601 atgcacaatg atatgggaac tactcggatt cgacaccgcg agtacatcac ggatatcttc
      661 agcaccgagg acttcacgaa tttgacattc gctatccaac caaccaacag ccagctgttt
      721 ccttggttgg ctgctgctgc gcagaactac gagcagcaca agtggctcgg agttatgttc
      781 gagttcaaga gcttgtcggc caatgctctg acgtcggcca acactgggtt gggatctgtg
      841 tcactcgcga cacagtacaa cgcactgaac gctccgttcg tgaacaaaca ggagctcctg
      901 aattaccagt ttgcatcttc atgcaaaccg gctgattcca tgcttcatcc ggtggaatgc
      961 gatcctggac aaacgcctaa ccagcctttg tacgtgcgga tcgggtcgca agctgcaggc
     1021 gatgctcgcc tgtatgacat tggagtgcta aaccttgtta cacaaggtat gcagagtgac
     1081 ggtgccacca tcggcgagct atgggtcacc tatgacatcc tgctgatcaa gcctcggatt
     1141 tcatccgggc ttggactagc attacgctcc gcgttttaca cctaccgagg aatcggtggt
     1201 ggagggtggg ttattaatga tgactcgcca atgggaactg aggccatcag ctcgtttgac
     1261 tccattggac tggagtttga atttgaggag gtggcgggcc ctgggatcga ttgtacgatc
     1321 acgttcccgc tgggctgcga aggtttgtac ctcgtacaga acactgactt cggcaacggg
     1381 acagcctcgg ttggtaatca aacgattgga actgttgtgt acacgaattg tactgcccgg
     1441 tccgggcttt acaacgtgac cagcgccaac acgacagggt cgattgacgt cggtttgaat
     1501 gggggcgtgg ctgattgcat tgcaatgtcg aatgacaaca tcatttcgat cccgaatccg
     1561 aatttgaggg caagcatcaa atacgtctcg cagactggtt ggattatgcc accctcggtc
     1621 acagggatct cggttggtaa tcttgttgtt tgccagctga acaaccaagt tgctccgccg
     1681 gcagcggcga atacgcctgg acacttcggt ctgcgcaagc gcattcacga agaggaaaaa
     1741 attgaacgag aacctgcgcc tggtgggcga aggattgtcg ttcaagatga tgatgacacg
     1801 tacgaaggtg tgggaccggt tccggtccat gtcgtacgtg aagatctgga tgaaaaaagg
     1861 gtgcggcgca ttgctgcacc gacccgggtc tccaagtcat agcatgctca atagcatgat
     1921 gaccatgtcc gctccacttg gagcctgaca ttattttgtg atagtgcggt gtttggcgcg
     1981 acttgtatag cacgatgcca cgagtcgttg actctcgtca taggtcgtat tgctttacag
     2041 catacccaga gattgagagg ccgaaaaaat ctggaagatt tattgagatc tttgactggg
     2101 actctgcaga tgcctatctc ggactcgaag ataaggtaaa ataccttatt gtgggtaaag
     2161 aaacctgccc gaaaacgggc agacagcatc atcaaggtta tgtcatctgg gagaatgcaa
     2221 aaaccttgag cgcttgctgt aaacttctac ctgaggctgc tcatgtagaa ccgcagaaag
     2281 ctcgcgacga cttcgacgct gcgcagtact gctggaaaga tgatgacctt gtcgtggagt
     2341 tcggagtacg tccagaacaa ggtaaaagaa acgatcttca tggcgttcgc aagatgatcg
     2401 aagatggaca tggcatgagg gatatcgtgg aagtgtgtga cagctaccaa gcgattcgcg
     2461 ggggcgaact cctcatgaag tacaaagaag cccaacgtga ctggcttccc gaggtcttct
     2521 ggtactgggg taaagccggc acgggtaaaa cccgcttggc tttagaaaaa gctggacccg
     2581 agccgtggat ctcgggtcga acgcttaaat ggtggtacga ctatgatgca catgaggatg
     2641 tgatcataga tgatttccgt ggtgatttct gtacctttca cgagctcctt cggattctcg
     2701 accggtaccc ttacgtcctt gagacgaaag gtggccatcg ccagcttctg gctcggcgga
     2761 tttggatcac gagctgctat ccacctgaga aggtgtatca atctcgtgaa gacgtggttc
     2821 agctcctgcg tcgcataacc cgtgtgatgg aattcaaaga cacgtcttcg aatgtcccac
     2881 gtcttccaga gcgatgggag tttgttgatg ggcgagaggt tctggtctcc gacccgaacc
     2941 cgcctgggca gcacgatgtg cagcccgatg tcgatgacgt caagatagac gcaatcgacg
     3001 acaaccgcga gcggctagaa cggctccaat caaaaatcgg agccttacta gcttctggca
     3061 attctgcaca gaagttgttg cacacaccac agaggtcggg ggtaatacta gccccgacct
     3121 cgcctgccca ccttgacgaa cctaaaaaga aggtcgtcag gtttgattgg gataacgtcc
     3181 tcgaggattt actcgacgac ttatagccct ataagggcta tgtttttggg tgattgcttg
     3241 gatcataaag tgtgttgatc cacataatga cccgctacag gtcgtaacct tcaggcccgg
     3301 gttatacaag tataccgggc ctgccggtta cgacccggtt gcagttaatt atgttacagc
     3361 aaccagaggt tatatttttg ggttaacaaa aaaataatca ccctatataa ttgaacgcta
     3421 tcgcgc
//
LOCUS       CruV-257                3434 bp    DNA     circular     24-FEB-2020
DEFINITION  Crucivirus-257 isolate 120_865_SvL1_3295.
ACCESSION   CruV-257
KEYWORDS    .
SOURCE      Crucivirus-257
  ORGANISM  Crucivirus-257
            Unclassified.
REFERENCE   1  (bases 1 to 3434)
  AUTHORS   de la Higuera,I., Kasun,G.W., Torrance,E.L., Pratt,A.A.,
            Maluenda,A., Colombet,J., Bisseux,M., Ravet,V., Dayaram,A.,
            Stainton,D., Kraberger,S., Zawar-Reza,P., Goldstien,S., Briskie,J.,
            White,R., Taylor,H., Gomez,C., Ainley,D.G., Harding,J.S.,
            Fontenele,R.S., Schreck,J., Ribeiro,S.G., Oswald,S.A., Arnold,J.M.,
            Varsani,A., Enault,F. and Stedman,K.M.
  TITLE     Direct Submission
  JOURNAL   Submitted (24-FEB-2020) Biology, Portland State University, 1719 SW
            10th Ave, Portland, Oregon 97201, USA
COMMENT     Bankit Comment: ALT EMAIL:ide@pdx.edu
            Bankit Comment: TOTAL # OF SEQS:451
            Bankit Comment: GAP: est. length - alignment

            ##Assembly-Data-START##
            Assembly Method       :: MetaSPAdes v. 3.12; IDBA-UD v. 1.1.1;
                                     MEGAHIT v. 1.0.3
            Sequencing Technology :: 454; Illumina
            ##Assembly-Data-END##
FEATURES             Location/Qualifiers
     source          1..3434
                     /organism="Crucivirus-257"
                     /mol_type="genomic DNA"
                     /isolate="120_865_SvL1_3295"
                     /isolation_source="Water"
                     /country="Norway"
                     /collection_date=2012
     CDS             1..1206
                     /codon_start=1
                     /product="Putative Replication Associated Protein"
                     /translation="MAVRPPPETKRSRSWVYTYFDYTEDTLEHLRTLDCVTYHVFGKE
                     VCPDTGRLHLQGYIEFKDAKPLGGVKKTLKNKKLHCENRRGTPEQASTYCKEDGDFWE
                     VGELPVKGKRNDLIGALALLRSEGIRSVALEMPMQYMLHHKALEAYKGLTTDHRKTRP
                     KVVWLWGLAGTGKSRTAKNCESYYLWAGGRFWCNYQNEQRVVIDDFSYDHAARESEHY
                     YRSLLRLMDFGQHQVEVKGSFAVFNSPEIYITCEFPPEHFWPATDVYGEVNNRHTQIM
                     RRLYACQEITQYKPDFVPTPLPHVGPDFFHPRAQEDEDLPDEDVSDSGTEESRRTDFT
                     FDSEEAAEAYLEQCVLEVARADDELHWVKCSNKHNHPACFDGINCPECMYPISQAERM
                     AIMDSRYID*"
     CDS             complement(1499..1801)
                     /codon_start=1
                     /product="putative ORF"
                     /translation="MYKKVFFVEFRARQRHGFYSIPYGYPNTPRREKPRVAGPQTRPI
                     CPLRLLKRAVREKLVDSDMEFSWRVLPVNLRVKNLQRVEAGRTGRSPGYNAIAVTP*"
     CDS             complement(1820..3223)
                     /codon_start=1
                     /product="Putative CruV Capsid Protein"
                     /translation="MPRAATAKRSKAATKHAYAKRTAVVGRGAYRKATATRAPARAKG
                     MRYPGAGAAIGSTIGSAFGPAGSAIGGLLGGAAHKLAHSIFGFGDYQVKRNSLILEET
                     NGPPMVMNGGKEFIVRHREYLTDVYSAAGTANSPSPFALQSFPINPGNFVTFPWLASI
                     ADKFEQYRIEGMVFEYKSNYSDAVVTNSGSLGTVILATEYNSGSPSFTSKQAMENYQF
                     AQSAKPSLSILHPIECARSQNVLTELYVRPGGVPAGEDVKTYDFADFQIASQGIPLGS
                     TGAAQNMGELWISYQLALIKPKIPTTAASYVDSGFAAFSTKTGSAPFGATAMPINNVS
                     RLTPSNIEVYVTADNTFTLQLGSTPMKYQIDAHWKAFSDTYSALDAWKAPTLGFTNAS
                     SVNSGSSGVYINELIPLRSPGSNGTGCSTTYFLQVPAATPSAPTATLVWANFLADQVP
                     AVRFDCYWNAVPINLVA*"
ORIGIN
        1 atggccgtcc gtcctccacc ggagacgaag cgatcccgct catgggtcta cacttatttc
       61 gactacactg aagacacgct cgagcacctc cgtacactcg attgcgtgac ctatcatgtc
      121 tttggtaaag aagtctgtcc cgacaccggc cgtctgcatt tacagggtta tatcgagttc
      181 aaagacgcta agcctctcgg cggcgtcaag aaaactctca agaacaaaaa gctgcactgc
      241 gagaaccgtc gtgggacacc tgaacaagca tccacttact gcaaggaaga tggagacttc
      301 tgggaagtcg gcgagctgcc tgttaaggga aaacgtaacg atctgattgg agcgctagca
      361 ctactcagaa gcgaaggaat tcgttctgta gcgcttgaga tgcctatgca gtatatgctg
      421 caccataagg ctttggaagc ttacaaaggc cttaccactg accaccgcaa aacacgacct
      481 aaagtggttt ggctatgggg tctagctggt actggcaaaa gtaggaccgc aaaaaattgc
      541 gagtcatact atttgtgggc tggcggacgt ttctggtgta actaccaaaa cgaacaacga
      601 gttgttattg atgacttctc ctacgaccac gctgcaagag aatcggagca ctactaccga
      661 tctctcttac gcctcatgga cttcggtcaa caccaggtcg aagttaaggg ttcattcgcc
      721 gttttcaact caccggagat ctacatcacc tgcgaattcc cccctgaaca cttctggcct
      781 gctactgacg tgtacggaga ggtcaacaac cgacacactc aaatcatgcg tagattatat
      841 gcgtgtcaag aaattactca gtataagccc gacttcgtcc ctacacctct acctcacgta
      901 gggcccgact tctttcaccc ccgagcacaa gaagatgagg acctacctga cgaggatgta
      961 tccgatagcg gtactgagga aagcagaagg actgacttca cattcgactc tgaagaggcc
     1021 gctgaggcct atctagagca atgtgtgcta gaagtcgcaa gagctgatga tgaactccac
     1081 tgggttaagt gctctaacaa gcacaaccac ccagcatgct tcgacggcat aaactgcccc
     1141 gaatgcatgt acccaatctc tcaagctgag aggatggcta ttatggattc acgctacatt
     1201 gattgagttg aacctctaca aacctttttt gtatatctcc agcaccagaa acaatatcgt
     1261 tagggctacg cccctttgga tatcgtctgg gtgccgggaa ggtaacactc cgcgccgaga
     1321 gaaacccaga gtggccggac cccaaacccg tccaatctgc cccctcatga tattcggtgg
     1381 tcggtacgtc ttccttcgga acagtgtccc actaaagcgg ggccctactt agaccgcgag
     1441 aatgttactc gccatttacc gctccctcac ctccgtgcat ttacacaatg cacaaaggtt
     1501 acggtgtaac ggcaatggcg ttatatccgg gacttcgccc cgtccttccc gcctcaaccc
     1561 tctgcaaatt ttttacacgc aggttgacag gtaatactcg ccatgaaaat tccatgtcgc
     1621 tgtcaactag tttttcccgt accgcccttt ttagtaagcg gagggggcag attggacggg
     1681 tttggggtcc ggccactctg ggtttctctc ggcgcggagt gttaggatat ccatatggta
     1741 tcgagtagaa accgtgtcgt tgtcgcgccc gaaactccac aaaaaagact tttttataca
     1801 ttatgacaag ctagagagct tatgcgacga gattgatggg gactgcattc cagtagcaat
     1861 cgaagcgcac agcgggcact tgatcagcaa ggaagttagc ccataccaac gtagcagtag
     1921 gtgcagacgg agtcgctgca ggcacttgca agaagtaagt agtggaacaa ccagttccat
     1981 tggatccagg ggatctaaga gggataagct cattgatgta cacaccagag gaaccagagt
     2041 tcacgctaga cgcgttggta aatccaagag taggagcttt ccatgcatcc agtgcactat
     2101 aagtgtcact gaaggccttc caatgagcat caatctgata cttcatggga gtggagccta
     2161 attgcaatgt gaacgtatta tcagccgtca catacacctc gatattcgag ggagtgagcc
     2221 tagaaacatt gttaataggc attgcagtcg ctccgaacgg tgctgagccc gtttttgtcg
     2281 agaacgctgc gaaaccagag tcgacgtaac tagccgccgt tgtaggaatc ttaggcttaa
     2341 tcagagctaa ctgataagag atccacaact cgcccatatt ctgggctgct cccgtagacc
     2401 ccagaggaat gccttgagag gcgatctgga agtctgcgaa atcgtacgtt ttcacgtcct
     2461 caccggctgg tacaccacct ggtcgaacat acaactcggt aagcacattt tgtgacctag
     2521 cacactcaat ggggtgcagt atcgacaatg acggcttggc ggactgggcg aactgatagt
     2581 tctccatagc ttgcttgcta gtaaacgagg gtgaacccga gttatactct gtagccagga
     2641 taaccgttcc taaagatcca ctattggtga ctacagcatc ggagtaattg ctcttgtact
     2701 caaagaccat tccttcaatc cgatactgct caaatttatc agcaatcgac gctaaccatg
     2761 ggaaagttac gaaatttcca gggttgatgg ggaaagactg caacgcgaag ggtgaaggcg
     2821 agttagctgt accagctgcc gagtacacat cggtcaagta ctcacgatga cggacgatga
     2881 actcttttcc tccattcatg accattggag gaccgttggt ctcctcaagg atcagtgagt
     2941 ttcttttgac ctggtaatca ccaaaaccga aaatagagtg agccagcttg tgagcagcac
     3001 cccccaagag accaccgata gcagatcctg cgggtccgaa ggcggaacca atggttgatc
     3061 caatggccgc tccggcgcct ggataacgca ttcccttagc acgtgccggt gccctagtcg
     3121 ctgtagcctt gcgatacgcc cctcttccga cgaccgcagt ccttttcgca taagcatgtt
     3181 ttgttgccgc cttcgaccgc ttagcagtgg ccgcacgagg catgttgacg aataatctaa
     3241 caggattaat aatctaatta attatataat taccggataa taagtccgat tgacacccac
     3301 tttcgggggt tatttattta tttccggcgc cgaaaaccgt cgatttcaga cttttcccgg
     3361 agaaaataat taactcagcc aataaatatt atcctcctag aggttattat gtgagatata
     3421 ttcgactacg cacc
//
LOCUS       CruV-258                3435 bp    DNA     circular     24-FEB-2020
DEFINITION  Crucivirus-258 isolate 120_1340_IR2_3297.
ACCESSION   CruV-258
KEYWORDS    .
SOURCE      Crucivirus-258
  ORGANISM  Crucivirus-258
            Unclassified.
REFERENCE   1  (bases 1 to 3435)
  AUTHORS   de la Higuera,I., Kasun,G.W., Torrance,E.L., Pratt,A.A.,
            Maluenda,A., Colombet,J., Bisseux,M., Ravet,V., Dayaram,A.,
            Stainton,D., Kraberger,S., Zawar-Reza,P., Goldstien,S., Briskie,J.,
            White,R., Taylor,H., Gomez,C., Ainley,D.G., Harding,J.S.,
            Fontenele,R.S., Schreck,J., Ribeiro,S.G., Oswald,S.A., Arnold,J.M.,
            Varsani,A., Enault,F. and Stedman,K.M.
  TITLE     Direct Submission
  JOURNAL   Submitted (24-FEB-2020) Biology, Portland State University, 1719 SW
            10th Ave, Portland, Oregon 97201, USA
COMMENT     Bankit Comment: ALT EMAIL:ide@pdx.edu
            Bankit Comment: TOTAL # OF SEQS:451
            Bankit Comment: GAP: est. length - alignment

            ##Assembly-Data-START##
            Assembly Method       :: MetaSPAdes v. 3.12; IDBA-UD v. 1.1.1;
                                     MEGAHIT v. 1.0.3
            Sequencing Technology :: 454; Illumina
            ##Assembly-Data-END##
FEATURES             Location/Qualifiers
     source          1..3435
                     /organism="Crucivirus-258"
                     /mol_type="genomic DNA"
                     /isolate="120_1340_IR2_3297"
                     /isolation_source="Water"
                     /country="Norway"
                     /collection_date=2011
     CDS             1..1314
                     /codon_start=1
                     /product="Putative Replication Associated Protein"
                     /translation="MCTFYLKIHLYKRVVKHVYHVKMQLDPANNYAPIDNLDGRSRAK
                     HWCFTVNNPTVQDVSMFTDITILTTYYVYGEEVADSGTQHLQCYICFDKATTLKACKG
                     FWPRAHFEVMRGTPLQAADYCKKDGKFKEHGILPKAKNIAGGEATKAKWIEISTAATE
                     NNLQLIQTKHPKEFVSNYRSLKQISFDFQPPMPDLDGDLDNLWLYGDSGVGKSMSVRE
                     EYGEDLYYKMCNKWWDNYEGEENVLIDDLDLTHSVIGHHIKIWSDRYAFRAEVKNHSK
                     MIRPKRIIITSQYHPNEIWSDKKLVDAISRRFKITQMLKLPDADPLISKKSTSKIVSA
                     PIKIKKHDRPYMKETDPTVKKKLRFTLNRSTTQSKIDKPPPMKPAFEFIRPITIDLTS
                     ESEEEEQEVYDSSLPDISEDEGSNHEDWDAQDAYREFISVNCNIK*"
     CDS             1807..3285
                     /codon_start=1
                     /product="Putative CruV Capsid Protein"
                     /translation="MFMQMSKRPSPNAIRPRAQSKYGQAGAKKVYPKGPIPYGVSRPN
                     AYWRIKKDLPAQDYWKQRYWRRRITGKGSYSSPRTMRGRGSYTGIGRRVGEYIGGYLG
                     EGAENLATSFITGLGDYSIRRNVFMAGSLPEVVNPTGPTGPGGTCIRYSEYLGDIITG
                     TSNTFDVQNYIINAANSRTFPFLSQIASNYEQFEIEGMLFEFKSTSADALNSTNTALG
                     TVMMATEYDVLDKAFSSKGEMLNYQYATCIKPSESTMHMVECDPRQTTTNLLYCLPSN
                     STVPVDVPDKADPRLYYLGNFFIATSGFQASNITIGQLHVTYQVRLLKPKLVSTIGED
                     FPLFRRFLTGGTDFPLWTNASPFGVFGTFETDIMNPATEFNSASIGVGGAIIAFNDNA
                     MPRTYLINIDWNAEVSTDPISLATFTTTNCTINEVTIAPSAIVSGIASMNMIVSTNGN
                     NITAAIQAGAGFNLPESTEAVPGSYVITITQVDPDILKVLVS*"
ORIGIN
        1 atgtgcactt tttatttgaa aatccacctt tataaacgtg tcgtaaaaca cgtttatcat
       61 gtcaaaatgc aattagatcc tgctaacaac tacgctccaa tagataactt agatggaaga
      121 tcacgcgcaa aacattggtg ttttacagtc aataatccca cagttcagga tgttagcatg
      181 tttacagata ttacaatcct taccacatac tatgtttatg gagaagaagt tgctgattct
      241 gggactcaac acttacaatg ttatatctgc tttgataaag ccactactct taaagcctgt
      301 aaaggattct ggcctagagc ccactttgaa gtcatgcgag gcactccttt acaagcagct
      361 gattattgta aaaaagatgg aaaatttaaa gagcatggga ttttaccaaa agccaaaaac
      421 attgctggag gagaggcgac gaaagctaaa tggatagaga tttcaactgc tgccacagaa
      481 aataatctcc agttaattca gactaaacat ccaaaagagt ttgtatctaa ttacagatca
      541 cttaaacaaa tctcttttga ctttcaaccg cctatgccag acttggatgg agatttagat
      601 aacctgtggc tatatggaga ctctggcgtt ggtaaatcaa tgtctgtcag agaagaatat
      661 ggagaagacc tttattacaa gatgtgtaat aaatggtggg ataattatga aggcgaagaa
      721 aatgtactta ttgatgatct tgatcttaca cattctgtca ttggacatca tatcaagatc
      781 tggtctgatc gctatgcatt ccgagccgaa gttaaaaatc attccaagat gatacgtcca
      841 aaacgtatca ttattacttc gcaatatcat ccaaatgaga tttggtctga caaaaaatta
      901 gttgatgcta tatctcgtag atttaaaatt actcagatgc ttaaacttcc cgatgctgat
      961 cctttaatct ccaaaaaatc tacttcaaag attgtaagtg ctccaattaa aatcaaaaaa
     1021 catgataggc cttatatgaa ggagactgat cctactgtaa agaagaaact ccgttttact
     1081 ctcaatagaa gtacaactca atctaagatt gataaacctc caccaatgaa acctgctttc
     1141 gaatttataa gaccaattac aattgatcta acatctgaat ctgaagaaga agaacaagaa
     1201 gtttatgatt catcattacc tgatatctct gaagatgaag gatctaatca tgaagactgg
     1261 gatgcacaag atgcatatcg tgaatttata agtgtaaact gtaacattaa ataaaaatat
     1321 aacccgttag ggtttacacg attgagatgt atgagactag ttagtccacg ggaccaagtg
     1381 tttgttaaac acgttaggtg ttgatacgaa tactcttcaa gcgtgggccc taacggccca
     1441 gagcgtaacg aataataaca gcacagcgca gcgtacccac ccatttaggg cgcgccgcag
     1501 gcataggtca tgtcatcttg tcccgtctac tacattaggg taaacaatta cccgtctact
     1561 acattagggt gaacaatcac ccgtctacta cattagggtg aacaatcacc cgtctataac
     1621 taaaacaaac acgagtttca ctcgagtgtt tgtccatcgg taccaagtaa ataattaact
     1681 atataatatc agccctctaa gccgaccgtg cgcagcagag gtcgtagtat tacccttaga
     1741 gggcgttaat ccttttaggg tgagttcact acactattta aaggttaacc agcacgtgct
     1801 gcacatttgt ttatgcaaat gagtaagcgc ccatcaccta acgcaatccg ccctagagct
     1861 cagtccaagt atggacaagc aggggcgaaa aaagtttatc ccaaaggtcc aataccttat
     1921 ggtgtatcca gacctaatgc ttactggcgg atcaaaaaag atctgccagc acaagattac
     1981 tggaaacaac gctattggcg acgtcgaata actggcaagg gcagttattc ctctcctcgt
     2041 acaatgcgtg gcagaggcag ttatactggt attggacgcc gagttggcga atacattgga
     2101 ggttatctcg gagaaggagc agaaaattta gctacttctt ttatcaccgg tctaggtgat
     2161 tattctatac gtaggaatgt atttatggca gggtcattac ctgaagttgt taatcctact
     2221 ggacctacag gaccaggagg tacatgtatc aggtattccg agtatctcgg tgatattatt
     2281 actggtacat caaatacttt tgatgtacag aattatatta tcaacgctgc gaattctcgt
     2341 actttcccat ttctctcaca aatcgcgtcc aattatgaac aatttgaaat agagggaatg
     2401 ctatttgaat ttaaatcaac atctgccgat gccttaaatt caaccaatac tgctttaggt
     2461 actgttatga tggctacaga atatgatgtc ttggacaaag cattctcctc aaaaggagaa
     2521 atgcttaatt accaatacgc tacttgtatt aagcctagcg aatcaactat gcatatggta
     2581 gaatgtgatc caagacaaac tacaacaaat ttgttgtatt gtctaccttc aaactccaca
     2641 gtacctgtag atgtgccaga taaagctgat cctagattat actatctagg taatttcttt
     2701 attgcaacat ctggttttca agcgtccaat ataactattg gacagcttca tgtaacttat
     2761 caggttaggt tactaaaacc taaacttgta agtactattg gtgaggactt tcctttattc
     2821 aggaggttcc tcacaggagg taccgacttt cctttatgga caaacgcttc accatttggt
     2881 gtatttggaa cctttgagac tgatattatg aatcccgcaa cagagtttaa ttcagcctcc
     2941 attggagtag gtggtgctat tatagctttt aatgataatg ctatgccacg tacttatctc
     3001 attaatattg attggaatgc tgaagttagt acagacccaa ttagtctggc tacttttact
     3061 acaacaaatt gtactattaa tgaagttaca atagctccat cagctattgt tagtggtata
     3121 gcatcaatga atatgattgt gtccacaaat ggaaacaata ttactgctgc tatccaagct
     3181 ggtgctggtt ttaatctccc agagagtact gaagctgtac ctggatctta tgttattaca
     3241 ataacacaag tagatccaga tatacttaaa gtattagtat cataacgaac cctaatttta
     3301 cgcccttata gttaattata atttctatta ataattaata ttataataat aataataata
     3361 ttataataat tgtaataaat aataaaaatt atttttaaaa taccgaattc catttctaaa
     3421 aacgtgcaaa agtgc
//
LOCUS       CruV-259                3437 bp    DNA     circular     26-FEB-2020
DEFINITION  Crucivirus-259 isolate 001503_virusbaton.
ACCESSION   CruV-259
KEYWORDS    .
SOURCE      Crucivirus-259
  ORGANISM  Crucivirus-259
            Unclassified.
REFERENCE   1  (bases 1 to 3437)
  AUTHORS   de la Higuera,I., Kasun,G.W., Torrance,E.L., Pratt,A.A.,
            Maluenda,A., Colombet,J., Bisseux,M., Ravet,V., Dayaram,A.,
            Stainton,D., Kraberger,S., Zawar-Reza,P., Goldstien,S., Briskie,J.,
            White,R., Taylor,H., Gomez,C., Ainley,D.G., Harding,J.S.,
            Fontenele,R.S., Schreck,J., Ribeiro,S.G., Oswald,S.A., Arnold,J.M.,
            Varsani,A., Enault,F. and Stedman,K.M.
  TITLE     Direct Submission
  JOURNAL   Submitted (24-FEB-2020) Biology, Portland State University, 1719 SW
            10th Ave, Portland, Oregon 97201, USA
COMMENT     Bankit Comment: ALT EMAIL:ide@pdx.edu
            Bankit Comment: TOTAL # OF SEQS:451
            Bankit Comment: GAP: est. length - alignment

            ##Assembly-Data-START##
            Assembly Method       :: MetaSPAdes v. 3.12; IDBA-UD v. 1.1.1;
                                     MEGAHIT v. 1.0.3
            Sequencing Technology :: 454; Illumina
            ##Assembly-Data-END##
FEATURES             Location/Qualifiers
     source          1..3437
                     /organism="Crucivirus-259"
                     /mol_type="genomic DNA"
                     /isolate="001503_virusbaton"
                     /isolation_source="Water"
                     /country="France"
                     /collection_date=2013
     misc_feature    join(3428..3437,1..17)
                     /note="stem-loop"
     misc_feature    1..9
                     /note="nonanucleotide"
     CDS             563..1735
                     /codon_start=1
                     /product="Putative Replication Associated Protein"
                     /translation="MQEQNDNSNNQQDLQITELKKSFLSQQGQRWTLTIWDKQECQNL
                     VDHKNTAYFQYGIEICPTTGKQHFQAYVELIKKQTAGQLKKIIPTAHILLSRGTEQHN
                     NIYTSKDGNVFKYGTPIKKGKRTDIEEIKQSIVQQIEDGVSIADILRNDHSNFRYINM
                     IEKCVVHLDQRRRTQAPDVFVYWGKSGCGKTHQAQQYSPNYYIVSKPSNNGILWFQGY
                     DNTKHDTIIFDEFTGWITFTDFKLLTDKYPCQVQTKGSSVQFLAKTIIFTSNKHPKQW
                     YDLQNPIDKVAFQRRIKKCKYFDVVHESVQQKYEDDTPQHILDEIDGIQTSQEDKENF
                     EMAGIESEQHQNNEIFKKYQQRQEEILTQTVIDKVNELMNQKNNMTIQECYDKLKI*"
                     /modified_by="nacho"
     CDS             1716..3047
                     /codon_start=1
                     /product="Putative CruV Capsid Protein"
                     /translation="MINLRYENIYLLFIIFFFQNCLCLKKAMPRIANKNKKSQPKQKI
                     VYVEKPVYKKKQYKERPKSARGPMQTIGETIGGYFGDTGRSVGKYLGKAAGVITGSGS
                     YQIHKNSLMNKQVPFMHSSDEVIRIRHREFLGNIKGSILYENQSYNINPGLKNSFPWL
                     YTTAQSFEQYKIEGLIVQFISTCGDAIASTNNSLGTVCLSTDYNVVNEDFQTMAQCQN
                     SMWSNTAKPSQNILMPIECDGKLNVLERLFLRDEFTVLSNTDLRLYDHCKINLSTEGM
                     QVATQIGQLWLSYDICFYKPVLHNPLSQNAHYLIPATTSGLLYGAVQKVNNIGVYFNQ
                     SLNANRLYFGKALVGKKVMVYYYQYASSGTYNWLDLSYSTSGCTPNNVLLNGIANALD
                     VPFSAGALAALTSGPSLLLSFNINYETDYVTFSGGIISSTVAAELMVWIYT*"
                     /modified_by="nacho"
ORIGIN
        1 tagtattacc ccccaaccta ggatattttt taaactaata caatattcga cggtttgaag
       61 tgtttaacat acaataatca attatcttta attgttgagc ctaaaaggcc taggatattt
      121 ttattttaaa cactttattg tttttaccat gggtcgaccc ctgtggggtg ggtcccagga
      181 cgaacaacaa ctaactcatt tatcgaccag gagctgtaag cccctccgca ggcggtacat
      241 tattttgttt aacacttttt attttaaaca ttttttattt ttaatagatg ggtcgcccct
      301 tggggggggt cccaggacaa ataacacttt taatatatat tatataaatg tatcgtctat
      361 atacctagga ggaacagctg taagcccctg ttccattata tacacataca tttagaccaa
      421 tactaatata taataaaata aacattaaat aaaaaatgta catatacaaa attaaagata
      481 attaataaat taaatatcct caacataata tttttatttt ttatttttta tttaaatatt
      541 tcttttactg tataaaaaaa taatgtaaga ataaaatgat aattcaaata atcaataaga
      601 tttataaatc actgaattaa aaaaatcatt tttatcttaa taaggttaaa ggtggacact
      661 cactatatgg gataaataag aatgttaaaa tttagttgat cataaaaata cagcttattt
      721 ttaatatggt attgaaattt gtccaactac tggtaaataa catttttaag catatgttga
      781 attgattaaa aaataaactg ctggataatt aaaaaaaatt attcctacag ctcatatatt
      841 attatctaga ggaacagaat aacacaataa tatatatact tctaaagatg gaaacgtttt
      901 taaatatggt actcctatta aaaaaggaaa aagaacagat atagaagaga taaagtaatc
      961 tattgtttaa taaatagagg atggtgtgag tatagcagac attttacgta atgatcatag
     1021 taattttaga tatataaata tgattgaaaa atgtgtcgta catttagact aaagaagacg
     1081 aacataagca cctgatgttt tcgtatattg gggtaaatct ggatgtggaa aaacacatta
     1141 agcctaataa tacagtccta attattatat agtgtcaaaa ccttcaaata atggtatatt
     1201 atggttttaa gggtatgata atactaaaca tgacacaata atatttgatg aatttacggg
     1261 atggattaca ttcactgatt ttaaactttt aactgataaa tacccttgtt aagtttaaac
     1321 aaaaggatca agtgtttaat ttttagctaa aactattata tttacatcca ataaacaccc
     1381 taaataatgg tatgatttat aaaatccaat tgataaagta gctttttaac gtaggattaa
     1441 aaaatgtaaa tattttgatg ttgtacatga gtcagtgtaa taaaaatatg aagatgatac
     1501 accataacat atattagatg aaatagatgg aatataaact tcataagaag ataaagaaaa
     1561 ttttgaaatg gcaggtattg aaagtgaata acatcaaaat aatgaaatat ttaaaaaata
     1621 ttaataaaga taggaagaaa ttttaacata aacagtaatt gataaagtaa atgaattaat
     1681 gaattaaaaa aataacatga caatataaga gtgttatgat aaacttaaga tatgaaaata
     1741 tatatttatt atttattatt tttttttttt aaaattgttt gtgcttaaaa aaagcaatgc
     1801 caagaatagc aaacaaaaat aaaaaatcat aaccaaaata aaaaatagtt tatgtcgaaa
     1861 aacctgttta caaaaaaaaa taatataaag aaagacctaa aagtgcaaga ggtccaatgt
     1921 aaacaatagg tgagacaatc ggtggatact tcggagacac tggaagatct gtggggaaat
     1981 atttaggaaa agcagctggt gtaattactg gtagtggatc atattagatc cataaaaatt
     2041 cattaatgaa taagcaagtt ccatttatgc actcatcaga tgaagtaata agaattagac
     2101 atagagaatt cttaggaaat attaaaggtt caatccttta tgaaaattaa agttataata
     2161 ttaacccagg gttaaaaaat tcattccctt ggttatatac aactgcttaa tcctttgaat
     2221 aatataaaat agagggatta attgtataat ttatatcaac atgtggtgac gctatagcat
     2281 ctacaaataa tagtttaggt actgtatgtt tatcaactga ttataatgtt gtaaatgaag
     2341 atttctaaac aatggcataa tgttaaaact caatgtggtc aaatacagca aaaccatcat
     2401 aaaacatttt aatgcctata gaatgcgacg gaaaattaaa cgtattagaa agattattct
     2461 taagagatga atttacggta ttatcaaata cagatttaag actatacgat cattgcaaaa
     2521 taaatttatc aactgagggt atgtaagtag caacataaat tggttaatta tggttatcat
     2581 atgatatctg tttttataaa cctgtattac ataacccatt atcataaaat gcacattatt
     2641 taatacctgc tactacatca ggtttattat atggagcagt ataaaaagta aacaatatag
     2701 gagtatattt taattaatca ttaaatgcaa acagactata ttttggaaag gcactagtag
     2761 gtaaaaaagt tatggtatat tattattaat atgcgtcctc tggtacttat aattggttag
     2821 atctttcata ttcaacatca ggatgtacac caaataatgt tttattaaat ggtatagcta
     2881 acgctttaga tgtccctttt agtgctgggg cattagcagc attaacctca ggtccttcat
     2941 tacttttatc attcaatata aattatgaga ctgattatgt tactttctca ggtggtataa
     3001 tttcatcaac tgttgcagct gaattaatgg tatggattta tacatgagta aatttaaatt
     3061 tttatatatt tcataaatta aaaaaacaaa cttttaaata gaaatatttt tttttcagtt
     3121 taacaaaaac aacaataaaa aaattaacaa caaataatat atcgccactg tggtgaacca
     3181 tcgtattcaa ttatgattta tcataatgga atatgatccc gcgttatggt ggcaaccata
     3241 acaagacaat cgactaggag ggcgcaactt taggcgcacc gacggaggag tattcactat
     3301 ataaacatat aataaaagag ttatataata aggagcaaag cgacaaacag aattataaaa
     3361 ataaaaaaat tatatgcata ttaacatatt gaaaaatcaa actgtaaaaa aaaaatcttg
     3421 tgtcctcgtt gggggtt
//
LOCUS       CruV-260                3439 bp    DNA     circular     24-FEB-2020
DEFINITION  Crucivirus-260 isolate 001502_virusbaton.
ACCESSION   CruV-260
KEYWORDS    .
SOURCE      Crucivirus-260
  ORGANISM  Crucivirus-260
            Unclassified.
REFERENCE   1  (bases 1 to 3439)
  AUTHORS   de la Higuera,I., Kasun,G.W., Torrance,E.L., Pratt,A.A.,
            Maluenda,A., Colombet,J., Bisseux,M., Ravet,V., Dayaram,A.,
            Stainton,D., Kraberger,S., Zawar-Reza,P., Goldstien,S., Briskie,J.,
            White,R., Taylor,H., Gomez,C., Ainley,D.G., Harding,J.S.,
            Fontenele,R.S., Schreck,J., Ribeiro,S.G., Oswald,S.A., Arnold,J.M.,
            Varsani,A., Enault,F. and Stedman,K.M.
  TITLE     Direct Submission
  JOURNAL   Submitted (24-FEB-2020) Biology, Portland State University, 1719 SW
            10th Ave, Portland, Oregon 97201, USA
COMMENT     Bankit Comment: ALT EMAIL:ide@pdx.edu
            Bankit Comment: TOTAL # OF SEQS:451
            Bankit Comment: GAP: est. length - alignment

            ##Assembly-Data-START##
            Assembly Method       :: MetaSPAdes v. 3.12; IDBA-UD v. 1.1.1;
                                     MEGAHIT v. 1.0.3
            Sequencing Technology :: 454; Illumina
            ##Assembly-Data-END##
FEATURES             Location/Qualifiers
     source          1..3439
                     /organism="Crucivirus-260"
                     /mol_type="genomic DNA"
                     /isolate="001502_virusbaton"
                     /isolation_source="Water"
                     /country="France"
                     /collection_date=2013
     CDS             1..1287
                     /codon_start=1
                     /product="Putative CruV Capsid Protein"
                     /translation="MPRKSAIPKKSISKKSSKVVTYRRIRGNGAYKVANAIPEVSAED
                     RKKNRFGTNFGGRLGAWLGNKAHQLFRHITGMGAYKIKGNTMIHGEQPAMFEKRENGH
                     RIQHREFVQDVVSTTSAYTCSTLDVSISNQAVFPWLANLSNAYEQYRVLGAVWEYVPL
                     SGSVSSNQSLGYVSLATEMDSNKPQPISKLQLENFDWSVQSVPCENMLHPIECDPAQT
                     SIPVLYVAPNGNPLPTENKNFVSFCNTYLSVGGQSASGQVLGSLYLAYDIELLKPRLQ
                     VSVPSQCELHYRGVGPSNAIPFGASAPTLRTASAIQSFWLRDSQVTITSNKISLTGLP
                     VGTQVLFTYYATGALITTGQFTVAPVGAVTENDFYGGSYLMQAPQTGVASVGNMMFGC
                     AFSTTAADSNFTFAATGAVPGTAYDLIISVMNAPDA*"
     CDS             complement(1337..2458)
                     /codon_start=1
                     /product="Putative Replication Associated Protein"
                     /translation="MTDTILQTDINSNLQTERQIVLKPKTENVYKRCFYTFNNYTEAD
                     YIALKAYDNYTYHICGKEIAPTTGTPHLQGFIVFKKNIRFSTFRNKISERNFFTETMK
                     KKSTPLQAANYCLKGEGTSRDPKNFNGFEQGSLEIQEPNPGKRNDIWECRDAIAANPK
                     LDVLQEFPHILAKYPNFIKLCQNANQKSRTVMTPIIWIHGTTGTHKSYLSRLLAEWEG
                     YTTFTKPPGQWWDGYQGQDVVILEDFRATQMSYNDLLTLSDRYEHIVMIKGGSVKFTS
                     KLIIINTPEDPEKTFEGFMQKQGSIDQLLRRIRMCMNRTPYLDANNNITSRPYEKWHF
                     AHDMSSFLTTYSEESQAKFKQNETAEICKMLEQTKKYKI*"
     misc_feature    2040..2062
                     /note="stem-loop"
     misc_feature    2048..2056
                     /note="nonanucleotide"
ORIGIN
        1 atgcctcgca aatctgcaat tcctaaaaaa tcaatctcta aaaagagttc aaaagttgta
       61 acgtataggc gtatccgtgg taatggtgct tataaagtcg cgaatgctat tcccgaagtt
      121 agcgcggaag atcgtaaaaa aaataggttt ggtaccaatt ttggtggacg tcttggggca
      181 tggttaggca acaaagctca tcagttattc cgtcatatta caggcatggg tgcctataag
      241 attaaaggaa acactatgat ccatggtgag caaccggcta tgtttgagaa acgagaaaac
      301 ggtcatcgta tccagcatcg tgaattcgtt caagacgttg tctctactac ttcagcgtat
      361 acttgttcta ctcttgacgt ttctatttct aaccaagcgg tctttccatg gttagctaat
      421 ctgtctaatg cttatgagca atatagggta ttaggcgcgg tatgggaata tgttccatta
      481 tctggtagcg tttcatctaa ccaaagtctt ggttatgtct cactagctac tgaaatggat
      541 tccaataagc cgcaacctat tagtaagctc caactggaaa atttcgattg gagtgttcag
      601 tctgttccct gcgaaaacat gcttcatcct atcgaatgcg acccagctca gaccagtatt
      661 cctgttcttt acgtcgctcc taatggtaat cctctgccta ctgagaataa gaattttgta
      721 tccttctgca atacttatct tagcgtaggt ggtcaatcag ctagtggtca agtacttggc
      781 agcctttact tagcttatga cattgaactt cttaagcctc gtctgcaagt aagtgtgcct
      841 agtcaatgcg aactacatta ccgcggtgtt ggacctagta atgctattcc ttttggtgct
      901 agtgctccta ctttaaggac tgcgtctgcg attcaatcgt tttggttacg tgatagtcaa
      961 gtcactatta cgtctaataa gatttcgctt actggtcttc ctgtgggaac acaagttctc
     1021 ttcacttatt atgctacagg cgctcttatc actactggtc aatttacagt tgctccggtt
     1081 ggtgcagtta ctgaaaatga cttttatggt ggctcgtatc ttatgcaagc tcctcaaaca
     1141 ggtgttgctt cagttggaaa tatgatgttt ggctgtgcgt tttcaacgac cgcggctgac
     1201 tccaatttca ctttcgctgc tacaggtgct gttccgggaa ctgcttatga tttaatcatt
     1261 tctgtcatga atgctcctga tgcgtaaatg aattaactaa gttgtagtaa ataattaaaa
     1321 aaaaagtttt ttttgtttaa attttatatt tttttgtttg ttcaagcatt ttacaaattt
     1381 cagctgtttc attttgttta aatttagctt gactttcttc agaataagtt gttaagaatg
     1441 aactcatatc atgtgcaaaa tgccattttt cgtagggcct actcgtaatg ttattatttg
     1501 catctagata aggtgtgcga ttcatacaca tcctgattcg acggagtaat tggtcaattg
     1561 atccttgttt ttgcataaat ccttcaaatg ttttttctgg atcttctggt gtatttataa
     1621 ttataagttt gctagtaaat tttactgagc ctcctttaat cataacgata tgttcgtacc
     1681 tatctgataa tgttaataag tcattatatg acatttgtgt tgcccggaaa tcttcgagga
     1741 ttacaacatc ctgtccttga taaccatccc accactggcc tggcggcttc gtgaaggtgg
     1801 tatatccttc ccattctgct agtaacctac ttagataact cttgtgagtt ccggtagttc
     1861 cgtgaatcca aataattggc gtcattacag tccttgattt ttggttcgcg ttttggcata
     1921 gttttataaa attcgggtat tttgctaaaa tatggggaaa ctcttgtaaa acatctaatt
     1981 taggattagc agcaattgca tcgcggcatt cccaaatgtc gtttcgtttt cctggatttg
     2041 gttcttgtat ttctaaagaa ccttgttcaa agccattaaa gtttttagga tctcggcttg
     2101 tgccttcacc tttcaaacaa taattggctg cttgaagggg tgttgatttc tttttcattg
     2161 tttctgtaaa gaaatttcgt tctgaaattt tgtttcgaaa agttgaaaat cgtatgtttt
     2221 ttttaaaaac aataaatcct tgtaaatgag gtgtgcctgt tgttggtgca atctctttac
     2281 cacatatatg atatgtgtaa ttatcatatg cttttaaagc aatataatcg gcttctgtgt
     2341 aattattaaa agtgtaaaaa caacgcttat aaacgttttc agtctttggc ttcaaaacta
     2401 tttggcgttc agtttgtaaa tttgaattta tgtcagtttg taaaattgta tcggtcattt
     2461 taaaatgtat aaaaagttaa acggtgaaaa atgcttttct tgttttcttg tatatatata
     2521 caaaaactat ttctttaaaa taaattaatt aattgtattt ttttgaaatt agtatattgt
     2581 gtataatttc ggttatttcg aaaactattg catagctctc tcaaaatcct catatcttag
     2641 aaaaatagaa tgtattggtt aataaatgta tttgtataag aggttagggg gtaatataga
     2701 agcaatttta tacaagtcta aaatttcttt tacccctaac acggcctgac ggcctctctt
     2761 attatcttaa ttggttactt aaggtgtagg ttatccatcg caacgccgcc gaaggttaaa
     2821 tttccaaata tcccaattgt cgcaaaaaaa actcttgaaa aaattaatct tccagatgaa
     2881 gttcttcgaa tcttcgattc tcataacttc acactccgtg ttaagtttat ttctcagttg
     2941 ttttcactta gcaatttgta tattttcaat ttacctccgt cgtctcttgc aggatgtata
     3001 actttattta tttgtgtata taatggtgtc taagacatct acggatatgt gtgcagtttc
     3061 ttctatgacg aaacaacaac atcgaagtgt cttaaaaaat ttaagtattg attgtatcat
     3121 tttatttaaa gtatatataa ttatttttta tttaattatt tattgtaaat tatatttttt
     3181 atttattttg taaatatgtc gtttttaaaa gatgttttaa cttatatttt tatgtttaaa
     3241 aggtgtttga aagattgttt ttatgtttaa aaggttattt tgatgtcaaa aaaaagatcg
     3301 tttttttctc cgtaggtgtt actatcaaaa tttttaggtg aaaaaagtga ttaattattt
     3361 aatttatttt tatattttaa tatatctata agtatgtttt tcttctagtt atttttcatc
     3421 gttttatatt ttatttttc
//
LOCUS       CruV-261                3444 bp    DNA     circular     24-FEB-2020
DEFINITION  Crucivirus-261 isolate 001498_virusbaton.
ACCESSION   CruV-261
KEYWORDS    .
SOURCE      Crucivirus-261
  ORGANISM  Crucivirus-261
            Unclassified.
REFERENCE   1  (bases 1 to 3444)
  AUTHORS   de la Higuera,I., Kasun,G.W., Torrance,E.L., Pratt,A.A.,
            Maluenda,A., Colombet,J., Bisseux,M., Ravet,V., Dayaram,A.,
            Stainton,D., Kraberger,S., Zawar-Reza,P., Goldstien,S., Briskie,J.,
            White,R., Taylor,H., Gomez,C., Ainley,D.G., Harding,J.S.,
            Fontenele,R.S., Schreck,J., Ribeiro,S.G., Oswald,S.A., Arnold,J.M.,
            Varsani,A., Enault,F. and Stedman,K.M.
  TITLE     Direct Submission
  JOURNAL   Submitted (24-FEB-2020) Biology, Portland State University, 1719 SW
            10th Ave, Portland, Oregon 97201, USA
COMMENT     Bankit Comment: ALT EMAIL:ide@pdx.edu
            Bankit Comment: TOTAL # OF SEQS:451
            Bankit Comment: GAP: est. length - alignment

            ##Assembly-Data-START##
            Assembly Method       :: MetaSPAdes v. 3.12; IDBA-UD v. 1.1.1;
                                     MEGAHIT v. 1.0.3
            Sequencing Technology :: 454; Illumina
            ##Assembly-Data-END##
FEATURES             Location/Qualifiers
     source          1..3444
                     /organism="Crucivirus-261"
                     /mol_type="genomic DNA"
                     /isolate="001498_virusbaton"
                     /isolation_source="Water"
                     /country="France"
                     /collection_date=2013
     misc_feature    join(3432..3444,1..18)
                     /note="stem-loop"
     misc_feature    1..9
                     /note="nonanucleotide"
     CDS             complement(236..1534)
                     /codon_start=1
                     /product="Putative Replication Associated Protein"
                     /translation="MMSRSPQKKANEEILATMISESQVLAMQSNLDNEILRSQNKRRN
                     AYGDVEEIPVEEKKDVVEDFDESVEDSIKEYVPKGKNWKYCKDHAWLTYKTQIPKDDL
                     EAFLKKIGNKREILFFRAAHEKSDKKHPYEHTHCLVKWSAALASTNVRVFDYQEIHPN
                     IGWVSDKNNHWQRIMRYMAKQDPANADLLDTPDVTGGGNCDQFDIEGIQACVDVNEAY
                     RKYCKKASDASGIKQVWVDRPIPQEHVDRPNFPWHQEFFEFLAFKPDRRKVYWIWEET
                     GNVGKSWMALYLFNYKLAYCVQQTGGGTNFSTIMYNAVKSGWDQKILIFDFPREAETK
                     SIYQCIEFVCNRMVTTTKYQGGSFVIKRPLIVVLANFRPNFKKMSLDRWILRSIGKDK
                     EFNHPLHSDEIDDQFEMDIVLKEIKLLETQEQNAKEFLKM*"
     CDS             complement(1737..3140)
                     /codon_start=1
                     /product="Putative CruV Capsid Protein"
                     /translation="MPAPKKEYVARKPRKPRKSTRSYKPRYEQIPVISPAPAPKKGSL
                     GRSLLKASGGLLGGLFGKTGSSLGSKLAGGLSNIVGLGEYKIEHNSLLEDPPRIKNPS
                     HPGETVLKHREYLGDLISHITPNTFKIQSFPLNPAQPSTFPWLSQIAANYEQYRFEGC
                     IFEFKSMSADALNSVNTALGSVIMATSYNAANPPFQTKSEMENYEFGMSCKPSSSMMH
                     PIECARMQTSLDELYTRPGSGAGLDIRLYDLGNFQIATTGCQGSQVNLGEIWVTFQVG
                     LLKPKLYSSLGNSNLMFSWMNDTFSPADPFGITAGPVNPVVYPISKANSLQVTITSGD
                     IYLPQSQVVQSFLLSLSWDGNTAGFLSPPTLTGFGGLVIDGVLNGPVTQPTAGAELMQ
                     GPWIGDEAFQGCFTYFIQTRGGGSYPIIRVGNDGIYPGEPIFVAVKLIQLPNQYYKVP
                     VIGALAPFTDYTKASYV*"
ORIGIN
        1 taatgttaag agcccccacg gggttagttg gagtttacga acaactcacc ccggtttatg
       61 gttagttttc ccaaaaatca ccattaacaa attatttttt atctaagata aaaaaaattg
      121 cttttgaatc atcacttcgt tattagtcac aagcaattat tattttccca aggaaataat
      181 aatttttaaa tcaaatagtt taacaattca tgaattttag aactctttta ttttatcaca
      241 tttttagaaa ctctttagca ttttgctctt gtgtttctaa caatttgatt tcttttaaga
      301 caatgtccat ttcaaattgg tcatcaattt catcactatg caatggatga ttaaattctt
      361 tgtctttacc gattgaccgt aggatccatc ggtccagact cattttttta aaattaggtc
      421 tgaaattggc taataccact atcaagggtc tttttattac gaaagatccg ccttggtatt
      481 tcgttgttgt aaccattcgg ttacaaacga actcaataca ttgataaatg gatttagttt
      541 cggcttctct tgggaaatca aaaatcaaaa ttttttgatc ccatccactt tttacggcgt
      601 tatacatgat cgtcgaaaaa ttggtaccac ctcctgtttg ttgtacacaa taagccaact
      661 tatagttaaa caaatagagc gccatccaag atttgccaac gttgccagtt tcctcccata
      721 tccaatacac ttttcgccta tcaggcttaa atgccaagaa ttcaaagaat tcttgatgcc
      781 atggaaaatt tggtctatcc acatgttctt gtggtattgg tctatcaacc catacttgct
      841 taatgccgga ggcgtcagaa gcttttttac aatattttct gtaagcttcg ttaacatcaa
      901 cacaagcttg aataccttca atatcaaatt ggtcacaatt tccaccacct gtaacatcag
      961 gtgtatccaa caaatcagcg tttgctgggt cttgctttgc catgtaacgc atgatccgtt
     1021 gccaatgatt atttttatca cttacccatc ctatgtttgg gtgtatttct tggtaatcaa
     1081 aaactcttac gtttgttgat gccaaggcag cactccattt taccaaacaa tgggtatgtt
     1141 cataagggtg ttttttgtcg ctcttttcat gagcggccct aaaaaaaagt atttcacgct
     1201 tattaccaat cttttttaaa aaagcctcaa gatcatcttt aggaatttgt gttttataag
     1261 tcagccacgc atgatcttta cagtatttcc aatttttacc tttaggtaca tattctttaa
     1321 tactgtcttc aacggattcg tcaaaatcct ccacaacatc ctttttttct tcaacaggga
     1381 tttcttcgac atccccgtac gcgtttctac gcttattctg cgatcttagt atttcgttat
     1441 ctaagttgga ctgcatggcc aaaacttgac tttccgaaat catagtcgct aaaatttctt
     1501 cgttcgcttt tttttgaggc gagcgagaca tcatttttta atagaatttt gctcctttat
     1561 gcaaatattc caaaaattac acatcatatc tttcccaaaa tatatagcgt atacgctcta
     1621 ttatgcctaa aatccaacat ttatccaaag tataaattac aagattttta ccaatgtacc
     1681 gaggcattat ttaaagatat caaaaaaaat aaatttgatc aaatttattt tttatattat
     1741 acataacttg cttttgtata atccgtaaaa ggcgccaagg caccgatgac gggtactttg
     1801 tagtattgat ttggtagctg aatcaattta acagctacaa atattggttc ccctggataa
     1861 ataccatcat ttccaactct aattattggg taacttcctc ctcccctagt ttggatgaag
     1921 taagtgaagc atccctgaaa tgcttcatcc ccaatccatg gaccttgcat caattcagcg
     1981 cctgccgttg gttgtgtcac gggtccattt agaaccccat caatcacaag acctccgaaa
     2041 ccagtaagtg taggagggct gaggaaaccg gcagtgtttc cgtcccaact aagggacaat
     2101 aggaacgatt ggaccacttg tgattgtggt aaataaatgt cacctgaggt aatagtaacc
     2161 tggaggctat ttgctttaga aataggatac actacagggt taacaggacc agctgtgata
     2221 ccgaaggggt cagctggact gaatgtatca ttcatccacg agaacattag gttactatta
     2281 cccagtgaac tatataattt gggtttcagg agtccaacct gaaaagtcac ccaaatttca
     2341 ccaagattaa cctgagaccc ttgacaaccg gtggttgcga tttggaaatt acccaaatcg
     2401 tataatctga tatcaagtcc agctcccgag cctggtcggg tgtatagttc atctaatgaa
     2461 gtttgcattc ttgcacattc tataggatgc atcatagatg aggacggttt gcaggacatg
     2521 ccaaactcat aattctccat ttcagacttc gtttggaaag gaggatttgc ggcattataa
     2581 gaggtcgcca taatgacgga accgagcgct gtgttcactg aatttagcgc gtctgcagac
     2641 atacttttga actcaaaaat acagccttca aagcggtatt gttcataatt cgcggctatc
     2701 tgtgagagcc acggaaaagt tgacggttga gcaggattta ggggaaacga ttggatttta
     2761 aacgtattag gtgtaatatg tgaaatcaaa tcgcctaaat actcacggtg ttttaatacc
     2821 gtttcccccg ggtgagaagg atttttaata cgaggtgggt cttcaagaag agaattatgc
     2881 tctatcttgt attcaccgag acctacaata ttagaaagac cacccgctag ttttgaccct
     2941 aaactagacc ctgttttacc aaacagacca cctaaaagtc ctccactagc ttttaggagt
     3001 gaacgaccaa gtgaaccttt cttaggcgca ggagctggag aaataaccgg gatttgttca
     3061 taccgaggtt tataacttcg tgttgacttt ctgggttttc ttggctttcg tgcgacatat
     3121 tctttttttg gagctggcat ttttatagca aaaatgtttc tttatgcaga tacttcaaaa
     3181 aattttcaaa aaaaattcca aagactatat atcatttagc gagcgatcga gcgagtaata
     3241 ccaaaaaaat acccccgtat tttcttcttt atgccaatat tccaaaattt ttccaaaatc
     3301 taaagttggc atttgcgcga agcgcatgaa aaagcgcgaa gcgcttcgta caaaaacgcg
     3361 aagcgttttt tatgaaatga aacgcgtttc gcgctgaatt tcatttgccg taggattgct
     3421 tgccgtaggc ttgggggctc tttt
//
LOCUS       CruV-262                3447 bp    DNA     circular     24-FEB-2020
DEFINITION  Crucivirus-262 isolate 120_1333_IR2_3297.
ACCESSION   CruV-262
KEYWORDS    .
SOURCE      Crucivirus-262
  ORGANISM  Crucivirus-262
            Unclassified.
REFERENCE   1  (bases 1 to 3447)
  AUTHORS   de la Higuera,I., Kasun,G.W., Torrance,E.L., Pratt,A.A.,
            Maluenda,A., Colombet,J., Bisseux,M., Ravet,V., Dayaram,A.,
            Stainton,D., Kraberger,S., Zawar-Reza,P., Goldstien,S., Briskie,J.,
            White,R., Taylor,H., Gomez,C., Ainley,D.G., Harding,J.S.,
            Fontenele,R.S., Schreck,J., Ribeiro,S.G., Oswald,S.A., Arnold,J.M.,
            Varsani,A., Enault,F. and Stedman,K.M.
  TITLE     Direct Submission
  JOURNAL   Submitted (24-FEB-2020) Biology, Portland State University, 1719 SW
            10th Ave, Portland, Oregon 97201, USA
COMMENT     Bankit Comment: ALT EMAIL:ide@pdx.edu
            Bankit Comment: TOTAL # OF SEQS:451
            Bankit Comment: GAP: est. length - alignment

            ##Assembly-Data-START##
            Assembly Method       :: MetaSPAdes v. 3.12; IDBA-UD v. 1.1.1;
                                     MEGAHIT v. 1.0.3
            Sequencing Technology :: 454; Illumina
            ##Assembly-Data-END##
FEATURES             Location/Qualifiers
     source          1..3447
                     /organism="Crucivirus-262"
                     /mol_type="genomic DNA"
                     /isolate="120_1333_IR2_3297"
                     /isolation_source="Water"
                     /country="Norway"
                     /collection_date=2011
     CDS             1..1413
                     /codon_start=1
                     /product="Putative Replication Associated Protein"
                     /translation="MPASAAAKSRKWCFTMFWDGEEPGADYFDDWECEYMYVGREICP
                     DSKREHYQGYIRFPNPRALMGVRKLFTHTIKLPSGNQGYPGHWEPCKGTEAQNVKYCS
                     KDSDLVIEFGKSEAEAKPDKQQGKRNDIVAVRKMVSEGAGMKDVILHCNSYQGIKVAE
                     TMLKYLEIRRSWIPEVYWIYGPAGVGKTQSALRVCDYPYMGLTDDTWWDGYDAHADVI
                     IDDFSDKWCTYKKFLRYTDSSPCKVPVKGSTREFLAKRIFITCDEPPDVIARNWSCVK
                     ANDLVQIGRRITQILYMPRPGEIYGCPGSEIGVEPSVATLFPERGPAQKVWGNTTEPA
                     TPTYDDVDEGGLSAPALTGPQTGLRYCKNHYHLCDGCLSCQYHNIEQNAIRGVIRECT
                     YCYPQEVKCTVQHGPDVVCDDCEFIEPPLVGDDARSLDDQLLAFLIEESAPPLPLSMP
                     PPLVIPAYSASEKGNRRRYL*"
     CDS             complement(1557..2855)
                     /codon_start=1
                     /product="Putative CruV Capsid Protein"
                     /translation="MKKVYAKAVPVKRAAAKRQTSSAGSSLGSAIGMALGAALGGPAG
                     AGVGGMIGNAGGSFISKMLGHGDYAVTNAPQLKENNLVLANAAQMPQFGTGKVACKFV
                     HREFLGDVYSSSTPGAFKITDFPINPGLDQTFPWLSGVVGAKFQQYRINGMSFEYRSM
                     SADALNSTNTALGSVIMSTDYDSADTTFASKQEMENTEYGVSCKPSVNMMHGIECSRF
                     QTPVSELYIRAFDVPAGKDIRLYDMGRFSVATVGCQAPNVNLGELWVSYDIDCFKAIE
                     QVPQYLTPLSQYILNDVDATHALGLTQTERLDQIGLVFDYTAGVASIIFPLDLPPGST
                     FSLYYYVYGAATAGVPDVSPPTLAGGLSAVGSTMGNPYGVAYTGSNGRGFYGVFRYDG
                     TGTPGVPPQLSIPGGVVPTAITQAVINISMMSAKYLSDGL*"
ORIGIN
        1 atgcctgcct cagctgctgc aaaatcacga aaatggtgct ttacgatgtt ttgggacggg
       61 gaagaacccg gcgccgacta cttcgacgac tgggaatgtg aatatatgta tgtcggccgt
      121 gaaatatgtc cagattccaa gcgcgaacac taccaaggct atattcggtt tcctaatcca
      181 cgtgccctta tgggtgtacg gaaattgttt acacacacga ttaagctccc ttctggcaac
      241 caaggttacc ccggtcattg ggagccgtgt aaaggaactg aagcgcagaa cgtaaagtac
      301 tgctctaaag attccgatct tgttatagag tttggtaagt ctgaggctga agctaagccc
      361 gacaaacaac aaggtaagcg caacgacatt gtagctgtcc gcaaaatggt ctctgaaggc
      421 gctggaatga aagatgtgat cttacattgt aatagttatc agggcatcaa agttgctgaa
      481 actatgttaa aatacttgga aatccgacgc tcctggattc ctgaagtata ttggatttac
      541 ggacccgccg gggttggtaa aacgcagtcc gctcttcgcg tctgcgacta tccttacatg
      601 gggctgacgg atgatacttg gtgggacggt tacgacgccc acgctgacgt tattatcgat
      661 gactttagtg ataagtggtg cacttataaa aagtttcttc gatatactga ctcttcaccc
      721 tgcaaagtgc cagtaaaagg cagcaccaga gagtttctcg cgaaacgtat ctttataaca
      781 tgtgatgagc cgcctgatgt cattgcacga aactggagtt gcgtgaaagc caacgattta
      841 gtgcaaatcg gtagaagaat tactcaaatt ctctacatgc cacgtcctgg ggaaatctat
      901 ggatgtcccg gatcagagat aggggtagaa ccctccgtgg cgactttgtt tccggaacgt
      961 ggcccggcac aaaaagtctg gggtaatact acggaaccgg ccacccctac ttacgacgat
     1021 gtcgacgagg ggggactatc ggcccctgcc ctaacgggcc cccagactgg tttgagatac
     1081 tgcaaaaatc actatcacct ttgcgatgga tgtttgtcct gccaatatca taatattgag
     1141 cagaatgcta tccgcggagt tatccgcgaa tgcacatatt gctatccgca agaggtcaaa
     1201 tgtactgtgc agcatggacc tgacgtcgta tgcgacgatt gcgaattcat cgagcctcct
     1261 ctcgtcggcg atgatgcgcg aagtttagat gatcaattgc tagcatttct cattgaagag
     1321 agtgctccgc cattgccttt gtcaatgcct ccgcctctag ttatacccgc gtattccgcg
     1381 agtgaaaaag ggaatcgacg cagatatcta tagatatctt caacccgcga ttcttataga
     1441 agtcacaaaa agttttttcg ggacttctaa gacattgaac atgtctcttt tgtctcatta
     1501 gtctcaataa actcgaaggc atcttcttca gaataatgag gggggccaat ccctagttat
     1561 aaaccgtccg aaagatattt cgcactcatc attgagatat taataacggc ctgcgtaatc
     1621 gcagtaggga ctacaccccc tggaatagat aattgtggtg gtactccagg agtaccggtt
     1681 ccgtcgtatc gaaatactcc atagaaccct ctaccattcg agccggtgta tgctacacca
     1741 tatgggttcc ccattgtcga accgacggcc gaaagccctc cagcaagtgt gggtggggat
     1801 acatcaggaa ctccagcggt agctgcacca tatacgtaat agtacagcga aaacgttgaa
     1861 cctggaggta aatctagtgg aaagattata gatgccacac ctgcggtgta atcaaacacg
     1921 agaccaatct gatcgagtcg ttcggtttgt gttaatccca atgcatgcgt tgcgtccaca
     1981 tcattcaaaa tgtattggga taatggtgta agatattgag gaacttgctc aatagccttg
     2041 aaacagtcaa tgtcataact aacccatagc tctccgagat tgacgttggg agcttgacag
     2101 ccaacagtag cgactgaaaa tcgccccata tcatataatc ggatatcttt acccgctggt
     2161 acatcgaatg ctcgaatata cagctcagat accggtgttt gaaagcgtga acattcgatg
     2221 ccatgcatca tgttaacact tggtttacag gatactccgt actctgtatt ttccatctcc
     2281 tgcttggatg caaacgttgt gtcagcactg tcatagtccg tagacataat aacagaaccc
     2341 aaagcagtat tcgttgaatt caacgcatcg gcggacatgc ttcgatactc gaaagacata
     2401 ccgttaatcc gatattgttg aaactttgcc cctacaacac ctgacagcca tggaaatgtc
     2461 tgatctaaac cgggattgat tggaaagtca gtaatcttaa acgcccctgg agttgaagaa
     2521 ctgtacacat ctccgagaaa ctctcgatgt acaaatttgc atgcaacttt gccagtacca
     2581 aactgaggca tctgtgccgc attcgctaac acaagattgt tctccttcag ttgtggagca
     2641 tttgtaacgg cgtaatctcc atgcccaagc atcttggaaa tgaagctgcc gccagcatta
     2701 ccaatcatac cacctacgcc agctccggcg ggtccgccca aagcagcgcc tagggccata
     2761 ccgattgctg aacccaaaga agaaccagcc gaagacgtct ggcgcttagc ggccgcacgc
     2821 tttacaggca ctgcctttgc gtacaccttt ttcattgggg cggccttctt tacgtaggcc
     2881 tttttggctg gcgcccgata cgctgccggt tttgcgactg cgagacgagg catcgtaacg
     2941 cgggtatatt acccgatttt tttcttctaa aatataaata tatgccgcat agctttccaa
     3001 ccagcgcata tcctcctcat gtttacgctc gattgcatcc cacgctgaca tgtaggatta
     3061 tttattgctc gcaataaata ttcacgaata tttttgctct tgtaatatga tgtcatatcc
     3121 tcggagttca tgacaggtct actcgctata caccgaatgc tggggcattg gggcatcacg
     3181 atcatatttc ccccacgggg aaccacgtgg agcactcagg gttgagagtg agccccttcg
     3241 gggctcagtg caacctgtga ggtttgcact tggcctagtc aggcatatga cccttgtggt
     3301 tcctccccac cgttccacta tgcgctattc agtgcatagg tcgggccata tattcatcct
     3361 cgtcacctct gacctcggat taattatcat ttgaactttt tgttccgcaa tgattatact
     3421 gttctcaccc gactacagcc tctcgac
//
LOCUS       CruV-263                3451 bp    DNA     circular     24-FEB-2020
DEFINITION  Crucivirus-263 isolate 001492_virusbaton.
ACCESSION   CruV-263
KEYWORDS    .
SOURCE      Crucivirus-263
  ORGANISM  Crucivirus-263
            Unclassified.
REFERENCE   1  (bases 1 to 3451)
  AUTHORS   de la Higuera,I., Kasun,G.W., Torrance,E.L., Pratt,A.A.,
            Maluenda,A., Colombet,J., Bisseux,M., Ravet,V., Dayaram,A.,
            Stainton,D., Kraberger,S., Zawar-Reza,P., Goldstien,S., Briskie,J.,
            White,R., Taylor,H., Gomez,C., Ainley,D.G., Harding,J.S.,
            Fontenele,R.S., Schreck,J., Ribeiro,S.G., Oswald,S.A., Arnold,J.M.,
            Varsani,A., Enault,F. and Stedman,K.M.
  TITLE     Direct Submission
  JOURNAL   Submitted (24-FEB-2020) Biology, Portland State University, 1719 SW
            10th Ave, Portland, Oregon 97201, USA
COMMENT     Bankit Comment: ALT EMAIL:ide@pdx.edu
            Bankit Comment: TOTAL # OF SEQS:451
            Bankit Comment: GAP: est. length - alignment

            ##Assembly-Data-START##
            Assembly Method       :: MetaSPAdes v. 3.12; IDBA-UD v. 1.1.1;
                                     MEGAHIT v. 1.0.3
            Sequencing Technology :: 454; Illumina
            ##Assembly-Data-END##
FEATURES             Location/Qualifiers
     source          1..3451
                     /organism="Crucivirus-263"
                     /mol_type="genomic DNA"
                     /isolate="001492_virusbaton"
                     /isolation_source="Water"
                     /country="France"
                     /collection_date=2013
     misc_feature    join(3440..3451,1..18)
                     /note="stem-loop"
     misc_feature    1..9
                     /note="nonanucleotide"
     CDS             727..2121
                     /codon_start=1
                     /product="Putative CruV Capsid Protein"
                     /translation="MPQRRRPAPAYPSLSNRRHVRRPAPSKATVNRAYIAGRADQATA
                     DQSRSRVPRYGKYLGGAAGTALAPFIAPYAPMLAAAAPAAGAYLGDQAQKLVKYVTGW
                     GDYKIGVNSLIKSADSVPEFKNTGSSRCTMIRHKEFIGDVIGGALVSSASVFTIDTFP
                     IQPGNSTTFPWLSKIAANYEQYRFHGLLFHYKTSSGVVSTTPMLGTTVMATQYNSLSN
                     PFQNKQEMENYEFAGSTVPSRNLIHPVECDPRQTQCNGIFNIQSAQNAYGGDKRLYDL
                     GDFSIATIGLPQASEVVGELWVTYDVCLMKPRLVPSGGSNADHWYGTGIGVNNTSNWL
                     GTDLALSINSDGFTTTDNASKIFFNKNFYGVVSVYYGVISAVTTTPVCPGIVGSNGVT
                     DYNMLRVVNGNNTQASYNSIFNNSQTAFYYTSWFRVQPLSNGTIPTLTFSSGTTGTLS
                     FIDIVIVQQPSDFS*"
     CDS             complement(2153..3256)
                     /codon_start=1
                     /product="Putative Replication Associated Protein"
                     /translation="MTSVWSDIKKLRRSPKYEKINIYVPYDENLEQDITKRNRKFCFV
                     INNPTENDWIDLEEIEFRYLCFEQEIGESGTPHIQGYVHFKDAHTIRDLSKKLRRAAI
                     FIARGTAAQNRAYCSKDNNGTFQEFGEIPKQGQRNDKLKMSDTIHMPETQIRELFPYQ
                     ADKLIANKKKLEVLRTPHRRCGDPKLILYIIGDTGAGKSRLGHDFFVRLDKEDIAFDK
                     IDCDGKFMAGYTQDRKPLLWDEFRDEEISHKQFLKICDPWYNPNQNVKNDFAKIDTDV
                     IIITSIIHPEDLYQDKKDKENITGQILRRINIIDMDKICKKDIYSSDVVNEYIAMREF
                     KKICEFKDEIDRIYEDQMSEEEQYITAEVEEFN*"
ORIGIN
        1 caatattacc tttagcctaa ttttttgtgc aataatttac agaatgtcaa atcttgcaga
       61 acttatcctt aatcctaagg atacgcaaca aattaccact aattttgaca tatttttaaa
      121 tattttaaca tttgtgtaat cattaataat tataatttta ttatcatttt aaggatgtat
      181 aacaccattt tatggtttta taacttctgt gtaagttgtg caattttcta aattgatgca
      241 attattggcg ttttctatcg cccataaatt catcttcaca gtaagagtga tttaggctca
      301 ttttatctaa atgatcttca atctctttat ttccgaaagc attgtcaaat ttactcataa
      361 ttagactgct tttaggtagt ctgttattaa tcagactatc acatttatgt tcatctatgt
      421 attcagattt ttctaaatca tagttctcat cttgtttatc taacgagtga gaatcacata
      481 aatcatgatt tggacaataa ctgtctaaat cttgcaaaat atttttaata ttattaaata
      541 atatttggaa ttcagaaaag tgtttacctt tttctgattc taatttcttt ttgtattctg
      601 tcaagattaa aaaagattca gcaattagat cagtcgcttg atctaacgct tcaaagtctg
      661 taacccagat ttctttgttc atatgtcttt tagatatatg ataatttttt ttatatctaa
      721 aataaaatgc cacaacgacg tagaccagct ccagcgtatc catcgttgtc aaacagaaga
      781 cacgtccgta gaccagcacc ttctaaggcg actgtcaata gagcttatat tgcaggacgt
      841 gctgatcaag caacagcgga ccaatctaga agtagagtac ctagatatgg taaatatctt
      901 ggaggtgcag caggtaccgc ccttgcaccg tttatcgccc catatgctcc tatgcttgct
      961 gcagcggctc cagctgcagg tgcatatctt ggagaccaag ctcagaaact agttaaatac
     1021 gttactggtt ggggagatta taagattggt gtcaattctc tgataaaatc cgccgattct
     1081 gttccagagt ttaaaaacac tggatcatct agatgtacca tgattcgtca caaagaattc
     1141 attggtgatg tcataggtgg tgctttagta tcatccgcat cagtatttac tattgatact
     1201 tttcccattc aaccaggaaa ttcaacaaca tttccatggt tgtcaaaaat tgcagccaat
     1261 tatgagcaat ataggttcca cggattgctt tttcattaca aaacaagttc cggggtagtt
     1321 tctactacac ctatgcttgg cacaaccgta atggctaccc agtacaattc actgtcaaac
     1381 ccctttcaga acaaacagga aatggaaaat tatgaatttg caggctcgac tgtcccttca
     1441 agaaatctaa ttcatcctgt tgagtgtgat cccagacaaa ctcaatgtaa tggtattttt
     1501 aacattcaat ctgctcaaaa tgcctatggt ggcgacaaac gtctttacga tcttggagac
     1561 ttttccattg caactatcgg attgcctcaa gcatcagaag ttgttggtga attatgggtt
     1621 acttatgacg tatgtcttat gaaacctaga cttgtgcctt caggcggatc taatgctgat
     1681 cattggtacg gaacaggtat tggtgttaac aatacttcta actggttagg taccgatcta
     1741 gctttatcta ttaatagtga tggatttact acaactgata acgccagtaa aatattcttt
     1801 aataaaaact tttacggtgt agtttcagtt tattatggtg taatctccgc agttacaacc
     1861 actccagtat gtccaggcat cgttggatca aatggagtta ccgattacaa tatgcttcgc
     1921 gttgttaatg gtaataatac tcaggcttct tataattcta tatttaataa ttctcaaacc
     1981 gcattctatt atacatcgtg gtttagagta cagcccctat caaatggaac aatccctacc
     2041 cttacattct caagcggaac aaccggtacg ctttcattca ttgatattgt gattgtacaa
     2101 caaccttcag atttttctta aaaatattat atgggtaatt tacccttata atttaattaa
     2161 attcttcgac ttcagccgta atatattgtt cttcttcaga catttgatct tcgtaaattc
     2221 tatcaatttc gtctttaaat tcacagattt tcttaaattc tctcatcgct atatattcat
     2281 ttaccacatc agatgagtag atatcttttt tacatatttt atccatatca attatgttaa
     2341 tccttcttaa tatttgacca gtaatatttt ctttatcttt tttatcctgg tacaaatctt
     2401 caggatgtat aattgaagtt atgataatta catctgtgtc aattttagcg aaatcattct
     2461 taacattttg attcgggtta taccaaggat cacatatttt aagaaattgt ttgtgagata
     2521 tttcttcatc tctaaattca tcccatagca gcggtttacg atcttgagta taccctgcca
     2581 taaatttacc atcacaatca attttatcaa aggcgatgtc ttctttgtct aatctaacaa
     2641 agaaatcatg accaagtctt gatttaccag cgcctgtatc accaataata tataaaatta
     2701 gttttggatc accacatctt ctatggggag ttctcaaaac ttctaatttt tttttatttg
     2761 ctattaattt atcagcttga taaggaaata attctctaat ttgagtttca ggcatatgaa
     2821 tagtgtcaga cattttcaat ttatcattgc gttgaccttg tttcggaatt tcaccaaatt
     2881 cctgaaaggt tccattgtta tctttagaac aataagctct attttgagct gcggtacctc
     2941 tagcaataaa gattgcagct cgtctcaatt ttttagataa atctcttata gtatgcgcat
     3001 ctttgaagtg aacatatcct tggatatgag gggtacctga ttcaccgatt tcttgttcaa
     3061 aacatagata tcggaattct atttcttcaa gatcaatcca atcgttttcc gttggattat
     3121 ttattacaaa gcaaaacttt ctattacgtt ttgtaatatc ttgttctaga ttttcatcgt
     3181 aaggaacata gatatttatc ttttcatatt taggtgatcg tcgaagcttt ttgatgtcac
     3241 tccacacaga agtcattctt ttttaacttc tgtgtaattt tatttttata tcaaattaat
     3301 tttttgagtc tatttttaga tcatcaaaat tcaatcacaa tgtcaaacga agaacgagct
     3361 tgcgagtgat gagttgacgc atttggccaa aagccaaatg cttgcttctg catcttggcg
     3421 tgacaaccta aaactcaaaa ggctaaagtt c
//
LOCUS       CruV-264                3454 bp    DNA     circular     24-FEB-2020
DEFINITION  Crucivirus-264 isolate H1_84897.
ACCESSION   CruV-264
KEYWORDS    .
SOURCE      Crucivirus-264
  ORGANISM  Crucivirus-264
            Unclassified.
REFERENCE   1  (bases 1 to 3454)
  AUTHORS   de la Higuera,I., Kasun,G.W., Torrance,E.L., Pratt,A.A.,
            Maluenda,A., Colombet,J., Bisseux,M., Ravet,V., Dayaram,A.,
            Stainton,D., Kraberger,S., Zawar-Reza,P., Goldstien,S., Briskie,J.,
            White,R., Taylor,H., Gomez,C., Ainley,D.G., Harding,J.S.,
            Fontenele,R.S., Schreck,J., Ribeiro,S.G., Oswald,S.A., Arnold,J.M.,
            Varsani,A., Enault,F. and Stedman,K.M.
  TITLE     Direct Submission
  JOURNAL   Submitted (24-FEB-2020) Biology, Portland State University, 1719 SW
            10th Ave, Portland, Oregon 97201, USA
COMMENT     Bankit Comment: ALT EMAIL:ide@pdx.edu
            Bankit Comment: TOTAL # OF SEQS:451
            Bankit Comment: GAP: est. length - alignment

            ##Assembly-Data-START##
            Assembly Method       :: MetaSPAdes v. 3.12; IDBA-UD v. 1.1.1;
                                     MEGAHIT v. 1.0.3
            Sequencing Technology :: 454; Illumina
            ##Assembly-Data-END##
FEATURES             Location/Qualifiers
     source          1..3454
                     /organism="Crucivirus-264"
                     /mol_type="genomic DNA"
                     /isolate="H1_84897"
                     /isolation_source="Water"
                     /country="New Zealand"
                     /collection_date=2012
     CDS             join(3225..3454,1..196)
                     /codon_start=1
                     /product="putative ORF"
                     /translation="MFFLTQILARAPRRSVLARSWGLGPPTQCAPMPTPYNTTQANLS
                     KIPHSTLSRLVVAAGRPPGWLFLAGWCRLCPGYIVTPGLPGAGATTCLAGGGWLLFWL
                     AGGGWLHRPASSGGGCWPAPIPGWPRAPWGGRGPGQARL*"
     CDS             join(complement(1..237),complement(3329..3454))
                     /codon_start=1
                     /product="putative ORF"
                     /translation="MTTRAFLEKHVPWPHSLAWPGPRPPQGARGQPGMGAGQQPPPEE
                     AGRWSQPPPASQKRSQPPPAKHVVAPAPGSPGVTIYPGQSRHQPAKKSQPGGRPAATT
                     SLLNVEWGILLKFACVVL*"
     misc_feature    join(3445..3454,1..17)
                     /note="stem-loop"
     misc_feature    1..9
                     /note="nonanucleotide"
     CDS             238..1755
                     /codon_start=1
                     /product="Putative CruV Capsid Protein"
                     /translation="MKRQREETQLVPYQPVTKRIKMDVQRAPYQRGPGFYRGKPLGPL
                     KAPKYTDRMWRVRRPRRKTPKAMAQYWRKRYRRRVLTGRGDYSYSDSRSTGANLGGWL
                     GSKAGEYLGGMAQTAGMALISGLGDYTVNKNVLLSGNMPSIQNMTSGAGITISFQEYL
                     GDVVTPLQVGNSSPFDVTTYVINASNPRTFPWLSQIAQNFEQYEMEGMLFGFRTTSAD
                     LVTNGTNNLQLGTVIMATQYDVADPNFASKGEMLNYQFSTSVKPSQPAIHMIECDPRQ
                     TSVNLLYTGTAAAGTDPRLYNLGRFSIATQGFQGSGIVIGELHVTYQVRLLKPKLWTT
                     LGENIPWSSGIYTMSSGSTKSWLGVSGTIATQTINNSNQTVSIDNNLDITQVQSKVIF
                     QRQRFPQRIYILIAWSAQSGATGGFFNTSDGNPVFSAGGGATLISQQIYPLSATNDSI
                     TGSFMGIVDIAPDQSAPYFLIKSFTPTFQPDYPNGQIIARLTLAPTDPTGNGNLN*"
     CDS             complement(1871..2884)
                     /codon_start=1
                     /product="Putative Replication Associated Protein"
                     /translation="MKSSSSSSSSSSEQVEDPIEHETELVISPYAGKPPARGERGNAQ
                     VCWLYTWFNYDPEKLPVVPDTECKYRIEGKELTPTKKTPHVQGFVIFKTKQRWSTVTT
                     NYPGINWLPMCKKATPWNNFEYCSKDGDFTEFGERPKQPKASGRGNDQQAFAEAFAAD
                     SVGAGIQIIKEKRPRDLALHGEAIERNLKKAKTEQWSHKYRENSFLVPLQDTGSNLLV
                     HGPSNTGKTHYAAAHFENPLVCSHVDDLKRLGPDNDGIIFDDMSFKHWPVEAIIHLLD
                     REFGRSINVRYGTVQIRAGIPKIFTHNTRNPFYEEATIEEEQKIAIERRFRRVNVLNK
                     LYA*"
ORIGIN
        1 tatagtaacc ccagggctcc ccggagccgg agccaccacg tgcttggctg gtggtggctg
       61 gctcctcttc tggctggctg gtggtggttg gctccaccgg ccggcctctt ctggtggtgg
      121 ctgctggccg gctcccatcc ctggctggcc ccgcgccccc tggggggggc gcggccccgg
      181 ccaggccagg ctgtgaggcc aaggcacgtg tttttccaag aacgcacgtg ttgtcatatg
      241 aagcgtcaac gtgaagagac ccaactcgtc ccttatcaac ctgttaccaa acgaatcaaa
      301 atggacgtac aacgagcacc ctatcaacga ggacctggat tttaccgtgg aaaacctctt
      361 ggcccgctta aagcacctaa atacacagat cgaatgtggc gagttcgccg accaaggcga
      421 aaaactccta aagcaatggc tcaatactgg cgaaagcgat accgaagacg agtcctcact
      481 ggacgaggag attatagtta ttcagatagt cgatctactg gtgcgaatct tggaggatgg
      541 cttggaagca aagcgggtga atacctcgga ggtatggccc aaaccgcagg aatggcctta
      601 atatccggtt tgggtgatta tactgttaac aaaaacgtgc tactatctgg aaatatgcct
      661 tcaattcaaa atatgacctc tggtgctgga atcactattt cctttcaaga atatcttggt
      721 gacgttgtca ctccactaca agtgggaaat tcttcacctt tcgatgttac aacatatgtt
      781 attaatgctt caaatcctcg aacattccct tggttatcac aaattgcaca aaattttgaa
      841 caatatgaaa tggaaggaat gctctttggt tttaggacaa cttcagctga cttagttact
      901 aatggcacaa ataacttaca acttggaact gttattatgg ctactcaata tgatgtagct
      961 gatccaaact ttgcatctaa aggtgaaatg ctcaattacc aattttcaac atctgttaaa
     1021 ccctcacaac ctgcaataca catgatagaa tgtgacccta gacaaacctc agtcaatctc
     1081 ctttatactg gtaccgcggc tgctggtacc gatcctcgac tttacaatct tgggagattt
     1141 tcaatcgcta ctcaaggatt tcaaggttct ggtattgtaa ttggtgaact acacgttacc
     1201 tatcaagtca gacttcttaa accaaaatta tggactacac ttggagaaaa tatcccttgg
     1261 agttcaggca tttatactat gagttcagga tcaactaaaa gttggcttgg tgtttctggt
     1321 actattgcta cacagactat taataattct aatcaaacgg tatcgataga taacaatctt
     1381 gatattaccc aggtacaatc aaaagttata ttccaacgtc aacgattccc tcaacgtata
     1441 tacatactta tagcatggtc agctcaatct ggagcaacag gaggattttt taatacctct
     1501 gatggtaatc ccgttttttc tgctggtggt ggtgcaacac tcatatcaca acaaatctac
     1561 cccctctctg ctactaatga ctctattact ggatctttta tgggaattgt agatattgcc
     1621 cctgatcagt cagcacctta ctttttgatt aaatcattca cacctacatt ccaacccgat
     1681 tacccaaatg gtcaaattat tgcgagatta actcttgcac ctacagaccc aactggtaat
     1741 ggcaatctta attgatccag caaccacatt accgcggctg cggacttata tattttttgg
     1801 gttaaaatgt caaacaaagt aataaacaac agtttagttc aaatataatt tattaaaatc
     1861 aacaatatac ttaagcgtat aatttattta agacatttac tcgtcgaaat cgacgctcaa
     1921 tagctatctt ttgctcctct tcaatagtgg cttcttcata gaacgggttt cttgtattgt
     1981 gggtaaagat ctttgggatt ccagctctga tttggactgt tccatatctg acattgattg
     2041 atcttccgaa ctccctatct agcaaatgaa taatggcttc aactggccag tgtttgaatg
     2101 acatatcatc gaagattatt ccatcattat caggccccaa cctctttaaa tcgtcaacat
     2161 gtgaacatac taagggattt tcgaaatggg ctgcagcata atgcgtcttt cctgtatttg
     2221 agggtccatg taccaataga tttgatcccg tgtcttgtag tggtacaagg aagctattct
     2281 ctctgtattt gtgagaccac tgttcggtct tggctttttt aaggtttctt tcaatggctt
     2341 ccccgtgtag agccaagtca cgtggtcttt tctccttgat gatttgtatt ccggctccaa
     2401 cactatcagc ggcaaaggct tcagcgaagg cttgttgatc gttaccgcgg ccgctggcct
     2461 taggttgttt gggtctttct ccaaactcag tgaaatctcc gtccttacta cagtactcga
     2521 agttgttcca aggtgtggct tttttgcaca tgggcaacca gttgattcct ggataattgg
     2581 ttgtaactgt tgaccatctc tgcttggtct taaaaatgac aaatccttgc acgtgtggag
     2641 tcttttttgt tggtgtaagt tcctttcctt caattctgta cttgcattcc gtgtcgggca
     2701 cgactggtaa cttttcggga tcataattga accacgtgta aagccaacag acttgtgcgt
     2761 ttcctctctc cccacgtgca ggtggctttc ctgcgtatgg actaatgacc agttccgttt
     2821 catgttcaat aggatcctca acctgttcag atgatgatga tgatgatgat gatgatgatt
     2881 tcatatatga ttaccgaaag agtagacgct gatgggtata atccttatcg gtaccttaca
     2941 gtcgagaact caatgggtat gatttataac cccccctata taagtataga attcgactcg
     3001 aactcttttc agatttgtac ttatactata tgagggctcc ccatttggga aagttttcaa
     3061 atattttttt tagaaatttt ggctatttgt ttttttatac tgtaaaaaca ttagaaaccg
     3121 ttttttggct atttgttttt ttatactgta aaaacattag aaaccgtttt ttggctattt
     3181 gtttttttat actgtaaaaa cattagaaac cgttttttgg ctatttgttt tttttaaccc
     3241 aaatcttagc cagagcgccg agaaggtcgg tgctagcacg gtcgtggggg ctggggcccc
     3301 cgacccagtg cgcgcctatg cccacaccct acaacaccac acaagcgaat ttaagcaaaa
     3361 ttccccactc tacattaagc aggctggtgg tggctgctgg ccggcctcct ggctggctct
     3421 tcttggctgg ctggtgccgg ctctgccctg ggta
//
LOCUS       CruV-265                3457 bp    DNA     circular     24-FEB-2020
DEFINITION  Crucivirus-265 isolate GP2_73310.
ACCESSION   CruV-265
KEYWORDS    .
SOURCE      Crucivirus-265
  ORGANISM  Crucivirus-265
            Unclassified.
REFERENCE   1  (bases 1 to 3457)
  AUTHORS   de la Higuera,I., Kasun,G.W., Torrance,E.L., Pratt,A.A.,
            Maluenda,A., Colombet,J., Bisseux,M., Ravet,V., Dayaram,A.,
            Stainton,D., Kraberger,S., Zawar-Reza,P., Goldstien,S., Briskie,J.,
            White,R., Taylor,H., Gomez,C., Ainley,D.G., Harding,J.S.,
            Fontenele,R.S., Schreck,J., Ribeiro,S.G., Oswald,S.A., Arnold,J.M.,
            Varsani,A., Enault,F. and Stedman,K.M.
  TITLE     Direct Submission
  JOURNAL   Submitted (24-FEB-2020) Biology, Portland State University, 1719 SW
            10th Ave, Portland, Oregon 97201, USA
COMMENT     Bankit Comment: ALT EMAIL:ide@pdx.edu
            Bankit Comment: TOTAL # OF SEQS:451
            Bankit Comment: GAP: est. length - alignment

            ##Assembly-Data-START##
            Assembly Method       :: MetaSPAdes v. 3.12; IDBA-UD v. 1.1.1;
                                     MEGAHIT v. 1.0.3
            Sequencing Technology :: 454; Illumina
            ##Assembly-Data-END##
FEATURES             Location/Qualifiers
     source          1..3457
                     /organism="Crucivirus-265"
                     /mol_type="genomic DNA"
                     /isolate="GP2_73310"
                     /isolation_source="Sediments"
                     /country="New Zealand"
                     /collection_date=2014
     misc_feature    join(3446..3457,1..19)
                     /note="stem-loop"
     misc_feature    1..9
                     /note="nonanucleotide"
     CDS             114..767
                     /codon_start=1
                     /product="putative ORF"
                     /translation="MFFGFSLEEHIPRPGTEAPGKKRKVVLDPFPAPKYTPAFTAKLM
                     EDGSGQAKKITPKVFNQELWQEYRNLKEGLRGMKKADERLVYAEAWLKKLGIDPFTYK
                     HQLKPIKYQVRTCMKYIPFAWFANEPNGLKAFVPLKEYMMVKRVTHKARRAKNPGFAR
                     WKPWRNTVARHHWKGVWRARFYGSRWRKGRSSFTNIVDGRTGRYTGYRMSRRRRYYG*
                     "
     CDS             complement(300..626)
                     /codon_start=1
                     /product="putative ORF"
                     /translation="MSRYSVTPRLPASKTRILRTSCLVSHAFDHHVLLERDERLQAVG
                     FVRKPSKRDVFHARSHLILDRLQLVLIRKRIDAEFLQPGFSIDKTFVGLLHSSKTFFQ
                     ITVLLP*"
     CDS             781..2118
                     /codon_start=1
                     /product="Putative CruV Capsid Protein"
                     /translation="MTAQPSRKRSRYLGPALSAGFGAAAVALGYGGLSPYASAAGGWL
                     GQKIKDITGFGDYTVNKNSLLSGTVPSVGNSSSHPDGLTISHREYLGDVITSSTAKEF
                     RISDYNLNPSDPQTWEWLSQIACNYEEWVPEGILFYYKSTSGEAFTSTDASMGTVIMA
                     TNYNPYNKAFTTKAEMSSYEFCTSGPPNSDLIHMIECDPHEGAISTYFMSSVNSSGSE
                     GLMDKRFNTLGTFYIATTGFQGTSCNIGELWVTYQVTLLKPKLYESLGLAVDSIRTEM
                     YPYGTATQGSGVYGLWPQTQGTNQARTADLTMPYTAQVPSDALWIDNNSNNVRIHWPV
                     YAFETYYYILLGILSAGLVGPTYTVTHNVGTGLKPPVTNAGPAQSPEVGDVSDYHTFR
                     MEVRVPGGFVCNDPANTPYTSISVAAGITGNFSWKIIQIPAVPNGLYSIDPPL*"
     CDS             complement(2115..3173)
                     /codon_start=1
                     /product="Putative Replication Associated Protein"
                     /translation="MQRSRGWCFTLNNWTHDEVLKVKENLAECRYWIMGREVAPDTST
                     PHVQGYLYCKNAVSFNTVRLWLLNRAHIEKAKGTPAQNYEYCSKDKDFEEYGSRPMSQ
                     EEKGEVGGDAQKEKWATIWNLAKQGKIEDIAEDYPKECFVYYKNIMLIRQLGAGATDD
                     LSNVCGLWLYGESGVGKSHKARHIEPDSYYIKGINKWWCNYHGQRTVILEDVGKDHAF
                     LGDFLKIWTDKWAFTAEFKGGSMARIRPARFVVTSQYRIEDIWMDKETRDALGRRFEV
                     QEVVKGPLDVMLRAEFPGVGAQDVVMSPIAYDVVPSDDEMALSLSPIDSEELSDYEKR
                     KRKREKCPFIEDECGESD*"
ORIGIN
        1 tagtattacc ctagacccct agtgtgtgtg taggggggcc ctaatctcgc caaatggtcc
       61 ctatcaggat agcctacact tttcactgaa aaagtgggcg gggaaaaaaa tttttgtttt
      121 ttggtttttc gctggaggag cacatccccc gtcccggaac cgaagcccca ggtaagaaga
      181 ggaaagttgt tctagaccct tttcccgcgc cgaagtatac tcctgccttt actgctaagc
      241 taatggaaga cggatctggt caagcgaaga agatcacgcc caaagtgttt aatcaggaac
      301 tatggcaaga gtaccgtaat ttgaaagaag gtcttcgagg aatgaagaag gccgacgaac
      361 gtcttgtcta tgctgaagcc tggctgaaga aactcggcat cgatcctttt acgtataagc
      421 accagctgaa gccgatcaag tatcaagtgc gaacgtgcat gaaatacatc ccttttgctt
      481 ggtttgcgaa cgaacccaac ggcttgaagg cgttcgtccc gctcaaggag tacatgatgg
      541 tcaaacgcgt gactcacaag gcacgacgtg cgaagaatcc tggttttgct cgctggaagc
      601 cgtggcgtaa cactgtagcg agacatcact ggaaaggagt gtggcgtgcc cgattttatg
      661 gttctcgctg gcgcaaaggc aggagctcgt tcacgaacat cgttgacggg cgaactggga
      721 gatatactgg ctatagaatg tctcgccgtc gtagatatta tggttaataa atctttttag
      781 atgactgcgc aaccttcacg gaaacgatca cgatacctgg gaccagcttt atctgcaggt
      841 tttggtgctg ctgcagtggc acttggctac ggtggcttgt caccgtatgc ctccgcagct
      901 ggtggatggc ttggacagaa aattaaagac atcacaggat ttggtgacta cacggtgaac
      961 aagaactctc tgctgtcagg tactgtaccg tcggtaggga attcttcttc tcatccagat
     1021 ggccttacga tatcgcatcg agagtactta ggagatgtga ttacatcaag caccgccaaa
     1081 gaattccgta tatctgatta caacttgaat ccgagcgatc cacaaacgtg ggagtggctg
     1141 tcacagattg cttgtaacta cgaagagtgg gtaccagaag ggatactctt ctactacaaa
     1201 agtacaagtg gagaagcttt tacctcgacc gatgcctcca tgggtactgt gatcatggca
     1261 accaattaca atccgtacaa caaggcattt acgacaaaag cagagatgtc aagctatgaa
     1321 ttttgcacca gcggtccacc aaactctgat ttgatccaca tgattgagtg cgatccgcac
     1381 gaaggagcga tatctacata ctttatgagc agtgtgaact cttcaggcag cgaaggactc
     1441 atggacaaga ggttcaacac tcttggaacc ttttacatcg ccactacagg atttcaaggg
     1501 acatcgtgca atatcggtga gctttgggtg acataccaag tgactttgtt gaagcccaaa
     1561 ctatacgaaa gtcttggact tgctgttgat tccatcagaa ccgagatgta cccgtacggg
     1621 acagctacgc aaggatcagg tgtgtatgga ctgtggccgc agacgcaagg aaccaatcaa
     1681 gctagaactg cggaccttac gatgccttat acagcacaag ttccatcaga tgcattgtgg
     1741 attgacaaca attccaacaa tgtccgcatc cattggccag tgtatgcttt tgagacgtac
     1801 tactacatcc ttttgggaat cctcagcgct ggacttgtag gaccgacgta cacagtgacc
     1861 cataatgtcg gtactggctt gaaaccacct gtaacaaacg caggaccagc tcaatcacca
     1921 gaagtaggag atgtgtccga ttatcatact tttcgtatgg aagtgagagt acctggcggg
     1981 tttgtctgta atgatccagc caatacaccg tatacatcaa tatctgtggc tgcaggaatc
     2041 acagggaact ttagttggaa aatcattcaa attccagcag tgccaaatgg actttattct
     2101 atcgatccac ctctctaatc actctctcca cactcatctt caataaaagg acatttctct
     2161 ctctttcttt ttcttttttc gtagtcgcta agctcctcag aatcaattgg agacagcgac
     2221 aatgccatct catcgtcaga cggcacaacg tcgtaggcga tgggactcat gacaacatct
     2281 tgtgccccaa cgccagggaa ctcggcacgt aacatcacgt ccagtggccc tttgaccact
     2341 tcttgcactt cgaacctccg gccaagggcg tcacgcgtct ctttgtccat ccaaatgtct
     2401 tcgatccggt actgcgaggt aacgacaaag cgagctggcc ggattcttgc catggacccg
     2461 cccttgaact ccgcggtgaa cgcccacttg tcagtccaga tcttgaggaa gtcgccgaga
     2521 aacgcgtggt cctttccgac gtcttcgagg atgacagtcc tttgaccgtg gtaattgcac
     2581 caccacttgt tgatcccttt gatgtaataa ctatcgggtt caatgtgtcg cgccttgtga
     2641 gatttcccaa caccagactc accgtacaac cacaagccgc acacgttaga gagatcatcc
     2701 gtagccccag cacctaactg acggataagc ataatattct tgtagtaaac gaaacactct
     2761 ttaggatagt cctctgcgat gtcttcgatc ttgccttgct ttgctaagtt ccaaatcgta
     2821 gcccacttct ctttctgcgc atcgccacca acctcaccct tttcttcttg agacattgga
     2881 cgagaaccgt actcttcgaa gtccttgtcc ttggaacagt attcgtaatt ctgcgcagga
     2941 gtgcccttcg ctttttcgat gtgagccctg ttcagcaacc aaaggcgaac cgtattgaaa
     3001 gacacagcgt ttttacagta caaataacct tgaacgtgag gagtagaagt atccggtgcg
     3061 acctcacggc ccataatcca atatcgacat tcagccaaat tctccttaac ttttaggact
     3121 tcgtcatgcg tccagttgtt tagggtaaaa caccagcctc tcgatctctg cattagaaaa
     3181 cacgaaacaa cgttcactcg aattagggtt tatttagggt ataataatat tcagctaatt
     3241 gcctataaaa ggggatcact atagataaag atgtgccccc cttggggggt tgggggggaa
     3301 aaggctcccg cagggtaact ggctcccgca gggcagtcga cccctttgct ttcccgggag
     3361 atttgccatg tcgcctaatc tccccgtagg ggaaagcggg gtctgggggc gcagccccca
     3421 ggcgcttttt tgacgtccaa cacacggggt ctaggca
//
LOCUS       CruV-266                3458 bp    DNA     circular     24-FEB-2020
DEFINITION  Crucivirus-266 isolate GP2_73310b.
ACCESSION   CruV-266
KEYWORDS    .
SOURCE      Crucivirus-266
  ORGANISM  Crucivirus-266
            Unclassified.
REFERENCE   1  (bases 1 to 3458)
  AUTHORS   de la Higuera,I., Kasun,G.W., Torrance,E.L., Pratt,A.A.,
            Maluenda,A., Colombet,J., Bisseux,M., Ravet,V., Dayaram,A.,
            Stainton,D., Kraberger,S., Zawar-Reza,P., Goldstien,S., Briskie,J.,
            White,R., Taylor,H., Gomez,C., Ainley,D.G., Harding,J.S.,
            Fontenele,R.S., Schreck,J., Ribeiro,S.G., Oswald,S.A., Arnold,J.M.,
            Varsani,A., Enault,F. and Stedman,K.M.
  TITLE     Direct Submission
  JOURNAL   Submitted (24-FEB-2020) Biology, Portland State University, 1719 SW
            10th Ave, Portland, Oregon 97201, USA
COMMENT     Bankit Comment: ALT EMAIL:ide@pdx.edu
            Bankit Comment: TOTAL # OF SEQS:451
            Bankit Comment: GAP: est. length - alignment

            ##Assembly-Data-START##
            Assembly Method       :: MetaSPAdes v. 3.12; IDBA-UD v. 1.1.1;
                                     MEGAHIT v. 1.0.3
            Sequencing Technology :: 454; Illumina
            ##Assembly-Data-END##
FEATURES             Location/Qualifiers
     source          1..3458
                     /organism="Crucivirus-266"
                     /mol_type="genomic DNA"
                     /isolate="GP2_73310b"
                     /isolation_source="Sediments"
                     /country="New Zealand"
                     /collection_date=2014
     misc_feature    join(3447..3458,1..19)
                     /note="stem-loop"
     misc_feature    1..9
                     /note="nonanucleotide"
     CDS             114..767
                     /codon_start=1
                     /product="putative ORF"
                     /translation="MFFGFSLEEHIPRPGTEAPGKKRKVVLDPFPAPKYTPAFTAKLM
                     EDGSGQAKKITPKVFNQELWQEYRNLKEGLRGMKKADERLVYAEAWLKKLGIDPFTYK
                     HQLKPIKYQVRTCMKYIPFAWFANEPNGLKAFVPLKEYMMVKRVTHKARRAKNPGFAR
                     WKPWRNTVARHHWKGVWRARFYGSRWRKGRSSFTNIVDGRTGRYTGYRMSRRRRYYG*
                     "
     CDS             complement(300..626)
                     /codon_start=1
                     /product="putative ORF"
                     /translation="MSRYSVTPRLPASKTRILRTSCLVSHAFDHHVLLERDERLQAVG
                     FVRKPSKRDVFHARSHLILDRLQLVLIRKRIDAEFLQPGFSIDKTFVGLLHSSKTFFQ
                     ITVLLP*"
     CDS             781..2313
                     /codon_start=1
                     /product="Putative CruV Capsid Protein"
                     /translation="MTAQPSRKRSRYLGPALSAGFGAAAVALGYGGLSPYASAAGGWL
                     GQKIKDITGFGDYTVNKNSLLSGTVPSVGNSSSHPDGLTISHREYLGDVITSSTAKEF
                     RISDYNLNPSDPQTWEWLSQIACNYEEWVPEGILFYYKSTSGEAFTSTDASMGTVIMA
                     TNYNPYNKAFTTKAEMSSYEFCTSGPPNSDLIHMIECDPHEGAISTYFMSSVNSSGSE
                     GLMDKRFNTLGTFYIATTGFQGTSCNIGELWVTYQVTLLKPKLYESLGLAVDSIRTEM
                     YPYGTATQGSGVYGLWPQTQGTNQARTADLTMPYTAQVPSDALWIDNNSNNVRIHWPV
                     YAFETYYYILLGILSAGLVGPTYTVTHNVGTGLKPPVTNAGPAQSPEVGDVSDYHTFR
                     MEVRVPGGFVCNDPANTPYTSISVAAGITGNFSWKIIQIPAVPNGLYSIDPPFFNPLS
                     TLIFNKRTFLSLSFSFFVVAKLLRINWRQRQCHLIVRRHNVVGDGTHDNILCPNAREL
                     GT*"
     CDS             complement(2017..3174)
                     /codon_start=1
                     /product="Putative Replication Associated Protein"
                     /translation="MQRSRGWCFTLNNWTHDEVLKVKENLAECRYWIMGREVAPDTST
                     PHVQGYLYCKNAVSFNTVRLWLLNRAHIEKAKDTPAQNYEYCSKDKDFEEYGSRPMSQ
                     EEKGEVGGDAQKEKWATIWNLAKQGKIEDIAEDYPKECFVYYKNIMLIRQLGAGATDD
                     LSNVCGLWLYGESGVGKSHKARHIEPDSYYIKGINKWWCNYHGQRTVILEDVGKDHAF
                     LGDFLKIWTDKWAFTAEFKGGSMARIRPARFVVTSQYRIEDIWMDKETRDALGRRFEV
                     QEVVKGPLDVMLRAEFPGVGAQDVVMSPIAYDVVPSDDEMALSLSPIDSEELSDYEKR
                     KRKREKCPFIEDECGERIKERWIDRIKSIWHCWNLNDFPTKVPCDSCSHRY*"
ORIGIN
        1 tagtattacc ctagacccct agtgtgtgtg taggggggcc ctaatctcgc caaatggtcc
       61 ctatcaggat agcctacact tttcactgaa aaagtgggcg gggaaaaaaa tttttgtttt
      121 ttggtttttc gctggaggag cacatccccc gtcccggaac cgaagcccca ggtaagaaga
      181 ggaaagttgt tctagaccct tttcccgcgc cgaagtatac tcctgccttt actgctaagc
      241 taatggaaga cggatctggt caagcgaaga agatcacgcc caaagtgttt aatcaggaac
      301 tatggcaaga gtaccgtaat ttgaaagaag gtcttcgagg aatgaagaag gccgacgaac
      361 gtcttgtcta tgctgaagcc tggctgaaga aactcggcat cgatcctttt acgtataagc
      421 accagctgaa gccgatcaag tatcaagtgc gaacgtgcat gaaatacatc ccttttgctt
      481 ggtttgcgaa cgaacccaac ggcttgaagg cgttcgtccc gctcaaggag tacatgatgg
      541 tcaaacgcgt gactcacaag gcacgacgtg cgaagaatcc tggttttgct cgctggaagc
      601 cgtggcgtaa cactgtagcg agacatcact ggaaaggagt gtggcgtgcc cgattttatg
      661 gttctcgctg gcgcaaaggc aggagctcgt tcacgaacat cgttgacggg cgaactggga
      721 gatatactgg ctatagaatg tctcgccgtc gtagatatta tggttaataa atctttttag
      781 atgactgcgc aaccttcacg gaaacgatca cgatacctgg gaccagcttt atctgcaggt
      841 tttggtgctg ctgcagtggc acttggctac ggtggcttgt caccgtatgc ctccgcagct
      901 ggtggatggc ttggacagaa aattaaagac atcacaggat ttggtgacta cacggtgaac
      961 aagaactctc tgctgtcagg tactgtaccg tcggtaggga attcttcttc tcatccagat
     1021 ggccttacga tatcgcatcg agagtactta ggagatgtga ttacatcaag caccgccaaa
     1081 gaattccgta tatctgatta caacttgaat ccgagcgatc cacaaacgtg ggagtggctg
     1141 tcacagattg cttgtaacta cgaagagtgg gtaccagaag ggatactctt ctactacaaa
     1201 agtacaagtg gagaagcttt tacctcgacc gatgcctcca tgggtactgt gatcatggca
     1261 accaattaca atccgtacaa caaggcattt acgacaaaag cagagatgtc aagctatgaa
     1321 ttttgcacca gcggtccacc aaactctgat ttgatccaca tgattgagtg cgatccgcac
     1381 gaaggagcga tatctacata ctttatgagc agtgtgaact cttcaggcag cgaaggactc
     1441 atggacaaga ggttcaacac tcttggaacc ttttacatcg ccactacagg atttcaaggg
     1501 acatcgtgca atatcgggga gctttgggtg acataccaag tgactttgtt gaagcccaaa
     1561 ctatacgaaa gtcttggact tgctgttgat tccatcagaa ccgagatgta cccgtacggg
     1621 acagctacgc aaggatcagg tgtgtatgga ctgtggccgc agacgcaagg aaccaatcaa
     1681 gctagaactg cggaccttac gatgccttat acagcacaag ttccatcaga tgcattgtgg
     1741 attgacaaca attccaacaa tgtccgcatc cattggccag tgtatgcttt tgagacgtac
     1801 tactacatcc ttttgggaat cctcagcgct ggacttgtag gaccgacgta cacagtgacc
     1861 cataatgtcg gtactggctt gaaaccacct gtaacaaacg caggaccagc tcaatcacca
     1921 gaagtaggag atgtgtccga ttatcatact tttcgtatgg aagtgagagt acctggcggg
     1981 tttgtctgta atgatccagc caatacaccg tatacatcaa tatctgtggc tgcaggaatc
     2041 acagggaact ttagttggaa aatcattcaa attccagcag tgccaaatgg actttattct
     2101 atcgatccac ctttctttaa tcctctctcc acactcatct tcaataaaag gacatttctc
     2161 tctctttctt tttctttttt cgtagtcgct aagctcctca gaatcaattg gagacagcga
     2221 caatgccatc tcatcgtcag acggcacaac gtcgtaggcg atgggactca tgacaacatc
     2281 ttgtgcccca acgccaggga actcggcacg taacatcacg tccagtggcc ctttgaccac
     2341 ttcttgcact tcgaacctcc ggccaagggc gtcacgcgtc tctttgtcca tccaaatgtc
     2401 ttcgatccgg tactgcgagg taacgacaaa gcgagctggc cggattcttg ccatggaccc
     2461 gcccttgaac tccgcggtga acgcccactt gtcagtccag atcttgagga agtcgccgag
     2521 aaacgcgtgg tcctttccga cgtcttcgag gatgacagtc ctttgaccgt ggtaattgca
     2581 ccaccacttg ttgatccctt tgatgtaata actatcgggt tcaatgtgtc gcgccttgtg
     2641 agatttccca acaccagact caccgtacaa ccacaagccg cacacgttag agagatcatc
     2701 cgtagcccca gcacctaact gacggataag cataatattc ttgtagtaaa cgaaacactc
     2761 tttaggatag tcctctgcga tgtcttcgat cttgccttgc tttgctaagt tccaaatcgt
     2821 agcccacttc tctttctgcg catcgccacc aacctcaccc ttttcttctt gagacattgg
     2881 acgagaaccg tactcttcga agtccttgtc cttggaacag tattcgtaat tctgcgcagg
     2941 agtgtccttc gctttttcga tgtgagccct gttcagcaac caaaggcgaa ccgtattgaa
     3001 agacacagcg tttttacagt acaaataacc ttgaacgtga ggagtagaag tatccggtgc
     3061 gacctcacgg cccataatcc aatatcgaca ttcagccaaa ttctccttaa cttttaggac
     3121 ttcgtcatgc gtccagttgt ttagggtaaa acaccagcct ctcgatctct gcattagaaa
     3181 acacgaaaca acgttcactc gaattagggt ttatttaggg tataataata ttcagctaat
     3241 tgcctataaa aggggatcac tatagataaa gatgtgcccc ccttgggggg ttggggggga
     3301 aaaggctccc gcagggtaac tggctcccgc agggcagtcg acccctttgc tttcccggga
     3361 gatttgccat gtcgcctaat ctccccgtag gggaaagcgg ggtctggggg cgcagccccc
     3421 aggcgctttt ttgacgtcca acacacgggg tctaggca
//
LOCUS       CruV-267                3465 bp    DNA     circular     24-FEB-2020
DEFINITION  Crucivirus-267 isolate 001535_virusbaton.
ACCESSION   CruV-267
KEYWORDS    .
SOURCE      Crucivirus-267
  ORGANISM  Crucivirus-267
            Unclassified.
REFERENCE   1  (bases 1 to 3465)
  AUTHORS   de la Higuera,I., Kasun,G.W., Torrance,E.L., Pratt,A.A.,
            Maluenda,A., Colombet,J., Bisseux,M., Ravet,V., Dayaram,A.,
            Stainton,D., Kraberger,S., Zawar-Reza,P., Goldstien,S., Briskie,J.,
            White,R., Taylor,H., Gomez,C., Ainley,D.G., Harding,J.S.,
            Fontenele,R.S., Schreck,J., Ribeiro,S.G., Oswald,S.A., Arnold,J.M.,
            Varsani,A., Enault,F. and Stedman,K.M.
  TITLE     Direct Submission
  JOURNAL   Submitted (24-FEB-2020) Biology, Portland State University, 1719 SW
            10th Ave, Portland, Oregon 97201, USA
COMMENT     Bankit Comment: ALT EMAIL:ide@pdx.edu
            Bankit Comment: TOTAL # OF SEQS:451
            Bankit Comment: GAP: est. length - alignment

            ##Assembly-Data-START##
            Assembly Method       :: MetaSPAdes v. 3.12; IDBA-UD v. 1.1.1;
                                     MEGAHIT v. 1.0.3
            Sequencing Technology :: 454; Illumina
            ##Assembly-Data-END##
FEATURES             Location/Qualifiers
     source          1..3465
                     /organism="Crucivirus-267"
                     /mol_type="genomic DNA"
                     /isolate="001535_virusbaton"
                     /isolation_source="Water"
                     /country="France"
                     /collection_date=2013
     CDS             1..1185
                     /codon_start=1
                     /product="Putative Replication Associated Protein"
                     /translation="MQLRYTYAMTTHDWPIWMDSDAFESKLVDVRHDMPAPQPTETKT
                     SSPVEPVKDSKWYKQRACNWCFTIFGYTPELVEQMKTEWPDRLAGFKCLMWEPEICPD
                     TKRPHLQGFICYEQVKSGKQITDKLMDKCRWSVMREPVVNNVIYCSKDKVGITIIGTL
                     PISNAMKGALGKTMGPWNQLKERTKAGASITEIDDEFTDLAGKFPNGVDRIYNRFKPK
                     GEFNLITKYGSLYPWQKDLLDIIELGANARSVIWCWEAQGDTGKSFFVKHLAVNHGFQ
                     PLTNAATRDIACAWERGNVCIDYARTDSTERNYDIIEKIKNGLMFSAKYQSCTKFSEQ
                     YKDVHVICFANEPPDVTKLSMDRWRIYNIVDKKLVPQAARDLLPTFGMPSPVIAPVSL
                     PA*"
     CDS             complement(1986..3332)
                     /codon_start=1
                     /product="Putative CruV Capsid Protein"
                     /translation="MPRAAAPKVVYVAAPPRKKAPKKKHASRVGGLRGHGDYTYDKPG
                     PWGRIGRSLGEHAGGMVGASYGAKGVGSAIGGKLGSYLHYIGKIFGSGDYVTSPNAVK
                     NNILVNQAQIPQFSDGKNSVRIAHREYICDIVSHGTANTFRLQSFPIQPGLASSFPWL
                     ANVVGGSFQQYRINGMVYEFRTMSADALNSVNTALGQVIMATDYDSKDANFSSKQQME
                     NTEFGVSCKPSSSMIHAIECARNQTSISELYVRPFAVPSGADQRLYDMGNFQIATNGV
                     QGTSVNLGELWVSYDITFFKTVEYPPGYQMGLANYLLNSVAAASPLGVGPTVQNGGAD
                     NIGLTLTSTTVGFPNNTPIGTTYELTYYLSLASSALTLPAITVSNGFTILAGFKAPSA
                     AATTTIGMCVTYVQLTSLPGNALLPTVTYGAYTGGAAGAGCYLDVYQISGAPIDAP*"
ORIGIN
        1 atgcagctaa ggtataccta cgctatgacg actcatgact ggcctatctg gatggatagc
       61 gacgcatttg aatctaagct ggtggacgtg cgccatgata tgccagcacc tcaaccaaca
      121 gagactaaaa cgtccagccc ggttgaaccg gttaaagatt cgaagtggta taagcagagg
      181 gcttgcaact ggtgcttcac catcttcggc tatacacccg aacttgttga acaaatgaag
      241 accgaatggc ctgatcggct agccggcttc aagtgtttga tgtgggaacc ggaaatctgc
      301 cccgatacga agcgaccgca tcttcagggt ttcatttgct acgagcaggt gaaatctggc
      361 aagcagatca ccgataagct catggacaaa tgcagatggt ccgtaatgcg cgaacctgtt
      421 gtcaataatg ttatttattg cagcaaggat aaagtcggca ttaccatcat cggaacgctc
      481 ccaatctcta acgcgatgaa gggtgcgctt ggcaagacca tggggccttg gaatcagcta
      541 aaggaacgca ctaaagcagg tgcgtccatt actgaaatag atgacgaatt cactgacctg
      601 gcaggtaagt tccccaacgg cgtagatcgg atctataacc gatttaagcc taagggtgaa
      661 tttaatctaa taactaaata cggttcctta tacccttggc agaaggacct attagatatc
      721 atcgagcttg gcgccaatgc gcgctccgtg atatggtgtt gggaagccca aggtgatacc
      781 ggcaagagtt tcttcgtgaa acacctcgcg gttaatcatg gttttcaacc acttaccaac
      841 gctgccacac gtgatatagc ctgcgcctgg gagcgcggca atgtgtgcat cgattacgca
      901 cgaactgact cgactgagcg caactatgac attattgaga aaatcaagaa cgggttgatg
      961 ttctctgcta aatatcagag ctgcactaag tttagcgagc agtataaaga cgtgcatgtt
     1021 atttgcttcg ccaacgaacc tcctgacgtt accaagctta gcatggaccg gtggaggatc
     1081 tataacatag tcgataaaaa gctagttccg caagctgcac gagacctgtt accgacattc
     1141 ggcatgcctt cccctgtgat tgcgcctgtg tcactgccag cctagataag tctgatgcaa
     1201 gctagcccac gcccgggcat tgatgcgcga gcatggttat cgctacgctc aaaccatgct
     1261 cgcgcacgcc tctacataac ggcggcccaa gcctgtcaac cattgcattt tttgatccaa
     1321 aaaacagctg cctgcgcatc gtggggagtc cctgcccttt tgaattaatt aattaaaaag
     1381 ccccttgcag ccaaggggta gacttgaaga aatccttcca ggctgtccta aagtcccatt
     1441 aaagcccatg gcgttaatgt ttacctgcac aagtctatgc agttaagggg tctggtaata
     1501 atacagccag ctctactgca cgcagtttag agccgtctgg tcagacccct ttttcgcctg
     1561 ccgcgggctt gtcctggtcg ccagatttgg cgaccgactt gttcggacat gctcatgact
     1621 gcgtcttagc ctgtgttacc ctcacgtccc cgttgacgcc aagaggcgag gccgacgaag
     1681 cgccccaaac aagtgtgggg gcgctccggc gccctcgccc ctcgacgtcc ccgcggacgt
     1741 ccctcggaac acacatagat ttgagctacg cgcgcttgct ctttttttgc tcatcctcgg
     1801 acccttctga tagaaggccc atgcactaaa tcccgtgcat caaaaaatat agcctaaagg
     1861 ctctgtttgt tctcttttcc gtccatcaaa tttggtggac gtgctatatg gaatggcgcg
     1921 tgccgctcct ctgagccata tagattgctc gttactccta acgatctatt ccccaaatct
     1981 ggggattagg gagcatcaat aggagcaccg gagatctgat agacgtccag ataacacccg
     2041 gcacctgcgg cacctccagt gtatgcccca tatgtcactg ttggcagcaa tgcattgccc
     2101 ggcaacgacg tgagctgaac gtaagtcaca cacataccga tcgttgtagt cgcagcagca
     2161 ctgggcgctt tgaatcctgc taggattgta aacccattac taaccgtaat ggctggcagc
     2221 gtcaaggcgc tggatgcgag cgaaagatag tatgttagtt catacgtagt ccctatcggg
     2281 gtattgtttg ggaaacctac ggttgtgctg gtcaaagtca acccgatgtt gtctgcgcct
     2341 ccattctgga ctgttggacc aactcccaaa gggcttgcag ccgcgacgct gttgagcaaa
     2401 taattcgcta aacccatctg atagcccgga ggatactcca cggtcttgaa gaaggtgatg
     2461 tcataggaaa cccagagctc gccaaggttc acactggtcc cctgcacacc attcgttgca
     2521 atctgaaaat tacccatatc gtataaacgt tgatccgcac cgcttggcac agcaaagggt
     2581 cgcacgtata actccgagat ggatgtttga ttgcgcgcgc actcaatcgc atggatcatg
     2641 cttgatgagg gcttgcatga gactccgaac tctgtgttct ccatctgttg cttggagctg
     2701 aaatttgcgt ccttcgagtc gtaatcagtt gccataatca cctgaccgag tgcagtattc
     2761 acagagttca acgcatcggc cgacattgtc cgaaactcat acaccatacc attgattcga
     2821 tactgttgga acgaaccacc aactacgttg gccagccaag gaaatgacga tgcaagtcct
     2881 ggttggatgg ggaaactctg aagcctaaag gtattcgccg ttccatgact gacgatatcg
     2941 cagatgtatt cacgatgcgc gatacgaaca ctgttcttac cgtctgaaaa ttgaggaatt
     3001 tgggcctgat tcacaaggat attgttcttc acggcattcg ggcttgtcac atagtcgcca
     3061 gagccgaata tcttgccgat gtagtgtaga tacgagccaa gctttccccc gatggcggac
     3121 ccgacgcctt tcgcgccgta acttgctcct accataccac cggcatgctc tcccagagag
     3181 cgaccaatac ggccccaggg tcccggcttg tcataagtat aatcaccatg accacgcaaa
     3241 ccaccgacac gagaagcatg cttcttcttg ggtgccttct tacgaggtgg tgcggcgacg
     3301 taaacgacct tcggtgctgc agctcttggc atgtgatgat cacttgatta attgttttcc
     3361 caagaaagtt tcggacgatg tcgtgtgacg agtcgtatat gctagtgccc gagacttctt
     3421 aagaacaaaa ttaaataatt aggtaattaa ttatcataag tcttt
//
LOCUS       CruV-268                3470 bp    DNA     circular     24-FEB-2020
DEFINITION  Crucivirus-268 isolate 120_848_SvL1_3295.
ACCESSION   CruV-268
KEYWORDS    .
SOURCE      Crucivirus-268
  ORGANISM  Crucivirus-268
            Unclassified.
REFERENCE   1  (bases 1 to 3470)
  AUTHORS   de la Higuera,I., Kasun,G.W., Torrance,E.L., Pratt,A.A.,
            Maluenda,A., Colombet,J., Bisseux,M., Ravet,V., Dayaram,A.,
            Stainton,D., Kraberger,S., Zawar-Reza,P., Goldstien,S., Briskie,J.,
            White,R., Taylor,H., Gomez,C., Ainley,D.G., Harding,J.S.,
            Fontenele,R.S., Schreck,J., Ribeiro,S.G., Oswald,S.A., Arnold,J.M.,
            Varsani,A., Enault,F. and Stedman,K.M.
  TITLE     Direct Submission
  JOURNAL   Submitted (24-FEB-2020) Biology, Portland State University, 1719 SW
            10th Ave, Portland, Oregon 97201, USA
COMMENT     Bankit Comment: ALT EMAIL:ide@pdx.edu
            Bankit Comment: TOTAL # OF SEQS:451
            Bankit Comment: GAP: est. length - alignment

            ##Assembly-Data-START##
            Assembly Method       :: MetaSPAdes v. 3.12; IDBA-UD v. 1.1.1;
                                     MEGAHIT v. 1.0.3
            Sequencing Technology :: 454; Illumina
            ##Assembly-Data-END##
FEATURES             Location/Qualifiers
     source          1..3470
                     /organism="Crucivirus-268"
                     /mol_type="genomic DNA"
                     /isolate="120_848_SvL1_3295"
                     /isolation_source="Water"
                     /country="Norway"
                     /collection_date=2012
     CDS             1..1344
                     /codon_start=1
                     /product="Putative Replication Associated Protein"
                     /translation="MGAALINGGALCARPVGRAGCTSWVSRRWVWVNWSQTGPLDPMD
                     QGRWTNRHKPAALRSLAKHWVFTAWDEPQDGGVLPSWATFLCYGQEVCPDTGRGHWQG
                     YAELVDKRRVGPLRELFAGLGVGRLWLGMRLGTPSQAREYALKDGIPVELGVLSSESS
                     HQGERTDIQRIISEIEDGSMSSVIQGCRSGVIKNHQQLKVADRLLAFCEPKRNFRPEV
                     IVHWGPTSTGKTRLVMHQWPDAYVAPSCAPRWWPGYDGHEVLLVDDFRGSFMPFHELL
                     RFLDRYPYRIEFKGGDRQLVARTMVITSPFHPSRWYPNCGEQLAQLLRRISRIRYFFA
                     EVDESSDVSAIKYTETDGLAPPLTAPLEHLAGDPLTANLLVPDRDPEVGGNISNRCPP
                     WGGGFVAGDPRPDNVVVGAGPFWARDEVDFVSLFEEVLTDLPQGHGLKCLSMAEK*"
     CDS             complement(1403..2761)
                     /codon_start=1
                     /product="Putative CruV Capsid Protein"
                     /translation="MPYTQKTGRYYKIVAGKRQYDPRFDKKPSGARALTGRGDYKTTN
                     AKPKARKQAKRAPRTVSASAGSRLMLEGGGALLGGALFGPAGAAVGGMAGGALSSFMG
                     LGDYEIKENVFLGGRLPKMINDTPSGGTIIRHQEYLGDIITSATANTFNIQAFGLNPA
                     DEGTHPWLAQIAANYEEYEYEGLVFQFRSTSADALNSTNTALGSVIMATSYDAADPLF
                     ASKTEMLNYEYSCSAKPSESFLHMIECAPNQTVLGHQYCRPGLPPAGTDIRFFDLGKF
                     QIATTGFQGTSVNIGELHCTYQVRLLKPKLFVALGNDVNYAHSTNAIGVVATSPLGTP
                     SGRVVLADNLGLTYTDKAVTFPVSRVIENYWVYLSFVGTTSAAMTYPSVTLTNATSDP
                     TLGLNTPANGTTADAASMHFKITTLGNSTVPVITWGAAGTIPTGSTNIRLVINQSPNN
                     LV*"
ORIGIN
        1 atgggcgcgg cattaattaa cggtggggca ctttgtgcgc ggcctgtcgg tcgcgctggg
       61 tgcacctcct gggtttccag gaggtgggtg tgggtcaatt ggagccagac tggtcccttg
      121 gatccaatgg atcagggacg ctggaccaat cgacacaagc ccgctgcgct gcgctcgctc
      181 gcgaagcact gggtgttcac ggcttgggac gagcctcagg acggtggtgt ccttccctcg
      241 tgggcgacct tcctgtgcta tggccaggag gtctgccctg acactgggcg gggccactgg
      301 cagggctacg ccgagcttgt tgacaagcgt cgcgttggcc cgcttcgcga gctgttcgcc
      361 ggcctcgggg tcggccgcct ctggttgggg atgcgccttg gtactccctc gcaggctcgt
      421 gagtacgctc tcaaggacgg catcccagtc gagctggggg tgctctcctc cgagtcctct
      481 caccagggcg agcggactga catccaacgc atcatctcag agatcgaaga tgggtcgatg
      541 tcctccgtca tccagggctg ccggagcggg gtcatcaaga accatcagca gctcaaggtt
      601 gctgaccggc ttcttgcctt ctgtgagccc aaacgtaact tccgaccgga agtcatcgtt
      661 cactgggggc ccacctccac tggcaagact cgtcttgtca tgcatcagtg gcctgacgca
      721 tacgtggctc catcctgcgc cccgcgctgg tggccgggct atgatggtca cgaagtcctc
      781 cttgtggatg actttcgtgg gtcgttcatg ccgtttcatg aactccttcg gttccttgac
      841 cgctatccgt accggatcga gttcaagggg ggtgaccggc aactcgttgc ccggaccatg
      901 gtgattacat cacccttcca cccctctcgt tggtatccca actgtggcga gcagctggct
      961 cagctgctcc gccgtatctc ccggatccgt tacttctttg cagaagtaga cgaatcctcc
     1021 gatgtctccg ctatcaagta cacggagacg gacgggctgg cgcccccgct caccgctcct
     1081 ctggagcatc tcgctggaga tccgcttaca gcaaatctcc tggttccgga tcgggatccg
     1141 gaagtcgggg gtaatattag caaccggtgc ccaccctggg ggggtgggtt cgttgctggc
     1201 gacccccgac cggacaacgt tgttgtcggc gctggcccat tctgggcgcg cgacgaggta
     1261 gacttcgtct ccctcttcga ggaggtccta acggacctgc ctcaaggcca tggtctcaag
     1321 tgtctctcaa tggccgaaaa ataacgcacc gtcaccgacc tgccggagat tgtccggagg
     1381 tcggccgggc ctctttttgc acctatacta ggttgtttgg ggactggttg atcaccagtc
     1441 tgatgttggt cgatcccgtt gggatggtcc ctgctgcacc ccaagttatc actggaactg
     1501 tgctgttgcc cagcgtggtg atcttgaagt gcatcgacgc cgcgtccgct gttgtgccgt
     1561 tcgctggcgt gtttagcccg agcgtggggt cgctcgtggc gtttgtcagt gtgactgacg
     1621 ggtaggtcat cgctgcgctt gttgtaccta cgaaagacag gtacacccag tagttctcga
     1681 ttactcgaga aactgggaat gtcactgcct tgtccgtgta cgtcagtccg aggttgtcgg
     1741 ccaggactac acggccgctt ggagttccaa gcgggcttgt ggcaacgacg cctatggcat
     1801 tcgtggaatg cgcgtagttg acgtcgtttc ccagcgcgac gaacagtttt ggcttgagca
     1861 gcctgacctg gtaggtgcag tgcagctcgc cgatgttcac gctggtgcct tggaatccgg
     1921 tggttgcgat ctggaatttt cccagatcga agaaccggat gtccgtgcct gctggtggca
     1981 ggcctgggcg acagtactgg tggcccagca ctgtctggtt gggtgcacat tcgatcatgt
     2041 gcaggaagct ttcgcttggt tttgccgagc agctgtactc gtagttcagc atctcggtct
     2101 tcgacgcgaa gagcgggtct gccgcgtcgt aactcgtcgc catgatcacg gatcctagcg
     2161 ccgtgttggt gctgttcagg gcgtctgccg acgtgctcct gaactggaat actagccctt
     2221 cgtactcgta ttcttcgtag ttggcggcta tctgcgctag ccacgggtgg gtgccttcat
     2281 ctgcagggtt cagtccgaac gcctgtatgt tgaaggtgtt ggcggtggcg gaggtgatga
     2341 tatccccaag atactcctgg tgacggataa tcgttcctcc gctgggggtg tcgttgatca
     2401 tcttcggcag tctgcctccg aggaacacat tttccttgat ctcgtagtct ccgagcccca
     2461 tgaaactgct caacgcgcct ccggccatgc ctcctacggc ggcaccggcc gggccaaaca
     2521 gggctcctcc aaggagggct cccccccctt ccaacatcaa gcggcttcct gcggatgcgc
     2581 ttactgttcg gggggcccgt ttggcttgtt tgcgagcctt cggcttcgca ttggtcgtct
     2641 tgtagtctcc gcgcccggtg agcgctctcg caccagacgg cttcttgtcg aagcgggggt
     2701 cgtactggcg tttgccagct actatcttat agtagcgacc tgtcttctgc gtgtacggca
     2761 tggtgggtat gccctacttt gcgcgctcca attgcccgcc cttgtggggc tgggggcaac
     2821 cgttcgctgg ttgtggggtg tccgccccct cgtgggggct agccgggtgc gacgctgcct
     2881 agctatggcc cggggtgccc ccgggcctta tatagctagt tgctgcaccc gctgtcaccg
     2941 tgggccactg gtgtgtctcc cggcgggtgg gttgcgcaat tatttcacgc gcggcagagt
     3001 atgtgcgggc ggggctccac catctctgcg cgccctgctg cgcgaaatat ttacgcggcg
     3061 cgcgcttctg tgcggccggt gcgctcgact cgggtgtccc tgcctcggac agcgccggaa
     3121 taatttcgcg cccgcgctct tttgcgcgcg ttgacgtggc ggctggctcc gctcactggg
     3181 cggtcaatta tttttgtgcg gccgcgactt tgtgcggccg gtgcactcgg ctctgctgct
     3241 cagtggcagg cagcgccgaa ataatttcgc gctcgcgctc ttttgtgcgc gttggcgtgg
     3301 cggctatctc cgcgcactgg gcggtcaatt atttttgtgc gacgctgtct ttgctcggca
     3361 ggggtgccga gcgcgtggcc tggtcgcgcg cgggtgggtg ccccattaat tacgtggccg
     3421 ggtgactctg cggcgcaggt cgggcggcgg ctcgggcgag atgcgcgcgc
//
LOCUS       CruV-269                3475 bp    DNA     circular     24-FEB-2020
DEFINITION  Crucivirus-269 isolate 120_1356_IR2_3297.
ACCESSION   CruV-269
KEYWORDS    .
SOURCE      Crucivirus-269
  ORGANISM  Crucivirus-269
            Unclassified.
REFERENCE   1  (bases 1 to 3475)
  AUTHORS   de la Higuera,I., Kasun,G.W., Torrance,E.L., Pratt,A.A.,
            Maluenda,A., Colombet,J., Bisseux,M., Ravet,V., Dayaram,A.,
            Stainton,D., Kraberger,S., Zawar-Reza,P., Goldstien,S., Briskie,J.,
            White,R., Taylor,H., Gomez,C., Ainley,D.G., Harding,J.S.,
            Fontenele,R.S., Schreck,J., Ribeiro,S.G., Oswald,S.A., Arnold,J.M.,
            Varsani,A., Enault,F. and Stedman,K.M.
  TITLE     Direct Submission
  JOURNAL   Submitted (24-FEB-2020) Biology, Portland State University, 1719 SW
            10th Ave, Portland, Oregon 97201, USA
COMMENT     Bankit Comment: ALT EMAIL:ide@pdx.edu
            Bankit Comment: TOTAL # OF SEQS:451
            Bankit Comment: GAP: est. length - alignment

            ##Assembly-Data-START##
            Assembly Method       :: MetaSPAdes v. 3.12; IDBA-UD v. 1.1.1;
                                     MEGAHIT v. 1.0.3
            Sequencing Technology :: 454; Illumina
            ##Assembly-Data-END##
FEATURES             Location/Qualifiers
     source          1..3475
                     /organism="Crucivirus-269"
                     /mol_type="genomic DNA"
                     /isolate="120_1356_IR2_3297"
                     /isolation_source="Water"
                     /country="Norway"
                     /collection_date=2011
     misc_feature    join(3466..3475,1..20)
                     /note="stem-loop"
     misc_feature    1..9
                     /note="nonanucleotide"
     CDS             50..1441
                     /codon_start=1
                     /product="Putative CruV Capsid Protein"
                     /translation="MKRLSVPSESSTGRILVSKSSIRKSRMKHKNKNNTTLTIIGNKM
                     CDCGDTKSGKYFSKVGGQIGDRLQRKGMSLANQAGKRFKNWSGLGDYNIVFNSLIEGS
                     SNSNNIAAMSEGRGLIFKHKEYLGDIITSSASPGAFHVTKFVVNPGNVITFPWLASIA
                     QQHDQYKPLGVIFEFVSTASDTTSSATLGSIVMSTQYDIKDPDPANKSDMLNRAYASE
                     ARMSQNMVHGLECDPGELQNDLFYTLPLNDTVDDTRFYNMANFYVATQGGNLPVSTII
                     GSLYVHYEYEFFKQIPFNGVPCKGNLYSIYSATIPAASGISITSMVPSLTHVSGRNLG
                     ITTHVTGNGGFIIPKYWQGASFMIEIFYRDGLPRTSQAAATMAFVNCTATVSALLPAS
                     NNYYLLAPRSPAITYLDSYTAVFVNINSNIASDAQVSQGSYLGCNDQFSSASGGDVLV
                     YFTMVQKDYQVKA*"
     CDS             1966..3111
                     /codon_start=1
                     /product="Putative Replication Associated Protein"
                     /translation="MRPKNIKLTHLKSQIKMDEEILNEESLNESENTEDENIEDQNFD
                     IGSDDLEDGGARFYNFIFVKHYPTPEDKVKIQAVKAKYIIYGEEMTAGTAEEPPHEHL
                     QGFICFKSQTTVRSARKHLCGAYVRSKAHYSTFKQASDYCRGNYYKKKQNIWKPKNEV
                     VYERGTLPMDPKAKAAFGGLDYDLAKAEALAGKEVSNSRIFIQYYDTMAKIRMNGHAK
                     RYKQVTTIFELRPWQKVIVDMIKLPPNPRKIHWIVDVVGSAGKSELAKYLRLNNGAYV
                     FTNSKVENMAYDLPLPAPNIFIMNLTRQIEGRVPYSYLECIKDGEVFSTKYYPIRKSF
                     DSPHLFVFANFAPDMSVMSADRWDIIDVGAPTFNPQQATEAREFNNF*"
ORIGIN
        1 tataactaga gcgcttataa taaggacgag gagcagaaaa acgcatagat tgaaaaggct
       61 ttcggtaccc tccgaatcct cgactggacg gatattggtt tccaaatcct ccataagaaa
      121 atccaggatg aaacataaaa ataaaaataa cacaaccctc acaattattg gaaataaaat
      181 gtgtgactgt ggagatacaa aaagtggaaa atatttttcc aaagttggtg gacaaattgg
      241 cgaccgttta caacgtaaag gaatgtcatt agcaaaccaa gctggtaaaa gatttaaaaa
      301 ttggtctgga ctaggtgatt acaatattgt atttaattca cttatcgaag gatcatcaaa
      361 ttctaataac attgcagcta tgagtgaagg aagaggactt atatttaaac acaaggaata
      421 ccttggtgat attataacat cttctgcgag ccctggtgca tttcatgtaa ctaaatttgt
      481 agttaatccc ggtaacgtaa ttacgtttcc atggctagca tcaattgcac aacaacacga
      541 tcaatataaa cctttaggtg taatatttga attcgtgtca acagcatcag atacaacaag
      601 ctcagcgaca ttaggatcga tagttatgtc aacccaatac gatattaaag accctgaccc
      661 tgcaaacaaa tcagatatgc tcaatcgtgc gtatgcaagt gaagcccgta tgtcacaaaa
      721 catggtacat ggattagagt gcgacccagg cgaattacaa aacgatttat tttatacatt
      781 accattaaat gatactgtag acgatacccg attttacaat atggcaaatt tttatgtagc
      841 cactcaaggt ggaaatctcc cagtcagtac gattatagga tcattgtatg tacattacga
      901 atatgaattc ttcaaacaaa tcccttttaa tggcgtgcca tgcaaaggaa acttatattc
      961 tatatattca gctactatcc cagcagcctc tggaattagt ataaccagta tggtaccctc
     1021 tttgacacat gtatctggaa gaaatttagg aattactaca catgttacag gaaatggagg
     1081 ttttattatt cccaaatatt ggcaaggagc gtcatttatg attgagatct tttacagaga
     1141 cggattacca agaacatcac aagctgctgc cacaatggca tttgttaatt gcactgctac
     1201 cgtgagcgca cttttaccag catctaataa ttattatttg ttagcaccta gatcaccagc
     1261 aattacatat ttagatagtt acactgcagt atttgtaaac attaattcca atatcgcgtc
     1321 tgatgcacaa gttagtcaag gatcttatct cggatgtaat gaccaatttt cgagtgccag
     1381 tggcggagat gtattggtat actttactat ggttcaaaag gattatcaag ttaaagctta
     1441 atatataacg aagttttcga ggccgcaggc cgagttttaa agcattaacg caatcctcac
     1501 actttacttc atcctttctg ttagcgtggt tactatgcgc tgcggccagc ttacccagtg
     1561 acagttctga cacctaccct aaccctaacc ctaaccctaa ccctaaccca ctatgcgctg
     1621 cggccaggtt cactccacta tgcgctgcgg ccaggttcac tccactatgc gctgcggcca
     1681 ggttcactcc actatgcgct gcggccaggt tcactccact atgcgctgcg gccaggttca
     1741 ctccactatg cgctgcggct aggttcactc cactatgcgc tgcggctagg ttaattttaa
     1801 aaaaaaatgt atccggttca aaacttgggg gtaagtatcc ttattacctt acccccaagt
     1861 gagtcatgag ttttcagttc ccatctcttg gttacagact ttggattccg ttattgagca
     1921 gtctcgaacg aaactttaac ggaatccaag ttctcgaacc gtcgcatgcg accaaaaaat
     1981 ataaaactca ctcacttaaa atcacaaatt aaaatggatg aagaaatttt gaacgaagaa
     2041 agtttgaacg aaagtgaaaa tactgaagat gaaaatatcg aagaccaaaa ttttgacatc
     2101 ggctctgacg atttagaaga tggaggagct cgattttaca atttcatctt cgtcaagcat
     2161 tatccgacgc cggaggataa agtaaaaata caagcagtta aagccaaata cataatttat
     2221 ggagaagaga tgacggccgg tacggccgaa gaaccacccc atgaacatct tcaaggcttt
     2281 atttgcttca agagccaaac gacagtgaga tcagctcgta aacatctttg cggcgcctac
     2341 gtgcgcagta aagcacatta tagtaccttc aaacaagcca gtgattattg ccgtggcaat
     2401 tattacaaga aaaaacaaaa tatttggaag cctaaaaatg aggtcgttta cgagcgcggt
     2461 acgcttccaa tggatcccaa agctaaagcc gcctttggtg gattagatta tgatttagct
     2521 aaagccgagg ctttagctgg aaaagaagtt agtaattcgc gtatatttat tcaatattat
     2581 gacactatgg caaagattag aatgaatggg cacgcgaaac gatataaaca agtaacaaca
     2641 atatttgaat tgcgcccttg gcaaaaagta atagtagata tgattaaatt acctccaaac
     2701 ccgcgaaaaa ttcattggat cgtcgatgtg gttggttccg caggtaaaag cgaattagcg
     2761 aagtatttaa gactaaataa tggagcatac gtatttacca attcaaaagt tgaaaatatg
     2821 gcatatgatt taccattacc agcccccaat attttcatta tgaatttgac acgtcaaatt
     2881 gaaggcagag taccctattc ttatttagaa tgcattaaag atggtgaagt attttcaaca
     2941 aaatattatc caattagaaa atcatttgat tcacctcatt tatttgtatt tgccaatttt
     3001 gctccggata tgagtgtaat gagtgctgat cgatgggata ttatagacgt aggcgctcct
     3061 acatttaatc cgcaacaagc aacagaagca agagaattta acaattttta atgatataat
     3121 taatcatctc tcattctttt agaaccaact ttatcaacgg cacccgttgt aacaagagca
     3181 gtaacagcat caaacattat agcgtctaca ccatctttat cataaaacat gaaattagtc
     3241 ctatcactcc tatgcttgat cacatattta gccatcgctt taagattatc acttttaaaa
     3301 gtgccaccaa gacgattaac tccatcttca ctaacaacta agcaggtata accacttgct
     3361 ggtttaacgt gtaatacggc gggaacgttg atgagtggct tattggatgt cttgtaatgc
     3421 actttggatt taataagctt tcggcaagat ttaacagcag ctgacttata agatt
//
LOCUS       CruV-270                3477 bp    DNA     circular     24-FEB-2020
DEFINITION  Crucivirus-270 isolate 120_133_SvL2_3298.
ACCESSION   CruV-270
KEYWORDS    .
SOURCE      Crucivirus-270
  ORGANISM  Crucivirus-270
            Unclassified.
REFERENCE   1  (bases 1 to 3477)
  AUTHORS   de la Higuera,I., Kasun,G.W., Torrance,E.L., Pratt,A.A.,
            Maluenda,A., Colombet,J., Bisseux,M., Ravet,V., Dayaram,A.,
            Stainton,D., Kraberger,S., Zawar-Reza,P., Goldstien,S., Briskie,J.,
            White,R., Taylor,H., Gomez,C., Ainley,D.G., Harding,J.S.,
            Fontenele,R.S., Schreck,J., Ribeiro,S.G., Oswald,S.A., Arnold,J.M.,
            Varsani,A., Enault,F. and Stedman,K.M.
  TITLE     Direct Submission
  JOURNAL   Submitted (24-FEB-2020) Biology, Portland State University, 1719 SW
            10th Ave, Portland, Oregon 97201, USA
COMMENT     Bankit Comment: ALT EMAIL:ide@pdx.edu
            Bankit Comment: TOTAL # OF SEQS:451
            Bankit Comment: GAP: est. length - alignment

            ##Assembly-Data-START##
            Assembly Method       :: MetaSPAdes v. 3.12; IDBA-UD v. 1.1.1;
                                     MEGAHIT v. 1.0.3
            Sequencing Technology :: 454; Illumina
            ##Assembly-Data-END##
FEATURES             Location/Qualifiers
     source          1..3477
                     /organism="Crucivirus-270"
                     /mol_type="genomic DNA"
                     /isolate="120_133_SvL2_3298"
                     /isolation_source="Water"
                     /country="Norway"
                     /collection_date=2012
     CDS             1..1419
                     /codon_start=1
                     /product="Putative Replication Associated Protein"
                     /translation="MKQNSYYLTKLAEYQKLEQEIGWVQPDQYETPAAKAKAAFILHK
                     ETEKTDKKDNINFISKKPTGAKFRLNCLRVFFTYKTHINMDEFIKWLDAKHPLESWII
                     CHEKSDKTNAYEHTHASVEFKTKINISDCRHFDYGIDPADGKIIHPNFSAVTKWSGSI
                     AYCIKQQPEAIYKSNFDVVAFLKKNKRNTELSPEQWKELVTKIVSQKNALEAIFNCAK
                     SLKDVSAINTIYGARVRGMDPLLIEEFKNFIPRPWQDDLVKILRSPQNNDDIYWIIDK
                     AGGKGKSTFLDWWELQPEGNSTVFMTDIGKPSDMNDYMKNWMDQDASRWPRVILINLP
                     REFNVFNEIYKVIEVLKDGRITCTKYKACPLKYKRPHVVIFANAEPKVTGMSLRKWKL
                     LGFDKIDGEDRLVSINTETLATKQKAKAKATKVTGGRNIMDYVLPPSSKENLKLLDPF
                     IESDDESSSIPIDEIAFIEDKD*"
     CDS             complement(1768..3162)
                     /codon_start=1
                     /product="Putative CruV Capsid Protein"
                     /translation="MPALVKRNYRPRRMTTAPYRRRRTPYSYSRTPRVGSAPRRRYTR
                     SYQNSGDAIRPPPTTAPTSNWGSNIGGGIGSLFGPAGTALGSLLGHGAQSLVKMVTGF
                     GDYSIQRNSLMPGSLEPPIINNSSKIGSKGGIMCRHREYIGDINPSIAFTVQTYAINA
                     GLVSTFPWLSQMASSFEEYCIHGMLFEFKSMSSDAVLSTAANSSLGTVIMATQYNALN
                     PAFADKREMENYEFSNSAKPSESFIHPIECKGSETPIKCLYVRTGAVPAGGDVRLYDI
                     GDFSIATQGMQSAAGVIGELWVTFEIEFFKPKLLAGLGLELLMDHYDIPVATVTSARP
                     FGTSRITPTLNSTLGSQVLGNLSPAVVVFPPYITDGTFLIEYNCTGTSAPLNPPVVVA
                     VSNCTLGTGFFNNQTLGSVFTPTGTTGPVYIYVGLVIITGPSATLTFGNAGTLPTSPT
                     AADFIITQINANVS*"
ORIGIN
        1 atgaaacaaa attcatacta tctcacaaag ctagctgaat accaaaaact cgaacaagag
       61 attggatggg tccaacctga ccaatacgag acacccgctg caaaagccaa agcagctttt
      121 atattacata aagaaacaga aaagactgat aaaaaagata acataaattt catcagcaaa
      181 aaacccacag gagcaaaatt cagactgaat tgtctaagag tttttttcac atacaaaact
      241 cacataaata tggatgagtt tataaaatgg ctagatgcaa agcatccact agaatcatgg
      301 ataatatgcc atgaaaaaag tgacaaaact aatgcatatg aacacacaca tgcatcagta
      361 gaattcaaaa ccaaaatcaa tatatccgac tgcagacatt ttgattatgg aatcgatcca
      421 gcagatggaa aaataatcca tcctaacttc agtgcagtta ccaaatggtc tggatctata
      481 gcatactgca taaagcagca acctgaagca atatacaaat caaactttga tgtagttgct
      541 ttcttaaaaa aaaataagag aaacactgaa ctttcacctg aacagtggaa agaattagtt
      601 actaaaattg tttctcaaaa aaacgcactc gaagcgattt ttaattgcgc aaaatcgctt
      661 aaagacgtga gcgcaataaa tacaatttac ggtgccagag tacgtggaat ggatccacta
      721 ctaatcgaag aattcaaaaa cttcattcca cgaccatggc aagatgactt agtaaaaata
      781 ctaagatctc cccaaaacaa cgatgacatc tattggataa tagataaagc cggcggcaaa
      841 ggcaaaagca ccttcttaga ttggtgggaa ctccaacctg aaggtaacag cacagttttc
      901 atgacagata tcggaaaacc atccgatatg aacgactaca tgaaaaactg gatggatcaa
      961 gatgcatccc gctggccacg agtaattctc ataaacttac caagagaatt caatgtattt
     1021 aatgaaatat acaaagttat tgaagtccta aaagacggcc gaataacttg tactaaatac
     1081 aaagcttgtc cattaaaata caagcgacca catgttgtta ttttcgcaaa tgcagagcct
     1141 aaagtgacag gaatgtcact acgaaaatgg aaacttctcg gcttcgataa aatcgatgga
     1201 gaagaccgct tagtctccat taacacagag accttagcta ccaaacaaaa ggctaaagct
     1261 aaagccacta aagtgaccgg ggggcgtaac attatggact atgtcctgcc cccctcatct
     1321 aaagagaatc tcaaattact agatcctttc atagaaagtg atgatgaaag ttcatctata
     1381 ccaatagatg aaatagcctt cattgaagat aaagattaat taatttctta tatataataa
     1441 attaattatt cctcaaataa ttgactaaag tcgctgacgc gacagtcccg ctcgccgcgg
     1501 ggcaaagttc gctacgctca caacaacatc cacaagtggg ttgcgagtcg ctacgctccc
     1561 gcaagccact tatagatatt gtagaaggga cctgcgcggt ccctcgaacc ctggcgccca
     1621 cgctggggcg aggtctccaa aaaagcgcta gcgcgctttt tttacgaccc gggattaatt
     1681 acttacttac ttaattttat taaaaatact atcatcataa tatttttaat taaaacgata
     1741 accataggaa ttaccgcgaa tattaaatta agatacattg gcattaattt gagtgattat
     1801 aaaatctgca gccgtaggtg aagttggtaa tgtaccagca ttcccaaatg ttaatgtagc
     1861 actcggacca gtaatgataa caagaccaac ataaatgtat actggaccag tcgttccagt
     1921 tggagtaaaa acagaaccta aagtctgatt attaaagaaa cctgtaccaa gagtacagtt
     1981 actaacagct actacaaccg gcggatttaa tggagctgaa gtcccagtac agttgtattc
     2041 tataagaaac gtcccatcag taatatatgg tggaaaaact acaacagctg gagataagtt
     2101 tcctaaaact tgagatccta aagtagaatt taatgtaggt gttatacgac tagtaccaaa
     2161 cggacgggca ctagtaacag ttgctacagg tatgtcataa tgatccatta ataattccaa
     2221 acctaaacca gcaagtaatt ttggcttaaa aaactcaatc tcaaaagtaa cccataattc
     2281 accgataaca ccggcagctg attgcatccc ttgagtcgca atactaaaat caccaatatc
     2341 atataatcgc acatcaccac ctgctggcac agctccagta cgaacataaa gacatttaat
     2401 tggtgtctca ctacccttac attcaatagg atgaatgaat gattcagatg gctttgctga
     2461 attagaaaat tcataatttt ccatctcacg tttatccgca aaagcgggat taagagcatt
     2521 atactgagtt gccataataa cagttcctaa agaactattt gcagcggttg acaaaacagc
     2581 atcagaactc atactcttaa attcaaacaa cattccatga atacaatatt cttcaaatga
     2641 actagccatt tgagataacc aagggaatgt tgacacaaga ccagcattta ttgcatatgt
     2701 ttgaacagta aatgcaatag atggatttat atcaccgata tactctcgat gacgacacat
     2761 aattccaccc ttagaaccta tcttactact attattaata ataggtggtt caagagaacc
     2821 aggcatcaac gaattacgct gaattgaata atcaccaaat ccagtaacca tcttaactaa
     2881 actctgggca ccatgaccta aaagcgaacc cagagctgtt ccggctggcc caaataatga
     2941 gcctatacca ccaccaatat tggatcccca attacttgtt ggagcagtgg ttggtggagg
     3001 ccggatagca tcaccagaat tttgataact cctagtatat cgacgacgag gcgccgatcc
     3061 aactctcgga gtacgagagt aagaataagg agttctcctt cgacgataag gagcagttgt
     3121 cattcgtcgc ggacggtaat ttcgtttaac taatgcgggc attttgtttg tatattatta
     3181 tataatattt taatttttta gtaatacccc ataaacgcca aatttgcaaa aacgccaaat
     3241 ttgtttttta aaaaacgcca aatttgtata ccaaaaaata gatacagcat aacaacacgc
     3301 gccaaatttg ctgaaaaacg ctgaaataac atattactct tataataaaa attattttta
     3361 tattccaacc attttgccac ccaatttact atagtaaatt attattaaaa ctcacaggca
     3421 aaatcgcgtt tgagactcta aaaataaaat ctcacgagtc tcatatacgc gccacaa
//
LOCUS       CruV-271                3479 bp    DNA     circular     24-FEB-2020
DEFINITION  Crucivirus-271 isolate 120_1311_IR2_3297.
ACCESSION   CruV-271
KEYWORDS    .
SOURCE      Crucivirus-271
  ORGANISM  Crucivirus-271
            Unclassified.
REFERENCE   1  (bases 1 to 3479)
  AUTHORS   de la Higuera,I., Kasun,G.W., Torrance,E.L., Pratt,A.A.,
            Maluenda,A., Colombet,J., Bisseux,M., Ravet,V., Dayaram,A.,
            Stainton,D., Kraberger,S., Zawar-Reza,P., Goldstien,S., Briskie,J.,
            White,R., Taylor,H., Gomez,C., Ainley,D.G., Harding,J.S.,
            Fontenele,R.S., Schreck,J., Ribeiro,S.G., Oswald,S.A., Arnold,J.M.,
            Varsani,A., Enault,F. and Stedman,K.M.
  TITLE     Direct Submission
  JOURNAL   Submitted (24-FEB-2020) Biology, Portland State University, 1719 SW
            10th Ave, Portland, Oregon 97201, USA
COMMENT     Bankit Comment: ALT EMAIL:ide@pdx.edu
            Bankit Comment: TOTAL # OF SEQS:451
            Bankit Comment: GAP: est. length - alignment

            ##Assembly-Data-START##
            Assembly Method       :: MetaSPAdes v. 3.12; IDBA-UD v. 1.1.1;
                                     MEGAHIT v. 1.0.3
            Sequencing Technology :: 454; Illumina
            ##Assembly-Data-END##
FEATURES             Location/Qualifiers
     source          1..3479
                     /organism="Crucivirus-271"
                     /mol_type="genomic DNA"
                     /isolate="120_1311_IR2_3297"
                     /isolation_source="Water"
                     /country="Norway"
                     /collection_date=2011
     CDS             1..933
                     /codon_start=1
                     /product="Putative Replication Associated Protein"
                     /translation="MTNSKLRSWEIEINNFDEVDIEEIFNQTEICDYSIVGFEGCDPL
                     SEYHPHIHCFMHYPNPISKSGLLKRFTRKPHVDLVRSPQRYVDYCKGYEDGVLKEPLV
                     GENIYFESGIPPISGINKKHNSEFVMKGIKEGKSLVELRDLYPMYMMHHESKVSAFID
                     KTRNIPPIQFYYINPISDVITEVQEIFGNKFEYVFVTELDELEAYDNYDVVVFMSEMF
                     RPIHALWPRGCKLTYKYGYQVKQIRCSKFVYVRGRYMDCDDEFKYYKKVNSDYGIDDK
                     PNMDVLHREITISNSPKKSKKSKKRSIPCGPFFE*"
     CDS             1259..2518
                     /codon_start=1
                     /product="Putative CruV Capsid Protein"
                     /translation="MPPKRKGKKKVYKKKSTSKAHRKSSFFAPVGKMIGGAAGGYIGG
                     PAGALIGSQLGSSAGSLISKITGFGSYHVSKNTIITNSIPQFQNRAGDGSIRLKHKEF
                     IADVKSSPTPGTFLPITYALSASDANTFPYLSQLAINFEEFAFEGLVFEYITSSGSLS
                     TTGQLGTVVGAVQFNSLAQAYLNKQQMEASTFGQSTVASQSCLFPVECDAMQTPSNGI
                     FYNSRPGITNVNNDKRWSQLGNFTIATVGMPLASETVGELYVSYDCVLMKPILIQDSG
                     SLADHWIASTGIVAPTNYFGTNAVLTAQSDNFTSLTPTTITFNPSFQGKVQVSYAVHG
                     ALGAWADPLMAVGAGVTALNILDSNITYQYLKVGDAATEMQLFTVAYFIISSVGGARP
                     VITLSGGAIFAPDYMDLIITSIPADFD*"
ORIGIN
        1 atgaccaatt ctaagcttag aagctgggaa atagaaataa ataatttcga tgaagtagat
       61 attgaagaga ttttcaatca gactgaaatt tgcgactatt ctattgtcgg ttttgaagga
      121 tgcgatccgt tatctgaata tcatccacat attcattgtt ttatgcacta tcctaatcct
      181 atttcaaaaa gtggacttct taaacgtttt acacgaaaac ctcacgtaga tctagtccgt
      241 agtcctcagc gctatgtcga ttactgtaaa ggctatgaag acggagttct gaaagagccc
      301 ttggttggcg agaatatata tttcgagagt ggaattcccc ctatttctgg gataaataag
      361 aaacataatt ctgaatttgt tatgaaagga ataaaagaag gaaaaagtct cgttgaatta
      421 cgagatcttt atccgatgta tatgatgcat cacgaatcta aggtttctgc ttttatagat
      481 aaaaccagaa atattcctcc aatacagttc tattatataa atcctatttc agacgttata
      541 acggaagttc aagaaatatt cggtaataaa ttcgaatatg tttttgttac tgaattagat
      601 gaattagagg cttacgataa ttatgatgtt gtagttttta tgtctgaaat gtttcgacca
      661 atacacgcat tatggccccg gggctgtaag ctcacatata agtatggtta tcaagttaaa
      721 cagattagat gttcaaaatt tgtatatgta cgtggacgtt atatggactg cgacgatgag
      781 tttaagtatt ataagaaagt taattcagat tacggaattg atgataaacc taatatggat
      841 gttttacatc gtgagattac tataagtaat tcacctaaaa aatctaagaa atctaagaag
      901 cgttcaatac cttgtgggcc attttttgaa taatgaataa aatacattta cttagtcaac
      961 tgacggtcga aatttggatt tctcagtaat atacagagat gaataattta ttgaaaatat
     1021 tcatggatta tgataaaaat aaaatatttt tattatatag tatgaatatt cccaataaag
     1081 aattcttcga taataacggt tatgatattt cattatatcc gtcgggcgcg atgattgagg
     1141 aaatgaccga ggcagaatgg cgagctttta tcaataatta cggcgactgg cgcttaaatc
     1201 ctgctttaca aaatttcatc gaacgaataa gaaataaaat atattcttag agtataagat
     1261 gcctccgaaa agaaaaggaa aaaagaaagt ctataaaaag aaaagtacat ctaaggcgca
     1321 ccgtaagtct tcattttttg cacctgttgg aaaaatgata ggaggtgctg ctggtggtta
     1381 tattggtggt ccagctggtg cacttatcgg atcacaactc ggatcaagtg ctggaagttt
     1441 aatttctaaa attactggat ttggatcata tcatgtatct aaaaatacta tcattacgaa
     1501 tagtattccg caattccaga atagagcagg tgatggttca atacgactta aacataagga
     1561 gtttattgct gatgtaaaat catctcctac tcctggtact tttttaccta ttacatatgc
     1621 gttatctgca tctgatgcaa acactttccc atatttatcg caacttgcta taaattttga
     1681 ggaatttgca tttgagggat tagtatttga atatataaca tcatcaggat cgctttcaac
     1741 tacaggacag ttaggtactg ttgttggagc agttcagttc aattccttgg cccaggctta
     1801 tttaaataaa caacagatgg aagcatctac gttcggtcag agtacggttg ctagtcaatc
     1861 ttgtttgttt ccagttgaat gtgatgctat gcagactcct tcaaatggaa tattttataa
     1921 ttctcgtcca ggaataacta atgtaaataa tgacaaacgc tggtcacagt taggaaattt
     1981 tactattgct acagttggta tgccacttgc tagtgaaact gttggagaat tgtatgtatc
     2041 atatgattgt gttttaatga agcctatttt gattcaagat agtggttctt tagcagatca
     2101 ttggattgca tcaactggta ttgttgcgcc aaccaattat tttggtacaa atgctgtttt
     2161 aactgctcaa tcagataatt ttacatcatt aactccaaca acaattactt ttaatcccag
     2221 tttccagggg aaagttcaag taagttatgc agtacatggt gcactcggag cttgggcaga
     2281 tccactcatg gctgtaggag ctggtgtaac agcgcttaat attttggaca gtaatatcac
     2341 gtatcaatat cttaaagttg gagatgccgc tacagaaatg caacttttca cggtcgcata
     2401 ttttataata tcgtctgtag gaggtgcacg tccagtaata actttatctg gaggtgcgat
     2461 atttgcgccc gattatatgg atttaattat aacatccatt cccgctgatt ttgattaata
     2521 atatatatca ttaataaaaa tcatcgtaaa gtaacagcga ataacttaat ttaacgcgaa
     2581 gcgacatggc ttgctttagc tcgttaacag aggccgtagg ccgaggataa tttataacat
     2641 tcgctgtata atacgatcat cgcgatgtta ttgggtccag ggcaaagcct tggtcacgga
     2701 gcgcgatttc gcgcgggctg aaagccagaa atcaatgaag tagtgaattg atttcgtaag
     2761 ataacacagt agtgtgatca aacataatcg cggcagcgaa cgcgaagcga gcgcgaaagc
     2821 gattatggtg ctgagtgatt agtctatcaa gcattagaaa tcccgattct atcgggattt
     2881 atcatgcggc cgggagaagg actatccgat tctatcggat tcctgaacag gccctcatta
     2941 gaattcgcga agctagcgaa ttatcatgac cacggtgatt agaatgccgt aggcattatc
     3001 atcaatttat tagaatgccg taggcattat cataaatagt ggcgcgaagc tcgcgactgg
     3061 ctgggccagg cgtaacccaa ctcgtaagac gccgacgagg cgtctttaag agttcacgct
     3121 gataatctga tatatcatat aagattattt ccgatatttt tattttgtga aaacatatag
     3181 aaaaatttcc gatattttta ttttgtgaaa acatatagaa aaaatcatca aaggctctag
     3241 ggaaggcgcc agcctagtat tacccctaga gccctgttcc gtgtgccaac acggacagct
     3301 agaatgccca cagcttgctg tgcgaccagg ggggaatttg cgaagcaaag gagggagcct
     3361 gttgtatgat aatttctctt tttaatattt tatagttttt catatagtaa aaactttttt
     3421 tggatcatta tttgaagacg ttccaaataa ttaaataatt tcttcttgta tattatacg
//
LOCUS       CruV-272                3479 bp    DNA     circular     24-FEB-2020
DEFINITION  Crucivirus-272 isolate DFL_65.
ACCESSION   CruV-272
KEYWORDS    .
SOURCE      Crucivirus-272
  ORGANISM  Crucivirus-272
            Unclassified.
REFERENCE   1  (bases 1 to 3479)
  AUTHORS   de la Higuera,I., Kasun,G.W., Torrance,E.L., Pratt,A.A.,
            Maluenda,A., Colombet,J., Bisseux,M., Ravet,V., Dayaram,A.,
            Stainton,D., Kraberger,S., Zawar-Reza,P., Goldstien,S., Briskie,J.,
            White,R., Taylor,H., Gomez,C., Ainley,D.G., Harding,J.S.,
            Fontenele,R.S., Schreck,J., Ribeiro,S.G., Oswald,S.A., Arnold,J.M.,
            Varsani,A., Enault,F. and Stedman,K.M.
  TITLE     Direct Submission
  JOURNAL   Submitted (24-FEB-2020) Biology, Portland State University, 1719 SW
            10th Ave, Portland, Oregon 97201, USA
COMMENT     Bankit Comment: ALT EMAIL:ide@pdx.edu
            Bankit Comment: TOTAL # OF SEQS:451
            Bankit Comment: GAP: est. length - alignment

            ##Assembly-Data-START##
            Assembly Method       :: MetaSPAdes v. 3.12; IDBA-UD v. 1.1.1;
                                     MEGAHIT v. 1.0.3
            Sequencing Technology :: 454; Illumina
            ##Assembly-Data-END##
FEATURES             Location/Qualifiers
     source          1..3479
                     /organism="Crucivirus-272"
                     /mol_type="genomic DNA"
                     /isolate="DFL_65"
                     /isolation_source="Drangonfly larvae"
                     /country="New Zealand"
                     /collection_date=2012
     CDS             1..873
                     /codon_start=1
                     /product="Putative Replication Associated Protein"
                     /translation="MPYRTKSGIKARYKYACFTDFVQLNHENYFTNKMDNVSYGVAGY
                     EICPDTKKPHIQGYVEFIKPINRDQIQTILGNKKMHVTERWATAQQAADYCKEDGTFT
                     EVGTISKSEQGKRNDLIDIRDKIINGISDKDLVMEHTEDYARHYKCFDRWRYMFQPHR
                     TWKTEVHILWGEPGCGKSHRAVLEEGGELISFENKFVLGYKNQEVVVLDDFNPGMIPL
                     EFMLRLTDKYHMVVNVKGGEMTWNPKKIYITTNRNPEAWYGKAGQWLRRVTSVTKLGD
                     DAYTGNNVDNIAGI*"
     misc_feature    32..50
                     /note="stem-loop"
     misc_feature    37..45
                     /note="nonanucleotide"
     CDS             complement(1916..3376)
                     /codon_start=1
                     /product="Putative CruV Capsid Protein"
                     /translation="MSFSDILSFSKFSLLCIFTFSFLLCNFESLRRLFFLNFFQKFSP
                     LFLFLFKGTISTIRIINYSTMPFVPRKKIIKRKRPTTRYMRPKAVKRVRKARAVPYSK
                     AISPASRPSIGSQIGGYLGNAAQNMFSKFLGFGAYDLKQNNMVQTLTPSNQVPFMKSS
                     STSTRIRHREFIQDFVGSSTPFAATYYPINIGMAQTFPWLSSIARNFSSYRFHGLIFE
                     FKSLSGDDQNKQSQGYVAGAVDYNSDSPFWVTKSQIENAMFSASSKPSTDIIFPVECD
                     PKHNQVSEFYVRSSALASTSSELLYDIGTFTIARGSNLNSTDTIGELWVSYDVELISP
                     ILGAGQLALPSAHYTSTNYTNLLPLGSTRTTVFDNMGLTISSTTITFPLGTLGKFALY
                     VQQTGANTGNVVPGSVSFNAGATALISLQNGSASGISAPLYTGAISGPNTSVYTTFTI
                     PSATVQAIITIGTAGTLPTSPSSCDIFVMPLNATLT*"
ORIGIN
        1 atgccttatc gaacgaagtc aggaatcaaa gcacgctata aatacgcgtg tttcacggat
       61 ttcgtgcaac tcaatcatga aaattatttc actaacaaaa tggataacgt gtcctatgga
      121 gtggcgggat acgagatctg cccggacact aagaaacctc atatccaggg atatgttgaa
      181 tttataaaac ccattaacag ggaccaaatt caaacgatct tgggaaacaa aaagatgcat
      241 gtgaccgaaa ggtgggcaac cgcgcagcaa gctgctgatt attgcaaaga agatgggact
      301 ttcacagaag ttgggaccat aagtaagtca gaacaaggga agaggaatga tctcatagat
      361 attcgtgaca aaattatcaa tggcatctca gataaagact tggtgatgga acacaccgaa
      421 gactacgcgc ggcactataa gtgttttgat cgttggcgct atatgtttca accgcacaga
      481 acctggaaaa cagaagtaca tattttatgg ggagaaccag gatgcgggaa gtcgcacaga
      541 gccgtgctcg aagaaggggg agaattgatt tcattcgaaa ataaattcgt actaggttac
      601 aagaaccaag aagtggtggt gttggacgat ttcaatccag gaatgattcc gttggaattt
      661 atgttgcgat taaccgacaa gtatcacatg gttgtgaatg tgaaaggagg agagatgacc
      721 tggaacccga agaagatcta tataacaaca aatcgaaacc cggaggcgtg gtacgggaag
      781 gccgggcagt ggctccggcg cgtgacttcc gtcactaaat tgggggatga cgcgtacaca
      841 ggaaataacg tcgataatat cgcagggata taaaagtttt aacagttcaa tatattttag
      901 tttatcaaaa aacgtattca tttcattcat gttagtcgcg cttcgcgctc tcgcactacg
      961 tgctcagtcc tggcgcttcg cgctgcggac ctcgtttcac tcgggtcgcc ttcggctctc
     1021 acgtcgcgct tcgcgctcct tctcgcttcg ctcgagttcg gcttcgcctc acgggctcgc
     1081 tgcgctcgcc gcaatcgctt cgctctcgct gatcgttggg atcgttgggg tagtggggtg
     1141 tttcctcggc ttggggcctc gtcaaggtgt ttcgcctgaa cggctcaagt cgcgctgcgc
     1201 gctccatcgt tgggatcgtt ggggttgtgt ggtgtttcct cggcttgggg cctcgtcaag
     1261 gtgtttcgcc tgaacggctc aagtcgctcg tacctcgctc catcgttggg atcgttggaa
     1321 ctgtagttat tgttataatc gttgaagatc gtaggcgctc gcattggctc gcttaattcg
     1381 tcgccttttt gggctcctca tacaccgcag gggtgataac gtgcacgtgc acgtgcactg
     1441 aagtggctgg taatactagg gctggctcgt tgcactcgct gcgcccttca gccacttcct
     1501 agagggatcg taggatttct agggatcgaa ggatcgaaga gatgatgact acgttcgggg
     1561 ctgcgcgccc tcactgcgcg ctgcgcttgc ctacgttacg gcgctacgcg gcctccactg
     1621 cgcggtcgta cctcccttgc ctgcgcgcgc tcgtacctcg cacgctgcgc cgccgctata
     1681 gtcgctgcgc tccttcgctc tgttgcgggg ggttagatag gttggctggt ttggtggtga
     1741 ggtgctagat gtgtgctttt gggctttttg ctcaccgcaa aaaactgcac cgcgctgcgc
     1801 gccgcccatg aaggtgccga aaatttctcg tagggttcgt aagaaattta attcacaaag
     1861 tgaatcgaat atgaaaaaaa accatttatt ggaaaaaatt aagtatagaa agaatttaag
     1921 ttaacgttgc gttcaacggc atcacgaaga tatcgcaaga cgacggagat gttggaaggg
     1981 tgccagctgt accgatggta atgatcgctt gtacggtcgc actcgggata gtaaacgtag
     2041 tgtacaccga ggtgttagga cccgaaatag caccggtgta gagaggtgct gagatgccgg
     2101 acgcgctccc gttttggaga gaaataagag ccgtagcacc agcgttaaaa gacactgatc
     2161 cagggaccac gttgcctgta tttgctcctg tttgctgaac gtataacgca aacttaccaa
     2221 gagtaccaag ggggaacgta atcgtggtgc tcgagatcgt caggcccata ttgtcgaaga
     2281 ccgtagtgcg tgtagatccg agagggagaa ggttggtata attcgtagaa gtataatgcg
     2341 cggatggaag cgcaagttgg cccgcgccaa gaatagggga aatgagctca acgtcatatg
     2401 aaacccaaag ctctccgatc gtatcagtcg agttcaagtt gctgccgcgc gcaatcgtga
     2461 aggtaccaat gtcgtaaaga agctcggaag aagtagaggc aagagcgctc gaacgaacgt
     2521 aaaattcgga aacctgattg tgcttagggt cgcactccac agggaaaata atatcagtcg
     2581 atggtttgct ggacgccgaa aacatcgcgt tttcgatctg tgacttagtt acccagaaag
     2641 gactatcgga gttgtagtcc accgcaccgg ctacatagcc ttgagactgc ttgttctgat
     2701 cgtcaccaga aagagatttg aactcaaata tcaaaccatg gaaccggtat gaagagaagt
     2761 ttcgcgcaat actgctcaac caagggaatg tttgggccat accgatatta attggataat
     2821 atgtcgcagc aaatggagta gaagaaccga cgaagtcttg gatgaactca cgatggcgaa
     2881 tcctcgtcga cgtactcgat gatttcatga acggcacttg attcgaagga gtaagggtct
     2941 gaaccatgtt gttttgtttg aggtcgtacg ctccaaaacc gagaaacttg ctgaacatgt
     3001 tttgtgcagc attacccaag tagccgccaa tttgactgcc aatagaaggg cgcgatgcag
     3061 gtgagatcgc tttggagtag gggacagcgc gcgccttacg aacacgttta acagctttgg
     3121 gccgcatgta acgagtagta ggacgcttac gcttaataat ttttttccga ggaacgaaag
     3181 gcattgttga ataatttatg attctaatag tagaaatcgt tcctttaaat agaaataaaa
     3241 ataggggtga aaatttttga aaaaaattta gaaaaaatag tcgtcttaaa gactcgaaat
     3301 tacatagtag aaaagaaaaa gtaaaaatgc aaagcaatga aaatttcgaa aacgacagga
     3361 tgtcagaaaa cgacatcaat tacgcgctgc tcacgccgcg cccagcgcct gacggcgtgg
     3421 atgaagaagt tgttgttatt gaaaatccta agaaacgtcg aatctcagat acggaagaa
//
LOCUS       CruV-273                3485 bp    DNA     circular     24-FEB-2020
DEFINITION  Crucivirus-273 isolate 001479_virusbaton.
ACCESSION   CruV-273
KEYWORDS    .
SOURCE      Crucivirus-273
  ORGANISM  Crucivirus-273
            Unclassified.
REFERENCE   1  (bases 1 to 3485)
  AUTHORS   de la Higuera,I., Kasun,G.W., Torrance,E.L., Pratt,A.A.,
            Maluenda,A., Colombet,J., Bisseux,M., Ravet,V., Dayaram,A.,
            Stainton,D., Kraberger,S., Zawar-Reza,P., Goldstien,S., Briskie,J.,
            White,R., Taylor,H., Gomez,C., Ainley,D.G., Harding,J.S.,
            Fontenele,R.S., Schreck,J., Ribeiro,S.G., Oswald,S.A., Arnold,J.M.,
            Varsani,A., Enault,F. and Stedman,K.M.
  TITLE     Direct Submission
  JOURNAL   Submitted (24-FEB-2020) Biology, Portland State University, 1719 SW
            10th Ave, Portland, Oregon 97201, USA
COMMENT     Bankit Comment: ALT EMAIL:ide@pdx.edu
            Bankit Comment: TOTAL # OF SEQS:451
            Bankit Comment: GAP: est. length - alignment

            ##Assembly-Data-START##
            Assembly Method       :: MetaSPAdes v. 3.12; IDBA-UD v. 1.1.1;
                                     MEGAHIT v. 1.0.3
            Sequencing Technology :: 454; Illumina
            ##Assembly-Data-END##
FEATURES             Location/Qualifiers
     source          1..3485
                     /organism="Crucivirus-273"
                     /mol_type="genomic DNA"
                     /isolate="001479_virusbaton"
                     /isolation_source="Water"
                     /country="France"
                     /collection_date=2013
     CDS             join(complement(1..43),complement(3139..3485))
                     /codon_start=1
                     /product="putative ORF"
                     /translation="MSSTLITRGGSGNTSRPPLYPRDQRRTRRTIPLFFDHLHGGRCY
                     VARWVRICDADFPCGVGGWVSLGGRHVRDAAVNRFAILRLTRNVRPVTQGPPPRASCG
                     HQRNLEKGIYAIYASLRSAGPISHLGG*"
     misc_feature    join(3475..3485,1..19)
                     /note="stem-loop"
     misc_feature    1..9
                     /note="nonanucleotide"
     CDS             complement(59..1147)
                     /codon_start=1
                     /product="Putative Replication Associated Protein"
                     /translation="MMQESGDEKKVEVTYVDDSKDPLGEGIKLDGDVKKRRQRNWTFT
                     LFYDDAPTADGVVKAWTSAPNFRGLMYEHEVCPKTHKPHLQGFICFDSIKSLAMMKLI
                     HNTAHWEAMGGSIEENNTYCSKEKNGTVIVYGVFPASAKEKGERGKAHGPKGATHGAK
                     APNLWDFLVSDIKGGMSKREVMEKYVRVVWGCEKSLDRYFDEFAPKPTFDIVAAHGSY
                     FPWQQQLLDVLAHKADNRSVVWIYSPEGGVGKSDMFKHLVWNCGFEPLMNAPSRDLAC
                     AWKGGHVVFDLARDLGDAPINYGFMEQVKNGFAFSAKYESRCKIAASSDPRWVICFAN
                     QLPDTSKLSRDRWALFRVVDNMLSWEDA*"
     CDS             complement(1608..3026)
                     /codon_start=1
                     /product="Putative CruV Capsid Protein"
                     /translation="MPRKSSAMVRPARRSVRRIKGKGDYRVGSMVRTARPRRLKGHGD
                     YSYDNPGPFGRAGSWIGDALGRSFAGEGGGKLGAKLGSYLHYIGKIFGSGDYVTAPNQ
                     VKKNVLVSDTQIPQFGSDSNTVHIRHREFLGDVISSGTANTFQTQSFNLNPGLKDTFP
                     WLSQVCGASFQQYRINGMVFEFRTMSADALNSVNTALGSVVMATDYDSADMAFNSKAQ
                     MENTEFGVSCKPSSCMIHAIECAKSQTTATELYVRAGAAQSGTDIRLYDWGKFYIATT
                     GCQGTSVNLGELWVSYDITFFKAIQQPAGWLIPSAHYYLDVSQVGTKPVQPATTAQVA
                     ALVAAGDVVPYDNIGITFDANGVALYLPTNLPLNSEWMITINNGTNAGGAANASQIVP
                     IFSQGLTALPLYQGGNGNAWPNPTPGTAVRSVGFVYYFYVSAPCTIAAPFKIAVGAPT
                     TPLVSYVASDLVINMINPNMAL*"
ORIGIN
        1 tagtattacc cgaccccccc ctagtgatca aggtcgaaga caacgtatca ttatggggtt
       61 atgcgtcctc ccatgatagc atattatcga ctaccctgaa tagcgcccaa cggtcgcgtg
      121 agagcttgct cgtgtcaggg agttgattag caaagcagat aacccaacgg ggatcgctag
      181 aggccgctat tttacatcgc gactcgtact ttgcggagaa cgcgaagccg ttcttgacct
      241 gctccatgaa cccgtagttt atgggcgcat ctcctagatc gcgggctagg tcgaatacta
      301 catgtcctcc cttccatgca cacgccaaat cacgtgaagg cgcgttcatt aagggttcaa
      361 aaccgcagtt ccatacgagg tgtttaaaca tgtcgctttt ccctactcca ccttcaggtg
      421 agtagatcca cacaacagat ctattatctg ctttgtgtgc taacacgtcg agtagttgtt
      481 gttgccatgg gaagtatgaa ccatgggcgg caacgatgtc aaacgtcggt ttgggagcga
      541 attcatcgaa gtagcgatcc agagactttt cacatcccca gactacacgt acgtatttct
      601 ccattacctc gcgcttggac atcccccctt tgatgtctga gacgaggaag tcccacaggt
      661 taggcgcttt agctccatgc gtagctcctt tgggaccgtg tgctttgcca cgctcaccct
      721 tttccttagc gctagcggga aatacaccgt agacgataac ggtaccgttc ttctctttag
      781 agcagtacgt attgttctct tctatggatc cacccatcgc ctcccaatga gccgtattgt
      841 ggatcagctt catcatcgcg agcgatttta tgctgtcgaa gcatatgaag ccctgaagat
      901 gcggcttatg cgtctttgga catacttcgt gctcatacat gagaccacgg aagttaggcg
      961 cagacgtcca tgcctttact acaccatcgg ctgtaggtgc atcgtcgtag aacagtgtaa
     1021 acgtccagtt tcgttgtcgg cgcttcttta cgtcaccgtc tagcttgata ccctccccca
     1081 agggatcttt gctatcgtcg acgtaagtta cctcaacctt cttttcatcg cctgattctt
     1141 gcatcagagg cctatatagg gccgtgcgat aaactgcgcg gatattaatt attatcgtgt
     1201 acgattctca tacttactat atagcgccct accatgtcta agactagtga tcaagtcgtg
     1261 cttgagcgtc tgcgtgacaa gtgccatatg atcatcgaag ccgttgaaga tgcgtgcctc
     1321 gacgaaggcg agtcacgcac aatggttcgt atgtatcaga tcttgaagga ggcctatcgc
     1381 actatctttg gggagcccca attgtgcttg cttctaacct ctatggagct caacgacaat
     1441 gaggaggaga agcaatggcc cccctatatg gatggcgacg cctttgagaa gtaagccaaa
     1501 aaagagatgt cagtggagat caatctccac tcacgaaaga ggtttgaacc tccttcgctc
     1561 gatgtacata cgttaccctg acaactttgt cagggtccat tttttgctta gagcgccata
     1621 ttcgggttga tcatgttgat caccaagtca cttgccacat aagataccaa cggtgtagtc
     1681 ggtgctccta ccgcaatctt aaatggtgca gcaatagtac atggcgcact cacgtaaaag
     1741 tagtaaacaa atccgacgct tcggacagct gtacctggtg ttggattagg ccatgcatta
     1801 ccgttcccac cttggtaaag tggcaacgcg gtcaatccct gcgagaatat cggaactatt
     1861 tgagatgcat ttgctgcgcc tcccgcattt gttccgttgt tgatggtgat catccattca
     1921 ctgtttagcg gtagattagt aggcagatac aatgccacac cattcgcatc gaacgttatg
     1981 ccaatgttat catatggcac tacgtcgcca gctgctacaa gtgcagcaac ctgagctgtc
     2041 gttgcaggtt gtactggttt tgttccgact tgactgacat ccagatagta gtgagccgaa
     2101 gggataagcc aaccggcagg ttgctgaatc gccttgaaga aggtgatatc atagctcacc
     2161 cataactccc ccagattcac acttgtgcct tggcatccag tggtcgcgat gtagaactta
     2221 ccccaatcgt acagacgaat atctgttccg ctctgtgctg ctcctgctct cacgtagagc
     2281 tctgttgctg tggtctgtga tttcgcgcat tcgatagcgt gaatcataca gctagacggc
     2341 ttgcagctta caccaaactc agtgttctcc atctgagctt tcgagttgaa cgccatgtca
     2401 gcactgtcgt agtcagtagc catgaccacc gatccgagtg ccgtatttac actgttcaat
     2461 gcgtcagcac tcatcgttcg aaactcgaac accataccgt tgatcctgta ctgttggaac
     2521 gaggcgccgc acacttgaga caaccaaggg aaggtatctt tcagtccagg gttcaggttg
     2581 aagctctgag tctgaaatgt gttggcagta cccgaggata tcacatcccc gaggaactca
     2641 cggtgacgga tgtgtaccgt gttgctgtcg ctgccaaact gcggaatttg agtatcgctt
     2701 accaacacat tcttcttgac ttgattgggc gcagtgacgt agtcgccgct gccaaagatt
     2761 ttgccgatgt agtggaggta gctgcctagt tttgctccta gttttcctcc accttcacct
     2821 gcgaagcttc gacccaatgc atctccaatc cagcttccag ctctaccgaa tggtccgggg
     2881 ttatcgtatg agtaatcccc gtgtcctttc agtcgtcgag gtctcgcggt tcttaccata
     2941 ctacctacgc ggtagtctcc cttacctttg atccgtctga cggatctcct tgccggtctt
     3001 accatggctg aactctttcg tggcatgtta ggctatagtg tgcctataca taccatcaaa
     3061 ataaatcctc tcgaaaataa ataattaata ttaattattt atctctctca cttattatct
     3121 atcctgaata ctaagcctct atccgcctaa atggctgatc gggccagcgg agcgtagcga
     3181 cgcgtatata gcataaatcc ccttctccaa gttgcgttgg tggccgcagc tggctcgtgg
     3241 gggtgggccc tgggtaacag ggcgcacgtt acgggtaagg cgtagaatcg cgaagcgatt
     3301 tacggccgca tccctaacgt gtcgaccacc cagggacacc caccccccga cgccacaagg
     3361 gaagtccgca tcgcagatgc ggacccacct agcaacgtag cacctccccc cgtgtaggtg
     3421 atcaaaaaag agaggtatcg ttcttcgtgt tcggcgctga tcacgtgggt ataagggggg
     3481 tcggc
//
LOCUS       CruV-274                3485 bp    DNA     circular     24-FEB-2020
DEFINITION  Crucivirus-274 isolate 120_1310_IR2_3297.
ACCESSION   CruV-274
KEYWORDS    .
SOURCE      Crucivirus-274
  ORGANISM  Crucivirus-274
            Unclassified.
REFERENCE   1  (bases 1 to 3485)
  AUTHORS   de la Higuera,I., Kasun,G.W., Torrance,E.L., Pratt,A.A.,
            Maluenda,A., Colombet,J., Bisseux,M., Ravet,V., Dayaram,A.,
            Stainton,D., Kraberger,S., Zawar-Reza,P., Goldstien,S., Briskie,J.,
            White,R., Taylor,H., Gomez,C., Ainley,D.G., Harding,J.S.,
            Fontenele,R.S., Schreck,J., Ribeiro,S.G., Oswald,S.A., Arnold,J.M.,
            Varsani,A., Enault,F. and Stedman,K.M.
  TITLE     Direct Submission
  JOURNAL   Submitted (24-FEB-2020) Biology, Portland State University, 1719 SW
            10th Ave, Portland, Oregon 97201, USA
COMMENT     Bankit Comment: ALT EMAIL:ide@pdx.edu
            Bankit Comment: TOTAL # OF SEQS:451
            Bankit Comment: GAP: est. length - alignment

            ##Assembly-Data-START##
            Assembly Method       :: MetaSPAdes v. 3.12; IDBA-UD v. 1.1.1;
                                     MEGAHIT v. 1.0.3
            Sequencing Technology :: 454; Illumina
            ##Assembly-Data-END##
FEATURES             Location/Qualifiers
     source          1..3485
                     /organism="Crucivirus-274"
                     /mol_type="genomic DNA"
                     /isolate="120_1310_IR2_3297"
                     /isolation_source="Water"
                     /country="Norway"
                     /collection_date=2011
     CDS             1..1062
                     /codon_start=1
                     /product="Putative Replication Associated Protein"
                     /translation="MSTQGPLCKVWDLTGWTSKGWVKDSVEELLKKHGDKWAFQLELA
                     GENEINYDYKYELVKGRYDENSEQTIKDSMEMEKSGGLHFQCRVCLRGNRAHKTAVVR
                     LFEGMHVSPTSTKSTKNFDYVMKEETRIDGPWTSENIEEEEDVFIDEEVKRIANELKP
                     WQTSILKVLEEQSRDKIAVVLDKIGGIGKTTLTDVARYKKIAGIIPSLDTTKLMMECI
                     FGQLTKNRKISKWIVDLPRATNEETIAQTYKAMETLKGGTAYDSRFTFREIITKRPSI
                     LVFTNKIPNLNYISKFRWDFFTVVNNELVSHDITQVEAMIREQNLQAMEIALERDLIK
                     KEDKERLLAKIKAKRLENK*"
     misc_feature    472..499
                     /note="stem-loop"
     misc_feature    486..494
                     /note="nonanucleotide"
     CDS             1813..3438
                     /codon_start=1
                     /product="Putative CruV Capsid Protein"
                     /translation="MPFTARKIRWKPRKYSTYPTKSRYAPSQPKRRYRKRIGNRPSYT
                     GTAAKSAMWELGRLGATALAGPLMSSIGVAAAQNAPSWGTIKQLGQNAYGSIKGYLPS
                     LTGSGDYKINGMGIKSNSFMDEMLSNGQPPMIINGSDRSFVFRHREYLGDVITGPDAN
                     FNLTAYQINPGLASTFPWLSGIASNFEQYRIEGMVFEFKSTSADALNSTNTALGTIIM
                     CTEYNSLNANFTNKQQMENHEFCSSARQSQSILHPVECKPSVTTLTELYVRSGAVPVG
                     QDGRFYDLGNFQIATVGQQGADVNIGELWISYDIVLLKPQLPEEEECPTDEPLSIHWT
                     GVEGINPPTSYFPTSPVTAFGSNLDGVTLSTSTIYMNSDMPNGRYQLTLIYQGAAANL
                     DVGFMVYLPSGGITILELLSDGGAAQVQASGVSGNYFSVVCFEKSDTGTIELTGFQTL
                     PTSLQYMDIFLTQLGPTQELIEVCDSEVDEEELELQFEFNEWKKWQRFEKMSLGAKEN
                     TIKKELEAASEQKQSRTAPINIPKKTSSVKSFK*"
ORIGIN
        1 atgtcaacac aaggtccact atgcaaagtg tgggatttaa caggttggac ttccaaagga
       61 tgggttaaag actcagtaga agagttacta aagaaacacg gagataaatg ggcttttcaa
      121 ctagaattag caggtgaaaa cgaaatcaat tacgattata aatacgaact tgttaaaggt
      181 cgatatgacg aaaactctga acaaaccatt aaagactcaa tggaaatgga aaaatcaggc
      241 ggattacatt tccaatgtag agtttgccta cgtggtaata gagcgcataa aacagcggta
      301 gttagacttt ttgaaggaat gcatgtttct ccgacatcaa caaaatctac taaaaatttt
      361 gattatgtaa tgaaagaaga aacacgaatt gatggaccgt ggacttccga aaacatagaa
      421 gaagaagaag atgttttcat tgatgaagaa gtaaaaagga tagccaatga attaaaacca
      481 tggcaaacta gtattttaaa agttctagaa gaacaatcaa gagataaaat agcagttgtt
      541 ttagacaaga taggaggtat tggcaaaaca acactaactg atgtggcaag atataaaaaa
      601 atagcaggaa taattccgtc attagatacc acaaaattaa tgatggaatg catatttggg
      661 cagttaacta aaaatcgaaa aattagcaaa tggattgttg atttacctag agccacaaac
      721 gaagaaacga ttgcacaaac gtacaaagca atggaaacgc tgaaaggagg aacagcgtat
      781 gatagtagat ttactttcag agaaataatc acaaaacgtc cttcgatatt agtatttaca
      841 aataaaatac ccaacttaaa ttacatatca aaatttaggt gggatttctt tacggtagta
      901 aataatgaac ttgtctctca cgacattact caagtagaag caatgatcag agaacaaaac
      961 cttcaagcaa tggaaatagc attggaacgt gatttgatca aaaaagaaga caaagaaaga
     1021 cttttagcaa aaataaaagc taaaaggctg gagaataagt gatttaatat aaaattttat
     1081 attaactaac aataaatcta aagaattaga ttagattatt ggtttgaaat gtatcaccaa
     1141 tgaatccctt aggcagtccg ctctcactaa gttcggctac cttgtaccag tacgtaaata
     1201 gtaattcaca ttcgttacac tcatgttcat tctatttact taagggattc ggttatattt
     1261 ggttaatttt tatagaactg actaataaat caaccaaatt tgatgaagat atttgatgat
     1321 atctgacgat aatatatcat aaccattata tttaattaaa ttaaaataat tcaaataact
     1381 tcatttttgt ttcggcgcta aacgaatttg ttaaaatgat caccacgcgc ccctccgcta
     1441 tcgcgtcgaa ccgtgctcaa ccgcacgggg cgctgaaatt gtttaaacga atttgtttaa
     1501 atgctcgcta tgctcgcgct gcgcaaatta catgctgcgc agtaggttcg taataattaa
     1561 ttaagtaatt attaagcaaa taaattatac taaattggct cgccaaatgt aaaattttga
     1621 agtgtcaaaa ttaagccatt tctctcgaaa aaaataatta gataaacggt taaaaggccc
     1681 caggatgtgc taaatatata acaggcaatg aataatgaaa tggttaatga aatgtactgt
     1741 taagggtagc aggagcagta atttgacttt taaaaaattt aaaaataaaa tgatgtcgta
     1801 ataaaaaaca acatgccttt caccgcaagg aaaataagat ggaagccacg aaaatactca
     1861 acatacccaa caaaatcaag gtatgcaccg tcacaaccga aacgtcgata tcgtaaacga
     1921 ataggaaacc gaccttcata taccgggaca gcagctaaat cagcaatgtg ggagcttggt
     1981 agattgggag ccactgcttt agcaggacca ttaatgagta gcattggcgt cgcagcagca
     2041 caaaatgcac caagttgggg aacaattaaa caactaggtc aaaacgcata cggaagcatt
     2101 aaaggatacc tgccgtccct tacaggaagt ggagactaca aaataaatgg aatgggcata
     2161 aagtctaata gctttatgga cgaaatgttg tcaaacggac aacctccaat gattattaac
     2221 ggatcagacc gtagctttgt ttttcgtcat agagaatatc taggagacgt aatcactgga
     2281 ccagatgcga actttaatct gacagcatat caaattaatc caggtttggc atcaactttt
     2341 ccatggctgt caggtattgc atcaaacttc gaacagtata ggatcgaagg aatggtattt
     2401 gaatttaaat caacatcagc agatgcatta aatagcacca ataccgcgct aggaacaatt
     2461 attatgtgca cagaatataa cagtttgaac gcgaacttca cgaacaaaca gcaaatggaa
     2521 aatcatgaat tttgctcaag cgcacgtcaa tctcaaagta tattgcatcc tgtagagtgc
     2581 aagccctcag ttactacttt gactgaattg tacgttagaa gcggagcagt tcctgtaggt
     2641 caagatggac gtttctatga cttaggaaac ttccaaattg caacagttgg tcagcaaggt
     2701 gcagatgtaa acatcggtga attgtggatt tcatacgaca ttgtactatt gaaaccgcaa
     2761 ttgcctgaag aagaggaatg cccaacagac gaaccgctta gtatccactg gaccggagtt
     2821 gagggaataa accctccaac ttcatacttt ccgacaagtc cagttaccgc ttttggttca
     2881 aatttagatg gagtaacttt gtcaacaagt accatatata tgaacagtga tatgccaaat
     2941 ggacgatacc aactaacact aatttatcaa ggagcagcag ctaatcttga tgttggtttc
     3001 atggtatatc ttccatcagg aggcataaca attttagaat tactaagtga tggtggagca
     3061 gctcaagtgc aagcaagtgg tgtgtctggt aactattttt cagtcgtatg ctttgagaaa
     3121 tcagacacag gaaccataga acttactggg ttccaaacgc taccaacatc gcttcagtat
     3181 atggatattt ttctcactca actaggaccg acacaagaac ttattgaagt ttgtgactcg
     3241 gaagttgacg aagaagagct tgaacttcaa ttcgaattta acgaatggaa gaaatggcaa
     3301 cgcttcgaaa aaatgagtct aggagcgaaa gaaaacacta ttaaaaaaga acttgaagca
     3361 gcttcagaac aaaaacaaag ccgaacagct ccaattaaca tcccaaaaaa aacgagttct
     3421 gtcaaatctt ttaaataagc aaaataaaat tgatgcttaa aagatttttt ataacactat
     3481 aaaaa
//
LOCUS       CruV-275                3497 bp    DNA     circular     24-FEB-2020
DEFINITION  Crucivirus-275 isolate RP_236.
ACCESSION   CruV-275
KEYWORDS    .
SOURCE      Crucivirus-275
  ORGANISM  Crucivirus-275
            Unclassified.
REFERENCE   1  (bases 1 to 3497)
  AUTHORS   de la Higuera,I., Kasun,G.W., Torrance,E.L., Pratt,A.A.,
            Maluenda,A., Colombet,J., Bisseux,M., Ravet,V., Dayaram,A.,
            Stainton,D., Kraberger,S., Zawar-Reza,P., Goldstien,S., Briskie,J.,
            White,R., Taylor,H., Gomez,C., Ainley,D.G., Harding,J.S.,
            Fontenele,R.S., Schreck,J., Ribeiro,S.G., Oswald,S.A., Arnold,J.M.,
            Varsani,A., Enault,F. and Stedman,K.M.
  TITLE     Direct Submission
  JOURNAL   Submitted (24-FEB-2020) Biology, Portland State University, 1719 SW
            10th Ave, Portland, Oregon 97201, USA
COMMENT     Bankit Comment: ALT EMAIL:ide@pdx.edu
            Bankit Comment: TOTAL # OF SEQS:451
            Bankit Comment: GAP: est. length - alignment

            ##Assembly-Data-START##
            Assembly Method       :: MetaSPAdes v. 3.12; IDBA-UD v. 1.1.1;
                                     MEGAHIT v. 1.0.3
            Sequencing Technology :: 454; Illumina
            ##Assembly-Data-END##
FEATURES             Location/Qualifiers
     source          1..3497
                     /organism="Crucivirus-275"
                     /mol_type="genomic DNA"
                     /isolate="RP_236"
                     /isolation_source="Robin feces"
                     /country="New Zealand"
                     /collection_date=2016
     CDS             complement(1..1476)
                     /codon_start=1
                     /product="Putative CruV Capsid Protein"
                     /translation="MSVGKKYTDAEKAAYYKKLAAASKAAPRTRAAAPAKKRAVTVRA
                     KAPVRRTVSGFGDYSTKKKNQSTGARIGGHVGSKIGTFLGGLASKLMGFGDYHIEQNS
                     LFMGGAMKPPEIVNSVHNGEVIVRHREYLGDVSATVAFTLTPYYLNPGLQASFPWLST
                     MAAAFEEYKWRGMVFEYISTSSDAILSSATSSSLGSVNMATQYNSIEGLFPDKAAMLN
                     HEFGNSTKPSCDLLHPIECLNSRTPVSQLYVRTGPVPAGADERLYDLGVFQIATSDQQ
                     ASTLNGLGQLWCSYEIGLFKPRLPSEDNFPSRVLSDHFQLAAFTNSFPLGTQTTPTST
                     SSLQGTIVASNRYIFPPRIVDGNYLVVINWRGATPVTAGYPTITGVNGSTIPNLWLGN
                     TAASATSPISGTVTITQMMMSFIVNVNGVGGGFNLGVAVLPGTPTPCDMYITQIDSDL
                     INLKVPKNMLKLTADEITTDDCSDEDFVRVSKNDLNKLLNQ*"
     misc_feature    248..274
                     /note="stem-loop"
     misc_feature    256..264
                     /note="nonanucleotide"
     CDS             1588..3045
                     /codon_start=1
                     /product="Putative Replication Associated Protein"
                     /translation="MDYRLLTDPLHPLNMDFSNNDTNHPHTSFEHINKLASTIPNNLA
                     SSRQIAASPKIIMKLDTSQPVVDTYDGLPLSRTNSSPPVTSGKNAVLQESDEPGVPES
                     GRPKYMNNKRILLTYGSRRASVHLHKEDFITAVTDLAVRHKNSISFIRLAHEYGTHDE
                     GEDPYLHTHIAIEFGKNLQSTNSQIFDYMEGYGFRVDKKCHIGFLHPHCRYLISKAHW
                     DNSLKYLAKEDPDNADLLINAKNDTPEFSVAALHAYATPADMLSAMITNKSDVKLATQ
                     YLAIYNCRPNKLMGIQKDPITLTGWQIPFFHEINNTPGKGRQIIWINDSIGGSGKSWL
                     IDHCFATNPSKYYMIGPPQGLPNTMNITMNALKAGWTGDTIFMNLTRSSESYKNTYEC
                     IEALIDGQCYNTKYIGGTYPYKACRVIVMANWMPLVSALTTDRWDIRRLSPISTPFVP
                     GPDTQELLRVSAYDLLHSSRNITDFTALVNNIPPL*"
ORIGIN
        1 ctactgattc aataacttat tcagatcatt cttcgatact cttacaaagt cttcgtctga
       61 acaatcatct gttgttatct catccgcagt taacttcaac atgttctttg gtaccttcaa
      121 attaatcaaa tcactatcaa tctgagtaat atacatatca catggtgtag gtgtgccagg
      181 aagaactgct actcctaaat taaatcctcc tccaactcca ttcacattaa ctataaatga
      241 catcatcatc tgagttattg ttactgtccc agatattggg gaagtagcgc tcgccgcggt
      301 gttccctagc cataaattag gtatcgtcga accgttaact cctgttatcg ttggataacc
      361 cgcagttacc ggcgtggctc ctctccaatt tataactacc aaatagtttc catctacaat
      421 tctcggtggg aatatgtatc tgttcgacgc aactatagtt ccttgtaaac tactcgttga
      481 agttggagtc gtctgtgttc ctagtggaaa cgaattagta aatgctgcga gctgaaaatg
      541 atccgacaat actctgctcg ggaaattatc ctccgatgga aggcggggtt taaaaagccc
      601 aatttcatat gaacaccata actgaccaag tccattcagt gttgaagctt gttgatcaga
      661 ggtcgcaatt tgaaacacac ccaaatcata caatctctca tccgctcctg ctggtactgg
      721 tccagttctc acataaagct gactcactgg cgttctggag ttcaaacact caattggatg
      781 taataaatca cagctcggtt tcgttgaatt accaaactca tgattcaaca tagccgcttt
      841 atctgggaaa agtccttcta tactattata ttgggtcgcc atatttaccg atcccaacga
      901 agaggatgtt gcactcgata atatcgcatc tgacgaagtc gatatatact cgaacaccat
      961 tcctctccat ttgtactcct caaaggctgc tgccattgtc gacaaccatg gaaaagatgc
     1021 ttgtaagccc ggattcaaat aatacggcgt caacgtaaat gctactgtcg cactcacatc
     1081 tcctaaatat tccctgtgtc ttacaatcac ttctccatta tgaacactat tcactatctc
     1141 tgggggcttc atagctcctc ccataaacag tgaattctgt tcaatatggt agtctccaaa
     1201 tcccatcagc tttgatgcca gtcctcctaa aaaagttcct attttcgatc ctacatggcc
     1261 tccaattcgg gctcctgttg attgattctt ctttttcgtc gaataatctc caaatcccga
     1321 cacagtcctt cgtacaggag cttttgcacg tacagtgact gctcttttct tcgctggtgc
     1381 tgctgctctt gttctcggag ctgcctttga tgcagccgcc aacttcttgt agtaagccgc
     1441 cttctctgca tctgtgtact tcttccctac tgacatcttt taaagtacta actaaatctt
     1501 tatacaacat ttatttcctt atacctctta ttataattta tactaggatg tgtctagtat
     1561 aaagatataa tcatccgtat aaaattgatg gattatagat tattaaccga tcctcttcat
     1621 ccattaaaca tggacttcag taataacgat actaatcatc cacatacttc tttcgaacat
     1681 attaacaaac ttgctagtac tattcctaat aacttggcat cgagccgtca gattgcagcc
     1741 tcaccaaaga ttattatgaa attagatact agtcaaccag ttgttgatac gtacgatggt
     1801 cttcctcttt cacgaactaa ctcttcacct cctgttacat ccggtaaaaa cgccgtccta
     1861 caggagagcg acgagcctgg cgttcctgaa agtggtagac caaagtatat gaataataaa
     1921 cgtatccttc ttacttacgg ttcacgtcga gccagtgtac atcttcataa agaagacttt
     1981 atcaccgctg tcactgatct cgccgttaga cacaaaaact ctatctcctt cattcgactc
     2041 gctcacgaat atggaactca tgacgaaggc gaagatcctt atcttcatac ccacatcgca
     2101 atcgagttcg gtaaaaatct tcagtctact aactcccaaa tttttgacta catggaggga
     2161 tatggttttc gtgtcgataa gaaatgccac attgggtttc ttcaccctca ctgtcgatat
     2221 cttatctcta aagctcattg ggacaattct cttaaatatc tagctaaaga agatccagat
     2281 aatgctgatc tactcattaa cgctaaaaat gatactcctg agttctctgt tgctgctctt
     2341 cacgcttacg caactcctgc tgatatgctt agcgctatga tcaccaacaa gtccgacgtt
     2401 aaattagcca ctcagtacct tgctatttat aactgccgtc ctaacaaact catgggtatc
     2461 cagaaagatc ctattaccct aacaggttgg caaattcctt tctttcatga aataaataat
     2521 actcccggta aaggtcgaca aattatttgg attaacgact ccatcggtgg ttccggtaaa
     2581 tcctggttaa ttgaccactg tttcgcaact aacccttcaa agtattacat gatcggtcct
     2641 ccacagggtc tgcctaatac tatgaatatc actatgaacg ccttaaaagc tggttggact
     2701 ggagatacca tctttatgaa cttaactcgt tctagtgaaa gctataaaaa cacatacgag
     2761 tgtattgaag ctcttatcga tggacaatgc tataatacaa aatatatcgg tggtacctat
     2821 ccatataaag catgtcgtgt catcgtcatg gccaattgga tgccacttgt cagtgccctc
     2881 actactgacc gttgggatat tcgtaggctt tctcctatct ctactccctt cgttcctgga
     2941 cctgatactc aggagctcct ccgcgtctcc gcgtatgatc tccttcacag ctcccgtaac
     3001 ataactgatt ttaccgctct ggtaaataat atacctcctc tataaaatgt tcccgggggg
     3061 ctctccgagg gtaggctacc tggtagccat tatagtttta aaaaactctt cacagagttc
     3121 tttaaaaaac ccgtcttgta aacaagccgt gtggggggtt aacattaaga attaaccccc
     3181 cacactattg tttacaacgc ctgcggcgcg ccttgttaat actgttatgt caatgtccac
     3241 aatgaacttc gtaagtggct tcgcccataa cactattaac aacatattaa aaaatttagt
     3301 gttatattta gcgatggatg gatgaccata gtatatatat tacacagggg ggtctaaaga
     3361 tataatatac taaatattac actaaatttt tttaacgatg gatggatgac catagtatat
     3421 atattacaca ggggggtcta aagatataat atactaaatc atcctccatt attttttttt
     3481 aattcttcta agttaac
//
LOCUS       CruV-276                3498 bp    DNA     circular     24-FEB-2020
DEFINITION  Crucivirus-276 isolate H10_24592.
ACCESSION   CruV-276
KEYWORDS    .
SOURCE      Crucivirus-276
  ORGANISM  Crucivirus-276
            Unclassified.
REFERENCE   1  (bases 1 to 3498)
  AUTHORS   de la Higuera,I., Kasun,G.W., Torrance,E.L., Pratt,A.A.,
            Maluenda,A., Colombet,J., Bisseux,M., Ravet,V., Dayaram,A.,
            Stainton,D., Kraberger,S., Zawar-Reza,P., Goldstien,S., Briskie,J.,
            White,R., Taylor,H., Gomez,C., Ainley,D.G., Harding,J.S.,
            Fontenele,R.S., Schreck,J., Ribeiro,S.G., Oswald,S.A., Arnold,J.M.,
            Varsani,A., Enault,F. and Stedman,K.M.
  TITLE     Direct Submission
  JOURNAL   Submitted (24-FEB-2020) Biology, Portland State University, 1719 SW
            10th Ave, Portland, Oregon 97201, USA
COMMENT     Bankit Comment: ALT EMAIL:ide@pdx.edu
            Bankit Comment: TOTAL # OF SEQS:451
            Bankit Comment: GAP: est. length - alignment

            ##Assembly-Data-START##
            Assembly Method       :: MetaSPAdes v. 3.12; IDBA-UD v. 1.1.1;
                                     MEGAHIT v. 1.0.3
            Sequencing Technology :: 454; Illumina
            ##Assembly-Data-END##
FEATURES             Location/Qualifiers
     source          1..3498
                     /organism="Crucivirus-276"
                     /mol_type="genomic DNA"
                     /isolate="H10_24592"
                     /isolation_source="Water"
                     /country="New Zealand"
                     /collection_date=2012
     misc_feature    join(3489..3498,1..17)
                     /note="stem-loop"
     misc_feature    1..9
                     /note="nonanucleotide"
     CDS             239..1756
                     /codon_start=1
                     /product="Putative CruV Capsid Protein"
                     /translation="MKRQREETQLVPYQPVTKRIKMDVQRAPYQRGPGFYRGKPLGPL
                     KAPKYTDRMWRVRRPRRKTPKAMAQYWRKRYRRRVLTGRGDYSYSDSRSTGANLGGWL
                     GSKAGEYLGGMAQTAGMALISGLGDYTVNKNVLLSGNMPSIQNMTSGAGITISFQEYL
                     GDVVTPLQVGNSSPFDVTTYVINASNPRTFPWLSQIAQNFEQYEMEGMLFGFRTTSAD
                     LVTNGTNNLQLGTVIMATQYDVADPNFASKGEMLNYQFSTSVKPSQPAIHMIECDPRQ
                     TSVNLLYTGTAAAGTDPRLYNLGRFSIATQGFQGSGIVIGELHVTYQVRLLKPKLWTT
                     LGENIPWSSGIYTMSSGSTKSWLGVSGTIATQTINNSNQTVSIDNNLDITQVQSKVIF
                     QRQRFPQRIYILIAWSAQSGATGGFFNTSDGNPVFSAGGGATLISQQIYPLSATNDSI
                     TGSFMGIVDIAPDQSAPYFLIKSFTSTFQPDYPNGQIIARLTLAPTDPTGNGNLN*"
     CDS             complement(1872..2882)
                     /codon_start=1
                     /product="Putative Replication Associated Protein"
                     /translation="MKSSSSSSSSSEQVEDPIEHETELVISPYAGKPPARGERGNAQV
                     CWLYTWFNYDPEKLPVVPDTECKYRIEGKELTPTKKTPHVQGFVIFKTKQRWSTVTTN
                     YPGINWLPMCKKATPWNNFEYCSKDGDFTEFGERPKQPKASGRGNDQQAFAEAFAADS
                     VGAGIQIIKEKRPRDLALHGEAIERNLKKAKTEQWSHKYRENSFLVPLQDTGSNLLVH
                     GPSNTGKTHYAAAHFENPLVCSHVDDLKRLGPDNDGIIFDDMSFKHWPVEAIIHLLDR
                     EFGRSINVRYGTVQIRAGIPKIFTHNTRNPFYEEATIEEEQKIAIERRFRRVNVLNKL
                     YA*"
ORIGIN
        1 tatagtaacc ccagggctcc ccggagccgg agccaccacg tgcttggctg gtggtggctg
       61 gctcctcttc tggctggctg gtggtggttg gctccaccgg ccggcctctt ctggtggtgg
      121 ctgctggccg gctcccatcc ctggctggcc ccgcgccccc tggggggggg cgcggccccg
      181 gccaggccag gctgtgaggc caaggcacgt gtttttccaa gaacgcacgt gttgtcatat
      241 gaagcggcaa cgtgaagaga cccaactcgt cccttatcaa cctgttacca aacgaatcaa
      301 aatggacgta caacgagcac cctatcaacg aggacctgga ttttaccgtg gaaaacctct
      361 tggcccgctt aaagcaccta aatacacaga tcgaatgtgg cgagttcgcc gaccaaggcg
      421 aaaaactcct aaagcaatgg ctcaatactg gcgaaagcga taccgaagac gagtcctcac
      481 tggacgagga gattatagtt attcagatag tcgatctact ggtgcgaatc ttggaggatg
      541 gcttggaagc aaagcgggtg aatacctcgg aggtatggcc caaaccgcag gaatggcctt
      601 aatatccggt ttgggtgatt atactgttaa caaaaacgtg ctactatctg gaaacatgcc
      661 ttcaattcaa aatatgacct ctggtgctgg aatcactatt tcctttcaag aatatcttgg
      721 tgacgttgtc actccactac aagtgggaaa ttcttcacct ttcgatgtta caacatatgt
      781 tattaatgct tcaaatcctc gaacattccc ttggttatca caaattgcac aaaattttga
      841 acaatatgaa atggaaggaa tgctctttgg ttttaggaca acttcagctg acttagttac
      901 taatggcaca aataacttac aacttggaac tgttattatg gctactcaat atgatgtagc
      961 tgatccaaac tttgcatcta aaggtgaaat gctcaattac caattttcaa catctgttaa
     1021 accctcacaa cctgcaatac acatgataga atgtgaccct agacaaacct cagtcaatct
     1081 cctttatact ggtaccgcgg ctgctggtac cgatcctcga ctttacaatc ttgggagatt
     1141 ttcaatcgct actcaaggtt ttcaaggttc tggtattgta attggtgaac tacacgttac
     1201 ctatcaagtc agacttctta aaccaaaatt atggactaca cttggagaaa atatcccttg
     1261 gagttcaggc atttatacta tgagttcagg atcaactaaa agttggcttg gtgtttctgg
     1321 tactattgct acacagacta ttaataattc taatcaaacg gtatcgatag ataacaatct
     1381 tgatattacc caggtacaat caaaagttat attccaacgt caacgattcc ctcaacgtat
     1441 atacatactt atagcatggt cagctcaatc tggagcaaca ggaggatttt ttaatacctc
     1501 tgatggtaat cccgtttttt ctgctggtgg cggtgcaaca ctcatatcac aacaaatcta
     1561 ccccctctct gctactaatg actctattac tggatctttt atgggaattg tagatattgc
     1621 ccctgatcag tcagcacctt actttttgat taaatcattc acatctacat tccaacccga
     1681 ttacccaaat ggtcaaatta ttgcgagatt aactcttgca cctacagacc caactggtaa
     1741 tggcaatctt aattgatcca gcaaccacat taccgcggct gcggacttat atattttttg
     1801 ggttaaaatg tcaaacaaag taataaacaa cagtttagtt caaatataat ttattaaaat
     1861 caacaatata cttaagcgta taatttattt aagacattta ctcgtcgaaa tcgacgctca
     1921 atagctatct tttgctcctc ttcaatagtg gcttcttcat agaacgggtt tcttgtattg
     1981 tgggtaaaga tctttgggat tccagctctg atttggactg ttccatatct gacattgatt
     2041 gatcttccga actccctatc tagcaaatga ataatggctt caactggcca gtgtttgaat
     2101 gacatatcat cgaagattat tccatcatta tcaggcccca acctctttaa atcgtcaaca
     2161 tgtgaacata ctaagggatt ttcgaaatgg gctgcagcat aatgcgtctt tcctgtattt
     2221 gagggtccat gtaccaatag atttgatccc gtgtcttgta gtggtacaag gaagctattc
     2281 tctctgtatt tgtgagacca ctgttcggtc ttggcttttt taaggtttct ttcaatggct
     2341 tccccgtgta gagccaagtc acgtggtctt ttctccttga tgatttgtat tccggctcca
     2401 acactatcag cggcaaaggc ttcagcgaag gcttgttgat cgttaccgcg gccgctggcc
     2461 ttaggttgtt tgggtctttc tccaaactca gtgaaatctc cgtccttact acagtactcg
     2521 aagttgttcc aaggtgtggc ttttttgcac atgggcaacc agttgattcc tggataattg
     2581 gttgtaactg ttgaccatct ctgcttggtc ttaaaaatga caaatccttg cacgtgtgga
     2641 gtcttttttg ttggtgtaag ttcctttcct tcaattctgt acttgcattc cgtgtcgggc
     2701 acgactggta acttttcggg atcataattg aaccacgtgt aaagccaaca gacttgtgcg
     2761 tttcctctct ccccacgtgc aggtggcttt cctgcgtatg gactaatgac cagttccgtt
     2821 tcatgttcaa taggatcctc aacctgttca gatgatgatg atgatgatga tgatgatttc
     2881 atatatgatt accgaaagag tagacgctga tgggtataat ccttatcggt accttacagt
     2941 cgagaactca atgggtatga tttataaccc cccctatata agtatagaat tcgactcgaa
     3001 ctcttttcag atttgtactt atactatatg agggctcccc atttgggaaa gttttcaaat
     3061 atttttttta gaaattttgg ctatttgttt ttttatactg taaaaacatt agaaaccgtt
     3121 ttttggctat ttgttttttt atactgtaaa aacattagaa accgtttttt ggctatttgt
     3181 ttttttatac tgtaaaaaca ttagaaaccg ttttttggct atttgttttt ttatactgta
     3241 aaaacattag aaaccgtttt ttggctattt gtttttttta acccaaatct tagccagagc
     3301 gccgagaagg tcggtgctag cacggtcgtg ggggctgggg cccccgaccc agtgcgcgcc
     3361 tatgcccaca ccctacaaca ccacacaagc gaatttaagc aaaattcccc actctacatt
     3421 aagcaggctg gtggtggctg ctggccggcc tcctggctgg ctcttcttgg ctggctggtg
     3481 ccggctctgc cctgggta
//
LOCUS       CruV-277                3499 bp    DNA     circular     24-FEB-2020
DEFINITION  Crucivirus-277 isolate SR5_34193.
ACCESSION   CruV-277
KEYWORDS    .
SOURCE      Crucivirus-277
  ORGANISM  Crucivirus-277
            Unclassified.
REFERENCE   1  (bases 1 to 3499)
  AUTHORS   de la Higuera,I., Kasun,G.W., Torrance,E.L., Pratt,A.A.,
            Maluenda,A., Colombet,J., Bisseux,M., Ravet,V., Dayaram,A.,
            Stainton,D., Kraberger,S., Zawar-Reza,P., Goldstien,S., Briskie,J.,
            White,R., Taylor,H., Gomez,C., Ainley,D.G., Harding,J.S.,
            Fontenele,R.S., Schreck,J., Ribeiro,S.G., Oswald,S.A., Arnold,J.M.,
            Varsani,A., Enault,F. and Stedman,K.M.
  TITLE     Direct Submission
  JOURNAL   Submitted (24-FEB-2020) Biology, Portland State University, 1719 SW
            10th Ave, Portland, Oregon 97201, USA
COMMENT     Bankit Comment: ALT EMAIL:ide@pdx.edu
            Bankit Comment: TOTAL # OF SEQS:451
            Bankit Comment: GAP: est. length - alignment

            ##Assembly-Data-START##
            Assembly Method       :: MetaSPAdes v. 3.12; IDBA-UD v. 1.1.1;
                                     MEGAHIT v. 1.0.3
            Sequencing Technology :: 454; Illumina
            ##Assembly-Data-END##
FEATURES             Location/Qualifiers
     source          1..3499
                     /organism="Crucivirus-277"
                     /mol_type="genomic DNA"
                     /isolate="SR5_34193"
                     /isolation_source="Water"
                     /country="New Zealand"
                     /collection_date=2012
     CDS             1..873
                     /codon_start=1
                     /product="Putative Replication Associated Protein"
                     /translation="MPYRTKSGIKARYKYACFTDFVQLNHENYFTNKMDNVSYGVAGY
                     EICPDTKKPHIQGYVEFIKPINRDQIQTILGNKKMHVTERWATAQQAADYCKEDGTFT
                     EVGTISKSEQGKRNDLIDIRDKIINGISDKDLVMEHTEDYARHYKCFDRWRYMFQPHR
                     TWKTEVHILWGEPGCGKSHRAVLEEGGELISFENKFVLGYKNQEVVVLDDFNPGMIPL
                     EFMLRLTDKYHMVVNVKGGEMTWNPKKIYITTNRNPEAWYGKAGQWLRRVTSVTKLGD
                     DAYTGNNVDNIAGI*"
     misc_feature    32..50
                     /note="stem-loop"
     misc_feature    37..45
                     /note="nonanucleotide"
     CDS             complement(1936..3396)
                     /codon_start=1
                     /product="Putative CruV Capsid Protein"
                     /translation="MSFSDILSFSKFSLLCIFTFSFLLCNFESLRRLFFLNFFQKFSP
                     LFLFLFKGTISTIRIINYSTMPFVPRKKIIKRKRPTTRYMRPKAVKRVRKARAVPYSK
                     AISPASRPSIGSQIGGYLGNAAQNMFSKFLGFGAYDLKQNNMVQTLTPSNQVPFMKSS
                     STSTRIRHREFIQDFVGSSTPFAATYYPINIGMAQTFPWLSSIARNFSSYRFHGLIFE
                     FKSLSGDDQNKQSQGYVAGAVDYNSDSPFWVTKSQIENAMFSASSKPSTDIIFPVECD
                     PKHNQVSEFYVRSSALASTSSELLYDIGTFTIARGSNLNSTDTIGELWVSYDVELISP
                     ILGAGQLALPSAHYTSTNYTNLLPLGSTRTTVFDNMGLTISSTTITFPLGTLGKFALY
                     VQQTGANTGNVVPGSVSFNAGATALISLQNGSASGISAPLYTGAISGPNTSVYTTFTI
                     PSATVQAIITIGTAGTLPTSPSSCDIFVMPLNATLT*"
ORIGIN
        1 atgccttatc gaacgaagtc aggaatcaaa gcacgctata aatacgcgtg tttcacggat
       61 ttcgtgcaac tcaatcatga aaattatttc actaacaaaa tggataacgt gtcctatgga
      121 gtggcgggat acgagatctg cccggacact aagaaacctc atatccaggg atatgttgaa
      181 tttataaaac ccattaacag ggaccaaatt caaacgatct tgggaaacaa aaagatgcat
      241 gtgaccgaaa ggtgggcaac cgcgcagcaa gctgctgatt attgcaaaga agatgggact
      301 ttcacagaag ttgggaccat aagtaagtca gaacaaggga agaggaatga tctcatagat
      361 attcgtgaca aaattatcaa tggcatctca gataaagact tggtgatgga acacaccgaa
      421 gactacgcgc ggcactataa gtgttttgat cgttggcgct atatgtttca accgcacaga
      481 acctggaaaa cagaagtaca tattttatgg ggagaaccag gatgcgggaa gtcgcacaga
      541 gccgtgctcg aagaaggggg agaattgatt tcattcgaaa ataaattcgt actaggttac
      601 aagaaccaag aagtggtggt gttggacgat ttcaatccag gaatgattcc gttggaattt
      661 atgttgcgat taaccgacaa gtatcacatg gttgtgaatg tgaaaggagg agagatgacc
      721 tggaacccga agaagatcta tataacaaca aatcgaaacc cggaggcgtg gtacgggaag
      781 gccgggcagt ggctccggcg cgtgacttcc gtcactaaat tgggggatga cgcgtacaca
      841 ggaaataacg tcgataatat cgcagggata taaaagtttt aacagttcaa tatattttag
      901 tttatcaaaa aacgtattca tttcattcat gttagtcgcg cttcgcgctc tcgcactacg
      961 tgctcagtcc tggcgcttcg cgctgcggac ctcgtttcac tcgggtcgcc ttcggctctc
     1021 acgtcgcgct tcgcgctcct tctcgcttcg ctcgagttcg gcttcgcctc acgggctcgc
     1081 tgcgctcgcc gcaatcgctt cgctctcgct gatcgttggg atcgttgggg tagtggggtg
     1141 tttcctcggc ttggggcctc gtcaaggtgt ttcgcctgaa cggctcaagt cgcgctgcgc
     1201 gctccaagtg agcgctcgta ttaactcgct tgacttcgtc gccctttggc tcctcgtgat
     1261 cgttgggatc gtaggggtgt gagcgctcgt attaactcgc ttgacttcgt cgccctttgg
     1321 ctcctcgtga tcgttggaac tgtagttatt gttataatcg ttgaagatcg taggcgctcg
     1381 cattggctcg cttaattcgt cgcctttttg ggctcctcat acaccgcagg ggtgataacg
     1441 tgcacgtgca tgtgcactga agtggctggt aatactaggg ctggctcgtt gcactcgctg
     1501 cgcccttcag ccacttccta gagggatcgt aggatttcta gggatcgaag gatcgaagag
     1561 atgatgacta cgttcggggc tgcgcgccct cactgcgcgc tgcgcttgcc tacgttacgg
     1621 cgctgcgcgg cctccactgc gcggtcgtac ctcccttgcc tacgcgcgct cgtacctcgc
     1681 acgctgcgcc ggcgctatag tcgctgcgct ccttcgctct gttgcggggg attagatagg
     1741 ttggctggtt tggtggtgag gtgctagatg tgtgcttttg ggctttttgc tcaccgcaaa
     1801 aaactgcacc gcgctgcgcg cccgcccatg aaggtgccga aaatttctcg tagggttcgt
     1861 aagaaattta attcacaaag tgaatcgaat atgaaaaaaa accatttatt ggaaaaaatt
     1921 aagtatagaa agaatttaag ttaacgttgc gttcaacggc atcacgaaga tatcgcaaga
     1981 cgacggagat gttggaaggg tgccagctgt accgatggta atgatcgctt gtacggtcgc
     2041 actcgggata gtaaacgtag tgtacaccga ggtgttagga cccgaaatag caccggtgta
     2101 gagaggtgct gagatgccgg acgcgctccc gttttggaga gaaataagag ccgtagcacc
     2161 agcgttaaaa gacactgatc cagggaccac gttgcctgta tttgctcctg tttgctgaac
     2221 gtataacgca aacttaccaa gagtaccaag ggggaacgta atcgtggtgc tcgagatcgt
     2281 caggcccata ttgtcgaaga ccgtagtgcg tgtagatccg agagggagaa ggttggtata
     2341 attcgtagaa gtataatgcg cggatggaag cgcaagttgg cccgcgccaa gaatagggga
     2401 aatgagctca acgtcatatg aaacccaaag ctctccgatc gtatcagtcg agttcaagtt
     2461 gctgccgcgc gcaatcgtga aggtaccaat gtcgtaaaga agctcggaag aagtagaggc
     2521 aagagcgctc gaacgaacgt aaaattcgga aacctgattg tgcttagggt cgcactccac
     2581 agggaaaata atatcagtcg atggtttgct ggacgccgaa aacatcgcgt tttcgatctg
     2641 tgacttagtt acccagaaag gactatcgga gttgtagtcc accgcaccgg ctacatagcc
     2701 ttgagactgc ttgttctgat cgtcaccaga aagagatttg aactcaaata tcaaaccatg
     2761 gaaccggtat gaagagaagt ttcgcgcaat actgctcaac caagggaatg tttgggccat
     2821 accgatatta attggataat atgtcgcagc aaatggagta gaagaaccga cgaagtcttg
     2881 gatgaactca cgatggcgaa tcctcgtcga cgtactcgat gatttcatga acggcacttg
     2941 attcgaagga gtaagggtct gaaccatgtt gttttgtttg aggtcgtacg ctccaaaacc
     3001 gagaaacttg ctgaacatgt tttgtgcagc attacccaag tagccgccaa tttgactgcc
     3061 aatagaaggg cgcgatgcag gtgagatcgc tttggagtag gggacagcgc gcgccttacg
     3121 aacacgttta acagctttgg gccgcatgta acgagtagta ggacgcttac gcttaataat
     3181 ttttttccga ggaacgaaag gcattgttga ataatttatg attctaatag tagaaatcgt
     3241 tcctttaaat agaaataaaa ataggggtga aaatttttga aaaaaattta gaaaaaatag
     3301 tcgtcttaaa gactcgaaat tacatagtag aaaagaaaaa gtaaaaatgc aaagcaatga
     3361 aaatttcgaa aacgacagga tgtcagaaaa cgacatcaat tacgcgctgc tcacgccgcg
     3421 cccagcgcct gacggcgtgg atgaagaagt tgttgttatt gaaaatccta agaaacgtcg
     3481 aatctcagat acggaagaa
//
LOCUS       CruV-278                3500 bp    DNA     circular     24-FEB-2020
DEFINITION  Crucivirus-278 isolate H2_34249.
ACCESSION   CruV-278
KEYWORDS    .
SOURCE      Crucivirus-278
  ORGANISM  Crucivirus-278
            Unclassified.
REFERENCE   1  (bases 1 to 3500)
  AUTHORS   de la Higuera,I., Kasun,G.W., Torrance,E.L., Pratt,A.A.,
            Maluenda,A., Colombet,J., Bisseux,M., Ravet,V., Dayaram,A.,
            Stainton,D., Kraberger,S., Zawar-Reza,P., Goldstien,S., Briskie,J.,
            White,R., Taylor,H., Gomez,C., Ainley,D.G., Harding,J.S.,
            Fontenele,R.S., Schreck,J., Ribeiro,S.G., Oswald,S.A., Arnold,J.M.,
            Varsani,A., Enault,F. and Stedman,K.M.
  TITLE     Direct Submission
  JOURNAL   Submitted (24-FEB-2020) Biology, Portland State University, 1719 SW
            10th Ave, Portland, Oregon 97201, USA
COMMENT     Bankit Comment: ALT EMAIL:ide@pdx.edu
            Bankit Comment: TOTAL # OF SEQS:451
            Bankit Comment: GAP: est. length - alignment

            ##Assembly-Data-START##
            Assembly Method       :: MetaSPAdes v. 3.12; IDBA-UD v. 1.1.1;
                                     MEGAHIT v. 1.0.3
            Sequencing Technology :: 454; Illumina
            ##Assembly-Data-END##
FEATURES             Location/Qualifiers
     source          1..3500
                     /organism="Crucivirus-278"
                     /mol_type="genomic DNA"
                     /isolate="H2_34249"
                     /isolation_source="Water"
                     /country="New Zealand"
                     /collection_date=2012
     CDS             join(3271..3500,1..196)
                     /codon_start=1
                     /product="putative ORF"
                     /translation="MFFLTQILARAPRRSVLARSWGLGPPTQCAPMPTPYNTTQANLS
                     KIPHSTLSRLVVAAGRPPGWLFLAGWCRLCPGYIVTPGLPGAGATTCLAGGGWLLFWL
                     AGGGWLHRPASSGGGCWPAPIPGWPRAPWGGRGPGQARL*"
     CDS             join(complement(1..237),complement(3375..3500))
                     /codon_start=1
                     /product="putative ORF"
                     /translation="MTTRAFLEKHVPWPHSLAWPGPRPPQGARGQPGMGAGQQPPPEE
                     AGRWSQPPPASQKRSQPPPAKHVVAPAPGSPGVTIYPGQSRHQPAKKSQPGGRPAATT
                     SLLNVEWGILLKFACVVL*"
     misc_feature    join(3491..3500,1..17)
                     /note="stem-loop"
     misc_feature    1..9
                     /note="nonanucleotide"
     CDS             238..1755
                     /codon_start=1
                     /product="Putative CruV Capsid Protein"
                     /translation="MKRQREETQLVPYQPVTKRIKMDVQRAPYQRGPGFYRGKPLGPL
                     KAPKYTDRMWRVRRPRRKTPKAMAQYWRKRYRRRVLTGRGDYSYSDSRSTGANLGGWL
                     GSKAGEYLGGMAQTAGMALISGLGDYTVNKNVLLSGNMPSIQNMTSGAGITISFQEYL
                     GDVVTPLQVGNSSPFDVTTYVINASNPRTFPWLSQIAQNFEQYEMEGMLFGFRTTSAD
                     LVTNGTNNLQLGTVIMATQYDVADPNFASKGEMLNYQFSTSVKPSQPAIHMIECDPRQ
                     TSVNLLYTGTAAAGTDPRLYNLGRFSIATQGFQGSGIVIGELHVTYQVRLLKPKLWTT
                     LGENIPWSSGIYTMSSGSTKSWLGVSGTIATQTINNSNQTVSIDNNLDITQVQSKVIF
                     QRQRFPQRIYILIAWSAQSGATGGFFNTSDGNPVFSAGGGATLISQQIYPLSATNDSI
                     TGSFMGIVDIAPDQSAPYFLIKSFTSTFQPDYPNGQIIARLTLAPTDPTGNGNLN*"
     CDS             complement(1871..2884)
                     /codon_start=1
                     /product="Putative Replication Associated Protein"
                     /translation="MKSSSSSSSSSSEQVEDPIEHETELVISPYAGKPPARGERGNAQ
                     VCWLYTWFNYDPEKLPVVPDTECKYRIEGKELTPTKKTPHVQGFVIFKTKQRWSTVTT
                     NYPGINWLPMCKKATPWNNFEYCSKDGDFTEFGERPKQPKASGRGNDQQAFAEAFAAD
                     SVGAGIQIIKEKRPRDLALHGEAIERNLKKAKTEQWSHKYRENSFLVPLQDTGSNLLV
                     HGPSNTGKTHYAAAHFENPLVCSHVDDLKRLGPDNDGIIFDDMSFKHWPVEAIIHLLD
                     REFGRSINVRYGTVQIRAGIPKIFTHNTRNPFYEEATIEEEQKIAIERRFRRVNVLNK
                     LYA*"
ORIGIN
        1 tatagtaacc ccagggctcc ccggagccgg agccaccacg tgcttggctg gtggtggctg
       61 gctcctcttc tggctggctg gtggtggttg gctccaccgg ccggcctctt ctggtggtgg
      121 ctgctggccg gctcccatcc ctggctggcc ccgcgccccc tggggggggc gcggccccgg
      181 ccaggccagg ctgtgaggcc aaggcacgtg tttttccaag aacgcacgtg ttgtcatatg
      241 aagcgtcaac gtgaagagac ccaactcgtc ccttatcaac ctgttaccaa acgaatcaaa
      301 atggacgtac aacgagcacc ctatcaacga ggacctggat tttaccgtgg aaaacctctt
      361 ggcccgctta aagcacctaa atacacagat cgaatgtggc gagttcgccg accaaggcga
      421 aaaactccta aagcaatggc tcaatactgg cgaaagcgat accgaagacg agtcctcact
      481 ggacgaggag attatagtta ttcagatagt cgatctactg gtgcgaatct tggaggatgg
      541 cttggaagca aagcgggtga atacctcgga ggtatggccc aaaccgcagg aatggcctta
      601 atatccggtt tgggtgatta tactgttaac aaaaacgtgc tactatctgg aaatatgcct
      661 tcaattcaaa atatgacctc tggtgctgga atcactattt cctttcaaga atatcttggt
      721 gacgttgtca ctccactaca agtgggaaat tcttcacctt tcgatgttac aacatatgtt
      781 attaatgctt caaatcctcg aacattccct tggttatcac aaattgcaca aaattttgaa
      841 caatatgaaa tggaaggaat gctctttggt tttaggacaa cttcagctga cttagttact
      901 aatggcacaa ataacttaca acttggaact gttattatgg ctactcaata tgatgtagct
      961 gatccaaact ttgcatctaa aggtgaaatg ctcaattacc aattttcaac atctgttaaa
     1021 ccctcacaac ctgcaataca catgatagaa tgtgacccta gacaaacctc agtcaatctc
     1081 ctttatactg gtaccgcggc tgctggtacc gatcctcgac tttacaatct tgggagattt
     1141 tcaatcgcta ctcaaggatt tcaaggttct ggtattgtaa ttggtgaact acacgttacc
     1201 tatcaagtca gacttcttaa accaaaatta tggactacac ttggagaaaa tatcccttgg
     1261 agttcaggca tttatactat gagttcagga tcaactaaaa gttggcttgg tgtttctggt
     1321 actattgcta cacagactat taataattct aatcaaacgg tatcgataga taacaatctt
     1381 gatattaccc aggtacaatc aaaagttata ttccaacgtc aacgattccc tcaacgtata
     1441 tacatactta tagcatggtc agctcaatct ggagcaacag gaggattttt taatacctct
     1501 gatggtaatc ccgttttttc tgctggtggt ggtgcaacac tcatatcaca acaaatctac
     1561 cccctctctg ctactaatga ctctattact ggatctttta tgggaattgt agatattgcc
     1621 cctgatcagt cagcacctta ctttttgatt aaatcattca catctacatt ccaacccgat
     1681 tacccaaatg gtcaaattat tgcgagatta actcttgcac ctacagaccc aactggtaat
     1741 ggcaatctta attgatccag caaccacatt accgcggctg cggacttata tattttttgg
     1801 gttaaaatgt caaacaaagt aataaacaac agtttagttc aaatataatt tattaaaatc
     1861 aacaatatac ttaagcgtat aatttattta agacatttac tcgtcgaaat cgacgctcaa
     1921 tagctatctt ttgctcctct tcaatagtgg cttcttcata gaacgggttt cttgtattgt
     1981 gggtaaagat ctttgggatt ccagctctga tttggactgt tccatatctg acattgattg
     2041 atcttccgaa ctccctatct agcaaatgaa taatggcttc aactggccag tgtttgaatg
     2101 acatatcatc gaagattatt ccatcattat caggccccaa cctctttaaa tcgtcaacat
     2161 gtgaacatac taagggattt tcgaaatggg ctgcagcata atgcgtcttt cctgtatttg
     2221 agggtccatg taccaataga tttgatcccg tgtcttgtag tggtacaagg aagctattct
     2281 ctctgtattt gtgagaccac tgttcggtct tggctttttt aaggtttctt tcaatggctt
     2341 ccccgtgtag agccaagtca cgtggtcttt tctccttgat gatttgtatt ccggctccaa
     2401 cactatcagc ggcaaaggct tcagcgaagg cttgttgatc gttaccgcgg ccgctggcct
     2461 taggttgttt gggtctttct ccaaactcag tgaaatctcc gtccttacta cagtactcga
     2521 agttgttcca aggtgtggct tttttgcaca tgggcaacca gttgattcct ggataattgg
     2581 ttgtaactgt tgaccatctc tgcttggtct taaaaatgac aaatccttgc acgtgtggag
     2641 tcttttttgt tggtgtaagt tcctttcctt caattctgta cttgcattcc gtgtcgggca
     2701 cgactggtaa cttttcggga tcataattga accacgtgta aagccaacag acttgtgcgt
     2761 ttcctctctc cccacgtgca ggtggctttc ctgcgtatgg actaatgacc agttccgttt
     2821 catgttcaat aggatcctca acctgttcag atgatgatga tgatgatgat gatgatgatt
     2881 tcatatatga ttaccgaaag agtagacgct gatgggtata atccttatcg gtaccttaca
     2941 gtcgagaact caatgggtat gatttataac cccccctata taagtataga attcgactcg
     3001 aactcttttc agatttgtac ttatactata tgagggctcc ccatttggga aagttttcaa
     3061 atattttttt tagaaatttt ggctatttgt ttttttatac tgtaaaaaca ttagaaaccg
     3121 ttttttggct atttgttttt ttatactgta aaaacattag aaaccgtttt ttggctattt
     3181 gtttttttat actgtaaaaa cattagaaac cgttttttgg ctatttgttt ttttatactg
     3241 taaaaacatt agaaaccgtt ttttggctat ttgttttttt taacccaaat cttagccaga
     3301 gcgccgagaa ggtcggtgct agcacggtcg tgggggctgg ggcccccgac ccagtgcgcg
     3361 cctatgccca caccctacaa caccacacaa gcgaatttaa gcaaaattcc ccactctaca
     3421 ttaagcaggc tggtggtggc tgctggccgg cctcctggct ggctcttctt ggctggctgg
     3481 tgccggctct gccctgggta
//
LOCUS       CruV-279                3501 bp    DNA     circular     24-FEB-2020
DEFINITION  Crucivirus-279 isolate 120_1295_IR2_3297.
ACCESSION   CruV-279
KEYWORDS    .
SOURCE      Crucivirus-279
  ORGANISM  Crucivirus-279
            Unclassified.
REFERENCE   1  (bases 1 to 3501)
  AUTHORS   de la Higuera,I., Kasun,G.W., Torrance,E.L., Pratt,A.A.,
            Maluenda,A., Colombet,J., Bisseux,M., Ravet,V., Dayaram,A.,
            Stainton,D., Kraberger,S., Zawar-Reza,P., Goldstien,S., Briskie,J.,
            White,R., Taylor,H., Gomez,C., Ainley,D.G., Harding,J.S.,
            Fontenele,R.S., Schreck,J., Ribeiro,S.G., Oswald,S.A., Arnold,J.M.,
            Varsani,A., Enault,F. and Stedman,K.M.
  TITLE     Direct Submission
  JOURNAL   Submitted (24-FEB-2020) Biology, Portland State University, 1719 SW
            10th Ave, Portland, Oregon 97201, USA
COMMENT     Bankit Comment: ALT EMAIL:ide@pdx.edu
            Bankit Comment: TOTAL # OF SEQS:451
            Bankit Comment: GAP: est. length - alignment

            ##Assembly-Data-START##
            Assembly Method       :: MetaSPAdes v. 3.12; IDBA-UD v. 1.1.1;
                                     MEGAHIT v. 1.0.3
            Sequencing Technology :: 454; Illumina
            ##Assembly-Data-END##
FEATURES             Location/Qualifiers
     source          1..3501
                     /organism="Crucivirus-279"
                     /mol_type="genomic DNA"
                     /isolate="120_1295_IR2_3297"
                     /isolation_source="Water"
                     /country="Norway"
                     /collection_date=2011
     CDS             complement(346..1731)
                     /codon_start=1
                     /product="Putative CruV Capsid Protein"
                     /translation="MSKRQRAPKKAPYVKKEYANPGIPYAVSQPNKFWRIKKDLPPAD
                     YWKKRYWRRRITGRGDYVMDASRSVGHQWGGYAGAKVGEFVGSTAQTLLGSLLTGMGD
                     YTVKKNVFMKGRLPQVVNQPTGGGTIIRFQEYLTDVITAPVAGDFKIQSFLINAANPV
                     TFPFLSQIAANYEQYEVEGMIFEFRSTSADSLNSTNTALGTVMQATQYDVGDDVFEAK
                     LDMLNYEYSTSCRPSVNCAHMIECAPSQTTVSKLYTLYNQGVPDNADPRLYHLGRYSI
                     ATTGFQGTSVNIGELHVTYQVRLLKPKLFVSLGETANFLEQILGDGTTTTPYSDPAPL
                     PGPATVGTNFFVNESNNSTFFIDTNLIRLPPTAAVLYYRIEILWTGTAPAGYSPPTLI
                     YLNCEQTDIDVAPDVGTIVAGKVALMITVKTKPTSIAPTITLANDGVFPSGNKQVEVR
                     IMQITTEFNGV*"
     CDS             join(complement(2754..3228),complement(2189..2712))
                     /codon_start=1
                     /product="Putative Replication Associated Protein"
                     /translation="MQCFVSFKKKKYFTAVKKLFPRAHIELKSQQSTMKQASDYCKKD
                     GNFHEKGTLPLDGNVAGGKGTKRKWDEIKSFATSGQLDQIDAEIFVLHYRNLKQISYD
                     YGNDRFVNLPDVCGEWIYGPPGVGKSHIARAENPDLYVKMINKWWDNYDGQKTVLLDD
                     FGHEHFMLGHHLKIWADKYPFRGEIKCHSRMFRPEKIVVTSNCHPAEIWDSEDDMCKA
                     IVRRFKIRRIDVLKKNDDTEPKLRKTANTKINLRREDAQHNLLHLSSINIPQIIDLTK
                     EEEAHTNKTLEMLQQGKMHSISLTPTQRKSSQSEEEMYDSDDDVIYSDSESMSESM*"
ORIGIN
        1 agatttcggg tataagacac ggtacctaat ttccttatat agttttctta ttttggaaca
       61 cgtgcgtgaa actgcctcat taggacggcc acacgttttg gtagttaaaa acgtgtgaac
      121 gtgcccttgc ggtaagtgtc attaccgaaa ataggagttt gacatgtgac acttttgaca
      181 tgtttgcaca attcacaccg cgtgaccttc ctaattccga aataggtaag tgtcaaaaat
      241 ttttgacagg tgtcaatcgg gatttgacac ttaccgaaac acacttgtga aaatgtgaaa
      301 attttaccgg cggacctacg cgtcaaaaac taaaaaattt tttatttaaa ctccattaaa
      361 ttccgtagtt atttgcataa tacgaacttc aacttgtttg ttgcctgaag ggaatactcc
      421 gtcgttcgcc agagtaattg ttggagcaat tgaagttggt ttggtcttga ctgtaatcat
      481 aagggccacc tttcctgcga caatagttcc tacgtcgggt gcgacgtcaa tgtcagtttg
      541 ttcgcagtta agatatatca aagttggagg ggaataaccg gctggagcag taccggtcca
      601 gaggatttca attctataat ataaaactgc cgccgttggt ggcagtctaa tgagattggt
      661 gtcgatgaaa aaggtgctgt tgttgctttc attaacaaag aaatttgttc cgaccgtagc
      721 tggaccgggt agtggtgctg gatctgaata aggggtagtt gttgttccat caccaagaat
      781 ttgttcaaga aaattagctg tctctccaag ggacacgaat agttttggtt ttaataatct
      841 tacttggtaa gtcacatgaa gttcgccgat gttcacgctt gtcccttgaa aacctgtggt
      901 cgcaattgaa taccggccga ggtgataaag acgaggatct gcgttgtcag gcactccttg
      961 gttgtagagt gtgtaaagct tgctgactgt tgtttggctg ggggcacatt ctatcatatg
     1021 tgcacagttg actgaagggc ggcaggaggt gctgtactca taatttaaca tatcaagttt
     1081 tgcttcgaaa acgtcgtctc cgacatcgta ttgagttgct tgcataactg ttcctagtgc
     1141 ggtgtttgtt gagtttaatg agtcagcact tgttgaacga aattcaaaaa tcatgccttc
     1201 gacttcatac tgttcgtagt ttgcagcgat ttgcgacagg aatggaaagg tgactggatt
     1261 tgccgcatta atgagaaagc tttgaatttt gaagtctccg gcaactggtg cagtgattac
     1321 atccgtaaga tactcctgga agcgaatgat agtccctcca cctgtcggtt gattgactac
     1381 ttgaggtaat ctgcccttca taaatacgtt ctttttaaca gtgtagtccc ccatcccagt
     1441 taataaactt ccgagaagag tttgcgccgt gctaccgacg aactctccaa ccttcgcgcc
     1501 agcgtatccc ccccactggt gtcccaccga gcgggaggca tccatgacat agtcccctcg
     1561 acctgtaatc ctccgtctcc aatatcgttt cttccaataa tcagccggag gtagatcttt
     1621 tttgatgcgc cagaatttgt tcggttgaga gacagcataa ggaattccag ggttagcata
     1681 ttcctttttg acataggggg ccttcttagg tgctctttgt cgtttagaca tttgtgaaat
     1741 aagttcgggg aaaagtgtgt ttgtttatat agtaccgtgt cacgtgcaca cgtggggaca
     1801 ttcaacgtga ctaaatcatg ccacgtgcgc acgtggtaag acggggcttc gataagacga
     1861 cgccctctag ggtaatacta ggccttgggg ccgacctaga gggcgccgga acgatcgagg
     1921 gggagttgga cgaatccgcg cctccgccgg cgctgttatg aattgtaact tcttactttc
     1981 cagggtcaaa catagactga acgggccttg gggccctgaa gatttttttt cccctggcca
     2041 ctgtattttg ccgtgatccc cctaccaccg tattgtggtg ttagcctgcc ggcggcgtgt
     2101 gagcctacgg cgtgggccgc gactgctgaa tgctgaatac tgaattctaa attatgaagg
     2161 gattcaatgc ccagttaagt ttatttaatc acatactttc agacatactc tcagagtcag
     2221 aataaataac atcgtcatcg ctgtcataca tctcttcctc agactgtgaa ctcttcctct
     2281 gtgtaggtgt taaagatata ctatgcatct tgccttgttg taacatttct agtgttttgt
     2341 ttgtatgtgc ttcttcttcc ttcgtcagat caattatttg tggtatatta atagatgata
     2401 gatgaagtag attatgctgt gcatcctctc ttcttaagtt tatcttcgtg ttagcagttt
     2461 ttcttaactt gggttcggta tcatcattct tcttcaatac atcaatcctt ctaatcttga
     2521 atcgtctgac tatagctttg cacatatcat cctcagaatc ccatatttca gctgggtggc
     2581 agttgcttgt gacaacaatt ttttctggtc tgaacattct tgaatgacac ttaatctctc
     2641 ctctgaaagg atacttgtca gcccagattt ttaagtggtg gcccaacata aagtgttcat
     2701 gtccaaagtc atctaaaagg taagtacatt acaacaagtt gttaatggtt tacctaataa
     2761 cacagttttt tgtccatcgt aattgtccca ccatttgttt atcatcttca catataaatc
     2821 ggggttctcg gctcgagcaa tatgagactt tccaactcct gggggtccat aaatccattc
     2881 tccgcaaacg tccggtaaat taacgaagcg gtcattacca taatcataag atatctgctt
     2941 caaattacga tagtgtaaca caaagatttc agcatctatc tgatctaact gtccactagt
     3001 agcgaaagat ttgatttcat cccacttgcg tttggtgcct tttcctccag caacattacc
     3061 atctaatgga agggttccct tctcatgaaa attaccatcc tttttacaat aatcagaggc
     3121 ttgcttcata gtagattgtt gtgacttcag ctctatatga gctcttggga agagcttctt
     3181 aacagcagta aaatacttct tctttttgaa agaaacaaaa cattgcatgt gttcagtgcc
     3241 actctcgcct ttctcgaggc cacagatagt ataatcgcat tcgtcagcaa cagcatcaaa
     3301 ggcagcgcca tcagtttcgt taggatggtt aataacaatg atccagtgtt tacctagtta
     3361 aaattatcaa taggattgag gatggattga ggatggattg aggttggatt cttacgcttt
     3421 tttcttacgc ttttttcttg cgcttttacc ttgcgattta ttatttgtgc ttgaggaggc
     3481 gttgctacta gtgtctgaca t
//
LOCUS       CruV-280                3507 bp    DNA     circular     24-FEB-2020
DEFINITION  Crucivirus-280 isolate 001460_virusbaton.
ACCESSION   CruV-280
KEYWORDS    .
SOURCE      Crucivirus-280
  ORGANISM  Crucivirus-280
            Unclassified.
REFERENCE   1  (bases 1 to 3507)
  AUTHORS   de la Higuera,I., Kasun,G.W., Torrance,E.L., Pratt,A.A.,
            Maluenda,A., Colombet,J., Bisseux,M., Ravet,V., Dayaram,A.,
            Stainton,D., Kraberger,S., Zawar-Reza,P., Goldstien,S., Briskie,J.,
            White,R., Taylor,H., Gomez,C., Ainley,D.G., Harding,J.S.,
            Fontenele,R.S., Schreck,J., Ribeiro,S.G., Oswald,S.A., Arnold,J.M.,
            Varsani,A., Enault,F. and Stedman,K.M.
  TITLE     Direct Submission
  JOURNAL   Submitted (24-FEB-2020) Biology, Portland State University, 1719 SW
            10th Ave, Portland, Oregon 97201, USA
COMMENT     Bankit Comment: ALT EMAIL:ide@pdx.edu
            Bankit Comment: TOTAL # OF SEQS:451
            Bankit Comment: GAP: est. length - alignment

            ##Assembly-Data-START##
            Assembly Method       :: MetaSPAdes v. 3.12; IDBA-UD v. 1.1.1;
                                     MEGAHIT v. 1.0.3
            Sequencing Technology :: 454; Illumina
            ##Assembly-Data-END##
FEATURES             Location/Qualifiers
     source          1..3507
                     /organism="Crucivirus-280"
                     /mol_type="genomic DNA"
                     /isolate="001460_virusbaton"
                     /isolation_source="Water"
                     /country="France"
                     /collection_date=2013
     misc_feature    join(3495..3507,1..18)
                     /note="stem-loop"
     misc_feature    1..9
                     /note="nonanucleotide"
     CDS             complement(108..1202)
                     /codon_start=1
                     /product="Putative Replication Associated Protein"
                     /translation="MTTLETKVPASPLGMEEKKEIIDPESDSVDSGKRTMNWAFTWNN
                     YTEEDVKTVNEDWLKLPDMRCVVYEKEVGKKGTPHLQGFFVFKKIKSFAQVKAMISPK
                     VRFSQMYKCLEANLKYCSKDKGAVFLGDVPMSKEQKGTHGYKGKESGKDAPDVWACFI
                     ADIKKGMSRKELMETYAEIGCRFSAGFDRYYDMFRPQVPFDIEKTHESFYPWQRQLLD
                     IIDLKADARSVIWVYSKRGNTGKTAMFKHLICMDETFEPFMNSTSRDLSCAWKGGSAI
                     FDLARDDGGVPMNYAFMEMIKNGYAFSAKYESKCKKATNMMGQRFVICFSNARPDVSK
                     LSIDRWAIFRIKEDLSLHLEDPYCFDENDN*"
     CDS             1365..2750
                     /codon_start=1
                     /product="Putative CruV Capsid Protein"
                     /translation="MPAPARKTYAAKPKKKYAKQQKVRSTYLKRDNRAPSEVALNASR
                     RAGTSDMGSMLGGLAGMALGGPTGAMIGNAVGGIGGNIISRLFGSGDYKTNAEKLTKN
                     NLVLSNAAQAPQFGSGRVMCKFRHREYLGDVFSSETANTFKIDSYAINPGVASTFPWL
                     SGVVGAKFQQYRINGCVFEFRSMSSDALNSTNTALGSVIMSTDYDSADNVFTSKQEME
                     NTEYGVSCKPSINMIHGIECKRSQTPVSELYIRAFDVPAGKDIRLYDLGRFSIATTGC
                     QGTNVNLGELWVSYDIDAFKAIEQVPQYLTPFAQYTLNAVDATHALGTSQTVIADEIG
                     ITFEYVAGVASVILPHSMPAGTIFSIFIGVLGSSTALVPTMDIPVISPSLDALGTGNG
                     WQGYAACPYPGPATTDFKALYTTYKYNGLGTPANPPKLSVAGGVVPTAITLANLTISF
                     MSGALPSDGQP*"
ORIGIN
        1 tagtattaca gaccccccct agttatcaac taggtactaa aactgggtaa cctataagct
       61 ctaaattgaa aaataagctt aaatcccagt tctaaaaatt agaaccccta gttatcattt
      121 tcatcaaagc aatagggatc ctctaaatgc aatgaaaggt cttctttgat tctaaaaatg
      181 gcccaacgat caatcgagag tttagacaca tcagggcgtg catttgagaa gcaaatgaca
      241 aatctttgtc ccatcatgtt cgtagccttt ttacatttag attcgtattt cgcactaaat
      301 gcataaccat tcttaatcat ttccatgaag gcgtaattca tgggaactcc tccatcatcg
      361 cgagcaagat cgaagatcgc gctaccgcct ttccatgcac atgaaaggtc acgagatgta
      421 gagttcataa atggttcaaa tgtttcatcc atacatatca aatgcttgaa catagcagtt
      481 ttgcctgtgt ttccacgctt tgaatatacc caaataacag atcttgcatc tgccttaaga
      541 tcgataatat ccagcagttg acgctgccac ggatagaaag actcatgagt cttttcgata
      601 tcaaaaggga cctgtggtcg aaacatgtca taatatctgt cgaatcccgc agagaatcta
      661 cagcctattt ctgcatatgt ttccattagc tccttgcggc tcatgccttt cttaatgtca
      721 gcaatgaagc aagcccacac atcgggcgca tctttgccag attccttacc cttgtaacca
      781 tgtgttcctt tctgttcttt agacatagga acatcaccaa ggaatacagc tcctttatcc
      841 ttgctacaat atttcaagtt cgcctcgaga cacttgtaca tttgtgagaa gcgaacctta
      901 ggtgagatca tagctttcac ttgtgcgaag cttttgatct tcttgaacac aaagaagcct
      961 tgaaggtgag gagttccctt tttgccgact tctttttcgt atacaacaca tcgcatgtcg
     1021 ggcagcttga gccaatcctc attgactgtc ttgacatctt cttcagtgta gttgttccac
     1081 gtgaacgccc agttcatcgt ccgtttacca ctgtcaaccg agtcactttc gggatcaata
     1141 atttccttct tctcttccat ccctaaagga gaagcaggaa cttttgtctc gagagtagtc
     1201 atcggtcaca agtatatcta acctagataa ctttcataaa acattttcaa ataaatgttg
     1261 ctccctcaat cgagggacca acccttctat tttagaaggg ataaatatat aaatgttata
     1321 aacatttttg gttacatgag tatacctgga ctcttgtcaa caacatgcca gcccctgctc
     1381 gtaaaactta tgctgcaaag cctaagaaaa agtatgcaaa gcaacaaaaa gtacgcagta
     1441 cttatcttaa gcgagacaat cgcgcacctt ctgaggtcgc attgaatgca tcgcgtcgtg
     1501 caggtacctc ggacatggga tccatgcttg gaggactcgc aggtatggca ctcggaggac
     1561 cgaccggtgc tatgatcgga aacgcagttg gaggtatcgg cggtaatatc atttcgcgcc
     1621 tgtttggtag cggcgactac aaaactaatg ctgaaaagct aacaaaaaac aaccttgtgt
     1681 tgtcaaacgc tgctcaagca ccacagttcg gaagcggacg tgttatgtgc aagtttaggc
     1741 atcgagagta tctcggtgac gtgttcagtt cggaaactgc caacacattt aagatcgaca
     1801 gttatgcgat caatccgggt gtggcttcca cattcccttg gctgtctggt gttgtcggag
     1861 caaagttcca acaatatcga atcaatggat gcgtcttcga gtttcgaagt atgtcatctg
     1921 atgcattgaa ttcaaccaat acagctctcg gatcagtcat catgtctact gattatgatt
     1981 cggcggacaa tgttttcaca tcgaaacaag agatggagaa cacagagtac ggggtgtctt
     2041 gcaagccttc gatcaacatg attcatggta tcgagtgcaa gcgctcacaa actccagtat
     2101 cggagttgta tatccgtgca tttgatgttc cggcaggaaa ggatattcgt ctgtatgatc
     2161 tgggacgatt ttcaatcgct acgactggat gtcaaggaac caacgtgaac cttggtgagc
     2221 tttgggtatc ctatgatatt gatgccttta aggctatcga acaggtccct cagtatctta
     2281 caccatttgc ccaatacact ctcaacgctg ttgatgcaac gcatgcgctc ggtacttcac
     2341 agaccgtgat cgcagatgaa attggcatta catttgagta tgtagccggt gtggcgagtg
     2401 tgattcttcc tcactcaatg ccagcaggga ctatcttctc aattttcatt ggggttttgg
     2461 gttcgagcac tgctcttgtt ccaacaatgg atattcctgt aatctcgcct agcttggatg
     2521 cactgggaac cggaaacggt tggcaaggct atgccgcatg tccttatcca ggcccagcta
     2581 cgacagattt taaggccttg tatacaacat acaagtataa cggattgggc acgcctgcaa
     2641 acccacctaa gctatcagta gctggtggag ttgttccaac cgcaattact ttagcgaatt
     2701 taactatcag tttcatgagt ggtgcgctgc catccgatgg acaaccgtaa ctagggattg
     2761 gccccactga acctgagttt ctcatggtct cagaggacaa aaccatcgag acagacgccc
     2821 taaaccaggg tatctgtcca gtacaataaa ggagaaaaat ctccgaatca aaagcccgaa
     2881 agggtacatt ttttgccgat caaatctgac cagcaaaccc cctagaatag agggtttcaa
     2941 aaaaagaaca aacgcggcat atccaaattt atgtatattc cgagggacgc ccggtggacg
     3001 tcgaggggtg ggggagccga ggcaccccac acttgtttgg gtgcctcgtc ttccccaccc
     3061 attgtcgtcc acggggcgtg agggtattac atgttaaatg taatcacgaa caatcattaa
     3121 aactaatgaa tcaatccaat gagtagtcgt agtcatatat ctcggcgaag tcctttgcaa
     3181 gaggatgaag tatccaccaa tgtggaaaag accagatatc tccccaacga ctattataat
     3241 aaccacgaaa gagacactcg agctcttgaa gtactaacat gttctcccac ggaggatata
     3301 cgtagtctat aacaatagac ataagattcg tatcgtagat cgaagccata acgaaacgtt
     3361 atacctatat ggagaaaaat ctccttatag aaataaacaa ctcaagggta ggcgaaacgg
     3421 ataccctgaa aaaacagctc agaatgagca aaaaataacg taggggagag atctgcataa
     3481 acatgcatga tatcgggggg tcgacta
//
LOCUS       CruV-281                3508 bp    DNA     circular     24-FEB-2020
DEFINITION  Crucivirus-281 isolate WNZ_406.
ACCESSION   CruV-281
KEYWORDS    .
SOURCE      Crucivirus-281
  ORGANISM  Crucivirus-281
            Unclassified.
REFERENCE   1  (bases 1 to 3508)
  AUTHORS   de la Higuera,I., Kasun,G.W., Torrance,E.L., Pratt,A.A.,
            Maluenda,A., Colombet,J., Bisseux,M., Ravet,V., Dayaram,A.,
            Stainton,D., Kraberger,S., Zawar-Reza,P., Goldstien,S., Briskie,J.,
            White,R., Taylor,H., Gomez,C., Ainley,D.G., Harding,J.S.,
            Fontenele,R.S., Schreck,J., Ribeiro,S.G., Oswald,S.A., Arnold,J.M.,
            Varsani,A., Enault,F. and Stedman,K.M.
  TITLE     Direct Submission
  JOURNAL   Submitted (24-FEB-2020) Biology, Portland State University, 1719 SW
            10th Ave, Portland, Oregon 97201, USA
COMMENT     Bankit Comment: ALT EMAIL:ide@pdx.edu
            Bankit Comment: TOTAL # OF SEQS:451
            Bankit Comment: GAP: est. length - alignment

            ##Assembly-Data-START##
            Assembly Method       :: MetaSPAdes v. 3.12; IDBA-UD v. 1.1.1;
                                     MEGAHIT v. 1.0.3
            Sequencing Technology :: 454; Illumina
            ##Assembly-Data-END##
FEATURES             Location/Qualifiers
     source          1..3508
                     /organism="Crucivirus-281"
                     /mol_type="genomic DNA"
                     /isolate="WNZ_406"
                     /isolation_source="Blood worms"
                     /country="New Zealand"
                     /collection_date=2012
     CDS             complement(1072..2532)
                     /codon_start=1
                     /product="Putative CruV Capsid Protein"
                     /translation="MSFSDILSFSKFSLLCIFTFSFLLCNFESLRRLFFLNFFQKFSP
                     LFLFLFKGTISTIRIINYSTMPFVPRKKIIKRKRPTTRYMRPKAVKRVRKARAVPYSK
                     AISPASRPSIGSQIGGYLGNAAQNMFSKFLGFGAYDLKQNNMVQTLTPSNQVPFMKSS
                     STSTRIRHREFIQDFVGSSTPFAATYYPINIGMAQTFPWLSSIARNFSSYRFHGLIFE
                     FKSLSGDDQNKQSQGYVAGAVDYNSDSPFWVTKSQIENAMFSASSKPSTDIIFPVECD
                     PKHNQVSEFYVRSSALASTSSELLYDIGTFTIARGSNLNSTDTIGELWVSYDVELISP
                     ILGAGQLALPSAHYTSTNYTNLLPLGSTRTTVFDNMGLTISSTTITFPLGTLGKFALY
                     VQQTGANTGNVVPGSVSFNAGATALISLQNGSASGISAPLYTGAISGPNTSVYTTFTI
                     PSATVQAIITIGTAGTLPTSPSSCDIFVMPLNATLT*"
     CDS             2483..3508
                     /codon_start=1
                     /product="Putative Replication Associated Protein"
                     /translation="MQSNENFENDRMSENDINYALLTPRPAPDGVDEEVVVIENPKKR
                     RISDTEEMPYRTKSGIKARYKYACFTDFVQLNHENYFTNKMDNVSYGVAGYEICPDTK
                     KPHIQGYVEFIKPINRDQIQTILGNKKMHVTERWATAQQAADYCKEDGTFTEVGTISK
                     SEQGKRSDLIDIRDKIINGISDKDLVMEHTEDYARHYKCFDRWRYMFQPHRTWKTEVH
                     ILWGEPGCGKSHRAVLEEGGELISFENKFVLGYKNQEVVVLDDFNPGMIPLEFMLRLT
                     DKYHMVVNVKGGEMTWNPKKIYITTNRNPEVWYGKAEQWLRRVTSVTKLGDDAYTGNN
                     VDNIAGI*"
     misc_feature    2667..2685
                     /note="stem-loop"
     misc_feature    2672..2680
                     /note="nonanucleotide"
ORIGIN
        1 aagttttaac agttcaatat attttagttt atcaaaaaac gtattcattt cattcatgtt
       61 agtcgcgctt cgcgctctcg cactacgtgc tcagtcctgg cgcttcgcgc tgcggacctc
      121 gtttcactcg ggtcgccttc ggctctcacg tcgcgcttcg cgctccttct cgcttcgctc
      181 gagttcggct tcgcctcacg ggctcgctgc gctcgccgca atcgcttcgc tctcgctgat
      241 cgttgggatc attggggtag tggggtgttt cctcggcttg gggcctcgtc aaggtgtttc
      301 gcctgaacgg ctcaagtcgc gctgcgcgct ccaagtgagc gctcgtatta actcgcttga
      361 cttcgtcgcc ctttggctcc tcgtgatcgt tgggatcgta ggggtgtgag cgctcgtatt
      421 aactcgcttg acttcgtcgc cctttggctc ctcgtgatcg ttgggatcgt tggaactgta
      481 gttattgtta taatcgttga agatcgtagg cgctcgcatt ggctcgctta attcgtcgcc
      541 tttttgggct cctcatacac cgcaggggtg ataacgtgca cgtgcacgtg cactgaagtg
      601 gctggtaata ctagggctgg ctcgttgcac tcgctgcgcc cttcagccac ttcctagagg
      661 gatcgtagga tttctaggga tcgaaggatc gaagagatga tgactacgtt cggggctgcg
      721 cgccctcact gcgcgctgcg cttgcctacg ttacggcgct gcgcggcctc cactgcgcgg
      781 tcgtacctcc cttgcctgcg cgcgctcgta cctcgcacgc tgcgccgccg ctatagtcgc
      841 tgcgctcctt cgctctgttg cggggggtta gataggttgg ctggtttggt ggtgaggtgc
      901 tagatgtgtg cttttgggct ttttgctcac cgcaaaaaac tgcaccgcgc tgcgcgcccg
      961 cccatgaagg tgccgaaaat ttctcgtagg gttcgtaaga aatttaattc acaaagtgaa
     1021 tcgaatatga aaaaaaacca tttattggaa aaaattaagt atagaaagaa tttaagttaa
     1081 cgttgcgttc aacggcatca cgaagatatc gcaagacgac ggagatgttg gaagggtgcc
     1141 agctgtaccg atggtaatga tcgcttgtac ggtcgcactc gggatagtaa acgtagtgta
     1201 caccgaggtg ttaggacccg aaatagcacc ggtgtagaga ggtgctgaga tgccggacgc
     1261 gctcccgttt tggagagaaa taagagccgt agcaccagcg ttaaaagaca ctgatccagg
     1321 gaccacgttg cctgtatttg ctcctgtttg ctgaacgtat aacgcaaact taccaagagt
     1381 accaaggggg aacgtaatcg tggtgctcga gatcgtcagg cccatattgt cgaagaccgt
     1441 agtgcgtgta gatccgagag ggagaaggtt ggtataattc gtagaagtat aatgcgcgga
     1501 tggaagcgca agttggcccg cgccaagaat aggggaaatg agctcaacgt catatgaaac
     1561 ccaaagctct ccgatcgtat cagtcgagtt caagttgctg ccgcgcgcaa tcgtgaaggt
     1621 accaatgtcg taaagaagct cggaagaagt agaggcaaga gcgctcgaac gaacgtaaaa
     1681 ttcggaaacc tgattgtgct tagggtcgca ctccacaggg aaaataatat cagtcgatgg
     1741 tttgctggac gccgaaaaca tcgcgttttc gatctgtgac ttagttaccc agaaaggact
     1801 atcggagttg tagtccaccg caccggctac atagccttga gactgcttgt tctgatcgtc
     1861 accagaaaga gatttgaact caaatatcaa accatggaac cggtatgaag agaagtttcg
     1921 cgcaatactg ctcaaccaag ggaatgtttg ggccataccg atattaattg gataatatgt
     1981 cgcagcaaat ggagtagaag aaccgacgaa gtcttggatg aactcacgat ggcgaatcct
     2041 cgtcgacgta cttgatgatt tcatgaacgg cacttgattc gaaggagtaa gggtctgaac
     2101 catgttgttt tgtttgaggt cgtacgctcc aaaaccgaga aacttgctga acatgttttg
     2161 tgcagcatta cccaagtagc cgccaatttg actgccaata gaagggcgcg atgcaggtga
     2221 gatcgctttg gagtagggga cagcgcgcgc cttacgaaca cgtttaacag ctttgggccg
     2281 catgtaacga gtagtaggac gcttacgctt aataattttt ttccgaggaa cgaaaggcat
     2341 tgttgaataa tttatgattc taatagtaga aatcgttcct ttaaatagaa ataaaaatag
     2401 gggtgaaaat ttttgaaaaa aatttagaaa aaatagtcgt cttaaagact cgaaattaca
     2461 tagtagaaaa gaaaaagtaa aaatgcaaag caatgaaaat ttcgaaaacg acaggatgtc
     2521 agaaaacgac atcaattacg cgctgctcac gccgcgccca gcgcctgacg gcgtggatga
     2581 agaagttgtt gttattgaaa atcctaagaa acgtcgaatc tcagatacgg aagaaatgcc
     2641 ttatcgaacg aagtcaggaa tcaaagcacg ctataaatac gcgtgtttca cggatttcgt
     2701 gcaactcaat catgaaaatt atttcactaa caaaatggat aacgtgtcct atggagtggc
     2761 gggatacgag atctgcccgg acactaagaa acctcatatc cagggatatg ttgaatttat
     2821 aaaacccatt aacagggacc aaattcaaac gatcttggga aacaaaaaga tgcatgtgac
     2881 cgaaaggtgg gcaaccgcgc agcaagctgc tgattattgc aaagaagatg ggactttcac
     2941 agaagttggg accataagta agtcagaaca agggaagagg agtgatctca tagatattcg
     3001 tgacaaaatt atcaatggca tctcagataa agacttggtg atggaacaca ccgaagacta
     3061 cgcgcggcac tataagtgtt ttgatcgttg gcgctatatg tttcaaccgc acagaacctg
     3121 gaaaacagaa gtacatattt tatggggaga accaggatgc gggaagtcgc acagagccgt
     3181 gctcgaagaa gggggagaat tgatttcatt cgaaaataaa ttcgtactag gttacaagaa
     3241 ccaagaagtg gtggtgttgg acgatttcaa tccaggaatg attccgttgg aatttatgtt
     3301 gcgattaacc gacaagtatc acatggttgt gaatgtgaaa ggaggagaga tgacctggaa
     3361 cccgaagaag atctatataa caacaaatcg aaacccggag gtgtggtacg ggaaggcaga
     3421 gcagtggctc cggcgcgtga cttccgtcac taaattgggg gatgacgcgt acacaggaaa
     3481 taacgtcgat aatatcgcag ggatataa
//
LOCUS       CruV-282                3509 bp    DNA     circular     24-FEB-2020
DEFINITION  Crucivirus-282 isolate H1_84525.
ACCESSION   CruV-282
KEYWORDS    .
SOURCE      Crucivirus-282
  ORGANISM  Crucivirus-282
            Unclassified.
REFERENCE   1  (bases 1 to 3509)
  AUTHORS   de la Higuera,I., Kasun,G.W., Torrance,E.L., Pratt,A.A.,
            Maluenda,A., Colombet,J., Bisseux,M., Ravet,V., Dayaram,A.,
            Stainton,D., Kraberger,S., Zawar-Reza,P., Goldstien,S., Briskie,J.,
            White,R., Taylor,H., Gomez,C., Ainley,D.G., Harding,J.S.,
            Fontenele,R.S., Schreck,J., Ribeiro,S.G., Oswald,S.A., Arnold,J.M.,
            Varsani,A., Enault,F. and Stedman,K.M.
  TITLE     Direct Submission
  JOURNAL   Submitted (24-FEB-2020) Biology, Portland State University, 1719 SW
            10th Ave, Portland, Oregon 97201, USA
COMMENT     Bankit Comment: ALT EMAIL:ide@pdx.edu
            Bankit Comment: TOTAL # OF SEQS:451
            Bankit Comment: GAP: est. length - alignment

            ##Assembly-Data-START##
            Assembly Method       :: MetaSPAdes v. 3.12; IDBA-UD v. 1.1.1;
                                     MEGAHIT v. 1.0.3
            Sequencing Technology :: 454; Illumina
            ##Assembly-Data-END##
FEATURES             Location/Qualifiers
     source          1..3509
                     /organism="Crucivirus-282"
                     /mol_type="genomic DNA"
                     /isolate="H1_84525"
                     /isolation_source="Water"
                     /country="New Zealand"
                     /collection_date=2012
     CDS             1..873
                     /codon_start=1
                     /product="Putative Replication Associated Protein"
                     /translation="MPYRTKSGIKARYKYACFTDFVQLNHENYFTNKMDNVSYGVAGY
                     EICPDTKKPHIQGYVEFIKPINRDQIQTILGNKKMHVTERWATAQQAADYCKEDGTFT
                     EVGTISKSEQGKRSDLIDIRDKIINGISDKDLVMEHTEDYARHYKCFDRWRYMFQPHR
                     SWKTEVHILWGEPGCGKSHRAVLEEGGELISFENKFVLGYKNQEVVVLDDFNPGMIPL
                     EFMLRLTDKYHMVVNVKGGEMTWNPKKIYITTNRNPETWYGKAEQWLRRVTSVTKLGD
                     DAYTGNNVDNIAGI*"
     misc_feature    32..50
                     /note="stem-loop"
     misc_feature    37..45
                     /note="nonanucleotide"
     CDS             complement(1946..3406)
                     /codon_start=1
                     /product="Putative CruV Capsid Protein"
                     /translation="MSFSDILSFSKFSLLCIFTFSFLLCNFESLRRLFFLNFFQKFSP
                     LFLFLFKGTISTIRIINYSTMPFVPRKKIIKRKRPTTRYMRPKAVKRVRKARAVPYSK
                     AISPASRPSIGSQIGGYLGNAAQNMFSKFLGFGAYDLKQNNMVQTLTPSNQVPFMKLS
                     STSTRIRHREFIQDFVGSSTPFAATYYPINIGMAQTFPWLSSIARNFSSYRFHGLIFE
                     FKSLSGDDQNKQSQGYVAGAVDYNSDSPFWVTKSQIENAMFSASSKPSTDIIFPVECD
                     PKHNQVSEFYVRSSALASTSSELLYDIGTFTIARGSNLNSTDTIGELWVSYDVELISP
                     ILGAGQLALPSAHYTSTNYTNLLPLGSTRTTVFDNMGLTISSTTITFPLGTLGKFALY
                     VQQTGANTGNVVPGSVSFNAGATALISLQNGSASGISAPLYTGAISGPNTSVYTTFTI
                     PSATVQAIITIGTAGTLPTSPSSCDIFVMPLNATLT*"
ORIGIN
        1 atgccttatc gaacgaagtc aggaatcaaa gcacgctata aatacgcgtg tttcacggat
       61 ttcgtgcaac tcaatcatga aaattatttc actaacaaaa tggataacgt gtcctatgga
      121 gtggcgggat acgagatctg cccggacact aagaaacctc atatccaggg atatgttgaa
      181 tttataaaac ccattaacag ggaccaaatt caaacgatct tgggaaacaa aaagatgcat
      241 gtgaccgaaa ggtgggcaac cgcgcagcaa gctgctgatt attgcaaaga agatgggact
      301 ttcacagaag ttgggaccat aagtaagtca gaacaaggga agaggagtga tctcatagat
      361 attcgtgaca aaattatcaa tggcatctca gataaagact tggtgatgga acacaccgaa
      421 gactacgcgc ggcactataa gtgttttgat cgttggcgct atatgtttca accgcacaga
      481 agctggaaaa cagaagtaca tattttatgg ggagaaccag gatgcgggaa gtcgcacaga
      541 gccgtgctcg aagaaggggg agaattgatt tcattcgaaa ataaattcgt actaggttac
      601 aagaaccaag aagtggtggt gttggacgat ttcaatccag gaatgattcc gttggaattt
      661 atgttgcgat taaccgacaa gtatcacatg gttgtgaatg tgaaaggagg agagatgacc
      721 tggaacccga agaagatcta tataacaaca aatcgaaacc cggagacgtg gtacgggaag
      781 gccgagcagt ggctccggcg cgtgacttcc gtcactaaat tgggggatga cgcgtacaca
      841 ggaaataacg tcgataatat cgcagggata taaaagtttt aacagttcaa tatattttag
      901 tttatcaaaa aacgtattca tttcattcat gttagtcgcg cttggcgctc tcgcactacg
      961 tgctcagtcc tggcgcttcg cgctgcggac ctcgtttcac tcgggtcgcc ttcggctctc
     1021 acgtcgcgct tcgcgctcct tctcgcttcg ctcgagttcg gcttcgcctc acgggctcgc
     1081 tgcgctcgcc gcaatcgctt cgctctcgct gatcgttggg atcattgggg tagtggggtg
     1141 tttcctcggc ttggggcctc gtcaaggtgt ttcgcctgaa cggctcaagt cgcgctgcgc
     1201 gctccaagtg agcgctcgta ttaactcgct tgacttcgtc gccctttggc tcctcgtgat
     1261 cgttgggatc gtaggggtgt gagcgctcgt attaactcgc ttgacttcgt cgccctttgg
     1321 ctcctcgtga tcgttgggat cgttggaact gtagttattg ttataatcgt tgaagatcgt
     1381 aggcgctcgc attggctcgc ttaattcgtc gcctttttgg gctcctcata caccgcaggg
     1441 ggtgataacg tgcacgtgca cgtgcactga agtggctggt aatactaggg ctgactcgtt
     1501 gcactcgctg cgcccttcag ccacttccta gagggatcgt aggatttcta gggatcgaag
     1561 gatcgaagag atgatgacta cgttcggggc tgcgcgccct cactgcgcgc tgcgcttgcc
     1621 tacgttacgg cgctgcgcgg cctccactgc gcggtcgtac ctcccttgcc tgcgcgcgct
     1681 cgtacctcgc acgctgcgcc gccgctatag tcgctgcgct ccttcgctct gttgcggggg
     1741 gttagatagg ttggctggtt tggtggtgag gtgctagatg tgtgcttttg ggctttttgc
     1801 tcaccgcaaa aaactgcacc gcgctgcgcg cccgcccatg aaggtgccga aaatttctcg
     1861 tagggttcgt aagaaattta attcacaaag tgaatcgaat atgaaaaaaa accatttatt
     1921 ggaaaaaatt aagtatagaa agaatttaag ttaacgttgc gttcaacggc atcacgaaga
     1981 tatcgcaaga cgacggagat gttggaaggg tgccagctgt accgatggta atgatcgctt
     2041 gtacggtcgc actcgggata gtaaacgtag tgtacaccga ggtgttagga cccgaaatag
     2101 caccggtgta gagaggtgct gagatgccgg acgcgctccc gttttggaga gaaataagag
     2161 ccgtagcacc agcgttaaaa gacactgatc cagggaccac gttgcctgta tttgctcctg
     2221 tttgctgaac gtataacgca aacttaccaa gagtaccaag ggggaacgta atcgtggtgc
     2281 tcgagatcgt caggcccata ttgtcgaaga ccgtagtgcg tgtagatccg agagggagaa
     2341 ggttggtata attcgtagaa gtataatgcg cggatggaag cgcaagttgg cccgcgccaa
     2401 gaatagggga aatgagctca acgtcatatg aaacccaaag ctctccgatc gtatcagtcg
     2461 agttcaagtt gctgccgcgc gcaatcgtga aggtaccaat gtcgtaaaga agctcggaag
     2521 aagtagaggc aagagcgctc gaacgaacgt aaaattcgga aacctgattg tgcttagggt
     2581 cgcactccac agggaaaata atatcagtcg atggtttgct ggacgccgaa aacatcgcgt
     2641 tttcgatctg tgacttagtt acccagaaag gactatcgga gttgtagtcc accgcaccgg
     2701 ctacatagcc ttgagactgc ttgttctgat cgtcaccaga aagagatttg aactcaaata
     2761 tcaaaccatg gaaccggtat gaagagaagt ttcgcgcaat actgctcaac caagggaatg
     2821 tttgggccat accgatatta attggataat atgtcgcagc aaatggagta gaagaaccga
     2881 cgaagtcttg gatgaactca cgatggcgaa tcctcgtcga cgtactggat aatttcatga
     2941 acggcacttg attcgaagga gtaagggtct gaaccatgtt gttttgtttg aggtcgtacg
     3001 ctccaaaacc gagaaacttg ctgaacatgt tttgtgcagc attacccaag tagccgccaa
     3061 tttgactgcc aatagaaggg cgcgatgcag gtgagatcgc tttggagtag gggacagcgc
     3121 gcgccttacg aacacgttta acagctttgg gccgcatgta acgagtagta ggacgcttac
     3181 gcttaataat ttttttccga ggaacgaaag gcattgttga ataatttatg attctaatag
     3241 tagaaatcgt tcctttaaat agaaataaaa ataggggtga aaatttttga aaaaaattta
     3301 gaaaaaatag tcgtcttaaa gactcgaaat tacatagtag aaaagaaaaa gtaaaaatgc
     3361 aaagcaatga aaatttcgaa aacgacagga tgtcagaaaa cgacatcaat tacgcgctgc
     3421 tcacgccgcg cccagcgcct gacggcgtgg atgaagaagt tgttgttatt gaaaatccta
     3481 agaaacgtcg aatctcagat acggaagaa
//
LOCUS       CruV-283                3511 bp    DNA     circular     24-FEB-2020
DEFINITION  Crucivirus-283 isolate 001459_virusbaton.
ACCESSION   CruV-283
KEYWORDS    .
SOURCE      Crucivirus-283
  ORGANISM  Crucivirus-283
            Unclassified.
REFERENCE   1  (bases 1 to 3511)
  AUTHORS   de la Higuera,I., Kasun,G.W., Torrance,E.L., Pratt,A.A.,
            Maluenda,A., Colombet,J., Bisseux,M., Ravet,V., Dayaram,A.,
            Stainton,D., Kraberger,S., Zawar-Reza,P., Goldstien,S., Briskie,J.,
            White,R., Taylor,H., Gomez,C., Ainley,D.G., Harding,J.S.,
            Fontenele,R.S., Schreck,J., Ribeiro,S.G., Oswald,S.A., Arnold,J.M.,
            Varsani,A., Enault,F. and Stedman,K.M.
  TITLE     Direct Submission
  JOURNAL   Submitted (24-FEB-2020) Biology, Portland State University, 1719 SW
            10th Ave, Portland, Oregon 97201, USA
COMMENT     Bankit Comment: ALT EMAIL:ide@pdx.edu
            Bankit Comment: TOTAL # OF SEQS:451
            Bankit Comment: GAP: est. length - alignment

            ##Assembly-Data-START##
            Assembly Method       :: MetaSPAdes v. 3.12; IDBA-UD v. 1.1.1;
                                     MEGAHIT v. 1.0.3
            Sequencing Technology :: 454; Illumina
            ##Assembly-Data-END##
FEATURES             Location/Qualifiers
     source          1..3511
                     /organism="Crucivirus-283"
                     /mol_type="genomic DNA"
                     /isolate="001459_virusbaton"
                     /isolation_source="Water"
                     /country="France"
                     /collection_date=2013
     CDS             1..798
                     /codon_start=1
                     /product="Putative Replication Associated Protein"
                     /translation="MAATHDEITIQPVELFPSLRRDCKAWCFQFEKSESGYLHFQGRL
                     SLKTKGRVQQAKLAIGFNWAHLTPTSTANRDNNFYVMKEETRLHGPWSDKDVVWPANW
                     GEWDLPHHWQAEIIDEIEQPAHDRKINVLIDQKGGIGKSKLVEHLERFHKARYIPAVF
                     EQALDIMGWVMSFDAAPIYLIDYPRSEETKRSHWAAIETIKGGRAFDKRYKGKEMRFD
                     KPHIWIFTNSCPKSNWLTKNRWLFWTVTPMTKRLRELNLVTALEENQ*"
     CDS             complement(1056..2336)
                     /codon_start=1
                     /product="Putative CruV Capsid Protein"
                     /translation="MGGFFGAERYARRVQRRQNFGKVALGALRMFPGIGAAIPGGVNM
                     SGMGDYNIRYNSIMNGGDGSTETDYVPALSNGGTRGSVRVSHREYIRDVSSTTGFLNL
                     PFAINPANSSCFPWLATLARNFQEYQIHGMVFHYVSQSSDALNSTNTALGSVVMSTDY
                     NSGNAPYASKQAAENAEYTVSCKPSCSLVHGIECDPAMTVNQGHLYISPFSDGTVPTG
                     QDIKFYNLANFQFMTQGSQASATIGELWVSYDISLIKPIDNGQLNQSAADHFALDTLG
                     QSTGTTYNFGSKTPVKLLSGVGTTITHVAGDNTITFPQSSIGGTVYMVSYNCTGSATT
                     GSAALGTVTVNSGAEFLDVLTTTGTASHTAAGNGAGVATQMSLFCIVRITSAATLTSP
                     AIITFGSTSQNLPNNGNGDLIIAQLPSLFGVLPP*"
     CDS             complement(2341..2781)
                     /codon_start=1
                     /product="putative ORF"
                     /translation="MSEPSPKRLRIDENLDLDAPVDPINDYAHPIGPINFRETLGAHN
                     VPADLKAAWRNDKKTHTKAWFRKSLKELPLEVKKIILRNTFRTRCLDSKKGRKKGSSK
                     KKRSVSKRKYKRTYKRRTTFRRGSFWGGARRRFYPRRRRSFWFR*"
     CDS             3065..3448
                     /codon_start=1
                     /product="putative ORF"
                     /translation="MSENETNSDSSDFGSFSGGTSSTSIDSQSATFEDAPDAEGSYNH
                     PILARYGTSSSFVRRGGINPIPESLVGRRLQPTNLRVDGATEINGSIAQGAASSSGIG
                     EKRKHKGKQVRFVPSNVIYTYYFKE*"
     CDS             3075..3401
                     /codon_start=1
                     /product="putative ORF"
                     /translation="MRPILTPLILEASLEEPLQQALTHRAQHLKMLQTLKGATITRYL
                     QDMAPHLPSSDEVESIQFLKAWLDDVYNLLICESMEQLKLMEALHKVQLQVLELEKKE
                     NTKENK*"
ORIGIN
        1 ttggctgcca cccatgatga aataacaatt caaccagttg aattgtttcc atcattacgt
       61 cgagattgta aggcttggtg cttccaattc gaaaaatcag agtctggcta cttgcatttc
      121 caaggaaggt tgagcttgaa aactaaaggt cgagttcagc aagcaaagct tgctattgga
      181 ttcaactggg cccatttgac tccaacctca actgcaaatc gtgacaacaa cttctatgtc
      241 atgaaggaag agacgcgctt gcacggacct tggagtgaca aagatgttgt ttggcctgcc
      301 aattggggag aatgggacct tccacaccat tggcaagctg aaataattga cgaaattgaa
      361 caacctgcgc atgatcgtaa gataaacgtg ctcatcgatc agaaaggagg tattggaaag
      421 tccaaattgg tggagcattt ggagagattt cacaaggcga ggtacatccc tgctgtcttc
      481 gagcaagctc tcgacatcat gggctgggtg atgtctttcg acgctgcccc catctaccta
      541 attgactacc caagatctga ggaaacaaag cgttcacatt gggccgcaat tgagacgatc
      601 aagggaggca gagcctttga taagcgctac aaaggtaagg aaatgcgctt tgataaacct
      661 catatttgga tcttcactaa cagctgtcct aagtccaact ggttgaccaa aaatcgctgg
      721 ctcttttgga ccgttacccc catgacaaaa agattaagag aattaaatct tgttactgct
      781 ttggaagaaa atcaataaat tgtttttttc tcaaatccct aacttggggg tggctaacgc
      841 cccccaaacc ccctgtttgc ctttggcact cggccttggg tccggaccct gcgggtcctg
      901 cggcctcggg tagagtagcc ccctgcgggg ggcgtaggcc ttagagaact aaccccatgc
      961 ccggccctgg ccctggccct ggccttggca ttttattttt tttacccttc gctcctaacc
     1021 gcaagtcttt tttattgaat tctcgagaat aaaaattaag gaggaaggac accaaaaaga
     1081 gatggaagtt gagcgatgat taaatcaccg tttccattgt taggtaggtt ttgagaagtt
     1141 gacccaaaag taataatggc tggacttgtc agagttgctg cagaggtgat tctcacaatg
     1201 cagaacaacg acatctgagt tgcaactcca gctccattac cagcagcagt atgactagct
     1261 gttcctgttg tggtcaaaac atcaagaaat tcagctccac tgttaacagt gacagttccg
     1321 agggctgcac ttcctgttgt tgcacttcct gtacaattgt aagaaaccat gtaaacagtg
     1381 cctccaattg aagattgtgg aaaagtaatt gtgttgtctc cagcaacatg ggtaatggtt
     1441 gttccaaccc ctgacaagag cttcacagga gttttgctac cgaagttgta ggtggtccct
     1501 gttgactgtc cgagtgtatc caatgcgaaa tgatcagcgg cagattgatt aagttgaccg
     1561 ttgtcaattg gcttaatcaa agagatgtca taactcaccc agagctcccc aatggtcgca
     1621 ctggcttgtg acccctgagt catgaattgg aagttggcta ggttgtaaaa tttgatgtcc
     1681 tgccctgttg gaactgtgcc atcagaaaat ggtgaaatgt acaagtggcc ttgattcaca
     1741 gtcatagcag gatcacattc aattccgtgc acaagtgagc aactgggctt gcagctaaca
     1801 gtgtactccg cattctcggc agcttgctta ctcgcatacg gtgcattccc ggaattgtaa
     1861 tctgtgctca taacaactga acccaaggcg gtgttagttg agttaagagc atcagatgat
     1921 tgagaaacgt aatgaaaaac cattccgtgg atttggtact cttgaaaatt tcgagcaagg
     1981 gtggcgagcc acggaaaaca actagaatta gccgggttaa tagcgaatgg aagattgagg
     2041 aatccggtgg tcgaactcac atctcgaatg tattctcgat gcgagacacg aacggatcct
     2101 cgagttccac cgttactaag ggcaggtacg tagtcggttt ccgtggaccc gtccccgccg
     2161 ttcataatcg agttgtacct tatgttgtag tctcccatgc cgctcatgtt tacaccgccg
     2221 ggaatggctg cgccaatccc tggaaacatg cgaagggctc ctaacgcgac ttttccgaaa
     2281 ttctgtctcc tttgcactcg tcgagcatac ctttctgcgc cgaaaaatcc tcccatttta
     2341 tcaacggaac cagaaactgc gtcttctcct tgggtaaaat cgtcgtcgag ctcctcccca
     2401 gaaactgcct cgtcggaaag tagttcgtcg cttgtaggtg cgcttgtact ttctcttgct
     2461 cacagaacgc ttcttcttcg aagaaccctt ctttcgacct ttctttgaat ctaaacaacg
     2521 tgtacgaaaa gtattacgga gaataatctt cttcacctca aggggcagct ctttcaacga
     2581 tttgcgaaac caagccttcg tgtgggtctt cttatcgttt ctccaagctg ctttcagatc
     2641 agcaggaacg ttatgtgcac ctagagtttc tctgaaatta ataggtccga ttgggtgggc
     2701 gtaatcgttg ataggatcaa ctggagcgtc taaatccagg ttttcgtcaa ttcgcaagcg
     2761 cttcggtgaa ggctcgctca taaaagaaaa gaaaaagggt tagaaaacgg tgatgttatc
     2821 gtttttttta tttttttttc ttgttctatt tatatttgtt atttttcaaa agggaacaaa
     2881 tgcgcggaaa gtgcgcggaa ataggtcgga aaatcggaaa atcggagcga taatttcgcc
     2941 gaagtgcttt tttttccgac ggttaattaa ctgacgttaa taatatatat aaagaggcgc
     3001 tacgctgtaa tactactctt tatatatatt attactttaa cgtgtggacc cagcggaccc
     3061 aattatgagc gaaaatgaga ccaattctga ctcctctgat tttggaagct tctctggagg
     3121 aacctcttca acaagcattg actcacagag cgcaacattt gaagatgctc cagacgctga
     3181 agggagctac aatcacccga tacttgcaag atatggcacc tcatcttcct tcgtcagacg
     3241 aggtggaatc aatccaattc ctgaaagctt ggttggacga cgtctacaac ctactaattt
     3301 gcgagtcgat ggagcaactg aaattaatgg aagcattgca caaggtgcag cttcaagttc
     3361 tggaattgga gaaaaaagaa aacacaaagg aaaacaagtg agatttgttc cttctaatgt
     3421 catttacact tattatttta aagaataaaa taaaaaggtt gtgagaattg acgatgagag
     3481 agaaacaaga gtctgcactt gggattttac t
//
LOCUS       CruV-284                3515 bp    DNA     circular     24-FEB-2020
DEFINITION  Crucivirus-284 isolate 120_1287_IR2_3297.
ACCESSION   CruV-284
KEYWORDS    .
SOURCE      Crucivirus-284
  ORGANISM  Crucivirus-284
            Unclassified.
REFERENCE   1  (bases 1 to 3515)
  AUTHORS   de la Higuera,I., Kasun,G.W., Torrance,E.L., Pratt,A.A.,
            Maluenda,A., Colombet,J., Bisseux,M., Ravet,V., Dayaram,A.,
            Stainton,D., Kraberger,S., Zawar-Reza,P., Goldstien,S., Briskie,J.,
            White,R., Taylor,H., Gomez,C., Ainley,D.G., Harding,J.S.,
            Fontenele,R.S., Schreck,J., Ribeiro,S.G., Oswald,S.A., Arnold,J.M.,
            Varsani,A., Enault,F. and Stedman,K.M.
  TITLE     Direct Submission
  JOURNAL   Submitted (24-FEB-2020) Biology, Portland State University, 1719 SW
            10th Ave, Portland, Oregon 97201, USA
COMMENT     Bankit Comment: ALT EMAIL:ide@pdx.edu
            Bankit Comment: TOTAL # OF SEQS:451
            Bankit Comment: GAP: est. length - alignment

            ##Assembly-Data-START##
            Assembly Method       :: MetaSPAdes v. 3.12; IDBA-UD v. 1.1.1;
                                     MEGAHIT v. 1.0.3
            Sequencing Technology :: 454; Illumina
            ##Assembly-Data-END##
FEATURES             Location/Qualifiers
     source          1..3515
                     /organism="Crucivirus-284"
                     /mol_type="genomic DNA"
                     /isolate="120_1287_IR2_3297"
                     /isolation_source="Water"
                     /country="Norway"
                     /collection_date=2011
     CDS             1..1077
                     /codon_start=1
                     /product="Putative Replication Associated Protein"
                     /translation="MVKNVATHKNPSNAVQCITFDFRISQMRTDESCFTADQIGQCLL
                     KLCKKFVFQLEQGEQTNYLHFQGRVSLIKPTLKMTLLSQIQKLLSCSIEDAPQYLSPT
                     VKGVHKTQNFNYVLKDSTKKEGPWTEKDFKPDDLKPVQYIPKQFQGIVDKLYPYQKTI
                     IDSRLVFDPRIVNIIYDPVGNNGKSTVASLAELLHGGIDVPPINDFKELMQLVCDECM
                     HTQNRSPSPILIDMPRGMDKFKLAGIYTAIEQIKKGKLFDTRNRYKKYWIDSPQIWVF
                     TNEYPELHFLSADRWKIWKISETKELVAYTAKEHNAHLVTKELKKRDKEVNINYFTYP
                     KKTSYLHDKSGCAGINHDKVISNM*"
     misc_feature    507..535
                     /note="stem-loop"
     misc_feature    516..524
                     /note="nonanucleotide"
     CDS             1428..2816
                     /codon_start=1
                     /product="Putative CruV Capsid Protein"
                     /translation="MPRKLFKKVAKVGKSLIKDVVSAVTSGNVDPASIARSVATKNVR
                     RLAKRAGVADAIGKHAAQKMVGNMESMIKGIKGRGDYISESADLDHNVLIHGVHGTAK
                     PIPQFSAKSMKDGRSMMIAHREFIKDVPGSIAFRSERYNIDPTNATTFPWLSTIAPLF
                     QKFRFHGCVFEFNSTSSNALTGTNTALGTVILVQQTNAHAANFTTKVQMENTYGAISA
                     KPSNNIACGVECSPALVIEPSYIRHAGDIGVMDVRLNQGYGYMQYATVGMQAQDFIIG
                     EMWVSYMVELIDPILGVAPNSILPVAHYSLDNTVGDAFFGQRIFGTTRTELVNTMGVT
                     IEPLSVSNRILIPASAFVDLPTPVNIVMSYVLQGDTSTSNLTGFGGTATNAVNNNIYT
                     LLGVPSSASQVASAGGLGSSFYENNAWTVTDPTQDVILDYITATCVFPLDASPVVPLC
                     GDLFISIYNASL*"
     CDS             2803..3138
                     /codon_start=1
                     /product="putative ORF"
                     /translation="MLHCNFNYRCRLYIEMGNYASYTELPINEENTPITIEDIPIVHR
                     RPKLPDTLRILMTIEGNETSEQFEYQIPTTLPNYKKLQHIITLLHSMDISIELIKPHS
                     NILQSITII*"
ORIGIN
        1 atggtaaaga atgtagcaac ccataagaac ccttctaatg ctgttcaatg catcactttt
       61 gattttcgaa ttagtcagat gcgaacagat gagtcatgtt ttacggccga tcaaataggg
      121 caatgtttgc tcaaactttg taaaaaattc gtttttcaat tggaacaagg tgaacaaacg
      181 aattatttgc attttcaggg gcgggtttct ttaataaaac cgacactaaa gatgacccta
      241 ttatcgcaaa tacaaaagct cttatcttgc tcaatagaag atgcgccaca atatctgtca
      301 ccaactgtaa agggtgttca taagacacag aattttaatt atgttctaaa agatagtaca
      361 aaaaaggaag gtccatggac tgaaaaagat tttaaacctg atgatttaaa accagtacaa
      421 tatataccga aacaatttca aggaattgtt gacaaattat atccctacca gaaaacaatt
      481 atcgatagta gattagtgtt tgaccccaga atcgtgaata ttatatacga tcccgttggt
      541 aataatggta aatctacagt agctagttta gctgaattat tacatggcgg gatcgacgtt
      601 ccccccatta atgattttaa ggaattaatg caattagtat gcgacgaatg tatgcacaca
      661 caaaatagat caccatcacc aattttaatt gatatgccga gagggatgga taaatttaag
      721 cttgctggaa tttacacggc cattgaacag atcaaaaaag gtaaactttt cgatactcgt
      781 aatcgttata aaaaatattg gatagatagt ccacagatat gggtctttac aaatgaatat
      841 cctgaacttc attttttaag tgccgataga tggaaaatat ggaaaatctc agaaacaaaa
      901 gaactagttg catatactgc aaaagagcat aatgctcatc tagtaacaaa agaattgaaa
      961 aagagagata aagaagtaaa tattaattac tttacgtatc ctaagaaaac atcataccta
     1021 catgataaaa gtggttgcgc tggcataaat catgataagg taattagtaa tatgtaacat
     1081 atacctcgtg tatttaactt ttccatttat aattattttt tatttcttga aaatgaataa
     1141 aaaataaata aaaatataaa attacattac cacgtaacga cgtcatagtg tctggaagac
     1201 attaacgtaa tattttagat tgtaagctga tgtatcaaaa atagtttgaa tggggagcat
     1261 ggcttcagcc tgctccagct ttggactatg atacatctga tcgcttgcaa agaaaatatg
     1321 agtgattggc ttacatggcg ctatgaaagg agtggagatc ccactcggga tttccgctcc
     1381 tgatcgtcgc tcataaaata atatcttaag atatagtata caaaacaatg ccccgcaaat
     1441 tgttcaaaaa agtagccaaa gtaggtaaaa gtcttattaa agacgtcgtg tccgctgtta
     1501 ccagtggaaa tgttgaccct gcatcaatag ctcgttctgt ggccactaaa aacgtccgta
     1561 gacttgctaa aagggcaggt gttgccgatg caattggtaa acacgccgcc caaaaaatgg
     1621 taggtaacat ggaatctatg attaagggta ttaagggccg aggcgattat atttcagaat
     1681 cagctgatct agatcataat gttcttattc atggagtcca tggaaccgct aaacccatac
     1741 ctcaattctc tgcaaagagt atgaaagatg gacgatcaat gatgattgcc catcgagagt
     1801 ttataaaaga cgttccgggg tcgatagctt ttcgatccga acgttacaat atagacccaa
     1861 ctaatgcgac aacctttcca tggttgtcga ccattgctcc gttgtttcaa aaattcagat
     1921 ttcatggctg tgtttttgaa tttaattcaa catcgagcaa tgcactaact ggcacaaata
     1981 ccgcccttgg aacggttatc cttgtccaac aaacaaatgc acatgctgca aactttacta
     2041 caaaagtaca aatggaaaac acctacggtg caatttccgc aaaaccttca aataacatcg
     2101 cttgtggtgt agagtgctcg cctgcactcg ttattgaacc atcatacata agacatgctg
     2161 gcgacatcgg cgtcatggat gtccgactca atcagggata cggctacatg caatatgcaa
     2221 cagtcgggat gcaggcccaa gactttatta ttggtgaaat gtgggtgtca tacatggttg
     2281 agcttataga tcctatcctt ggcgtagccc ctaatagcat acttccagtg gctcattatt
     2341 ctctagataa taccgttggc gatgcattct ttggccagag aatcttcgga acaactcgta
     2401 cagagttggt caatacaatg ggtgttacta tagagccctt aagtgtttct aatagaatac
     2461 tcatcccagc gtcagcgttc gtggaccttc ctacaccggt aaacatagtt atgtcatacg
     2521 tccttcaagg cgatacatca acaagtaacc ttacaggctt tggcggtact gcgacaaatg
     2581 ccgttaataa caatatttat actctcttgg gagttcctag ctctgcctct caagtggcat
     2641 cagctggagg cctcggttca tcgttttatg aaaataatgc gtggactgtg accgacccta
     2701 cacaagatgt aattttagat tacatcacag ctacatgtgt ctttcctcta gacgcatcac
     2761 cagtagttcc attatgtggt gatttattta tttccatata taatgcttca ttgtaatttt
     2821 aattatcgat gtagattata tatagaaatg ggaaattatg catcatatac ggaactacca
     2881 attaatgaag agaatacccc gattacaatc gaggatatac caatcgtaca tagaagacct
     2941 aaattacctg atacattaag aatattaatg actatcgaag ggaatgaaac atcagaacaa
     3001 tttgagtatc aaattccaac aacattacca aattataaaa agcttcagca tataattact
     3061 ttattacatt cgatggatat atcaatcgaa ttaataaagc cacatagtaa tatattacaa
     3121 tctattacaa taatataaca atctaataag tcaccttatt acttatatta ataatataat
     3181 gtttacaatt atattattag aatacttttt tttacggacg agaatatttc gagtgttctt
     3241 ccagattgct cgccaatcgc cgtaaaaaaa agcttatttt aaattcattt caaaaagcgc
     3301 atttgtcggg cagaccatcg atgaaatcga ttgtcgtggt gccagatagt cattatcttt
     3361 taattttgtg ggccctcata agatcgttta cgattgttgt gatctcataa aattatatta
     3421 gaatggctat tggcatcgtc gacataatgg catataatta tcataagtat aatattacaa
     3481 tattaaatat cttattacaa tatatagtaa actaa
//
LOCUS       CruV-285                3517 bp    DNA     circular     24-FEB-2020
DEFINITION  Crucivirus-285 isolate 001462_virusbaton.
ACCESSION   CruV-285
KEYWORDS    .
SOURCE      Crucivirus-285
  ORGANISM  Crucivirus-285
            Unclassified.
REFERENCE   1  (bases 1 to 3517)
  AUTHORS   de la Higuera,I., Kasun,G.W., Torrance,E.L., Pratt,A.A.,
            Maluenda,A., Colombet,J., Bisseux,M., Ravet,V., Dayaram,A.,
            Stainton,D., Kraberger,S., Zawar-Reza,P., Goldstien,S., Briskie,J.,
            White,R., Taylor,H., Gomez,C., Ainley,D.G., Harding,J.S.,
            Fontenele,R.S., Schreck,J., Ribeiro,S.G., Oswald,S.A., Arnold,J.M.,
            Varsani,A., Enault,F. and Stedman,K.M.
  TITLE     Direct Submission
  JOURNAL   Submitted (24-FEB-2020) Biology, Portland State University, 1719 SW
            10th Ave, Portland, Oregon 97201, USA
COMMENT     Bankit Comment: ALT EMAIL:ide@pdx.edu
            Bankit Comment: TOTAL # OF SEQS:451
            Bankit Comment: GAP: est. length - alignment

            ##Assembly-Data-START##
            Assembly Method       :: MetaSPAdes v. 3.12; IDBA-UD v. 1.1.1;
                                     MEGAHIT v. 1.0.3
            Sequencing Technology :: 454; Illumina
            ##Assembly-Data-END##
FEATURES             Location/Qualifiers
     source          1..3517
                     /organism="Crucivirus-285"
                     /mol_type="genomic DNA"
                     /isolate="001462_virusbaton"
                     /isolation_source="Water"
                     /country="France"
                     /collection_date=2013
     CDS             1..1875
                     /codon_start=1
                     /product="Putative Replication Associated Protein"
                     /translation="MLGHWVSRSYFPHGEPRGALRVECAPFGGSATCEVCTWPSQAYD
                     PYSSPHRSTMRYSVHRSGHVFTLAPVTWVNSFFRDCAGTLIPSSSLLIDMPAKARTAD
                     LGLDEKNEQLPLDDYKVVAGKLKRSDDKSYPKRKNYQHPAGKYRGFVFTNFKVDWDTE
                     EMKEWTSVNFASIGLEKTKTGRLHHQGCCYFKNPRVCSGFSKLLPPNTWFRPARGDEC
                     SNNKYTSKEGNRILLIGRPIAQGERVDLEEIRDRIINGGATPNEIACESGTSFEDIRF
                     AETIFRLAGSAPKRTWKTTVIWLYGSAGRGKSHIAKALIGDDDCWKSSAKLDYWQQYQ
                     GQETVWFDEFRGDKCTFTALLEIMDSTPYEVNIKHGSHQLLARRLIVTSPMHPDKIYP
                     SCAENVNQMLRRIDVLWEIDELYEAAASGFEFPAVPEFPEFGAVIAACSASRLVAKNP
                     LRCEGTKCLELINNCWVPKVIADVEQPGVPGTLVVRTQSRRGRAPTTIDSVSGTEVGG
                     NIRSDTGVVPRPVSSAPTSGLTSEPPCEYKEDKFLLSVDDCQHEASAEDEAGNEICYG
                     CGAMGIVNNDGIFEPFVTGLPSDDDEGDEGDTFDDDAMSDPPYEDEEFGQVQIAQPLR
                     *"
     CDS             complement(1932..3443)
                     /codon_start=1
                     /product="Putative CruV Capsid Protein"
                     /translation="MYACIHCDVMPRPAAAKVASPAGYAAYLKYMGSSAKRAPVKRRT
                     VRRKTNESHGSIARRSVRRAGTSDLGSMLGGLAGMALGGPTGAMIGNAVGGVGGNMIS
                     RIFGSGDYQVSNAGSLKKNNIALANAANIPQFGTGKVASKFVHREFLGDVISSSTAGA
                     FKIDSYAINPGQAGTFPWLSGVVGAKYQQYRINGLTFEYRSMSADALNSTNTALGSVI
                     MSTDYDSADVVFTTKQQMENTENGVSCKPSVNMMHGIECQRSQTPVSELFVRAFGVPT
                     SLADIRLYDLGRFSIASTGCQGTNVTLGELWVTYDIDCFKAIEQVPLYLQPFSQYSLV
                     GQAAAFPLGTSQPQLLTAASNSIPLLITGNRIRFPISTVIGSIFHVEWHVLGSSAALT
                     PPVVTSGAGMFINTNTVWKSTASTTPNAFLTCDVQYQGGATPANPPYLNLANYTWATT
                     VVQADLYVYQMSGQYPGDYSSQLEVLPPEDEDVETVYVQQLEEQKKPQHGRGR*"
ORIGIN
        1 atgctggggc attgggtctc acgatcatat ttcccccacg gggaaccacg tggagcactc
       61 agggttgagt gtgccccctt cgggggcagt gcaacctgtg aggtttgcac ttggcctagt
      121 caggcatatg acccttatag ctccccccac cgttccacta tgcgctattc agtgcatagg
      181 tcgggccatg tattcaccct tgctcctgta acgtgggtta attctttttt tagagattgt
      241 gctggcacac ttataccgag ttcctcgcta ctcatcgaca tgcctgctaa agcccgcacc
      301 gcggatttag gtctcgacga gaaaaatgaa cagctcccgc ttgatgatta taaagtggtc
      361 gctggaaagc taaagagatc ggacgacaag tcttatccca agcgcaaaaa ttaccagcat
      421 cctgctggga aatacagagg tttcgtattt acaaatttta aagtcgattg ggacaccgaa
      481 gagatgaaag agtggacttc cgtcaacttt gcatctattg gacttgaaaa gaccaaaact
      541 ggacggcttc accatcaagg ttgttgctat ttcaagaacc ctcgcgtctg ctctggtttc
      601 agcaagctgc tcccgcctaa cacttggttt agacctgcgc gtggcgatga atgctcgaat
      661 aacaagtata cgagcaaaga aggcaacagg attctcctta ttggccggcc aattgcccaa
      721 ggcgaacgtg tcgaccttga agaaattcgt gaccgaatta ttaacggagg tgctactcct
      781 aatgagattg catgtgaatc tggcacttcg tttgaagata ttcgtttcgc ggaaactatc
      841 tttcgtttgg ctggctctgc acctaaacga acctggaaaa ctacggttat ctggttgtat
      901 ggctccgctg gtcgcggaaa atcgcacatc gctaaagcgc tgattggcga tgatgattgc
      961 tggaaatcgt cagccaagct ggattactgg cagcaatacc aaggacagga gactgtttgg
     1021 tttgatgaat ttcgcggtga taaatgcacc tttaccgctt tgctcgaaat aatggactct
     1081 actccgtacg aggtcaatat caagcatggt tctcaccaat tgcttgcccg gcggctaatc
     1141 gtaacaagtc cgatgcatcc tgacaagatc tatccatctt gtgccgagaa cgtaaatcaa
     1201 atgctgcgcc gtattgatgt actatgggaa atcgacgagc tttatgaagc tgccgcctct
     1261 ggcttcgagt tccctgccgt gcccgaattt cctgaattcg gtgccgtcat cgccgcgtgt
     1321 tccgcgtcga gattagttgc taaaaatccg ctacgctgcg aaggcacgaa atgcctcgag
     1381 ctcattaata actgttgggt tccaaaagtg attgccgatg ttgagcaacc tggtgttccg
     1441 ggaactctag tggtgaggac gcaatctcgg cgcggccgtg ctcccacaac tatagattct
     1501 gtttccggaa cagaagtcgg gggtaatata cgctctgata ccggtgtcgt tcctcgaccg
     1561 gtgagctctg ccccgacttc tgggctaaca agtgagcccc catgtgaata taaagaggat
     1621 aaattcctct taagtgtgga cgattgtcaa cacgaagctt ccgctgaaga cgaagctggc
     1681 aatgaaatat gctatggctg tggagcaatg ggcatagtta ataacgatgg aatatttgaa
     1741 cctttcgtca caggattacc atctgatgac gatgaaggtg atgaaggtga tacctttgat
     1801 gatgacgcta tgagcgaccc accatacgag gatgaagaat ttggccaagt acaaattgct
     1861 cagcctcttc gttaaatatt cacaaaaaga actttagttc tattgctttc taagcaatta
     1921 cgcagtcctg cctatcggcc tcggccatgc tggggctttt tttgctcctc gagttgttga
     1981 acataaactg tttcaacatc ttcatcctct ggaggaagaa cctcaagctg agacgaataa
     2041 tctcccggat actgtccgga catctgatag acgtacaaat cagcctgaac tactgttgta
     2101 gcccatgtat aatttgcgag attcaaatac ggaggattcg caggggtggc accaccctga
     2161 tactgcacat cacaagtcag aaaggcattc ggagttgtac tcgctgttga tttccaaacc
     2221 gtatttgtgt tgataaacat tccggcaccg gatgttacaa caggaggagt caaagcagct
     2281 gaagatccca atacatgcca ttcaacatgg aagatacttc caataactgt tgaaattggg
     2341 aatcgaatac gatttcctgt gattaacaaa ggaatagaat tcgatgctgc tgtaagaagc
     2401 tgcggctgag acgtacccaa tgggaaagct gccgcttgtc ctaccaatga gtactgcgag
     2461 aatggctgaa gatacaaagg cacttgctcg atggccttaa agcaatcgat atcataagtc
     2521 acccaaagct cgcccagcgt cacattcgtg ccctggcagc cggttgaggc aatagaaaat
     2581 cgccccaaat cgtaaagacg aatatcggct aatgatgttg gcacaccaaa cgcacgaaca
     2641 aacagctcac tcactggtgt ttgcgatcgc tggcattcaa tcccatgcat catgttgact
     2701 gaaggcttac acgacactcc gttttccgtg ttttccatct gttgttttgt tgtaaaaacc
     2761 acatctgctg aatcatagtc tgtcgacatg ataactgacc ccaaagctgt gttggttgag
     2821 tttaaagcgt ctgccgacat tgaccggtac tcaaaggtaa gaccattgat tcggtactgt
     2881 tggtactttg caccaactac tccggaaagc cacggaaagg ttcctgcttg tccaggattg
     2941 atagcataac tgtctatctt aaaagctcct gccgtggagg agctaataac atctccaagg
     3001 aattctcgat ggacaaattt ggaagctacc ttaccggtac caaattgtgg aatatttgct
     3061 gcatttgcca atgcaatatt gttcttcttt agggaacctg cattcgacac ttgatagtct
     3121 ccactaccaa agatgcgaga aatcatgttt ccaccaacgc cgccaacagc gtttccaatc
     3181 atggcaccgg tgggaccacc taacgccatt cctgccaggc ctcccaacat cgaaccgagg
     3241 tcggaagtac ctgcacgacg aaccgatcta cgagctattg acccgtgcga ttcgttcgtt
     3301 ttacgacgta ctgtacgtcg tttcacggga gctcgctttg cggagctccc catatacttt
     3361 agatatgcgg catatcctgc cggtgatgcc actttcgctg ctgcgggacg aggcatgacg
     3421 tcgcaatgta tgcacgcata cattcctcta atttaaaata tttaaatgac tgttatggct
     3481 tcgggattca tgaacaggtc tactcgctat acaccga
//
LOCUS       CruV-286                3518 bp    DNA     circular     24-FEB-2020
DEFINITION  Crucivirus-286 isolate 000653_ADNa22.
ACCESSION   CruV-286
KEYWORDS    .
SOURCE      Crucivirus-286
  ORGANISM  Crucivirus-286
            Unclassified.
REFERENCE   1  (bases 1 to 3518)
  AUTHORS   de la Higuera,I., Kasun,G.W., Torrance,E.L., Pratt,A.A.,
            Maluenda,A., Colombet,J., Bisseux,M., Ravet,V., Dayaram,A.,
            Stainton,D., Kraberger,S., Zawar-Reza,P., Goldstien,S., Briskie,J.,
            White,R., Taylor,H., Gomez,C., Ainley,D.G., Harding,J.S.,
            Fontenele,R.S., Schreck,J., Ribeiro,S.G., Oswald,S.A., Arnold,J.M.,
            Varsani,A., Enault,F. and Stedman,K.M.
  TITLE     Direct Submission
  JOURNAL   Submitted (24-FEB-2020) Biology, Portland State University, 1719 SW
            10th Ave, Portland, Oregon 97201, USA
COMMENT     Bankit Comment: ALT EMAIL:ide@pdx.edu
            Bankit Comment: TOTAL # OF SEQS:451
            Bankit Comment: GAP: est. length - alignment

            ##Assembly-Data-START##
            Assembly Method       :: MetaSPAdes v. 3.12; IDBA-UD v. 1.1.1;
                                     MEGAHIT v. 1.0.3
            Sequencing Technology :: 454; Illumina
            ##Assembly-Data-END##
FEATURES             Location/Qualifiers
     source          1..3518
                     /organism="Crucivirus-286"
                     /mol_type="genomic DNA"
                     /isolate="000653_ADNa22"
                     /isolation_source="Water"
                     /country="France"
                     /collection_date=2013
     CDS             1..1095
                     /codon_start=1
                     /product="Putative Replication Associated Protein"
                     /translation="MTEQTNNCTGYDLTIYETVKGKTYTYLDMGDMLKQVASKFVFQL
                     EEGEEKKRHFQIRMHAMKKTRASPLLKLVFKCLNITDAKAFIFCKPTSKDVHNKKNFN
                     YVMKAEGRIAGPWTDEFFKEHVNYNIIDEFIDFKEENLRPFQKSILDIINTKDMRKID
                     IIYDPDGNRGKGFIADYLEIFADCYILPNIDDYKLIMQDAHNAVMRRITRLGIANKNI
                     KAFILDMPRAMELREMKKLICAIESVKGGRLQDYRNRSTEPIRINKPRVILFTNEPID
                     MTLYSKDRYAIWTIDDKDALIPFTFPKVDKAYYEYEPAKPTTRILYDNAGTATKVCKK
                     RDEDDDDDDDTPPPVIPKKLIKNKKLVYLE*"
     CDS             1187..2614
                     /codon_start=1
                     /product="Putative CruV Capsid Protein"
                     /translation="MPRRPATAAARPRRAYKAQMKTARRTNRVNRVIAQPERQARRRA
                     AGNAFLSGVGRTIAPLAGSVINALGKRAMTMISGQGDYDISGITHNSLVGKISPTVPV
                     FSSNTAGMIRVQHREYLTDILSTTDFNNLEFPINPALASTFPWLSKIAANFEQYQIHG
                     LIFEFKSGSSDALNSTNTALGYVVMATEYNSLAPGYQNKLEMENALFACSTKPSLSTI
                     HAVECAPTQSPLTVLYTRTGFENIGESDIRFYDLGKFNIATVGMQAIGANIGELFVSY
                     DISLSKTRYIPPGLTIPTYSVQFGDVDNARALLGSNVSQVIKINTLNVNLAQTFNGAT
                     NICSSIFTLPPGSAGSYIFNYQLVGDLSTNAGGVSGTFPFQSNPFNSLVNITERNIYP
                     SDEAKLGFNHDVRPYNANVVVSAQLPTSVSQFLTYSFTIVNNNLPASFTFSITNAALL
                     QLVKTLGVSPFTLGVCYITQVNSQL*"
ORIGIN
        1 atgactgaac agacaaacaa ctgcaccggc tacgacttaa ctatttacga gacagtaaaa
       61 ggtaagacat acacatattt agacatgggc gacatgttaa agcaggtggc cagtaagttt
      121 gtttttcaac ttgaagaggg cgaagaaaaa aagcgccatt ttcaaattag aatgcacgcg
      181 atgaagaaga cacgggcgag ccctttatta aaacttgttt ttaaatgttt aaatataaca
      241 gatgctaaag cttttatatt ttgtaagccg acatcaaaag atgtacataa taaaaaaaat
      301 tttaattatg taatgaaggc agagggcaga atcgccggac catggactga tgaatttttt
      361 aaagaacatg ttaattataa tattattgat gaatttatag attttaaaga agagaattta
      421 aggccgtttc aaaaatctat attagatata attaacacaa aagacatgcg gaagatagac
      481 attatctatg accccgacgg aaaccgcggg aaaggcttta ttgcggatta tttagagatt
      541 ttcgcagatt gttatatatt acctaatatt gacgattaca aattaattat gcaggatgca
      601 cacaacgcag ttatgcggcg cattacacga ttaggcatag cgaataagaa tataaaagct
      661 tttatattag atatgccgcg cgccatggaa cttagagaaa tgaaaaagct tatatgcgct
      721 atagaaagtg taaaaggtgg acgtttgcag gattacagaa accgcagcac ggagcccata
      781 aggataaata agccacgtgt tattttattt actaatgaac caatagacat gactttatat
      841 agtaaagacc gttacgcgat atggactatt gatgataaag acgctttaat accttttact
      901 tttccgaaag ttgataaagc ttattatgaa tatgaaccgg ccaaacctac gacacgtata
      961 ttatatgata atgcaggcac cgccacgaag gtttgtaaaa aaagggatga agacgacgac
     1021 gacgacgacg acacaccccc gcccgttata cccaaaaaat taattaaaaa taaaaaactt
     1081 gtttatttag aataatttat aaaaaattta aaaatatata gaaaaaaatt tatttattaa
     1141 tatttaattt aatttaattt aaatattatc ttttccggat atataaatgc ccagacgacc
     1201 agcgacagca gcagcacgac cacgaagagc ctataaggcg cagatgaaga cagcacgacg
     1261 cacaaaccgt gtaaatagag tgatagcaca accagaacga caggcccgca gacgcgcagc
     1321 cggtaatgcc tttttatcag gcgtaggacg aactatagcc cctttggcgg gttccgttat
     1381 taacgcatta ggtaagcgag cgatgactat gattagcggg cagggcgact atgatatatc
     1441 agggattaca cataacagtt tagtaggtaa aataagccct acagtccccg tgttttccag
     1501 taatacagcg ggcatgatta gagtacagca ccgcgaatat ctcacggata ttttaagcac
     1561 aactgatttt aataatttag agtttcctat taatccggcg cttgcttcta cttttccatg
     1621 gttaagtaag attgccgcga attttgaaca gtaccagata cacggcttaa tttttgaatt
     1681 taaaagcggt tcgtcagatg ctttgaacag cacaaatacc gcacttggtt atgtagtgat
     1741 ggctacagag tataacagct tggccccagg atatcaaaat aagcttgaaa tggagaacgc
     1801 actttttgca tgttctacta aacccagttt aagcactatt cacgcggtag aatgcgcacc
     1861 cacgcagagc ccattgacag tattatatac ccgtacaggt tttgaaaata ttggcgaaag
     1921 tgatataaga ttttacgact taggaaagtt taatattgct acagtaggta tgcaggcgat
     1981 aggcgcgaac ataggggagc tttttgtcag ttatgatata agcctctcaa aaacacgcta
     2041 tataccccca ggtcttacta tacccacata tagtgtccag tttggagatg tagataacgc
     2101 gcgcgcgctt ttaggttcta atgtaagcca ggttattaaa attaatactt taaatgttaa
     2161 tcttgcacaa accttcaacg gcgccacgaa tatatgtagt tcaatattta cacttccgcc
     2221 agggtcggca ggttcatata tttttaatta tcaattagtc ggagatttaa gcactaacgc
     2281 cggcggagtg tcagggactt ttccttttca gtctaacccg tttaatagtt tagttaatat
     2341 tacagagcgt aatatatacc cgtcagacga ggctaaactt ggctttaatc atgatgtacg
     2401 cccttataat gctaatgttg tagtatcagc acaattaccc acgagtgtaa gccagttttt
     2461 aacatattca tttactattg taaataataa tttgcccgcg tcttttactt tttctataac
     2521 aaatgccgca ttattacaac ttgttaaaac tttaggagta tcacctttta ctttaggagt
     2581 atgttatatt acccaggtaa attcacaatt ataaataata atacgactta gatataaagc
     2641 agttataata aaccttttat atttaagcat tttaatatag tttatctgtt gtaaatgttt
     2701 atgtgtgcgt gctttaaaaa taaagccttt taataaaata gttccgtaaa atatatttaa
     2761 tagttccgta tagttccatt aaataaacct aattttaaca taatttttta ttaccatata
     2821 caccttttta taaatagttc cgtatgttcc atataccgga tatattatat atatagtatt
     2881 aacaacaata acttaaagta tgaatatgtg taacctatga atatatgtat cagatacttt
     2941 acgtatcgcc tttaagttat tgtttgacga tgcccccaac gacgaacaat atattatatc
     3001 cttacgcgaa gcggttttat atggcgcttg ccgttacccg gccgccctat aaaattataa
     3061 gttttcacgc tttgcgtgtt cctatatccg cccccccgcg gggggcgcta tgatgttatt
     3121 taacataaaa ctataaaaag gatataatga tattacttat aaaaaattta cgcgaagcgc
     3181 cctgggcttg gccggggtat taggcccccg cgggatacgt ccataaatta atagtatacc
     3241 cgacatttaa aaaaatttat ttttttaatg aagggtataa cgtaattaat ttacagaggc
     3301 cgatattttt ttttaagtca tatacgaagc atttatatat atcttttcaa tttaggctat
     3361 tttaatattt tcaaaaactt tttaaatata tagaaaaaaa atagttccga aaaaaattta
     3421 aaatatatag aaaaataaaa agttccgatt tatttatttt aaatttaatt taagcgccta
     3481 aatataaatt tattatctat tattataata tataaaag
//
LOCUS       CruV-287                3521 bp    DNA     circular     24-FEB-2020
DEFINITION  Crucivirus-287 isolate 120_435_IR1_3296.
ACCESSION   CruV-287
KEYWORDS    .
SOURCE      Crucivirus-287
  ORGANISM  Crucivirus-287
            Unclassified.
REFERENCE   1  (bases 1 to 3521)
  AUTHORS   de la Higuera,I., Kasun,G.W., Torrance,E.L., Pratt,A.A.,
            Maluenda,A., Colombet,J., Bisseux,M., Ravet,V., Dayaram,A.,
            Stainton,D., Kraberger,S., Zawar-Reza,P., Goldstien,S., Briskie,J.,
            White,R., Taylor,H., Gomez,C., Ainley,D.G., Harding,J.S.,
            Fontenele,R.S., Schreck,J., Ribeiro,S.G., Oswald,S.A., Arnold,J.M.,
            Varsani,A., Enault,F. and Stedman,K.M.
  TITLE     Direct Submission
  JOURNAL   Submitted (24-FEB-2020) Biology, Portland State University, 1719 SW
            10th Ave, Portland, Oregon 97201, USA
COMMENT     Bankit Comment: ALT EMAIL:ide@pdx.edu
            Bankit Comment: TOTAL # OF SEQS:451
            Bankit Comment: GAP: est. length - alignment

            ##Assembly-Data-START##
            Assembly Method       :: MetaSPAdes v. 3.12; IDBA-UD v. 1.1.1;
                                     MEGAHIT v. 1.0.3
            Sequencing Technology :: 454; Illumina
            ##Assembly-Data-END##
FEATURES             Location/Qualifiers
     source          1..3521
                     /organism="Crucivirus-287"
                     /mol_type="genomic DNA"
                     /isolate="120_435_IR1_3296"
                     /isolation_source="Water"
                     /country="Norway"
                     /collection_date=2011
     misc_feature    join(3512..3521,1..18)
                     /note="stem-loop"
     misc_feature    1..9
                     /note="nonanucleotide"
     CDS             63..1136
                     /codon_start=1
                     /product="Putative Replication Associated Protein"
                     /translation="MAAKKIYRPSGAGFYEVTNDEPDLLTPVEDKSKQAYVYDFTYFI
                     NPTLQSACETDQDAKIDFADIEEYLKTFCKAYCFQLEETPTTKRWHLQGRFKTQKITR
                     IQTLVNKFQKDSCPFYPCHLSYTSRLNHQAPAFYRYPMKGTTRVPGYEPIADMVFEDE
                     NPQQLDILEDVHELNAWQKYVYEERITKYRDKDMRRKITWIHCSHGNTGKTTFKSWLR
                     FNKHAQIIPNCENYQMIMQCVLQLETRPCYVIDIPRAAQYNKSTMRGIYSAIETIKDG
                     YAYDTRFRLREKHMKSQPMVVVMSNFLPDPTALSIDRWHIISLVKKDKEVVYKDITKS
                     FLIGIEEKKSEKSPKRRIKVIED*"
     CDS             1709..3397
                     /codon_start=1
                     /product="Putative CruV Capsid Protein"
                     /translation="MISRKYSQAEKAAYWKAKALGAAKGGRSRSYEPGVVRQYSGRRP
                     GGRKGKDFSLHWDNSWLGSGGLLYSKESQVPKGMSRSIAPSSMSTMVKTSTPAIKAGV
                     NRSFVIENRELLSNVTWNGTPDFQVTQYSINPGLSDILPYAANIAKSYTRYVMEFCLE
                     YAPSVPSTTSGTISLAYDRNSQASPPTSIQDLMSYEGSTVGSVWAGTNFPANAARVRP
                     SEKKLYVRFGTLTAGEDINLYDMCKVFVCLNGIAGDSVVNNTSVGNLYINYKITYMNQ
                     KLTQIVNSNIFSVTPALTTTSFSPLLVNTIIPENEAFDYTYQFGQGNIQFLNQNQSLD
                     SAPRIVFPTQGIYRLRFYMYPVVRSGTLAGSVYTTDVSYIFAPIVTHGSQVIVADAFE
                     TYYRQENAVLAPSIGTQAYPIQEHILEVLTSGSYISQGGVVGEGWISLDPETEVVATL
                     MAHSNIGTGVIANILSWLEVDQISGDEATFYDSIVFPPPSTMSDVYRTVAHHSTHRAI
                     TGGNGHRRIQRKMIPISSSSMQPESISETTPEDEAIVEIDREMEEFRKWKKSQQ*"
ORIGIN
        1 taaaacaaaa taacaaaaaa gtgattagta aataaatttt aaatcagtta aaattaacaa
       61 aaatggcagc gaaaaaaatc tatagaccat caggggcggg attttatgaa gttacaaacg
      121 acgaaccgga tttgcttact cccgtagagg ataagtccaa acaggcgtat gtttatgatt
      181 ttacatattt catcaaccca accttgcaaa gtgcctgtga aacggatcaa gatgctaaga
      241 ttgattttgc agatatagaa gaatatctca aaacattttg caaggcttac tgttttcagt
      301 tagaagagac accgacaacg aaaaggtggc atttacaagg gagatttaag acacagaaaa
      361 tcacccgtat acaaacttta gttaataagt ttcaaaagga tagttgtccg ttttacccgt
      421 gtcatctatc ctatacatca agattaaatc accaagctcc tgcgttttat agatatccga
      481 tgaaaggaac aacaagagtt ccaggatatg aacctattgc agatatggta tttgaagacg
      541 agaatccaca acagttggat atactcgagg atgttcacga acttaacgct tggcagaaat
      601 acgtttatga agagcgtata acgaagtaca gagataagga tatgagaaga aagatcacat
      661 ggatccattg ttctcatggg aatacaggaa aaacaacttt taaatcctgg ttaaggttta
      721 ataaacatgc ccagatcata ccgaattgtg aaaattacca aatgataatg cagtgtgttt
      781 tacagttaga aacgagacct tgttatgtta ttgatatacc tcgtgctgct cagtataata
      841 aatccactat gcggggtatt tattctgcca ttgagactat aaaggatggt tatgcttatg
      901 atacaaggtt ccgtttaaga gaaaaacata tgaagtcaca gcctatggta gtggtaatgt
      961 ctaattttct gccggatcct acggcattat cgattgatcg ttggcatatt atttcactag
     1021 tgaagaagga caaagaagtt gtttataagg atattacgaa aagtttcctt atcggtatag
     1081 aagagaagaa gtctgaaaaa tctccaaaaa ggagaattaa ggtgattgaa gattaaatta
     1141 tataaatttt atataatgat cattttgtga caagagcgcg agccagcgag cgcaccctaa
     1201 aatgccggtc gaataaaaat cagaaatgat gttttatgac tatatatatg actcggaaat
     1261 tttattctga ctaacaattt ccgttagtgt gttaccttcc gttaataaat aaataagttt
     1321 tatttaaaga caccaaggca gtctcagaca tagcttttag gatattatta caggtaaacg
     1381 aaggttaccg tcctaaaagc tatgcgtgga acagtagagc gccttttgag ccccccgctt
     1441 tgtcgggttt caggcgaggc gcgattttgc cgctaatttt tccgcaatgg atcggaaaag
     1501 cggcccaaaa aaatatttaa agataatttt tttacctttt taaagtttaa aaagttaatt
     1561 ttctcacctg aaaattaact ctgaccaata tttgcaagaa tatttagctg ataaagcatc
     1621 ttgggagtct gtctggagtc aagaccctga gtattacggt ggtgtcgacg cggtttatcc
     1681 tgattttgat ttgtggttgc gaagaggaat gatttctcgt aaatattctc aagcagaaaa
     1741 ggcagcctat tggaaggcta aagcacttgg tgcagcgaag ggtggtcggt cacgatcgta
     1801 tgaacccgga gtggtgcgtc aatattctgg acgcagacct ggtggaagaa aggggaaaga
     1861 tttttccctc cactgggata actcatggtt gggttctggc ggtttgttat attcaaagga
     1921 aagccaggtt ccaaagggaa tgtcccgctc cattgctcct tcatccatgt caactatggt
     1981 gaaaacatca actcccgcaa tcaaagccgg tgtgaacagg tcttttgtta ttgagaatcg
     2041 agagttgtta tctaatgtta cttggaacgg tactccggat ttccaagtga ctcaatattc
     2101 aattaatcct ggtttgagtg atattctgcc ctacgcagct aatattgcga aatcctatac
     2161 tcgctacgtg atggagtttt gtcttgaata tgctccatca gttccgtcga ctacttcagg
     2221 aacaatttcg cttgcatacg atcgaaattc acaagcatct cctcccacat caattcaaga
     2281 tttgatgtct tacgaaggtt ctactgtcgg gtcagtttgg gcaggaacta atttcccagc
     2341 taatgctgcc cgagtccgtc cttctgagaa gaagttatac gttcgattcg gaacgttgac
     2401 ggcaggagaa gatattaact tatacgatat gtgtaaggtt tttgtctgtt tgaatggtat
     2461 tgccggtgat tccgttgtca ataacacttc tgttggtaat ctgtatatta attacaagat
     2521 cacctatatg aatcagaagt tgactcagat tgtcaattca aacatttttt cagttacccc
     2581 tgcgttaact actacttcat tttcgccatt gttggttaac acaattattc cagaaaatga
     2641 ggcctttgat tatacttatc agtttggaca ggggaatatt caatttttga atcagaatca
     2701 gtctcttgat tctgccccca ggattgtttt cccgacacag ggtatatatc gattacgatt
     2761 ttacatgtac cctgtggtta ggagtggaac ccttgctggg tccgtttata ctactgatgt
     2821 cagttatatt tttgcaccga tagtaactca tggttctcag gttattgttg ccgatgcatt
     2881 tgagacatat tatcgtcaag aaaatgcagt tcttgctccc agtattggga cacaggctta
     2941 tcctattcaa gagcacatcc ttgaggtact gacatctggc agttatattt ctcaaggtgg
     3001 cgttgttgga gagggctgga tttcacttga tccagagaca gaagttgttg cgactttgat
     3061 ggcccattcc aatattggaa ctggagttat tgctaacatt ctgtcttggt tggaagtcga
     3121 tcagatcagt ggtgatgaag ccacttttta tgattctatc gtatttcctc caccctcgac
     3181 aatgtcagat gtttatcgta ctgtcgcgca tcacagtacc caccgagcga ttaccggtgg
     3241 gaatggtcac agacgtattc agagaaagat gattcctata tcatctagtt ccatgcaacc
     3301 tgagtccatt tctgagacta ccccagaaga tgaggccatc gttgagattg atagggaaat
     3361 ggaggagttc cgtaaatgga agaaatctca acaataggga gatcgagact cccgaaccga
     3421 catacggagt tcaaataaca gtataatatt ataatattat agcgaaataa cttttaaaat
     3481 aaattatttt aaaaatggtt taaagacaca attttgttat c
//
LOCUS       CruV-288                3526 bp    DNA     circular     24-FEB-2020
DEFINITION  Crucivirus-288 isolate 001443_virusbaton.
ACCESSION   CruV-288
KEYWORDS    .
SOURCE      Crucivirus-288
  ORGANISM  Crucivirus-288
            Unclassified.
REFERENCE   1  (bases 1 to 3526)
  AUTHORS   de la Higuera,I., Kasun,G.W., Torrance,E.L., Pratt,A.A.,
            Maluenda,A., Colombet,J., Bisseux,M., Ravet,V., Dayaram,A.,
            Stainton,D., Kraberger,S., Zawar-Reza,P., Goldstien,S., Briskie,J.,
            White,R., Taylor,H., Gomez,C., Ainley,D.G., Harding,J.S.,
            Fontenele,R.S., Schreck,J., Ribeiro,S.G., Oswald,S.A., Arnold,J.M.,
            Varsani,A., Enault,F. and Stedman,K.M.
  TITLE     Direct Submission
  JOURNAL   Submitted (24-FEB-2020) Biology, Portland State University, 1719 SW
            10th Ave, Portland, Oregon 97201, USA
COMMENT     Bankit Comment: ALT EMAIL:ide@pdx.edu
            Bankit Comment: TOTAL # OF SEQS:451
            Bankit Comment: GAP: est. length - alignment

            ##Assembly-Data-START##
            Assembly Method       :: MetaSPAdes v. 3.12; IDBA-UD v. 1.1.1;
                                     MEGAHIT v. 1.0.3
            Sequencing Technology :: 454; Illumina
            ##Assembly-Data-END##
FEATURES             Location/Qualifiers
     source          1..3526
                     /organism="Crucivirus-288"
                     /mol_type="genomic DNA"
                     /isolate="001443_virusbaton"
                     /isolation_source="Water"
                     /country="France"
                     /collection_date=2013
     CDS             join(complement(1..10),complement(3165..3526))
                     /codon_start=1
                     /product="putative ORF"
                     /translation="MSSLLYNIIKQIFKIINPFGVGPIFLGPHLVPRRVPASIPRLFG
                     LKSYGRRNTFLLFLDSPKARHFAQTIYYNPLPGGNNWLAAKLALRVGVRSFGSPPAPC
                     LPMVSFVFLVYLLAIPSRGTC*"
     CDS             1..1128
                     /codon_start=1
                     /product="Putative Replication Associated Protein"
                     /translation="MTTKVNLKPDVPFVNGKYCVFTAWQVAHLKFNDETMQYLIYQRE
                     CCPDTKRLHWQGYVEFKANTRRKAAQLQLGIIDADTIDSDGKLLAGYWRAFNRRGTGS
                     EAAAYCEKRDSAVNEYVYEWGSMEKKQPGQRTDLEKPAEALRKGASLRDLAREFTPAM
                     IKYSRGFERVYQLLKPEIKAPDPCIELRFWQRELIKRIDKGFKKRQILWVWSEASATG
                     KSTMYDYMCFKYGMDKVLPGAWTKTDLLHAYSQHTVVSFNVPRNQPMTKAMIDVLESI
                     SDGGVQMSIKYDSCAKLINAVVIVFANIPPPNVDMPDRCIDICVDSDEVQQDRILKGL
                     PVPSKTLASVKAEERVLKFQQFMESERELMDSDIVKLDEYS*"
     CDS             1493..3109
                     /codon_start=1
                     /product="Putative CruV Capsid Protein"
                     /translation="MYMLSKSDYAKLSKAGKAKHKASLLKSKVRKPRSLAPKRKKPAA
                     RKSSGIASSIGEAVGGFFGPAGARLGKTAGSMFAKLSGYGDYKVESNSISMGTDPPMF
                     ASSGRGTLVRHREFIQDIVGSTSFNIQGFGINAGLAGTFPWLSQLAVNFEQYVVRGMV
                     FEFKTTSATSLASGTNTAMGTVIMATEYNSSATNPASGGALFSSKAQMENHEFCTSSI
                     PSQSFIHAIECARGETPVSCLYVRPGTVPSGTDQRLYDLGTFEIATVGMQAANIVGEL
                     WCTYDIELLKPVLYDAVGLGILTDHYQLPVATVSTASPFGTTYPVAKVASSSLGSTIT
                     GTTIINLPYSSVYQSYFLVYAVKGASTVLTTAIPQPTYSTGVSAPSLFYLDALSRLSN
                     PNGVTSTVQWYVTFVDVVGGTVSPTITWGTTATMPTSIDGADLFIQQANPGLLTRYRK
                     LSPAKIVAAIVEADSDDDVDADYEKMIREKKRAEIVKWKAESESKEEKKPKADDDDED
                     EYAAFKRVRAKVLSVAEPESPHPGKSASKK*"
     misc_feature    2734..2756
                     /note="stem-loop"
     misc_feature    2740..2748
                     /note="nonanucleotide"
ORIGIN
        1 atgacgacaa aagttaattt gaagccagac gtgccgtttg ttaacgggaa gtattgtgtt
       61 tttactgctt ggcaagtggc tcatttgaag tttaatgatg aaactatgca gtatttaatt
      121 tatcagcgag agtgttgtcc agatactaag cgtttgcatt ggcaaggtta tgttgaattt
      181 aaagcaaata cccgccgtaa ggcggcacaa ctacagttgg gtattattga tgcagatact
      241 atagattctg atggaaagtt gctcgcaggg tactggcggg catttaatcg tagaggaact
      301 ggttctgaag ctgcagcata ttgtgaaaag cgtgatagcg ctgttaatga atatgtttat
      361 gaatggggtt ctatggagaa gaagcagcct gggcagcgaa ctgatctaga aaaacctgct
      421 gaagcattgc gtaaaggagc atcgctccgt gatttagcta gggagtttac tcctgctatg
      481 attaagtatt ctcgaggttt cgagagggtt tatcaattat tgaagcctga aatcaaagcc
      541 ccagatcctt gtatcgaact tcggttttgg cagcgtgagc tgataaagag aatcgataaa
      601 ggatttaaga aacgtcagat tctttgggtt tggtcagaag cttcagctac tggtaaatct
      661 actatgtatg actatatgtg tttcaaatat ggaatggata aggttttacc tggtgcttgg
      721 actaagactg atctgttgca tgcgtattcg cagcatacag ttgtttcgtt taatgttcca
      781 aggaatcagc caatgacgaa agcgatgata gatgttctag aatctatttc ggatggcggt
      841 gttcaaatgt caataaagta tgattcttgc gctaagctaa tcaatgctgt tgttattgtg
      901 tttgctaata ttccacctcc taatgtagat atgcctgatc gttgtatcga tatttgtgtt
      961 gattctgatg aagttcaaca agatcgtatt ttgaaaggtt tacctgttcc ttcaaaaact
     1021 ttagcttctg tcaaagctga agagcgtgtt ttaaagtttc aacaatttat ggaatccgag
     1081 agggaactca tggattctga tattgttaaa cttgatgaat attcttgata aaaaagttgc
     1141 cgtaaggcta tattttttga attcttaagg tgggggcaag cgttatatgc gcttattatt
     1201 accccccacc tccataacat aacgatggag atgacgaagg aatcgactga gagatggcta
     1261 tgcaccttag ggtgcaggcc aatgtatgga gtgttatatc aaatctattg tgattttgat
     1321 aggtcttttg ggtatgaaat atctaaaaaa gactacggca tccggagacc tttaggtcgg
     1381 agaggtgagg aatcgacgcc gggacccgcc gaggaacgag gtggggacca aggaagatgg
     1441 gatgtccccc gtagggggac taattatttt aatttagact tttatagatg gcatgtatat
     1501 gctgtcaaaa agtgactatg ctaaattatc taaagccggt aaggctaaac ataaggcttc
     1561 tttacttaaa agtaaagtta gaaagcctcg gagcttagca cctaagcgga aaaagccagc
     1621 agcccgaaaa tcttctggga ttgcatcatc tattggtgag gcagttggtg gcttctttgg
     1681 tcctgctgga gctcggcttg gtaaaactgc tggaagtatg tttgccaagt tgtctgggta
     1741 tggagattac aaagtagaat cgaattcgat ttctatgggt acagatcctc ccatgtttgc
     1801 ttccagcgga aggggaactt tagtacgaca tcgcgaattt attcaagata ttgttggttc
     1861 tacttctttt aatatccaag gatttgggat taatgcagga ttggcaggaa catttccgtg
     1921 gttatctcaa ctagctgtga attttgaaca atatgttgta cgaggaatgg tatttgaatt
     1981 taagaccaca agtgctacgt cacttgcgtc gggtacaaat acagccatgg gtactgtgat
     2041 tatggctaca gaatataaca gttctgctac aaatcctgca tcaggtggtg cgttgttttc
     2101 aagtaaagca caaatggaaa atcacgagtt ttgtacgagt agtattcctt ctcaatcgtt
     2161 tattcatgct atcgaatgtg caaggggtga aacgccagtt tcatgtttgt atgttcgtcc
     2221 gggaactgtt ccttctggaa ctgatcagcg attgtatgat ttgggaacat ttgagattgc
     2281 gaccgtaggt atgcaagctg caaatattgt tggcgaattg tggtgtacgt atgatattga
     2341 actgcttaag ccagttttgt atgatgcagt tggtttgggt attttgactg atcattatca
     2401 gctacccgta gctactgttt cgactgcatc accttttgga acgacatatc ctgtcgcaaa
     2461 agttgcatcg tcaagtcttg gtagtactat tacaggaact actattataa atttgcctta
     2521 tagcagtgtt tatcaatctt acttcttagt gtatgcggta aaaggtgcgt caactgtatt
     2581 gacaactgct ataccacagc ctacttattc tacaggagtt tcagctcctt ctttgttcta
     2641 tttggatgct ttgtctcgtt tgtcaaatcc aaatggtgtt acatctacag ttcaatggta
     2701 tgttacgttt gttgatgttg ttggtggaac tgtttctcca actattacat ggggaactac
     2761 tgcaactatg ccaacttcta ttgatggtgc tgatttattt attcagcaag caaatcctgg
     2821 attgctaact cgatatcgaa agctgtctcc agctaagatt gttgctgcta tcgtagaggc
     2881 tgattctgat gatgatgttg atgctgatta cgagaagatg attcgagaaa agaagcgtgc
     2941 cgaaattgtt aaatggaagg ctgagtctga atctaaagag gaaaagaagc caaaggctga
     3001 tgacgatgat gaagacgaat atgctgcttt caagcgcgtt cgtgcaaaag ttttgtctgt
     3061 tgcagagcct gaatcacctc atcctggtaa gtctgcttca aaaaagtaag atttattaag
     3121 attttttttt aagatattag gaacgtgcgc gatgcccgaa gggctcagca cgttccgcgt
     3181 gaaggtattg ctaaaaggta tactagaaat acgaaagata ccattggcaa gcaaggagcg
     3241 ggtggagatc cgaaggatcg aaccccgacg cgcagagcga gcttggcagc gagccagtta
     3301 ttgccccccg ggaggggatt atagtatatt gtttgcgcaa aatgacgagc cttaggcgag
     3361 tccaggaata gcaaaaaagt attacggcgc ccgtaggact ttagtccgaa gaggcgagga
     3421 atcgacgccg ggacccgccg aggaacgagg tggggaccaa ggaagatggg tccaacccca
     3481 aaggggttaa ttattttaaa gatttgtttg atgatgttat ataaca
//
LOCUS       CruV-289                3536 bp    DNA     circular     24-FEB-2020
DEFINITION  Crucivirus-289 isolate 001430_virusbaton.
ACCESSION   CruV-289
KEYWORDS    .
SOURCE      Crucivirus-289
  ORGANISM  Crucivirus-289
            Unclassified.
REFERENCE   1  (bases 1 to 3536)
  AUTHORS   de la Higuera,I., Kasun,G.W., Torrance,E.L., Pratt,A.A.,
            Maluenda,A., Colombet,J., Bisseux,M., Ravet,V., Dayaram,A.,
            Stainton,D., Kraberger,S., Zawar-Reza,P., Goldstien,S., Briskie,J.,
            White,R., Taylor,H., Gomez,C., Ainley,D.G., Harding,J.S.,
            Fontenele,R.S., Schreck,J., Ribeiro,S.G., Oswald,S.A., Arnold,J.M.,
            Varsani,A., Enault,F. and Stedman,K.M.
  TITLE     Direct Submission
  JOURNAL   Submitted (24-FEB-2020) Biology, Portland State University, 1719 SW
            10th Ave, Portland, Oregon 97201, USA
COMMENT     Bankit Comment: ALT EMAIL:ide@pdx.edu
            Bankit Comment: TOTAL # OF SEQS:451
            Bankit Comment: GAP: est. length - alignment

            ##Assembly-Data-START##
            Assembly Method       :: MetaSPAdes v. 3.12; IDBA-UD v. 1.1.1;
                                     MEGAHIT v. 1.0.3
            Sequencing Technology :: 454; Illumina
            ##Assembly-Data-END##
FEATURES             Location/Qualifiers
     source          1..3536
                     /organism="Crucivirus-289"
                     /mol_type="genomic DNA"
                     /isolate="001430_virusbaton"
                     /isolation_source="Water"
                     /country="France"
                     /collection_date=2013
     CDS             1..858
                     /codon_start=1
                     /product="Putative Replication Associated Protein"
                     /translation="MSIDNARARNWCFTLNNYTEEEFNESEELGARSSYIVVGKEIGT
                     NSTPHLQGYVEYTNAVRGSAIRKLFNGRAHWEVRKGTAKQASEYCKKEEKFFEMGSLS
                     AQGKRTDLEEIGAAVLSGASTKEIACTFPGTYIKYHKGIQQLQFASKGLFDFEHRTTK
                     PTVIWRWGATGVGKTRTAREAHTSFYIKDGTPWWDGYEQQQAIIIDDFDGKWPYRDLL
                     RLLDFGPYQGQFKGGYVKINSPYIYITCEYPPETAFRNIEGDVSQVIRRIDKVIHVEA
                     PPPTESKTA*"
     CDS             1215..1601
                     /codon_start=1
                     /product="putative ORF"
                     /translation="MCQPYLPHPQPLKSVLAYFFMGGNSPDMSLFVQCRLVCTGIYYT
                     CRYSLMAYCCNDAYMIPRDLMSIVIDYLYPPWECVNVLNELHREMRNWRANYYFGEEN
                     YFALYFFWSKEDYDYDPADIFRDIDA*"
     CDS             1453..1866
                     /codon_start=1
                     /product="putative ORF"
                     /translation="MSLMNCIEKCAIGARTIISVKKTTSRYTSFGLKKTMTMILRIYF
                     AILMRSCKGHGTGREALWYAHELWPSGALVGALGVCPLASLAKAIWVMSRTTLRYVLL
                     VLNRPFFDQKIYPSGLLCSPNLEIKILDSNLCSDS*"
     CDS             complement(1849..3357)
                     /codon_start=1
                     /product="Putative CruV Capsid Protein"
                     /translation="MPRATVTVRKPAKKKMVTVTRARAPRLKVPRPVGTASYKTKSGS
                     KSGLFGSAGGTVGGMIGTALGGPAGGMLGSTLGRLGGAALSSIFGHGDYEATNAPAIK
                     NNNIVLSNSAQAPQFGSGKVACKFVHREYLGDVFSSSSANTFKIDSYAINPGNSDTFP
                     WLSGVVGAKFQQYRINGMAFEFRSMSSDALNSTNTALGSVIMSTDYDSADATFASKQQ
                     MENTEYGVSCKPSSNMMHGIECERFQTPISELFIRAFDVPSGKDIRLYDLGRFSIASV
                     GCQGTSVNLGELWVAYDIDCFKAIEQPPGYLIPFSQYALVGTLPASAPLGTSQSQLLT
                     QAANEINMTFTANKMSWPSSIDVGSVWHVQINWLRGSSALTAPTITSGNGMFINSNTI
                     WKTTTGTTCTDIRVDIDVQYQGGASPGALPYLNIANFTHAGANVQSDLYISQMSGLWP
                     GTYTATTAISIQEQHDEDYEEETDSPEYVAVHEDEEKKTSVSRQMSRLRIRT*"
ORIGIN
        1 atgagtatcg acaatgcacg tgcgcgtaac tggtgcttca cacttaacaa ctacacggaa
       61 gaagaattca atgagagtga agagctcgga gctcgttcat cttacattgt tgtaggcaag
      121 gagattggca ctaacagcac accacacttg caaggttatg ttgaatatac gaacgcggtt
      181 cgaggatctg caattcgtaa attattcaat ggacgtgctc actgggaagt ccgcaaaggg
      241 actgccaagc aagcgtccga atattgtaag aaagaggaaa aattctttga aatgggttct
      301 ctttctgctc aaggtaagcg cactgaccta gaggaaatag gcgctgcggt tctctcagga
      361 gcaagcacca aagaaatagc atgcacgttc ccaggaacat acattaaata ccataaaggc
      421 atccagcaac tgcaatttgc atcgaaaggt ttgttcgatt ttgaacaccg cacaactaaa
      481 cctacggtga tttggcgatg gggagcgaca ggtgtcggta aaacacgcac tgcgcgcgag
      541 gctcatacga gtttttacat caaggacggg acgccctggt gggacggtta cgaacaacaa
      601 caggcaatta tcatcgatga tttcgacggt aaatggccct atcgtgacct cttacgtcta
      661 cttgattttg gaccttacca aggacagttt aaaggcgggt atgtcaagat aaacagtcca
      721 tacatataca tcacatgtga gtatccacct gaaacagcct ttcgaaacat cgagggcgat
      781 gtgtcacagg tcattcgtag aattgataaa gtcattcatg tagaagcacc acctcctacg
      841 gagtctaaaa cagcataaac aagcgcttct acgaagacgt cagactatcc tgaaaggagt
      901 atctgatttg agcctttttt ttgcaattct catgtatggc ttgatcattt tgttcttctg
      961 tgggtaactt tctgccgatt gtataacacc ggcgttatgg gggacaagcc cccataacgt
     1021 catgtggcta cgccacatga gcgtcgctac gctccgcccg gccccacgcc taggcggcgg
     1081 gccggatctg tccagattta cagaaacctt cccttggcac agaagttttt tcacttatgt
     1141 taacgcatat agcttatttg gtcattctgc ccacgctatg cgttttggca cagaagtggt
     1201 agggtaatac taggttgtgc caaccttacc taccacatcc acagcctcta aagtctgttc
     1261 tggcctattt tttcatgggc ggcaattcgc ctgacatgtc attgtttgtt cagtgcagat
     1321 tagtctgcac tggtatatac tatacgtgtc gatactcgct aatggcttac tgttgcaacg
     1381 atgcatacat gatcccaagg gatcttatga gtattgtaat agactactta taccctccgt
     1441 gggagtgtgt aaatgtcctt aatgaattgc atagagaaat gcgcaattgg cgcgcgaact
     1501 attatttcgg tgaagaaaac tacttcgcgt tatacttctt ttggtctaaa gaagactatg
     1561 actatgatcc tgcggatata tttcgcgata ttgatgcgta gttgtaaagg acatgggacg
     1621 ggccgtgagg ccctatggta tgcccatgaa ctgtggccat cgggcgcctt agtcggcgcc
     1681 cttggcgttt gtcctctcgc ttcgctcgca aaggcaatat gggttatgag tcgcactacg
     1741 cttcgctacg tgctccttgt cctaaatcgt cctttttttg atcaaaaaat atacccttcc
     1801 gggcttttat gctctccaaa tttggagatc aagatactgg acagtaactt atgttctgat
     1861 tcgtaaccga ctcatctgac gtgatactga tgtttttttt tcctcatctt catgaaccgc
     1921 tacatactca ggtgaatcgg tttcctcttc atagtcttcg tcatgttgct cttgaatgga
     1981 gattgcggta gttgccgtgt aagttccggg ccatagacca gacatttggg agatgtatag
     2041 atctgattga acattagcgc ccgcgtgcgt aaagttcgcg atattgagat acgggagagc
     2101 tccgggggat gctccacctt gatactgaac gtcaatatct acacgaatgt cggtgcatgt
     2161 agtgccggtt gttgtcttcc aaatagtatt ggaattgatg aacattccgt ttccgctagt
     2221 aatggttggt gcggttaatg cagatgaacc tcgaagccaa ttgatttgaa cgtgccatac
     2281 actaccaaca tcaatagatg acggccagct cattttattt gctgtgaatg tcatgttgat
     2341 ctcatttgca gcttgtgtta gcaattggga ttgtgatgtt cccaatggag ctgatgccgg
     2401 aagagttccg acgagagcat attgagagaa tggaataaga tatccgggcg gttgttcgat
     2461 tgctttgaag cagtcaatgt catatgcaac ccagagctca ccaaggttca cactggttcc
     2521 ttgacatcct acggacgcaa tggaaaatcg accgagatca tacagtctga tatctttgcc
     2581 cgatggaacg tcaaatgccc tgataaacag ttcagaaata ggagtttgaa agcgttcaca
     2641 ttcgatgcca tgcatcatat tgctcgaagg cttgcacgac acaccgtatt cggtgttctc
     2701 catttgttgc ttggatgcga atgtcgcatc agcactgtca taatcagtag acataatgac
     2761 cgagcctagg gcagtgtttg ttgaattgag agcatcagat gacatacttc ggaactcgaa
     2821 tgccatgcca ttgatgcgat attgttgaaa cttagctccg acaactccag acaaccaagg
     2881 aaacgtatcc gaatttccag ggttgattgc gtaactgtca attttgaatg tattagcgct
     2941 cgaagagctg aacacatcgc cgagatactc gcggtggacg aacttgcacg caactttgcc
     3001 actcccaaat tgtggagctt gagctgagtt tgataataca atgttattgt ttttgattgc
     3061 tggagcgttg gtagcctcgt agtctccgtg accgaagata ctcgatagtg ctgcgcctcc
     3121 caatcgtcca agtgttgaac caagcatacc acctgcaggt cctccgagag ctgtcccgat
     3181 cattccgcct acagtgccac cggcacttcc aaacagtccg cttttactcc ctgatttcgt
     3241 cttgtatgat gctgtcccga caggtcgcgg aaccttcagc cgaggtgcac gtgctcgagt
     3301 tactgtaacc atctttttct tcgcgggctt acgaactgta acagttgcac gaggcatgtt
     3361 gttggttgag cgatcggtta ccttcggtag acgagtatac cttagggtcc gatcttgtta
     3421 tgaacatttt attaatgtgc attatttgtt tttcttctaa gctttggcac agaagtatat
     3481 atacgagtag tcagtgacca cacggaatta ttgagactca tttctttggc gggatc
//
LOCUS       CruV-290                3538 bp    DNA     circular     24-FEB-2020
DEFINITION  Crucivirus-290 isolate 001428_virusbaton.
ACCESSION   CruV-290
KEYWORDS    .
SOURCE      Crucivirus-290
  ORGANISM  Crucivirus-290
            Unclassified.
REFERENCE   1  (bases 1 to 3538)
  AUTHORS   de la Higuera,I., Kasun,G.W., Torrance,E.L., Pratt,A.A.,
            Maluenda,A., Colombet,J., Bisseux,M., Ravet,V., Dayaram,A.,
            Stainton,D., Kraberger,S., Zawar-Reza,P., Goldstien,S., Briskie,J.,
            White,R., Taylor,H., Gomez,C., Ainley,D.G., Harding,J.S.,
            Fontenele,R.S., Schreck,J., Ribeiro,S.G., Oswald,S.A., Arnold,J.M.,
            Varsani,A., Enault,F. and Stedman,K.M.
  TITLE     Direct Submission
  JOURNAL   Submitted (24-FEB-2020) Biology, Portland State University, 1719 SW
            10th Ave, Portland, Oregon 97201, USA
COMMENT     Bankit Comment: ALT EMAIL:ide@pdx.edu
            Bankit Comment: TOTAL # OF SEQS:451
            Bankit Comment: GAP: est. length - alignment

            ##Assembly-Data-START##
            Assembly Method       :: MetaSPAdes v. 3.12; IDBA-UD v. 1.1.1;
                                     MEGAHIT v. 1.0.3
            Sequencing Technology :: 454; Illumina
            ##Assembly-Data-END##
FEATURES             Location/Qualifiers
     source          1..3538
                     /organism="Crucivirus-290"
                     /mol_type="genomic DNA"
                     /isolate="001428_virusbaton"
                     /isolation_source="Water"
                     /country="France"
                     /collection_date=2013
     CDS             1..1398
                     /codon_start=1
                     /product="Putative CruV Capsid Protein"
                     /translation="MSGKKTKLNSMASGWTEMNEGERKAYRLGKQNGAKSAAAGAPPK
                     KRANGAKSKAKKPYAKNSKKGPAVFSHAGSFLGGMAGAALVPEGGPISEAIGSFLGGK
                     IGHLADKIIGFGDYKVQSNSIMRGGMSAPQITNSIRNGGVVVRYREYLGDITSTTAFT
                     TDVYYLNPGQRKGFPWLSTFAACWQQFRWRGLIFEFGSTSSDAVLSSATSSALGTVSM
                     ATDYDVLDTTYMTKREMLNTMFANSSKPSCTFIHPIECARKQTPIPIQYVRTGAYPSN
                     ADPRLYDLGKMVIATEGMQADGGAVGELWVTYEVEFFKQQLAATALSDNYALSNGSSA
                     LWLGIVAPTLVDGSTIGGICGTDGTNSYYQFPSWITNGKYLMTFDRQGTTAVLGQITP
                     TVTHGSLFDIWESGITNFVQSPATGTNSPTIMCQLVVVITGASCRVTFGTAAVPTSST
                     GNLVVVQINYDSSAD*"
     CDS             complement(1913..3103)
                     /codon_start=1
                     /product="Putative Replication Associated Protein"
                     /translation="MCPEMDDNRRKFRRFRWTHNNYTAEDIARYSKPDKLHEWIDYIT
                     FSYEIGEKCGTPHLQGYIEFDRMQTIAQACDKMEPGKGKAKTSVPHGVRLMQADANRQ
                     ANIVYVRKGRTKDPIYVDEAGNPLIFEWQKEGAQRTRGAGTGKEAWEAKLEHLKAEPN
                     LLAFAGEYPEEAFKYSSGVEALCKAIGRTMTDDKLTGMYPMNLRLYKWQRAIANIVGR
                     KDVLPEGRKVLWIYDPLGKCGKTVFAAWLMVNHKALYLNNAKSENMAHAWSKDRNDII
                     IIDLMKTTEDFVNYTAMEAGKNGIWFSPKYDSSTQVSIRPWVIVLSNSLPRLDAMAQE
                     RFLTIRIRDNPAGMELEDDPEGKLEKFNDSDRTGEFLGDPILGPMILGDEWHHHPSNP
                     KRQG*"
     misc_feature    2570..2600
                     /note="stem-loop"
     misc_feature    2580..2588
                     /note="nonanucleotide"
ORIGIN
        1 ttgtcgggta aaaaaaccaa gctgaacagc atggcatccg gatggacaga gatgaacgaa
       61 ggagagagga aagcataccg tctcggtaag cagaatggag cgaagtcagc tgcagctggc
      121 gctccaccaa agaagagggc taacggtgcc aagtccaaag ccaaaaagcc ctacgctaag
      181 aactccaaga agggaccagc ggtgttttcg cacgccgggt cgttccttgg aggcatggca
      241 ggtgccgcct tagtaccaga aggcggtcct atctcagagg ctataggctc attccttggt
      301 ggcaagatcg ggcatcttgc cgacaaaatc attggattcg gagactacaa ggtccaatcc
      361 aactccatca tgcgtggtgg catgtccgcc ccgcagatca ctaactccat tagaaatgga
      421 ggagtggtag tcaggtacag agagtaccta ggtgacatta cgtcgacaac ggcgtttacc
      481 accgatgtct attatctcaa tccaggccag aggaaaggct tcccctggtt gtccactttt
      541 gccgcttgtt ggcaacaatt caggtggaga ggtctgatct ttgaatttgg atcaacctcg
      601 tctgacgcag tcctctcgtc agccacctcc tccgcattag gtacagtcag tatggctact
      661 gactatgatg tcctcgacac aacctacatg acaaagagag agatgctgaa cactatgttc
      721 gcaaactcat ccaagccctc ttgcacgttc atccacccca ttgaatgcgc aagaaagcag
      781 actcctattc ctatccagta tgttaggaca ggagcctatc cttctaatgc agaccctcgt
      841 ctgtacgatc ttggaaagat ggttattgca acagaaggca tgcaggccga tggtggtgca
      901 gtaggtgagc tctgggtcac ctatgaagtc gagttcttca aacagcaact cgcagctacc
      961 gctctttcag acaattatgc cttgagcaac ggctctagcg ctctttggtt aggcattgtt
     1021 gctccaaccc tcgttgatgg ttcgaccatc ggaggtatct gtggaactga cggaaccaac
     1081 agttactacc agttcccttc atggattacc aacggtaagt atctcatgac ttttgaccgc
     1141 caagggacaa cagctgtact cggacagatt acacctactg tgacccacgg gtcactcttt
     1201 gatatctggg agtctggtat taccaacttt gtccagtccc cagctacagg cactaactct
     1261 ccgaccatta tgtgtcagtt agtcgttgtt atcacaggag caagctgtcg agtaacgttt
     1321 ggtaccgcag ccgttcccac ttcgtcaact ggtaacttag ttgtagtgca gattaattat
     1381 gattcatctg cagattagaa tagtgttggc caacactata tagtaaccgc gggtttttat
     1441 aagtgaccgg gagaggagga tccctttagg gaggtgcctc ccggctccaa agaccagcgg
     1501 gaattatgcc ttgtttaacc acagcttgtc tgggattgta tcatagtttg catgtctcat
     1561 tgcatacttc cagagaccat cgtcgataca gctcacaagt tcatcgcagt agtgcttaca
     1621 gaaccaagag cgcagctctc tgtaattcca aagagcatct tttagatcga agagtttttc
     1681 ttcggatata gtaagcctgc ccaggctgtc ttgatactca ccaagctcat ctttgatagt
     1741 cttgggtagt gattccctcg gctgcttgag caagtagtcg taggctgatg tgttcatttc
     1801 ttggtcaaga aattaccttt atgttataac tgggggacgt ccggcgcttg cgccggacga
     1861 tggggggcta ttatctctat tacgcccccc cccccaaccc caacacaatg gtttatcctt
     1921 gtcgtttagg gttactagga tggtgatgcc attcatcgcc caatatcatt gggccgagaa
     1981 taggatcacc aaggaattca ccggttcggt cggaatcgtt gaatttctct agcttcccct
     2041 cgggatcgtc ttcgagctcc atgcctgcag ggttgtctcg aatgcgtatc gtgaggaagc
     2101 gttcttgggc catggcgtcg agtctgggca acgagttgct cagtacaatc acccatggcc
     2161 ttattgagac ttgggtactg gagtcgtact tcggagagaa ccagataccg ttcttacccg
     2221 cctccatggc ggtgtaatta acaaagtctt ccgtcgtctt catcaggtcg atgatgatga
     2281 tgtcgttacg gtccttgctc caagcatggg ccatattctc tgacttagca ttgttaaggt
     2341 agagagcttt gtggttgacc attagccatg cggcgaatac ggtctttcca cacttgccca
     2401 acgggtcata gatccataag accttcctcc cctcggggag gacgtctttg cgcccaacga
     2461 tgttagcgat cgctctctgc cacttgtaga gacggaggtt catcgggtac atgccagtaa
     2521 gcttgtcgtc ggtcattgta cgccctattg ctttacatag ggcttcaacc cctgaggagt
     2581 atttaaatgc ctcctcaggg tattctccgg cgaaggccag tagattgggc tccgctttga
     2641 ggtgctccaa tttggcctcc cacgcctctt tgccagtgcc agcaccacgg gtacgctgtg
     2701 caccttcctt ttgccactcg aatatgagag ggttcccggc ttcgtctacg tagatcgggt
     2761 ccttggttct gcccttcctg acatagacaa tgttggcttg tcgattggcg tcggcttgca
     2821 tgaggcgtac gccatgtggg acgctggttt tggctttgcc tttgccaggt tccattttgt
     2881 cgcatgcttg agcgatggtc tgcatgcggt caaactcgat gtagccttgc aggtgaggtg
     2941 tgccacactt ctcgcctatc tcatagctga aggttatgta gtcgatccac tcgtggagct
     3001 tgtctggttt agagtacctg gcgatgtctt cggcagtgta gttgttatgc gtccagcgga
     3061 agcgtcggaa ttttctacgg ttgtcgtcca tttctgggca caggagttac gcttatcaca
     3121 accctgatga tactgttctt gttactgttc ccgttccacg atttttttct gaaaaatctc
     3181 gtcgtagaaa acccatggag tggcgagacg atcggctaga acagaggata gaacagcatg
     3241 tggcatgtgg ccgtgctgag agtgccatgt tggcgtctca cacgatgaga caagaggcct
     3301 acgatgagat cgagctcacc tggcgcgagc tccaagagca caagcagtta cacccccggt
     3361 gtgtggctga tgtagtgagg ctagaggacc gtatcctcac acttaaggca gccgtatggg
     3421 agtatgagga cactctcgat gctgccatgc gcgatagaga tgactattgg gcagcccacc
     3481 gcggcgctcc acaggatgtt gactagatct acttccgcaa aacaaaacgc gcgacgat
//
LOCUS       CruV-291                3538 bp    DNA     circular     24-FEB-2020
DEFINITION  Crucivirus-291 isolate SR3_42523.
ACCESSION   CruV-291
KEYWORDS    .
SOURCE      Crucivirus-291
  ORGANISM  Crucivirus-291
            Unclassified.
REFERENCE   1  (bases 1 to 3538)
  AUTHORS   de la Higuera,I., Kasun,G.W., Torrance,E.L., Pratt,A.A.,
            Maluenda,A., Colombet,J., Bisseux,M., Ravet,V., Dayaram,A.,
            Stainton,D., Kraberger,S., Zawar-Reza,P., Goldstien,S., Briskie,J.,
            White,R., Taylor,H., Gomez,C., Ainley,D.G., Harding,J.S.,
            Fontenele,R.S., Schreck,J., Ribeiro,S.G., Oswald,S.A., Arnold,J.M.,
            Varsani,A., Enault,F. and Stedman,K.M.
  TITLE     Direct Submission
  JOURNAL   Submitted (24-FEB-2020) Biology, Portland State University, 1719 SW
            10th Ave, Portland, Oregon 97201, USA
COMMENT     Bankit Comment: ALT EMAIL:ide@pdx.edu
            Bankit Comment: TOTAL # OF SEQS:451
            Bankit Comment: GAP: est. length - alignment

            ##Assembly-Data-START##
            Assembly Method       :: MetaSPAdes v. 3.12; IDBA-UD v. 1.1.1;
                                     MEGAHIT v. 1.0.3
            Sequencing Technology :: 454; Illumina
            ##Assembly-Data-END##
FEATURES             Location/Qualifiers
     source          1..3538
                     /organism="Crucivirus-291"
                     /mol_type="genomic DNA"
                     /isolate="SR3_42523"
                     /isolation_source="Water"
                     /country="New Zealand"
                     /collection_date=2012
     CDS             1..873
                     /codon_start=1
                     /product="Putative Replication Associated Protein"
                     /translation="MPYRTKSGIKARYKYACFTDFVQLNHENYFTNKMDNVSYGVAGY
                     EICPDTKKPHIQGYVEFIKPINRDQIQTILGNKKMHVTERWATAQQAADYCKEDGTFT
                     EVGTISKPEQGKRNDLIDIRDKIINGISDKDLVMEHTEDYARHYKCFDRWRYMFQPHR
                     TWKTEVHILWGEPGCGKSHRAVLEEGGELISFENKFVLGYKNQEVVVLDDFNPGMIPL
                     EFMLRLTDKYHMVVNVKGGEMTWNPKKIYITTNRNPEVWYGKAEQWLRRVTSVTKLGD
                     DAYTGNNVDNIAGI*"
     misc_feature    32..50
                     /note="stem-loop"
     misc_feature    37..45
                     /note="nonanucleotide"
     CDS             complement(1975..3435)
                     /codon_start=1
                     /product="Putative CruV Capsid Protein"
                     /translation="MSFSDILSFSKFSLLCIFTFSFLLCNFESLRRLFFLNFFQKFSP
                     LFLFLFKGTISTIRIINYSTMPFVPRKKIIKRKRPTTRYMRPKAVKRVRKARAVPYSK
                     AISPASRPSIGSQIGGYLGNAAQNMFSKFLGFGAYDLKQNNMVQTLTPSNQVPFMKSS
                     STSTRIRHREFIQDFVGSSTPFAATYYPINIGMAQTFPWLSSIARNFSSYRFHGLIFE
                     FKSLSGDDQNKQSQGYVAGAVDYNSDSPFWVTKSQIENAMFSASSKPSTDIIFPVECD
                     PKHNQVSEFYVRSSALASTSSELLYDIGTFTIARGSNLNSTDTIGELWVSYDVELISP
                     ILGAGQLALPSAHYTSTNYTNLLPLGSTRTTVFDNMGLTISSTTITFPLGTLGKFALY
                     VQQTGANTGNVVPGSVSFNAGATALISLQNGSASGISAPLYTGAITGPNTSVYTTFTI
                     PSATVQAIITIGTAGTLPTSPSSCDIFVMPLNATLT*"
ORIGIN
        1 atgccttatc gaacgaagtc aggaatcaaa gcacgctata aatacgcgtg tttcacggat
       61 ttcgtgcaac tcaatcatga aaattatttc actaacaaaa tggataacgt gtcctatgga
      121 gtggcgggat acgagatctg cccggacact aagaaacctc atatccaggg atatgttgaa
      181 tttataaaac ccattaacag ggaccaaatt caaacgatct tgggaaacaa aaagatgcat
      241 gtgaccgaaa ggtgggcaac cgcgcagcaa gctgctgatt attgcaaaga agatgggact
      301 ttcacagaag ttgggaccat aagtaagcca gaacaaggga agaggaatga tctcatagat
      361 attcgtgaca aaattatcaa tggcatctca gataaagact tggtgatgga acacaccgaa
      421 gactacgcgc ggcactataa gtgttttgat cgttggcgct atatgtttca accgcacaga
      481 acctggaaaa cagaagtaca tattttatgg ggagaaccag gatgcgggaa gtcgcacaga
      541 gccgtgctcg aagaaggggg agaattgatt tcattcgaaa ataaattcgt actaggttac
      601 aagaaccaag aagtggtggt gttggacgat ttcaatccag gaatgattcc gttggaattt
      661 atgttgcgat taaccgacaa gtatcacatg gttgtgaatg tgaaaggagg agagatgacc
      721 tggaacccga agaagatcta tataacaaca aatcgaaacc cggaggtgtg gtacgggaag
      781 gccgagcagt ggctccggcg cgtgacttcc gtcactaaat tgggggatga cgcgtacaca
      841 ggaaataacg tcgataatat cgcagggata taaaagtttt aacagttcaa tatattttag
      901 tttatcaaaa aacgtattca tttcattcat gttagtcgcg cttcgcgctc tcgcactacg
      961 tgctcagtcc tggcgcttcg cgctgcggac ctcgtttcac tcgggtcgcc ttcggctctc
     1021 acgtcgcgct tcgcgctcct tctcgcttcg ctcgagttcg gcttcgcctc acgggctcgc
     1081 tgcgctcgcc gcaatcgctt cgctctcgct gatcgttggg atcgttgggg tagtggggtg
     1141 tttcctcggc ttggggcctc gtcaaggtgt ttcgcctgaa cggctcaagt cgcgctgcgc
     1201 gctccaagtt cggcttcgcc tcacgggctc gctgcgctcg ccgcaatcgc ttcgctctcg
     1261 ctgatcgttg ggatcgttgg ggttgtgtgg tgtttcctcg gcttggggcc tcgtcaaggt
     1321 gtttcgcctg aacggctcaa gtcgctcgta cctcgctcca tcgttgggat cgttggaact
     1381 gtagttattg ttataatcgt tgaagatcgt aggcgctcgc attggctcgc ttaattcgtc
     1441 gcctttttgg gctcctcata caccgcaggg gtgataacgt gcacgtgcac gtgcactgaa
     1501 gtggctggta atactagggc tggctcgttg cactcgctgc gcccttcagc cacttcctag
     1561 agggatcgta ggatttctag ggatcgaagg atcgaagaga tgatgactac gttcggggct
     1621 gcgcgccctc actgcgcgct gcgcttgcct acgttacggc gctgcgcggc ctccactgcg
     1681 cggtcgtacc tcccttgcct gcgcgcgctc gtacctcgca cgctgcgccg gcgctatagt
     1741 cgctgcgctc cttcgctctg ttgcgggggg ttagataggt tggctggttt ggtggtgagg
     1801 tgctagatgt gtgcttttgg gctttttgct caccgcaaaa aactgcaccg cgctgcgcgc
     1861 ccgcccatga aggtgccgaa aatttctcgt agggttcgta agaaatttaa ttcacaaagt
     1921 gaatcgaata tgaaaaaaaa ccatttattg gaaaaaatta agtatagaaa gaatttaagt
     1981 taacgttgcg ttcaacggca tcacgaagat atcgcaagac gacggagatg ttggaagggt
     2041 gccagctgta ccgatggtaa tgatcgcttg tacggtcgca ctcgggatag taaacgtagt
     2101 gtacaccgag gtgttaggac ccgttatagc accggtgtag agaggtgctg agatgccgga
     2161 cgcgctcccg ttttggagag aaataagagc cgtagcacca gcgttaaaag acactgatcc
     2221 agggaccacg ttgcctgtat ttgctcctgt ttgctgaacg tataacgcaa acttaccaag
     2281 agtaccaagg gggaacgtaa tcgtggtgct cgagatcgtc aggcccatat tgtcgaagac
     2341 cgtagtgcgt gtagatccga gagggagaag gttggtataa ttcgtagaag tataatgcgc
     2401 ggatggaagc gcaagttggc ccgcgccaag aataggggaa atgagctcaa cgtcatatga
     2461 aacccaaagc tctccgatcg tatcagtcga gttcaagttg ctgccgcgcg caatcgtgaa
     2521 ggtaccaatg tcgtaaagaa gctcggaaga agtagaggca agagcgctcg aacgaacgta
     2581 aaattcggaa acctgattgt gcttagggtc gcactccaca gggaaaataa tatcagtcga
     2641 tggtttgctg gacgccgaaa acatcgcgtt ttcgatctgt gacttagtta cccagaaagg
     2701 actatcggag ttgtagtcca ccgcaccggc tacatagcct tgagactgct tgttctgatc
     2761 gtcaccagaa agagatttga actcaaatat caaaccatgg aaccggtatg aagagaagtt
     2821 tcgcgcaata ctgctcaacc aagggaatgt ttgggccata ccgatattaa ttggataata
     2881 tgtcgcagca aatggagtag aagaaccgac gaagtcttga atgaactcac gatggcgaat
     2941 cctcgtcgac gtactcgatg atttcatgaa cggcacttga ttcgaaggag taagggtctg
     3001 aaccatgttg ttttgtttga ggtcgtacgc tccaaaaccg agaaacttgc tgaacatgtt
     3061 ttgtgcagca ttacccaagt agccgccaat ttgactgcca atagaagggc gcgatgcagg
     3121 tgagatcgct ttggagtagg ggacagcgcg cgccttacga acacgtttaa cagctttggg
     3181 ccgcatgtaa cgagtagtag gacgcttacg cttaataatt tttttccgag gaacgaaagg
     3241 cattgttgaa taatttatga ttctaatagt agaaatcgtt cctttaaata gaaataaaaa
     3301 taggggtgaa aatttttgaa aaaaatttag aaaaaatagt cgtcttaaag actcgaaatt
     3361 acatagtaga aaagaaaaag taaaaatgca aagcaatgaa aatttcgaaa acgacaggat
     3421 gtcagaaaac gacatcaatt acgcgctgct cacgccgcgc ccagcgcctg acggcgtgga
     3481 tgaagaagtt gttgttattg aaaatcctaa gaaacgtcga atctcagata cggaagaa
//
LOCUS       CruV-292                3538 bp    DNA     circular     24-FEB-2020
DEFINITION  Crucivirus-292 isolate 001429_virusbaton.
ACCESSION   CruV-292
KEYWORDS    .
SOURCE      Crucivirus-292
  ORGANISM  Crucivirus-292
            Unclassified.
REFERENCE   1  (bases 1 to 3538)
  AUTHORS   de la Higuera,I., Kasun,G.W., Torrance,E.L., Pratt,A.A.,
            Maluenda,A., Colombet,J., Bisseux,M., Ravet,V., Dayaram,A.,
            Stainton,D., Kraberger,S., Zawar-Reza,P., Goldstien,S., Briskie,J.,
            White,R., Taylor,H., Gomez,C., Ainley,D.G., Harding,J.S.,
            Fontenele,R.S., Schreck,J., Ribeiro,S.G., Oswald,S.A., Arnold,J.M.,
            Varsani,A., Enault,F. and Stedman,K.M.
  TITLE     Direct Submission
  JOURNAL   Submitted (24-FEB-2020) Biology, Portland State University, 1719 SW
            10th Ave, Portland, Oregon 97201, USA
COMMENT     Bankit Comment: ALT EMAIL:ide@pdx.edu
            Bankit Comment: TOTAL # OF SEQS:451
            Bankit Comment: GAP: est. length - alignment

            ##Assembly-Data-START##
            Assembly Method       :: MetaSPAdes v. 3.12; IDBA-UD v. 1.1.1;
                                     MEGAHIT v. 1.0.3
            Sequencing Technology :: 454; Illumina
            ##Assembly-Data-END##
FEATURES             Location/Qualifiers
     source          1..3538
                     /organism="Crucivirus-292"
                     /mol_type="genomic DNA"
                     /isolate="001429_virusbaton"
                     /isolation_source="Water"
                     /country="France"
                     /collection_date=2013
     CDS             1..909
                     /codon_start=1
                     /product="Putative Replication Associated Protein"
                     /translation="MVSIDNIRVRNWCFTLNNYTPEEEEGLKSWAIANVDYLVFGREV
                     GEEKGTPHLQGYVEFANAHRGSALKKLFRGRISWRPRKGTAYQASMYCKKGTQLHAEW
                     VSQKELGPNFGKDAVLFEHGTISNQGKRNDLADVGQAVLAGETMKDIATEYPSTFIKY
                     HKGIFAMRSAIKGIFDFEDRTTKPKVIWRWGATGVGKSRTAREAHKSFYIKDGTPWWD
                     GYEQQEAIIIDDFDGKWPFRDLLRLLDFGPYQGQFKGGYVKINSPFIYITCEFPPELA
                     FANATGDISQVLRRIDQVIHVEAQPS*"
     CDS             1589..1915
                     /codon_start=1
                     /product="putative ORF"
                     /translation="MLKSVINGLYGWTVTHLKRQNYLWYEVYLSPERHINEESMTTEW
                     KCRAGVSMHGGDTRASFPFRLTSPPHLGPPKHRFGRYIGIARDCSFFIKKYSHGGSLD
                     CRMPSS*"
     CDS             complement(2032..3363)
                     /codon_start=1
                     /product="Putative CruV Capsid Protein"
                     /translation="MPRTAVAVRKRAKPKAKRAGASVRGHGDYGVSPSMRYGSTYSNP
                     GPVGKAGRYLGRLAGQAVAGPIGGFLGEKAGGLAHYIGKIFGAGDYVASAGVKMNSIT
                     GGDFQPQALNFNSGKTSVRIRRREFLGDVISSATANTFQIKSYSINPGLAATFPWMSE
                     VCGSTFQQYRINGMVFEFRSMSADALNSTNTALGSVVMATDYDSADVAFTSKQQMENS
                     EFGVSCKPSSCMIHAIECAKGATSISEQYIRAFAIPTNTDIRVYDLGKFYIATTGCQG
                     TSVNLGELWVSYDVTLIKAIDQVPGYIMPLANYVITGAVPASAPLGSSRAIASGGVDT
                     IGLTFTANRIIFSKDYPVGSTFAWTYAYNMASGVVTPPTVTYATGLTNLALYSAPLGG
                     ETATRMVLSGFFQVTAMSTTDYYVNIANFTAGATTGCILDVYQVSGVPQAP*"
ORIGIN
        1 atggtgagta ttgataacat acgtgtacgt aactggtgct tcacacttaa caactatact
       61 ccagaggagg aagaaggatt gaaatcttgg gcgattgcaa atgttgacta tcttgttttc
      121 ggacgtgaag tcggtgaaga aaaaggtact ccacatcttc aaggttatgt agaatttgct
      181 aatgcacatc gtggaagtgc actgaaaaaa ttgtttcgag gacgaatcag ctggcgtccc
      241 cggaaaggta cagcttatca agcttcgatg tactgtaaaa aaggaacgca attacatgcc
      301 gaatgggtgt cccagaagga actcggaccc aatttcggaa aggatgcagt actttttgaa
      361 catggaacta tcagtaatca aggtaaacgt aatgaccttg ctgatgttgg ccaagcggtg
      421 ttggctggtg aaactatgaa agatatcgct acggagtatc cttctacgtt cataaaatac
      481 cataaaggta tcttcgcgat gagatcagcg attaaaggta tcttcgattt tgaagatcgc
      541 acaactaaac ccaaggtgat ttggcgatgg ggtgcgacag gtgtcggaaa gagtcgtaca
      601 gctcgtgaag ctcacaaatc tttctatata aaggatggta cgccatggtg ggatggctat
      661 gagcagcaag aagcaatcat tattgatgat tttgatggaa agtggccttt ccgtgactta
      721 ttgcggctgc ttgattttgg accttaccaa ggacaattca agggaggtta tgtcaagata
      781 aactctccat tcatttacat tacatgtgaa tttcctcccg aacttgcctt cgcaaatgcc
      841 acaggtgaca ttagtcaggt cttacgacgc atcgatcaag tgatacatgt cgaagctcaa
      901 ccctcctaat ttggagaggt tgcggattaa atcttgaatc tattatatgc aataatagag
      961 caatatttga tcaaaggttg agggtaccta aaggtaccct caattcgggg actaaagtcc
     1021 ccgaacatgc gtctgcgacg catgccatgc ctcatacggc ggcaaactgc ctaactgatc
     1081 attttttgct ccaaagttgc ataaaactga ttctaaaaaa aatccatgtt tgacacagat
     1141 gttttttcca cttatgttat tcctatgtgc cctaaaaccc gtttagaagc ccatatccat
     1201 tttggcacag aagtggtggg gtaatactaa gcgtgtcaag tcacgctttg cccaccacaa
     1261 cagtgctttg ctcacttctg agctactttc caggtaccaa atttggtacc tgggatattt
     1321 atttgttcat agaactttac aatactaggt ataacgaatc gttacaccta gatgaatatc
     1381 cctcgtgaac tcgtagatat tatagcagaa tatgtttgtc ctccggacga aaacatggat
     1441 gtgttaaatg aattaacaca acttcatctt cgctggaagg aagatgtatg tgatggattt
     1501 gatgaaacaa aaacattctt cgaatggtgg acatttgaac aatgccagga gtcgagatat
     1561 ctagactggc tgtgggaaca gcagtgtatt gctgaagagc gtcatcaatg gcctatatgg
     1621 atggacagtg acgcatttga agcgtcagaa ctatctttgg tatgaggttt atcttagccc
     1681 ggagaggcat atcaatgagg agtcgatgac gacagaatgg aaatgccgcg cgggggtatc
     1741 aatgcacggg ggcgataccc gcgcgtcatt cccattccgt cttacatcgc cacctcatct
     1801 cggccctcca aaacatagat ttgggagata cattggtatt gcccgagatt gttctttttt
     1861 tatcaaaaaa tatagccatg gaggctcttt agactgccga atgcccagca gctaggaggg
     1921 agacgcgagt ctcgatacat tgtcattggc aaacatttgc cgacgccgta tctttttttt
     1981 gtttattgtc gggaccattt ttgggtccca tccttgagag actttgtact cttaaggggc
     2041 ctgtggcact cctgacactt gatagacatc aagtatacaa ccagtggttg cacctgcggt
     2101 gaaatttgca atattgacat aataatcagt tgttgacatc gcggttactt ggaaaaatcc
     2161 agaaagaacc atacgcgttg ctgtctcacc acctaacggg gcagagtaca gagcaagatt
     2221 tgtcaatcca gtagcataag taacagtagg aggagtgaca actcctgaag ccatgttata
     2281 tgcataggtc cacgcaaatg tagagccaac aggataatcc tttgaaaata tgatccgatt
     2341 ggcagtaaat gtcaaaccaa ttgtgtcaac tccaccagac gcgattgcgc gtgaagaacc
     2401 aagtggagcg cttgcaggaa ctgcacccgt aatgacatag tttgccaagg gcatgatgta
     2461 gcctggaacc tggtcaatag ccttgatcag ggtcacatca tacgagaccc agagctcacc
     2521 aaggttcaca ctggttccct gacaacccgt tgtagcgatg tagaactttc caagatcata
     2581 cacgcgaata tccgtgttcg taggaatagc gaatgcccga atatactgct cactgataga
     2641 agtagcgccc tttgcgcatt caattgcgtg aatcatacaa gaagacggtt tgcatgagac
     2701 tccgaactct gagttctcca tctgttgctt ggatgtaaac gcaacgtcag cactgtcata
     2761 gtccgtagcc ataactactg aacccagtgc cgtgtttgtc gagttgagag catcagcact
     2821 catacttcga aactcaaaca ccattccatt gatacgatac tgctggaatg tagatccgca
     2881 tacttcagac atccaaggaa acgttgccgc caacccaggg ttgatcgagt aactcttgat
     2941 ctggaaggta ttagcggttg cagaactgat cacatcgccg agaaactcgc gacgtctgat
     3001 ccgtactgaa gtctttcctg aattgaaatt caaagcctga ggttgaaaat ccccgccagt
     3061 gatactgttc atcttcacgc cagcggaagc cacgtaatca cctgcaccga agatttttcc
     3121 gatatagtgg gctaagcctc ctgccttctc tccgagaaag cccccaatcg gtccagcaac
     3181 tgcctgtccg gcaagacgcc caaggtaacg cccagcctta cccacggggc ccggattgct
     3241 gtaagttgat ccataccgca tagacggact aacgccgtag tcgccgtgac cgcgaacact
     3301 agctccggca cgtttcgctt tcggcttagc acgcttacgc actgcaacag cggtacgagg
     3361 catctaattt tttcttcgaa aaagttttgg tccaggtgag agtatacact atcaccgaga
     3421 cttcttaaaa agaaaattaa ataaatgaat atttattttc cttcttagca tggcacagaa
     3481 gttacctata tagtcagtga cataccctcg tgtaatcaag actcatttct ttggacgg
//
LOCUS       CruV-293                3545 bp    DNA     circular     24-FEB-2020
DEFINITION  Crucivirus-293 isolate LSLA_53418.
ACCESSION   CruV-293
KEYWORDS    .
SOURCE      Crucivirus-293
  ORGANISM  Crucivirus-293
            Unclassified.
REFERENCE   1  (bases 1 to 3545)
  AUTHORS   de la Higuera,I., Kasun,G.W., Torrance,E.L., Pratt,A.A.,
            Maluenda,A., Colombet,J., Bisseux,M., Ravet,V., Dayaram,A.,
            Stainton,D., Kraberger,S., Zawar-Reza,P., Goldstien,S., Briskie,J.,
            White,R., Taylor,H., Gomez,C., Ainley,D.G., Harding,J.S.,
            Fontenele,R.S., Schreck,J., Ribeiro,S.G., Oswald,S.A., Arnold,J.M.,
            Varsani,A., Enault,F. and Stedman,K.M.
  TITLE     Direct Submission
  JOURNAL   Submitted (24-FEB-2020) Biology, Portland State University, 1719 SW
            10th Ave, Portland, Oregon 97201, USA
COMMENT     Bankit Comment: ALT EMAIL:ide@pdx.edu
            Bankit Comment: TOTAL # OF SEQS:451
            Bankit Comment: GAP: est. length - alignment

            ##Assembly-Data-START##
            Assembly Method       :: MetaSPAdes v. 3.12; IDBA-UD v. 1.1.1;
                                     MEGAHIT v. 1.0.3
            Sequencing Technology :: 454; Illumina
            ##Assembly-Data-END##
FEATURES             Location/Qualifiers
     source          1..3545
                     /organism="Crucivirus-293"
                     /mol_type="genomic DNA"
                     /isolate="LSLA_53418"
                     /isolation_source="Odonatan larvae"
                     /country="New Zealand"
                     /collection_date=2013
     CDS             1..1083
                     /codon_start=1
                     /product="Putative Replication Associated Protein"
                     /translation="MFTYYIYGREVGEQGTPHLQFYVAGAKKITFKCLKKIFPRAHLL
                     VCKGSPDQNYKYCSKDGNFVTYGDLPAAKTAKATKAAKDKWDEVKELAIAGDLDAIDS
                     EVFIKHYPTLKRIRSDHRMKAKLPTLDWTASPNIWIYGPPGVGKSRRARLTYPDAYIK
                     APQNKWWGGYNDEPVVLIDDLRMDQDYQLSNLITWADRYPFQAEIKLDSTGMIRPRVI
                     VVTSNFHPSQIWKRPEDINSILRRFRMVNMELLEQFDTTESTKKRKVVEEVTPEKPPL
                     FRQNATGNLVPFVDLQPKITKVFDLTSSVTGIDMELTQDDEPAVIDLTDVDNAIAEAT
                     QIYKMCDRCGDKVIECSCDMANPSMY*"
     CDS             complement(1116..2567)
                     /codon_start=1
                     /product="Putative CruV Capsid Protein"
                     /translation="MSYYAGRDVAMRGNDVQERGRYPTYYRIKKGLAPDQYWRARYYR
                     RRFTGRGDYRSVRRPVLKRRKVARRSLRGRGDYSGGTGYGAAIGSYLGSKAGGYLGGM
                     AEHAVMGLVSGLGDYKVKKNVLLGGNMPKIENHSGPGGITVQFQEYLGDITTPVQAGN
                     SSPFDIKTYLINPAVTQTFPWLSQIAANFEQYEIEGMLFGFRTTSADMVTNNSNNLQL
                     GTVIMATQYDVADAVFASKAEMLNYEFASSVKPSEGAVHMIECDPRQTSVNLLYTSPG
                     PTPAGTDPRLYHLGRFAIATQGFQGSGVTIGELHITYQVRLLKPKLWTGLGQTIPFSY
                     FTNQWINANTQSWMGPVNTISLGGVANGCGITFTNLPTADDVLVNIPGNPKPVSYLFT
                     VEWLTTSGAMFATTLPVPDPLANCAVFGASYAPSQANAASTRAIAVWKVTTSPSTGAA
                     LFQLGVPTQATSLPPGNVVVNMQVTVVDPDFVI*"
     CDS             2569..2919
                     /codon_start=1
                     /product="putative ORF"
                     /translation="MCSDVSCTCAPRNVWVWGCGYVQRGVGYGHGLARVCPTVPCPTV
                     GGNTKAFGAPDRFKGLFGAFVFLYLAASPGLCVTMVNMVSLWCYGGKCVVFPSLDCAV
                     GGTRGPSPLGPPAR*"
ORIGIN
        1 ctgttcactt attatatcta tggtcgagag gtgggtgaac agggaacgcc gcatttgcaa
       61 ttctacgtag ctggggctaa gaagataacc ttcaaatgct taaagaagat ttttcctcgt
      121 gcgcatctgt tggtctgtaa gggttctcct gatcaaaatt acaagtactg ttccaaagat
      181 ggtaattttg tgacctatgg tgacttgccc gcagcaaaga ccgctaaagc tactaaggct
      241 gctaaggaca agtgggacga agtaaaagag ttggcaatcg ctggtgactt ggacgctata
      301 gatagcgaag tgttcattaa acactaccca actcttaaac gtattcgatc agaccatcgg
      361 atgaaggcca aattacctac gctggattgg actgcttctc caaatatttg gatctatggc
      421 ccccctggag tcggtaagtc tcgtcgtgct cgccttacct accctgatgc atacatcaaa
      481 gctccgcaga ataagtggtg ggggggctac aatgatgagc ctgttgtcct cattgatgac
      541 ttgcgcatgg atcaagatta ccagctttcg aatcttatta cttgggctga tcggtaccct
      601 ttccaggctg agatcaaact cgattcgaca ggtatgatta ggcctagagt gattgttgtc
      661 acttcaaatt ttcatccgag tcaaatatgg aaacgtcctg aggatatcaa ttctattttg
      721 cgccgctttc gtatggtaaa catggagctt ttggagcaat ttgataccac tgagtctact
      781 aagaagcgga aagttgtgga agaagtcact ccggaaaagc ctcctctctt tcgacagaat
      841 gctactggta acttagttcc gttcgtggat cttcaaccga agattaccaa ggtgtttgat
      901 cttacttcct ctgttactgg aattgatatg gaacttaccc aggatgatga acctgccgtt
      961 attgatttaa ctgatgtgga taacgccatt gctgaagcta ctcagatcta taagatgtgt
     1021 gaccgatgcg gtgataaagt tatagaatgt tcatgtgata tggctaatcc atccatgtat
     1081 tgatttattt tgttattaca gaaataaata ttttgttaaa ttacgaaatc tgggtcaact
     1141 acggtgactt gcatgtttac aacaacgttg ccgggtggta atgaagtcgc ttgtgttggg
     1201 actccgagtt ggaagagtgc cgcgcctgta gatggactgg ttgtaacttt ccatacggcg
     1261 atggctcggg tagatgctgc attagcttgc gagggtgcgt aggacgctcc aaacactgca
     1321 caattggcta aagggtccgg tactggtaaa gttgtggcaa acatagcacc ggaagttgtg
     1381 agccattcga ctgtaaacag ataggataca ggtttgggat ttcctggaat atttaccaaa
     1441 acgtcatctg ccgtaggtaa attggtaaag gtaatgccgc atccattggc tactcctcct
     1501 aatgaaattg tattaacagg gcccatccaa gattgcgtgt tcgcattgat ccattggttg
     1561 gtaaagtagg agaacggaat cgtctggcct aagccggtcc ataacttggg cttgagaaga
     1621 cgaacttgat atgtgatatg tagctcccca atagttacgc cactgccttg gaatccttgg
     1681 gtagctatag cgaagcgccc caggtgatac agcctagggt ctgtgcctgc gggagtaggc
     1741 cctggggaag tgtagagtaa attaacggac gtttgtctag ggtcacattc gatcatgtgc
     1801 acggctcctt cggaaggttt cactgatgat gcgaattcgt aatttagcat ttcagcttta
     1861 ctggcaaata ccgcgtctgc gacatcgtat tgagttgcca taataactgt tccaagctgt
     1921 aagttgtttg agttgttggt gaccatgtca gcggaggtag tccggaaacc aaacaacatt
     1981 ccttcgattt catattgttc aaagttagca gcgatctgcg ataaccatgg gaacgtctgc
     2041 gtaactgctg gattaataag ataagttttg atatcgaagg gagaactgtt gccagcttgg
     2101 acaggtgttg taatgtctcc taagtactct tgaaattgaa cggtaatgcc accgggtccg
     2161 gaatgatttt caattttagg catattgcca cctaggagaa cgttcttctt cactttgtaa
     2221 tctccgagtc cggagaccaa gcccataaca gcgtgttcgg ccatgcctcc taagtatcct
     2281 ccggcttttg atccgagata tgagccaata gctgcgccat agccagttcc tccagaataa
     2341 tcacctctac ctcgcaagga gcgtcgtgcg acttttcgtc gcttaaggac aggtcttcta
     2401 acagatcgat aatcgcctct accagtaaat cttcgtcgat agtaacgcgc tctccaatac
     2461 tgatcaggag cgagaccttt tttgatgcgg tagtatgtag ggtaccttcc tcgctcttga
     2521 acatcgttcc ctcgcattgc aacatctctt cctgcatagt atgacatcct gtgttctgac
     2581 gtttcatgca cgtgcgcacc gagaaacgtg tgggtgtggg ggtgtggata tgtccaacgg
     2641 ggcgttggat atggacatgg gttggcacgt gtgtgtccaa cggttccatg tccaacggtc
     2701 gggggtaata ctaaggcctt cggcgccccc gaccgtttta aaggcctttt tggggccttt
     2761 gtatttcttt atttagcggc ttcgccgggg ttgtgtgtca ctatggtcaa tatggtgtca
     2821 ctgtggtgtt atggagggaa atgtgtggta tttccctcgc tggattgtgc cgtggggggg
     2881 acccggggcc ccagtcccct gggtcccccc gcacggtagt ttaaccttct cgacccctga
     2941 ctaagagtca agcatagttt ctctgaagct taccagatta cctctgatat aaggggttcg
     3001 agtgtagatt gattggattt tgatccaact agggttattg ggtgaattag ggttattgac
     3061 caaactaggg ttagcggttt cgtaacccta gtaccgcggc tgctggcact accgcggctg
     3121 ctgacagcca ctgccgcggc tgctggtata taaataaaaa aaaaaaaaaa aatagcccaa
     3181 tgcatttatc gggtaatgca tttttcgggt cggccctgaa aaaatgattt tgggctttgc
     3241 aatccacatc ctcattttct gaactgcatg gaccctacac cacacgtcga aaatttatcc
     3301 aatttgttta ctggcgatga atcaaccaag ggatacgcta ttcactggta tgatgttctc
     3361 ccatcatttt catttctggg ggggtgagga ttcaaaagcc caaattggtt ttgtcatcac
     3421 gtgcgttttg gacttagcga aattttcaaa aaaaaaaaaa aaaaaattgt tattaatttt
     3481 ttaggtgtgg cacaatcaat aatttttctg aagttgacga agcttgtttt actgctaatc
     3541 gcagt
//
LOCUS       CruV-294                3546 bp    DNA     circular     24-FEB-2020
DEFINITION  Crucivirus-294 isolate 001423_virusbaton.
ACCESSION   CruV-294
KEYWORDS    .
SOURCE      Crucivirus-294
  ORGANISM  Crucivirus-294
            Unclassified.
REFERENCE   1  (bases 1 to 3546)
  AUTHORS   de la Higuera,I., Kasun,G.W., Torrance,E.L., Pratt,A.A.,
            Maluenda,A., Colombet,J., Bisseux,M., Ravet,V., Dayaram,A.,
            Stainton,D., Kraberger,S., Zawar-Reza,P., Goldstien,S., Briskie,J.,
            White,R., Taylor,H., Gomez,C., Ainley,D.G., Harding,J.S.,
            Fontenele,R.S., Schreck,J., Ribeiro,S.G., Oswald,S.A., Arnold,J.M.,
            Varsani,A., Enault,F. and Stedman,K.M.
  TITLE     Direct Submission
  JOURNAL   Submitted (24-FEB-2020) Biology, Portland State University, 1719 SW
            10th Ave, Portland, Oregon 97201, USA
COMMENT     Bankit Comment: ALT EMAIL:ide@pdx.edu
            Bankit Comment: TOTAL # OF SEQS:451
            Bankit Comment: GAP: est. length - alignment

            ##Assembly-Data-START##
            Assembly Method       :: MetaSPAdes v. 3.12; IDBA-UD v. 1.1.1;
                                     MEGAHIT v. 1.0.3
            Sequencing Technology :: 454; Illumina
            ##Assembly-Data-END##
FEATURES             Location/Qualifiers
     source          1..3546
                     /organism="Crucivirus-294"
                     /mol_type="genomic DNA"
                     /isolate="001423_virusbaton"
                     /isolation_source="Water"
                     /country="France"
                     /collection_date=2013
     CDS             complement(619..2019)
                     /codon_start=1
                     /product="Putative CruV Capsid Protein"
                     /translation="MPPKYDIKKAFLGANNQRRLKSASKAYTGKNAERKRRNTAGAIR
                     RYVNAYGPTLAGALTGMGDYQVRDPGSLGRSYRVGRGIHPESGSMGYVHGKTHHSHSS
                     HHPHTSMEKGMMSIKHSEYIGDLISSATTGSSNFVSQSYALNPGNSGTFPWLASTAIN
                     FQHYRFKKLVFEYRPLTSESTSSTAVTLLSMGSVIAATQYNSVLGPYTTKQQMAESDF
                     SVTTKPSEHMLHAVECDPRFNPLGELFISAQTSLTIGANSSDIRMQNLGIFQISSCNI
                     PVAASTAIDLGEIWVHYEVELYKPQINAYLSGLLSSHYTGTSSTGNPTNSNPFGPNIL
                     ATVQPVATANNLLALTFGVSSFTFPLQVTSGQYLCVYYAKGGAAACAYTAPTCTNGTI
                     DVLWTGGTLGTDDVSTVAGAPQTTLAGATQLSIAFVVNVNAPGASLCVVTMAVTVVPV
                     TGSFDLIVTPYNSIMN*"
     CDS             complement(2110..3546)
                     /codon_start=1
                     /product="Putative Replication Associated Protein"
                     /translation="MSEIPKKSSKSLATIISDVVIVKTITKASKKPSVSKASSTPKRA
                     RATESTEEEEGASPPTMPKSSKKTKASSATKSHEREEGDGSGPPTKARGWCFTINNYT
                     LEDPEIFRTYAAKIDYSIFGFEVAPTTGTPHMQCYMNHNNTIRFQTIRKLFPRAANIV
                     QAKGTAKHNQRYCKKGEQSKEEYDELLDQGPNYGKNAVWEEFGECPESVGAGHRTDLD
                     IIGDQLRDGTTTVDELARSLPLDTFNRYSRTFAYMQNLYQRERRREGDLQCFFLYGTT
                     GSAKTREYTRGYREDEIFNQLPDSVRFSGYTGQRCFVMNEVRGDVPFRYILGIADPSA
                     PYPIEIKNERLLQLMTATRIVITSCHPPEKLWPDESPTDMLQLHRRYTVVKLDGNRRD
                     VVTNKQVYTLPWVDHPNFLSEVDKWEGTKQEREQRLQFAFEAYREDKSSWDEHMKDTQ
                     YEYRHRRLIKYQELGLFRLHDDESLDNP*"
     misc_feature    3241..3266
                     /note="stem-loop"
     misc_feature    3246..3254
                     /note="nonanucleotide"
ORIGIN
        1 tttaaaaaat atgacgtaga tgtttttttt gaatgaatga atgttaaaaa tggttttaac
       61 ctatgtggca gaaaataaaa aaaggatgtt ttaacctaaa aagtaaaata tgacctatgt
      121 ggcaaattaa aaaaaatcta attaattggt aactccttac acgtttataa ataaactccc
      181 gcataatctt acgctatgct tggttacgtc gtcctcttcg aaatcaatag attttgccac
      241 agaggtactc taaaggggta ataataaccc tttagattct caaaggaact aaagttcctt
      301 atcgaaatcg tgcggtaaac cgccctcgta cctctaattt aaattaatat gtaaaacata
      361 cagcacatta tactgtaata catatctagg gggcttcgcc ccagtctatt ctgtggtggg
      421 gtgtgtgtgc cttcggcctt tcttatttat atcttttgca gtagttcaat gctccattac
      481 ctcgcatcgt aattttaaag catcattgtg tatggttcat taccaacagt cataattaat
      541 gaacttcgtt ctcacggttt ttgtaaatta ataattataa ttacaaaaca cctacaattc
      601 tctttaaaac aaaaatacct aattcatgat agagttatac ggagtaacta tcaaatcgaa
      661 actacccgta actggtacaa cagttacagc catagtaacc acacatagtg aagcacctgg
      721 ggcattcaca ttaaccacaa atgcaattga cagctgagta gctcccgcta atgttgtttg
      781 tggggcacct gcaacagtac tgacatcatc agtaccaagt gtaccgccag tccacagcac
      841 atcaattgta ccattagtgc aagtaggagc agtatacgca caagctgctg cacctccttt
      901 tgcataataa acacataaat actgtccgga agtaacctgt aatggaaacg taaatgatga
      961 aacaccaaac gttaaagcta acaaattatt agcagttgca actggttgaa cagtagcaag
     1021 aatattaggt ccaaaaggat tactattagt aggattaccg gttgacgaag tacccgtgta
     1081 atgcgaagat aacaacccgg acaaataagc attaatctgc ggcttataaa gctctacctc
     1141 ataatgcacc catatctcac caagatcgat tgcagtactt gcagcaacag gtatgttgca
     1201 acttgatatc tgaaaaatac caagattctg catgcgaata tcagatgaat tcgctccaat
     1261 agttaagctt gtttgtgccg atataaacag ctcacctaat ggattaaaac gaggatcaca
     1321 ttctacagca tgtaacatgt gctctgatgg cttagtagtg acagaaaaat cactctccgc
     1381 catttgctgc tttgtagtat agggtcccaa tacactgtta tattgagtcg ctgcaataac
     1441 agatcccata cttaacaaag taacagcagt agaagaagta gattcagatg ttagaggacg
     1501 atactcaaac accaacttct taaaacgata atgctgaaag ttaatcgcag tagacgctaa
     1561 ccacgggaat gttcccgaat tacccggatt aagagcgtac gattgagaaa caaaattgga
     1621 agatccagtt gtagctgatg aaataagatc tccaatatat tctgaatgtt taattgacat
     1681 catacctttc tccatactag tatgcggatg atgcgaacta tgtgaatgat gtgttttccc
     1741 atgcacatat cccatagaac cagattcagg gtgaattcca cgaccaacac gatacgaacg
     1801 tcctaacgat ccaggatcac gtacttgata atcacccata cctgtcaatg caccagctaa
     1861 agtaggtcca tacgcattga cataccgacg aatagctcca gcagtattcc gtcgtttacg
     1921 ttcagcattc tttcctgtat acgctttaga cgctgacttc agacgtcttt gattattagc
     1981 tcccaggaaa gcctttttga tgtcatattt tggaggcata ttagaaatat ttgattggtt
     2041 gaaaatgtaa ttagattatg aattacattt acaaaagact aaaaacaaag aaaatacctt
     2101 tgtggcaaat taaggattat ctaaagattc atcatcatgt aatctaaata accctagttc
     2161 ttgatactta ataagacgac gatgacgata ctcatattga gtatccttca tatgttcatc
     2221 ccatgaagat ttgtcctcgc gataagcttc aaaagcaaat tgcaaccgtt gctcacgctc
     2281 ttgcttggtt ccttcccatt tatcaacttc actgagaaaa ttaggatggt caacccaagg
     2341 caaagtataa acttgtttat tagtaacaac atcacgacga ttaccatcga gtttaacaac
     2401 ggtatatcgg cgatgtaact gtagcatatc agtcggagat tcatccggcc acagtttttc
     2461 cggtggatga catgacgtaa taacaattcg tgttgctgtc ataagctgca gtagccgctc
     2521 atttttaatt tcaataggat atggtgcaga aggatctgca ataccaagta tatatctaaa
     2581 agggacatcg ccccttactt cgttcatcac aaaacatctc tgtcctgtgt atcccgaaaa
     2641 tctaacagaa tcaggaagct gattgaagat ttcatcttca cggtatcccc gcgtatactc
     2701 tcttgttttt gcggagccag ttgtcccgta caaaaaaaaa cattgtaaat ctccctcacg
     2761 acgccgctct cgctgatata aattttgcat ataagcaaaa gttcgagaat aacgattaaa
     2821 tgtatctaac ggtaatgatc tagcaagttc atcgacagta gtcgtaccat cacgcaattg
     2881 atcgccaata atatccaaat ctgtacgatg tcctgcacca acggattccg gacattcacc
     2941 aaattcttcc cagacggcat tcttgccata atttggacct tgatcaagga gctcgtcata
     3001 ctcctctttg ctctgctcac ctttcttaca atacctttga ttatgctttg cagttccttt
     3061 tgcctgcact atgttagcag cacgagggaa caacttacgt atagtttgaa agcgtatagt
     3121 gttattatga ttcatataac actgcatatg tggagtaccg gttgtaggcg caacctcaaa
     3181 cccaaaaata gaataatcta ttttcgcagc atatgtcctg aaaatttcag gatcttcgag
     3241 ggtgtaatta ttaatagtga aacaccaccc tcttgccttg gtcggagggc cggatccgtc
     3301 accctcctcc cgctcgtgcg acttcgtcgc actcgatgcc ttcgtcttct tcgaagactt
     3361 cggcatagtc ggagggcttg cgccctcctc ctcctcggtc gactccgtcg ccctcgctct
     3421 cttcggcgtc gaagacgcct tcgagacact cggcttcttc gaagccttcg tgattgtttt
     3481 cacaatgaca acatcactta tgatagttgc caaagatttg gatgactttt taggaatttc
     3541 tgacat
//
LOCUS       CruV-295                3548 bp    DNA     circular     24-FEB-2020
DEFINITION  Crucivirus-295 isolate BS_189.
ACCESSION   CruV-295
KEYWORDS    .
SOURCE      Crucivirus-295
  ORGANISM  Crucivirus-295
            Unclassified.
REFERENCE   1  (bases 1 to 3548)
  AUTHORS   de la Higuera,I., Kasun,G.W., Torrance,E.L., Pratt,A.A.,
            Maluenda,A., Colombet,J., Bisseux,M., Ravet,V., Dayaram,A.,
            Stainton,D., Kraberger,S., Zawar-Reza,P., Goldstien,S., Briskie,J.,
            White,R., Taylor,H., Gomez,C., Ainley,D.G., Harding,J.S.,
            Fontenele,R.S., Schreck,J., Ribeiro,S.G., Oswald,S.A., Arnold,J.M.,
            Varsani,A., Enault,F. and Stedman,K.M.
  TITLE     Direct Submission
  JOURNAL   Submitted (24-FEB-2020) Biology, Portland State University, 1719 SW
            10th Ave, Portland, Oregon 97201, USA
COMMENT     Bankit Comment: ALT EMAIL:ide@pdx.edu
            Bankit Comment: TOTAL # OF SEQS:451
            Bankit Comment: GAP: est. length - alignment

            ##Assembly-Data-START##
            Assembly Method       :: MetaSPAdes v. 3.12; IDBA-UD v. 1.1.1;
                                     MEGAHIT v. 1.0.3
            Sequencing Technology :: 454; Illumina
            ##Assembly-Data-END##
FEATURES             Location/Qualifiers
     source          1..3548
                     /organism="Crucivirus-295"
                     /mol_type="genomic DNA"
                     /isolate="BS_189"
                     /isolation_source="Water"
                     /country="New Zealand"
                     /collection_date=2012
     CDS             1..1275
                     /codon_start=1
                     /product="Putative Replication Associated Protein"
                     /translation="MPATAATKSRKWCFTMFWNGEEPGASYFDDWEVEYIYVGKELCP
                     ETKRDHYQGYVRFPNPRALGGVRKLFTHTVKLPSGRDGYPGHWEACKGSEADNVKYCS
                     KDGVLVVEAGDSEKTANDVKQQGKRSDIIAVRQLIQAGARMHEIVSTVNSYQAIKVAE
                     TMLKFLEPGRDWVPEVYWIYGPTGTGKSKFAFGSCADPWISGKDGKWFEGYDAHEDVI
                     FDDFRQDFCAFHILLRLLDRYPYRVEIKGASRQFLARRIFITSCHPPDKVYPLSGEDL
                     GQLGRRISQIIYMPNLGEAYSRPGSGIGLEPSDCFVIPERGPEQKVWGNTTGPEVPDD
                     VGPEAPALARTQTGFTQVPEHKCSVAHGADVVCDDCEPEDESSGNANGCKFEHSADEF
                     CTYCMDPDEAEVLGELLGEMDLDGSYGDEIDG*"
     misc_feature    965..989
                     /note="stem-loop"
     misc_feature    972..980
                     /note="nonanucleotide"
     CDS             complement(1363..2715)
                     /codon_start=1
                     /product="Putative CruV Capsid Protein"
                     /translation="MPRVAAKQKVVYVKPAAKRATAVVRKPRAVAVKPAPAAKRKPAS
                     SMVSSLLSGGGSLLGGAMGGPAGAALGGAAGKLVGKLFGHGDYTVSNVESINKNNLVL
                     SNAANIPQFGTGKVATKFVHREFLGDIYSAATANTFKIQSYPLNPGQASTFPWLADVV
                     KAKYQQYRFNGCVFEFRSMSSDALNSTNTALGSVIMSTDYDSADVVFASKQEMENTEF
                     SSSCKPSVNMLHAIECERTQTPVNELYIRAYDVPAGKDIRLYDLGRVSIATTGCQGTN
                     VNLGELWVTYDIDCFKAIEQSPYYGTPVSEFKLSGVVSTNALGDLPPTPLVDQIGCTF
                     VRDSGTDTVILPYDMKKGTIFSVYYAVYGASTASIPYQSMTVAGGVVGYGSYAAHPYS
                     ASLTTDNKTRYQVFKYDGTGTPAAPPTLAFTGGLVPNTITNASLVIAVIGGGYVPDGN
                     *"
     CDS             complement(3107..3457)
                     /codon_start=1
                     /product="putative ORF"
                     /translation="MTVELSMFALAKQCAGTREYLKEVDESTNSLGRRCMRLEHEVML
                     LKQAMAEQQDQLHKILNVVLPGLAGNTTGPEVPDDVGPEAKKEISSAQQARRGNGPTR
                     TTKPSAHRVAIEES*"
ORIGIN
        1 atgcccgcta ccgctgctac gaagtctcgc aaatggtgct tcaccatgtt ttggaatggt
       61 gaagaacctg gggcttctta ctttgacgac tgggaagtag agtatatcta cgtcggcaaa
      121 gaactctgcc ctgaaactaa acgcgaccat taccaaggtt atgttcgctt tccgaatccc
      181 cgcgcgcttg gcggcgttcg gaaactcttt acacacaccg ttaaactccc ctccggccgg
      241 gatggttatc cgggtcactg ggaagcgtgc aaaggctctg aagcagataa cgtgaagtac
      301 tgctccaaag atggtgtcct tgttgttgag gctggcgact ctgaaaagac ggctaacgac
      361 gtcaaacagc aaggcaagcg ctcggacatc attgctgtcc ggcagctcat ccaagctggc
      421 gctaggatgc atgagatcgt cagcactgtg aatagctatc aagctatcaa agtcgcagag
      481 acgatgctta aatttctgga acccggtcgc gactgggttc ctgaagttta ttggatttat
      541 ggccctactg gtacgggcaa atccaagttt gctttcggat cctgcgctga cccttggatc
      601 tctggcaaag acggaaaatg gttcgagggc tatgacgccc acgaagatgt catctttgat
      661 gatttccgtc aagatttctg cgcctttcac atcctgctca ggctcctcga tcggtacccc
      721 tatcgcgtcg aaatcaaggg tgcgagccgc cagttccttg ctcgccgcat ctttatcaca
      781 agctgccacc ctcccgacaa agtttacccc ttgtcgggcg aagatttggg ccagctgggg
      841 cgccggattt cccaaatcat ctatatgcct aacctaggtg aagcatacag tcggcccgga
      901 tcgggaatag ggctggagcc ctccgactgc tttgtgattc cggaacgtgg cccggaacaa
      961 aaagtctggg gtaatactac ggggccagag gtacctgacg atgtcggtcc cgaggcccct
     1021 gcgctggcgc gcacccagac tggttttacc caagtgcccg agcataaatg ctctgtcgca
     1081 catggggctg acgtcgtctg cgacgactgt gagccagagg atgaatcctc cggcaatgca
     1141 aacggttgca aattcgagca ttctgcggac gaattctgca cttattgcat ggatccggac
     1201 gaggccgagg tgctcggtga gctactcggt gaaatggacc tcgacggatc ttatggtgac
     1261 gaaattgatg gctaagtacc gctaaagctc gcgaaaaaat tttttagcga ctgattaatc
     1321 aatatcggtc accaggagat attcctcttc tgcggaaact agtcagtttc catcgggaac
     1381 atatcctcca ccaatcactg cgataaccaa actcgcgttt gtgatcgtgt tgggcactag
     1441 tcctcctgtg aaggctagcg ttggtggtgc ggcaggcgtt cctgtgccgt cgtatttgaa
     1501 aacctgataa cgcgttttgt tatcggttgt caaggacgcg gagtagggat gggcggcata
     1561 actgccataa cccaccactc ctccggcaac agtcatactc tggtacggaa tgctcgcggt
     1621 ggacgcaccg taaactgcat agtacacaga gaaaatcgtg cctttcttca tgtcgtaagg
     1681 caagatgact gtgtccgtac cgctatcgcg tacgaacgta caccctattt gatccaccaa
     1741 gggcgttggc ggtaaatccc ccaatgcatt ggtcgatacc accccggaga gcttgaactc
     1801 tgataccggg gttccataat agggcgattg ctcaattgcc ttgaagcaat cgatatcata
     1861 ggtcacccaa agctcaccga ggttcacgtt ggttccctga catccggtcg ttgcgatcga
     1921 gactcgtccc aagtcgtaca accggatatc ctttcctgcc ggaacgtcat aagcgcgaat
     1981 gtacagctca ttgactggcg tctgcgttcg ctcgcattca atggcgtgta gcatattcac
     2041 gcttggcttg catgagctag agaactctgt gttctccatc tcctgctttg atgcgaacac
     2101 cacgtcggcg gaatcatagt cggttgacat gatgacgctt ccgagggctg tgttcgtcga
     2161 gttcaatgcg tcgctggaca ttgagcggaa ctcaaagaca cagccattga atcgatactg
     2221 ttgatatttc gctttgacga catctgcgag ccagggaaac gttgatgcct gtccaggatt
     2281 caacgggtaa ctctggatct tgaatgtatt cgcagtcgct gctgaataaa tatcgccgag
     2341 gaactctcgg tggacaaact tcgttgccac ctttcccgtt ccaaattgcg gaatgttggc
     2401 cgcatttgac aagacgaggt tgttcttgtt aatggactcg acattggaaa ccgtatagtc
     2461 tccatgtcca aacagttttc ctaccagctt tcccgccgcg cctcccagcg cggctcctgc
     2521 tggccccccc attgctcctc cgagcaatga ccccccgccg cttagtaagc tcgacaccat
     2581 actcgatgct ggcttccgct ttgcagcggg ggcaggtttt acagcaacgg cgcggggctt
     2641 tcgcaccact gctgttgccc gctttgcagc gggcttgaca tataccactt tctgctttgc
     2701 agcgacgcgt ggcatgtgcg cgatatgctt tcgcgcataa tctttgggaa aaaaaaatat
     2761 ttaatccgcc aaagctcgga gttataatgg tctgctccca ggacaactcc agccgtatat
     2821 cctgtgccta agaagaaatc actccatttt ggagtgagat attaggatcg gtaaacttgg
     2881 tcccccaggg gcctgaatca ggatgtaatc tatgttaaat ccgatcataa agctggtgtt
     2941 gttactttcg ttcccactcg ttcccactcg cgcccgctcg cgcccacttt gatgccgctt
     3001 gatccctctt tcttcccttc tgtcgcattt ggctgcggtc tattacgtta cgattcgcgc
     3061 ccctggggga cgaacgttac cgaccttgat atcttaccgc taagcgctat gattcctcta
     3121 tagccacccg atgggcggat ggcttcgtcg tcctggtcgg accatttcct ctcctcgcct
     3181 gttgggccga ggaaatctct tttttggcct cgggaccgac atcgtcaggt acctctggcc
     3241 ccgtagtatt acccgctaac ccaggtaaaa ctacgttaag aatcttatga agctgatctt
     3301 gctgctcagc catggcttgc ttgaggagca tcacttcatg ttctaatctc atacatcgtc
     3361 ttccgagaga attcgtactc tcgtcgacct ctttcaaata ctcacgagtg cccgcacatt
     3421 gcttcgcgag cgcgaacatt gatagctcta ctgtcatcgt gagtgatata cctgcaatta
     3481 ttttttcatt ttgaaaaata ttgccggcac tgataccgag ttccaccccg gctcagtcgc
     3541 tctccttc
//
LOCUS       CruV-297                3549 bp    DNA     circular     24-FEB-2020
DEFINITION  Crucivirus-297 isolate 120_1258_IR2_3297.
ACCESSION   CruV-297
KEYWORDS    .
SOURCE      Crucivirus-297
  ORGANISM  Crucivirus-297
            Unclassified.
REFERENCE   1  (bases 1 to 3549)
  AUTHORS   de la Higuera,I., Kasun,G.W., Torrance,E.L., Pratt,A.A.,
            Maluenda,A., Colombet,J., Bisseux,M., Ravet,V., Dayaram,A.,
            Stainton,D., Kraberger,S., Zawar-Reza,P., Goldstien,S., Briskie,J.,
            White,R., Taylor,H., Gomez,C., Ainley,D.G., Harding,J.S.,
            Fontenele,R.S., Schreck,J., Ribeiro,S.G., Oswald,S.A., Arnold,J.M.,
            Varsani,A., Enault,F. and Stedman,K.M.
  TITLE     Direct Submission
  JOURNAL   Submitted (24-FEB-2020) Biology, Portland State University, 1719 SW
            10th Ave, Portland, Oregon 97201, USA
COMMENT     Bankit Comment: ALT EMAIL:ide@pdx.edu
            Bankit Comment: TOTAL # OF SEQS:451
            Bankit Comment: GAP: est. length - alignment

            ##Assembly-Data-START##
            Assembly Method       :: MetaSPAdes v. 3.12; IDBA-UD v. 1.1.1;
                                     MEGAHIT v. 1.0.3
            Sequencing Technology :: 454; Illumina
            ##Assembly-Data-END##
FEATURES             Location/Qualifiers
     source          1..3549
                     /organism="Crucivirus-297"
                     /mol_type="genomic DNA"
                     /isolate="120_1258_IR2_3297"
                     /isolation_source="Water"
                     /country="Norway"
                     /collection_date=2011
     CDS             complement(1..1077)
                     /codon_start=1
                     /product="Putative Replication Associated Protein"
                     /translation="METGGSGKEKWEHIPQAGHDKGDKSKTRNWCFTINNPVRTKEEW
                     LVILQGKNTRYVAMGDEVGENGTPHIQGYVDFVNPHTFGGIVKIFENLAHVEKRRGSW
                     KQASDYCKKDSKFVEFGKPPRQGARGDIDDVKDCVLENMSMNEMIDEGYINNLQQLRF
                     AEGLKKYKETPRDPSNPPRILWYWGPTGTGKTRTAIELYPDCWLSGKNLKWWDSYDGH
                     KHVLIDDFRKDFCTFHELLRILDRYPFKVECKGGSRELMAEVIIITCPFRPEELYEGR
                     TEEDISQVTRRITEIRHFVGSPQLININNGGTEQKSAGNTIAADVLINNSSAIINSSV
                     PISVMKVNEVKVPVVPSPQVFNCY*"
     misc_feature    118..145
                     /note="stem-loop"
     misc_feature    129..137
                     /note="nonanucleotide"
     CDS             1711..3303
                     /codon_start=1
                     /product="Putative CruV Capsid Protein"
                     /translation="MVQYTAAQRAAYGRKMAKLRAARPVYRKSNYSAPRSTRRVRGYG
                     SYAEDWQKVGTEWGGKVGNVAGGIADLATGFEKAVSGFGSYKVKRNSLLSEGNDPPEI
                     RNAKDGRFIIRHREYLRDIVAPSVGVTSTAFNLDRFNIQPGDSSSFPWLATLAQNFEQ
                     YSLRGAIYEFKSTYSDSSVAGSLGTVVMATEYDSTRPDFLNKAAMENHQFANSCKPSH
                     SMMHIIECARSQTPVSELYIRDDEIPVTGDQRLYDFGTFQIASVGVPNPSSDKSEIVL
                     GELWVTYEIQLIKPKLSSLNSRTVASAFFRQESSTPSTSAYFGTLSLVEHSTKNNVDV
                     ELGNTTILFPQQAACSYLITYMVSGTSNALVVLPTFTATSTGEGTFVTLSTANDPFDG
                     NMTAPGSGQSSNRMMISFIAHLDGLGDGLITLSGGTFPATAINAYLYITRVVSGMSLG
                     SVILPAEYDEEEWAPQQRLSHRPDSVFFHDEKDIETPKSSPVDEGEWEDMSKSTMSSI
                     QQIVERSKAKKALLDKISSAKQ*"
ORIGIN
        1 ttaatagcaa ttaaatactt gaggtgaggg tacaactggt accttaacct cattaacctt
       61 cattacacta ataggaacag aagaattaat aatagcggag ctattattaa tcagaacgtc
      121 agcagctata gtattacctg ctgacttctg ttctgttcca ccattattaa tattaattaa
      181 ttgaggacta cccacgaagt gtctaatctc agtgatgcga cgcgtcactt gagatatgtc
      241 ctcttccgtt ctaccttcat ataactcctc gggacggaaa ggacaagtta taataataac
      301 ttcggccatt agttctctag aaccaccctt acattccacc ttgaaggggt acctatcaag
      361 aatgcgtagc agctcatgga aggtacaaaa atcctttcta aaatcatcaa taaggacatg
      421 tttatgtcca tcataactgt cccaccactt cagatttttg cctgataacc aacaatcggg
      481 gtatagctct atagctgtac gcgtttttcc agtgcctgta gggccccagt accaaagaat
      541 acgaggagga ttcgaaggat cccgaggtgt ttctttgtat ttcttcaatc cttcggcaaa
      601 gcgtagctgc tgaaggttgt taatataccc ttcatcaatc atttcattca tactcatatt
      661 ctctaataca caatctttaa catcgtcaat gtcaccacgt gctccttgac gaggaggttt
      721 accaaattcg acaaatttac tatccttctt acaataatca gaagcttgct tccaactacc
      781 tctacgtttt tcgacatgcg ccaagttttc gaaaatctta acaattccac cgaaggtgtg
      841 aggattgaca aagtcaacat atccctggat atgcggggta ccgttctcac caacttcatc
      901 acccatagca acatatctag tattcttgcc ttgcaaaata actagccatt cttccttagt
      961 gcgtaccggg ttgttaatag taaagcacca attacgcgtc ttgctcttgt cacctttatc
     1021 atgaccagct tgaggaatgt gttcccactt ttccttacca ctaccaccag tttccataaa
     1081 gagtttacca ttaacgatct tagttctagg ctcaccattt tccgcaggaa taataggagc
     1141 attatgaaaa tcttcattac cattacctat tttacctata ctatcgacta cactcttaat
     1201 aagagtagga ttaggccgag cgatgggatg agcgatggtt ttgcgagttg ttaacattgc
     1261 aattaatttt ggaacggaac ggatgtcttt aggccctatt atacctctat taattttact
     1321 aaaggcctaa agacatccat attattatac taataataaa taaataaata aatagtctaa
     1381 agtaaaaata tttatacata taaataccta actataaatg ccacgtagag ttcttcttga
     1441 tcctgcctac gtctattatt gtcgggccaa aggctatacc gacaagcaaa ttgtctccca
     1501 taagaacaag caagctaaag ctgatggaga ctctattagc ttttacatac ataagagtaa
     1561 taaagcccct gtccctttta agcgtacgta caaacgtact tataaacgct atggcggtta
     1621 ttaaaattat tagtaataat actaataaat aaataaataa atataaaata aaaaatatat
     1681 atattactaa atactaccat taacaacaat atggttcaat acactgctgc tcaaagagct
     1741 gcctatggca gaaagatggc taaacttcgt gctgcccgtc ccgtttatcg taaaagtaat
     1801 tattctgccc ctaggtctac gcgccgtgtt cgcggttatg gatcttatgc tgaggactgg
     1861 caaaaggtcg gtactgaatg gggaggcaag gttggaaacg ttgccggggg tatagctgat
     1921 ttagctactg gctttgaaaa agctgtatca ggattcggtt cctataaggt taaacgaaat
     1981 tctctccttt ctgaaggtaa cgatccccct gagattcgta atgctaagga tggtcgattt
     2041 attattcgcc accgtgaata tctaagagat attgttgctc cttctgttgg tgtgactagc
     2101 acagctttca atcttgacag gtttaatatc caacctggag attctagttc ttttccgtgg
     2161 ttggctactc ttgctcaaaa cttcgagcaa tatagcttac gaggtgctat ttacgagttc
     2221 aagtctactt atagcgattc ttcagtcgcc ggcagccttg gtactgttgt catggctact
     2281 gagtacgatt ctaccagacc tgacttcctc aataaagctg cgatggagaa tcaccaattc
     2341 gctaattctt gtaagccttc tcattcaatg atgcatatta ttgaatgtgc caggagccaa
     2401 actcctgtaa gtgaattata tatccgtgat gatgaaattc ctgtcactgg tgaccaacgt
     2461 ctatatgact tcggaacctt ccaaattgct tcggttggtg tccctaaccc atctagtgat
     2521 aaatctgaga tcgttctcgg agaactgtgg gtaacttatg agattcagct tattaagccc
     2581 aagctttctt ccctcaactc tcgtacagtt gcctcagctt tcttccgtca agagagctcc
     2641 accccctcta cctcagctta ctttggtaca ttgtctcttg tagaacattc tacgaagaac
     2701 aatgtagatg ttgagcttgg aaacacaacc atcttgttcc cacaacaagc tgcttgttct
     2761 tatttaatta cttacatggt aagtggtact tctaacgcat tagttgtact tcctacattt
     2821 actgctactt ctactggaga aggaacattc gtcaccttgt ctactgcaaa tgacccattt
     2881 gatggtaaca tgactgctcc tggttccggt caatcttcaa atagaatgat gatatcattt
     2941 attgctcact tagatggtct cggagatggc cttatcactc tcagtggtgg tacattccca
     3001 gccactgcta ttaacgctta cctttacatt acccgtgttg tttccggtat gtcgttaggt
     3061 agtgtcatac ttccagcgga atatgatgaa gaggaatggg cgccccaaca gcgactcagt
     3121 catcgtcctg attcagtctt ctttcatgat gaaaaagata ttgagactcc taaatcatct
     3181 ccagttgacg aaggagaatg ggaagatatg tcaaagtcta caatgagttc gattcaacaa
     3241 atcgttgagc gttccaaagc aaagaaagcc ttactagata agatttcttc tgccaaacaa
     3301 taaacaacaa tatatctata aactagatat attatcatac acacactcac acactatcac
     3361 ataactcatg tatacttcgg tatgaccccc tcgccgggag gcgcccccgg agggtccatc
     3421 aagtcctacg tgaagaaata taataatgga gggcaacgtt ggttggccga cattatttat
     3481 ttatggagcg gcaccactat actgtgcatc ctgttcatta tatgtacact atacatacaa
     3541 tattagtat
//
LOCUS       CruV-298                3551 bp    DNA     circular     24-FEB-2020
DEFINITION  Crucivirus-298 isolate AZ_lake_25994.
ACCESSION   CruV-298
KEYWORDS    .
SOURCE      Crucivirus-298
  ORGANISM  Crucivirus-298
            Unclassified.
REFERENCE   1  (bases 1 to 3551)
  AUTHORS   de la Higuera,I., Kasun,G.W., Torrance,E.L., Pratt,A.A.,
            Maluenda,A., Colombet,J., Bisseux,M., Ravet,V., Dayaram,A.,
            Stainton,D., Kraberger,S., Zawar-Reza,P., Goldstien,S., Briskie,J.,
            White,R., Taylor,H., Gomez,C., Ainley,D.G., Harding,J.S.,
            Fontenele,R.S., Schreck,J., Ribeiro,S.G., Oswald,S.A., Arnold,J.M.,
            Varsani,A., Enault,F. and Stedman,K.M.
  TITLE     Direct Submission
  JOURNAL   Submitted (24-FEB-2020) Biology, Portland State University, 1719 SW
            10th Ave, Portland, Oregon 97201, USA
COMMENT     Bankit Comment: ALT EMAIL:ide@pdx.edu
            Bankit Comment: TOTAL # OF SEQS:451
            Bankit Comment: GAP: est. length - alignment

            ##Assembly-Data-START##
            Assembly Method       :: MetaSPAdes v. 3.12; IDBA-UD v. 1.1.1;
                                     MEGAHIT v. 1.0.3
            Sequencing Technology :: 454; Illumina
            ##Assembly-Data-END##
FEATURES             Location/Qualifiers
     source          1..3551
                     /organism="Crucivirus-298"
                     /mol_type="genomic DNA"
                     /isolate="AZ_lake_25994"
                     /isolation_source="Water"
                     /country="USA"
                     /collection_date=2012
     CDS             1..828
                     /codon_start=1
                     /product="Putative Replication Associated Protein"
                     /translation="MAARRYCWTLNNPTDDERRRIRSIDGDNLVGYIICGNEVGQSGT
                     PHLQGYLELKSPQRINRVKRILGVDRVHLEAARGTPTQAAEYCKKEGDTFHEAGAISR
                     QGKRNDLLTLQESIDGGASVMDIAREHFGSFIKYGKGIRGYLALHVQPRDWRTQVVWW
                     HGPTGTGKSRRAHTESQNLCPNSVCWLPDQTLTWFDGYEGHKGVIIDDFDGRAPIAQL
                     LRLFDRYPLKVAIKGGFVEWVPRIIWVTSNKCPEDMYGDHHQWDALRRRIDEIDEIN*
                     "
     CDS             complement(859..1419)
                     /codon_start=1
                     /product="putative ORF"
                     /translation="MSKRSRLQQQAGAASGYTRAELIAAERARQRRVAWDDVAEARYR
                     VEAEALFWSDDVKQREQYWEDHPIIGDDTMGHFTYGYMYDHYPREISRVANALMVPPA
                     ERFNNYSRGWLVVLRGWYATYNEAYRPAVIASLSNFPGLSNSVDVIMEYLCMPFDMFL
                     VKMFRTLKPRRAPPGQSRSRYRYVSI*"
     CDS             complement(1437..3011)
                     /codon_start=1
                     /product="Putative CruV Capsid Protein"
                     /translation="MEPNVPRPDYPRQRKRPVYKSDTSKLRYISGKQKWFAKAYRNTD
                     PNDPYRQMGRQLMGNTYRGANTAQKVVRNALQYYGPGDYRNVVKWIPRGIGAAVGGAL
                     GWQGAGVGGISGGMKSGWDTGAGWSKWMGWGDYAPNGSGHGHTSVNQIMGGGPPLSVN
                     STGDYSGDIIMQRTEFVSNISCSTGVGQTVTPYQSTAFEINPGLSSTFPWLSQIASNF
                     ELYDWEGLMFMYRPTSGEFGASAVSNALGKVVMATKYGVTQFIPFQSTVEAQNYDYAN
                     STKPSLSMVHGVETKNSSQVGGDMLVIRDDIVPNTSNIVLYDLGRLFVMTEGIPLAAS
                     NTAIIGELWVTYKIRLSRAKLWNALSENFSYSRGQWRRNLTQFIIPISWQKRDSGAFT
                     LSRTNISASVGDFTVTNSSPTFLNTTFMVLIYGYDETVIPQAICFDSNPAVTNCTRVD
                     NGITLAGPNNAWNGPDVGVGSSVRGHHITWFLKVTNPNLPFTFRVSTNQVGFPDAAAY
                     QILVVSIDNNVVDSIV*"
ORIGIN
        1 atggccgcaa ggcgatactg ctggacgctg aacaacccta ccgatgatga aagacgacga
       61 atacgaagca ttgatggaga caacttggtc ggctacatca tctgtggcaa cgaggttggg
      121 caatctggaa cgccacatct tcagggctac ctggaactca agtcacctca gcgaattaac
      181 agagttaaga gaatacttgg agtggatcga gtccatttgg aagcagctcg tggaactccc
      241 actcaagccg cagaatattg caagaaagag ggtgacacgt ttcatgaagc cggagcaata
      301 tcacgacagg gaaagcgcaa tgatctactt acattgcaag agtctatcga cggcggcgca
      361 tctgttatgg atattgctcg agagcacttt ggatccttca tcaaatacgg taaaggcatc
      421 agaggttacc tcgctttaca tgtgcaacct cgagattgga gaacacaagt cgtctggtgg
      481 catggcccta cgggtaccgg caaatcaaga agggctcata cagaaagtca aaacttatgc
      541 cctaactcgg tttgctggtt gccagaccag acacttacgt ggtttgacgg atacgaagga
      601 cataaaggag ttattattga tgattttgac gggagagcac ctattgctca gctcttacga
      661 ttgtttgaca gatatcctct aaaggtagcc atcaagggag gttttgtcga gtgggtaccg
      721 aggattatct gggtaacaag caacaagtgc cccgaggata tgtacggcga tcatcatcaa
      781 tgggatgccc taagaagacg tattgatgaa atcgatgaaa taaactaatg tcttacaccg
      841 caagcggtgc gctttgtatt agatagaaac ataacgataa cgagacctag attgacctgg
      901 cggtgctctc ctgggcttca atgtcctaaa catcttaact aaaaacatgt cgaatggcat
      961 gcataaatat tccataataa catcgacact gttagaaagc cctggaaaat tggacaatga
     1021 cgctataaca gcagggcgat acgcctcatt gtatgtagca taccaacctc ttaaaacaac
     1081 taaccaccct ctagaataat tattaaatcg ctccgcggga ggaaccatta aagcattcgc
     1141 taccctagat atctccctag gataatggtc atacatatat ccatacgtaa aatgacccat
     1201 agtatcgtct ccaataatcg ggtgatcttc ccaatactgt tcgcgttgtt taacatcatc
     1261 cgaccaaaac aatgcttcag cttcaacacg atacctagct tccgcaacat catcccatgc
     1321 aacacgtcgt tgacgagctc tttcggcagc aataagttca gctctggtat aaccactagc
     1381 agcgccagct tgttgttgta atctagatcg tttcgacatt aggcaaaatg ttttatttag
     1441 acgatagagt caaccacatt attgtcaata gaaacaacca atatctgata agccgctgcg
     1501 tctggaaaac ctacttgatt agtactaaca cggaatgtaa acggaaggtt cgggtttgta
     1561 actttcaaaa accaagtaat atgatgaccc ctaacagaag agccaacacc aacatcgggg
     1621 ccattccagg cattattcgg tccagccaat gtaattccgt tgtcaacgcg ggtacaattt
     1681 gtaacagcag gattagaatc gaaacaaatt gcctgcggaa tgacagtctc atcgtaacca
     1741 taaatcaaaa ccataaacgt ggtgttcaag aatgtgggac tagagtttgt aacagtaaaa
     1801 tcaccaacac tagcgcttat gttagtacga ctaagagtaa aagcaccgct atctctcttc
     1861 tgccaagaaa taggaataat aaactgggtc aaatttcttc tccattgtcc acgactatat
     1921 gaaaagtttt cagacaaggc attccacaac ttcgcgcgac ttaatctaat cttgtacgtt
     1981 acccataatt ccccaataat agcagtattg ctcgctgcta agggaatacc ttcggtcata
     2041 acaaataatc gaccaagatc atacaaaaca atattagacg tgttgggaac aatatcatct
     2101 ctgataacaa gcatgtctcc accaacttga ctagaattct tggtctctac accatgtacc
     2161 attgataaag aaggctttgt agaatttgca taatcatagt tctgtgcttc tacggtactt
     2221 tgaaaaggta taaactgtgt aacaccatac ttcgtagcca taacaacctt acccaatgca
     2281 ttgctgactg cagaagcacc aaattctcca gaagtaggac gatacataaa cataaggcct
     2341 tcccaatcat ataattcaaa attgctcgca atctgagata accaaggaaa cgtactggat
     2401 aaaccaggat tgatttcaaa cgcggtgctt tgatagggag taacggtctg accgacacca
     2461 gtagaacatg aaatattgga tacaaactca gtacgctgca taataatatc cccactgtaa
     2521 tcacctgtag aattcacaga caatggcgga cctcctccca taatttgatt gacactagtg
     2581 tgaccatgac ccgaaccatt aggagcataa tctccccatc ccatccattt actccagccg
     2641 gcaccagtat cccaaccaga tttcatacca ccggaaatac ccccgacacc agcaccttgc
     2701 caacctaagg caccaccaac tgctgcacca attccacgag gtatccattt tactacgttg
     2761 cggtaatctc ctggaccata gtattgaaga gcgttcctaa ctactttttg agcagtgttg
     2821 gcacctctat atgtgtttcc cataagctgt ctacccattt gtctataagg atcgttgggg
     2881 tctgtgtttc tatacgcttt cgcaaaccat ttttgtttac cagaaatata tctaagttta
     2941 cttgtgtcgc ttttgtatac aggacgcttt ctttgccgcg ggtaatcagg tctaggtacg
     3001 ttaggctcca tcactaacct ttactttctt gtttggtgtt gtttggctaa atgacttgat
     3061 atcgtcgtcg tcgtctgaag aaacttcaac ataaaccttg tacttcttgt ccgaccctga
     3121 cataacagcg acaatacctt tagtaatcgg gtcttgacat atgatacatt tctcctcctc
     3181 ctcttctctt ggttccgcat cgccgtccga aagtaactcg ttccacgcag tgatgtcaat
     3241 aggatacgac atactgaaag aatgactgag caaagaagca tgctccctta aatacacaaa
     3301 aaatatatgt cacatgagca cacaaccgtt acacgatcct gcagaggttt cccactggtg
     3361 caaccggcca acactgtaca gtcaaaaaaa atttatacta tggtacccaa ctggtgcaac
     3421 cggccaacac atacacattt cgggatgggg agggggttgt aacaagtaac gaagtggaca
     3481 gggtaatact ggaaacttcg tttcacctgt ccactattta agcgctatgc cacaagcagc
     3541 tattcacaag c
//
LOCUS       CruV-299                3552 bp    DNA     circular     24-FEB-2020
DEFINITION  Crucivirus-299 isolate 120_1265_IR2_3297.
ACCESSION   CruV-299
KEYWORDS    .
SOURCE      Crucivirus-299
  ORGANISM  Crucivirus-299
            Unclassified.
REFERENCE   1  (bases 1 to 3552)
  AUTHORS   de la Higuera,I., Kasun,G.W., Torrance,E.L., Pratt,A.A.,
            Maluenda,A., Colombet,J., Bisseux,M., Ravet,V., Dayaram,A.,
            Stainton,D., Kraberger,S., Zawar-Reza,P., Goldstien,S., Briskie,J.,
            White,R., Taylor,H., Gomez,C., Ainley,D.G., Harding,J.S.,
            Fontenele,R.S., Schreck,J., Ribeiro,S.G., Oswald,S.A., Arnold,J.M.,
            Varsani,A., Enault,F. and Stedman,K.M.
  TITLE     Direct Submission
  JOURNAL   Submitted (24-FEB-2020) Biology, Portland State University, 1719 SW
            10th Ave, Portland, Oregon 97201, USA
COMMENT     Bankit Comment: ALT EMAIL:ide@pdx.edu
            Bankit Comment: TOTAL # OF SEQS:451
            Bankit Comment: GAP: est. length - alignment

            ##Assembly-Data-START##
            Assembly Method       :: MetaSPAdes v. 3.12; IDBA-UD v. 1.1.1;
                                     MEGAHIT v. 1.0.3
            Sequencing Technology :: 454; Illumina
            ##Assembly-Data-END##
FEATURES             Location/Qualifiers
     source          1..3552
                     /organism="Crucivirus-299"
                     /mol_type="genomic DNA"
                     /isolate="120_1265_IR2_3297"
                     /isolation_source="Water"
                     /country="Norway"
                     /collection_date=2011
     CDS             1..1518
                     /codon_start=1
                     /product="Putative Replication Associated Protein"
                     /translation="MPKLTIPLKGTRPRPAAMPAGPANVQLKKSTEYTEKRKTFKHPG
                     GKYRGFVFTNFKMDWDVMECKEWTNVVFLSIGLEKCPSTGTPHHQGAVYFKNQRSSSG
                     FSKLLPPNCWMEPAYGDEFSNEKYTQKESRLLIIGAPMAQGERNDIEGIRERIKNGAT
                     PADLIAEAGITYNDMKFAEMAFKYLGKPPVRNWKPSVVWLWGESGRGKTYIAKKLLEG
                     DDYWKSSKDLTFWNGYVGQESVWFDEFRGCKTTFNDLLDILGDSDYECNIKGSSQQLF
                     ARRIIFTSPMHPKDVYPGLTESFNQLKRRIDKIINIDTMYTKVDQGFKYAPLAEFPKF
                     NEILDLCAKSRKVTENPVCEEDGDLVELTGNCWIPKVDQSDYVTDEPARMVVKTESYR
                     GRAPNTIPGVSGSEVGGNIGPIGTTSLHDSGRAAPTSSSADIKCSIEHTPEEFCADCV
                     LADECSHEDVFDDGFGNTLCYACGESIIEEFEPGAEPELEINDEFGEVQICSPLR*"
     CDS             complement(1655..3061)
                     /codon_start=1
                     /product="Putative CruV Capsid Protein"
                     /translation="MPRYADEKLPAYKRGLIRKPVYTRAAPVRRAAPLRRAAPRNGGA
                     TNATIAMRSARRAGKSDMGSMLGGLAGMALGGPTGAMIGNAVGGIGGNVLSRIFGSGD
                     YGVTNANSLKKNNIVLSNSANIPKFGTGKVATKFVHREFLGDVFSSATANTFDIKSYS
                     INPGVAETFPWLSGVLGAKYQQYRINGCVFEFRSMSADALNSTNTALGSVIMSTDYDS
                     ADVVFSSKQQMENTENGVSSKPSVNMMHGIECARSQTPVNELYIRAYDVPAGKDIRLY
                     DLGRFSIATTGCQGTSVNLGELWVTYDIDAFKAIEQPPQFLTSMAQYTLNAVDATHAL
                     GTSQTEVVDQIGLTFEYTAGTAYVVFPYDMPVGTIFSFYYGVTGASTIGVATGDVPVE
                     SGGLAIWQGYCATPYPGPTATADLRSFYTTYRYDGSGTPAAPPKLTVAGGVVPTAITV
                     AKLTVSMMSALLPQDGLA*"
ORIGIN
        1 atgcccaaac taaccatacc actaaagggt actcgcccaa ggcctgcggc aatgccagca
       61 gggccggcaa atgtacaact taaaaagagt acagagtata ctgaaaagcg caagactttt
      121 aagcatcctg gtgggaaata tcgcggattt gtattcacga atttcaaaat ggattgggac
      181 gttatggagt gtaaagaatg gaccaatgtg gtcttcttat ccataggact tgaaaaatgc
      241 ccaagcacgg gcacaccgca tcaccaaggt gctgtttact tcaagaacca acgtagttcg
      301 tccggattta gcaagcttct cccacccaat tgctggatgg agcctgctta tggtgatgaa
      361 ttctcaaatg agaagtatac acaaaaagag tcgagactct taatcattgg cgcgccgatg
      421 gctcaaggtg aacgcaatga tatcgaaggt attcgagaac gcattaaaaa tggcgcaact
      481 cctgcggact tgatagctga agcaggcata acttacaatg atatgaagtt tgctgaaatg
      541 gctttcaagt acctcggtaa acctccggta cgtaactgga aacctagtgt ggtttggtta
      601 tggggagagt ctggtcgagg caagacttac atcgcgaaga aacttctaga aggcgacgat
      661 tattggaagt catctaaaga tttgacattc tggaacggct atgtgggaca agaatccgtt
      721 tggtttgatg agtttcgcgg ctgcaaaacc accttcaatg atctcttgga tatccttggt
      781 gacagtgatt acgaatgtaa catcaaaggc tcgagccaac aattgtttgc tcgccggatc
      841 atctttacat ctccgatgca tcctaaagat gtatatccgg ggttgactga aagcttcaat
      901 caacttaaac gtagaattga taaaatcatc aacatcgaca ccatgtatac taaggtcgat
      961 caaggtttca agtatgctcc cttggctgag tttcctaaat tcaacgagat tctggacttg
     1021 tgcgccaagt caagaaaagt gacagaaaat ccggtatgcg aagaagatgg cgatctcgtc
     1081 gaattaaccg gtaactgttg gatccctaaa gtggaccaaa gtgattatgt cactgatgag
     1141 ccggcgcgta tggtggtcaa gaccgagtcg tatcgcgggc gcgctccgaa caccatcccc
     1201 ggtgtttccg gctcagaagt cgggggtaat ataggcccta ttggcactac atcattacat
     1261 gattccggcc gggccgcccc gacttccagc tccgctgata taaaatgctc tatcgagcac
     1321 actccagagg agttctgcgc ggactgcgtc cttgctgacg aatgctcaca cgaagatgtc
     1381 ttcgatgatg gattcgggaa cactttgtgt tatgcttgtg gagaaagcat tattgaagag
     1441 ttcgaacccg gagcggaacc agaactcgaa atcaatgatg aatttggcga agtccaaatt
     1501 tgtagtccgc tgcgctaacc tacaataata aggtccacaa aaaatgtttt tggtacctct
     1561 acgataatga acaaggcgcg acctcttccg cggtctcttt ttctgtatcg aagatatcta
     1621 catactcgaa atcgggaagg gggccaatcc ccatttatgc gagtccatcc tgcggcaata
     1681 aagcactcat catgcttact gtcagctttg ccacagtaat tgctgtagga actacacctc
     1741 cagctacggt cagctttgga ggcgcggctg gtgtaccaga cccatcatat ctatacgtgg
     1801 tatagaatga tcgaagatca gctgttgctg ttggacctgg atatggcgtt gcgcaatatc
     1861 cttgccatat tgctaacccg ccagactcaa cgggaacatc accggtggct acacctattg
     1921 tggatgctcc tgtaactccg taatagaacg agaatattgt gcctaccggc atgtcgtacg
     1981 gaaatacgac ataagctgta ccagctgtat actcgaacgt caaccctatt tggtcgacaa
     2041 cctctgtttg agaggttccc agcgcatgcg ttgcatccac tgcattcaga gtgtattgag
     2101 ccattgatgt gagaaactgg ggcggctgct cgattgcttt gaatgcatcg atgtcatatg
     2161 tcacccataa ctctcccaga tttacagaag tgccttggca ccctgtagtg gcgattgaaa
     2221 atcgtcccaa atcataaaga cgaatatcct tgcctgccgg cacatcatac gctcggatat
     2281 acagctcatt cacaggcgtt tgagagcgtg cacattcgat gccatgcatc atgttcaccg
     2341 atggcttact agacactccg ttttctgtgt tctccatctg ctgtttagat gagaagacaa
     2401 catctgcaga atcatagtcc gttgacatga tcactgaacc caaagcggta tttgttgaat
     2461 tcagggcatc tgctgacatt gatcgaaact caaagacaca gccattgatt cgatactgct
     2521 ggtacttcgc accaagaact ccagacagcc acggaaatgt ttccgctact cctggattaa
     2581 tagaatagct cttaatatca aaagtgtttg ctgttgcaga actaaacaca tcacccaaga
     2641 actcacgatg cacgaacttg gttgctacct tgcccgttcc gaacttcgga atgtttgcag
     2701 aatttgaaag aacaatgttg ttcttcttca aggaatttgc gttagtcaca ccataatctc
     2761 cgctcccgaa gattcttgat aacacatttc ctccgatacc gcctacagcg ttaccgatca
     2821 tagcaccagt ggggccacct agtgccatac ccgcaaggcc tcccaacatg gaacccatgt
     2881 ctgatttgcc tgctctacga gcgcttcgca ttgcgattgt tgcattcgta gcgcctccat
     2941 tacgtggagc cgctcgcctc aacggtgccg cacgccttac aggagcagca cgagtgtaca
     3001 cgggctttct tatgagtccg cgtttgtacg ctggcaactt ttcgtccgcg taccgaggca
     3061 taatcgatcg ggtatactac ctgattattt tcctctaatt tattattata tgctgcatgg
     3121 ctctgcagcc atcgcacatc tctcacatgc caccactcta gctctgccat cgtcggcatg
     3181 attattaaaa gaacttatct tttatttttc gtaatatggg cggcacttcg tacctcccgt
     3241 caagctctat actaggggcg tcgcttccat attggtctgc tcgctacgca cctgttgctt
     3301 aaattaagtc tttaaacctc ttcccctgtg ggggttaacg tggctttaaa cactgattga
     3361 ggttgccccc taaagggggc aacgcaataa gcgtgtctag tcctgttagc ttctgcagca
     3421 agacgttgct tgccttaccg ttacactata cgcaccaagt gtgtaggtcg gaccacgttg
     3481 tttgctcctg cactctagac atgggataat tttttatttt aaagaatatc ccggctcggt
     3541 atactttgag ct
//
LOCUS       CruV-300                3558 bp    DNA     circular     24-FEB-2020
DEFINITION  Crucivirus-300 isolate Ga0132255_100115160.
ACCESSION   CruV-300
KEYWORDS    .
SOURCE      Crucivirus-300
  ORGANISM  Crucivirus-300
            Unclassified.
REFERENCE   1  (bases 1 to 3558)
  AUTHORS   de la Higuera,I., Kasun,G.W., Torrance,E.L., Pratt,A.A.,
            Maluenda,A., Colombet,J., Bisseux,M., Ravet,V., Dayaram,A.,
            Stainton,D., Kraberger,S., Zawar-Reza,P., Goldstien,S., Briskie,J.,
            White,R., Taylor,H., Gomez,C., Ainley,D.G., Harding,J.S.,
            Fontenele,R.S., Schreck,J., Ribeiro,S.G., Oswald,S.A., Arnold,J.M.,
            Varsani,A., Enault,F. and Stedman,K.M.
  TITLE     Direct Submission
  JOURNAL   Submitted (24-FEB-2020) Biology, Portland State University, 1719 SW
            10th Ave, Portland, Oregon 97201, USA
COMMENT     Bankit Comment: ALT EMAIL:ide@pdx.edu
            Bankit Comment: TOTAL # OF SEQS:451
            Bankit Comment: GAP: est. length - alignment

            ##Assembly-Data-START##
            Assembly Method       :: MetaSPAdes v. 3.12; IDBA-UD v. 1.1.1;
                                     MEGAHIT v. 1.0.3
            Sequencing Technology :: 454; Illumina
            ##Assembly-Data-END##
FEATURES             Location/Qualifiers
     source          1..3558
                     /organism="Crucivirus-300"
                     /mol_type="genomic DNA"
                     /isolate="Ga0132255_100115160"
                     /isolation_source="Soil"
                     /country="USA"
                     /collection_date=2009
     CDS             1..1173
                     /codon_start=1
                     /product="Putative Replication Associated Protein"
                     /translation="MPQIVSPLINNLINPNTAAVQDSRCCVWDATIGETINNVVVEPA
                     TIKAWMEKNCKKWCFQLEKAPNTGWRHYQCRFSLKEKARIKQVFDLLDADDLTGHLSV
                     TSKENRDNHFYVCKDTSRVDGPWMDSDTNYYVPRQVRSIKEWLPWQQTLMTILTVWDD
                     RGIHVVFDPDGGIGKSTFCTFMGSTGKAVKIPYCNDFRDINRIVMDKPKAGGYLMDMP
                     RASSKDKLFQMYAALESLKDGYAYDDRYKFRDMYFDSPNIIIFTNKMPDAGLLTRNRW
                     HVWKVNDEQELDDITAELDVREPRRQIDKSKIDIVSNIDSNGFLTISRKEKPGSPLES
                     RTVDIVYDKTGHIGISNNMPVESISLSDEIKAISELAALKAEVAKQASNPPPTNNM*"
     misc_feature    1153..1181
                     /note="stem-loop"
     misc_feature    complement(1160..1168)
                     /note="nonanucleotide"
     misc_feature    1161..1169
                     /note="nonanucleotide"
     CDS             complement(1643..3277)
                     /codon_start=1
                     /product="Putative CruV Capsid Protein"
                     /translation="MSKRYSPAQKAAIAAAYARKAQYGSNKYYKKPAYKKPAYNKYAN
                     YKSVKGYGKYTGMGSRIGSVLGEGAERLIEFGGGLASVLGFGDYVQPGFRVQKNSFLN
                     GGNDPPTIKNAKDGRFILRHREFLRDVITGAANAFNLVSIPINPGIVTSFPWLAAVAQ
                     AFEQYKLRGMIFEFKSTSADALSSTNTALGTVVMATEYDSTRPNFANKQQMENHQFAS
                     SARQSSSMYHPIECDRSQTPISELYVRTGSPSDSTDNDLDLRLSDWGNFQIATTGQQA
                     ANVNIGELWVTYEVEFLKPCINENGPIQLASFYRSDDSSYTTTNYLGNPATQKANTGN
                     NIGMDLSVGNIIRWPKGTYGCFLCLIEWTGSALVNQTIPTFAGSIITEGVTLGATISP
                     FSTVLSAPLNTEQASKMVVAFLVQCYGQTATGAGPGVVISGGTLPSGPLGVKFYVASV
                     MDGLINANNPPLPEELEEDEEFQKYLAEEVAKLKLNYIESHTPQQIEDTPTPKSEVKE
                     EDLIDKFSGMDSSQLLGFLQKVKEHKTSQQTGSTSK*"
ORIGIN
        1 atgccccaaa ttgttagccc ccttatcaac aacttgataa atcctaatac tgcagcagta
       61 caagacagca gatgctgcgt gtgggatgca acaatcggcg aaacaattaa caatgttgtc
      121 gtcgaaccag ctaccattaa ggcctggatg gaaaagaatt gcaagaaatg gtgcttccaa
      181 cttgaaaagg cccctaatac tggctggcgt cactaccaat gtagattctc tcttaaagag
      241 aaagctagaa tcaaacaagt atttgattta cttgatgctg atgacttaac cggtcatcta
      301 agtgtgactt ctaaagaaaa tcgcgataac cacttctacg tctgtaaaga tacttctcgc
      361 gttgacggac cttggatgga tagtgatacc aactactatg tccctcgtca agtccgcagt
      421 attaaggaat ggctaccttg gcaacaaaca ctaatgacaa ttttaactgt ctgggatgac
      481 cgcggtattc atgttgtctt cgaccctgat ggaggtatcg gcaaaagtac cttctgcacc
      541 ttcatgggta gcactggcaa agctgtcaag attccttatt gtaacgattt ccgtgatatt
      601 aatcgtatcg tcatggataa acctaaggca ggcggttatc tcatggatat gcctagagct
      661 agcagtaaag ataaactgtt ccagatgtat gccgctcttg aatcccttaa ggatggctac
      721 gcttatgatg atcgctacaa attccgcgat atgtacttcg actcaccgaa tattataata
      781 ttcactaata aaatgcctga cgctggatta ctcacccgta accgttggca tgtctggaaa
      841 gttaatgatg aacaagaact tgatgatatt actgctgaac ttgatgtacg cgaacctcgt
      901 aggcaaatcg ataagtctaa aatcgatatt gtaagtaata ttgactctaa tggattctta
      961 accataagtc gtaaagagaa gcctggaagt cctcttgaaa gtcgaacagt tgatattgtc
     1021 tatgacaaaa ctggccatat tggtatatcc aataatatgc ctgtcgaaag catttcttta
     1081 tctgacgaaa taaaagctat ttctgaatta gctgctctta aagcagaagt tgctaagcaa
     1141 gcttctaatc ctcctcccac taataatatg taagtgggag gctaaaattt attattgcta
     1201 ataataaatg tatttttaac cctggggggt ttttttcggc ggttccctgt aatagtacga
     1261 aaaaaacccg gttcacatcc aacttcgtct ctcaatcttt aatctcaaat tataaaaata
     1321 atgtaatatt atttttaact ttctggacct actggaccta tttgaaagtt tacatacatg
     1381 tcaaccacct ccttcgtcgg tggtgcacgt taatgtttat tcggtgtact gcctccggcg
     1441 tactatacta taatagtagt ctaccagaat gtaaaagaga tataactatt ctcaatatta
     1501 tctccttgac ttatcacaac agtaataaat catatgccta acgtctttct tttttttata
     1561 tgtttttatt tgaatgcctc tctgcatctg tcttattatt gttgtttctt cgtctcttat
     1621 ctaatatagc aaactggaag tattacttag aagtacttcc agtttgctga ctagtcttat
     1681 gctctttcac cttctgcaag aagccaagca actgcgatga atccattcct gaaaatttat
     1741 ctatcaaatc ttcctcctta acctctgatt taggtgtagg agtatcctct atttgttgag
     1801 gtgtatgcga ctcaatataa ttgagcttca acttagcaac ctcctccgcc aaatacttct
     1861 ggaactcctc atcttcctct aactcctctg gaagaggcgg attattagca ttaattaatc
     1921 catccataac tgaagcaacg tagaatttaa ctcctaacgg tcctgatggt aaagtacctc
     1981 ctgaaatcac aactcctgga ccagcaccag tagccgtttg accataacat tgaactaaaa
     2041 atgcaacaac cattttagaa gcttgttctg tattcaatgg agcagaaagt actgtactga
     2101 atggactaat agtcgctcca agagtaactc cttctgtaat aatagaacct gcaaatgtag
     2161 gaatcgtttg attaaccaat gccgatcctg tccattcaat aagacaaagg aaacatccat
     2221 aagttccttt cggccacctt ataatattac caactgaaag atccatccca atattatttc
     2281 ccgtattagc cttctgcgta gcaggattac caaggtaatt agtagtcgta taactagaat
     2341 catcactacg atagaaagat gctaactgaa tgggtccatt ttcattaata caaggtttga
     2401 gaaattcaac ctcataagta acccaaagct ctccaatatt cacattagca gcttgctgcc
     2461 cagttgtagc aatctggaaa ttaccccaat ctgataaacg aagatctaaa tcattatcag
     2521 tagaatctga tggagatccc gtacgaacat ataattcaga aatgggagtc tgcgaacgat
     2581 cacactcgat cggatgatac atcgaactag actgacgagc cgatgaagca aactgatgat
     2641 tctccatctg ttgcttattc gcaaaattag gacgagtact atcatactct gtagccatca
     2701 caactgttcc taatgcagta ttagttgaac tcaacgcatc cgctgatgta ctcttaaact
     2761 caaatatcat ccctcttaac ttatactgct caaatgcctg agctacagcc gctaaccatg
     2821 gaaatgatgt cacaattcct ggattaatcg gaattgatac caaattgaac gcattcgctg
     2881 ctccagtaat cacatcgcga agaaattctc gatgtctcaa gataaacctc ccatctttcg
     2941 cattcttaat cgtcggagga tcgttcccac catttagaaa actattcttc tgaactctaa
     3001 acccaggttg aacataatct ccaaacccaa gcactgatgc aagacctcct ccaaactcta
     3061 ttaaccgttc agctccttca ccaagaactg atcctatacg cgatcccata cctgtatatt
     3121 taccataacc tttcacactt ttgtaattag catacttgtt atatgctggc ttcttatacg
     3181 caggcttctt ataatatttg ttagatccat attgagcctt acgtgcgtat gcggccgcaa
     3241 ttgcggcctt ctgagctgga gaatatctct tcgacatctt aaacttttat ttaataaata
     3301 aataaaaatt attttcaatt ttcatcccaa tcttcatcat accaaaatac aaattcatca
     3361 tatatattca tccttaccca atttggtaaa tattgaagat catctccata ataccacata
     3421 attaatggtg tatcggtatt aacgataaat aaatcttcca tctttttctt ctacaacttt
     3481 cttttttaaa tcttaaaaaa atttttttta tttctaaaaa tctggatcta ttggacctaa
     3541 aataatcaga aacgaaag
//
LOCUS       CruV-301                3560 bp    DNA     circular     24-FEB-2020
DEFINITION  Crucivirus-301 isolate 001415_virusbaton.
ACCESSION   CruV-301
KEYWORDS    .
SOURCE      Crucivirus-301
  ORGANISM  Crucivirus-301
            Unclassified.
REFERENCE   1  (bases 1 to 3560)
  AUTHORS   de la Higuera,I., Kasun,G.W., Torrance,E.L., Pratt,A.A.,
            Maluenda,A., Colombet,J., Bisseux,M., Ravet,V., Dayaram,A.,
            Stainton,D., Kraberger,S., Zawar-Reza,P., Goldstien,S., Briskie,J.,
            White,R., Taylor,H., Gomez,C., Ainley,D.G., Harding,J.S.,
            Fontenele,R.S., Schreck,J., Ribeiro,S.G., Oswald,S.A., Arnold,J.M.,
            Varsani,A., Enault,F. and Stedman,K.M.
  TITLE     Direct Submission
  JOURNAL   Submitted (24-FEB-2020) Biology, Portland State University, 1719 SW
            10th Ave, Portland, Oregon 97201, USA
COMMENT     Bankit Comment: ALT EMAIL:ide@pdx.edu
            Bankit Comment: TOTAL # OF SEQS:451
            Bankit Comment: GAP: est. length - alignment

            ##Assembly-Data-START##
            Assembly Method       :: MetaSPAdes v. 3.12; IDBA-UD v. 1.1.1;
                                     MEGAHIT v. 1.0.3
            Sequencing Technology :: 454; Illumina
            ##Assembly-Data-END##
FEATURES             Location/Qualifiers
     source          1..3560
                     /organism="Crucivirus-301"
                     /mol_type="genomic DNA"
                     /isolate="001415_virusbaton"
                     /isolation_source="Water"
                     /country="France"
                     /collection_date=2013
     misc_feature    join(3549..3560,1..19)
                     /note="stem-loop"
     misc_feature    1..9
                     /note="nonanucleotide"
     CDS             177..1079
                     /codon_start=1
                     /product="Putative Replication Associated Protein"
                     /translation="MPVAMSLSRRYCFTVNNPSEEDCEQVYALSKHKHHKYLIVGSEK
                     GECGTPHLQGYVVFAKPVRFNTFKALIPRAHVEAAKGSSEQNRVYCMKEGDYEEFGDV
                     PTDGGQTLQSCEWDQARANAKLGKFDDIPSNLYIRYKNAFHSIFDDACNASECIVELD
                     HLWITGPTGIGKSRYCFESFPNAYRKGANKWWCHYTNQDVVILEDVEPSQEKWIGYYL
                     KIWSDHYPFIAERKGGSRSIRPKRIVITSNYSIKEIFTDAKIRDPLLRRFKERTIKDG
                     ELIASISQEILFRQIGVDHYEAIE*"
     CDS             1097..2665
                     /codon_start=1
                     /product="Putative CruV Capsid Protein"
                     /translation="MLTRSKRRRSVEAFDPEPYQGPRRRLNALSVEEAVMPEMATGIE
                     QGAGPYYAPPRNLDFYKDKPLDYTTYTALKQQGYTLSKRKKKAATRKRPTRRRRSYSY
                     NGSSSRNLSGMGPIVITGRGGYWGDKFGEGASAAWKSLKRATPEGTFGRLGRAAGGAM
                     FGKPGATVGSWLGGGVSDILGFGDYAVKRNDLMMLDEGVPVPTFQNMNDGVIVCHREY
                     IGDVTQSTNFVNNQFIIQPGDSTTFPWLNQIAHQFEQYEILGMIFQYRSTASDFGTTS
                     NMAMGSVIMATEYDTVDAAYGSKLEMENSQYAMSGKPSHDLIHPIECDPGVVGPMGLK
                     YVRTGPVPTGKDNRLYDHGNFQIATTGMPSASGTIGELWVTYKIAFYKPQFNLGSDIK
                     SDIFFGTAGVASNSPFPVSPQPGNSTGCIVSQGAITFPSNAPVGSKWLISLFVVGAST
                     GGMVVSAFNLAGCTSLYQNSNPSVATTTDRVNQNMLIRITAPGAVVGGNTMTVPGSVT
                     SAQLFITQFDSEAV*"
     CDS             complement(2673..2990)
                     /codon_start=1
                     /product="putative ORF"
                     /translation="MAPLPVLSGSSKTPTIYIDSPGRTDTSAENCTTEETAGRCRSHS
                     EGYATTTSQSDREDSPSVKKKKRPKFTYTWLRLRNHCPDCDYLDSEATQPQSYDVRVR
                     LPK*"
ORIGIN
        1 tagtattacc ccgcccccct gtaacaggcg taatttgcct gttattatat ggggagccct
       61 aatattagct gttacggagg ctgcgccagt gtaatttgtc gatgtaacag ggtttaggtc
      121 ccataatggt tgtatcgtta tgggactcta catagggact aatggcgctt tagctcctgc
      181 cagtcgctat gtcgctttct cgccgttatt gctttacggt gaacaatccg agtgaggagg
      241 attgtgagca ggtttatgcc ttgtcgaagc ataagcatca caagtatttg attgttggta
      301 gcgaaaaggg ggagtgtggt accccgcatt tgcaaggcta tgttgttttt gctaagcctg
      361 ttcgatttaa tacgtttaaa gctttaatac cgagggcgca tgtggaggct gctaaaggtt
      421 cttctgagca gaatcgggta tactgtatga aggagggtga ttatgaggaa tttggtgatg
      481 ttcctacgga tggtggtcaa acgttgcaaa gttgtgagtg ggaccaagct cgtgctaacg
      541 cgaagcttgg aaaatttgat gatattccat cgaatttgta catccgttat aagaatgcct
      601 tccacagtat atttgacgat gcatgtaacg caagtgagtg tattgtcgaa ttggaccatt
      661 tatggataac tggtccaacc gggattggga aatctcggta ttgttttgaa tcgtttccta
      721 atgcttatcg aaagggtgca aacaagtggt ggtgtcatta caccaaccag gatgttgtta
      781 ttctcgaaga tgttgagcca tctcaagaaa aatggatagg atactatctg aagatttggt
      841 ccgaccatta tccttttatt gctgaaagaa agggaggatc gcgcagtatt cggccgaagc
      901 gaatagtgat aacgtcaaat tatagtatca aggagatttt tactgatgcc aagattcgag
      961 atccgctttt gcgtcgtttt aaagaacgta ctatcaagga tggggaatta atagcgagta
     1021 tttcgcaaga gattttattt cggcagattg gtgttgacca ttacgaagct attgaataaa
     1081 gtgcgtattc gcatttatgt taactcgttc taaacgtcgt cgttctgtcg aggcgtttga
     1141 tccggagcca tatcaaggtc ctcgtcgtcg tcttaacgct ctttctgttg aagaagcggt
     1201 aatgcctgag atggctactg gcattgagca gggtgctggt ccttactatg cgcccccgcg
     1261 caaccttgat ttttacaaag ataagccttt ggattatacg acttacactg ctcttaagca
     1321 gcagggatat acgcttagta agcgtaaaaa gaaagccgcc actcgcaaaa gacccacccg
     1381 tcgtaggcgc agttactctt acaatggatc ctcttctcgt aatctctctg ggatgggtcc
     1441 tattgttatt actggccgtg gtggttattg gggtgataag tttggcgagg gggcatctgc
     1501 agcttggaaa tctttgaagc gtgctacgcc agaaggcacg tttggacgtt tgggtcgtgc
     1561 cgctggcggt gctatgtttg gaaagccagg agcgactgtt ggtagctggc ttggaggcgg
     1621 agtctctgat attttaggtt ttggagacta cgctgttaaa agaaacgatt taatgatgtt
     1681 ggacgaaggt gttcctgttc caacatttca gaatatgaac gatggcgtta tagtttgcca
     1741 tcgagagtat attggtgatg ttactcaatc taccaatttt gttaataatc agtttattat
     1801 tcaacctggt gatagtacca cttttccgtg gttgaatcag attgctcatc agtttgagca
     1861 atatgaaata ttgggtatga tatttcagta tcgttctaca gcctctgatt ttggtaccac
     1921 ttcaaacatg gcgatgggca gtgttattat ggctacagaa tacgacactg ttgatgctgc
     1981 ctatggcagt aaattggaaa tggagaattc ccaatatgct atgagtggta aaccatctca
     2041 tgatcttatt catccaatcg aatgtgatcc cggtgttgtt ggacctatgg gtcttaaata
     2101 tgttcgtact ggtccagttc ctactggcaa ggacaatcgt ttgtacgatc atggcaattt
     2161 ccaaattgcc actaccggaa tgccttctgc tagtggaaca attggtgaat tatgggttac
     2221 ttacaagatt gctttttata agccgcagtt taatcttggt agtgatatta agagcgatat
     2281 tttctttggt accgctggcg ttgccagcaa ttcgcctttc ccggtttctc ctcaaccggg
     2341 aaatagtacc ggttgtattg tttcacaagg tgctataaca tttccttcta acgctccagt
     2401 tggttcaaag tggttgattt cgctttttgt tgttggtgct tctaccggcg gaatggtcgt
     2461 tagtgcgttc aatttggctg gatgcacttc tttgtatcaa aactccaatc ctagtgttgc
     2521 tactacaact gatcgtgtta atcaaaacat gttgattcgg attactgcac ctggtgctgt
     2581 tgttggtggc aatactatga cggtgcccgg atctgtgact tctgcacagt tgtttataac
     2641 tcagtttgat tctgaagctg tttaatataa aattattttg gtagacgaac tcgtacgtca
     2701 tagctctgtg gttgtgttgc ttcactgtct aggtagtcac agtcagggca atgattacgc
     2761 agccggagcc acgtgtaagt gaatttcggc ctcttcttct tcttcactga aggagagtct
     2821 tcgcggtcgg actgggatgt cgtcgttgcg tagccttccg agtgcgagcg acatctccca
     2881 gctgtctctt ctgttgtaca gttctcggcg gaagtatcgg tacggccggg cgaatcgatg
     2941 tagatggtcg gtgttttgga agacccagac aacaccggca acggagccat atgttaccta
     3001 ggcaattcag attgattgta tcagagtgct ctaatccgaa ctgaccattt aaggttcaat
     3061 gttaagcgct gttctgataa ggtaagtatg ggagtttgtc cctcctatat agggaggctc
     3121 cgccacaagt tgtttatggg acctttaaaa ctcattgttg agtttttgtg cgtgactgtt
     3181 cacagtcaga aaaaaagtaa aagtagtttg tgttttctat cgcaaagtaa aggggcctac
     3241 cttgtgtagg gctttatgtt gttgatgctg cgccccgtag gccagtcatc ttgtttttgc
     3301 aaacccctca aacaattagg gcggccgaag gccgggccgc cctaggcggc ctagggccgc
     3361 cgtaggcggc ttccgggccc cgcaggggcc ctttaggggg ttttgggccc gaagggcctt
     3421 taggggtttt tgtgtgtttg gtttttaaag gcttttgcct ttggggcccg tagggccctg
     3481 cgcgccgcag gcggcccccg gcaggggagc gcagcttttg ggccacggag ttttggcgtg
     3541 gcctttaggg ggggcgggtc
//
LOCUS       CruV-302                3563 bp    DNA     circular     24-FEB-2020
DEFINITION  Crucivirus-302 isolate 120_805_SvL1_3295.
ACCESSION   CruV-302
KEYWORDS    .
SOURCE      Crucivirus-302
  ORGANISM  Crucivirus-302
            Unclassified.
REFERENCE   1  (bases 1 to 3563)
  AUTHORS   de la Higuera,I., Kasun,G.W., Torrance,E.L., Pratt,A.A.,
            Maluenda,A., Colombet,J., Bisseux,M., Ravet,V., Dayaram,A.,
            Stainton,D., Kraberger,S., Zawar-Reza,P., Goldstien,S., Briskie,J.,
            White,R., Taylor,H., Gomez,C., Ainley,D.G., Harding,J.S.,
            Fontenele,R.S., Schreck,J., Ribeiro,S.G., Oswald,S.A., Arnold,J.M.,
            Varsani,A., Enault,F. and Stedman,K.M.
  TITLE     Direct Submission
  JOURNAL   Submitted (24-FEB-2020) Biology, Portland State University, 1719 SW
            10th Ave, Portland, Oregon 97201, USA
COMMENT     Bankit Comment: ALT EMAIL:ide@pdx.edu
            Bankit Comment: TOTAL # OF SEQS:451
            Bankit Comment: GAP: est. length - alignment

            ##Assembly-Data-START##
            Assembly Method       :: MetaSPAdes v. 3.12; IDBA-UD v. 1.1.1;
                                     MEGAHIT v. 1.0.3
            Sequencing Technology :: 454; Illumina
            ##Assembly-Data-END##
FEATURES             Location/Qualifiers
     source          1..3563
                     /organism="Crucivirus-302"
                     /mol_type="genomic DNA"
                     /isolate="120_805_SvL1_3295"
                     /isolation_source="Water"
                     /country="Norway"
                     /collection_date=2012
     misc_feature    join(3556..3563,1..16)
                     /note="stem-loop"
     misc_feature    1..9
                     /note="nonanucleotide"
     CDS             336..1712
                     /codon_start=1
                     /product="Putative Replication Associated Protein"
                     /translation="MSQVDDRLLHNSFYCFKFTWNNPPSDAKDCLEAFQVEGLAYTRK
                     TALTDQGLKPFHKVSALIGAFEICPTTNTPHIQGAVRFVDKIYWRQLQARFPGVWFGP
                     ADGKFINHVRYIQGPYESDDGIKKKPINPTFFCFGDLPRQGKRTDLSSLREAMMLGKP
                     FTEICHEHFDTVMKFPSGTRLLYSTLKPAEYGRKVLIELRSWQKKLLSHLSTPYIPRQ
                     IIWIWSLSSTTGKTTFMQHVDSKYVVTTIQEFKDWNGLVHSFRKDTQVVHIDIPRAYT
                     YADKTAETLLEILEKLSDHSAMSTTKYEGKRVYCDAHIVVTSNLPPPRAKLPNRFTEI
                     CLDNNLEEGEIINHITVSDPRYVFNVREIDRTSEFRHVTFKEEPAIPKVTLDAYKQRN
                     YSHDNYIGQSMEASLEESPSESSRSSLGSRGDSNLDVNEEDIHIFGFEPARVSEAAGY
                     PFVRQSTN*"
     CDS             1888..3360
                     /codon_start=1
                     /product="Putative CruV Capsid Protein"
                     /translation="MVISCKTFLHKKYKMPRSIRRSGSVRSSGSRRSARSYRRRAYVP
                     RAYPPQRRTYVPRVMTAPRESRSRSEVIGGQIGKGIDLVGKGAGGIYSMVKQLFGSGD
                     YKIGASPAVNSLFVGSKTPDTINAGFGSKTIRLQHREYLTDVMTSPTANTFQIQTWSL
                     NPGLFSTFPWLNAIANQFQCYKLHGCVLEFKTTSADALNSVNTALGTVILAADYDSVS
                     GPFINKQDMLNSFGAIDCKPSQSCMAGIECDPKRLPLSELYTRSGSASASADIRLYDI
                     CQFSVATTGMQGTSVNVGELYIAYDIEFYMPIQSVVGNTCPGACYQLTAPGITDIAGQ
                     SPFGSAQTALYDNIGMTFNTSVDVGYTSTMCSVDPKIWNSMSNALLLVTYKLYGTTTG
                     GSAAETFDPIAAGGGTTIVQAFPGVAAGEQSIQANQNLAPTGAVQVGLSFFVSVQNLQ
                     SSGGLLFGLQNPVGIGAIVPTAATGVIYISVVNPSAIASY*"
ORIGIN
        1 tatagataaa gtatacagat tagacataat ctagaaaatc aaagatttct tatacgcttt
       61 cttcttagag cgcgaagcgc cgcaagccga gcaaagctcg gcgcgcagtg ctgtgtaaat
      121 atataaaatg aaccccccgt attcgtttct ccattgtggc ctgataatac catcggcttt
      181 agacgatata ttaatcaggc cacatttggt tattgaatac agattatttt atgatcgtca
      241 ataaccatat aaaaactcac agttttttca cgtttaagtt caaaattact atttttaact
      301 tccagttttt ggttttttac ataaaaacta taaaaatgtc tcaagttgat gatcgattgt
      361 tacacaatag cttttattgc ttcaagttta cttggaataa tccaccatct gatgcgaaag
      421 attgccttga agcattccaa gtggaaggcc tcgcatacac acggaaaacg gcattaaccg
      481 atcagggatt aaagcctttc cataaggtct cagctcttat aggtgcattt gaaatatgcc
      541 ctactaccaa tacgccacac atccagggtg ctgttcgttt cgtcgataaa atttattggc
      601 gtcaactcca agctaggttt ccgggcgtct ggttcggtcc tgctgacggt aagttcatta
      661 atcatgttcg atatatacaa ggaccatatg aatcagacga cggtataaag aaaaagccca
      721 taaatccaac attcttttgt tttggcgatt taccacgtca gggtaaacgt accgatttat
      781 cttctctccg ggaagctatg atgttaggta agccatttac tgaaatatgt catgaacatt
      841 tcgacactgt catgaaattt ccatctggga ctagattgtt atattctaca ttaaaaccgg
      901 ctgaatatgg tcgaaaggtt cttattgaac ttcgttcttg gcagaagaaa ttactttctc
      961 atctgtccac accatatata ccccggcaga ttatctggat atggtccttg tcttctacca
     1021 caggaaagac cacatttatg caacatgttg actctaaata tgtcgtaacg actattcagg
     1081 aatttaagga ttggaacggt ttagttcatt cattccgtaa agatacacag gtcgtgcata
     1141 ttgatattcc gcgtgcatat acttatgctg ataaaacagc agaaacacta cttgaaatac
     1201 tcgagaaact tagcgatcat agcgcaatgt ctacgactaa atatgagggg aagcgcgtat
     1261 attgcgatgc ccatattgtc gttacgagta atctaccacc cccacgcgca aagctcccta
     1321 atcgatttac tgaaatttgt ctcgacaata atttagagga gggggaaatt atcaatcata
     1381 taaccgtttc ggatccacga tatgtattta atgttcgaga aattgatcga acttctgaat
     1441 ttaggcatgt tacatttaag gaagagcccg ctattcctaa ggttaccctt gatgcttata
     1501 agcagcgaaa ttattcccac gataattata ttggacaatc catggaggct tcgctagaag
     1561 aatcgccatc agaatcaagt cgttcatctc tgggttcccg tggtgattct aaccttgacg
     1621 taaatgaaga agatattcac attttcggct tcgaaccggc tcgcgtcagc gaggcggcgg
     1681 gatatccatt tgtgcgtcag tctacaaact aatttataga ttatatcgtt aggcgtccgg
     1741 gcaaagcccg gacgctttac cttcaattct atagcttttt tcgttcattt atatgtcaca
     1801 tatatctaaa tatttattaa aatatagatt atatcgttag gcgtccgggc aaagcccgga
     1861 cgctttacct tcaattctat attacatctg gttataagtt gtaaaacttt tttacataaa
     1921 aaatataaaa tgccacgatc aattcgtaga tcaggttctg ttagatcctc aggttctcgt
     1981 cgttcagctc gttcttatcg acgacgcgct tatgtaccgc gtgcttaccc tccacagagg
     2041 cgcacgtatg tcccacgagt gatgactgct ccgagagagt ctcggtctcg ctctgaagtt
     2101 attggtggtc aaatcggcaa aggcattgac ttagtcggaa agggcgccgg tggtatttat
     2161 tccatggtta agcaactttt cggttccggt gattacaaga tcggagcttc gccagctgtt
     2221 aattcgttat tcgtgggttc taagactccg gatacgatta atgcaggatt tggtagcaaa
     2281 actattcgac ttcagcacag ggagtatctc acagatgtca tgacatctcc cacagctaat
     2341 acgtttcaga ttcagacatg gtcattaaat ccggggttgt tttcaacgtt cccgtggttg
     2401 aatgctattg ctaatcagtt tcagtgttac aaacttcatg gctgcgtcct tgaatttaag
     2461 actacttctg ctgatgcgct taattcagta aacacagctc tcggcacagt tattttagct
     2521 gctgattacg acagcgttag cggtccgttt atcaataagc aggatatgct taattctttt
     2581 ggagctatcg attgtaagcc ttcgcagtca tgcatggctg gtattgaatg cgatccaaag
     2641 cgactaccac tttctgaact ttatactcgc tctggttctg cttcagctag tgctgatatt
     2701 cgattgtatg atatctgtca attttcagtc gctaccaccg gtatgcaggg gacatcagtt
     2761 aatgttggtg agctttacat cgcatatgat atcgagttct acatgcccat tcaaagtgtt
     2821 gttggtaata catgtcccgg tgcatgttac cagttgacag ctccaggaat tactgatatt
     2881 gcgggtcaat caccatttgg ttcagctcag actgcgttgt atgacaatat cggtatgacc
     2941 tttaatactt cagtcgatgt cggatatact agtactatgt gtagcgtaga ccctaagatt
     3001 tggaattcta tgagtaatgc tttactcctt gtcacgtaca aattgtatgg taccacaacc
     3061 ggcggctcgg ctgcagaaac ctttgatcca attgcagcag gaggtggaac aactattgtt
     3121 caagcttttc ctggtgttgc tgctggtgag caatcaatac aagctaatca aaatctcgct
     3181 cctactggtg cagttcaagt cggtctttca ttctttgttt ccgtccaaaa tctccagagt
     3241 tcgggaggtt tattgtttgg actacaaaat ccagtcggta ttggtgctat tgtaccaacc
     3301 gctgcgacag gtgttattta catttccgtg gttaatcctt cggctattgc ctcatattag
     3361 acgtacatct cgtctttttt gcaaaatcta accccctttt ttcatatatg acacatataa
     3421 tttctcatgc ggagcccaga gagtactatt acggaatttt ttctagatgg agggataccc
     3481 agtgaagctt ttgaggcgtt ttagcagacg ggagcaaagc gagcggatgc aaaaacgtga
     3541 taaaagaggc attgggtata ccg
//
LOCUS       CruV-303                3564 bp    DNA     circular     24-FEB-2020
DEFINITION  Crucivirus-303 isolate 001412_virusbaton.
ACCESSION   CruV-303
KEYWORDS    .
SOURCE      Crucivirus-303
  ORGANISM  Crucivirus-303
            Unclassified.
REFERENCE   1  (bases 1 to 3564)
  AUTHORS   de la Higuera,I., Kasun,G.W., Torrance,E.L., Pratt,A.A.,
            Maluenda,A., Colombet,J., Bisseux,M., Ravet,V., Dayaram,A.,
            Stainton,D., Kraberger,S., Zawar-Reza,P., Goldstien,S., Briskie,J.,
            White,R., Taylor,H., Gomez,C., Ainley,D.G., Harding,J.S.,
            Fontenele,R.S., Schreck,J., Ribeiro,S.G., Oswald,S.A., Arnold,J.M.,
            Varsani,A., Enault,F. and Stedman,K.M.
  TITLE     Direct Submission
  JOURNAL   Submitted (24-FEB-2020) Biology, Portland State University, 1719 SW
            10th Ave, Portland, Oregon 97201, USA
COMMENT     Bankit Comment: ALT EMAIL:ide@pdx.edu
            Bankit Comment: TOTAL # OF SEQS:451
            Bankit Comment: GAP: est. length - alignment

            ##Assembly-Data-START##
            Assembly Method       :: MetaSPAdes v. 3.12; IDBA-UD v. 1.1.1;
                                     MEGAHIT v. 1.0.3
            Sequencing Technology :: 454; Illumina
            ##Assembly-Data-END##
FEATURES             Location/Qualifiers
     source          1..3564
                     /organism="Crucivirus-303"
                     /mol_type="genomic DNA"
                     /isolate="001412_virusbaton"
                     /isolation_source="Water"
                     /country="France"
                     /collection_date=2013
     CDS             1..1059
                     /codon_start=1
                     /product="Putative Replication Associated Protein"
                     /translation="MSEMKTADVATPPPPATQPKKSKSKPSPIDNKRVRGWCFTLNNY
                     TPDDVKAAKALEHEFEYIVFGKEHGEQKDTPHLQGFVHANNARRGSAMRKLFNDRAHW
                     EPQSGTFLEAAMYCKKGTQPHTEWLTFKPEGWKGPNFGKDADIFEAGEACEQGERSDL
                     EKVCKMIIEGHSLDDVATEAPSVFVRNVKGLAALRATRFTDRDPNKPPQVIWRWGLAG
                     VGKTRKPIEAHKSYFIKDGTMWWDGYEQQEAIIVDDFDGKWPYRDFLRFLDRYPYQGQ
                     FKGGYVSVNSPFIYITCEHPPEFFFGLNAGVGRPDGSAPSQNELDQVLRRITRIEHVV
                     RADNAPPPTGPERVEVTS*"
     CDS             1455..2969
                     /codon_start=1
                     /product="Putative CruV Capsid Protein"
                     /translation="MVPFGKQRTDSLRVRLCTDKPYRDYLFVFRQTIGCIYIPALFVK
                     VLVPMPRPAAPPRRRTVRRISGRGDYKVRPMVSSARPRRALRGHGDYTYDKPGPWGKA
                     GRWIGNALGSHFAGSGGGKLGEKIGSYAHYLGKIFGSGDYVTAASSVKSNTLVNSSQV
                     PQFANGLHTMKIQHREYLGDIFSSGTANTFAIQDYAINPGQVLSYPWLANVVGVSYQQ
                     YRINGQVYEFRSMSSDALNSTNTALGSVAMATDYDSKDNTFVSKAQMENTEFGVSCKP
                     SCNMIHAIECARNQTSVSELYMRPAGVPSGADQRLYDLGRFSIASTGMQGTNVNLGEL
                     WVSYDIDFLKPIQLLPLANGLEAAYEILGTDATHPLGTSSILDDVDSFGLVLPAAAST
                     SGVIAFPANLPIGTTFMFHYQITGNSTNSVAAPVVTWAHGLSSIGNTLAFPASGTATA
                     SVWYYSSVIRYTGGASASAPPTATISAGVVPASGTGILNLMQVSGNVLNDILLG*"
     CDS             complement(3148..3495)
                     /codon_start=1
                     /product="putative ORF"
                     /translation="MRTSHIHAHLCVMTSVDNAELEEWRDKCYMIIEAVEDECKKVES
                     ASHMRAAYVYLKDFYRGVCGVEALRELVNSFEPRLVMDFNDTDDGSDEEPSQCEPERC
                     GGCQFCPEEEQKK*"
ORIGIN
        1 atgtctgaga tgaagactgc tgatgtagct acgccgcccc caccggctac gcaacctaag
       61 aagtccaaat ctaagccaag tccgattgac aacaagcgcg tacgtggttg gtgcttcaca
      121 ctgaacaact acacgcctga tgacgttaaa gcggctaaag cccttgaaca tgaattcgaa
      181 tacattgtat tcggtaaaga gcatggtgaa caaaaagaca cgcctcattt gcaaggcttt
      241 gtccatgcta acaacgctcg aagaggcagt gcaatgcgca aactattcaa tgatcgcgca
      301 cactgggaac cgcaaagcgg aacctttctc gaagcagcta tgtactgcaa gaaaggaacg
      361 caaccccata ccgaatggtt gacattcaaa cctgaaggtt ggaaaggtcc caatttcggc
      421 aaggatgctg acattttcga agccggcgaa gcctgtgagc aaggtgaacg ctcagacctc
      481 gaaaaggtat gtaaaatgat catcgaagga catagccttg atgatgtcgc tacagaagcg
      541 ccaagcgtct ttgtacgcaa cgtaaaagga ctcgcagcac tacgcgcaac tcgctttact
      601 gatcgtgacc caaacaagcc gccccaagtc atctggcgat gggggctcgc tggtgttgga
      661 aaaacacgca aaccaattga agcccacaaa tcctacttca tcaaagacgg tacgatgtgg
      721 tgggatggtt acgagcagca agaagcaatc atcgttgacg atttcgacgg taaatggcct
      781 taccgtgact tcttgcgctt ccttgatcga tacccttacc agggacaatt caagggcgga
      841 tatgtgagtg tgaacagtcc gttcatctac atcacatgtg aacatccccc ggagttcttc
      901 tttggcttaa acgctggcgt tgggcgtcct gacggatcag ctccatcgca aaacgagctt
      961 gatcaagtgc tacgtcgtat cactcgaata gagcatgttg tacgcgctga caatgcaccc
     1021 ccaccaaccg gtccggaacg cgttgaagtt acgagctgat aatctgtcct acggactgtt
     1081 ctaagctcat ctcttttttt ggcaaccttt gtgccaagtg tctgtgccag aacacagaaa
     1141 aaggtgtgcc aaatctacgt gccaaacgcg ctacgcgctc tatttcgact aagaggatac
     1201 ctctgattct tttggcacag aagtgtctaa ggtaatactg gaggctatgc ctcctcctta
     1261 tccacttctg taaccccgtc agatcatacc tatccgacgt attgcctcca gacatgagca
     1321 agatgcatcg tgccaggggc ctaaacccca cctacggtgg gggcggcccc tgtcgcgatg
     1381 cttgttcagg ccttgcctcc gcgcttacgc gcggccacct gtgcattttt ttagagtagg
     1441 aagggtacca ttgcatggta cccttcggaa agcaacgtac tgactcgcta cgcgtgagac
     1501 tctgtacgga taaaccctat cgagattatt tatttgtttt ccgacaaact atcggctgca
     1561 tctatatacc tgcacttttt gtcaaagtac tcgtaccaat gccacgtccc gcagctccgc
     1621 cgcgtagaag aactgttagg cgcatttcag gccgcggtga ttacaaagtt cgcccaatgg
     1681 tttcaagtgc tcgccctcgt cgggctctgc gtggacatgg ggactacaca tatgataaac
     1741 ccggtccttg gggtaaagct ggtcgttgga ttggtaacgc cttgggaagt cactttgctg
     1801 gttcaggagg cggcaaactt ggtgagaaaa tcggctcata tgctcattac ctgggcaaaa
     1861 tctttggttc tggtgactac gtcacagctg catcgtctgt caaaagcaac acgcttgtga
     1921 acagctcgca agtaccacaa ttcgctaacg gactgcacac aatgaaaatc caacatcgcg
     1981 agtacctcgg cgacatcttc tcttctggaa cagcaaacac atttgccatc caggactacg
     2041 caatcaaccc tggacaagta ctatcctatc cttggctggc taacgtcgtt ggcgtcagct
     2101 accagcaata ccgcatcaac ggtcaagtct atgaattccg ttcaatgtca tcagatgcac
     2161 tgaacagcac aaacactgct ctcggttccg tggcaatggc cacagactac gacagcaaag
     2221 acaacacatt cgtttcaaaa gcccaaatgg aaaacacaga gttcggtgtc tcttgcaaac
     2281 cctcttgtaa catgattcac gcaatcgaat gcgcgcgaaa ccaaacatca gtctctgaac
     2341 tctatatgag acctgcagga gtacccagcg gagctgatca acgtctgtat gatctcggaa
     2401 gattcagcat cgcatcaact gggatgcaag gaacaaacgt caatctcggg gaactctggg
     2461 taagctacga tatagacttc ctcaaaccaa ttcagctgct accgcttgcg aatgggcttg
     2521 aagctgcata tgaaatcctg ggaacggatg cgacccatcc acttggaact tcaagcatat
     2581 tggacgatgt ggacagtttc ggcctcgtgc ttcctgccgc cgcaagcact tcaggggtaa
     2641 tcgccttccc ggcgaacctc cccataggaa ccacattcat gtttcactac caaatcaccg
     2701 gaaacagcac gaactccgta gctgcacctg tagtaacatg ggcacatggg ctatcatcca
     2761 tcggaaacac tctcgcgttc cctgcatctg gaactgcaac agctagcgtc tggtattaca
     2821 gttctgtaat acgatacaca ggtggagcct cggcaagtgc cccgcctacg gccacaattt
     2881 cggctggagt cgtacccgcc tcaggtacag gtatattgaa cctcatgcaa gtaagcggaa
     2941 atgtcttgaa tgacattctt cttggttaag aggatttgcc gacttcgcgg tcatgcctca
     3001 tgccaaaaaa actaccggaa ggagatgaac agaagcattg cacatgcttc taccgatcgg
     3061 aatttttaac gaccaaaccc ggttaccgac accggtacac gggacccggc gggaatttct
     3121 tcccgcccaa cccgcaaggt tgcgggttca ctttttttgc tcctcttcag ggcaaaactg
     3181 acaacctccg catcgctccg gttcgcattg tgatggttct tcatcggacc cgtcatccgt
     3241 gtcgttgaag tccataacaa ggcgaggttc gaaggagtta actaactccc tcagggcttc
     3301 caccccacac acgccacggt agaaatcctt caaatataca taggcggctc gcatatgcga
     3361 ggcagactca accttcttac actcgtcttc caccgcctca atgatcatgt agcatttatc
     3421 acgccactct tcgagctcgg cgttatctac tgatgtcatg acgcacaaat gcgcgtgtat
     3481 atgagaggta cgcaaactta ttgagatatt tattatctga agaagtctcg cgacacacta
     3541 tatacgtgtg ccaatcgtgc caac
//
LOCUS       CruV-304                3567 bp    DNA     circular     24-FEB-2020
DEFINITION  Crucivirus-304 isolate Sty_232.
ACCESSION   CruV-304
KEYWORDS    .
SOURCE      Crucivirus-304
  ORGANISM  Crucivirus-304
            Unclassified.
REFERENCE   1  (bases 1 to 3567)
  AUTHORS   de la Higuera,I., Kasun,G.W., Torrance,E.L., Pratt,A.A.,
            Maluenda,A., Colombet,J., Bisseux,M., Ravet,V., Dayaram,A.,
            Stainton,D., Kraberger,S., Zawar-Reza,P., Goldstien,S., Briskie,J.,
            White,R., Taylor,H., Gomez,C., Ainley,D.G., Harding,J.S.,
            Fontenele,R.S., Schreck,J., Ribeiro,S.G., Oswald,S.A., Arnold,J.M.,
            Varsani,A., Enault,F. and Stedman,K.M.
  TITLE     Direct Submission
  JOURNAL   Submitted (24-FEB-2020) Biology, Portland State University, 1719 SW
            10th Ave, Portland, Oregon 97201, USA
COMMENT     Bankit Comment: ALT EMAIL:ide@pdx.edu
            Bankit Comment: TOTAL # OF SEQS:451
            Bankit Comment: GAP: est. length - alignment

            ##Assembly-Data-START##
            Assembly Method       :: MetaSPAdes v. 3.12; IDBA-UD v. 1.1.1;
                                     MEGAHIT v. 1.0.3
            Sequencing Technology :: 454; Illumina
            ##Assembly-Data-END##
FEATURES             Location/Qualifiers
     source          1..3567
                     /organism="Crucivirus-304"
                     /mol_type="genomic DNA"
                     /isolate="Sty_232"
                     /isolation_source="Water"
                     /country="New Zealand"
                     /collection_date=2012
     CDS             1..1083
                     /codon_start=1
                     /product="Putative Replication Associated Protein"
                     /translation="MFTYYIYGREVGEQGTPHLQFYVAGAKKITFKCLKKIFPRAHLL
                     VCKGSPDQNYKYCSKDGNFVTYGDLPAAKTAKATKAAKDKWDEVKELAIAGDLDAIDS
                     EVFIKHYPTLKRIRSDHRMKAKLPTLDWTASPNIWIYGPPGVGKSRRARLTYPDAYIK
                     APQNKWWGGYNDEPVVLIDDLRMDQDYQLSNLITWADRYPFQAEIKLDSTGMIRPRVI
                     VVTSNFHPSQIWKRPEDINSILRRFRMVNMELLEQFDTTESTKKRKVVEEVTPEKPPL
                     FRQNATGNLVPFVDLQPKITKVFDLTSSVTGIDMELTQDDEPAVIDLTDVDNAIAEAT
                     QIYKMCDRCGDKVIECSCDMANPSMY*"
     CDS             complement(1116..2684)
                     /codon_start=1
                     /product="Putative CruV Capsid Protein"
                     /translation="MEPLDTHVPTHVHIQRPPVGQIHTTHHTFLGAHVHETSEHRMSY
                     YAGRDVAMRGNDVQERGRYPAYYRIKKGLAPDQYWRARYYRRRFTGRGDYRSVRRPVA
                     KRRKLSRRPLRGRGDYSGGTGLGSSIGSYLGSKAGGYLGGMAEHAVMGLVSGLGDYKV
                     KKNVLLGGNMPKIENHSGPGGITVQFQEYLGEVTTPIQAGNSSPFDIKTYLINPAVST
                     TFPWLSQLAANFEQYEIEGMLFGFRTTSADMVTNATNNLQLGTVIMATQYDVADAVFG
                     SKAEMLNYEFSSSVKPSEGAVHMIECDPRQTSVNLLYTSPGAVPTGTDPRLYHLGRFA
                     IATQGFQGSGITIGELHITYQVRLLKPKLWSGLGQTIPFSSFQNIWTNDNALGYLGPI
                     TTIASRQTNGLGMTFGFVTSGPATVNFPIQGKPLRVLVRFRWSTTAAGVYFLTNPPAI
                     NNAQNGTIISSVLSPQTGTNNSTTGMVTTVFQLGVGGNVSPSFDVGLNTVALPLAQVT
                     LLIDATVVDPDVVS*"
ORIGIN
        1 ctgttcactt attatatcta tggtcgagag gtgggtgaac agggaacgcc gcatttgcaa
       61 ttctacgtag ctggggctaa gaagataacc ttcaaatgct taaagaagat ttttcctcgt
      121 gcgcatctgt tggtctgtaa gggttctcct gatcaaaatt acaagtactg ttccaaagat
      181 ggtaattttg tgacctatgg tgacttgccc gcagcaaaga ccgctaaagc tactaaggct
      241 gctaaggaca agtgggacga agtaaaagag ttggcaatcg ctggtgactt ggacgctata
      301 gatagcgaag tgttcattaa acactaccca actcttaaac gtattcgatc agaccatcga
      361 atgaaggcca aattacctac gctggattgg actgcttctc caaatatttg gatctatggc
      421 ccccctggag tcggtaagtc tcgtcgtgct cgccttacct accctgatgc atacatcaaa
      481 gctccgcaga ataagtggtg ggggggctac aatgatgagc ctgttgtcct cattgatgac
      541 ttgcgcatgg atcaagatta ccagctttcg aatcttatta cttgggctga tcggtaccct
      601 ttccaggctg agatcaaact cgattcgaca ggtatgatta ggcctagagt gattgttgtc
      661 acttcaaatt ttcatccgag tcaaatatgg aaacgtcccg aggatatcaa ttctattttg
      721 cgccgctttc gtatggtaaa catggagctt ttggagcaat ttgataccac tgagtctact
      781 aagaagcgga aagttgtgga agaagtcact ccggaaaagc ctcctctctt tcgacagaat
      841 gctactggta acttagttcc gttcgtggat cttcagccga agattaccaa ggtgtttgat
      901 cttacttcct ctgttactgg aattgatatg gagcttaccc aggatgatga acctgccgtt
      961 attgatttaa ctgatgtgga taacgccatt gctgaagcta ctcagatcta taagatgtgt
     1021 gaccgatgtg gtgataaagt tatagaatgt tcatgtgata tggctaatcc atccatgtat
     1081 tgatttattt tgttattaca gaaataaata ttttgttaag atactacgtc aggatctact
     1141 actgtggcgt ctattaacaa ggtgacttga gccagtggaa gagcaacggt gtttaatcct
     1201 acgtcaaaag atgggcttac atttccacca actccaagtt gaaacacggt ggttaccata
     1261 ccggtggtgg agttgttagt ccccgtttgc ggtgacaata cggaagaaat gatggtcccg
     1321 ttttgggcgt tatttatagc tggcgggttt gtcagaaaat acacgcctgc agcagtggtg
     1381 gaccacctaa aacgtactag cacgcgaagt ggcttcccct gtattgggaa gtttacggtg
     1441 gcgggacctg aggtgacgaa gccaaatgtc atgccaaggc cgttggtttg tctcgaagca
     1501 attgttgtaa ttggaccaag gtaaccaaga gcgttgtcat ttgtccaaat gttctggaag
     1561 gaggaaaacg gaatcgtttg gcctaagccg gaccataatt tgggtttgag aagacgaact
     1621 tgataagtaa tgtgtagctc cccaatagtt atgccactgc cttggaatcc ttgggtagct
     1681 atagcgaagc gccccaggtg atagagtctc gggtctgttc ccgtaggaac tgcacctggg
     1741 gaagtgtaaa gtaaattaac ggacgtttgt ctagggtcgc attctatcat atgcacggct
     1801 ccttcggaag gtttcactga tgatgagaat tcgtaattta gcatttcagc cttgctgcca
     1861 aaaactgcat ctgcgacatc atattgagtt gccataataa ctgttccaag ttgtaagttg
     1921 tttgtggcgt ttgtaaccat gtcggcggag gtagtccgga acccaaacaa catgccttcg
     1981 atttcatatt gttcaaagtt cgccgcgagt tgtgacaacc aggggaatgt agttgatacc
     2041 gcgggattaa tgagataggt ttttatatcg aagggagaac tgtttccagc ttggataggt
     2101 gttgttactt ctcctaggta ctcttgaaat tgaacggtaa tgccaccggg tccggaatga
     2161 ttttcaattt taggcatatt gccacctagg aggacgttct tcttcacttt gtaatctccg
     2221 agtccggaga ccaaacccat aacagcgtgt tcggccatgc ctcctaaata tcctccggct
     2281 ttgcttccaa ggtatgagcc aatagatgag ccaagtcctg ttcctccaga atagtcacct
     2341 ctacctcgca atggtcttct ggagagcttt cgtcgcttcg cgacaggtct tctaacagat
     2401 cgataatcgc ctctaccagt aaatcttcgt cgatagtaac gcgctctcca atactgatca
     2461 ggagcgagac cttttttgat ccggtaatat gccggatacc tacctcgctc ttgaacatcg
     2521 tttcctcgca ttgcgacatc tcttcctgca tagtatgaca tcctgtgttc tgacgtttca
     2581 tgcacgtgcg caccgagaaa cgtgtggtga gtggtgtgga tctgtccaac gggtgggcgt
     2641 tggatgtgga catgggttgg cacgtgtgtg tccaacggtt ccatgtccaa cggtcggggg
     2701 taatactaag gccttcggcg cccccgaccg ttttaaaggc ctttttgggg cctttgtatt
     2761 tctttattta gcggcttcgc cggggttgtg tgtcactatg gtcaatatgg tgtcactgtg
     2821 gtgttatgga gggaaatgtg tggtatttcc ctcgctggat tgtgccgtgg gggggacccg
     2881 gggccccagt cccctgggtc cccccgcacg gtagtttaac cttctcgacc cctgactaag
     2941 agtcaagcat agtttctctg aagcttacca gattacctct gatataaggg gttcgagtgt
     3001 agattgattg gattttgatc caactagggt tattgggtga attagggtta ttgaccaaac
     3061 tagggttagc ggtttcgtaa ccctagtacc gcggctgctg gcactaccgc ggctgctggt
     3121 atataaataa aaaaaaaaaa aaaaaatagc ccaatgcatt tatcgggtaa tgcatttttc
     3181 gggtcggccc tgaaaaaatg attttgggct ttgcaatcca catcctcatt ttctgaactg
     3241 catggaccct acaccacacg tcgaaaattt atccaatttg tttactggcg atgaatcaac
     3301 caagggatac gctattcact ggtatgatgt tctcccatca ttttcatttc tgggggggtg
     3361 aggattcaaa agcccaaatt ggttttgtca tcacgtgcgt tttggactta gcgaaatttt
     3421 caaaaaaaaa aaaaaaaaaa aaaaaaaaaa aaaatttttn aaaaaaaaaa aaaaaaaaaa
     3481 aaaaaaaatt gttattaatt ttttaggtgt ggcacaatca ataatttttc tgaagttgac
     3541 gaagcttgtt ttactgctaa tcgcagt
//
LOCUS       CruV-305                3569 bp    DNA     circular     24-FEB-2020
DEFINITION  Crucivirus-305 isolate H4_30587.
ACCESSION   CruV-305
KEYWORDS    .
SOURCE      Crucivirus-305
  ORGANISM  Crucivirus-305
            Unclassified.
REFERENCE   1  (bases 1 to 3569)
  AUTHORS   de la Higuera,I., Kasun,G.W., Torrance,E.L., Pratt,A.A.,
            Maluenda,A., Colombet,J., Bisseux,M., Ravet,V., Dayaram,A.,
            Stainton,D., Kraberger,S., Zawar-Reza,P., Goldstien,S., Briskie,J.,
            White,R., Taylor,H., Gomez,C., Ainley,D.G., Harding,J.S.,
            Fontenele,R.S., Schreck,J., Ribeiro,S.G., Oswald,S.A., Arnold,J.M.,
            Varsani,A., Enault,F. and Stedman,K.M.
  TITLE     Direct Submission
  JOURNAL   Submitted (24-FEB-2020) Biology, Portland State University, 1719 SW
            10th Ave, Portland, Oregon 97201, USA
COMMENT     Bankit Comment: ALT EMAIL:ide@pdx.edu
            Bankit Comment: TOTAL # OF SEQS:451
            Bankit Comment: GAP: est. length - alignment

            ##Assembly-Data-START##
            Assembly Method       :: MetaSPAdes v. 3.12; IDBA-UD v. 1.1.1;
                                     MEGAHIT v. 1.0.3
            Sequencing Technology :: 454; Illumina
            ##Assembly-Data-END##
FEATURES             Location/Qualifiers
     source          1..3569
                     /organism="Crucivirus-305"
                     /mol_type="genomic DNA"
                     /isolate="H4_30587"
                     /isolation_source="Water"
                     /country="New Zealand"
                     /collection_date=2012
     misc_feature    join(3557..3569,1..20)
                     /note="stem-loop"
     misc_feature    1..9
                     /note="nonanucleotide"
     CDS             189..1079
                     /codon_start=1
                     /product="Putative Replication Associated Protein"
                     /translation="MSLSRRYCFTVNNPSEEDCEQVYALSKHKHHKYLIVGSEKGECG
                     TPHLQGYVVFAKPVRFNTFKALIPRAHVEAAKGTSEQNRVYCMKEGDYEEFGDVPTDG
                     GQSLQVCEWDQARANAKLGKFDDIPSNLYIRYKNAFHSIFDDACNASECIVELDHLWI
                     TGPTGIGKSRYCFESFPNAYRKGANKWWCHYTNQDVVILEDVEPSQEKWIGYYLKIWS
                     DHYPFIAERKGGSRSIRPKRIVITSNYSIKEIFTDAKIRDPLLRRFKERTIKDGELIA
                     SISQEILFRQIGVDHYEAIE*"
     CDS             1095..2702
                     /codon_start=1
                     /product="Putative CruV Capsid Protein"
                     /translation="MVSTRSRRRSSGSSSSSSAFPLPVNPFVPPTQRSTQQGLTEAVA
                     EMSAETQTQPSSDGYISYYTGEPLTASGAVGPQNWVDPVTYRALRKKFTKKRKRTTTK
                     KRSTRRSSRSYGTSGRSLSGMGPIVITGRGGYWGDKFGEGASAAWKSLKRATPEGTFS
                     RLGRAAGGAMFGKPGASVGNWLGGGVSGILGFGDYAVKKNDLMMLDEGVPVPTFQNMN
                     DGVIVCHREYIGDITQTTAFTNNQFIIQPGDSTTFPWLNQIAHQFEQYEILGMIFQYR
                     STASDFGTTSNMAMGSVIMATEYDTVDSSYGSKLEMENSQYAMSGKPSHDILHPIECD
                     PGVVGPMGLKYIRTGPVPTGKDNRLYDHGNFQVATTGMPANSGTIGELWVTYKIAFYK
                     PVFNLGSDIKTDIFFATTGASSGAYFTGYNPQPANSLGCTLSVATCTFPANAITGNKY
                     LVSYFVLGASNPGIFINTATLVNCVVLYQNANPSAVTTTDRATISFSLRVLITNPAVP
                     VSFNLSGNIPASTLGQLYITQIDSEAV*"
ORIGIN
        1 tagtattacc ccgccccccc gtaacccatg taacacctgg gttattatat ggggagccct
       61 aatattagct gttacggagg ccgcgccagt gtaatttgtc gatgtaacag ggcctaggtc
      121 ccataatggt ggtatcgtta tgggactcta catagggact aatggtgctt tagctcctgc
      181 cagtcgctat gtcgctttct cgccgttatt gctttacggt gaacaatccg agtgaggagg
      241 attgtgagca ggtttatgcc ttgtcgaagc ataagcatca caagtatttg attgttggta
      301 gcgaaaaggg ggagtgtggt accccgcatt tgcaaggata tgttgtgttt gccaagccag
      361 ttcgatttaa cacgtttaaa gctttaatac cgagggcgca tgtggaggct gctaaaggta
      421 cctctgagca gaatcgggta tactgtatga aggagggtga ttatgaggaa tttggtgatg
      481 ttcctacgga tggtggtcaa tcgctgcaag tttgtgagtg ggaccaagct cgtgctaacg
      541 cgaagcttgg aaaatttgat gatattccat cgaatttgta catccgttat aagaacgcct
      601 tccacagtat atttgacgat gcatgtaacg caagtgagtg tattgtcgaa ttggaccatt
      661 tatggataac tggtccaacc gggattggga aatctcggta ttgttttgaa tcgtttccta
      721 atgcttatcg aaagggtgca aacaagtggt ggtgtcatta caccaaccag gatgttgtca
      781 ttctcgaaga tgtggagcca tcacaagaaa aatggatagg atactatctg aagatttggt
      841 ccgaccatta tccttttatt gctgaaagaa agggaggatc gcgcagtatt cgacctaagc
      901 gaatagtgat aacgtcaaat tatagtatca aggagatttt tactgatgcc aagattcgag
      961 atccgctttt gcgtcgtttt aaagaacgta ctattaagga tggggaatta atagcgagta
     1021 tatcgcaaga gattttattt cggcagattg gtgttgacca ttacgaagct attgaataaa
     1081 gtgcgtattc atttatggta tcgacacgta gtcgacgtcg ttctagtggg tcgtcgtcgt
     1141 cgtcttctgc attccctctt cctgttaatc cgttcgttcc tccgacgcag cgctctactc
     1201 agcaaggtct tacagaagca gttgctgaaa tgtctgctga aacgcagact cagccgtcat
     1261 cggacggcta cattagttac tacaccggag agcctttaac ggcctcaggt gcagttgggc
     1321 cacaaaattg ggtggaccct gttacttatc gtgcacttcg aaagaagttc accaagaagc
     1381 gtaagcgtac gaccacgaag aagaggtcca cgcggcgttc cagtcgcagt tatggaacct
     1441 ctggtcgcag cctctctggg atgggtccta ttgttattac tgggcgtggt ggttattggg
     1501 gtgataagtt tggcgagggt gcatctgcag cctggaaatc tttaaagcgc gccactcctg
     1561 aaggcacgtt tagtcgttta ggtcgtgccg ctggtggtgc gatgtttgga aagccaggag
     1621 ccagtgtagg gaactggctt ggtggaggcg tgtctggaat tcttggattt ggagactacg
     1681 ccgttaagaa gaacgacctg atgatgttgg atgaaggggt tcctgttccg acatttcaaa
     1741 atatgaacga tggagtgatt gtttgtcatc gggagtatat tggcgatata acgcagacga
     1801 ctgcgtttac taataatcag tttattattc aaccaggtga ttctactacg tttccgtggt
     1861 tgaatcagat tgctcatcag ttcgagcagt atgaaattct tggtatgata tttcagtatc
     1921 gttctactgc cagtgacttt ggaactacgt cgaatatggc aatgggaagt gttataatgg
     1981 ctacagaata tgacactgtt gattcctctt acgggagtaa gttggaaatg gagaattcgc
     2041 agtatgcaat gagtgggaaa ccgtctcatg atattttgca tcctattgaa tgcgatcctg
     2101 gtgttgttgg accaatgggt ttgaaatata ttcggactgg tccagttcca actggcaaag
     2161 ataatcggtt gtatgatcat ggtaatttcc aggttgctac aaccggtatg ccagcgaatt
     2221 caggcactat tggtgaatta tgggtaacgt acaagattgc gttttacaag ccggtgttta
     2281 atcttggtag cgatatcaag actgacatat tctttgcaac aactggcgca tcatctggcg
     2341 cttacttcac cggatataat cctcaacctg cgaacagtct tggttgtaca ttaagcgtag
     2401 ctacttgtac ctttcctgct aacgctatta ctggaaacaa gtacctagtg agttacttcg
     2461 tgctcggtgc aagcaaccca ggtattttca ttaatactgc gactttggtc aattgcgttg
     2521 tattgtacca aaacgcaaac ccaagcgccg ttactaccac agatcgcgcc acaattagct
     2581 tctctcttcg cgttctaata acaaatccag ctgttcctgt atctttcaac ttatcaggta
     2641 acatcccagc gtcaacgctg ggacagctgt atattacgca gattgattct gaagctgttt
     2701 aaataaaaaa agttatttta ttgtgttgat cggacgcgaa tttcgtagtt cgtggctccg
     2761 ggctcgttac gcagccggag ccacgtgtaa gtgaatttcg ctttcttctt cttcttcaga
     2821 gaaggagagt cttctcgatc ggatcgggag gtcgtcgtct cgtagtcgcc cgagggcgag
     2881 cgacatctcc cagctgtcgc tccggctgta gagttctcgt ctgaagtagc ggtatggtct
     2941 ggcgaatcgg tgtagatggt ccgtgttgtt gaagacccag acgacgccgg caacagagcc
     3001 ataagtgacc taaggaattc agattgatta tgtcagactg ctctaatccg tactgaccat
     3061 taaaggttca atgttaagca ctagtctgat aaggtaagta tggtaagtct tcgttcctat
     3121 atagggaggc tccgccagaa gtactttatg ggacctttaa aactcacagt tgagttttaa
     3181 tgcgtgactg ggtacagtca ggaaaaaagt aaaagtagtg tttgttttct atcgcaaagt
     3241 aaaggggcct accttgtgta gggctttatg ttttttgatg ctgcgccccg taggccagtc
     3301 atctttgctt tttagaaccc ctaaacttta gggcggccga aggccgggcc gccgtaggcg
     3361 gcttaggccg ccgtaggcgg cttccgggcc ccgaaggggc cctttagggg ctttaggggc
     3421 ccgaagggcc ttttaggggc tttagatagg ttttttaaag cctttgtttt tggggcccgc
     3481 agggccctgc gcgccgcagg cggcccccgg caggggagcg cagcttttgg gccacggact
     3541 ttttgcgtgg cctttagggg gggcgggtc
//
LOCUS       CruV-306                3576 bp    DNA     circular     24-FEB-2020
DEFINITION  Crucivirus-306 isolate GP2_73319.
ACCESSION   CruV-306
KEYWORDS    .
SOURCE      Crucivirus-306
  ORGANISM  Crucivirus-306
            Unclassified.
REFERENCE   1  (bases 1 to 3576)
  AUTHORS   de la Higuera,I., Kasun,G.W., Torrance,E.L., Pratt,A.A.,
            Maluenda,A., Colombet,J., Bisseux,M., Ravet,V., Dayaram,A.,
            Stainton,D., Kraberger,S., Zawar-Reza,P., Goldstien,S., Briskie,J.,
            White,R., Taylor,H., Gomez,C., Ainley,D.G., Harding,J.S.,
            Fontenele,R.S., Schreck,J., Ribeiro,S.G., Oswald,S.A., Arnold,J.M.,
            Varsani,A., Enault,F. and Stedman,K.M.
  TITLE     Direct Submission
  JOURNAL   Submitted (24-FEB-2020) Biology, Portland State University, 1719 SW
            10th Ave, Portland, Oregon 97201, USA
COMMENT     Bankit Comment: ALT EMAIL:ide@pdx.edu
            Bankit Comment: TOTAL # OF SEQS:451
            Bankit Comment: GAP: est. length - alignment

            ##Assembly-Data-START##
            Assembly Method       :: MetaSPAdes v. 3.12; IDBA-UD v. 1.1.1;
                                     MEGAHIT v. 1.0.3
            Sequencing Technology :: 454; Illumina
            ##Assembly-Data-END##
FEATURES             Location/Qualifiers
     source          1..3576
                     /organism="Crucivirus-306"
                     /mol_type="genomic DNA"
                     /isolate="GP2_73319"
                     /isolation_source="Sediments"
                     /country="New Zealand"
                     /collection_date=2014
     misc_feature    join(3571..3576,1..22)
                     /note="stem-loop"
     misc_feature    1..9
                     /note="nonanucleotide"
     CDS             complement(6..1064)
                     /codon_start=1
                     /product="Putative Replication Associated Protein"
                     /translation="MAEIKLEYVADVDNEVGRIVYTGDFLKQRARNWCFTHNNYTQED
                     IQVIRTAWPALHGFMGVMFEEEKGEAKATPHLQGFVCFSTMKSGKQMLEIGPRTHWAV
                     MKGSIEANKTYCSKDNCGVVVIGQFPMSAKDKGIAGAKHGAKGASAPNEWAELVKDIK
                     AGMKRSDLMEKYAEIGIRCPKGFNDYIAEFTPKHTFSILERYGSYLPWQEQLIDIIAL
                     GAGPREVIWIYSPEGNVGKSDMAKHLIYNQGFQPLQNATSRDLSCAWQCGSVVIDLAR
                     DQGGSPINYGFMECVKDGRSFSSKYESTCKLADPKRNRYVICFANERPDIQKLSADRW
                     AIFRVVDGVLQWEDSGLV*"
     CDS             complement(1283..2668)
                     /codon_start=1
                     /product="Putative CruV Capsid Protein"
                     /translation="MPAVSRRAAPVRKQRAPAKPKIGAARRGAPVRSKVAVPHERGMT
                     RAGKAFRTGGQGLGSAIGSAFGGPIGGMAGGALGRGAGAILSKIFGHGDYSVSNVEQI
                     RKNNLVLSNSAQMPQFGSGKVACKLVHREFLGDLISSATAGAFKIQTYAINPGIAETF
                     PWLSGVVGAKFQQYRINGMTFEFRSMSADALNSTNTALGSVIMSTDYDSADVDFASKQ
                     EMENTEYGVSCKPSANMLHGIECERFQTPVSELYIRAFDVPSGKDIRLYDLGKFQIAS
                     VGCQGTNVNLGELWVSYDIDVFKAIEQPPGYLSPVASYTLSGVGSASALGLSTTTPVE
                     VVDQIGLTFVRSSGTDTVQFPLSILPGVKYMMHYGILGASTASVAYQAITLAGGLVAL
                     GSDNANPSFSQGTLTTASKQHSMAFYYNGTGTQASPPTLAFTGGVVPTSLTLMTLSIA
                     EIGNAYPADGW*"
ORIGIN
        1 caatattaga ctaaaccaga gtcctcccat tggagtactc cgtcgactac tcggaagatc
       61 gcccagcggt cagctgagag cttctgtatg tcgggacgtt catttgcgaa gcagataacg
      121 tatctattgc gtttagggtc cgccagtttg caggtggact cgtatttgga tgagaaggac
      181 cttccgtcct tcacgcactc cataaagcca tagttgatcg gcgaacctcc ctggtccctc
      241 gctagatcga taacgacaga accgcactgc caagcgcacg acaggtccct gctcgtggcg
      301 ttttgcagcg gttggaagcc ttgattgtaa atcaggtgct tagccatgtc ggacttacct
      361 acgttaccct caggggagta tatccagatc acttcccgag gtcccgcgcc gagagcgata
      421 atgtcgatta gctgctcttg ccacgggaga tacgatccgt atctctccag gatggagaac
      481 gtatgtttag gtgtaaactc cgcgatataa tcattgaacc ccttggggca ccgaatccca
      541 atttcggcgt acttttccat gaggtcgcta cgtttcattc cagccttgat atccttaaca
      601 agctctgccc attcgttggg tgccgaagct ccctttgcgc cgtgcttggc tccagcgatc
      661 cccttgtcct tcgcggacat ggggaactgt ccaatgacca ctaccccgca gttgtccttg
      721 ctgcagtagg tcttgttggc ctcgatagag cctttcatga ccgcccaatg cgtacgaggt
      781 cctatctcga gcatttgctt ccctgacttc atcgtagaga agcagacgaa gccttggaga
      841 tgtggggttg ctttagcttc tcccttctcc tcttcgaaca tcactcccat gaatccatgc
      901 agggcaggcc acgccgtcct aatgacctgg atgtcttcct gggtgtagtt gttgtgtgtg
      961 aaacaccagt tccgagctct ttgcttgagg aagtcgccag tgtagacaat gcgtcctact
     1021 tcattgtcca cgtcggctac gtactctagc ttgatttcgg ccatgttgct ccgctatatg
     1081 gtggtcatta caataccaaa ccaaaaaaac aattaatgat cagcccgaag gctatcgggc
     1141 tgaacatcgt gaaggccaat agacgcgcat ctcttcatca tcttaatgtc ggacgttcag
     1201 atcatgaaac cgagcatgtg gttgtctctt gcccgtctcc tgtgactatg acgagttcct
     1261 cgcattctac aggcccaagc attcaccagc cgtcagctgg gtatgcgtta ccgatctcag
     1321 ctatactcag ggtcatgagt gtgagactcg ttggcactac tccaccggta aaggctaaag
     1381 tgggcggtga cgcctgcgta cccgtgccgt tgtagtagaa cgccatgctg tgctgcttgc
     1441 ttgcagtagt gagggtccct tgtgagaaag atgggttcgc gttgtcggag cccagagcta
     1501 cgaggcctcc ggccagagtg atagcctggt atgcgaccga agccgttgag gctccaagga
     1561 tcccgtaatg catcatgtat ttaactcctg gtagaataga cagcggaaac tgtaccgtgt
     1621 ctgttccaga tgatcttacg aaagtcaggc caatctggtc cactacctcc actggagtag
     1681 ttgtcgatag ccccaatgca cttgcacttc ccactccact cagtgtatat gaggccactg
     1741 ggctcagata ccctggtggt tgctctatag ccttgaatac gtcaatgtcg tatgaaaccc
     1801 agagctctcc caagttcacg ttggtgcctt ggcacccgac cgatgcgatc tggaactttc
     1861 cgagatcgta gagcctaatg tccttcccgg agggaacatc gaatgctcgg atgtacaact
     1921 cagatacggg cgtttggaag cgctcgcact cgataccgtg tagcatgtta gccgatggct
     1981 tacaggatac gccgtattct gtgttttcca tctcctgctt tgacgcaaag tctacgtcgg
     2041 ctgagtcgta gtcagtcgac ataatcactg atcccaacgc tgtgttggtg gagttcagcg
     2101 cgtccgcaga catgcttcga aactcgaatg tcatgccatt gatgcggtat tgctggaact
     2161 tcgctccaac tacgccagat aaccatggga acgtctctgc tattcccggg ttgatagcgt
     2221 atgtctgaat cttgaatgca ccggctgtag ccgagctaat caggtctccg aggaattccc
     2281 tatgcaccaa cttgcaagca accttccccg aaccaaactg gggcatttgc gctgagttgg
     2341 acagcactag gttattcttc cggatctgtt ctacgtttga tactgagtag tcaccatgtc
     2401 cgaatatctt acttaggatg gctccagctc ctcgaccgag agctcctcca gccatcccac
     2461 caattgggcc tccgaaggcg gatccaatcg cacttcctag gccttggcct ccagttctga
     2521 atgcctttcc ggctcttgtc attcctctct cgtgaggtac tgccaccttg ctcctaacag
     2581 gagcgcctcg ccttgctgct ccaattttgg gcttagcggg cgcgcgctgt ttgcgaacag
     2641 gggcagctct acgggaaacg gcaggcatct tcgagtgatg ataggtcgct ataggtagcg
     2701 tccgacaatt cttccccaaa ataataatta atgtcctcga cttaattact taattgccag
     2761 tagaactctc cggaaagtcc ttattccgga actttctact cgtaaataat aattaatcca
     2821 atagtgccac gaccaggccc accaggcccg ccgtcagtgc tctcttgatg gtccacatgt
     2881 gtgccatata ttatctcccc taaaaaaata attaatcaat taaatgcctt atgtcgtaga
     2941 aggcgggaac tcggccccgg gaggggatcc gtaggattca gccgtgagtt gcatagcaac
     3001 gatcggagga ccgattatct agcctggtga acgacttcag gcagtcatct aaagggccct
     3061 taggtccgaa atagggttgg cccagatggt ctcggccact gatcagttca aatgaacaat
     3121 gcagccgtag gctataacat taacaccatc accccttccc agcagggcga agcgggccga
     3181 aggcgcgcgc ccgagcccct gcagtcacgc gaagcgggac gagggccgaa gaccgaagcg
     3241 aagcgaggtt tcgggccctc gcgcccgatg cagcgtgcgc aggaataggc gaagggcagc
     3301 acgcatgagg ggagccggag cgaagcggag gcaccgtaag ccatgcgcat acccctcccc
     3361 caaatctatg ggcaaaaaat gtaaaagagg tgtgatcttg aggggggtct agtggcgtag
     3421 cctactatta cctagacccc ccctcgcgct cagctcaggg ggtaaagtga agtacaaggt
     3481 acggaacgtt aaaaatgtcg tttatatgca gataactctt tccgtccgcc gcaggcggcg
     3541 ctccacgccc cccgcagggg aaacacatgt actctg
//
LOCUS       CruV-307                3577 bp    DNA     circular     24-FEB-2020
DEFINITION  Crucivirus-307 isolate 120_1246_IR2_3297.
ACCESSION   CruV-307
KEYWORDS    .
SOURCE      Crucivirus-307
  ORGANISM  Crucivirus-307
            Unclassified.
REFERENCE   1  (bases 1 to 3577)
  AUTHORS   de la Higuera,I., Kasun,G.W., Torrance,E.L., Pratt,A.A.,
            Maluenda,A., Colombet,J., Bisseux,M., Ravet,V., Dayaram,A.,
            Stainton,D., Kraberger,S., Zawar-Reza,P., Goldstien,S., Briskie,J.,
            White,R., Taylor,H., Gomez,C., Ainley,D.G., Harding,J.S.,
            Fontenele,R.S., Schreck,J., Ribeiro,S.G., Oswald,S.A., Arnold,J.M.,
            Varsani,A., Enault,F. and Stedman,K.M.
  TITLE     Direct Submission
  JOURNAL   Submitted (24-FEB-2020) Biology, Portland State University, 1719 SW
            10th Ave, Portland, Oregon 97201, USA
COMMENT     Bankit Comment: ALT EMAIL:ide@pdx.edu
            Bankit Comment: TOTAL # OF SEQS:451
            Bankit Comment: GAP: est. length - alignment

            ##Assembly-Data-START##
            Assembly Method       :: MetaSPAdes v. 3.12; IDBA-UD v. 1.1.1;
                                     MEGAHIT v. 1.0.3
            Sequencing Technology :: 454; Illumina
            ##Assembly-Data-END##
FEATURES             Location/Qualifiers
     source          1..3577
                     /organism="Crucivirus-307"
                     /mol_type="genomic DNA"
                     /isolate="120_1246_IR2_3297"
                     /isolation_source="Water"
                     /country="Norway"
                     /collection_date=2011
     CDS             1..1497
                     /codon_start=1
                     /product="Putative CruV Capsid Protein"
                     /translation="MDAGNKMPAQSRYRNNRAYGGQGAYRGRGAYRGRGDYSVDNAPW
                     ANRGAGIGATIGGAFGIPPEVGSWLGRRAFYYPSKWFGGTGAYRPFGGRGAYRHMMYG
                     GQGAYRGRGAYRGRGAYRGRGAYRGRGSYTVENPVPQGTLAPTMPSFVGGSSDSVVLS
                     YKEYIGDIITSASANTFSIQDFLINPGNAQTFPWLANLCQGSYQHFQFLQCLFYFKSS
                     SADALNSTNTALGQVISAINYDVSDPTFRSRMEMENTNWSNSCKPSNDMEIPVECDPG
                     QTPARGLLHISPVGVIPSGADPKSYNLGRLSIATTGFQGTSVLIGSLYVVYKIRLFKP
                     ITLAPLSNGNRMLRVRGSVTNAAPFGSIYLSPTAGAECDTLGVTFSTTVMTLPKARLQ
                     NGMRFFVYLEFSGGSVAITLPSYTVSSNVTRHAAFGAAGTFQNSDTPAATTATQGSMA
                     AFWEITNDNADATLTLGGAGTLPTGCSLNVSIWQVNGTPSATLGIYVA*"
     misc_feature    7..26
                     /note="stem-loop"
     misc_feature    12..20
                     /note="nonanucleotide"
     CDS             complement(1777..3081)
                     /codon_start=1
                     /product="Putative Replication Associated Protein"
                     /translation="MPHTRRTCWKSVVLPTSALAGKLLLGRTTLNICIYIGTLVFLIF
                     FSMWEAPKVSLILKVDVDCPRQSTRPCRNPYQHHTSIEWKMNNNQRMPTSRNAMSPDD
                     KERLMAFIRRTQSDPKTPRYESNEVVSRIEQGNCKAASAGPPRESPAPNGSVEQPEER
                     DEEPDPYECGPDENYTVKKGGGGKPFCDWLTTYWPKTKEVYKTEDKLVKYFATQLEIC
                     PNTGKLHGHMYIELRAPRSRNWLKVLFADSTMHCEPRKGTQTQAKDYVTKELTRVPGT
                     TYEWGTKKRQGHRSDLDMMTAGALAGMSRMELLAEFKGGALRHLGMLERAQRCANGWD
                     ALDNAILNRRIEAERLDIHPQFVTTSFDSLKTENDETAARRREVFFGAEHWAIGVLEE
                     AEAEKSDDDNEEPPARLQIKDRRTMPRVNTLASRSSNDDDDG*"
ORIGIN
        1 ctggatgctg gtaataaaat gccagctcag tcccgttatc ggaacaatcg tgcgtatggt
       61 ggccagggcg cctaccgtgg taggggcgct tatcgtggtc gtggagacta cagtgtcgat
      121 aatgctccct gggctaatcg gggggctggt atcggtgcca ccatcggtgg cgctttcggt
      181 attccccctg aggttggctc ttggcttggc cgtagagctt tctattatcc ttccaagtgg
      241 tttggaggga cgggcgccta ccgtccgttc ggaggacgcg gcgcttatcg ccatatgatg
      301 tatggtggcc agggtgccta tcgtggccga ggcgcttaca ggggccgagg cgcttatcgc
      361 ggccgaggtg cctatcgcgg ccgcggctct tatactgttg agaacccagt cccgcagggg
      421 acgcttgcgc ccacaatgcc gtcgtttgtg ggtggttcga gtgatagtgt tgtgctttct
      481 tataaggagt atattggtga tatcatcacc agtgccagtg cgaatacgtt tagtattcag
      541 gactttttga tcaacccagg aaatgcgcag acgtttccgt ggctggcaaa tctttgccag
      601 ggttcgtatc agcacttcca atttttgcag tgtctctttt atttcaagag ctcgtcggcg
      661 gatgctctga attctacgaa tactgcgttg gggcaggtta tttcggctat taactatgat
      721 gtgtcagacc cgacattccg ctcgcgaatg gagatggaga atacgaactg gtctaattca
      781 tgcaagcctt cgaatgatat ggagatccca gtggagtgtg atccagggca gactcctgcc
      841 cgcggtctgc ttcatatttc acctgtgggt gttattcctt ctggtgcgga tccaaagtcg
      901 tacaatcttg gtcgtctttc gatcgctacg actggtttcc agggaaccag tgttcttatt
      961 gggtcgcttt atgtggttta caagatcagg ctgttcaagc ctatcaccct tgcgcctctt
     1021 tcaaacggta acagaatgct tcgcgttcgt ggctcggtta caaatgctgc gccttttggt
     1081 tcgatttacc ttagtccaac cgctggtgcg gagtgcgata cattgggcgt tacattcagt
     1141 accaccgtga tgacgttacc caaggctcgt cttcagaatg gtatgcgttt ctttgtctac
     1201 cttgagttca gcggaggttc agttgctatc actcttcctt cgtatacggt gtcgtccaac
     1261 gttactcgtc acgctgcgtt tggcgcggcg ggcacgttcc agaactctga tacgcctgct
     1321 gcgactacag ctacgcaggg tagtatggct gccttctggg agatcacgaa tgataacgct
     1381 gatgcgacgc ttactctggg tggagcgggc acgctcccta cgggatgttc gctcaatgtg
     1441 tcgatttggc aggtcaatgg tacgccctct gctacccttg gtatttatgt tgcttagaga
     1501 gtataactat atacttagcg tttctacagg cgcggggggg aggtgaacgg acagaaatga
     1561 gtttacgaat ttcagtatta ccgttcacct cttgtccaaa tattaagttt cactttaaat
     1621 gtggacccag aaataatttt aatttttcga tttgttaagg ttaagggtct cgtgtccata
     1681 ggcctatggc gcgggatccg tcttgggttc gcggaacaca aaaccgtatg aacgtagtga
     1741 tttgtatagg atatatacat tttagatgat atgggtttag ccatcatcat cgtcgttgct
     1801 acttcttgac gcgagggtat ttactcgtgg cattgtgcga cggtctttaa tttggagcct
     1861 cgcgggaggc tcctcattat cgtcgtcgct cttttcagct tcagcttctt cgaggacgcc
     1921 gatagcccag tgttctgccc cgaagaacac ctctcggcgt cgtgcggcgg tttcatcatt
     1981 ctcagtcttt aggctgtcga atgaggttgt gacgaattga gggtgtatgt caagacgttc
     2041 agcctcaatt ctgcgattca aaatggcgtt gtcgagggcg tcccagccat ttgcgcatcg
     2101 ttgggcgcgc tcaagcatcc ccagatggcg gagcgcgccg cctttgaact cagcgagtag
     2161 ttccattcta ctcattccag cgagggctcc agctgtcatc atatcgaggt cagagcgatg
     2221 tccttggcgc ttcttggtgc cccattcgta ggtggtgccg ggcacacggg ttagctcttt
     2281 cgttacatag tctttagcct gcgtttgggt gccctttcgg ggctcacaat gcatagtgct
     2341 gtccgcgaag aggactttca gccagttcct cgaacgtggg gctctgagct caatatacat
     2401 gtgaccgtgg agctttccag tattcgggca gatctcgagt tgagttgcaa agtatttgac
     2461 taacttgtct tcagtcttgt agacttcttt tgtcttaggc cagtatgtgg tcagccagtc
     2521 acagaagggt ttgcctccgc cacccttctt tactgtataa ttttcgtcag gcccgcactc
     2581 gtacgggtct ggttcttcgt ctctttcttc tggctgctcg acggaaccgt taggggcggg
     2641 gctttcccgc ggcggtcccg ctgaagcagc cttacaattc ccttgttcga ttctcgagac
     2701 tacttcgttg ctctcgtatc tcggggtttt agggtcgctt tgggttcttc ggatgaaggc
     2761 catcagtctt tccttgtcgt cagggctcat agcattgcgg gatgttggca tccgttggtt
     2821 gttgttcatt ttccattcaa tactcgtgtg atgctggtaa ggatttctac acggtcgagt
     2881 tgactgccgc gggcagtcaa catcgacctt caatatcagg gatacttttg gagcctccca
     2941 catggaaaag aatattaaaa aaaccagcgt gccaatatat atacatatat ttagagtggt
     3001 ccgtcccagt aacaacttgc ccgcgagagc gctcgtgggc aaaaccacag atttccaaca
     3061 agttcgcctt gtgtgtggca ttcagggcat tgtttgctct ctccacagtg cgcattaacg
     3121 caggagagtg ttaatcctcc gtgacttctt cctggtgata cgcgaagcat ctctccacat
     3181 gcttcgcaac tatattcttc tccagggaga taccagtatc gccagacagg cgtgtctggc
     3241 tcttggaaga ggtctcgttg gagcgcttgg atgttgagca taccagccat attcctcaca
     3301 gagcattccc aatcaaaatg ttttgtttcg ctacggcttc ccctgtggta gcgaactacg
     3361 atagggttcg ccattgttcc tgggaccgtg actactttta atttcgtgta tgtttctttg
     3421 tagatttcaa atctatcatt tacttcaact gcaagaagaa tcgttctctg ttcttgttgc
     3481 tgttcagcaa agtctaaacg tgccatcacg ttttcaatct tcttcatatg cactttctgt
     3541 tcctcaacgg ttgtagacat tttcttaacg gcactcg
//
LOCUS       CruV-308                3578 bp    DNA     circular     24-FEB-2020
DEFINITION  Crucivirus-308 isolate 001406_virusbaton.
ACCESSION   CruV-308
KEYWORDS    .
SOURCE      Crucivirus-308
  ORGANISM  Crucivirus-308
            Unclassified.
REFERENCE   1  (bases 1 to 3578)
  AUTHORS   de la Higuera,I., Kasun,G.W., Torrance,E.L., Pratt,A.A.,
            Maluenda,A., Colombet,J., Bisseux,M., Ravet,V., Dayaram,A.,
            Stainton,D., Kraberger,S., Zawar-Reza,P., Goldstien,S., Briskie,J.,
            White,R., Taylor,H., Gomez,C., Ainley,D.G., Harding,J.S.,
            Fontenele,R.S., Schreck,J., Ribeiro,S.G., Oswald,S.A., Arnold,J.M.,
            Varsani,A., Enault,F. and Stedman,K.M.
  TITLE     Direct Submission
  JOURNAL   Submitted (24-FEB-2020) Biology, Portland State University, 1719 SW
            10th Ave, Portland, Oregon 97201, USA
COMMENT     Bankit Comment: ALT EMAIL:ide@pdx.edu
            Bankit Comment: TOTAL # OF SEQS:451
            Bankit Comment: GAP: est. length - alignment

            ##Assembly-Data-START##
            Assembly Method       :: MetaSPAdes v. 3.12; IDBA-UD v. 1.1.1;
                                     MEGAHIT v. 1.0.3
            Sequencing Technology :: 454; Illumina
            ##Assembly-Data-END##
FEATURES             Location/Qualifiers
     source          1..3578
                     /organism="Crucivirus-308"
                     /mol_type="genomic DNA"
                     /isolate="001406_virusbaton"
                     /isolation_source="Water"
                     /country="France"
                     /collection_date=2013
     CDS             1..1383
                     /codon_start=1
                     /product="Putative CruV Capsid Protein"
                     /translation="MRKLKINFFCPPYFKDLFLKMSYSRKRRRTNTYRKVYGYGDYNY
                     GGRIARGIGGALGGLAGTLIPVPGASAVTKWMGDAAGGLFHKITGIGDYEDQKLMGIQ
                     TGGGSVTRGAVFGSGQDSVVITKREFLADIKSTISFSNQAFPINPGLVETFPWLSNMA
                     SLFEQWVPMGILFEFESQSGNAFSSTNNALGNIAMTIEYNPQQPNFGTMSQVLNNIWT
                     VSQKPTCSFTLPVECSPAERATTLFYVRDGGVPDNQDQKFYDLGNVQVSTEGMQQDGV
                     LIGKLWISYMIGFMKPKLPTNDTLGGEGFGINFQCGSANAGGTLPFGHATDLPEPNYD
                     SIGVTIHNSPTVQTITFPSNLSGTFSLEFTYRSLGATLFGPLTGVTASAGIDLDSFNN
                     LSNLSGGTMTSNGQIAYTLYFAIDSVLPVPKTIYLSPSVMSTICGNANGTVLSTVICN
                     QIQNVLSDMN*"
     CDS             complement(1541..2596)
                     /codon_start=1
                     /product="Putative Replication Associated Protein"
                     /translation="MSVVFPSASLSSFVGSNIILPTQPLMIPSDAKEINEERKKFRLQ
                     SKNLVLTWPKCYTSREQVREKCELHFAGNIRGYAIAQEAHKDGTPHIHVSMMLKERID
                     TKYENELDVLAGQHGNYQSARSLKKWTNYVLKSDVNAIKFGDILDDETWTTILQSSTV
                     KEMWENLVKNKPRDAAINGKRIVENYHYHKGLKRKRQEKPEKKLLPFKITIPMERWVI
                     DNVHRPNLFERTRCLVVHGGPNLGKTEWARSLFPKENVCYLQGIFNLKKLEEIEDLKL
                     IIFDDIEWHDLPQKKMLLQAKGETELTDKYMKKCTVHIHAPAIYLTNKDDFMSDVEFN
                     SYWKSELDVVYITSRLY*"
     misc_feature    2532..2557
                     /note="stem-loop"
     misc_feature    2542..2550
                     /note="nonanucleotide"
ORIGIN
        1 ttgaggaaat tgaagattaa ttttttttgt cctccttatt ttaaagatct ttttctaaaa
       61 atgtcttatt cacgaaagag acggcgtacc aacacgtacc gaaaggttta cggatatggc
      121 gattacaatt acggaggtag aattgctcga ggtattggtg gagctcttgg tggactcgcc
      181 ggtactctta ttccagttcc tggggcttct gcagtcacaa aatggatggg tgatgcagca
      241 ggaggtttat ttcacaaaat aaccggtatc ggagattatg aagatcaaaa acttatgggt
      301 attcaaaccg gaggtggatc cgttactcgc ggagcagttt ttggatcggg tcaagactct
      361 gttgtgatca ccaaacgaga gtttttggct gacatcaaga gtactattag tttcagcaac
      421 caggcatttc ctattaatcc tggattggtg gaaacgtttc catggctttc caacatggct
      481 tcacttttcg aacaatgggt cccaatggga attcttttcg agtttgaaag tcagtctggg
      541 aatgcctttt cttcaacgaa caatgctttg ggtaatattg caatgacaat tgaatacaat
      601 ccccaacaac ctaatttcgg aaccatgtct caagtgttga ataacatctg gactgtgtca
      661 cagaaaccaa cttgttcgtt tacacttcct gttgaatgct cacctgcaga acgagctact
      721 acactcttct acgtaagaga tggtggcgtt cctgacaacc aagatcagaa attctatgat
      781 ttgggtaatg ttcaagtatc aaccgaaggg atgcaacagg atggagtcct cattgggaaa
      841 ctctggattt cctacatgat tggcttcatg aaacctaaac ttccaacaaa tgatactttg
      901 ggtggagaag gtttcggaat taattttcaa tgtggatctg ctaatgcagg aggtacactt
      961 ccatttggtc atgcaacgga tctacctgaa ccgaactatg acagtattgg tgttactatc
     1021 cataattcac ctacagtcca aactattact tttccttcaa atctgagtgg taccttcagt
     1081 ttggagttca cttaccgttc tcttggagcc acattatttg gtccacttac gggagtgaca
     1141 gcaagtgctg gtatagattt ggattcattt aacaatttaa gtaatttgtc cggaggtaca
     1201 atgacttcca atggtcaaat agcgtacacc ttgtattttg ccattgattc cgtattacca
     1261 gttccaaaga ctatttatct gagtccttca gtaatgtcta caatttgtgg taatgcaaac
     1321 ggaactgttt tatctacagt tatatgcaac cagatacaga atgttctgag tgatatgaat
     1381 taaactcaaa tttttgcgca aacaaatgtt cgattagcac aggagcgaag cgacccctgt
     1441 aggagcgaag cgaccaaaag actaaaaatt ttaagggagc gacgggtccc ccgagtgtag
     1501 cgagtggggc ggtcgcctag aacaagggat tagcgcaaga ttagtagagt cttgacgtta
     1561 tgtaaactac atctaattct gatttccagt atgaattgaa ttctacgtct gacatgaaat
     1621 cgtccttgtt tgttaagtat attgcaggag catggatatg aacggtacac tttttcatat
     1681 acttgtctgt gagttccgtc tcacccttag cttgcaataa cattttcttt tgaggaagat
     1741 cgtgccattc gatatcgtca aaaatgatta acttcaggtc ttcaatttct tcaagtttct
     1801 tgagattgaa tattccttgt aagtaacata cattctcctt tggaaacaga ctacgagccc
     1861 attctgtttt tccaaggttg ggtccaccat gtacaacaag acaccttgtt ctttcaaaaa
     1921 gattaggtcg gtgtacattg tcaataaccc atctttccat tggaatggtt attttgaagg
     1981 gaagtaactt tttctcaggt ttttcctgac gtttcctctt caaccccttg tggtaatgat
     2041 aattttcaac aatacgcttt ccattgattg cagcgtctct tggtttattc ttcacaaggt
     2101 tttcccacat ctccttgact gtggaagatt gaaggatggt tgtccaagtt tcatcgtcca
     2161 agatgtcacc aaatttgatc gcattgacgt cagatttcaa cacataattg gtccactttt
     2221 tcaaagatct cgcagactgg tagtttccat gctgaccagc gaggacatcc aattcatttt
     2281 catacttggt atcaattcgt tccttcaaca tcatcgagac gtgtatgtga ggggtaccat
     2341 ctttatgggc ttcttgggca atagcgtaac cacgaatatt cccagcaaaa tgcaattcgc
     2401 atttctctcg aacttgttca cgactcgtat aacatttggg ccaggtaagt acaagatttt
     2461 tactttggag cctaaatttt ttccgttcct cgttgatttc ctttgcgtcg cttggaatca
     2521 tcagaggctg agtaggcaat ataatattac tgcctacgaa tgaggataat gaggcgctgg
     2581 ggaagacgac cgacattaca atactcgcgg agaaaaaata tttcacccct gatgaaattt
     2641 tacgtcctca ttctacaatt gaagggtttt agggaaaatc ttggacacat ctaagtttcc
     2701 cttatttcac tactgtcttt agatggggac taattaaacc tggaatttcg tttgcacaag
     2761 caagacaata ttcattagtc cagaggttag ggtatctgca aatgttacat ttcctctttt
     2821 tgggttccct aatcatccac ttgttgcatg ggaagtatgc ggagcatgtc tttcctgcac
     2881 actgatcgat atgtttcgga tccattgaaa aaaaaacttt ttgtctttct ttattaaaga
     2941 agttgatttt atttttcaaa atggcatatc gtcgaaaacg ccgtgcttat tcaaaacgcc
     3001 caaagggtct ccgttggaga gctgtttata aacctgtacg aaaacgtctg acacctgctc
     3061 agcgagcaaa tcgtactgct cgacaggtat tcagggccca tcagattatt caaagagctg
     3121 ggggtactat ttcataattt tttttgtctt cctttattaa aagaagtatt ctacaatgcc
     3181 tggtcgttgg agagccgttt acaagcctgt ttacaagcgt aggagacgaa gggtagctaa
     3241 gttaagacct aacttaccca ttatgaacac tattccacgt ttgcctgttt taccagcatt
     3301 aacgttacca ggaaatatgg atgtcccaat ggttgacttt gatcagaagt atggtattcc
     3361 attacctttg agtattccat tgatgaatcc cgcaggggga cctattttac aaccagcagc
     3421 ttctattgca ggtacactat tgcaaggagc tggtcaatta cttggttctg ctgtggagtc
     3481 ttacattcac aatcgtaagc gacaacgaga ggaaccgtat tcacttccaa gtccagaaaa
     3541 aatggcaaga cttatgccac caccagttgg tccgataa
//
LOCUS       CruV-309                3584 bp    DNA     circular     24-FEB-2020
DEFINITION  Crucivirus-309 isolate H6_8319.
ACCESSION   CruV-309
KEYWORDS    .
SOURCE      Crucivirus-309
  ORGANISM  Crucivirus-309
            Unclassified.
REFERENCE   1  (bases 1 to 3584)
  AUTHORS   de la Higuera,I., Kasun,G.W., Torrance,E.L., Pratt,A.A.,
            Maluenda,A., Colombet,J., Bisseux,M., Ravet,V., Dayaram,A.,
            Stainton,D., Kraberger,S., Zawar-Reza,P., Goldstien,S., Briskie,J.,
            White,R., Taylor,H., Gomez,C., Ainley,D.G., Harding,J.S.,
            Fontenele,R.S., Schreck,J., Ribeiro,S.G., Oswald,S.A., Arnold,J.M.,
            Varsani,A., Enault,F. and Stedman,K.M.
  TITLE     Direct Submission
  JOURNAL   Submitted (24-FEB-2020) Biology, Portland State University, 1719 SW
            10th Ave, Portland, Oregon 97201, USA
COMMENT     Bankit Comment: ALT EMAIL:ide@pdx.edu
            Bankit Comment: TOTAL # OF SEQS:451
            Bankit Comment: GAP: est. length - alignment

            ##Assembly-Data-START##
            Assembly Method       :: MetaSPAdes v. 3.12; IDBA-UD v. 1.1.1;
                                     MEGAHIT v. 1.0.3
            Sequencing Technology :: 454; Illumina
            ##Assembly-Data-END##
FEATURES             Location/Qualifiers
     source          1..3584
                     /organism="Crucivirus-309"
                     /mol_type="genomic DNA"
                     /isolate="H6_8319"
                     /isolation_source="Water"
                     /country="New Zealand"
                     /collection_date=2012
     CDS             join(complement(1..111),complement(3378..3584))
                     /codon_start=1
                     /product="putative ORF"
                     /translation="MLHRQNYTGAASVTANIRAPHIITQVLHGLRGGGVILDPPPLKA
                     TRKVRGPKAALPCRGPPAARRALRAPKTKALKNLSKAPKRPFGPLKPLKGPFGARKPP
                     TAA*"
     misc_feature    join(3572..3584,1..20)
                     /note="stem-loop"
     misc_feature    1..9
                     /note="nonanucleotide"
     CDS             190..1080
                     /codon_start=1
                     /product="Putative Replication Associated Protein"
                     /translation="MSLSRRYCFTVNNPSEEDCEQVYALSKHKHHKYLIVGSEKGECG
                     TPHLQGYIVFAKPVRFNTFKALIPRAHVEAAKGTSEQNRVYCMKEGDYEEYGDVPSDG
                     GQSLQVCEWDQARANAKLGKFDDIPSNLYIRYKNAFHSIFDDACNASECIVELDHLWI
                     TGPTGIGKSRYCFESFPNAYRKGANKWWCHYTNQDVVILEDVEPSQEKWIGYYLKIWS
                     DHYPFIAERKGGSRSIRPKRIVITSNYSIKEIFTDAKIRDPLLRRFKERTIKDGELIA
                     SISQEILFRQIGVDHYEAIE*"
     CDS             1096..2718
                     /codon_start=1
                     /product="Putative CruV Capsid Protein"
                     /translation="MVSTRSRRRSSGSSSSSSAFPLPVNPYVPPTQRSTQQGLTEAVA
                     EMSAETQTQPSSDGYISYYTGEPLTASGAVGPQNWVDPVTYRALRKKFTKKRKRTTTK
                     KRSTRRSSRSYGNSGRSLSGMGPIVITGRGGYWGDKFGEGASAAWKSLKRVTPEGTFS
                     RLGRAAGGAMFGKPGASVGNWLGGGVSGILGFGDYAVKKNDLMVLDEGVPVPTFQNMN
                     DGVIVCHREYIGDIGQTVNFTNTAFPIQPGDATTFPWLHQIASQFEQYEILGMLFQFR
                     STSSDFGSTANMALGTVIMATEYDSVDASFGSKLEMENAQYSMSGKPSHDILHPVECD
                     PGVVGPMGLKYVRTGPLPSGKDNRLYDHGNFQIATTGMPVGSGTIGELWVTYKIAFYK
                     PQFNHGVDIKSDFFFHSSADNSNMFGTVPIAVSNSSLGCTISGNTITFPKTAVVGQVY
                     MIAYQCNGGAAVTSAGGCIYTGCAAINRQASLPGAGTVTRYMENQYIRITNVSPTAAI
                     TVVWSAMTLPTAITDVRTVITQLDSDLLSQLG*"
ORIGIN
        1 tagtattacc ccgccccccc gtaacccatg taacacctgg gttattatat ggggagccct
       61 aatattagct gttacggagg ccgcgccagt gtaattttgt cgatgtaaca gggcttaggt
      121 cccataatgg tggtatcgtt atgggactct acatagggac taatggtgct ttagctcctg
      181 ccagtcgcta tgtcgctttc tcgccgttat tgctttacgg tgaacaatcc gagtgaggag
      241 gattgtgagc aggtttatgc cttgtctaag cataagcatc acaagtattt gattgttggt
      301 agcgaaaagg gggagtgtgg taccccgcat ttgcaaggct atattgtttt tgctaagcct
      361 gttcgattta atacgtttaa agctttaata ccgagggcgc atgtggaggc tgctaaaggt
      421 acttctgagc agaatcgggt atactgtatg aaggagggtg attatgagga atatggcgat
      481 gttccttcgg atggtggtca atcgctgcaa gtttgtgagt gggaccaagc tcgggcgaat
      541 gccaagcttg gaaaatttga tgatattcca tcgaatttgt acatccgtta taagaatgcc
      601 ttccacagta tatttgacga tgcatgtaac gcaagtgagt gtattgtcga attggaccat
      661 ttatggataa ctggtccaac cgggattggg aaatctcggt attgttttga atcgtttcct
      721 aatgcttatc gaaagggtgc aaacaagtgg tggtgtcatt acaccaacca ggatgttgtc
      781 attctcgaag atgtggagcc atcgcaagaa aaatggatag gatactatct gaagatttgg
      841 tccgaccatt atccttttat tgctgaaaga aagggaggat cgcgcagtat tcgacctaag
      901 cgaatagtga taacgtcaaa ttatagtatc aaggagattt ttactgatgc caagattcga
      961 gatccgcttt tgcgtcgttt taaagaacgt actattaagg atggggaatt aatagcgagt
     1021 atatcgcaag agattttatt tcggcagatt ggtgttgacc attacgaagc tattgaataa
     1081 agtgcgtatt catttatggt atcgacacgt agtcgacgtc gttctagtgg gtcgtcgtcg
     1141 tcgtcttctg cattccctct tcctgttaat ccgtacgttc ctccgacgca gcgttctact
     1201 cagcaaggtc ttacagaagc agttgctgaa atgtctgctg aaacgcagac tcagccgtca
     1261 tcggacggct acattagtta ctacaccgga gagcctttaa cggcgtctgg tgcagttggg
     1321 ccacaaaatt gggtggaccc tgttacttat cgtgcacttc gaaagaagtt caccaagaag
     1381 cgaaagcgta cgaccacgaa gaagaggtcc acgcggcgtt ccagtcgcag ttatggaaac
     1441 tctggtcgca gcctctctgg gatgggtcct attgttatta ctgggcgtgg tggttattgg
     1501 ggtgataagt ttggcgaggg tgcatctgca gcctggaaat ctttaaagcg cgtcactcct
     1561 gaaggcacgt ttagtcgttt aggtcgtgcc gctggtggtg ctatgtttgg gaagccagga
     1621 gccagtgttg ggaactggct cggtggaggc gtgtctggaa ttcttggatt tggagactac
     1681 gccgttaaga agaacgacct gatggttctt gatgaaggcg ttcctgttcc cacttttcag
     1741 aatatgaacg atggagttat tgtttgccat cgggagtata ttggtgatat tggtcaaact
     1801 gtgaacttta ctaacactgc ttttccgatt caacctggtg atgcaactac gttcccatgg
     1861 ttgcatcaga ttgcgtcgca atttgaacag tatgaaattt tagggatgtt atttcagttt
     1921 cgttcgacat cttctgattt cggttcaact gccaacatgg cgcttggcac tgttattatg
     1981 gctacggaat atgatagtgt tgatgcttct tttggaagca aattggaaat ggagaatgcc
     2041 cagtattcga tgagtgggaa accatctcat gatattctac atcctgttga atgtgatcct
     2101 ggtgttgttg ggccaatggg tttgaagtat gttcgaaccg gtcctcttcc atcaggaaag
     2161 gataatcgtt tgtatgatca tggcaatttt cagattgcca caacgggaat gcctgtaggg
     2221 tctggcacca ttggtgagtt gtgggttacc tacaaaattg cgttttataa gccgcaattt
     2281 aatcatggtg ttgatatcaa gagcgatttc ttctttcatt ctagtgctga taattctaat
     2341 atgtttggaa ctgttcctat tgctgttagt aacagttctc ttggatgtac tatttctggg
     2401 aatacgatta cgtttcccaa aacagctgtt gttggacaag tgtatatgat tgcatatcaa
     2461 tgcaatggtg gtgctgccgt cacaagtgcc ggagggtgta tttatacggg ttgtgctgct
     2521 attaatcggc aggcatcgtt gcctggtgca ggcaccgtta ctcgttatat ggagaatcag
     2581 tatattcgta taacgaacgt tagtccaaca gctgctatta cagtagtttg gtcagcaatg
     2641 actttaccaa ctgctattac ggatgttcgt actgtaatta cgcagttgga tagtgattta
     2701 ctctctcaac ttggttaaat aaataaggaa ttttattgta tagaccgtac gcgaatttcg
     2761 tagtttgggg ctccgggctc attacgcagc cggagccacg tgtaattgaa tttcgctttc
     2821 ttcttcttct tcagagaagg agagtcttct tgatcggatc gggaggtcgt cgtctcgtag
     2881 tcgaccgagt gcgagcgaca tttcccagct gtcgctccgg ctgtagagtt ctcgtcggaa
     2941 atatctgtac ggcctggcga atcggtggag atggtctgtg ttgttgaaga cccagacgac
     3001 gccggcaaca gagccataag tgacctaagg aattcagatt gattatgtca gactgctcta
     3061 atccgtactg accattaaag gttcaatgtt aagcactagt ctgataaggt aagtatggta
     3121 agtcttcgtt cctatatagg gaggctccgc cagaagtact ttatgggacc tttaaaactc
     3181 acagttgagt tttaatgcgt gactgggtac agtcaggaaa aaagtaaaag tagtgtgtgt
     3241 tttctatcgc aaagtaaagg ggcctacctt gtgtagggct ttatgttttt tgatgctgcg
     3301 ccccgtaggc cagtcatctt tgctttttag aacccctaaa ctttagggcg gccgaaggcc
     3361 gggccgccgt aggcggctta ggccgccgta ggcggcttcc gggccccgaa ggggcccttt
     3421 aggggcttta ggggcccgaa gggcctttta ggggctttag ataggttttt taaagccttt
     3481 gtttttgggg cccgcagggc cctgcgcgcc gcaggcggcc cccggcaggg gagcgcagct
     3541 tttgggccac ggacttttcg cgtggccttt agggggggcg ggtc
//
LOCUS       CruV-310                3584 bp    DNA     circular     24-FEB-2020
DEFINITION  Crucivirus-310 isolate SR5_34553.
ACCESSION   CruV-310
KEYWORDS    .
SOURCE      Crucivirus-310
  ORGANISM  Crucivirus-310
            Unclassified.
REFERENCE   1  (bases 1 to 3584)
  AUTHORS   de la Higuera,I., Kasun,G.W., Torrance,E.L., Pratt,A.A.,
            Maluenda,A., Colombet,J., Bisseux,M., Ravet,V., Dayaram,A.,
            Stainton,D., Kraberger,S., Zawar-Reza,P., Goldstien,S., Briskie,J.,
            White,R., Taylor,H., Gomez,C., Ainley,D.G., Harding,J.S.,
            Fontenele,R.S., Schreck,J., Ribeiro,S.G., Oswald,S.A., Arnold,J.M.,
            Varsani,A., Enault,F. and Stedman,K.M.
  TITLE     Direct Submission
  JOURNAL   Submitted (24-FEB-2020) Biology, Portland State University, 1719 SW
            10th Ave, Portland, Oregon 97201, USA
COMMENT     Bankit Comment: ALT EMAIL:ide@pdx.edu
            Bankit Comment: TOTAL # OF SEQS:451
            Bankit Comment: GAP: est. length - alignment

            ##Assembly-Data-START##
            Assembly Method       :: MetaSPAdes v. 3.12; IDBA-UD v. 1.1.1;
                                     MEGAHIT v. 1.0.3
            Sequencing Technology :: 454; Illumina
            ##Assembly-Data-END##
FEATURES             Location/Qualifiers
     source          1..3584
                     /organism="Crucivirus-310"
                     /mol_type="genomic DNA"
                     /isolate="SR5_34553"
                     /isolation_source="Water"
                     /country="New Zealand"
                     /collection_date=2012
     CDS             join(complement(1..111),complement(3378..3584))
                     /codon_start=1
                     /product="putative ORF"
                     /translation="MLHRQNYTSAASVTANIRAPHIITQVLHGLRGGGVILDPPPLKA
                     TRKVRGPKAALPCRGPPAARRALRAPKTKALKNLSKAPKRPFGPLKPLKGPFGARKPP
                     TAA*"
     misc_feature    join(3572..3584,1..20)
                     /note="stem-loop"
     misc_feature    1..9
                     /note="nonanucleotide"
     CDS             190..1080
                     /codon_start=1
                     /product="Putative Replication Associated Protein"
                     /translation="MSLSRRYCFTVNNPSEEDCEQVYALSKHKHHKYLIVGSEKGECG
                     TPHLQGYIVFAKPVRFNTFKALIPRAHVEAAKGSSEQNRVYCMKEGDYEEFGDVPTDG
                     GQTLQVCEWDQARANAKLGKFDDIPSNLYIRYKNAFHSIFDDACNASECIVELDHLWI
                     TGPTGIGKSRYCFESFPNAYRKGANKWWCHYTNQDVVILEDVEPSQEKWIGYYLKIWS
                     DHYPFIAERKGGSRSIRPKRIVITSNYSIKEIFTDAKIRDPLLRRFKERTIKNGELIA
                     SISQEILFRQIGVDHYEAIE*"
     CDS             1096..2718
                     /codon_start=1
                     /product="Putative CruV Capsid Protein"
                     /translation="MVSTRSRRRSSGSSSSSSAFPLPVNPYVPPTQRSTQQGLTEAVA
                     EMSAETQTQPSSDGYISYYTGEPLTASGAVGPQNWVDPVTYRALRKKFTKKRKRTTTK
                     KRSTRRSSRSYGSSGRSLSGMGPIVITGRGGYWGDKFGEGASAAWKSLKRVTPEGTFS
                     RLGRAAGGAMFGKPGASVGNWLGGGVSGILGFGDYAVKKNDLMVLDEGVPVPTFQNMN
                     DGVIVCHREYIGDIGQTVNFTNTVFPIQPGDATTFPWLHQIASQFEQYEILGMLFQFR
                     STSSDFGSTANMALGTVIMATEYDSVDASFGSKLEMENAQYSMSGKPSHDILHPVECD
                     PGVVGPMGLKYVRTGPVPSGKDNRLYDHGNFQIATTGMPVGSGTIGELWVTYKIAFYK
                     PQFNHGVDIKSDFFFHSSADNSNMFGTVPIAVSNSSLGCTISGNTITFPKTAVVGQVY
                     MIAYQCNGGAAVTSAGACTYTGCAVIHRQASLPGAGTVTRYLENQYIRITNVSPTAAI
                     TVIWSAMTLPTAITDVRTVITQLDSDLLSQLG*"
ORIGIN
        1 tagtattacc ccgccccccc gtaacccatg taacacctgg gttattatat ggggagccct
       61 aatattagct gttacggagg ccgcgctagt gtaattttgt cgatgtaaca gggcctaggt
      121 cccataatgg tggtatcgtt atgggactct acatagggac taatggtgct ttagctcctg
      181 ccagtcgcta tgtcgctttc tcgccgttat tgttttacgg tgaacaatcc gagtgaggag
      241 gattgtgagc aggtttatgc cttgtcgaag cataagcatc acaagtattt gattgttggg
      301 agcgaaaagg gggagtgtgg taccccgcat ttgcaaggct atattgtttt tgctaagcct
      361 gttcgattta atacgtttaa agctttaata ccgagggcgc atgtggaggc tgctaaaggt
      421 agttccgagc agaatcgggt atactgtatg aaggaaggtg attatgagga atttggtgat
      481 gttcctacgg atggtggtca aacgctgcaa gtttgtgagt gggatcaagc tcgtgctaac
      541 gcgaagcttg gaaaatttga tgatattcca tcgaatttgt acatccgtta taagaacgcc
      601 ttccacagta tatttgacga tgcatgtaac gcaagtgagt gtattgtcga attggaccat
      661 ttatggataa ctggtccaac cgggattggg aaatctcggt attgttttga atcgtttcct
      721 aatgcttatc gaaagggtgc aaacaagtgg tggtgtcatt acaccaacca ggatgttgtc
      781 attctcgaag atgtggagcc atcgcaagaa aaatggatag gatactatct gaagatttgg
      841 tccgaccatt atccttttat tgctgaaaga aagggaggat cgcgcagtat tcgaccgaag
      901 cgaatagtga taacgtcaaa ttatagtatc aaggagattt ttactgatgc aaagattcga
      961 gatccgcttt tgcgtcggtt taaagaacgt actatcaaga atggggaatt aatagcgagt
     1021 atatcgcaag agattttatt tcggcagatt ggtgttgacc attacgaagc tattgaataa
     1081 agtgcgtatt catttatggt atcgacacgt agtcgacgtc gttctagtgg gtcgtcgtcg
     1141 tcgtcttctg cattccctct tcctgttaat ccgtacgttc ctccgacgca gcgttctact
     1201 cagcaaggtc ttacagaagc agttgctgaa atgtctgctg aaacgcagac tcagccgtca
     1261 tcggacggct acattagtta ctacaccgga gagcctttaa cggcgtctgg tgcagttggg
     1321 ccacaaaatt gggtggaccc tgttacttat cgtgcacttc gaaagaagtt caccaagaag
     1381 cgaaagcgta cgaccacgaa gaagaggtcc acgcggcgtt ccagtcgcag ttatggaagc
     1441 tctggtcgca gcctctctgg gatgggtcct attgttatta ctgggcgtgg tggttattgg
     1501 ggtgataagt ttggcgaggg tgcatctgca gcctggaaat ctttaaagcg cgtcactcct
     1561 gaaggcacgt ttagtcgttt aggtcgtgcc gctggtggtg ctatgtttgg gaagccagga
     1621 gccagtgttg ggaactggct cggtggaggc gtgtctggaa ttcttggatt tggagactac
     1681 gccgttaaga agaacgacct gatggttctt gatgaaggcg ttcctgttcc cacttttcag
     1741 aatatgaacg atggagttat tgtttgccat cgggagtata ttggtgatat tggtcaaact
     1801 gtgaacttta ctaacactgt ttttccgatt caacctggtg atgcaactac gtttccatgg
     1861 ttgcatcaga ttgcgtcgca atttgaacag tatgaaattt tagggatgtt atttcagttt
     1921 cgttcgacat cttctgattt cggttcaact gccaacatgg cgcttggcac tgttattatg
     1981 gctacggaat atgatagtgt tgatgcttct tttggaagca aattggaaat ggagaatgca
     2041 cagtattcga tgagtgggaa accatctcat gatattctac atcctgttga atgtgatcct
     2101 ggtgttgttg ggccaatggg tttgaagtat gttcgaaccg gtcctgttcc atcaggaaag
     2161 gataatcgtt tgtatgatca tggcaatttt cagattgcca caacgggaat gcctgtaggg
     2221 tctggcacca ttggtgagtt gtgggttacc tacaaaattg cgttttataa gccgcaattt
     2281 aatcatggtg ttgatatcaa gagcgatttc ttctttcatt ctagtgctga taattctaat
     2341 atgtttggaa ctgttcctat tgctgttagt aacagttctc ttggatgtac tatttctggg
     2401 aatacgatta cgtttcccaa aacagctgtt gttggacaag tgtatatgat tgcatatcaa
     2461 tgcaatggtg gtgctgccgt cacaagtgcc ggagcgtgta cttatacggg ttgtgctgtt
     2521 attcatcggc aggcatcgtt gcctggtgca ggcaccgtta ctcgttattt ggagaatcag
     2581 tatattcgta taacgaacgt tagtccaaca gctgctatta cagtaatttg gtcagcaatg
     2641 actttaccaa ctgctattac ggatgttcgt actgtaatta cgcagttgga tagtgattta
     2701 ctctctcaac ttggttaaat aaataaggaa ttttattgta tagaccgtac gcgaatttcg
     2761 tagtttgggg ctccgggctc attacgcagc cggagccacg tgtaattgaa tttcgctttc
     2821 ttcttcttct tcagagaagg agagtcttct tgatcggatc gggaggtcgt cgtctcgtag
     2881 tcgaccgagt gcgagcgaca tttcccagct gtcgctccgg ctgtagagtt ctcgtcggaa
     2941 atatctgtac ggcctggcga atcggtggag atggtctgtg ttgttgaaga cccagacgac
     3001 gccggcaaca gagccataag tgacctaagg aattcagatt gattatgtca gactgctcta
     3061 atccgtactg accattaaag gttcaatgtt aagcactagt ctgataaggt aagtatggta
     3121 agtcttcgtt cctatatagg gaggctccgc cagaagtact ttatgggacc tttaaaactc
     3181 acagtcgagt tttaatgcgt gactgggtac agtcaggaaa aaagtaaaag tagtgtgtgt
     3241 tttctatcgc aaagtaaagg ggcctacctt gtgtagggct ttatgttttt tgatgctgcg
     3301 ccccgtaggc cagtcatctt tgctttttag aacccctaaa ctttagggcg gccgaaggcc
     3361 gggccgccgt aggcggctta ggccgccgta ggcggcttcc gggccccgaa ggggcccttt
     3421 aggggcttta ggggcccgaa gggcctttta ggggctttag ataggttttt taaagccttt
     3481 gtttttgggg cccgcagggc cctgcgcgcc gcaggcggcc cccggcaggg gagcgcagct
     3541 tttgggccac ggacttttcg cgtggccttt agggggggcg ggtc
//
LOCUS       CruV-311                3584 bp    DNA     circular     24-FEB-2020
DEFINITION  Crucivirus-311 isolate SR5_34554.
ACCESSION   CruV-311
KEYWORDS    .
SOURCE      Crucivirus-311
  ORGANISM  Crucivirus-311
            Unclassified.
REFERENCE   1  (bases 1 to 3584)
  AUTHORS   de la Higuera,I., Kasun,G.W., Torrance,E.L., Pratt,A.A.,
            Maluenda,A., Colombet,J., Bisseux,M., Ravet,V., Dayaram,A.,
            Stainton,D., Kraberger,S., Zawar-Reza,P., Goldstien,S., Briskie,J.,
            White,R., Taylor,H., Gomez,C., Ainley,D.G., Harding,J.S.,
            Fontenele,R.S., Schreck,J., Ribeiro,S.G., Oswald,S.A., Arnold,J.M.,
            Varsani,A., Enault,F. and Stedman,K.M.
  TITLE     Direct Submission
  JOURNAL   Submitted (24-FEB-2020) Biology, Portland State University, 1719 SW
            10th Ave, Portland, Oregon 97201, USA
COMMENT     Bankit Comment: ALT EMAIL:ide@pdx.edu
            Bankit Comment: TOTAL # OF SEQS:451
            Bankit Comment: GAP: est. length - alignment

            ##Assembly-Data-START##
            Assembly Method       :: MetaSPAdes v. 3.12; IDBA-UD v. 1.1.1;
                                     MEGAHIT v. 1.0.3
            Sequencing Technology :: 454; Illumina
            ##Assembly-Data-END##
FEATURES             Location/Qualifiers
     source          1..3584
                     /organism="Crucivirus-311"
                     /mol_type="genomic DNA"
                     /isolate="SR5_34554"
                     /isolation_source="Water"
                     /country="New Zealand"
                     /collection_date=2012
     CDS             join(complement(1..111),complement(3378..3584))
                     /codon_start=1
                     /product="putative ORF"
                     /translation="MLHRQNYTSAASVTANIRAPHIITQVLHGLRGGGVILDPPPLKA
                     TRKVRGPKAALPCRGPPAARRALRAPKTKALKNLSKAPKRPFGPLKPLKGPFGARKPP
                     TAA*"
     misc_feature    join(3572..3584,1..20)
                     /note="stem-loop"
     misc_feature    1..9
                     /note="nonanucleotide"
     CDS             190..1080
                     /codon_start=1
                     /product="Putative Replication Associated Protein"
                     /translation="MSLSRRYCFTVNNPSEEDCEQVYALSKHKHHKYLIVGSEKGECG
                     TPHLQGYIVFAKPVRFNTFKALIPRAHVESAKGTSEQNRVYCMKEGDYEEFGDVPTDG
                     GQSLQVCEWDQARANAKLGKFDDIPSNLYIRYKNAFHSIFDDACNASECIVELDHLWI
                     TGPTGIGKSRYCFESFPNAYRKGANKWWCHYTNQDVVILEDVEPSQEKWIGYYLKIWS
                     DHYPFIAERKGGSRSIRPKRIVITSNYSIKEIFTDAKIRDPLLRRFKERTIKNGELIA
                     SISQEILFRQIGVDHYEAIE*"
     CDS             1096..2718
                     /codon_start=1
                     /product="Putative CruV Capsid Protein"
                     /translation="MVSTRSRRRSSGSSSSSSAFPLPVNPYVPPTQRSTQQGLTEAVA
                     EMSAETQTQPSSDGYISYYTGEPLTASGAVGPQNWVDPVTYRALRKKFTKKRKRTTTK
                     KRSTRRSSRSYGSSGRSLSGMGPIVITGRGGYWGDKFGEGASAAWKSLKRVTPEGTFS
                     RLGRAAGGAMFGKPGASVGNWLGGGVSGILGFGDYAVKKNDLMVLDEGVPVPTFQNMN
                     DGVIVCHREYIGDIGQTVNFTNTVFPIQPGDATTFPWLHQIASQFEQYEILGMLFQFR
                     STSSDFGSTANMALGTVIMATEYDSVDASFGSKLEMENAQYSMSGKPSHDILHPVECD
                     PGVVGPMGLKYVRTGPVPSGKDNRLYDHGNFQIATTGMPVGSGTIGELWVTYKIAFYK
                     PQFNHGVDIKSDFFFHSSADNSNMFGTVPIAVSNSSLGCTISGNTITFPKTAVVGQVY
                     MIAYQCNGGAAVTSAGACTYTGCAVIHRQASLPGAGTVTRYLENQYIRITNVSPTAAI
                     TVIWSAMTLPTAITDVRTVITQLDSDLLSQLG*"
ORIGIN
        1 tagtattacc ccgccccccc gtaacccatg taacacctgg gttattatat ggggagccct
       61 aatattagct gttacggagg ccgcgctagt gtaattttgt cgatgtaaca gggcctaggt
      121 cccataatgg tggtatcgtt atgggactct acatagggac taatggtgct ttagctcctg
      181 ccagtcgcta tgtcgctttc tcgccgttat tgttttacgg tgaacaatcc gagtgaggag
      241 gattgtgagc aggtttatgc cttgtcgaag cataagcatc acaagtattt gattgttggg
      301 agcgaaaagg gggagtgtgg taccccgcat ttgcaaggct atattgtttt tgctaagcct
      361 gttcgattta atacgtttaa agctttaata ccgagggcgc atgtggagtc tgctaaaggt
      421 acttctgagc agaatcgggt atactgtatg aaggagggtg attatgagga atttggtgat
      481 gttcctacgg atggtggtca atcgctgcaa gtttgtgagt gggaccaagc tcgtgctaac
      541 gcgaagcttg gaaaatttga tgatattcca tcgaatttgt acatccgtta taagaacgcc
      601 ttccacagta tatttgacga tgcatgtaac gcaagtgagt gtattgtcga attggaccat
      661 ttatggataa ctggtccaac cgggattggg aaatctcggt attgttttga atcgtttcct
      721 aatgcttatc gaaagggtgc aaacaagtgg tggtgtcatt acaccaacca ggatgttgtc
      781 attctcgaag atgtggagcc atcgcaagaa aaatggatag gatactatct gaagatttgg
      841 tccgaccatt atccttttat tgctgaaaga aagggaggat cgcgcagtat tcgaccgaag
      901 cgaatagtga taacgtcaaa ttatagtatc aaggagattt ttactgatgc aaagattcga
      961 gatccgcttt tgcgtcggtt taaagaacgt actatcaaga atggggaatt aatagcgagt
     1021 atatcgcaag agattttatt tcggcagatt ggtgttgacc attacgaagc tattgaataa
     1081 agtgcgtatt catttatggt atcgacacgt agtcgacgtc gttctagtgg gtcgtcgtcg
     1141 tcgtcttctg cattccctct tcctgttaat ccgtacgttc ctccgacgca gcgttctact
     1201 cagcaaggtc ttacagaagc agttgctgaa atgtctgctg aaacgcagac tcagccgtca
     1261 tcggacggct acattagtta ctacaccgga gagcctttaa cggcgtctgg tgcagttggg
     1321 ccacaaaatt gggtggaccc tgttacttat cgtgcacttc gaaagaagtt caccaagaag
     1381 cgaaagcgta cgaccacgaa gaagaggtcc acgcggcgtt ccagtcgcag ttatggaagc
     1441 tctggtcgca gcctctctgg gatgggtcct attgttatta ctgggcgtgg tggttattgg
     1501 ggtgataagt ttggcgaggg tgcatctgca gcctggaaat ctttaaagcg cgtcactcct
     1561 gaaggcacgt ttagtcgttt aggtcgtgcc gctggtggtg ctatgtttgg gaagccagga
     1621 gccagtgttg ggaactggct cggtggaggc gtgtctggaa ttcttggatt tggagactac
     1681 gccgttaaga agaacgacct gatggttctt gatgaaggcg ttcctgttcc cacttttcag
     1741 aatatgaacg atggagttat tgtttgccat cgggagtata ttggtgatat tggtcaaact
     1801 gtgaacttta ctaacactgt ttttccgatt caacctggtg atgcaactac gtttccatgg
     1861 ttgcatcaga ttgcgtcgca atttgaacag tatgaaattt tagggatgtt atttcagttt
     1921 cgttcgacat cttctgattt cggttcaact gccaacatgg cgcttggcac tgttattatg
     1981 gctacggaat atgatagtgt tgatgcttct tttggaagca aattggaaat ggagaatgca
     2041 cagtattcga tgagtgggaa accatctcat gatattctac atcctgttga atgtgatcct
     2101 ggtgttgttg ggccaatggg tttgaagtat gttcgaaccg gtcctgttcc atcaggaaag
     2161 gataatcgtt tgtatgatca tggcaatttt cagattgcca caacgggaat gcctgtaggg
     2221 tctggcacca ttggtgagtt gtgggttacc tacaaaattg cgttttataa gccgcaattt
     2281 aatcatggtg ttgatatcaa gagcgatttc ttctttcatt ctagtgctga taattctaat
     2341 atgtttggaa ctgttcctat tgctgttagt aacagttctc ttggatgtac tatttctggg
     2401 aatacgatta cgtttcccaa aacagctgtt gttggacaag tgtatatgat tgcatatcaa
     2461 tgcaatggtg gtgctgccgt cacaagtgcc ggagcgtgta cttatacggg ttgtgctgtt
     2521 attcatcggc aggcatcgtt gcctggtgca ggcaccgtta ctcgttattt ggagaatcag
     2581 tatattcgta taacgaacgt tagtccaaca gctgctatta cagtaatttg gtcagcaatg
     2641 actttaccaa ctgctattac ggatgttcgt actgtaatta cgcagttgga tagtgattta
     2701 ctctctcaac ttggttaaat aaataaggaa ttttattgta tagaccgtac gcgaatttcg
     2761 tagtttgggg ctccgggctc attacgcagc cggagccacg tgtaattgaa tttcgctttc
     2821 ttcttcttct tcagagaagg agagtcttct tgatcggatc gggaggtcgt cgtctcgtag
     2881 tcgaccgagt gcgagcgaca tttcccagct gtcgctccgg ctgtagagtt ctcgtcggaa
     2941 atatctgtac ggcctggcga atcggtggag atggtctgtg ttgttgaaga cccagacgac
     3001 gccggcaaca gagccataag tgacctaagg aattcagatt gattatgtca gactgctcta
     3061 atccgtactg accattaaag gttcaatgtt aagcactagt ctgataaggt aagtatggta
     3121 agtcttcgtt cctatatagg gaggctccgc cagaagtact ttatgggacc tttaaaactc
     3181 acagtcgagt tttaatgcgt gactgggtac agtcaggaaa aaagtaaaag tagtgtgtgt
     3241 tttctatcgc aaagtaaagg ggcctacctt gtgtagggct ttatgttttt tgatgctgcg
     3301 ccccgtaggc cagtcatctt tgctttttag aacccctaaa ctttagggcg gccgaaggcc
     3361 gggccgccgt aggcggctta ggccgccgta ggcggcttcc gggccccgaa ggggcccttt
     3421 aggggcttta ggggcccgaa gggcctttta ggggctttag ataggttttt taaagccttt
     3481 gtttttgggg cccgcagggc cctgcgcgcc gcaggcggcc cccggcaggg gagcgcagct
     3541 tttgggccac ggacttttcg cgtggccttt agggggggcg ggtc
//
LOCUS       CruV-312                3593 bp    DNA     circular     24-FEB-2020
DEFINITION  Crucivirus-312 isolate SR5_33644.
ACCESSION   CruV-312
KEYWORDS    .
SOURCE      Crucivirus-312
  ORGANISM  Crucivirus-312
            Unclassified.
REFERENCE   1  (bases 1 to 3593)
  AUTHORS   de la Higuera,I., Kasun,G.W., Torrance,E.L., Pratt,A.A.,
            Maluenda,A., Colombet,J., Bisseux,M., Ravet,V., Dayaram,A.,
            Stainton,D., Kraberger,S., Zawar-Reza,P., Goldstien,S., Briskie,J.,
            White,R., Taylor,H., Gomez,C., Ainley,D.G., Harding,J.S.,
            Fontenele,R.S., Schreck,J., Ribeiro,S.G., Oswald,S.A., Arnold,J.M.,
            Varsani,A., Enault,F. and Stedman,K.M.
  TITLE     Direct Submission
  JOURNAL   Submitted (24-FEB-2020) Biology, Portland State University, 1719 SW
            10th Ave, Portland, Oregon 97201, USA
COMMENT     Bankit Comment: ALT EMAIL:ide@pdx.edu
            Bankit Comment: TOTAL # OF SEQS:451
            Bankit Comment: GAP: est. length - alignment

            ##Assembly-Data-START##
            Assembly Method       :: MetaSPAdes v. 3.12; IDBA-UD v. 1.1.1;
                                     MEGAHIT v. 1.0.3
            Sequencing Technology :: 454; Illumina
            ##Assembly-Data-END##
FEATURES             Location/Qualifiers
     source          1..3593
                     /organism="Crucivirus-312"
                     /mol_type="genomic DNA"
                     /isolate="SR5_33644"
                     /isolation_source="Water"
                     /country="New Zealand"
                     /collection_date=2012
     CDS             1..906
                     /codon_start=1
                     /product="Putative Replication Associated Protein"
                     /translation="METVAKDTQVYRYDFTSYFDDIEECKKFLRTHCSKWTFQQEKCP
                     STGKLHLQGRVSLKTKKTLKSAALLFKALNNAHISKTSKNCTDDAYVMKNESRVAGPW
                     KHDDKPPPYIPIQLRITEFLPWQKAVIEQLLNFRKINMVLDKKGNIGKTTLVGNLCCQ
                     NKARQIPFCNDFKDIMRMMMCMEEDHGSCPLVFIDLPRAIKKDHLYQMYSGIEVIKGG
                     YLFDDRYHLKFKYIDSPNIWVFTNVEPEWSFMSSDRWIVWEVNKDFELVQKEPDGLKK
                     WKDSNEGCEDIITLANKLKSELSTK*"
     CDS             complement(1198..2589)
                     /codon_start=1
                     /product="Putative CruV Capsid Protein"
                     /translation="MFYKKHMSTKYSDAQRADYWKAKAMAKPKSKARPAKRTYKKKAY
                     RKKAYSRRGGSGGNRSINISGLGDYQMNEGDNFGRRYGGLVGSKLGEYAGGALHGLMT
                     SFTGLGDYKVVHNSLMEGGMMPPIINPHTTGGDVFRRSEYLGDLISASTANTFKLQSF
                     PVNAGLESTFQWLSQIAGNYTQYQFEGIYFEFRSMSADALNSTNTALGQVVMAANYNA
                     SEDNFTNKQEMENYYSGVSGKPSGNIRYFVECHPAVNVLTDLYVRQGEVPPGEDQRLF
                     DLCNFQIATNGCQGTNVNLGEIWVTYQVCLRKPRLFAALGEYNSWASMVPNTLDFSNA
                     NPLNTNWFISPTSNIIGLSFTSTTIRWTAPTLPQVYNVFIRWVGSGSVALTTPSFLGS
                     NGLTQRNLYQTPGATTTTSSYTISFQVSYNPANNTSGGDPTVTVGTGGVLPTTPTSFV
                     LQITQMPNTTYGV*"
     CDS             3143..3505
                     /codon_start=1
                     /product="putative ORF"
                     /translation="MCVDEIKKINVIKIFFIKYFLYIKMEVNLKYDYIYGISEGSYND
                     MSPCARASVILDNWKCELENCRQLKPRDATHPDFEDELEDYQTWKSEVLDHIAIYQAF
                     IKGWFMCLEYNKNNLIKS*"
ORIGIN
        1 atggagacag tggctaaaga cacacaagtc tacagatacg actttacatc gtattttgat
       61 gatatcgaag aatgtaaaaa attcttacga actcattgtt caaaatggac ctttcagcaa
      121 gaaaaatgtc catcaaccgg taaattacat ttacaaggta gagtgtctct aaagacaaaa
      181 aagaccctaa aatcagctgc actgcttttt aaggctctta ataatgctca tatttctaaa
      241 acaagtaaaa attgtactga tgacgcttac gtcatgaaaa atgagtctag agtcgccggt
      301 ccttggaaac acgacgacaa accacctccg tatattccaa tacaacttag aattacggag
      361 ttccttccat ggcaaaaggc tgttattgaa caacttttaa actttcgtaa aatcaatatg
      421 gttttagaca aaaaaggtaa tattggaaag actacattag ttggaaactt atgttgtcag
      481 aacaaagcaa ggcaaatacc tttttgtaat gattttaaag acatcatgcg tatgatgatg
      541 tgcatggaag aagatcatgg atcttgtcca ctagtcttta ttgatctccc aagggcgatc
      601 aaaaaagatc atttatatca aatgtatagt ggaatagaag taataaaagg aggatatctg
      661 ttcgatgaca gatatcacct taaatttaaa tacattgact cacccaatat ttgggtattc
      721 accaatgttg aaccagaatg gagctttatg agctccgata gatggatagt gtgggaagtc
      781 aataaagact ttgaactggt tcagaaagag cccgatgggc ttaaaaagtg gaaagattcg
      841 aatgaagggt gtgaagacat tattacctta gctaataagt taaaatcgga actttccaca
      901 aaataaatat atcaattagg atatattttt ttaatccatg tcctggaggc catagattga
      961 gttggcgtgg gtacaagtac cgcactgtaa tactgcaact caatctatgc gctacgcgga
     1021 accaaaaagc gctttgcgct ttttgcttct atatatatat tattttaagt gtatggataa
     1081 atggataaaa tgagtgtttg taccatttcc cattaagttt ttttgttttg ctgtggctaa
     1141 actttggata cggctggcta aaatagccgt gtaatatata tccctggata tatattatca
     1201 aacaccataa gtcgtgtttg gcatttgtgt tatctgtaaa acaaatgaag taggggtagt
     1261 aggtaaaacc ccaccggtac caactgttac agttgggtca ccgccagatg tattattagc
     1321 aggattataa ctgacttgaa aagaaatagt ataagatgag gtagtagttg tagcaccggg
     1381 agtttgatac aaattacgtt gcgtaagacc attactacca agaaaagatg gagtagtaag
     1441 tgctacagaa ccagaaccga cccatctaat aaaaacatta taaacttgtg gtaaagttgg
     1501 agcagtccat ctaatagtag tactagtaaa agataaacct ataatgtttg atgttggact
     1561 gataaaccaa tttgtattta atggatttgc attagaaaaa tccaatgtat taggaaccat
     1621 agaagcccat gagttatatt ctcccaaagc agcaaataac ctaggctttc tcaagcaaac
     1681 ttgataagtg acccaaattt cccctaaatt gacgtttgta ccttgacaac cgtttgtagc
     1741 aatttggaaa ttacataaat cgaagagtct ttgatcttct cctggaggca cttcaccctg
     1801 acgaacatat aaatctgtta atacgttaac agcagggtga cattcgacaa aataccttat
     1861 gttaccagaa ggttttcccg aaacaccgga ataataattt tccatctctt gtttgttcgt
     1921 aaaattatct tctgacgcgt tgtaattggc tgccattaca acttgcccta aagcggtgtt
     1981 agtagaattt aacgcatcag ctgacattga gcgaaattca aaataaatac cctcaaattg
     2041 atactgtgta taatttccgg ctatttgact cagccattga aaagtacttt ccaaaccagc
     2101 attcacagga aacgactgaa gtttgaaagt attggctgtg gaagcactaa ttaaatcccc
     2161 caagtactcg cttcgtctaa agacatcacc tcctgtggta tggggattta ttattggggg
     2221 catcattcct ccttccatga gagaattatg gacgacctta tagtcgccta aacccgtaaa
     2281 tgacgtcatt aaaccgtgta atgcgcctcc agcgtattcc ccgagttttg atcctacaag
     2341 accaccataa cgtctaccga agttgtcacc ttcgttcatt tggtaatcac caagtcctga
     2401 tatgtttatt gagcgattac ctccggatcc acctcgacgg gaataagctt tctttcgata
     2461 agccttcttc ttgtaagtcc ttttagctgg tctagctttg gacttaggct tagccatagc
     2521 cttagccttc caatagtctg ctctttgagc gtctgagtac ttggtggaca tgtgtttttt
     2581 atagaacatg aaaaattaaa aaaaaataaa ttttttgttt ttttatttgt aattatggaa
     2641 tggcgactca cgctccgctt ccggcgccgt attgtcgggg acgtaaccgc ccgccaaggt
     2701 taatgataaa tatatgaaaa gagtatgttt agtagtgttt tatatatgta tacacaaatt
     2761 tttggtgaat ttgtgatttt tgtttttgaa ttgaactgac ggaagcgttg cttaaggggc
     2821 ttcgcccctg gtcaaaaaaa agtggtataa agaaatgtgt tgtatataaa aaataacagt
     2881 gggcaatggc taacatggct acaatggcta catacggacc tgaaatgtat atcggtggtc
     2941 atttcattga ggatgatgaa atagaagatg cgataattac ttatcttaaa agaacaactg
     3001 gatatcatat aaatatgata gatgcatgct ttgatgttat aacagataaa ttgttagatg
     3061 ctcctggagc acctaatagt aaatattctc aaaatcatat tgtattagaa atagctgatg
     3121 atttgatgaa attaatcgac acatgtgtgt ggatgagatt aagaagatta atgttataaa
     3181 aatatttttt ataaaatatt ttttatatat aaaaatggaa gtcaacttga agtacgacta
     3241 tatttatggt ataagcgaag gttcttataa tgacatgagt ccctgtgcca gagcctctgt
     3301 cattctagac aactggaagt gtgaattaga aaattgtcgc caattaaaac ctcgcgatgc
     3361 aactcaccca gattttgagg atgagttaga agattaccaa acttggaaaa gtgaagtttt
     3421 ggatcatata gctatttatc aggcctttat aaagggttgg tttatgtgtt tggaatataa
     3481 taaaaacaac ctgataaaat cataaaaata taattttcaa ttatattttt ttttgaattt
     3541 atttcaaaaa tatggtataa agaaataaat ttagccataa aaaagtggct aac
//
LOCUS       CruV-313                3594 bp    DNA     circular     24-FEB-2020
DEFINITION  Crucivirus-313 isolate 120_125_SvL2_3298.
ACCESSION   CruV-313
KEYWORDS    .
SOURCE      Crucivirus-313
  ORGANISM  Crucivirus-313
            Unclassified.
REFERENCE   1  (bases 1 to 3594)
  AUTHORS   de la Higuera,I., Kasun,G.W., Torrance,E.L., Pratt,A.A.,
            Maluenda,A., Colombet,J., Bisseux,M., Ravet,V., Dayaram,A.,
            Stainton,D., Kraberger,S., Zawar-Reza,P., Goldstien,S., Briskie,J.,
            White,R., Taylor,H., Gomez,C., Ainley,D.G., Harding,J.S.,
            Fontenele,R.S., Schreck,J., Ribeiro,S.G., Oswald,S.A., Arnold,J.M.,
            Varsani,A., Enault,F. and Stedman,K.M.
  TITLE     Direct Submission
  JOURNAL   Submitted (24-FEB-2020) Biology, Portland State University, 1719 SW
            10th Ave, Portland, Oregon 97201, USA
COMMENT     Bankit Comment: ALT EMAIL:ide@pdx.edu
            Bankit Comment: TOTAL # OF SEQS:451
            Bankit Comment: GAP: est. length - alignment

            ##Assembly-Data-START##
            Assembly Method       :: MetaSPAdes v. 3.12; IDBA-UD v. 1.1.1;
                                     MEGAHIT v. 1.0.3
            Sequencing Technology :: 454; Illumina
            ##Assembly-Data-END##
FEATURES             Location/Qualifiers
     source          1..3594
                     /organism="Crucivirus-313"
                     /mol_type="genomic DNA"
                     /isolate="120_125_SvL2_3298"
                     /isolation_source="Water"
                     /country="Norway"
                     /collection_date=2012
     misc_feature    join(3581..3594,1..23)
                     /note="stem-loop"
     misc_feature    join(complement(1..8),3594)
                     /note="nonanucleotide"
     misc_feature    1..9
                     /note="nonanucleotide"
     CDS             154..1458
                     /codon_start=1
                     /product="Putative Replication Associated Protein"
                     /translation="MSSRGRGGAPRQEKKGVSITWTLNNYTDAEVSDIKANWPKMAKY
                     VCFGKEIGGAELTPHLQGYMSLKKQTAYSSIHKDFPGMIRAHFLNAGGDHVQNRNYCG
                     KGAMSTIEYDRYRKVAVPNSKPKVFKEGDRYGQDVRSHPEYGIGADFWESGPLSEAIQ
                     PGKSKDLHEFVEALGSKSITSIVRSSMENKIVFLKHNKYLEALESKIVRTRSAKDGFP
                     ETHWICGKTGQAKTRLAGEFARRYMGADDDAEDQELFALIRPGRAGLAPWFGNWEYQP
                     VAIIDEVRAKSHDFVWMLGFCDRYDQVCEPKGSQKPWNAEVVIYTSPRSIDDTYRGRT
                     DEELDQLHRRIRESGGREWRFPDDAAEWLERLGRLQPRSGTGLADPRIRPQYASAVVG
                     PLRSNEVRLAEPADEESLRSGTEMMDEIDAEIQRLVDEHFAD*"
     CDS             1502..3253
                     /codon_start=1
                     /product="Putative CruV Capsid Protein"
                     /translation="MSYVPDFTESWLHDPATNLEALKMSEEYIPQTLLPFPPSARQDY
                     PRVIRDRPSARGQSARKKARADVQQIAAQMFVEELKSEGRSIPQAVSDQARGITSKST
                     KMSLPPKDYAASLSRDDLEKKYAASIERSKGKRKTTGKGRAGASKKRRYSSSRSSGRY
                     GGTNIVYPNVVGRGAYGIKGGLNFGHEDDWFRGGVGGYYNSDRAAGGTVSGRGAYNIK
                     RNSLVVDEGQGPPLIENLTNGQVVIRHREYLKDMTCPTGAPSPYNINFQEKINPGNPA
                     MFPWLSQIAPNFQEWEALGMIFEVKTMSSDYTQNVSMGTVFAATDYNTQAPAPINKQQ
                     LENMEFSQSCKPSCSIMMPIECNPRLNVAQHLYVAPNGDYNGGDPQFYNLGQVFLGSQ
                     GLPNAAGDVGTIAELWVSYEIVFYKPKISPIEDTRLLDLSQYLCLNITDSQPMGSVKS
                     PLRAIQGSAVLTDQTIQLPPIVGSTWMITVFYGGEDFRANAPTSSPNYDFSLGCALGG
                     QQSFPDAGGTSFAWDDNVTALSVSGDATIDGMSFGINVYVTPTAIAIPPTITFSAMVL
                     PGTATEPTATVTILRLS*"
ORIGIN
        1 tactattacc caagaaaccc cctatcgcat agcgatagcg ccgattcaca aagttgaaat
       61 cggctggaat ttcgtgaaat ttcaaaaact ccgagatctc gtaactccga gttcgtataa
      121 gaactcggag taactcggag ttaagcattt ataatgtctt ctcgcgggcg aggaggtgct
      181 cctcgacaag agaaaaaagg tgtttcgata acttggactt tgaacaatta tacggacgct
      241 gaagtttccg atattaaggc caactggccg aagatggcaa aatatgtgtg ttttggcaag
      301 gaaataggcg gtgcggagct aacgccacat cttcaaggtt atatgagctt gaagaaacaa
      361 actgcttatt cgagcattca taaggatttt cctggaatga tccgagctca ttttttgaac
      421 gcgggcggag accacgttca aaatcgcaat tactgtggta agggcgcgat gtcaacgatc
      481 gaatacgatc gatatcgcaa agttgcggtt ccaaactcaa aacctaaggt ttttaaagag
      541 ggcgatcgat acggccagga cgtacgatcg catcctgaat atggaatcgg tgccgatttt
      601 tgggaaagcg gccctttatc cgaagcgata caaccgggga aatccaaaga tttacacgag
      661 ttcgtagaag ctctcggatc gaagtcgata acttcgattg ttcgctcatc tatggagaac
      721 aaaattgtgt tcctcaaaca caataaatat ttggaagcat tagaatccaa gatcgttcgc
      781 actcgtagtg cgaaagatgg ctttccggag acccactgga tttgcggaaa aacgggacag
      841 gcgaagacac gactcgctgg tgagttcgcc cgacgatata tgggcgccga tgatgatgcc
      901 gaagaccaag agctcttcgc gctcatccga cctggacgag ctggactcgc cccttggttc
      961 ggaaactggg agtatcaacc tgtcgccatc attgacgagg ttcgagctaa atcccacgac
     1021 ttcgtttgga tgctcggatt ctgcgatcga tacgatcaag tctgcgagcc caaaggatcg
     1081 cagaaacctt ggaacgcaga agtcgtcatt tatacttctc caagaagtat tgacgacacc
     1141 tatagaggac gaactgatga ggagctcgat cagctccacc gaagaattcg agagtcagga
     1201 ggaagagagt ggcgattccc cgacgatgct gcggagtggc tcgagcggct cggccgactc
     1261 cagccacgat ctgggaccgg gctcgccgat ccccgcatta gaccccaata cgcctctgcc
     1321 gtcgttgggc cgcttcgcag caatgaagtt cggttggcag agcccgccga tgaagaatca
     1381 cttcgatcgg gtaccgaaat gatggatgaa attgatgcgg agatacagcg acttgttgat
     1441 gaacacttcg ctgattaata aaaaattcgt attacgaagt ttgtgttata tttaagtatg
     1501 gatgtcgtac gttcctgatt ttaccgaaag ttggttgcat gaccctgcga ccaatcttga
     1561 agcgttaaaa atgtctgaag agtatattcc tcagacattg ttaccttttc ctccctccgc
     1621 gaggcaggat tatcctcgtg ttatacgtga tcgacctagt gccagaggtc agtcggcgcg
     1681 taaaaaggca cgagctgatg ttcaacaaat tgctgctcaa atgtttgttg aagaattaaa
     1741 atctgagggt agatctattc ctcaggctgt cagtgatcaa gctagaggaa ttacatcgaa
     1801 gtctacgaaa atgtcgttac caccgaaaga ttatgcggct tcgctttctc gtgatgatct
     1861 tgagaagaag tatgctgctt cgattgaacg ttctaaaggt aaacgtaaaa ccactggtaa
     1921 aggacgagct ggcgcgtcta aaaaacgacg atattcgtcg tctcgatctt cgggacgcta
     1981 tggtggcacg aatatcgtat atcccaacgt tgtcggacgt ggagcgtacg gcattaaggg
     2041 aggccttaac ttcggacacg aagacgattg gtttagagga ggtgttggag gttactacaa
     2101 ctccgaccga gccgcgggag gaacagtttc cggacgaggc gcttataaca ttaagcgaaa
     2161 ttcgttggtt gttgacgaag gacaaggtcc tcctctaatc gaaaacttga ccaacgggca
     2221 agttgtaatt agacaccgtg agtatctcaa ggatatgact tgtccaactg gcgctccttc
     2281 gccttacaat atcaattttc aagagaaaat taatccagga aaccctgcga tgttcccttg
     2341 gcttagccaa attgctccta attttcagga gtgggaagca ttaggaatga tctttgaggt
     2401 caagacgatg agttctgatt acacgcagaa cgtcagtatg ggaaccgtat ttgcggcgac
     2461 cgattacaat actcaagctc ctgcgccgat taacaaacaa caactcgaaa atatggagtt
     2521 ttcgcagtcg tgtaaaccaa gttgtagcat tatgatgcct attgagtgca atcctagatt
     2581 gaatgtcgct caacatctct acgttgcgcc aaacggcgat tacaacggag gtgatccaca
     2641 attttacaat ttgggtcaag ttttccttgg ttctcaagga ttacccaacg cagcgggcga
     2701 tgttggtacg atagctgaac tttgggtcag ttatgagatc gttttttata aaccgaagat
     2761 ctctcctatc gaagacacta gattgctcga tttatcgcag tatttgtgtc tgaatattac
     2821 tgacagtcaa ccaatgggat cggttaagtc acctttacga gccattcaag gcagtgccgt
     2881 tttgactgat caaactattc aacttccacc aattgtcggt tcgacgtgga tgattaccgt
     2941 tttttatggc ggtgaagatt ttagagctaa tgctcctacg tcatctccaa attacgattt
     3001 ttcgcttggt tgtgcgcttg gtggacaaca aagttttcct gatgctggag gaacatcatt
     3061 tgcatgggac gacaacgtta ctgcgttatc tgtttcaggt gatgctacaa ttgacggtat
     3121 gtcttttgga attaacgttt acgttactcc aaccgcaatt gcaattccac ctacaatcac
     3181 gttttcggcg atggttcttc ctgggacagc gactgaacca acggcaaccg tgactatttt
     3241 acgtctttcg taaataaaaa tactttactt cactcgtgct taaagttttt aatttatagg
     3301 tggggcggtt cggcgcccgc agggcggcga agcgtccaca ccgacgcgac cgaagggcgc
     3361 gagttcggcg cctccgggcg ccgcatatac gtaaatacgg gtcccgaccg ttacgtataa
     3421 aataaaaacg tgcttactat ttttttgtct tctggcgtta gtcgaaccga cgaacgaccg
     3481 acgaccaacg acgaagtcgg tggttcgatt cccgctgtag gcgttttttt tcttaaaaaa
     3541 aaagtcgaaa atctgctaag acttttcgac ttagcgaaaa agggggtttc ttgc
//
LOCUS       CruV-314                3597 bp    DNA     circular     24-FEB-2020
DEFINITION  Crucivirus-314 isolate 120_795_SvL1_3295.
ACCESSION   CruV-314
KEYWORDS    .
SOURCE      Crucivirus-314
  ORGANISM  Crucivirus-314
            Unclassified.
REFERENCE   1  (bases 1 to 3597)
  AUTHORS   de la Higuera,I., Kasun,G.W., Torrance,E.L., Pratt,A.A.,
            Maluenda,A., Colombet,J., Bisseux,M., Ravet,V., Dayaram,A.,
            Stainton,D., Kraberger,S., Zawar-Reza,P., Goldstien,S., Briskie,J.,
            White,R., Taylor,H., Gomez,C., Ainley,D.G., Harding,J.S.,
            Fontenele,R.S., Schreck,J., Ribeiro,S.G., Oswald,S.A., Arnold,J.M.,
            Varsani,A., Enault,F. and Stedman,K.M.
  TITLE     Direct Submission
  JOURNAL   Submitted (24-FEB-2020) Biology, Portland State University, 1719 SW
            10th Ave, Portland, Oregon 97201, USA
COMMENT     Bankit Comment: ALT EMAIL:ide@pdx.edu
            Bankit Comment: TOTAL # OF SEQS:451
            Bankit Comment: GAP: est. length - alignment

            ##Assembly-Data-START##
            Assembly Method       :: MetaSPAdes v. 3.12; IDBA-UD v. 1.1.1;
                                     MEGAHIT v. 1.0.3
            Sequencing Technology :: 454; Illumina
            ##Assembly-Data-END##
FEATURES             Location/Qualifiers
     source          1..3597
                     /organism="Crucivirus-314"
                     /mol_type="genomic DNA"
                     /isolate="120_795_SvL1_3295"
                     /isolation_source="Water"
                     /country="Norway"
                     /collection_date=2012
     CDS             1..1656
                     /codon_start=1
                     /product="Putative Replication Associated Protein"
                     /translation="MIIFSFRVFCGYAYLRVYTMDCKTKPLSRAQREARDEAIATTDA
                     QPMEASVRKKKPSKETPEAAAPVDSDVSPYKSGLRSRGWVFTWNNPVLPAGYANFVDF
                     YRAWEPQVDYLVVGNEHAPTTGTLHHQGAVWFKNQRTWPACRSSIKCWWKPAKGSGEQ
                     NFNYSNKEAILIEFGIRKQQGNRADLEDVRDLIREGASMREVTERASNLQAIKVGQLF
                     LQHHPELQQRRNWNMQIFWIKGPAGIGKTRWCAEQAPNAHFSSRNLQWWDGYADQEDV
                     IVDDFRGDFCTYHELLRIQDRYPYRVQIKGSSCELLAKRIFFTSCLLPTECYATREDI
                     WQLLRRITAVYEFNEDGSANIWRYVNKVDTTKVEQLGKLPPRTVELSAREEIILPGLG
                     TALPPRDFELIIKNPADSTQKSGGNKCPLRNNLIIPPRAAPDNLVCACTNCGSTELTH
                     RCCNYCKMTDPPMDHHMSCNEETKYAAETRECQHVDTCDDGAGNELCLTCGEYVEDCD
                     ADNEEEQEMCPVCATPIIAYIDGVLACKCHIAYPDGYDYPPDG*"
     CDS             1729..3204
                     /codon_start=1
                     /product="Putative CruV Capsid Protein"
                     /translation="MPRVAAKKKVVRKRVAATPRYIPRVSGRGAYSYEKPGPWGRYGR
                     TAGGIAGDVLGRTFLGPAGGVVGKVLGNKVGSYAHYIGRIFGSGDYVTSKGVRMNNIL
                     SPQTPAFEDPKGTVRVRHREYLGDVISSGTAAAFKIDSYALNPGIADSFPWLSNVCGA
                     TFQQYRINGMVFEFRSMSSDALNSTNTALGSVVMCTDYDSADTAFTSKQQMENSEWGV
                     SCKPSCNMIHGIECAPRMTTATELYVRAFDNPSGTDIRLYDWGKTYVATVGCQGTSVN
                     LGELWVSYDVTFFKTIEQVPGYLIPFSLYALTSASATAPLGTSRAQWSTTTGGNNNLD
                     LTLTENKVSFPLTIPVNSIWHVTLAYESSEGKAITVPACTTGNGMYMDAAKAWKTPNT
                     GSEIQYSFAWNIDVQYQGGGTPAALPYVNFADFTWTGAVITQADLYVYQLPGLYQGTY
                     TDQSEVPRVVKEEEEEVDTAPSYISVHQNEEKKPQVRRPVR*"
ORIGIN
        1 atgattattt ttagttttag agttttttgc ggttatgcat acctgcgtgt ttacactatg
       61 gattgcaaaa caaagcctct ttcgcgtgcc caaagagagg ccagagatga agctatcgcc
      121 acgacagacg cacaaccgat ggaagcgtct gttcgaaaaa agaaaccctc taaggagaca
      181 ccagaagctg ccgctcccgt cgattctgac gtcagcccat ataaatctgg actgcgctcc
      241 cgaggatggg tatttacttg gaataaccca gtactgcctg ccggctatgc caacttcgtt
      301 gacttctaca gggcttggga gcctcaagta gactacttag tcgtaggaaa tgaacacgcg
      361 cccactactg ggacgctaca tcaccaagga gctgtttggt tcaaaaatca gcggacttgg
      421 cccgcttgta gatcttcgat caaatgctgg tggaaaccag ctaaaggttc tggcgagcaa
      481 aactttaact actcaaacaa agaggccatc ctcattgaat ttggtatacg gaaacaacaa
      541 ggtaatcgag ctgaccttga ggatgtccgt gacttgattc gcgagggcgc atcaatgcgt
      601 gaagtgactg aacgagctag taaccttcaa gctatcaaag ttggtcaact tttcttacag
      661 catcatcccg agttgcagca acgccgaaac tggaatatgc agatcttttg gatcaaaggg
      721 cctgcaggaa tcggtaaaac acgatggtgt gcggagcaag ctcctaatgc gcacttctcg
      781 tcgcgcaacc tgcagtggtg ggacggttat gcagaccagg aagacgtcat tgttgacgat
      841 ttccgtggtg atttctgcac ctatcatgaa ctgcttcgca tccaagaccg atatccctat
      901 cgggttcaga tcaaaggcag ttcttgcgaa ctcctagcca aacgtatctt ctttacgagc
      961 tgtttgcttc ctaccgagtg ctacgctaca cgggaggata tttggcaact ccttagacgc
     1021 attactgccg tctatgagtt taacgaggat ggaagtgcaa atatctggcg atatgtgaac
     1081 aaagtggaca cgactaaagt cgagcaactt ggcaagctgc cacctcgaac tgttgagctg
     1141 agtgcaagag aagagattat cttgcccggc cttggtacgg cgctcccgcc gagagatttt
     1201 gaattaatta ttaaaaatcc ggccgactct actcaaaagt cggggggtaa taaatgcccc
     1261 ctccggaata atttaattat ccctccgcgg gcggcccccg acaatttggt ttgtgcatgc
     1321 acaaactgtg gtagcactga attgacacat aggtgttgca attattgcaa aatgactgat
     1381 cctccgatgg atcatcatat gtcatgcaac gaagaaacaa agtacgctgc agagacaagg
     1441 gagtgtcaac atgttgacac gtgtgacgac ggggcaggca atgaattgtg cctcacctgt
     1501 ggtgaatacg tcgaggactg cgatgcagat aatgaagaag agcaagaaat gtgccctgtc
     1561 tgcgcaacgc ccattattgc ttatatagat ggcgtacttg catgcaagtg ccatattgca
     1621 taccctgacg gatatgacta tcctccggat ggttaaagat gctgatatgc atatttattt
     1681 ttgtgttaga ctttttttag cgcataatat gttcaagctt tcacaagtat gcctcgcgtt
     1741 gctgcaaaaa agaaggtggt gcggaaacgt gtcgctgcta ctccacgata cattccacgt
     1801 gtaagtggcc gcggggccta cagttacgaa aaaccaggac catggggccg ctacggtcgt
     1861 accgcgggcg gtattgccgg tgatgtgctg gggcgcacct tccttggtcc ggctggtggc
     1921 gttgttggta aggttcttgg caacaaggtc ggctcctacg cccattacat tggaagaatc
     1981 tttgggtctg gcgactacgt tacctcaaaa ggtgtaagaa tgaacaacat tttgtctcct
     2041 caaactcccg cgttcgaaga ccctaaggga accgttaggg ttcgacaccg tgagtatctc
     2101 ggagatgtga tttcgtctgg aacagctgcc gctttcaaga tcgattcata cgcactaaac
     2161 ccgggaatcg ctgattcatt tccgtggttg tcgaacgtat gcggtgctac cttccagcag
     2221 taccgcatta atggtatggt gttcgaattt cgatcaatgt cttccgacgc tctcaactca
     2281 acaaacacag ctctgggatc tgtggtcatg tgtactgatt acgattcagc agatactgct
     2341 ttcacatcga aacaacaaat ggagaacagt gaatgggggg tttcttgcaa accctcatgc
     2401 aacatgattc atggtattga atgtgcgcct cgcatgacta cggctacaga attgtacgtc
     2461 agggcttttg ataacccttc cggaacagac attcgtctgt atgattgggg gaagacttac
     2521 gtcgcaaccg ttggatgcca aggcacctca gttaacctcg gtgaactgtg ggtgtcttac
     2581 gacgttacgt tcttcaaaac tattgaacag gtacctgggt accttattcc gttctcgttg
     2641 tacgcgctaa cgagtgcctc cgctacggca cccctgggta catcgcgtgc acagtggtcc
     2701 acgaccactg gtggcaacaa caacctcgac ctgactttaa cagagaataa agtcagcttt
     2761 ccgctgacaa ttcctgtaaa ttccatctgg catgtgacgc tcgcatacga gtcgtctgag
     2821 ggcaaagcta taactgtgcc cgcatgcacg actggaaacg gaatgtatat ggatgctgct
     2881 aaagcatgga agacaccaaa cactggatct gagattcaat actccttcgc atggaatatc
     2941 gacgtccagt atcaaggtgg tggtacgcct gctgcgctgc cgtatgtaaa ctttgctgac
     3001 ttcacctgga ctggcgccgt gatcacacag gcagacctgt acgtgtatca attgcccggg
     3061 ttgtaccaag gtacatacac cgatcaatct gaagttccaa gagttgtaaa agaagaagaa
     3121 gaagaagtgg ataccgcacc gtcttacatt tccgttcatc aaaatgagga aaaaaagcct
     3181 caggtccgaa gacctgttcg gtaacgtgat atgctcgcac caagccggca tcgcgggact
     3241 tacagccccg cagttctgct gccgtttgtc gctcgcgcct acggcaaaat attttgtgat
     3301 atttattaaa taattataca tatgttagtc gctcgctctc cagtgaggtc tgctctctat
     3361 gtacatcgtg cgtatcctcc gtgacgctaa tttgcgtcca acccctatgg cctaaaggcc
     3421 accacgggaa gtatctgatc aaggcaaacc cgcttcgcgg gttggcgtga aaagccgata
     3481 cctcacttgg tctcgtcaga ctagcagttc gtagctcatt acggtcgcgg accgtattca
     3541 ctggatgcac gaagtgcata ggtcggacca cgttgttcgc gctcccggga ataacat
//
LOCUS       CruV-315                3600 bp    DNA     circular     24-FEB-2020
DEFINITION  Crucivirus-315 isolate 120_1237_IR2_3297.
ACCESSION   CruV-315
KEYWORDS    .
SOURCE      Crucivirus-315
  ORGANISM  Crucivirus-315
            Unclassified.
REFERENCE   1  (bases 1 to 3600)
  AUTHORS   de la Higuera,I., Kasun,G.W., Torrance,E.L., Pratt,A.A.,
            Maluenda,A., Colombet,J., Bisseux,M., Ravet,V., Dayaram,A.,
            Stainton,D., Kraberger,S., Zawar-Reza,P., Goldstien,S., Briskie,J.,
            White,R., Taylor,H., Gomez,C., Ainley,D.G., Harding,J.S.,
            Fontenele,R.S., Schreck,J., Ribeiro,S.G., Oswald,S.A., Arnold,J.M.,
            Varsani,A., Enault,F. and Stedman,K.M.
  TITLE     Direct Submission
  JOURNAL   Submitted (24-FEB-2020) Biology, Portland State University, 1719 SW
            10th Ave, Portland, Oregon 97201, USA
COMMENT     Bankit Comment: ALT EMAIL:ide@pdx.edu
            Bankit Comment: TOTAL # OF SEQS:451
            Bankit Comment: GAP: est. length - alignment

            ##Assembly-Data-START##
            Assembly Method       :: MetaSPAdes v. 3.12; IDBA-UD v. 1.1.1;
                                     MEGAHIT v. 1.0.3
            Sequencing Technology :: 454; Illumina
            ##Assembly-Data-END##
FEATURES             Location/Qualifiers
     source          1..3600
                     /organism="Crucivirus-315"
                     /mol_type="genomic DNA"
                     /isolate="120_1237_IR2_3297"
                     /isolation_source="Water"
                     /country="Norway"
                     /collection_date=2011
     CDS             1..1479
                     /codon_start=1
                     /product="Putative Replication Associated Protein"
                     /translation="MDCKTKPLSRAERQAADEARQALSAPAAASAALPSEASVRKKKP
                     SKEIPEATAPEESDVSPYKAGLRSRGWIFTWNNPVLPTGFANFVDFYKAWEPQVDYLV
                     VGNEHAPTTGTPHHQGAVWFKNQRTWPACRSSIKCWWKPAKGSGEQNFNYSNKEAILI
                     EFGVRKQQGNRSDLEDVRDLIREGASMREVTERASNLQAIKVGQLFLQHHPELQQRRN
                     WNMQIFWIKGPAGIGKTRWCAEQAPNAHFSSRNLQWWDGYADQEDVIVDDFRGDFCTY
                     HELLRIQDRYPYRVQIKGSSCELLAKRIFFTSCLLPTECYATREDIWQLLRRITAVYE
                     YNEDGSANIWRYVDKVDTTKVEQLGKLPPRTVELSAREEIILPGLGTALPPRDFELII
                     KNPAASTQKSGGNKCQVENNFIIPSLASPDIGQLRCTLCNVYDSLCECEGEQKCSTPH
                     APTEICVECCTHADSISDGCNEGYEMCLDCGAFCEVQYEVDG*"
     CDS             complement(1524..3023)
                     /codon_start=1
                     /product="Putative CruV Capsid Protein"
                     /translation="MPAPRKVVTVRVPAKKKMVVVTKAKAVRKPRAVGTSAYKAKGGS
                     KSGFFGTMGGTAGGMLGTALGGPAGGMIGSTLGKYAGAALSSIFGHGDYEASNSNQIK
                     NNNLVLSNSAQVPQFGTGKVACKFVHREFLGDVFSSATANTFKIDSYPINPGQSKTFP
                     WLSGVVGAKFQQYRINGMVFEFRSMSSDALNSTNTALGSVVMSTDYDSADATFRSKQE
                     MENTEYGVSCKPSSNMMHGIECARFQTPVSELFIRAFAVPEDKDIRLYDLGRFSIASS
                     GCQGTDVNLGELWVAYDIDCFKAIEQVPAYLTPYSQYALTGQSATAPLGTAQPTLLTN
                     VDSPDFNSQQQIGLTFTENKISWPIDTSVGSVYMVDLAVLGAAAAALTPPVVTTGNGM
                     FINVNGFWKSYTAGTTDVMHLWYTIKYEGGATPGALPYLNVADYTWAPAGGILQADLV
                     VSQISGQFGGVFSSQSEITHDDDDTAAPGYISVREDEKKTVTVHRRAVR*"
ORIGIN
        1 atggattgca aaactaagcc actttcgcgt gccgaaagac aagccgcaga tgaagcacgt
       61 caagcgttga gtgcacccgc cgccgcgagc gccgcgctac cttcggaagc gtctgtacgg
      121 aaaaagaaac cctctaaaga gatacctgaa gcaaccgctc cagaggaatc tgacgtcagt
      181 ccgtacaaag ccggcctacg ctcacgaggg tggatattca cttggaataa ccctgtactg
      241 cctaccggct ttgccaactt cgttgacttc tacaaggctt gggagcctca agtagactac
      301 ttggtcgtag ggaatgaaca cgcgcccact actgggacgc cacatcacca aggagctgtt
      361 tggttcaaaa atcagcggac ttggcccgct tgtagatctt cgatcaaatg ctggtggaaa
      421 ccagctaaag gttctggcga gcagaacttt aattactcca acaaagaggc tatccttatt
      481 gaatttggag tgcggaaaca acaaggtaac cgcagtgatc ttgaagatgt ccgtgatttg
      541 atccgcgagg gcgcatcaat gcgtgaagtt actgaacgag ccagtaacct tcaagctata
      601 aaagttggtc aacttttctt acagcatcat cctgagttgc agcaacgccg aaactggaat
      661 atgcaaatct tttggattaa gggtcctgca ggcatcggta aaacgcgctg gtgtgcggag
      721 caagctccta atgcgcactt ctcgtcgcgc aatctgcagt ggtgggacgg ttatgcagac
      781 caggaagacg tcattgttga cgatttccgt ggtgatttct gcacctatca tgaactgctt
      841 cgcatccaag accgatatcc ctatcgggtt cagattaagg gctcatcttg tgagcttttg
      901 gccaaacgta tcttctttac gagctgtttg cttcctaccg agtgctatgc tacccgggag
      961 gatatttggc aactccttcg acgcattact gccgtctatg agtataacga ggatggaagt
     1021 gcaaatatct ggcgatatgt ggacaaagtg gacacgacta aagtcgagca acttggcaag
     1081 ctgccacctc gaactgttga gttgagtgca agagaagaga ttatcttgcc cggcctgggt
     1141 acggcgctac cgccgagaga ttttgaatta attattaaaa atccggccgc ctctactcaa
     1201 aagtcggggg gtaataaatg ccaggtcgaa aataatttca ttattccctc gctggcgtcc
     1261 cccgacattg gtcaacttcg ctgtacatta tgtaatgtat atgattcgct atgcgaatgt
     1321 gaaggtgaac aaaaatgctc tacgccgcac gcgcctactg agatttgtgt tgaatgttgc
     1381 acacatgctg actccatcag cgacggttgc aacgagggtt acgagatgtg tctcgattgt
     1441 ggtgcattct gcgaggtgca gtatgaagtt gatggataaa caccaacaac caacacaaaa
     1501 gttttttcgc taatgttatt agcttatcgg acagctcgtc ggtgaacggt cactgtcttt
     1561 ttttcgtctt cacgcactga aatgtagccc ggggctgccg tgtcatcatc gtcatgagtg
     1621 atctcagatt gagatgagaa gactcctccg aactggccag atatctgcga tactaccaga
     1681 tcggcctgaa gtatgcctcc cgcgggtgcc caagtgtagt cagccacgtt gagatacgga
     1741 agtgcgcctg gcgttgcacc tccttcatac ttaatagtat accacagatg catcacatct
     1801 gttgttcctg ccgtgtatga cttccagaag ccatttacgt taataaacat tccgttccct
     1861 gtggtcacta caggtggtgt aagagcggct gccgcggctc ctaacactgc caaatcgacc
     1921 atgtaaacac ttcctacgga cgtgtcgatt ggccaactga tcttgttttc tgtgaatgtg
     1981 agcccgattt gctgctgaga gttgaaatca ggcgaatcta cattcgttag caaggttggt
     2041 tgagcagtcc ctaaaggggc tgtcgctgac tgcccagtca gcgcgtattg cgaatacgga
     2101 gtcagatagg caggcacctg ctcaattgct ttgaaacagt cgatatcata tgctacccaa
     2161 agctcaccaa ggttcacatc ggtcccttga catcctgagg acgcaatgga gaatcgaccc
     2221 aaatcataaa gccgaatatc cttgtcttct ggcactgcaa aagctcggat gaacaattcg
     2281 gacacgggag tctgaaagcg cgcgcattca atcccgtgca tcatatttga tgatggttta
     2341 caactaacac cgtactcggt attttccatc tcttgtttgg acctaaaggt cgcatctgct
     2401 gaatcatagt ctgtcgacat gaccacagac ccaagagctg tgttagtgga attcagcgca
     2461 tcagatgaca tgcttcgaaa ctcgaagacc atcccattaa tcctgtactg ctgaaattta
     2521 gcaccaacta caccggaaag ccacggaaag gtcttcgact gtcctggatt gatcgggtag
     2581 ctatcaattt tgaacgtgtt cgctgtggcc gagctaaaca catctccgag gaattctcga
     2641 tgaacaaatt tgcacgcaac tttgcccgtt ccaaactgtg gtacttgagc cgaattcgac
     2701 aatacaagat tattattctt gatctggttt gaattcgacg cttcataatc tccgtgcccg
     2761 aagattgatg aaagggcggc tccggcatat ttgcccaatg ttgagccaat catgccccct
     2821 gcgggtccac ccaatgctgt acccagcata ccgccagcgg ttccacccat agtaccaaag
     2881 aatccacttt tggatccacc tttggcctta tatgcggaag tacctacagc acgtggtttt
     2941 ctcacggctt tggcttttgt gacgacgacc atcttcttct ttgcgggcac acgtaccgtg
     3001 acaactttcc gaggggcggg catgttgtag aagattgtcg gactacgacg tcagggtatg
     3061 ataccgaatt atattccttt aatttgcaat ttaattgcac atacattgca gcatgacgcg
     3121 cccatgccgc tgatgccgga tcatatcgct ctagctcttt tgcaagtgct agctgctcga
     3181 gtaagacttt atcatactca tgccaccaat cgagttcctc tactgtcggc atcctcgtat
     3241 gattaatatt tataatttct taaaaatata aatcccctgt tatgacttcg ggattcatga
     3301 acaggtctac tcgctataca ccgaatgctg gggcattagg gattatacat ccaagtcacc
     3361 cacgggtggg caccacgtgg agcgctcagg gttgagcttg tccccttcgg ggacagtgca
     3421 acctgtgagt ttgcacttgg tccattcgga cactcggatc tttagtataa ccctaccgtt
     3481 ccactatgcg ctattcagtg cataggtcgg ggcatgtatt cacccttgtc cctcgaacga
     3541 ggtgattagt ttttggttta gacttttttc cgcttggggt atacctgagt gttacgcacc
//
LOCUS       CruV-316                3603 bp    DNA     circular     24-FEB-2020
DEFINITION  Crucivirus-316 isolate 001391_virusbaton.
ACCESSION   CruV-316
KEYWORDS    .
SOURCE      Crucivirus-316
  ORGANISM  Crucivirus-316
            Unclassified.
REFERENCE   1  (bases 1 to 3603)
  AUTHORS   de la Higuera,I., Kasun,G.W., Torrance,E.L., Pratt,A.A.,
            Maluenda,A., Colombet,J., Bisseux,M., Ravet,V., Dayaram,A.,
            Stainton,D., Kraberger,S., Zawar-Reza,P., Goldstien,S., Briskie,J.,
            White,R., Taylor,H., Gomez,C., Ainley,D.G., Harding,J.S.,
            Fontenele,R.S., Schreck,J., Ribeiro,S.G., Oswald,S.A., Arnold,J.M.,
            Varsani,A., Enault,F. and Stedman,K.M.
  TITLE     Direct Submission
  JOURNAL   Submitted (24-FEB-2020) Biology, Portland State University, 1719 SW
            10th Ave, Portland, Oregon 97201, USA
COMMENT     Bankit Comment: ALT EMAIL:ide@pdx.edu
            Bankit Comment: TOTAL # OF SEQS:451
            Bankit Comment: GAP: est. length - alignment

            ##Assembly-Data-START##
            Assembly Method       :: MetaSPAdes v. 3.12; IDBA-UD v. 1.1.1;
                                     MEGAHIT v. 1.0.3
            Sequencing Technology :: 454; Illumina
            ##Assembly-Data-END##
FEATURES             Location/Qualifiers
     source          1..3603
                     /organism="Crucivirus-316"
                     /mol_type="genomic DNA"
                     /isolate="001391_virusbaton"
                     /isolation_source="Water"
                     /country="France"
                     /collection_date=2013
     misc_feature    join(3594..3603,1..18)
                     /note="stem-loop"
     misc_feature    1..9
                     /note="nonanucleotide"
     CDS             265..1752
                     /codon_start=1
                     /product="Putative Replication Associated Protein"
                     /translation="MSEIAQKPSKSINELVKDVVVVKTKAKAAKPPSEPKAPKEPKVP
                     KAPKEPRPPKVPKAPKEPRASKAPSEEEDQGPPTKARGYCFTINNYSPEEVESFRAYA
                     VKIDYAIFGYEVAPTTGTPHMQCYMNHNNTIRFQTLRKLFPRAANIVQAKGTAKQNQI
                     YCKKGVQSKEEYTEHGAEGPNYGKDAVWEEFGECPETVGSGHRTDLDIIGQQLREGKT
                     TVEKLATELPTDTFHRYLRTFSYYEHHYLRNKRRTGPPRCFFLYGTTGSAKTREFTNG
                     IPEEEIYTQPKDSLKFSGYAGQRAFIMNEVRHTVPFEYLLDIADPTVALTVEVKNERK
                     HIQMTAEIIVITSCSSPEELYPEESRERMLQFYRRYICVEMNGNDRNVIVDEEVYKRP
                     FVDHPNFISEVVTWEGTKQECEQRLQDAFASRRKDKALWAERMAETKAQYDERRRVER
                     EAEEAAERENNALIRANPLSDQELLDMFNDSPREYEEYMAIISSK*"
     CDS             1838..3241
                     /codon_start=1
                     /product="Putative CruV Capsid Protein"
                     /translation="MPPKYNAVKAFQGVNNQRRLKSAAKAYAGKNAARKRQNTVGAVR
                     RAVNAYGPMISKALSGMGDYAIRDPSSSLGHSYRTGRGIEQGHMGTKHPGAHHGHHPH
                     SSLEKGMMSICHSEYIGDLISSGTTSTTNFVSQSYALNPGNSGTFPWLSSTAINFQHY
                     RFKKLVFEYRPLVSESTASTTATLLSMGSVIMATQYNSVVGPYTTKLGMTESDFAVTT
                     KPSEHSLHAVECDPKFNPLGELFVSAQTSLTVGANSSDIRMQNLGIFEIASSNIPIAG
                     NAAIDLGEIWVHYEVELYKPQINAYLSGLLSSHYYQNTTVGNATASNLFGPNVLSTAQ
                     PTAATNNLLGLTFTTNSFSFPLQVTSGSYLCIYKVYASTSQAITIATPTVATNTGTLL
                     TCWSSSAAADINSVANAPETGDSAKNFHLSFIVQVNAPGATLCTVTMGATTPPLVNGG
                     TSFWELFVTPYNSAMLV*"
ORIGIN
        1 tattattacc cttttagagt acctatgtgg caaaatattg gtaaatattg gctcatgtac
       61 gggaggaagt ctggctacat atgcggtagc attagcggat cttgagaata caagacagat
      121 gacgactaac caatattgtt cgttttatat tttgccacag tagtaatatt ttgcttttgg
      181 gttaaaaact ccttttttta ttttctgcca caaaggttta atcaaacctc aaaccaaaaa
      241 agtaacacat tcattttttt gtcaatgtca gaaattgccc aaaagccatc caaatctatc
      301 aacgaattgg taaaagatgt tgtcgtagtg aaaacgaagg cgaaggcggc gaagccgccg
      361 agtgaaccga aggctccgaa ggagccgaag gttccgaagg ctccgaagga gccgagacct
      421 ccgaaggtgc cgaaggcacc gaaggagccg agggcgtcga aggcgccctc ggaggaggag
      481 gaccagggtc ctccgactaa ggcaaggggg tattgtttta ctataaacaa ttactccccc
      541 gaagaagttg aaagcttcag agcatatgct gtgaaaatag attatgctat ttttgggtat
      601 gaggttgctc ctacaaccgg tactccacat atgcagtgtt atatgaatca taataacact
      661 atacgctttc agactttacg taaattgttt cctcgtgctg ctaacatagt gcaggcaaag
      721 gggactgcaa agcagaatca aatttattgc aagaaaggtg tgcagagtaa ggaggagtat
      781 accgaacatg gtgcagaagg accaaattac ggcaaagatg ccgtttggga agaatttggc
      841 gaatgtcctg agaccgtagg ttccggtcac cgtacagatt tggatattat cggccaacag
      901 ctgcgggaag gtaagacaac tgtggaaaag ttggcgacgg aattgcctac agatacattt
      961 catcgttatc tgcgcacatt ctcgtattat gagcatcatt atctccggaa taaacgtagg
     1021 actggaccac cgcgttgttt ttttttgtac gggacgactg gctccgcaaa aacccgggaa
     1081 tttactaatg gaatcccgga ggaagagatt tatactcagc ctaaggactc tctcaagttt
     1141 tccggctatg ctggacagcg tgcctttatt atgaatgaag tacggcatac cgtacctttt
     1201 gaatatctgc ttgatattgc agatcctact gtggcactaa ccgttgaggt taaaaatgag
     1261 aggaaacata ttcagatgac agctgaaatt attgtgatta catcttgttc tagtcctgaa
     1321 gaactgtatc ctgaagaatc acgggagcgt atgttacagt tctatcgccg atatatttgc
     1381 gtcgagatga atggaaatga tcgaaatgtc atcgttgatg aggaggtgta taaacgacct
     1441 tttgtagatc atcctaattt tatcagcgag gtggttacct gggaaggaac caagcaggaa
     1501 tgtgagcaac gattacaaga tgcgtttgcg tcacgccgta aggataaagc attatgggct
     1561 gagcgtatgg ctgagaccaa ggctcagtac gacgaacgtc gtcgagttga acgagaagca
     1621 gaggaagctg ctgaacgtga aaataatgca cttatacgag caaatccgtt gagtgatcag
     1681 gaattgcttg atatgtttaa tgattctcct cgtgaatacg aagaatatat ggctattatt
     1741 tcatctaaat aatgccacta aggtatgaac ctatttttag ttcttttgta agtgtaattc
     1801 aaactttaat tacaccttca aatattcaac tttttaaatg cctccaaaat ataacgccgt
     1861 taaagccttt caaggcgtta acaatcaacg acgtctcaaa tccgcagcta aagcttatgc
     1921 tggaaagaat gcggcacgaa agcgacaaaa tactgttggt gctgttcgta gagcagttaa
     1981 tgcgtatggt ccaatgattt caaaagcatt gtccggtatg ggtgactacg ccatccggga
     2041 tccttcatcg tcgttaggac attcgtatag aactggccgt ggtatagagc agggacatat
     2101 gggaactaag catcccggtg cccatcatgg ccatcatcct cattcttctt tggagaaagg
     2161 aatgatgtct atttgtcatt cagagtatat tggggatttg attagttcgg gaacgacttc
     2221 tactacaaat tttgtttcac aaagctatgc cctcaacccg ggaaattcgg gaacttttcc
     2281 atggttgtct tctactgcta ttaattttca acattatcgc tttaagaagc tggtgtttga
     2341 atatcgtccg ttggtgtcgg aatcaaccgc atcaactacg gcaactcttt tgagtatggg
     2401 gtctgttata atggcaacac agtataatag tgtggtggga ccttacacca ctaagttggg
     2461 aatgacagag tctgattttg cagttactac gaagccgtca gaacattcgc tccatgcagt
     2521 agaatgtgat cccaaattca atcctttggg tgaattgttt gtatcagcac agactagttt
     2581 gactgttggc gcgaattcat ctgatattcg catgcagaat ttaggcatat ttgagattgc
     2641 cagctcaaac attcctattg cagggaatgc tgctattgat cttggtgaga tctgggtaca
     2701 ttatgaggtt gaattataca agccgcagat taatgcgtat ctgtcaggat tgttgtcatc
     2761 tcattattat cagaacacta cagttggtaa tgctaccgca tcgaatttat tcggcccaaa
     2821 tgtattgtcg actgcacagc ctactgcggc tactaacaat ttgttggggt taacatttac
     2881 aacaaattcg ttttcgtttc ctctacaagt tacatctgga agttatcttt gtatttataa
     2941 agtgtatgca tcgacgtccc aggcgatcac tatagctact ccaactgtag caacgaatac
     3001 tggaacactt cttacgtgtt ggtcttcgtc cgctgctgcc gatattaact cggtggctaa
     3061 tgctcctgaa actggagata gcgctaagaa ttttcatctt tcttttattg tgcaagtaaa
     3121 tgcaccaggc gctacgttat gcactgtaac gatgggggct actactccac ctcttgtaaa
     3181 tggtggtact tccttttggg agttgtttgt aactccgtac aacagtgcta tgttagttta
     3241 attgttcttt tgtgtataaa aaaatagcag gtgttttgaa tttttgtaag tataaaaatc
     3301 aaaaaccgaa tgtaacgatt tacctccagc aagcataatg gaatctattt tgtttataga
     3361 ttatgtgcgc agttccaagc gcataactgc tgcaaaagac acatattggt gggccgaagg
     3421 catacgcgcc gcaggcccca cgttgaatag ccccgcaggg agccccgcag ggcataacac
     3481 attggtgtta atagcagcta tatccgcatt aaatgctgta tattgaaata aattaggtac
     3541 gagggcaact tgttgcccga atttgaaaga caaactttag tttgtcttga aaatctaaaa
     3601 ggt
//
LOCUS       CruV-317                3611 bp    DNA     circular     24-FEB-2020
DEFINITION  Crucivirus-317 isolate 000633_ADNa22.
ACCESSION   CruV-317
KEYWORDS    .
SOURCE      Crucivirus-317
  ORGANISM  Crucivirus-317
            Unclassified.
REFERENCE   1  (bases 1 to 3611)
  AUTHORS   de la Higuera,I., Kasun,G.W., Torrance,E.L., Pratt,A.A.,
            Maluenda,A., Colombet,J., Bisseux,M., Ravet,V., Dayaram,A.,
            Stainton,D., Kraberger,S., Zawar-Reza,P., Goldstien,S., Briskie,J.,
            White,R., Taylor,H., Gomez,C., Ainley,D.G., Harding,J.S.,
            Fontenele,R.S., Schreck,J., Ribeiro,S.G., Oswald,S.A., Arnold,J.M.,
            Varsani,A., Enault,F. and Stedman,K.M.
  TITLE     Direct Submission
  JOURNAL   Submitted (24-FEB-2020) Biology, Portland State University, 1719 SW
            10th Ave, Portland, Oregon 97201, USA
COMMENT     Bankit Comment: ALT EMAIL:ide@pdx.edu
            Bankit Comment: TOTAL # OF SEQS:451
            Bankit Comment: GAP: est. length - alignment

            ##Assembly-Data-START##
            Assembly Method       :: MetaSPAdes v. 3.12; IDBA-UD v. 1.1.1;
                                     MEGAHIT v. 1.0.3
            Sequencing Technology :: 454; Illumina
            ##Assembly-Data-END##
FEATURES             Location/Qualifiers
     source          1..3611
                     /organism="Crucivirus-317"
                     /mol_type="genomic DNA"
                     /isolate="000633_ADNa22"
                     /isolation_source="Water"
                     /country="France"
                     /collection_date=2013
     CDS             10..741
                     /codon_start=1
                     /product="Putative Replication Associated Protein"
                     /translation="MTKIKPIYNNSTTYKSNTHGDDLSDMGKNRSAIKQWFITYPKIL
                     SSMTREKFMDDLKNVLGQKIQYLRVVLEHHEDGGEHLHSILLMKGSGLSYSAINKFFK
                     DLYPNENKRIHFRPIRVLSYAIDYCDKEDIEDITYGELSIENGGVRQSDEVKLFLNEI
                     KRKDLFQVPQDILLKKNYQMEILNSCNQYLLFTRHFNNDERTILYDCRNHMSNKKELV
                     RFMLLFHRLNDFLITFQVEQKYIDI*"
     CDS             1755..3047
                     /codon_start=1
                     /product="Putative CruV Capsid Protein"
                     /translation="MPRVNAKSSRIPRKRIIKGKGAYINLKQPYKRYAKNALRAVSIG
                     AGAYLGRSSYGIMGKNAVFMDTVAKKTGDIIFGQGAYKTKLNRNSLIYASPVPAVHSV
                     GESIRIRHTECLYDISSSTTSFVQTRYAINPAVSATFPWLSTIASSFQEYRFEGLIFE
                     FRSSAGESINSASNNLGSIMMTTQYNPYDLPFINKNQLLNESGAVEAKPSQNLIHGVE
                     CSPQLNTLSTLYTRYGAAPAGSDIRLFDLGVTTVATYGSQAVNDIGSLYVSYDVVLIK
                     SKIVSALALTNETTHFSQTGTSATLTQPLISLLNRFDFIGITITGGNTIVLPVGSQGK
                     YFINLTWAGTTAQIAPLVGTTLTNATIVPSFTNNNYVQSPQNTVITASLSFQLVINII
                     DPSVLTTILILGVTGTFPTGTTGAGCDLYINQINNAVV*"
ORIGIN
        1 ataatcaata tgacaaaaat taaacccata tataataatt caaccactta taaaagtaat
       61 actcacggtg acgatttatc cgatatgggt aaaaatcgaa gtgctattaa acagtggttt
      121 ataacttatc ctaaaatttt atcgtcaatg acgagagaaa aatttatgga cgatttaaaa
      181 aatgtattag gtcaaaaaat tcaatatctt cgggtcgtat tagaacacca cgaagacgga
      241 ggcgaacatt tacacagtat attattaatg aagggttcag gtttatcata ttctgctata
      301 aataaatttt ttaaggattt atacccaaat gaaaataaac gaattcattt tagaccgata
      361 agagtgttaa gttatgctat tgactactgt gataaagaag acattgagga tataacatat
      421 ggagaacttt ctatagagaa tggaggagtt agacaatccg acgaagttaa actttttctt
      481 aatgaaataa aaagaaaaga tttatttcaa gttcctcagg atatactttt aaagaaaaat
      541 taccaaatgg agattttaaa tagttgtaat caatatttac tatttacgag acattttaat
      601 aacgatgaac gaactatttt atatgattgc cgtaatcata tgagtaataa aaaagaatta
      661 gttcgattta tgttattatt tcataggtta aatgattttt taataacttt tcaagtagaa
      721 caaaaatata tagacattta aatatataaa ttcattttta tatatttttt atcaataatt
      781 agaacaacta ttaaaagttg tactaattta aattataaat atttataggg ttgttatgcc
      841 gtttttttca atatttataa tttaaaaaga aaatcaaaaa gtgcgactta ccccattcaa
      901 aaattttaaa aaactttttc aactttttca acttttttaa aatttttcaa aaaaagaaaa
      961 acttttaaat attttatgaa aacaagacga cattttttaa aaaatgataa aaaaacaata
     1021 tttaaactga aaaaaaaaaa ttttccaagt ctgggggggt ggttagttca ttacccaccc
     1081 ccccaactac agaggcgaca attattaaaa acggatgaca ttttttaact gtcgtcaatg
     1141 tagttaatca attaaaaaca aaaataattt ttaattgata tttaaagata aggattatat
     1201 aattaagtat tacaattttt taaaatttta ctacttttaa tttttaatta tggtttaaag
     1261 atttataaat attaatatag taagtattac aaccgttaag gttttactac tttttaataa
     1321 acattaatta attttaatat agtataatat catattatga tatgctacta ataaattaat
     1381 tatggtttaa agacatatta ttatttataa aatatgacga catttaaaaa taatttagat
     1441 tattctaaac tcttatttga caatgtttta aatgatttga aaaaatcttt tatatattat
     1501 aagatatatt ctaaacaaac tatagaccaa tgtgccgaat gtgataaaca cggtcttcaa
     1561 gttgaagaac cgtgttataa aatttacagt gatattatgg ctatagaatt ttcagacata
     1621 aaatattatg ttttttatag ggttagtcat tgtattaatt ctatttttta tcatacatga
     1681 tttttaatta tttataattt taaattttaa ttgatattta aagaaatgaa tttattaata
     1741 aaaaacttta aaaaatgccc cgcgtaaatg ctaaatcttc aagaattcca agaaaaagaa
     1801 ttattaaagg taaaggtgct tatattaatc taaaacaacc gtataaacga tatgctaaga
     1861 atgctcttcg tgctgtttct attggtgctg gtgcatattt aggtcgttct tcttatggca
     1921 taatgggtaa gaatgccgtt tttatggata ctgtcgctaa aaaaacaggt gatattattt
     1981 ttggacaagg tgcttataag accaaactta atagaaacag tcttatttac gcatctcccg
     2041 ttccggctgt tcattctgtc ggtgagagta taagaatacg tcacacagag tgtttatatg
     2101 atatatcgag ttctacgact tcgttcgttc agacaagata tgctattaat cccgctgtaa
     2161 gtgctacttt tccgtggttg tcaactattg ctagttcttt tcaagaatat cgctttgaag
     2221 gcttgatttt cgaatttagg agcagtgctg gtgaaagtat aaattccgct tcgaataatc
     2281 tcggttctat tatgatgacg acacaatata atccgtatga tttaccattt attaacaaaa
     2341 atcagttgtt aaatgaaagt ggtgcggttg aagctaaacc aagtcagaat ttaatccacg
     2401 gtgttgaatg ctctccacaa ttaaatacat tatcaacttt atatactcgt tacggtgccg
     2461 cacccgctgg gtctgacatt agattatttg atttgggtgt tacaaccgtg gcaacttacg
     2521 gaagtcaggc ggttaatgat ataggaagtt tatatgtatc atatgatgta gttttaataa
     2581 aatctaaaat agtttcggct cttgccttga ctaacgaaac tacccatttt tcacaaactg
     2641 ggacaagtgc cactttaacc caaccattaa ttagtcttct taatcgtttt gattttatcg
     2701 gtattactat aactggtggc aatactatcg ttttacccgt aggcagtcaa ggtaaatatt
     2761 ttataaatct tacttgggct ggcactacgg cacaaattgc tccacttgta gggacaactt
     2821 tgacaaatgc gactatagtg ccgtctttta caaataataa ttatgttcaa tcaccgcaaa
     2881 acaccgttat aactgcttct ttaagttttc aacttgttat taatattata gacccgtctg
     2941 tattaacaac tattttaatt ttaggtgtta ctgggacttt tccaactggg acaactgggg
     3001 ccggttgtga tttatatatt aatcaaataa ataatgcagt tgtttaaaaa actttttcaa
     3061 ttttttcaac ttttttaact tttttaaaaa taataagaaa tacttattat tttttctaat
     3121 taatttttat atttttataa ttttattttc atttttaaaa ataatttttc ataatctata
     3181 attttttata atgtgggctc gatttattga aaaaggaccg agggcccaaa aggaccgcca
     3241 cggtcatttt tcaattaata gagagaccgc tataattatt tatatatact attatatata
     3301 aatataatta aacataatta aacatagtct atataatatt tatatagact atgcgatatg
     3361 tttttttaag aagaaattaa atgaattctt aaattaaaac atatcaagat aagatatatc
     3421 atatatatta taattataat atttttatca tacgagtaat aaacttaatt attacagatt
     3481 taaaaaaacc tactggattt ttttacaatc tttaattatt aatttattaa gagttataat
     3541 tttattaatg taataaaaca ttaattaaag atattttaat tattatttaa agatttaaat
     3601 aatattataa a
//
LOCUS       CruV-318                3611 bp    DNA     circular     24-FEB-2020
DEFINITION  Crucivirus-318 isolate 001394_virusbaton.
ACCESSION   CruV-318
KEYWORDS    .
SOURCE      Crucivirus-318
  ORGANISM  Crucivirus-318
            Unclassified.
REFERENCE   1  (bases 1 to 3611)
  AUTHORS   de la Higuera,I., Kasun,G.W., Torrance,E.L., Pratt,A.A.,
            Maluenda,A., Colombet,J., Bisseux,M., Ravet,V., Dayaram,A.,
            Stainton,D., Kraberger,S., Zawar-Reza,P., Goldstien,S., Briskie,J.,
            White,R., Taylor,H., Gomez,C., Ainley,D.G., Harding,J.S.,
            Fontenele,R.S., Schreck,J., Ribeiro,S.G., Oswald,S.A., Arnold,J.M.,
            Varsani,A., Enault,F. and Stedman,K.M.
  TITLE     Direct Submission
  JOURNAL   Submitted (24-FEB-2020) Biology, Portland State University, 1719 SW
            10th Ave, Portland, Oregon 97201, USA
COMMENT     Bankit Comment: ALT EMAIL:ide@pdx.edu
            Bankit Comment: TOTAL # OF SEQS:451
            Bankit Comment: GAP: est. length - alignment

            ##Assembly-Data-START##
            Assembly Method       :: MetaSPAdes v. 3.12; IDBA-UD v. 1.1.1;
                                     MEGAHIT v. 1.0.3
            Sequencing Technology :: 454; Illumina
            ##Assembly-Data-END##
FEATURES             Location/Qualifiers
     source          1..3611
                     /organism="Crucivirus-318"
                     /mol_type="genomic DNA"
                     /isolate="001394_virusbaton"
                     /isolation_source="Water"
                     /country="France"
                     /collection_date=2013
     CDS             1..1461
                     /codon_start=1
                     /product="Putative Replication Associated Protein"
                     /translation="MTTDPNHTYGELRAKDAAFGKHRVWTPNKDQPAGKWRAFCFTAF
                     DMRWDIRECLEWKNVKYLTIGFEHCKNGRPHYQGAVYFVNARACGGFMKALPAGTNFR
                     VARGSEASNEAYTQKESTVLTIGVPSRQGERTDLESIRIRIINEGATPADLVCEQGVD
                     FKKLQFAEAVFRLAGNAPKRAWKTSVVWLWGGAGKGKSTVARSLMRNIDACDIWTSSA
                     KLDYWQQYNGQDTVWFDEFRGDKCTFTNLLQILDSTPFECNVKYGSQQLLAKRIVITS
                     SMHPKDAYSGCAENVNQLLRRIEVIWNIDELYAACADKYVFPEVPFFDDFGTSVNLCA
                     ESKLTAKNPIRYEGRVCFELVSDEWVPCVEQKVLGSCVPALGVKGDDSAQAQAPLTQP
                     TVPAQKWGGNTGPDSGTTSLHASTTGPPPTSDIKCSVEHVPTEYCEDCCPHEATADDG
                     LTEDGSEICLYCGAYVMEVDADGDEIPSDIDEANAE*"
     CDS             complement(1521..3026)
                     /codon_start=1
                     /product="Putative CruV Capsid Protein"
                     /translation="MPQRKVVTVRVPAKKKMVVVTRAKAPAARKPRAVGTPAYKAKGG
                     SKSGFFGTMGGTAGGMLGTALGGPAGGMIGSTLGKYAGAALSSIFGHGDYEASNANQI
                     KNNNLVLSNSAQAPQFGSGKVACKFVHREYLGDVFSSSTANTFKIDSYPINPGQSKTF
                     PWLSGVVGAKFQQYRINGCVFEFRSMSSDALNSTNTALGSVIMSTDYDSADATFRSKQ
                     EMENTEYGVSCKPSSNMMHGIECARFQTPVSELFIRAFDVPADKDIRLYDLGRFSIAS
                     TGCQGTNVNLGELWVAYDIDGFKAIEQVPAYLTPYSQYGLTGQSATAPLGTARSALIT
                     DTTTPDFNSQQQIALTFTENKISWPIDIDVGSVYLVDLAVLGAAAAALTPPAVTTGNG
                     MFININGFWKSFTAGTTNVMHLMYTIKYEGGATPGALPYLNVADYTWAPAGGILQADL
                     IVTQISGQFGGTFSSQSEITHDDDHEDTAPGYISVCEDEKKTVTVHRRAVR*"
ORIGIN
        1 atgaccaccg accctaatca tacttatggg gaactccgcg ctaaagacgc agcgtttggc
       61 aagcatcgtg tgtggacacc taataaagat caacccgctg gcaaatggcg agcattctgc
      121 tttaccgcgt ttgatatgag gtgggacatt cgtgaatgtc ttgaatggaa aaacgtcaag
      181 tacttgacta ttggttttga gcattgcaaa aacggcaggc cgcactacca gggggcagta
      241 tactttgtga atgctcgcgc gtgtggaggc tttatgaaag ctcttcccgc gggcaccaac
      301 tttcgcgtcg ctaggggtag cgaggcatct aacgaggcct acacgcaaaa agaatctacg
      361 gttcttacca ttggtgttcc gtctcgccaa ggcgaacgga ctgatcttga gagtattcgt
      421 atccgaatca taaatgaggg ggctactccc gctgatcttg tttgtgaaca aggtgtagat
      481 ttcaagaaac tacagttcgc agaggcggtg ttccgccttg ctggtaacgc acctaaacgt
      541 gcttggaaaa caagtgttgt ttggctttgg ggcggggccg gcaaaggtaa gagtacggtg
      601 gcccgttctc ttatgcgcaa tatcgatgcc tgtgacatct ggacgtcttc agcgaagctg
      661 gactattggc aacaatacaa tggtcaggac actgtatggt ttgatgagtt caggggcgat
      721 aaatgcacct tcactaatct gttgcaaata cttgacagca ctcctttcga gtgcaacgta
      781 aagtacggtt cgcaacagtt gctcgccaaa cgaatcgtga tcacgagttc gatgcatccg
      841 aaggacgcat actctggctg cgcggaaaac gtcaatcagc tcctccgtcg gattgaagtt
      901 atctggaaca tcgatgaact ctatgcagct tgcgccgaca agtacgtgtt ccccgaggtt
      961 cccttcttcg acgatttcgg cacctcagtg aacttatgtg ccgagtcgaa gttgaccgcc
     1021 aaaaacccaa tacgctacga aggccgcgtt tgcttcgagc tcgtctctga cgaatgggta
     1081 ccttgtgtgg aacaaaaggt attggggtct tgtgtgccgg cacttggggt gaagggagac
     1141 gattctgcac aagcgcaggc tcccctaacc caacctacgg ttccggcaca gaagtggggg
     1201 ggtaatactg gccctgatag tggtactacg tcattacatg cctcgaccac agggccgccc
     1261 cccacttctg atattaaatg ctctgtcgag catgttccta cggagtattg cgaggactgc
     1321 tgtcctcacg aagccactgc cgatgatggt ctcaccgaag atggctctga aatatgcctc
     1381 tattgtggag catatgtcat ggaggttgat gctgatggtg atgaaatccc atcagatatt
     1441 gatgaagcta atgccgaata atcaacattg atgatccgca tacttcacaa aaaatttttt
     1501 agacacgtta agcctttagc ttatcgaaca gctcgtcgat ggacggtcac tgtctttttt
     1561 tcgtcctcgc acaccgagat atagccgggg gctgtatcct catgatcgtc atcatgggta
     1621 atctcagact gtgacgagaa cgttcctcca aactggcccg atatctgtgt gactatcagg
     1681 tcagcttgta gaataccacc cgcgggcgcc catgtatagt cggccacgtt caaatacggg
     1741 agtgcgcctg gcgttgcgcc tccttcatac ttgatggtat acatcagatg catcacattt
     1801 gttgttccag cagtgaaaga tttccagaat ccgttgatgt ttataaacat cccgtttccg
     1861 gttgtcactg ctggtggtgt aagtgcggct gccgcggcac ctaacactgc caaatccacc
     1921 agatacacac ttccaacgtc aatgtcgata ggccagctaa tcttattttc cgtgaaggtg
     1981 agtgcaattt gctgctgcga attgaaatct ggtgttgtgg tatctgtgat taatgcagat
     2041 cgagcagtcc ctaaaggggc tgtcgctgac tgcccagtca gaccgtattg agaatacgga
     2101 gtcagatagg ctggcacctg ctcaattgct ttgaacccgt cgatatcgta tgctacccaa
     2161 agctcaccaa ggttcacatt ggtcccttgg catcctgtgg acgcaatgga gaatcgaccc
     2221 aaatcgtaca accgaatatc cttatccgct ggtacgtcaa aggctcggat gaacaattcg
     2281 gacacgggag tctgaaagcg cgcgcattca ataccgtgca tcatgtttga tgatggtttg
     2341 caactaacac cgtactcggt gttttccatc tcttgtttgg acctaaatgt cgcatctgct
     2401 gaatcatagt ccgtcgacat gatcacagac ccaagagctg tgttggttga attcagcgca
     2461 tcagatgaca tgcttcgaaa ctcgaagaca cagccattga ttctgtactg ctgaaattta
     2521 gcacctacta ctcccgacaa ccaaggaaag gtcttcgact gtcctgggtt gatcggatag
     2581 ctatcaattt tgaacgtatt cgctgtagac gagctaaaca catctccgag atattctcga
     2641 tggacaaact tgcaagcaac cttgcccgat ccaaattgcg gtgcctgagc cgaattcgac
     2701 aatacaagat tattattctt gatttgattc gcattcgacg cttcataatc tccgtgcccg
     2761 aagattgatg aaagggcggc tccggcatac ttgcctagcg ttgagccaat catgccccct
     2821 gcgggtccgc ctaatgctgt tcccagcata ccgccagcgg ttccacccat agtaccaaag
     2881 aatccgctct tgcttcctcc tttggcctta taggcggggg tgcctactgc cctcggcttt
     2941 cttgcagccg gggctttggc ccttgtgacg acgaccatct tcttctttgc gggcacacgt
     3001 accgtgacaa ctttacgctg aggcatgttg taagaggaag tcgggctgcg acgtcaggat
     3061 atgataccga attatattcc tttaatttgc aatttaattg cccatacatt gcagcatgac
     3121 gcgcccatgc cgctgatgcc gaatcatatc gctctagctc tttcgcaagt gctagctgct
     3181 cgagtaagac tttgtcatac tcatgccacc aatcgagttc ctctactgtc ggcatcctcg
     3241 tatgattaat atttataatt gcttaaaaat ataaatcccc tgttgcaact cggggttgcg
     3301 tgaacaggtc tactcgctat acaccacctg ctggggcatt aatgtaaaga tcatatttcc
     3361 cccacgggga accacgtgga gcactcaggg ttgagtgtgc ccccttcggg ggcagtgcaa
     3421 cctgtgaggt ttgcacttgg cctagtcagg catatgaccc ttttttacat caccgttcca
     3481 ctatgcgcag ttagtgcata ggtcgggcca tgtactgacc ctcgtacctc gaacggggtg
     3541 attaatttta ttttagaaaa agatataccg atggtatacc tgcgcgaact tctgttccag
     3601 cttgttccaa a
//
LOCUS       CruV-319                3613 bp    DNA     circular     24-FEB-2020
DEFINITION  Crucivirus-319 isolate 120_418_IR1_3296.
ACCESSION   CruV-319
KEYWORDS    .
SOURCE      Crucivirus-319
  ORGANISM  Crucivirus-319
            Unclassified.
REFERENCE   1  (bases 1 to 3613)
  AUTHORS   de la Higuera,I., Kasun,G.W., Torrance,E.L., Pratt,A.A.,
            Maluenda,A., Colombet,J., Bisseux,M., Ravet,V., Dayaram,A.,
            Stainton,D., Kraberger,S., Zawar-Reza,P., Goldstien,S., Briskie,J.,
            White,R., Taylor,H., Gomez,C., Ainley,D.G., Harding,J.S.,
            Fontenele,R.S., Schreck,J., Ribeiro,S.G., Oswald,S.A., Arnold,J.M.,
            Varsani,A., Enault,F. and Stedman,K.M.
  TITLE     Direct Submission
  JOURNAL   Submitted (24-FEB-2020) Biology, Portland State University, 1719 SW
            10th Ave, Portland, Oregon 97201, USA
COMMENT     Bankit Comment: ALT EMAIL:ide@pdx.edu
            Bankit Comment: TOTAL # OF SEQS:451
            Bankit Comment: GAP: est. length - alignment

            ##Assembly-Data-START##
            Assembly Method       :: MetaSPAdes v. 3.12; IDBA-UD v. 1.1.1;
                                     MEGAHIT v. 1.0.3
            Sequencing Technology :: 454; Illumina
            ##Assembly-Data-END##
FEATURES             Location/Qualifiers
     source          1..3613
                     /organism="Crucivirus-319"
                     /mol_type="genomic DNA"
                     /isolate="120_418_IR1_3296"
                     /isolation_source="Water"
                     /country="Norway"
                     /collection_date=2011
     misc_feature    join(3598..3613,1..22)
                     /note="stem-loop"
     misc_feature    1..9
                     /note="nonanucleotide"
     CDS             93..926
                     /codon_start=1
                     /product="Putative Replication Associated Protein"
                     /translation="MPPRHRNFVFTYNNYPNTDLVDNVACKYIAYSHEVAPTTNTPHL
                     QGFITFNSVKSYNQVINLLPGCHVEAMRGSIAENEDYCSKAGNLIERGEKPISNDNRG
                     RAESERWQRAKDLAKEGKIDDIDADIYIRCYSTLKSIAKDHMIKPPPAEVQCFWIHGD
                     TGTGKSHAVETKFPDCYKKSMDDLKWFDGYEDEEVVYLEDFDVYQIKWGGMLKRLADR
                     WPMQASIKGSMKYIRPKMVIVTSNYTPDQIWSDPQTLNPLMRRFQLIHKQNQEQVIEF
                     E*"
     CDS             1059..1571
                     /codon_start=1
                     /product="putative ORF"
                     /translation="MKKNRGVKKRSYPKRKPKQWYRPGTGYVRTYVRKGPVYTPIGQK
                     RERKRYLGPAALAALRKLLPGTVDVIMGPPVVPVTGGQKASTPHAVVALDQYPLTPES
                     KKIIANYVTPPIIRDRPMHPAIRRKAAELFPKMYAMRGFYADKATRTRFKKALGPSYI
                     VDGVNRRRMK*"
     CDS             1591..2817
                     /codon_start=1
                     /product="Putative CruV Capsid Protein"
                     /translation="MKRKGSYMRGRGDYTQGYVNNKGITNYSLINWGQTNSMQSNALT
                     GSGDYKMGHTPSSNSIWNSTATPKFQQRGRGNIITNREYIRDITIPALSNGQFNVERV
                     VVQPGLATTFPWLATIAQNYEQFKILGMIFEFKSTLGDNSLVQNLGTVIMGTEYNVSS
                     ADFVNKQVMENSEFAQSARSTASQIHGIECASKESPTKLLYVRTGVLPLANVAAGDNL
                     KWYDYANFQIATVGFPLIGAAPLNIGELWVSYQIEFFKPQIPSNIGGVVQTGHVYRTG
                     AGDTDGLSILGQNTVYSSGSLSLEVLNEQMTIKGVFPSNVYALSLMWIGGGVNYVSPG
                     VVFTGADLKALYNNKGNSAVEGAGAPGATDTAQAMYEIAFTANATSVTVALTDYAIPV
                     NSGLDIWVRGIDNLTV*"
     CDS             complement(2840..3232)
                     /codon_start=1
                     /product="putative ORF"
                     /translation="MLSRVVFFYDYVSHPLYKNAIPPRLFYHKYFPNLMNPDSLFFLN
                     PSPDYPTPEEPTAATTQILTRSQAQQEYDAAQCLLKGNLLSTLSDHLHCAWNGWEMAV
                     KFEDEERQDYYMKFVVSLHDQIKKVRKF*"
ORIGIN
        1 tactattacc tagacctcct gatcatgatc actacgccac acctggcgcg agattatgat
       61 cagaaatcac cctagtgatt taatcaaaaa aaatgccacc ccgtcatcgt aattttgtat
      121 ttacttacaa taattaccct aacactgatt tggtggataa tgtcgcgtgt aagtatattg
      181 catactctca tgaagttgct cctactacta acactccgca tctgcaagga tttatcacgt
      241 ttaattctgt caagagttac aaccaggtta tcaaccttct tcctggatgt cacgttgaag
      301 caatgcgcgg aagcattgct gaaaacgaag actattgctc taaagctgga aatcttattg
      361 aacgtggcga gaaaccaatt tccaacgata atcgagggcg tgctgagagc gaacgatggc
      421 aacgcgctaa agatctcgcg aaagaaggca agattgacga tattgatgct gatatttata
      481 ttcgttgtta ctcaactctt aagtctatcg ccaaggacca tatgatcaaa ccaccacctg
      541 cggaagtcca atgcttttgg attcacggtg acactggcac tggcaagtcg catgctgtgg
      601 aaacgaaatt tcctgattgc tacaagaaaa gtatggacga cttgaaatgg tttgatggct
      661 acgaagacga ggaagtagtg tatttggagg attttgatgt atatcaaatt aaatggggcg
      721 gtatgttgaa gagactcgca gatcgctggc caatgcaagc aagtatcaaa ggctcgatga
      781 agtatatccg ccctaaaatg gtaattgtta cttctaatta cactcctgac caaatttggt
      841 ctgatcctca aacacttaat ccgttaatgc gacgatttca gcttatccac aaacaaaatc
      901 aagaacaagt tattgagttt gaataaatga acgattttta cagtgttatg acatttattg
      961 ccgcgtttac atctattttg actggtttgg ttgctgcgtt tcattttcgt tcgaagtgtg
     1021 ttgttgggga taatacaaca acagtttaac caataaaaat gaaaaagaac agaggtgtta
     1081 aaaaacgttc gtatccaaaa cgcaaaccaa aacagtggta ccgtccggga actgggtatg
     1141 ttcggacata tgttcgaaaa ggacctgttt atactccgat tgggcagaaa cgtgagagaa
     1201 aacgatattt aggacctgca gcgttggcag ctctaagaaa actgctgcca gggactgttg
     1261 atgttattat gggaccacca gtggtaccag taacaggcgg gcaaaaagct tcgacacctc
     1321 atgctgtcgt tgctttggat cagtatcctt tgacgccgga gtcaaaaaaa ataattgcca
     1381 attatgtaac tccgcctatt atcagagatc ggcctatgca tccagcaatt cgccgaaaag
     1441 cggcagaatt atttccgaaa atgtatgcta tgagagggtt ctatgcggat aaggcgaccc
     1501 gaacgagatt taagaaggcg cttggtcctt cttatattgt tgacggagtt aatagacgac
     1561 gtatgaaata attctttcct aaataaaaat atgaaacgta aaggttctta tatgagagga
     1621 cgtggtgatt acacccaagg ttatgtaaac aacaagggta tcaccaatta ttctctgatt
     1681 aattggggtc aaacgaacag tatgcaaagc aatgctctca ctgggagtgg ggattataag
     1741 atgggtcaca ccccatcttc taattctatt tggaattcca cagcaacacc gaaatttcaa
     1801 caaagaggac gcgggaatat tatcactaat agggagtata ttagggatat tactattcca
     1861 gcgttatcaa acggccagtt taatgttgaa agggttgtag tacaaccagg tttggctact
     1921 acttttccat ggttggctac tattgcgcaa aattatgaac aatttaaaat attgggaatg
     1981 atctttgagt ttaagtcaac acttggtgat aactctcttg tccagaattt gggaactgtg
     2041 attatgggta cggagtacaa tgtcagctct gctgattttg ttaacaaaca agtcatggaa
     2101 aatagtgaat tcgcccaatc tgctagatca acagcatctc aaatccacgg gattgaatgt
     2161 gctagcaagg agtctccaac taaattgtta tatgtccgaa caggtgttct acctttagcc
     2221 aatgttgccg caggggacaa tttgaaatgg tatgattatg ccaactttca aattgcaact
     2281 gttgggtttc ctcttattgg agctgctcct cttaacattg gcgaattgtg ggtttcttat
     2341 caaatagaat tctttaaacc tcaaattcca tcaaatattg gtggagttgt gcaaactggc
     2401 catgtttatc gaactggagc tggtgacacg gatgggttat ctattcttgg acagaatacc
     2461 gtgtattctt caggaagtct aagtttggaa gttctaaatg aacaaatgac catcaaaggt
     2521 gtattcccta gtaacgttta cgcgttgtct cttatgtgga taggaggagg tgtgaattat
     2581 gttagtcctg gtgtagtttt cactggagcc gatttgaaag ctctgtataa caataagggc
     2641 aattcagctg ttgaaggtgc cggagctcct ggtgccacag acactgccca agcaatgtat
     2701 gaaatagcgt ttactgccaa tgctacttcg gtgacagtag cactcactga ctatgcaatc
     2761 cctgtaaatt cagggttgga tatttgggtt cgtggcattg ataatttgac cgtttaggtt
     2821 aaccaaaata tattaagtat taaaactttc gtactttttt gatttgatcg tggagagaga
     2881 ctacaaactt catgtagtag tcttggcgct cttcgtcttc aaatttaaca gccatttccc
     2941 acccattcca agcacaatgt aagtggtcgg agagggtgga gagcaagttt cctttcaaca
     3001 aacattgcgc ggcatcgtat tcttgttgag cttgagaacg cgttagaatt tgagtagtag
     3061 cagcagttgg ttcttctgga gtaggataat caggagaagg gttgagaaag aagagagaat
     3121 cagggttcat tagattagga aaatatttgt ggtaaaatag tcgcgggggg attgcgttct
     3181 tatatagtgg atggcttacg taatcataaa aaaaaacaac acgtgaaagc agatcggatc
     3241 gagatgctta cgtaatcccc gatctattat tccgcagatc ttgatctaat tatccgctga
     3301 tctgtccgaa cacttcggac atgtccggac atttcggaca taataaaaac ataaactagg
     3361 gttcagctac tctgccggga gtttgataga gcgagcgtga agaaatcgct tgcgatttta
     3421 gtcgagtgat atcaacacga ccagtccctt agaagcagtc gctcaacctt tactgcagcg
     3481 catagtttat gttggcccct gccaggcctc cggaggtgca taacatacta ttaacccagt
     3541 ataattcata ctgggcgagc ctccgcggcg actgagcggc acacgttagc gcggtgatca
     3601 agaggtctag cat
//
LOCUS       CruV-320                3613 bp    DNA     circular     24-FEB-2020
DEFINITION  Crucivirus-320 isolate 120_1225_IR2_3297.
ACCESSION   CruV-320
KEYWORDS    .
SOURCE      Crucivirus-320
  ORGANISM  Crucivirus-320
            Unclassified.
REFERENCE   1  (bases 1 to 3613)
  AUTHORS   de la Higuera,I., Kasun,G.W., Torrance,E.L., Pratt,A.A.,
            Maluenda,A., Colombet,J., Bisseux,M., Ravet,V., Dayaram,A.,
            Stainton,D., Kraberger,S., Zawar-Reza,P., Goldstien,S., Briskie,J.,
            White,R., Taylor,H., Gomez,C., Ainley,D.G., Harding,J.S.,
            Fontenele,R.S., Schreck,J., Ribeiro,S.G., Oswald,S.A., Arnold,J.M.,
            Varsani,A., Enault,F. and Stedman,K.M.
  TITLE     Direct Submission
  JOURNAL   Submitted (24-FEB-2020) Biology, Portland State University, 1719 SW
            10th Ave, Portland, Oregon 97201, USA
COMMENT     Bankit Comment: ALT EMAIL:ide@pdx.edu
            Bankit Comment: TOTAL # OF SEQS:451
            Bankit Comment: GAP: est. length - alignment

            ##Assembly-Data-START##
            Assembly Method       :: MetaSPAdes v. 3.12; IDBA-UD v. 1.1.1;
                                     MEGAHIT v. 1.0.3
            Sequencing Technology :: 454; Illumina
            ##Assembly-Data-END##
FEATURES             Location/Qualifiers
     source          1..3613
                     /organism="Crucivirus-320"
                     /mol_type="genomic DNA"
                     /isolate="120_1225_IR2_3297"
                     /isolation_source="Water"
                     /country="Norway"
                     /collection_date=2011
     misc_feature    join(3598..3613,1..22)
                     /note="stem-loop"
     misc_feature    1..9
                     /note="nonanucleotide"
     CDS             93..926
                     /codon_start=1
                     /product="Putative Replication Associated Protein"
                     /translation="MPPRHRNFVFTYNNYPNTDLVDNVACKYIAYSHEVAPTTNTPHL
                     QGFITFNSVKSYNQVINLLPGCHVEAMRGSIAENEDYCSKAGNLIERGEKPISNDNRG
                     RAESERWQRAKDLAKEGKIDDIDADIYIRCYSTLKSIAKDHMIKPPPAEVKCFWIHGD
                     TGTGKSHAVETKFPDCYKKSMDDLKWFDGYEDEEVVYLEDFDVYQIKWGGMLKRLADR
                     WPMQASIKGSMKYIRPKMVIVTSNYTPDQIWSDPQTLNPLMRRFQLIHKQNQEQVIEF
                     E*"
     CDS             1591..2817
                     /codon_start=1
                     /product="Putative CruV Capsid Protein"
                     /translation="MKRKGSYMRGRGDYTQGYVNNKGITNYSLINWGQTNSMQSNALT
                     GSGDYKMGHTPSSNSIWNSTATPKFQQRGRGNIITNREYIRDITIPALSNGQFHVERV
                     VVQPGLATTFPWLATIAQNYEQFKILGMIFEFKSTLGDNSLVQNLGTVIMGTEYNVSS
                     ADFVNKQVMENSEFAQSARSTASQIHGIECASKESPTKLLYVRTGVLPSANVAAGDNL
                     KWYDYANFQIATVGFPLIGAAPLNIGELWVSYQIEFFKPQIPSNIGGVVQTGHVYRTG
                     AGDTDGLSILGQNTVYSSGNLSLEVQNEQMTIKGVFPSNVYALSLMWIGEGVNYVSPG
                     VVFTGADLKALYNNKGNSAVEGAGAPGATDTVQAMYEIAFTANATSVTVALTDYAIPV
                     NSGLDIWVRGIDNLTV*"
     CDS             complement(2840..3232)
                     /codon_start=1
                     /product="putative ORF"
                     /translation="MLSRVVFFYDYVSHPLYKNAIPPRLFYHKYFPNLMNPDSLFFLN
                     PSPDYPTPEEPTAATTQILTRSQAQQEYDAAQCLLKGNLLSTLSDHLHCAWNGWEMAV
                     KFEDEERQDYYMKFVVSLHDQIKKVRKF*"
ORIGIN
        1 tactattacc tagacctcct gatcatgatc actacgccac acctggcgcg agattatgat
       61 cagaaatcac cctagtgatt taatcaaaaa aaatgccacc ccgtcatcgt aattttgtat
      121 ttacttacaa taattaccct aacactgatt tggtggataa tgtcgcgtgt aagtatattg
      181 catactctca tgaagttgct cctactacta acactccgca tctgcaagga tttatcacgt
      241 ttaattctgt caagagttac aaccaggtta tcaaccttct tcctggatgt cacgttgaag
      301 caatgcgcgg aagcattgct gaaaacgaag actattgctc taaagctgga aatcttattg
      361 aacgtggcga gaaaccaatt tccaacgata atcgagggcg tgctgagagc gaacgatggc
      421 aacgcgctaa agatctcgcg aaagaaggca agattgacga tattgatgct gatatttata
      481 ttcgttgtta ctcaactctt aagtctatcg ccaaggacca tatgatcaaa ccaccacctg
      541 cggaagtcaa atgcttttgg attcacggtg acactggcac tggcaagtcg catgctgtgg
      601 aaacgaaatt tcctgattgc tacaagaaaa gtatggacga cttgaaatgg tttgatggct
      661 acgaagacga ggaagtagtg tatttggagg attttgatgt atatcaaatt aaatggggcg
      721 gtatgttgaa gagactcgca gatcgctggc caatgcaagc aagtatcaaa ggctcgatga
      781 agtatatccg ccctaaaatg gtaattgtta cttctaatta cacccctgac caaatttggt
      841 ctgatcctca aacacttaat ccgttaatgc gacgatttca gcttatccac aaacaaaatc
      901 aagaacaagt tattgagttt gaataaatga acgattttta cagtgttatg acatttattg
      961 ccgcgtttac atctattttg actggtttgg ttgctgcgtt tcattttcgt tcgaagtgtg
     1021 ttgttgggga taatacaaca acagtttaac caataaaaat gaaaaagaac agaggtgtta
     1081 aaaaacgttc gtatccaaaa cgcaaaccaa aacagtggta ccgtccggga actgggtatg
     1141 ttcggacata tgttcgaaaa ggacctgttt atactccgat tgggcagaaa cgtgagagaa
     1201 aacgatattt aggacctgca gcgttggcag ctctaagaaa actgctgcca gggactgttg
     1261 atgttattat gggaccacca gtggtaccag taacaggcgg gcaaaaagct tcgacacctc
     1321 atgctgtcgt tgctttggac cagtatcctt tgacgccgga gtcaaaaaaa ataattgcca
     1381 attatgtaac tccgcctgtt atcagagatc ggcctatgca tccagcaatt cgccgaaaag
     1441 cggcagaatt atttccgaaa atgtatgcta tgagagggtt ctatgcggat aaggcgaccc
     1501 gaacgagatt taagaaggcg cttggtcctt cttatattgt tgacggagtt aatagacgac
     1561 gtatgaaata attctttcct aaataaaaat atgaaacgta aaggttctta tatgagagga
     1621 cgtggtgatt acacccaagg ttatgtaaac aacaagggta tcaccaatta ttctctgatt
     1681 aattggggtc aaacgaacag tatgcaaagc aatgctctca ctgggagtgg ggattataag
     1741 atgggtcaca ccccatcttc taattctatt tggaattcca cagcaacacc gaaatttcaa
     1801 caaagaggac gcgggaatat tatcactaat agggagtata ttagggatat tactattcca
     1861 gcgttatcaa acggccagtt tcatgttgaa agggttgtag tacaaccagg tttggctact
     1921 acttttccat ggttggctac tattgcgcaa aattatgaac aatttaaaat attgggaatg
     1981 atctttgagt ttaagtcaac acttggtgat aactctcttg tccagaattt gggaactgtg
     2041 attatgggta cggagtacaa tgtcagctct gctgattttg ttaacaaaca agtcatggaa
     2101 aatagtgaat ttgcccaatc tgctagatca acagcatctc aaatccacgg gattgaatgt
     2161 gctagcaagg agtctccaac taaattgtta tatgtccgaa caggtgttct accttcagcc
     2221 aatgttgccg caggggacaa tttgaaatgg tatgattatg ccaactttca aattgcaact
     2281 gttgggtttc ctcttattgg agctgctcct cttaacattg gcgaattgtg ggtttcttat
     2341 caaatagaat tctttaaacc tcaaattcca tcaaatattg gtggagttgt gcaaactggc
     2401 catgtttatc gaactggagc tggtgacacg gacgggttat ctattcttgg acagaatacc
     2461 gtgtattctt caggaaatct aagtttggaa gttcaaaatg aacaaatgac catcaaaggt
     2521 gtattcccta gtaacgttta cgcgttgtct cttatgtgga taggagaagg tgtgaattat
     2581 gttagtcctg gggtagtttt cactggagcc gatttgaaag ctctgtataa caataagggc
     2641 aattcagctg ttgaaggtgc cggagctcct ggtgccacag acactgtcca agcaatgtat
     2701 gaaatagcgt ttactgccaa tgctacttcg gtgacagtag cactcactga ctatgcaatc
     2761 cctgtaaatt cagggttgga tatttgggtt cgtggcattg ataatttgac cgtttaggtt
     2821 aaccaaaata tattaagtat taaaactttc gtactttttt gatttgatcg tggagagaga
     2881 ctacaaactt catgtagtag tcttggcgct cttcgtcttc aaatttaaca gccatttccc
     2941 acccattcca agcacaatgt aagtggtcgg agagggtgga gagcaagttt cctttcaaca
     3001 aacattgcgc ggcatcgtat tcttgttgag cttgagaacg cgttaaaatt tgagtagtag
     3061 cagcagttgg ttcttctgga gtaggataat caggagaagg gttgagaaag aagagagaat
     3121 cagggttcat tagattagga aaatatttgt ggtaaaatag tcgcgggggg attgcgttct
     3181 tatatagtgg atggcttacg taatcataaa aaaaaacaac acgtgaaagc agatcggatc
     3241 gagatgctta cgtaatcccc gatctattat tccgcagatc ttgatctaat tatccgctga
     3301 tctgtccgaa cacttcggac atgtccggac atttcggaca taataaaaac ataaactagg
     3361 gtttagctac tctgccggga gtttgataga gcgagcgtga agaaatcgct tgcgatttta
     3421 gtcgagtgat atcaacacga ccagtccctt agaagcagtc gctcaacctt tactgcagcg
     3481 catagtttat gttggcccct gccaggcctc cggaggtgca taacatacta ttaacccagt
     3541 ataattcata ctgggcgagc ctccgcggcg actgagcggc acacgttagc gcggtgatca
     3601 agaggtctag cat
//
LOCUS       CruV-321                3614 bp    DNA     circular     24-FEB-2020
DEFINITION  Crucivirus-321 isolate 001383_virusbaton.
ACCESSION   CruV-321
KEYWORDS    .
SOURCE      Crucivirus-321
  ORGANISM  Crucivirus-321
            Unclassified.
REFERENCE   1  (bases 1 to 3614)
  AUTHORS   de la Higuera,I., Kasun,G.W., Torrance,E.L., Pratt,A.A.,
            Maluenda,A., Colombet,J., Bisseux,M., Ravet,V., Dayaram,A.,
            Stainton,D., Kraberger,S., Zawar-Reza,P., Goldstien,S., Briskie,J.,
            White,R., Taylor,H., Gomez,C., Ainley,D.G., Harding,J.S.,
            Fontenele,R.S., Schreck,J., Ribeiro,S.G., Oswald,S.A., Arnold,J.M.,
            Varsani,A., Enault,F. and Stedman,K.M.
  TITLE     Direct Submission
  JOURNAL   Submitted (24-FEB-2020) Biology, Portland State University, 1719 SW
            10th Ave, Portland, Oregon 97201, USA
COMMENT     Bankit Comment: ALT EMAIL:ide@pdx.edu
            Bankit Comment: TOTAL # OF SEQS:451
            Bankit Comment: GAP: est. length - alignment

            ##Assembly-Data-START##
            Assembly Method       :: MetaSPAdes v. 3.12; IDBA-UD v. 1.1.1;
                                     MEGAHIT v. 1.0.3
            Sequencing Technology :: 454; Illumina
            ##Assembly-Data-END##
FEATURES             Location/Qualifiers
     source          1..3614
                     /organism="Crucivirus-321"
                     /mol_type="genomic DNA"
                     /isolate="001383_virusbaton"
                     /isolation_source="Water"
                     /country="France"
                     /collection_date=2013
     misc_feature    join(3604..3614,1..17)
                     /note="stem-loop"
     misc_feature    1..9
                     /note="nonanucleotide"
     CDS             complement(161..1378)
                     /codon_start=1
                     /product="Putative Replication Associated Protein"
                     /translation="MCFDECTMWSTCLGFGRGCKCTPCRERASSREEKKEVTPSIEGP
                     TPTLGETPTVDEGIIPSEAPKESAFYKLKARNWVFTINNYDDEVIALVKTWEQAKDFK
                     CLMYGKEVAPTTGTPHLQGFICFETIKSGKQVAALRANNYWAPMRMKVENNAIYCSKD
                     NTDVTVLGTLPMSQAAKGKCGVLGKEHGKKAANQWDDLVKDVEAGMTKKEVERKYAKL
                     VGQYGKGVDRLYESFKPKGDFDILKIYGSFYPWQRTLMDEIEKGADKRTVHWFWSKDG
                     NAGKSDMCKHLAFQNGFQPLMNGTTKDLACAWKCGSVCMDYARDTEAKELNYSVLEAI
                     KNKYLFSSKYDSQTKQSEAFKDVFVVCFSNAPPNVSKLSTDRWKIYRIENNTAILQTI
                     VGESVIDALMVTP*"
     CDS             1513..2910
                     /codon_start=1
                     /product="Putative CruV Capsid Protein"
                     /translation="MPQVRRAAPARKVARPPARRVAAAPARRAPARRAAPAPRAPKKR
                     SEAGGAFRSGGSVLGGMLGTALGGPAGGAVGGLIGRGAGAMFSKLLGHGDYTVSNVDQ
                     LKHNNIALSNAAQLPIFGSGKVACKFVHREFLGDVYSSATPGAFKIDSYPINPGQSET
                     FPWLSGVVGAKFQQYRINGLSFEFRSMSSDALNSTNTALGSVIMSTDYDSADNVFTSK
                     QEMENTEYGVSCKPSANMLHGIECERFQTPVSELYIRAFDVPAGKDIRLYDLGRFSIA
                     TVGCQGAGVNLGELWATYDIDAFKAIEQPPQYLTPVAQYKLVGVDATHALGTSQTQTI
                     DQIGVQFEYAAGVASVIMPHDMPPGSIFSFYWAVYGASTAAIPPGDVPVISPSLDALG
                     TGNGWQGYSAMPYPTTQTSDAKGFYSTYKYDGTGTPSNPPKLSVAGGVVPATITVAGL
                     TISMMSALTPADGLA*"
ORIGIN
        1 tattattacc gaccccccct aatgatattt tgtgtaacat aaatctctag gattcacggt
       61 ttttttgaac tttagagcta aaaaaagggg gtagcgtttc gcgaccccta gtgattttta
      121 tttctgttca atagaaccta gtgatattga catttagagc ttatggcgta accataagcg
      181 catcaataac agattcacct acaatcgttt gtaagatcgc tgtattgttt tcaatcctgt
      241 agatcttcca acgatcagta gatagcttag aaacgttggg aggtgcattt gagaagcaaa
      301 caacgaaaac gtctttgaac gcttcagact gcttcgtctg gctgtcatat ttggaactga
      361 acaagtactt gttcttgatc gcttccaata ctgaatagtt caattccttt gcctctgtgt
      421 cacgggcgta gtccatgcat acagagccgc atttccaagc gcaagctaga tccttagtag
      481 ttccattcat gagtggctgg aagccatttt ggaacgctaa gtgcttgcac atgtccgatt
      541 taccggcatt gccgtccttg gaccagaacc agtgaacggt ccttttatcg gcaccttttt
      601 cgatctcatc cataagagtt ctctgccatg gatagaacga accatagatt ttgagaatat
      661 caaagtctcc tttaggtttg aacgactcgt agaggcgatc cacaccttta ccatattgac
      721 ctactagttt agcgtattta cgttcaactt ccttcttggt cataccagct tcgacatctt
      781 tgacgagatc gtcccattga ttcgcagcct tctttccatg ctctttacca agcacaccac
      841 acttgccttt ggcagcttgt gacattggaa gagttccgag gacagtaacg tcagtgttgt
      901 ctttggaaca gtagatagcg ttgttctcaa ccttcatacg cattggagcc caatagttat
      961 tggctctcaa ggcagctact tgcttgccag atttgattgt ttcgaaacag ataaatccct
     1021 gtaagtgagg agttccggtc gttggcgcaa cctctttgcc gtacataaga cacttgaagt
     1081 ccttcgcttg ctcccaagtc ttaaccaggg cgatcacttc atcatcgtag ttgttgattg
     1141 tgaagaccca gttacgagcc tttagcttat agaatgcaga ctctttcgga gcttcgctcg
     1201 gaatgatgcc ttcgtcaaca gtgggcgttt cgcctagtgt tggagttggt ccctcgatag
     1261 agggagtaac ttctttcttc tcttctcttg atgatgctcg ttcacgacat ggtgtacatt
     1321 tgcaacctcg gccgaagccc agacaggttg accacatggt acattcatcg aagcacattg
     1381 tcgtaagggg taagtcgtca gactatatac ccctaatgat aattgttagg aaggaaatta
     1441 attaattgtc tttaggagtt cacggcctat agtatatgct gtgcacttac ctaagtacat
     1501 actactaaca tcatgcctca agttcgccgt gctgctccag ctcggaaagt tgctcgtccc
     1561 cccgctcggc gtgtagccgc agctcctgct cgtcgtgctc ctgctcgtcg tgctgcacct
     1621 gcccctcggg ctcccaagaa acgatccgaa gccggtggcg catttcgctc cggtggtagt
     1681 gtccttggag gtatgttagg aaccgctctc ggaggccctg ccggaggagc cgtcggtggg
     1741 ttgattggac gtggcgctgg tgccatgttc agcaaactcc tcggtcacgg agactataca
     1801 gtctcaaacg tagaccaact caagcataac aacattgctt tatcaaatgc agctcagctt
     1861 ccgatctttg gatctggaaa ggtcgcgtgc aagttcgtgc atcgcgagtt ccttggtgac
     1921 gtgtacagct cggccacccc tggcgcattc aaaattgaca gttatcctat caatccagga
     1981 cagtcggaga ctttcccttg gttatccggc gtagtcggtg ccaagtttca gcagtatcgt
     2041 atcaacggct tgtccttcga gtttcgaagc atgtcgtccg atgctctgaa ttcaaccaac
     2101 acagctcttg gctcagtgat catgtctaca gactatgaca gtgcggacaa tgtgttcacc
     2161 tccaaacagg agatggaaaa cacagagtac ggtgtctcgt gcaagccctc ggctaacatg
     2221 ttgcatggta tcgagtgcga acgatttcag actccagtgt ctgagctcta cattagagcc
     2281 ttcgatgttc ccgcaggtaa ggacattcga ctctatgatc ttgggcgatt ctccatcgct
     2341 actgttgggt gtcaaggagc aggagtgaac ctcggtgagc tctgggctac ctatgacatt
     2401 gatgccttca aggctatcga acaaccacct caatatctca ctccagtcgc acaatacaag
     2461 ctcgttggtg ttgatgctac tcatgccctc ggcacttctc aaactcaaac gatcgatcag
     2521 attggtgtcc aattcgagta tgcagccggt gtggctagtg tcattatgcc tcatgacatg
     2581 ccaccgggat ctatcttctc gttctattgg gcagtgtatg gagcaagcac agctgcaatc
     2641 cctcctgggg atgttccagt aatttctcca agtttggatg cactcggaac aggtaacgga
     2701 tggcaaggat actctgctat gccgtaccca acaacacaga cttccgatgc aaaagggttc
     2761 tactctactt acaagtatga tggaacaggc actccttcga accctcctaa attgtctgtc
     2821 gcaggtggag ttgtccctgc aactatcacc gtagcaggtc taacaatcag catgatgagt
     2881 gctctcactc ctgcggatgg tcttgcataa ctagggattg gccccgaaga gactgagttt
     2941 actatggtct caggagacaa aaccatacaa gaagagacag gatactgtcc cgtccagtaa
     3001 caaaaaagaa caaaaaaaag ccgaaaggca ctattttttg acactctcgc gccctttccg
     3061 cacgaagtcg gccaattaca gagcaaaaaa agatctcgtc cagatatctt aacccatatg
     3121 tattggaggg ccgagaggaa gtgaaggctg acgagacgtg agtggcgggc gggtatcacc
     3181 tttaggtaca gatactcccg cccgacactt acgtcttgtc tgcctttact ttctcgataa
     3241 gccctcgagg cgtatgcctc gatgatcaca ttgatcttaa tcgtactcat agtcaaggtc
     3301 aaagacactt gcatgtagta tcgcacggtc tatagcaccg tcgtaatcgt ccatggattc
     3361 aagcacttct tggttgtaag catacatctc aagagtgtcg aagtaccact gagagaacca
     3421 aaaaccagtc ctctgctggt tacattcacg agcaagtttg ttcacttgtt gaacaagcgc
     3481 gaggtgataa ctaggagcat aagcatagcc gatgacaatg tccataagga cgtttggaag
     3541 agacatgctt cgctatatag cagtgcgcga agcggcacag aacaaaaaag tatcattatc
     3601 gtagggggtc gtta
//
LOCUS       CruV-322                3616 bp    DNA     circular     24-FEB-2020
DEFINITION  Crucivirus-322 isolate H7_8267.
ACCESSION   CruV-322
KEYWORDS    .
SOURCE      Crucivirus-322
  ORGANISM  Crucivirus-322
            Unclassified.
REFERENCE   1  (bases 1 to 3616)
  AUTHORS   de la Higuera,I., Kasun,G.W., Torrance,E.L., Pratt,A.A.,
            Maluenda,A., Colombet,J., Bisseux,M., Ravet,V., Dayaram,A.,
            Stainton,D., Kraberger,S., Zawar-Reza,P., Goldstien,S., Briskie,J.,
            White,R., Taylor,H., Gomez,C., Ainley,D.G., Harding,J.S.,
            Fontenele,R.S., Schreck,J., Ribeiro,S.G., Oswald,S.A., Arnold,J.M.,
            Varsani,A., Enault,F. and Stedman,K.M.
  TITLE     Direct Submission
  JOURNAL   Submitted (24-FEB-2020) Biology, Portland State University, 1719 SW
            10th Ave, Portland, Oregon 97201, USA
COMMENT     Bankit Comment: ALT EMAIL:ide@pdx.edu
            Bankit Comment: TOTAL # OF SEQS:451
            Bankit Comment: GAP: est. length - alignment

            ##Assembly-Data-START##
            Assembly Method       :: MetaSPAdes v. 3.12; IDBA-UD v. 1.1.1;
                                     MEGAHIT v. 1.0.3
            Sequencing Technology :: 454; Illumina
            ##Assembly-Data-END##
FEATURES             Location/Qualifiers
     source          1..3616
                     /organism="Crucivirus-322"
                     /mol_type="genomic DNA"
                     /isolate="H7_8267"
                     /isolation_source="Water"
                     /country="New Zealand"
                     /collection_date=2012
     misc_feature    join(3605..3616,1..19)
                     /note="stem-loop"
     misc_feature    1..9
                     /note="nonanucleotide"
     CDS             complement(217..1914)
                     /codon_start=1
                     /product="Putative CruV Capsid Protein"
                     /translation="MVFHLILSFLLHFTKTEYDLVPDYPKNLGKRKRTYDAQEEADDQ
                     YYLRKRRRTEPTVPAIAEPVYAIALRDNPPPPKPARTRRSATGGVQFASSRPYRESFS
                     FGPSGPPKPPRTPRSRSYTRRRAPARRPYTRRARVTRAVYRQKRFAFQRSYRLAPALG
                     VLGSLAGTYLGGPSTAALGATVGNALGHGFKTLTGFGDYHVRENTLSPGSPPAMANGA
                     LPGGACIISHREYLGDIISNSVANTFHSQNFSINPGLANTFPWLSQLATNFTQFRIMG
                     MAFEFRSMSADALNSTNTALGQVIIATNYDPASPAFSSKAEMENSAYAQSIKPSSSVL
                     HLIECDTSTMPVSEMYVRSGDIPTGTDIRFYDLGTTTVATNGFQGTSVNAGELWVTYQ
                     VLLLKPKIVDGLGEDVSYWAGYFAGGITNALPLGPNDPVLPSDPNTNSLAVDFPTSGI
                     IDIGVQPYKKCFQFDFAWSGSLTSSLSAPSYALVGLSTTEAVYNPTAADWTTEFQTPS
                     VSASQSSTRLMWTIILQCDGSSSACRLILSTGTLPSAATACALRISELPSDAAFPDS*
                     "
     CDS             complement(1943..3094)
                     /codon_start=1
                     /product="Putative Replication Associated Protein"
                     /translation="MSTARRISSVRSRGRSWCFTLNNPVDSDYGILEDVGAISRYLIY
                     GKEHFSSCSSDSTPHLQGYVMFENPKEFNTVKSLLGSRCHLEVARGTPQDNIDYCSKG
                     NQPKKEWEDLGVLGPNYGVGVDITSFGIPPMTQKRKGEKGGLAQKEKWAKIRKLADEG
                     NLDEIGENEPKVYVTHYRTLKQIAVDHLSKPANLESPAAIWCCGVTGVGKSTKYRSLF
                     PDAYVKNHSKWWDGYQGQDIVILEDISKFHVKLGDLMKLWSDKWSFICEYKGGSRWIR
                     PKLFIVTSQYTIEGIWDDEETRTALKRRFRYDFMSDIDGYSADPISESDGIFVPFLGG
                     GFLPNPLDDISRVPETPPFGAQPLMTPSPLHEVFSPINLDDLDILDLEL*"
ORIGIN
        1 cagtattacc taggacccta gtgtgccagt gccgaaaaaa taacagcttt agctaattat
       61 tttgaaggca tgtaaggcag tcactgatag cctagtgttt agatcggctc gcgttagacg
      121 cttgtgcttg ggatcgagac acgccctgta aatatattta tttattttca gtgagtgtct
      181 ttttattttt tttattattt ttaatcaaaa aacggattat gaatccggga atgcagcatc
      241 agagggtagt tcactgatgc gtaaagcaca agcggttgcc gcagaaggaa gcgtgccggt
      301 acttagtatt aaacggcacg cggacgagct tccatcgcat tggaggatga tagtccacat
      361 aagacgagtg gaagactgcg atgcgctaac gctgggcgtt tggaactccg tagtccaatc
      421 cgctgcggta ggattgtata cagcttcggt agtagacaat cctactagag cgtaggaagg
      481 agcagacaaa cttgaggtaa gtgatcctga ccatgcgaaa tcgaattgga aacatttttt
      541 gtaaggttga actccaatat cgattatccc tgatgtgggg aaatccacag caagggagtt
      601 agtatttgga tcggatggta gtacaggatc atttggaccc aatggtaatg cgttggtgat
      661 tccaccagcg aagtaaccag cccaatagga gacatcttct cccaagccat caacgatttt
      721 gggtttcagg agcaagactt ggtatgtaac ccataattcc ccggcgttaa cggaagtacc
      781 ttggaaaccg ttggtagcaa cggttgtagt accaaggtcg taaaatcgga tatccgttcc
      841 ggttggtata tcccctgaac gtacgtacat ctcagatact ggcattgtgg aagtatcgca
      901 ctcaataagg tgtaaaacac tcgaagaggg ttttatgctt tgtgcgtaag cggagttttc
      961 catctccgct ttagaagaga aggcaggact cgcaggatca taattggtag caattatgac
     1021 ttgtcccaat gcagtatttg tagagttcag ggcgtctgca gacatggaac ggaattcgaa
     1081 agccattccc atgatacgga actgagtgaa gttggttgcg agttgagaca accatgggaa
     1141 tgtattggct aagccgggat ttatggagaa gttttgcgag tgaaacgtat tggcgaccga
     1201 gttggagatg atatctccga ggtattctcg gtgggagatg atgcaggctc ctccggggag
     1261 tgcgccgttt gccatcgccg gaggcgatcc aggagagaga gtattctcac gaacgtgata
     1321 gtcaccgaac ccagtaaggg ttttaaaacc atgacccaga gcatttccga cggtagcacc
     1381 gagggccgca gtggagggtc ccccaagata tgttcctgcg aggctgccta gtacgcctaa
     1441 cgccggagcg agtcggtaag agcgctgaaa cgcgaagcgt ttctggcggt aaaccgctct
     1501 agtaacacgc gctcttctag tataaggccg acgagcgggt gcccggcgtc tagtatatga
     1561 acgtgaacga ggcgttcttg ggggttttgg cggcccggaa ggtccgaagg aaaatgactc
     1621 tcggtaaggt cgactagagg caaactgcac gcctccggta gctgatcgtc tcgttcttgc
     1681 tggctttgga ggaggaggat tgtcacgtag tgcgatcgcg taaacaggtt ctgctattgc
     1741 aggaactgtt ggttctgttc ttctacgctt gcgtagatag tattgatcgt cagcctcctc
     1801 ttgagcgtcg taggttcttt tcctttttcc taaattttta ggataatcag gaacaagatc
     1861 gtactccgtt ttcgtaaaat gcagcaagaa cgatagtatt aggtgaaata ccattgtatt
     1921 gtaatacgat ggtaatgatt tattagagtt caagatctaa aatatcaaga tcatcgaggt
     1981 ttatcggtga gaaaacctca tgaagaggag acggagtcat taatggctga gcaccaaatg
     2041 gcggagtctc cggaactcta gatatatcat ctaaagggtt tggaagaaac cctccgccaa
     2101 ggaacggcac gaagatgcca tctgactcgc tgataggatc tgcactgtaa ccatcgatgt
     2161 cggacatgaa atcatatcgg aatcttctct taagtgccgt tctcgtctct tcgtcgtccc
     2221 aaataccctc gatggtatac tgcgaagtga cgataaaaag ctttggccga atccaacgcg
     2281 atcctccctt gtactcacaa ataaatgacc atttatcaga ccaaagcttc atcaaatcac
     2341 ctaatttgac atggaacttc gagatgtctt cgagaatgac aatgtcctgg ccctggtatc
     2401 catcccacca cttggagtgg ttcttcacgt aagcatccgg aaatagcgat ctgtatttgg
     2461 tggacttccc gacacccgta acaccacaac accagatcgc tgcaggtgat tccagattcg
     2521 caggcttgct gagatgatcc actgcgatct gcttgagggt acgataatga gtaacataaa
     2581 ccttaggctc attctctcca atctcatcga gattaccctc atcagcaagt ttccggattt
     2641 tagcccactt ttctttctgg gccagtccac ctttttctcc ttttctcttc tgagtcatcg
     2701 gaggaattcc aaaggaagta atatcaacac caactccgta gttgggacca agaacaccta
     2761 aatcttccca ttccttcttt ggttgatttc ctttggaaca ataatcgatg ttgtcttgcg
     2821 gagtgccacg agcaacttct agatgacaac gagatccgag aagactctta acagtattaa
     2881 attctttggg attctcgaac ataacgtatc cttgaagatg cggagtagaa tcggaggaac
     2941 aagacgagaa atgttctttt ccgtagatca gataacgaga gattgctccg acatcctcga
     3001 gaattccata atcagaatct actggattgt tcagtgtaaa gcaccaactg cgacctctag
     3061 acctaactga agaaatcctc cttgcagtag acattcttga tttatttgaa tttaaaagag
     3121 aaattgaaaa cactagctaa atgagcacat ttttttgacc aataaaatgg tgccacgtgt
     3181 acaagtgcgt catgtcatga cgtatttttt tagaccacaa cgcggcaaca ccgcgtaaag
     3241 gtctaaaagt gacaccatct caaaaaggag atatccgcaa ccaatgaaat gctgccacgt
     3301 cagcatcact aaaaaaggag atatccgcaa ccaatcagtg actgacacgt gtcactagat
     3361 catcaaatga ctagatgccc aagtgactag atcaactgat gactagatgc ccagtgataa
     3421 ctaaaaacaa tccagaaaac aatgtcttgg cgaagccacg cgggagtcaa atgaccacag
     3481 gcccccctac cttgtataca acaaggaaac aattgacttg gcgttagcca cgcgggaaac
     3541 attgacttgg cggagccacg cgggagccaa taaaaaaacc ctttgaaaaa agagtgtggc
     3601 acagagggtc ctagta
//
LOCUS       CruV-323                3616 bp    DNA     circular     24-FEB-2020
DEFINITION  Crucivirus-323 isolate H10_16055.
ACCESSION   CruV-323
KEYWORDS    .
SOURCE      Crucivirus-323
  ORGANISM  Crucivirus-323
            Unclassified.
REFERENCE   1  (bases 1 to 3616)
  AUTHORS   de la Higuera,I., Kasun,G.W., Torrance,E.L., Pratt,A.A.,
            Maluenda,A., Colombet,J., Bisseux,M., Ravet,V., Dayaram,A.,
            Stainton,D., Kraberger,S., Zawar-Reza,P., Goldstien,S., Briskie,J.,
            White,R., Taylor,H., Gomez,C., Ainley,D.G., Harding,J.S.,
            Fontenele,R.S., Schreck,J., Ribeiro,S.G., Oswald,S.A., Arnold,J.M.,
            Varsani,A., Enault,F. and Stedman,K.M.
  TITLE     Direct Submission
  JOURNAL   Submitted (24-FEB-2020) Biology, Portland State University, 1719 SW
            10th Ave, Portland, Oregon 97201, USA
COMMENT     Bankit Comment: ALT EMAIL:ide@pdx.edu
            Bankit Comment: TOTAL # OF SEQS:451
            Bankit Comment: GAP: est. length - alignment

            ##Assembly-Data-START##
            Assembly Method       :: MetaSPAdes v. 3.12; IDBA-UD v. 1.1.1;
                                     MEGAHIT v. 1.0.3
            Sequencing Technology :: 454; Illumina
            ##Assembly-Data-END##
FEATURES             Location/Qualifiers
     source          1..3616
                     /organism="Crucivirus-323"
                     /mol_type="genomic DNA"
                     /isolate="H10_16055"
                     /isolation_source="Water"
                     /country="New Zealand"
                     /collection_date=2012
     misc_feature    join(3605..3616,1..19)
                     /note="stem-loop"
     misc_feature    1..9
                     /note="nonanucleotide"
     CDS             complement(217..1914)
                     /codon_start=1
                     /product="Putative CruV Capsid Protein"
                     /translation="MVFHLILSFLLHFTKTEYDLVPDYPKNLGKRKRTYDAQEEADDQ
                     YYLRKRRRTEPTVPAIAEPVYAIALRDNPPPPKPARTRRSATGGVQFASSRPYRESFS
                     FGPSGPPKPPRTPRSRSYTRRRAPARRPYTRRARVTRAVYRQKRFAFQRSYRLAPALG
                     VLGSLAGTYLGGPSTAALGATVGNALGHGFKTLTGFGDYHVRENTLSPGSPPAMANGA
                     LPGGACIISHREYLGDIISNSVANTFHSQNFSINPGLANTFPWLSQLATNFTQFRIMG
                     MAFEFRSMSADALNSTNTALGQVIIATNYDPASPAFSSKAEMENSAYAQSIKPSSSVL
                     HLIECDTSTMPVSEMYVRSGDIPTGTDIRFYDLGTTTVATNGFQGTSVNAGELWVTYQ
                     VLLLKPKIVDGLGEDVSYWAGYFAGGITNSLPLGTNDPVLPSDPNTNSLAVDFPTSGI
                     IDIGVQPYKKCFQFDFAWSGSLTSSLSAPSYALVGLSTTEAVYNPTAANWTTEFQTPS
                     VSASQSSTRLMWTIILQCDGSSSACRLILSTGTLPSAATACALRISELPSDAAFPDS*
                     "
     CDS             complement(1943..3094)
                     /codon_start=1
                     /product="Putative Replication Associated Protein"
                     /translation="MSTARRISSVRSRGRSWCFTLNNPVDSDYGILEDVGAISRYLIY
                     GKEHFSSCSSDSTPHLQGYVMFENPKEFNTVKSLLGSRCHLEVARGTPQDNIDYCSKG
                     NQPKKEWEDLGVLGPNYGVGVDITSFGIPPMTQKRKGEKGGLAQKEKWAKIRKLADEG
                     NLDEIGENEPKVYVTHYRTLKQIAVDHLSKPANLESPAAIWCCGVTGVGKSTKYRSLF
                     PDAYVKNHSKWWDGYQGQDIVILEDISKFHVKLGDLMKLWSDKWSFICEYKGGSRWIR
                     PKLFIVTSQYTIEGIWDDEETRTALKRRFRYDFMSDIDGYSADPISESDGIFVPFLGG
                     GFLPNPLDDISRVPETPPFGAQPLMTPSPLHEVFSPINLDDLDILDLEL*"
ORIGIN
        1 cagtattacc taggacccta gtgtgccagt gccgaaaaaa taacagcttt agctaattat
       61 tttgaaggca tgtaaggcag tcactgatag cctagtgttt agatcggctc gcgttagacg
      121 cttgtgcttg ggatcgagac acgccctgta aatatattta tttattttca gtgagtgtct
      181 ttttattttt tttattattt ttaatcaaaa aacggattat gaatccggga atgcagcatc
      241 agagggtagt tcactgatgc gtaaagcaca agcggttgcc gcagaaggaa gcgtgccggt
      301 acttagtatt aaacggcacg cggacgagct tccatcgcat tggaggatga tagtccacat
      361 aagacgagtg gaagactgcg atgcgctaac gctgggcgtt tggaactccg tagtccaatt
      421 cgctgcggta ggattgtata cagcttcggt agtagacaat cctactagag cgtaggaagg
      481 agcagacaaa cttgaggtaa gtgatcctga ccatgcgaaa tcgaattgga aacatttttt
      541 gtaaggttga actccaatat cgattatccc tgatgtgggg aaatccacag caagggagtt
      601 agtatttgga tcggatggta gtacaggatc atttgtaccc aatggtaatg agttggtgat
      661 tccaccagcg aagtaaccag cccaatagga gacatcttct cccaagccat caacgatttt
      721 gggtttcagg agcaagactt ggtatgtaac ccataattcc ccggcgttaa cggaagtacc
      781 ttggaaaccg ttggtagcaa cggttgtagt accaaggtcg taaaatcgga tatccgttcc
      841 ggttggtata tcccctgaac gtacgtacat ctcagatact ggcattgtgg aagtatcgca
      901 ctcaataagg tgtaaaacac tcgaagaggg ttttatgctt tgtgcgtaag cggagttttc
      961 catctccgct ttagaagaga aggcaggact cgcaggatca taattggtag caattatgac
     1021 ttgtcccaat gcagtatttg tagagttcag ggcgtctgca gacatggaac ggaattcgaa
     1081 agccattccc atgatacgga actgagtgaa gttggttgcg agttgagaca accatgggaa
     1141 tgtattggct aagccgggat ttatggagaa gttttgcgag tgaaacgtat tggcgaccga
     1201 gttggagatg atatctccga ggtattctcg gtgggagatg atgcaggctc ctccggggag
     1261 tgcgccgttt gccatcgccg gaggcgatcc aggagagaga gtattctcac gaacgtgata
     1321 gtcaccgaac ccagtaaggg ttttaaaacc atgacccaga gcatttccga cggtagcacc
     1381 gagggccgca gtggagggtc ccccaagata tgttcctgcg aggctgccta gtacgcctaa
     1441 cgccggagcg agtcggtaag agcgctgaaa cgcgaagcgt ttctggcggt aaaccgccct
     1501 agtaacacgc gctcttctag tataaggccg acgagcgggt gcccggcgtc tagtatatga
     1561 acgtgaacga ggcgttcttg ggggttttgg cggcccggaa ggtccgaagg aaaatgactc
     1621 tcggtaaggt cgactagagg caaactgcac gcctccggta gctgatcgtc tcgttcttgc
     1681 tggctttgga ggaggaggat tgtcacgtag tgcgatcgcg taaacaggtt ctgctattgc
     1741 aggaactgtt ggttctgttc ttctacgctt gcgtagatag tattgatcgt cagcctcctc
     1801 ttgagcgtcg taggttcttt tcctttttcc taaattttta ggataatcag gaacaagatc
     1861 gtactccgtt ttcgtaaaat gcagcaagaa cgatagtatt aggtgaaata ccattgtatt
     1921 gtaatacgat ggtaatgatt tattagagtt caagatctaa aatatcaaga tcatcgaggt
     1981 ttatcggtga gaaaacctca tgaagaggag acggagtcat taatggctga gcaccaaatg
     2041 gcggagtctc cggaactcta gatatatcat ctaaagggtt tggaagaaac cctccgccaa
     2101 ggaacggcac gaagatgcca tctgactcgc tgataggatc tgcactgtaa ccatcgatgt
     2161 cggacatgaa atcatatcgg aatcttctct taagtgccgt tctcgtctct tcgtcgtccc
     2221 aaataccctc gatggtatac tgcgaagtga cgataaaaag ctttggccga atccaacgcg
     2281 atcctccctt gtactcacaa ataaatgacc atttatcaga ccaaagcttc atcaaatcac
     2341 ctaatttgac atggaacttc gagatgtctt cgagaatgac aatgtcctgg ccctggtatc
     2401 catcccacca cttggagtgg ttcttcacgt aagcatccgg aaatagcgat ctgtatttgg
     2461 tggacttccc gacacccgta acaccacaac accagatcgc tgcaggtgat tccagattcg
     2521 caggcttgct gagatgatcc actgcgatct gcttgagggt acgataatga gtaacataaa
     2581 ccttaggctc attctctcca atctcatcga gattaccctc atcagcaagt ttccggattt
     2641 tagcccactt ttctttctgg gccagtccac ctttttctcc ttttctcttc tgagtcatcg
     2701 gaggaattcc aaaggaagta atatcaacac caactccgta gttgggacca agaacaccta
     2761 aatcttccca ttccttcttt ggttgatttc ctttggaaca ataatcgatg ttgtcttgcg
     2821 gagtgccacg agcaacttct agatgacaac gagatccgag aagactctta acagtattaa
     2881 attctttggg attctcgaac ataacgtatc cttgaagatg cggagtagaa tcggaggaac
     2941 aagacgagaa atgttctttt ccgtagatca gataacgaga gattgctccg acatcctcga
     3001 gaattccata atcagaatct actggattgt tcagtgtaaa gcaccaactg cgacctctag
     3061 acctaactga agaaatcctc cttgcagtag acattcttga tttatttgaa tttaaaagag
     3121 aaattgaaaa cactagctaa atgagcacat ttttttgacc aataaaatgg tgccacgtgt
     3181 acaagtgcgt catgtcatga cgtatttttt tagaccacaa cgcggcaaca ccgcgtaaag
     3241 gtctaaaagt gacaccatct caaaaaggag atatccgcaa ccaatgaaat gctgccacgt
     3301 cagcatcact aaaaaaggag atatccgcaa ccaatcagtg actgacacgt gtcactagat
     3361 catcaaatga ctagatgccc aagtgactag atcaactgat gactagatgc ccagtgataa
     3421 ctaaaaacaa tccagaaaac aatgtcttgg cgaagccacg cgggagtcaa atgaccacag
     3481 gcccccctac cttgtataca acaaggaaac aattgacttg gcgttagcca cgcgggaaac
     3541 attgacttgg cggagccacg cgggagccaa taaaaaaacc ctttgaaaaa agagtgtggc
     3601 acagagggtc ctagta
//
LOCUS       CruV-324                3616 bp    DNA     circular     24-FEB-2020
DEFINITION  Crucivirus-324 isolate PCD_14.
ACCESSION   CruV-324
KEYWORDS    .
SOURCE      Crucivirus-324
  ORGANISM  Crucivirus-324
            Unclassified.
REFERENCE   1  (bases 1 to 3616)
  AUTHORS   de la Higuera,I., Kasun,G.W., Torrance,E.L., Pratt,A.A.,
            Maluenda,A., Colombet,J., Bisseux,M., Ravet,V., Dayaram,A.,
            Stainton,D., Kraberger,S., Zawar-Reza,P., Goldstien,S., Briskie,J.,
            White,R., Taylor,H., Gomez,C., Ainley,D.G., Harding,J.S.,
            Fontenele,R.S., Schreck,J., Ribeiro,S.G., Oswald,S.A., Arnold,J.M.,
            Varsani,A., Enault,F. and Stedman,K.M.
  TITLE     Direct Submission
  JOURNAL   Submitted (24-FEB-2020) Biology, Portland State University, 1719 SW
            10th Ave, Portland, Oregon 97201, USA
COMMENT     Bankit Comment: ALT EMAIL:ide@pdx.edu
            Bankit Comment: TOTAL # OF SEQS:451
            Bankit Comment: GAP: est. length - alignment

            ##Assembly-Data-START##
            Assembly Method       :: MetaSPAdes v. 3.12; IDBA-UD v. 1.1.1;
                                     MEGAHIT v. 1.0.3
            Sequencing Technology :: 454; Illumina
            ##Assembly-Data-END##
FEATURES             Location/Qualifiers
     source          1..3616
                     /organism="Crucivirus-324"
                     /mol_type="genomic DNA"
                     /isolate="PCD_14"
                     /isolation_source="Soil"
                     /country="New Zealand"
                     /collection_date=2012
     misc_feature    join(3605..3616,1..19)
                     /note="stem-loop"
     misc_feature    1..9
                     /note="nonanucleotide"
     CDS             complement(217..1914)
                     /codon_start=1
                     /product="Putative CruV Capsid Protein"
                     /translation="MVFHLILSFLLHFTKTEYDLVPDYPKNLGKRKRTYDAQEEADDQ
                     YYLRKRRRTEPTVPAIAEPVYAIALRDNPPPPKPARTRRSATGGVQFASSRPYRESFS
                     FGPSGPPKPPRTPRSRSYTRRRAPARRPYTRRARVTRAVYRQKRFAFQRSYRLAPALG
                     VLGSLAGTYLGGPSTAALGATVGNALGHGFKTLTGFGDYHVRENTLSPGSPPAMANGA
                     LPGGACIISHREYLGDIISNSVANTFHSQNFSINPGLANTFPWLSQLATNFTQFRIMG
                     MAFEFRSMSADALNSTNTALGQVIIATNYDPASPAFSSKAEMENSAYAQSIKPSSSVL
                     HLIECDTSTMPVSEMYVRSGDIPTGTDIRFYDLGTTTVATNGFQGTSVNAGELWVTYQ
                     VLLLKPKIVDGLGEDVSYWAGYFAGGITNALPLGTNDPVLPSDPNTNSLAVDFPTSGI
                     IDIGVQPYKKCFQFDFAWSGSLTSSLSAPSYALVGLSTTEAVYNPTAADWTTEFQTPS
                     VSASQSSTRLMWTIILQCDGSSSACRLILSSGTLPSAATACALRISELPSDAAFPYS*
                     "
     CDS             complement(1943..3094)
                     /codon_start=1
                     /product="Putative Replication Associated Protein"
                     /translation="MSTARRISSVRSRGRSWCFTLNNPVDSDYGILEDVGAISRYLIY
                     GKEHFSSCSSDSTPHLQGYVMFENPKEFNTVKSLLGSRCHLEVARGTPQDNIDYCSKG
                     NQPKKEWEDLGVLGPNYGVGVDITSFGIPPMTQKRKGEKGGLAQKEKWAKIRKLADEG
                     NLDEIGENEPKVYVTHYRTLKQIAVDHLSKPANLESPAAIWCCGVTGVGKSTKYRSLF
                     PDAYVKNHSKWWDGYQGQDIVILEDISKFHVKLGDLMKLWSDKWSFICEYKGGSRWIR
                     PKLFIVTSQYTIEGIWDDEETRTALKRRFRYDFMSDIDGYSADPISESDGIFVPFLGG
                     GFLPNPLDDISRVPETPPFGAQPLMTPSPLHEVFSPINLDDLDILDLEL*"
ORIGIN
        1 cagtattacc taggacccta gtgtgccagt gccgaaaaaa taacagcttt agctaattat
       61 tttgaaggca tgtaaggcag tcactgatag cctagtgttt agatcggctc gcgttagacg
      121 cttgtgcttg ggatcgagac acgccctgta aatatattta tttattttca gtgagtgtct
      181 ttttattttt tttattattt ttaatcaaaa aacggattat gaatacggga atgcagcatc
      241 agagggtagt tcactgatgc gtaaagcaca agcggttgcc gcagaaggaa gcgtgccgct
      301 acttagtatt aaacggcacg cggacgagct tccatcgcat tggaggatga tagtccacat
      361 aagacgagtg gaagactgcg atgcgctaac gctgggcgtt tggaactccg tagtccaatc
      421 cgctgcggta ggattgtata cagcttcggt agtagacaat cctactagag cgtaggaagg
      481 agcagacaaa cttgaggtaa gtgatcctga ccatgcgaaa tcgaattgga aacatttttt
      541 gtaaggttga actccaatat cgattatccc tgatgtgggg aaatccacag caagggagtt
      601 agtatttgga tcggatggta gtacaggatc atttgtaccc aatggtaatg cgttggtgat
      661 tccaccagcg aagtaaccag cccaatagga gacatcttct cccaagccat caacgatttt
      721 gggtttcagg agcaagactt ggtatgtaac ccataattcc ccggcgttaa cggaagtacc
      781 ttggaaaccg ttggtagcaa cggttgtagt accaaggtcg taaaatcgga tatccgttcc
      841 ggttggtata tcccctgaac gtacgtacat ctcagatact ggcattgtgg aagtatcgca
      901 ctcaataagg tgtaaaacac tcgaagaggg ttttatgctt tgtgcgtaag cggagttttc
      961 catctccgct ttagaagaga aggcaggact cgcaggatca taattggtag caattatgac
     1021 ttgtcccaat gcagtatttg tagagttcag ggcgtctgca gacatggaac ggaattcgaa
     1081 agccattccc atgatacgga actgagtgaa gttggttgcg agttgagaca accatgggaa
     1141 tgtattggct aagccgggat ttatggagaa gttttgcgag tgaaacgtat tggcgaccga
     1201 gttggagatg atatctccga ggtattctcg gtgggagatg atgcaggctc ctccggggag
     1261 tgcgccgttt gccatcgccg gaggcgatcc aggagagaga gtattctcac gaacgtgata
     1321 gtcaccgaac ccagtaaggg ttttaaaacc atgacccaga gcatttccga cggtagcacc
     1381 gagggccgca gtggagggtc ccccaagata tgttcctgcg aggctgccta gtacgcctaa
     1441 cgccggagcg agtcggtaag agcgctgaaa cgcgaagcgt ttctggcggt aaaccgctct
     1501 agtaacacgc gctcttctag tataaggccg acgagcgggt gcccggcgtc tagtatatga
     1561 acgtgaacga ggcgttcttg ggggttttgg cggcccggaa ggtccgaagg aaaatgactc
     1621 tcggtaaggt cgactagagg caaactgcac gcctccggta gctgatcgtc tcgttcttgc
     1681 tggctttgga ggaggaggat tgtcacgtag tgcgatcgcg taaacaggtt ctgctattgc
     1741 aggaactgtt ggttctgttc ttctacgctt gcgtagatag tattgatcgt cagcctcctc
     1801 ttgagcgtcg taggttcttt tcctttttcc taaattttta ggataatcag gaacaagatc
     1861 gtactccgtt ttcgtaaaat gcagcaagaa cgatagtatt aggtgaaata ccattgtatt
     1921 gtaatacgat ggtaatgatt tattagagtt caagatctaa aatatcaaga tcatcgaggt
     1981 ttatcggtga gaaaacctca tgaagaggag acggagtcat taatggctga gcaccaaatg
     2041 gcggagtctc cggaactcta gatatatcat ctaaagggtt tggaagaaac cctccgccaa
     2101 ggaacggcac gaagatgcca tctgactcgc tgataggatc tgcactgtaa ccatcgatgt
     2161 cggacatgaa atcatatcgg aatcttctct taagtgccgt tctcgtctct tcgtcgtccc
     2221 aaataccctc gatggtatac tgcgaagtga cgataaaaag ctttggccga atccaacgcg
     2281 atcctccctt gtactcacaa ataaatgacc atttatcaga ccaaagcttc atcaaatcac
     2341 ctaatttgac atggaacttc gagatgtctt cgagaatgac aatgtcctgg ccctggtatc
     2401 catcccacca cttggagtgg ttcttcacgt aagcatccgg aaatagcgat ctgtatttgg
     2461 tggacttccc gacacccgta acaccacaac accagatcgc tgcaggtgat tccagattcg
     2521 caggcttgct gagatgatcc actgcgatct gcttgagggt acgataatga gtaacataaa
     2581 ccttaggctc attctctcca atctcatcga gattaccctc atcagcaagt ttccggattt
     2641 tagcccactt ttctttctgg gccagtccac ctttttctcc ttttctcttc tgagtcatcg
     2701 gaggaattcc aaaggaagta atatcaacac caactccgta gttgggacca agaacaccta
     2761 aatcttccca ttccttcttt ggttgatttc ctttggaaca ataatcgatg ttgtcttgcg
     2821 gagtgccacg agcaacttct agatgacaac gagatccgag aagactctta acagtattaa
     2881 attctttggg attctcgaac ataacgtatc cttgaagatg cggagtagaa tcggaggaac
     2941 aagacgagaa atgttctttt ccgtagatca gataacgaga gattgctccg acatcctcga
     3001 gaattccata atcagaatct actggattgt tcagtgtaaa gcaccaactg cgacctctag
     3061 acctaactga agaaatcctc cttgcagtag acattcttga tttatttgaa tttaaaagag
     3121 aaattgaaaa cactagctaa atgagcacat ttttttgacc aataaaatgg tgccacgtgt
     3181 acaagtgcgt catgtcatga cgtatttttt tagaccacaa cgcggcaaca ccgcgtaaag
     3241 gtctaaaagt gacaccatct caaaaaggag atatccgcaa ccaatgaaat gctgccacgt
     3301 cagcatcact aaaaaaggag atatccgcaa ccaatcagtg actgacacgt gtcactagat
     3361 catcaaatga ctagatgccc aagtgactag atcaactgat gactagatgc ccagtgataa
     3421 ctaaaaacaa tccagaaaac aatgtcttgg cgaagccacg cgggagtcaa atgaccacag
     3481 gcccccctac cttgtataca acaaggaaac aattgacttg gcgttagcca cgcgggaaac
     3541 attgacttgg cggagccacg cgggagccaa taaaaaaacc ctttgaaaaa agagtgtggc
     3601 acagagggtc ctagta
//
LOCUS       CruV-325                3619 bp    DNA     circular     24-FEB-2020
DEFINITION  Crucivirus-325 isolate 120_1229_IR2_3297.
ACCESSION   CruV-325
KEYWORDS    .
SOURCE      Crucivirus-325
  ORGANISM  Crucivirus-325
            Unclassified.
REFERENCE   1  (bases 1 to 3619)
  AUTHORS   de la Higuera,I., Kasun,G.W., Torrance,E.L., Pratt,A.A.,
            Maluenda,A., Colombet,J., Bisseux,M., Ravet,V., Dayaram,A.,
            Stainton,D., Kraberger,S., Zawar-Reza,P., Goldstien,S., Briskie,J.,
            White,R., Taylor,H., Gomez,C., Ainley,D.G., Harding,J.S.,
            Fontenele,R.S., Schreck,J., Ribeiro,S.G., Oswald,S.A., Arnold,J.M.,
            Varsani,A., Enault,F. and Stedman,K.M.
  TITLE     Direct Submission
  JOURNAL   Submitted (24-FEB-2020) Biology, Portland State University, 1719 SW
            10th Ave, Portland, Oregon 97201, USA
COMMENT     Bankit Comment: ALT EMAIL:ide@pdx.edu
            Bankit Comment: TOTAL # OF SEQS:451
            Bankit Comment: GAP: est. length - alignment

            ##Assembly-Data-START##
            Assembly Method       :: MetaSPAdes v. 3.12; IDBA-UD v. 1.1.1;
                                     MEGAHIT v. 1.0.3
            Sequencing Technology :: 454; Illumina
            ##Assembly-Data-END##
FEATURES             Location/Qualifiers
     source          1..3619
                     /organism="Crucivirus-325"
                     /mol_type="genomic DNA"
                     /isolate="120_1229_IR2_3297"
                     /isolation_source="Water"
                     /country="Norway"
                     /collection_date=2011
     CDS             1..927
                     /codon_start=1
                     /product="Putative Replication Associated Protein"
                     /translation="MAQVSQIHNVMIRISKPDNITDETWFSEVMQKLFVEIFSSLGKK
                     YIFQYEYTESCNRDEDDNIIESSKRHNHHIQGMAKLHRKQRPAAVAKTIKNMLTLEHI
                     SVVVQPAFNLAALEKYSMKDESRQRNLVFADHAIYMGEDLPSLDNLYPYQQTLYKMCK
                     NKPNDRHIYWIYDQIGNTGKSKFCKFMTYHEKTPMLTYGKNADILNLASKYPNSRTYL
                     FNLSRAKPSQIGIQDLYATLEALKDGHYVNTKYDTCAVLQNPAHIIVMANVLPDLTLL
                     SSDRWKVMTIDKNKVLQEYKQDKVEELINSLS*"
     misc_feature    17..43
                     /note="stem-loop"
     misc_feature    29..37
                     /note="nonanucleotide"
     CDS             1258..2835
                     /codon_start=1
                     /product="Putative CruV Capsid Protein"
                     /translation="MSYAPRRKAYAPRAAARPKRVYRPRAIEEVVIAAPPPPPQKKRG
                     LFSKLVRGVTKFASPLVNAGLSILQKKAMAKMGMASGHGNYSVSNNTILSPNSQIPQF
                     SNLGQSVRCAHREFIGDINSSTAFALQSYAINPGLSGTFPWLSAIANQFEQYKIHGLM
                     FEFRTTSSDALNSTNTALGTMILATEYNVLLPSFTNKQQMENTIFSTSGKPSTTLLHP
                     IECDPAQTPNQPAYVRSGATLIGDNRLYDLGNFQIASVGSQASSVVGELWITYDITFY
                     KPILNNSAAFQAKTAHYLTTNPGNVAFTSIGDPTLLVTSMDTIGGGLFDSLPLAPLSA
                     GYEFPLGSIGIYQFVYWSESGFNVFNTPATITGFGCTVLNVWSNAAGPFNRPVVYDAA
                     SVVGSAQNLLVFIINITSPTAIASVAVDLTTTYFAGFGDWTITQLNGAYPTTLAVLSA
                     AQERKQLMINNSMLSKQLAQERANKYISCASVEPTNIEEEKEDDNDELEFTQEDIKLI
                     LEMREKKKKEVKTSQKN*"
ORIGIN
        1 atggctcaag tttctcaaat tcataatgta atgataagaa tttctaagcc agataatata
       61 actgacgaaa catggttttc tgaagttatg caaaagttat tcgtagaaat attttcatct
      121 ttaggtaaga aatatatttt ccaatatgaa tatacagaaa gttgtaatcg tgatgaagat
      181 gataacataa ttgaatcttc aaagcgacat aatcatcaca ttcagggaat ggctaaacta
      241 catcgtaaac aaagaccagc agcagtagct aaaacaataa aaaatatgct aacgttagaa
      301 cacatatcag ttgtagttca accagcattc aatctagcag cgttagaaaa atactcaatg
      361 aaagatgaaa gcagacaaag aaatctagtc ttcgctgatc atgcaatata catgggtgaa
      421 gatctaccat cacttgataa tttatatcct tatcaacaaa cattatataa aatgtgtaaa
      481 aataaaccaa acgatagaca tatatactgg atatatgatc aaataggaaa tacaggtaaa
      541 tcaaaatttt gtaaatttat gacttaccac gaaaaaacac caatgttaac atatggtaaa
      601 aatgcagaca ttttaaactt agcttcaaag tatccaaatt caagaactta cttatttaat
      661 ttatcaagag ctaaaccttc tcaaatagga atacaagatc tttatgcgac attagaagct
      721 cttaaagacg gtcattacgt taatactaag tacgacacat gtgcagtact tcaaaatcca
      781 gcacatataa tagttatggc taatgtgctt cctgatctaa ctcttctttc ttctgataga
      841 tggaaagtta tgacaataga taaaaacaaa gttttacaag aatacaaaca agacaaagtt
      901 gaagagttaa taaacagtct ttcataactg tctttttcaa tgatccatta ggtccaaaga
      961 atatatatat gtatattcga taagaaataa gagggacaat tacttccagc gtagcgcaag
     1021 tccctctgta ataaaattat aataattttt tactaaaata ggagcgtagc gactttccaa
     1081 cataattaaa aattcttaat tatttctgat gaaatttgta ctcactttta agaccgtagg
     1141 gattgtacaa cattaatcat cattttttaa aaacttttaa aaacttttaa aaactaatta
     1201 attaattaat tatatttaaa catttatata tccatattta tatactcaga tattataatg
     1261 tcttatgctc ctcgaagaaa agcatatgcg cctcgtgctg ctgcaagacc aaaacgcgtt
     1321 tatcgtcctc gtgcgattga agaagtggta attgctgcac ctccacctcc tcctcaaaag
     1381 aagcgtggat tatttagtaa actcgtaaga ggtgttacta agtttgcttc tcctttagta
     1441 aatgctggtc tcagtatttt gcaaaaaaaa gcaatggcta agatgggaat ggcaagtggc
     1501 catggtaatt attcagtcag taataatact atcttatctc ctaactcaca gattcctcaa
     1561 ttttccaatt taggtcaaag tgttagatgt gctcatagag aatttatcgg agatattaat
     1621 tcttctacag cattcgcatt acaatcttat gcgatcaatc ctggcttgtc aggtactttt
     1681 ccatggttat ctgctatagc aaatcaattt gagcagtata aaatacatgg attaatgttt
     1741 gaatttcgta ctacatcttc agatgcactt aattccacta atactgcact tggtacaatg
     1801 atattagcta ctgagtataa cgtgttgtta ccatcgttta cgaataaaca acaaatggag
     1861 aataccattt tctctacaag tggtaaacct tcaaccactt tacttcatcc tattgaatgt
     1921 gatcctgcac aaacaccaaa tcaacctgca tacgtacgtt ctggtgctac tcttatcggt
     1981 gataatcgat tatatgatct tggaaatttc caaattgcat cagttggttc tcaggcatca
     2041 tccgtagttg gagaactatg gattacatat gatattacat tctataaacc cattcttaat
     2101 aattcagctg cttttcaagc taagactgca cattatttaa ccacaaatcc aggcaatgtt
     2161 gcattcactt caatcggaga tcctacatta cttgttacat caatggatac tattggtggt
     2221 ggattatttg attcattacc tcttgcacct ttaagtgctg gttatgaatt tccattaggt
     2281 tctatcggta tttaccaatt tgtatactgg tctgaatcag gattcaacgt atttaacact
     2341 cctgctacaa ttacaggttt tggatgtacg gttttaaacg tatggtctaa tgctgctgga
     2401 ccttttaatc gacccgtcgt atatgacgct gcatcagttg ttggtagtgc tcaaaatctt
     2461 ttagtattca ttattaatat tacttcacca actgctattg catctgttgc tgttgatctt
     2521 acaactactt atttcgctgg ctttggagat tggacaatca ctcaattaaa tggcgcatac
     2581 cctactacac ttgctgtatt aagcgctgca caagaaagaa aacaactaat gataaacaat
     2641 tccatgttat ctaaacaatt agctcaagaa agagcaaaca aatatataag ctgtgcttca
     2701 gtagaaccaa caaatatcga agaggaaaaa gaagacgata atgatgaact agagttcaca
     2761 caagaagata taaaactcat tctagaaatg agagaaaaaa agaagaaaga agtaaagact
     2821 tcacagaaga attaaataat tattggaaaa ataaatattt agacttaaaa gatttagtaa
     2881 ttaaatattg tacactaaca caagaagaaa acacgctcat agactccgaa ggagcaccct
     2941 actttaattg aaagtattac ataaaagaaa aattttaaaa ataagaataa attataatat
     3001 aatttttttt acattttttc aaattttaat ttattttcaa aataaatcta attgaagctt
     3061 tattacctct tagcgaattt tttgacatta aaaaaaccca aaaacttcaa tcaaaataat
     3121 aaaaattatt aataattttt taacacattt aattaaactt ttttttaatg atttacaaaa
     3181 aagtattaaa ggtttaagaa gatctatttt tactaaaaat tacaattaga aataagtaaa
     3241 aaaaattata ttataattta ttcttatttt taaaattttt ctaggtaaaa attccaatac
     3301 ctttagggta agattacgga gtaatagaac aaataaaaat tcaaacataa ataaaattat
     3361 ttgatattcc aaataaattc atttcaactc acactagtta attaatcatt ttacataatt
     3421 tagggtttac ctaaagttat tatcacatta attaaaattt aatcaaaatt cgaacaaagt
     3481 tcgtatgagg gccccgccac tcgagcgcag cgacgtggca ggcctcaatc agatatgaac
     3541 aaaacaatac caaagttcaa ataaaattat ttgctatttc aaataattaa ataatatata
     3601 aacctatata tatactatc
//
LOCUS       CruV-326                3620 bp    DNA     circular     24-FEB-2020
DEFINITION  Crucivirus-326 isolate PCD_205.
ACCESSION   CruV-326
KEYWORDS    .
SOURCE      Crucivirus-326
  ORGANISM  Crucivirus-326
            Unclassified.
REFERENCE   1  (bases 1 to 3620)
  AUTHORS   de la Higuera,I., Kasun,G.W., Torrance,E.L., Pratt,A.A.,
            Maluenda,A., Colombet,J., Bisseux,M., Ravet,V., Dayaram,A.,
            Stainton,D., Kraberger,S., Zawar-Reza,P., Goldstien,S., Briskie,J.,
            White,R., Taylor,H., Gomez,C., Ainley,D.G., Harding,J.S.,
            Fontenele,R.S., Schreck,J., Ribeiro,S.G., Oswald,S.A., Arnold,J.M.,
            Varsani,A., Enault,F. and Stedman,K.M.
  TITLE     Direct Submission
  JOURNAL   Submitted (24-FEB-2020) Biology, Portland State University, 1719 SW
            10th Ave, Portland, Oregon 97201, USA
COMMENT     Bankit Comment: ALT EMAIL:ide@pdx.edu
            Bankit Comment: TOTAL # OF SEQS:451
            Bankit Comment: GAP: est. length - alignment

            ##Assembly-Data-START##
            Assembly Method       :: MetaSPAdes v. 3.12; IDBA-UD v. 1.1.1;
                                     MEGAHIT v. 1.0.3
            Sequencing Technology :: 454; Illumina
            ##Assembly-Data-END##
FEATURES             Location/Qualifiers
     source          1..3620
                     /organism="Crucivirus-326"
                     /mol_type="genomic DNA"
                     /isolate="PCD_205"
                     /isolation_source="Soil"
                     /country="New Zealand"
                     /collection_date=2012
     CDS             1..1266
                     /codon_start=1
                     /product="Putative Replication Associated Protein"
                     /translation="MSNPQQHSDEEEEVPQSNAVAKPVVQAKPVVQEVMEGEPVYNQR
                     NRRWIFTWNNYPALTDQQFLQLFQNYFKETLRYVAWSREIGKKGTPHLQGFIYFHEAK
                     VLGKPLQNGNYTKGSMKGFEREVRWAIMITDFSTNTSYCSKKGTEDLFEWGKPPAEEG
                     EGGGRGARNDLSGLCRQIIDGQLTRDQMYEQFPEMMVKHSRGFENLFSYIQEHRRKAP
                     YVVWVYGQSGSGKSQFAEDMHGRDNVYRKMNGGLFFNGYNGQLACVIEEFRVISSSNP
                     RGWVFIELLQFLDKYQISCEIKGGSMKFNSPFLYITSVEHPRDVFKDDYEFEQIVRRM
                     FCFCYIDNNGNQIEDEKYRGQYFDPNRPPIITKASERFSALMAREARERETHTRERAM
                     NQLMKSAERELALKGKVASATLPKYDDEK*"
     CDS             1396..1941
                     /codon_start=1
                     /product="putative ORF"
                     /translation="MSNPNQQDDGDVSSTHSENPMQVIDLKMEAYLSVVEEMCESHAQ
                     GVGNDIYVKRIQALHVLDDILGTSKHFAVLLEDLYRSVARIPSRSVGETLKHFARTRI
                     FRMLSEQLNNVAEMLEDVENEWNDLPAVAPTLTSDMGAPAKSVSIRAPDDDDTIVLPT
                     VRHTRSQSRSSKGAQPSPFFK*"
     CDS             complement(1964..3382)
                     /codon_start=1
                     /product="Putative CruV Capsid Protein"
                     /translation="MSNLPMNQLIYAYRGRDGKTRDMREPLRTSITRQYAQLGPYQGG
                     GAYRRMRGRGSYWSDFGRKWGARAGQYLGKMIPAWAGGGAYRKKRNAGKGAYRRIRGR
                     GSYEEASSGKFAPTPPQFGTQGDANIISKREYIGDVYSSATAGAFSVEQYYLNAGDTK
                     TNPWLATLCNSSYQQYKYMGALMEFVSMSADALNSTNTALGTVVASVNYDSTDSAPTN
                     RAQMENTDWSRASKPSESFTIGVECDRKETFAGGLLNIRSSQTLPTGTDRKTYDLGVI
                     SVASVGVQGQSVLLGSLYMNYKVALYKTIQLAPGAQQGRFHWYSSTGVAVTTAYFGTD
                     TATRSPSVNNIGATINGTTLTIPRKYLVPNACYVLDVFWNSGSGVAVVLPTLTPTANQ
                     TTATNYWLSTASAANGLNVGYITNGGSTCSQLFGRWFINIVANPTSDLSLVMSVGTIP
                     TAATVSVDVQLTQVNGDPNLIL*"
ORIGIN
        1 atgtccaacc ctcaacaaca ttcagatgaa gaggaagaag ttcctcaatc caatgcggta
       61 gccaaacctg tggttcaagc taaacctgtg gttcaagaag ttatggaagg ggaaccagtc
      121 tataatcagc gtaatagacg atggatattt acttggaaca attatcctgc tctgactgat
      181 caacaattcc ttcagttatt tcagaattat ttcaaggaga ctttgcggta tgtagcgtgg
      241 tctcgggaga ttggaaagaa aggcacacct catctacaag gatttatcta tttccatgaa
      301 gcgaaggttc ttggaaaacc actccagaat ggtaattata cgaaagggag catgaaagga
      361 tttgaacgag aagttcgctg ggctattatg attactgatt tttctaccaa tacttcttat
      421 tgttcaaaga aaggtacaga agacttattt gaatggggca agccacctgc tgaagaaggt
      481 gaaggaggtg gtagaggtgc cagaaatgat ctttctggct tatgtcgtca gattattgat
      541 ggacaactca cacgagacca gatgtatgag cagtttccag agatgatggt taagcattct
      601 cgtggatttg agaatttatt ctcttatatt caagaacata ggcgaaaggc tccctatgtt
      661 gtatgggttt atggacaatc aggttcaggt aaatctcagt ttgctgagga catgcacgga
      721 cgtgataatg tctatcgtaa gatgaatggt ggtttattct ttaatggata taatggacaa
      781 cttgcttgtg ttatagaaga gtttagagtg atttcgtctt ccaatccgag aggatgggtt
      841 tttattgagc tcttacaatt cttagataag tatcagattt cctgtgaaat caaaggaggt
      901 tcaatgaaat ttaactctcc atttctatat attacttctg tggagcatcc gcgagatgta
      961 ttcaaggatg attatgaatt tgaacagatt gtcagacgta tgttctgctt ctgttatatt
     1021 gataataatg gcaatcaaat tgaagatgag aaataccgag gtcagtattt cgatcctaat
     1081 agaccgccta ttattactaa agcctcagaa aggttctccg cactgatggc acgtgaagca
     1141 cgtgagagag agacacacac tcgcgaacga gctatgaatc agctgatgaa atctgctgaa
     1201 agagagcttg ctctcaaagg caaagttgct tcagcaactt tacctaaata tgatgatgag
     1261 aaataggtgg cctggaaaca cgtagtgtta gtattaccca ggccacctct gtgtaaaata
     1321 attttttata gcttataaaa atttttctgt caccgagacc actttactag catttatgga
     1381 atcacttttt ccaaaatgtc aaaccccaac caacaagatg acggagacgt ctcctctact
     1441 cattctgaaa atccaatgca agtcattgat ttgaagatgg aagcctatct ttctgtcgtt
     1501 gaagaaatgt gcgaatcaca cgcacaaggc gttggaaatg atatttatgt gaagcgcatt
     1561 caagcgcttc atgtattgga tgatattctt gggactagca agcattttgc tgttcttctt
     1621 gaggatttgt atagatccgt tgctaggatt ccttctcgat cagtgggcga aactctcaag
     1681 cactttgcac gcacacgcat cttcagaatg ctttctgaac agcttaataa tgttgcggaa
     1741 atgcttgaag atgttgagaa tgaatggaat gatcttccag ctgtagcacc aacacttaca
     1801 agcgatatgg gcgcgcccgc caagtctgtc tctattcgtg ctcctgatga cgatgacact
     1861 attgttctac caacggttcg tcatactcgt tctcagagta ggtcttctaa gggcgcacaa
     1921 ccttctcctt tcttcaagta aatcttatat atatataaga aatttagaga atgagatttg
     1981 gatctccatt cacttgtgtg agttggacat ctacggatac ggtcgcggca gtcggaatcg
     2041 ttcccacact cataaccaga gacagatccg aggtaggatt ggcaacaata ttgatgaacc
     2101 acctgccgaa gagttgagag caggtagagc caccattggt gatataacca acgtttaggc
     2161 cgttcgctgc agaagcagta gaaagccagt agttagtcgc agttgtttga tttgcggtag
     2221 gtgtcagtgt cggaaggaca actgccacac cgcttcctga gttccagaat acatctaaca
     2281 cgtagcacgc gttagggact agatatttcc ttggaattgt cagagttgtc ccgttgatag
     2341 ttgcacctat gttattgaca ctcggagaac gagtggcagt atcagttccg aaatacgcag
     2401 tcgtaacagc gacaccagta gaggaatacc agtggaacct tccttgctga gcaccaggtg
     2461 cgagttgaat cgtcttgtaa agagcgactt tgtagttcat atacagagaa cccagaagaa
     2521 ctgattgtcc ttgtactcct acagatgcca cactgatgac accaagatcg tatgtcttac
     2581 gatcagtgcc agtgggaagt gtctgtgaag aacggatgtt caataggcct ccagcgaaag
     2641 tctccttcct atcacattct acaccaatcg tgaagctctc agaaggcttt gaggcacgag
     2701 accagtcagt attttccatc tgagcacgat ttgtaggcgc gctgtcagtg ctgtcataat
     2761 tgacggatgc tactacagta ccaagagcag tgttcgtaga gttgagtgcg tcagctgaca
     2821 tactcacgaa ctccatcaaa gctcccatat acttatattg ctgatacgag ctgttgcaaa
     2881 gcgtagcgag ccacggattt gtcttcgtat caccagcatt tagatagtat tgttctacgc
     2941 tgaacgcacc agcagtcgca gaagaataca catcaccgat atattcacgc ttgctgatga
     3001 tgttggcatc accttgagtg ccaaactgag gaggagtagg ggcaaacttg ccacttgatg
     3061 cttcctcata ggaaccacga ccacggatgc gacgatatgc tccctttcct gcattccgtt
     3121 tcttgcgata tgcaccacca ccagcccaag ctggaatcat ctttccaaga tactgaccag
     3181 cacgagctcc ccacttcctt ccgaaatcac tccagtatga gccacggcca cgcatgcgac
     3241 gatatgcacc accaccttga tatggtccca gttgtgcgta ttgccgtgtg atacttgttc
     3301 ggagaggttc tctcatatct cttgtttttc catctctacc gcggtaggcg tagatcaatt
     3361 ggttcattgg gaggtttgac attttatctc tacacaactc aaataatgct ggtaatcata
     3421 cccccaaatt gaggagggcg caaagcgccc tatctgagca cactatttgt cgttagacca
     3481 atagtctgca aacccccctt gccccctatg attatttgat gacttgtaag cgggcgacga
     3541 cgacgacgag gtgacgagga ggaggaggaa gcccgcgact ccaattctta gtccctttct
     3601 tcaacctcac cttctacaaa
//
LOCUS       CruV-327                3630 bp    DNA     circular     24-FEB-2020
DEFINITION  Crucivirus-327 isolate 120_782_SvL1_3295.
ACCESSION   CruV-327
KEYWORDS    .
SOURCE      Crucivirus-327
  ORGANISM  Crucivirus-327
            Unclassified.
REFERENCE   1  (bases 1 to 3630)
  AUTHORS   de la Higuera,I., Kasun,G.W., Torrance,E.L., Pratt,A.A.,
            Maluenda,A., Colombet,J., Bisseux,M., Ravet,V., Dayaram,A.,
            Stainton,D., Kraberger,S., Zawar-Reza,P., Goldstien,S., Briskie,J.,
            White,R., Taylor,H., Gomez,C., Ainley,D.G., Harding,J.S.,
            Fontenele,R.S., Schreck,J., Ribeiro,S.G., Oswald,S.A., Arnold,J.M.,
            Varsani,A., Enault,F. and Stedman,K.M.
  TITLE     Direct Submission
  JOURNAL   Submitted (24-FEB-2020) Biology, Portland State University, 1719 SW
            10th Ave, Portland, Oregon 97201, USA
COMMENT     Bankit Comment: ALT EMAIL:ide@pdx.edu
            Bankit Comment: TOTAL # OF SEQS:451
            Bankit Comment: GAP: est. length - alignment

            ##Assembly-Data-START##
            Assembly Method       :: MetaSPAdes v. 3.12; IDBA-UD v. 1.1.1;
                                     MEGAHIT v. 1.0.3
            Sequencing Technology :: 454; Illumina
            ##Assembly-Data-END##
FEATURES             Location/Qualifiers
     source          1..3630
                     /organism="Crucivirus-327"
                     /mol_type="genomic DNA"
                     /isolate="120_782_SvL1_3295"
                     /isolation_source="Water"
                     /country="Norway"
                     /collection_date=2012
     CDS             1..1308
                     /codon_start=1
                     /product="Putative Replication Associated Protein"
                     /translation="MIEEIKEESKEEYHLQNKKQVSFCIDNNEYFALEESIKELTIAN
                     EKPAQDKPKFRLNACRFLLTYKTHIDKNELILFFNKLCKIKKGENKPEIKFCRSGHEF
                     GTNHESDKEDYEHTHTCIEFTKNFVSQDTEIFNFVTKDKEIIHPHMKYCGKTENKKWN
                     DMKRYISKEDPENADLLLETLPLVDRVFNCETKYDVAKLCNKPGDIIGLLALHALKPI
                     DNNKSLIYKELGGDKFNLYQWQNEVYQEMINMTGNTRKVTWIFDPMGNSGKSTFAKDL
                     QSKDITYRVEGTGNGYENCAYLLGNAYKNKLWNGKNIIFDFPRGLNMMQSTSIYNMIE
                     MLCNGEISIGKYQSEKITMKQNTQVYVFANKEPFFTVDGNPTLSFDRWKCLGVKSTKD
                     GSDKVFSKKYYKETCHKINAMAKHFDQQTVISDDDEPMSLKRE*"
     misc_feature    1218..1243
                     /note="stem-loop"
     misc_feature    1224..1232
                     /note="nonanucleotide"
     CDS             complement(1924..3501)
                     /codon_start=1
                     /product="Putative CruV Capsid Protein"
                     /translation="MPTRKGTRKSRKSSRKYVVKGSGSYRRRPITSRIKRVVGSGSYH
                     TKRDYGNSRDFIQPTYGSYKSDLSSGIKSIGSGLGGMFSSLLGLGDYKVKANTLTGVG
                     PGMNDQIPAMHDINGGVRICHREYLGDIYGSINFNNVSFAIQPANSTTFPWLSQLALN
                     FEQWKPLGLIFEFRSMSSDTQIASSTALGSVVLATEYNALAPGFASKQAMENSQFVVS
                     TKPSASVIHPIECDLSQTPNQPLYIRLPQSSTGDARLFDLGNFQIATQGMLTDGSNQG
                     ELWVSYDVILLKPIIGNTLGVDIPFTHTYNKFNVGDCNDVAIFGVAPLESQNSVLNMT
                     INNTTNVVAFTPSVTYEASLLYIEFSWIYHTPQLAPLPVPNFILTNCTQANILWGSRQ
                     SFINTALSSCRVSFYLIPTDVGLPFEVAFLSALIGLVGDADVDVYATQVNSNVLNVTS
                     SFEAVSASSVAAIIDRDDAFLDRLARRMLQINDKKEEEKECDTETDEVSLLKAEVEQY
                     RALQASARKITANILKS*"
ORIGIN
        1 atgattgaag aaatcaaaga agaatcaaaa gaagaatatc atttacaaaa taagaaacaa
       61 gtttcttttt gtattgataa taacgaatac tttgctttag aagaaagtat caaagaacta
      121 actattgcaa atgagaaacc ggcacaagac aaacctaaat ttaggttaaa tgcttgccga
      181 ttcttattaa cttataaaac tcatattgat aaaaatgaat taattttatt tttcaataaa
      241 ctttgtaaaa taaagaaagg tgaaaataaa cctgaaatta aattctgtag atcaggtcat
      301 gaatttggaa caaaccatga atctgataaa gaagattatg aacatacaca tacatgtatt
      361 gagttcacta aaaattttgt aagtcaagat actgaaattt ttaattttgt tactaaggat
      421 aaagaaatta tccatccaca tatgaaatat tgtggaaaaa cagaaaacaa aaagtggaac
      481 gatatgaaaa gatacatatc gaaagaagat cctgaaaatg cggatctttt attagaaact
      541 ctaccattag ttgatcgtgt atttaattgt gaaacaaaat acgatgttgc taaattatgc
      601 aataaacctg gtgatataat tggactactt gcacttcatg cacttaagcc aattgataat
      661 aacaaatctt taatatataa agaattaggt ggtgataaat ttaatttata ccaatggcaa
      721 aatgaagttt accaagaaat gattaacatg accggtaata caagaaaagt tacttggata
      781 tttgatccaa tgggtaactc aggcaaaagt acttttgcta aagatcttca atcgaaagac
      841 attacttata gagttgaagg aaccggaaat ggttatgaaa attgcgcgta cttattaggt
      901 aacgcttata aaaataaatt gtggaatgga aagaatatta tcttcgattt tccaagagga
      961 ttaaatatga tgcaaagcac aagtatttac aatatgattg aaatgttatg taatggagaa
     1021 atttctattg gaaaatacca atcagaaaaa attacaatga agcaaaatac acaagtttat
     1081 gtttttgcaa ataaagaacc attcttcaca gtagatggaa acccaacact ttcatttgat
     1141 agatggaaat gtttaggtgt taaatccact aaagatggat ctgataaagt atttagtaaa
     1201 aaatactata aagaaacttg ccataaaata aatgcaatgg caaaacactt tgatcaacaa
     1261 actgtaatct ctgatgatga tgaaccaatg tcactaaaac gtgaataggt ataattatat
     1321 ttttttaaca aaaataaaag taacactcgc acgactcgtc gtaaactcct cgtcgtgcga
     1381 gtgtctaacg aaagtaacta tcttaataaa taagatatgg gctatgatat gttctccgac
     1441 aaatgctccg acaatcatta cttattaata aataagatat gaactatgat atgttctccg
     1501 acaaatgctc cgacaatcat tacttattag tgaataagat atggactatg atatgttctc
     1561 cgacaaatgc tccgacaatc ataaagggtc tactaaagta gagtttaggg ataagactaa
     1621 cattaaagcc ttgcaggcta tggtcttatc cttattataa taattaagaa aaacataaag
     1681 aaaaccacaa ggatttcaaa agcttttttt aatttttaga aagccatagt tttctaaaac
     1741 cgataaaaac cgttaaaaac gacccacaag gggtcgtttt taagctttgg acccgtcgtt
     1801 gaaaaaaaat ccacacaaat ttttttttcc aatttttact attaaaaaaa cttttggaac
     1861 cgtgctaaaa aaaacgttac tggtaaagca taaaaaaata atttaatcta ttataactta
     1921 gaattaactt ttgagtatat tcgcagtaat tttacgagca ctcgcttgta gtgctctata
     1981 ctgttcaacc tcagctttta aaagagaaac ttcatcagtt tcagtatcac attctttttc
     2041 ttcttctttt ttatcattaa tttgtaacat acgacgagca agacgatcaa gaaatgcgtc
     2101 atctctatcg ataatagcag caacagacga agccgaaaca gcttcaaaac tacttgtaac
     2161 atttaataca ttagaattaa cttgtgttgc ataaacatca acatcggcat cgcctacaag
     2221 tccaataaga gcagataaaa aagctacttc aaaaggtaaa ccaacatcag taggaatcaa
     2281 ataaaatgaa acacgacaac ttgacaatgc ggtattaata aatgattgac gagaacccca
     2341 aagaatattt gcttgtgtac aattagtaag tataaaatta ggaactggca aaggtgccaa
     2401 ttgaggagta tgataaatcc aactaaattc aatataaaga agagatgctt cataagtaac
     2461 agaaggagta aaagcaacta catttgttgt attattgata gtcatattaa gaacactatt
     2521 ttgagattct aaaggcgcaa caccaaagat agcaacatca ttacaatcac caacattaaa
     2581 tttattatat gtatgagtaa atggaatatc aacacccaaa gtattaccaa taatcggttt
     2641 aagtaaaata acatcataag atacccataa ctcaccctga ttagaaccat cagtcaacat
     2701 tccttgagta gcaatttgaa aattaccaag atcaaacaaa cgagcatcac cagtagatga
     2761 ttgaggaagt ctaatataca aaggctgatt aggagtttga gaaagatcac attcaatagg
     2821 atggattaca gaagcagaag gtttagtaga aactacgaat tgagaattct ccatagcctg
     2881 tttagaagca aatcccggtg ccaaagcatt atattcagta gccaatacaa cagaaccaag
     2941 agcagtagaa gatgcaattt gagtatcaga agacatagat ctaaattcaa aaatcaaacc
     3001 taatggtttc cattgttcaa aattcaatgc taactgactg agccaaggaa aagtggtaga
     3061 attagcaggt tgaattgcaa atgaaacatt attaaaatta attgaaccat aaatatcacc
     3121 aagatactca cgatgacaaa tacgaacacc accattaata tcatgcattg caggaatttg
     3181 atcattcata ccaggaccaa caccagtaag agtattagct ttaactttat aatcaccaag
     3241 tccaagaaga gaactaaaca taccacctaa tccagaacca atcgatttaa taccagaaga
     3301 caaatcagat ttgtaacttc cataagtagg ctgaataaaa tcacggctat tgccgtaatc
     3361 ccgttttgta tgataagaac cggaaccaac aactcgctta atacgcgaag taataggacg
     3421 acgcctataa gaaccagaac ctttaacaac atacttgcga gaactcttac gagattttcg
     3481 cgtacctttc cgagtcggca tgataatgat actatatcta acaatctttt ttttatttaa
     3541 ttaaataata caaaaaaaaa taaataaata attcctatta gagaagtata caagtatata
     3601 gattaatata ctatactacc caaggtccaa
//
LOCUS       CruV-328                3630 bp    DNA     circular     24-FEB-2020
DEFINITION  Crucivirus-328 isolate 120_1228_IR2_3297.
ACCESSION   CruV-328
KEYWORDS    .
SOURCE      Crucivirus-328
  ORGANISM  Crucivirus-328
            Unclassified.
REFERENCE   1  (bases 1 to 3630)
  AUTHORS   de la Higuera,I., Kasun,G.W., Torrance,E.L., Pratt,A.A.,
            Maluenda,A., Colombet,J., Bisseux,M., Ravet,V., Dayaram,A.,
            Stainton,D., Kraberger,S., Zawar-Reza,P., Goldstien,S., Briskie,J.,
            White,R., Taylor,H., Gomez,C., Ainley,D.G., Harding,J.S.,
            Fontenele,R.S., Schreck,J., Ribeiro,S.G., Oswald,S.A., Arnold,J.M.,
            Varsani,A., Enault,F. and Stedman,K.M.
  TITLE     Direct Submission
  JOURNAL   Submitted (24-FEB-2020) Biology, Portland State University, 1719 SW
            10th Ave, Portland, Oregon 97201, USA
COMMENT     Bankit Comment: ALT EMAIL:ide@pdx.edu
            Bankit Comment: TOTAL # OF SEQS:451
            Bankit Comment: GAP: est. length - alignment

            ##Assembly-Data-START##
            Assembly Method       :: MetaSPAdes v. 3.12; IDBA-UD v. 1.1.1;
                                     MEGAHIT v. 1.0.3
            Sequencing Technology :: 454; Illumina
            ##Assembly-Data-END##
FEATURES             Location/Qualifiers
     source          1..3630
                     /organism="Crucivirus-328"
                     /mol_type="genomic DNA"
                     /isolate="120_1228_IR2_3297"
                     /isolation_source="Water"
                     /country="Norway"
                     /collection_date=2011
     CDS             complement(1..1062)
                     /codon_start=1
                     /product="Putative Replication Associated Protein"
                     /translation="MEEDNPTPDFDEDDDTQEEIGGKHRNWCFTLNNYTHVEWVNIQT
                     ILTSWKYLVVAQEIGEKKGTPHLQGYVDFNSPRGLMALKKLNKRIRWGKRGGTWEQAS
                     NYCKKGEQRKAEWKKLGIKGPNFGKNAIFVEAGEGPKQGQRNDISDVRLAIKAGKGMR
                     AICEIADSYQAIRHAEVLLKYTEKKRDFETRVYWFWGPTGSGKTRHALEMFPDAWMSG
                     KNLKWWEGYDAHEEVVIDDFRGDFCTFHELIRILDRYQYPIENKGGSRQLLARDIVIT
                     SCRGPEGTYETREDIWQLLRRIDCSYDFGSRTARYLKTEFNENSEQHMEQKSGGNTMP
                     QTGGLFGRVHAPRANSNTL*"
     misc_feature    55..83
                     /note="stem-loop"
     misc_feature    66..74
                     /note="nonanucleotide"
     CDS             1540..2832
                     /codon_start=1
                     /product="Putative CruV Capsid Protein"
                     /translation="MPNYSRRTRYARKARRIPYAQWKARQQGKYRSRRGGGWNLAPIG
                     SAIGSYFGGPVGGAIGGVAGMAANRLITGISGRGDYTVTKNTLVHPQSVPQFVSHMTD
                     GSIRIRHKEFIQDVVTSATPGGFKVETFPICASENNAFPYLSQLAVNFEEFMFEGLVF
                     TYIPSSGSLSTTGQLGTVILATQYNSLSPPFANKQAAEASTYATSQVVSQAAIHPIEC
                     DGKVTPSNGIFYNRRPDQQIGGDVRWNQLGTFSLMSQGAPNASETIGELHVSYDVVLA
                     KPILNQGTNAQADHWKATTGIAVGAGAGPYFGTDTVLTPGSDNFTNFSDNQIFIEPEF
                     NGNLSVTYAVHGAAGAWVDVLMTASTGVTNLLLLDDNITQQYVKTGAAGQLVTYTTAY
                     FTVKSTGVDSVITLSSGTFFTPDSMDLIIVQMPNELNT*"
     CDS             complement(2885..3457)
                     /codon_start=1
                     /product="putative ORF"
                     /translation="MSSSSHAGYDYEVGPSGHYVKVCPKQPSNPQLWSNVRSSGKKYP
                     RKFQEKSKFLWPKPAKPAKYQPFTQVGNGKFQWSNNDGKIVGWIPSDWKLPHPNYREG
                     IRLAKLKSLQDEIDAEGRAIEQAIFDEERAVSMKRSQRISRLEKEEAMVFKRIGHGRI
                     TAFDKERGLMRHAEYQRYLARKNHGRCDKK*"
ORIGIN
        1 ctacagtgtg ttgctgttcg cgcgaggcgc gtgtacgcga ccgaataggc cgccagtctg
       61 gggcatagta ttacccccag acttctgttc catgtgctgt tccgaatttt cattaaattc
      121 agttttcaaa tatctagcag ttctggaccc gaaatcgtat gagcaatcta ttctgcgtaa
      181 aagttgccat atgtcttccc tggtttcgta agttccttct ggtccacgac aagatgttat
      241 cactatatct cttgctaaga gttgtctaga accaccttta ttttctattg gatattggta
      301 tctgtctagt attctgatta gttcatgaaa agtgcaaaaa tctcctctaa agtcgtctat
      361 gactacttct tcgtgcgcgt cgtaaccttc ccaccattta aggttctttc cagacatcca
      421 agcatcaggg aacatttcca aagcgtgtct cgtctttcct gatccggtag gaccccagaa
      481 ccagtatact cttgtttcaa agtccctttt cttctccgta tatttcaaga gtacttcagc
      541 gtgtctgatt gcttgataac tatcagctat ttcgcatatt gcgcgcatac cttttccagc
      601 ttttatcgct agtcttacgt cggagatatc atttctttga ccttgtttag gaccttctcc
      661 ggcttcaaca aagatagcat tctttccaaa gttaggaccc tttattccca atttcttcca
      721 ttcagctttt ctttgttcac ctttcttaca gtagttagaa gcttgttccc aagtacctcc
      781 tcgttttccc caacgaattc ttttgttgag tttcttaagt gccataagac ctcttggact
      841 attaaaatca acgtaacctt gcaagtgagg tgtacctttc ttttctccta tttcttgagc
      901 gaccactaaa tatttccatg aagttaagat cgtttgaatg tttacccatt ctacgtgcgt
      961 gtagttattt agggtgaagc accaatttcg atgtttacct ccaatctctt cttgagtgtc
     1021 atcgtcttcg tcaaaatctg gagtaggatt gtcttcttcc attttaccta gtaattttta
     1081 attaagtaca tcagtttata cgagcgtagc gagcgcggta aaaggcctgt ttgactttct
     1141 aattcgcata catgacatcg tacatctaac tagtaaccac taactattaa ttatccttag
     1201 taagcggcac aaggcctact agacgacccc gcgccgaagg cggagttact ggaaacaata
     1261 ctactcatct tgtcatctaa tagattattt acaaaacatt atcaagcccc gtcgcgagta
     1321 gccttcatgg atcacgaggc cgcgaagcga tggctttcgt ggatcatgaa ttccttaagc
     1381 aattatttcc ttactaaaaa tgtgctatta tcctctatgt tctcacgagt atctcgttta
     1441 tgttttagtc tccgtccctg ataacaggat tttcatacct atataccatg gcatgcaaaa
     1501 ttgattgtct aaaaaaaaat attgcctata taaattaaca tgcccaatta ctctcgtcga
     1561 acacgatatg ctcgtaaggc ccgccgcata ccatatgcgc aatggaaggc cagacagcaa
     1621 ggtaaatacc gttctcgtag aggaggcggg tggaatctcg cccctattgg ctctgctatt
     1681 ggctcttact ttggcggtcc cgttggaggc gctatcggtg gggtagctgg aatggctgcc
     1741 aaccgtctta ttacaggtat ttctggacgt ggagattaca cagtcactaa gaatacactt
     1801 gttcatcctc aatcagtccc acaatttgtg tcacacatga ctgatggttc gatccggatt
     1861 cgtcacaaag aattcatcca ggatgttgtt acttccgcta cgccaggagg atttaaggtc
     1921 gaaacgttcc caatttgtgc gtctgaaaat aacgcatttc cttatctatc tcagcttgcg
     1981 gttaactttg aagagttcat gttcgagggc cttgtattca catatattcc tagttcagga
     2041 tctctctcaa ctactggtca gttaggtact gttattcttg ctactcagta caacagtctt
     2101 tctcctccgt ttgccaataa gcaagcagct gaagcatcca cgtatgctac ttcccaagtt
     2161 gtctctcagg ctgctattca tccaattgaa tgcgatggca aggttacacc tagtaacggt
     2221 atattctaca atcgtagacc agatcaacag attggtggcg atgttcgttg gaatcaactt
     2281 ggtactttca gtttgatgag tcaaggagct cccaatgctt ccgagactat tggtgaactt
     2341 catgttagtt atgatgttgt cctcgcaaaa ccaattctca atcaaggtac taacgctcaa
     2401 gccgatcatt ggaaggctac tactggtatc gctgttggtg caggtgctgg tccttacttt
     2461 ggcacagata ccgtacttac tcccggttct gacaacttta ccaatttctc cgacaatcag
     2521 atctttattg aacccgagtt caacggtaac ctctctgtta cttatgcagt ccatggtgcg
     2581 gctggtgctt gggttgacgt tcttatgact gccagtactg gtgtcactaa cttgcttctc
     2641 cttgatgata acattaccca gcaatacgtc aaaacgggcg ctgccggtca acttgttact
     2701 tacacaaccg cttattttac ggttaaatct actggtgtcg acagtgtaat tacgctctcc
     2761 agtggtactt tctttactcc tgattcgatg gatctcatca tcgttcagat gcccaatgag
     2821 cttaacactt aaaaaataca taaacattcg actaaacgtc gtacgttttt taatctcgat
     2881 ttttttactt cttatcacaa cgtccgtgat tctttctagc aagatatctt tgatattcag
     2941 cgtgtctcat caatccacgt tctttatcga acgctgtgat gcgaccatgt ccaattctct
     3001 tgaataccat agcttcttct ttttccaatc tggaaattcg ttgagatctt ttcatagaga
     3061 cagctctttc ttcatcgaag attgcttgtt caatcgctct accttcggca tcgatttcat
     3121 cttgaagaga tttcaattta gctagtctaa taccttctcg gtagtttgga tgaggaagtt
     3181 tccaatcact tggaatccaa ccgacgattt taccatcgtt gttactccat tggaatttac
     3241 catttcctac ttgcgtgaaa ggttgatatt ttgcaggttt cgcaggtttt ggccatagga
     3301 acttggattt ttcctgaaac ttccgaggat atttctttcc tgaagaacga acattcgacc
     3361 acaattgtgg attcgaaggt tgtttagggc aaactttgac atagtgccct gacggaccta
     3421 cttcgtagtc gtaaccggcg tggctcgaac tagacatact ttagtatgaa taaatgattt
     3481 tctagagcac aaattaaatt tgtacaatca tttttctagt cgccacgctg acgagggagt
     3541 gtccgaggac acgaccgagc ttagatcggt aaatacagca aaatgatttt gcaatatttt
     3601 tttttagcga gagaaagtca tttgttcata
//
LOCUS       CruV-329                3635 bp    DNA     circular     24-FEB-2020
DEFINITION  Crucivirus-329 isolate 120_1222_IR2_3297.
ACCESSION   CruV-329
KEYWORDS    .
SOURCE      Crucivirus-329
  ORGANISM  Crucivirus-329
            Unclassified.
REFERENCE   1  (bases 1 to 3635)
  AUTHORS   de la Higuera,I., Kasun,G.W., Torrance,E.L., Pratt,A.A.,
            Maluenda,A., Colombet,J., Bisseux,M., Ravet,V., Dayaram,A.,
            Stainton,D., Kraberger,S., Zawar-Reza,P., Goldstien,S., Briskie,J.,
            White,R., Taylor,H., Gomez,C., Ainley,D.G., Harding,J.S.,
            Fontenele,R.S., Schreck,J., Ribeiro,S.G., Oswald,S.A., Arnold,J.M.,
            Varsani,A., Enault,F. and Stedman,K.M.
  TITLE     Direct Submission
  JOURNAL   Submitted (24-FEB-2020) Biology, Portland State University, 1719 SW
            10th Ave, Portland, Oregon 97201, USA
COMMENT     Bankit Comment: ALT EMAIL:ide@pdx.edu
            Bankit Comment: TOTAL # OF SEQS:451
            Bankit Comment: GAP: est. length - alignment

            ##Assembly-Data-START##
            Assembly Method       :: MetaSPAdes v. 3.12; IDBA-UD v. 1.1.1;
                                     MEGAHIT v. 1.0.3
            Sequencing Technology :: 454; Illumina
            ##Assembly-Data-END##
FEATURES             Location/Qualifiers
     source          1..3635
                     /organism="Crucivirus-329"
                     /mol_type="genomic DNA"
                     /isolate="120_1222_IR2_3297"
                     /isolation_source="Water"
                     /country="Norway"
                     /collection_date=2011
     CDS             1..987
                     /codon_start=1
                     /product="Putative Replication Associated Protein"
                     /translation="MRGIFIFKMNPVAVYDLTISLNEYDEEVHTLQWFVKQFVSLGKK
                     WTFQQERGEETGYDHIQARVSLFKKKRLGELKKLLVAIGGPFAGAHISPTSSNGVDSG
                     DFYCCKIDTRVAGPWSDKDELVKELIDPPYIPRQVRETPNLYSWQQAIIDTKDQWCTR
                     SINCVINEKGNIGKSILVAHCRAYKHARVLPPVNDNKDLLRMVCDMPTEKMYMFDMPK
                     AMCKDRLFQFYQAIETIKDGYAYDDRYHFQEKVFDCPNVWIFTNKIPNVKLLSRDRWK
                     FWEVEDGELVKKELSEEDELDPEEAKEIVVRENNDLKVQLKLLKKRMKEASF*"
     CDS             complement(1317..1832)
                     /codon_start=1
                     /product="putative ORF"
                     /translation="MIKMEKTQDLETQAHNFSPFFSSFSVFENAVISVLEFETTVEPQ
                     EIYARITDVFETLLTEFIQKHNIDLDDALQPSEDCEMYEEVDNEPQIPDPPVTPVQPK
                     KQLGQTFLPEETRDFPLRSLAPVNRKERKSVEVPFFEDPYHQHTCKECDSPELPRHHS
                     LCYTCFRAYNY*"
     CDS             complement(2017..3420)
                     /codon_start=1
                     /product="Putative CruV Capsid Protein"
                     /translation="MKMPPRRHYNFSRAFHGKQNRQFYKKEFIPTMRNTAVYAAGKAG
                     QMAGTYYGGPFGGIAGRAAGTLTARRILGRGDYSIAPPRRAYTAGALVKQSAGFQRGH
                     SGYSRPKMSTTNDHELMITHSEYIGDLISSGTTNTFIAQNYAINPANSGTFPWLSSTA
                     INFQNYKFDKLAFEFKPLVSESTSSSTGALVSMGKVVLATQYDSCLGAYTNTQLAENS
                     EGSASTKPSEHIIMGVECKSQLNPMGVYYTSGQGSLTVGANASDIRMQNVGIFCAMAN
                     GIPTASSTAVDLGELWVHYTVRLMKPQLNAGLSNVLTAHYYGATACGSPATTSPFGAS
                     VSATVQPTATTGSYLPLTFTTNKFTFPLAITEGSYLCSYYCNGTGATSSSTAPTITNG
                     SFLPIYNAGVLNATSVNTYVSCPNGLASNISYMQQFVVSVNAPGSSLCAVTINTTVVP
                     TSGQFDLIITPYNINTL*"
ORIGIN
        1 ctgagaggaa tttttatatt taaaatgaat ccagtagccg tttatgattt aactatttcg
       61 ttaaatgagt atgatgaaga ggtgcacact cttcaatggt ttgtcaaaca gtttgtttct
      121 ttaggaaaga agtggacttt ccaacaagag agaggtgagg aaacaggata tgatcatatc
      181 caagcaaggg tttctctctt taagaagaaa agattgggcg aactaaagaa actattagtc
      241 gcaatcggag gaccatttgc aggagcacat atcagtccaa cttcatctaa cggtgtagat
      301 agtggggatt tttactgttg taaaatagac acaagggttg caggaccttg gtcagataaa
      361 gatgagttgg ttaaagaact gattgatcct ccgtatattc cacgtcaagt acgggaaaca
      421 ccaaacttgt actcatggca acaagctata attgatacta aagatcaatg gtgtacgagg
      481 tcgattaatt gtgtaattaa cgaaaaaggt aacatcggga aatctatttt ggttgcacat
      541 tgtcgagcgt ataaacacgc aagagtgtta ccacctgtaa acgacaataa agatttgtta
      601 agaatggttt gcgatatgcc aaccgagaag atgtacatgt ttgacatgcc taaagcgatg
      661 tgtaaggata ggctttttca attttatcaa gcaatagaaa ccattaaaga tggttatgct
      721 tatgatgaca ggtaccattt ccaagaaaag gtttttgact gtccaaatgt gtggattttt
      781 acaaataaga ttccgaatgt taaactatta tcgagggacc gatggaaatt ctgggaagtc
      841 gaagacgggg aacttgtaaa aaaagagctg agtgaagaag atgagttaga tccagaagag
      901 gctaaagaga tcgttgtaag agaaaacaac gacttaaaag ttcagctcaa attattgaaa
      961 aagcgaatga aggaggcgag tttttaaaaa atcgaaaaat cgaaaaaact cgtaggtaga
     1021 gggggggcct tggcgcttcg ctgtaacact gtaaggcccc cagactctaa ttattcttga
     1081 aaaagatggc acaattggaa cattggaaca gaaatctttt tcatggattc catggatgga
     1141 ttgcaataat tttattaaat tattgaccct ttgggaattg ggcgcttcgc gcattagcct
     1201 tacggcactt cacttgcgca cgctcacgcg tgcacaagct cgattagagc aaacactctg
     1261 ggtatgaaac gaggcgttta ggtgtttgta tttatttttc tgtttagaaa aattatttag
     1321 tagttatatg ctcgaaagca agtataacat aaagaatgat gtcgaggtaa ttcaggtgaa
     1381 tcacattctt tacaggtatg ttgatggtaa ggatcttcga aaaacggaac ttctacagat
     1441 ttacgttcct ttcgattgac aggggcaaga gatctaaggg gaaagtctct tgtttcttcg
     1501 ggaagaaaag tctgtcctaa ttgttttttt ggttggacag gtgtgactgg agggtcaggt
     1561 atttggggtt cattgtcaac ttcttcatac atttcacaat cttctgaagg ttgtaaggca
     1621 tcgtcaaggt cgatgttatg cttctgaata aattcagtaa gtaatgtttc aaatacatca
     1681 gtgattcggg cgtatatttc ttgcggttca acagtcgtct caaattccag aacggaaatt
     1741 acagcatttt cgaaaaccga aaacgacgaa aaaaaggggg aaaaattgtg cgcttgtgtt
     1801 tctaagtctt gtgtcttttc cattttaatc aaaaaatctc tctcgcattt ttttcttcat
     1861 tttcattttc cgaaaattgt gaaatttcgc taaccgaaat ttgagaggac tgtgaacagc
     1921 agatgcatcg cataaaagtg ataaatcgta gaaagataga gggcatttta aaataaattt
     1981 ttatttattt ttttttacga tttatcactg atgtatttac aaagtattga tattgtatgg
     2041 agtgataatg agatcaaact gaccagaggt tggtaccacg gttgtgttaa ttgtgacagc
     2101 acaaagagac gaacctggtg cgttgacaga gacaacgaac tgttgcatgt aactgatgtt
     2161 cgatgcgagt ccattaggac atgaaacgta agtgtttaca ctagtggcat tgagtacacc
     2221 agcgttgtag attggaagga aagatccatt ggtaatcgtg ggagcagtac tagaggatgt
     2281 tgcacccgtt ccgttacagt agtaagaaca caaataggat ccttcagtaa ttgccaaagg
     2341 aaacgtaaac ttatttgtgg tgaatgtgag tgggaggtaa ctaccagttg tagcagttgg
     2401 ttgtacggtg gcactgacac ttgcaccgaa cggggatgta gtagcgggag aaccacaggc
     2461 agtagcaccg tagtagtggg cggtaagaac gttggataaa ccagcattca attggggttt
     2521 catgagtcgt acagtgtagt gaacccagag ttctccaaga tcaactgcag tggaggaagc
     2581 agttgggata ccgttggcca ttgcgcaaaa gataccgacg ttttgcattc taatgtctga
     2641 agcattagca ccaacagtga gagaaccttg accggaagtg tagtaaacac ccatagggtt
     2701 caattgagat ttacactcaa cacccataat gatgtgttcg gacggtttag tagaagcaga
     2761 gccttcacta ttttctgcaa gttgtgtatt agtataagct ccaagacaac tgtcgtactg
     2821 agtagctaac acaacttttc ccatcgagac caatgcacca gtggaactgg acgttgattc
     2881 agagaccaac ggtttgaatt cgaaggcgag cttatcgaat ttgtaatttt ggaaattgat
     2941 ggctgtagaa gaaagccaag ggaaggtgcc ggaattagcg gggttaattg cgtaattctg
     3001 agcgatgaaa gtattggttg tacctgaaga aataaggtca ccaatatact cggagtgggt
     3061 gatcattaat tcatgatcgt ttgtagtaga cattttgggt cttgaataac cagaatgtcc
     3121 tcgttggaaa ccagcagact gtttgacgag tgcaccagcg gtgtaagcgc ggcgtggagg
     3181 agcgattgag taatcaccgc gaccaagaat tcgacgagca gtaagtgtac ctgcagcgcg
     3241 tccagcaatt ccaccaaatg gaccaccgta gtaggttcca gccatttgac cagctttacc
     3301 agcagcataa actgcggtat ttcgcattgt gggaataaat tccttcttgt agaattgtcg
     3361 attctgtttt ccatgaaaag cacgactaaa gttataatga cgacgaggag gcattttcat
     3421 taaaattctt ctctcaagaa tttacagatt ttttattttt caaaaaggga aaaaatggtg
     3481 aaaagaggaa aacggcgcag aatatttatg ataaaattat gcttttggag cggattttta
     3541 gcgtaaaaat tttgaccgca aaacgggtct gggaggggga tccatgacat ttttttgttc
     3601 caatcggttc cggaacagtg ccggaacata aattc
//
LOCUS       CruV-330                3656 bp    DNA     circular     24-FEB-2020
DEFINITION  Crucivirus-330 isolate 120_1204_IR2_3297.
ACCESSION   CruV-330
KEYWORDS    .
SOURCE      Crucivirus-330
  ORGANISM  Crucivirus-330
            Unclassified.
REFERENCE   1  (bases 1 to 3656)
  AUTHORS   de la Higuera,I., Kasun,G.W., Torrance,E.L., Pratt,A.A.,
            Maluenda,A., Colombet,J., Bisseux,M., Ravet,V., Dayaram,A.,
            Stainton,D., Kraberger,S., Zawar-Reza,P., Goldstien,S., Briskie,J.,
            White,R., Taylor,H., Gomez,C., Ainley,D.G., Harding,J.S.,
            Fontenele,R.S., Schreck,J., Ribeiro,S.G., Oswald,S.A., Arnold,J.M.,
            Varsani,A., Enault,F. and Stedman,K.M.
  TITLE     Direct Submission
  JOURNAL   Submitted (24-FEB-2020) Biology, Portland State University, 1719 SW
            10th Ave, Portland, Oregon 97201, USA
COMMENT     Bankit Comment: ALT EMAIL:ide@pdx.edu
            Bankit Comment: TOTAL # OF SEQS:451
            Bankit Comment: GAP: est. length - alignment

            ##Assembly-Data-START##
            Assembly Method       :: MetaSPAdes v. 3.12; IDBA-UD v. 1.1.1;
                                     MEGAHIT v. 1.0.3
            Sequencing Technology :: 454; Illumina
            ##Assembly-Data-END##
FEATURES             Location/Qualifiers
     source          1..3656
                     /organism="Crucivirus-330"
                     /mol_type="genomic DNA"
                     /isolate="120_1204_IR2_3297"
                     /isolation_source="Water"
                     /country="Norway"
                     /collection_date=2011
     misc_feature    join(3646..3656,1..16)
                     /note="stem-loop"
     misc_feature    1..9
                     /note="nonanucleotide"
     CDS             92..496
                     /codon_start=1
                     /product="putative ORF"
                     /translation="MSPLHPNSRPDELHRERPALPPALEALAAHAIAQEQIRQLRIRL
                     IMAVLFQHNRHQDYDIRFHQASTFYGLLVPAQISLNLTFNQVLEYIPDFYLYRQAMEQ
                     IQVEFLFQPHTLTFTAFIQDQLIGNPERLPPP*"
     misc_feature    362..386
                     /note="stem-loop"
     misc_feature    complement(370..378)
                     /note="nonanucleotide"
     misc_feature    371..379
                     /note="nonanucleotide"
     CDS             532..1890
                     /codon_start=1
                     /product="Putative Replication Associated Protein"
                     /translation="MSNSRSSILCKGTSGNRMQDQLALAAQAFNCDPQDLIPNYQDYQ
                     LYLERFPQLQAESPYPMARLNFLDFLEEIRLSSTEQEQEEHLPSEQVAGGGAAAYQKP
                     LEREFPSAPETLPDARSLRQSVSSSQHVRSQAIQELTPSIQSLQSLQAQPTPPSLEDE
                     IFEAPPFEKYRIRNWFLTYWPKPNENGTYELYIPPPGAKAKYYAQQLERCPHTGRIHA
                     HMYIEFINLQSLSTIKRMFNDNTINCLFRKGTQQQAIDYCTKEDTRLEGYEPLIFGTP
                     KLSGQRNDLDGMTELAMRGLTKFELLRLYGGNAFRHLGMIDRVHAVVLGHDKHDNELM
                     FRRSRLEAINKKHPTNHSILTIHRDIEYDQRLPEAIDKLPITTILSYPNKYPYNCVAE
                     ELQQQHQRVIDDFEQDQTVDVELIIDQLQNDKELKIKSNPDNEQEDTKSNNGSESDKE
                     LE*"
     CDS             2076..3506
                     /codon_start=1
                     /product="Putative CruV Capsid Protein"
                     /translation="MSSYPQRRRPMYQAAYPQRRPAPVFARPVYQKRRTYQMAKPSIP
                     FTRRIGGNGAYTLENGPWANRGAYLGGAIGGAYAGPLGRTVGSWLGRRLAHYPAKLFG
                     SGSYLQTAPGAKTMAPQTPSFQHGSDGQVYICHREYIGDIISSATAGAFSVQSFDLNP
                     GVASTFPWLSNLSQSTFQQYKFEGLVFEFRSSSGDALNSTNTALGKVIASINYDFSDD
                     SPNSRYEMENMSWSMNCKPSQNAMIPVECKPSISGMNGGLLYVMNAATIPTGTDPKTY
                     FFGKLFIATIGLQGTNVNTGSLYVTYKVKLLKPMMTRPLSQAHTVWQYRSAVDGTNHF
                     GTSQLADVNSCDTIGVTFGTSTMTLAKKRLIVGQRFMLTYQVVGVNTASLAVPTVAIS
                     AGAQGVSYFIGSSIGSEIPSISAPRTTAFTDTQILVVITFVIDENDADIVITLSGGTY
                     PGTGCTALVFLTQVNGTLLSRTGTVV*"
ORIGIN
        1 cagtgttacc cggcacctct gaccacagac cagatgtgtt tttttcataa aaaaatacaa
       61 aaatcaattt ttaaaaactt gttttctaaa aatgtctcca ctacacccta attcacgtcc
      121 agatgaactt catcgcgaac gtcccgctct cccaccagcc ctcgaggcat tagcggctca
      181 tgctatagca caagaacaaa tacgacaact gcgtattagg cttattatgg ctgttttatt
      241 ccaacataat cgtcatcaag attatgacat tcgctttcat caagcatcca cgttttatgg
      301 actcttagta ccagctcaaa tttctcttaa tttaactttc aatcaggttc tcgaatatat
      361 ccctgatttc tatttatacc gtcaggctat ggaacaaatt caagttgaat ttttatttca
      421 accacatact ctaactttta cagcatttat ccaagatcag ctaatcggaa atcccgaacg
      481 tttacctcct ccataaaaca aaaaccgatt attttttata aatattataa aatgtccaac
      541 tcacgatcat ccatcttatg caaaggtact tcaggtaatc gcatgcaaga tcaattagct
      601 ttagcagcac aagcatttaa ttgcgatccg caagatctta ttcccaacta tcaagattat
      661 caactttatc ttgaacgctt tccacaatta caagcagaat caccatatcc tatggcaaga
      721 cttaattttc tcgattttct cgaagaaatt agactctctt caacagaaca agaacaagaa
      781 gaacacttac cttcagaaca agtcgccgga ggcggagcgg cagcatatca aaaaccactc
      841 gaaagagaat tcccctcagc accagaaact ctcccagacg caagatctct tcgtcaatca
      901 gtatcatcct cacaacatgt ccgctctcag gctattcaag aactcacacc ttcaatccaa
      961 tctttacaat ctttacaagc tcaaccaaca ccaccttctc tagaagacga aattttcgaa
     1021 gcacctcctt ttgagaaata tcgcattaga aactggtttc tcacctactg gcctaaacca
     1081 aatgagaacg gaacgtacga attatatatc cctccaccag gagcaaaagc aaaatactat
     1141 gcacaacaac tcgaacgctg tcctcataca ggcagaattc atgctcacat gtatatcgag
     1201 ttcatcaatc tccaatcact ctcaactatt aaacgtatgt ttaatgacaa tactatcaat
     1261 tgcctattcc gaaaaggaac tcaacaacaa gcaatcgact attgcaccaa agaagacact
     1321 cgtctcgaag gttacgaacc actcatcttc ggcactccta aactctcagg acaaagaaac
     1381 gacctcgacg gtatgaccga actagctatg agaggtctaa caaaattcga actattacgc
     1441 ctttatggcg gcaacgcatt ccgacacctc ggaatgatcg atagagtaca cgcagtagtc
     1501 cttggacacg ataaacatga caacgaatta atgttccgaa gatctagact ggaagcaatc
     1561 aacaaaaaac atcctactaa tcattctata ttaacaattc atcgagatat cgagtatgat
     1621 caaaggcttc cagaggcaat tgataaacta cctatcacaa caatcctctc ttacccaaac
     1681 aaatatccat acaactgcgt agcagaagaa ctacaacaac aacatcaaag agtcatagac
     1741 gactttgaac aagatcaaac ggtagatgta gagctcatta tagatcaact tcaaaacgac
     1801 aaagaactaa aaatcaaatc aaatccagac aacgaacaag aagacacaaa atctaataat
     1861 ggatcggaat cggataaaga attagaataa ttattacaat aaaaaaaaag aaaaaatata
     1921 taaaatatat ggatcaatca ggacttaaac aataatagcc ctcctgaccc ccggaggccc
     1981 gcgcgtacgc gcgttaggac gacccgcttc tataagtcta aaaatatcta aagaataaaa
     2041 acaaaaatta attattaatt tcagggtata ttaaaatgtc ctcatacccg caacgtcgta
     2101 gacctatgta tcaagcagca tatcctcaac gcagacctgc accagtattc gctagacctg
     2161 tatatcaaaa acgaagaact tatcaaatgg caaaaccatc tattcctttt actcgtcgca
     2221 tcggcggaaa tggagcttat accctagaaa acggcccttg ggcaaatcgc ggagcatatc
     2281 ttggcggagc aatcggcgga gcatacgcag gaccactcgg aagaacagtc ggttcatggc
     2341 tcggtcgaag actcgcacac taccctgcaa aactattcgg ctcaggttct tacctccaaa
     2401 cagctcccgg agctaaaact atggcacctc aaactccatc atttcaacat ggctcagacg
     2461 gacaagtcta tatatgccat agagaataca tcggagatat catctcatcg gcaacggcag
     2521 gagcattctc tgttcaatca ttcgatctca atccaggcgt agcatcaaca ttcccttggc
     2581 tctcaaatct atcacaatca acatttcaac aatataaatt cgaaggtctt gtctttgaat
     2641 tcagatcatc ctcgggagac gctcttaact ctactaatac agcccttggc aaagtaatcg
     2701 cttcaattaa ttacgatttc tcagatgatt cacccaattc tagatacgaa atggagaata
     2761 tgtcatggtc aatgaactgc aaaccttctc aaaatgcaat gatcccagtc gaatgcaaac
     2821 cctcaatctc aggcatgaat ggaggtctcc tctatgttat gaacgctgct acaatcccaa
     2881 ctggcacaga tcctaaaaca tacttcttcg gtaaactctt tattgcaacc atcggccttc
     2941 aaggcacaaa tgtaaataca ggctctcttt acgtaactta taaagtcaaa cttcttaaac
     3001 caatgatgac ccgtcctcta tctcaagcac ataccgtatg gcaataccgc agcgcagtcg
     3061 acggtacaaa tcatttcggt acatcacaac tcgcagatgt aaattcttgc gacaccatcg
     3121 gtgttacatt cggcacaagt actatgacac tggcaaaaaa acggcttata gtcgggcaaa
     3181 gatttatgtt aacttatcaa gtagtcggtg taaatacagc atctctcgcc gtacctacgg
     3241 tcgcaatctc agcaggagca caaggcgtat catacttcat cggatcatcc atcggatcag
     3301 aaatcccctc tatctcggcc cctcgaacca cagcattcac agatacccaa atcctcgttg
     3361 ttattacttt tgtaatcgac gaaaacgacg cagacattgt aatcactctc tcgggaggaa
     3421 cctatcccgg aaccggttgc acagcactag tcttcctcac tcaagtcaac ggtactcttc
     3481 tctcaagaac aggcactgta gtctaaaatc ttttcaacaa aatttaatca ttcttataaa
     3541 aattataagc ttttcattca ctttctactc acccaaattt tctcaacctc gaataaactc
     3601 ttttattcac gtcaaaaaaa taaaaattat ttttttaact ttaatgtgcc ggtctc
//
LOCUS       CruV-331                3657 bp    DNA     circular     24-FEB-2020
DEFINITION  Crucivirus-331 isolate DelMOSum2011_c10008323.
ACCESSION   CruV-331
KEYWORDS    .
SOURCE      Crucivirus-331
  ORGANISM  Crucivirus-331
            Unclassified.
REFERENCE   1  (bases 1 to 3657)
  AUTHORS   de la Higuera,I., Kasun,G.W., Torrance,E.L., Pratt,A.A.,
            Maluenda,A., Colombet,J., Bisseux,M., Ravet,V., Dayaram,A.,
            Stainton,D., Kraberger,S., Zawar-Reza,P., Goldstien,S., Briskie,J.,
            White,R., Taylor,H., Gomez,C., Ainley,D.G., Harding,J.S.,
            Fontenele,R.S., Schreck,J., Ribeiro,S.G., Oswald,S.A., Arnold,J.M.,
            Varsani,A., Enault,F. and Stedman,K.M.
  TITLE     Direct Submission
  JOURNAL   Submitted (24-FEB-2020) Biology, Portland State University, 1719 SW
            10th Ave, Portland, Oregon 97201, USA
COMMENT     Bankit Comment: ALT EMAIL:ide@pdx.edu
            Bankit Comment: TOTAL # OF SEQS:451
            Bankit Comment: GAP: est. length - alignment

            ##Assembly-Data-START##
            Assembly Method       :: MetaSPAdes v. 3.12; IDBA-UD v. 1.1.1;
                                     MEGAHIT v. 1.0.3
            Sequencing Technology :: 454; Illumina
            ##Assembly-Data-END##
FEATURES             Location/Qualifiers
     source          1..3657
                     /organism="Crucivirus-331"
                     /mol_type="genomic DNA"
                     /isolate="DelMOSum2011_c10008323"
                     /isolation_source=2011
                     /country="USA"
     CDS             1..924
                     /codon_start=1
                     /product="Putative Replication Associated Protein"
                     /translation="MSGRVRAWCFTLNNYTPEDRNELSALYDEGGVKYLVYQPERGQS
                     GTPHLQGYIVFVNARTPGGVRQLFGKRGHWEQAHGSAESNIAYCTKESTRDGSAGFEV
                     VELGARLDVRGSGQGRGHRTDVEVVADRVKSGDSLAEIAQDHPKAIVLHARGLAALQA
                     LCQQRRDAPPSVYWLWGPTGTGKSRAANEESPDAYWKDPCSSWWDGYDGKSDVIIDDY
                     RRDFCTFAALLRLFDRYPLQLQIKGGTTNMTARRIYITTPKDPQNTWEGRTAEDIGQL
                     MRRITLVKHYPGTESVPVPRLPGLFLESCHN*"
     CDS             925..2544
                     /codon_start=1
                     /product="Putative CruV Capsid Protein"
                     /translation="MSCTKLMQYNIIEEEGEISNKMSTPFAGRGRESYRRVPAQRVAR
                     KDVLSAYGPSMKDAWTVAAMGGNKNFYDNRVADGFYGRGAYKRKKYSGRGMYTGRVEG
                     RGMYSMAAPLAYAAKTGIDYAVQNPRATRKFFKNIGNKFLGRGEYEDSSSNELMSSST
                     NSPPMFQSAGDEAGALMINHREYVGDIFAPASGATSDFTVQSFPLNPGLEQSFPWLSQ
                     IAQNYEEYEMKQCVFEFVSTVQDINSSNGQVGTIISATQYNPSRPDFEDKPAMAAYAH
                     SVSGKSTDNQTHGVECDPSKLSGSEGKYIRANPVMTGEDLKTYDHGRFQLATHNIPEA
                     MASGTLGELYVSYTVVLRKPKFFTGRGLGLTRYLEVGDTDSGTAATYQLPFGAGGDEL
                     TAKQNNLSVKTTKIDNYTSITFPAYYGGRLRITGVIEGVGLGGLLWGANTTTGNVSLV
                     KDLYAAYQTIPSGDAPDSTLSVASTGRHLFIISINVEPSTNATDNVLTIRHDMTAGTI
                     NQSSLEVTEYNTFQHSGAPDLVNSAGTSIVL*"
     CDS             complement(3039..3473)
                     /codon_start=1
                     /product="putative ORF"
                     /translation="MIRTEIRIIINYFFCVGALHIVVRVILRATSATYLTTIMSRRQQ
                     VPTDIERLIQDYYGAFPQEGEWPIGNVEELTDLAFSAPNNYLPSMRPALNPKFESPGF
                     KVGPRGYYWNKTRGQWIKRHVYRPGSFVPARVYGTSKKPVNK*"
ORIGIN
        1 atgtcgggtc gcgttagagc gtggtgtttt accttaaata attatacccc cgaagatcga
       61 aatgagctat cagccttata cgatgaagga ggagttaaat atttggtata ccaacccgaa
      121 cgagggcagt caggcacccc gcacctccag gggtacattg tctttgtcaa tgcccgaacc
      181 cctggagggg tccgtcagct ctttggaaag cgaggacatt gggagcaagc ccatggatcc
      241 gcagagtcca atattgccta ttgcaccaaa gagagtacac gagacggaag tgccgggttt
      301 gaagttgtcg aactcggagc tagacttgat gtgcgaggga gtggacaagg tcgcggacat
      361 cgtacagacg ttgaagtcgt tgccgaccgt gtcaagtctg gtgattccct cgctgaaatc
      421 gcgcaggatc accccaaagc aattgtactg catgcccgtg gacttgccgc attgcaggca
      481 ctttgccaac agcgccgaga tgcgccccca agcgtatatt ggctttgggg acccactgga
      541 actggtaaga gcagagctgc gaatgaagag agcccagatg catactggaa agacccatgt
      601 tcctcctggt gggatggata cgatgggaaa tccgacgtta taattgatga ttatcgtcgt
      661 gacttttgca cattcgcagc tttgctgcga ttgtttgatc gttatccgtt acaacttcag
      721 ataaagggtg gtacaacaaa tatgactgca aggcgtattt atattactac cccgaaagac
      781 cctcagaata cctgggaggg tcgcaccgct gaagacattg gacaactgat gcgtcgcatc
      841 acacttgtaa aacattatcc aggtaccgaa tctgtaccag ttccgaggtt accgggattg
      901 tttttagaat cttgtcataa ttaattgagt tgcacaaagt taatgcaata taatataatt
      961 gaagaagaag gtgaaatcag caacaaaatg tctacgccat ttgcaggaag aggtcgtgag
     1021 agctataggc gcgttcctgc gcagcgtgtc gctcgcaagg atgtattgag cgcatacggt
     1081 ccgtctatga aggacgcttg gactgttgcc gctatgggtg gcaataagaa tttttatgac
     1141 aatcgtgttg ctgatggttt ctatggccgt ggcgcttata agcgcaagaa gtatagcggc
     1201 cgtggaatgt atactggcag ggtcgaggga cgtggcatgt acagcatggc cgctcccctc
     1261 gcatacgctg ccaagactgg gatcgattac gcagtacaga atcccagggc aaccagaaag
     1321 tttttcaaga atattggaaa taagtttctg ggtcgtggag aatacgagga tagcagctct
     1381 aatgagctga tgtctagttc tactaattct cctcctatgt ttcagagtgc tggcgacgag
     1441 gccggcgctt taatgatcaa ccatcgtgag tacgttggtg acatttttgc acccgccagt
     1501 ggtgctacct ctgattttac tgttcagtcg ttccctttga accctgggct tgagcaatct
     1561 ttcccttggt tgagtcagat tgctcagaac tacgaagagt atgaaatgaa acaatgtgtt
     1621 ttcgaatttg tcagtacggt acaggacatt aattcgagta acggtcaggt cggtactatt
     1681 atttctgcta cccagtataa ccccagccgt cctgattttg aggataagcc tgctatggct
     1741 gcctacgcgc atagcgtgtc aggcaagagc actgataacc agactcatgg agtcgagtgt
     1801 gatccttcta aactgtctgg ttccgagggt aagtacattc gtgctaatcc cgttatgaca
     1861 ggcgaagatc ttaaaactta cgatcatgga agatttcagt tagctactca taatattcct
     1921 gaggctatgg ccagtggtac attgggcgag ctatacgtta gttacaccgt tgtattgcgt
     1981 aagcccaagt tcttcaccgg tagaggtctt ggattaacga gatatctaga agttggcgat
     2041 accgatagtg gtaccgctgc tacttaccag ctgccttttg gcgctggtgg tgatgagttg
     2101 actgctaagc agaacaatct gtctgtgaag accactaaga ttgataatta caccagcatt
     2161 acgttccctg cgtattatgg tggccgtctt cgtatcacgg gagtcatcga aggagtcggc
     2221 ctcggaggat tgctttgggg tgctaatacc acaaccggta atgtgtcgct tgtaaaagac
     2281 ctttatgcag cttaccaaac gattccgtct ggtgacgcac ctgactctac attgtcagtg
     2341 gcttcgacgg gtagacacct ttttatcata tcaatcaatg tggagccgtc taccaatgct
     2401 actgataatg tcttgactat tcgtcatgat atgacagccg gtaccattaa ccaaagttct
     2461 cttgaagtaa cggagtacaa cactttccag cattcaggcg ctcctgatct ggttaatagt
     2521 gctggtacct ccattgttct ttaaaacatg gtattttata taaaattgaa atttgacggt
     2581 aacaggttta ccagttcgtg ctgtagtcat gacgttacac ttgacgttga cgacaacgtt
     2641 actaaaccta aagaccctga gaataaggac gataaaatgc tcaacgaaac ctctaacgag
     2701 caaggtgtag atatatcaga gcatggcaca attaattgta attaacaaat ttgatgccgt
     2761 ttctcccaag taaaactcat tattctagcg agagaaagga tgtcgagttt agaaatagat
     2821 ataaaacaga cgctggtaaa tttatttacc gttcttatcc ttggtctact cgtaactctg
     2881 agcggcgtga tatcgagcgc cgtcgcgagg ctaagcgaga gagagatgca gatcttagaa
     2941 tgattgagat tgccgatcga gtatttgctg ctcaggctag agaacaaaag aatatacaaa
     3001 ggtctgtaaa gagatttgct catgtattaa aaaaataatt atttatttac aggtttttta
     3061 gaagtaccgt atactcttgc aggaacaaaa gaacctggtc gatatacatg tctttttatc
     3121 cattgtcctc tagttttatt ccaataatat cctcgaggcc cgactttgaa tcccggactt
     3181 tcaaatttcg ggttcagagc tggcctcatg gatggcaagt aattatttgg tgctgagaat
     3241 gctaagtcgg tgagttcctc cacgttgccg attggccatt ctccttcttg agggaatgct
     3301 ccataataat cttggattaa tctttcgatg tccgttggca cttgttgacg acgagacata
     3361 atagttgtta ggtaagttgc gcttgtcgca cgtagtatta ccctaacaac tatgtgtagt
     3421 gcacctacac aaaaaaagta attaataatg attcgaatct cagttcgaat catttgcgaa
     3481 atttatttcg caaaagtcga gcggtcgcga ccgctcgcca aattttggat ttttcgctat
     3541 taaattcgca cggtttcgcg gatttcggag catattccga cccttcacat ttgtgaaggt
     3601 gcacgaaaat aaaatttcag attacctgac ttctgtaata tttggtcaca attatta
//
LOCUS       CruV-332                3658 bp    DNA     circular     24-FEB-2020
DEFINITION  Crucivirus-332 isolate 120_779_SvL1_3295.
ACCESSION   CruV-332
KEYWORDS    .
SOURCE      Crucivirus-332
  ORGANISM  Crucivirus-332
            Unclassified.
REFERENCE   1  (bases 1 to 3658)
  AUTHORS   de la Higuera,I., Kasun,G.W., Torrance,E.L., Pratt,A.A.,
            Maluenda,A., Colombet,J., Bisseux,M., Ravet,V., Dayaram,A.,
            Stainton,D., Kraberger,S., Zawar-Reza,P., Goldstien,S., Briskie,J.,
            White,R., Taylor,H., Gomez,C., Ainley,D.G., Harding,J.S.,
            Fontenele,R.S., Schreck,J., Ribeiro,S.G., Oswald,S.A., Arnold,J.M.,
            Varsani,A., Enault,F. and Stedman,K.M.
  TITLE     Direct Submission
  JOURNAL   Submitted (24-FEB-2020) Biology, Portland State University, 1719 SW
            10th Ave, Portland, Oregon 97201, USA
COMMENT     Bankit Comment: ALT EMAIL:ide@pdx.edu
            Bankit Comment: TOTAL # OF SEQS:451
            Bankit Comment: GAP: est. length - alignment

            ##Assembly-Data-START##
            Assembly Method       :: MetaSPAdes v. 3.12; IDBA-UD v. 1.1.1;
                                     MEGAHIT v. 1.0.3
            Sequencing Technology :: 454; Illumina
            ##Assembly-Data-END##
FEATURES             Location/Qualifiers
     source          1..3658
                     /organism="Crucivirus-332"
                     /mol_type="genomic DNA"
                     /isolate="120_779_SvL1_3295"
                     /isolation_source="Water"
                     /country="Norway"
                     /collection_date=2012
     CDS             1..1197
                     /codon_start=1
                     /product="Putative Replication Associated Protein"
                     /translation="MRFIVIKDQRGIPWCRALAEFVATNPSAMAACPEHPPEGNKRKR
                     PSRAGKRAQAYCFTYYSGGSLSPEEWLCEWHACDMPPQIKFLIGQLEKCPESGRLHVQ
                     GYVELHNGCEIPAVQQMLDLGKSHMEKRRKTSAEAAAYCEKEESWVAGSQRVCRGERS
                     QQGKRTDLSAMAAMVMAAPDVGLQQVMMADPSAIIRYGRGLQLLNGLAHPPRVRGRPW
                     ILYLVGPSRCGKSVLARTLAPDAYRCSDSPNGWFDGYAGQDTVIFSEFAGKMPVRDLL
                     QIIDSDAVRVWIKGSSAPLRATRFIFTSNYRPELLYTNPLDSDPWLKRIEEFGIIMEE
                     DAIRREVADRRVVFHDSEPPVWWPRPEGAAAAASDAPTVVLGSLEVEGSPPAQPDLAS
                     PVWDYE*"
     CDS             1550..1861
                     /codon_start=1
                     /product="putative ORF"
                     /translation="MCHMFMSLGDIKLIGRVGALVCAALSFVRGVVLSCRRRHVVPPS
                     AHADEAPPSVQRAPVCPPPSPAEASGGSGELSGQCCACFGSSRVFHAGLWRPASGCQG
                     C*"
     CDS             1987..3183
                     /codon_start=1
                     /product="Putative CruV Capsid Protein"
                     /translation="MGRSAKRLIGSTLTGSTMSMRRTRRSGRVLPGSTVRLREYDPAN
                     ADVWCAGSGDYSLGPGISGSNSQAPQFGSGTDGVRIRHREYVRDISTSVAFTNTTFEM
                     NPGLDTMFPWLSAIAQNFEQYEWKGVIIELRSISSDAIASSTNLALGSIIAAAEYNVN
                     QAAYLNKAQMENSFFAKSCKPSESLLMAIECDSKENAQKNFYVRTGAVPSGQDARLYD
                     LCNVQIATVGSQAAYVVSELWVSYDIILRKPQLDSGLGLSLDSAHFRLVAPAVTTSYL
                     GTSRTTAFDSIGMTLTSTTATFPLGTTGKFLVNYSARGASTASLVPPVVTPANATILS
                     VFNNDADQAAQTAGTPTSTIVQQDFIVNIADPNLAAVLTWSVGTLCGTPTYGDLIITQ
                     VNGNLN*"
     CDS             complement(3204..3656)
                     /codon_start=1
                     /product="putative ORF"
                     /translation="MHLRHNFTQLHQSHTLNVNGGWDRPVVQELHSCSSRPIGNGEPD
                     DERHLERARNNPVGAGLRVEVRQRDRVRVVHTRGHRHRGDAILPRHDENAPGPARNGI
                     RKDQELRTTSTSRPKIGAQRVLLNLPHRTQRVRVGTVGRVPPVHRRRG*"
ORIGIN
        1 ttgcggttta ttgttataaa ggaccaacgc ggaatcccgt ggtgtcgcgc gttggctgag
       61 tttgtggcca caaatccatc tgccatggct gcctgccctg agcacccgcc tgagggcaac
      121 aagcgcaagc ggccttcgcg tgcggggaag cgtgcgcagg cgtactgctt cacgtactac
      181 agtggcggca gcctgtctcc ggaggagtgg ctctgcgagt ggcatgcgtg cgacatgccg
      241 cctcagatca agtttctgat cggtcagctg gagaagtgcc cagagagcgg tcggctccat
      301 gtgcagggct acgtggagct gcacaacggg tgcgagatcc cggcggtgca gcagatgctc
      361 gatctgggca agtcgcatat ggagaagcgg cgcaagacgt cggcggaggc ggccgcgtac
      421 tgcgagaagg aggagagctg ggttgcgggt tcccagcgtg tgtgccgcgg tgagcgttcg
      481 cagcagggca agcgcacgga tctctccgct atggcggcca tggtgatggc cgcgcctgac
      541 gtcggtctcc agcaggtgat gatggcggat ccttccgcca ttatccgcta tggccgtggc
      601 cttcaactgt tgaatggcct ggcccatcca ccacgcgtta gaggacggcc gtggatcctg
      661 tacctggtgg gtccgtcccg gtgtggcaag tctgtgcttg cgaggacgct cgcgcctgac
      721 gcctaccgct gcagtgattc gccgaacgga tggttcgacg gctatgccgg gcaggacacg
      781 gtcatcttca gcgagtttgc cgggaagatg cctgttcgcg atctgctgca gatcatcgat
      841 agcgatgccg tgcgtgtgtg gatcaagggt tcgtctgcgc ctttgcgcgc aacgcgcttc
      901 attttcacgt cgaactatcg gccggagttg ttgtatacca acccgctcga tagcgacccg
      961 tggttgaagc gaatcgagga gtttggcatc atcatggagg aggatgctat ccgcagggag
     1021 gtcgccgatc gtcgtgtcgt gttccacgac agcgagcctc ctgtgtggtg gcctcgaccg
     1081 gagggagcag cagcggctgc gtctgacgcc ccgacggttg tcctcggttc tcttgaggtt
     1141 gagggctccc ccccggccca gcccgacctc gcctccccgg tgtgggatta tgagtaaaag
     1201 aaactcccca caccatgaaa gcggagggca gggcgggcgc gaagcgctcc gccggcccgg
     1261 agcccggccc tgcccggttt aaaggcggcc cccaaccgcc gaacattcca gcgtcccccc
     1321 gcgcgccttg aacagagttg acgtcgagca agggggcgct acatacgttt ctcggacacg
     1381 ccgacgttat aaagggagcg cagcgactct aaggaggtgt gtccgatcac caattattgc
     1441 gtttgggtca caaacgcccg ggaagtcacg gcgtagtatt acccgtgact ttccgggtca
     1501 cttttcgggg accaacgcgg atttgcgtgg gtgaaaagtg ggttgtggct tgtgccacat
     1561 gttcatgtcg cttggcgaca tcaagctcat aggccgtgtg ggagcacttg tgtgcgctgc
     1621 cttgagcttt gttcgtggag tggtgttgag ttgccgccgt cgtcatgtcg taccgccgtc
     1681 ggcgcacgcc gatgaagcgc cgccgagcgt acagcgcgcg cccgtttgcc cgccgccgag
     1741 ccccgctgaa gcgtcgggtg gctcggggga gctatctggg cagtgctgcg cgtgctttgg
     1801 gtcgtctcgg gtcttccacg ctgggctatg gcgcccggcg tctggctgcc aagggtgttg
     1861 acgcggcgat tgccatgggt ctcgacgcca gtgcggcgat gattcccggc ggttcagcgg
     1921 cgctggcggc ggcgcgcgcc ggttacaaca tggcccacaa ctacgtgaca ggtgttcgcg
     1981 cgcagcctgg ggcgaagcgc gaagcgcctg attggaagca cactgactgg ttcgacgatg
     2041 agtatgcgga ggacccgaag gtccggaagg gtgcttcccg ggtcgacggt acgcttgcgc
     2101 gagtacgacc ctgctaatgc tgatgtttgg tgcgcaggct ctggagacta ctcgctgggg
     2161 ccgggtatct ccgggagcaa cagccaagca cctcagttcg ggtctggcac cgatggagtg
     2221 cgcattcgcc accgcgagta cgtccgcgac atctccactt cggtggcctt caccaacacc
     2281 acgttcgaga tgaatcccgg tctcgacacg atgttcccct ggctgtcggc gatcgcgcag
     2341 aacttcgagc agtacgagtg gaaaggagtc atcatcgagc tccgctcgat ctcgtccgac
     2401 gccattgcca gttccacgaa tctggcgctc ggcagcatca tcgctgccgc ggagtacaac
     2461 gtaaaccagg ctgcgtacct gaacaaggcg cagatggaga atagcttctt cgcgaagagc
     2521 tgcaagccga gcgagagtct gctgatggcg atcgagtgcg acagcaagga gaacgcccag
     2581 aagaacttct acgtgcggac gggggctgtc ccctcggggc aagacgcgcg tctctacgat
     2641 ctgtgcaacg tgcagatcgc cacggtcggc tcgcaagccg cgtacgtggt gtcggagctc
     2701 tgggtcagct acgacatcat cctgcgcaag cctcagttgg actccggtct tgggctgtcg
     2761 cttgactcgg cgcacttccg tctggttgcg cctgcggtca cgacgagcta cctggggacg
     2821 agtcgtacga ccgcgttcga cagcatcggg atgacgttga ccagcactac ggctacgttc
     2881 cctctgggga cgacgggcaa gttcttggtg aactattctg cccgtggcgc gtcgactgca
     2941 tctctggtgc ctcccgtcgt gacaccggcg aatgccacca ttctgtcggt gttcaacaac
     3001 gatgcggatc aagcagcgca gacggcgggc accccgacct cgacgattgt ccaacaggac
     3061 ttcatcgtca acattgccga tccgaacctc gcggcggtgc tgacctggag cgtgggcacg
     3121 ttgtgcggca cgccgaccta cggcgatttg attatcacgc aggtcaacgg caatttaaac
     3181 taggcgccgg ggctgagacg ggctcagcct cttcgacgat gtaccggggg tactcgacca
     3241 acggttccga cccggaccct ctgggtacgg tgtggcaggt tcagcagaac acgttgggcg
     3301 cctatcttgg gcctgctggt ggaggtggtt cggagttctt gatccttccg aataccgttc
     3361 cgagcgggac ccggtgcgtt ctcatcgtgg cgtggcaaga tggcgtcacc gcggtgcctg
     3421 tggcccctag tatgaactac gcgaacgcgg tcccgttgcc gtacttcgac gcgcaaacca
     3481 gctccgactg ggttgttccg ggcgcgttca agatgtcgtt cgtcgtctgg ttcaccgtta
     3541 ccgatgggtc ggctactgca ggagtgcagt tcctggacga cgggacgatc ccagccaccg
     3601 ttgacattca atgtgtggct ttggtgtagt tgagtaaaat tgtgccttag atgcaatt
//
LOCUS       CruV-333                3665 bp    DNA     circular     24-FEB-2020
DEFINITION  Crucivirus-333 isolate 001350_virusbaton.
ACCESSION   CruV-333
KEYWORDS    .
SOURCE      Crucivirus-333
  ORGANISM  Crucivirus-333
            Unclassified.
REFERENCE   1  (bases 1 to 3665)
  AUTHORS   de la Higuera,I., Kasun,G.W., Torrance,E.L., Pratt,A.A.,
            Maluenda,A., Colombet,J., Bisseux,M., Ravet,V., Dayaram,A.,
            Stainton,D., Kraberger,S., Zawar-Reza,P., Goldstien,S., Briskie,J.,
            White,R., Taylor,H., Gomez,C., Ainley,D.G., Harding,J.S.,
            Fontenele,R.S., Schreck,J., Ribeiro,S.G., Oswald,S.A., Arnold,J.M.,
            Varsani,A., Enault,F. and Stedman,K.M.
  TITLE     Direct Submission
  JOURNAL   Submitted (24-FEB-2020) Biology, Portland State University, 1719 SW
            10th Ave, Portland, Oregon 97201, USA
COMMENT     Bankit Comment: ALT EMAIL:ide@pdx.edu
            Bankit Comment: TOTAL # OF SEQS:451
            Bankit Comment: GAP: est. length - alignment

            ##Assembly-Data-START##
            Assembly Method       :: MetaSPAdes v. 3.12; IDBA-UD v. 1.1.1;
                                     MEGAHIT v. 1.0.3
            Sequencing Technology :: 454; Illumina
            ##Assembly-Data-END##
FEATURES             Location/Qualifiers
     source          1..3665
                     /organism="Crucivirus-333"
                     /mol_type="genomic DNA"
                     /isolate="001350_virusbaton"
                     /isolation_source="Water"
                     /country="France"
                     /collection_date=2013
     misc_feature    join(3654..3665,1..19)
                     /note="stem-loop"
     misc_feature    1..9
                     /note="nonanucleotide"
     CDS             complement(215..1417)
                     /codon_start=1
                     /product="Putative Replication Associated Protein"
                     /translation="MCVKECPNWSHCCIGGTAPCRCKTTACRELLHEEKKIMCPPDLA
                     DTTTEGPNEGIIPSEAPKESAFYKLKARNWVFTINNYDDEVITLVKTWEQAKDFKCLM
                     FGKEIAPTTGTPHLQGFICFETIKSGKQIAALRANNYWAPMRMKVENNAIYCSKDNTD
                     VTVLGTIPMSQAAKGKCGVLGKEHGKKAANAWEELVKDVEEGMTKKEIERKYAKLVGQ
                     YGKGVDRLFESFKPKGDFDLLKIYGSYLPWQEVLLSEVAKGPDKRTIHWFWEHKGNAG
                     KSDMCKHLAFQHGFQPLMNGTTKDLACAWKCGSVCFDYARDTECKELNYSVLEAIKNK
                     YLFSSKYDSQTKQSESFKDVFVVCFANEPPDVRKLSADRWKIYRIEDGKAIPQILVGE
                     SVIDAIQV*"
     CDS             1554..2936
                     /codon_start=1
                     /product="Putative CruV Capsid Protein"
                     /translation="MPQVRRAAPARRVAPAPARRVAVAPARRAPARRAAPAPQRAPKQ
                     RSQAGSAFRAGGSTLGGILGSAIGGPGGGAIGSMVGRGAGAMISKLFGHGDYAVSNVD
                     QLKKNNLVLSNSAQIPQFGTGKVACKFVHREFLGDVYSSATAGAFKIDSYAINPGLDE
                     TFPWLSGVVGAKFQQYRINGMTFEFRSMSSDALNSTNTALGSVVMSTDYDSADNVFAS
                     KQEMENTEYGVSCKPSANMLHGIECARFQTPVSELYIRAFGVPSGKDIRLYDLGRFSI
                     ASVGCQGTNVNLGELWVTYDIDAFKAIEQPPQYLTPFAQYDLNAVDATHALGTSQTEV
                     KDQIGLVFDYTDGIASIIFPHDMPPGTKFWFFWELIGSSTASVPTGNVPALSGGLTNM
                     SSTHALPYPGPTATTTRKSYSTLYSYDGTGTPAAPPSLAVAGGVVPTAITVAVVTVSL
                     VSSGITADGT*"
ORIGIN
        1 tattattacc gaccccccct aatgatattt agggtgtgct aaatcttgca caggtagccc
       61 ccgatcggcg aatgagatcg catagtgagt gcaaaaaaaa gtagacctag tgttattttt
      121 gctttacctt gtgatatttc aatacagaat gttatttagg cttggagagt tgaaattcgt
      181 atatcatctt catgtgatat agaattgatg cactctagac ttgaatagca tcaatcacag
      241 actctcccac aagtatttgt ggaatagctt taccatcctc gattctgtag atcttccagc
      301 gatcagcgga tagtttacgg acatcaggtg gttcattcgc gaagcaaaca acgaagacgt
      361 ctttaaatga ttctgattgt ttggtttgac tgtcatattt ggagctaaac aaatacttgt
      421 ttttgattgc ttctaggacc gaatagttca attctttgca ttctgtgtca cgcgcataat
      481 caaagcatac agatccacat ttccaagcac atgcgagatc tttagtcgtg ccattcataa
      541 gaggttggaa accatgttga aaggccaagt gcttgcacat gtcggattta ccagcattcc
      601 ctttgtgttc ccagaaccaa tggatggtgc gtttatctgg tcccttagca acttcgctaa
      661 gtaatacttc ctgccaagga agataagagc cgtagatctt taacagatca aagtctcctt
      721 taggtttgaa tgactcgaag agacgatcaa cacctttacc atattgacca actagtttgg
      781 cgtatttacg ttcaatttct tttttggtca tgccttcttc aacgtctttg acgagttctt
      841 cccaagcgtt agcggctttc tttccatgtt ctttaccaag caccccacac ttgcccttgg
      901 cagcttgtga cattggaata gttcctagga cagtaacatc tgtgttgtct ttggaacagt
      961 agatagcatt gttctctacc ttcatacgca ttggagccca atagttattg gctctcaacg
     1021 cagctatttg cttacctgat ttgatcgttt cgaaacaaat gaacccttgt aagtgaggag
     1081 ttccagttgt cggtgcaatc tctttgccaa acatgagaca cttgaagtct ttcgcttgtt
     1141 cccaggtctt tactagggtt atcacttcat catcgtagtt gttgatagtg aagacccagt
     1201 tacgagcctt cagcttatag aatgcagact ctttcggagc ctcgctcgga ataatgcctt
     1261 cgttaggacc ctctgtggta gtgtccgcca aatctggtgg acacataatc ttcttctctt
     1321 cgtgtaagag ttcacgacat gctgttgtct tgcaacgaca aggtgctgtg ccaccgatac
     1381 agcagtgaga ccagttggga cactctttga cacacattgt aaattgtcgt aaggggtaag
     1441 tcgttagact atatacccct aatgataatt cctaagaaga aaattaatta atgcgtttag
     1501 gaagttcacg gtccatagta tatgctgtgt acctgaagtt cacttaacct aacatgcctc
     1561 aagttcgtcg tgcagctcct gctcgtcgtg ttgctcctgc tcctgctcgg cgtgtagccg
     1621 tagctcctgc tcgtcgtgct cctgctcgtc gtgctgcacc tgcccctcag cgtgcaccta
     1681 agcaacgttc acaagccgga agtgcatttc gcgctggagg ttccactctc ggtggtatcc
     1741 tcggatcagc gatcggaggt cccggaggcg gagccatcgg aagtatggtt ggtcgtggcg
     1801 ctggagccat gatcagtaaa ctctttggac acggagacta tgctgtctct aacgtggacc
     1861 aattaaagaa aaacaatctt gttctatcaa actcagcaca gatcccacag ttcgggactg
     1921 gaaaggtagc atgtaagttc gtgcaccgtg agttcctggg tgatgtatac agcagtgcaa
     1981 cagctggtgc attcaaaatt gatagttatg ctatcaatcc cggccttgat gaaacattcc
     2041 cttggttatc aggagtagtt ggagctaagt tccagcagta tcggatcaat ggcatgacct
     2101 tcgaatttcg aagtatgtcg tccgatgcat tgaactctac caatacagcg ctcggatcag
     2161 tggtcatgtc aaccgattat gattcggctg ataatgtttt tgcatccaag caagaaatgg
     2221 aaaacactga gtatggagtc tcttgtaaac ccagtgcaaa tatgttgcat gggattgaat
     2281 gtgcaaggtt ccaaactcca gtttctgagc tctatattag agcgttcggt gtcccctcag
     2341 gtaaggacat ccgcctctat gatctcggtc gattctcgat cgcatcggtt gggtgtcaag
     2401 gaaccaatgt gaacctcggt gaactgtggg tcacttatga catcgatgca ttcaaggcta
     2461 tcgaacaacc accccagtat cttactccct tcgcacagta tgatctcaac gcagttgatg
     2521 ctactcatgc tttgggtacg tcccagacag aagttaaaga tcaaatcggt cttgtatttg
     2581 attatacgga tgggatagca tcaatcattt ttccacatga tatgccaccc ggaacaaagt
     2641 tctggttctt ttgggaattg ataggtagtt ctaccgcatc tgttcccaca ggaaatgttc
     2701 ctgcactatc tggaggtctt actaatatgt caagcactca tgcgcttcca tatcctggac
     2761 caacagcaac aaccactcgt aaatcgtatt caactctcta cagttatgac ggaacgggaa
     2821 ctcccgcagc tcctcctagt ctcgccgtgg caggaggtgt tgttccaact gctatcactg
     2881 ttgcagtagt tacggtgtct ctcgtcagct caggtatcac cgctgacgga acataaatgg
     2941 ggattggccc caatgagact gagtttttca tggtctcaga ggacaaaacc atacaggatc
     3001 aggaacagaa tggatactgt cctgttcaat aacgggaaaa atcccggaat aaaaaaacaa
     3061 taaatggccc gaaagggtct cttttttgat gcattaatat agaacaaaaa ataaggagtt
     3121 aattaactct cactcatccc atattgcccc tcgcggcgaa tagccgcgct gtacaggcgt
     3181 cgaggctggc gacttagcca gccgctgacc acaatcatta tgcaggaggt ggcccccccc
     3241 cgtggggggc cacccggccc tcagccgcag catagttcat agtatatgct cgttcagatt
     3301 aacgagcaat caatcatatt catagtcaag gtcaaaaaca cttgcatgca gtatcgcacg
     3361 gtcaatagta ccgtcgtaat catccattga atggagtact tcttgattgt aagcatacat
     3421 ctcagtgtca aaataccatt gatgaaacca aaaaccagtt cttctctggt tacattctcg
     3481 cgccaatcta tctatctgat cgattatagc gaggtgtgga gcaggagcgt atgcatagct
     3541 catgatcaag ttcaagaggt tgagatcgaa gatcgaagcc atagcgtgtc agatatgaca
     3601 ggctatatac tagtgcagat agttctgcag tgaacaaaac tatcaattat cgtggggggg
     3661 tcgta
//
LOCUS       CruV-334                3666 bp    DNA     circular     24-FEB-2020
DEFINITION  Crucivirus-334 isolate 001349_virusbaton.
ACCESSION   CruV-334
KEYWORDS    .
SOURCE      Crucivirus-334
  ORGANISM  Crucivirus-334
            Unclassified.
REFERENCE   1  (bases 1 to 3666)
  AUTHORS   de la Higuera,I., Kasun,G.W., Torrance,E.L., Pratt,A.A.,
            Maluenda,A., Colombet,J., Bisseux,M., Ravet,V., Dayaram,A.,
            Stainton,D., Kraberger,S., Zawar-Reza,P., Goldstien,S., Briskie,J.,
            White,R., Taylor,H., Gomez,C., Ainley,D.G., Harding,J.S.,
            Fontenele,R.S., Schreck,J., Ribeiro,S.G., Oswald,S.A., Arnold,J.M.,
            Varsani,A., Enault,F. and Stedman,K.M.
  TITLE     Direct Submission
  JOURNAL   Submitted (24-FEB-2020) Biology, Portland State University, 1719 SW
            10th Ave, Portland, Oregon 97201, USA
COMMENT     Bankit Comment: ALT EMAIL:ide@pdx.edu
            Bankit Comment: TOTAL # OF SEQS:451
            Bankit Comment: GAP: est. length - alignment

            ##Assembly-Data-START##
            Assembly Method       :: MetaSPAdes v. 3.12; IDBA-UD v. 1.1.1;
                                     MEGAHIT v. 1.0.3
            Sequencing Technology :: 454; Illumina
            ##Assembly-Data-END##
FEATURES             Location/Qualifiers
     source          1..3666
                     /organism="Crucivirus-334"
                     /mol_type="genomic DNA"
                     /isolate="001349_virusbaton"
                     /isolation_source="Water"
                     /country="France"
                     /collection_date=2013
     CDS             complement(1..1416)
                     /codon_start=1
                     /product="Putative Replication Associated Protein"
                     /translation="MAEEKSDNVQCEIPSIVKYGRKGKKITKSDQTINAFWKKPTHPR
                     LPRVVEREKEYDNIKFSGCPIYTVGNEPNEHEETEEINEEEDEEYDFIEEPQNTDDDP
                     ASTESETSSTISRGSNDDKKTHRSYCFTINNPETKTLSFPPIVRYAVWQLEQGNEGTF
                     HLQGYMELSKPSRFLPIIESMKELNGIGHFHMKHRIATRDQAREYCMKKDETYREGPW
                     EFGKFEKGGQGTRNDLIAIYDEIKSGASRSSIIDSFPSEYIRYHNGIDKVLADRSKPR
                     DWKPVVIVIYGEPGCGKTRLAKRICGKNMYLFNSANEPWWNNYIGQQCVLFDDFDPID
                     IKYNRMKHYLDRHEMLIQTKGGMVQFSPKIIVITSNDSPDEWYDRFTALERRISIVLT
                     MDVPKGKSGTGNNEPSLKREIFTKTRKSDFKFKKEIQADNDTDWSSSLSPYTVGELTA
                     FINSELEKSINEIDTPLSLNL*"
     misc_feature    192..216
                     /note="stem-loop"
     misc_feature    201..209
                     /note="nonanucleotide"
     CDS             1501..2778
                     /codon_start=1
                     /product="Putative CruV Capsid Protein"
                     /translation="MPRVAVKSSKKSSKKPAKKSSKKKVRGGYIVKDRSYAEARSYKP
                     SLGRKMLTAGGGLAGSMFGPAGAALGAKAGDLMSTVLGMGAYSLNHNSLVGASAVPVM
                     HSARDGVRVRHREFICNISSSSTFSNTTFSINPGMNETFPWLSAMAQNFEQYKFDGLL
                     FEFVSTSADALNSTNTALGAIIMAAEYNATQPAYINKQQMENSWFAMSSRPSACQIMP
                     IECDPQENPLKIMYIRTGDIAANQDLRMYDLGNFQIASVGSQAIAVVGELWATYDVVL
                     YKPQLQSALGYDINSSRYTFSTGIAVTTGYFGTSRTENLDAIGLTVNATSITFPIGSQ
                     GYYLICYNVTGDSTALTAETRTLSNCTSATTGFAGSPSISSSGSTSTKYISIYMIYIA
                     DPTLQASITYSSGTYPANATIGNLHVTQFNGSY*"
     CDS             2896..3240
                     /codon_start=1
                     /product="putative ORF"
                     /translation="MSVSLIVSPGFGNAILIPANTFSLSGVRIFISVTYSNFSGAIVM
                     PGTVKTNFTNLLYYNNGTAVATVSGSPSGDGMSYQTWLLIADSTLDASIAFDTTGTIA
                     NYVCNITVMSFY*"
ORIGIN
        1 ttacagattt aaggaaagag gcgtatcaat ttcatttatc gacttttcca attctgagtt
       61 aataaaggct gttaattcac caactgtata tggactgagt gatgaagacc aatcagtatc
      121 attatcagct tgaatttctt tcttgaattt aaagtcggat tttctagttt tcgtaaaaat
      181 ctctctttta agggacggct cattattacc cgtcccggat tttcccttag gaacatccat
      241 agttaagact atgctaatac gtctttcaag tgctgtgaat cggtcatacc attcatctgg
      301 gctatcatta cttgtaatga ctataatttt aggggaaaat tgtaccatac cacccttggt
      361 ttgtattaac atttcatgtc tatcaagata atgtttcatt ctattgtatt ttatatcaat
      421 agggtcgaaa tcgtcgaata gtacgcattg ttgaccaata tagttattcc accatggctc
      481 attagccgag ttgaataaat acatattctt tccgcaaatt ctttttgcta atctggtttt
      541 accacaacct ggttcaccat agattacaat aactacgggt ttccaatccc ggggttttga
      601 tctatcagct aatactttat ctataccgtt atggtatcgt atgtattcag atggaaagct
      661 atctattatg ctagatcgag aggctcccga tttgatttca tcataaatag ctataaggtc
      721 atttcttgta ccttgtcctc ctttttcgaa tttaccaaat tcccatggac cttctctata
      781 ggtctcatct tttttcatac aatactcacg ggcttgatct cgtgtagcta ttctatgttt
      841 catatgaaaa tgtccaatac cgttaagttc tttcatactc tcaatgatag gtaagaatct
      901 actaggtttt gacagttcca tatatccttg tagatgaaac gttccctcat taccttgttc
      961 taattgccat acagcatatc gaacaatagg aggaaaactt aatgttttag tttctggatt
     1021 attgattgta aaacaatacg atctatgcgt ttttttatca tcattggatc ctctagaaat
     1081 ggttgatgaa gtttcacttt ccgtgcttgc cgggtcatca tctgtattct gaggctcctc
     1141 aatgaagtca tattcttcat cttcttcttc attaatttct tcggtttctt catgttcatt
     1201 tggttcatta ccaactgtat atataggaca accagaaaat ttaatgttat catattcttt
     1261 ttctctttct actactcgtg gtaatcgagg atgagtcggt ttcttccaga aagcgttaat
     1321 agtttggtca gatttggtaa tttttttacc ttttctacca tatttgacaa ttgaaggtat
     1381 ttcacattgt acattatctg atttttcttc agccatgatg ataattaatt ttttttctta
     1441 gaagtataag agttggtata tatacccaat cttctaaatc tttcacaaaa ataattaact
     1501 atgccacgtg ttgctgttaa gtcatctaaa aaatcttcta aaaaacctgc taagaaatct
     1561 tctaaaaaga aagttcgtgg aggttatatt gttaaagata gatcatatgc ggaagctaga
     1621 agctataaac catctttagg acgcaagatg ttaacagctg ggggaggttt agctgggtct
     1681 atgtttggac ctgctggcgc tgctttaggt gcgaaagctg gagatcttat gtcaaccgta
     1741 cttggaatgg gtgcatattc tcttaatcat aactcattgg taggagctag cgccgtacct
     1801 gttatgcatt ctgctcgaga tggcgtacgt gttagacacc gagaatttat atgtaatatt
     1861 tcgtctagtt ctactttctc gaatactaca ttttctatca atcctggaat gaacgaaact
     1921 tttccttggt taagtgcaat ggcgcaaaat ttcgaacagt acaagtttga tggtttacta
     1981 tttgagttcg ttagtacttc tgctgatgcg ttgaattcaa cgaataccgc tttaggtgca
     2041 attataatgg ctgccgaata taacgctact caacctgctt atattaataa acaacaaatg
     2101 gaaaactcgt ggtttgccat gtcaagtcgt ccatctgctt gtcagattat gcctattgag
     2161 tgcgaccctc aggagaaccc acttaagatt atgtatattc gtacaggcga tattgccgcg
     2221 aatcaggatc ttagaatgta cgatcttggt aatttccaaa ttgctagcgt aggatctcaa
     2281 gccatagcgg tagttggaga actttgggct acctatgatg tagtgctgta taagcctcaa
     2341 cttcaaagtg cacttggtta tgatatcaat agttcgcgat acacatttag cactggtatt
     2401 gctgtaacta ctggatattt tggtacgtct cgtactgaga atcttgatgc tattggatta
     2461 actgtaaatg ctacaagcat aactttccca attggatcac aaggatatta tttaatttgc
     2521 tataacgtta ctggtgattc tactgcatta actgcagaaa ccagaacact aagcaactgc
     2581 acatcagcaa caactggatt tgcaggtagt ccatcgatta gcagctctgg gtctactagc
     2641 acaaaataca tatcgatcta tatgatttac attgccgatc ctactctaca ggcgtcaatt
     2701 acttattcga gcggaacata tccggcaaat gctactatcg gtaatcttca tgttactcaa
     2761 tttaatggat cttattaggt attatccccg gatagtgtta ctcagactac ttctgtattt
     2821 agggcttatt catcaaatcc aacatctggg ttacctttag gttctacatt ttcgatactc
     2881 gaaaacagtg gtgatttgtc agtctcccta attgtatccc ctggatttgg taatgctatt
     2941 ctaatacctg ctaatacgtt ttcattatct ggggtacgaa tatttatttc agtaacatat
     3001 tctaatttta gcggtgctat agttatgcct ggtaccgtaa agactaattt tactaattta
     3061 ttatattata ataatggtac agcggttgcc actgtatctg gatcacctag tggagatggt
     3121 atgagttacc aaacttggtt actaatagct gattctacat tagatgctag tattgctttt
     3181 gatacgactg gtactattgc taactatgtc tgcaatataa ctgtgatgag tttctattaa
     3241 agcatattca aaaaatattg tatattagtt tttttgttct atttctgtct cccataaata
     3301 cacccatgtg ggggcacatg tatttttata attcagtatt tttcgttgat tatacttccc
     3361 ccctttagcc cgtagggtga gcgaagcgaa gggggaatat aataacggaa aaataatcaa
     3421 ttataaaaat catgtatcct ctcatatgta tgtattaata atttattttg atttatcaaa
     3481 aaactagtaa aaataccata aaagtatact ttttgatatt attacccgtc cacttcgtaa
     3541 cggtaattat ttatttaatt ttttatttgt tgaaatcttc atgcaagata gggggtaccc
     3601 cgtgctgaaa attgaaaaaa ataaataaat atcttttttt aaaaaaagga attaatcaga
     3661 tctttt
//
LOCUS       CruV-335                3667 bp    DNA     circular     24-FEB-2020
DEFINITION  Crucivirus-335 isolate 120_1192_IR2_3297.
ACCESSION   CruV-335
KEYWORDS    .
SOURCE      Crucivirus-335
  ORGANISM  Crucivirus-335
            Unclassified.
REFERENCE   1  (bases 1 to 3667)
  AUTHORS   de la Higuera,I., Kasun,G.W., Torrance,E.L., Pratt,A.A.,
            Maluenda,A., Colombet,J., Bisseux,M., Ravet,V., Dayaram,A.,
            Stainton,D., Kraberger,S., Zawar-Reza,P., Goldstien,S., Briskie,J.,
            White,R., Taylor,H., Gomez,C., Ainley,D.G., Harding,J.S.,
            Fontenele,R.S., Schreck,J., Ribeiro,S.G., Oswald,S.A., Arnold,J.M.,
            Varsani,A., Enault,F. and Stedman,K.M.
  TITLE     Direct Submission
  JOURNAL   Submitted (24-FEB-2020) Biology, Portland State University, 1719 SW
            10th Ave, Portland, Oregon 97201, USA
COMMENT     Bankit Comment: ALT EMAIL:ide@pdx.edu
            Bankit Comment: TOTAL # OF SEQS:451
            Bankit Comment: GAP: est. length - alignment

            ##Assembly-Data-START##
            Assembly Method       :: MetaSPAdes v. 3.12; IDBA-UD v. 1.1.1;
                                     MEGAHIT v. 1.0.3
            Sequencing Technology :: 454; Illumina
            ##Assembly-Data-END##
FEATURES             Location/Qualifiers
     source          1..3667
                     /organism="Crucivirus-335"
                     /mol_type="genomic DNA"
                     /isolate="120_1192_IR2_3297"
                     /isolation_source="Water"
                     /country="Norway"
                     /collection_date=2011
     CDS             join(complement(1..210),complement(3494..3667))
                     /codon_start=1
                     /product="putative ORF"
                     /translation="MSISFFVSLISFIQISWFLITISFTHPTNESSPNSAFNSSIFIS
                     LTFVEHLCADVSGIPLIHLCEISVIVLFGISLVTNLWRSILFIEEKYFLFFSFFFLLI
                     SSLDFYSFLILYAVRLRNFFLYSNT*"
     CDS             join(3627..3667,1..454)
                     /codon_start=1
                     /product="putative ORF"
                     /translation="MLRQRFVTNEIPKRTITEISHRCISGIPDTSAHRCSTKVREMKI
                     DELNALLGLDSLVGCVNEMVIKNQEICIKEINETKKDIDRTLLRISILECNNLLLKNN
                     MKEIIENNQFLRLKIQKLTDRIEDVEEDMEEYLSSVDTLLNENIEINDRLDCIETDNT
                     SEDD*"
     misc_feature    join(3653..3667,1..19)
                     /note="stem-loop"
     misc_feature    1..9
                     /note="nonanucleotide"
     CDS             complement(494..1846)
                     /codon_start=1
                     /product="Putative CruV Capsid Protein"
                     /translation="MPKYYKKYAAPVARYVTNSAYQYARKYAPPGTAKAVGAAAGAAT
                     GLPYAKYVGAQVGKQFSNITGVGAYYKSTPKRTYTKSTSIMNPRTATPRVKNADDDVY
                     ISKCEYLMDIRSGPTGTPTEFNQQVMYLNPGLDTDEGGLFTFLPALAQNFTQYSFEQC
                     VIEYRPISGNATGSNTTLGSVIMCANYIPSSDPCENKLQALNSQYAVSTAPSKGVFFP
                     IECKKSQQNLRMHQIRNGELKANQNIDLFDYAMIEVDTQGCQVPDIVLGELWITYRVR
                     LSKFKFTKTICPVVTEPLFAKFRYSGAAADWSTNPLGPSSGTVLALTDTTSNFTPVLN
                     RTARTITIPSTVVSKHFWLYCKYRSVSPQLWATFGITCSGNTSPLVSLFSVGAGQTDV
                     VTAGSGSDEDEFCVSILISVSSGGGGTCTFTTTGSLDDVTDVEIGLIEQPFDAFTAGA
                     *"
     CDS             1962..3278
                     /codon_start=1
                     /product="Putative Replication Associated Protein"
                     /translation="MNKKVKFEDKKVPKEEIDDEFLKEELPEGKGFRLTSKRVMLTYK
                     THIPKEYLTGFLQDKANKDRPFKFILLAHELGDKTNNYEHTHVLLDFGEKTDIKTSRV
                     FDFYYEVFDEEEEEDTAYMHDEKGVFIGEKNPLHPHIKPVRPGENNFKKCINYLCKED
                     AENEDFMVEINDKSGISMDEVHDAPTLYDALKNANLHLTRANDIKLVYETREVESNIA
                     LYSKDMFIVELPEIFRNIFLHGGSGIGKTQLALAQFKNPLLVRHLDNLKNFSPKFHDG
                     IVFDDINLKYHGREQCIAITDWDMPSDINVKHSVARIPANTRKIFTSNIMFDEYFPED
                     EASAIKRRFSFIIKLGDNKLFKEEEKNAIKTGTLDFDTIVTKDGKKHLNKLKAEKKVK
                     DEADAKAKADAKEKCGCDEPAKVAPPPIKEIKKNRIQIVNDDTLKG*"
ORIGIN
        1 aactattaca gaaatttcac atagatgtat cagtggaatt ccagacacat cagcacatag
       61 atgctctaca aaagtcagag aaatgaaaat agatgaatta aatgcactat taggactaga
      121 ttcattagta ggatgtgtaa atgaaatggt aattaaaaac caagaaattt gtataaaaga
      181 gattaatgaa acaaaaaaag atatcgacag aacattatta agaatatcaa ttcttgaatg
      241 taataattta ttattgaaaa ataatatgaa agaaattata gaaaataatc agtttttaag
      301 attgaagatt caaaaattaa cagatagaat cgaagatgtt gaagaagata tggaagaata
      361 tctaagtagt gttgatacat tactaaatga aaatattgaa attaatgata ggttagactg
      421 tattgaaaca gataatacat ctgaagatga ttaaaatatt tatatttgag aaatataaat
      481 gtgagaaata aatttatgca ccagcagtaa atgcatcaaa gggttgttca ataagaccaa
      541 tttcaacgtc agtgacatca tcaagagaac cagtagtagt aaaggtacac gttccaccac
      601 caccagagct aacactaata agaatagaaa cacaaaattc atcttcatca gaaccagaac
      661 cggcagtaac aacatcagtt tgaccagcac caacactaaa aagggataca agaggagaag
      721 tattaccaga acaagtaatg ccaaaagtag cccaaagttg aggagaaaca gagcgatatt
      781 tacaatataa ccaaaagtgt ttggaaacaa cagtggaagg tatagttatt gttcttgctg
      841 ttcgattaag aacaggagta aaattagaag ttgtatcagt taaggctaaa acagttccgg
      901 aagaaggacc taaaggattt gttgaccagt cagcagcagc accagaatat ctgaattttg
      961 caaataaagg ttcagtaaca acaggacaaa ttgtttttgt aaatttaaat ttagagagac
     1021 gaacacgata agtaatccat aactcaccca aaacaatatc aggaacctga cagccttgtg
     1081 tatccacttc aatcatagca taatcgaata aatcgatgtt ttggttggct tttaattcgc
     1141 cattacgaat ttgatgcatg cgcaagtttt gttgggattt tttacattca attggaaaaa
     1201 aaacaccttt agaaggagca gtagaaacag catattgact attaagagct tggagcttgt
     1261 tttcacaagg atcagatgat ggaatataat tcgcacacat aataactgaa ccaagagtag
     1321 tgttcgagcc agtagcgtta ccagagatag gacggtattc aatgacacat tgttcgaaag
     1381 aatattgagt aaaattctga gctaaagcag gaagaaaagt aaaaagacca ccttcatcgg
     1441 tgtctaagcc gggattaaga tacataacct gttggttaaa ttccgtaggg gttcctgtag
     1501 gacctgaacg aatatccatt aaatattcgc acttagaaat ataaacatca tcatctgcat
     1561 ttttaacacg tggtgtggca gtccgaggat tcatgattga tgtagactta gtataagtcc
     1621 ttttaggggt gcttttataa taagcaccta ctcctgtaat attggaaaat tgttttccaa
     1681 cctgagcacc aacatattta gcatagggta aaccggtagc agcaccggcg gcagctccaa
     1741 ctgctttggc agtaccaggg ggagcatact ttctagcata ttggtaagca gaatttgtga
     1801 cataacgagc aactggtgca gcatattttt tatagtattt gggcatttag agaaatgtta
     1861 agaaaatatt ttttaaaaat caaaaaaatt cctggttaga atttttttga ggaggaagaa
     1921 aaatgaaaaa ataaaaaaaa atttttttta gagttaataa aatgaacaag aaagtgaaat
     1981 ttgaagataa aaaagttcct aaagaagaaa tcgatgatga atttcttaaa gaagaattac
     2041 ctgagggtaa aggattcaga cttacgtcaa agagagttat gttaacatat aaaacccata
     2101 ttcctaaaga atatttaaca ggttttttac aagacaaggc taataaggat agaccattta
     2161 agtttatatt acttgcccat gaactgggag ataaaactaa caattatgaa cacacacatg
     2221 ttttacttga ttttggagag aaaacagata tcaaaacctc tagagttttt gacttttatt
     2281 atgaagtctt cgatgaagaa gaagaagaag atacagcata catgcatgat gaaaaaggtg
     2341 tgtttattgg cgaaaaaaat ccgcttcatc cacatattaa acctgtaaga ccaggcgaga
     2401 ataactttaa aaaatgcatc aattatttat gtaaggaaga tgcagagaat gaggatttta
     2461 tggttgaaat taatgacaaa tccgggataa gtatggatga agtacacgat gcgccaacat
     2521 tatatgatgc attaaaaaat gcaaacctac atttgactag agcaaatgat attaagcttg
     2581 tttatgaaac aagagaagtt gaatcgaata tagcgcttta ttctaaagat atgtttatag
     2641 ttgagttgcc agaaatattt agaaatatat tcttacatgg aggtagcgga attggtaaaa
     2701 ctcaactcgc gttggcgcag tttaaaaacc cactattagt gagacattta gataatctaa
     2761 aaaatttttc gccaaaattt cacgatggta tcgtgtttga tgatattaat ttaaaatatc
     2821 atggaagaga gcaatgtatt gctattacag attgggatat gccttcagac ataaatgtga
     2881 aacattctgt tgcacgtatt cctgcgaata caagaaagat atttacatcc aacattatgt
     2941 ttgatgaata ctttccagaa gatgaagcta gcgctataaa aagaagattt agttttataa
     3001 taaaactagg agataataaa ctcttcaagg aagaagaaaa aaatgccatt aaaactggca
     3061 cactagattt tgatactatc gtaacaaaag atggaaaaaa acaccttaat aaattaaagg
     3121 ctgagaaaaa agttaaagac gaagcagatg ctaaagcaaa agcagatgct aaagaaaaat
     3181 gtggttgcga tgaacctgct aaggttgcac cacccccaat taaggaaata aaaaagaata
     3241 gaattcagat cgtaaacgat gataccctaa agggataaac taacaaatat aatttaatat
     3301 tatatttgaa ataaagctaa agcttaaagc gtcacgtata aatatatacg agtggacgag
     3361 agagggccca taacattatt ggggccctca ttgaaccctg cggggcacca aaaaattcga
     3421 cttcgtcgaa ttttgttggg gaaggttgga taatttttgt aacatagact cctccggagt
     3481 ctggggaatg gaattatgta ttgctgtaca gaaaaaagtt tctgagccgt acagcgtaaa
     3541 gaattaagaa agaataaaaa tctaacgagg aaataaggag gaagaaaaat gaaaaaaata
     3601 aaaaatattt ttcttcgata aataaaatgc tacgccagag atttgttacc aatgaaattc
     3661 caaaaag
//
LOCUS       CruV-336                3676 bp    DNA     circular     24-FEB-2020
DEFINITION  Crucivirus-336 isolate 120_1195_IR2_3297.
ACCESSION   CruV-336
KEYWORDS    .
SOURCE      Crucivirus-336
  ORGANISM  Crucivirus-336
            Unclassified.
REFERENCE   1  (bases 1 to 3676)
  AUTHORS   de la Higuera,I., Kasun,G.W., Torrance,E.L., Pratt,A.A.,
            Maluenda,A., Colombet,J., Bisseux,M., Ravet,V., Dayaram,A.,
            Stainton,D., Kraberger,S., Zawar-Reza,P., Goldstien,S., Briskie,J.,
            White,R., Taylor,H., Gomez,C., Ainley,D.G., Harding,J.S.,
            Fontenele,R.S., Schreck,J., Ribeiro,S.G., Oswald,S.A., Arnold,J.M.,
            Varsani,A., Enault,F. and Stedman,K.M.
  TITLE     Direct Submission
  JOURNAL   Submitted (24-FEB-2020) Biology, Portland State University, 1719 SW
            10th Ave, Portland, Oregon 97201, USA
COMMENT     Bankit Comment: ALT EMAIL:ide@pdx.edu
            Bankit Comment: TOTAL # OF SEQS:451
            Bankit Comment: GAP: est. length - alignment

            ##Assembly-Data-START##
            Assembly Method       :: MetaSPAdes v. 3.12; IDBA-UD v. 1.1.1;
                                     MEGAHIT v. 1.0.3
            Sequencing Technology :: 454; Illumina
            ##Assembly-Data-END##
FEATURES             Location/Qualifiers
     source          1..3676
                     /organism="Crucivirus-336"
                     /mol_type="genomic DNA"
                     /isolate="120_1195_IR2_3297"
                     /isolation_source="Water"
                     /country="Norway"
                     /collection_date=2011
     CDS             join(complement(1..243),complement(3566..3676))
                     /codon_start=1
                     /product="putative ORF"
                     /translation="MWVSRSAVRVSVYIILYRIKGSITGIMYGPLAALAFIAPVSNPT
                     PVSCGCRGAPLGFSALQAEGSPKGPRSERTHVPPLVLPGGTYVLRPKIYFFARLLCHL
                     SVVPVAFRRLTDIKF*"
     misc_feature    join(3666..3676,1..18)
                     /note="stem-loop"
     misc_feature    1..9
                     /note="nonanucleotide"
     CDS             complement(280..819)
                     /codon_start=1
                     /product="putative ORF"
                     /translation="MDNDSNEWDQQMRQLADMHRMNAEVNSHRALLISITDDIQTAID
                     HIEETIWQLEFDKESPYDNRRDQQYYSRFINRILNALNPVVTRLTQGVQTQRILDVLA
                     PIQDIMNRLNNIIPISDHDEALHHMRDIIGDLFEEREDALASIVQLSRMMDRMVGYAE
                     AIGLEESQPFTNNKTPKIE*"
     CDS             complement(882..2474)
                     /codon_start=1
                     /product="Putative CruV Capsid Protein"
                     /translation="MAYGYRRRYGGYRRRSYGGYRRRSYGNSGGGITSAGIVRAMRTA
                     NKQTENNQYRKAYKNAMAAEYPKGSEGSQRYYGKTWDSASAIQRGARMSNRYYGDGDY
                     TDYLGYIPRAIGAAGGAMLGYGSRGTIDGAYKAAKAGWNAGANFSKGYLGWGDYAPVT
                     NQIMGGQGSSSDTISVNRDNLSGDIFFSHSEYIGNVVATGTAGGLSAFQITEYPLNCG
                     LSETFPFLAQIAQNFTLYDFEGLIFQYKPNYGESGGTSQALGKVIMSTNYDPDAETYV
                     NSVQMENADYAASCKPSLAMHHGVETHHAAQMVTNYYVRTGVSAKSKTFTDLGVFCVA
                     TESIPLPAASTTIDIGELWVTYRVKLSRAILYGSLLGLNIKQDSFIVTTNAGALMAGF
                     TAKATNTIGVNLTGLGTTTFRVDFPVNISLGAFFVHIHVLDSTTPLGLGVLNAPGTLT
                     NCQFFQPQLTLPQNAGTTTIRAPEVGVAANSKAEQAFGIVVKAPGNLVASFIVTSSAA
                     IPVGTMRMFITQVNQPMSLTIQ*"
     CDS             complement(2532..3545)
                     /codon_start=1
                     /product="Putative Replication Associated Protein"
                     /translation="MLRSFRLMTTKTKAWCFTIFDANEETEEDMAGWQYTYLVYGREV
                     CPDTNRPHLQGYVEFHNRKVLKTVKRIHKTAHWEPRRGSAHEASEYCKKDKVFVELGA
                     ISLGQGHRTDLEIIRSEIADGKSEIDIADCHFAIWVQHRRAFSEYRHLMQPPLYRPNL
                     QIWSVSGPTGTGKTSMSMAMYPDAFIVSDPTLQWFDGYNGERVVIIDEYRGGARGDIL
                     LRLTDIYKFRVPVKGGFVPWNPLLIIFTSNLYCPWGHDDISEPIARRVQHNINLTEKR
                     DFNDDAQSIRIAAKFPVCPFIRAEEVVEGTPVDIEIGTQDNTQEDLSWLLPDETPDMF
                     HFE*"
ORIGIN
        1 taatactaat gggggtacat gtgtcctttc gctccgcggg cccttcgggg agccttcggc
       61 ttggagagcg ctaaacccta acggcgctcc gcgacaccca catgacacag gggtcgggtt
      121 gctaacgggc gctataaagg ctagggccgc aagcggccca tacataattc ctgttattga
      181 acccttgatg cggtataaga taatgtatac gctaacccta accgcgctcc gcgacaccca
      241 catgacacag gggtcgggtt gctaacaagc gctcagtatt tattcaattt taggcgtctt
      301 gttattagta aatggttgac tttcttctaa acctattgct tcagcgtatc caaccattct
      361 gtccatcatt ctagataatt ggacgatgct agctagtgcg tcttctcgtt cttcaaacag
      421 atcgccgatt atgtcacgca tgtgatgaag tgcttcgtca tgatcagaaa ttggaataat
      481 attgttaagt ctattcataa tgtcttgaat tggtgccaat acgtctagta tacgctgagt
      541 ctgtactcct tgtgtcaacc gggtaacaac aggatttaac gcatttagaa tacgattaat
      601 gaacctagaa tagtattgtt gatccctcct attatcataa ggactttctt tgtcgaactc
      661 cagttgccat atagtttcct ctatatgatc tatggctgtt tgaatatcgt ccgtaataga
      721 tattaataac gcccggtgac tatttacttc agcgttcatt ctatgcatgt cggccaattg
      781 gcgcatttgt tgatcccatt cattagaatc attgtccatt ttcgcaaaat gacgtattta
      841 ttttgtcgtg cttaatgtaa tgtcgaagac acatgttacg tttattgaat tgtcaacgac
      901 ataggttggt taacttgtgt gataaacata cgcatagttc ccactggaat tgctgcacta
      961 gaagtcacaa taaatgacgc aactaaatta ccaggagcct tcaccactat accaaacgct
     1021 tgttcagctt ttgaattagc cgcaaccccc acctcgggag ctctaatagt agttgtaccc
     1081 gcattttgtg ggagggttaa ctgaggctgg aagaattgac aatttgtcaa ggttcccggt
     1141 gcatttagta cccctaaccc caacggtgta gtggaatcta atacatgaat atgaacaaag
     1201 aatgctccta gactgatatt aacagggaag tcgactctaa aggtagttgt ccctaaacct
     1261 gtaaggttca ctccgatcgt attagttgcc ttggcagtaa atccagccat caatgcccct
     1321 gcattagtag tgacaatgaa agaatcttgt ttgatattaa gaccaagtaa tgatccatac
     1381 aaaatagccc tagataattt cacacggtat gttacccata gttcaccaat gtcaattgta
     1441 gtcgatgcag ctggtaacgg aatgctctct gtagcaacac aaaacactcc taaatcggta
     1501 aacgtcttag acttggccga aacgcctgta cgaacataat aattagtaac catttgggca
     1561 gcatgatgag tttctacacc atggtgcata gccaaactcg gtttacacga agcagcataa
     1621 tctgcatttt ccatctggac ggaattaaca tatgtttctg catccgggtc gtagttagta
     1681 gacataatga ctttaccaag agcctgcgac gtaccgccgc tttctccata attaggttta
     1741 tattgaaaga taagaccttc gaagtcatat aatgtaaaat tttgggcaat ctgcgccaaa
     1801 aatggaaatg tctcactcaa accgcaattg agtggatatt cggtgatttg gaatgcggat
     1861 aatccgcccg ctgtacccgt agccactaca ttgccgatgt attcagaatg actaaaaaat
     1921 atatctccag ataaattatc acgattgacc gaaatagtat ctgaagaact accttgaccg
     1981 cccataatct gattagtgac aggagcataa tctccccagc ccaagtatcc cttggaaaaa
     2041 ttagcgcccg cgttccaacc agctttagca gctttatagg cgccatcaat agtaccacga
     2101 ctaccatatc ctagcatagc accaccagct gcaccaatag cacggggaat ataaccgaga
     2161 tagtcagtat agtctccatc accgtaataa cggttagaca tacgtgcgcc acgttgaata
     2221 gcactagcag aatcccaagt ttttccataa tatcgttgac tcccttctga cccctttggg
     2281 tattccgcgg ccatagcgtt tttatacgcc tttctgtatt gattattctc agtttgctta
     2341 tttgcagtac gcatagcgcg aacaataccg gcacttgtaa tgccgccgcc actgtttccg
     2401 taagacctgc gtctgtagcc tccatatgat ctacgtctgt acccgccata acggcgtcta
     2461 taaccgtatg ccattgaagc agctacaaat acactttata aatacgttta tacgtttaca
     2521 cttaatcgtc tttattcaaa atgaaacata tccggcgttt cgtccggaag taaccaagat
     2581 aagtcctctt gcgtattatc ttgtgtccca atttcaatat caacaggagt gccttcaaca
     2641 acttcctcag ccctaataaa cgggcacaca ggaaatttag cagctattct tatagactgg
     2701 gcatcatcat tgaaatcccg cttttcggta aggttaatgt tgtgctgtac ccgtctggcg
     2761 atgggttcgg agatgtcgtc atgcccccaa gggcagtaga ggttggacgt gaatataata
     2821 agtaaagggt tccatggaac gaagcctccc ttgacaggga ctctgaactt gtaaatatcc
     2881 gtgagtctaa gaaggatgtc gcctcttgcg ccacctctat attcatcgat gataacgact
     2941 ctttcaccgt tatagccgtc aaaccactga agtgtcggat ccgaaacgat gaaggcatcg
     3001 ggatacattg ccatggacat ggacgttttg ccggtacctg taggtccgga aacggaccag
     3061 atctgtaagt taggtctata caaaggcggt tgcattagat gacggtattc actaaaagcc
     3121 cgacgatgtt gaacccaaat agcaaagtga cagtcggcta tatcaatttc agacttaccg
     3181 tcagcaatct cagatcgaat gatttctaaa tcagtccgat gtccctggcc aagagatatt
     3241 gcaccaagtt caacaaatac cttatccttt ttacaatatt ctgaggcctc gtgagccgag
     3301 ccacgcctag gttcccaatg agccgtcttg tgaatacgtt tgacggtctt gagtacctta
     3361 cggttatgaa attcgacata cccttgtaaa tggggacggt tagtgtctgg acacacctct
     3421 ctaccataca caagataagt atactgccac ccagccatat cttcttcagt ttcctcattt
     3481 gcgtcaaaaa tagtgaaaca ccaggctttc gtcttggttg tcattagtcg aaatgaccgt
     3541 aacaaaaaat aacattacaa atatgttaaa attttatgtc agtcaaccgc ctaaaagcga
     3601 cgggcacgac ggacagatgg cataagagtc ttgcgaaaaa ataaatttta ggacgcaaga
     3661 cataagtacc cccagg
//
LOCUS       CruV-337                3682 bp    DNA     circular     24-FEB-2020
DEFINITION  Crucivirus-337 isolate 120_403_IR1_3296.
ACCESSION   CruV-337
KEYWORDS    .
SOURCE      Crucivirus-337
  ORGANISM  Crucivirus-337
            Unclassified.
REFERENCE   1  (bases 1 to 3682)
  AUTHORS   de la Higuera,I., Kasun,G.W., Torrance,E.L., Pratt,A.A.,
            Maluenda,A., Colombet,J., Bisseux,M., Ravet,V., Dayaram,A.,
            Stainton,D., Kraberger,S., Zawar-Reza,P., Goldstien,S., Briskie,J.,
            White,R., Taylor,H., Gomez,C., Ainley,D.G., Harding,J.S.,
            Fontenele,R.S., Schreck,J., Ribeiro,S.G., Oswald,S.A., Arnold,J.M.,
            Varsani,A., Enault,F. and Stedman,K.M.
  TITLE     Direct Submission
  JOURNAL   Submitted (24-FEB-2020) Biology, Portland State University, 1719 SW
            10th Ave, Portland, Oregon 97201, USA
COMMENT     Bankit Comment: ALT EMAIL:ide@pdx.edu
            Bankit Comment: TOTAL # OF SEQS:451
            Bankit Comment: GAP: est. length - alignment

            ##Assembly-Data-START##
            Assembly Method       :: MetaSPAdes v. 3.12; IDBA-UD v. 1.1.1;
                                     MEGAHIT v. 1.0.3
            Sequencing Technology :: 454; Illumina
            ##Assembly-Data-END##
FEATURES             Location/Qualifiers
     source          1..3682
                     /organism="Crucivirus-337"
                     /mol_type="genomic DNA"
                     /isolate="120_403_IR1_3296"
                     /isolation_source="Water"
                     /country="Norway"
                     /collection_date=2011
     CDS             1..1134
                     /codon_start=1
                     /product="Putative Replication Associated Protein"
                     /translation="MTSDYENEMSTGPVNVHSVRWSIPKSGVLTVSQFKDAVTPLEDM
                     FKTISDDYVFQLELTVEDGRDNWHYQGFCHTNEKVRSKQLGKRFNGPFLGIAFRTSST
                     AGKKNLKEYCIKTETKQAGPWGAKKLASGEWRQETYYGGKDIILDQQMYPWQREIFQR
                     IQGEVHSRYVDCVADFDGGKGKSSLCKLLAYKKLGIMIPYCSAQNAINAVFHNQGLKS
                     YLFNLSRSRPKNFDMAELYSSIEYIKDGHMLNTKYEVKPILMDQPHCWVFTNEPPRAD
                     CFSPDRWRFWFVVNGRLVQKTLKEVVEAHKLHRRLEVVKKREADALRLREEEEEKNLE
                     HEILYSTGSELDPTPPSSPRSELGYDDGPTIVLLDEYDEVIEE*"
     CDS             1700..3076
                     /codon_start=1
                     /product="Putative CruV Capsid Protein"
                     /translation="MYVLYVVFCSKKIIFFFIYFFFSIYKKMVLKRNYKKKPAPARIY
                     GQGAYRTTRRAPVRRAPSRMIRGKGGYWSDIGRGLTSVGGAVLGGLAGGPAGALAGLS
                     AGALAGQNIFGKGAYRVKKNTILTPSSVPAVHSDNDSVRVFHKEYICDIVSSQAFALK
                     SYSVNPGLSASFPWLSGVAGDSFSQFDIQGMVYYFKSTCGDTSSSTNSLGTVSMCAEY
                     NVNTPAFSTKQQMENSMFCASGKPTQDIMAPIECDDRLNVQDSHYIRTGPVPTGGTLS
                     QYDHCNMMIATSGQQADDVVIGELWCTYDIILRKSILNNTNLGGGSVLHYAFDSTLVP
                     TASLPFNGKVACNSTNLVYESGSKISWPAGVSGTYKVICWYKGTSAVLGFPTSVVLVN
                     ATRLDLYQFGSQDSNDTRTSDVFFYMFCFNITNPSVAGSLTVTGGTYPTSITASDLFI
                     VEVPNTIA*"
     CDS             3119..3592
                     /codon_start=1
                     /product="putative ORF"
                     /translation="MYNKMSNTQPENNTDYELRCKELEEELLQLKEHKETEEEEGLIL
                     AFEEELSKHYDLQMEVQALNKGLYALPARIYDRYNDLMSYTQVLFDYVFDSEESEEEE
                     EESKEEKRETFSDISLSKEEKLFKLFQDWKKKDPDSYPRPHYRESDDYHYSEKSS*"
ORIGIN
        1 atgacaagtg attatgaaaa cgaaatgtca accggaccgg ttaacgttca tagtgtgaga
       61 tggtctattc caaagagtgg agttttaacg gtatctcaat ttaaagatgc tgttactccc
      121 ttggaggata tgtttaaaac tatatccgac gattatgtct ttcagctgga gcttacagtt
      181 gaagatggtc gtgataattg gcattatcag gggttttgtc atacgaatga gaaggtgcga
      241 agcaagcaac ttggaaagag gtttaatggg ccttttttag gtatagcttt tcgtactagc
      301 tcaacagctg gtaagaagaa cctaaaggaa tattgtatta agacagaaac taaacaggct
      361 ggtccttggg gtgctaagaa actagcttca ggcgagtgga gacaggagac ttattatggt
      421 ggcaaggata ttattttgga ccaacagatg tatccctggc aacgagagat atttcaacgt
      481 attcaaggtg aagtacactc tcgatatgta gattgtgtgg ctgattttga tggaggtaaa
      541 ggtaagagtt cattgtgcaa actacttgct tataaaaagc ttggcattat gataccttac
      601 tgtagtgctc aaaatgctat aaatgctgtc ttccacaatc aagggttgaa gagttatctt
      661 tttaatctct ctcgttcccg tcctaagaac tttgatatgg cggagcttta ctcgagtata
      721 gagtatatta aagatggtca tatgttgaat acaaaatatg aggtgaaacc tattttgatg
      781 gaccaacctc attgttgggt atttaccaac gaaccaccca gagctgattg tttcagccct
      841 gataggtgga ggttttggtt tgtagttaat ggtcgtctcg ttcagaagac tttgaaggaa
      901 gtcgttgagg cccataaact tcatagacga ttggaggttg taaagaagag ggaagccgat
      961 gctttacgac tcagagagga ggaagaggag aaaaatcttg aacatgagat tttatactct
     1021 acagggagtg aacttgatcc aactcctcct tcttctccta ggtcagaact tggttatgac
     1081 gatggtccaa cgatagtttt gttggatgag tatgacgagg taatagagga ataattttgg
     1141 agagactttt aaatatttta gatatttaaa acttgtttat taaataaaaa gcgaagcagt
     1201 tttttttttt tgattttttt tttttctcgt tgtcagtttt ttataaaaaa gggaggttgt
     1261 gaacgaaagg gggggtccac cccgagcgac ggaggagcga gtgggcttgg cccgaagggc
     1321 tggacggtgc cttgaggaat caacctccat tataacaaac ttacataaga gctggttttt
     1381 tactctccag taaaaaagat tggtggaggt agcgtgcgat agcacgccca atagagattt
     1441 ttaaaatcgt taagagtttt aaaaaatgtt ccattttgtt ccaaatgttc ctaattgttc
     1501 caattagttc catcctactt ttgacgtagt ttaaagagga tttcggggaa aaaagggtca
     1561 aaacaggctg ttttgttcca tttttctata gaatataggg ataaacataa acgagcgtag
     1621 cgagttggca ttccagcgga gcgccgttta tcccgtgggt ttttcctacg tggtgaaaaa
     1681 atgttctatt ttgttccaga tgtatgtttt atacgtcgtt ttttgttcca agaaaataat
     1741 tttttttttt atttattttt ttttttctat atataaaaaa atggttctca aaagaaatta
     1801 taagaaaaaa cctgctccgg cacgtattta tggtcaaggt gcttatcgca ctactagacg
     1861 tgctcccgtt agacgggctc cttctcgtat gatacgaggt aagggggggt attggagtga
     1921 tattggtcgt ggactgactt ccgtaggagg tgcagtctta ggtgggttag cgggaggtcc
     1981 ggcaggagct cttgcgggtt tgtcagcggg ggctctcgct ggtcaaaata tttttggtaa
     2041 aggtgcttat cgtgttaaga agaatactat tcttacacct tcttcagttc ctgcagttca
     2101 ttcggacaac gatagtgttc gagtgtttca caaggagtat atttgtgata ttgtgtcttc
     2161 tcaagctttt gctttgaaga gttattcggt taatcctggt ttgagtgctt catttccttg
     2221 gttatctggt gtcgctggag attcattctc ccagttcgac attcaaggta tggtttatta
     2281 ttttaagtct acttgtggtg atacaagttc gtctaccaat tcgttgggga cggtttctat
     2341 gtgtgctgag tataatgtta atactccggc tttttcaact aaacagcaga tggagaatag
     2401 tatgttctgt gcttcgggta agccgacaca ggatattatg gctcctatcg agtgtgatga
     2461 tagattaaat gtccaagata gtcattatat aagaactggt cctgttccaa ctggtggaac
     2521 tttatctcag tatgaccatt gtaatatgat gatagctact tcaggtcaac aagcagatga
     2581 tgttgttata ggtgagttgt ggtgtactta tgatattata cttcgtaaga gtatattgaa
     2641 taatactaat ttaggaggtg gttcagtgct tcattatgca tttgattcca ccttagttcc
     2701 aactgcttct cttcctttta atggtaaagt agcttgtaat tctactaatt tggtttatga
     2761 gtcaggttct aaaatcagct ggcctgctgg tgttagtggt acttataagg ttatatgttg
     2821 gtataagggc acaagtgccg ttttaggatt tccaacttca gttgttttgg ttaatgctac
     2881 tcgtttggac ttgtatcagt ttggaagtca agattccaat gataccagaa cttcagatgt
     2941 atttttctat atgttctgtt ttaatattac taatccttca gtcgctggta gtcttacagt
     3001 tacaggagga acttatccta ccagtattac agcttcagat ttgtttatag ttgaagtgcc
     3061 aaatactata gcatagaaat ttatttttaa aataaataat tttatttttt attatcttat
     3121 gtataataaa atgtctaata ctcaaccaga aaataatacc gattacgaat taagatgtaa
     3181 ggaactcgaa gaggaactcc ttcagcttaa agagcataaa gaaactgaag aggaagaagg
     3241 cttaatttta gctttcgaag aagagctttc taagcattat gatttacaaa tggaggttca
     3301 ggctttgaac aagggtttgt atgctttacc cgctcgtata tacgaccgat ataatgactt
     3361 aatgtcatat acgcaggttt tgtttgacta tgtctttgat tcggaagaat cggaagaaga
     3421 ggaagaagag tcaaaagagg aaaaaaggga aactttttcc gatatttccc tttctaaaga
     3481 ggaaaaacta tttaaacttt ttcaagattg gaagaaaaag gatccggata gttatccccg
     3541 tccacattat cgtgaaagcg atgattatca ctatagtgaa aaatcatctt aaatacatat
     3601 ttaggataag atgtatattt tttttgttcc aagaaaaaaa tattttaata aaattaaaaa
     3661 caataatcta ttgtaatata aa
//
LOCUS       CruV-338                3686 bp    DNA     circular     24-FEB-2020
DEFINITION  Crucivirus-338 isolate Ga0116229_10059760.
ACCESSION   CruV-338
KEYWORDS    .
SOURCE      Crucivirus-338
  ORGANISM  Crucivirus-338
            Unclassified.
REFERENCE   1  (bases 1 to 3686)
  AUTHORS   de la Higuera,I., Kasun,G.W., Torrance,E.L., Pratt,A.A.,
            Maluenda,A., Colombet,J., Bisseux,M., Ravet,V., Dayaram,A.,
            Stainton,D., Kraberger,S., Zawar-Reza,P., Goldstien,S., Briskie,J.,
            White,R., Taylor,H., Gomez,C., Ainley,D.G., Harding,J.S.,
            Fontenele,R.S., Schreck,J., Ribeiro,S.G., Oswald,S.A., Arnold,J.M.,
            Varsani,A., Enault,F. and Stedman,K.M.
  TITLE     Direct Submission
  JOURNAL   Submitted (24-FEB-2020) Biology, Portland State University, 1719 SW
            10th Ave, Portland, Oregon 97201, USA
COMMENT     Bankit Comment: ALT EMAIL:ide@pdx.edu
            Bankit Comment: TOTAL # OF SEQS:451
            Bankit Comment: GAP: est. length - alignment

            ##Assembly-Data-START##
            Assembly Method       :: MetaSPAdes v. 3.12; IDBA-UD v. 1.1.1;
                                     MEGAHIT v. 1.0.3
            Sequencing Technology :: 454; Illumina
            ##Assembly-Data-END##
FEATURES             Location/Qualifiers
     source          1..3686
                     /organism="Crucivirus-338"
                     /mol_type="genomic DNA"
                     /isolate="Ga0116229_10059760"
                     /isolation_source="Soil"
                     /country="USA"
                     /collection_date=2010
     CDS             1..1479
                     /codon_start=1
                     /product="Putative CruV Capsid Protein"
                     /translation="MPRIINGRRIYSQAEKAAYYKKKASSYVPRISGRGDYSVFKRRI
                     ASNYRKPYRHPGVGRKIGRTAGRYIGDQIPFIGSQLGGMVGGLAGQAVHSGIKTITGY
                     GDYVVKENSVLYNRDAVPQFGMSNPRCTIIAHREFVTDINGSTLFNIDTFNINASNPA
                     IFPWLSQVARNYEQVVWQGLVFEFKTTCATAIGSTNTALGTVVMATQYDSKSAPFLNK
                     QQMENYEFAQSTVPSQSIMHAIECDPKLTYNQGMFYIPQPDDDQNDNDSRMSDIGRFS
                     IATVGMQAISTIGELWVTYKVCLLKPKLTSQVLQADAWNLDTANITATAPYGDTPQLR
                     PQSTSAAYNSNLVASVVYTDPPMSDQNFTAIGRCPWFNDTDPTACFINPSFTGFLLVV
                     YELHGGSIPKGDPLATSYGNCTIITLPTINEGPFITYQKTYSTAPVVNYDAVLYTFVV
                     RVTGGYDENGFSPYFTLTAGVPASPTQGALVIYSIPGNLYYP*"
     misc_feature    1233..1264
                     /note="stem-loop"
     misc_feature    1242..1250
                     /note="nonanucleotide"
     CDS             complement(1821..2906)
                     /codon_start=1
                     /product="Putative Replication Associated Protein"
                     /translation="MEHMEHNKSPLAMYDITAPCDRYTLDQMKEWFRLHCKRWCFQQE
                     RSDNTGYLHWQCRISLISKKRADTMRHWIANTMTGWHESPTSNPSFHSGDEFYVIKVD
                     TRINGPWSDRDDVNPNNIPKRLRNDNMTWRPWQQSVINLINEEPDDRTSDRDDVNPNN
                     IPKRLRNDNMTWRPWQQSVINLINEEPDDRTVNIIINPTGQVGKSTLALWLMARKKAQ
                     RIPQQKDARDIMRMIMCCPIRSCYFIDLPKATSHRDMHSIYAAIEEIKNGYAYDDRYK
                     FTEKLFEPPHIWVFTNVKPDEKLLSRDRWKYWSINSQLQLVEHSDTIPSPSSLEIASL
                     SFVNNQLIRLQSITSKPLTLNVIREHT*"
     CDS             3157..3639
                     /codon_start=1
                     /product="putative ORF"
                     /translation="MNIHEICEGVYAVTCDISRDASGADDAGLPDDIEESEPGICQCI
                     ITPEKLLADSKLYAEQYINRGPPYGDTLDFMKFILNVTLTGNEQYTGKYEVVPPQEYP
                     VTEKQFMFKYFQEWFCVKYNQQEDEEMCEGCGYIGGDINIDGYCYDCYCIMNESDDDY
                     *"
ORIGIN
        1 atgccaagaa taataaatgg acgaagaatt tattcacaag ctgagaaagc agcttattat
       61 aagaagaaag cttcaagtta tgtaccacga ataagtggta gaggagatta tagcgtattt
      121 aagaggagaa ttgcgtctaa ttataggaag ccgtataggc atccaggggt tggacgaaaa
      181 attgggagaa ctgcaggtag atatatagga gatcaaattc cttttatagg gtcacaactt
      241 ggtggtatgg taggtggtct agctggacaa gctgtacatt cagggatcaa aacaatcact
      301 gggtatggag actatgtagt taaggaaaat tcagttttat ataatagaga tgcagttcca
      361 caatttggaa tgtctaatcc tagatgtact attatagctc atagagagtt tgtgacggat
      421 ataaatggat cgacattatt taatattgat acatttaata ttaatgcgtc taatcctgca
      481 atatttccgt ggctatctca agtagcacga aattatgaac aagtagtttg gcaaggttta
      541 gtttttgaat ttaagactac ttgtgctaca gcaattggat cgactaatac agcattgggt
      601 acggtggtaa tggcaacgca atatgattct aaatcagcac cgtttctaaa taagcaacag
      661 atggaaaatt acgagtttgc tcaatcaact gttccatcac agagtataat gcatgcaatt
      721 gaatgtgatc caaaacttac ttataatcaa ggaatgtttt atattcctca gcctgatgat
      781 gatcaaaatg ataatgattc tcgaatgtca gatataggta ggtttagtat tgcaacagtt
      841 gggatgcaag cgatatctac gattggagaa ttatgggtta catataaagt ttgtttgctt
      901 aaacctaagc taacatctca agttctacaa gcggatgctt ggaatttaga tacagctaat
      961 attacagcaa cagcaccata tggtgatact ccacagttaa ggccacagtc tacatcagca
     1021 gcttataatt caaatcttgt agcaagtgta gtatatacag atccgccaat gtctgatcaa
     1081 aattttacgg cgataggacg atgtccttgg tttaatgata cagatccaac ggcttgtttt
     1141 attaatccct catttacagg gtttttgctc gttgtttatg agttgcatgg tggtagtata
     1201 ccaaagggtg atccattggc gacatcttat ggtaattgta ctattataac acttcctaca
     1261 attaatgaag ggccttttat tacgtatcag aagacttatt ctacagctcc tgttgtgaat
     1321 tatgatgctg tgctgtatac ttttgttgtt cgagttacgg gtggatatga tgaaaatgga
     1381 ttttcaccat attttacttt gacagctggt gttccagcta gtccaactca aggggcatta
     1441 gttatttatt ctattccagg taatttatat tatccttaat atatattatc tttaatttat
     1501 atacgtattt tgacgtatat aggcaatggc gaagccgaag gcttcgacac agtagacgat
     1561 gctttcggca tcgtcctagg cgcgaagcgc atagaagtgt ttggcgcgaa gcgcccgctc
     1621 agcaggctcg aagagactgc aaaaatatat tattgattct atgggtaggt atggcgcgaa
     1681 gcgccatacc gcagtacacg attcagtgtg ttccaggtgt tccggatttt taaaataata
     1741 atatatatat attattatta gtgttacagt gacagcgcct aaaaatacgg agaacctggg
     1801 tacctgggtg ggtggtaggg ttaagtgtgt tctctgatca cattcaatgt gagtggtttt
     1861 gaagtgattg attgaagacg tatcaactga ttgttgacga atgataatga ggcaatttca
     1921 aggcttgaag gacttggaat tgtatctgag tgttcaacta gttgaagttg actgttgatt
     1981 gaccaatatt tccatctatc tctagatagt aatttttcat ctggttttac gttagtaaat
     2041 acccagatgt gaggaggttc aaataatttt tcagtaaatt tatatctatc atcataggcg
     2101 tagccgttct taatttcttc gatggctgca tatatagagt gcatatctct atgtgaagtt
     2161 gcttttggta agtcaatgaa ataacaactt ctgattgggc agcacattat cattctcatg
     2221 atatctctag catctttttg ctgaggtatt ctttgagctt tcttgcgtgc cattagccaa
     2281 agagccagag tagatttacc aacttgacca gtggggttaa taataatatt aactgttcta
     2341 tcatctggct cttcattgat gaggttaatg actgattgtt gccaaggtct ccaagtcatg
     2401 ttatcatttc gtagacgttt agggatgtta ttagggttga catcatctct gtcggatgtt
     2461 ctatcatctg gctcttcatt gatgaggtta atgactgatt gttgccaagg tctccaagtc
     2521 atgttatcat ttcgtagacg tttagggatg ttattagggt tgacatcatc tctgtcggac
     2581 caaggtccgt tgattctggt gtcgactttg atgacgtaga attcgtcccc ggaatgaaat
     2641 gaagggttag aagtaggact ttcgtgccaa ccagtcatag tattagcgat ccaatgtctc
     2701 atagtatcag ctctcttctt actaataaga ctaattctgc attgccagtg tagataacca
     2761 gtattatcag aacgttcttg ttgaaaacac caacgtttac agtgtaatct gaaccattct
     2821 ttcatttggt ctaaagtgta tctatcacag ggtgcagtaa tgtcgtacat agctagtggt
     2881 gatttattat gttccatgtg ttccaggttt aataataaaa aaggttgctt tagactattt
     2941 atttcaattt gtaataaaaa taaaaataaa aataataaga ggtctaaggt agtatactta
     3001 ctacaaaata tttattaatg tgtgataaat aaaataaaaa taataagagg tctaaggtag
     3061 tatacttact acataatata tattaatgcg aaaaattgaa tttttaaaaa ataaataaaa
     3121 taataagtgg tataaagagg agtgtgttat ataaaaatga acatccacga aatctgtgaa
     3181 ggagtgtatg cggtgacgtg tgatatatct agggatgctt ctggcgctga tgatgctgga
     3241 ctgcctgatg atatagagga atcggaaccg ggaatatgtc agtgtataat aactccagag
     3301 aaactgcttg cagattctaa gttatatgcg gagcagtata taaatagagg accaccttat
     3361 ggtgatactt tagattttat gaaatttata ttaaatgtta cattaactgg gaatgaacaa
     3421 tatacaggga agtatgaagt agttccacct caagaatatc cagtgactga gaagcaattt
     3481 atgtttaagt attttcagga gtggttttgc gtgaagtata accaacaaga ggatgaagaa
     3541 atgtgtgaag gttgtggata tataggtgga gatataaata tagatggtta ttgttatgat
     3601 tgttattgta ttatgaatga atctgatgat gattattaat aaatgaaata agtagtctaa
     3661 agcaatcatg ttgcttgtaa aacaac
//
LOCUS       CruV-339                3691 bp    DNA     circular     24-FEB-2020
DEFINITION  Crucivirus-339 isolate GP1_93221.
ACCESSION   CruV-339
KEYWORDS    .
SOURCE      Crucivirus-339
  ORGANISM  Crucivirus-339
            Unclassified.
REFERENCE   1  (bases 1 to 3691)
  AUTHORS   de la Higuera,I., Kasun,G.W., Torrance,E.L., Pratt,A.A.,
            Maluenda,A., Colombet,J., Bisseux,M., Ravet,V., Dayaram,A.,
            Stainton,D., Kraberger,S., Zawar-Reza,P., Goldstien,S., Briskie,J.,
            White,R., Taylor,H., Gomez,C., Ainley,D.G., Harding,J.S.,
            Fontenele,R.S., Schreck,J., Ribeiro,S.G., Oswald,S.A., Arnold,J.M.,
            Varsani,A., Enault,F. and Stedman,K.M.
  TITLE     Direct Submission
  JOURNAL   Submitted (24-FEB-2020) Biology, Portland State University, 1719 SW
            10th Ave, Portland, Oregon 97201, USA
COMMENT     Bankit Comment: ALT EMAIL:ide@pdx.edu
            Bankit Comment: TOTAL # OF SEQS:451
            Bankit Comment: GAP: est. length - alignment

            ##Assembly-Data-START##
            Assembly Method       :: MetaSPAdes v. 3.12; IDBA-UD v. 1.1.1;
                                     MEGAHIT v. 1.0.3
            Sequencing Technology :: 454; Illumina
            ##Assembly-Data-END##
FEATURES             Location/Qualifiers
     source          1..3691
                     /organism="Crucivirus-339"
                     /mol_type="genomic DNA"
                     /isolate="GP1_93221"
                     /isolation_source="Water"
                     /country="New Zealand"
                     /collection_date=2014
     CDS             1..1185
                     /codon_start=1
                     /product="Putative Replication Associated Protein"
                     /translation="MSKYGKVMSRFTFTWNNYSEENVINLKKLTYENIKYLIFGKEIG
                     ECGTPHLQGYVELWTRQRETGVKKILDPILKLKSKIHIEIAGGTRADSIRYCSKGEQS
                     KTEFEEFKLLEDFGPNYGKNADVYEMKTENNSETTFADILEKAKHGAKFVDILEDHPE
                     ECIKYHHGIDRVITEQKKQIIENKLNKEMEKMIPRTWQQNLLDEMKNFIDGSLFDDRS
                     IIWYYDKDGCAGKSSLIDLAYYHYSEEVCVIENGRSADIAESYNGETIVIVDLSRTIQ
                     DHINYGAIESLKNKHSYKSKWHSGSFRHPSPMIVVMANFKPKYDAFSLDRWDIRELRG
                     FNKIIDVKNIKKTIIYNDEGPRGGNTVDINVSNAPPISQRKDNIDYDNLIDDIDYDLN
                     EI*"
     misc_feature    494..518
                     /note="stem-loop"
     misc_feature    504..512
                     /note="nonanucleotide"
     CDS             1243..1680
                     /codon_start=1
                     /product="putative ORF"
                     /translation="MSAYHKRMLKQSIKMEEIKKKELKKLNNEEKNIENETIKDFNKN
                     LNSHFIINNEMCGGNENLCEGLNIMNNNIWKHYAIYDAHIKKLNEKIECLEKQYELFN
                     NKMIVIVNVVEETKNNFNRFLDDLAEEEKAFEEFEKIENIKKK*"
     misc_feature    1586..1612
                     /note="stem-loop"
     misc_feature    complement(1595..1603)
                     /note="nonanucleotide"
     CDS             complement(1981..3354)
                     /codon_start=1
                     /product="Putative CruV Capsid Protein"
                     /translation="MVNYTAAQKRAYARRMAAQRKQIAPAIFAGSGAYYQKRYPSAFA
                     PVVARGQGAYNLGNLIKAEARRISKKKGFSRAVGGSIGAALGNRAVGANVGATIGRAL
                     GMGDYVVKENTLLQGGIPMVMSSNDRSFVVRHREFLQNIVKAAGAPTFTLLAFPLQPG
                     LSSVFPWLSQMAVGYQQYRIRGMIWEYKGTSSDSLDSTQTSLGSVLMATEYDSSAPPF
                     QNQQQMLNHEFANSCKPSDTMLHAIECKKAQSVLSELYIRSGAVPQGDDERFFDFGTF
                     QIATVGGPTAASDQVLGELWCSYEVEFLKPQLITGLGLQLKTDTWSSSDTVNTTNYFG
                     ADYASAPTAGSNLGCTFTQTKIKFPVNISEGTYQVNIYYNGDDVGPGAVAPPLITANA
                     SCSILSDLAIPERAAADTSNNLMVQDIVKILAQGAELTLSAATLPTGTKAVRVVINQF
                     NGGTPAV*"
ORIGIN
        1 atgtcaaaat acggtaaagt aatgtcaaga tttacattta cttggaataa ttattctgaa
       61 gaaaatgtca taaatctaaa aaaacttact tacgaaaata ttaaatactt aatatttggt
      121 aaagaaattg gtgaatgtgg gacacctcat ttacaaggtt atgtcgaatt atggactaga
      181 caaagagaaa caggagttaa aaaaattctt gatccaattt taaaattaaa atcaaaaatt
      241 catatcgaaa tcgctggtgg aacacgggca gacagtataa gatactgttc taaaggcgaa
      301 caatcgaaaa ctgaatttga agaatttaaa ttattagaag attttggtcc aaattatgga
      361 aaaaatgctg atgtttatga aatgaaaact gaaaataata gcgaaactac gtttgctgat
      421 attttagaaa aagctaaaca tggcgctaaa tttgttgata ttttagaaga tcatcctgaa
      481 gaatgtatca aatatcacca tggtatagat agggtgatta ctgaacaaaa gaaacaaatt
      541 attgaaaata aattaaataa agaaatggaa aaaatgatac caagaacatg gcaacaaaat
      601 ttattggatg aaatgaaaaa ttttattgat ggatcattat tcgatgatcg ttcgatcatt
      661 tggtattatg ataaagatgg ttgtgctggc aaatcatctt taatagatct tgcatactat
      721 cactatagtg aagaagtatg tgttattgaa aatgggagat ctgccgatat agctgaaagc
      781 tataatggtg aaacaattgt aatcgttgat ctatcaagaa caatacaaga tcatatcaat
      841 tacggagcaa ttgaatcatt aaaaaataaa cactcgtata aatctaaatg gcactctggt
      901 tcatttagac atccctcgcc aatgatcgtt gtcatggcta acttcaaacc aaaatacgat
      961 gcatttagtt tagacagatg ggatattaga gaattacgag gtttcaataa aataatagat
     1021 gttaaaaaca ttaaaaaaac tattatttat aatgatgagg gtcccagggg gggtaatact
     1081 gtcgacataa atgtctctaa cgcccccccc atttcacaaa gaaaagataa tatagattat
     1141 gataatttaa tcgatgatat cgattatgac ttaaacgaaa tttgaattaa ttaattaaat
     1201 ttccatttaa aaatatattt tttatataat atataaaaaa aaatgtcagc atatcataaa
     1261 cgtatgttaa aacaatccat taaaatggaa gaaataaaaa agaaagaact taaaaaatta
     1321 aataatgaag aaaaaaatat tgaaaatgaa acaatcaaag attttaacaa aaacctaaat
     1381 tcacatttta ttataaataa tgaaatgtgc ggtggtaatg aaaatttatg tgaaggttta
     1441 aatattatga ataataatat ttggaaacat tacgctatct acgatgctca cataaaaaaa
     1501 ttgaatgaaa aaattgaatg tttagaaaaa caatatgaac tttttaacaa caaaatgatt
     1561 gtaattgtta atgttgttga agaaactaaa aataatttta atagattttt agatgattta
     1621 gctgaagaag aaaaagcttt tgaagaattt gaaaaaattg aaaatattaa aaaaaaataa
     1681 attttgctaa tatataggcg caaaataaat aaattaattt tttttgcata tatgcctcgc
     1741 gggggcgcca ctccgtggag gccatttcat ggagaccctt cgccagggag gcgcagcact
     1801 cgccggcgtg ctgcactgcc tcgatatata tagcgcgcac aagtgtgtgc aggtataaaa
     1861 aaaaatggga ttttttttta accttgcaca tcccacccct tgggtgggac gctgtcggag
     1921 ttaatctata ttaatgaaaa aaacctccga caatatacta tacttatttt ttatccaaat
     1981 ttaaacagct ggtgtacctc cgttgaattg gttaattaca acgcgaacgg ctttagttcc
     2041 ggttggcaat gttgcagctg aaagtgttaa ttcagcacct tgtgccaata ttttaacaat
     2101 atcttgaacc atcaaattat ttgatgtgtc tgctgcagct cgttcaggta tagctaaatc
     2161 gctaagaatt gaacagctag catttgctgt aataagtggt ggtgctacgg ctccgggtcc
     2221 gacatcatca ccattataat aaatattaac ttgataagtt ccttcggaaa tatttactgg
     2281 aaacttaatt tttgtctgtg taaatgtaca tcctaaatta cttcctgcag ttggtgcact
     2341 tgcgtaatca gctccgaaat aatttgtagt attaacggta tctgaggatg accatgtatc
     2401 tgtcttcaat tgaagaccta aaccagtaat aagctgtggt ttcaaaaatt caacctcata
     2461 agaacaccat aattctccta atacttgatc agatgctgcg gttggacctc caactgttgc
     2521 gatttgaaaa gttccaaaat cgaaaaatcg ctcatcatct ccttgtggaa cagctccact
     2581 tctaatatat aattctgaca aaacagactg tgcttttttg cattcaatag cgtgaagcat
     2641 cgtgtcagat ggtttacaag aatttgcaaa ctcatgattt aacatttgtt gttgattctg
     2701 aaatggtggt gcacttgaat catattcagt tgccattaac acacttccga gcgaagtttg
     2761 tgtagaatcc aacgagtcgg atgaagttcc tttatactcc caaatcatac ctctgatacg
     2821 atattgttga taacctacag ccatctgaga taaccatgga aataccgatg aaagtcctgg
     2881 ttgtaatggg aatgctaaca acgtaaacgt tggagcacct gcggctttta caatattttg
     2941 taaaaattca cgatgtctta caacaaaact tctgtcattt gatgacataa ccatcggaat
     3001 acctccttga agtaaagtat tttcttttac tacataatca cccataccta gagcccttcc
     3061 aatagtagca ccgacgttcg cgccgactgc tctatttcca agtgcggcac caatggatcc
     3121 accgactgct ctagaaaaac ctttcttttt tgaaattcgc cttgcttcgg ctttaattaa
     3181 attacctaaa ttatatgcgc cttgtcctct tgcgacaact ggagcgaaag cggatggata
     3241 tcgcttttga taataagcac cactacctgc aaatatggcg ggtgcaattt gtttacgttg
     3301 agctgccatt cttctggcat atgctctttt ttgtgctgct gtataattaa ccattatttt
     3361 gaatataaat tattgtacct tttaattatt ttaaatttat taaattataa aatttattaa
     3421 aatttatttt tgatgttaat cgaattttgt ttgatggtag tttatgatat ataaatatat
     3481 ttataaatat ataaaatata gaaaaataaa tatttaaatg aaaattcgaa aattttgttc
     3541 cgagttggaa caaagaatag aagtaagtaa attaaaatta aagttgatat atcaattgtt
     3601 ctttaatttg ttccattttt ataaaaacct tttttgaatt tctatttaaa aaaataaaaa
     3661 ttatgatttt ataaatatac atatttaaaa a
//
LOCUS       CruV-340                3708 bp    DNA     circular     24-FEB-2020
DEFINITION  Crucivirus-340 isolate Sty_223.
ACCESSION   CruV-340
KEYWORDS    .
SOURCE      Crucivirus-340
  ORGANISM  Crucivirus-340
            Unclassified.
REFERENCE   1  (bases 1 to 3708)
  AUTHORS   de la Higuera,I., Kasun,G.W., Torrance,E.L., Pratt,A.A.,
            Maluenda,A., Colombet,J., Bisseux,M., Ravet,V., Dayaram,A.,
            Stainton,D., Kraberger,S., Zawar-Reza,P., Goldstien,S., Briskie,J.,
            White,R., Taylor,H., Gomez,C., Ainley,D.G., Harding,J.S.,
            Fontenele,R.S., Schreck,J., Ribeiro,S.G., Oswald,S.A., Arnold,J.M.,
            Varsani,A., Enault,F. and Stedman,K.M.
  TITLE     Direct Submission
  JOURNAL   Submitted (24-FEB-2020) Biology, Portland State University, 1719 SW
            10th Ave, Portland, Oregon 97201, USA
COMMENT     Bankit Comment: ALT EMAIL:ide@pdx.edu
            Bankit Comment: TOTAL # OF SEQS:451
            Bankit Comment: GAP: est. length - alignment

            ##Assembly-Data-START##
            Assembly Method       :: MetaSPAdes v. 3.12; IDBA-UD v. 1.1.1;
                                     MEGAHIT v. 1.0.3
            Sequencing Technology :: 454; Illumina
            ##Assembly-Data-END##
FEATURES             Location/Qualifiers
     source          1..3708
                     /organism="Crucivirus-340"
                     /mol_type="genomic DNA"
                     /isolate="Sty_223"
                     /isolation_source="Water"
                     /country="New Zealand"
                     /collection_date=2012
     misc_feature    join(3692..3708,1..12)
                     /note="stem-loop"
     CDS             856..2331
                     /codon_start=1
                     /product="Putative CruV Capsid Protein"
                     /translation="MLDDLIEEMQAATPRETTRATGNFQEPMLSAQDYVNRYRAKREA
                     YPLIKAQKQAFREQYRADLNNPQSMVYQQRLGKLVRGGGRKRYRKRYRRKGRRGGRRY
                     YGRGTYVASAGANLGGRIGGWLGATGGEMLGNWGGRMIGLGDYEVNKNVFSGRLPEVT
                     NIAGNGGTVIRYQEYLGDVTTSATAGAFKIDSYLLNAANENTFPWLSQIAANYDQFEI
                     QGILFEFRSTSGNALNSTNTALGTVMFATQYDTVDTPFASKSEMLNYEFSTSGMPSHN
                     QTHMIECDPHQTPLPLLFTEQGSTNPPNTDPRLYFLGRTQIATTGFQAASVNIGELHV
                     TYQVKLLKPKLVTTLGLTNGFYRAVSNTITPTLPLGDFANANVIFSNSDYDITATDIL
                     FPTFGTDKQYSIELVWVGTVNAAVATYPTVSYFNGASDGGGAFQVPPPTSANVIRTLM
                     YQQVNVAGGQLRPFIRLGTSGAYPTGTTSVTIVINELPVNA*"
     CDS             join(complement(2838..3411),complement(2378..2775))
                     /codon_start=1
                     /product="Putative Replication Associated Protein"
                     /translation="MESCSLVQSEAATSVGVPSTNKSNAAPVRRVQLRAKHWCITLNN
                     YNDSDCLRFNQPTQFDYAICGIERGKDGTPHLQGYVVYKQRVVLSRLQRDFPRGHFEI
                     AKGTPLQNKTYCSKEMDFTEYGDLPLTGSQATSRNWQEAKEFAIAGKLDDIDPEVYIK
                     YIKNLEHIQARHRPRLESNVSLDNEWHWGPTGTGKSRYVRTAHPDAYIKSAATEWWNG
                     YDNQDVVIIEDLDIYHVKLGYYLKIWTDHYPFPANIKGSGEIMIRPKKVIITSNYTPE
                     EIWTDSKTIDPIRRRFNFIEYKVHQILHALNGNLVSFDGDTYTVLDQ*"
     misc_feature    complement(3700..3708)
                     /note="nonanucleotide"
ORIGIN
        1 taatctaggg gcacaaccac acgcacgtgg gagaaaaaaa gaaaaaaaaa atagtcccgg
       61 atggaattga accatcgacc ttgtgttaaa ccatatgaaa cacacgcact accactatgc
      121 tatcggaact gaatgaaaat gtgtaaagcg aataggctat ggcacgtagc cttcggctac
      181 tccgcttttt agatattggt gacttcacgc tccgctggga gtctgccgtg attttgtctg
      241 ccgcgagtcc tcgccgccga acaagtcggc tcggaatatt agagtcatag taataaaaaa
      301 gatttattta agatttacag aagatctacg taaacaaggt ttctttttag gagactcgtc
      361 tgagtcctcc agatcatcgg aatagtcatc atcttgatca gcccaaagaa cttcattagc
      421 aatttcacgg agtaaggtaa acatttcagt tagagtttgt tcaatatcaa aaagtctttg
      481 ttgatacaca tgtttgtaaa catcaggatg gatttctccc atatcgtgaa acacaacttt
      541 gactggttct acttccatgt ttctaagtaa acatgtgcaa cacgtgctaa gaatattcga
      601 agaatggacc cgatagtaga tgattggaca gatgatattg caaattattg ggatagtttc
      661 caaaaacaag tattagatgt tcctgatagt ccaagagtaa ggaatcaaat tacaccttat
      721 aaaaggaatg cagaacatgc aaatatgttt actaattggt tttttccaca gcaaagaaga
      781 agaatagatt ataatgtacc tgatacacaa gaattaagtg taacaccaat tagagaagag
      841 gaaaatcctt atcaaatgct agatgattta attgaagaaa tgcaagctgc aacaccacgt
      901 gaaacaacac gtgctactgg aaatttccaa gaaccgatgc tatcggcaca agactacgtc
      961 aatcgttatc gagcgaaacg agaagcgtat cctttaatca aagcacagaa acaagcgttt
     1021 cgagaacaat acagagcaga tttaaataac ccacaatcaa tggtttatca acaacgtctt
     1081 ggaaaactag tcagaggagg aggaagaaaa cgttaccgaa aacgttatcg aagaaaagga
     1141 agacgtggtg gaagaagata ttacggacga ggaacctacg ttgcatcagc cggtgcaaac
     1201 ttaggtggtc gtattggtgg gtggctaggc gccactggag gagaaatgct tggaaactgg
     1261 ggaggacgaa tgattggatt aggagattac gaagtcaaca agaatgtatt ctctggtaga
     1321 ttacctgaag taactaacat tgctggaaat ggaggtacag taatcagata tcaagaatat
     1381 cttggtgatg taacaacatc agcaactgct ggagcattca aaatagattc atacttatta
     1441 aacgccgcaa atgaaaatac atttccatgg ttatcacaaa tagcagcaaa ttacgatcaa
     1501 tttgaaatcc aaggaatctt attcgaattt agaagtacat ctggaaatgc cttaaattct
     1561 accaacacag cattaggaac agtgatgttt gctacacaat atgatacagt agacacacct
     1621 ttcgcttcaa agagtgaaat gctaaattac gaattttcca caagcggaat gccttcacac
     1681 aatcaaacac atatgataga atgcgatcca catcaaacac cactaccgtt attattcaca
     1741 gaacaaggaa gtactaatcc accaaacact gacccaagat tatacttcct aggaagaact
     1801 caaatagcaa caactggatt tcaagcagca agtgtcaaca ttggagaatt gcacgtaact
     1861 tatcaagtaa agttattaaa accaaaatta gttacaacac taggattaac taatggtttt
     1921 tacagagctg taagtaacac aataacacca actttacctt taggagattt tgcaaatgcc
     1981 aacgtaatat tcagcaactc agattacgac ataactgcta cggatatatt attccctaca
     2041 tttggaacag acaaacaata ttcaatagaa ttagtttggg taggaacagt aaacgcagca
     2101 gtagcaactt atcctacagt atcatatttt aatggagcaa gcgatggagg aggagcattt
     2161 caagtaccgc cacctacaag tgcaaatgta ataagaacat taatgtatca acaagttaat
     2221 gtagctggag gacaattaag accgtttatc agattaggaa cttctggagc ctatccaaca
     2281 ggaacaacta gtgtaaccat tgttatcaat gagttacctg ttaatgctta attatgaaat
     2341 gttaaaaaga aataaaaaga tttttttttt aaaattatta ttgatctagt acagtataag
     2401 tatcaccatc aaaactaact aagttaccat taagagcatg taagatttga tgtactttat
     2461 attcaataaa gttaaaacga cgtcgaatcg gatcgatcgt cttactatca gtccaaattt
     2521 cttcaggagt ataattggaa gtaataatca ccttcttagg tcggatcata atttcaccag
     2581 atcctttgat attagcaggg aatggataat gatcggtcca aatcttgagg tagtaaccaa
     2641 gtttgacatg ataaatatca agatcttcaa taatgacaac atcttgattg tcataaccat
     2701 tccaccattc agtagcagca gatttgatat aggcatcggg atgagctgtc ctgacataac
     2761 gggacttacc agtacctaaa tttttagcat cattgccgtt atcaacggct atgaaaaaca
     2821 ttcttataaa aatttacctg taggacccca atgccattca ttatccaagg aaacatttga
     2881 ttcgaggcga ggacgatgac gagcttgaat atgttctaaa tttttgatat atttgatata
     2941 gacttcagga tctatatcat caagcttacc agctatagca aattccttag cttcttgcca
     3001 attacgggaa gtagcttgag aaccagtaag aggtaaatca ccatattcag taaaatccat
     3061 ttctttggag caataggtct tattttgaag aggagtacct tttgcaattt cgaaatgacc
     3121 tctgggaaag tcacgttgaa gtctagaaag tacaacacgt tgtttgtaaa caacgtaacc
     3181 ttgaagatgg ggagtaccgt cttttcccct ctcaatacca caaatagcat aatcgaactg
     3241 ggttggttgg ttaaagcgaa gacagtccga atcattgtag ttgttgagag taatacacca
     3301 atgcttggct ctaagttgaa cacgacggac aggagcagca ttagatttgt tggtcgaggg
     3361 caccccaaca gaagtggctg cttctgattg tacgaggcta caagactcca tctaaggcaa
     3421 taagacagta aaaactgaat atttgaccgg gggggggtcg gccttttaag gaaatttgaa
     3481 aaaaaaattt tgtggtttta tatatttaaa tatttcaatt tgaatttgaa attgaaattg
     3541 aaaaacgtgc gcacatgcgt tggttgaact tcaccctgga gggtcgaaaa tatgtttttc
     3601 agactggggt catatatgaa aaatatgtct agagggtcga caggaaaatc gcacgtgggg
     3661 atcgggctag ggagatgagg acagaggatg cgcccctagg taatactg
//
LOCUS       CruV-341                3708 bp    DNA     circular     24-FEB-2020
DEFINITION  Crucivirus-341 isolate 120_1171_IR2_3297.
ACCESSION   CruV-341
KEYWORDS    .
SOURCE      Crucivirus-341
  ORGANISM  Crucivirus-341
            Unclassified.
REFERENCE   1  (bases 1 to 3708)
  AUTHORS   de la Higuera,I., Kasun,G.W., Torrance,E.L., Pratt,A.A.,
            Maluenda,A., Colombet,J., Bisseux,M., Ravet,V., Dayaram,A.,
            Stainton,D., Kraberger,S., Zawar-Reza,P., Goldstien,S., Briskie,J.,
            White,R., Taylor,H., Gomez,C., Ainley,D.G., Harding,J.S.,
            Fontenele,R.S., Schreck,J., Ribeiro,S.G., Oswald,S.A., Arnold,J.M.,
            Varsani,A., Enault,F. and Stedman,K.M.
  TITLE     Direct Submission
  JOURNAL   Submitted (24-FEB-2020) Biology, Portland State University, 1719 SW
            10th Ave, Portland, Oregon 97201, USA
COMMENT     Bankit Comment: ALT EMAIL:ide@pdx.edu
            Bankit Comment: TOTAL # OF SEQS:451
            Bankit Comment: GAP: est. length - alignment

            ##Assembly-Data-START##
            Assembly Method       :: MetaSPAdes v. 3.12; IDBA-UD v. 1.1.1;
                                     MEGAHIT v. 1.0.3
            Sequencing Technology :: 454; Illumina
            ##Assembly-Data-END##
FEATURES             Location/Qualifiers
     source          1..3708
                     /organism="Crucivirus-341"
                     /mol_type="genomic DNA"
                     /isolate="120_1171_IR2_3297"
                     /isolation_source="Water"
                     /country="Norway"
                     /collection_date=2011
     misc_feature    join(3695..3708,1..20)
                     /note="stem-loop"
     misc_feature    1..9
                     /note="nonanucleotide"
     CDS             130..1518
                     /codon_start=1
                     /product="Putative CruV Capsid Protein"
                     /translation="MAYRRRVYRKRPVRRYKRKVYGRGAYGDWPEIDLSGAYKSKGNS
                     YISKMPVSTGGKGRTGLGNTMQAVGTVLGSLLGNYGFPGIGGALGGVGGSIAGSVLSK
                     ITGQGAYKRPVKANSLYLPTGSGVLHMAKAGGSENGNVRIRHREYVQDIISASVAGAF
                     KSEAIRIYPSNSAIFKWLGPVANQFDQYQIHGISFEYKTTSGDVLTSTNSAMGTVILA
                     PQYNTSDPLFTNKRDMVNYESAISSIPSQSMILFMECERSQTPFKILYTYRPTIHTQD
                     LRLNVFANIAMGSSGFQGTSINAGELWVSYDIELMKPRMGSAAALLGDHYEFTTGVST
                     SAYFGTSNLLLKQTSSNLGTTLTGTTIVFDGFFSGAVVVHYTMFGDSTAWVQPTTTGT
                     LGATSLNILQQDVNNTLAVNAVSTVGQMTEYFNIIPANDGTFPTITFSAGTVILNSQK
                     GDLIISTISLFN*"
     CDS             complement(2459..3457)
                     /codon_start=1
                     /product="Putative Replication Associated Protein"
                     /translation="MTPEELEERDRLYNGDLGKKRFRNWCFTEFDNFDELYYKLVEQQ
                     MNGSVIYTVFQLEKCPETGRKHMQGYITLKNPCGMNWVKKVLTATAHLIVANGTALEN
                     QKYCTKELSRVKGPWEFGIMPVGQGKRTDLEDLKKLIDESGITDPALMWELDFKNMLK
                     YKKSIEAYILSKSKMRTEPIYVVALIGKSGSGKTKYVYDKHKYEDVYVKDTTNKWWDG
                     YNGQPVVLLDDFKGGMDVTTMKQLLDRYPMNIEIKGGNTKISNATRWVYITSNHNPEF
                     WWNDGSVNEKDMEAVERRIDETIEFTHILTNGRKINRKFRINDKGEKEEIITAVAE*"
ORIGIN
        1 tagtattacc ctcctcttcc tctgtgcaaa tttttcttat ccttttaact tttttaattt
       61 actttttttc tattattgaa ttattttttt ttatcccatt ttttaaaatt tgctatttat
      121 ttacaaaaaa tggcctatag acgtagagtt tatcgtaaaa gaccagttcg cagatacaaa
      181 cgcaaggttt atggtcgtgg tgcttatggt gattggcctg aaattgattt gtcaggtgct
      241 tacaaatcca aaggaaattc ctatatttct aaaatgcctg tatcgacagg cggaaaaggc
      301 cgtacaggac ttggtaacac aatgcaagca gttggtactg ttcttggttc attgttaggt
      361 aactacggat ttccaggcat tggtggcgca cttggtggtg taggtggatc cattgctggt
      421 tctgttttat ccaaaatcac cggacaagga gcttataagc gtcctgttaa ggccaattct
      481 ttatatcttc caacaggttc aggagtcctt catatggcga aagctggtgg ttcagagaac
      541 ggtaacgttc gaattagaca tcgtgagtat gttcaagata tcattagcgc atctgttgct
      601 ggagctttta aatctgaagc aataagaatt tatccatcta atagtgcaat ctttaagtgg
      661 ttaggtccag tagccaatca atttgatcaa taccaaattc acggtatttc atttgaatat
      721 aagactacat ccggagatgt tcttacttct actaattcag caatgggtac agttatattg
      781 gctcctcaat ataatacttc agaccctctt tttaccaata aaagagatat ggttaattat
      841 gagtcagcta tttcatccat tccttctcaa tctatgattt tgtttatgga atgtgaaaga
      901 tcacaaacac ctttcaaaat actctatact tatagaccaa ctattcatac ccaagattta
      961 cgtcttaatg tatttgctaa catagcaatg ggttcatccg gatttcaagg tacctctatc
     1021 aatgctggtg aactttgggt atcttatgat attgaattaa tgaagccacg tatgggttca
     1081 gctgctgcac ttcttggtga tcattatgaa ttcaccactg gagtttcaac ttcagcatat
     1141 tttggaacgt caaacttgtt attaaaacaa acatcttcaa atcttggaac tactttaaca
     1201 ggtacaacca tagtatttga tggattcttt tctggtgctg ttgttgtcca ttatactatg
     1261 tttggagatt ctactgcttg ggttcaacca acaactacag gaacacttgg agcaacatct
     1321 cttaatattc ttcaacaaga tgttaataac actcttgcag ttaatgcagt ttcaacagtt
     1381 ggccaaatga ctgaatattt caacatcatt ccagctaatg atggtacttt cccaacaatt
     1441 accttttcag ctggtacagt aattttaaat tctcaaaagg gtgatttaat tatcagtacc
     1501 atttccttat ttaattaaaa ggatggtgtg tgaataaaaa acttgtttaa ggtacaagaa
     1561 aaaaagtaaa aacctattaa aactcaattg agtttaatca ataattaatt ataaattaat
     1621 tattaataat tttatttaat tttttttaat taatttatat ccatttaatt ctatataatc
     1681 atttatagaa tctcttaatt cacattcaag tgaatctcta ttaattggac ttaatttttt
     1741 atttaattca tctaatatac atacaatttt ttcttttaat tgtgtttttt ctgcttgatt
     1801 attcataaac cgattgcttc gcttgattga ggttttgaat gtcaaataaa tatttaaaat
     1861 tcgattttaa atatcgaaac ttcggcgtcg ccttcattca taggcccttt tgggactcat
     1921 aatagcgaca caaatccctc ccattccgaa gcgacaaaac aaggcccaaa agggcctatg
     1981 aatgaaggcg acgccgaatt ccgatattta aaatcgaatt ttaaatattt atttaattat
     2041 tcaaaacctc aatcaagcga agcaatcggt ttatgaataa tcaatcagaa aactcccaat
     2101 taaaagaaaa aattccccaa accccactca cctcctcaac cctatcatcc tctgatgacc
     2161 ctattgctca aatcctattg gttctccagc aatttgaaaa ggaaatcaaa gatttaagag
     2221 aagtcgttga tggaattgaa gaacaccttc ttgaagacga caaagaacca ctcacccctc
     2281 aaactcaaca agtacaacaa cagcaatatc ctcctaattt tcaaaaacct ttttatcagt
     2341 atcctcaaaa taattaccgt aagtattaat ttatttatga gttatatatt tatgttaatt
     2401 tacatattag tccttaaata ttagttatat tatgatcata atataactaa cttgttgttt
     2461 attcagctac tgctgttata atttcttctt tttctccttt gtcgttaatt ctaaattttc
     2521 tattaatttt tcttccgttg gtgaggatat gggtgaattc aatagtttca tcgattctcc
     2581 gctccacagc ttccatatct ttctcattaa cagaaccatc gttccaccaa aattcagggt
     2641 tatgattaga tgtaatgtat acccacctgg tagcatttga aattttggta tttcctcctt
     2701 ttatttcgat attcatcgga taacgatcaa gtaattgttt catagttgtg acgtccatac
     2761 ctcctttaaa gtcatctagt aaaacaacag gttgcccatt ataaccatcc caccatttat
     2821 ttgtggtatc cttgacatat acatcttcgt atttatgttt gtcatataca tattttgttt
     2881 taccactacc gcttttacct attaaggcaa ctacataaat tggttctgtt ctcattttag
     2941 actttgataa aatatatgct tcaatagatt ttttatactt caacatattt ttaaagtcta
     3001 attcccacat taaagctggg tctgttatgc cactttcatc aatcaacttt tttaagtctt
     3061 caaggtctgt tcttttacct tgaccaactg gcataatacc gaattcccaa ggtcctttga
     3121 ctctgctcaa ttccttagta caatattttt gattttctag agcagtacca ttggcaacta
     3181 tcaaatgagc tgttgctgtt aaaacttttt taacccaatt cattccacaa ggatttttca
     3241 atgttatata tccttgcata tgttttctac cagtttctgg acatttctcc aattgaaaaa
     3301 cagtataaat gacgcttccg ttcatttgtt gttcgactag tttataataa agttcgtcga
     3361 aattgtcaaa ttcagtaaaa caccagtttc tgaacctttt tttacctaga tcaccgttat
     3421 aaagtctatc tctttcttct aattcttcgg gtgtcatagg tataattaat tcttttaatt
     3481 tttttaaata tagttcgttt atctcttccc cagaatcaag tttatcttct tttttttctg
     3541 gcggatttat agttaaaatt ttaagaactt tttcattttt ttgagctctc agtttactca
     3601 ttttcaattg atttttacaa ttgcccaaaa ctaaaaaaaa ttcaattttt acatcagtgc
     3661 aaaaacagta aagaatatta gaaaataata ttcgggaaga ggaggaac
//
LOCUS       CruV-342                3709 bp    DNA     circular     24-FEB-2020
DEFINITION  Crucivirus-342 isolate 120_1175_IR2_3297.
ACCESSION   CruV-342
KEYWORDS    .
SOURCE      Crucivirus-342
  ORGANISM  Crucivirus-342
            Unclassified.
REFERENCE   1  (bases 1 to 3709)
  AUTHORS   de la Higuera,I., Kasun,G.W., Torrance,E.L., Pratt,A.A.,
            Maluenda,A., Colombet,J., Bisseux,M., Ravet,V., Dayaram,A.,
            Stainton,D., Kraberger,S., Zawar-Reza,P., Goldstien,S., Briskie,J.,
            White,R., Taylor,H., Gomez,C., Ainley,D.G., Harding,J.S.,
            Fontenele,R.S., Schreck,J., Ribeiro,S.G., Oswald,S.A., Arnold,J.M.,
            Varsani,A., Enault,F. and Stedman,K.M.
  TITLE     Direct Submission
  JOURNAL   Submitted (24-FEB-2020) Biology, Portland State University, 1719 SW
            10th Ave, Portland, Oregon 97201, USA
COMMENT     Bankit Comment: ALT EMAIL:ide@pdx.edu
            Bankit Comment: TOTAL # OF SEQS:451
            Bankit Comment: GAP: est. length - alignment

            ##Assembly-Data-START##
            Assembly Method       :: MetaSPAdes v. 3.12; IDBA-UD v. 1.1.1;
                                     MEGAHIT v. 1.0.3
            Sequencing Technology :: 454; Illumina
            ##Assembly-Data-END##
FEATURES             Location/Qualifiers
     source          1..3709
                     /organism="Crucivirus-342"
                     /mol_type="genomic DNA"
                     /isolate="120_1175_IR2_3297"
                     /isolation_source="Water"
                     /country="Norway"
                     /collection_date=2011
     CDS             1..1275
                     /codon_start=1
                     /product="Putative Replication Associated Protein"
                     /translation="MPMENKEERHVTFCIDNNEYFALEESMKELTEITLPNVGSPKFR
                     INACRFLLTYKTHINKDELVLFFNKLCKTKKGENKPEVKFCRSGHEFGKNHESDKEDY
                     EHTHTCIEFTKHFVSQDPEIFNFTTKDKEVIHPHMKYCGKVENKKWNDMKRYISKEDP
                     ENADLLTETLPLVDRVFACDTKYDVAKLCNRPGDIIGLLAIHALKPNEDKKNLIYHEL
                     GGNKFNLYQWQYEILDEMKDMSGNSRKVTWIYDPMGNSGKSTFAKDLQSNGIAYRVEG
                     TGNGYENCAYLLGNAYKNKAWNGKNIIFDFPRGLNLMQSISIYNMIEMLCNGEISIGK
                     YQSEKITMANNAQVYVFANKEPIFCYDDNPTLSFDRWKCLGIKSTKDGTNKVFSKKYY
                     KEICCKINALATKYDEKTVISDDDEPMILKRE*"
     CDS             complement(2087..3583)
                     /codon_start=1
                     /product="Putative CruV Capsid Protein"
                     /translation="MPTRKGTRKSHKGSRKVIRKVIVKGGGDYRRKSYGPRVIRGRGD
                     YKADLRSGLRSVGSGIGGFFSNALGFGDYKVRSNSLMNPGMNEQIPGMHNGRGSVRVS
                     HREYLGDVFGTTLFTNTVYPINPSDSDTFPWLSQIALNFEQWKPHGMIFEYRTMSSDT
                     QVGSTTALGSIVLATEYNSLAPGFPSKQAMENSQFVVSVKPSSSVIHPIECDLSQTPN
                     QPLYVRLPSSAAGDARLFDLGKFQIATQGMTVDGSNQGELWLSYDIEFFKPIIGNLGG
                     ADIAYSHAYQGSGTGDCTVARPVGITSLFTHYSDNILGLGVDNVTRVVYFAPTPAFRI
                     SPIQIVFSWIFEDLQVAPAGVPTLTYANCAVNTTALFGTTGIFRVGDLYQMSLVVYID
                     PTPSASTFSVIVGTDGSWTGNATLDIYATEVNSLSILPSIVSLQQKNKLQSIPLVIEE
                     DRSEKLLLEKKDKEIDFLISELEEYRILKERAKQVTALINEQKSSKKE*"
ORIGIN
        1 ttgccaatgg aaaacaaaga agaacgacac gttacttttt gtattgacaa taatgaatac
       61 tttgctttag aagaaagtat gaaagaactt acagagatta cattaccaaa tgttggtagt
      121 cccaaattta gaattaatgc ttgtagattt ttattaactt ataaaacaca catcaataaa
      181 gatgaattag ttttattctt taataaactt tgtaaaacaa agaaaggcga aaacaaacct
      241 gaagttaagt tttgtagatc aggacatgaa tttggaaaaa atcatgaatc cgataaagaa
      301 gattatgaac acacacatac gtgtatagag ttcacaaaac atttcgtaag ccaagatccc
      361 gaaatattta attttactac aaaggataaa gaagtaatcc atcctcatat gaaatattgc
      421 ggcaaagtag aaaataagaa atggaacgac atgaagcgat acatttcaaa agaagatcct
      481 gaaaatgcgg atcttttaac agaaactctc cctttagttg atagagtatt cgcatgcgat
      541 actaaatatg acgtggctaa attatgtaat agaccaggtg atattattgg acttttagca
      601 attcatgctt taaagccaaa tgaagataaa aagaatttga tctatcatga attaggtggt
      661 aataaattta atttatacca atggcaatat gagatattgg atgaaatgaa agatatgagt
      721 ggtaactcaa gaaaagttac ttggatatat gatccaatgg gtaactcagg caaaagtact
      781 tttgctaaag atttacaatc taatggcata gcatatagag ttgaaggaac aggcaatggt
      841 tatgaaaact gtgcctatct tttaggtaat gcttataaaa ataaagcttg gaacggaaag
      901 aatataatat tcgatttccc aagaggatta aacttaatgc aaagcataag tatttacaat
      961 atgattgaaa tgttatgtaa tggggaaatt agtattggca aataccaatc agaaaaaata
     1021 actatggcaa ataatgcaca agtttatgtt tttgcaaaca aagaacctat cttttgttat
     1081 gacgataacc ctacactatc attcgacaga tggaagtgtt taggaattaa atccactaaa
     1141 gatggaacta ataaagtatt cagtaagaaa tactataaag aaatttgttg caagataaat
     1201 gccttggcaa ctaaatatga tgagaaaacg gttatatctg acgatgatga acctatgata
     1261 ttaaaacggg aataaatccc ttaatatatt tttttgtgaa aaatgatgcg tcactcgcac
     1321 gtctcgccgc taaagcgtct cgacgtgcga gtgactaaag tttgtaacta acgtacactc
     1381 taaagaaacc ctaagcatag taacaagcta tgcttctaag ttaaatattc ccgtgaaata
     1441 ttcccgtgaa atattcaagt aacttgaaaa attaactttg tgattcactt tgaaattaac
     1501 ttggaaacca agattataat ttacaaaagg gtaaaccttg agtgaaagaa aactactaaa
     1561 gtagtaaaag gtttgtaacc aacagttaca ctctaaagaa accccaagca tagaaacaag
     1621 ctatgctctt aagtaaaata ttctgtcaaa tattccgtga aaaatttcag tgaaatattc
     1681 actttatgat tcactttgaa attaacttaa aagccaaggc tgtaattcca aaaaggggta
     1741 aacccttgag tgaacctcaa cgaccaaggt cgagtttggg ataagactaa cattaaagcc
     1801 ttgcggctgt cttatccttt tataataata aatgtgttaa tagttaaaca caagtgggtt
     1861 aaaaagattt ttttaaattt taaaagactt acgctattta aaacctgaaa ataccctttt
     1921 tacgaaccct taacgggttc gtaaaaagtt tggattccac gttgaaaaat atagtctggg
     1981 gactattttt ttccaatatt tttttttaaa aaaacttttt gaatgagtgt taaagttagt
     2041 aataacgtta acccttatag caaaaaaaaa tttaacttag tttaatttat tccttttttg
     2101 aggatttttg ttcgttaata agtgcagtaa cttgtttagc cctttctttc aaaatacgat
     2161 actcttcgag ttcagaaata agaaaatcaa tttctttatc ttttttttca agcaataact
     2221 tttcggaacg atcttcctca ataacaagag gtatagattg taatttattt ttttgttgta
     2281 aagaaacaat agacggaaga attgataaag aattaacttc agtagcgtaa atatcaagag
     2341 tagcattacc agtccaagaa ccgtcagtac caacaataac tgaaaacgta gaagcagaag
     2401 gggtaggatc aatatagact acaagactca tttgatataa atctccaaca cggaaaatac
     2461 cagtagtacc aaataaagcc gtagtattca ctgcacaatt agcatatgta agagtaggaa
     2521 caccagcagg tgcaacttga agatcttcga atatccaact aaaaacaatt tgaataggag
     2581 aaattcggaa agcaggagtt ggtgcaaaat atacgacacg ggttacgttg tcaacaccga
     2641 gtcccaaaat attgtcagaa taatgtgtaa acaatgaagt aatgccaaca ggccttgcca
     2701 ctgtacaatc accagtacca gaaccttgat aagcatgaga ataagcaata tccgcaccac
     2761 cgagatttcc aataataggt ttgaaaaatt caatatcata agacaaccat agttcacctt
     2821 gattagaacc atctacggtc ataccttgag tagcaatctg aaatttacca agatcaaata
     2881 atcgagcatc gcctgcagcg cttgaaggca atcggacata aagtggttga ttcggcgttt
     2941 gagaaagatc acattcaata ggatgaatta ctgatgaaga aggtttaacg gaaacaacaa
     3001 attgagaatt ttccattgct tgtttggaag gaaatccagg agcaagagaa ttgtattctg
     3061 tagcaagaac aatagatcct aaagcagtag tagaaccaac ttgagtatca gaagacatag
     3121 tacgatattc aaaaatcatt ccatgaggtt tccattgttc aaaattgaga gcaatttgtg
     3181 aaagccaagg aaaagtatca gaatctgaag gattaatagg atagacagta ttggtaaata
     3241 acgtagtacc aaatacatca cctaagtatt cacgatgaga aacacggaca gaaccccgtc
     3301 cattatgcat accaggaatt tgttcgttca taccaggatt cataagagaa ttggaacgaa
     3361 ctttataatc accaaatcca agtgcattag agaaaaatcc accgatacct gaaccaacgc
     3421 ttcttaaacc agaacgtaag tcagctttat aatcaccacg accacgaata acacgtggac
     3481 catatgattt acgacgataa tcgccaccgc cttttactat aaccttacgg ataactttac
     3541 gagaaccctt atgagacttt cgtgtacctt tacgagtcgg catgatgttc gatgatcgat
     3601 gatcctatat aataggtaat ctttttttaa aaaaaataat aaataaataa ttccaattaa
     3661 acaaagtata caagtatata tataagtata ttattattac tcgggtcct
//
LOCUS       CruV-343                3719 bp    DNA     circular     24-FEB-2020
DEFINITION  Crucivirus-343 isolate 001315_virusbaton.
ACCESSION   CruV-343
KEYWORDS    .
SOURCE      Crucivirus-343
  ORGANISM  Crucivirus-343
            Unclassified.
REFERENCE   1  (bases 1 to 3719)
  AUTHORS   de la Higuera,I., Kasun,G.W., Torrance,E.L., Pratt,A.A.,
            Maluenda,A., Colombet,J., Bisseux,M., Ravet,V., Dayaram,A.,
            Stainton,D., Kraberger,S., Zawar-Reza,P., Goldstien,S., Briskie,J.,
            White,R., Taylor,H., Gomez,C., Ainley,D.G., Harding,J.S.,
            Fontenele,R.S., Schreck,J., Ribeiro,S.G., Oswald,S.A., Arnold,J.M.,
            Varsani,A., Enault,F. and Stedman,K.M.
  TITLE     Direct Submission
  JOURNAL   Submitted (24-FEB-2020) Biology, Portland State University, 1719 SW
            10th Ave, Portland, Oregon 97201, USA
COMMENT     Bankit Comment: ALT EMAIL:ide@pdx.edu
            Bankit Comment: TOTAL # OF SEQS:451
            Bankit Comment: GAP: est. length - alignment

            ##Assembly-Data-START##
            Assembly Method       :: MetaSPAdes v. 3.12; IDBA-UD v. 1.1.1;
                                     MEGAHIT v. 1.0.3
            Sequencing Technology :: 454; Illumina
            ##Assembly-Data-END##
FEATURES             Location/Qualifiers
     source          1..3719
                     /organism="Crucivirus-343"
                     /mol_type="genomic DNA"
                     /isolate="001315_virusbaton"
                     /isolation_source="Water"
                     /country="France"
                     /collection_date=2013
     CDS             1..882
                     /codon_start=1
                     /product="Putative Replication Associated Protein"
                     /translation="MSIDNCRARNWCFTLNNYTPEEYEEAKSITCSYIIIGKEVGANG
                     TPHLQCYIEFTNAVRGSAIRKRFNERAHWEVRKGTSKQASEYCKKENNFIELGTLSAQ
                     GRRTDLETIGQAVVDGDSIKEIATTYPGTFIKYHKGINALRNALYEHRTEPPTVVWRW
                     GLAGVGKSRGAYEAHSTFYIKDGTPWWDGYEQQVAIVIDDFDVGKWPFRDMLRLLDRY
                     PYQGQYKGGYIPINSPFIYITCEHPPYHFWSGNELAQVMRRLAQVIEVKPPILGCYAQ
                     TPQNGAPVEETKTGLST*"
     CDS             complement(2057..3604)
                     /codon_start=1
                     /product="Putative CruV Capsid Protein"
                     /translation="MNKHIPIGIPVNVVQLDNMPQPKAGRVAVRRPAKKRVVVAPRRR
                     VAAPRPRAAPRGSYKTKSGSKSGLFGTAGGAVGGMIGTALGGPGGGAIGSVLGRLGGA
                     ALSSIFGHGDYAVSNVPAIKENNLVLSNAAQVPQFGTGKVASKFVHREFLGDIYSSST
                     AGAFKIDNYPINPGVGTTFPWLSGVVGAKFQQYRINGMTFEYRSMSSDALNSTNTAFG
                     SVVMSTDYDSADIVFASKQEMENTEYGVSCKPSCNMLHAIECARAQTPVSELYIRSFD
                     VPSGKDIRLYDLGRFSIATVGFQGTSVNCGELWVSYDIDAFKAIEQVPGYQTPFAAYA
                     LNSASATSPLGTSQSLFATGADQIGLTFASENKVTWPLTTPIGSTYMVNYVLIGGSAA
                     AITPPAVTTGGGMYVSSTVLLFTPHSGVTTQYAQGVWVIRYNGGGTPAAPPYFNIADF
                     TWAPSSIPECDLFVTQVSGLWPGTFTGTTGEVPPSLADETEVPEYISVCEEKKAHTLL
                     PVCVRRR*"
ORIGIN
        1 atgagcatcg ataactgtcg tgcacgtaac tggtgtttca cactgaacaa ctacactcct
       61 gaggagtatg aagaggctaa gagcattaca tgctcttaca tcatcatcgg caaagaggtc
      121 ggtgccaatg gcacccccca cttgcaatgt tacattgaat tcactaacgc tgtccgaggg
      181 tcagcgattc gtaaacgatt caacgaacgc gctcattggg aagttcgcaa aggaacttcc
      241 aaacaagctt ccgaatattg taagaaagag aacaacttta tcgagttggg tactctttct
      301 gctcaaggca gaagaactga ccttgaaaca ataggtcagg ccgtagtcga cggcgactcg
      361 atcaaagaga tcgcgactac ttaccctggc acctttatca agtatcataa aggaatcaac
      421 gcgttgagaa acgcattgta cgaacaccgc acagaaccac ctacggtggt ctggcgatgg
      481 ggcctcgccg gcgtcggcaa gagccgtggc gcttacgaag cgcacagcac tttctacatc
      541 aaggacggaa caccctggtg ggatggttac gagcaacaag tcgccatcgt catcgatgat
      601 ttcgatgtcg gcaaatggcc attcagagac atgttacgtc tccttgatcg ttacccttat
      661 cagggccagt acaaaggcgg ttacattccg attaactcac ccttcattta cattacgtgc
      721 gagcatcctc cataccactt ttggtctgga aatgaactag ctcaagtcat gagacgactt
      781 gcgcaagtca ttgaggtcaa accccccata ctggggtgtt atgcacagac accccagaac
      841 ggggcgcctg tagaagagac gaaaaccggt ttatcaacct aatttcaggg tgcgcgctaa
      901 gcgacccctt attttttgat ctattctaat aaaaacctac ggttcccatt tctgggaacc
      961 tggcacagag gttttttcca cttctgacaa acccataggg tttttccagc gattaaagac
     1021 gctggcaata atatttaatt cttggcacag aagtggcgtg gtaatactag tgtgccaaag
     1081 cacacttaca cgccacatac ggtctttgac ctattctgac cactttttcc caaggagcga
     1141 attcgctcct tgaggtacca aatttggtac ctggttctga gagaggaaga gtaaagttat
     1201 cttttttttg cgacattgga tcgccgattg ttatgtaact ttggactcgc tcactccggt
     1261 cgagctcggt cttgtgatcc tcttctcact cattaggcgg gggttttttt acccccgcta
     1321 tagcgggggc aaggtcttga tgactccgac ccaccctttt gaccccctaa tttctagggg
     1381 gttatcagtg cagattgttc tgcactgtat atagctatcg cgttacacga tgacctcatt
     1441 atgcccagac ataatgagca taatctacga ttacgtctat cccccttggg aatgtcggca
     1501 agttcttagt gaacttacct ctctccatga cgaatggaag agagaaaata tatcaggatg
     1561 gtctttcgcc cattgggtca cagtatggac catgtactca gaaccgagtg acgaagatca
     1621 ctactaccct gacatagact cgtcattctt cgatgacgaa gattgataca ggcatggtgc
     1681 tgctcgcaac gctcgcaggc cgctccgcgg cctcctgcac aactttatgt cgtgcggctt
     1741 acgccgcagc cagaggcgct gccgcgcggc cccctgcggg gggcataagg cagatagggg
     1801 gtttatagtt tattcttttt tttcaggtta cggctccaga ggcgcctaac tgactgatgt
     1861 tgttcatcga ccggcccgat gggccaccgt ctcacatgtt ccggacattg tcttttttaa
     1921 attcacatcg tttcattccg caaatttgcg gacaaaaaaa atccacactc ctcacactaa
     1981 ccgcagcaca taagctgcgg tcgctacgct tgctccccat ttttggggag ctctctttgc
     2041 tcgttaaatt taacgatcaa cgcctacgca cacacacagg caaaagcgta tgcgcttttt
     2101 tttcctcgca cacagagatg tactcgggca cctctgtctc gtcagcgaga cttggaggta
     2161 cctcccctgt ggtacccgta aaagtgcctg gccataggcc actcacctga gtgacgaaca
     2221 agtcacactc tgggatactt gatggagccc aagtaaaatc agcgatatta aaatatggtg
     2281 gagcagcagg agtgcctcca ccgttatatc tgatcaccca tacgccttgg gcgtactggg
     2341 tagtcacacc agaatgtggt gtgaacagaa ggactgtact agacacatac atacccccgc
     2401 ccgttgtaac ggcggggggt gtgatagcag cagctgagcc tccgataagg acataattaa
     2461 ccatgtacgt ggaccctata ggggtcgtaa gaggccacgt aaccttattc tccgacgcaa
     2521 acgtaagacc gatctgatcg gcccccgttg caaacaagga ctgcgacgtc cctaaagggg
     2581 acgttgcaga tgcactattc agcgcataag cggcgaacgg cgtctgataa cccgggacct
     2641 gctcgatcgc tttgaaagcg tcgatgtcat aagacaccca aagttcccca caattcacac
     2701 tcgtaccttg gaagccaacg gtagcgattg agaaacgacc aaggtcgtac aatcttatgt
     2761 ctttgcctga aggcacgtca aaggatcgaa tatacaactc agacacaggc gtctgcgctc
     2821 gagcgcattc gattgcgtgc agcatgttgc acgatggctt acatgacaca ccatactccg
     2881 tattctccat ctcctgcttg gacgcgaaca cgatatcggc cgaatcgtag tccgtcgaca
     2941 tgaccaccga cccaaaggcg gtgttcgtcg aattcagagc atccgaagac atgcttcgat
     3001 actcaaacgt cataccgtta atacggtact gttggaactt agctccaaca acacctgaca
     3061 accaggggaa tgtagtccct acccctggat tgataggata gttatcaatt ttgaacgcac
     3121 ctgcagttga tgaactgtag atgtcaccga ggaactctcg atgaacaaat ttactcgcaa
     3181 ccttcccagt tccaaactgg ggaacttgcg cagcatttga gagaacaaga ttgttctcct
     3241 tgatagcagg cacattagac acggcgtagt cgccgtgtcc gaagatactc gacaaggctg
     3301 cgccgcctaa ccgaccgagt acacttccaa ttgcgcctcc accaggaccg ccaagagcgg
     3361 tcccgatcat gccaccaacc gctcctcctg ctgtaccgaa cagtccgctt ttgctacctg
     3421 atttcgtctt gtacgaaccc ctcggggctg cccgagggcg tggcgctgcc actctccgac
     3481 ggggtgcgac aacaactctc ttcttcgcgg gtctacggac cgcgactcga ccagctttgg
     3541 gctgaggcat gttgtcaagt tggacaacgt tgactggtat acctataggt atatgtttgt
     3601 tcagaacatt tattaattaa atgaattatt taatttttct tcttagcatt ggcacagaag
     3661 ttactatata gtcagtgaca tacacatagt catagatact caacaacttt ggacgggag
//
LOCUS       CruV-344                3726 bp    DNA     circular     24-FEB-2020
DEFINITION  Crucivirus-344 isolate 120_1169_IR2_3297.
ACCESSION   CruV-344
KEYWORDS    .
SOURCE      Crucivirus-344
  ORGANISM  Crucivirus-344
            Unclassified.
REFERENCE   1  (bases 1 to 3726)
  AUTHORS   de la Higuera,I., Kasun,G.W., Torrance,E.L., Pratt,A.A.,
            Maluenda,A., Colombet,J., Bisseux,M., Ravet,V., Dayaram,A.,
            Stainton,D., Kraberger,S., Zawar-Reza,P., Goldstien,S., Briskie,J.,
            White,R., Taylor,H., Gomez,C., Ainley,D.G., Harding,J.S.,
            Fontenele,R.S., Schreck,J., Ribeiro,S.G., Oswald,S.A., Arnold,J.M.,
            Varsani,A., Enault,F. and Stedman,K.M.
  TITLE     Direct Submission
  JOURNAL   Submitted (24-FEB-2020) Biology, Portland State University, 1719 SW
            10th Ave, Portland, Oregon 97201, USA
COMMENT     Bankit Comment: ALT EMAIL:ide@pdx.edu
            Bankit Comment: TOTAL # OF SEQS:451
            Bankit Comment: GAP: est. length - alignment

            ##Assembly-Data-START##
            Assembly Method       :: MetaSPAdes v. 3.12; IDBA-UD v. 1.1.1;
                                     MEGAHIT v. 1.0.3
            Sequencing Technology :: 454; Illumina
            ##Assembly-Data-END##
FEATURES             Location/Qualifiers
     source          1..3726
                     /organism="Crucivirus-344"
                     /mol_type="genomic DNA"
                     /isolate="120_1169_IR2_3297"
                     /isolation_source="Water"
                     /country="Norway"
                     /collection_date=2011
     CDS             1..1287
                     /codon_start=1
                     /product="Putative Replication Associated Protein"
                     /translation="MPEISKCIERRFNSNNYLFYKVTFLHKYIKVKMSSRHYCFTVNL
                     QDNSEIWSVVPEACVDEDEKVIEILSEEDVEEYLENSFIKYIIYQREVAPTTGRHHYQ
                     GYVEFKKNLTIKQAKSVLNDDTVHLEARKGTQQQAIDYVSKLSTAIPETQFTWGEPGR
                     QGARTDIKKTKAALLSNIDSSTNVRDFAIANPDLYVRMHQGIDKLVYHHKKPRDFKTE
                     VHIFWGVSDSGKTRKVYDDALASNQSVYKKPNGQWWDGYEGQDVVLIDDYDSECVLPF
                     REFLQLNDRYPHQVPIKGAFRQFTSKKIVYTSNQDPIDWYPNISEESLKAFKRRITTV
                     LHFPDLYNPPIEDLSFYMRSGSSPYDEPYDEPHNSSPDIQVFGDVSEAKLRNKIKALS
                     IPAVEPRRNNVSTIRDDIESIVEVAKSAVETADSCC*"
     misc_feature    70..97
                     /note="stem-loop"
     misc_feature    82..90
                     /note="nonanucleotide"
     CDS             1727..3064
                     /codon_start=1
                     /product="Putative CruV Capsid Protein"
                     /translation="-MPRPAYRRPRARPRAKPRAKPRYRRQVQGPGFIRRTAGYLAPV
                     LAGAAIRSGMHRLMGQPRDPGVYDYAKAAYDAAGGLSGIGSSASALIANTAGRILGFG
                     DYKVKRNSLMHGSQVPVMHSAHDGVRIRHREFIQDLSGSVAFTNTSFSMNPGLPESFP
                     WLSAIAQNFEEYKWEGLVFEFKSTSADALNSTNTALGTVVLAAEYNTAQSAYVNKQQM
                     ENSMWAMSGKPADCHIMPVECEPSLNPLSNQYIRLGAVASNQDIRMYDLCNVQVATVG
                     MQAAAVIGELWVSYDVLLMKPQLDSGLALSANSASYQLTAPVVTTAYFGTARTADFDS
                     IGLSISGTVITFPLGMQGVYSMWYQVVGDSTAVSSPTFTLVNATELSILVGNSVGTIN
                     NSGNTSTKYLRNAYFRITDPNIACTITASVGTLPANPTSGDLVITQLNGSLAA*"
     CDS             3110..3508
                     /codon_start=1
                     /product="putative ORF"
                     /translation="MSVDATNVLGTSQTNQYDNIGLTFDSATRKISFTSGNEGIYLMT
                     YNVSGGAVIVDGPTISYTNASAFNLYENGSDNGFSNSGLLSARYYELAAFRITTNTQT
                     ASVNFTTAVTFPTAVSSADIVITRLADFIS*"
ORIGIN
        1 atgcctgaaa taagcaaatg tattgagcgc aggtttaatt ctaataatta tttattttat
       61 aaagttactt ttttacataa atatataaaa gtaaaaatgt catctcgtca ttactgtttt
      121 actgttaacc ttcaagataa ctctgaaatc tggtccgtcg ttcctgaggc ttgtgtagat
      181 gaagatgaga aagtcatcga gatcttatca gaagaagatg tagaagaata tcttgaaaac
      241 tcttttatta aatatatcat ttatcaaaga gaagttgctc cgaccaccgg tcgtcatcat
      301 taccaaggtt acgttgaatt taaaaaaaat ttaactatta aacaggctaa gtctgtcttg
      361 aatgatgata cagtacatct cgaagctcgt aagggtactc agcaacaggc aatagattat
      421 gtttctaagc tttctactgc tattcctgaa acccaattta cttggggaga acctggtcgt
      481 caaggtgctc ggacagatat taaaaaaact aaagctgctc tattgtctaa cattgattct
      541 tctactaacg ttagggattt tgcaattgca aatcctgatt tatatgttcg tatgcatcaa
      601 ggtattgata aacttgttta tcatcataaa aagccacgcg attttaaaac tgaagtccat
      661 atcttttggg gagtttcaga ttctggtaag accagaaagg tttacgatga cgctttagct
      721 tcaaatcaat cggtctataa aaagccaaac ggacaatggt gggacggata cgaaggccaa
      781 gacgtagtat tgatagatga ctatgattca gaatgtgttt taccattccg tgagttttta
      841 caactcaatg atcgttatcc tcatcaagtt ccaataaaag gtgctttcag gcaatttact
      901 tctaagaaaa tagtttatac ttctaatcaa gatcctatcg actggtatcc taatatctca
      961 gaagaatcgc ttaaagcttt taagcgtcgt ataactactg ttctacattt tccagattta
     1021 tataaccctc caatagagga tcttagtttc tacatgaggt ctggttcaag tccttacgat
     1081 gaaccttacg acgaacctca taattcatct cctgatattc aggtatttgg tgatgttagc
     1141 gaagctaaat tgcgtaataa aataaaagct ctttcaatac ctgctgtaga acctcgccga
     1201 aacaacgtgt caacaatacg tgatgatatc gaatcaattg ttgaagtagc taagtctgct
     1261 gtagaaactg cagattcatg ttgttaattt ataaaggaag actctttata aatccccccc
     1321 cccttcatta tacaatccgg ttcaagggca gcatacttgc ccttgcctcc ccaaaaaaga
     1381 aaaccttttt tttgccgtaa ggcatttagt caacctaaaa attgtaccat aattcttata
     1441 tcctggccat gtgtattaca ctttcagatt gcagtggggt ccccgtttaa acgacgattt
     1501 aaacgacgaa tacggggtgc tgcaatccca tagtaagaat accatcggcc atatccacaa
     1561 agctgtaccg aaggtaccgt aattcttaat gtcaactaca aatattcaaa ctttttgcga
     1621 cactctaatc taacaaatat aaaaaaaaac actatcagca atctttgaat attatttatt
     1681 ttacattaaa cattattttc tattatatat atcttataca taaaaaatca tgcctcgtcc
     1741 agcttatcgt agacctcgag cccgacctcg agctaaacct cgtgcaaaac ctcgttatcg
     1801 tcggcaagtt cagggtccag gttttatccg cagaactgct ggttacttag cacctgttct
     1861 tgctggtgct gccattagat caggtatgca tcggcttatg ggtcagcctc gtgaccctgg
     1921 agtctacgat tatgccaaag ctgcttatga tgcagctggc ggtctttctg gtattggttc
     1981 gtcagcttca gctcttattg ccaatacagc tggccgaatt ttaggtttcg gcgattataa
     2041 agttaagaga aattctttaa tgcatggcag tcaagttcct gttatgcatt ctgctcacga
     2101 tggagttcgt attagacatc gagagtttat tcaggaccta tcaggttccg ttgcattcac
     2161 taatacttcc ttctctatga atccaggtct tcctgagtct ttcccttggc tatcggctat
     2221 agctcaaaat tttgaggaat ataaatggga aggattagta tttgaattta aatctacatc
     2281 agctgatgct cttaactcta caaacactgc tttaggtaca gttgttttag ctgcagaata
     2341 caatactgca cagtccgctt atgttaataa gcagcaaatg gagaattcta tgtgggctat
     2401 gtcaggcaaa cctgctgact gtcatattat gcctgtagaa tgtgagccct ctttaaatcc
     2461 tctatctaat caatacatcc gtcttggtgc tgtagcatct aaccaagata tacgtatgta
     2521 tgatctctgc aatgttcagg ttgcaactgt tggtatgcaa gccgcagccg ttattggtga
     2581 actttgggtt tcttatgatg ttttactcat gaaacctcaa cttgactctg gtttggcgtt
     2641 gagcgcaaat tcggctagtt atcagttgac agcaccagtt gtcactacag cttattttgg
     2701 tactgctaga actgcagact ttgatagtat tggtctgtca atatctggca ccgttattac
     2761 atttccttta ggaatgcaag gtgtttattc tatgtggtat caagtagtcg gagatagtac
     2821 agctgtatct tctcctacat ttacattagt aaatgcgacc gaactaagta ttttagttgg
     2881 aaattcggtc ggtactatta ataattcagg aaatacatca accaaatatt tgagaaatgc
     2941 ttatttcaga atcacagatc cgaacattgc ttgtactatt acagcttcgg tcggtacttt
     3001 gccagcaaat cctacatctg gtgatttagt cattactcaa ttgaatggca gtttggccgc
     3061 ataggaagat tcggtggaat cagatggtaa atctgcccat tatcgcctat tgtcagtcga
     3121 tgcgactaat gttttgggta caagtcaaac taatcaatat gataatattg gacttacttt
     3181 tgattctgcc acacgtaaaa tatcatttac ttcaggtaat gagggcatat atcttatgac
     3241 ctataatgtt tcgggtggtg ctgtgattgt tgacggtcct accatttcat atacgaatgc
     3301 ttcagcgttt aatttatacg aaaatggttc tgataacggc tttagtaatt caggcttatt
     3361 aagcgccagg tattatgagc tggctgcttt tcgtataaca acaaacactc aaacggcttc
     3421 agttaatttt actacagccg tcacattccc taccgctgtt tcgtcagcgg atatagttat
     3481 aactcgttta gctgatttta ttagctaata atatttttgg taaaccaaaa aaaatgcgaa
     3541 gcatagtgga tagcaaacta atttaaaaat gaaatttttt tttagtgtag tattacctat
     3601 ccacttcata aaactgtcta tctttatcgc tgtctttatg aattgaccct attagcaaag
     3661 ctattgggtt gacacttcgt ttttcacttt gtaaaagaca ttgtaaatga agtagtcttt
     3721 attata
//
LOCUS       CruV-345                3728 bp    DNA     circular     24-FEB-2020
DEFINITION  Crucivirus-345 isolate C11_167.
ACCESSION   CruV-345
KEYWORDS    .
SOURCE      Crucivirus-345
  ORGANISM  Crucivirus-345
            Unclassified.
REFERENCE   1  (bases 1 to 3728)
  AUTHORS   de la Higuera,I., Kasun,G.W., Torrance,E.L., Pratt,A.A.,
            Maluenda,A., Colombet,J., Bisseux,M., Ravet,V., Dayaram,A.,
            Stainton,D., Kraberger,S., Zawar-Reza,P., Goldstien,S., Briskie,J.,
            White,R., Taylor,H., Gomez,C., Ainley,D.G., Harding,J.S.,
            Fontenele,R.S., Schreck,J., Ribeiro,S.G., Oswald,S.A., Arnold,J.M.,
            Varsani,A., Enault,F. and Stedman,K.M.
  TITLE     Direct Submission
  JOURNAL   Submitted (24-FEB-2020) Biology, Portland State University, 1719 SW
            10th Ave, Portland, Oregon 97201, USA
COMMENT     Bankit Comment: ALT EMAIL:ide@pdx.edu
            Bankit Comment: TOTAL # OF SEQS:451
            Bankit Comment: GAP: est. length - alignment

            ##Assembly-Data-START##
            Assembly Method       :: MetaSPAdes v. 3.12; IDBA-UD v. 1.1.1;
                                     MEGAHIT v. 1.0.3
            Sequencing Technology :: 454; Illumina
            ##Assembly-Data-END##
FEATURES             Location/Qualifiers
     source          1..3728
                     /organism="Crucivirus-345"
                     /mol_type="genomic DNA"
                     /isolate="C11_167"
                     /isolation_source="Biofilm"
                     /country="New Zealand"
                     /collection_date=2012
     CDS             1..981
                     /codon_start=1
                     /product="Putative Replication Associated Protein"
                     /translation="MTAAKACVYERQWCYTWNNFTCEDYASMKEWCEKYCTWWIMGIE
                     KGEEGTPHIQGAFRTKNTRTFKNLKNKFGKVHWEMMKGTCQQSLTYCSKGGNFEVGGK
                     LPAQGKRQDLDNLKDAIIKDSVKLDDIICKMPMLYHQYGRTLEKLEDIALRKKSRTEM
                     TEGIWYWGKTAVGKSHLAFEGYHPSTHYLLSLEDKGWWEGYNGQDTVIINDFRGEIKY
                     SELLQLIDKWPYNVTRRNRGTVPFISKRVIITSSLPPDKCYRHRQDEDCIEQLLRRLK
                     VVELVKEPMKVPLAESSGSGRSGQGNTIHDHSASATKKSCICDATFCECI*"
     CDS             complement(1982..3601)
                     /codon_start=1
                     /product="Putative CruV Capsid Protein"
                     /translation="MNRSVRTSKTGGVTRKASGTSRASSSKMAAPRAKRWTFAADAKG
                     FGIGGGIKISSKRKGMSRAVAPSATSTTVSQYKPKVSGGSAANSFTIENRELLSSCLW
                     QGSNGFELTEFSINPGLSSIFQFASNASKNYTRYAMNMRLVWAPSVPSTTSGQIAFAM
                     DRDNTSVAPSSMQEVLSYESNVVTNIWSCVNFPENGMFASSEKLLYLRYGPLDDLSQI
                     NLYDLMKCYICLNGIAGASLGTGQSLGSVYIEYKITFFNQKLQDIVESNIFSVQSTSA
                     VGPGTVAIADDDDTTKYLYQYGEGDVQYLLQNSSTGHFYLIFPTAGYYTVDFYMFVSQ
                     DTGTITGPIQALSLVSELITVGTEVINGDLGTGSFNIETNLYTALTTNATNAQAHTKY
                     VCQVLSSGSYSGSNGAPIGEGWLYFDPGTILASIAADPNIGTAVFSCYQSYCIVNRIS
                     AAEAYWFYPLAFPEGASPSPLTQTISHRRKFHPVKGHARQNRRPPHPTSSSKQMPPLL
                     EDQSEKEESKEPKSEYDEFLAWKKKSTYLSK*"
ORIGIN
        1 atgaccgccg ctaaagcttg tgtttacgaa cgacaatggt gttatacgtg gaataacttc
       61 acgtgtgaag attatgcatc catgaaggaa tggtgtgaaa agtactgcac ctggtggatt
      121 atgggtatag aaaaaggtga agaaggtaca cctcatatac aaggtgcttt tagaactaaa
      181 aacactagaa catttaagaa cttaaaaaat aagttcggta aggtgcactg ggaaatgatg
      241 aaaggcacat gtcagcaaag tctaacgtac tgttccaaag gaggtaattt tgaagtagga
      301 ggcaaactac ctgcccaagg aaagcgtcaa gacttagata acctgaaaga tgctatcatt
      361 aaggatagtg ttaagttaga tgacattata tgtaaaatgc ctatgcttta tcaccagtac
      421 ggtagaaccc tcgaaaaact cgaggacatt gctcttcgta aaaagtcgcg aacagaaatg
      481 actgaaggta tctggtactg gggaaaaaca gctgtaggca aaagtcatct ggcttttgag
      541 ggctatcacc catctacaca ctatctttta tctttagaag ataaagggtg gtgggaaggc
      601 tataatggac aggataccgt cattattaat gatttcagag gtgagataaa gtactctgag
      661 ttattacagc tcattgacaa gtggccgtat aacgtcacac gtcgaaacag gggaacggta
      721 cccttcattt cgaaaagagt tatcataaca tcatcattac cgcctgataa gtgttacaga
      781 catcggcaag acgaggattg catcgagcag cttttgcgta ggttaaaagt tgtcgaactc
      841 gttaaagagc cgatgaaagt gccgctagca gaaagtagcg gcagtggcag aagtggtcaa
      901 ggtaatacta tacatgacca ctctgcctct gccaccaaaa agtcttgcat ctgcgatgcg
      961 actttttgtg agtgtatata ggcttacatg taaaacacga accctattct gtgttcgcgt
     1021 tttccccata atgtatcctc aatatcggat acattcttat gtaagccgag gacaccgtcc
     1081 cataaattta cccttacagg gataaatttc tgcggtgccc atgatatata tcatcatccc
     1141 cattttgtga tgatgatata tatcgttgtt gtgtatctat actcgatcaa attcacgatc
     1201 gtcatatgat cgaggggctc ccctataatt tacattccct gcgggaatgt aaattatagt
     1261 gtcctttctc tggtctcatg tgaacacaaa atgtgtcaga tctaccgtcg aatgtgtacg
     1321 ccctgacaga taagatctgc tcacttggaa tgacaaatta ttatacgaat ccattcccat
     1381 aaaaatgtcg caagctccat tttgtatggg cccctttgcg tataataatt tatcattcct
     1441 ggccgtgaag cagatcatat ctgctttggg gcgtacttca cgacgggatc atgtgacaca
     1501 tttgcgtgtg tgagtcggag agatattttt aatatacatg caagtatatg gttacaggta
     1561 tattatagta gtggtgtcgc tcacgcaagc tccgctaggg tccgctcgca cttcgcttcg
     1621 ctacgttacg cgggagtttt tattatggtc gccaattagc gccctaacgc aggcgctcgc
     1681 cgccgctgct cgcgctgtgt gtcggcgcta attattgaaa aaggtcaact tcctcaacat
     1741 agatagaacc ggacatagct aatctagtgt ttaagttatg taccttctct gttaaggaga
     1801 gtatgtgcga tttgagctta agtatagtag ccttataaga ttcgatttcc tcatccattt
     1861 gagaggctac ctctttgagg agacgatact tgagcatttt ttcattatgg taagtatggg
     1921 tgactgacat cttatatact attactaata tataagatat ttttttataa gttttcactt
     1981 attacttaga aaggtaggta cttttcttct tccatgctaa gaactcatca tattcactct
     2041 taggttcttt agattcttct ttttcagatt gatcttccaa aagaggaggc atttgtttag
     2101 aggaagaagt agggtgaggt ggtcgcctat tctgacgtgc gtgacccttt acaggatgga
     2161 acttacggcg atgtgaaata gtctgtgtta gcggtgatgg agaagcacct tcggggaaag
     2221 ctaaaggata gaaccaataa gcttccgcag cagatatgcg attcactata cagtatgact
     2281 gataacacga aaaaacagca gttccgatat tggggtctgc tgcgatacta gcaagtatgg
     2341 tacctggatc aaaatataac catccctctc cgataggtgc accattactt ccggaataag
     2401 aaccggaaga aagaacctgg catacgtact tagtatgagc ttgagcatta gtagcattag
     2461 tagtaagagc ggtgtaaaga ttagtttcga tattgaaact accggtacca aggtctccat
     2521 taatcacttc agttcctact gtaattagtt cagagaccag tgataaagcc tggatgggcc
     2581 cagttatagt accggtatcc tgtgagacaa acatataaaa gtcgacagta taatatccag
     2641 ctgtaggaaa gataagatag aaatgtcctg tagaagaatt ctggagaaga tactgaacat
     2701 caccttctcc atactggtag agatacttag tcgtatcatc atcatcagct atggctacag
     2761 ttccaggacc tacagctgag gtagactgaa cagaaaagat attagactcg actatatctt
     2821 ggagcttctg attaaagaat gtgatcttat actcgatata tactgaaccg agagactgtc
     2881 cagttccgag agaagcacca gcgataccat taagacagat atagcacttc ataagatcat
     2941 aaaggttaat ctgagaaaga tcatctaatg gaccataacg aaggtatagt aacttctctg
     3001 aagaagcaaa cataccattc tcggggaagt taacgcaaga ccaaatatta gtcactacgt
     3061 tactttcata agaaaggact tcctgcatag atgatggagc tacagaagtg ttatcacgat
     3121 ccatagcgaa agcgatttgt ccactagtcg tagatgggac tgaaggagcc cacactaatc
     3181 gcatattcat agcatatctg gtataattct tagaagcgtt cgaagcgaac tgaaagatac
     3241 tagaaaggcc tggattaata gagaactcag taagttcaaa accattagat ccttgccaga
     3301 gacagctaga aagaagctcg cgattctcga tagtaaagga gttagcggca gaacctccac
     3361 taaccttagg cttatactga gaaacagtag tagatgtggc tgatggagct accgcacggg
     3421 acattccttt acgcttgcta gaaattttga taccgcctcc gataccaaag cccttagcgt
     3481 cagcagcaaa tgtccatcgc ttggctctag gtgcagccat cttagaacta gatgcgcgag
     3541 atgtgcctga ggcttttcgt gttacaccac ccgtttttga agtacgaact gatctattca
     3601 ttttgctagt tatactagtt atatatggga cagactttat tttatagctt tttagcgttt
     3661 ttttcaaaac tagaaaatta ttaaaaaaag taagctataa aaaaagaaag tatatataac
     3721 aataaaaa
//
LOCUS       CruV-346                3749 bp    DNA     circular     24-FEB-2020
DEFINITION  Crucivirus-346 isolate 120_1193_IR2_3297.
ACCESSION   CruV-346
KEYWORDS    .
SOURCE      Crucivirus-346
  ORGANISM  Crucivirus-346
            Unclassified.
REFERENCE   1  (bases 1 to 3749)
  AUTHORS   de la Higuera,I., Kasun,G.W., Torrance,E.L., Pratt,A.A.,
            Maluenda,A., Colombet,J., Bisseux,M., Ravet,V., Dayaram,A.,
            Stainton,D., Kraberger,S., Zawar-Reza,P., Goldstien,S., Briskie,J.,
            White,R., Taylor,H., Gomez,C., Ainley,D.G., Harding,J.S.,
            Fontenele,R.S., Schreck,J., Ribeiro,S.G., Oswald,S.A., Arnold,J.M.,
            Varsani,A., Enault,F. and Stedman,K.M.
  TITLE     Direct Submission
  JOURNAL   Submitted (24-FEB-2020) Biology, Portland State University, 1719 SW
            10th Ave, Portland, Oregon 97201, USA
COMMENT     Bankit Comment: ALT EMAIL:ide@pdx.edu
            Bankit Comment: TOTAL # OF SEQS:451
            Bankit Comment: GAP: est. length - alignment

            ##Assembly-Data-START##
            Assembly Method       :: MetaSPAdes v. 3.12; IDBA-UD v. 1.1.1;
                                     MEGAHIT v. 1.0.3
            Sequencing Technology :: 454; Illumina
            ##Assembly-Data-END##
FEATURES             Location/Qualifiers
     source          1..3749
                     /organism="Crucivirus-346"
                     /mol_type="genomic DNA"
                     /isolate="120_1193_IR2_3297"
                     /isolation_source="Water"
                     /country="Norway"
                     /collection_date=2011
     CDS             1..1473
                     /codon_start=1
                     /product="Putative CruV Capsid Protein"
                     /translation="MVKRRKQHYPLAPIHDQYHAQAIMDQYDPKRARGTDFSKSRYGA
                     TALLATPEQRLNRRLGRFRGQGGYKDYLKWIPRGIGALAGGALALESGGDVIGGAKSG
                     WGRGGQFSKWAGWGDYAPVTMNSIMSGNEGMSSQQQIQVSNGAVDGSGDVLISNTEFV
                     QNVVVTASAAGTSSFSLQSYALNPGMSDAFPWLSQISNNFSLYEFQQLVFQYKPTSGE
                     FGSSGSNSLGKIIMATNYDADSENFANSRQMENYDYASACKPSTGMLHGVECAKKQRM
                     TDQLYVRSGATSKDKVLTDLGTFQIATEGITTSAAGTFVIGELWVSYTLKLSRKLLNS
                     KLGVDIEFDQASQTGSATPGSAVTYQARQIGGTLSYISTTTFRYTFPLNVNSGFYHIT
                     AVIKSDNLASNRQFSTTVPALNCTVRAVNNSLGFNISHPQTLTGVGALASNGSIEFFV
                     SVDAPGALLAHVDFVVGAGGFEATTGVSVLVMQANPQVVS*"
     misc_feature    907..929
                     /note="stem-loop"
     misc_feature    915..923
                     /note="nonanucleotide"
     CDS             complement(1474..1899)
                     /codon_start=1
                     /product="putative ORF"
                     /translation="MPILDKIEWEAQLRNVPVETIWDEYIAMGGGRFLDLTKEKDPRS
                     PPPSPRTPSPTPEDSSLSPEMKVEQRRRSQRLRQRSMISPPIPIRMRGSEYWNSLCAP
                     PLNLNLSIIDFEFELPDVDALEIDTNLINHMKRNANCID*"
     CDS             join(2013..2394,2444..2949)
                     /codon_start=1
                     /product="Putative Replication Associated Protein"
                     /translation="MIRLFYFAGLFFDRIKMSRSRNWCFTLNNYSEDDVKTFENVECE
                     YIIFGFEVGESGTPHLQGYIRFKNACRLSTLSSLCARAHYEVAKGDALSNIKYCSKQF
                     NEKQLLGEEWRPFFEKGDRPFTKSEASVARYDDAYKLAREGNFEDIQKDILIRCYNSL
                     RRIHSDFKQPVLMLQPGSKHEWFYGPTGSGKSRAAREHNGYFLKNASNKWWDGYNAEE
                     VVILDDFDKKFEYNGYYLKIWADIYPFSAEVKNGSTGSIRPKKIIVTSNYHPNEIWTD
                     EQTLEPLLRRFNVTKYNVL*"
ORIGIN
        1 atggttaaaa gacgtaaaca acattatcca cttgctccta ttcatgatca gtatcatgct
       61 caagccataa tggatcagta tgatcctaag cgtgcgcggg gtaccgattt ttccaaaagc
      121 aggtatggtg ctactgccct tcttgccact cctgagcaaa gattgaatcg gagattagga
      181 agatttcgcg gtcaaggagg ttataaagat tatttaaaat ggataccccg cggtattggc
      241 gctcttgctg gcggggcttt agcactcgaa agtggcgggg acgtcattgg gggtgcgaag
      301 agcggttggg ggcgaggagg acagttctct aaatgggctg gttggggtga ttatgcccct
      361 gttactatga attctattat gagtggtaat gaaggcatgt ctagccaaca acaaattcag
      421 gtttctaacg gtgcagtcga cggttctggt gatgtcctta tttcaaatac tgaatttgtt
      481 caaaatgttg ttgtgactgc gtctgctgct ggtacatcta gtttttcgtt gcagtcctat
      541 gctctaaatc caggaatgag cgatgccttt ccttggttgt ctcagatatc taataatttt
      601 tcattgtatg agtttcaaca gcttgtcttt cagtataaac ctacttctgg tgaatttggt
      661 tcttctggtt ctaattcact tggaaaaatt attatggcta cgaattatga tgctgattct
      721 gaaaactttg caaattctcg ccaaatggag aattatgatt atgcatctgc atgtaaacca
      781 tcaactggta tgttgcatgg agttgaatgc gctaagaagc agcgcatgac cgaccagttg
      841 tacgtaagga gtggtgcgac aagcaaggac aaagtattaa ccgatttggg tacatttcaa
      901 attgctacag aaggtataac aacttctgct gcaggcactt ttgttatagg cgagttatgg
      961 gtgagttaca ccctaaaact gagccgcaag cttcttaata gcaaacttgg agttgatatt
     1021 gaatttgatc aagcgtccca aaccggtagt gccactcctg gtagcgctgt tacttatcaa
     1081 gcccgtcaaa ttggaggaac gcttagctac attagcacaa cgacttttcg ctatacattt
     1141 cctttgaatg ttaattctgg cttttatcat attactgctg ttattaagtc agataatctg
     1201 gctagtaaca ggcagttctc tactactgtt cctgctctta attgcactgt tcgagccgtt
     1261 aataattcac ttggatttaa tatttcacat cctcaaactc tcaccggtgt tggtgcattg
     1321 gcttctaatg gtagtattga gttttttgtg tctgttgatg ctcctggtgc tttattagca
     1381 catgtcgact tcgttgttgg agcaggagga tttgaagcca ctacaggtgt atctgtatta
     1441 gttatgcaag ccaatcctca agttgtttct taattaatca atacaatttg catttctttt
     1501 catatgatta attaaattag tgtcaatctc taacgcgtcc acatctggta attcaaattc
     1561 aaagtctatt attgagagat ttaaattaag cggtggcgca cataaactat tccaatactc
     1621 acttcctctc atgcgtatcg gtattggcgg gcttatcata gaccgttgtc tcaacctttg
     1681 tgatcgtcgt cgctgctcga ccttcatctc tggtgaaagt gaagagtctt ctggtgtagg
     1741 acttggtgtt cgaggcgagg gaggcggtga acgagggtct ttttcctttg ttaaatctag
     1801 gaatctccca ccgcccattg cgatgtattc gtcccatatt gtttccacag gaacatttct
     1861 cagttgagct tcccattcta ttttgtccaa aattggcatt cttaatactg ttagaagact
     1921 taattgtaac acgcgacttg aaaagtcaaa tttcttttgt cgtgatttct atgtttacgt
     1981 ttggaaccgg ggtctaaaca acagataacc tgatgattag gttattttat ttcgcgggtt
     2041 tattttttga cagaataaaa atgagcaggt cccgaaattg gtgttttact ttgaacaact
     2101 actctgaaga tgatgttaag acatttgaaa atgttgaatg tgaatacatt atatttggtt
     2161 ttgaagtcgg ggaaagtggt actcctcacc tgcaggggta cattcgtttt aagaacgctt
     2221 gtcgtttgtc cactttgagc tctctatgcg ctcgcgctca ttatgaggtc gctaaaggcg
     2281 atgctcttag caatattaag tattgttcta agcagttcaa tgagaaacaa cttttaggtg
     2341 aagagtggag gccctttttt gaaaagggtg accgcccatt tactaaaagc gaaggtgaat
     2401 taagcatatt tggtgttggt aattttgaca tttggttttg tagccagtgt tgctagatac
     2461 gacgatgctt ataaattagc tcgtgaaggt aattttgagg atattcaaaa agacatcctc
     2521 attcgttgtt ataacagttt aaggcgtatt cattctgatt ttaagcaacc tgtgttaatg
     2581 ttacagcctg gttcaaagca tgagtggttc tatggtccca caggtagtgg gaaatctcgt
     2641 gctgctcgtg agcataatgg ttattttttg aagaatgcat ctaacaagtg gtgggatggt
     2701 tataatgctg aagaagttgt tattctggat gattttgata aaaagtttga gtataatggg
     2761 tattatttga agatttgggc ggatatttat ccatttagtg ctgaagtaaa aaacggcagt
     2821 actggatcta tccgacccaa aaagattatt gttactagca attatcatcc aaatgaaatt
     2881 tggactgatg aacaaacttt agaacctctt ttaagaagat ttaatgttac taaatataat
     2941 gttttataag tataaatgta aattgaacat tattggtaaa accagcaaat atatggttgt
     3001 ggccacggaa agagcaggat aatctacgca gtaatgcgag tatgttgtcc atccccttga
     3061 ttcaaggtat ccgtctaaca acagtagcaa tattatgcta cctaattgca ctataaaaat
     3121 taataatgga aacaggttca ttatgtatgt cacaacattc atttttttaa tatcgcctaa
     3181 attgtctttg tctgttgttt gcgtacagtc ttcttcttgc tctttgcaat acagccatac
     3241 gatgattatc cgctaatagc ctcctttgcc acggtgcaaa gagccgccgc cttatatggt
     3301 ggtgtggcgg tgttcccaaa cgaaatgaat gccggtcatc tatatgtctt gtttgggctc
     3361 ttgtatttaa aatgagaggc gagctattgc ttgaagaatt ggaactcatt ttattatggc
     3421 tgtcacgcgc gacttttggg agcaaaaagc aggtgcagct gcaagctgct taagagcggt
     3481 gggtagggac ccgccgcgta catgctattt caaacaacat caaccccctg gcggcaatag
     3541 tgtttgtttc attgcggagc cacagttgtt gactaagttc agtattacct tagtcaacag
     3601 ctgtgtcagt gtcagcccta agtaacccta aaaaatagcc tcgaaacacc ttatattatg
     3661 atgtacccag cgactaccca gtgaattttg tgacacattg gattgtgaca acttacttac
     3721 tcctgtgtca gtgtgtttta cataacaaa
//
LOCUS       CruV-347                3766 bp    DNA     circular     24-FEB-2020
DEFINITION  Crucivirus-347 isolate 120_1156_IR2_3297.
ACCESSION   CruV-347
KEYWORDS    .
SOURCE      Crucivirus-347
  ORGANISM  Crucivirus-347
            Unclassified.
REFERENCE   1  (bases 1 to 3766)
  AUTHORS   de la Higuera,I., Kasun,G.W., Torrance,E.L., Pratt,A.A.,
            Maluenda,A., Colombet,J., Bisseux,M., Ravet,V., Dayaram,A.,
            Stainton,D., Kraberger,S., Zawar-Reza,P., Goldstien,S., Briskie,J.,
            White,R., Taylor,H., Gomez,C., Ainley,D.G., Harding,J.S.,
            Fontenele,R.S., Schreck,J., Ribeiro,S.G., Oswald,S.A., Arnold,J.M.,
            Varsani,A., Enault,F. and Stedman,K.M.
  TITLE     Direct Submission
  JOURNAL   Submitted (24-FEB-2020) Biology, Portland State University, 1719 SW
            10th Ave, Portland, Oregon 97201, USA
COMMENT     Bankit Comment: ALT EMAIL:ide@pdx.edu
            Bankit Comment: TOTAL # OF SEQS:451
            Bankit Comment: GAP: est. length - alignment

            ##Assembly-Data-START##
            Assembly Method       :: MetaSPAdes v. 3.12; IDBA-UD v. 1.1.1;
                                     MEGAHIT v. 1.0.3
            Sequencing Technology :: 454; Illumina
            ##Assembly-Data-END##
FEATURES             Location/Qualifiers
     source          1..3766
                     /organism="Crucivirus-347"
                     /mol_type="genomic DNA"
                     /isolate="120_1156_IR2_3297"
                     /isolation_source="Water"
                     /country="Norway"
                     /collection_date=2011
     misc_feature    join(3752..3766,1..21)
                     /note="stem-loop"
     misc_feature    1..9
                     /note="nonanucleotide"
     CDS             95..1789
                     /codon_start=1
                     /product="Putative CruV Capsid Protein"
                     /translation="MSAVLTNFDIEQAIKKRRLSLPYQNQSDQIMGDILYAGAAPTAA
                     APKKTYKRRYKRRYGARKYKRRSYQYGRYARELRDFYAPRGSEQSINAFGADRYSANE
                     AQLKLRRATRFYGKGGYWDFLKPVGKWAARGVGALAGGGLGFEAGGLTGAVEGAQAGY
                     DRGAAFSKWAGWGAYNPAAGFGDGHSGGDVATNSLMQMTGGGPSQQISVNSDSVEGDV
                     IISYTEFVQNVTVSSLAAGSSPFTVQSFPLNPGFSTTFPWLSQLANNFTLYEMNGLIF
                     QYKPTSGDYGSTNSNALGKVIMCTNYDPDAPAFFGAQQMENYDYAVACKPSNGMYHAV
                     ETHPKQRVTNQMYVRAGSTTKDLTLCDLGLFQVATEGIYTGAAGTYVIGELWVTYSVK
                     LSRKNLNDASLGISIPVDYFTQRSLVNSAPGGTMVSQLSRIGCVISSQTSTRIRVTFP
                     TSISSGFYSINCWGCDATGTPFASSQNFTSVGAVSNCGLRSINSGAQAVQRAPNISTL
                     SVANNVINYFILVTINAPGTLQAYVELNLSATLNASSQLALIVEQVPQALYAAAIL*"
     CDS             join(2727..3048,3143..3666)
                     /codon_start=1
                     /product="Putative Replication Associated Protein"
                     /translation="MSARARNWCFTLNNYTDEEVQLVMKEHTPRNYIVFGYETGENGT
                     PHLQGYVMLKNPAKLDVMKKWIPRAHFEIARGKPQQNIDYCIKEVFNAEINGVEHVPF
                     FEEGVQTKRYDEAYELAKQGDFESIPKDILIRCYRSLKSIHSDQKKTIESLTPGVKHF
                     WFHGSTGTGKSMKAREIFSPFYLKNASNKWWDGYNGEDNVIIDDFDKKFEYNGYYLKI
                     WGDMYGFVGEVKNGNTGLIRPRNIVVTSNFHPEEIWQDKQTLEPLLRRFQVICFPVSY
                     DILKN*"
ORIGIN
        1 tagtattacc ctaaggtaag tgcttgtgtt tgtgtttcga atgccatttc cgaaaacaca
       61 aaattgtcca aacggatttt tatagacaca ttcaatgtca gctgtactta ccaattttga
      121 tatagaacaa gctataaaaa aaagacgatt gtctttgcca tatcaaaatc agtcagatca
      181 aatcatggga gacattttat atgcaggagc ggctccaaca gccgctgcgc ccaagaaaac
      241 gtacaagaga agatacaaga ggaggtacgg tgcgcgcaaa tacaaaaggc gctcgtatca
      301 atacggacgt tacgccagag agctccgcga cttctacgcc cctcgtgggt cagaacaatc
      361 aataaacgct tttggcgctg atcgatattc agctaacgaa gctcaattaa aattacgacg
      421 agccacccgg ttttacggta aaggtggtta ttgggacttt ttaaaaccag taggcaagtg
      481 ggctgcccgc ggagtaggtg cattagcagg tggtggttta ggtttcgaag ctggaggatt
      541 aaccggagct gtagaaggag cgcaagctgg ttatgaccgt ggtgcagcgt tttcaaagtg
      601 ggctggttgg ggcgcctata atcctgctgc tggatttggg gatggacaca gcggaggaga
      661 cgtggccact aatagtttaa tgcaaatgac aggtggtggt ccgtcacaac aaatatcagt
      721 taactctgat tctgtcgaag gcgacgttat catttcatat acagagtttg tgcagaacgt
      781 taccgtatct tcactagctg caggatcatc tcctttcact gttcaaagct tccctttaaa
      841 cccaggtttt tcaacaacat ttccatggct ttcgcaatta gccaataatt ttactcttta
      901 tgaaatgaat ggtttgatat tccaatacaa acctacatcc ggagattatg gttcaactaa
      961 ttcaaatgct ttaggtaaag ttattatgtg taccaattat gatccagacg cacctgcatt
     1021 ttttggtgcg caacaaatgg aaaattatga ttatgctgtt gcctgtaaac cttcaaatgg
     1081 catgtaccat gcagttgaaa cccatccaaa gcagagagtt actaatcaaa tgtatgttcg
     1141 tgctggaagc acaaccaaag atttaacttt atgtgattta ggcctcttcc aggtagcaac
     1201 agaaggtatt tatactggtg cagctggtac ctacgtaata ggtgaattgt gggttactta
     1261 ttccgtgaag ttatcccgta aaaatttgaa tgatgcttct ttaggaatct caatccccgt
     1321 tgattacttt acacaacgta gcttagttaa ctctgcacca ggaggaacta tggtttctca
     1381 attatctcgc attggatgtg taatctctag tcaaacttcc actcgtatca gagtaacgtt
     1441 ccctaccagc atcagttccg gattctactc cattaattgc tggggttgcg atgcaactgg
     1501 aactccattt gctagtagtc aaaacttcac tagtgttggt gctgttagta attgtggatt
     1561 acgttctatt aattctggtg cacaagcagt gcaacgtgct cctaatatct caactctgag
     1621 tgttgctaat aacgttatta attatttcat tttagttact ataaacgctc caggtacttt
     1681 acaagcttat gttgaactga acctcagcgc tacactcaat gcatcatccc aattagctct
     1741 aattgtagaa caagttccgc aagcgttgta cgctgcagct atactttaaa cacaattata
     1801 acaatatgcc tttttaattt ttttataatg tctcaaccca ttcaacataa aattagatat
     1861 gaacaaatac aggtgcaact ccgtcccctc actactcgta ggttgcatct tattggaagg
     1921 atggatcata tcattaaacg cgaggtagaa agcaaaacgt acagcgaata taagtattgg
     1981 aatcaacaac aaatccttgt tttcgccgag aaacaaagga tagattccat tatcaaagga
     2041 ttaaatgatg aattaaatgt atatgattca tacatgtatt gttaaaattt aaaacatttt
     2101 atttatctgt aacattaaaa acatcatcat tacttaaatt tgacaaagat gatccatccg
     2161 atgaagtatc gctaacttca gtcatatcat aatatcgtga ctgcgtttta gctaacagaa
     2221 tatcttcttc caatctctcc cgaataagct ctagttcagc aaccgcgtga agcgagtcat
     2281 acaattccca atcaaaatct ggcctagatt gtttcattgc aatcagcata tctctagctt
     2341 gttccgattc gctccgctta atggacattt agtttgtaac ttttcttctt gtatgtaaag
     2401 gtataggtaa cgcaccttgt tttttatata ttagtaaaca gttttctcgc ctacttacat
     2461 atcaacgtgt ttcccgcgct gcgagcacgc tcagcgcagc gtggtatccg tataaactat
     2521 gttttaggtt caccatgttg aataactatg ttttaggttc accatgtata ggtttagtaa
     2581 caactaacgt tagactataa gctcccccgc gctagtgggg gcgggggggg agcacagcgc
     2641 agcgagttta gcaagacccc aagaacacag gattgagatt actatgttct cgtggtcttt
     2701 tcaaatcata atttttttaa aaaatcatgt ctgcaagagc aagaaactgg tgttttacgt
     2761 taaataatta tacagatgaa gaagttcaat tggtaatgaa agagcacaca cctcgtaact
     2821 acattgtttt tggttatgaa actggtgaaa atggaactcc acatttgcaa ggttatgtaa
     2881 tgcttaaaaa ccccgcgaaa ttagacgtaa tgaaaaaatg gatacctcgt gctcattttg
     2941 aaattgctcg tggtaaacca caacagaata tagactactg tattaaggag gtttttaatg
     3001 ctgaaattaa tggcgtggaa catgttcctt tttttgaaga aggagtacgt cctaaaatca
     3061 atgatagttc aggtgcgttc gcttttttta tggataacaa caacgatttt cgtgtatgtc
     3121 atgacatatc tttatggttt agagacgaaa agatatgatg aagcttatga gcttgccaaa
     3181 caaggcgatt ttgaaagcat acctaaagat atattaattc gttgttatag atctttgaaa
     3241 agtattcact ctgaccaaaa aaaaacaatt gaaagtttaa cacctggcgt taaacatttt
     3301 tggtttcacg gttctacagg tacagggaaa tccatgaaag ctagagaaat attttctcct
     3361 ttctatctga aaaacgcttc taacaaatgg tgggatggtt ataatggaga agacaatgtt
     3421 attattgatg attttgacaa aaaatttgaa tataatggat attatttaaa aatatggggc
     3481 gacatgtatg gatttgtcgg tgaagttaag aatggaaaca ctgggctcat acgccctcgt
     3541 aatatagtgg tgacttccaa ctttcatccg gaggaaatct ggcaggataa gcagactcta
     3601 gagcccttat tgcgaagatt tcaagttata tgctttcccg tatcatatga tatattaaag
     3661 aattaatata cctcacaata ccataaagta ttattattat tataactaca accaattttt
     3721 caaaatccgt aagtacggac atcaaacaca aacttacctt aggata
//
LOCUS       CruV-348                3769 bp    DNA     circular     24-FEB-2020
DEFINITION  Crucivirus-348 isolate 120_104_SvL2_3298.
ACCESSION   CruV-348
KEYWORDS    .
SOURCE      Crucivirus-348
  ORGANISM  Crucivirus-348
            Unclassified.
REFERENCE   1  (bases 1 to 3769)
  AUTHORS   de la Higuera,I., Kasun,G.W., Torrance,E.L., Pratt,A.A.,
            Maluenda,A., Colombet,J., Bisseux,M., Ravet,V., Dayaram,A.,
            Stainton,D., Kraberger,S., Zawar-Reza,P., Goldstien,S., Briskie,J.,
            White,R., Taylor,H., Gomez,C., Ainley,D.G., Harding,J.S.,
            Fontenele,R.S., Schreck,J., Ribeiro,S.G., Oswald,S.A., Arnold,J.M.,
            Varsani,A., Enault,F. and Stedman,K.M.
  TITLE     Direct Submission
  JOURNAL   Submitted (24-FEB-2020) Biology, Portland State University, 1719 SW
            10th Ave, Portland, Oregon 97201, USA
COMMENT     Bankit Comment: ALT EMAIL:ide@pdx.edu
            Bankit Comment: TOTAL # OF SEQS:451
            Bankit Comment: GAP: est. length - alignment

            ##Assembly-Data-START##
            Assembly Method       :: MetaSPAdes v. 3.12; IDBA-UD v. 1.1.1;
                                     MEGAHIT v. 1.0.3
            Sequencing Technology :: 454; Illumina
            ##Assembly-Data-END##
FEATURES             Location/Qualifiers
     source          1..3769
                     /organism="Crucivirus-348"
                     /mol_type="genomic DNA"
                     /isolate="120_104_SvL2_3298"
                     /isolation_source="Water"
                     /country="Norway"
                     /collection_date=2012
     misc_feature    join(3757..3769,1..18)
                     /note="stem-loop"
     misc_feature    1..9
                     /note="nonanucleotide"
     CDS             complement(304..1956)
                     /codon_start=1
                     /product="Putative Replication Associated Protein"
                     /translation="MFSQKCDQKFQINEYSHRYSDWREDPNMDLINSSEDLDDFCKFY
                     NGRISRSMVNEEIQRSKDDKCIKKLAFTHHTLQDEVDAKNGAPFPKPLRIPGTYGKSS
                     RVEGCKCINACFNPEDCPMVRAAKKKPGIRKIKVKSTPPVTPVSVEPEEEEDKCTYSK
                     DKSYRGYAITDFEMDVNFWEDQWNTDKYTYLLVCEETCPKTGELHWQTFVYYTNKSKY
                     SACLKRFKPRHIGAIYATPSDNARYCKKGDQSKAEYREFGSKGPNYGLNCIIIFEQGE
                     CPQQGARTDLETIYEQIKKGEIRTKTGLANANISIACQYGKPLMELVEEAQESRSWKT
                     EVWAIIGKGGTGKSRLARKLDPDVKAIKFTGDKKCPFILGYKGHPEVVMIEEFDPDCC
                     TQEFWLEACDRYEMPVNIKGSETTWNPRKIYFTSNKDPSTWWGGWNRQTERRFTQILE
                     CDVDVEPEDLHPVRRKILMNKDGVILKDDVIPWKPEEEPKEVSLEESLAAGVQTKDGY
                     TFTAKAVGEKVDANALTGHDRIAWEEAKRAEAEYKKSLVRLK*"
     CDS             2061..3566
                     /codon_start=1
                     /product="Putative CruV Capsid Protein"
                     /translation="MPAPKRVIRRAPAKKAAPKRIIRRANPGASVQRAMLRDLGPRRR
                     AAAPKRSSGGSSLGTAIGSAIGLALGGPGGAAAGGMIGSAGGNFLSKIFGHGDYQVSN
                     SNMIKSNNLISNSANIPQFGTGKVATNFRHREFLGDVISSSTANTFKIDSFSINPGVD
                     ATFPWLAGVVGAKYQQYRINGMTFEFRSMSADALNSTNTALGSVIMSTDYDSADTAFT
                     SKQQMENTEYGVSCKPSVNMMHGIECERSQTPVSELYIRAFDVPSGKDVRFYDIGRFS
                     IASTGCQGTNVNLGELWVSYDIDCFKAIEQVPGFLIPFSQYALVGTLPASAPLGTSQS
                     QLLTQSGNEVAMTFTANKMSWPLSIDVGSIWHVQINWLRTSSALTAPVITSGNGMFFN
                     TNTVWKTITGTTCTDIRVDVDVQYQGGANAANLPYLNIANFTHAGANQQSDLYITQMS
                     GLWPGTYTSQTQSLPPEDEEETSPEYIEEMEQKKPQQTISKGMSKMRIGFL*"
ORIGIN
        1 tagtattact tgaggcactt aaggttttaa atttaaccgg ttgtttaccg gttaaaataa
       61 aacattcagt cgtcttaaaa gttctccaca gaacttttat aggaaaatag aatagtaaag
      121 tcatagcaac tagtagctat gctttcactc ttttcttttc aaaatttctc cacaggatag
      181 attttttaac ttttccatat aacaatatag aaaatttttt tatttttcgt taactaacta
      241 aacataaact gctcccccgc tggcgggggc cggggggtga cctatatact tatcgtttat
      301 aagttatttg agtctaacaa gagacttctt gtattcggct tcagcacgct ttgcttcttc
      361 ccaagcaata cgatcatgac ctgtcaaggc attagcgtct accttttctc ccactgcttt
      421 agctgtaaaa gtgtagccat ctttagtctg cactcctgca gccaacgatt cttcgagaga
      481 tacttctttc ggttcttcct ctggtttcca aggaataaca tcgtctttga ggatgacacc
      541 atccttattc attaagatct tgcgtcttac tggatgtagg tcctctggtt ctacatcgac
      601 atcacattct agaatttgtg taaatcgacg ttccgtctgt cgattccaac cgccccacca
      661 ggttgatgga tctttgttcg aagtaaaata aattttgcga ggattccatg tagtttcact
      721 acccttgata ttaactggca tttcatatcg atcgcaagct tctaaccaga actcttgcgt
      781 acaacaatca ggatcgaact cttctatcat gaccacttct ggatgtccct tgtatccaag
      841 gataaaaggg cattttttgt cacctgtaaa tttgatagct tttacatcgg gatctagttt
      901 gcgtgcaagt cttgatttac ctgtcccgcc tttaccgatg atggcccata cttcggtttt
      961 ccaagaacgc gattcctgag cttcttcaac taattccatt agtggctttc catactggca
     1021 agctatagag atattagcat tagctaaacc agtcttggtt cttatctcac ccttttttat
     1081 ctgttcataa attgtttcga ggtcggttct cgcaccttgc tggggacact ccccctgctc
     1141 gaatatgatt atgcagttca aaccataatt tggtcccttt gatccaaatt ctcggtattc
     1201 agctttagac tgatctccct ttttacaata tctcgcattg tcagaaggag ttgcataaat
     1261 agcacctatg tgtctaggtt taaaccgttt cagacacgcg ctatatttgc ttttgttcgt
     1321 atagtacacg aacgtctgcc aatgcagttc acctgtcttt gggcaagttt cttcgcaaac
     1381 taacaggtaa gtatacttat ctgtattcca ctgatcttcc cagaaattga catccatttc
     1441 gaagtctgtg atagcgtatc ctctgtaaga tttatcttta ctataggtac acttgtcttc
     1501 ttcctcttcc ggttcaactg aaaccggagt aacaggaggt gtagacttaa ccttgatctt
     1561 tctgattcca ggtttcttct ttgctgctct caccataggg cagtcctctg gattgaaaca
     1621 tgcgtttatg catttacaac cttctactct agaacttttc ccataagttc ctggtatcct
     1681 taaaggtttc ggaaaaggtg caccgttttt cgcatcgact tcatcttgaa gcgtatggtg
     1741 agtaaaggca agtttcttga tacacttgtc gtctttactt ctttgtatct cttcattgac
     1801 cattgaacgg gatattctac cattgtagaa cttgcaaaaa tcatctaggt cttcagagct
     1861 attgattaga tccatatttg gatcctctct ccaatcagaa tatctatgag agtattcgtt
     1921 aatttggaac ttctggtcac acttctggct gaacatctgg ttttatttac aacggaagtt
     1981 ttaaaacaat cacttttggt tttttttcgt taattttaaa ttaaaatttg atattaaaaa
     2041 caattttaaa ggcataataa atgcctgccc ccaagcgagt catacgccga gccccagcca
     2101 aaaaggctgc ccccaaacgc ataatacgaa gagccaaccc aggcgcttca gtacagcgtg
     2161 caatgctgag agatctaggc ccacgccgaa gagctgcagc acctaaaaga tcctcaggtg
     2221 gatcttctct cggaactgca atcggttccg ccatcggtct tgcactcgga ggccccggag
     2281 gtgcagcagc cggtggtatg ataggtagtg ctggaggcaa cttcttatct aaaatctttg
     2341 gacatggtga ctatcaggtc tcgaattcta atatgattaa gtcaaacaac ttaattagta
     2401 attcagccaa cattccgcaa tttggcactg gcaaagtagc tactaacttt agacatcgag
     2461 aattccttgg agatgttatt agctcatcca cagctaatac ctttaagatt gatagcttct
     2521 ctatcaatcc aggtgttgat gcaactttcc cttggctcgc aggagtcgtt ggggcaaagt
     2581 atcaacagta ccgtatcaac ggtatgacat ttgaatttag aagtatgtca gcagacgcac
     2641 ttaattcaac aaacactgct ctaggttcag taatcatgtc aacagactat gattcagctg
     2701 acaccgcttt tacatctaaa caacagatgg aaaatacaga gtacggagta tcctgtaagc
     2761 cctcagtgaa catgatgcac ggcattgaat gtgaacgctc tcagacacct gtttctgagt
     2821 tatatatcag agcctttgac gtcccttcag gcaaagatgt caggttctat gatataggta
     2881 gattctcaat agcatctacc ggttgccaag gtactaatgt aaaccttgga gagttatggg
     2941 tatcttatga tatcgattgc tttaaagcca tcgaacaagt acctgggttc ttgatcccat
     3001 tctcacaata tgccctagta ggaacactac cggcttccgc cccactaggt acttcacaat
     3061 cacaattgtt aactcaatct ggaaacgagg tagcgatgac tttcacagct aataaaatga
     3121 gttggcccct atctatcgat gtagggagta tctggcatgt ccagattaac tggttaagaa
     3181 catcttcagc tctaaccgcc ccagtcatta cttcaggtaa tggcatgttt ttcaacacca
     3241 atacagtgtg gaaaactatt acaggtacta cctgtacaga tattagagtc gatgtagacg
     3301 ttcaatatca aggtggtgct aatgcagcta accttcctta tttaaatatc gctaatttta
     3361 cacatgcagg tgcaaatcag caatcagatc tttatattac ccaaatgtct ggtttatggc
     3421 ctggtacata cacatcacag acccaatctc ttcctccaga ggatgaagaa gagacatctc
     3481 cagaatacat tgaagagatg gaacaaaaaa aacctcagca gactatctcg aaagggatgt
     3541 ccaagatgag gattggtttt ctctagagta agccaatata ttaatctact acaccaaata
     3601 gtagattatt ctttctcgtt ctcgtcttac actatttggt actacaccat atatgaaaaa
     3661 gcgcaaaaga acgccaaggc ccccagttgg ggccgcggag aggacgtgat tctacgaata
     3721 agacatcgtt tcgttgtccg tccgttaatc tcccaggtgc ctcaattat
//
LOCUS       CruV-349                3770 bp    DNA     circular     24-FEB-2020
DEFINITION  Crucivirus-349 isolate H2_26680.
ACCESSION   CruV-349
KEYWORDS    .
SOURCE      Crucivirus-349
  ORGANISM  Crucivirus-349
            Unclassified.
REFERENCE   1  (bases 1 to 3770)
  AUTHORS   de la Higuera,I., Kasun,G.W., Torrance,E.L., Pratt,A.A.,
            Maluenda,A., Colombet,J., Bisseux,M., Ravet,V., Dayaram,A.,
            Stainton,D., Kraberger,S., Zawar-Reza,P., Goldstien,S., Briskie,J.,
            White,R., Taylor,H., Gomez,C., Ainley,D.G., Harding,J.S.,
            Fontenele,R.S., Schreck,J., Ribeiro,S.G., Oswald,S.A., Arnold,J.M.,
            Varsani,A., Enault,F. and Stedman,K.M.
  TITLE     Direct Submission
  JOURNAL   Submitted (24-FEB-2020) Biology, Portland State University, 1719 SW
            10th Ave, Portland, Oregon 97201, USA
COMMENT     Bankit Comment: ALT EMAIL:ide@pdx.edu
            Bankit Comment: TOTAL # OF SEQS:451
            Bankit Comment: GAP: est. length - alignment

            ##Assembly-Data-START##
            Assembly Method       :: MetaSPAdes v. 3.12; IDBA-UD v. 1.1.1;
                                     MEGAHIT v. 1.0.3
            Sequencing Technology :: 454; Illumina
            ##Assembly-Data-END##
FEATURES             Location/Qualifiers
     source          1..3770
                     /organism="Crucivirus-349"
                     /mol_type="genomic DNA"
                     /isolate="H2_26680"
                     /isolation_source="Water"
                     /country="New Zealand"
                     /collection_date=2012
     misc_feature    join(3757..3770,1..19)
                     /note="stem-loop"
     misc_feature    1..9
                     /note="nonanucleotide"
     CDS             309..1604
                     /codon_start=1
                     /product="Putative CruV Capsid Protein"
                     /translation="MPGRYVRKYPARRASSSRAVVYRGKGAYRRSAPARRYSRRVRYV
                     RPVKAPQQSSGSWLSDVGSKAGSALGSIVGKFLGLGEYKMARNSLINGSSPNQVPFMH
                     SAKDGVRIRHREFIADITTQAAFTNTSYSVNPGLSLSFPWLSALAQNFEEYRFEGLVF
                     EYVPMCGDAVSSTNSTMGVVVMAAEYNPTQAAYVNKQQMENSMWAMSDKPSKPIALPI
                     ECAPSLNPLSNQYIRIGAVASGQDVRFYDLCNIQVATVGAQASFKAGELWATYDIVLM
                     KPQLDSGLALSAEIAHYTLVAPSESGSAWFGTSRSAYYDNIGLTVAGASITFPLGSQG
                     TYHISYSQNGASTVLTNINTPTYTNCAQVTSYLNNNTLSAIRNGGTTSSIYFNSSYVY
                     IADPTLQAVVTFSGGTLCTTPTYGDLIVTQVNDNFRLNP*"
     CDS             1632..2033
                     /codon_start=1
                     /product="putative ORF"
                     /translation="MSAFYLLNTPSNGVYFGSSRTMSFDSIGLTVSTDTITFPLGLWG
                     YYYVNWVVYGGSTTVIAPSVTYTNCNTVSRFNNFSVNYITNSGATDSKLIYCFQIYIP
                     SPVSQASVGFSSGLMSISTTTGDLFVTEYDP*"
     CDS             2060..2572
                     /codon_start=1
                     /product="putative ORF"
                     /translation="MLKNYFFVCILLNMSDPVCCSDPVCCSECVKLRDDVNKIIHVVS
                     VMDKKFDEMFERIQVLEEIIDESHIDESDDEDNCRRLVSSPVQITCPDESEDEKSPAS
                     TALEWKDYSQSVEKLFKVDPPKNGVKYTAIEHDTFLKNGYDQRVGFCWLNFKNDEGAS
                     WWEVGHRRSG*"
     CDS             complement(2755..3735)
                     /codon_start=1
                     /product="Putative Replication Associated Protein"
                     /translation="MSIRKTIITSYLFQEREFEANLKTKEFAQVKFAVIQEELCPKTN
                     KMHHQGYLEFNKPVTLAFIKKLTGDNTTHVVTQKMKGTPLQGAEYCGYNNGGETEKNK
                     EKAIERIKNNWYYFEHGKLEWQEQGKRNDINEVVDMIHEGKQLKEIVEEYTSTYIRNY
                     KGIERAMQIMGKPLTTPKIEEKQVFLNYGESGTGKTELIYNSHPIHEIFRKPPGQWWD
                     GYCGQKYILIDDIQPNEYKIEEFLQWLDKYPISVPIKGSFVPLTCTHIYLTSNFDPNK
                     WFNYKSIESYKAIIRRITKIKYHEKEPLYDILKREMETEWVWEVAQGTAV*"
ORIGIN
        1 tagtattacc tatagctacc tctgtatttt ttttgaaaaa aaaaataaaa aattggattt
       61 atcctaattt ttcggtttat tcgctgaggt tacgcgaaag agaatttttt tttttataaa
      121 ataaataatt atcggtcccg aagggattcg caatcataaa atgtcttaga atgcaaaata
      181 taattttgag gaaaaataaa tatttatttg ttccgttgca tttttttagt taattaatta
      241 tttatttatt ttttaaaaat aatataatac ttaatttata tctttgatag aaaatatttt
      301 tggaaaaaat gcctggtcgt tatgttcgta agtatccagc tagacgagct tcaagctctc
      361 gagctgttgt ttatagaggt aagggtgctt accgtaggtc tgctcctgct agacgttatt
      421 ctcgtagagt tcggtatgta agacctgtta aggctcctca acaatcttca ggttcttggc
      481 tttctgacgt aggaagtaaa gcaggttctg ctttgggtag tattgttggt aaatttttgg
      541 gattaggtga atataagatg gctcgtaatt ctttaattaa cggatcatct cctaatcaag
      601 tgccgtttat gcattctgcg aaggatggtg taagaattag acatcgtgag tttattgcag
      661 atattacaac tcaagccgcg tttactaata ctagttattc agtaaatccc ggattatctt
      721 tgtcttttcc ttggttgtct gctttagctc aaaacttcga agagtatcga tttgaaggtt
      781 tagtttttga gtatgttcct atgtgtggtg atgctgtatc atcaactaat tcaacaatgg
      841 gtgttgttgt aatggctgct gagtataatc caacgcaagc ggcttatgtt aataagcaac
      901 agatggagaa ttcaatgtgg gcgatgtctg ataagcctag taagccaatt gctttaccta
      961 ttgaatgtgc tccttctttg aatcctttgt ctaatcaata tattcgtatt ggtgctgttg
     1021 ctagtggtca agatgttaga ttttatgatt tgtgtaatat tcaagttgca acggtaggcg
     1081 ctcaagcgtc ttttaaagct ggtgagcttt gggcaactta tgatattgtt ttaatgaaac
     1141 ctcaattaga ttctggatta gcgttatctg ctgagattgc tcattatact ttagtagcac
     1201 cctcagaatc tggttctgca tggtttggaa catcacgtag tgcatattat gataatattg
     1261 ggttaacggt tgctggagct agcattacgt tccctcttgg ttctcaagga acttatcata
     1321 tttcatatag tcaaaatgga gcatctactg ttttgactaa tattaatact cctacgtata
     1381 caaactgcgc tcaagtcaca tcatacttga ataataacac attgagtgcc attcgtaatg
     1441 gtggaactac tagttctatt tattttaatt caagttatgt gtatattgct gatccaacac
     1501 ttcaagctgt tgttacgttt tctggtggca cattgtgcac tactcctacc tatggagatc
     1561 ttattgttac gcaagttaat gataatttta gattgaatcc ttaggacagt gatccttttt
     1621 gtagcagttc tatgtctgcc ttttatcttc ttaatacccc ttctaatggt gtttatttcg
     1681 gtagtagtcg aaccatgtct tttgatagta ttggtttgac ggtatctact gatactatta
     1741 catttccttt aggattatgg ggttattatt atgttaattg ggttgtttat ggtggatcta
     1801 ctactgttat tgctccttct gttacttata caaattgtaa cactgtttct cgatttaata
     1861 atttttctgt aaattatatt actaattcag gagcaactga tagtaagttg atatattgtt
     1921 ttcagattta tattcctagt cctgttagcc aagcttctgt tggtttttca tctggtttga
     1981 tgtctatatc aactactact ggtgatcttt ttgttactga atatgatcct taaaggttag
     2041 aactaattat ttaattattt tgttaaaaaa ttattttttt gtatgtatat tattaaatat
     2101 gtctgatcct gtttgttgtt ctgatcctgt ttgttgttct gaatgtgtta aacttcgtga
     2161 tgatgttaat aagatcattc acgtagtttc tgttatggat aaaaaatttg atgagatgtt
     2221 tgagcgaatt caagttttag aggagattat tgatgaatct catattgatg agtctgatga
     2281 cgaagataat tgcagacgtt tagtttcatc tcctgttcag attacttgtc ctgatgaatc
     2341 agaggatgaa aagagtcctg catctactgc attggaatgg aaagattaca gtcaatcagt
     2401 agagaagtta tttaaggttg atcctccaaa gaatggagtt aaatatactg caattgagca
     2461 tgatactttt cttaaaaatg gttatgatca aagagttgga ttttgttggt taaattttaa
     2521 aaatgatgaa ggtgccagtt ggtgggaagt tggtcataga cgttctggtt aattatttta
     2581 atcagtattt tttttaaatt ttgattttag ctgtatcacg cgcgacctgc agttttttgt
     2641 ataaaaatga acaaaaaaaa cataacttat cccattaggg tgcgtagcgt agctacgcat
     2701 ctgaaagaat aattattttt ttaatttttt tatacgtgga actgcatacg tgagttaaac
     2761 agcggtccct tgggcaactt cccacaccca ttctgtttcc atttctcttt ttaaaatatc
     2821 atataatggt tctttttcgt gatattttat ttttgttatt cttcttatta ttgctttgta
     2881 actttctata cttttgtagt tgaaccattt gtttgggtcg aagttacttg ttaaatatat
     2941 gtgtgtgcat gttagtggaa cgaatgatcc ttttattggc acacttatag ggtatttatc
     3001 aagccattgt aagaattctt caattttgta ttcatttggt tgtatatcat caattagaat
     3061 atatttttgt ccacaatatc cgtcccacca ttgtcccggt ggttttctaa atatttcgtg
     3121 aattggatgt gaattatata ttaattcagt ttttcctgtt cctgattcac cgtagtttaa
     3181 gaaaacttgt ttttcttcaa ttttaggtgt tgttaatggt ttacccatta tttgcattgc
     3241 tctttcaatg cctttgtagt ttcttatgta ggttgatgta tattcttcaa ctatttcttt
     3301 tagttgtttt ccttcgtgaa tcatatcaac tacttcattt atgtcgtttc ttttgccttg
     3361 ttcttgccat tctaattttc cgtgttcaaa ataataccaa ttatttttta ttctttctat
     3421 tgctttttct ttgttttttt ctgtttctcc tccgttgttg taaccacaat attcagctcc
     3481 ttgtaaaggt gttcctttca ttttttgtgt tacaacatgt gttgtattgt ctccagttaa
     3541 ttttttaata aatgctaatg ttactggttt gttaaattca aggtatcctt gatgatgcat
     3601 tttatttgtt tttggacata attcttcttg aattactgcg aatttaactt gagcaaattc
     3661 ttttgttttt aaatttgctt caaattctct ttcttgaaaa agatatgaag ttataattgt
     3721 ttttctaatt gacatcttta tttatttttt tttattgtag ctatagtttt
//
LOCUS       CruV-350                3777 bp    DNA     circular     24-FEB-2020
DEFINITION  Crucivirus-350 isolate H_268.
ACCESSION   CruV-350
KEYWORDS    .
SOURCE      Crucivirus-350
  ORGANISM  Crucivirus-350
            Unclassified.
REFERENCE   1  (bases 1 to 3777)
  AUTHORS   de la Higuera,I., Kasun,G.W., Torrance,E.L., Pratt,A.A.,
            Maluenda,A., Colombet,J., Bisseux,M., Ravet,V., Dayaram,A.,
            Stainton,D., Kraberger,S., Zawar-Reza,P., Goldstien,S., Briskie,J.,
            White,R., Taylor,H., Gomez,C., Ainley,D.G., Harding,J.S.,
            Fontenele,R.S., Schreck,J., Ribeiro,S.G., Oswald,S.A., Arnold,J.M.,
            Varsani,A., Enault,F. and Stedman,K.M.
  TITLE     Direct Submission
  JOURNAL   Submitted (24-FEB-2020) Biology, Portland State University, 1719 SW
            10th Ave, Portland, Oregon 97201, USA
COMMENT     Bankit Comment: ALT EMAIL:ide@pdx.edu
            Bankit Comment: TOTAL # OF SEQS:451
            Bankit Comment: GAP: est. length - alignment

            ##Assembly-Data-START##
            Assembly Method       :: MetaSPAdes v. 3.12; IDBA-UD v. 1.1.1;
                                     MEGAHIT v. 1.0.3
            Sequencing Technology :: 454; Illumina
            ##Assembly-Data-END##
FEATURES             Location/Qualifiers
     source          1..3777
                     /organism="Crucivirus-350"
                     /mol_type="genomic DNA"
                     /isolate="H_268"
                     /isolation_source="Water"
                     /country="New Zealand"
                     /collection_date=2012
     CDS             1..1413
                     /codon_start=1
                     /product="Putative Replication Associated Protein"
                     /translation="MFLITTNSPSLMEGQEKCHAIYSGLRKGHMDFLNKARSCRWSWT
                     LFNYTEEMIEGVATAFKTNEKLSYIVWGREVCPDTGRKHLQGYLEMKIKSGFRTVLKH
                     AGLPQNTFICVSKGNAKENRAYCTKDGNQVVELGKARDGQGHRNDLSDVKEMVKQGCS
                     LEEIADVATSYQGLRSINTYRAIYDKPRDFETKVVWLWGDTGTGKTLFARLITDNAWI
                     SAANGIEWFDGYYNNAYAILDDIRAESGRIEKFLRLFDRTGCPVPIKGSTVQWKPRLI
                     VVTGPFHPQVLFRKGAPCFEENVQQLLRRCDYVIHADKWNTDVLVDIMREKEIAESES
                     SDECPFVWDPLSVEEIYEMMDISIDDKSIERPKLALSKLSSPNVDGTRKTHYLASRSR
                     KTSNVSGCTSASSFVCNSAKPFSFEKWPKVGGNIPRASPLGRSPPETDPRPGFGKEEM
                     EESNAVLGSALARAAERLSH*"
     CDS             complement(2053..3663)
                     /codon_start=1
                     /product="Putative CruV Capsid Protein"
                     /translation="MPYGRKSGPYRAYVRGRGAYTVGRGIAALRPSVRKYFSKPSRAA
                     RVARPQSEKLFDAVALGASIGSRIGRTAGSAIAPGIGTVAGSTFGSVAGSAAGRGFQR
                     ITGWGDYKVGSKKGGTDFSVPVFGNDAIRIRKREFIATLNCTTDFQNNIFPIQPGDET
                     TFPWLSAIANCYEQYRFNGCIFSYVSLTSDAIASSTQLGLGQVIAATDYNASELPFLN
                     EIEMLGNTFAVSGKPSNDLMHAVECAPKQTAQKVYYTRTGAVPYGSDPRLYDVGTFQI
                     ATVGAQANYTGAGSLYVSYDITFMKSKMINQLGLAVNTDHFAFGEVDATADDYFSTIA
                     KQNHILPGSNLGCTLEENNIIFPSTLQTGYYMITYCVWGDGPAADATVETDSNPEVKG
                     FVSIKSPSSAKKPSTLISPGPVAAPDINCNNCELLAAFPRPSTVAAQTSGYLWNDGQY
                     ASTSTSGNLQTLVMSFIVRIVARDASITFSGGILPLNDGGLSSGDLIIQQVNGGIYIN
                     CEVETSATMTKKQPKNRIRTRKPFGVFT*"
ORIGIN
        1 atgtttttga tcactacaaa ctcgccatcg ctcatggaag gtcaagagaa gtgtcatgcg
       61 atatacagcg gtctgaggaa ggggcatatg gatttcttga acaaagctcg tagttgcaga
      121 tggtcgtgga ctctgttcaa ctacactgaa gaaatgattg aaggtgttgc taccgcgttc
      181 aagactaacg agaaactctc gtatatagtg tggggtagag aagtgtgtcc tgacactggc
      241 aggaagcact tacaaggata tctcgaaatg aagatcaaat cggggtttag aactgtattg
      301 aagcatgctg gactgcctca aaatacgttc atatgtgtgt cgaagggtaa cgctaaagag
      361 aatcgtgcgt actgtactaa agacggcaat caagtagttg agcttggtaa agctcgcgac
      421 ggacagggcc atcgcaacga cttgagtgac gtgaaagaga tggtaaagca agggtgctct
      481 cttgaggaga tcgctgatgt agcgacttcc tatcaagggt taagaagtat aaatacgtat
      541 cgtgctatct acgataagcc gagagacttt gagaccaaag ttgtgtggct ctggggagac
      601 actggtacag gtaaaacctt gttcgcgaga cttatcactg ataatgcctg gatcagtgcc
      661 gcgaatggga tcgaatggtt cgatggttat tataataatg cttatgccat acttgatgac
      721 ataagagctg agtctggtcg tatagagaag ttcttacgac tgtttgacag gactggttgt
      781 cctgttccta ttaaaggcag cacggtacaa tggaagcctc gactgatcgt cgtaactggt
      841 ccctttcatc cgcaagtgct gtttagaaag ggggctccgt gctttgagga gaacgtgcag
      901 cagctacttc gtcgttgtga ctacgtgata catgctgaca agtggaatac tgatgtgctg
      961 gttgacatca tgcgtgaaaa agagattgct gaaagcgaat cctctgacga gtgcccattt
     1021 gtatgggacc cactgtcggt tgaagagatc tatgagatga tggatatctc aatagacgat
     1081 aagagtatcg aacgtcctaa gcttgcgctc agtaagttgt cctcgcccaa tgttgatggg
     1141 acgagaaaga ctcactatct ggcaagcaga tctcgtaaga cttcgaacgt gagtggttgc
     1201 acaagtgcaa gttcctttgt ttgcaattct gcaaaaccat tttcttttga aaagtggcca
     1261 aaggtcgggg gtaatatccc aagggcttcg ccgcttggga ggtctcctcc ggagacggac
     1321 ccccgacctg gctttgggaa ggaggaaatg gaagagagta acgctgtgtt gggcagcgca
     1381 cttgcgcgtg ctgccgagcg tctctcacat tagacgaatg cgagtgtatt gctttttcgg
     1441 aagcaaatgt ttacatggga tattgtacgt gcgcttactc gtatgcgcta actcgtgtgc
     1501 gctaactcgt gtctgttata gtctgtgaca taacacggta cgatgtggtc acttgatacc
     1561 tctgacccca taccccccat gtttgtgctc ggtcgacatc ttcccgcact gtgcgcatca
     1621 agcactcggt cgagcaacta gtaagcgaca taccatgtgt atgctcctat tatgtgcgta
     1681 actaaagtgt atcactcgcg ttgatgcata cgaagacaat cgttcgacgc attggtgcgg
     1741 cgttgcggaa cgggtggggc gtgggtcccg tatatatccc acttccgtcc ttatccgcgt
     1801 cctagccacc aatgcactga acgattgcca tcgtatgaat cgacgtgaat gataagcttt
     1861 agtcacgtac ataataggag catacccatg gtgtgtctct tattagttgt tcgaccgagt
     1921 acttgatacg catgcgtgcg tgtcgagtct cggtcgccca gttcggcatg tccttcgtcc
     1981 cgcccaacga ttgctttgcg aaaaagtaag tatactcttt ttgtactaat gcatgtgtat
     2041 gtctatgtgt gtctaagtga agactccgaa aggctttcgt gttctaatcc tattcttagg
     2101 ttgctttttg gtcatagtgg cggatgtctc aacttcacag ttgatataga tacctccatt
     2161 gacttgttga ataatgagat ctccggaact caatccacca tcgttaagtg gaaggatacc
     2221 gcctgagaat gtgatacttg cgtcgcgagc gacgatgcgg acaatgaaag acataactaa
     2281 tgtctgtaga ttgccggaag tgctagtact agcgtactgc ccatcgttcc aaagatagcc
     2341 agatgtctgt gctgctactg ttgaagggcg tgggaatgcg gctaacaact cacagttgtt
     2401 acaattgatg tcgggtgctg caactggtcc tggagaaata agcgtagatg gcttcttggc
     2461 tgaagaaggc gacttgatcg agacgaagcc cttgacttcg ggattcgagt cagtctcaac
     2521 agtggcatcc gctgcaggac catcgcccca aacacaatac gtaatcatgt aatatcccgt
     2581 ctgaagagta gaggggaaga tgatattgtt ctcttctaag gtacaaccaa ggttgctacc
     2641 tgggagaata tggttctgtt tagcaatagt gctaaagtag tcgtctgctg tggcatccac
     2701 ttcaccaaag gcgaagtgat cagtattgac ggcaagtcca agttgattga tcatctttga
     2761 tttcatgaag gtgatatcat aactaacata gagactgcct gctcctgtgt agttcgcctg
     2821 tgctcctact gttgcaatct gaaatgtgcc aacatcatac aaacgtggat cggatccgta
     2881 tgggaccgct ccagtgcgag tgtaatacac tttttgtgct gtctgcttag gtgcacactc
     2941 aaccgcgtgc ataagatcat tcgacggctt gcctgaaacc gcgaatgtgt tgccaagcat
     3001 ctcgatctcg ttcaagaacg ggagttcgga tgcgttgtag tctgtggctg caataacttg
     3061 cccaaggcca agttgtgtgc tacttgcgat ggcatctgat gtcaatgaga catatgagaa
     3121 gatgcatccg ttgaagcgat actgctcgta gcagttcgcg atggctgaca accaaggaaa
     3181 ggttgtctca tctcctggtt ggatagggaa gatgttgttc tggaagtcgg tggtgcaatt
     3241 gagagtcgcg ataaactctc gctttctaat cctaattgca tcgtttccga agactgggac
     3301 actgaagtcg gttcctccct tcttgctgcc aactttgtaa tccccccatc cggtaatgcg
     3361 ttgaaatccg cgtccggcag ctgatccggc gacactgccg aaagttgatc cagcaactgt
     3421 cccaatacct ggagcaatgg cgctccctgc tgtacggcca atcctgctac cgatcgaagc
     3481 tccgagtgcg actgcgtcga atagcttctc gctttgcggt cttgcaactc tagctgctct
     3541 gctaggttta ctaaagtact ttcgtactga tgggcgcaat gctgcaattc ctctaccaac
     3601 tgtataagct cctcttcctc gaacgtaagc tcggtaaggt cctgactttc ttccataagg
     3661 catgtgtgtg cgtgtgcgtg ttgagcgcgg gtttgagcaa cttaaaaagt tgactatttt
     3721 ttttgaaaag agatcaaatg agatttgagt ccttttgata gtgtgtgata tgaaact
//
LOCUS       CruV-351                3786 bp    DNA     circular     24-FEB-2020
DEFINITION  Crucivirus-351 isolate 120_763_SvL1_3295.
ACCESSION   CruV-351
KEYWORDS    .
SOURCE      Crucivirus-351
  ORGANISM  Crucivirus-351
            Unclassified.
REFERENCE   1  (bases 1 to 3786)
  AUTHORS   de la Higuera,I., Kasun,G.W., Torrance,E.L., Pratt,A.A.,
            Maluenda,A., Colombet,J., Bisseux,M., Ravet,V., Dayaram,A.,
            Stainton,D., Kraberger,S., Zawar-Reza,P., Goldstien,S., Briskie,J.,
            White,R., Taylor,H., Gomez,C., Ainley,D.G., Harding,J.S.,
            Fontenele,R.S., Schreck,J., Ribeiro,S.G., Oswald,S.A., Arnold,J.M.,
            Varsani,A., Enault,F. and Stedman,K.M.
  TITLE     Direct Submission
  JOURNAL   Submitted (24-FEB-2020) Biology, Portland State University, 1719 SW
            10th Ave, Portland, Oregon 97201, USA
COMMENT     Bankit Comment: ALT EMAIL:ide@pdx.edu
            Bankit Comment: TOTAL # OF SEQS:451
            Bankit Comment: GAP: est. length - alignment

            ##Assembly-Data-START##
            Assembly Method       :: MetaSPAdes v. 3.12; IDBA-UD v. 1.1.1;
                                     MEGAHIT v. 1.0.3
            Sequencing Technology :: 454; Illumina
            ##Assembly-Data-END##
FEATURES             Location/Qualifiers
     source          1..3786
                     /organism="Crucivirus-351"
                     /mol_type="genomic DNA"
                     /isolate="120_763_SvL1_3295"
                     /isolation_source="Water"
                     /country="Norway"
                     /collection_date=2012
     CDS             1..1347
                     /codon_start=1
                     /product="Putative Replication Associated Protein"
                     /translation="MMKELTGLFNSKKLDKLLGKFYKEKSSVNFIDEKIDEEINTDNI
                     KKVRFLDEIEIKVIENCLAELDNGEKDDGSFRIQKMMKCMLTYPDHLPKEEYIKWFED
                     KIGKCKFIRIAHETGKSGEHDYEHSHVLIELEKPFSSTKKNIFHFKDFRHPQFKYFKM
                     SNMLHWKNSVRYLGKEDPDNKDLLTVHTNFVDEIWSKNKEDALQSCQRPADAMGILTL
                     WKNKPMVMKPPKYLKELGGETCPHNFMRWQHNIHHMMKFNTQGNRKITWIYDAIGGKG
                     KSCFADDLVAAKIAYKVKSNGGGYIHCNEMIGEAYESGLWNGENIIFDWPRQTDFIDR
                     KGLYGLIEAIKDGTLSRTKYIGKTINIQLDGNKNTQVFIFANFLPQYKDEHGHLTLSL
                     DRWDVHEIGKHGGFNSRFHKTQNSIIKNIRNEFEKNQDEHTEDEEESWFDDTKIYE*"
     CDS             complement(1985..3688)
                     /codon_start=1
                     /product="Putative CruV Capsid Protein"
                     /translation="MPRVRKPTGKSSRKYSRSVKGSGDYRRPSRKQLVQRQPIRTLRG
                     RGDYKSVLGTILKGAGIVAGGMTGGLPGALKGGLTAGALGNLMGLGDYRVKTNSLVRP
                     GMNDQIPSMHNSKGCVRIQHREYLGDILGSTTFENRSYAINPADVITFPWLSQIAVNF
                     EQWVPKGIIFEFRTMSSDTQIGSTVSLGTVILATEYNVLLNRFISKQQMENSQFSMSV
                     KPSNSCIHPVECDPSQTPNMPLFVKVPSASLGDARLYDLGNFQLATQGMTATGAGQVI
                     GELWCSYDIEFCKPVLSAGDSVFADSASYNLQDLQTGIGYTSPGLLGDITFLQAAANK
                     NYDQIGLTFPGGASDRILFPASANGRYEVDIYFSGSILSASDFDVAPLITTSGTVSLV
                     SVLPSSGGYLASSSVQRVYSGVAPDPATFAAHSFHLTAQVQVNGNGVDTGLLFLKPAL
                     AATGANYPTGAPTTFGLLMVNQVYTPPVSSVTLSREDNLLQSLQDQISALQVQRNDKS
                     AAVSISEDINDSDSINCVINREVYDRMCRTIDSYDNKKKEIDEIVSRVESSIKKSSSK
                     Q*"
     misc_feature    2125..2151
                     /note="stem-loop"
     misc_feature    2134..2142
                     /note="nonanucleotide"
ORIGIN
        1 ctgatgaaag aattgacagg attatttaat tcaaaaaagt tagataaact tttgggaaaa
       61 ttttataaag aaaaatcttc agtaaatttt attgatgaaa aaattgatga agaaattaat
      121 actgacaata ttaaaaaagt tagatttctt gatgaaatag aaatcaaagt tattgaaaat
      181 tgtttagcag aattagataa tggcgaaaaa gatgatggat catttcgcat tcaaaaaatg
      241 atgaaatgta tgttaacata tccagatcat ttacctaaag aagaatatat taaatggttt
      301 gaagataaaa ttggcaaatg caaatttata agaattgccc atgaaacagg caaaagtggt
      361 gaacatgatt atgaacattc acacgttcta atagaattag aaaaaccatt ttcttcaaca
      421 aagaaaaata tatttcactt taaagatttc agacatccac aatttaaata ttttaaaatg
      481 tccaatatgt tacattggaa aaatagtgtg agatatcttg gaaaagaaga cccagataat
      541 aaagatttat taactgtgca tactaatttt gttgacgaaa tttggtcaaa aaataaagaa
      601 gatgctttac aatcatgtca aagaccagca gatgcaatgg gtattttgac attatggaaa
      661 aataaaccaa tggttatgaa accacctaaa tacttaaaag aattaggagg tgaaacttgt
      721 ccacataatt ttatgagatg gcaacataat attcatcata tgatgaaatt taatacacaa
      781 ggtaatcgaa agattacttg gatatatgat gcaattggtg gaaaaggtaa atcttgtttt
      841 gcagatgact tagttgcagc aaagattgct tacaaagtaa aaagtaacgg tggaggttac
      901 attcattgca acgagatgat tggagaagca tatgaatcag gattatggaa tggcgaaaat
      961 attattttcg attggccaag acaaactgat ttcatagata gaaaaggatt gtatggtttg
     1021 attgaagcaa tcaaagatgg aactttgtca agaacaaagt atattggtaa aacaattaac
     1081 atacaattag atggaaacaa aaatactcaa gtatttatat ttgctaattt tctaccacaa
     1141 tataaagatg aacatggtca tttgacatta tctttagata gatgggatgt tcatgaaata
     1201 ggaaaacacg gtggttttaa ttcccgattt cataaaactc agaattcaat tattaaaaat
     1261 ataagaaatg aatttgaaaa aaaccaagat gaacatactg aagatgaaga agaatcttgg
     1321 tttgatgata ctaaaatata tgaataaata atatatgtta gtatataact tagaatttgg
     1381 atagtatcaa aatccagtaa agtagggccc caaccaactg tgatgtgcgc aagcgcacat
     1441 cacacttggg accatcccta ctccttacgt aataggttag atactaacat attacttagc
     1501 ctattaacta cgctaaagct acgtgaatag aggctaaaac tcttaacatt aaaggagttt
     1561 tagcaatact ataataaata gactaataaa tgtataatgc ggttgcccac gaaaagttaa
     1621 aattgtaatt tcttttataa attattaaac taatttttta attttgaaaa aaggttattt
     1681 tcttccataa aaaataaatt agtgatgaga cttaaaattt ttttgatgct taagttgacg
     1741 gggcaactga agcatcggag gaaaaagaat gttagactac atctctaatt tattttttat
     1801 gttcagaaaa taactttttt ctaaaatcaa aaaaataaac taataattta taagaaatta
     1861 caattttgaa acttttgtgg gcaacctaaa aaagtaaata aaaaaataat ttgtataaat
     1921 aaagatgtag taattagttt aatccgctca ctatcgttcg cgtcataaac agtaaaatag
     1981 aaatttattg cttagaagaa gatttcttaa ttgatgattc aactcgagaa acgatttcat
     2041 ctatttcttt cttcttgttg tcataagaat caatagtgcg gcacatacga tcatatacct
     2101 cacgattaat aacacaatta atactatcag aatcattaat atcttctgat atagaaacag
     2161 cagcagattt atcattgcgt tgaacttgca atgcagaaat ttgatcttgt aaagattgaa
     2221 gaagattatc ttcacgtgat aaagtaaccg aagatacagg aggtgtataa acttgattga
     2281 ccataagtaa accaaatgta gttggtgcac cagtaggata atttgcacca gtagcagcga
     2341 gtgcaggttt aagaaataat aaaccagtat cgacaccatt tccattaact tgaacttgag
     2401 cagtaagatg aaatgaatgt gcagcaaaag ttgcaggatc gggtgcaaca ccagagtaaa
     2461 ctctttgaac agaagatgat gcgagataac caccagaact tggtaaaaca gaaaccaaag
     2521 atacagttcc ggaagttgta ataagtggtg caacatcaaa atcagatgca gataaaatag
     2581 aaccactaaa ataaatatcg acttcatatc taccatttgc agatgcagga aatagaattc
     2641 gatcagaagc accaccagga aaagtaagcc caatttggtc ataattttta tttgcagcag
     2701 cttgaagaaa tgtaatatca cccaataaac caggtgatgt gtaaccaata ccagtttgta
     2761 aatcttgaag attatatgat gcagaatcag caaacacaga atcaccagca gataaaacag
     2821 gtttacaaaa ttcaatatca taagaacacc ataattcacc gataacttgt ccagcaccag
     2881 ttgcagtcat gccttgtgtt gcaagttgaa aattacccaa atcataaaga cgagcatcac
     2941 cgagtgaagc cgatggaact ttaacgaata atggcatatt tggtgtttgt gatggatcac
     3001 attcaacagg atgaatacat gaatttgaag gtttaacaga catagagaat tgagaattct
     3061 ccatttgttg tttagaaata aaacgattaa gtaatacgtt atattcggtt gcaagaataa
     3121 ccgttcctaa tgaaacagtt gaaccgattt gagtatcaga agacattgtt ctaaactcaa
     3181 agataattcc tttcggaacc cattgttcaa aattaacagc aatttgtgat aaccaaggga
     3241 aagtaataac atccgcagga ttaatagcat aagatctatt ttcaaaagtt gtcgatccaa
     3301 gaatatcacc aagatattca cgatgttgaa tacgaacaca acctttagaa ttatgcatag
     3361 atgggatttg atcattcata ccaggacgaa ctaatgaatt agtcttaaca cgataatcac
     3421 ctaaacccat aagattacct aatgcaccgg cggttaaacc acctttcaaa gcaccaggta
     3481 aaccaccagt cattccaccc gcaacgatac cagcaccttt caaaattgtg ccaagcacag
     3541 atttataatc gccacgacca cgcaatgttc tgattggttg gcgttggact aattgtttgc
     3601 gagaaggtct tcgataatct ccacttcctt ttacagatcg tgaatatttt cgagatgact
     3661 taccagttgg tttgcgaacg cgaggcattt ttgtaattat atattacaac gcaaatttaa
     3721 ttaattaata attaattaaa taaataatat attatatatt atataactaa ctaactaact
     3781 aactaa
//
LOCUS       CruV-352                3790 bp    DNA     circular     24-FEB-2020
DEFINITION  Crucivirus-352 isolate 120_384_IR1_3296.
ACCESSION   CruV-352
KEYWORDS    .
SOURCE      Crucivirus-352
  ORGANISM  Crucivirus-352
            Unclassified.
REFERENCE   1  (bases 1 to 3790)
  AUTHORS   de la Higuera,I., Kasun,G.W., Torrance,E.L., Pratt,A.A.,
            Maluenda,A., Colombet,J., Bisseux,M., Ravet,V., Dayaram,A.,
            Stainton,D., Kraberger,S., Zawar-Reza,P., Goldstien,S., Briskie,J.,
            White,R., Taylor,H., Gomez,C., Ainley,D.G., Harding,J.S.,
            Fontenele,R.S., Schreck,J., Ribeiro,S.G., Oswald,S.A., Arnold,J.M.,
            Varsani,A., Enault,F. and Stedman,K.M.
  TITLE     Direct Submission
  JOURNAL   Submitted (24-FEB-2020) Biology, Portland State University, 1719 SW
            10th Ave, Portland, Oregon 97201, USA
COMMENT     Bankit Comment: ALT EMAIL:ide@pdx.edu
            Bankit Comment: TOTAL # OF SEQS:451
            Bankit Comment: GAP: est. length - alignment

            ##Assembly-Data-START##
            Assembly Method       :: MetaSPAdes v. 3.12; IDBA-UD v. 1.1.1;
                                     MEGAHIT v. 1.0.3
            Sequencing Technology :: 454; Illumina
            ##Assembly-Data-END##
FEATURES             Location/Qualifiers
     source          1..3790
                     /organism="Crucivirus-352"
                     /mol_type="genomic DNA"
                     /isolate="120_384_IR1_3296"
                     /isolation_source="Water"
                     /country="Norway"
                     /collection_date=2011
     CDS             1..1347
                     /codon_start=1
                     /product="Putative Replication Associated Protein"
                     /translation="MSKSQNIHWCFTINKGESILLGPKLMDEFEDIVRYAAWELEEGT
                     TTGNLHIQGYVELKEKTSLRGICSLFPNWNAHFEPRMGTREQARDYCMGIENGEPKVG
                     VLQGPWEFGEFKAGGQGARNDLRSVANSIIAGTNIMEIALEHPVEFIKFNKGIERLHS
                     LIQPRRNWKTEIIVLWGKSGTGKSKLAWDCDPNAYEKAAEHTWWDGYNGEDTIIIDDY
                     AGELPFRYLLRLADRYPMQVQIKGGTTQFRSRRIIFTTNIHPKYWYKNEDYATFERRI
                     TMLCEVQVDENNLIKVNSEKNKYLSEVAGNTKIPRNFINFCNLLPPLIPLDLITDIED
                     ESEALNKYRIMLNNLASEIIPHLSEFPSSEEFPEPEELIKLQIAQDEKLAQEQIYRQE
                     EYEKNQVKNNINQQKSMLGKINDDEEFEAKCREKKRKEKEERNKRRAIRLQGKINN*"
     CDS             complement(1840..2367)
                     /codon_start=1
                     /product="putative ORF"
                     /translation="MGTTQIMQHDSMGVTKNGDSFTLPSSQYGTFLINWVLYLPLGGT
                     LGVDGTQDLTNCVGVNLWDGMSETGWKTPASILNISHISNTFTISKAEGVLATIKPFD
                     GADFTGLYTGDLFITKIDDTVTSFSINYPSSEEDFSNIETDEEFSDDEEKSTEGIILF
                     EKVINTNEGCASTIM*"
     CDS             complement(2446..3714)
                     /codon_start=1
                     /product="Putative CruV Capsid Protein"
                     /translation="MPRIAEKVKKIRKPKDDEEVVVVKKKKTKKPAARSSYWDQFSRY
                     VPYAKQAGQLKDAATAAYKGKYSDAAGHIFDVLIGAGGYRVKKNSLTNRSQVPSMHSI
                     KDGFRIRHREYLGDVSSSIAFTNTVYNVNPGLDETFPWLSAIAQNFDQYEIKGMIVEF
                     KSTSANALNSTNTALGTMILCAEYDSADSTYVNKQQMENSMYACSTKPSESMIMPIEC
                     DPRQNPIGNLYTRSGAVPSGKDSRLYDLCNFQIASVGSQAAAVVGELWISYDIILRKP
                     QMSSGLGLALDSYHLQMTNAAGDNPLGTARTELFDTYGITATNVALTMMQGHSGIHKL
                     TYNVTGDSVAVTSPIVTYTNAAEVLVYNNSASGHVQSGGTTTLFQFESVFRITDPSLP
                     VVITFGVAGTLPTSPGVSDCSISQLNGDYV*"
ORIGIN
        1 atgtctaaaa gtcaaaatat tcattggtgt ttcacaataa ataaaggaga atctatctta
       61 cttggtccta aattaatgga tgaatttgag gacatcgtaa gatatgctgc atgggaatta
      121 gaagaaggaa caacaactgg aaatctccat atccaaggct atgtagaact aaaggaaaaa
      181 acatctcttc gaggtatttg ttctttattt cccaattgga acgctcattt cgaacctcgc
      241 atgggtactc gcgagcaagc ccgggattac tgcatgggga tagaaaatgg tgaacctaaa
      301 gttggagttt tacagggacc ttgggaattc ggtgaattta aggcaggtgg tcaaggagca
      361 cgcaatgacc ttcgttcagt tgctaattca ataatagctg gcactaatat tatggaaatt
      421 gctcttgagc atcccgtaga atttattaag tttaataaag gaatagaaag acttcattca
      481 cttatccaac cacgacgtaa ttggaaaact gaaataatag tcttatgggg aaaatcaggt
      541 acaggcaaat ctaaattagc ttgggattgt gatccaaatg catatgagaa agcagccgaa
      601 catacctggt gggatggata taatggcgaa gacacaataa taatagatga ttatgcgggt
      661 gaactacctt tcaggtatct tctaagactg gcagataggt accctatgca agtccaaatt
      721 aagggcggaa ctactcaatt tcgtagtaga cgtattattt tcacgaccaa tattcatcct
      781 aaatattggt ataaaaatga agactatgct acatttgaac gtcgaataac tatgttatgc
      841 gaggttcaag ttgatgaaaa taatttaatc aaagtcaatt cggaaaaaaa taaatattta
      901 tccgaagtgg cgggtaatac taagattccg agaaatttca taaatttctg caatctttta
      961 ccgccactaa ttcctttaga tttaattacc gatatagaag acgaatcgga agctttaaat
     1021 aaataccgaa taatgttaaa taatttagca agcgaaataa ttccccattt atccgaattc
     1081 cctagttccg aagaatttcc tgaaccagaa gaattaatta aattgcaaat tgcacaagat
     1141 gaaaaattag ctcaagaaca aatatatcgt caggaagaat atgaaaaaaa ccaagttaaa
     1201 aataatatta atcaacaaaa atctatgtta ggaaagatta atgatgatga agaatttgaa
     1261 gcaaaatgcc gagagaaaaa acgtaaagaa aaagaagaaa gaaataaacg aagagcaatt
     1321 agattacaag gaaaaattaa taattaacta attatatatt ttttttgtaa acgtttcacg
     1381 cggaaaagat attttccgcg gagtttcgtg tggttttgaa acttttagaa aatccgtaat
     1441 taatataaaa ttcaaatctc gctccgctcg atttgaagtt tcacagttaa tctacgattt
     1501 tgaaaaattc caaaaccgcg cgagagtcag tggagagtcc ctttcaggtt tcgttcgaaa
     1561 tcagaatatt tattatttat tctaaataat ataatataca agggcatata aaacacacat
     1621 cactattttt ctacaaaaca gcaatctcta tgcctagtga ttttagtttt tttaaaaaaa
     1681 taattattca cataattacg tttacatatt ttttatattt atttattact ttttcaagta
     1741 atataatata taaggatact taagatacat gtcgctattt ttctacaaaa caataatctc
     1801 tatgcctagt gattttagtt tttttaaaaa aataattatt cacataattg tactagcaca
     1861 accttcattt gtgtttatta ctttttcaaa taatataatt ccttcagtcg acttttcttc
     1921 atcatcagaa aattcttcat cagtctcaat attagaaaaa tcttcttccg aagatggata
     1981 attaatacta aaagaagtaa ctgtatcatc aattttagtt ataaataaat caccagtata
     2041 taaccctgta aaatcagcac catcgaatgg ttttatcgta gctaaaacac cctctgcttt
     2101 actaattgta aaggtattag aaatgtgact tatatttaaa atagaagctg gagttttcca
     2161 tccagtttct gacattccat cccataaatt tacacctaca caattcgtga ggtcctgcgt
     2221 accgtctact cctagtgttc cacctaaagg tagatacagg acccaattta ttaagaatgt
     2281 accatattgg ctacttggca atgtaaagct atctccgttt tttgtcacac ccatggaatc
     2341 gtgctgcata atttgtgtag tacccaaagt atttgtctca ccggctccag taaaccgtag
     2401 atgagcactg gcaattgtgc aaccggaatc cgatgggtcg gatccctaaa cataatcacc
     2461 attcaattga gaaatggaac aatctgaaac tcctggcgaa gttggcagag taccagcaac
     2521 accaaaagta ataacaacag gcaaactcgg atcagtaatt ctaaagacac tttcgaattg
     2581 aaacagagta gtcgtacctc cagactgaac atgtcccgaa gctgaattat tataaacaag
     2641 tacttcggcc gcattagtgt acgtcactat aggtgaagtc acagcaactg aatcgcccgt
     2701 tacattatat gtcaacttat gaataccact atgaccttgc atcatagtta atgcaacatt
     2761 tgttgcagta ataccataag tatcaaacaa ctcagtacga gcagtgccca atggattatc
     2821 acctgcagca ttagtcattt gcaaatgata actatctaaa gctagaccca aaccactact
     2881 catctgaggt ttacgaagaa taatatcata tgaaatccat agttctccaa ccaccgctgc
     2941 tgcttggcta cccacggatg caatctgaaa attacacaaa tcataaagac gagagtcttt
     3001 accactaggt actgctccag aacgagtgta aagatttcca atcggatttt gacgagggtc
     3061 acattcgata ggcataatca tactttcgct aggcttagta gaacaagcat acatactatt
     3121 ttccatttgc tgcttattta cataagtaga atctgccgag tcatattcag cacatagaat
     3181 catagtaccc agggcagtgt ttgttgaatt tagcgcattc gcagaagtgc ttttaaattc
     3241 cacaatcata cccttaatct catattggtc aaagttttga gcaatagcag atagccaagg
     3301 aaatgtctca tccaatcctg gatttacatt atacacagta ttagtaaacg caattgaact
     3361 agaaacgtcc cctagatact ctcggtgcct aattctaaac ccatctttaa tactatgcat
     3421 agaaggaact tgagacctat tcgttaaaga attcttttta actctataac ctccagcacc
     3481 aatcaaaaca tcaaatatat gaccagcggc atcagaatat ttgcccttat acgcagctgt
     3541 agcggcatct ttcaattgcc ctgcttgctt tgcataagga acataacgag aaaattgatc
     3601 ccaataagaa ctcctagcag caggcttctt agtcttcttc ttttttacaa caacaacttc
     3661 ttcatcatct ttgggtttcc taattttctt tactttttca gcaattcgtg gcatgtttat
     3721 ttaattgttt tcgcaaaaag tatgaggggt atatatacaa cttaccttac ctttaacatt
     3781 ttatacatac
//
LOCUS       CruV-353                3791 bp    DNA     circular     24-FEB-2020
DEFINITION  Crucivirus-353 isolate SR1_21045.
ACCESSION   CruV-353
KEYWORDS    .
SOURCE      Crucivirus-353
  ORGANISM  Crucivirus-353
            Unclassified.
REFERENCE   1  (bases 1 to 3791)
  AUTHORS   de la Higuera,I., Kasun,G.W., Torrance,E.L., Pratt,A.A.,
            Maluenda,A., Colombet,J., Bisseux,M., Ravet,V., Dayaram,A.,
            Stainton,D., Kraberger,S., Zawar-Reza,P., Goldstien,S., Briskie,J.,
            White,R., Taylor,H., Gomez,C., Ainley,D.G., Harding,J.S.,
            Fontenele,R.S., Schreck,J., Ribeiro,S.G., Oswald,S.A., Arnold,J.M.,
            Varsani,A., Enault,F. and Stedman,K.M.
  TITLE     Direct Submission
  JOURNAL   Submitted (24-FEB-2020) Biology, Portland State University, 1719 SW
            10th Ave, Portland, Oregon 97201, USA
COMMENT     Bankit Comment: ALT EMAIL:ide@pdx.edu
            Bankit Comment: TOTAL # OF SEQS:451
            Bankit Comment: GAP: est. length - alignment

            ##Assembly-Data-START##
            Assembly Method       :: MetaSPAdes v. 3.12; IDBA-UD v. 1.1.1;
                                     MEGAHIT v. 1.0.3
            Sequencing Technology :: 454; Illumina
            ##Assembly-Data-END##
FEATURES             Location/Qualifiers
     source          1..3791
                     /organism="Crucivirus-353"
                     /mol_type="genomic DNA"
                     /isolate="SR1_21045"
                     /isolation_source="Water"
                     /country="New Zealand"
                     /collection_date=2012
     misc_feature    join(3778..3791,1..19)
                     /note="stem-loop"
     misc_feature    1..9
                     /note="nonanucleotide"
     CDS             308..1603
                     /codon_start=1
                     /product="Putative CruV Capsid Protein"
                     /translation="MPGRYVRKYPARRASSSRAVVYRGKGAYRRSAPARRYSRRVRYV
                     RPVKAPQQSSGSWLSDVGSKAGSALGSIVGKFLGLGEYKMARNSLINGSSPNQVPFMH
                     SAKDGVRIRHREFIADITTQAAFTNTSYSVNPGLSLSFPWLSALAQNFEEYRFEGLVF
                     EYVPMCGDAVSSTNSTMGVVVMAAEYNPTQAAYVNKQQMENSMWAMSDKPSKPIALPI
                     ECAPSLNPLSNQYIRIGAVASGQDVRFYDLCNIQVATVGAQASFKAGELWATYDIVLM
                     KPQLDSGLALSAEIAHYTLVAPSESGSAWFGTSRSAYYDNIGLTVAGASITFPLGSQG
                     TYHISYSQNGASTVLTNINTPTYTNCAQVTSYLNNNTLSAIRNGGTTSSIYFNSSYVY
                     IADPTLQAVVTFSGGTLCTTPTYGDLIVTQVNDNFRLNP*"
     CDS             1679..2041
                     /codon_start=1
                     /product="putative ORF"
                     /translation="MGTSQTLGFDNIGITFDPLGEYFDFPLGAYGRYYVNYVIYGASN
                     AVVKPTLSFTNCSSDSLFKGFSQNYISNSGSTDVQLIYSFCVFVTSVSLKARISFTGG
                     ILPGAVSSGDLFICEYFP*"
     CDS             2072..2593
                     /codon_start=1
                     /product="putative ORF"
                     /translation="MLNKLFFLMYIILNMSDPSCCSECVKLRDDFNKIIHVVSCMDQK
                     FDELFERIQVLEEIIDESHLAESDEDEDEKKPNDDCRRLATSPIHILGELDSDEDCKS
                     PASTALEWKDYSQSVEKLFKVDPPKNGVKYTHIEHDTFLKSGYDQRVGFCWLNFKNDE
                     GASWWEVGHRRSG*"
     CDS             complement(2776..3756)
                     /codon_start=1
                     /product="Putative Replication Associated Protein"
                     /translation="MSIRKTIITSYLFQEREFEANLKTKEFAQVKFAVIQEELCPKTN
                     KMHHQGYLEFNKPVTLAFIKKLTGDNTTHVVTQKMKGTPLQGAEYCGYNNGGETEKNK
                     EKAIERIKNNWYYFEHGKLEWQEQGKRNDINEVVDMIHEGKQLKEIVEEYTSTYIRNY
                     KGIERAMQIMGKPLTTPKIEEKQVFLNYGESGTGKTELIYNSHPIHEIFRKPPGQWWD
                     GYCGQKYILIDDIQPNEYKIEEFLQWLDKYPISVPIKGSFVPLTCTHIYLTSNFDPNK
                     WFNYKSIESYKAIMRRITKIKYHEKELLYDILKKEIETDWVWDIAQGTAV*"
ORIGIN
        1 tagtattacc tatagctacc tctgtatttt ttttgaaaaa aaaaataaaa aattggattt
       61 atcctaattt ttcggtttat tcgctgaggt tacgcgaaag agaatttttt ttttataaaa
      121 taaataatta tcggtcccga agggattcgc aatcataaaa tgtcttagaa tgcaaaatat
      181 aattttgagg aaaaataaat atttatttgt tccgttgcat ttttttagtt aattaattat
      241 ttatttattt tttaaaaata atataatact taatttatat ctttgataga aaatattttt
      301 ggaaaaaatg cctggtcgtt atgttcgtaa gtatccagct agacgagctt caagctctcg
      361 agctgttgtt tatagaggta agggtgctta ccgtaggtct gctcctgcta gacgttattc
      421 tcgtagagtt cggtatgtaa gacctgttaa ggctcctcaa caatcttcag gttcttggct
      481 ttctgacgta ggaagtaaag caggttctgc tttgggtagt attgttggta aatttttggg
      541 attaggtgaa tataagatgg ctcgtaattc tttaattaac ggatcatctc ctaatcaagt
      601 gccgtttatg cattctgcga aggatggtgt aagaattaga catcgtgagt ttattgcaga
      661 tattacaact caagccgcgt ttactaatac tagttattca gtaaatcccg gattatcttt
      721 gtcttttcct tggttgtctg ctttagctca aaacttcgaa gagtatcgat ttgaaggttt
      781 agtttttgag tatgttccta tgtgtggtga tgctgtatca tcaactaatt caacaatggg
      841 tgttgttgta atggctgctg agtataatcc aacgcaagcg gcttatgtta ataagcaaca
      901 gatggagaat tcaatgtggg cgatgtctga taagcctagt aagccaattg ctttacctat
      961 tgaatgtgct ccttctttga atcctttgtc taatcaatat attcgtattg gtgctgttgc
     1021 tagtggtcaa gatgttagat tttatgattt gtgtaatatt caagttgcaa cggtaggcgc
     1081 tcaagcgtct tttaaagctg gtgagctttg ggcaacttat gatattgttt taatgaaacc
     1141 tcaattagat tctggattag cgttatctgc tgagattgct cattatactt tagtagcacc
     1201 ctcagaatct ggttctgcat ggtttggaac atcacgtagt gcatattatg ataatattgg
     1261 gttaacggtt gctggagcta gcattacgtt ccctcttggt tctcaaggaa cttatcatat
     1321 ttcatatagt caaaatggag catctactgt tttgactaat attaatactc ctacgtatac
     1381 aaactgcgct caagtcacat catacttgaa taataacaca ttgagtgcca ttcgtaatgg
     1441 tggaactact agttctattt attttaattc aagttatgtg tatattgctg atccaacact
     1501 tcaagctgtt gttacgtttt ctggtggcac attgtgcact actcctacct atggagatct
     1561 tattgttacg caagttaatg ataattttag attgaatcct taggacagtg aacccgatat
     1621 tgatgacaca gcagcatcta cttattatcg gcttattgct cctaccactg tttttcattt
     1681 gggaacttct caaactcttg gatttgataa tattggtata acatttgatc cattaggtga
     1741 atattttgat tttcctcttg gtgcatatgg tcgatattat gttaattatg ttatttatgg
     1801 tgcttctaac gcagttgtaa aacctacttt atcatttact aattgttctt ctgatagtct
     1861 ttttaaaggt ttttcccaga attatatttc taatagtggt tcaactgatg ttcagttaat
     1921 ttattctttt tgcgtttttg tcacaagtgt ttctttaaaa gctcgtattt cttttaccgg
     1981 tggaatttta cctggtgctg tttcttcggg tgatttattt atttgtgaat attttccttg
     2041 atttaaatcg ggttcagagc taattaatta tttgttaaat aaattatttt ttttaatgta
     2101 tataatttta aatatgtctg atccttcttg ttgttctgaa tgtgttaaac ttcgtgatga
     2161 ttttaataag attattcatg ttgtttcttg tatggatcaa aagtttgatg agttatttga
     2221 acgaattcaa gttttggaag aaattattga tgaatcacat ttggctgaat ctgatgaaga
     2281 tgaagatgaa aaaaaaccaa atgatgattg taggcgatta gcaacatctc ctattcatat
     2341 tttgggagaa ttagacagtg atgaggattg taagagtcct gcatctactg ctttggaatg
     2401 gaaagattat tctcagtctg ttgagaagtt atttaaggtt gatcctccaa agaatggagt
     2461 taagtatact catattgaac atgatacttt tcttaaaagt ggttatgatc aaagagttgg
     2521 attttgttgg ttaaatttta aaaatgatga aggtgccagt tggtgggaag ttggtcatag
     2581 acgttctggt taattatttt aatcagtatt ttttttaaat tttgatttta gctgtatcac
     2641 gcgcgacctg cagttttttg tataaaaatg aacaaaaaaa acataactta tcccattagg
     2701 gtgcgtagcg tagctacgca tctgaaagaa taattatttt tttaattttt ttatacgtgg
     2761 aactgcatac gtgagttaaa cagcggtccc ttgggcaata tcccataccc aatctgtttc
     2821 tatttctttt tttaaaatat catataataa ttctttttcg tgatatttta tttttgttat
     2881 tctcctcatt attgctttgt aactttctat acttttgtag ttgaaccatt tgtttgggtc
     2941 gaagttactt gttaaatata tgtgtgtgca tgttagtgga acgaatgatc cttttattgg
     3001 cacacttata gggtatttat caagccattg taagaattct tcaattttgt attcatttgg
     3061 ttgtatatca tcaattagaa tatatttttg tccacaatat ccgtcccacc attgtcccgg
     3121 tggttttcta aatatttcgt gaattggatg tgaattatat attaattcag tttttcctgt
     3181 tcctgattca ccgtagttta agaaaacttg tttttcttca attttaggtg ttgttaatgg
     3241 tttacccatt atttgcattg ctctttcaat gcctttgtag tttcttatgt aggttgatgt
     3301 atattcttca actatttctt ttagttgttt tccttcgtga atcatatcaa ctacttcatt
     3361 tatgtcgttt cttttgcctt gttcttgcca ttctaatttt ccgtgttcaa aataatacca
     3421 attatttttt attctttcta ttgctttttc tttgtttttt tctgtttctc ctccgttgtt
     3481 gtaaccacaa tattcagctc cttgtaaagg tgttcctttc attttttgtg ttacaacatg
     3541 tgttgtattg tctccagtta attttttaat aaatgctaat gttactggtt tgttaaattc
     3601 aaggtatcct tgatgatgca ttttatttgt ttttggacat aattcttctt gaattactgc
     3661 gaatttaact tgagcaaatt cttttgtttt taaatttgct tcaaattctc tttcttgaaa
     3721 aagatatgaa gttataattg tttttctaat tgacatcttt atttattttt ttttattgta
     3781 gctatagttt t
//
[truncated: 1,944,713 more chars]
